# Recurrent Signature Patterns in HIV-1 B Clade Envelope Glycoproteins Associated with either Early or Chronic Infections

**DOI:** 10.1371/journal.ppat.1002209

**Published:** 2011-09-29

**Authors:** S. Gnanakaran, Tanmoy Bhattacharya, Marcus Daniels, Brandon F. Keele, Peter T. Hraber, Alan S. Lapedes, Tongye Shen, Brian Gaschen, Mohan Krishnamoorthy, Hui Li, Julie M. Decker, Jesus F. Salazar-Gonzalez, Shuyi Wang, Chunlai Jiang, Feng Gao, Ronald Swanstrom, Jeffrey A. Anderson, Li-Hua Ping, Myron S. Cohen, Martin Markowitz, Paul A. Goepfert, Michael S. Saag, Joseph J. Eron, Charles B. Hicks, William A. Blattner, Georgia D. Tomaras, Mohammed Asmal, Norman L. Letvin, Peter B. Gilbert, Allan C. DeCamp, Craig A. Magaret, William R. Schief, Yih-En Andrew Ban, Ming Zhang, Kelly A. Soderberg, Joseph G. Sodroski, Barton F. Haynes, George M. Shaw, Beatrice H. Hahn, Bette Korber

**Affiliations:** 1 Theoretical Biology, Los Alamos National Laboratory, Los Alamos, New Mexico, United States of America; 2 Santa Fe Institute, Santa Fe, New Mexico, United States of America; 3 SAIC-Frederick, National Cancer Institute, Frederick, Maryland, United States of America; 4 Departments of Medicine and Microbiology, University of Alabama at Birmingham, Birmingham, Alabama, United States of America; 5 Center for Molecular Biophysics and Department of Biochemistry, Cellular & Molecular Biology, University of Tennessee, Knoxville, Tennessee, United States of America; 6 National Engineering Laboratory of AIDS Vaccine School of Life Science, Jilin University, Changchun, China; 7 Duke University Medical Center, the Departments of Medicine and Surgery, and the Duke Human Vaccine Institute, Duke University, Durham, North Carolina, United States of America; 8 Department of Biochemistry and Biophysics and the Division of Infectious Diseases Center for AIDS Research, University of North Carolina at Chapel Hill, Chapel Hill, North Carolina, United States of America; 9 Aaron Diamond AIDS Research Center, an affiliate of the Rockefeller University, New York, New York, United States of America; 10 Institute of Human Virology, University of Maryland, School of Medicine, Baltimore, Maryland, United States of America; 11 Beth Israel Deaconess Medical Center, Boston, Massachusetts, United States of America; 12 Division of Viral Pathogenesis, Department of Medicine, Harvard Medical School, Boston, Massachusetts, United States of America; 13 Vaccine Infectious Disease Division, Fred Hutchinson Cancer Research Center, Seattle, Washington, United State of America; 14 Department of Biochemistry, University of Washington, Seattle, Washington, United States of America; 15 Arzeda Corporation, Seattle, Washington, United States of America; 16 Department of Epidemiology and Biostatistics, College of Public Health, University of Georgia, Athens, Georgia, United States of America; 17 Dana-Farber Cancer Institute, Department of Cancer Immunology and AIDS, Boston, Massachusetts, United States of America; The Salk Institute for Biological Studies, United States of America

## Abstract

Here we have identified HIV-1 B clade Envelope (Env) amino acid signatures from early in infection that may be favored at transmission, as well as patterns of recurrent mutation in chronic infection that may reflect common pathways of immune evasion. To accomplish this, we compared thousands of sequences derived by single genome amplification from several hundred individuals that were sampled either early in infection or were chronically infected. Samples were divided at the outset into hypothesis-forming and validation sets, and we used phylogenetically corrected statistical strategies to identify signatures, systematically scanning all of Env. Signatures included single amino acids, glycosylation motifs, and multi-site patterns based on functional or structural groupings of amino acids. We identified signatures near the CCR5 co-receptor-binding region, near the CD4 binding site, and in the signal peptide and cytoplasmic domain, which may influence Env expression and processing. Two signatures patterns associated with transmission were particularly interesting. The first was the most statistically robust signature, located in position 12 in the signal peptide. The second was the loss of an N-linked glycosylation site at positions 413–415; the presence of this site has been recently found to be associated with escape from potent and broad neutralizing antibodies, consistent with enabling a common pathway for immune escape during chronic infection. Its recurrent loss in early infection suggests it may impact fitness at the time of transmission or during early viral expansion. The signature patterns we identified implicate Env expression levels in selection at viral transmission or in early expansion, and suggest that immune evasion patterns that recur in many individuals during chronic infection when antibodies are present can be selected against when the infection is being established prior to the adaptive immune response.

## Author Summary

A single virus most often establishes HIV-1 infection. As a consequence, virus sampled early in infection is usually very homogeneous. A few months into the infection, the virus begins to accumulate mutations as it evolves to evade HIV-specific immune responses mounted by the infected host. During chronic infection, the viral population diversifies, reflecting the history of mutations that arose within that infected individual. We hypothesized that particular amino acids might confer a selective advantage during transmission or early infection, and others might recur during chronic infection because they provide common and effective strategies of immune escape. We compared a large number of viral sequences from several hundred infected people sampled soon after transmission or during chronic infection to identify such infection-status “signature” patterns. A particularly robust signature was identified in the signal peptide of Envelope, a region that regulates its expression. Other signatures were found in regions of Envelope that interact with its cellular receptors, or are implicated in immune escape.

## Introduction

It has proven to be very difficult to elicit protective immunity through an HIV vaccine [1], although a recent vaccine trial in Thailand, RV144, yielded encouraging results [2]. A protective vaccine will need to elicit immune responses that interact effectively with the spectrum of circulating viral strains, and HIV is a remarkably diverse virus [3], [4], [5]. Against this backdrop of variation, if viruses sampled early in infection exhibit a more constrained pattern of diversity at than chronic viruses, i.e. exhibit statistically enriched signature patterns related to transmission or establishing infection, then designing vaccines that incorporate such signatures may be beneficial, and such signatures may yield insight into the biology of viral transmission and disease progression.

Several aspects of the biology of sexual transmission of HIV motivated this systematic search for early versus chronic infection signatures. First was the genetic bottleneck at transmission. It has long been apparent that HIV-1 undergoes extensive diversification during the course of an infection [6], [7], [8], [9], [10], and that viruses sampled from early in infection are less diverse than chronic samples [11], [12], [13], [14], [15]. Improved sampling, modeling strategies, and experimental methods have added greater clarity to this, and recent studies indicate new infections are established by a single virus in approximately 80% of HIV-1 heterosexual transmission cases [16], [17], [18], [19], [20]. By an infection being established by a single virus, we mean that only one lineage is apparent in the viral population sampled early in infection, and that the sampled data is fully consistent with a single founder virus that was transmitted and that expanded in accord with a model of early viral diversification using established parameters for HIV mutation rates and generation time [17], [21], [22]. In addition, the estimated time of infection in homogeneous infections based on experimentally defined Fiebig staging is consistent with estimated times to the most recent common ancestor based on viral diversity [17], [18]. In these cases, the virus that established the infection and was presumably transmitted can be modeled and reconstructed from sequences sampled in early infection, and synthesized for further study [23]. The appropriateness of these models has been confirmed experimentally in macaques where the inoculum, infecting strains and time of infection were known [24], [25]. The rates of multi-variant transmission in men who have sex with men (MSM) [26] and in individuals with inflammatory genital infection [20] are higher, indicating that barriers to transmission may be reduced in these circumstances. The high mutation and replication rates of the virus in a newly infected host provides the baseline for acquisition of genetic diversity, enabling escape from host cytotoxic T lymphocyte (CTL) [27], [28], [29], [30], [31] and antibody [30], [32], [33] responses, and adaptation in a rapidly changing landscape of in vivo selection pressures.

Our second motivation for this study was that a sequence pattern associated with early viruses had already been defined, so a systematic extended search for more patterns seemed likely to yield results. The known pattern was that hypervariable loops of HIV-1 Env tend to be shorter and to carry fewer potential N-linked glycosylation sites (PNLGs) than their chronic counterparts [34], [35]. One hypothesis to explain this is that while larger loops may mask epitopes recognized by neutralizing antibodies, and so may be acquired during the course of infection under immune pressure, these same variable loop insertions may reduce CD4 receptor or CCR5 co-receptor access, and be disfavored at transmission [6], [36]. Our third motivation was the evidence for phenotypic trait selection at transmission: Viruses isolated during acute infection almost exclusively use the CCR5 co-receptor, while during progression HIV-1 can utilize different co-receptors, most commonly CXCR4 [17], [23], [26], [37]. In addition, cloned early viruses replicate efficiently in activated human CD4+T cells, but not in monocyte-derived macrophages [23], [26].

Here we performed a search for amino acids in Env sequences to discern patterns in amino acid substitutions (signatures) that were statistically associated either with transmission or with frequent recurrence across individuals during viral diversification in the chronic phase of the infection. We based our analyses on thousands of sequences from several hundred subjects (summarized in Table 1, with subjects individually described in Table S1). The analyses involved a series of strategies to identify signatures in single sites or sets of functionally related sites. By putting the signatures in a structural, functional, and immunological context, we then discuss what is known about the sites and the protein regions they are embedded in, to raise hypothesis regarding their possible modes of action.

**Table 1:** Number of subjects and SGA sequences used in this study.

Dataset	Stage	Total Number	
		Subjects	Sequences
Original	Early	48	1340
	Chronic	43	892
Holdout	Early	43	1375
	Chronic	43	1230
Plasma Donors	Early	44	1466
LANL Database	Chronic	54	760

## Results

### Sequence data

All sequences were derived using single genome amplification (SGA) methods [38] from individuals with sexually transmitted subtype B infections. We assembled as many well-characterized samples as we could that met these criteria, with contributions from many groups, with the goal of making this study as well powered as possible. Most samples were collected within the United States, with a subset from Trinidad. The demographic and clinical information relating to the subjects and samples are described in Supplement Table S1. Sequences were separated into two data sets: the ‘original’ hypothesis-raising set, and the ‘holdout’ hypothesis-validating set. Data sets were matched as described in the methods. In a second series of hypothesis-forming analyses, to increase our sample size and statistical power, we also generated a third set of sequences from acute/early infections, from infected plasma donors, and added additional sequences reported to be sampled during chronic infection from the Los Alamos database, and combined them with the original set.

### Analyses strategies

We performed a series of exploratory tests to identify signatures that were significantly associated with Env protein sequences from either viruses sampled in early infection or viruses collected during chronic infection. We used an approach that accounts for the non-independence of the sequences due to phylogenetic relationships and adjusts for multiple tests (see the results and methods sections for more details) [39]. By “signature” we mean a mutational pattern that compared to expectations from unselected inheritance either (i) is enriched among the early virus, or (ii) recurs in chronic infection and yet is rare among the early variants,. We began with a search for statistically significant enrichment of single amino acids found at each position in the Env alignment. We next grouped small sets of alignment positions based on their contribution to a potential N-linked glycosylation site (PNLG) motif, membership in an inferred functional domain (functional groups), or spatial proximity defined using structural models (contact sets). We then systematically looked for signatures based on combinations of amino acid changes within these three groups, enabling us to identify additional patterns that were significantly different between early and chronic sequences.

The first approach we used tested for correlations between early versus chronic status and the amino acids found in the consensus sequences derived from individual patients, using the same methods as we have used previously [39], [40]. A consensus sequence represents the most common amino acid found at each alignment position within an individual. Consensus sequences from homogeneous early infection cases generally correspond to the modeled transmitted virus [17], [18]. The second approach we used included all sequences from each subject, modifying our earlier published methods to enable inclusion of multiple sequences per subject, as illustrated in Supplement Fig. S1. Fig. S1 shows the phylogenetic tree based on all of the available data, highlights characteristic phylogenetic patterns from examples of early and chronic infection, and illustrates the strategy we used to incorporate all sequences from every subject into the signature analysis. We initially required associations both be statistically supported in the “test” data set with a q-value of <0.2, and that they show a consistent association in a separate analysis in the “holdout” data test set. A q-value is a false discovery rate [41] that adjusts for multiple tests, critical in this study as thousands of tests were conducted. We chose a relatively high q-value cut off in our initial analysis; thus we expect approximately 20% of our sites from our first round of analysis to be by chance. We then used then conservative strategy of requiring validation in a completely separate holdout set to minimize false positives (Type I errors). This was very stringent, and we only found a small number of signatures. Therefore, we subsequently did an analysis combining data from all subjects, test and holdout and plasma donors, using a cross-validation strategy to test the statistical robustness of the observed signature sites. This provided an alternate view of the data that minimizes false negatives (Type II errors).

### Identification of a signature at position 12 in the Envelope signal peptide

Using just the consensus sequences from each subject, only one signature amino acid at position 12 in Env was identified through an analysis of all amino acids found at each single alignment position in Env in both the test and holdout sets. Mutating away from His at position 12 (expressed here as !H12) was statistically enriched in chronic viruses, while a stable His was enriched in early viruses (p = 0.001, for details see Table 2). The distribution of amino acids at position 12 for each subject is shown in Supplement Fig. S2A. H12 is the most common amino acid among both early and chronic viruses, but it was enriched among early sequences. This was true for the within-subject consensus sequences (74% in early versus 57% in chronics were His), as well as all of the natural sequences (3114/4181, 74%, of early sequences were His, as compared to 1150/2122, 54%, of chronic sequences). Thus H12 is enriched among early infection relative to chronic sequences (odds ratio = 2.5, 95% CI 2.2–2.8, Fisher's p<2×10^−16^). However, as demonstrated in Bhattacharya et al. [39], a simple analysis testing for enrichment can be profoundly biased by lineage effects, as sequences are not independent but related by shared phylogenetic history. Thus without a phylogenetic correction even such apparently strong associations should be viewed with caution. In the case of the !H12 chronic signature we have such support (Table 2), and in all of the other signature identification strategies employed here (Tables 3–5) we have used a phylogenetic correction.

**Table 2:** Summary statistics for the only single-site signature found in Env based on within-subject consensus sequence analysis, His at position 12.

Data Analysis	HXB2 Pos	Align Pos	Original		Holdout		Fiebig stage	Direction	Change Early	Stasis Early
			p-value	q-value	p-value	q-value			Change Chronic	Stasis Chronic
Consensus Tree	12 H	12	0.001	0.07	0.12	0.30	F1–F5	H ->!H	2	35
								chronic	13	21
Full Tree, strong	12 H	12	4×10^−9^	9×10^−8^	9×10^−5^	0.0005	F1–F5	H ->!H	8	67
chronic signatures								chronic	57	54
Full Tree	12 H	12	8×10^−5^	0.0007	0.08	0.19	F1–F6	!R ->R	2	14
								chronic	20	6
Full Tree	12 H	12	1×10^−5^	0.0002	ns	ns	F1–F6	!P ->P	0	91
								chronic	20	127

The full tree analysis and summary of common changes in position 12 support this signature, and are also provided. The direction indicates the signature amino acids, and **H** ->!H is read as H changes to “not His” (i.e. any other amino acid). The Fiebig stage indicates the group included in the comparison that gave the p-value shown. For example, F1-F5 means that Fiebig stages F1–F5 were included in the early group, and the p-values for this set are given, as they have the lowest p- and q-values. Five increasing inclusive levels of Fiebig stages were compared, however; all 5 groupings of Fiebig stages had a trend indicting support of this signature, although not always meeting the q-value threshold. The contingency table on the right of each row indicates the number of times the ML tree indicated a change between the ancestral state immediately preceding the consensus sequence, versus when the amino state did not change. Thus H is enriched among transmitted variants. In the consensus tree, it mutates away from H in only 2/37 times in acute/early, versus and 13/34 times in the chronic cases (5% in acutes versus 38% in chronics). In the full tree including all of the sequences, the distinction was similarly pronounced, changing 8/75 in acute cases and 57/111 in chronics (10% versus 51%). H most frequently mutates to R or P during the course of an infection; changes to P were statistically not supported (ns) in the holdout set.

We did not see significant increases in changes *towards* H12 in early Envs when using a phylogenetic correction, only the reciprocal signature, *away from* H12 (!H12), in chronics. This could be because these two tests, both based on frequencies of changes from ancestral states, not just simple counts, had different powers in our dataset. The statistic that captures inferred H12 to !H12 changes in the phylogenetic tree in chronic infection was powered by H being the most common amino acid in this position, and so the most commonly inferred ancestral amino acid. In contrast, a statistic looking for changes towards H12 in early sequences required the relatively rare !H12 as an ancestor. In other words, we were statistically better powered to see changes away from His in chronics than towards His in early infection, and this simple explanation may account for the lack of significant association with changes towards H in early infection despite a high level of significance for !H12 in chronic infection.

### Identification of a transmission signature at position 415, near the CCR5-binding site

**Figure 1:**
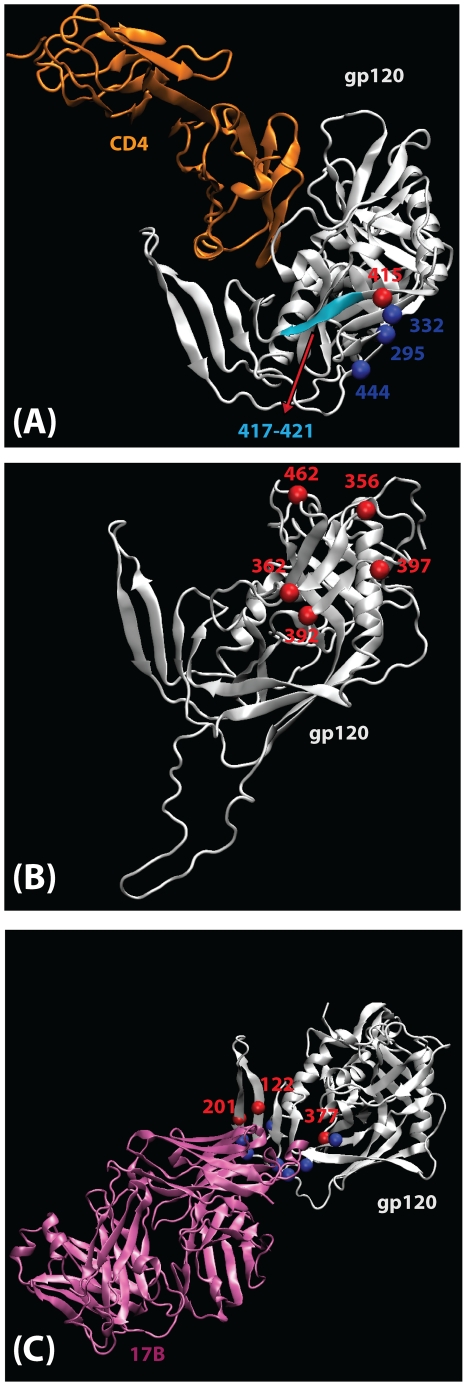
Mapping of signature sites (red) on the three-dimensional structure of gp120 (silver). A ribbon structure of the HIV-1 gp120 core +V3 in the CD4-bound conformation is shown in white. (**A**) Key residues involved in co-receptor and antibody (2G12, b12, b13 and F105) binding that are proximal to the position 415 are shown. Residues 295 and 332, that contribute to the 2G12 epitope, and residue 444, that is important for co-receptor binding, are shown as blue balls. A motif spanning the region 417 to 421 (cyan color) that is proximal to position 415 and contains residues that take part in binding to coreceptor (419), b12 (417–419), b13 (419–421) and F105 (421). CD4 (orange) is shown for better visualization of receptor binding site region. (**B**) Locations of signature patterns involving glycan motifs (N-notP-[ST]). (**C**) Spatial locations of signature sites within a set of functional sites (blue) associated with CCR5 binding. The 17b antibody Fab is included to mark the region in gp120 that takes part in CCR5 binding. Signature sites are labeled with HXB2 reference numbers.

After detecting only a single signature in our first analysis of consensus sequences, we were concerned that we did not have adequate power to detect potentially important but subtle signatures. Thus, to improve our power in the hypothesis-raising context, we extended our original data set with the set of samples from acute and early infection plasma donors, and a set of chronic samples from the Los Alamos database (www.hiv.lanl.gov); our holdout set remained the same (Table 1). A factor complicating our analysis was that although 80% of early patients were productively infected with only one HIV-1 strain, the rest were clearly infected by multiple transmitted viruses. Given that this latter group might have multiple transmissions because of a less restricted transmission bottleneck, we next analyzed only the subset of the early infection cases that were established by a single virus [17]. When one consensus sequence per patient was analyzed after excluding heterogeneous acute infections, a signature pattern of not having a Thr at HXB2 position 415 (!T415), was found to be enriched in acute infection samples (Table 3). This position is part of a PNLG sequon at N413, lies at the end of the flexible part of the gp120 V4 loop, and is in the conformationally conserved part of the outer domain. It is structurally proximal to three regions of potential interest: the binding site of several CD4-binding site (CD4bs) antibodies (Fig. 1a) [42]; two sites that have been implicated in co-receptor binding by mutational studies, positions 419 and 444 [43], [44]; and two key residue for mannose addition for the 2G12 epitope, N295 and N332 [45], [46]. We therefore checked if there was a correlation between the presence or absence of T415 and neutralizing antibody (NAb) IC50 scores that were available for a set of SGA-derived pseudotyped Envs (Table S3). !T415 (Envs lacking the PNLG) was associated with increased b12 neutralization sensitivity (p = 0.0001, Wilcoxon rank test). In contrast, neutralization by sCD4 was not significantly correlated with the !T415 signature (p = 0.2756, Wilcoxon rank test). Detectable neutralization by the CD4-inducible (CD4i) monoclonal antibody 17b, or by 17b with sCD4, was extremely rare in this dataset and observed only 3/113 times. In all three cases, a T415 was present, suggesting that its presence did not inhibit access to the 17b binding site, but this result was not statistically significant. Finally, this site was not significantly correlated with neutralization susceptibility to monoclonal antibody 2G12, which critically depends on other nearby PNLG sites in Env [45], [46].

**Table 3 ppat-1002209-t003:** Summary statistics additional signatures identified with additional searches, using the combined original and PD/DB sets to identify potential signatures and comparing to the holdout set. For legend see table 2.

Data Analysis^a^	HXB2 Pos	Align Pos	Original+PD/DB		Holdout		Fiebig stage	Direction	Change Acute	Stasis Acute
			p-value	q-value	p-value	q-value			Change Chronic	Stasis Chronic
Homogeneous	415	525	0.003	0.40	0.05	0.11	F1–F2	T ->**!T**	14	30
Early, consensus								**acute**	9	78
Full tree, strong chronic signatures	397	487	3×10^−11^	5×10^−9^	1×10^−9^	6×10^−8^	F1–F4	N ->!N	6	146
									66	154
Full tree, strong chronic signatures	399	489	5×10^−11^	5×10^−9^	3×10^−6^	3×10^−6^	F1–F6	T ->!T	17	184
									77	148
Full tree, strong chronic signatures	362	445	6×10^−11^	1×10^−8^	1×10^−8^	1×10^−6^	F1–F2	N ->!N	11	82
									130	138
Consensus Tree	Ref 1	Ref 2	0.007	0.23	0.01	0.28	F1–F5	L[IV]---N ->	0	36
CCR5 model set^b^								!L[IV]---N	8	35

a One new acute signature site was identified through restricting the search to just the homogeneous early infection samples, !T415. This association was significant only for a grouping of the earliest samples, from Fiebig stages 1 and 2. Three sites in addition to site 12 (already included in Table 2) were strongly supported signatures of recurrent change in the chronic subjects using full tree analyses. One combination of sites was found through more intensive examination of the functional domain sets. It was found in the CCR5 CoRbs model, defined based on a heavy-atom based distance criterion to identify the proximal amino acids to the CCR5 CoRbs.

b Region explored for Ref-1 HXB2 amino acid and positions, complex signature positions in bold; Ref-2 refers to the alignment position given in parenthesis. Q114 (133), **L122 (141),** **I201 (271**), Q203 (273), A204 (274), S209 (279), **N377 (463**), Y384 (470), A436 (546) and P437 (547)

### Analysis combining consensus data from all subjects using cross-validation

In a hypotheses-raising framework, we also did an exploratory signature test on consensus sequences across all positions, combining the subjects listed in Table 1 to further increase our power. For this analysis we compared consensus sequences representing the 135 acute or early infection subjects to the 86 chronic infection subjects sampled and sequenced through this project. To further minimize Type II error and be inclusive in a hypothesis-raising framework, a liberal q-value of 0.5 was used. As stated above, this analysis, with a larger N but without a strict separation of hypothesis generating and validation sets, is not as statistically robust as the original analysis with a distinct validation set. We used a stratified 10-fold cross-validation test as an assessment of the robustness of the predictor. Ten potentially interesting signatures were identified with this strategy, including continuing support for the signatures !H12 and !T415 with a range of cross-validation support, with the signature at position 12 yielding a high degree of support (Table 4). 2 of these 10 associations were early infection signatures (!T415 and F712), the other 8 were chronic. We also performed an additional 10-fold cross validation analysis to reduce the possibility that the observed signatures were the result of an alignment artifact (see methods for alignment details). Our primary alignment for our original analysis was created using the Genecutter alignment tool coupled with a HMMER model [47]; we we repeated the procedures on a second distinct alignment generated with the alignment program MAFFT [48], [49]. 8/10 of the signature sites defined using the HMMER alignment were also found in the MAFFT alignment; the two that were not found in the MAFFT alignment also had only low level support in the cross-validation test.

**Table 4 ppat-1002209-t004:** Signature hypotheses raised based on analysis of all within-subject consensus sequences.

HXB2 Pos	Align Pos	p value mafft	p value hmmer	q value	OR	Cross Validate train	Cross Validate holdout	Fiebig stage	Direction	Change Early	Stasis Early	Region
										Change Chronic	Stasis Chronic	
12 H	12	0.0067	0.0039	0.46	0.39	8	6	F1–F5	H to !H	19	108	Signal peptide
									chronic	38	85	
192 K	262	0.0005	0.0029	0.28	0	10	9	F1–F3	R to !R	0	86	V2
									chronic	11	107	base
309 I	381	0.0006	0.0010	0.29	0.27	6	2	F1–F4	I to !I	9	83	V3 near tip
									chronic	35	88	
415 T	525	0.0100	0.0031	0.48	3.35	6	6	F1–F2	T to !T	18	43	V4 PNLG
									early	14	113	
446 V	556	0.0010	0.0010	0.40	0	4	3	F1–F6	!V to V	0	145	PNLG
									chronic	9	121	
455 T	565	0.0019	0.0014	0.23	0	6	6	F1–F4	T to !T	0	103	V5 CD4bs VRC01
									chronic	12	117	
543 Q	681	na	0.0047	0.42	0.14	3	3	F1–F6	L to !L	2	37	gp41
									chronic	13	32	
700 A	851	na	0.0064	0.43	0.21	0	0	F1–F4	A to !A	4	50	Trans-membrane
									chronic	16	42	
703 S	854	0.0200	0.0033	0.37	7.51	2	0	F1–F4	S to !S	11	93	Cytoplasmic tail
									chronic	2	128	
721 L	873	0.0002	0.0005	0.14	8.39	1	0	F1–F2	!F to F	11	54	Cytoplasmic tail
									early	3	125	

Consensus sequences from each subject from all three sets (Table 1, main text) were combined in a hypothesis-raising context (the Test set “All con”). 2 acute signatures were observed (in bold): selecting for a loss of T in acutes at position 415 (discussed in the text), and selecting for F at 721. **Key: HXB2 Pos:** the HXB2 Env position and amino acid. **Aln Pos:** The corresponding position in the Env protein alignment. **Sig AA:** The signature amino acid. **Test set:** “All con” was based on comparing acute and chronic data using a consensus from each patient and combining all three datasets described in Table 1 in the main text. We raised the q value threshold to 0.5 for this exploratory summary, so we could identify a few potentially interesting sites; only half would be expected to be of interest. “Original” are the six sites for which a signature hypothesis was raised based on the original data; only position 12 H was later supported in the holdout data, so it is discussed further in the main text and was subsequently experimentally validated to regulate expression levels. Here we used our standard q threshold of 0.2. **Pattern:** “A to !A” means the signature amino acid is predicted in the maximum likelihood tree to be A in the most recent ancestral node of the subject, but to have changed to not being the signature amino acid (“!A” means “not A”) in the subject. This change contrasted to the signature amino acid remaining the same in the contingency table (The signature amino acid A it found in the recent ancestor and the leaf node). “!A to A” is the inverse situation where the ancestral state is not the signature amino acid. **FS:** Fiebig Stage.

### Identification of signatures using all sequence data from individuals

We also systematically explored the complete Env glycoprotein using all available sequences from individuals, not just the per-individual consensus sequences. To do this, the sequence at the node *preceding* the ancestral node within each subject in the reconstructed phylogenetic tree was estimated by maximizing the marginal likelihood [39], [50], and the number of times each ancestral amino-acid was estimated to have changed between that node and the sampled sequences within each subject was calculated (See Fig. S1 for an illustration of the strategy). As with our first exploration of the consensus sequences we validated the results from the test data with the holdout data. Position 12 was again found to commonly mutate away from H, most often to R or P, during chronic infection (Fisher's exact p-value of 4×10^−9^, Table 2). Although we found changes specifically associated with early signatures at a number of positions in the original test set, none of these associations were also supported in the holdout validation set. In contrast, many chronic signatures (specific changes found repeatedly in chronic patients) were supported in both the test and holdout sets. 25 signature patterns were found that were indicative of recurrent change during chronic infection, using the criteria of q<0.2 in the test set and q<0.3 in the holdout set; these signatures are listed in Supplement Table S2. Interestingly, 8 of these 25 chronic signatures, including !H12, were found in either the signal peptide or the cytoplasmic tail, supporting the possibility that modulation of Env expression levels may play a role in selection at transmission, and lowered Env expression levels may be important for immune evasion during chronic infection.

**Figure 2:**
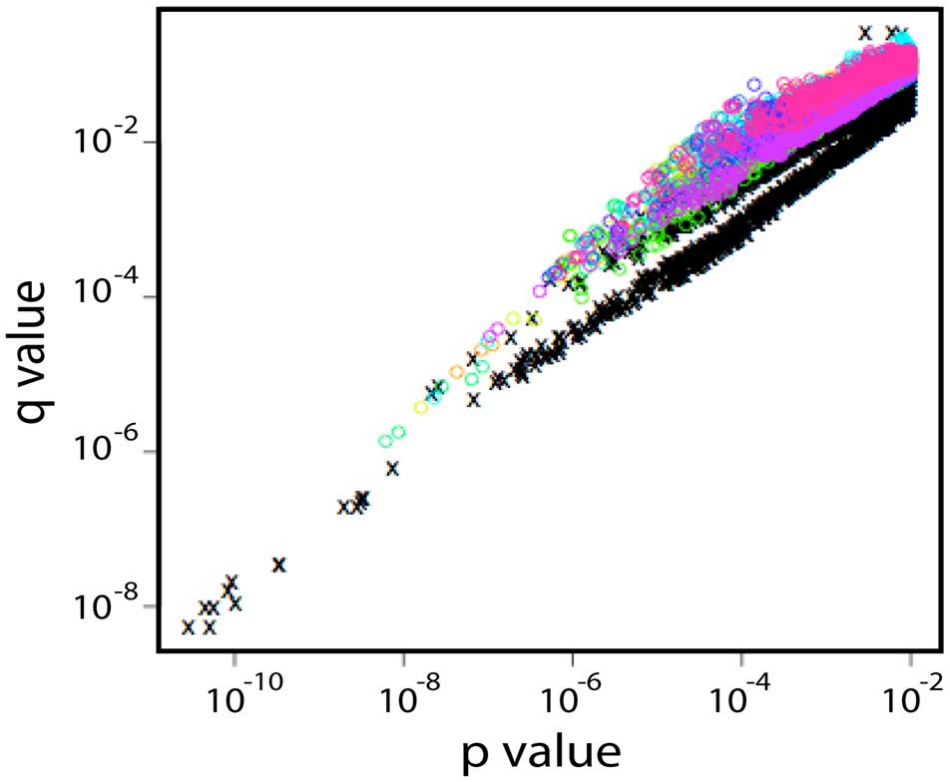
p- and q-values found in shuffling experiments in which the entire sequence signature strategy was repeated 10 times after randomizing the early and chronic designation of each subject. The black x's represent the distribution of p- and q-values in the real data, while the colored circles represent the findings for incremental inclusion of Fiebig stages 2–6 in shuffled data. The lower quadrant of part of the graph is almost exclusively occupied by the real data, indicating a signature dependent on early versus chronic status; p-values of less than 10^−6^ were rare in the randomized data, and value less than 10^−8^ were exclusively found among real data classifications.

The interpretation of chronic signatures identified by analyzing the full-sequence alignment, not just one sequence per person, is complicated by the fact that chronic sequences are inherently more heterogeneous, and hence display more changes than acute sequences, and we can not distinguish between associations arising due to repeated mutations in a small number of very complex chronic infections, and a pattern repeated across multiple patients. Thus we did one further computational experiment to help interpret our observed levels of significance. Since we were interested in identifying recurring patterns across multiple patients, we performed a shuffling test where we randomized the acute/chronic classification categories and redid the signature analysis 10 times (these analyses are extremely computationally intensive, so it was only feasible to do 10 such randomizations for this study). This randomization should maintain significance if it arose as recurrent pattern that was distributed across many distinct infections, but would remove the signal if it was an anomaly resulting from a single or very small set of complex patients. The results of this re-sampling experiment showed that while low p-values did indeed occur even after randomization, p-values of less than 10^−8^ were not found in the analyses of these randomly classified data (Fig. 2). Four of the chronic mutational signatures were found to both be significant in the test data with p-values of less than 10^−8^, and also were supported in the holdout data: !H12, !N397, !T399, and !N362 (Tables 2 and 3). Thus, these 4 signatures were singled out as being the most robust. Like the consensus signature analysis, the full tree signature captured the !H12 chronic infection signature (Table 2). Two additional full tree chronic signatures at position 12 shown in Table 2 (12R and 12P); they represent the most common amino acid substitutions in position 12 as it mutates away from His. The other three robust chronic amino acid signature patterns all impact PNLGs: positions 397 and 399 are part of the same PNLG, and 362 is in a PNLG in the C3 region.

Next, associations between the presence or absence of intact PNLG motifs with early versus chronic sequences were examined. Glycans can play an important role in immune escape and immunogenicity, can contribute to transmissibility and impact cell entry [51], [52], [53], and several of the single site signatures already described are part of PNLGs. We identified six PNLG motifs (N-X-[ST], where X is any amino acid other than Pro) that were significantly associated with a repeated pattern of loss during chronic infection (Table 5). These PNLGs spatially mapped on an X-ray structure of gp120 are shown in Figure 1b. The per-subject frequency of one of these patterns, the PNLG motif at position 397–399, is illustrated in Supplement Figure S2B–the PNLG at position 397 was conserved overall (Fig. S2B), although it was more likely to be present early in infection (Table 5, q-value = 3×10^−10^ in the original data, 0.0001 in the holdout data). One of the PNLG signatures, that enables glycosylation at position 392, is part of the monoclonal antibody 2G12 epitope [45], [46], [54]. Experimental data from Nab IC50 scores 2G12 from 113 clones representing SGA clones from early transmission cases (Table S3), and confirmed that the glycosylation motif at position 392 was highly correlated with 2G12 neutralization (p = 0.006, Wilcoxon rank test).

**Table 5:** Summary statistics using the combined original and PD/DB sets and holdout set to the gain or loss of PNLGs, defined as the motif NX[ST], where N is Asp, X is any amino acid besides Pro, and [ST] is a Ser or Thr.

HXB2 Pos	Align Pos	Original+PD/DB		Holdout		Fiebig stage	Direction
		p-value	q-value	p-value	q-value		
397	487	2×10^−11^	3×10^−10^	9×10^−5^	1×10^−4^	F1–F4	Recurrent loss of potential N-linked glycosylation sites during chronic infection
362	445	6×10^−7^	6×10^−6^	0.02	0.02	F1–F6	
356	438	1×10^−7^	6×10^−7^	0.002	0.002	F1–F6	
392	478	1×10^−5^	6×10^−5^	2×10^−5^	3×10^−5^	F1–F3	
462	576	1×10^−5^	6×10^−5^	3×10^−11^	2×10^−10^	F1–F4	
188	249	1×10^−5^	8×10^−5^	3×10^−^	7×10^−5^	F1–F4	

### Identification of a complex signature near the CCR5 Coreceptor-binding site (CCR5 CoRbs)

Clearly, analysis of single amino acid positions may miss complex mutational patterns in functionally or conformationally important regions. Given the vast number of combinations of alignment positions and the range of different amino acids at each position, we are limited in our ability to look at arbitrary combinations of sites and amino acids across the full Env sequence, , due to multiple test issues and limited power due to sampling constraints (Table 1) compounded by computational feasibility. Thus, we performed a focused in depth exploration for signatures based on a small number of combinations of sites, including only amino acids within narrowly defined sets of functionally related sites [3] (Table S4). How extensively we searched combinations of sites within these sets was determined dynamically as described in the methods; however, at a minimum, all combinations of up to 3 amino acids at each of 2 positions were searched within each functional region, using a sliding window approach to span different amino acid subsets and combinations within each functional domain. These functional regions included: the CD4bs in gp120; the CCR5 CoRbs region in gp120; positions known to impact R5/X4 tropism; a subset of the V3 loop positions; the b12 binding site in gp120; residues predicted to reside at the gp120/gp41 trimer interface; the gp120 V2 region implicated in binding the gut homing receptor; 2F5/4E10 binding sites in gp41; the lentivirus lytic peptide LLP1 and LLP2 regions of the gp41 cytoplasmic domain; and sites that have been related to membrane fusion, including sites in which changes were shown to result in increased or decreased entry (see Table S4 for positions included). Despite this extensive search, only one statistically significant association with a complex signature was identified and validated in both the test data and holdout data; it was found in a CCR5 CoRbs set and the signature was defined as: L122-[IV]201-N377, with repeated mutation away from this pattern in chronic samples. The statistical summary of this signature pattern is given in Table 3, and the spatial locations of these sites are mapped on gp120 in Fig. 1c. The CCR5 model set contains residues that are proximal to the highly conserved critical residues that take part in the binding to CCR5, but that are clearly amenable to positive selection since they are variable at the population level.

### Biochemical patterns in structure-based regional clusters

In our final exploration of this data, we searched for early infection or chronic signatures defined by changes in amino acid chemistry in spatially defined local regions. Our reasoning was that transmission signatures would not necessarily have to involve particular amino acid substitutions at a single site or a collection of sites, but rather might reflect a complicated amino acid substitution pattern that could in turn affect the structure or chemical nature of specific spatial regions within the Env structure. Such regional changes may impact expression or binding to receptors and antibodies. To explore this possibility, we first defined 395 contact sets of spatially defined clusters structurally centered on the amino acids included in the X-ray structure of the gp120 core from the YU2 strain [55], as described in the methods. Each set contained a up to10 amino acids that were less than 10 Å from the center amino acid of the contact set, based on all-atom molecular dynamic simulations. To capture the effects of dynamic interaction between flexible and core regions, no distinction was made for surface residues.

**Figure 3:**
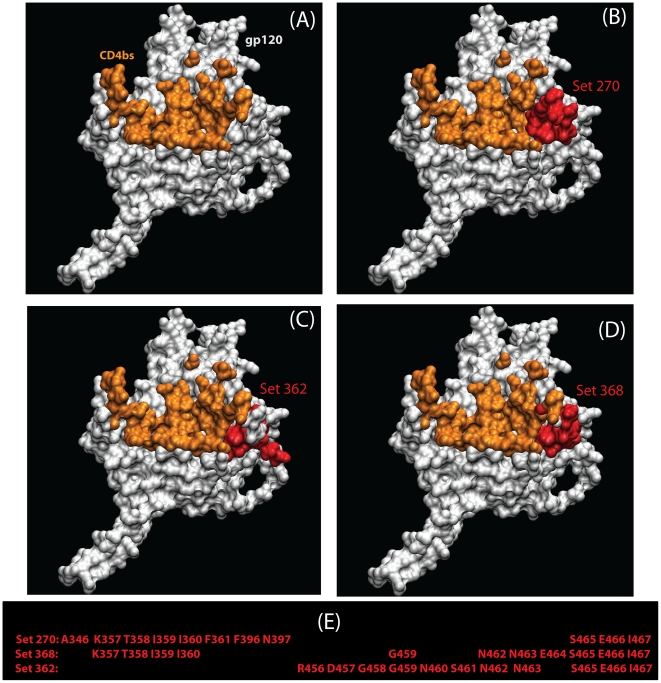
Three statistically significant structures-based regional clusters in gp120 (white) associated with changes in polarity. These regional clusters occur near the CD4-binding site (orange) shown in (**A**). The CD4-bound conformation of the HIV-1 gp120 core+V3 is shown, from the perspective seen by CD4. The three clusters (**B–D**) are shown in red. The residues that form these sets are shown in panel (**E**). All maps are based on HXB2 numbering.

It was not feasible to analyze all neighborhood lists with all combinations of explicit amino acid transitions, so we simplified the data by calculating a regional additive polarity score for the amino acids in each neighborhood cluster (see Methods). Unlike the discrete change-stasis nature of the variables (acute versus chronic) used for the other signature analyses in this study, this score was a continuous variable, so we used the method of phylogenetically independent contrasts [56] to identify changes in polarity that correlated with early or chronic infection sequences. Three statistically significant regions were identified (Table 6), and mapped on the three-dimensional structure of gp120 (Fig. 3). In all three regions, the region became more polar during chronic infection. All three sets have amino acids that share or border the binding sites of CD4, and b12 [57], [58]. The polarity scores did not correlate significantly with sCD4 or b12 neutralization when compared the with experimental binding data (Table S3). Sets 270 and 368 border the highly conserved CD4 binding loop region (HXB2 positions 364–373). Sets 362 and 368 consist of additional residues from β23 strand and V5 loop region that take part in binding to CD4 and b12. All three sets shared a three amino acid segment (465–467) that constitutes part of the binding site for the potent broadly neutralizing monoclonal antibody VRC01 [57], [58].

**Table 6:** Summary statistics regarding changes in regional hydrophobicity associated with chronic infection.

Data Analysis	Set number^a^	Original+PD/DB			Holdout		Correlation Coefficient Original	Correlation Coefficient Test	Direction
		p-value	q-value		p-value	q-value			
Change in Polarity	270	1×10^−12^		1×10^−11^	0.04	0.01	0.64	0.18	Chronic sets are more polar
	368	1×10^−6^		1×10^−5^	0.05	0.01	0.47	0.18	
	362	1×10^−4^		1×10^−3^	1×10^−4^	1×10^−3^	0.38	0.34	

a Sets of amino acids including in the three statistically interesting regions. These tests compared sequences from all Fiebig stages, F1–F6, to chronic samples.

**Spatial Region 270**: I359,T358,I360,E466,N397,K357,F396,S465,A346,I467,F361.

**Spatial Region 368**: S465,E466,E464,T358,K357,N463,N462,I359,I467,I360,G459.

**Spatial Region 362**: G459,G458,N460,D457,S461,N462,E466,I467,R456,N463,S465.

### Hypervariable loop length and number of glycosylation site differences between acute and chronic samples

We tested whether the hypervariable regions V1–V2, V4, or full gp120 revealed a pattern of reduced loop length or number of PNLG sites in the acute/early samples relative to the chronic samples, as would be expected from the literature [59]. When we compared the distributions of all of the within-subject Env consensus sequences in the acute/early versus chronic subjects, fewer PNLG sites overall were found in gp120s from early infection (p = 0.008, Wilcoxon signed rank test). There was also a trend towards fewer PNLG sites in the V1V2 loops (Wilcoxon p = 0.03), as well as a trend toward reduced V4 loop lengths ((Wilcoxon p = 0.03).

### Signature analyses methods that did not incorporate a phylogenetic correction

Several other strategies were employed to look for signatures among the sequences by treating the samples as independent, and not accounting for phylogenetic relationships [60]. These methods did not yield any consistent signature patterns between the hypothesis-forming test (with a q-value of <0.2) and hold-out sets (with a q-value of <0.3), although additional support for a signature at position 12 was observed; these methods and results are fully summarized in the Supplement (Text S1, Figs. S3, S4, S5, S6, S7 and Table S8). In these analyses, a lack of concordance between the hypothesis forming and test-sets could arise as a consequence of a lineage effect dominating the signal in the hypothesis-forming set; alternatively, the subjects and sampling may have been too dissimilar to reproduce subtle effects.

## Discussion

In this study we performed a comprehensive analysis of HIV-1 Env sequences to identify signature patterns in proteins that are significantly different in chronic versus early sequences. Here we focus on interpreting the strongly statistically supported signature patterns in the context of what is known about the biological role of these sites.

### Signature sites in the signal peptide and cytoplasmic domain

It was intriguing that among the 25 significant signatures identified upon combining all of the data (Table S2), 3 were located in the signal peptide of gp160, and 4 in the cytoplasmic domain. The recurrence of patterns of mutational change in these two regions during chronic infection raises the possibility that they may indirectly influence immune evasion by altering Env protein folding, modification or expression levels. The signal peptide directs Env in its co-translational translocation to the endoplasmic reticulum (ER), where it undergoes further folding, glycosylation, and trimerization [61]; it may also serve as a gatekeeper for the release of correctly folded proteins [62]. It is unusually long (30 amino acids on average), and contains a number of highly charged residues in the N-terminal region [63], [64] spanning position 12, one of our most robust signatures (Table 2). Signal peptides play a role in the efficiency of the protein secretion and in orienting proteins in membrane, influence folding and the exit from the ER [65], [66], and can impact cleavage rates [63], [67]. A slower cleavage rate down-regulates the rates of folding, intracellular transport and secretion [63], [65], [68], [69].

The Env cytoplasmic domain of HIV-1 is also unusually long; at 150 amino acids long, three times longer than that found in typical lentiviruses [70]. It contains three helical fragments called lentivirus lytic peptides (LLPs) [71] that have been implicated in cell surface Env expression [72], [73], incorporation into virus particles [74], [75], fusogenicity [76], [77], and Env's localization in lipid rafts [71]. The chronic infection signatures in the cytoplasmic tail (Table S3) are all concentrated on the LLP-3 segment. This segment has a strong potential to associate with and perturb the membrane [78], and a di-aromatic motif of Y802 W803 in this region has been associated with retrograde transport of Env to the trans-Golgi network [79].

### The acute signature site at position 415

!T415 was strongest early sequence signature observed, indicating that the PNLG at 413–415 is selected against at or immediately after transmission. This PNLG is glycosylated when present [80], and is located near the C terminal end of the V4 loop, proximal to both the CCR5 CoRbs and the CD4bs regions that impact both antibody access (Fig. 1a). A highly conserved sequence motif that takes part in CCR5 binding, RIKQ (HXB2 419–422), is just a few residues upstream [43], [44], [81]. The conserved sequence motif PCR (HXB2 417–419) that participates in the binding to monoclonal b12 is also in the neighborhood of this site [58], consistent with our finding that the presence of the PNLG motif at 413–415 is highly correlated with reduced b12 susceptibility. The glycosylation site at 413–415 has repeatedly been singled out as a relevant immune escape site in recent neutralizing antibody studies. Acquisition of a PNLG at 413–15 has been demonstrated to confer escape from autologous antibodies in longitudinal studies of the trajectory of escape in both an HIV-1 infected person (David Montefiori, personal communication), and in a rhesus macaque infected with SIVmac239 [82]. Furthermore, this region in association with the C3 α-2 helical domain is thought to contribute to patterns of neutralization susceptibility [83], [84], [85].

Two studies have found the presence of a glycosylation site 413–415 to be associated with virus isolated from individuals capable of eliciting potent or broadly neutralizing antibodies [40], [86]. This correlation was proposed to either result from a recurrent pattern of escape in people who make potent broad neutralizing antibodies, or as common feature in Envs able to elicit good antibodies [40]. We have tested a strain that has the glycosylation site at 413–415 present (strain CH0219), isolated from an individual who had made very potent broadly neutralizing antibodies in response to infection [40]. This Env was resistant to autologous antibodies in sera from CH0219, supporting its role in antibody escape. Furthermore it was found to be an extremely poor immunogen for eliciting neutralizing antibodies in guinea pigs (BFH, unpublished data). These findings are consistent with the intuitive hypothesis raised by our current signature analysis, that the addition of a glycosylation site at 413–415 provides a common escape mechanism during chronic infection by blocking access to a key epitope, but that it is selected against in early viruses, resulting in the observed !T415 signature pattern.

### Implications of the repeated patterns of loss of glycosylation motifs during chronic infection

Changes in glycosylation play a key role in chronic infection, and either the gain or the loss of a particular glycosylation sites can both result in immune escape [32], [87]. As discussed earlier, reduced loops lengths and numbers of PNLGs are characteristic of early viruses, and although the pattern can be subtle and difficult to discern in the B subtype [34], [35], we did find supporting evidence for an overall pattern of reduced numbers of PNLGs after transmission in this data set; this reduction in PNLG sites occurs in the hypervariable loops. In contrast, most of the specific signature PNLGs we have identified are clustered in the outer domain, and these are lost not at transmission but in the course of chronic infection (Fig, 1b). The statistical counterpoint to the chronic loss-of-glycosylation-motif signatures is relative conservation of these PNLG sites at transmission, consistent with a scenario that these specific sites facilitate transmission in early infection, and their loss contributes to immune escape in chronic infection.

Several of the signature PNLGs have known functional roles which support the scenario described above. First, the glycan at N188 facilitates interactions with CD4 and CCR5 [88], and the loss of glycosylation sites in this region have been associated with diminished replicative capacity [55], [57], [58], [89]. Changes in this region have also been associated with immune escape from some of the first neutralizing antibodies in natural infection [33], [85], and a glycan knock-out at position 188 impacts the neutralization potency of the recently isolated broadly neutralizing antibodies PG9 and PG16 [90]. Thus selection for the glycan may occur at transmission, and selection away from in during immune escape from antibodies similar to PG9 and PG16. Similarly, N362 has been shown to contribute specifically to enhanced fusogenicity [91], a property that might be favored during transmission. PNLG 362 and 462 are near the CD4bs, and the b12 and VRC01 monoclonal antibody binding sites [55], [57], [58], and the CD4bs is a common target of neutralizing antibodies in natural infection [92], [93]. Finally, the PNLGs at positions 392, 397 and 356 are all part of the “silent face” of gp120 [94], [95]. The oligomannose glycans that are clustered on the silent face of HIV are ligands for DC-SIGN, a lectin found on the surface of dendritic cells [96]. Dendritic cells encounter HIV soon after mucosal exposure [97], and may have a role in enhancing the efficiency of HIV transmission [88], [91], [98]. A mannose at position 392 is also a critical component of the epitope of the neutralizing antibody 2G12 [45], [54], and our data confirm this previously well-established relationship. Although the 2G12 epitope may not be a common a target of neutralizing antibodies in natural infection [99], antibodies to the 2G12 epitope in neutralizing sera have been found in long-term non-progressors [100], suggesting the glycan shield at the silent face of HIV can be a point of vulnerability in some circumstances. Creating high-density mannose clusters that mimic HIV's glycan shield are being explored as a vaccine strategy [101], [102].

### Complex chronic signatures in localized regions of Env

Despite testing for complex multi-site signatures within several functional domains in Env, only one multi-site signature was identified, a chronic signature in the CCR5 CoRbs set (Table 3). The CCR5 CoRbs can be a target for broadly neutralizing antibodies [92], [93], and non-neutralizing antibodies against the CCR5 CoRbs may also be able to impose selection on the virus [103]. Interestingly, the only identified signatures found associated with Env glycoproteins that were isolated from individuals that made broad and potent neutralizing antibody responses were also localized in the CCR5 CoRbs [40]. We also tested for distinctive biochemical patterns in local spatial regions in the gp120 structure, and identified three regions that are proximal to the CD4 binding site [57], [58] that undergo change in polarity (Fig. 3). The regions of gp120 surrounding the CD4bs are the most conserved in Env when considered at a structural level [3], thus providing a vulnerable target for cross-reactive HIV antibodies [57], [58]. Changes in electrostatic potential may enable antibody escape from at least some antibodies in HIV-infected individuals who naturally mount a potent and cross-reactive anti-CD4bs antibody response [93], [99], [104], [105].

### A summary view

While the signature patterns we have identified are significantly enriched in terms of association with either early or chronic viruses, still there are exceptions to any given pattern (Table 2–5), and thus the signatures cannot be used to accurately predict whether a given sequence is derived from an acute or chronic infection. This is not surprising, but worth noting. It is a reminder that tests that involve site-directed mutagenesis might fail to result in a phenotypic change even when a site is relevant, because the phenotypic consequences of change in a single amino acid can be context dependent. Furthermore, there may be multiple paths to the same end, and the immune responses that drive repeated patterns of escape in chronic infection are likely to be shared only by a subset of individuals who target a particular Env region. Similarly, reversion in early viruses is likely to be context dependent, depending on the presence of compensatory mutations as well as other selective pressures acting on the virus. It is also of interest that some signature patterns that might have been expected were not observed. We did not see amino acids in the V3 loop that have been noted to be associated with CCR5 co-receptor use predominate in acute infection [17], [23], [26], [37] or those associated with CXCR4 use in chronic infection [106], [107]. We think this is because of inadequate statistical power: CXCR4-using viruses rare among both our early and chronic sequences (Table S4) and there are multiple ways to manifest a CXCR4 phenotype, thus it is likely that no CXCR4-associated substitution was repeated enough to enable identification of a signature.

Despite these issues, several interesting and consistent signature patterns emerged through our study. First, multiple signal peptide and cytoplasmic domain signature patterns were found (Table S2), raising the possibility that Env expression levels may be an important generalized aspect of immune escape during chronic infection. Second, two signatures were found near the CCR5 CoRbs region; this domain is emerging as a key region for neutralizing antibody escape and induction of antibodies in a number of studies, and merits close attention as vaccine design and evaluation strategies progress. Third, the recurrent loss of glycosylation sites in key positions during chronic infection suggests that this pattern typifies an essential aspect of immune escape, leaving a profound and recurring trace at the population level. If the loss of these specific glycosylation sites mediates immune escape from common transmitted forms, in may be advantageous to include these sites in vaccines. In contrast, the loss of the PNLG at position 413–415 was enriched among early sequences, so it may be advantageous to also exclude PNLGs at 413–415 from a vaccine immunogen. Thus the signature patterns identified in this study point to post-translational regulation of Env having a role in selection of early sequences, and indicate particular protein modifications that merit consideration for immunogen design and evaluation.

## Methods

### Ethics statement

Written informed consent was provided by all study participants. The Duke University Health System Institutional Review Board for Clinical Investigations (DUHS IRB), has determined the specific components above under the protocol, “Acute HIV-1 Infection Prospective Cohort Study” (CR3_Pro00006579) to be in compliance with all applicable Health Insurance Portability and Accountability Act ("HIPAA") regulations.

### Data sets

The acute samples were collected from individuals sampled at varying time post-infection, and were clinically staged according to Fiebig et al. [17] to estimate the time between infection and sampling [17], [21], [22]. Chronic samples were selected from individuals who were not on anti-retroviral therapy, and infected for a minimum of two years. All represented subtype B infections, and most samples were collected in the United States, although a small number were from Trinidad/Tobago, included to increase our sample size and power (Table S1). This was a retrospective study involving many cohorts, to enable us to get a large enough sample to perform signature analysis. Table S1 includes demographic and clinical information related to these samples, including viral load at the time of sampling, Fiebig stage, year of sample collection, sampling country, primary risk factor for infection, and whether the sequence evidence indicates that the new infections were established by single or multiple strains. All acute and early samples were obtained from people with sexually acquired HIV. Alignments of the full set of 6303 early and chronic SGA Env sequences used are available in the supplement, and GenBank accession numbers are provided in each of the sequence names (Tables S5–S7). As this study involved samples from HIV-1 infected human subjects, informed consent was obtained from all subjects.

The data were originally separated into two sets: the original hypothesis-raising ‘test’ set, the ‘holdout’ hypothesis-validating set. It was critical that the test and holdout sets each had a good representation of early Fiebig stages, so we ensured that the test and holdout sets each had 19 samples with a Fiebig stage of 3 or less. Each set was also matched for samples that were suggested by the data to be consequence of single infection (68% in the test set, and 65% in the holdout). The early and chronic groups within each set were matched in terms of country of origin (the early and the chronic groups each had ∼30% from Trinidad in the test set, and the early and chronic groups each had ∼5% in holdout set); this was important because the Trinidad sequences formed a distinct clade in phylogenetic analysis and such geographically localized clades can have systematically different patterns of mutations in early or chronic infections. Although these were sexual transmission cohorts, the risk factors for infection were not always known; heterosexuals were well represented in each group.

A third set was added to increase our statistical power for hypothesis forming (Table 1). This set was based on adding early infection samples from plasma donors in the United States, and a set of B clade chronic sequences from the Los Alamos HIV database that were from individuals who were documented in the database entry to not be on anti-retroviral therapy and who had been infected for a minimum of two years. This third set was not as well matched in terms of the clinical and geographic origin as other two sets.

### Sequencing and sample characterization methods

All sequences were obtained from plasma of infected individuals using single genome amplification (SGA) methods, as previously described [17], [38]. A full alignment of all sequences used in this study is available in **Supplement Table S5–S7**; all sequences have been submitted to GenBank in conjunction with this paper, or else were previously submitted, and the accession number of each sequence is included in the sequence name, and at the end of this article. The positions numbers in the paper are generally given as HXB2 position numbers (http://www.hiv.lanl.gov/content/sequence/HIV/REVIEWS/HXB2.html), unless it is specified in the text that the numbering refers to the alignment position used in this study. For signature analysis, all sequences were analyzed in maximum likelihood trees, including multiple sequences from each individual; subject-specific phylogenetic clusters were consistently formed, so there were no overt contamination issues in this study.

Sequences were aligned using a HMMER alignment [47] and then codon aligned with GeneCutter (http://www.hiv.lanl.gov/), with hand correction at the borders of the regions with many insertions and deletions to rectify obvious alignment errors. The hand editing was done because the hypervariable region indels in HIV are particularly difficult for multiple alignment programs [48]-ot only do they exhibit extensive length variation, but the insertions are generally comprised of distinctive direct repeats from neighboring regions in the gene [108]. The alignment was done in iterative steps; first each subject was aligned internally, then a majority consensus sequence representing each subject was generated. For within-subject consensus generation, we considered the codons that that bases were imbedded in, and selected the most common codon for the consensus. This step was required because otherwise simple position-wise consensus sequences occasionally created codons that did not exist within the subject, as the most common bases in highly variable codon positions are not always found in combination. The subject consensus sequences were aligned, then the within-subject sequences sets were aligned to their own consensus in the framework of the full population alignment, and then the whole process was iterated. This alignment was 3120 bases long. To test for dependence on the alignment strategy used, we repeated the consensus sequence signature analysis using an unedited MAFFT alignment [48], [49]; this alignment was 3735 bases long, so had many more gaps; a SATe alignment of this same data was even longer, at 3790 bases (http://phylo.bio.ku.edu/software/sate/sate.html).

### Phylogenetically-based analysis

To identify signature patterns in HIV that relate to a particular phenotype (in this case, early versus chronic status), sampled viruses cannot be treated as independent samples from a random distribution of genotypes. Any population substructure in the data exacerbates the problem. To correct for this we employed a tree corrected contingency table approach used previously [39], but with the addition of more extensive searching capabilities such as the ability to look for statistically interesting combinations of sites in functional domains and loss and gain of glycosylation site motifs [40]. The phylogeny of all sequences was inferred using a maximum likelihood method, and ancestral states were inferred at the internal nodes in the tree [39]. We used a GTR model and a maximum likelihood assignment of rates per site.

The method we originally developed to study the correlation between HIV genotypic variation and host immunological parameters was used directly to correlate the early/chronic status with the consensus genotype in each patient. This method has been previously shown to enable identification of signature sites that could be experimentally validated as biologically meaningful [39], [40], [109]. In particular, when applied to finding mutational associations with host class I HLA genotyping, the associations identified were in known or predicted cytoxic T cell epitopes with the expected frequency [39], [109], and when applied to neutralizing antibody sensitivity, critical mutational patterns were identified among the natural variants [40]. To fully utilize the availability of multiple sequences per subject in this study, we have adapted the original signature identification method to enable tracking of changes in character states observed within each individual defined relative to the most likely state at the last (closest) ancestral node outside the patient. These changes were correlated with the patient being early or chronic (Fig. S1 illustrates the method). The number of sequences sampled varied widely among the patients (indicated by the heights of the bars in Fig. S2), and the diversity at some positions was much greater than others, so a bootstrap approach was used to determine appropriate significance levels for identification of interesting signals (Fig. 1).

For quantitative signatures of continuous variables (in particular, regional polarity scores) Felsenstein's phylogenetic contrast approach was used [56] to estimate a covariance matrix, and Student's t-statistic was used to obtain significance levels for the differences between the early and chronic patients. Since the variables of interest had a bounded domain, we verified manually that the signatures did not arise from saturation of the bounds where the model was strongly violated.

### Statistical testing criteria

Given that we were in a hypothesis raising mode and our expectation was that transmission signatures would be relatively subtle, and we were of necessity in a framework of limited sampling, we decided to require that associations be statistically supported in an initial training data set with a q-value of <0.2, and show at least a trend (q <0.3) towards a consistent pattern of association in a separate analysis of the holdout data. A q-value is a false discovery rate that adjusts for multiple tests [41]. There were many associations with a q<0.2 that were found either only the training or confirmatory data sets that were not supported in both sets, which we do not list here. Retaining a holdout set that is excluded from the initial analysis is not often done in this kind of correlation analysis [109], [110]. Our decision balanced the value of increasing the sample size and the potential for identifying more correlated sites, with the additional level of confidence in our primary findings provided by the holdout set analysis; we opted for the latter to limit our type I false-positive error, although potentially missing interesting signature sites and increasing type II false-negative error. A more comprehensive listing of the non-validated sites provided in the supplement reverses this, and these tables are far more likely to contain false positives, but less likely to miss true positives.

Signatures were sought comparing sequences classified as early by combining data sets incrementally from Fiebig stage 2 up to 6, such that all sequences up to a given stage were combined and then analyzed, and then contrasted with chronic data. The reason we explored the data in this increasingly inclusive fashion was to balance the increasing power that is a consequence of including additional sequences from later Fiebig stages, against the possibility that as samples are taken at progressively later Fiebig stages, transmission signatures may no longer be evident in the sample due to early immune or fitness selection pressures [23], [30]. The Fiebig stage of the data combination that produces the most significant signature associations for a given amino acid pattern is provided in the Tables manuscript to simplify presentation; the use of q-values for statistical significance guards against increasing Type I errors by this procedure.

Cross-validation strategies can provide reasonably unbiased accuracy estimates for classifiers [111], but their use in hypothesis testing suffers from the absence of reliable estimates of their variance [112], [113], [114]. In particular, they are known to have inflated type 1 error rates [115], [116] and can sometimes lead to incorrect choice [117] when used for model/feature selection; we have encountered such issues in a previous study [40], hence we did not use this approach initially for this study, rather we used the strategy described above involving a strictly maintained holdout set. When very few signatures were evident by this conservative approach, however, we turned to cross-validation; even though it has limitations, it often works well in practice [118] and is commonly used for data mining. We used a stratified 10-fold cross validation approach [119] to check robustness of our findings when analyzing the combined test and holdout datasets, to raise hypotheses for further work. We stratified by the early/chronic status, as well as by the sample's geographic origin (i.e. whether the sample originated in the United State or in Trinidad and Tobago, given that Trinidad and Tobago B subtype viruses formed a distinct lineage relative to the B subtype US viruses). 90% of the sample was selected randomly for a training set, and 10% was retained as a test set. As with the full data set analysis of all patient consensus sequences, a q-value of 0.5 was used for the training set criteria of positive, and the test was considered a match if the direction of the odds ratio was preserved (<1 or > = 1).

### Grouping of positions and amino acids for signature analysis based on alignment positions

Our primary analysis was concerned with single site signatures. In addition to the single site signatures we considered the loss or gain of aligned PNLG motifs, where the motif is: NX[T/S], and N is Asn and T/S is either Thr or Ser [120]. Regions of the alignment that could not be reliably aligned due to insertion/deletion events were essentially excluded, by systematically excluding positions where more than 10% gaps had been included to maintain the alignment. One important consequence of this is the exclusion of hypervariable domains where we did not feel confident of the alignment, so associations would be missed in these regions as they could not be reliably identified.

### Signature analysis of combinations of sites in functional domains of HIV-1 Env

We also defined sets of amino acid based on the computed structure and presumed function of the envelope protein. Three sources were used to define these sets, as described in Korber and Gnanakaran [3]. A search of the literature provided critical residues obtained through site directed mutational experiments that probed sites within functional domains as well as antibody binding motifs in gp120 and gp41. We compiled those sites classified according to corresponding functional activities and antibody epitopes. Second, x-ray crystal structures of gp120 are available with different binding partners, including neutralizing monoclonal antibodies. In these cases, we identified the set of relevant key sites based on spatial contacts.

The amino acid positions included in these sets, and the references used to select them, are provided in the supplement (Table S4). The functional domains in gp120 that we considered included CD4 and co-receptor (CCR5 and CXCR4) binding sites, sites that correlate with CCR5 and CXCR4 co-receptor usage, exclusive sites within V3 loop that take part in binding to co-receptor, and V2 gut mucosal homing receptor binding sites. In gp41, we included sites in LLP1 associated with virion incorporation, LLP2 sites associated with Tyrosine-dependent sorting signal and exposure of CD4 binding site, and an additional set of sites associated with modulating entry during fusion process. We also included a set of amino acid positions in both gp120 and gp41 thought important to maintain the Env trimer, and those sites that lie on the interface between gp120 and gp41. The gp120 epitope sites included the binding sites of monoclonal antibodies b12 and 17b. In gp41, the epitope sets included two sets in MPER region covering 4E10 and 2F5 binding sites.

We looked as exhaustively as was feasible given our data and computational constraints for early or chronic signatures in functional domains. How extensively the combinations of sites and amino acids in a given functional domain were explored was determined dynamically. All sets were initially explored based on combinations of 3 positions in the functional domain and up to three amino acids per position; if this resulted in more than 5 million patterns, we then considered only 2 positions and 3 amino acid combinations in the first series of tests. We then tested incrementally more combinations of amino acids at the each of the positions until we reached 5 million patterns per domain, a limit based on computational feasibility; however, if at this point the p- and q-values were still improving, we increased this to up to 10 million tests. We then repeated the incremental iterations including more sites rather than more amino acids per site. When this was done, the combinations with the best p- and q-values were compared between the test and holdout sets; then end result was that essentially only one complex combination signature, in the CCR5 model set, was supported in both sets.

### Contact matrix based signature analysis

A third kind of amino acid set analyzed was based on spatial proximity; we called these ‘contact’ sets. These sets were created from the contact matrix obtained from long timescale molecular dynamics simulations of liganded gp120. The gp120 structure of YU2 strain with modeled loops was carried out with molecular dynamics simulations in explicit aqueous solvent [79], [80], thus incorporating into our model the dynamics, the influence of solvent, relative flexibility of both flexible and conserved regions and the interaction between core and variable regions. Contact profiles were obtained from the simulation trajectories. For each residue in the simulated structure a contact set was generated such that it contained at most the 10 closest contact amino acids, and all amino acids included were within 10 Å of the center. We made a total of 395 contact sets corresponding to the total number of residues in the simulated gp120 molecule informed by the crystal structure. The definition of these contact sets was based both on the distance between amino acids obtained during the entire dynamics and the duration in the dynamical conformation. Sets were excluded from consideration if they contained regions of alignment uncertainty caused by insertion/deletion events.

It was not computationally feasible to analyze all contact lists with all explicit amino acid substitutions. Therefore a few contact sets were chosen for an in-depth analysis based on the full tree single amino acid scan identifying an amino acid within the contact set as potentially interesting. Combinations of positions in the sets containing these positions were analyzed in the same manner as the functional domains; this yielded no complex signatures that were supported in both the test and the holdout sets. We then simplified the information in the contact sets by grouping amino acids into standard side-chain chemically motivated equivalence classes, J = [A I L M F W V], X = [S Y T Q N H], Z = [K R], O = [D E] and U = [G P C], and their unions, and used this to test of complex signatures within all contact sets; this effort identified no new signatures in both test and holdout analysis. We then computed a polarity score for each of the contact sets, a single number representing the chemistry of each local spatial region in gp120. To do this, we used the Hopp and Woods scale, which has been used previously to identify antigenic sites [121], to assign scores to individual amino acids, and then summed these scores over the contact sets. In this case three contact sets yielded statistically interesting correlations in both the test and hold out sets. Because this score could vary almost continuously through small changes in amino acid composition, we used the method of phylogenetically independent contrasts [56] to identify changes in polarity that either correlated with transmission or were recurrent during chronic infection based on the full dataset. Though the range of polarity is finite, violating the assumptions of the method, however we found the observed signatures did not arise from saturating the bounds.

### Correlation of signature sites with neutralization by antibodies and sCD4

For each of a panel of MAbs, or sCD4, concentrations required for 50% neutralization (IC50) were determined for 113 SGA-derived Envs expressed as pseudovirions [122] from 73 individuals sampled either in early or chronic infection (Table S3). This represents an extension of the set previously reported in Keele et al. [17], using the same experimental methods. To determine if there were significant correlations between the presence or absence of signatures patterns and neutralization phenotypes we used non-parametric Wilcoxon rank statistics as implemented in the R project for statistical computing http://www.r-project.org/).

### Testing for correlations of between lengths and number of glycosylation sites in hypervariable loops and early versus chronic sampling

Because of pre-existing literature on this subject leading to an expectation that early Env hypervariable loops would be shorter with few glycosylation sequons, we grouped all early and all chronic samples for this study, and did not separate our data into a hypothesis forming and holdout sets. Furthermore, since we have no good models to reconstruct ancestral states for the variable loops that are subject to rapid within-subject insertions and deletions, in this study we did not correct for phylogenetic relationship between the sequences. Instead, we compared the tallies of number of glycosylation sites or loop lengths based on a single consensus sequence from each subject in early versus chronic infections using a Wilcoxon rank statistic; this simple test revealed there were less glycosylation sites overall in gp120 among early infections, supporting previous findings. We next compared the spectrum of variants found in each subject. Because the within-subject sequences are not independent and the number of such samples varied widely from patient-to-patient, we re-sampled the sequences from each subject 1,000 times to create sets with a constant sample size across subjects, which we chose to be the smallest number of sequences obtained from a single subject in the real data. We then compared the distributions found in the early versus chronic data with a Wilcoxon test, and then did a Monte Carlo test shuffling the early/chronic designations 1000 times based on each of the re-samplings, to see how often the level of distinction based on the real data was found in the randomized data.

### GenBank accession mumbers

GenBank accession numbers for sequences used in this study. New sequence accession numbers for newly introduced sequences in this study: HQ216367-HQ218052, HQ238279-HQ238288. Previously published CHAVI sequence accession numbers: EU574937-EU575065, EU575067-EU575212, EU575214-EU575231, EU575233,EU575235-EU575251, EU575253-EU575265, EU575267-EU575272, EU575274-EU575441, EU575443-EU575468, EU575470-EU575552, EU575554-EU575636, EU575638-EU575704, EU575706-EU575775, EU575777-EU575852, EU575854-EU575943, EU575945-EU575980, EU575982-EU575990, EU575992-EU576064, EU576066-EU576089,EU576091-EU576237, EU576239-EU576292, EU576294-EU576296, EU576298-EU576619, EU576621-EU576642, EU576644, EU576646-EU576774, EU576776-EU576799, EU576801-EU576814, EU576816-EU576817, EU576819-EU576840, EU576842-EU576936, EU576938-EU577005, EU577007-EU577100, EU577102-EU577114, EU577116-EU577310, EU577312-EU577350, EU577352-EU577433, EU577435-EU577440, EU577442-EU577478, EU577480-EU577662, EU577664-EU578089, EU578091-EU578109, EU578111-EU578174, EU578176-EU578239, EU578241-EU578292, EU578294-EU578307, EU578309-EU578321, EU578323-EU578328, EU578330-EU578331, EU578333-EU578375, EU578377-EU578512, EU578514-EU578559, EU578561-EU578576, EU578578, EU578580-EU578636, EU578638-EU578677, EU578679-EU578686, FJ495818, FJ495937, FJ496000-FJ496001, GU330247-GU330646, GU330648-GU330861, GU330938-GU331030, GU331032-GU331095, GU331098-GU331102, GU331114, GU331116, GU331121-GU331122, GU331127, GU331129-GU331133, GU331183-GU331217, GU331634 GU331721. Previously published database chronics selected from the Los Alamos based on their annotation clearly indicating chronic infection, including one representative sequence chosen per subject sample: AY223734, AY223785, AY535433, AY535511, AY535480, AY535461, AY842807, AY842816, AY842830, DQ410613, DQ410520, DQ410533, DQ410542, DQ410569, DQ410586, DQ410599, AY357342, DQ410219, DQ410057, DQ410068, DQ410075, DQ410099, DQ410105, DQ410120, DQ410177, DQ410196, DQ222216, DQ410454, DQ410483, DQ410623, DQ410280, DQ410322, DQ410338, DQ410384, DQ410412, AY423385, DQ410232, DQ976380, DQ976394, AJ535593, AJ535602, DQ853433, DQ976430, AY314044.

## Supporting Information

Figure S1 **Phylogenetic tree of all sequences in this study, highlighting the signature at position 12.** This figure illustrates the abundance of the underlying data, the within sample diversity found in early and chronic subjects highlighting typical examples, and the basic principals of our signature identification strategy when all available sequences in the data set are used. The branches of this maximum likelihood phylogenetic tree are shown as black lines. If sequences were isolated from an early sample, they are labeled with a magenta line to the right of the leaf node of the tree. If the sample was chronic, the sequences are labeled with a grey line. The different amino acids found in sequences in position 12 are represented by different colors, thus a bright spot of color at every leaf node indicates the amino acid found in position 12 in a sampled sequence, and a bright spot on an interior nodes represents the most likely amino acid found at that node based on the phylogenetic model. A His is the signature amino acid of interest for this comparison, it is labeled bright red. There are thousands of sequences in this tree, thus it is impossible to see detail, so the region boxed in red was enlarged to illustrate the method we used to identify signatures. A representative acutely infected subject was boxed in green, and further expanded. The ancestral node just prior to the infection in position 12 was predicted to be a His (H12), and H12 was also the most likely amino acid in the recent common ancestor sequence (MRCA), or founder virus, in this subject. H12 was also found in all other sequences sampled in this subject. Thus this subject would be counted as an early subject with H12 carried in at transmission, that remained unchanged (H stasis), i.e. no H to !H changes in position 12 were observed between the last ancestral node outside the subject, through the MRCA, and out to all of the sampled sequences from within the subject. In contrast, a chronic infection case boxed in blue represents a typically diverse population of sequences found in chronic infection. Again, the ancestral state at the node immediately preceding the infection, as well as the MRCA of the subject's sequences, were both most likely to be a His (the red X on the left hand side of the blue box). This subject would be counted as a chronic subject with H12 as the most recent ancestor prior to transmission, and would contribute a total of 2 “H to !H” changes to the total tally of chronic infection H to !H changes. This is because in this subject an H12 ancestral state was predicted to have changed to Pro, represented by the yellow “0”, twice independently. It is not possible to distinguish whether within-subject recombination carried the mutation into two lineages, or if it arose from two distinct convergent mutations, but by either mechanism the Pro arose in two distinctive lineages in the within-subject clade. To search for significant signatures, similar tallies were made across all patients for every amino acid at every position, Fisher's exact tests were performed, and q-tests were conducted to control for multiple tests.
(EPS)

Figure S2 **Distributions of amino acids in each subject for representative signature patterns.** (A) Each vertical bar represents a subject, and the height of the bar indicates the number of sequences sampled. The subjects are grouped according to the data set breakdown in Table 1. A solid color in a bar indicates the single amino acid found in that subject, while bars with multiple colors indicate that multiple amino acids are found in the position, and the contribution of each color to the bar indicates how often each amino acid is found. The color key shows which amino acids are represented by which color. The signature of interest at position 12, His, is black, and as is illustrated here, is more common in early than in chronic infection. The pattern illustrated here, with His the most common amino acid in position 12, is shared by most subtypes (A, B, D, F and G), but in the C subtype, Gln dominates www.hiv.lanl.gov). Thus, there is likely to be a subtype-specific context relevant to this signature. (B) The basic structure of this figure is like (A), but instead of amino acids the colors indicate the presence or absence of the PNLG motif at positions 397–399. This again illustrates the complexity of the sample, yet signature motif is clearly frequently lost in chronic infection. Chronic sequences from the database that met our inclusion criteria included a small number of chronically infected elite controllers; these sequences had unusual patterns so they are marked in both (A) and (B).
(EPS)

Figure S3 **Difference in estimated probabilities of mismatch.** For each amino acid position, a test statistic was computed to evaluate whether the frequency distribution of amino acids at the position differed between the acute/early sequences and the chronic sequences. The test statistic is the estimated probability of inter-subject amino acid mismatch for all pairs of acute sequences minus the estimated probability of inter-subject amino acid mismatch for all pairs of acute versus chronic sequences. This test statistic is plotted for each position, and a large departure from zero constitutes evidence for different amino acid frequencies in the two groups of sequences. Four positions had evidence for a significant difference (based on a q-value<0.20) and are highlighted red.
(EPS)

Figure S4 **Receiver operating characteristic curves for evaluating accuracy of amino acids to classify acute/chronic status.** Threshold gradient descent regularization (TGDR) was used to evaluate the set of amino acids at certain positions that best predict whether a sequence is from an acute/early or chronic subject. One hundred random splits of the Original data set were made, with 2∶1 allocation into training:test sets. For each of the 100 splits, the left panel shows receiver operating characteristic (ROC) curves that summarize the ability of the selected best model to classify subjects into acute/early versus chronic for the same data that were used to build the model; in contrast the right panel shows the 100 ROC curves computed on the data left out of the model-building. The right-panel provides information on how well the model can classify independent data, and the fact that the ROC curves tend to be slightly to the left of and above the y = x line suggest weak ability of the amino acids to classify acute/chronic status.
(EPS)

Figure S5 **Antibody footprint sphere scanning Q-values for Original and Holdout data-sets.** 226 sphere set clusters of residues on gp120 (centered around each exposed surface residue) that might be targeted by antibodies were identified, with radii selected to be the normal size of known antibody-antigen complexes. For each sphere set, Poisson regression for repeated measures data was used to evaluate whether the number of mismatched amino acids in the sphere set compared to the reference sphere set (acute/early consensus) differed for acute/early versus chronic sequences. 226 Wald-based p-values were obtained, from which q-values were computed. For the Original and Holdout data-sets, q-values are plotted, where we show q-values only for the sphere sets/clusters that had the smallest p-values. The sphere sets are indexed by the exposed surface ‘anchor’ residue.
(EPS)

Figure S6 **Antibody footprint sphere scanning Holm-Bonferroni adjusted p-values for Original and Holdout data-sets.** The figure shows the results of the same analysis as presented in Figure S3, except Holm-Bonferroni family-wise error rate adjusted p-values are shown instead of q-values. These adjusted p-values are stringent, supporting that cluster 437 is a signature sphere set for the Holdout data after accounting for the multiplicity of hypothesis tests.
(EPS)

Figure S7 **Cluster 437: Numbers of amino acid mismatches to the reference (acute consensus) sphere set.** The figure describes the nature of the significant Holdout data signature sphere set number 437. For each of the Original and Holdout data-sets, histograms of the number of sequences with 0, 1, 2, 3, 4, 5, or 6 mismatches (the maximum observed) relative to the reference sequence (acute/early consensus) are plotted, stratified by acute/early (labeled Stage I-VI) versus chronic and stratified by phylogenetic sub-clade a/d/e (all from subjects not from Trinidad) versus b/c (all from Trinidad subjects).
(EPS)

Table S1 **Background information regarding subjects.** This table summarizes basic information regarding the sample and subject, including the subject ID, the cohort that was source of the sample, the risk factor for infection and sex of the subject, Fiebig stage of infection, viral load, CD4 counts, year of sampling, country of sampling, state if known, and whether or not the diversity in the sample was indicative of a single virus established infection (homogeneous) or if multiple viruses established infection (heterogeneous). The number of sequences per sample is indicated in the N_seqs column. Sex of the subject is M for male, F for female, and risk factors are: sexual, heterosexual (SH), sexual male sex with male (SM), sexual bisexual (SB), IV drug use (ID), not reported (NR). US stands for The United States of America, TT for The Republic of Trinidad and Tobago. If samples from later Fiebig stages showed exclusively highly clustered diversity, it was considered to be likely immune escape-driven diversity. These samples were obtained retrospectively from several cohorts and collections, hence not all samples have equivalent information available. The GenBank accession numbers of each sequence can be found in the complete sequence alignment files.
(XLS)

Table S2 **Signature hypotheses raised by including all sequences per patient.** Here we compared just the original data to the holdout, and these sites illustrate the complete set of sites with a p<0.2 in the original and q<0.3 in the holdout. All signatures that were identified across both data sets where chronic; in the full analysis this means that the pattern of change observed was repeated enriched during chronic infection relative to acute. These sets on the whole were not as profoundly significant as the 4 sites we included in the main text based on combining the data from the original set with the plasma donors/database set to increase our sample size. Several signatures were supported by the two analyses: 12H, 362K, and 399T. Sites in regions with uncertain alignment were excluded from this table, 3 sites that were significant in both sets but had a reversed pattern of amino acid substitutions were also excluded (for example A to !A was enriched in chronics in the original sets and in acutes in the holdout).
(DOC)

Table S3 **MAb and sCD4 IC50 data and coreceptor usage of SGA clones used in our study, comparing chronic versus acute sequences, augmenting the data set first described in Keele et al (ref 1).** The values given are 50% neutralization titers of monoclonal antibodies b12, 2G12, 2F5, 4E10, Z13e1, 447-52D, 447, F425, 17b, soluble CD4 (sCD4), and HIVIG, co-receptor usage, and infectious units per ul.
(DOC)

Table S4 **List of functional domain HXB2 positions.** Combinations of sites in these functional regions were tested for correlations with acute/early versus chronic infection. The only one that provided a combination of sites that was significant was the CCR5coR model set, the set of variable positions proximal to the conserved CCR5 binding sites; it is indicated in bold.
(DOC)

Table S5 **All sequences generated for this study under** ***Original Set*** **, aligned.** The first character of each sequence name indicates either the Fiebig stage at time of sampling, or C for chronic infection (see material and methods for details). The GenBank numbers are all included in the name of each sequence in the files; as there are thousands of sequences, and the numbers are not continuous, this seemed the most parsimonious presentation.
(DOC)

Table S6 **All sequences generated for this study under** ***Holdout Set*** **, aligned.** The first character of each sequence name indicates either the Fiebig stage at time of sampling, or C for chronic infection (see material and methods for details). The GenBank numbers are all included in the name of each sequence in the files; as there are thousands of sequences, and the numbers are not continuous, this seemed the most parsimonious presentation.
(DOC)

Table S7 **All sequences generated for this study under** ***PlasmaDonors Set*** **, aligned.** The first character of each sequence name indicates either the Fiebig stage at time of sampling, or C for chronic infection (see material and methods for details). The GenBank numbers are all included in the name of each sequence in the files; as there are thousands of sequences, and the numbers are not continuous, this seemed the most parsimonious presentation.
(DOC)

Table S8 **Distributions of Amino Acid Sets for Cluster 437: Sites 207, 326, 327, 422, 436, 437, 439.** Sphere-sets (sets/clusters of amino acid sites within a sphere centered on a surface residue with diameter selected to fit a typical conformational antibody epitope) potentially containing antibody epitopes were evaluated using a generalized linear model fit by generalized estimating equations, to assess a different distribution of amino acid patterns in the acute/early group versus the chronic group, relative to the reference set (the consensus among acute/early sequences). Cluster 437 (comprised of sites 207, 326, 327, 422, 436, 437, 439) had q-value<0.001 and Hom-Bonferroni adjusted p-value<0.001 for the Holdout data, and is noteworthy because the q-value and adjusted p-value are small, and because it includes CD4-induced epitope sites originally described in Wyatt et al. (1998). The table shows the distribution of amino acid sets in cluster 437 for the Original data and for the Holdout data. For the Holdout data several patterns of mutation away from the reference set KIRQAPI are over-represented in the chronic sequences. However, for the Original data the pattern of amino acid mutations was opposite to that for the holdout data, with mutation away from the reference set KIRQAPI slightly over-represented in acute/early sequences.
(DOC)

Text S1 **This part summarizes strategies to define Acute versus Chronic HIV Signature Analysis via Non-Phylogenetically-Corrected Statistical Analyses.**
(DOC)
