# Supplementary material for: Recurrent Signature Patterns in HIV-1 B Clade Envelope Glycoproteins Associated with either Early or Chronic Infections
Source: PLoS Pathog. 2011 Sep 29;7(9):e1002209. doi: 10.1371/journal.ppat.1002209 (PMC3182927; doi:10.1371/journal.ppat.1002209)
Supplement: Table S2 — Signature hypotheses raised by including all sequences per patient. Here we compared just the original data to the holdout, and these sites illustrate the complete set of sites with a p<0.2 in the original and q<0.3 in the holdout. All signatures that were identified across both data sets where chronic; in the full analysis this means that the pattern of change observed was repeated enriched during chronic infection relative to acute. These sets on the whole were not as profoundly significant as the 4 sites we included in the main text based on combining the data from the original set with the plasma donors/database set to increase our sample size. Several signatures were supported by the two analyses: 12H, 362K, and 399T. Sites in regions with uncertain alignment were excluded from this table, 3 sites that were significant in both sets but had a reversed pattern of amino acid substitutions were also excluded (for example A to !A was enriched in chronics in the original sets and in acutes in the holdout). (DOC) [file ppat.1002209.s009.doc]

| **HXB2** | **Aln** | **AA** | **Pattern** | **p-value** | **r1c1** | **r1c2** | **r2c1** | **r2c2** | **q-value** | **OR** | **data** | **Note** |
| --- | --- | --- | --- | --- | --- | --- | --- | --- | --- | --- | --- | --- |
| 8R | 8 | K | A to !A | 0.00014 | 7 | 58 | 28 | 42 | 0.0011 | 0.18 | Original | Signal peptide |
|  |  |  |  | 0.010 | 8 | 62 | 25 | 62 | 0.020 | 0.32 | Holdout |  |
|  | 8 | R | !A to A | 6.4e-05 | 5 | 60 | 26 | 44 | 0.0016 | 0.14 | Original |  |
|  |  |  |  | 0.16 | 6 | 64 | 15 | 72 | 0.30 | 0.45 | Holdout |  |
| 12H | 12 | H | A to !A | 1.8e-08 | 10 | 69 | 57 | 54 | 5.2e-07 | 0.14 | Original | Signal peptide |
|  |  |  |  | 8.9e-05 | 14 | 66 | 49 | 61 | 0.00023 | 0.27 | Holdout |  |
| 29S | 43 | S | A to !A | 8.6e-05 | 10 | 81 | 36 | 67 | 0.00079 | 0.23 | Original | Signal peptide |
|  |  |  |  | 0.0050 | 5 | 76 | 21 | 77 | 0.011 | 0.24 | Holdout |  |
| 30A | 44 | A | A to !A | 0.00082 | 7 | 87 | 25 | 72 | 0.0047 | 0.23 | Original | Signal peptide cleavage |
|  |  |  |  | 0.043 | 19 | 66 | 42 | 74 | 0.069 | 0.51 | Holdout |  |
| 232T | 303 | K | A to !A | 0.00060 | 18 | 57 | 37 | 34 | 0.0035 | 0.29 | Original | PNLG gp41 interface |
|  |  |  |  | 0.038 | 38 | 40 | 105 | 62 | 0.064 | 0.56 | Holdout |  |
| 270V | 342 | I | !A to A | 0.00050 | 15 | 73 | 42 | 63 | 0.0077 | 0.31 | Original | gp120 |
|  |  |  |  | 0.038 | 23 | 63 | 49 | 70 | 0.081 | 0.52 | Holdout |  |
|  | 342 | V | A to !A | 0.00030 | 15 | 73 | 43 | 62 | 0.0019 | 0.30 | Original |  |
|  |  |  |  | 0.019 | 23 | 63 | 51 | 68 | 0.037 | 0.49 | Holdout |  |
| 277F | 349 | F | A to !A | 6.6e-05 | 7 | 86 | 29 | 65 | 0.00064 | 0.18 | Original | gp120 Loop D  CD4bs/VRC01 |
|  |  |  |  | 0.00012 | 11 | 70 | 46 | 74 | 0.00029 | 0.25 | Holdout |  |
| 346A | 424 | V | A to !A | 3.2e-05 | 15 | 72 | 43 | 50 | 0.00037 | 0.24 | Original | gp120 |
|  |  |  |  | 0.0061 | 14 | 44 | 58 | 70 | 0.012 | 0.39 | Holdout |  |
| 347S | 425 | E | A to !A | 0.00018 | 11 | 14 | 33 | 4 | 0.0014 | 0.10 | Original | gp120 |
|  |  |  |  | 0.20 | 16 | 14 | 76 | 38 | 0.28 | 0.57 | Holdout |  |
| 351E | 430 | E | A to !A | 0.00014 | 5 | 90 | 25 | 77 | 0.0011 | 0.17 | Original | gp120 |
|  |  |  |  | 4.0e-07 | 16 | 73 | 93 | 95 | 2.0e-06 | 0.23 | Holdout |  |
| 362K | 445 | N | A to !A | 3.0e-05 | 20 | 62 | 67 | 58 | 0.00034 | 0.28 | Original | PNLG |
|  |  |  |  | 1.1e-06 | 24 | 56 | 117 | 69 | 4.7e-06 | 0.25 | Holdout |  |
| 399T | 489 | T | A to !A | 7.0e-06 | 9 | 84 | 41 | 71 | 0.00014 | 0.19 | Original | PNLG V4 |
|  |  |  |  | 3.5e-06 | 19 | 67 | 84 | 76 | 1.0e-05 | 0.26 | Holdout |  |
| 424I | 534 | I | A to !A | 0.0015 | 3 | 83 | 17 | 73 | 0.0081 | 0.16 | Original | CCR5 CoR CD4bs β20/β21 |
|  |  |  |  | 0.053 | 16 | 66 | 39 | 80 | 0.078 | 0.50 | Holdout |  |
| 624N | 772 | N | A to !A | 0.0013 | 0 | 11 | 8 | 4 | 0.0075 | 0.00 | Original | gp41 |
|  |  |  |  | 0.025 | 4 | 12 | 17 | 9 | 0.041 | 0.18 | Holdout |  |
| 633R | 782 | R | A to !A | 0.00037 | 17 | 64 | 50 | 57 | 0.0023 | 0.30 | Original | gp41 |
|  |  |  |  | 0.10 | 18 | 54 | 40 | 66 | 0.14 | 0.55 | Holdout |  |
| 668S | 819 | N | !A to A | 1.1e-05 | 9 | 81 | 35 | 58 | 0.00027 | 0.19 | Original | gp41 |
|  |  |  |  | 1.7e-06 | 2 | 79 | 29 | 74 | 1.8e-05 | 0.07 | Holdout |  |
|  | 819 | S | A to !A | 0.00013 | 13 | 77 | 37 | 56 | 0.0010 | 0.26 | Original |  |
|  |  |  |  | 1.8e-06 | 5 | 76 | 36 | 67 | 7.1e-06 | 0.12 | Holdout |  |
| 700A | 851 | A | A to !A | 0.00034 | 9 | 55 | 31 | 43 | 0.0022 | 0.23 | Original | gp41 |
|  |  |  |  | 0.020 | 12 | 30 | 36 | 34 | 0.036 | 0.38 | Holdout |  |
| 807L | 984 | I | !A to A | 0.00017 | 0 | 91 | 13 | 84 | 0.0031 | 0 | Original | Cytoplasmic tail  LLP-3 |
|  |  |  |  | 3.1e-05 | 1 | 86 | 20 | 84 | 0.00015 | 0.05 | Holdout |  |
|  | 984 | L | A to !A | 8.0e-05 | 0 | 91 | 14 | 83 | 0.00076 | 0 | Original |  |
|  |  |  |  | 3.1e-05 | 1 | 86 | 20 | 84 | 0.00010 | 0.05 | Holdout |  |
| 808K | 985 | K | A to !A | 0.0016 | 7 | 87 | 25 | 76 | 0.0088 | 0.25 | Original | Cytoplasmic tail  LLP-3 |
|  |  |  |  | 0.0015 | 9 | 79 | 33 | 83 | 0.0033 | 0.29 | Holdout |  |
| 809N | 986 | N | A to !A | 0.00022 | 9 | 85 | 33 | 74 | 0.0016 | 0.24 | Original | Cytoplasmic tail  LLP-3 |
|  |  |  |  | 0.0016 | 13 | 73 | 44 | 83 | 0.0033 | 0.34 | Holdout |  |
| 812V | 989 | V | A to !A | 0.00016 | 9 | 83 | 32 | 66 | 0.0013 | 0.23 | Original | Cytoplasmic tail LLP-3 |
|  |  |  |  | 1.3e-08 | 4 | 78 | 42 | 64 | 1.1e-07 | 0.08 | Holdout |  |

**Table S2. Signature hypotheses raised by including all sequences per patient.** Here we compared just the original data to the holdout, and these sites illustrate the complete set of sites with a p < 0.2 in the original and q < 0.3 in the holdout. All signatures that were identified across both data sets where chronic; in the full analysis this means that the pattern of change observed was repeated enriched during chronic infection relative to acute. These sets on the whole were not as profoundly significant as the 4 sites we included in the main text based on combining the data from the original set with the plasma donors/database set to increase our sample size. Several signatures were supported by the two analyses: 12H, 362K, and 399T. Sites in regions with uncertain alignment were excluded from this table, 3 sites that were significant in both sets but had a reversed pattern of amino acid substitutions were also excluded (for example A to !A was enriched in chronics in the original sets and in acutes in the holdout).
