# Supplementary material for: Recurrent Signature Patterns in HIV-1 B Clade Envelope Glycoproteins Associated with either Early or Chronic Infections
Source: PLoS Pathog. 2011 Sep 29;7(9):e1002209. doi: 10.1371/journal.ppat.1002209 (PMC3182927; doi:10.1371/journal.ppat.1002209)
Supplement: Table S3 — MAb and sCD4 IC50 data and coreceptor usage of SGA clones used in our study, comparing chronic versus acute sequences, augmenting the data set first described in Keele et al (ref 1). The values given are 50% neutralization titers of monoclonal antibodies b12, 2G12, 2F5, 4E10, Z13e1, 447-52D, 447, F425, 17b, soluble CD4 (sCD4), and HIVIG, co-receptor usage, and infectious units per ul. (DOC) [file ppat.1002209.s010.doc]

|  | b12 | 2G12 | 2F5 | 4E10 | Z13e1 | 447-52D | 447+sCD4 | F425-B4e8 | F425+sCD4 | 17b | 17b+sCD4 | sCD4 | HIVIG | co-receptor | | IU/ul |  | |
| --- | --- | --- | --- | --- | --- | --- | --- | --- | --- | --- | --- | --- | --- | --- | --- | --- | --- | --- |
| **Transmitted Env**  **clones** | ug/ml | ug/ml | ug/ml | ug/ml | ug/ml | ug/ml | ug/ml | ug/ml | ug/ml | ug/ml | ug/ml | nM | ug/ml | usage | |  |  | |
| BORId9.4F8.1413 | 0.8 | >50 | >50 | 9.8 | >50 | >25 | 0.2 | >10 | 1.7 | >10 | >10 | 306 | 461 | R5 | | 631 |  | |
| BORId9.4D7.1410 | 2.1 | >50 | >50 | 8.1 | >50 | >25 | 16 | >10 | >10 | >10 | >10 | 343 | 240 | R5 | | 1393 |  | |
| BORId9.4F12.1412 | 4.2 | >50 | >50 | 6.4 | >50 | >25 | >25 | >10 | >10 | >10 | >10 | 303 | 429 | R5 | | 80 |  | |
| BORId9.2F8.1396 | 5.4 | >50 | >50 | 18.5 | >50 | >25 | 17.2 | >10 | >10 | >10 | >10 | >1000 | 346 | R5 | | 1129 |  | |
| BORId9.2E12.1395 | 0.7 | >50 | >50 | 6.5 | >50 | >25 | 5.1 | >10 | 10 | >10 | >10 | 589 | 413 | R5 | | 135 |  | |
| 1051-12.C22.3325 | 1 | >50 | 9.8 | 31.7 | >50 | >25 | 5.9 | >10 | >10 | >10 | >10 | 339 | >1000 | R5/X4^3^ | | 1998 |  | |
| 1051-12.TD12.3291 | 0.4 | >50 | 5 | 20 | >50 | >25 | 18.8 | >10 | >10 | >10 | >10 | 85 | 890 | R5/X4^3^ | | 6550 |  | |
| 1051-09.11D.5899 | 1.28 | >50 | 2.61 | 10.34 | >50 | >25 | 20.46 | >10 | >10 | >10 | >10 | 255 | 831 | R5 | | 2604 |  | |
| 1006-11.C3.1601 | 0.8 | 1.6 | 5.6 | 11.5 | >50 | >25 | 1.6 | >10 | 7.9 | >10 | >10 | 241 | 688 | R5 | | 347 |  | |
| 1053-07.B15.1648 | >50 | 4.5 | 9.1 | 7.3 | >50 | >25 | 16.6 | >10 | 0.8 | >10 | >10 | 428 | 322 | R5 | | 149 |  | |
| 1054-07.TC4.1499 | 4.2 | >50 | >50 | 4.8 | >50 | >25 | >25 | >10 | >10 | >10 | >10 | 113 | 449 | R5 | | 1148 |  | |
| 1056-10.TA11.1826 | 1.2 | 14.3 | 0.7 | 3.4 | 23.4 | >25 | >25 | >10 | >10 | >10 | >10 | 635 | >1000 | R5 | | 219 |  | |
| 1012-11.TC21.3257 | >50 | 10.9 | 1.7 | 5.8 | 36.2 | >25 | >25 | >10 | >10 | >10 | >10 | 331 | >1000 | R5 | | 1325 |  | |
| 6240-08.TA5.4622 | >50 | 0.3 | 9.6 | 15.5 | >50 | >25 | >25 | >10 | >10 | >10 | >10 | 478 | >1000 | R5 | | 2813 |  | |
| 6244-13.B5.4576 | >50 | 26 | >50 | 2.3 | 15.4 | >25 | >25 | >10 | >10 | >10 | >10 | 254 | 455 | R5 | | 1480 |  | |
| 62357-14.D3.4589 | 47.8 | 19.3 | 5 | 8.7 | 33.5 | >25 | >25 | >10 | >10 | >10 | >10 | 83 | 655 | R5 | | 753 |  | |
| 9021-14.B2.4571 | 2.7 | 45.9 | 6.4 | 9.7 | 31.4 | >25 | >25 | >10 | >10 | >10 | >10 | 378 | 618 | R5 | | 167 |  | |
| 9020-20.TB1.4597 | 30.9 | 11.6 | 0.6 | 1.4 | >50 | 12.7 | 4.5 | 3.1 | 3.3 | >10 | >10 | 217 | 44 | R5 | | 9 |  | |
| SC51.4B2.2673 | 0.1 | 2.7 | 0.2 | 0.9 | 5 | >25 | >25 | >10 | >10 | >10 | >10 | 122 | 32 | R5 | | 260 |  | |
| SC51.4G3.2649 | 1.1 | 3.5 | 0.1 | 0.3 | 6 | >25 | >25 | >10 | >10 | >10 | >10 | 282 | 107 | R5 | | 222 |  | |
| TT29P.3A1.2769 | 0.7 | 38.8 | 3.4 | 4.9 | 23.6 | >25 | >25 | >10 | >10 | >10 | >10 | 207 | 373 | R5 | | 1216 |  | |
| TT31P.2E10.2789 | 0.1 | 3.7 | 0.8 | 4.1 | 13.3 | >25 | >25 | >10 | >10 | >10 | >10 | 288 | 110 | R5 | | 627 |  | |
| TT31P.2F10.2792 | 0.1 | 4.7 | 2.5 | 6 | 29 | >25 | >25 | >10 | 10 | >10 | >10 | 246 | 311 | R5 | | 1408 |  | |
| WEAUd15.410.787 | 1.2 | 0.3 | 0.8 | 1.1 | >50 | >25 | >25 | >10 | >10 | >10 | >10 | 72 | 507 | R5/X4 | | 1165 |  | |
| 9014-01.TB1.4769 | 7.6 | >50 | 2.2 | 11 | >50 | >25 | 17.3 | >10 | 10 | >10 | >10 | >1000 | >1000 | R5 | | 1117 |  | |
| 9015-07.A1.4729 | 3.5 | 4 | 2.6 | 4.9 | >50 | >25 | >25 | >10 | >10 | >10 | >10 | >1000 | 413 | R5 | | 1893 |  | |
| 62615-03.P4.3964 | 10.5 | >50 | >50 | 25 | >50 | >25 | >25 | >10 | >10 | >10 | >10 | 354 | >1000 | R5 | | 1948 |  | |
| 63068-05.A10.4784 | 18.2 | 5 | 7.9 | 15 | >50 | >25 | 2.6 | >10 | 4.3 | >10 | >10 | >1000 | 565 | R5 | | 1267 |  | |
| 700010040.C9.4520 | 0.7 | >50 | 5.1 | 9 | >50 | >25 | >25 | >10 | >10 | >10 | >10 | 97 | 441 | R5 | | 2554 |  | |
| PRB931-06.TC3.4930 | 22.7 | 1.4 | 7.2 | 20.9 | >50 | >25 | 14.5 | >10 | 7.6 | >10 | >10 | 1000 | 440 | R5 | | 1038 |  | |
| PRB956-04.B20.4265 | 1.7 | 2.5 | 1.8 | 5.1 | >50 | >25 | 4.9 | >10 | 4 | >10 | >10 | 152 | 426 | R5 | | 338 |  | |
| SC22.3C2.2466 | 5.3 | 2.1 | >50 | 5.8 | >50 | >25 | >25 | >10 | >10 | >10 | >10 | 514 | 453 | R5 | | 225 |  | |
| SC33.4H1.2589 | 0.6 | 2.9 | 1.8 | 3.2 | 50 | 25 | 8.2 | 4.3 | 9.3 | >10 | >10 | 61 | 339 | R5 | | 3785 |  | |
| SC33.4A4.2576 | 0.7 | >50 | 2.6 | 2.9 | 30.8 | >25 | >25 | >10 | >10 | >10 | >10 | 928 | 461 | R5 | | 3229 |  | |
| SC05.8C11.2344 | 1.9 | 19.1 | 1.4 | 1.7 | 28 | >25 | >25 | >10 | >10 | >10 | >10 | 267 | 323 | R5 | | 1067 |  | |
| SC45.4B5.2631 | 0.7 | 7.4 | 0.8 | 3.2 | 50 | >25 | 5.1 | 9.6 | 6.5 | >10 | >10 | 268 | 209 | R5 | | 988 |  | |
| 1058-11.B11.1550 | 3 | >50 | 2.4 | 2.4 | 34 | >25 | >25 | >10 | >10 | >10 | >10 | 298 | 642 | R5/X4^3^ | | 1625 |  | |
| 1059-09.A4.1460 | 1 | 11.4 | 0.3 | 0.6 | 22 | >25 | 15.9 | >10 | 6.5 | >10 | >10 | 201 | 55 | R5 | | 229 |  | |
| TT27P.8H1.2730 | 1.6 | >50 | 0.7 | 1.5 | >50 | >25 | >25 | >10 | >10 | >10 | >10 | 1000 | 393 | R5 | | 131 |  | |
| TT27P.8C2.2714 | 0.6 | >50 | 1.3 | 1.1 | >50 | >25 | >25 | >10 | >10 | >10 | >10 | 188 | 496 | R5 | | 667 |  | |
| TT34P.8C11.2833 | >50 | 8.5 | 2.1 | 2.5 | 20 | >25 | >25 | >10 | >10 | >10 | >10 | 129 | 227 | R5 | | 1225 |  | |
| TT35P.11H8.2874 | >50 | >50 | 1.8 | 3.5 | 30 | >25 | 3 | >10 | >10 | >10 | >10 | 643 | 228 | R5 | | 942 |  | |
| 63358-04.P3.4013 | >50 | >50 | 3.1 | 4 | >50 | >25 | >25 | >10 | >10 | >10 | >10 | 538 | 1000 | R5 | | 6244 |  | |
| RHPA.A19.2000 | 0.5 | >50 | 3.6 | 6.5 | >50 | 11.7 | 20.6 | >10 | >10 | >10 | >10 | 288 | 166 | R5 | | 163 |  | |
| 62130-04.B10.4768 | 25.5 | >50 | 0.8 | 5.2 | >50 | >25 | 0.5 | >10 | 8.6 | >10 | >10 | 460 | 127 | R5 | | 802 |  | |
| 700010058.A4.4375 | 1.5 | 0.4 | 0.3 | 1.9 | 21.3 | >25 | 12.2 | >10 | >10 | >10 | >10 | 413 | 378 | R5 | | 625 |  | |
| SC31.4E11.2549 | 0.5 | 10.8 | 0.4 | 1.2 | 16.1 | >25 | >25 | >10 | >10 | >10 | >10 | 208 | 186 | R5 | | 956 |  | |
| REJO.D12.1972 | 5.5 | >50 | 1.3 | 2.3 | 21.37 | >25 | >25 | >10 | >10 | >10 | >10 | 72 | 389 | R5 | | 2385 |  | |
| PRB958-06.TB1.4305 | 0.8 | 1.1 | 0.3 | 0.7 | >50 | >25 | >25 | >10 | >10 | >10 | >10 | 141 | 252 | R5 | | 3875 |  | |
| 1018-10.A5.1732 | 4.1 | 0.8 | 0.7 | 1.4 | 19.97 | >25 | >25 | >10 | >10 | >10 | >10 | 359 | 176 | R5 | | 181 |  | |
| 12008-09.B33.4830 | >50 | >50 | 1 | 5 | >50 | >25 | >25 | >10 | >10 | >10 | >10 | 147 | 835 | R5 | | 2594 |  | |
| PRB926-04.A9.4237 | 0.5 | >50 | 0.6 | 1.4 | 30.8 | >25 | >25 | >10 | >10 | >10 | >10 | 93 | 285 | R5 | | 14479 |  | |
| 9076-08.C14.4863 | >50 | 0.4 | 0.7 | 1.2 | 15.5 | >25 | >25 | >10 | >10 | >10 | >10 | 332 | 70 | R5 | | 460 |  | |
| 9077-12.B5.4655 | 8.7 | 6.1 | 2.6 | 9.3 | >50 | >25 | >25 | >10 | >10 | >10 | >10 | 300 | >1000 | R5 | | 952 |  | |
| SC20.8A8.2437 | 0.4 | >50 | 2.9 | 12.3 | >50 | >25 | >25 | >10 | >10 | >10 | >10 | 290 | 616 | R5 | | 3271 |  | |
| WITO.B10.2062 | 8.0 | 1.4 | 0.8 | 1.1 | >50 | >25 | 14.3 | >10 | >10 | >10 | >10 | 482 | >1000 | R5 | | 195 |  | |
| 700010077.A10.4429^1^ | 0.8 | 29.0 | 2.0 | 6.9 | 37 | >25 | >25 | >10 | >10 | >10 | >10 | 345 | 855 | R5/X4^3^ | | 133 |  | |
| 9032.08.A1.4685 | 6.5 | 6.4 | >50 | 16.0 | nd | nd | nd | nd | nd | nd | nd | >1000 | nd | R5 | | 43 |  | |
| MEMI.B6.3226^2^ | >50 | 29.6 | 9.6 | 4.2 | >50 | >25 | 16.78 | >10 | >10 | >10 | >10 | 228 | 498 | R5 | | 124 |  | |
| MEMI.E1.3237^2^ | >50 | 15.0 | 9.0 | 4.1 | >50 | >25 | 13.92 | >10 | >10 | >10 | >10 | 709 | 498 | R5 | | 808 |  | |
| MEMI.A6.3222^2^ | >50 | 20.7 | 10.5 | 4.1 | >50 | >25 | >25 | >10 | >10 | >10 | >10 | 720 | 815 | R5 | | 463 |  | |
|  |  |  |  |  |  |  |  |  |  |  |  |  |  |  | |  |  | |
| **Chronic Env clones** |  |  |  |  |  |  |  |  |  |  |  |  |  |  | |  |  | |
| SC24.3B4.2498 | 0.5 | 4.5 | 0.7 | 1.3 | >50 | >25 | >25 | >10 | >10 | >10 | >10 | 48 | 323 | X4 | | 767 |  | |
| SC24.3B10.2497 | 0.4 | 28.2 | 1.5 | 2.2 | >50 | >25 | >25 | >10 | >10 | >10 | >10 | 200 | 403 | X4 | | 1065 |  | |
| SC24.3E9.2500 | 0.1 | >50 | 2 | 3.5 | >50 | >25 | >25 | 5.4 | 7.2 | >10 | >10 | 545 | 260 | R5 | | 1385 |  | |
| SC24.3B9.2499 | 0.19 | 5.64 | 1.44 | 6.94 | >50 | >25 | >25 | >10 | >10 | >10 | >10 | >1000 | 166 | R5 | | 3448 |  | |
| SC25.8H5.2517 | 0.2 | >50 | 1.2 | 1.4 | 18.42 | >25 | >25 | >10 | >10 | >10 | >10 | 144 | 92 | R5 | | 558 |  | |
| SC24.8B3.2514 | 1.26 | >50 | 2.52 | 8.23 | >50 | >25 | >25 | >10 | >10 | >10 | >10 | 369 | 605 | R5 | | 1442 |  | |
| SC51.8A6.2684 | 0.6 | 1.4 | 0.4 | 0.8 | 37.09 | >25 | >25 | >10 | >10 | >10 | >10 | 66 | 514 | X4 | | 50 |  | |
| SC51.8A8.2685 | 0.2 | 0.3 | 0.7 | 1.1 | 9.68 | >25 | >25 | >10 | >10 | >10 | >10 | 452 | 277 | R5 | | 498 |  | |
| SC51.8A12.2681 | 0.48 | 0.54 | 0.65 | 1.49 | 15.48 | >25 | >25 | >10 | >10 | >10 | >10 | 186 | 375 | R5 | | 1833 |  | |
| SC05.8H2.3243 | 45.8 | >50 | 1.2 | 1.1 | >50 | >25 | >25 | >10 | >10 | >10 | >10 | 65 | 470 | R5 | | 3615 |  | |
| SC05.8A10.2362 | >50 | 5.2 | 1 | 1.7 | >50 | >25 | >25 | >10 | >10 | >10 | >10 | 791 | 603 | R5 | | 2375 |  | |
| SC05.8A11.2363 | >50 | >50 | 0.83 | 1.72 | >50 | >25 | >25 | >10 | >10 | >10 | >10 | 332 | 735 | R5 | | 4677 |  | |
| SC13.2G9.2402 | 0.3 | >50 | 1.2 | 1.7 | >50 | >25 | >25 | >10 | >10 | >10 | >10 | 181 | 703 | R5 | | 858 |  | |
| SC13.2H9.2406 | 0.5 | >50 | 1.1 | 2.2 | >50 | >25 | >25 | >10 | >10 | >10 | >10 | 424 | 372 | R5 | | 948 |  | |
| TT114P.3C6.2954 | 0.2 | 0.7 | 0.3 | 0.4 | 10.24 | >25 | >25 | >10 | >10 | >10 | >10 | 198 | 475 | R5/x4 | | 2479 |  | |
| TT114P.3E1.2955 | 0.3 | 1.3 | 0.4 | 0.7 | 11.55 | >25 | >25 | >10 | >10 | >10 | >10 | 266 | 450 | R5/x4 | | 1875 |  | |
| TT31P.2H3.2816 | 0.3 | 1.8 | 1.4 | 2.3 | 30.19 | >25 | >25 | >10 | >10 | >10 | >10 | 115 | 679 | R5 | | 4073 |  | |
| SAMI.A8.1863 | >50 | 2.1 | 2.2 | 5.4 | >50 | >25 | >25 | >10 | >10 | >10 | >10 | 470 | 500 | R5 | | 873 |  | |
| WICU.C1.2992 | >50 | >50 | 2.8 | 3.9 | 32.27 | >25 | >25 | >10 | >10 | >10 | >10 | 421 | 750 | R5 | | 888 |  | |
| WICU.B1.2971 | >50 | >50 | 1.6 | 3.3 | >50 | >25 | >25 | >10 | >10 | >10 | >10 | 141 | >1000 | R5 | | 18 |  | |
| WICU.B4.2973 | 15.1 | >50 | 0.3 | 1.0 | 38 | >25 | >25 | >10 | >10 | >10 | >10 | 184 | 697 | R5 | | 193 |  | |
| SC02.3D9.2293 | 3.5 | 1.7 | 1.3 | 2.5 | 17.9 | >25 | >25 | >10 | >10 | >10 | >10 | 442 | 654 | R5 | | 2146 |  | |
| SC02.3B7.2286 | 3.4 | 0.34 | 0.57 | 1.82 | 13.7 | >25 | >25 | >10 | >10 | >10 | >10 | 696 | 271 | R5 | | 2063 |  | |
| SC02.3A4.2283 | 2.14 | 0.79 | 0.63 | 1.55 | 14.24 | >25 | 8.9 | >10 | 5.4 | >10 | >10 | 477 | 361 | R5 | | 5792 |  | |
| SC02.3F2.2297 | 2.78 | 0.75 | 1.07 | 2.54 | 10.7 | >25 | 15 | >10 | >10 | >10 | >10 | 979 | 268 | R5 | | 819 |  | |
| SC03.8A9.2326 | 3.3 | 1.7 | 1.2 | 1.9 | 17.6 | >25 | >25 | >10 | >10 | >10 | >10 | 818 | 393 | R5 | | 3104 |  | |
| SC03.8C6.2327 | 0.31 | 13.24 | 2.09 | 5.14 | >50 | >25 | >25 | >10 | >10 | >10 | >10 | 697 | 823 | R5 | | 3542 |  | |
| SC03.4E5.2314 | 0.02 | >50 | 0.67 | 0.94 | 6.64 | 0.008 | 0.011 | 2.45 | 1.2 | 3.56 | 5.79 | 1.5 | 86 | R5 | | 272 |  | |
| TT103.2E4.2900 | 0.02 | 39.97 | 0.01 | 0.07 | 0.55 | >25 | >25 | 5.73 | >10 | 5.87 | 0.885 | 15 | 34 | R5 | | 688 |  | |
| TT103.2E1.2898 | 0.34 | 18.37 | 1.33 | 5.31 | 34.15 | >25 | >25 | >10 | >10 | >10 | >10 | 299 | 221 | R5 | | 2771 |  | |
| TT112P.3E6.2920 | 10.89 | 0.05 | 1.69 | 1.77 | 26.12 | 0.03 | 0.01 | >10 | >10 | >10 | 0.073 | 60 | 93 | R5 | | 1619 |  | |
| TT113PC.2G2.2941 | 48.3 | 2.8 | 1.7 | 2.6 | 45 | >25 | >25 | >10 | >10 | >10 | >10 | 377 | 355 | R5 | | 253 |  | |
| JOTO.TA1.2247 | >50 | >50 | 0.9 | 1.4 | >50 | >25 | >25 | >10 | >10 | >10 | >10 | 461 | 857 | X4 | | 245 |  | |
| SHKE.A26.4112 | >50 | 2.8 | 14.9 | 27.0 | >50 | >25 | >25 | >10 | >10 | >10 | >10 | 470 | 625 | R5 | | 2915 |  | |
| SHKE.A4.2116 | >50 | >50 | 19.7 | 35.7 | >50 | >25 | >25 | >10 | >10 | >10 | >10 | 219 | 539 | R5 | | 3094 |  | |
| SHKE.A7.2118 | >50 | 2.9 | 15.3 | 23.7 | >50 | >25 | >25 | >10 | >10 | >10 | >10 | 369 | 425 | R5 | | 2535 |  | |
| CRPE.B28.4072 | >50 | >50 | 36.6 | 5.7 | >50 | >25 | 1.88 | >10 | >10 | >10 | >10 | 388 | 569 | R5/X4 | | 758 |  | |
| OLLA.A14.1923 | >50 | 2.1 | 1.4 | 3.9 | >50 | >25 | >25 | >10 | >10 | >10 | >10 | 413 | >1000 | R5 | | 771 |  | |
| SMRE.A13.4127 | 2.1 | 0.6 | 2.4 | 3.3 | >50 | >25 | 7.99 | >10 | >10 | >10 | >10 | 189 | 479 | R5 | | 1800 |  | |
| 1632.TA9 | >50 | >50 | 4.0 | 4.9 | >50 | >25 | >25 | >10 | >10 | >10 | >10 | 978 | 921 | R5 | | 1876 |  | |
| 1632.A17 | >50 | >50 | 0.6 | 2.6 | >50 | >25 | >25 | >10 | >10 | >10 | >10 | 118 | 384 | R5 | | 188 |  | |
| 1632.A6 | >50 | >50 | 12.6 | 7.2 | >50 | >25 | >25 | >10 | >10 | >10 | >10 | 303 | >1000 | R5 | | 1931 |  | |
| 1632.TA1 | >50 | >50 | 30.0 | 4.2 | >50 | >25 | >25 | >10 | >10 | >10 | >10 | 522 | >1000 | R5 | | 775 |  | |
| 1632.A7 | >50 | >50 | 3.0 | 2.8 | >50 | >25 | >25 | >10 | >10 | >10 | >10 | 144 | 963 | R5 | | 533 |  | |
| 1632.A23 | >50 | >50 | 4.1 | 7.6 | >50 | >25 | >25 | >10 | >10 | >10 | >10 | 926 | >1000 | R5 | | 1283 |  | |
| 1588.TA3 | >50 | 14.5 | 0.9 | 2.2 | 32 | >25 | >25 | >10 | >10 | >10 | >10 | 780 | 525 | R5/X4 | | 821 |  | |
| 1588.A8 | >50 | >50 | 1.0 | 3.2 | 36 | >25 | >25 | >10 | >10 | >10 | >10 | 947 | 738 | R5 | | 1056 |  | |
| 1588.TA7 | >50 | >50 | 2.6 | 4.2 | 51 | >25 | >25 | >10 | >10 | >10 | >10 | >1000 | >1000 | R5 | | 2979 |  | |
| 1470.D27 | >50 | >50 | 3.1 | 2.6 | 47 | >25 | >25 | >10 | 7.15 | >10 | >10 | 247 | 345 | R5 | | 33 |  | |
| 1451.D17 | >50 | 0.5 | 4.1 | 5.1 | >50 | >25 | >25 | >10 | >10 | >10 | >10 | 605 | >1000 | R5 | | 227 |  | |
| 1451.C16 | >50 | 3.6 | >50 | 14.6 | >50 | >25 | >25 | >10 | >10 | >10 | >10 | >1000 | >1000 | R5 | | 2854 |  | |
| 1451.D1 | >50 | 0.8 | >50 | 14.8 | >50 | >25 | >25 | >10 | >10 | >10 | >10 | >1000 | >1000 | R5 | | 3292 |  | |
| 1451.C8 | >50 | 2.6 | >50 | 9.2 | nd | >25 | >25 | >10 | >10 | >10 | >10 | 813 | nd | R5 | | 2271 |  | |
| 1599.B11 | 29.1 | >50 | 11.1 | 10.9 | >50 | >25 | >25 | >10 | >10 | >10 | >10 | >1000 | >1000 | R5 | | 877 |  | |
| 1444.A21 | 2.3 | 8.7 | 1.1 | 2.1 | >50 | >25 | >25 | >10 | >10 | >10 | >10 | 129 | 801 | R5 | | 1690 |  | |
| YOMI.F2.4137 | >50 | 1.9 | 1.1 | 2.9 | >50 | >25 | >25 | >10 | >10 | >10 | >10 | 215 | >1000 | R5 | | 388 |  | |
| TALA.A2.1780 | >50 | >50 | 1.2 | 2.5 | 21 | >25 | 21 | >10 | >10 | >10 | >10 | >1000 | 281 | R5 | | 63 |  | |
|  | | |  |  |  |  |  |  |  |  |  |  |  |  |  | |  |  |
|  | | |  |  |  |  |  |  |  |  |  |  |  |  |  | |  |  |
|  | | | | | | |  |  |  |  |  |  |  |  |  | |  |  |

| ^1^ Not Transmitted virus. CTL escape mutant |  |  |  |  |
| --- | --- | --- | --- | --- |
| ^2^ Transmitted sequence not possible to identify |  |  |  |  |
| ^3^ Initially typed as R5 only in TZM-bl cells but subsequently typed as R5/X4 on NP-2 cells | | | | |

**Table S3. MAb and sCD4 IC50 data and coreceptor usage of SGA clones used in our study, comparing chronic versus acute sequences, augmenting the data set first described in Keele et al (ref 1).** The values given are 50% neutralization titers of monoclonal antibodies b12, 2G12, 2F5, 4E10, Z13e1, 447-52D, 447, F425, 17b, soluble CD4 (sCD4), and HIVIG, co-receptor usage, and infectious units per ul.
