# Supplementary material for: Recurrent Signature Patterns in HIV-1 B Clade Envelope Glycoproteins Associated with either Early or Chronic Infections
Source: PLoS Pathog. 2011 Sep 29;7(9):e1002209. doi: 10.1371/journal.ppat.1002209 (PMC3182927; doi:10.1371/journal.ppat.1002209)
Supplement: Table S4 — List of functional domain HXB2 positions. Combinations of sites in these functional regions were tested for correlations with acute/early versus chronic infection. The only one that provided a combination of sites that was significant was the CCR5coR model set, the set of variable positions proximal to the conserved CCR5 binding sites; it is indicated in bold. (DOC) [file ppat.1002209.s011.doc]

**gp120 sites**

CD4bs X-ray D368 E370 I371 N425 M426 W427 D457 G473

CD4bs Model L125 S128 T194 D279 A281 T283 S365 Q428 K429 N460 D474

CCR5coR Sodroski K117 K121 K207 N377 E381 R419 I420 K421 Q422 P438 R440 G441 R444

CCR5coR Mutation T123A/D T202D/G N279A P369A F383A R419A/D Q422A W427A P438A I439D R/S440D/A R444

**CCR5coR Model set Q114 L122 I201 Q203 A204 S209 N377 Y384 A436 P437**

CCR5coR V3 Loop N301A T303A R/S306A R308A P313A R315A F317A Y318A I323A D325A H330A

R5/X4 gp120 core R166K 195H N197D T198 200T A204 A221T I424V M434K R440D

R5/X4 V3 Loop N301 302 T303 306K/R I309R A316V 317 322K 323 I326

b12bs A281 S364 S365 G367 P369 T373 Y384 N386 P417 C418 R419 D474

main-main T257 N280 A281 S365 D368 P369 E370 I371 Y384 N386 P417 R419 V430 D457 D474 M475

main-side S256 G366 G367 C418 G431 R456 G458 P470 G471 G472 G473

side-side chain S364 V372 T373 K432 L453 T455

glycosylation cluster N262 S264 N295 T297 N332 S334 N339 T341 K362 S364 N386 T388 N392 T394 N448 T450

trimer interface E102 Q103 Q114 A204 V208 S209 E211 P214 H216 I491

gp41 interface set V36 Y40 V85 N92 T232 N234 P238 R252 F382 W427

gut mucosal homing S164 I165 V169 K171 Y177 K178 L179 D180 I181 I182 P183

**gp41 sites**

2F5 bs E662 L663 D664 K665 W666 A667

4E10 bs N671 W672 F673 N/D674 I675 T676 L679 W680

gp120 Interface, set 1 L555 Q562 L593 I595 W596 S599 V608 W610 S618

gp120 Interface, set 2 S528 M530 L555 Q562 L593 W596 G597 K601 V608 W610 S618

Fusion up-entry T605 P609 S613 E620 Q621

Fusion down-entry I595 L602 I603 K617

LLP1 virion incorp. I843 P844 R845 R846 I847 R848

LLP2 Tyr-sorting sig Y768 H769 R770 L771 R772

Expose CD4bs C764 L765 F766 S767 Y768 H769 R770 L771

**Table S4. List of functional domain HXB2 positions.** Combinations of sites in these functional regions were tested for correlations with acute/early versus chronic infection. The only one that provided a combination of sites that was significant was the CCR5coR model set, the set of variable positions proximal to the conserved CCR5 binding sites; it is indicated in bold.
