# Supplementary material for: Recurrent Signature Patterns in HIV-1 B Clade Envelope Glycoproteins Associated with either Early or Chronic Infections
Source: PLoS Pathog. 2011 Sep 29;7(9):e1002209. doi: 10.1371/journal.ppat.1002209 (PMC3182927; doi:10.1371/journal.ppat.1002209)
Supplement: Table S5 — All sequences generated for this study under Original Set , aligned. The first character of each sequence name indicates either the Fiebig stage at time of sampling, or C for chronic infection (see material and methods for details). The GenBank numbers are all included in the name of each sequence in the files; as there are thousands of sequences, and the numbers are not continuous, this seemed the most parsimonious presentation. (DOC) [file ppat.1002209.s012.doc]

2.BORI0637.UAB.EU576279 ATGAGAGCGAAGGAGATCAGGAAGAATTGTCAGCGCTTG---------TGGAGATGGGGC------------------------ACCATGCTCCTTGGGATGTTGATGATC------------TGTAGTGCTGCA---------GGCAATTTGTGGGTCACAGTCTATTATGGGGTACCTGTGTGGAAAGAAGCAACCACCACTTTATTTTGTGCATCAGATGCTAAAGCATATGATACAGAGGTACATAAT---GTTTGGGCCACACATGCCTGTGTACCCACAGACCCCAACCCACAAGAAGTAGTACTG---AAAAATGTGACAGAAAATTTTAACATGTGGAAAAATAACATGGTAGAACAGATGCATGAGGATATAATCAGTTTATGGGATCAAAGCCTAAAGCCATGTGTAAAACTAACTCCACTCTGTGTTACTTTAAATTGCACTGATTATTTGAATAATGCTACTAATACC------------------------------------------------------------------------------------------ACTAGTAGTAAGGAGGGAGAAATGAGAGGAGAAATAAAAAAATGCTCTTTCAATGTCACC---ACAAGAATAAGAGAT------AAGGTGCAGAAAGAATATGCACTTTTTTATAAACTTGATGTAGTACCAATAGGTAATGGT------------------------------AATACTAGC---------------------------TATAGGTTGATAAATTGTAATACCTCAGTCATTACACAGGCCTGTCCAAAGGTATCCTTTGAACCAATTCCCATACATTATTGTACCCCGGCTGGTTTTGCAATTCTAAAGTGT---AAAGATAAGAAGTTCAATGGAACAGGATCATGTAAAAATGTCAGCACAGTACAATGTACACATGGAATTAGACCAGTAGTGTCAACTCAACTGTTGTTAAATGGCAGTCTAGCAGAAGAA---GAGGTAGTAATTAGATCTGAAAATTTTACGAACAATGCTAAAACCATAATAGTACAGCTGAACGAAACTGTAGAAATTAATTGTACAAGACCCAACAACAATACAAGAAAAAGTATACATATAGGA------------CCAGGGAGA---ACATTTTATACAACAGGAGACATAATAGGAGATATAAGGCAGGCATATTGTATCCTT------AGTAGAGCAAAATGGGAAGACACTTTACAAAAGATAGTCACAAAATTAGGA---GAACAATATGGG---------AATAATAAAACA---ATAGTCTTTAATCAC---------TCCTCAGGAGGGGACCCAGAAATTGTAATGCACAGTTTTAATTGTGGAGGGGAATTTTTCTACTGTAATTCAACACAACTGTTTAATAGTACTTGGAGTTTT---------AATAGTACTTGG------------------------AATAAGAATTTTAATAGTACTTGG------------AATATTACTGAAGGGACA---AATAACACTGAA------ATCACACTCCCATGTAGAATAAAACAAATTATAAACATGTGGCAGGAAGTAGGAAAAGCAATGTATGCCCCTCCCATCAGAGGACAAATTAAATGTTCATCTAACATTACAGGGCTGCTATTAACAAGAGATGGTGGGGGTAATGGG---------AGCGGGACC---------------------------ACT---GAGGTCTTCAGACCTGGAGGGGGAGATATGAGGGACAATTGGAGA---AGTGAATTATACAAATATAAAGTAGTAAAAATTGAACCA---TTAGGAGTAGCACCT---ACCAAGGCAAAGAGAAGAGTGGTGCAAAGAGAA---AAAAGAGCAGTG---GGAGCAATAGGA---GCTATG---TTCCTT---GGG---------TTCTTGGGA---GCAGCAGGAAGCACTATGGGCGCAGCGTCAGTG---GCGCTGACGGTACAGGCCAGACAATTATTGTCTGGTATAGTGCAACAGCAGAACAATTTGCTGAGGGCTATTGAGGCGCAACAGCATCTGTTGCAACTCACAGTCTGGGGCATCAAGCAGCTCCAGGCAAGA---GTCCTGGCTGTGGAAAGATACCTAAAGGATCAACAGCTCCTAGGGATTTGGGGTTGCTCTGGAAAACTCATTTGCACCACTACTGTGCCTTGGAATACTAGTTGGAGT---------------------------AATAAATCTCTGGATGATATTTGGCAT---AACATGACCTGGATGCAGTGGGAAAAAGAGATTAAC------AATTACACAGACTTAATATACACCTTAATTGAAGAATCGCAAAACCAACAAGAAAAGAATGAACAAGAATTATTGGAATTAGATAAATGGGACAGTTTGTGGAATTGGTTTAACATAACAAACTGGCTGTGGTATATAAAAATATTCATAATGATAGTAGGAGGCTTGATAGGTTTAAGAATAGTTTTTATTGTACTTTCTGTAGTGAATAGAGTTAGGCAGGGATACTCACCATTATCATTTCAGACCCGC---CTCCCAGTCCCGAGGGAA------CCCGACAGGCCCGAAGGAACAGAAGAAGAAGGTGGAGAGAAAGACAGAGGCAGATCCGGACGTTTAGTGGATGGATTCTTAGCACTTATCTGGGACGATCTGCGGAGCCTGTTCCTCTTCAGCTACCACCGCTTGAGAGACTTACTCTTGATTGTAGCGAGGATTGTGGAACTTCTGGGACGCAGG---------------GGGTGGGGAGTTCTCAAATATTGGTGG---AATCTCCTGCAGTATTGG---------------------------------------------------AGTCAGGAACTAAAGAATAGTGCTGTTAGCTTGCTCAATGCCACAGCTATCGCAGTAGCTGAGGGGACAGATAGGGTTATAGAAATATTACAAAGA------------------ATTTATAGAGCTTTTCTCCACATACCTAGAAGAATAAGACAGGGCCTCGAAAGGGCTTTGTTATAA

2.BORI0637.UAB.EU576287 ATGAGAGCGAAGGAGATCAGGAAGAATTGTCAGCGCTTG---------TGGAGATGGGGC------------------------ACCATGCTCCTTGGGATGTTGATGATC------------TGTAGTGCTGCA---------GGCAATTTGTGGGTCACAGTCTATTATGGGGTACCTGTGTGGAAAGAAGCAACCACCACTTTATTTTGTGCATCAGATGCTAAAGCATATGATACAGAGGTACATAAT---GTTTGGGCCACACATGCCTGTGTACCCACAGACCCCAACCCACAAGAAGTAGTACTG---AAAAATGTGACAGAAAATTTTAACATGTGGAAAAATAACATGGTAGAACAGATGCATGAGGATATAATCAGTTTATGGGATCAAAGCCTAAAGCCATGTGTAAAACTAACTCCACTCTGTGTTACTTTAAATTGCACTGATTATTTGAATAATGCTACTAATACC------------------------------------------------------------------------------------------ACTAGTAGTAAGGAGGGAGAAATGAGAGGAGAAATAAAAAAATGCTCTTTCAATGTCACC---ACAAGAATAAGAGAT------AAGGTGCAGAAAGAATATGCACTTTTTTATAAACTTGATGTAGTACCAATAGGTAATGGT------------------------------AATACTAGC---------------------------TATAGGTTGATAAATTGTAATACCTCAGTCATTACACAGGCCTGTCCAAAGGTATCCTTTGAACCAATTCCCATACATTATTGTACCCCGGCTGGTTTTGCAATTCTAAAGTGT---AAAGATAAGAAGTTCAATGGAACAGGATCATGTAAAAATGTCAGCACAGTACAATGTACACATGGAATTAGACCAGTAGTGTCAACTCAACTGTTGTTAAATGGCAGTCTAGCAGAAGAA---GAGGTAGTAATTAGATCTGAAAATTTTACGAACAATGCTAAAACCATAATAGTACAGCTGAACGAAACTGTAGAAATTAATTGTACAAGACCCAACAACAATACAAGAAAAAGTATACATATAGGA------------CCAGGGAGA---ACATTTTATACAACAGGAGACATAATAGGAGATATAAGGCAGGCATATTGTATCCTT------AGTAGAGCAAAATGGGAAGACACTTTACAAAAGATAGTCACAAAATTAGGA---GAACAATATGGG---------AATAATAAAACA---ATAGTCTTTAATCAC---------TCCTCAGGAGGGGACCCAGAAATTGTAATGCACAGTTTTAATTGTGGAGGGGAATTTTTCTACTGTAATTCAACACAACTGTTTAATAGTACTTGGAGTTTT---------AATAGTACTTGG------------------------AATAAGAATTTTAATAGTACTTGG------------AATATTACTGAAGGGACA---AATAACACTGAA------ATCACACTCCCATGTAGAATAAAACAAATTATAAACATGTGGCAGGAAGTAGGAAAAGCAATGTATGCCCCTCCCATCAGAGGACAAATTAAATGTTCATCTAACATTACAGGGCTGCTATTAACAAGAGATGGTGGGGGTAATGGG---------AGCGGGACC---------------------------ACT---GAGGTCTTCAGACCTGGAGGGGGAGATATGAGGGACAATTGGAGA---AGTGAATTATACAAATATAAAGTAGTAAAAATTGAACCA---TTAGGAGTAGCACCT---ACCAAGGCAAAGAGAAGAGTGGTGCAAAGAGAA---AAAAGAGCAGTG---GGAGCAATAGGA---GCTATG---TTCCTT---GGG---------TTCTTGGGA---GCAGCAGGAAGCACTATGGGCGCAGCGTCAGTG---GCGCTGACGGTACAGGCCAGACAATTATTGTCTGGTATAGTGCAACAGCAGAACAATTTGCTGAGGGCTATTGAGGCGCAACAGCATCTGTTGCAACTCACAGTCTGGGGCATCAAGCAGCTCCAGGCAAGA---GTCCTGGCTGTGGAAAGATACCTAAAGGATCAACAGCTCCTAGGGATTTGGGGTTGCTCTGGAAAACTCATTTGCACCACTACTGTGCCTTGGAATACTAGTTGGAGT---------------------------AATAAATCTCTGGATGATATTTGGCAT---AACATGACCTGGATGCAGTGGGAAAAAGAGATTAAC------AATTACACAGACTTAATATACACCTTAATTGAAGAATCGCAAAACCAACAAGAAAAGAATGAACAAGAATTATTGGAATTAGATAAATGGGACAGTTTGTGGAATTGGTTTAACATAACAAACTGGCTGTGGTATATAAAAATATTCATAATGATAGTAGGAGGCTTGATAGGTTTAAGAATAGTTTTTATTGTACTTTCTGTAGTGAATAGAGTTAGGCAGGGATACTCACCATTATCATTTCAGACCCGC---CTCCCAGTCCCGAGGGAA------CCCGACAGGCCCGAAGGAACAGAAGAAGAAGGTGGAGAGAAAGACAGAGGCAGATCCGGACGTTTAGTGGATGGATTCTTAGCACTTATCTGGGACGATCTGCGGAGCCTGTTCCTCTTCAGCTACCACCGCTTGAGAGACTTACTCTTGATTGTAGCGAGGATTGTGGAACTTCTGGGACGCAGG---------------GGGTGGGGAGTTCTCAAATATTGGTGG---AATCTCCTGCAGTATTGG---------------------------------------------------AGTCAGGAACTAAAGAATAGTGCTGTTAGCTTGCTCAATGCCACAGCTATCGCAGTAGCTGAGGGGACAGATAGGGTTATAGAAATATTACAAAGA------------------ATTTATAGAGCTTTTCTCCACATACCTAGAAGAATAAGACAGGGCCTCGAAAGGGCTTTGTTATAA

2.BORI0637.UAB.EU576276 ATGAGAGCGAAGGAGATCAGGAAGAATTGTCAGCGCTTG---------TGGAGATGGGGC------------------------ACCATGCTCCTTGGGATGTTGATGATC------------TGTAGTGCTGCA---------GGCAATTTGTGGGTCACAGTCTATTATGGGGTACCTGTGTGGAAAGAAGCAACCACCACTTTATTTTGTGCATCAGATGCTAAAGCATATGATACAGAGGTACATAAT---GTTTGGGCCACACATGCCTGTGTACCCACAGACCCCAACCCACAAGAAGTAGTACTG---AAAAATGTGACAGAAAATTTTAACATGTGGAAAAATAACATGGTAGAACAGATGCATGAGGATATAATCAGTTTATGGGATCAAAGCCTAAAGCCATGTGTAAAACTAACTCCACTCTGTGTTACTTTAAATTGCACTGATTATTTGAATAATGCTACTAATACC------------------------------------------------------------------------------------------ACTAGTAGTAAGGAGGGAGAAATGAGAGGAGAAATAAAAAAATGCTCTTTCAATGTCACC---ACAAGAATAAGAGAT------AAGGTGCAGAAAGAATATGCACTTTTTTATAAACTTGATGTAGTACCAATAGGTAATGGT------------------------------AATACTAGC---------------------------TATAGGTTGATAAATTGTAATACCTCAGTCATTACACAGGCCTGTCCAAAGGTATCCTTTGAACCAATTCCCATACATTATTGTACCCCGGCTGGTTTTGCAATTCTAAAGTGT---AAAGATAAGAAGTTCAATGGAACAGGATCATGTAAAAATGTCAGCACAGTACAATGTACACATGGAATTAGACCAGTAGTGTCAACTCAACTGTTGTTAAATGGCAGTCTAGCAGAAGAA---GAGGTAGTAATTAGATCTGAAAATTTTACGAACAATGCTAAAACCATAATAGTACAGCTGAACGAAACTGTAGAAATTAATTGTACAAGACCCAACAACAATACAAGAAAAAGTATACATATAGGA------------CCAGGGAGA---ACATTTTATACAACAGGAGACATAATAGGAGATATAAGGCAGGCATATTGTATCCTT------AGTAGAGCAAAATGGGAAGACACTTTACAAAAGATAGTCACAAAATTAGGA---GAACAATATGGG---------AATAATAAAACA---ATAGTCTTTAATCAC---------TCCTCAGGAGGGGACCCAGAAATTGTAATGCACAGTTTTAATTGTGGAGGGGAATTTTTCTACTGTAATTCAACACAACTGTTTAATAGTACTTGGAGTTTT---------AATAGTACTTGG------------------------AATAAGAATTTTAATAGTACTTGG------------AATATTACTGAAGGGACA---AATAACACTGAA------ATCACACTCCCATGTAGAATAAAACAAATTATAAACATGTGGCAGGAAGTAGGAAAAGCAATGTATGCCCCTCCCATCAGAGGACAAATTAAATGTTCATCTAACATTACAGGGCTGCTATTAACAAGAGATGGTGGGGGTAATGGG---------AGCGGGACC---------------------------ACT---GAGGTCTTCAGACCTGGAGGGGGAGATATGAGGGACAATTGGAGA---AGTGAATTATACAAATATAAAGTAGTAAAAATTGAACCA---TTAGGAGTAGCACCT---ACCAAGGCAAAGAGAAGAGTGGTGCAAAGAGAA---AAAAGAGCAGTG---GGAGCAATAGGA---GCTATG---TTCCTT---GGG---------TTCTTGGGA---GCAGCAGGAAGCACTATGGGCGCAGCGTCAGTG---GCGCTGACGGTACAGGCCAGACAATTATTGTCTGGTATAGTGCAACAGCAGAACAATTTGCTGAGGGCTATTGAGGCGCAACAGCATCTGTTGCAACTCACAGTCTGGGGCATCAAGCAGCTCCAGGCAAGA---GTCCTGGCTGTGGAAAGATACCTAAAGGATCAACAGCTCCTAGGGATTTGGGGTTGCTCTGGAAAACTCATTTGCACCACTACTGTGCCTTGGAATACTAGTTGGAGT---------------------------AATAAATCTCTGGATGATATTTGGCAT---AACATGACCTGGATGCAGTGGGAAAAAGAGATTAAC------AATTACACAGACTTAATATACACCTTAATTGAAGAATCGCAAAACCAACAAGAAAAGAATGAACAAGAATTATTGGAATTAGATAAATGGGACAGTTTGTGGAATTGGTTTAACATAACAAACTGGCTGTGGTATATAAAAATATTCATAATGATAGTAGGAGGCTTGATAGGTTTAAGAATAGTTTTTATTGTACTTTCTGTAGTGAATAGAGTTAGGCAGGGATACTCACCATTATCATTTCAGACCCGC---CTCCCAGTCCCGAGGGAA------CCCGACAGGCCCGAAGGAACAGAAGAAGAAGGTGGAGAGAAAGACAGAGGCAGATCCGGACGTTTAGTGGATGGATTCTTAGCACTTATCTGGGACGATCTGCGGAGCCTGTTCCTCTTCAGCTACCACCGCTTGAGAGACTTACTCTTGATTGTAGCGAGGATTGTGGAACTTCTGGGACGCAGG---------------GGGTGGGGAGTTCTCAAATATTGGTGG---AATCTCCTGCAGTATTGG---------------------------------------------------AGTCAGGAACTAAAGAATAGTGCTGTTAGCTTGCTCAATGCCACAGCTATCGCAGTAGCTGAGGGGACAGATAGGGTTATAGAAATATTACAAAGA------------------ATTTATAGAGCTTTTCTCCACATACCTAGAAGAATAAGACAGGGCCTCGAAAGGGCTTTGTTATAA

2.BORI0637.UAB.EU576299 ATGAGAGCGAAGGAGATCAGGAAGAATTGTCAGCGCTTG---------TGGAGATGGGGC------------------------ACCATGCTCCTTGGGATGTTGATGATC------------TGTAGTGCTGCA---------GGCAATTTGTGGGTCACAGTCTATTATGGGGTACCTGTGTGGAAAGAAGCAACCACCACTTTATTTTGTGCATCAGATGCTAAAGCATATGATACAGAGGTACATAAT---GTTTGGGCCACACATGCCTGTGTACCCACAGACCCCAACCCACAAGAAGTAGTACTG---AAAAATGTGACAGAAAATTTTAACATGTGGAAAAATAACATGGTAGAACAGATGCATGAGGATATAATCAGTTTATGGGATCAAAGCCTAAAGCCATGTGTAAAACTAACTCCACTCTGTGTTACTTTAAATTGCACTGATTATTTGAATAATGCTACTAATACC------------------------------------------------------------------------------------------ACTAGTAGTAAGGAGGGAGAAATGAGAGGAGAAATAAAAAAATGCTCTTTCAATGTCACC---ACAAGAATAAGAGAT------AAGGTGCAGAAAGAATATGCACTTTTTTATAAACTTGATGTAGTACCAATAGGTAATGGT------------------------------AATACTAGC---------------------------TATAGGTTGATAAATTGTAATACCTCAGTCATTACACAGGCCTGTCCAAAGGTATCCTTTGAACCAATTCCCATACATTATTGTACCCCGGCTGGTTTTGCAATTCTAAAGTGT---AAAGATAAGAAGTTCAATGGAACAGGATCATGTAAAAATGTCAGCACAGTACAATGTACACATGGAATTAGACCAGTAGTGTCAACTCAACTGTTGTTAAATGGCAGTCTAGCAGAAGAA---GAGGTAGTAATTAGATCTGAAAATTTTACGAACAATGCTAAAACCATAATAGTACAGCTGAACGAAACTGTAGAAATTAATTGTACAAGACCCAACAACAATACAAGAAAAAGTATACATATAGGA------------CCAGGGAGA---ACATTTTATACAACAGGAGACATAATAGGAGATATAAGGCAGGCATATTGTATCCTT------AGTAGAGCAAAATGGGAAGACACTTTACAAAAGATAGTCACAAAATTAGGA---GAACAATATGGG---------AATAATAAAACA---ATAGTCTTTAATCAC---------TCCTCAGGAGGGGACCCAGAAATTGTAATGCACAGTTTTAATTGTGGAGGGGAATTTTTCTACTGTAATTCAACACAACTGTTTAATAGTACTTGGAGTTTT---------AATAGTACTTGG------------------------AATAAGAATTTTAATAGTACTTGG------------AATATTACTGAAGGGACA---AATAACACTGAA------ATCACACTCCCATGTAGAATAAAACAAATTATAAACATGTGGCAGGAAGTAGGAAAAGCAATGTATGCCCCTCCCATCAGAGGACAAATTAAATGTTCATCTAACATTACAGGGCTGCTATTAACAAGAGATGGTGGGGGTAATGGG---------AGCGGGACC---------------------------ACT---GAGGTCTTCAGACCTGGAGGGGGAGATATGAGGGACAATTGGAGA---AGTGAATTATACAAATATAAAGTAGTAAAAATTGAACCA---TTAGGAGTAGCACCT---ACCAAGGCAAAGAGAAGAGTGGTGCAAAGAGAA---AAAAGAGCAGTG---GGAGCAATAGGA---GCTATG---TTCCTT---GGG---------TTCTTGGGA---GCAGCAGGAAGCACTATGGGCGCAGCGTCAGTG---GCGCTGACGGTACAGGCCAGACAATTATTGTCTGGTATAGTGCAACAGCAGAACAATTTGCTGAGGGCTATTGAGGCGCAACAGCATCTGTTGCAACTCACAGTCTGGGGCATCAAGCAGCTCCAGGCAAGA---GTCCTGGCTGTGGAAAGATACCTAAAGGATCAACAGCTCCTAGGGATTTGGGGTTGCTCTGGAAAACTCATTTGCACCACTACTGTGCCTTGGAATACTAGTTGGAGT---------------------------AATAAATCTCTGGATGATATTTGGCAT---AACATGACCTGGATGCAGTGGGAAAAAGAGATTAAC------AATTACACAGACTTAATATACACCTTAATTGAAGAATCGCAAAACCAACAAGAAAAGAATGAACAAGAATTATTGGAATTAGATAAATGGGACAGTTTGTGGAATTGGTTTAACATAACAAACTGGCTGTGGTATATAAAAATATTCATAATGATAGTAGGAGGCTTGATAGGTTTAAGAATAGTTTTTATTGTACTTTCTGTAGTGAATAGAGTTAGGCAGGGATACTCACCATTATCATTTCAGACCCGC---CTCCCAGTCCCGAGGGAA------CCCGACAGGCCCGAAGGAACAGAAGAAGAAGGTGGAGAGAAAGACAGAGGCAGATCCGGACGTTTAGTGGATGGATTCTTAGCACTTATCTGGGACGATCTGCGGAGCCTGTTCCTCTTCAGCTACCACCGCTTGAGAGACTTACTCTTGATTGTAGCGAGGATTGTGGAACTTCTGGGACGCAGG---------------GGGTGGGGAGTTCTCAAATATTGGTGG---AATCTCCTGCAGTATTGG---------------------------------------------------AGTCAGGAACTAAAGAATAGTGCTGTTAGCTTGCTCAATGCCACAGCTATCGCAGTAGCTGAGGGGACAGATAGGGTTATAGAAATATTACAAAGA------------------ATTTATAGAGCTTTTCTCCACATACCTAGAAGAATAAGACAGGGCCTCGAAAGGGCTTTGTTATAA

2.BORI0637.UAB.EU576278 ATGAGAGCGAAGGAGATCAGGAAGAATTGTCAGCGCTTG---------TGGAGATGGGGC------------------------ACCATGCTCCTTGGGATGTTGATGATC------------TGTAGTGCTGCA---------GGCAATTTGTGGGTCACAGTCTATTATGGGGTACCTGTGTGGAAAGAAGCAACCACCACTTTATTTTGTGCATCAGATGCTAAAGCATATGATACAGAGGTACATAAT---GTTTGGGCCACACATGCCTGTGTACCCACAGACCCCAACCCACAAGAAGTAGTACTG---AAAAATGTGACAGAAAATTTTAACATGTGGAAAAATAACATGGTAGAACAGATGCATGAGGATATAATCAGTTTATGGGATCAAAGCCTAAAGCCATGTGTAAAACTAACTCCACTCTGTGTTACTTTAAATTGCACTGATTATTTGAATAATGCTACTAATACC------------------------------------------------------------------------------------------ACTAGTAGTAAGGAGGGAGAAATGAGAGGAGAAATAAAAAAATGCTCTTTCAATGTCACC---ACAAGAATAAGAGAT------AAGGTGCAGAAAGAATATGCACTTTTTTATAAACTTGATGTAGTACCAATAGGTAATGGT------------------------------AATACTAGC---------------------------TATAGGTTGATAAATTGTAATACCTCAGTCATTACACAGGCCTGTCCAAAGGTATCCTTTGAACCAATTCCCATACATTATTGTACCCCGGCTGGTTTTGCAATTCTAAAGTGT---AAAGATAAGAAGTTCAATGGAACAGGATCATGTAAAAATGTCAGCACAGTACAATGTACACATGGAATTAGACCAGTAGTGTCAACTCAACTGTTGTTAAATGGCAGTCTAGCAGAAGAA---GAGGTAGTAATTAGATCTGAAAATTTTACGAACAATGCTAAAACCATAATAGTACAGCTGAACGAAACTGTAGAAATTAATTGTACAAGACCCAACAACAATACAAGAAAAAGTATACATATAGGA------------CCAGGGAGA---ACATTTTATACAACAGGAGACATAATAGGAGATATAAGGCAGGCATATTGTATCCTT------AGTAGAGCAAAATGGGAAGACACTTTACAAAAGATAGTCACAAAATTAGGA---GAACAATATGGG---------AATAATAAAACA---ATAGTCTTTAATCAC---------TCCTCAGGAGGGGACCCAGAAATTGTAATGCACAGTTTTAATTGTGGAGGGGAATTTTTCTACTGTAATTCAACACAACTGTTTAATAGTACTTGGAGTTTT---------AATAGTACTTGG------------------------AATAAGAATTTTAATAGTACTTGG------------AATATTACTGAAGGGACA---AATAACACTGAA------ATCACACTCCCATGTAGAATAAAACAAATTATAAACATGTGGCAGGAAGTAGGAAAAGCAATGTATGCCCCTCCCATCAGAGGACAAATTAAATGTTCATCTAACATTACAGGGCTGCTATTAACAAGAGATGGTGGGGGTAATGGG---------AGCGGGACC---------------------------ACT---GAGGTCTTCAGACCTGGAGGGGGAGATATGAGGGACAATTGGAGA---AGTGAATTATACAAATATAAAGTAGTAAAAATTGAACCA---TTAGGAGTAGCACCT---ACCAAGGCAAAGAGAAGAGTGGTGCAAAGAGAA---AAAAGAGCAGTG---GGAGCAATAGGA---GCTATG---TTCCTT---GGG---------TTCTTGGGA---GCAGCAGGAAGCACTATGGGCGCAGCGTCAGTG---GCGCTGACGGTACAGGCCAGACAATTATTGTCTGGTATAGTGCAACAGCAGAACAATTTGCTGAGGGCTATTGAGGCGCAACAGCATCTGTTGCAACTCACAGTCTGGGGCATCAAGCAGCTCCAGGCAAGA---GTCCTGGCTGTGGAAAGATACCTAAAGGATCAACAGCTCCTAGGGATTTGGGGTTGCTCTGGAAAACTCATTTGCACCACTACTGTGCCTTGGAATACTAGTTGGAGT---------------------------AATAAATCTCTGGATGATATTTGGCAT---AACATGACCTGGATGCAGTGGGAAAAAGAGATTAAC------AATTACACAGACTTAATATACACCTTAATTGAAGAATCGCAAAACCAACAAGAAAAGAATGAACAAGAATTATTGGAATTAGATAAATGGGACAGTTTGTGGAATTGGTTTAACATAACAAACTGGCTGTGGTATATAAAAATATTCATAATGATAGTAGGAGGCTTGATAGGTTTAAGAATAGTTTTTATTGTACTTTCTGTAGTGAATAGAGTTAGGCAGGGATACTCACCATTATCATTTCAGACCCGC---CTCCCAGTCCCGAGGGAA------CCCGACAGGCCCGAAGGAACAGAAGAAGAAGGTGGAGAGAAAGACAGAGGCAGATCCGGACGTTTAGTGGATGGATTCTTAGCACTTATCTGGGACGATCTGCGGAGCCTGTTCCTCTTCAGCTACCACCGCTTGAGAGACTTACTCTTGATTGTAGCGAGGATTGTGGAACTTCTGGGACGCAGG---------------GGGTGGGGAGTTCTCAAATATTGGTGG---AATCTCCTGCAGTATTGG---------------------------------------------------AGTCAGGAACTAAAGAATAGTGCTGTTAGCTTGCTCAATGCCACAGCTATCGCAGTAGCTGAGGGGACAGATAGGGTTATAGAAATATTACAAAGA------------------ATTTATAGAGCTTTTCTCCACATACCTAGAAGAATAAGACAGGGCCTCGAAAGGGCTTTGTTATAA

2.BORI0637.UAB.EU576295 ATGAGAGCGAAGGAGATCAGGAAGAATTGTCAGCGCTTG---------TGGAGATGGGGC------------------------ACCATGCTCCTTGGGATGTTGATGATC------------TGTAGTGCTGCA---------GGCAATTTGTGGGTCACAGTCTATTATGGGGTACCTGTGTGGAAAGAAGCAACCACCACTTTATTTTGTGCATCAGATGCTAAAGCATATGATACAGAGGTACATAAT---GTTTGGGCCACACATGCCTGTGTACCCACAGACCCCAACCCACAAGAAGTAGTACTG---AAAAATGTGACAGAAAATTTTAACATGTGGAAAAATAACATGGTAGAACAGATGCATGAGGATATAATCAGTTTATGGGATCAAAGCCTAAAGCCATGTGTAAAACTAACTCCACTCTGTGTTACTTTAAATTGCACTGATTATTTGAATAATGCTACTAATACC------------------------------------------------------------------------------------------ACTAGTAGTAAGGAGGGAGAAATGAGAGGAGAAATAAAAAAATGCTCTTTCAATGTCACC---ACAAGAATAAGAGAT------AAGGTGCAGAAAGAATATGCACTTTTTTATAAACTTGATGTAGTACCAATAGGTAATGGT------------------------------AATACTAGC---------------------------TATAGGTTGATAAATTGTAATACCTCAGTCATTACACAGGCCTGTCCAAAGGTATCCTTTGAACCAATTCCCATACATTATTGTACCCCGGCTGGTTTTGCAATTCTAAAGTGT---AAAGATAAGAAGTTCAATGGAACAGGATCATGTAAAAATGTCAGCACAGTACAATGTACACATGGAATTAGACCAGTAGTGTCAACTCAACTGTTGTTAAATGGCAGTCTAGCAGAAGAA---GAGGTAGTAATTAGATCTGAAAATTTTACGAACAATGCTAAAACCATAATAGTACAGCTGAACGAAACTGTAGAAATTAATTGTACAAGACCCAACAACAATACAAGAAAAAGTATACATATAGGA------------CCAGGGAGA---ACATTTTATACAACAGGAGACATAATAGGAGATATAAGGCAGGCATATTGTATCCTT------AGTAGAGCAAAATGGGAAGACACTTTACAAAAGATAGTCACAAAATTAGGA---GAACAATATGGG---------AATAATAAAACA---ATAGTCTTTAATCAC---------TCCTCAGGAGGGGACCCAGAAATTGTAATGCACAGTTTTAATTGTGGAGGGGAATTTTTCTACTGTAATTCAATACAACTGTTTAATAGTACTTGGAGTTTT---------AATAGTACTTGG------------------------AATAAGAATTTTAATAGTACTTGG------------AATATTACTGAAGGGACA---AATAACACTGAA------ATCACACTCCCATGTAGAATAAAACAAATTATAAACATGTGGCAGGAAGTAGGAAAAGCAATGTATGCCCCTCCCATCAGAGGACAAATTAAATGTTCATCTAACATTACAGGGCTGCTATTAACAAGAGATGGTGGGGGTAATGGG---------AGCGGGACC---------------------------ACT---GAGGTCTTCAGACCTGGAGGGGGAGATATGAGGGACAATTGGAGA---AGTGAATTATACAAATATAAAGTAGTAAAAATTGAACCA---TTAGGAGTAGCACCT---ACCAAGGCAAAGAGAAGAGTGGTGCAAAGAGAA---AAAAGAGCAGTG---GGAGCAATAGGA---GCTATG---TTCCTT---GGG---------TTCTTGGGA---GCAGCAGGAAGCACTATGGGCGCAGCGTCAGTG---GCGCTGACGGTACAGGCCAGACAATTATTGTCTGGTATAGTGCAACAGCAGAACAATTTGCTGAGGGCTATTGAGGCGCAACAGCATCTGTTGCAACTCACAGTCTGGGGCATCAAGCAGCTCCAGGCAAGA---GTCCTGGCTGTGGAAAGATACCTAAAGGATCAACAGCTCCTAGGGATTTGGGGTTGCTCTGGAAAACTCATTTGCACCACTACTGTGCCTTGGAATACTAGTTGGAGT---------------------------AATAAATCTCTGGATGATATTTGGCAT---AACATGACCTGGATGCAGTGGGAAAAAGAGATTAAC------AATTACACAGACTTAATATACACCTTAATTGAAGAATCGCAAAACCAACAAGAAAAGAATGAACAAGAATTATTGGAATTAGATAAATGGGACAGTTTGTGGAATTGGTTTAACATAACAAACTGGCTGTGGTATATAAAAATATTCATAATGATAGTAGGAGGCTTGATAGGTTTAAGAATAGTTTTTATTGTACTTTCTGTAGTGAATAGAGTTAGGCAGGGATACTCACCATTATCATTTCAGACCCGC---CTCCCAGTCCCGAGGGAA------CCCGACAGGCCCGAAGGAACAGAAGAAGAAGGTGGAGAGAAAGACAGAGGCAGATCCGGACGTTTAGTGGATGGATTCTTAGCACTTATCTGGGACGATCTGCGGAGCCTGTTCCTCTTCAGCTACCACCGCTTGAGAGACTTACTCTTGATTGTAGCGAGGATTGTGGAACTTCTGGGACGCAGG---------------GGGTGGGGAGTTCTCAAATATTGGTGG---AATCTCCTGCAGTATTGG---------------------------------------------------AGTCAGGAACTAAAGAATAGTGCTGTTAGCTTGCTCAATGCCACAGCTATCGCAGTAGCTGAGGGGACAGATAGGGTTATAGAAATATTACAAAGA------------------ATTTATAGAGCTTTTCTCCACATACCTAGAAGAATAAGACAGGGCCTCGAAAGGGCTTTGTTATAA

2.BORI0637.UAB.EU576301 ATGAGAGCGAAGGAGATCAGGAAGAATTGTCAGCGCTTG---------TGGAGATGGGGC------------------------ACCATGCTCCTTGGGATGTTGATGATC------------TGTAGTGCTGCA---------GGCAATTTGTGGGTCACAGTCTATTATGGGGTACCTGTGTGGAAAGAAGCAACCACCACTTTATTTTGTGCATCAGATGCTAAAGCATATGATACAGAGGTACATAAT---GTTTGGGCCACACATGCCTGTGTACCCACAGACCCCAACCCACAAGAAGTAGTACTG---AAAAATGTGACAGAAAATTTTAACATGTGGAAAAATAACATGGTAGAACAGATGCATGAGGATATAATCAGTTTATGGGATCAAAGCCTAAAGCCATGTGTAAAACTAACTCCACTCTGTGTTACTTTAAATTGCACTGATTATTTGAATAATGCTACTAATACC------------------------------------------------------------------------------------------ACTAGTAGTAAGGAGGGAGAAATGAGAGGAGAAATAAAAAAATGCTCTTTCAATGTCACC---ACAAGAATAAGAGAT------AAGGTGCAGAAAGAATATGCACTTTTTTATAAACTTGATGTAGTACCAATAGGTAATGGT------------------------------AATACTAGC---------------------------TATAGGTTGATAAATTGTAATACCTCAGTCATTACACAGGCCTGTCCAAAGGTATCCTTTGAACCAATTCCCATACATTATTGTACCCCGGCTGGTTTTGCAATTCTAAAGTGT---AAAGATAAGAAGTTCAATGGAACAGGATCATGTAAAAATGTCAGCACAGTACAATGTACACATGGAATTAGACCAGTAGTGTCAACTCAACTGTTGTTAAATGGCAGTCTAGCAGAAGAA---GAGGTAGTAATTAGATCTGAAAATTTTACGAACAATGCTAAAACCATAATAGTACAGCTGAACGAAACTGTAGAAATTAATTGTACAAGACCCAACAACAATACAAGAAAAAGTATACATATAGGA------------CCAGGGAGA---ACATTTTATACAACAGGAGACATAATAGGAGATATAAGGCAGGCATATTGTATCCTT------AGTAGAGCAAAATGGGAAGACACTTTACAAAAGATAGTCACAAAATTAGGA---GAACAATATGGG---------AATAATAAAACA---ATAGTCTTTAATCAC---------TCCTCAGGAGGGGACCCAGAAATTGTAATGCACAGTTTTAATTGTGGAGGGGAATTTTTCTACTGTAATTCAACACAACTGTTTAATAGTACTTGGAGTTTT---------AATAGTACTTGG------------------------AATAAGAATTTTAATAGTACTTGG------------AATATTACTGAAGGGACA---AATAACACTGAA------ATCACACTCCCATGTAGAATAAAACAAATTATAAACATGTGGCAGGAAGTAGGAAAAGCAATGTATGCCCCTCCCATCAGAGGACAAATTAAATGTTCATCTAACATTACAGGGCTGCTATTAACAAGAGATGGTGGGGGTAATGGG---------AGCGGGACC---------------------------ACT---GAGGTCTTCAGACCTGGAGGGGGAGATATGAGGGACAATTGGAGA---AGTGAATTATACAAATATAAAGTAGTAAAAATTGAACCA---TTAGGAGTAGCACCT---ACCAAGGCAAAGAGAAGAGTGGTGCAAAGAGAA---AAAAGAGCAGTG---GGAGCAATAGGA---GCTATG---TTCCTT---GGG---------TTCTTGGGA---GCAGCAGGAAGCACTATGGGCGCAGCGTCAGTG---GCGCTGACGGTACAGGCCAGACAATTATTGTCTGGTATAGTGCAACAGCAGAACAATTTGCTGAGGGCTATCGAGGCGCAACAGCATCTGTTGCAACTCACAGTCTGGGGCATCAAGCAGCTCCAGGCAAGA---GTCCTGGCTGTGGAAAGATACCTAAAGGATCAACAGCTCCTAGGGATTTGGGGTTGCTCTGGAAAACTCATTTGCACCACTACTGTGCCTTGGAATACTAGTTGGAGT---------------------------AATAAATCTCTGGATGATATTTGGCAT---AACATGACCTGGATGCAGTGGGAAAAAGAGATTAAC------AATTACACAGACTTAATATACACCTTAATTGAAGAATCGCAAAACCAACAAGAAAAGAATGAACAAGAATTATTGGAATTAGATAAATGGGACAGTTTGTGGAATTGGTTTAACATAACAAACTGGCTGTGGTATATAAAAATATTCATAATGATAGTAGGAGGCTTGATAGGTTTAAGAATAGTTTTTATTGTACTTTCTGTAGTGAATAGAGTTAGGCAGGGATACTCACCATTATCATTTCAGACCCGC---CTCCCAGTCCCGAGGGAA------CCCGACAGGCCCGAAGGAACAGAAGAAGAAGGTGGAGAGAAAGACAGAGGCAGATCCGGACGTTTAGTGGATGGATTCTTAGCACTTATCTGGGACGATCTGCGGAGCCTGTTCCTCTTCAGCTACCACCGCTTGAGAGACTTACTCTTGATTGTAGCGAGGATTGTGGAACTTCTGGGACGCAGG---------------GGGTGGGGAGTTCTCAAATATTGGTGG---AATCTCCTGCAGTATTGG---------------------------------------------------AGTCAGGAACTAAAGAATAGTGCTGTTAGCTTGCTCAATGCCACAGCTATCGCAGTAGCTGAGGGGACAGATAGGGTTATAGAAATATTACAAAGA------------------ATTTATAGAGCTTTTCTCCACATACCTAGAAGAATAAGACAGGGCCTCGAAAGGGCTTTGTTATAA

2.BORI0637.UAB.EU576292 ATGAGAGCGAAGGAGATCAGGAAGAATTGTCAGCGCTTG---------TGGAGATGGGGC------------------------ACCATGCTCCTTGGGATGTTGATGATC------------TGTAGTGCTGCA---------GGCAATTTGTGGGTCACAGTCTATTATGGGGTACCTGTGTGGAAAGAAGCAACCACCACTTTATTTTGTGCATCAGATGCTAAAGCATATGATACAGAGGTACATAAT---GTTTGGGCCACACATGCCTGTGTACCCACAGACCCCAACCCACAAGAAGTAGTACTG---AAAAATGTGACAGAAAATTTTAACATGTGGAAAAATAACATGGTAGAACAGATGCATGAGGATATAATCAGTTTATGGGATCAAAGCCTAAAGCCATGTGTAAAACTAACTCCACTCTGTGTTACTTTAAATTGCACTGATTATTTGAATAATGCTACTAATACC------------------------------------------------------------------------------------------ACTAGTAGTAAGGAGGGAGAAATGAGAGGAGAAATAAAAAAATGCTCTTTCAATGTCACC---ACAAGAATAAGAGAT------AAGGTGCAGAAAGAATATGCACTTTTTTATAAACTTGATGTAGTACCAATAGGTAATGGT------------------------------AATACTAGC---------------------------TATAGGTTGATAAATTGTAATACCTCAGTCATTACACAGGCCTGTCCAAAGGTATCCTTTGAACCAATTCCCATACATTATTGTACCCCGGCTGGTTTTGCAATTCTAAAGTGT---AAAGATAAGAAGTTCAATGGAACAGGATCATGTAAAAATGTCAGCACAGTACAATGTACACATGGAATTAGACCAGTAGTGTCAACTCAACTGTTGTTAAATGGCAGTCTAGCAGAAGAA---GAGGTAGTAATTAGATCTGAAAATTTTACGAACAATGCTAAAACCATAATAGTACAGCTGAACGAAACTGTAGAAATTAATTGTACAAGACCCAACAACAATACAAGAAAAAGTATACATATAGGA------------CCAGGGAGA---ACATTTTATACAACAGGAGACATAATAGGAGATATAAGGCAGGCATATTGTATCCTT------AGTAGAGCAAAATGGGAAGACACTTTACAAAAGATAGTCACAAAATTAGGA---GAACAATATGGG---------AATAATAAAACA---ATAGTCTTTAATCAC---------TCCTCAGGAGGGGACCCAGAAATTGTAATGCACAGTTTTAATTGTGGAGGGGAATTTTTCTACTGTAATTCAACACAACTGTTTAATAGTACTTGGAGTTTT---------AATAGTACTTGG------------------------AATAAGAATTTTAATAGTACTTGG------------AATATTACTGAAGGGACA---AATAACACTGAA------ATCACACTCCCATGTAGAATAAAACAAATTATAAACATGTGGCAGGAAGTAGGAAAAGCAATGTATGCCCCTCCCATCAGAGGACAAATTAAATGTTCATCTAACATTACAGGGCTGCTATTAACAAGAGATGGTGGGGGTAATGGG---------AGCGGGACC---------------------------ACT---GAGGTCTTCAGACCTGGAGGGGGAGATATGAGGGACAATTGGAGA---AGTGAATTATACAAATATAAAGTAGTAAAAATTGAACCA---TTAGGAGTAGCACCT---ACCAAGGCAAAGAGAAGAGTGGTGCAAGGAGAA---AAAAGAGCAGTG---GGAGCAATAGGA---GCTATG---TTCCTT---GGG---------TTCTTGGGA---GCAGCAGGAAGCACTATGGGCGCAGCGTCAGTG---GCGCTGACGGTACAGGCCAGACAATTATTGTCTGGTATAGTGCAACAGCAGAACAATTTGCTGAGGGCTATTGAGGCGCAACAGCATCTGTTGCAACTCACAGTCTGGGGCATCAAGCAGCTCCAGGCAAGA---GTCCTGGCTGTGGAAAGATACCTAAAGGATCAACAGCTCCTAGGGATTTGGGGTTGCTCTGGAAAACTCATTTGCACCACTACTGTGCCTTGGAATACTAGTTGGAGT---------------------------AATAAATCTCTGGATGATATTTGGCAT---AACATGACCTGGATGCAGTGGGAAAAAGAGATTAAC------AATTACACAGACTTAATATACACCTTAATTGAAGAATCGCAAAACCAACAAGAAAAGAATGAACAAGAATTATTGGAATTAGATAAATGGGACAGTTTGTGGAATTGGTTTAACATAACAAACTGGCTGTGGTATATAAAAATATTCATAATGATAGTAGGAGGCTTGATAGGTTTAAGAATAGTTTTTATTGTACTTTCTGTAGTGAATAGAGTTAGGCAGGGATACTCACCATTATCATTTCAGACCCGC---CTCCCAGTCCCGAGGGAA------CCCGACAGGCCCGAAGGAACAGAAGAAGAAGGTGGAGAGAAAGACAGAGGCAGATCCGGACGTTTAGTGGATGGATTCTTAGCACTTATCTGGGACGATCTGCGGAGCCTGTTCCTCTTCAGCTACCACCGCTTGAGAGACTTACTCTTGATTGTAGCGAGGATTGTGGAACTTCTGGGACGCAGG---------------GGGTGGGGAGTTCTCAAATATTGGTGG---AATCTCCTGCAGTATTGG---------------------------------------------------AGTCAGGAACTAAAGAATAGTGCTGTTAGCTTGCTCAATGCCACAGCTATCGCAGTAGCTGAGGGGACAGATAGGGTTATAGAAATATTACAAAGA------------------ATTTATAGAGCTTTTCTCCACATACCTAGAAGAATAAGACAGGGCCTCGAAAGGGCTTTGTTATAA

2.BORI0637.UAB.EU576302 ATGAGAGCGAAGGAGATCAGGAAGAATTGTCAGCGCTTG---------TGGAGATGGGGC------------------------ACCATGCTCCTTGGGATGTTGATGATC------------TGTAGTGCTGCA---------GGCAATTTGTGGGTCACAGTCTATTATGGGGTACCTGTGTGGAAAGAAGCAACCACCACTTTATTTTGTGCATCAGATGCTAAAGCATATGATACAGAGGTACATAAT---GTTTGGGCCACACATGCCTGTGTACCCACAGACCCCAACCCACAAGAAGTAGTACTG---AAAAATGTGACAGAAAATTTTAACATGTGGAAAAATAACATGGTAGAACAGATGCATGAGGATATAATCAGTTTATGGGATCAAAGCCTAAAGCCATGTGTAAAACTAACTCCACTCTGTGTTACTTTAAATTGCACTGATTATTTGAATAATGCTACTAATACC------------------------------------------------------------------------------------------ACTAGTAGTAAGGAGGGAGAAATGAGAGGAGAAATAAAAAAATGCTCTTTCAATGTCACC---ACAAGAATAAGAGAT------AAGGTGCAGAAAGAATATGCACTTTTTTATAAACTTGATGTAGTACCAATAGGTAATGGT------------------------------AATACTAGC---------------------------TATAGGTTGATAAATTGTAATACCTCAGTCATTACACAGGCCTGTCCAAAGGTATCCTTTGAACCAATTCCCATACATTATTGTACCCCGGCTGGTTTTGCAATTCTAAAGTGT---AAAGATAAGAAGTTCAATGGAACAGGATCATGTAAAAATGTCAGCACAGTACAATGTACACATGGAATTAGACCAGTAGTGTCAACTCAACTGTTGTTAAATGGCAGTCTAGCAGAAGAA---GAGGTAGTAATTAGATCTGAAAATTTTACGAACAATGCTAAAACCATAATAGTACAGCTGAACGAAACTGTAGAAATTAATTGTACAAGACCCAACAACAATACAAGAAAAAGTATACATATAGGA------------CCAGGGAGA---ACATTTTATACAACAGGAGACATAATAGGAGATATAAGGCAGGCATATTGTATCCTT------AGTAGAGCAAAATGGGAAGACACTTTACAAAAGATAGTCACAAAATTAGGA---GAACAATATGGG---------AATAATAAAACA---ATAGTCTTTAATCAC---------TCCTCAGGAGGGGACCCAGAAATTGTAATGCACAGTTTTAATTGTGGAGGGGAATTTTTCTACTGTAATTCAACACAACTGTTTAATAGTACTTGGAGTTTT---------AATAGTACTTGG------------------------AATAAGAATTTTAATAGTACTTGG------------AATATTACTGAAGGGACA---AATAACACTGAA------ATCACACTCCCATGTAGAATAAAACAAATTATAAACATGTGGCAGGAAGTAGGAAAAGCAATGTATGCCCCTCCCATCAGAGGACAAATTAAATGTTCATCTAACATTACAGGGCTGCTATTAACAAGAGATGGTGGGGGTAATGGG---------AGCGGGACC---------------------------ACT---GAGGTCTTCAGACCTGGAGGGGGAGATATGAGGGACAATTGGAGA---AGTGAATTATACAAATATAAAGTAGTAAAAATTGAACCA---TTAGGAGTAGCACCT---ACCAAGGCAAAGAGAAGAGTGGTGCAAAGAGAA---AAAAGAGCAGTG---GGAGCAATAGGA---GCTATG---TTCCTT---GGG---------TTCTTGGGA---GCAGCAGGAAGCACTATGGGCGCAGCGTCAGTG---GCGCTGACGGTACAGGCCAGACAATTATTGTCTGGTATAGTGCAACAGCAGAACAATTTGCTGAGGGCTATTGAGGCGCAACAGCATCTGTTGCAACTCACAGTCTGGGGCATCAAGCAGCTCCAGGCAAGA---GTCCTAGCTGTGGAAAGATACCTAAAGGATCAACAGCTCCTAGGGATTTGGGGTTGCTCTGGAAAACTCATTTGCACCACTACTGTGCCTTGGAATACTAGTTGGAGT---------------------------AATAAATCTCTGGATGATATTTGGCAT---AACATGACCTGGATGCAGTGGGAAAAAGAGATTAAC------AATTACACAGACTTAATATACACCTTAATTGAAGAATCGCAAAACCAACAAGAAAAGAATGAACAAGAATTATTGGAATTAGATAAATGGGACAGTTTGTGGAATTGGTTTAACATAACAAACTGGCTGTGGTATATAAAAATATTCATAATGATAGTAGGAGGCTTGATAGGTTTAAGAATAGTTTTTATTGTACTTTCTGTAGTGAATAGAGTTAGGCAGGGATACTCACCATTATCATTTCAGACCCGC---CTCCCAGTCCCGAGGGAA------CCCGACAGGCCCGAAGGAACAGAAGAAGAAGGTGGAGAGAAAGACAGAGGCAGATCCGGACGTTTAGTGGATGGATTCTTAGCACTTATCTGGGACGATCTGCGGAGCCTGTTCCTCTTCAGCTACCACCGCTTGAGAGACTTACTCTTGATTGTAGCGAGGATTGTGGAACTTCTGGGACGCAGG---------------GGGTGGGGAGTTCTCAAATATTGGTGG---AATCTCCTGCAGTATTGG---------------------------------------------------AGTCAGGAACTAAAGAATAGTGCTGTTAGCTTGCTCAATGCCACAGCTATCGCAGTAGCTGAGGGGACAGATAGGGTTATAGAAATATTACAAAGA------------------ATTTATAGAGCTTTTCTCCACATACCTAGAAGAATAAGACAGGGCCTCGAAAGGGCTTTGTTATAA

2.BORI0637.UAB.EU576291 ATGAGAGCGAAGGAGATCAGGAAGAATTGTCAGCGCTTG---------TGGAGATGGGGC------------------------ACCATGCTCCTTGGGATGTTGATGATC------------TGTAGTGCTGCA---------GGCAATTTGTGGGTCACAGTCTATTATGGGGTACCTGTGTGGAAAGAAGCAACCACCACTTTATTTTGTGCATCAGATGCTAAAGCATATGATACAGAGGTACATAAT---GTTTGGGCCACACATGCCTGTGTACCCACAGACCCCAACCCACAAGAAGTAGTACTG---AAAAATGTGACAGAAAATTTTAACATGTGGAAAAATAACATGGTAGAACAGATGCATGAGGATATAATCAGTTTATGGGATCAAAGCCTAAAGCCATGTGTAAAACTAACTCCACTCTGTGTTACTTTAAATTGCACTGATTATTTGAATAATGCTACTAATACC------------------------------------------------------------------------------------------ACTAGTAGTAAGGAGGGAGAAATGAGAGGAGAAATAAAAAAATGCTCTTTCAATGTCACC---ACAAGAATAAGAGAT------AAGGTGCAGAAAGAATATGCACTTTTTTATAAACTTGATGTAGTACCAATAGGTAATGGT------------------------------AATACTAGC---------------------------TATAGGTTGATAAATTGTAATACCTCAGTCATTACACAGGCCTGTCCAAAGGTATCCTTTGAACCAATTCCCATACATTATTGTACCCCGGCTGGTTTTGCAATTCTAAAGTGT---AAAGATAAGAAGTTCAATGGAACAGGATCATGTAAAAATGTCAGCACAGTACAATGTACACATGGAATTAGACCAGTAGTGTCAACTCAACTGTTGTTAAATGGCAGTCTAGCAGAAGAA---GAGGTAGTAATTAGATCTGAAAATTTTACGAACAATGCTAAAACCATAATAGTACAGCTGAACGAAACTGTAGAAATTAATTGTACAAGACCCAACAACAATACAAGAAAAAGTATACATATAGGA------------CCAGGGAGA---ACATTTTATACAACAGGAGACATAATAGGAGATATAAGGCAGGCATATTGTATCCTT------AGTAGAGCAAAATGGGAAGACACTTTACAAAAGATAGTCACAAAATTAGGA---GAACAATATGGG---------AATAATAAAACA---ATAGTCTTTAATCAC---------TCCTCAGGAGGGGACCCAGAAATTGTAATGCACAGTTTTAATTGTGGAGGGGAATTTTTCTACTGTAATTCAACACAACTGTTTAATAGTACTTGGAGTTTT---------AATAGTACTTGG------------------------AATAAGAATTTTAATAGTACTTGG------------AATATTACTGAAGGGACA---AATAACACTGAA------ATCACACTCCCATGTAGAATAAAACAAATTATAAACATGTGGCAGGAAGTAGGAAAAGCAATGTATGCCCCTCCCATCAGAGGACAAATTAAATGTTCATCTAACATTACAGGGCTGCTATTAACAAGAGATGGTGGGGGTAATGGG---------AGCGGGACC---------------------------ACT---GAGGTCTTCAGACCTGGAGGGGGAGATATGAGGGACAATTGGAGA---AGTGAATTATACAAATATAAAGTAGTAAAAATTGAACCA---TTAGGAGTAGCACCT---ACCAAGGCAAAGAGAAGAGTGGTGCAAAGAGAA---AAAAGAGCAGTG---GGAGCAATAGGA---GCTGTG---TTCCTT---GGG---------TTCTTGGGA---GCAGCAGGAAGCACTATGGGCGCAGCGTCAGTG---GCGCTGACGGTACAGGCCAGACAATTATTGTCTGGTATAGTGCAACAGCAGAACAATTTGCTGAGGGCTATTGAGGCGCAACAGCATCTGTTGCAACTCACAGTCTGGGGCATCAAGCAGCTCCAGGCAAGA---GTCCTGGCTGTGGAAAGATACCTAAAGGATCAACAGCTCCTAGGGATTTGGGGTTGCTCTGGAAAACTCATTTGCACCACTACTGTGCCTTGGAATACTAGTTGGAGT---------------------------AATAAATCTCTGGATGATATTTGGCAT---AACATGACCTGGATGCAGTGGGAAAAAGAGATTAAC------AATTACACAGACTTAATATACACCTTAATTGAAGAATCGCAAAACCAACAAGAAAAGAATGAACAAGAATTATTGGAATTAGATAAATGGGACAGTTTGTGGAATTGGTTTAACATAACAAACTGGCTGTGGTATATAAAAATATTCATAATGATAGTAGGAGGCTTGATAGGTTTAAGAATAGTTTTTATTGTACTTTCTGTAGTGAATAGAGTTAGGCAGGGATACTCACCATTATCATTTCAGACCCGC---CTCCCAGTCCCGAGGGAA------CCCGACAGGCCCGAAGGAACAGAAGAAGAAGGTGGAGAGAAAGACAGAGGCAGATCCGGACGTTTAGTGGATGGATTCTTAGCACTTATCTGGGACGATCTGCGGAGCCTGTTCCTCTTCAGCTACCACCGCTTGAGAGACTTACTCTTGATTGTAGCGAGGATTGTGGAACTTCTGGGACGCAGG---------------GGGTGGGGAGTTCTCAAATATTGGTGG---AATCTCCTGCAGTATTGG---------------------------------------------------AGTCAGGAACTAAAGAATAGTGCTGTTAGCTTGCTCAATGCCACAGCTATCGCAGTAGCTGAGGGGACAGATAGGGTTATAGAAATATTACAAAGA------------------ATTTATAGAGCTTTTCTCCACATACCTAGAAGAATAAGACAGGGCCTCGAAAGGGCTTTGTTATAA

2.BORI0637.UAB.EU576289 ATGAGAGCGAAGGAGATCAGGAAGAATTGTCAGCGCTTG---------TGGAGATGGGGC------------------------ACCATGCTCCTTGGGATGTTGATGATC------------TGTAGTGCTGCA---------GGCAATTTGTGGGTCACAGTCTATTATGGGGTACCTGTGTGGAAAGAAGCAACCACCACTTTATTTTGTGCATCAGATGCTAAAGCATATGATACAGAGGTACATAAT---GTTTGGGCCACACATGCCTGTGTACCCACAGACCCCAACCCACAAGAAGTAGTACTG---AAAAATGTGACAGAAAATTTTAACATGTGGAAAAATAACATGGTAGAACAGATGCATGAGGATATAATCAGTTTATGGGATCAAAGCCTAAAGCCATGTGTAAAACTAACTCCACTCTGTGTTACTTTAAATTGCACTGATTATTTGAATAATGCTACTAATACC------------------------------------------------------------------------------------------ACTAGTAGTAAGGAGGGAGAAATGAGAGGAGAAATAAAAAAATGCTCTTTCAATGTCACC---ACAAGAATAAGAGAT------AAGGTGCAGAAAGAATATGCACTTTTTTATAAACTTGATGTAGTACCAATAGGTAATGGT------------------------------AATACTAGC---------------------------TATAGGTTGATAAATTGTAATACCTCAGTCATTACACAGGCCTGTCCAAAGGTATCCTTTGAACCAATTCCCATACATTATTGTACCCCGGCTGGTTTTGCAATTCTAAAGTGT---AAAGATAAGAAGTTCAATGGAACAGGATCATGTAAAAATGTCAGCACAGTACAATGTACACATGGAATTAGACCAGTAGTGTCAACTCAACTGTTGTTAAATGGCAGTCTAGCAGAAGAA---GAGGTAGTAATTAGATCTGAAAATTTTACGAACAATGCTAAAACCATAATAGTACAGCTGAACGAAACTGTAGAAATTAATTGTACAAGACCCAACAACAATACAAGAAAAAGTATACATATAGGA------------CCAGGGAGA---ACATTTTATACAACAGGAGACATAATAGGAGATATAAGGCAGGCATATTGTATCCTT------AGTAGAGCAAAATGGGAAGACACTTTACAAAAGATAGTCACAAAATTAGGA---GAACAATATGGG---------AATAATAAAACA---ATAGTCTTTAATCAC---------TCCTCAGGAGGGGACCCAGAAATTGTAATGCACAGTTTTAATTGTGGAGGGGAATTTTTCTACTGTAATTCAACACAACTGTTTAATAGTACTTGGAGTTTT---------AATAGTACTTGG------------------------AATAAGAATTTTAATAGTACTTGG------------AATATTACTGAAGGGACA---AATAACACTGAA------ATCACACTCCCATGTAGAATAAAACAAATTATAAACATGTGGCAGGAAGTAGGAAAAGCAATGTATGCCCCTCCCATCAGAGGACAAATTAAATGTTCATCTAACATTACAGGGCTGCTATTAACAAGAGATGGTGGGGGTAATGGG---------AGCGGGACC---------------------------ACT---GAGGTCTTCAGACCTGGAGGGGGAGATATGAGGGACAATTGGAGA---AGTGAATTATACAAATATAAAGTAGTAAAAATTGAACCA---TTAGGAGTAGCACCT---ACCAAGGCAAAGAGAAGAGTGGTGCAAAGAGAA---AAAAGAGCAGTG---GGAGCAATAGGA---GCTATG---TTCCTT---GGG---------TTCTTGGGA---GCAGCAGGAAGCACTATGGGCGCAGCGTCAGTG---GCGCTGACGGTACAGGCCAGACAATTATTGTCTGGTATAGTGCAACAGCAGAACAATTTGCTGAGGGCTATTGAGGCGCAACAGCATCTGTTGCAACTCACAGTCTGGGGCATCAAGCAGCTCCAGGCAAGA---GTCCTGGCTGTGGAAAGATACCTAAAGGATCAACAGCTCCTAGGGATTTGGGGTTGCTCTGGAAAACTCATTTGCACCACTACTGTGCCTTGGAATACTAGTTGGAGT---------------------------AATAAATCTCTGGATGATATTTGGCAT---AACATGACCTGGATGCAGTGGGAAAAAGAGATTAAC------AATTACACAGACTTAATATACACCTTAATTGAAGAATCGCAAAACCAACAAGAAAAGAATGAACAAGAATTATTGGAATCAGATAAATGGGACAGTTTGTGGAATTGGTTTAACATAACAAACTGGCTGTGGTATATAAAAATATTCATAATGATAGTAGGAGGCTTGATAGGTTTAAGAATAGTTTTTATTGTACTTTCTGTAGTGAATAGAGTTAGGCAGGGATACTCACCATTATCATTTCAGACCCGC---CTCCCAGTCCCGAGGGAA------CCCGACAGGCCCGAAGGAACAGAAGAAGAAGGTGGAGAGAAAGACAGAGGCAGATCCGGACGTTTAGTGGATGGATTCTTAGCACTTATCTGGGACGATCTGCGGAGCCTGTTCCTCTTCAGCTACCACCGCTTGAGAGACTTACTCTTGATTGTAGCGAGGATTGTGGAACTTCTGGGACGCAGG---------------GGGTGGGGAGTTCTCAAATATTGGTGG---AATCTCCTGCAGTATTGG---------------------------------------------------AGTCAGGAACTAAAGAATAGTGCTGTTAGCTTGCTCAATGCCACAGCTATCGCAGTAGCTGAAGGGACAGATAGGGTTATAGAAATATTACAAAGA------------------ATTTATAGAGCTTTTCTCCACATACCTAGAAGAATAAGACAGGGCCTCGAAAGGGCTTTGTTATAA

2.BORI0637.UAB.EU576277 ATGAGAGCGAAGGAGATCAGGAAGAATTGTCAGCGCTTG---------TGGAGATGGGGC------------------------ACCATGCTCCTTGGGATGTTGATGATC------------TGTAGTGCTGCA---------GGCAATTTGTGGGTCACAGTCTATTATGGGGTACCTGTGTGGAAAGAAGCAACCACCACTTTATTTTGTGCATCAGATGCTAAAGCATATGATACAGAGGTACATAAT---GTTTGGGCCACACATGCCTGTGTACCCACAGACCCCAACCCACAAGAAGTAGTACTG---AAAAATGTGACAGAAAATTTTAACATGTGGAAAAATAACATGGTAGAACAGATGCATGAGGATATAATCAGTTTATGGGATCAAAGCCTAAAGCCATGTGTAAAACTAACTCCACTCTGTGTTACTTTAAATTGCACTGATTATTTGAATAATGCTACTAATACC------------------------------------------------------------------------------------------ACTAGTAGTAAGGAGGGAGAAATGAGAGGAGAAATAAAAAAATGCTCTTTCAATGTCACC---ACAAGAATAAGAGAT------AAGGTGCAGAAAGAATATGCACTTTTTTATAAACTTGATGTAGTACCAATAGGTAATGGT------------------------------AATACTAGC---------------------------TATAGGTTGATAAATTGTAATACCTCAGTCATTACACAGGCCTGTCCAAAGGTATCCTTTGAGCCAATTCCCATACATTATTGTACCCCGGCTGGTTTTGCAATGCTAAAGTGT---AAAGATAAGAAGTTCAATGGAACAGGATCATGTGAAAATGTCAGCACAGTACAATGTACACATGGAATTAGACCAGTAGTGTCAACTCAACTGTTGTTAAATGGCAGTCTAGCAGAAGAA---GAGGTAGTAATTAGATCTGAAAATTTTACGAACAATGCTAAAACCATAATAGTACAGCTGAATGAAACTGTAGAAATTAATTGTACAAGACCCAACAACAATACAAGAAAAAGTATACATATAGGA------------CCAGGGAGA---ACATTTTATACAACAGGAGACATAATAGGAGATATAAGGCAAGCATATTGTATCCTT------AGTAGAGCAAAATGGGAAGACACTTTAAAAAAGATAGTCACAAAATTAGGA---GAACAATATGGG---------AATAATAAAACA---ATAGTCTTTAATCAC---------TCCTCAGGAGGGGACCCAGAAATTGTAATGCACAGTTTTAATTGTGGAGGGGAATTTTTCTACTGTAATTCAACACAACTGTTTAATAGTACTTGGAGTTTT---------AATAGTACTTGG------------------------AATAAGAATTTTAATAGTACTTGG------------AATATTACTGAAGGGACA---AATAACACTGAA------ATCACACTCCCATGTAGAATAAAACAAATTATAAACATGTGGCAGGAAGTGGGAAAAGCAATGTATGCCCCTCCCATCAGAGGACAAATTAAATGTTCATCTAACATTACAGGGCTGCTATTAACAAGAGATGGTGGGGGTAATGGG---------AGCGGGACC---------------------------ACT---GAGGTCTTCAGACCTGGAGGGGGAGATATGAGGGACAATTGGAGA---AGTGAATTATACAAATATAAAGTAGTAAAAATTGAACCA---TTAGGAGTAGCACCC---ACCAAGGCAAAGAGAAGAGTGGTGCAAGGAGAA---AAAAGAGCAGTG---GGAGCAATAGGA---GCTATG---TTCCTT---GGG---------TTCTTGGGA---GCAGCAGGAAGCACTATGGGCGCAGCGTCAATG---GCGCTGACGGTACAGGCCAGACAATTATTGTCTGGTATAGTGCAACAGCAGAACAATTTGCTAAGGGCTATTGAGGCGCAACAGCATCTGTTGCAACTCACAGTCTGGGGCATCAAGCAGCTCCAGGCAAGA---GTCCTGGCTGTGGAAAGATACCTAAAGGATCAACAGCTCCTAGGGATTTGGGGTTGCTCTGGAAAACTCATTTGCACCACTACTGTGCCTTGGAATACTAGTTGGAGT---------------------------AATAAATCTCTGGATGATATTTGGCAT---AACATGACCTGGATGCAGTGGGAAAAAGAGATTAAC------AATTACACAGATTTAATATACACCTTAATTGAAGAATCGCAAAACCAACAAGAAAAGAATGAACAAGAATTATTGGAATTAGATAAATGGGACAGTTTGTGGAATTGGTTTAACATAACAAACTGGCTGTGGTATATAAAAATATTCATAATGATAGTAGGAGGCTTGATAGGTTTAAGAATAGTTTTTATTGTACTTTCTGTAGTGAATAGAGTTAGGCAGGGATACTCACCATTATCATTTCAGACCCGC---CTCCCAGTCCCGAGGGAA------CCCGACAGGCCCGAAGGAACAGAAAAAGAAGGTGGAGAGAAAGACAGAGGCAGATCCGGACGATTAGTGGATGGATTCTTAGCACTTATCTGGGACGATCTGCGGAGCCTGTGCCTCTTCAGCTACCACCGCTTGAGAGACTTACTCTTGATTGTAGCGAGGATTGTGGAACTTCTGGGACGCAGG---------------GGGTGGGGAGCTCTCAAATATTGGTGG---AATCTCCTGCAGTATTGG---------------------------------------------------AGTCAGGAACTAAAGAATAGTGCTGTTAGCTTGCTCAATGCCACAGCTATCGCAGTAGCTGAGGGGACAGATAGGGTTATAGAAATATTACAAAGA------------------ATTTATAGAGCTTTTCTCCACATACCTAGAAGAATAAGACAGGGCCTCGAAAGGGCTTTGTTATAA

2.BORI0637.UAB.EU576294 ATGAGAGCGAAGGAGATCAGGAAGAATTGTCAGCGCTTG---------TGGAGATGGGGC------------------------ACCATGCTCCTTGGGATGTTGATGATC------------TGTAGTGCTGCA---------GGCAATTTGTGGGTCACAGTCTATTATGGGGTACCTGTGTGGAAAGAAGCAACCACCACTTTATTTTGTGCATCAGATGCTAAAGCATATGATACAGAGGTACATAAT---GTTTGGGCCACACATGCCTGTGTACCCACAGACCCCAACCCACAAGAAGTAGTACTG---AAAAATGTGACAGAAAATTTTAACATGTGGAAAAATAACATGGTAGAACAGATGCATGAGGATATAATCAGTTTATGGGACCAAAGCCTAAAGCCATGTGTAAAACTAACTCCACTCTGTGTTACTTTAAATTGCACTGATTATTTGAATAATGCTACTAATACC------------------------------------------------------------------------------------------ACTAGTAGTAAGGAGGGAGAAATGAGAGGAGAAATAAAAAAATGCTCTTTCAATGTCACC---ACAAGAATAAGAGAT------AAGGTGCAGAAAGAATATGCACTTTTTTATAAACTTGATGTAGTACCAATAGGTAATGGT------------------------------AATACTAGC---------------------------TATAGGTTGATAAATTGTAATACCTCAGTCATTACACAGGCCTGTCCAAAGGTATCCTTTGAACCAATTTCCATACATTATTGTACCCCGGCTGGTTTTGCAATTCTAAAGTGT---AAAGATAAGAAGTTCAATGGAACAGGATCATGTAAAAATGTCAGCACAGTACAATGTACACATGGAATTAGACCAGTAGTGTCAACTCAACTGTTGTTAAATGGCAGTCTAGCAGAAGAA---GAGGTAGTAATTAGATCTGAAAATTTTACGAACAATGCTAAAACCATAATAGTACAGCTGAATGAGACTGTAGAAATTAATTGTACAAGACCCAACAACAATACAAGAAAAGGTATACATATAGGA------------CCAGGAAGA---ACATTTTATACAACAGGAGACATAATAGGAGATATAAGGCAAGCATATTGTATCCTT------AGTAGAGCAAAATGGGAAGACACTTTAAAAAAGATAGTCACAAAATTAGGA---GAACAATATGGG---------AATAATAAAACA---ATAGTCTTTAATCAC---------TCCTCAGGAGGGGACCCAGAAATTGTAATGCACAGTTTTAATTGTGGAGGGGAATTTTTCTACTGTAATTCAACACAACTGTTTAATAGTACTTGGAGTTTT---------AATAGTACTTGG------------------------AATAAAAATTCTAATAGTACTTGG------------AATAATACTGAAGGGACA---AATGACACA---------ATCACACTCCCATGCAGAATAAAACAAATTATAAACATGTGGCAGGAAGTAGGAAAAGCAATGTATGCCCCTCCCATCAGAGGACAAATTAGATGTTCATCTAATATTACAGGGCTGCTATTAACAAGAGATGGTGGGAATAATGGG---------AGCGGGACC---------------------------ACT---GAGATCTTCAGACCTGGAGGGGGAGATATGAGGGACAATTGGAGA---AGTGAACTATACAAATATAAAGTAGTAAAAATTGAACCA---TTAGGAGTAGCACCC---ACCAAGGCAAAGAGAAGAGTGGTGCAAAGAGAA---AAAAGAGCGGTG---GGAGCAATAGGA---GCTATG---TTCCTT---GGG---------TTCTTGGGA---GCAGCAGGAAGCACTATGGGCGCAGCGTCAATG---GCGCTGACGGTACAGGCCAGACAATTATTGTCTGGTATAGTGCAACAGCAGAACAATTTGCTGAGGGCTATTGAGGCGCAACAGCATCTGTTGCAACTCACAGTCTGGGGCATCAAGCAGCTCCAGGCAAGA---GTCCTGGCTGTGGAAAGATACCTAAAGGATCAACAGCTCCTAGGGATTTGGGGTTGCTCTGGAAAACTCATTTGCACCACTACTGTGCCTTGGAATACTAGTTGGAGT---------------------------AATAAATCTCTGGATGATATTTGGCAT---AACATGACCTGGATGCAGTGGGAAAAAGAGATTAAC------AATTACACAGATTTAATATACACCTTAATTGAAGAATCGCAAAACCAACAAGAAAAGAATGAACAAGAATTATTGGAATTAGATAAATGGGACAATTTGTGGAATTGGTTTAACATAACAAACTGGCTGTGGTATATAAAAATATTCATAATGATAGTAGGAGGCTTGATAGGTTTAAGAATAGTTTTTATTGTACTTTCTGTAGTGAATAGAGTTAGGCAGGGATACTCACCATTATCATTTCAGACCCGC---CTCCCAGTCCCGAGGGAA------CCCGACAGGCCCGAAGGAACAGAAGAAGAAGGTGGAGAGAAAGACAGAGGCAGATCCGGACGATTAGTGGATGGATTCTTAGCACTTATCTGGGACGATCTGCGGAGCCTGTGCCTCTTCAGCTACCACCGCTTGAGAGACTTACTCTTGATTGTAGCGAGGATTGTGGAACTTCTGGGACGCAGG---------------GGGTGGGGAGCTCTCAAATATTGGTGG---AATCTCCTGCAGTATTGG---------------------------------------------------AGTCAGGAACTAAAGAATAGTGCTGTTAGCTTGCTCAATGCCACAGCTATTGCAGTAGCTGAGGGGACAGATAGGATTATAGAAGTATTACAAAGA------------------CTTTATAGAGCTTTTCTTCACATACCTAGAAGAATAAGACAGGGCTTCGAAAGGGCTTTGTTATAA

2.BORI0637.UAB.EU576286 ATGAGAGCGAAGGAGATCAGGAAGAATTGTCAGCGCTTG---------TGGAGATGGGGC------------------------ACCATGCTCCTTGGGATGTTGATGATC------------TGTAGTGCTGCA---------GGCAATTTGTGGGTCACAGTCTATTATGGGGTACCTGTGTGGAAAGAAGCAACCACCACTTTATTTTGTGCATCAGATGCTAAAGCATATGATACAGAGGTACATAAT---GTTTGGGCCACACATGCCTGTGTACCCACAGACCCCAACCCACAAGAAGTAGTACTG---AAAAATGTGACAGAAAATTTTAACATGTGGAAAAATAACATGGTAGAACAGATGCATGAGGATATAATCAGTTTATGGGATCAAAGCCTAAAGCCATGTGTAAAACTAACTCCACTCTGTGTTACTTTAAATTGCACTGATTATTTGAATAATGCTACTAATACC------------------------------------------------------------------------------------------ACTAGTAGTAAGGAGGGAGAAATGAGAGGAGAAATAAAAAAATGCTCTTTCAATGTCACC---ATAAGAATAAGAGAT------AAGGTGCAGAAAGAATATGCACTTTTTTATAAACTTGATGTAGTACCAATAGGTAATGGT------------------------------AATACTAGC---------------------------TATAGGTTGATAAATTGTAATACCTCAGTCATTACACAGGCCTGTCCAAAGGTATCCTTTGAACCAATTCCCATACATTATTGTACCCCGGCTGGTTTTGCAATTCTAAAGTGT---AAAGATAAGAAGTTCAATGGAACAGGATCATGTAAAAATGTCAGCACAGTACAATGTACACATGGAATTAGACCAGTAGTGTCAACTCAACTGTTGTTAAATGGCAGTCTAGCAGAAGAA---GAGGTAGTAATTAGATCTGAAAATTTTACGAACAATGCTAAAACCATAATAGTACAGCTGAACGAAACTGTAGAAATTAATTGTATAAGACCCAACAACAATACAAGAAAAGGTATACATATAGGA------------CCAGGGAGA---ACATTTTATACAACAGGAGAAATAATAGGGGATATAAGGCAAGCACATTGTAACCTT------AATAGAACAAAATGGGAAGACACTTTAAAAAAGATAGTCACAAAATTAGGA---GAACAATATGGG---------AATAATAAAACA---ATAGTCTTTAATCAC---------TCCTCAGGAGGGGACCCAGAAATTGTAATGCACAGTTTTAATTGTGGAGGGGAATTTTTCTACTGTAATTCAACACAACTGTTTAATAGTACTTGGAGTTTT---------AATAGTACTTGG------------------------AATAAGAATTTTAATAGTACTTGG------------AATATTACTGAAGGGACA---AATAACACTGAA------ATCACACTCCCATGTAGAATAAAACAAATTATAAACATGTGGCAGGAAGTAGGAAAAGCAATGTATGCCCCTCCCATCAGAGGACAAATTAAATGTTCATCTAACATTACAGGGCTGCTATTAACAAGAGATGGTGGGGGTAATGGG---------AGCGGGACC---------------------------ACT---GAGGTCTTCAGACCTGGAGGGGGAGATATGAGGGACAATTGGAGA---AGTGAACTATACAAATATAAAGTAGTAAAAATTGAACCA---TTAGGAGTAGCACCT---ACCAAGGCAAAGAGAAGAGTGGTGCAAAGAGAA---AAAAGAGCAGTG---GGAGCGATAGGA---GCTATG---TTCCTT---GGG---------TTCTTGGGA---GCAGCAGGAAGCACTATGGGCGCAGCGTCAATG---GCGCTGACGGTACAGGCCAGACAATTATTGTCTGGTATAGTGCAACAGCAGAACAATTTGCTGAGGGCTATTGAGGCGCAACAGCATCTGTTGCAACTCACAGTCTGGGGCATCAAGCAGCTCCAGGCAAGA---GTCCTGGCTGTGGAAAGATACCTAAAGGATCAACAGCTCCTAGGGATTTGGGGTTGCTCTGGAAAACTCATTTGCACCACTACTGTGCCTTGGAATACTAGTTGGAGT---------------------------AATAAATCTCTGGATGATATTTGGCAT---AACATGACCTGGATGCAGTGGGAAAAAGAGATTAAC------AATTACACAGATTTAATATACACCTTAATTGAAGAATCGCAAAACCAACAAGAAAAGAATGAACAAGAATTATTGGAATTAGATAAATGGGACAGTTTGTGGAATTGGTTTAACATAACAAACTGGCTGTGGTATATAAAAATATTCATAATGATAGTAGGAGGCTTGATAGGTTTAAGAATAGTTTTTATTGTCCTTTCTGTAGTGAATAGAGTTAGGCAGGGATACTCACCATTATCATTTCAGACCCGC---CTCCCAGTCCCGAGGGAA------CCCGACAGGCCCGAAGGAACAGAAGAAGAAGGTGGAGAGAAAGACAGAGGCAGATCCGGACGATTAGTGGATGGATTCTTAGCACTTATCTGGGACGATCTGCGGAGCCTGTGCCTCTTCAGCTACCACCGCTTGAGAGACTTACTCTTGATTGTAGCGAGGATTGTGGAACTTCTGGGACGCAGG---------------GGGTGGGGAGCTCTCAAATATTGGTGG---AATCTCCTGCAGTATTGG---------------------------------------------------AGTCAGGAACTAAAGAATAGTGCTGTTAGCTTGCTCAATGCCACAGCTATTGCAGTAGCTGAGGGGACAGATAGGATTATAGAAGTATTACAAAGA------------------CTTTATAGAGCTTTTCTTCACATACCTAGAAGAATAAGACAGGGCTTCGAAAGGGCTTTGTTATAA

2.BORI0637.UAB.EU576300 ATGAGAGCGAAGGAGATCAGGAAGAATTGTCAGCGCTTG---------TGGAGATGGGGC------------------------ACCATGCTCCTTGGGATGTTGATGATC------------TGTAGTGCTGCA---------GGCAATTTGTGGGTCACAGTCTATTATGGGGTACCTGTGTGGAAAGAAGCAACCACCACTTTATTTTGTGCATCAGATGCTAAAGCATATGATACAGAGGTACATAAT---GTTTGGGCCACACATGCCTGTGTACCCACAGACCCCAACCCACAAGAAGTAGTACTG---AAAAATGTGACAGAAAATTTTAACATGTGGAAAAATAACATGGTAGAACAGATGCATGAGGATATAATCAGTTTATGGGATCAAAGCCTAAAGCCATGTGTAAAACTAACTCCACTCTGTGTTACTTTAAATTGCACTGATTATTTGAATAATGCTACTAATACC------------------------------------------------------------------------------------------ACTAGTAGTAAAGAGGGAGAAATGAGAGGAGAAATAAAAAAATGCTCTTTCAATGTCACC---ACAAGAATAAGAGAT------AAGGTGCAGAAAGAATATGCACTTTTTTATAAACTTAATGTAGTACCAATAGGTAATGGT------------------------------AATACTAGC---------------------------TATAGGTTGATAAATTGTAATACCTCAGTCATTACACAGGCCTGTCCAAAGGTATCCTTTGAACCAATTCCCATACATTATTGTACCCCGGCTGGTTTTGCAATTCTAAAGTGT---AAAGATAAGAAGTTCAATGGAACAGGATCATGTAAAAATGTCAGCACAGTACAATGTACACATGGAATTAGGCCAGTAGTGTCAACTCAACTGTTGTTAAATGGCAGTCTAGCAGAAGAA---GAGGTAGTAATTAGATCTGAAAATTTTACGAACAATGCTAAAACCATAATAGTACAGCTGAACGAAACTGTAGAAATTACTTGTATAAGACCCAACAACAATACAAGAAAAGGTATACATATAGGA------------CCAGGGAGA---ACATTTTATACAACAGGAGAAATAATAGGGGATATAAGGCAAGCACATTGTAACCTT------AATAGAACAAAATGGGAAGACACTTTAAAAAGGATAGTCACAAAATTAGGA---GAACAATATGGG---------AATAATAAAACA---ATAGTCTTTAATCAC---------TCCTCAGGAGGGGACCCAGAAATTGTAATGCACAGTTTTAATTGTGGAGGGGAATTTTTCTACTGTAATTCAACACAACTGTTTAATAGTACTTGGAGTTTT---------AATAGTACTTGG------------------------AATAAGAATTTTAATAGTACTTGG------------AATATTACTGAAGGGACA---AATAACACTGAA------ATCACACTCCCATGTAGAATAAAACAAATTATAAACATGTGGCAGGAAGTAGAAAAAGCAATGTATGCCCCTCCCATCAGAGGACAAATTAAATGTTCATCTAACATTACAGGGCTGCTATTAACAAGAGATGGTGGGGGTAATGGG---------AGCGGGACC---------------------------ACT---GAGGTCTTCAGACCTGGAGGGGGAGATATGAGGGACAATTGGAGA---AGTGAACTATACAAATATAAAGTAGTAAAAATTGAACCA---TTAGGAGTAGCACCT---ACCAAGGCAAAGAGAAGAGTGGTGCAAAGAGAA---AAAAGAGCAGTG---GGAGCGATAGGA---GCTATG---TTCCTT---GGG---------TTCTTGGGA---GCAGCAGGAAGCACTATGGGCGCAGCGTCAGTG---GCGCTGACGGTACAGGCCAGACAATTATTGTCTGGTATAGTGCAACAGCAGAACAATTTGCTAAGGGCTATTGAGGCGCAACAGCATCTGTTGCAACTCACAGTCTGGGGCATCAAGCAGCTCCAGGCAAGA---GTCCTGGCTGTAGAAAGATACCTAAAGGATCAACAGCTCCTAGGGATTTGGGGTTGCTCTGGAAAACTCATTTGCACCACTACTGTGCCTTGGAATACTAGTTGGAGT---------------------------AATAAATCTCTGGATGATATTTGGCAT---AACATGACCTGGATGCAGTGGGAAAAAGAGATTAAC------AATTACACAGATTTAATATACACCTTAATTGAAGAATCGCAAAACCAACAAGAAAAGAATGAACAAGAATTATTGGAATTAGATAAATGGGACAGTTTGTGGAATTGGTTTAACATAACAAACTGGCTGTGGTATATAAAAATATTCATAATGATAGTAGGAGGCTTGATAGGTTTAAGAATAGTTTTTATTGTACTTTCTGTAGTGAATAGAGTTAGGCAGGGATACTCACCATTATCATTTCAGACCCGC---CTCCCAGTCCCGAGGGAA------CCCGACAGGCCCGAAGGAACAGAAGAAGAAGGTGGAGAGAAAGACAGAGGCAGATCCGGACGATTAGTGGATGGATTCTTAGCACTTATCTGGGACGATCTGCGGAGCCTGTGCCTCTTCAGCTACCACCGCTTGAGAGACTTACTCTTGATTGTAGCGAGGATTGTGGAACTTCTGGGACGCAGG---------------GGGTGGGGAGTTCTCAAATATTGGTGG---AATCTCCTGCAGTATTGG---------------------------------------------------AGCCAGGAACTAAAGAATAGTGCTGTTAGCTTGCTCAATGCCACAGCTATCGCAGTAGCTGAGGGGACAGATAGGGTTATAGAAATATTACAAAGA------------------CTTTATAGAGCTTTTCTTCACATACCTAGAAGAATAAGACAGGGCTTCGAAAGGGCTTTGTTATAA

2.BORI0637.UAB.EU576282 ATGAGAGCGAAGGAGATCAGGAAGAATTGTCAGCGCTTG---------TGGAGATGGGGC------------------------ACCACGCTCCTTGGGATGTTGATGATC------------TGTAGTGCTGCA---------GGCAATTTGTGGGTCACAGTCTATTATGGGGTACCTGTGTGGAAAGAAGCAACCACCACTCTATTTTGTGCATCAGATGCTAAAGCATATGATACAGAGGTACATAAT---GTTTGGGCCACACATGCCTGTGTACCCACAGACCCCAACCCACAAGAAGTAGTACTG---AAAAATGTGACAGAAAATTTTAACATGTGGAAAAATAACATGGTAGAACAGATGCATGAGGATATAATCAGTTTATGGGATCAAAGCCTAAAGCCATGTGTAAAACTAACTCCACTCTGTGTTACTTTAAATTGCACTGAT---TTGAAGAATGCTACTAATACC------------------------------------------------------------------------------------------ACTAGTATTAAGGAGAGAGAAATGAGAGGAGAAATAAAAAACTGCTCTTTCAATGTCACC---ACAAGCATAAGAGAT------AAGGTGCAGAAAGAATATGCACTTTTTTATAAACTTGATGTAGTACCAATAGATAATGATAATGAT------------------------AATACTAGC---------------------------TATAGGTTGATAAATTGTAATACCTCAGTCATTACACAGGCCTGTCCAAAGGTATCCTTTGAGCCAATTCCCATACATTATTGTACCCCGGCTGGTTTTGCAATTCTAAAGTGT---AAAGATAAGAAGTTCAATGGAACAGGACTATGTGAAAATGTCAGCACAGTACAATGTACACATGGAATTAGACCAGTAGTGTCAACTCAACTGTTGTTAAATGGCAGTCTAGCAGAAGAA---GAGGTAGTAATTAGATCTGAAAATTTTACGAACAATGCTAAAACCATAATAGTACAGCTGAATGAAACTGTAGAAATTAATTGTATAAGACCCAACAACAATACAAGAAAAGGTATACATATAGGA------------CCAGGGAGA---ACATTTTATACAACAGGAGACATAATAGGAGATATAAGGCAAGCATATTGTAACCTT------AGTAGAACAAAATGGGAAGACACTTTAAAAAAGATAGTCACAAAATTAGGA---GAACAATATGGG---------AATAATAAAACA---ATAGTCTTTAATCAC---------TCCTCAGGAGGGGACCCAGAAATTGTAATGCACAGTTTTAATTGTGGAGGGGAATTTTTCTACTGTAATTCAACACAACTGTTTAATAGTACTTGGAATTTT---------AATGGTACTTGG------------------------AATAAGAATTTTAATAATACTTGG------------AATAATACTGAAGGGACA---AATGACACA---------ATCACACTCCCATGCAGAATAAAACAAATTATAAACATGTGGCAGGAAGTAGGAAAAGCAATGTATGCCCCGCCCATCAGAGGACAAATTAGATGTTCATCTAATATTACAGGGCTGCTATTAACAAGAGATGGTGGGGGTAATGGG---------AGCAGGACC---------------------------ACT---GAGATCTTCAGACCTGGAGGGGGAGATATGAGGGACAATTGGAGA---AGTGAACTATACAAATATAAAGTAGTAAAAATTGAACCA---TTAGGAGTAGCACCT---ACCAAGGCAAAGAGAAGAGTGGTGCAAAGAGAA---AAAAGAGCGGTG---GGAGCGATAGGA---GCTATG---TTCCTT---GGG---------TTCTTGGGA---GCAGCAGGAAGCACTATGGGCGCAGCGTCAATG---GCGCTGACGGTACAGGCCAGGCAATTATTGTCTGGTATAGTGCAACAGCAGAACAATTTGCTGAGGGCTATTGAGGCGCAACAGCATCTGTTGCAACTCACAGTCTGGGGCATCAAGCAGCTCCAGGCAAGA---GTCCTGGCTGTGGAAAGATACCTAAGGGATCAACAGCTCCTAGGGATTTGGGGTTGCTCTGGAAAACTCATTTGCACCACTACTGTGCCTTGGAATGCTAGTTGGAGT---------------------------AATAGATCTCTGGATGATATTTGGCAT---AACATGACCTGGATGCAGTGGGAAAGAGAGATTGAC------AATTACACAAGCTTAATATACACCTTAATTGAAGAATCGCAAAACCAACAAGAAAAGAATGAACAAGAATTGTTGGAATTGGATAAATGGGAAAATTTGTGGAGTTGGTTTACCATAACAAATTGGCTGTGGTATATAAAAATATTCATAATGATAGTAGGAGGCTTGGTAGGTTTAAGAATAGTTTTTATTGTACTTTCTGTAGTGAATAGAGTTAGGCAGGGATACTCACCATTATCATTCCAGATCCGC---CTCCCAGTCCCGAGGGAA------CCCGACAGGCCCGAAGGAACAGAAGAAGAAGGTGGAGAGAAAGACAGAGGCAGATCCGGACGTTTAGTGGATGGATTCTTAGCACTTATCTGGGACGATCTGCGGAGCCTGTTCCTCTTCAGCTACCACCGCTTGAGAGACTTACTCTTGATTGTAGCGAGGATTGTGGAACTTCTGGGACGCAGG---------------GGGTGGGGAGTTCTCAAATATTGGTGG---AATCTCCTGCAGTATTGG---------------------------------------------------AGTCAGGAACTAAAGAATAGTGCTGTTAGCTTGCTCAATGCCACAGCTATCGCAGTAGCTGAGGGGACAGATAGGGTTATAGAAGTATTACAAAGA------------------ATTTATAGAGCTTTTCTCCACATACCTAGAAGAATAAGACAGGGCCTCGAAAGGGCTTTGTTATAA

2.BORI0637.UAB.EU576283 ATGAGAGCGAAGGAGATCAGGAAGAATTGTCAGCGCTTG---------TGGAGATGGGGC------------------------ACCATGCTCCTTGGGATGTTGATGATC------------TGTAGTGCTGCA---------GGCAATTTGTGGGTCACAGTCTATTATGGGGTACCTGTGTGGAAAGAAGCAACCACCACTCTATTTTGTGCATCAGATGCTAAAGCATATGATACAGAGGTACATAAT---GTTTGGGCCACACATGCCTGTGTACCCACAGACCCCAACCCACAAGAAGTAGTACTG---AAAAATGTGACAGAAAATTTTAACATGTGGAAAAATAACATGGTAGAACAGATGCATGAGGATATAATCAGTTTATGGGATCAAAGCTTAAAGCCATGTGTAAAGCTAACCCCACTCTGTGTTACTTTAAATTGCACTGATCGGGTGGGGAATGCTACTCATACCAATAGT------------------------------------------------------------------------------------------AGTATGGAAGGAGAA------------ATAAAAAACTGCTCTTTCAATGTCACC---ACAAGAATAAGAGAT------AAGGTGCAGAAAGAATATGCACTTTTTTATAAACTTGATGTAGTACCAATAGATAAGAAT------------------------------AATACTAGC---------------------------TATAGGTTGATAAATTGTAATACCTCAGTCATTACACAAGCCTGTCCAAAGGTATCCTTTGAGCCAATTCCCATACATTATTGTACCCCGGCTGGTTTTGCAATTCTAAAATGT---AAAGATAAGAAGTTCAATGGAACAGGATCATGTGAAAATGTCAGCACGGTACATTGTACACATGGAATTAGACCAGTAGTGTCAACTCAACTGCTGTTAAATGGCAGTCTAGCAGAAGAA---GAGGTAGTAATTAGATCTGAAAATTTTACGAACAATGCTAAAACCATAATAGTACAGTTGAATGAGACTGTAGAAATTAATTGTACAAGACCCAACAACAATACAAGAAAAGGTATACATATAGGA------------CCAGGAAAA---ACATTTTATACAACAGGAGGAATAATAGGAGATATAAGGCAAGCATATTGTAACCTT------AGTAGAGCAAAATGGGAGGACACTTTAAAAAAGATAGTCACAAAATTAGGA---GAACAATATGGG---------AATAATAAAACA---ATAGTCTTTAATCAC---------TCCTCAGGAGGGGACCCAGAAATTGTAATGCACAGTTTTAATTGTGGAGGAGAATTTTTCTACTGTAATTCAACACAACTGTTTAATAGTACTTGGAAGTTT------AATAATAGTACTTGG------------------------------AATTTTAATAGTACTTGG------------AATAATACTGAAGGGACA---AATAACACTGAA------ATCACACTCCCATGCAGAATAAAGCAAATTATAAACATGTGGCAGGAAGTAGGAAAAGCAATGTATGCCCCTCCCATCAGCGGACAAATTAGATGTTCATCTAATATTACAGGGCTGCTATTAACAAGAGATGGTGGGGGTAATGGG---------AGTGGGACC---------------------------ACT---GAGATCTTCAGACCTGGAGGGGGAGATATGAGGGACAATTGGAGA---AGTGAACTATACAAATATAAAGTAGTAAAAATTGAACCA---TTAGGAGTAGCACCT---ACCAAGGCAAAGAGAAGAGTGGTGCAAAGAGAA---AAAAGAGCGGTG---GGAGCGCTAGGA---GCTATG---TTCCTT---GGG---------TTCTTGGGA---GCAGCAGGAAGCACTATGGGCGCAGCGTCAATG---GCGCTGACGGTACAGGCCAGACAATTATTGTCTGGTATAGTGCAACAGCAGAACAATTTGCTGAGGGCTATTGAGGCGCAACAGCATCTGTTGCAACTCACAGTCTGGGGCATCAAGCAGCTCCAGGCAAGA---GTCCTGGCTGTGGAAAGATACCTAAAGGATCAACAGCTCCTAGGGATTTGGGGTTGCTCTGGAAAACTCATTTGCACCACTACTGTGCCTTGGAATGCTAGTTGGAGT---------------------------AATAAATCTCTGGATGATATTTGGCAT---AACATGACCTGGATGCAGTGGGAAAAAGAGATTAAC------AATTACACAGATTTAATATACACCTTAATTGAAGAATCGCAAAACCAACAAGAAAAGAATGAACAAGAATTACTGGAATTAGATAAATGGGACAGTTTGTGGAATTGGTTTAACATAACAAACTGGCTGTGGTATATAAAAATATTCATAATGATAGTAGGAGGCTTGGTAGGTTTAAGAATAGTTTTTATTGTACTTTCTGTAGTGAATAGAGTTAGGCAGGGATACTCACCATTATCATTTCAGACCCGC---CTCCCAGTCCCGAGGGAA------CCCGACAGGCCCGAAGGAACAGAAGAAGAAGGTGGAGAGAAAGACAGAGGCAGATCCGGACGTTTAGTGGATGGATTCTTAGCACTTATCTGGGACGATCTGCGGAGCCTGTGCCTCTTCAGCTACCACCGCTTGAGAGACTTACTCTTGATTGTAGCGAGGATTGTGGAACTTCTGGGACGCAGG---------------GGGTGGGGAGTTCTCAAATATTGGTGG---AATCTCCTGCAGTATTGG---------------------------------------------------AGCCAGGAACTAAAGAATAGTGCTGTTAGCTTGCTCAATGCCACAGCTATTGCAGTAGCTGAGGGGACAGATAGGATTATAGAAGTATTACAAAGA------------------CTTTATAGAGCTTTTCTTCACATACCTAGAAGAATAAGACAGGGCTTCGAAAGGGCTTTGTTATAA

2.BORI0637.UAB.EU576298 ATGAGAGCGAAGGAGATCAGGAAGAATTGTCAGCGCTTG---------TGGAGATGGGGC------------------------ACCATGCTCCTTGGGATGTTGATGATC------------TGTAGTGCTGCA---------GGCAATTTGTGGGTCACAGTCTATTATGGGGTACCTGTGTGGAAAGAAGCAACCACCACTCTATTTTGTGCATCAGATGCTAAAGCATATGATACAGAGGTACATAAT---GTTTGGGCCACACATGCCTGTGTACCCACAGACCCCAACCCACAAGAAGTAGTACTG---AAAAATGTGACAGAAAATTTTAACATGTGGAAAAATAACATGGTAGAACAGATGCATGAGGATATAATCAGTTTATGGGATCAAAGCTTAAAGCCATGTGTAAAGCTAACCCCACTCTGTGTTACTTTAAATTGCACTGATCGGGTGGGGAATGCTACTCATACCAATAGT------------------------------------------------------------------------------------------AGTATGGAAGGAGAA------------ATAAAAAACTGCTCTTTCAATGTCACC---ACAAGAATAAGAGAT------AAGGTGCAGAAAGAATATGCACTTTTTTATAAACTTGATGTAGTACCAATAGATAAGAAT------------------------------AATACTAGC---------------------------TATAGGTTGATAAATTGTAATACCTCAGTCATTACACAAGCCTGTCCAAAGGTATCCTTTGAGCCAATTCCCATACATTATTGTACCCCGGCTGGTTTTGCAATTCTAAAATGT---AAAGATAAGAAGTTCAATGGAACAGGATCATGTGAAAATGTCAGCACGGTACATTGTACACATGGAATTAGACCAGTAGTGTCAACTCAACTGCTGTTAAATGGCAGTCTAGCAGAAGAA---GAGGTAGTAATTAGATCTGAAAATTTTACGAACAATGCTAAAACCATAATAGTACAGTTGAATGAGACTGTAGAAATTAATTGTACAAGACCCAACAACAATACAAGAAAAGGTATACATATAGGA------------CCAGGAAAA---ACATTTTATACAACAGGAGGAATAATAGGAGATATAAGGCAAGCATATTGTAACCTT------AGTAGAGCAAAATGGGAGGACACTTTAAAAAAGATAGTCACAAAATTAGGA---GAACAATATGGG---------AATAATAAAACA---ATAGTCTTTAATCAC---------TCCTCAGGAGGGGACCCAGAAATTGTAATGCACAGTTTTAATTGTGGAGGAGAATTTTTCTACTGTAATTCAACACAACTGTTTAATAGTACTTGGAAGTTT------AATAATAGTACTTGG------------------------------AATTTTAATAGTACTTGG------------AATAATACTGAAGGGACA---AATAACACTGAA------ATCACACTCCCATGCAGAATAAAGCAAATTATAAACATGTGGCAGGAAGTAGGAAAAGCAATGTATGCCCCTCCCATCAGCGGACAAATTAGATGTTCATCTAATATTACAGGGCTGCTATTAACAAGAGATGGTGGGGGTAATGGG---------AGTGGGACC---------------------------ACT---GAGATCTTCAGACCTGGAGGGGGAGATATGAGGGACAATTGGAGA---AGTGAACTATACAAATATAAAGTAGTAAAAATTGAACCA---TTAGGAGTAGCACCT---ACCAAGGCAAAGAGAAGAGTGGTGCAAAGAGAA---AAAAGAGCGGTG---GGAGCGCTAGGA---GCTATG---TTCCTT---GGG---------TTCTTGGGA---GCAGCAGGAAGCACTATGGGCGCAGCGTCAATG---GCGCTGACGGTACAGGCCAGACAATTATTGTCTGGTATAGTGCAACAGCAGAACAATTTGCTGAGGGCTATTGAGGCGCAACAGCATCTGTTGCAACTCACAGTCTGGGGCATCAAGCAGCTCCAGGCAAGA---GTCCTGGCTGTGGAAAGATACCTAAAGGATCAACAGCTCCTAGGGATTTGGGGTTGCTCTGGAAAACTCATTTGCACCACTACTGTGCCTTGGAATGCTAGTTGGAGT---------------------------AATAAATCTCTGGATGATATTTGGCAT---AACATGACCTGGATGCAGTGGGAAAAAGAGATTAAC------AATTACACAGATTTAATATACACCTTAATTGAAGAATCGCAAAACCAACAAGAAAAGAATGAACAAGAATTACTGGAATTAGATAAATGGGACAGTTTGTGGAATTGGTTTAACATAACAAACTGGCTGTGGTATATAAAAATATTCATAATGATAGTAGGAGGCTTGGTAGGTTTAAGAATAGTTTTTATTGTACTTTCTGTAGTGAATAGAGTTAGGCAGGGATACTCACCATTATCATTTCAGACCCGC---CTCCCAGTCCCGAGGGAA------CCCGACAGGCCCGAAGGAACAGAAGAAGAAGGTGGAGAGAAAGACAGAGGCAGATCCGGACGTTTAGTGGATGGATTCTTAGCACTTATCTGGGACGATCTGCGGAGCCTGTGCCTCTTCAGCTACCACCGCTTGAGAGACTTACTCTTGATTGTAGCGAGGATTGTGGAACTTCTGGGACGCAGG---------------GGGTGGGGAGTTCTCAAATATTGGTGG---AATCTCCTGCAGTATTGG---------------------------------------------------AGCCAGGAACTAAAGAATAGTGCTGTTAGCTTGCTCAATGCCACAGCTATTGCAGTAGCTGAGGGGACAGATAGGATTATAGAAGTATTACAAAGA------------------CTTTATAGAGCTTTTCTTCACATACCTAGAAGAATAAGACAGGGCTTCGAAAGGGCTTTGTTATAA

2.BORI0637.UAB.EU576284 ATGAGAGCGAAGGAGATCAGGAAGAATTGTCAGCGCTTG---------TGGAGATGGGGC------------------------ACCATGCTCCTTGGGATGTTGATGATC------------TGTAGTGCTGCA---------GGCAATTTGTGGGTCACAGTCTATTATGGGGTACCTGTGTGGAAAGAAGCAACCACCACTCTATTTTGTGCATCAGATGCTAAAGCATATGATACAGAGGTACATAAT---GTTTGGGCCACACATGCCTGTGTACCCACAGACCCCAACCCACAAGAAGTAGTACTG---AAAAATGTGACAGAAAATTTTAACATGTGGAAAAATAACATGGTAGAACAGATGCATGAGGATATAATCAGTTTATGGGATCAAAGCTTAAAGCCATGTGTAAAGCTAACCCCACTCTGTGTTACTTTAAATTGCACTGATCGGGTGGGGAATGCTACTCATACCAATAGT------------------------------------------------------------------------------------------AGTATGGAAGGAGAA------------ATAAAAAACTGCTCTTTCAATGTCACC---ACAAGAATAAGAGAT------AAGGTGCAGAAAGAATATGCACTTTTTTATAAACTTGATGTAGTACCAATAGATAAGAAT------------------------------AATACTAGC---------------------------TATAGGTTGATAAATTGTAATACCTCAGTCATTACACAAGCCTGTCCAAAGGTATCCTTTGAGCCAATTCCCATACATTATTGTACCCCGGCTGGTTTTGCAATTCTAAAATGT---AAAGATAAGAAGTTCAATGGAACAGGATCATGTGAAAATGTCAGCACGGTACATTGTACACATGGAATTAGACCAGTAGTGTCAACTCAACTGCTGTTAAATGGCAGTCTAGCAGAAGAA---GAGGTAGTAATTAGATCTGAAAATTTTACGAACAATGCTAAAACCATAATAGTACAGTTGAATGAGACTGTAGAAATTAATTGTACAAGACCCAACAACAATACAAGAAAAGGTATACATATAGGA------------CCAGGAAAA---ACATTTTATACAACAGGAGGAATAATAGGAGATATAAGGCAAGCATATTGTAACCTT------AGTAGAGCAAAATGGGAGGACACTTTAAAAAAGATAGTCACAAAATTAGGA---GAACAATATGGG---------AATAATAAAACA---ATAGTCTTTAATCAC---------TCCTCAGGAGGGGACCCAGAAATTGTAATGCACAGTTTTAATTGTGGAGGAGAATTTTTCTACTGTAATTCAACACAACTGTTTAATAGTACTTGGAAGTTT------AATAATAGTACTTGG------------------------------AATTTTAATAGTACTTGG------------AATAATACTGAAGGGACA---AATAACACTGAA------ATCACACTCCCATGCAGAATAAAGCAAATTATAAACATGTGGCAGGAAGTAGGAAAAGCAATGTATGCCCCTCCCATCAGCGGACAAATTAGATGTTCATCTAATATTACAGGGCTGCTATTAACAAGAGATGGTGGGGGTAATGGG---------AGTGGGACC---------------------------ACT---GAGATCTTCAGACCTGGAGGGGGAGATATGAGGGACAATTGGAGA---AGTGAACTATACAAATATAAAGTAGTAAAAATTGAACCA---TTAGGAGTAGCACCT---ACCAAGGCAAAGAGAAGAGTGGTGCAAAGAGAA---AAAAGAGCGGTG---GGAGCGCTAGGA---GCTATG---TTCCTT---GGG---------TTCTTGGGA---GCAGCAGGAAGCACTATGGGCGCAGCGTCAATG---GCGCTGACGGTACAGGCCAGACAATTATTGTCTGGTATAGTGCAACAGCAGAACAATTTGCTGAGGGCTATTGAGGCGCAACAGCATCTGTTGCAACTCACAGTCTGGGGCATCAAGCAGCTCCAGGCAAGA---GTCCTGGCTGTGGAAAGATACCTAAAGGATCAACAGCTCCTAGGGATTTGGGGTTGCTCTGGAAAACTCATTTGCACCACTACTGTGCCTTGGAATGCTAGTTGGAGT---------------------------AATAAATCTCTGGATGATATTTGGCAT---AACATGACCTGGATGCAGTGGGAAAAAGAGATTAAC------AATTACACAGATTTAATATACACCTTAATTGAAGAATCGCAAAACCAACAAGAAAAGAATGAACAAGAATTACTGGAATTAGATAAATGGGACAGTTTGTGGAATTGGTTTAACATAACAAACTGGCTGTGGTATATAAAAATATTCATAATGATAGTAGGAGGCTTGGTAGGTTTAAGAATAGTTTTTATTGTACTTTCTGTAGTGAATAGAGTTAGGCAGGGATACTCACCATTATCATTTCAGACCCGC---CTCCCAGTCCCGAGGGAA------CCCGACAGGCCCGAAGGAACAGAAGAAGAAGGTGGAGAGAAAGACAGAGGCAGATCCGGACGTTTAGTGGATGGATTCTTAGCACTTATCTGGGACGATCTGCGGAGCCTGTGCCTCTTCAGCTACCACCGCTTGAGAGACTTACTCTTGATTGTAGCGAGGATTGTGGAACTTCTGGGACGCAGG---------------GGGTGGGGAGTTCTCAAATATTGGTGG---AATCTCCTGCAGTATTGG---------------------------------------------------AGCCAGGAACTAAAGAATAGTGCTGTTAGCTTGCTCAATGCCACAGCTATTGCAGTAGCTGAGGGGACAGATAGGATTATAGAAGTATTACAAAGA------------------CTTTATAGAGCTTTTCTTCACATACCTAGAAGAATAAGACAGGGCTTCGAAAGGGCTTTGTTATAA

2.BORI0637.UAB.EU576285 ATGAGAGCGAAGGAGATCAGGAAGAATTGTCAGCGCTTG---------TGGAGATGGGGC------------------------ACCATGCTCCTTGGGATGTTGATGATC------------TGTAGTGCTGCA---------GGCAATTTGTGGGTCACAGTCTATTATGGGGTACCTGTGTGGAAAGAAGCAACCACCACTCTATTTTGTGCATCAGATGCTAAAGCATATGATACAGAGGTACATAAT---GTTTGGGCCACACATGCCTGTGTACCCACAGACCCCAACCCACAAGAAGTAGTACTG---AAAAATGTGACAGAAAATTTTAACATGTGGAAAAATAACATGGTAGAACAGATGCATGAGGATATAATCAGTTTATGGGATCAAAGCTTAAAGCCATGTGTAAAGCTAACCCCACTCTGTGTTACTTTAAATTGCACTGATCGGGTGGGGAATGCTACTCATACCAATAGT------------------------------------------------------------------------------------------AGTATGGAAGGAGAA------------ATAAAAAACTGCTCTTTCAATGTCACC---ACAAGAATAAGAGAT------AAGGTGCAGAAAGAATATGCACTTTTTTATAAACTTGATGTAGTACCAATAGATAAGAAT------------------------------AATACTAGC---------------------------TATAGGTTGATAAATTGTAATACCTCAGTCATTACACAAGCCTGTCCAAAGGTATCCTTTGAGCCAATTCCCATACATTATTGTACCCCGGCTGGTTTTGCAATTCTAAAATGT---AAAGATAAGAAGTTCAATGGAACAGGATCATGTGAAAATGTCAGCACGGTACATTGTACACATGGAATTAGACCAGTAGTGTCAACTCAACTGCTGTTAAATGGCAGTCTAGCAGAAGAA---GAGGTAGTAATTAGATCTGAAAATTTTACGAACAATGCTAAAACCATAATAGTACAGTTGAATGAGACTGTAGAAATTAATTGTACAAGACCCAACAACAATACAAGAAAAGGTATACATATAGGA------------CCAGGAAAA---ACATTTTATACAACAGGAGGAATAATAGGAGATATAAGGCAAGCATATTGTAACCTT------AGTAGAGCAAAATGGGAGGACACTTTAAAAAAGATAGTCACAAAATTAGGA---GAACAATATGGG---------AATAATAAAACA---ATAGTCTTTAATCAC---------TCCTCAGGAGGGGACCCAGAAATTGTAATGCACAGTTTTAATTGTGGAGGAGAATTTTTCTACTGTAATTCAACACAACTGTTTAATAGTACTTGGAAGTTT------AATAATAGTACTTGG------------------------------AATTTTAATAGTACTTGG------------AATAATACTGAAGGGACA---AATAACACTGAA------ATCACACTCCCATGCAGAATAAAGCAAATTATAAACATGTGGCAGGAAGTAGGAAAAGCAATGTATGCCCCTCCCATCAGCGGACAAATTAGATGTTCATCTAATATTACAGGGCTGCTATTAACAAGAGATGGTGGGGGTAATGGG---------AGTGGGACC---------------------------ACT---GAGATCTTCAGACCTGGAGGGGGAGATATGAGGGACAATTGGAGA---AGTGAACTATACAAATATAAAGTAGTAAAAATTGAACCA---TTAGGAGTAGCACCT---ACCAAGGCAAAGAGAAGAGTGGTGCAAAGAGAA---AAAAGAGCGGTG---GGAGCGCTAGGA---GCTATG---TTCCTT---GGG---------TTCTTGGGA---GCAGCAGGAAGCACTATGGGCGCAGCGTCAATG---GCGCTGACGGTACAGGCCAGACAATTATTGTCTGGTATAGTGCAACAGCAGAACAATTTGCTGAGGGCTATTGAGGCGCAACAGCATCTGTTGCAACTCACAGTCTGGGGCATCAAGCAGCTCCAGGCAAGA---GTCCTGGCTGTGGAAAGATACCTAAAGGATCAACAGCTCCTAGGGATTTGGGGTTGCTCTGGAAAACTCATTTGCACCACTACTGTGCCTTGGAATGCTAGTTGGAGT---------------------------AATAAATCTCTGGATGATATTTGGCAT---AACATGACCTGGATGCAGTGGGAAAAAGAGATTAAC------AATTACACAGATTTAATATACACCTTAATTGAAGAATCGCAAAACCAACAAGAAAAGAATGAACAAGAATTACTGGAATTAGATAAATGGGACAGTTTGTGGAATTGGTTTAACATAACAAACTGGCTGTGGTATATAAAAATATTCATAATGATAGTAGGAGGCTTGGTAGGTTTAAGAATAGTTTTTATTGTACTTTCTGTAGTGAATAGAGTTAGGCAGGGATACTCACCATTATCATTTCAGACCCGC---CTCCCAGTCCCGAGGGAA------CCCGACAGGCCCGAAGGAACAGAAGAAGAAGGTGGAGAGAAAGACAGAGGCAGATCCGGACGTTTAGTGGATGGATTCTTAGCACTTATCTGGGACGATCTGCGGAGCCTGTGCCTCTTCAGCTACCACCGCTTGAGAGACTTACTCTTGATTGTAGCGAGGATTGTGGAACTTCTGGGACGCAGG---------------GGGTGGGGAGTTCTCAAATATTGGTGG---AATCTCCTGCAGTATTGG---------------------------------------------------AGCCAGGAACTAAAGAATAGTGCTGTTAGCTTGCTCAATGCCACAGCTATG------------GGGACAGATAGGATTATAGAAGTATTACAAAGA------------------CTTTATAGAGCTTTTCTTCACATACCTAGAAGAATAAGACAGGGCTTCGAAAGGGCTTTGTTATAA

2.BORI0637.UAB.EU576281 ATGAGAGCGAAGGAGATCAGGAAGAATTGTCAGCGCTTG---------TGGAGATGGGGC------------------------ACCATGCTCCTTGGGATGTTGATGATC------------TGTAGTGCTGCA---------GGCAATTTATGGGTCACAGTCTATTATGGGGTACCTGTGTGGAAAGAAGCAACCACTACTCTATTTTGTGCATCAGATGCTAAAGCATATGATACAGAGGTACATAAT---GTTTGGGCCACACATGCCTGTGTACCCACAGACCCCAACCCACAAGAAGTAGTACTG---AAAAATGTGACAGAAAATTTTAACATGTGGAAAAATAACATGGTAGAACAGATGCATGAGGATATAATCAGTTTATGGGATCAAAGCTTAAAGCCATGTGTAAAACTAACTCCACTCTGTGTTACTTTAAATTGCACTGATCGGGTGGGGAATGTTACTCATACCAAT---------------------------------------------------------------------------------------------AGTAGTGATAAGGAAATGAGGGGAGAAATAAAAAACTGCTCTTTCAACGTCACC---ACAAGAATAAGAGAT------AAGGTGCAGAAAGAATATGCACTTTTTTATAAACTTGATGTAGTACCAATAGAT---GATAATGAT------------------------AATACCAGC---------------------------TATAGATTGATAAATTGTAATACCTCAGTCATTACACAGGCCTGTCCAAAGGTATCCTTTGAGCCAATTCCCATACATTATTGTACCCCGGCTGGTTTTGCAATTCTAAAATGT---AAAGATAAGAAATTCAATGGAACAGGACAATGTAAAAATGTCAGCACAGTACAATGTACACATGGAATTAGACCAGTAGTGTCAACTCAACTGTTGTTAAATGGCAGTCTAGCAGAAGAA---GAGGTAGTAATTAGATCTGAAAATTTTACGAACAATGCTAAAACCATAATAGTACAGCTGAATGAAACTGTAGAAATTAATTGTACAAGACCCAACAACAATACAAGAAAAAGTATACATATAGGA------------CCAGGGAGA---ACATTTTATACAACAGGAGACATAATAGGAGATATAAGGCAAGCATATTGTATCCTT------AGTAGAGCAAAATGGGAAGACACTTTAAAAAAGATAGTCACAAAATTAGGA---GAACAATATGGG---------AATAATAAAACA---ATAGTCTTTAATCAC---------TCCTCAGGAGGGGACCCAGAAATTGTAATGCACAGTTTTAATTGTGGAGGGGAATTTTTCTACTGTAATTCAACACAACTGTTTAATAGTACTTGGAGTTTT---------AATAGTACTTGG------------------------AATAAGAATTTTAATAGTACTTGG------------AATATTACTGAAGGGACA---AATAACACTGAA------ATCACACTCCCATGCAGAATAAAGCAAATTATAAACATGTGGCAGGAAGTAGGAAAAGCAATGTATGCCCCTCCCATCAGCGGACAAATTAAATGTTCATCTAATATTACAGGGCTGCTATTAACAAGAGATGGTGGGAATAATGAG---------AACAAGACC---------------------------ACT---GAGATCTTCAGACCTGGAGGGGGAGATATGAGGGACAATTGGAGA---AGTGAACTATACAAATATAAAGTAGTAAAAATTGAACCA---TTAGGAGTAGCACCT---ACCAAGGCAAAGAGAAGAGTGGTGCAAAGAGAA---AAAAGAGCAGTG---GGAGCAATAGGA---GCTATG---TTCCTT---GGG---------TTCTTGGGA---GCAGCAGGAAGCACTATGGGCGCAGCGTCAATA---GCGCTGACGGTACAGGCCAGACAATTATTGTCTGGTATAGTGCAACAGCAGAACAATTTGCTAAGGGCTATTGAGGCGCAACAGCATCTGTTGCAACTCACAGTCTGGGGCATCAAGCAGCTCCAGGCAAGA---GTCCTGGCTGTGGAAAGATACCTAAAGGACCAACAGCTCCTAGGGATTTGGGGTTGCTCTGGAAAACTCATTTGCACCACTACTGTGCCTTGGAATACTAGTTGGAGT---------------------------AATAAATCTCTGGATGATATTTGGCAT---AACATGACCTGGATGCAGTGGGAAAAAGAGATTAAC------AATTACACAGATTTAATATACACCTTAATTGAAGAATCGCAAAACCAACAAGAAAAGAATGAACAAGAATTATTGGAATTAGATAAATGGGACAGTTTGTGGAATTGGTTTAACATAACAAACTGGCTGTGGTATATAAAAATATTCATAATGATAGTAGGAGGCTTGATAGGTTTAAGAATAGTTTTTATTGTACTTTCTGTAGTGAATAGAGTTAGGCAGGGATACTCACCATTGTCATTTCAGACCCGC---CTCCCAGTCCCGAGGGAA------CCCGACAGGCCCGAAGGAACAGAAGAAGAAGGTGGAGAGAAAGACAGAGGCAGATCCGGACGATTAGTGGATGGATTCTTAGCACTTATCTGGGACGATCTGCGGAGCCTGTGCCTCTTCAGCTACCACCGCTTGAGAGACTTACTCTTGATTGTAGCGAGGATTGTGGAACTTCTGGGACGCAGG---------------GGGTGGGGAGTTCTCAAATATTGGTGG---AATCTCCTGCAGTATTGG---------------------------------------------------AGTCAGGAACTAAAGAATAGTGCTGTTAGCTTGCTCAATGCCACAGCTATTGCAGTAGCTGAGGGGACAGATAGGATTATAGAAGTATTACAAAGA------------------CTTTATAGAGCTTTTCTTCACATACCTAGAAGAATAAGACAGGGCTTCGAAAGGGCTTTGTTATAA

2.BORI0637.UAB.EU576288 ATGAGAGCGAAGGAGATCAGGAAGAATTGTCAGCGCTTG---------TGGAGATGGGGC------------------------ACCATGCTCCTTGGGATGTTGATGATC------------TGTAGTGCTGCA---------GGCAATTTATGGGTCACAGTCTATTATGGGGTACCTGTGTGGAAAGAAGCAACCACTACTCTATTTTGTGCATCAGATGCTAAAGCATATGATACAGAGGTACATAAT---GTTTGGGCCACACATGCCTGTGTACCCACAGACCCCAACCCACAAGAAGTAGTACTG---AAAAATGTGACAGAAAATTTTAACATGTGGAAAAATAACATGGTAGAACAGATGCATGAGGATATAATCAGTTTATGGGATCAAAGCTTAAAGCCATGTGTAAAACTAACTCCACTCTGTGTTACTTTAAATTGCACTGATCGGGTGGGGAATGTTACTCATACCAAT---------------------------------------------------------------------------------------------AGTAGTGATAAGGAAATGAGGGGAGAAATAAAAAACTGCTCTTTCAACGTCACC---ACAAGAATAAGAGAT------AAGGTGCAGAAAGAATATGCACTTTTTTATAAACTTGATGTAGTACCAATAGAT---GATAATGAT------------------------AATACCAGC---------------------------TATAGATTGATAAATTGTAATACCTCAGTCATTACACAGGCCTGTCCAAAGGTATCCTTTGAGCCAATTCCCATACATTATTGTACCCCGGCTGGTTTTGCAATTCTAAAATGT---AAAGATAAGAAATTCAATGGAACAGGACAATGTAAAAATGTCAGCACAGTACAATGTACACATGGAATTAGACCAGTAGTGTCAACTCAACTGTTGTTAAATGGCAGTCTAGCAGAAGAA---GAGGTAGTAATTAGATCTGAAAATTTTACGAACAATGCTAAAACCATAATAGTACAGCTGAATGAAACTGTAGAAATTAATTGTACAAGACCCAACAACAATACAAGAAAAAGTATACATATAGGA------------CCAGGGAGA---ACATTTTATACAACAGGAGACATAATAGGAGATATAAGGCAAGCATATTGTATCCTT------AGTAGAGCAAAATGGGAAGACACTTTAAAAAAGATAGTCACAAAATTAGGA---GAACAATATGGG---------AATAATAAAACA---ATAGTCTTTAATCAC---------TCCTCAGGAGGGGACCCAGAAATTGTAATGCACAGTTTTAATTGTGGAGGGGAATTTTTCTACTGTAATTCAACACAACTGTTTAATAGTACTTGGAGTTTT---------AATAGTACTTGG------------------------AATAAGAATTTTAATAGTACTTGG------------AATATTACTGAAGGGACA---AATAACACTGAA------ATCACACTCCCATGCAGAATAAAGCAAATTATAAACATGTGGCAGGAAGTAGGAAAAGCAATGTACGCCCCTCCCATCAGCGGACAAATTAAATGTTCATCTAATATTACAGGGCTGCTATTAACAAGAGATGGTGGGAATAATGAG---------AACAAGACC---------------------------ACT---GAGATCTTCAGACCTGGAGGGGGAGATATGAGGGACAATTGGAGA---AGTGAACTATACAAATATAAAGTAGTAAAAATTGAACCA---TTAGGAGTAGCACCT---ACCAAGGCAAAGAGAAGAGTGGTGCAAAGAGAA---AAAAGAGCAGTG---GGAGCAATAGGA---GCTATG---TTCCTT---GGG---------TTCTTGGGA---GCAGCAGGAAGCACTATGGGCGCAGCGTCAATA---GCGCTGACGGTACAGGCCAGACAATTATTGTCTGGTATAGTGCAACAGCAGAACAATTTGCTAAGGGCTATTGAGGCGCAACAGCATCTGTTGCAACTCACAGTCTGGGGCATCAAGCAGCTCCAGGCAAGA---GTCCTGGCTGTGGAAAGATACCTAAAGGACCAACAGCTCCTAGGGATTTGGGGTTGCTCTGGAAAACTCATTTGCACCACTACTGTGCCTTGGAATACTAGTTGGAGT---------------------------AATAAATCTCTGGATGATATTTGGCAT---AACATGACCTGGATGCAGTGGGAAAAAGAGATTAAC------AATTACACAGATTTAATATACACCTTAATTGAAGAATCGCAAAACCAACAAGAAAAGAATGAACAAGAATTATTGGAATTAGATAAATGGGACAGTTTGTGGAATTGGTTTAACATAACAAACTGGCTGTGGTATATAAAAATATTCATAATGATAGTAGGAGGCTTGATAGGTTTAAGAATAGTTTTTATTGTACTTTCTGTAGTGAATAGAGTTAGGCAGGGATACTCACCATTGTCATTTCAGACCCGC---CTCCCAGTCCCGAGGGAA------CCCGACAGGCCCGAAGGAACAGAAGAAGAAGGTGGAGAGAAAGACAGAGGCAGATCCGGACGATTAGTGGATGGATTCTTAGCACTTATCTGGGACGATCTGCGGAGCCTGTGCCTCTTCAGCTACCACCGCTTGAGAGACTTACTCTTGATTGTAGCGAGGATTGTGGAACTTCTGGGACGCAGG---------------GGGTGGGGAGTTCTCAAATATTGGTGG---AATCTCCTGCAGTATTGG---------------------------------------------------AGTCAGGAACTAAAGAATAGTGCTGTTAGCTTGCTCAATGCCACAGCTATTGCAGTAGCTGAGGGGACAGATAGGATTATAGAAGTATTACAAAGA------------------CTTTATAGAGCTTTTCTTCACATACCTAGAAGAATAAGACAGGGCTTCGAAAGGGCTTTGTTATAA

2.BORI0637.UAB.EU576280 ATGAGAGCGAAGGAGATCAGGAAGAATTGTCAGCGCTTG---------TGGAGATGGGGC------------------------ACCATGCTCCTTGGGATGTTGATGATC------------TGTAGTGCTGCA---------GGCAATTTGTGGGTCACAGTCTATTATGGGGTACCTGTGTGGAAAGAAGCAACCACCACTCTATTTTGTGCATCAGATGCTAAAGCATATGATACAGAGGTACATAAT---GTTTGGGCCACACATGCCTGTGTACCCACAGACCCCAACCCACAAGAAGTAGTACTG---AAAAATGTGACAGAAGATTTTAACATGTGGAAAAATAACATGGTAGAACAGATGCATGAGGATATAATCAGTTTATGGGATCAAAGCCTAAAGCCATGTGTAAAACTAACTCCACTCTGTGTTACTTTAAATTGCACTGATCATTTGTGGAATGTTACTAATACCATGAGGAATGCT---------------------------------------------------------------------ACTAATACCACTAGTAGTAAGGAGGGAGAAATGAGAGGAGAAATAAAAAACTGCTCTTTCAATGTCACC---ACAAGCATAAGAGAT------AAGGTGCAGAAAGAATATGCACTTTTTTATAAACTTGATGTAGTACCAATAGATAATGATAATGAT------------------------AATACTAGC---------------------------TATAGGTTGATAAATTGTAATACCTCAGTCATTACACAAGCCTGTCCAAAAGTATCTTTTGAGCCAATTCCCATACATTATTGTACCCCGGCTGGTTTTGCAATTCTAAAGTGT---AAAGATAAGAAATTCAATGGAACAGGACAATGTGAAAATGTCAGCACAGTACAATGTACACATGGAATTAGACCAGTAGTGTCAACTCAACTGTTGTTAAATGGCAGTCTAGCAGAAGAA---GAGGTAGTAATTAGATCTGAAAATTTTACGAACAATGCTAAAACCATAATAGTACAGCTGAATGAAACTGTAGAAATTAATTGTATAAGACCCAACAACAATACAAGAAAAGGTATACATATAGGA------------CCAGGGAGA---ACATTTTATACAACAGGAGATATAATAGGAGATATAAGGCAAGCATATTGTAACCTT------AGTAGAACAAAATGGGAAGACACTTTAAAAAAGATAGTCACAAAATTAGGA---GAACAATATGGG---------AATAATAAAACA---ATAGTCTTTAATCAC---------TCCTCAGGAGGGGACCCAGAAATTGTAATGCACAGTTTTAATTGTGGAGGGGAATTTTTCTACTGTAATTCAACACAACTGTTTAATAGTACTTGGAAGTTT------AATAATAGTACTTGG------------------------------AATTTTAATAGTACTTGG------------AATAATACTGAAAGGACA---AATAACACA---------ATCACACTCCCATGCAGAATAAAACAAATTATAAACATGTGGCAGGAAGTAGGAAAAGCAATGTATGCCCCTCCCATCAGAGGACAAATTAGATGTTCATCTAATATTACAGGGCTGCTATTAACAAGAGATGGTGGGAATAATGAG---------AACAAGACC---------------------------ACT---GAGATCTTCAGACCTGGAGGGGGAGATATGAGGGACAATTGGAGA---AGTGAACTATACAAATATAAAGTAGTAAAAATTGAACCA---TTAGGAGTAGCACCT---ACCAAGGCAAAGAGAAGAGTGGTGCAAAGAGAA---AAAAGAGCGGTG---GGAGCAATAGGA---GCTATG---TTCCTT---GGG---------TTCTTGGGA---GCAGCAGGAAGCACTATGGGCGCAGCGTCAATG---GCGCTGACGGTACAGGCCAGACAATTATTGTCTGGTATAGTGCAACAGCAGAACAATTTGCTGAGGGCTATTGAGGCGCAACAGCATCTGTTGCAACTCACAGTCTGGGGCATCAAGCAGCTCCAGGCAAGA---GTCCTGGCTGTGGAAAGATACCTAAGGGATCAACAGCTCCTAGGGATTTGGGGCTGCTCTGGAAAACTCATTTGCACCACTACTGTGCCTTGGAATACTAGTTGGAGT---------------------------AATAAATCTCTGGATGATATTTGGCAT---AACATGACCTGGATGCAGTGGGAAAAAGAGATTAAC------AATTACACAGATTTAATATACACCTTAATTGAAGAATCGCAAAACCAACAAGAAAAGAATGAACAAGAATTATTGGAATTAGATAAATGGGACAGTTTGTGGAATTGGTTTAACATAACAAACTGGCTGTGGTATATAAAAATATTCATAATGATAGTAGGAGGCTTGATAGGTTTAAGAATAGTTTTTATTGTACTTTCTGTAGTGAATAGAGTTAGGCAGGGATACTCACCATTATCATTTCAGACCCGC---CTCCCAGTCCCGAGGGAA------CCCGACAGGCCCGGAGGAACAGAAGAAGAAGGTGGAGAGAAAGACAGAGGCAGATCCGGACGTTTAGTGGATGGATTCTTAGCACTTATCTGGGACGATCTGCGGAGCCTGTTCCTCTTCAGCTACCAGCGCTTGAGAGACTTACTCTTGATTGTAGCGAGGATTGTGGAACTTCTGGGACGCAGG---------------GGGTGGGGAGTTCTCAAATATTGGTGG---AATCTCCTGCAGTATTGG---------------------------------------------------AGTCAGGAACTAAAGAATAGTGCTGTTAGCTTGCTCAATGCCACAGCTATCGCAGTAGCTGAGGGGACAGATAGGGTTATAGAAGTATTACAAAGA------------------ATTTATAGAGCCTTTCTCCACATCCCTAGAAGAATAAGACAGGGCTTCGAAAGGGCTTTGTTATAA

2.BORI0637.UAB.EU576296 ATGAGAGCGAAGGAGATCAGGAAGAATTGTCAGCGCTTG---------TGGAGATGGGGC------------------------ACCATGCTCCTTGGGATGTTGATGATC------------TGTAGTGCTGCA---------GGCAATTTGTGGGTCACAGTCTATTATGGGGTACCTGTGTGGAAAGAAGCAACCACCACTCTATTTTGTGCATCAGATGCTAAAGCATATGATACAGAGGTACATAAT---GTTTGGGCCACACATGCCTGTGTACCCACAGACCCCAACCCACAAGAAGTAGTACTG---AAAAATGTGACAGAAGATTTTAACATGTGGAAAAATAACATGGTAGAACAGATGCATGAGGATATAATCAGTTTATGGGATCAAAGCCTAAAGCCATGTGTAAAACTAACTCCACTCTGTGTTACTTTAAATTGCACTGATCATTTGTGGAATGTTACTAATACCATGAGGAATGCT---------------------------------------------------------------------ACTAATACCACTAGTAGTAAGGAGGGAGAAATGAGAGGAGAAATAAAAAACTGCTCTTTCAATGTCACC---ACAAGCATAAGAGAT------AAGGTGCAGAAAGAATATGCACTTTTTTATAAACTTGATGTAGTACCAATAGATAATGATAATGAT------------------------AATACTAGC---------------------------TATAGGTTGATAAATTGTAATACCTCAGTCATTACACAAGCCTGTCCAAAAGTATCTTTTGAGCCAATTCCCATACATTATTGTACCCCGGCTGGTTTTGCAATTCTAAAGTGT---AAAGATAAGAAATTCAATGGAACAGGACAATGTGAAAATGTCAGCACAGTACAATGTACACATGGAATTAGACCAGTAGTGTCAACTCAACTGTTGTTAAATGGCAGTCTAGCAGAAGAA---GAGGTAGTAATTAGATCTGAAAATTTTACGAACAATGCTAAAACCATAATAGTACAGCTGAATGAAACTGTAGAAATTAATTGTATAAGACCCAACAACAATACAAGAAAAGGTATACATATAGGA------------CCAGGGAGA---ACATTTTATACAACAGGAGATATAATAGGAGATATAAGGCAAGCATATTGTAACCTT------AGTAGAACAAAATGGGAAGACACTTTAAAAAAGATAGTCACAAAATTAGGA---GAACAATATGGG---------AATAATAAAACA---ATAGTCTTTAATCAC---------TCCTCAGGAGGGGACCCAGAAATTGTAATGCACAGTTTTAATTGTGGAGGGGAATTTTTCTACTGTAATTCAACACAACTGTTTAATAGTACTTGGAAGTTT------AATAATAGTACTTGG------------------------------AATTTTAATAGTACTTGG------------AATAATACTGAAAGGACA---AATAACACA---------ATCACACTCCCATGCAGAATAAAACAAATTATAAACATGTGGCAGGAAGTAGGAAAAGCAATGTATGCCCCTCCCATCAGAGGACAAATTAGATGTTCATCTAATATTACAGGGCTGCTATTAACAAGAGATGGTGGGAATAATGAG---------AACAAGACC---------------------------ACT---GAGATCTTCAGACCTGGAGGGGGAGATATGAGGGACAATTGGAGA---AGTGAACTATACAAATATAAAGTAGTAAAAATTGAACCA---TTAGGAGTAGCACCT---ACCAAGGCAAAGAGAAGAGTGGTGCAAAGAGAA---AAAAGAGCGGTG---GGAGCAATAGGA---GCTATG---TTCCTT---GGG---------TTCTTGGGA---GCAGCAGGAAGCACTATGGGCGCAGCGTCAATG---GCGCTGACGGTACAGGCCAGACAATTATTGTCTGGTATAGTGCAACAGCAGAACAATTTGCTGAGGGCTATTGAGGCGCAACAGCATCTGTTGCAACTCACAGTCTGGGGCATCAAGCAGCTCCAGGCAAGA---GTCCTGGCTGTGGAAAGATACCTAAGGGATCAACAGCTCCTAGGGATTTGGGGCTGCTCTGGAAAACTCATTTGCACCACTACTGTGCCTTGGAATACTAGTTGGAGT---------------------------AATAAATCTCTGGATGATATTTGGCAT---AACATGACCTGGATGCAGTGGGAAAAAGAGATTAAC------AATTACACAGATTTAATATACACCTTAATTGAAGAATCGCAAAACCAACAAGAAAAGAATGAACAAGAATTATTGGAATTAGATAAATGGGACAGTTTGTGGAATTGGTTTAACATAACAAACTGGCTGTGGTATATAAAAATATTCATAATGATAGTAGGAGGCTTGATAGGTTTAAGAATAGTTTTTATTGTACTTTCTGTAGTGAATAGAGTTAGGCAGGGATACTCACCATTATCATTTCAGACCCGC---CTCCCAGTCCCGAGGGAA------CCCGACAGGCCCGGAGGAACAGAAGAAGAAGGTGGAGAGAAAGACAGAGGCAGATCCGGACGTTTAGTGGATGGATTCTTAGCACTTATCTGGGACGATCTGCGGAGCCTGTTCCTCTTCAGCTACCAGCGCTTGAGAGACTTACTCTTGATTGTAGCGAGGATTGTGGAACTTCTGGGACGCAGG---------------GGGTGGGGAGTTCTCAAATATTGGTGG---AATCTCCTGCAGTATTGG---------------------------------------------------AGTCAGGAACTAAAGAATAGTGCTGTTAGCTTGCTCAATGCCACAGCTATCGCAGTAGCTGAGGGGACAGATAGGGTTATAGAAGTATTACAAAGA------------------ATTTATAGAGCCTTTCTCCACATCCCTAGAAGAATAAGACAGGGCTTCGAAAGGGCTTTGTTATAA

2.BORI0637.UAB.EU576275 ATGAGAGCGAAGGAGATCAGGAAGAATTGTCAGCGCTTG---------TGGAGATGGGGC------------------------ACCATGCTCCTTGGGATGTTGATGATC------------TGTAGTGCTGCA---------GGCAATTTGTGGGTCACAGTCTATTATGGGGTACCTGTGTGGAAAGAAGCAACCACCACTCTATTTTGTGCATCAGATGCTAAAGCATATGATACAGAGGTACATAAT---GTTTGGGCCACACATGCCTGTGTACCCACAGACCCCAACCCACAAGAAGTAGTACTG---AAAAATGTGACAGAAGATTTTAACATGTGGAAAAATAACATGGTAGAACAGATGCATGAGGATATAATCAGTTTATGGGATCAAAGCCTAAAGCCATGTGTAAAACTAACTCCACTCTGTGTTACTTTAAATTGCACTGATCATTTGTGGAATGTTACTAATACCATGAGGAATGCT---------------------------------------------------------------------ACTAATACCACTAGTAGTAAGGAGGGAGAAATGAGAGGAGAAATAAAAAACTGCTCTTTCAATGTCACC---ACAAGCATAAGAGAT------AAGGTGCAGAAAGAATATGCACT-TTTTATAAACTTGATGTAGTACCAATAGATAATGATAATGAT------------------------AATACTAGC---------------------------TATAGGTTGATAAATTGTAATACCTCAGTCATTACACAAGCCTGTCCAAAAGTATCTTTTGAGCCAATTCCCATACATTATTGTACCCCGGCTGGTTTTGCAATTCTAAAGTGT---AAAGATAAGAAATTCAATGGAACAGGACAATGTGAAAATGTCAGCACAGTACAATGTACACATGGAATTAGACCAGTAGTGTCAACTCAACTGTTGTTAAATGGCAGTCTAGCAGAAGAA---GAGGTAGTAATTAGATCTGAAAATTTTACGAACAATGCTAAAACCATAATAGTACAGCTGAATGAAACTGTAGAAATTAATTGTATAAGACCCAACAACAATACAAGAAAAGGTATACATATAGGA------------CCAGGGAGA---ACATTTTATACAACAGGAGATATAATAGGAGATATAAGGCAAGCATATTGTAACCTT------AGTAGAACAAAATGGGAAGACACTTTAAAAAAGATAGTCACAAAATTAGGA---GAACAATATGGG---------AATAATAAAACA---ATAGTCTTTAATCAC---------TCCTCAGGAGGGGACCCAGAAATTGTAATGCACAGTTTTAATTGTGGAGGGGAATTTTTCTACTGTAATTCAACACAACTGTTTAATAGTACTTGGAAGTTT------AATAATAGTACTTGG------------------------------AATTTTAATAGTACTTGG------------AATAATACTGAAAGGACA---AATAACACA---------ATCACACTCCCATGCAGAATAAAACAAATTATAAACATGTGGCAGGAAGTAGGAAAAGCAATGTATGCCCCTCCCATCAGAGGACAAATTAGATGTTCATCTAATATTACAGGGCTGCTATTAACAAGAGATGGTGGGAATAATGAG---------AACAAGACC---------------------------ACT---GAGATCTTCAGACCTGGAGGGGGAGATATGAGGGACAATTGGAGA---AGTGAACTATACAAATATAAAGTAGTAAAAATTGAACCA---TTAGGAGTAGCACCT---ACCAAGGCAAAGAGAAGAGTGGTGCAAAGAGAA---AAAAGAGCGGTG---GGAGCAATAGGA---GCTATG---TTCCTT---GGG---------TTCTTGGGA---GCAGCAGGAAGCACTATGGGCGCAGCGTCAATG---GCGCTGACGGTACAGGCCAGACAATTATTGTCTGGTATAGTGCAACAGCAGAACAATTTGCTGAGGGCTATTGAGGCGCAACAGCATCTGTTGCAACTCACAGTCTGGGGCATCAAGCAGCTCCAGGCAAGA---GTCCTGGCTGTGGAAAGATACCTAAGGGATCAACAGCTCCTAGGGATTTGGGGCTGCTCTGGAAAACTCATTTGCACCACTACTGTGCCTTGGAATACTAGTTGGAGT---------------------------AATAAATCTCTGGATGATATTTGGCAT---AACATGACCTGGATGCAGTGGGAAAAAGAGATTAAC------AATTACACAGATTTAATATACACCTTAATTGAAGAATCGCAAAACCAACAAGAAAAGAATGAACAAGAATTATTGGAATTAGATAAATGGGACAGTTTGTGGAATTGGTTTAACATAACAAACTGGCTGTGGTATATAAAAATATTCATAATGATAGTAGGAGGCTTGATAGGTTTAAGAATAGTTTTTATTGTACTTTCTGTAGTGAATAGAGTTAGGCAGGGATACTCACCATTATCATTTCAGACCCGC---CTCCCAGTCCCGAGGGAA------CCCGACAGGCCCGGAGGAACAGAAGAAGAAGGTGGAGAGAAAGACAGAGGCAGATCCGGACGTTTAGTGGATGGATTCTTAGCACTTATCTGGGACGATCTGCGGAGCCTGTTCCTCTTCAGCTACCAGCGCTTGAGAGACTTACTCTTGATTGTAGCGAGGATTGTGGAACTTCTGGGACGCAGG---------------GGGTGGGGAGTTCTCAAATATTGGTGG---AATCTCCTGCAGTATTGG---------------------------------------------------AGTCAGGAACTAAAGAATAGTGCTGTTAGCTTGCTCAATGCCACAGCTATCGCAGTAGCTGAGGGGACAGATAGGGTTATAGAAGTATTACAAAGA------------------ATTTATAGAGCCTTTCTCCACATCCCTAGAAGAATAAGACAGGGCTTCGAAAGGGCTTTGTTATAA

2.BORI0637.UAB.EU576290 ATGAGAGCGAAGGAGATCAGGAAGAATTGTCAGCGCTTG---------TGGAGATGGGGC------------------------ACCATGCTCCTTGGGATGTTGATGATC------------TGTAGTGCTGCA---------GGCAATTTGTGGGTCACAGTCTATTATGGGGTACCTGTGTGGAAAGAAGCAACCACCACTCTATTTTGTGCATCAGATGCTAAAGCATATGATACAGAGGTACATAAT---GTTTGGGCCACACATGCCTGTGTACCCACAGACCCCAACCCACAAGAAGTAGTACTG---AAAAATGTGACAGAAGATTTTAACATGTGGAAAAATAACATGGTAGAACAGATGCATGAGGATATAATCAGTTTATGGGATCAAAGCCTAAAGCCATGTGTAAAACTAACTCCACTCTGTGTTACTTTAAATTGCACTGATCATTTGTGGAATGTTACTAATACCATGAGGAATGCT---------------------------------------------------------------------ACTAATACCACTAGTAGTAAGGAGGGAGAAATGAGAGGAGAAATAAAAAACTGCTCTTTCAATGTCACC---ACAAGCATAAGAGAT------AAGGTGCAGAAAGAATATGCACTTTTTTATAAACTTGATGTAGTACCAATAGATAATGATAATGAT------------------------AATACTAGC---------------------------TATAGGTTGATAAATTGTAATACCTCAGTCATTACACAAGCCTGTCCAAAAGTATCTTTTGAGCCAATTCCCATACATTATTGTACCCCGGCTGGTTTTGCAATTCTAAAGTGT---AAAGATAAGAAATTCAATGGAACAGGACAATGTGAAAATGTCAGCACAGTACAATGTACACATGGAATTAGACCAGTAGTGTCCACTCAACTGTTGTTAAATGGCAGTCTAGCAGAAGAA---GAGGTAGTAATTAGATCTGAAAATTTTACGAACAATGCTAAAACCATAATAGTACAGCTGAATGAAACTGTAGAAATTAATTGTATAAGACCCAACAACAATACAAGAAAAGGTATACATATAGGA------------CCAGGGAGA---ACATTTTATACAACAGGAGATATAATAGGAGATATAAGGCAAGCATATTGTAACCTT------AGTAGAACAAAATGGGAAGACACTTTAAAAAAGATAGTCACAAAATTAGGA---GAACAATATGGG---------AATAATAAAACA---ATAGTCTTTAATCAC---------TCCTCAGGAGGGGACCCAGAAATTGTAATGCACAGTTTTAATTGTGGAGGGGAATTTTTCTACTGTAATTCAACACAACTGTTTAATAGTACTTGGAAGTTT------AATAATAGTACTTGG------------------------------AATTTTAATAGTACTTGG------------AATAATACTGAAAGGACA---AATAACACA---------ATCACACTCCCATGCAGAATAAAACAAATTATAAACATGTGGCAGGAAGTAGGAAAAGCAATGTATGCCCCTCCCATCAGAGGACAAATTAGATGTTCATCTAATATTACAGGGCTGCTATTAACAAGAGATGGTGGGAATAATGAG---------AACAAGACC---------------------------ACT---GAGATCTTCAGACCTGGAGGGGGAGATATGAGGGACAATTGGAGA---AGTGAACTATACAAATATAAAGTAGTAAAAATTGAACCA---TTAGGAGTAGCACCT---ACCAAGGCAAAGAGAAGAGTGGTGCAAAGAGAA---AAAAGAGCGGTG---GGAGCAATAGGA---GCTATG---TTCCTT---GGG---------TTCTTGGGA---GCAGCAGGAAGCACTATGGGCGCAGCGTCAATG---GCGCTGACGGTACAGGCCAGACAATTATTGTCTGGTATAGTGCAACAGCAGAACAATTTGCTGAGGGCTATTGAGGCGCAACAGCATCTGTTGCAACTCACAGTCTGGGGCATCAAGCAGCTCCAGGCAAGA---GTCCTGGCTGTGGAAAGATACCTAAGGGATCAACAGCTCCTAGGGATTTGGGGCTGCTCTGGAAAACTCATTTGCACCACTACTGTGCCTTGGAATACTAGTTGGAGT---------------------------AATAAATCTCTGGATGATATTTGGCAT---AACATGACCTGGATGCAGTGGGAAAAAGAGATTAAC------AATTACACAGATTTAATATACACCTTAATTGAAGAATCGCAAAACCAACAAGAAAAGAATGAACAAGAATTATTGGAATTAGATAAATGGGACAGTTTGTGGAATTGGTTTAACATAACAAACTGGCTGTGGTATATAAAAATATTCATAATGATAGTAGGAGGCTTGATAGGTTTAAGAATAGTTTTTATTGTACTTTCTGTAGTGAATAGAGTTAGGCAGGGATACTCACCATTATCATTTCAGACCCGC---CTCCCAGTCCCGAGGGAA------CCCGACAGGCCCGGAGGAACAGAAGAAGAAGGTGGAGAGAAAGACAGAGGCAGATCCGGACGTTTAGTGGATGGATTCTTAGCACTTATCTGGGACGATCTGCGGAGCCTGTTCCTCTTCAGCTACCAGCGCTTGAGAGACTTACTCTTGATTGTAGCGAGGATTGTGGAACTTCTGGGACGCAGG---------------GGGTGGGGAGTTCTCAAATATTGGTGG---AATCTCCTGCAGTATTGG---------------------------------------------------AGTCAGGAACTAAAGAATAGTGCTGTTAGCTTGCTCAATGCCACAGCTATCGCAGTAGCTGAGGGGACAGATAGGGTTATAGAAGTATTACAAAGA------------------ATTTATAGAGCCTTTCTCCACATCCCTAGAAGAATAAGACAGGGCTTCGAAAGGGCTTTGTTATAA

2.BORI0637.UAB.EU576274 ATGAGAGCGAAGGAGATCAGGAAGAATTGTCAGCGCTTG---------TGGAGATGGGGC------------------------ACCATGCTCCTTGGGATGTTGATGATC------------TGTAGTGCTGCA---------GGCAATTTGTGGGTCACAGTCTATTATGGGGTACCTGTGTGGAAAGAAGCAACCACCACTCTATTTTGTGCATCAGATGCTAAAGCATATGATACAGAGGTACATAAT---GTTTGGGCCACACATGCCTGTGTACCCACAGACCCCAACCCACAAGAAGTAGTACTG---AAAAATGTGACAGAAGATTTTAACATGTGGAAAAATAACATGGTAGAACAGATGCATGAGGATATAATCAGTTTATGGGATCAAAGCCTAAAGCCATGTGTAAAACTAACTCCACTCTGTGTTACTTTAAATTGCACTGATCATTTGTGGAATGTTACTAATACCATGAGGAATGCT---------------------------------------------------------------------ACTAATACCACTAGTAGTAAGGAGGGAGAAATGAGAGGAGAAATAAAAAACTGCTCTTTCAATGTCACC---ACAAGCATAAGAGAT------AAGGTGCAGAAAGAATATGCACTTTTTTATAAACTTGATGTAGTACCAATAGATAATGATAATGAT------------------------AATACTAGC---------------------------TATAGGTTGATAAATTGTAATACCTCAGTCATTACACAAGCCTGTCCAAAAGTATCTTTTGAGCCAATTCCCATACATTATTGTACCCCGGCTGGTTTTGCAATTCTAAAGTGT---AAAGATAAGAAATTCAATGGAACAGGACAATGTGAAAATGTCAGCACAGTACAATGTACACATGGAATTAGACCAGTAGTGTCAACTCAACTGTTGTTAAATGGCAGTCTAGCAGAAGAA---GAGGTAGTAATTAGATCTGAAAATTTTACGAACAATGCTAAAACCATAATAGTACAGCTGAATGAAACTGTAGAAATTAATTGTATAAGACCCAACAACAATACAAGAAAAGGTATACATATAGGA------------CCAGGGAGA---ACATTTTATACAACAGGAGATATAATAGGAGATATAAGGCAAGCATATTGTAACCTT------AGTAGAACAAAATGGGAAGACACTTTAAAAAAGATAGTCACAAAATTAGGA---GAACAATATGGG---------AATAATAAAACA---ATAGTCTTTAATCAC---------TCCTCAGGAGGGGACCCAGAAATTGTAATGCACAGTTTTAATTGTGGAGGGGAATTTTTCTACTGTAATTCAACACAACTGTTTAATAGTACTTGGAAGTTT------AATAATAGTACTTGG------------------------------AATTTTAATAGTACTTGG------------AATAATACTGAAAGGACA---AATAACACA---------ATCACACTCCCATGCAGAATAAAACAAATTATAAACATGTGGCAGGAAGTAGGAAAAGCAATGTATGCCCCTCCCATCAGAGGACAAATTAGATGTTCATCTAATATTACAGGGCTGCTATTAACAAGAGATGGTGGGAATAATGAG---------AACAAGACC---------------------------ACT---GAGATCTTCAGACCTGGAGGGGGAGATATGAGGGACAATTGGAGA---AGTGAACTATACAAATATAAAGTAGTAAAAATTGAACCA---TTAGGAGTAGCACCT---ACCAAGGCAAAGAGAAGAGTGGTGCAGAGAGAA---AAAAGAGCGGTG---GGAGCAATAGGA---GCTATG---TTCCTT---GGG---------TTCTTGGGA---GCAGCAGGAAGCACTATGGGCGCAGCGTCAATG---GCGCTGACGGTACAGGCCAGACAATTATTGTCTGGTATAGTGCAACAGCAGAACAATTTGCTGAGGGCTATTGAGGCGCAACAGCATCTGTTGCAACTCACAGTCTGGGGCATCAAGCAGCTCCAGGCAAGA---GTCCTGGCTGTGGAAAGATACCTAAGGGATCAACAGCTCCTAGGGATTTGGGGCTGCTCTGGAAAACTCATTTGCACCACTACTGTGCCTTGGAATACTAGTTGGAGT---------------------------AATAAATCTCTGG--------------------ATGACCTGGATGCAGTGGGAAAAAGAGATTAAC------AATTACACAGATTTAATATACACCTTAATTGAAGAATCGCAAAACCAACAAGAAAAGAATGAACAAGAATTATTGGAATTAGATAAATGGGACAGTTTGTGGAATTGGTTTAACATAACAAACTGGCTGTGGTATATAAAAATATTCATAATGATAGTAGGAGGCTTGATAGGTTTAAGAATAGTTTTTATTGTACTTTCTGTAGTGAATAGAGTTAGGCAGGGATACTCACCATTATCATTTCAGACCCGC---CTCCCAGTCCCGAGGGAA------CCCGACAGGCCCGGAGGAACAGAAGAAGAAGGTGGAGAGAAAGACAGAGACAGATCCGGACGTTTAGTGGATGGATTCTTAGCACTTATCTGGGACGATCTGCGGAGCCTGTTCCTCTTCAGCTACCAGCGCTTGAGAGACTTACTCTTGATTGTAGCGAGGATTGTGGAACTTCTGGGACGCAGG---------------GGGTGGGGAGTTCTCAAATATTGGTGG---AATCTCCTGCAGTATTGG---------------------------------------------------AGTCAGGAACTAAAGAATAGTGCTGTTAGCTTGCTCAATGCCACAGCTATCGCAGTAGCTGAGGGGACAGATAGGGTTATAGAAGTATTACAAAGA------------------ATTTATAGAGCCTTTCTCCACATCCCTAGAAGAATAAGACAGGGCTTCGAAAGGGCTTTGTTATAA

C.SC05.Trinidad.EU578360 ATGAGAGTGATGGAGACAAGGAGGAATTGGCAGCACTTG---------TGGAAATGGGGC------------------------ACGATGCTCTTTGGGATGTTGATGATC------------TGTAGTGCTGCA---------GAAGATATGTGGGTCACAGTCTATTATGGGGTACCTGTGTGGAAAGACACGACCACCACTCTATTTTGTGCATCAGATGCTAAAGCATATGATACAGAGGTACATAAT---GTCTGGGCCACACATGCCTGTGTACCCACAGACCCCAACCCACAAGAAGTAGTATTG---GAAAATGTGACAGAAAATTTTAACATGTGGAAAAATAACATGGTAGAACAGATGCATGAGGATATAATCAGTTTATGGGATCAAAGCCTAAAGCCATGTGTAAAATTAACCCCACTCTGTGTTACTTTAAATTGTATTGACATGAATGGTACTAGTGCTAATGTCACT---------------------------------------------------------------------------------------------AGTATAGAAAAAGTAGAAAAAGGAGAAATAAAAAACTGCTCTTTTAATATCACC---ACAGAAGTAAGAGAT------AAGATGCAAAAAACATATGCAACTTTTTATAGCCTTGATGTAGTACCAATAGATAAAGAT------------------------------AATATTAGC---------------------------TATAGGCTGGCAAGTTGTAATACCTCAGTCATTAGGCAAGCCTGTCCAAAGGTGTCCTTTGAACCAATTCCCATACATTATTGTGCCCCGGCTGGTTTTGCGCTTCTAAAATGT---AATGATAAAAAGTTCAATGGAACAGGACCATGTAAAAATGTCAGCACAGTACAATGTACACATGGAATTAGACCAGTAGTGTCAACTCAACTGTTGTTAAATGGCAGTCTAGCAGAAGAA---GAGGTAGTAATTAGATCTGAAAATTTCACAAACAATGCTAAAACCATAATAGTACAGCTGAATAGCTCTGTAGTAATTAATTGTACAAGACCCAACAATAATACAAGAAAAAGTATAACTATAGGA------------CCAGGGAGT---GTATTTTAT---ACAGGAGAAATAATAGGAGATATAAGACGAGCACATTGTACCCTT------AATAAAACAGCTTGGAATAACACTCTAAAACAGGTAGTTATAAAATTAAGA---GAACAATTTGGG------------AATAAAACA---ATAGTCTTTAACCAT---------TCCGCAGGAGGGGATCCAGAAATTGTATTGCACAGTTTTAATTGTGGAGGGGAATTTTTCTACTGTAATACAACACCACTGTTTAATAGTACTTGGAATATT---------AATGATACTTGGAATGATACG---------------------AATGGTACTACAGAGTCA------------AATGACACT------------------------------ATCACACTCCCATGCAGAATAAAACAAATTATAAACATGTGGCAGGAAGTAGGCAAAGCAATGTATGCCCCTCCCATTAGAGGAGAGATTAGATGTTTATCAAATATTACAGGATTACTATTAACAAGAGATGGTGGTAATACT------------AGCACGAAT------------------------GACACC---GAGACCTTCAGACCTGGAGGAGGAGATATGAGGGACAATTGGAGA---AGTGAATTATACAAATATAAAGTAGTAAAAATAGAACCA---TTAGGAATAGCACCC---ACCAAGGCAAAGAGAAGAGTGGTGCAGAGAGAA---AAAAGAGCAGTG---GGAACAATAGGA---GCTATG---TTCCTT---GGG---------TTCTTGGGA---GCAGCAGGAAGCACTATGGGCGCAGCATCAGTA---ACGCTGACGGTACAAGCCAGACTATTATTGTCTGGTATAGTGCAACAGCAGAACAATCTGCTGAGGGCTATTGAGGCGCAACAGCATCTGTTGCAACTCACGGTCTGGGGCATTAAACAGCTCCAAGCAAGA---GTCCTGGCTGTAGAAAGATACCTACAGGATCAACAGCTCCTGGGGATTTGGGGTTGCTCTGGAAAGCTCATCTGCACCACTACTGTGCCTTGGAATGATAGTTGGAGT---------------------------AATAAATCTTACAGTGATATTTGGGAT---AACATGACCTGGATGCAGTGGGATAGAGAAATTAAC------AATTACACAACCCTCATATACACCTTACTTGAAGGTGCGCAGAACCAGCAAGAAAAGAATGAACACGAATTATTAGAATTGGATAAGTGGGCAAGTTTGTGGAATTGGTTTAGCATAACACAGTGGCTGTGGTATATAAAAATATTCATAATGATAGTAGGTGGTCTGATAGGTTTAAGAATAGTTTTTGCTGTGCTTTCTATAGTAAATAGAGTTAGGCAGGGATACTCACCACTATCATTTCAGACCCAC---CTCCCAGCCAGGAGGGAA------CCCGACAGGCCCGAAGGAATCGAAGAAGAAGGTGGAGAGAAAGACAAAGACAGATCAGTTCGATTAGTGCATGGATTCTTAGCACTCATCTGGGACGACCTACGGAGCCTGTGCCTCTTCAGCTACCACCGCTTGAGAGACTTACTATTGATTGTAACGAGGGTTGTGGAGACTCTGGGACGCAGG---------------GGGTGGGAAATCCTCAAGTATTGGTGG---AGTCTCCTTCAGTATTGG---------------------------------------------------AGTCAGGAACTAAAGAAGAGTGCTGTTAGCTTGTTTGATGCCATAGCTATCACAGCAGCTGAGGGGACAGATAGGGTTCTAGAAGTAGTGCAAAGA------------------CTTTTTAGAGCTTTTCTCCACATACCTACAAGAATCAGACAGGGATTGGAAAGGGCTTTGTTATAA

C.SC05.Trinidad.EU578357 ATGAGAGTGATGGAGACAAGGAGGAATTGGCAGCACTTG---------TGGAAATGGGGC------------------------ACGATGCTCCTTGGGATGTTGATGATC------------TGTAGTGCTACA---------GAAAAGATGTGGGTCACAGTCTATTATGGGGTACCTGTGTGGAAAGACACGACCACCACTCTATTTTGTGCATCAGATGCTAAAGCATATGATGCAGAGGTACATAAT---GTCTGGGCCACACATGCCTGTGTACCCACAGACCCCAACCCACAAGAAGTAGTATTG---GAAAATGTGACAGAAAATTTTAACATGTGGAAAAATAACATGGTAGAACAGATGCATGAGGATATAATCAGTTTATGGGATCAAAGCCTAAAGCCATGTGTAAAATTAACCCCACTCTGTGTTACTTTAAATTGTATTGATATGAATGGTACTAGTACTAATGTCACT---------------------------------------------------------------------------------------------AGTATAGAAAAAATAGAAAAAGGAGAAATAAAAAACTGCTCTTTTAATATCACC---ACAGAAGTAAGAGAT------AAGATGCAAAAAACATATGCAACTTTTTATAGCCTTGATGTAGTACCAATAGATAAAGAT------------------------------AATATTAGC---------------------------TATAGGCTGGCAAGTTGTAATACCTCAGTCATTAGGCAAGCCTGTCCAAAGGTGTCCTTTGAACCAATTCCCATACATTATTGTGCCCCGGCTGGTTTTGCGCTTCTAAAATGT---AATGATAAAAAGTTCAATGGAACAGGACCATGTAAAAATGTCAGCACAGTACAATGTACACATGGAATTAAACCAGTAGTGTCAACTCAACTGTTGTTAAATGGCAGTCTAGCAGAAGAA---GAGGTAGTAATTAGATCTGAAAATTTCACAAACAATGCTAAAACCATAATAGTACAGCTGAATAGCTCTGTAGTAATTAATTGTACAAGACCCAACAATAATACAAGAAAAAGTATAACTATAGGA------------CCAGGGAGT---GTATTTTAT---ACAGGAGAAATAATAGGAGATATAAGACGAGCACATTGTACCCTT------AATAAAACAGCTTGGAATAACACTCTAAAACAGGTAGTTATAAAATTAAGA---GAACAATTTGGG------------AATAAAACA---ATAGTCTTTAACCAT---------TCCGCAGGAGGGGATCCAGAAATTGTATTGCACAGTTTTAATTGTGGAGGGGAATTTTTCTACTGTAATACAACACCACTGTTTAATAGTACCTGGAATATT---------AATGATACTTGGAATGATACG---------------------AATGGTACTACAGAGTCA------------AATGACACT------------------------------ATCACACTCCCATGCAGAATAAAACAAATTATAAACATGTGGCAGGAAGTAGGCAAAGCAATGTATGCCCCTCCCATTAGAGGAGAGATTAGATGTTTATCAAATATTACAGGATTACTATTAACAAGAGACGGTGGTAATAGT------------AGCACGAAT------------------------GACACC---GAGATCTTCAGACCTGGAGGAGGAGATATGAGGGACAATTGGAGA---AGTGAATTATATAAATATAAAGTAGTAAAAATTGAACCA---TTAGGAATAGCACCC---ACCAAGGCAAAGAGAAGAGTGGTGCAGAGAGAA---AAAAGAGCAGTG---GGAACAATAGGA---GCTATG---TTCCTT---GGG---------TTCTTGGGA---GCAGCAGGAAGCACTATGGGCGCAGCATCAGTA---ACGCTGACGGTACAAGCCAGACTATTATTGTCTGGTATAGTGCAACAGCAGAACAATCTGCTGAGGGCTATTGAGGCGCAACAGCATCTGTTGCAACTCACAGTCTGGGGCATTAAACAGCTCCAGGCAAGA---GTCCTGGCTGTGGAAAGATACCTACAGGATCAACAGCTCCTGGGGATTTGGGGTTGCTCTGGAAAACTCATCTGCACCACTACTGTGCCTTGGAATGATAGTTGGAGT---------------------------AATAAAACTTATAATGATATTTGGGAT---AACATGACCTGGATGCAGTGGAATAGAGAAATTAAC------AATTACACAACCCTCATATACACCTTACTTGAAGGTGCACAGAACCAGCAAGAAAAGAATGAACACGAATTATTAGAATTGGATAAGTGGGCAAGTTTGTGGAATTGGTTTGACATAACACAATGGCTGTGGTATATAAAAATATTCATAATGATAGTAGGTGGTCTGATAGGTTTAAGAATAGTTTTTGCTGTGCTTTCTATAGTGAATAGAGTTAGGCAGGGATACTCACCACTATCATTTCAGACCCAC---CTCCCAGCCAGGAGGGAA------CCCGACAGGCCCGAAGGAATCGAAGAAGAAGGTGGAGAGAAAGACAAAGACAGATCAGTTCGATTAGTGCATGGATTCTTAGCACTCATCTGGGACGACCTACGGAGCCTGTGCCTCTTCAGCTACCACCGCTTGAGAGACTTACTCTTGATAATAACGAGGATTGTGGAAACTCTGGGACGCAGG---------------GGGTGGGAAATCCTCAAGTATTGGTGG---AATCTCCTTCAGTATTGG---------------------------------------------------AGTCAGGAGCTAAAGAAGAGTGCTGTTAGCTTGTTTGATGCCATAGCTATCACAGCAGCTGAGGGGACAGATAGGGTTATAGAAGTAGTGCAAAGA------------------CTTTTTAGAGCTTTTCTCCACATACCTACAAGAATCAGGCAGGGATTGGAAAGGGCTTTGTTATAA

C.SC05.Trinidad.EU578358 ATGAGAGTGATGGAGACAAGGAGGAATTGGCAGCACTGG---------TGGAAATGGGGC------------------------ACGATGCTCTTTGGGATGTTGATGATC------------TGTAGTGCTGCA---------GAAGATATGTGGGTCACAGTCTATTATGGGGTACCTGTGTGGAAAGACGCGACCACCACTCTATTTTGTGCATCAGATGCTAAAGCATATGATACAGAGGTACATAAT---GTCTGGGCCACACATGCCTGTGTACCCACAGACCCCAACCCACAAGAAGTAGTATTG---GAAAATGTGACAGAAAATTTTAACATGTGGAAAAATAACATGGTAGAACAGATGCATGAGGATATAATCAGTTTATGGGATCAAAGCCTAAAGCCATGTGTAAAATTAACCCCACTCTGTGTTACTTTAAATTGTATTGATATGAATGGTACTAGTGCTAATGTCACT---------------------------------------------------------------------------------------------AGTATAGAAAAAGTAGAAAAAGGAGAAATAAAAAACTGCTCTTTTAATATCACC---ACAGCAATAAGAGAT------AAGGTGCAAAAAACATATGCAACTTTTTATAGCCTTGATGTAGTACCAATAGATAAAGAT------------------------------AATAGTAGC---------------------------TATAGGTTGGCAAGTTGTAATACCTCAGTCATTAGACAAGCCTGTCCAAAGGTGTCCTTTGAACCAATTCCCATACATTATTGTGCCCCGGCTGGTTTTGCGCTTCTAAAATGT---AATGATAAAAAGTTCAATGGAACAGGACCATGTAAAAATGTCAGCACAGTACAATGTACACATGGAATTAGACCAGTAGTGTCAACTCAACTGTTGTTAAATGGCAGTCTAGCAGAAGAA---GAGGTAGTAATTAGATCTGAAAACTTCACAAACAATGCTAAAACCATAATAGTACAGCTGAAGAACTCTGTAGTAATTAATTGTACAAGACCCAACAATAATACAAGAAAAAGTATACATATAGGA------------CCAGGGAGT---GTATTTTAT---ACAGGAGAAATAATAGGAAATATAAGACAAGCACATTGTAACCTT------AGTAAAACAGCTTGGAATAACACTTTAAAAAGGGTAGTTATAAAATTAAGA---GAACAATTTGGG------------AATAAAACA---ATAGTCTTTAACCAT---------TCCGCAGGAGGGGATCCAGAAATTGTATTGCACAGTTTTAATTGTGGAGGGGAATTTTTCTACTGTAATGCAACACAACTGTTTAATAGTACTTGGAATATT---------AATGGTACTTGGAATGATACA---------------------AATGGTACTACAGAGTCA------------AATGACACT------------------------------ATCTCACTTCCATGCAGAATAAAACAAATTATAAACATGTGGCAGGAAGTAGGCAAAGCGATGTATGCCCCTCCTATTAGAGGACAGATTAGATGTTCATCAAATATTACAGGATTACTATTAACAAGAGACGGTGGTAATACT------------AGCACGATT------------------------GACACC---GAGATCTTCAGACCTGGAGGAGGAGACATGAGGGACAATTGGAGA---AGTGAATTATATAAATATAAAGTAGTAAGAATTGAACCA---TTAGGAATAGCACCC---ACCAAGGCAAAGAGAAGAGTGGTGCAGAGAGAA---AAAAGAGCAGTG---GGAACAATAGGA---GCTATG---TTCCTT---GGG---------TTCTTGGGA---GCAGCAGGAAGCACTATGGGCGCAGCATCAGTA---ACGCTGACGGTACAGGCCAGACTATTATTGTCTGGTATAGTGCAACAGCAGAACAATCTGCTGAGGGCTATTGAGGCGCAACAGCATCTGTTGCAACTCACGGTCTGGGGCATTAAACAGCTCCAGGCAAGA---GTCCTGGCTGTGGAAAGATACCTACAGGATCAACAGCTCCTGGGGATTTGGGGTTGCTCTGGAAAGCTCATCTGCACCACTACTGTGCCTTGGAATGATAGTTGGAGT---------------------------AATAAATCTTACAGTGATATTTGGAAT---AACATGACCTGGATGCAGTGGGATAGAGAAATTAAC------AATTACACAACCCTCATATACACCTTACTTGAAGGTGCGCAGAACCAGCAAGAAAAGAATGAACACGAATTATTAGAATTGGATAAGTGGGCAAGTTTGTGGAATTGGTTTAGCATAACACAGTGGCTGTGGTATATAAAAATATTCATAATGATAGTAGGTGGTCTGATAGGTTTAAGAATAGTTTTTGCTGTGCTTTCTATAGTGAATAGAGTTAGGCAGGGATACTCACCACTGTCATTTCAGACCCAC---CTCCCAGCCAGGAGGGAA------CCCGACAGGCCCGAAGGAATCGAAGGAGAAGGTGGAGAGAAAGACAAAGACAGATCAGTTCGATTAGTGCATGGATTCTTAGCACTCATCTGGGACGACCTACGGAGCCTGTGCCTCTTCAGCTACCACCGCTTGAGAGACTTACTCTTGATTATAACGAGGATTGTGGAAACTCTGGGACGCAGG---------------GGGTGGGAAATCCTCAAGTATTGGTGG---AATCTCCTTCAGTATTGG---------------------------------------------------AGTCAGGAACTGAAGAATAGTGCTGTTAGCTTGTTTGATGCCATAGCTATCGCAACAGCTGAGGGGACAGATAGGGTTATTGAAGTAGTGCGAAGA------------------CTTTTTAGAGCTTTTCTCCACATACCTACAAGAATCAGACAGGGATTGGAAAGGGCTTTGTTATAA

C.SC05.Trinidad.EU578354 ATGAGAGTGATGGAGACAAGGAGGAATTGGCAGCACTGG---------TGGAAATGGGGC------------------------ACGATGCTCTTTGGGATGTTGATGATC------------TGTAGTGCTGCA---------GAAGATATGTGGGTCACAGTCTATTATGGGGTACCTGTGTGGAAAGACGCGACCACCACTCTATTTTGTGCATCAGATGCTAAAGCATATGATACAGAGGTACATAAT---GTCTGGGCCACACATGCCTGTGTACCCACAGACCCCAACCCACAAGAAGTAGTATTG---GAAAATGTGACAGAAAATTTTAACATGTGGAAAAATAACATGGTAGAACAGATGCATGAGGATATAATCAGTTTATGGGATCAAAGCCTAAAGCCATGTGTAAAATTAACCCCACTCTGTGTTACTTTAAATTGTATTGATATGAATGGTACTAGTACTAATGTCACT---------------------------------------------------------------------------------------------AGTATAGAAAGAGTAGAAAAAGGAGAAATAAAAAACTGCTCTTTTAATATCACC---ACAGCAATAAGAGAT------AAGATGCAAAAAACATATGCAACTTTTTATAGCCTTGATGTAGTACCAATAGATAAAGAT------------------------------AATATTAGC---------------------------TATAGGCTGGCAAGTTGTAATACCTCAGTCATTAGGCAAGCCTGTCCAAAGGTGTCCTTTGAACCAATTCCCATACATTATTGTGCCCCGGCTGGTTTTGCGCTTCTAAAATGT---AATGATAAAAAGTTCAATGGAACAGGACCATGTAAAAATGTCAGCACAGTACAATGTACACATGGAATTAAACCAGTAGTGTCAACTCAACTGTTGTTAAATGGCAGTCTAGCAGAAGAA---GAGGTAGTAATTAGATCTGAAAATTTCACAAACAATGCTAAAACCATAATAGTACAGCTGAATAGCTCTGTAATAATTAATTGTACAAGACCCAACAATAATACAAGAAAAAGTATAACTATAGGA------------CCAGGGAGT---GTATTTTAT---ACAGGAGAAATAATAGGAGATATAAGACGAGCACATTGTACCCTT------AATAAAACAGCTTGGAATAACACTCTAAAACAGGTAGTTATAAAATTAAGA---GAACAATTTGGG------------AATAAAACA---ATAGTCTTTAACCAT---------TCCGCAGGAGGGGATCCAGAAATTGTATTGCACAGTTTTAATTGTGGAGGGGAATTTTTCTACTGTAATACAACACCACTGTTTAATAGTACCTGGAATATT---------AATGATACTTGGAATGATACG---------------------AATGGTACTACAGAGTCA------------AATGACACT------------------------------ATCACACTCCCATGCAGAATAAAACAAATTATAAACATGTGGCAGGAAGTAGGCAAAGCAATGTATGCCCCTCCCATTAGAGGACAGATTAGATGTTTATCAAATATTACAGGATTACTATTAACAAGAGATGGTGGTAATACT------------AGCACGAAT------------------------GACACC---GAGATCTTCAGACCTGGAGGAGGAGATATGAGGGACAATTGGAGA---AGTGAATTATATAAATATAAAGTAGTAAGAATTGAACCA---TTAGGAATAGCACCC---ACCAAGGCAAAGAGAAGAGTGGTGCAGAGAGAA---AAAAGAGCAGTG---GGAACAATAGGA---GCTATG---TTCCTT---GGG---------TTCTTGGGA---GCAGCAGGAAGCACTATGGGCGCAGCATCAGTA---ACGCTGACGGTACAGGCCAGACTATTATTGTCTGGTATAGTGCAACAGCAGAACAATCTGCTGAGGGCTATTGAGGCGCAACAACATCTGTTGCAACTCACGGTCTGGGGCATTAAACAGCTCCAAGCAAGA---GTCCTGGCTGTAGAAAGATACCTACAGGATCAACAGCTCCTGGGGATTTGGGGTTGCTCTGGAAAACTCATCTGCACCACTACTGTGCCTTGGAATGCTAGTTGGAGT---------------------------AATAAATCTTATAGTGATATTTGGGAT---AACATGACCTGGATGCAGTGGGATAGAGAAATTAAC------AATTACACAACCCTCATATACACCTTACTTGAAGGTGCGCAGAACCAGCAAGAAAAGAATGAACACGAATTATTAGAATTGGATAAGTGGGCAAGTTTGTGGAATTGGTTTAGCATAACACAGTGGCTGTGGTATATAAAAATATTCATAATGATAGTAGGTGGTCTGATAGGTTTAAGAATAATTTTTGCTGTGCTTTCTATAGTGAATAGAGTTAGGCAGGGATACTCACCACTGTCATTTCAGACCCAC---CTCCCAGCCAGGAGGGAA------CCCGACAGGCCCGAAGGAATCGAAGGAGAAGGTGGAGAGAAAGACAAAGACAGATCAGTTCGATTAGTGCATGGATTCTTAGCACTCATCTGGGACGACCTACGGAGCCTGTGCCTCTTCAGCTACCACCGCTTGAGAGACTTACTCTTGATAATAACGAGGATTGTGGAAACTCTGGGACGCAGG---------------GGGTGGGAAATCCTCAAGTATTGGTGG---AATCTCCTTCAGTATTGG---------------------------------------------------AGTCAGGAACTAAAGAAGAGTGCTGTTAGCTTGTTTGATGCCATAGCTATCACAGCAGCTGAGGGGACAGATAGGGTTATAGAAGTAGTGCAAAGA------------------CTTTTTAGAGCTTTTCTCCACATACCTACAAGAATCAGACAGGGATTAGAAAGGGCTTTGTTATAA

C.SC05.Trinidad.EU578355 ATGAGAGTGATGGAGACAAGGAGGAATTGGCAGCACTTG---------TGGAAATGGGGC------------------------ACGATGCTCCTTGGGATGTTGATGATC------------TGTAGTGCTACA---------GAAAAGATGTGGGTCACAGTCTATTATGGGGTACCAGTGTGGAAAGAAGCGACCACCACTCTATTTTGTGCATCAGATGCTAAAGCATATGATACAGAGGTACATAAT---GTCTGGGCCACACATGCCTGTGTACCCACAGACCCCAACCCACAAGAAGTAGTATTA---GAAAATGTGACAGAAAATTTTAACATGTGGAAAAATAACATGGTAGAACAGATGCATGAGGATATAATCAGTTTATGGGATCAAAGCCTAAAGCCATGTGTAAAATTAACCCCACTCTGTGTTACTTTAAATTGTATTGATATGAATGGTACTAGTACTAATGTCACT---------------------------------------------------------------------------------------------AGTATAGAAAAAGTAGAAAAAGGAGAAATAAAAAACTGCTCTTTTAATATCACC---ACAGAAGTAAGAGAT------AAGATGCAAAAAACATATGCAACTTTTTATAGCCTTGATGTAGTACCAATAGATAAAGAT------------------------------AATATTAGC---------------------------TATAGGCTGGCAAGTTGTAATACCTCAGTCATTAGGCAAGCCTGTCCAAAGGTGTCCTTTGAACCAATTCCCATACATTATTGTGCCCCGGCTGGTTTTGCGCTTCTAAAATGT---AATGATAAAAAGTTCAATGGAACAGGACCATGTAAAAATGTCAGCACAGTACAATGTACACATGGAATTAAACCAGTAGTGTCAACTCAATTGTTGTTAAATGGCAGTCTAGCAGAGGAA---GAGGTAGTGATTAGATCTGAAAATTTTACAAACAATGCTAAAACCATAATAGTACAGCTGAATGAATCTGTAGTAATTAATTGTACAAGACCCAACAATAATACAAGAAAAAGTATAACTATAGGA------------CCAGGGAGT---GTATTTTAT---ACAGGAGAAATAATAGGAAATATAAGACAAGCACATTGTAACCTT------AGTAAAACAGCTTGGAATAACACTTTAAAAAGGGTAGTTATAAAATTAAGA---GAACAATTTGGG------------AATAAAACA---ATAGTCTTTAACCAT---------TCCGCAGGAGGGGATCCAGAAATTGTATTGCACAGTTTTAATTGTGGAGGGGAATTTTTCTACTGTAATGCAACACAACTGTTTAATAGTACTTGGAATATT---------AATGGTACTTGGAATGATACA---------------------AATGGTACTACAGAGTCA------------AATGACACT------------------------------ATCTCACTTCCATGCAGAATAAAACAAATTATAAACATGTGGCAGGAAGTAGGCAAAGCAATGTATGCCCCTCCCATTAGAGGACAGATTAGATGTTCATCAAATATTACAGGATTACTATTAACAAGAGACGGTGGTAATACT------------AGCACGATT------------------------GACACC---GAGATCTTCAGACCTGGAGGAGGAGATATGAGGGACAATTGGAGG---AGTGAATTATATAAATATAAAGTAGTAAGAATTGAACCA---TTAGGAATAGCACCC---ACCAAGGCAAAGAGAAGAGTGGTGCAGAGAGAA---AAAAGAGCAGTG---GGAACAATAGGA---GCTATG---TTCCTT---GGG---------TTCTTGGGA---GCAGCAGGAAGCACTATGGGCGCAGCATCAGTA---ACGCTGACGGTACAGGCCAGGCTATTATTGTCTGGTATAGTGCAACAGCAGAACAATCTGCTAAGGGCTATTGAGGCGCAACAGCATCTGTTGCAACTCACAGTCTGGGGCATTAAACAGCTCCAGGCGAGA---GTCCTGGCTGTGGAAAGATACCTACAGGATCAACAGCTCCTGGGGATTTGGGGTTGCTCTGGAAAACTCATCTGCACCACTACTGTGCCTTGGAATGATAGTTGGAGT---------------------------AATAAATCTTACAGTGATATTTGGAAT---AACATGACCTGGATGCAGTGGGATAGAGAAATTAAC------AATTACACAACCCTCATATACACCTTACTTGAAGGTGCGCAGAACCAGCAAGAAAAGAATGAACACGAATTATTAGAATTGGATAAGTGGGCAAGTTTGTGGAATTGGTTTAGCATAACACAGTGGCTGTGGTATATAAAAATATTCATAATGATAGTAGGTGGTCTGATAGGTTTAAGAATAGTTTTTGCTGTGCTTTCTATAGTGAATAGAGTTAGGCAGGGATACTCACCACTATCATTTCAGACCCAC---CTCCCAGCCAGGAGGGAA------CCCGACAGGCCCGAAGGAATCGAAGGAGAAGGTGGAGAGAAAGACAAAGACAGATCAGTTCGATTAGTGCATGGATTCTTAGCACTCATCTGGGACGACCTACGGAGCCTGTGCCTCTTCAGCTACCACCGCTTGAGAGACTTACTCTTGATAATAACGAGGATTGTGGAAACTCTGGGACGCAGG---------------GGGTGGGAAATCCTCAAGTATTGGTGG---AATCTCCTTCAGTATTGG---------------------------------------------------AGTCAGGAACTGAAGAATAGTGCTGTTAGCTTGTTTGATGCCATAGCTATCACAGCAGCAGAGGGGACAGATAGGGTTATAGAAGTAGTGCAAAGA------------------CTTTTTAGAGCTTTTCTCCACATACCTACAAGAATCAGACAGGGATTGGAAAGGGCTTTGTTATAA

C.SC05.Trinidad.EU578359 ATGAGAGTGATGGAGACAAGGAGGAATTGGCAGCACTTG---------TGGAAATGGGGC------------------------ATGATGCTCTTTGGGATGTTGATGATC------------TGTAGTGCTACA---------GAAGATATGTGGGTCACAGTCTATTATGGGGTACCTGTGTGGAAAGACGCGACCACCACTCTATTTTGTGCATCAGATGCTAAAGCATATGATACAGAGGTACATAAT---GTCTGGGCCACACATGCCTGTGTACCCACAGACCCCAACCCACAAGAAGTAGTATTG---GAAAATGTGACAGAAAATTTTAACATGTGGAAAAATAACATGGTAGAACAGATGCATGAGGATATAATCAGTTTATGGGATCAAAGCCTAAAGCCATGTGTAAAATTAACCCCACTCTGTGTTACTTTAAATTGTATTGATATGAATGGTACTAGTACTAATGTCACT---------------------------------------------------------------------------------------------AGTATAGAAAAAGTAGAAAAAGGAGAAATAAAAAACTGTTCTTTTAATATCACC---ACAGAAGTAAGAGAT------AAGATGCAAAAAACATATGCAACTTTTTATAGCCTTGATGTAGTACCAATAGATAAAGAT------------------------------AATATTAGC---------------------------TATAGGCTGGCAAGTTGTAATACCTCAGTCATTAGGCAAGCCTGTCCAAAGGTGTCCTTTGAACCAATTCCCATACATTATTGTGCCCCGGCTGGTTTTGCGCTTCTAAAATGT---AATGATAAAAAGTTCAATGGAACAGGACCATGTAAAAATGTCAGCACAGTACAATGTACACATGGAATTAGACCAGTAGTGTCAACTCAACTGTTGTTAAATGGCAGTCTAGCAGAAGAA---GAGGTAGTAATTAGATCTGAAAATTTCACAAACAATGCTAAAACCATAATAGTACAGCTGAATAGCTCTGTAGTAATTAATTGTACAAGACCCAACAATAATACAAGAAAAAGTATAACTATAGGA------------CCAGGGAGT---GTATTTTAT---ACAGGAGAAATAATAGGAGATATAAGACGAGCACATTGTACCCTT------AATAAAACAGCTTGGAATAACACTCTAAAACAGGTAGTTATAAAATTAAGA---GAACAATTTGGG------------AATAAAACA---ATAGTCTTTAACCAT---------TCCGCAGGAGGGGATCCAGAAATTGTATTGCACAGTTTTAATTGTGGAGGGGAATTTTTCTACTGTAATACAACACCACTGTTTAATAGTACCTGGAATATT---------AATGATACTTGGAATGATGCG---------------------AATGGTACTACAGAGTCA------------AATGACACT------------------------------ATCACACTCCCATGCAGAATAAAACAAATTATAAACATGTGGCAGGAAGTAGGCAAAGCAATGTATGCCCCTCCCATTAGAGGACAGATTAGATGTTTATCAAATATTACAGGATTACTATTAACAAGAGATGGTGGTAATACT------------AGCACGAAT------------------------AACACC---GAGATCTTCAGACCTGGAGGAGGAGATATGAGGGACAATTGGAGA---AGTGAATTATATAAATATAAAGTAGTAAGAATTGAACCA---TTAGGAATAGCACCC---ACCAAGGCAAAGAGAAGAGTGGTGCAGAGAGAA---AAAAGAGCAGTG---GGAACAATAGGA---GCTATG---TTCCTT---GGG---------TTCTTGGGA---GCAGCAGGAAGCACTATGGGCGCAGCATCAGTA---ACGCTGACGGTACAGGCCAGACTATTATTGTCTGGTATAGTGCAACAGCAGAACAATCTGCTGAGGGCTATTGAGGCGCAACAACATCTGTTGCAACTCACGGTCTGGGGCATTAAACAGCTCCAAGCAAGA---GTCCTGGCTGTAGAGAGATACCTACAGGATCAACAGCTCCTGGGGATTTGGGGTTGCTCTGGAAAGCTCATCTGCACCACTACTGTGCCTTGGAATGATAGTTGGAGT---------------------------AATAAATCTTACAGTGATATTTGGAAT---AACATGACCTGGATGCAGTGGGATAGAGAAATTAAC------AATTACACAACCCTCATATACACCTTACTTGAAGGTGCGCAGAACCAGCAAGAAAAGAATGAACACGAATTATTAGAATTGGATAAGTGGGCAAGTTTGTGGAATTGGTTTAGCATAACACAGTGGCTGTGGTATATAAAAATATTCATAATGATAGTAGGTGGTCTGATAGGTTTAAGAATAGTTTTTGCTGTGCTTTCTATAGTGAATAGAGTTAGGCAGGGATACTCACCACTATCATTTCAGACCCAC---CTCCCAGCCAGGAGGGAA------CCCGACAGGCCCGAAGGAATCGAAGGAGAAGGTGGAGAGAAAGACAAAGACAGATCAGTTCGATTAGTGCATGGATTCTTAGCACTCATCTGGGACGACCTACGGAGCCTGTGCCTCTTCAGCTACCACCGCTTGAGAGACTTACTCTTGATAATAACGAGGATTGTGGAAACTCTGGGACGCAGG---------------GGGTGGGAAATCCTCAAGTATTGGTGG---AATCTCCTTCAGTATTGG---------------------------------------------------AGTCAGGAGCTAAAGAATAGTGCTGTTAGCTTGTTTGATGCCATAGCTATCGCAGCAGCTGAGGGGACAGATAGGGTTATAGAAGTAGTGCAAAGA------------------CTTTTTAGAGCTTTTCTCCACATACCTACAAGAATCAGACAGGGATTGGAAAGGGCTTTGTTATAA

C.SC05.Trinidad.EU578363 ATGAGAGTGATGGAGACAAGGAGGAATTGGCAGCACTTG---------TGGAAATGGGGC------------------------ACGATGCTCCTTGGGATGTTGATGATC------------TGTAGTGCTGCA---------GATGATATGTGGGTCACAGTCTATTATGGGGTACCTGTGTGGAAAGACGCGACCACCACTCTATTTTGTGCATCAGATGCTAAAGCATATGATACAGAGGTACATAAT---GTCTGGGCCACACATGCCTGTGTACCCACAGACCCCAATCCACAAGAAGTAGTATTG---GAAAATGTGACAGAAAATTTTAACATGTGGAAAAATAACATGGTAGAACAGATGCATGAGGATATAATCAGTTTATGGGATCAAAGCCTAAAGCCATGTGTAAAATTAACCCCACTCTGTGTTACTTTAGATTGTATTGATATGAATGGTACTAGTGCTAATGTCACT---------------------------------------------------------------------------------------------AGTATAGAAAAAGTAGAAAAAGGAGAAATAAAAAACTGCTCTTTTAATATCACC---ACAGCAATAAGAGAT------AAGGTGCAAAAAACATATGCAACTTTTTATAGCCTTGATGTAGTACCAATAGATAAGGAT------------------------------AATAGTAGC---------------------------TATAGGTTGGCAAGTTGTAATACCTCAGTCATTAGGCAAGCCTGTCCAAAGGTGTCCTTTGAACCAATTCCCATACATTATTGTGCCCCGGCTGGTTTTGCGCTTCTAAAATGT---AATGATAAAAAGTTCAATGGAACAGGACCATGTAAAAATGTCAGCACAGTACAATGTACACATGGAATTAGACCAGTAGTGTCAACTCAACTGTTGTTAAATGGCAGTCTAGCAGAAGAA---GAGGTAGTAATTAGATCTGAAAATTTCACAAACAATGCTAAAACCATAATAGTACAGCTGAATAGCTCTGTAGTAATTAATTGTACAAGACCCAACAATAATACAAGAAAAGGTATACATATAGGA------------CCAGGGAGG---GTATTTTAT---ACAGGAGAAATAATAGGAAATATAAGACGAGCACATTGTAACCTT------AATAAAACAGATTGGAATAACACTTTAAAACAGGTAGTTATAAAGTTAAGA---GAACAATTTGGA------------AATAAAACA---ATAGTATTTAATCAA---------TCTGCAGGAGGGGATCTAGAAATTGTATTGCACAGTTTTAATTGTGGAGGGGAATTTTTCTACTGTAATGCAACACAACTGTTTAATAGTACTTGGAATATT---------AATGATATTTGGAATGATACG---------------------AATGGTACTACAGAGTTA------------AATAACACT------------------------------ATCATACTCCCATGCAGAATAAAACAAATTATAAACATGTGGCAGGAAGTAGGCAAAGCAATGTATGCCCCTCCCATTAGAGGACAGATTAGATGTTCATCAAATATTACAGGATTACTATTAACAAGAGATGGTGGTAATACT------------AGCACGAAT------------------------GACACC---GAGATCTTCAGACCTGGAGGAGGAGATATGAGGGACAATTGGAGA---AGTGAATTATATAAATATAAAGTAGTAAGAATTGAACCA---TTAGGAATAGCACCC---ACCAAGGCAAAGAGAAGAGTGGTGCAGAGAGAA---AAAAGAGCAGTG---GGAACAATAGGA---GCTATG---TTCCTT---GGG---------TTCTTGGGA---GCAGCAGGAAGCACTATGGGCGCAGCATCAGTA---ACGCTGACGGTACAAGCCAGACTATTATTGTCTGGTATAGTGCAACAGCAGAACAATCTGCTGAGGGCTATTGAGGCGCAACAGCATCTGTTGCAACTCACAGTCTGGGGCATTAAACAGCTCCAGGCAAGA---GTCCTGGCTGTGGAAAGATACCTACAGGATCAACAGCTCCTGGGGATTTGGGGTTGCTCTGGAAAACTCATCTGCACCACTACTGTGCCTTGGAATGCTAGTTGGAGT---------------------------AATAAATCTTACAGTGATATTTGGGAT---AACATGACCTGGATGCAGTGGGATAGAGAAATTAAC------AATTACACAACCCTCATATACACCTTACTTGAAGGTGCGCAGAACCAGCAAGAAAAGAATGAACACGAATTATTAGAATTGGATAAGTGGGCAAGTTTGTGGAATTGGTTTAGCATAACACAGTGGCTGTGGTATATAAAAATATTCATAATGATAGTAGGTGGTCTGATAGGTTTAAGAATAGTTTTTGCTGTGCTTTCTATAGTAAATAGAGTTAGGCAGGGATACTCACCACTATCATTTCAGACCCAC---CTCCCAGCCAGGAGGGAA------CCCGACAGGCCCGAAGGAATCGAAGGAGAAGGTGGAGAGAAAGACAAAGACAGATCAGTTCGATTAGTGCATGGATTCTTAGCACTCATCTGGGACGACCTACGGAGCCTGTGCCTCTTCAGCTACCACCGCTTGAGAGACTTACTCTTGATTGTAACGAGGATTGTGGAAACTCTGGGACGCAGG---------------GGGTGGGAAATCCTCAAGTATTGGTGG---AATCTCCTTCAGTATTGG---------------------------------------------------AGTCAGGAACTAAAGAATAGTGCTGTTAGCTTGTTTGATGCCATAGCTATCGCAGCAGCTGAGGGGACAGATAGGGTTATAGAAGTAGTGCGAAGA------------------CTTTTTAGAGCTTTTCTCCACATACCTACAAGAATCAGACAGGGATTGGAAAGGGCTTTGTTATAA

C.SC05.Trinidad.EU578362 ATGAGAGTGATGGAGACAAGGAGGAATTGGCAGCACTTG---------TGGAAATGGGGC------------------------ACGATGCTCTTTGGGATGTTGATGATC------------TGTAGTGCTACA---------GAAAAGATGTGGGTCACAGTCTATTATGGGGTACCTGTGTGGAAAGACGCGACCACCACTCTATTTTGTGCATCAGATGCTAAAGCATATGATACAGAGGTACATAAT---GTCTGGGCCACACATGCCTGTGTACCCACAGACCCCAACCCACAAGAAGTAGTATTG---GAAAATGTGACAGAAAATTTTAACATGTGGAAAAATAACATGGTAGAACAGATGCATGAGGATATAATCAGTTTATGGGATCAAAGCCTAAAGCCATGTGTAAAATTAACCCCACTCTGTGTTACTTTAAATTGTATTGATATAAATGGTACTAGTACTAATGTCACT---------------------------------------------------------------------------------------------AGTATAGAAAAAGTAGAAAAAGGAGAAATAAAAAACTGCTCTTTTAATATCACC---ACAGAAGTAAGAGAT------AAGATGCAAAAAACATATGCAACTTTTTATAGCCTTGATGTAGTACCAATAGATAAAGAT------------------------------AATATTAGC---------------------------TATAGGCTGGCAAGTTGTAATACCTCAGTCATTAGGCAAGCCTGTCCAAAGGTGTCCTTTGAACCAATTCCCATACATTATTGTGCCCCGGCTGGTTTTGCGCTTCTAAAATGT---AATGATAAAAAGTTCAATGGAACAGGACCATGTAAAAATGTCAGCACAGTACAATGTACACATGGAATTAGACCAGTAGTGTCAACTCAATTGTTGTTAAATGGCAGTCTAGCAGAAGAA---GAGGTAGTAATTAGATCTGAAAATTTCACAAACAATGCTAAAACCATAATAGTACAGCTGAATAGCTCTGTAGTAATTAATTGTACAAGACCCAACAATAATACAAGAAAAAGTATAACTATAGGA------------CCAGGGAGT---GTATTTTAT---ACAGGAAAAATAATAGGAGATATAAGACGAGCACATTGTACCCTT------AATAAAACAGCTTGGAATAACACTCTAAAACAGGTAGTTATAAAATTAAGA---GAACAATTTGGG------------AATAAAACA---ATAGTCTTTAACCAT---------TCCGCAGGAGGGGATCCAGAAATTGTATTGCACAGTTTTAATTGTGGAGGGGAATTTTTCTACTGTAATACAACACCACTGTTTAATAGTACCTGGAATATT---------AATGATACTTGGAATGATACG---------------------AATGGTACTACAGAGTCA------------AATGACACT------------------------------ATCACACTCCCATGCAGAATAAAACAAATTATAAACATGTGGCAGGAAGTAGGCAAAGCAATGTATGCCCCTCCCATTAGAGGAGAGATTAGATGTTTATCAAATATTACAGGATTACTATTAACAAGAGACGGTGGTAATACT------------AGCACGAAT------------------------GACACC---GAGATCTTCAGACCTGGAGGAGGAGATATGAGGGACAATTGGAGA---AGTGAATTATATAAATATAAAGTAGTAAGAATTGAACCA---TTAGGAATAGCACCC---ACCAAGGCAAAGAGAAGAGTGGTGCAGAGAGAA---AAAAGAGCAGTG---GGAACAATAGGA---GCTATG---TTCCTT---GGG---------TTCTTAGGA---GCAGCAGGAAGCACTATGGGCGCAGCATCAGTA---ACGCTGACGGTACAGGCCAGACTATTATTGTCTGGTATAGTGCAACAGCAGAACAATCTGCTGAGGGCTATTGAGGCGCAACAGCATCTGTTGCAACTCACGGTCTGGGGCATTAAACAGCTCCAAGCAAGA---GTCCTGGCTGTAGAAAGATACCTACAGGATCAACAGCTCCTGGGGATTTGGGGTTGCTCTGGAAAACTCATCTGCACCACTACTGTGCCTTGGAATGCTAGTTGGAGT---------------------------AATAAAACTTATAATGATATTTGGGAT---AACATGACCTGGATGCAGTGGGATAGAGAAATTAAC------AATTACACAACCCTCATATACACCTTACTTGAAGGTGCGCAGAACCAGCAAGAAAAGAATGAACACGAATTATTAGAATTGGATAAATGGGCAAGTTTGTGGAATTGGTTTAACATAACACAGTGGCTGTGGTATATAAAAATATTCATAATGATAGTAGGTGGTCTGATAGGTGTAAGAATAGTTTTTGCTGTGCTTTCTATAGTGAATAGAGTTAGGCAGGGATACTCACCACTATCATTTCAGACCCAC---CTCCCAGCCAGGAGGGAA------CCCGACAGGCCCGAAGGAATCGAAGGAGAAGGTGGAGAGAAAGACAAAGACAGATCAGTTCGATTAGTGCATGGATTCTTAGCACTCATCTGGGACGACCTACGGAGCCTGTGCCTCTTCAGCTACCACCGCTTGAGAGACTTACTCTTGATAATAACGAGGATTGTGGAAACTCTGGGACGCAGG---------------GGGTGGGAAATCCTCAAGTATTGGTGG---AATCTCCTTCAGTATTGG---------------------------------------------------AGTCAGGAACTAAAGAATAGTGCTGTTAGCTTGTTTGATGCCATAGCTATCGCAGCAGCTGAGGGGACAGATAGGATTATAGAAGTAGTGCAAAGA------------------CTTTTTAGAGCTTTTCTCCACATACCTACAAGAATCAGACAGGGATTGGAAAGGGCTTTGTTATAA

C.SC05.Trinidad.EU578361 ATGAGAGTGATGGAGACAAGGAGGAATTGGCAGCACTGG---------TGGAAATGGGGC------------------------ACGATGCTCTTTGGGATGTTGATGATC------------TGTAGTGCTGCA---------GAAGATATGTGGGTCACAGTCTATTATGGGGTACCTGTGTGGAAAGACGCGACCACCACTCTATTTTGTGCATCAGATGCTAAAGCATATGATACAGAGGTACATAAT---GTCTGGGCCACACATGCCTGTGTACCCACAGACCCCAACCCACAAGAAGTAGTATTG---GAAAATGTGACAGAAAATTTTAACATGTGGAAAAATAACATGGTAGAACAGATGCATGAGGATATAATCAGTTTATGGGATCAAAGCCTAAAGCCATGTGTAAAATTAACCCCACTCTGTGTTACTTTAAATTGTATTGATATGAATGGTACTAGTACTAATGTCACT---------------------------------------------------------------------------------------------AGTATAGAAAGAGTAGAAAAAGGAGAAATAAAAAACTGCTCTTTTAATATCACC---ACAGAAGTAAGAGAT------AAGATGCAAAAAACATATGCAACTTTTTATAGCCTTGATGTAGTACCAATAGATAAAGAT------------------------------AATATTAGC---------------------------TATAGGCTGGCAAGTTGTAATACCTCAGTCATTAGACAAGCCTGTCCAAAGGTGTCCTTTGAACCAATTCCCATACATTATTGTGCCCCGGCTGGTTTTGCGCTTCTAAAATGT---AATGATAAAAAGTTCAATGGAACAGGACCATGTAAAAATGTCAGCACAGTACAATGTACACATGGAATTAGACCAGTAGTGTCAACTCAACTGTTGTTAAATGGCAGTCTAGCAGAAGAA---GAGGTAGTAATTAGATCTGAAAATTTCACAAACAATGCTAAAACCATAATAGTACAGCTGAATAGCTCTGTAGTAATTAATTGTACAAGACCCAACAATAATACAAGAAAAAGTATAACTATAGGA------------CCAGGGAGT---GTATTTTAT---ACAGGAGAAATAATAGGAGATATAAGACGAGCACATTGTACCCTT------AATAAAACAGCTTGGAATAACACTCTAAAACAGGTAGTTATAAAATTAAGA---GAACAATTTGGG------------AATAAAACA---ATAGTCTTTAACCAT---------TCCGCAGGAGGGGATCCAGAAATTGTATTGCACAGTTTTAATTGTGGAGGGGAATTTTTCTACTGTAATACAACACCACTGTTTAATAGTACCTGGAATATT---------AATGATACTTGGAATGATACG---------------------AATGGTACTACAGAGTCA------------AATGACACT------------------------------ATCACACTCCCATGCAGAATAAAACAAATTATAAACATGTGGCAGGAAGTAGGCAAAGCAATGTATGCCCCTCCCATTAGAGGACAGATTAGATGTTTATCAAATATTACAGGATTACTATTAACAAGAGATGGTGGTAATAGT------------AGCATGAAT------------------------GACACC---GAGATCTTCAGACCTGGAGGAGGAGATATGAGGGACAATTGGAGA---AGTGAATTATATAAATATAAAGTAGTAAGAATTGAACCA---TTAGGAATAGCACCC---ACCAAGGCAAAGAGAAGAGTGGTGCAGAGAGAA---AAAAGAGCAGTG---GGAACAATAGGA---GCTATG---TTCCTT---GGG---------TTCTTGGGA---GCAGCAGGAAGCACTATGGGCGCAGCATCAGTA---ACGCTGACGGTACAGGCCAGACTATTATTGTCTGGTATAGTGCAACAGCAGAACAATCTGCTGAGGGCTATTGAGGCGCAACAGCATCTGTTGCAACTCACAGTCTGGGGCATTAAACAGCTCCAGGCAAGA---GTCCTGGCTGTGGAAAGATACCTACAGGATCAACAGCTTCTGGGGATTTGGGGTTGCTCTGGAAAACTCATCTGCACCACTACTGTGCCTTGGAATGATAGTTGGAGT---------------------------AATAAATCTTACAGTGATATTTGGAAT---AACATGACCTGGATGCAGTGGGATAGAGAAATTAAC------AATTACACAACCCTCATATACACCTTACTTGAAGGTGCGCAGAACCAGCAAGAAAAGAATGAACACGAATTATTAGAATTGGATAAGTGGGCAAGTTTGTGGAATTGGTTTAGCATAACACAGTGGCTGTGGTATATAAAAATATTCATAATGATAGTAGGTGGTCTGATAGGTTTAAGAATAGTTTTTGCTGTGCTTTCTATAGTGAATAGAGTTAGGCAGGGATACTCACCACTATCATTTCAGACCCAC---CTCCCAGCCAGGAGGGAA------CCCGACAGGCCCGAAGGAATCGAAGGAGAAGGTGGAGAGAAAGACAAAGACAGATCAGTTCGATTAGTGCATGGATTCTTAGCACTCATCTGGGACGACCTACGGAGCCTGTGCCTCTTCAGCTACCACCGCTTGAGAGACTTACTCTTGATAATAACGAGGATTGTGGAAACTCTGGGACGCAGG---------------GGGTGGGAAATCCTCAAGTATTGGTGG---AATCTCCTTCAGTATTGG---------------------------------------------------AGTCAGGAACTGAAGAATAGTGCTGTTAGCTTGTTTGATGCCATAGCTATCGCAGCAGCTGAGGGGACAGATAGGGTTATAGAAGTAGTGCGCAGA------------------CTTGTTAGAGCTTTTCTCCACATACCTACAAGAATCAGACAGGGATTGGAACGGGCTTTGTTATAA

C.SC05.Trinidad.EU578364 ATGAGAGTGATGGAGACAAGGAGGAATTGGCAGCACTTG---------TGGAAATGGGGC------------------------ACGATGCTCTTTGGGATGTTGATGATC------------TGTAGTGCTACA---------GAAAAGATGTGGGTCACAGTCTATTATGGGGTACCAGTGTGGAAAGACGCGACCACCACTCTATTTTGTGCATCAGATGCTAAAGCATATGATACAGAGGTACATAAT---GTCTGGGCCACACATGCCTGTGTACCCACAGACCCCAACCCACAAGAAGTAGTATTG---GAAAATGTGACAGAAAATTTTAACATGTGGAAAAATAACATGGTAGAACAGATGCATGAGGATATAATCAGTTTATGGGATCAAAGCCTAAAGCCATGTGTAAAATTAACCCCACTCTGTGTTACTTTAAATTGTATTGATATGAATGGTACTAGTACTAATGTCACT---------------------------------------------------------------------------------------------AGTATAGAAAAAGTAGAAAAAGGAGAAATAAAAAACTGCTCTTTTAATATCACC---ACAGAAGTAAGAGAT------AAGATGCAAAAAACATATGCAACTTTTTATAGCCTTGATGTAGTACCAATAGATAAAGAT------------------------------AATATTAGC---------------------------TATAGGCTGGCAAGTTGTAATACCTCAGTCATTAGGCAAGCCTGTCCAAAGGTGTCCTTTGAACCAATTCCCATACATTATTGTGCCCCGGCTGGTTTTGCGCTTCTAAAATGT---AATGATAAAAAGTTCAATGGAACAGGACCATGTAAAAATGTCAGCACAGTACAATGTACACATGGAATTAGACCAGTAGTGTCAACTCAACTGTTGTTAAATGGCAGTCTAGCAGAAGAA---GAGGTAGTAATTAGATCTGAAAATTTCACAAACAATGCTAAAACCATAATAGTACAGCTGAATAGCTCTGTAGTAATTAATTGTACAAGACCCAACAATAATACAAGAAAAAGTATAACTATAGGA------------CCAGGGAGT---GTATTTTAT---ACAGGAGAAATAATAGGAGATATAAGACGAGCACATTGTACCCTT------AATAAAACAGCTTGGAATAACACTCTAAAACAGGTAGTTATAAAATTAAGA---GAACAATTTGGG------------AATAAAACA---ATAGTCTTTAACCAT---------TCCGCAGGAGGGGATCCAGAAATTGTATTGCACAGTTTTAATTGTGGAGGGGAATTTTTCTACTGTAATACAACACCACTGTTTAATAGTACTTGGAATATT---------AATGATACTTGGAATGATACG---------------------AATGGTACTACAGAGTCA------------AATGACACT------------------------------ATCACACTCCCATGCAGAATAAAACAAATTATAAACATGTGGCAGGAAGTAGGCAAAGCAATGTATGCCCCTCCCATTAGAGGAGAGATTAGATGTTTATCAAATATTACAGGATTACTATTAACAAGAGATGGTGGTAATACT------------AGCACGAAT------------------------GACACC---GAGACCTTCAGACCTGGAGGAGGAGATATGAGGGACAATTGGAGA---AGTGAATTATACAAATATAAAGTAGTAAAAATAGAACCA---TTAGGAATAGCACCC---ACCAAGGCAAAGAGAAGAGTGGTGCAGAGAGAA---AAAAGAGCAGTG---GGAACAATAGGA---GCTATG---TTCCTT---GGG---------TTCTTGGGA---GCAGCAGGAAGCACTATGGGCGCAGCATCAGTA---ACGCTGACGGTACAAGCCAGACTATTATTGTCTGGTATAGTGCAACAGCAGAACAATCTGCTGAGGGCTATTGAGGCGCAACAGCATCTGTTGCAACTCACGGTCTGGGGCATTAAACAGCTCCAAGCAAGA---GTCCTGGCTGTAGAAAGATACCTACAGGATCAACAGCTCCTGGGGATTTGGGGTTGCTCTGGAAAGCTCATCTGCACCACTACTGTGCCTTGGAATGATAGTTGGAGT---------------------------AATAAATCTTACAGTGATATTTGGGAT---AACATGACCTGGATGCAGTGGGATAGAGAAATTAAC------AATTACACAACCCTCATATACACCTTACTTGAAGGTGCGCAGAACCAGCAAGAAAAGAATGAACACGAATTATTAGAATTGGATAAGTGGGCAAGTTTGTGGAATTGGTTTAGCATAACACAGTGGCTGTGGTATATAAAAATATTCATAATGATAGTAGGTGGTCTGATAGGTTTAAGAATAGTTTTTGCTGTGCTTTCTATAGTGAATAGAGTTAGGCAGGGATACTCACCACTATCATTTCAGACCCAC---CTCCCAGCCAGGAGGGAA------CCCGACAGGCCCGAAGGAATCGAAGAAGAAGGTGGAGAGAAAGACAAAGACAGATCAGTTCGATTAGTGCATGGATTCTTAGCACTCATCTGGGACGACCTACGGAGCCTGTGCCTCTTCAGCTACCACCGCTTGAGAGACTTACTATTGATTGTAACGAGGGTTGTGGAGACTCTGGGACGCAGG---------------GGGTGGGAAATCCTCAAGTATTGGTGG---AGTCTCCTTCAGTATTGG---------------------------------------------------AGTCAGGAACTAAAGAAGAGTGCTGTTAGCTTGTTTGATGCCATAGCTATCACAGCAGCTGAGGGGACAGATAGGGTTATAGAAGTAGTGCAAAGA------------------CTTTTTAGAGCTTTTCTCCACATACCTACAAGAATCAGACAGGGATTGGAAAGGGCTTTGTTATAA

C.SC05.Trinidad.EU578356 ATGAGAGTGATGGAGACAAGGAGGAATTGGCAGCACTTG---------TGGAAATGGGGC------------------------ACGATGCTCTTTGGGATGTTGATGATC------------TGTAGTGCTGCA---------GAGGATATGTGGGTCACAGTCTATTATGGGGTACCTGTGTGGAAAGAAGCGACCACCACTCTATTTTGTGCATCAGATGCTAAAGTATATGATACAGAGGTACATAAT---GTCTGGGCCACACATGCCTGTGTACCCACAGACCCCAACCCACAAGAAGTAGTATTG---GAAAATGTGACAGAAAATTTTAACATGTGGAAAAATAACATGGTAGAACAAATGCATGAGGATATAATCAGTTTATGGGATCAAAGCCTAAAGCCATGTGTAAAATTAACCCCACTCTGTGTTACTTTAAATTGTGCTGATGTGAATGGTACTAGAGATAATGCCACTAGT------------------------------------------------------------------------------------------------------ATAGAAAAAGGAGAAATAAAAAACTGCTCTTTTAATATCACC---ACAGCAATAAGGGAT------AAGGTGCAGAAAGCATATGCAACTTTTTATAGCCTTGATGTAGTACCAATAGATAATAAT------------------------------AGCAGTAGC---------------------------TATAGGTTGGCAAGTTGTAATACCTCAGTCATTACACAAGCCTGTCCAAAGGTGTCCTTTGAACCAATTCCCATACATTATTGTGCCCCGGCTGGTTTTGCGCTTCTAAAATGT---AATGATAAAAAGTTCAATGGAACAGGACCATGTAAAAATGTCAGCACAGTACAATGTACACATGGAATTAGACCAGTAGTGTCAACTCAACTGTTGTTAAATGGCAGTCTAGCAGAAGAA---GAGGTAGTAATTAGATCTGAAAATTTCACAGACAATGCTAAAACCATAATAGTACAGCTAAATAGCTCTGTAGTAATTAATTGTACAAGACCCAACAATAATACAAGAAAAGGTATACATATAGGA------------CCAGGGAGG---GTATTTTAT---ACAGGAAAAATAATAGGAAATATAAGACGAGCACATTGTAACCTT------AATAAAACAGATTGGAATAACACTTTAAAACAGGTAGTTATAAAGTTAAGA---GAACAATTTGGA------------AATAAAACA---ATAGTATTTAATCAA---------TCTGCAGGAGGGGATCTAGAAATTGTATTGCACAGTTTTAATTGTGGAGGGGAATTTTTCTACTGTAATGCAACACAACTGTTTAATAGTACTTGGAATATT---------AATGATACTTGGAATGATACG---------------------AATGGTACTACAGAGTTA------------AATGACACT------------------------------ATCATACTCCCATGCAGAATAAAACAAATTATAAACATGTGGCAGGAAGTAGGCAAAGCAATGTATGCCCCTCCCATTAGAGGACAGATTAGATGTTCATCAAATATTACAGGATTACTATTAACAAGAGATGGTGGTAATACT------------AGCACGAAT------------------------GACACC---GAGATCTTCAGACCTGGAGGAGGAGATATGAGGGACAATTGGAGA---AGTGAATTATATAAATATAAAGTAGTAAGAATTGAACCA---TTAGGAATAGCACCC---ACCAAGGCAAAGAGAAGAGTGGTGCAGAGAGAA---AAAAGAGCAGTG---GGAACAATAGGA---GCTATG---TTCCTT---GGG---------TTCTTGGGA---GCAGCAGGAAGCACTATGGGCGCAGCATCAGTA---ACGCTGACGGTACAAGCCAGACTATTATTGTCTGGTATAGTGCAACAGCAGAACAATCTGCTGAGGGCTATTGAGGCGCAACAGCATCTGTTGCAACTCACAGTCTGGGGCATTAAACAGCTCCAGGCAAGA---GTCCTGGCTGTGGAAAGATACCTACAGGATCAACAGCTCCTGGGGATTTGGGGTTGCTCTGGAAAACTCATCTGCACCACTACTGTGCCTTGGAATGCTAGTTGGAGT---------------------------AATAAATCTTACAGTGATATTTGGGAT---AACATGACCTGGATGCAGTGGGATAGAGAAATTAAC------AATTACACAACCCTCATATACACCTTACTTGAAGGTGCGCAGAACCAGCAAGAAAAGAATGAACACGAATTATTAGAATTGGATAAGTGGGCAAGTTTGTGGAATTGGTTTAGCATAACACAGTGGCTGTGGTATATAAAAATATTCATAATGATAGTAGGTGGTCTGATAGGTTTAAGAATAGTTTTTGCTGTGCTTTCTATAGTAAATAGAGTTAGGCAGGGATACTCACCACTATCATTTCAGACCCAC---CTCCCAGCCAGGAGGGAA------CCCGACAGGCCCGAAGGAATCGAAGGAGAAGGTGGAGAGAAAGACAAAGACAGATCAGTTCGATTAGTGCATGGATTCTTAGCACTCATCTGGGACGACCTACGGAGCCTGTGCCTCTTCAGCTACCACCGCTTGAGAGACTTACTCTTGATTGTAACGAGGATTGTGGAAACTCTGGGACGCAGG---------------GGGTGGGAAATCCTCAAGTATTGGTGG---AATCTCCTCCAGTATTGG---------------------------------------------------AGTCAGGAACTAAAGAATAGTGCTGTTAGCTTGTTTGATGCCACAGCTATCGCAGCAGCTGAGGGGACAGATAGGGTTATAGAAGTAGTACAAAGA------------------CTTTTTAGAGCTTTTCTCCACATACCTACAAGAATCAGACAGGGATTGGAAAGGGCTTTGTTATAA

2.SC05.Trinidad.EU576758 ATGAGAGTGAAGGAGACAAGGAGGAATTGGCAGCACTTG---------TGGAAATGGGGC------------------------ACGATGCTCCTTGGGATGTTGATGATC------------TGTAGTGCTGCA---------GGAAATTTGTGGGTCACAGTCTATTATGGGGTACCTGTGTGGAAAGAAGCGACCACCACTCTATTTTGTGCATCAGATGCTAAAGCATATGATACAGAGGTACATAAT---GTCTGGGCCACACATGCCTGTGTACCCACAGACCCCAACCCACAAGAAGTAGTATTG---GGAAATGTGACAGAAAATTTTAACATGTGGAAAAATAACATGGTAGAACAGATGCATGAGGATATAATCAGTTTATGGGATCAAAGCCTAAAGCCATGTGTAAAATTAACCCCACTCTGTGTTACTTTAAATTGTACTGATGTGAATGGTACTAGTGCTAATGTCACTAGT------------------------------------------------------------------------------------------------------ATAGAAAAAGGAGAAATAAAAAACTGCTCTTTTAATATCACC---ACAACAATAAGAGAT------AAGGTGCAGAAAGCATATGCAACTTTTTATAGCCTTGATGTAGTACCAATAGATAATGATCAAGAT------------------------AATAGTAGC---------------------AGTAGCTATAGGTTGACAAATTGTAATACCTCAGTCATTACACAGGCCTGTCCAAAGGTGTCCTTTGAACCAATTCCCATACATTATTGTGCCCCGGCTGGTTTTGCGCTTCTAAAATGT---AATGATAAAAAGTTCAATGGAACAGGACCATGTACAAATGTCAGCACAGTACAATGTACACATGGAATTAGACCAGTAGTGTCAACTCAACTGTTGTTAAATGGCAGTCTAGCAGAAGAA---GAGGTAGTAATTAGATCTGAAAATTTCACAGACAATGCTAAAACCATAATAGTACAGCTGAATAACTCTGTAGTAATTAATTGTACAAGACCCAACAATAATACAAGAAAAAGTATACCTATAGGA------------CCAGGGAGT---GTATTTTAT---ACAGGAGAAATAATAGGAGATATAAGACAAGCACATTGTAACCTT------AGTAATACAGATTGGAATAACACTTTAAAACAGGTAGTTATAAAATTAAGA---GAACAATTTGAG------------AATAAAACA---ATAGTCTTTAATCAA---------TCCGCAGGAGGGGATCCAGAAATTGTATTGCACAGTTTTAATTGTGGAGGGGAATTTTTCTACTGTAATGCAACACAACTGTTTAATAGTACTTGGAATATT---------AATGGTACTTGGAATGGTACG---------------------AATGGTACTACAGAGTCA------------AATGACACT------------------------------ATCACACTCCCATGCAGAATAAAACAAATTATAAACATGTGGCAGGAAGTAGGCAAAGCAATGTATGCCCCTCCCATTAGAGGACAGATTAGATGTTCATCAAATATTACAGGATTACTATTAACAAGAGACGGTGGTAATAAT------------AACACGAGT------------------------GACACC---GAGATCTTCAGACCTGGAGGAGGAGATATGAGGGACAATTGGAGA---AGTGAATTATATAAATATAAAGTAGTAAAAATTGAACCA---TTAGGAATAGCACCC---ACCAAGGCAAAGAGAAGAGTGGTGCAGAGAGAA---AAAAGAGCAGTG---GGAACAATAGGA---GCTATG---TTCCTT---GGG---------TTCTTGGGA---GCAGCAGGAAGCACTATGGGCGCAGCATCATTA---ACGCTGACGGTACAGGCCAGACTATTATTGTCTGGTATAGTGCAACAGCAGAACAATCTGCTGAGGGCTATTGAGGCGCAACAGCATCTGTTGCAACTCACGGTCTGGGGCATCAAACAGCTCCAGGCAAGA---GTCCTGGCTGTGGAAAGATACCTAAGGGATCAACAGCTCCTGGGGATTTGGGGTTGCTCTGGAAAACTCATTTGCACCACTACTGTGCCTTGGAATGCTAGTTGGAGT---------------------------AATAAATCTTTGAATTATATTTGGGAT---AACATGACCTGGATGCAGTGGGATAGAGAAATTAAC------AATTACACAACCCTCATATACACCTTACTTGAAGATGCGCAGAACCAGCAAGAAAAGAATGAACACGAATTATTAGAATTGGATAAGTGGGCAAGTTTGTGGAATTGGTTTGACATAACACATTGGCTGTGGTATATAAAAATATTCATAATGATAGTAGGTGGTCTGATAGGTTTAAGAATAGTTTTTGCTGTGCTTTCTATAGTGAATAGAGTTAGGCAGGGATACTCACCATTATCATTTCAGACCCAC---CTCCCAGCCAGGAGGGAA------CCCGACAGGCCCGAAGGAATCGAAGGAGAAGGTGGAGAGAAAGACAAAGACAGATCAGTTCGATTAGTGCATGGATTCTTAGCACTCATCTGGGACGACCTACGGAGCCTGTGCCTCTTCAGCTACCACCTCTTGAGAGACTTACTCTTGATTGTAACGAGGATTGTGGAAACTCTGGGACGCAGG---------------GGGTGGGAAGTCCTCAAGTATTGGTGG---AATCTCCTTCAGTATTGG---------------------------------------------------AGTCAGGAACTAAAGAATAGTGCTGTTAGCTTGTTTGATGCCATAGCTATCGCAGCAGCTGAGGGGACAGATAGGGCTATAGAAGTAGTGCGAAGA------------------CTTTTTAGAGCTTTTCTCCACATACCTACAAGAATCAGACAGGGATTGGAAAGGGCTTTGTTATAA

2.SC05.Trinidad.EU576767 ATGAGAGTGAAGGAGACAAGGAGGAATTGGCAGCACTTG---------TGGAAATGGGGC------------------------ACGATGCTCCTTGGGATGTTGATGATC------------TGTAGTGCTGCA---------GGAAATTTGTGGGTCACAGTCTATTATGGGGTACCTGTGTGGAAAGAAGCGACCACCACTCTATTTTGTGCATCAGATGCTAAAGCATATGATACAGAGGTACATAAT---GTCTGGGCCACACATGCCTGTGTACCCACAGACCCCAACCCACAAGAAGTAGTATTG---GGAAATGTGACAGAAAATTTTAACATGTGGAAAAATAACATGGTAGAACAGATGCATGAGGATATAATCAGTTTATGGGATCAAAGCCTAAAGCCATGTGTAAAATTAACCCCACTCTGTGTTACTTTAAATTGTACTGATGTGAATGGTACTAGTGCTAATGTCACTAGT------------------------------------------------------------------------------------------------------ATAGAAAAAGGAGAAATAAAAAACTGCTCTTTTAATATCACC---ACAACAATAAGAGAT------AAGGTGCAGAAAGCATATGCAACTTTTTATAGCCTTGATGTAGTACCAATAGATAATGATCAAGAT------------------------AATAGTAGC---------------------AGTAGCTATAGGTTGACAAATTGTAATACCTCAGTCATTACACAGGCCTGTCCAAAGGTGTCCTTTGAACCAATTCCCATACATTATTGTGCCCCGGCTGGTTTTGCGCTTCTAAAATGT---AATGATAAAAAGTTCAATGGAACAGGACCATGTACAAATGTCAGCACAGTACAATGTACACATGGAATTAGACCAGTAGTGTCAACTCAACTGTTGTTAAATGGCAGTCTAGCAGAAGAA---GAGGTAGTAATTAGATCTGAAAATTTCACAGACAATGCTAAAACCATAATAGTACAGCTGAATAACTCTGTAGTAATTAATTGTACAAGACCCAACAATAATACAAGAAAAAGTATACCTATAGGA------------CCAGGGAGT---GTATTTTAT---ACAGGAGAAATAATAGGAGATATAAGACAAGCACATTGTAACCTT------AGTAATACAGATTGGAATAACACTTTAAAACAGGTAGTTATAAAATTAAGA---GAACAATTTGAG------------AATAAAACA---ATAGTCTTTAATCAA---------TCCGCAGGAGGGGATCCAGAAATTGTATTGCACAGTTTTAATTGTGGAGGGGAATTTTTCTACTGTAATGCAACACAACTGTTTAATAGTACTTGGAATATT---------AATGGTACTTGGAATGGTACG---------------------AATGGTACTACAGAGTCA------------AATGACACT------------------------------ATCACACTCCCATGCAGAATAAAACAAATTATAAACATGTGGCAGGAAGTAGGCAAAGCAATGTATGCCCCTCCCATTAGAGGACAGATTAGATGTTCATCAAATATTACAGGATTACTATTAACAAGAGACGGTGGTAATAAT------------AACACGAGT------------------------GACACC---GAGATCTTCAGACCTGGAGGAGGAGATATGAGGGACAATTGGAGA---AGTGAATTATATAAATATAAAGTAGTAAAAATTGAACCA---TTAGGAATAGCACCC---ACCAAGGCAAAGAGAAGAGTGGTGCAGAGAGAA---AAAAGAGCAGTG---GGAACAATAGGA---GCTATG---TTCCTT---GGG---------TTCTTGGGA---GCAGCAGGAAGCACTATGGGCGCAGCATCATTA---ACGCTGACGGTACAGGCCAGACTATTATTGTCTGGTATAGTGCAACAGCAGAACAATCTGCTGAGGGCTATTGAGGCGCAACAGCATCTGTTGCAACTCACGGTCTGGGGCATCAAACAGCTCCAGGCAAGA---GTCCTGGCTGTGGAAAGATACCTAAGGGATCAACAGCTCCTGGGGATTTGGGGTTGCTCTGGAAAACTCATTTGCACCACTACTGTGCCTTGGAATGCTAGTTGGAGT---------------------------AATAAATCTTTGAATTATATTTGGGAT---AACATGACCTGGATGCAGTGGGATAGAGAAATTAAC------AATTACACAACCCTCATATACACCTTACTTGAAGATGCGCAGAACCAGCAAGAAAAGAATGAACACGAATTATTAGAATTGGATAAGTGGGCAAGTTTGTGGAATTGGTTTGACATAACACATTGGCTGTGGTATATAAAAATATTCATAATGATAGTAGGTGGTCTGATAGGTTTAAGAATAGTTTTTGCTGTGCTTTCTATAGTGAATAGAGTTAGGCAGGGATACTCACCATTATCATTTCAGACCCAC---CTCCCAGCCAGGAGGGAA------CCCGACAGGCCCGAAGGAATCGAAGGAGAAGGTGGAGAGAAAGACAAAGACAGATCAGTTCGATTAGTGCATGGATTCTTAGCACTCATCTGGGACGACCTACGGAGCCTGTGCCTCTTCAGCTACCACCTCTTGAGAGACTTACTCTTGATTGTAACGAGGATTGTGGAAACTCTGGGACGCAGG---------------GGGTGGGAAGTCCTCAAGTATTGGTGG---AATCTCCTTCAGTATTGG---------------------------------------------------AGTCAGGAACTGAAGAATAGTGCTGTTAGCTTGTTTGATGCCATAGCTATCGCAGCAGCTGAGGGGACAGATAGGGCTATAGAAGTAGTGCGAAGA------------------CTTTTTAGAGCTTTTCTCCACATACCTACAAGAATCAGACAGGGATTGGAAAGGGCTTTGTTATAA

2.SC05.Trinidad.EU576780 ATGAGAGTGAAGGAGACAAGGAGGAATTGGCAGCACTTG---------TGGAAATGGGGC------------------------ACGATGCTCCTTGGGATGTTGATGATC------------TGTAGTGCTGCA---------GGAAATTTGTGGGTCACAGTCTATTATGGGGTACCTGTGTGGAAAGAAGCGACCACCACTCTATTTTGTGCATCAGATGCTAAAGCATATGATACAGAGGTACATAAT---GTCTGGGCCACACATGCCTGTGTACCCACAGACCCCAACCCACAAGAAGTAGTATTG---GGAAATGTGACAGAAAATTTTAACATGTGGAAAAATAACATGGTAGAACAGATGCATGAGGATATAATCAGTTTATGGGATCAAAGCCTAAAGCCATGTGTAAAATTAACCCCACTCTGTGTTACTTTAAATTGTACTGATGTGAATGGTACTAGTGCTAATGTCACTAGT------------------------------------------------------------------------------------------------------ATAGAAAAAGGAGAAATAAAAAACTGCTCTTTTAATATCACC---ACAACAATAAGAGAT------AAGGTGCAGAAAGCATATGCAACTTTTTATAGCCTTGATGTAGTACCAATAGATAATGATCAAGAT------------------------AATAGTAGC---------------------AGTAGCTATAGGTTGACAAATTGTAATACCTCAGTCATTACACAGGCCTGTCCAAAGGTGTCCTTTGAACCAATTCCCATACATTATTGTGCCCCGGCTGGTTTTGCGCTTCTAAAATGT---AATGATAAAAAGTTCAATGGAACAGGACCATGTACAAATGTCAGCACAGTACAATGTACACATGGAATTAGACCAGTAGTGTCAACTCAACTGTTGTTAAATGGCAGTCTAGCAGAAGAA---GAGGTAGTAATTAGATCTGAAAATTTCACAGACAATGCTAAAACCATAATAGTACAGCTGAATAACTCTGTAGTAATTAATTGTACAAGACCCAACAATAATACAAGAAAAAGTATACCTATAGGA------------CCAGGGAGT---GTATTTTAT---ACAGGAGAAATAATAGGAGATATAAGACAAGCACATTGTAACCTT------AGTAATACAGATTGGAATAACACTTTAAAACAGGTAGTTATAAAATTAAGA---GAACAATTTGAG------------AATAAAACA---ATAGTCTTTAATCAA---------TCCGCAGGAGGGGATCCAGAAATTGTATTGCACAGTTTTAATTGTGGAGGGGAATTTTTCTACTGTAATGCAACACAACTGTTTAATAGTACTTGGAATATT---------AATGGTACTTGGAATGGTACG---------------------AATGGTACTACAGAGTCA------------AATGACACT------------------------------ATCACACTCCCATGCAGAATAAAACAAATTATAAACATGTGGCAGGAAGTAGGCAAAGCAATGTATGCCCCTCCCATTAGAGGACAGATTAGATGTTCATCAAATATTACAGGATTACTATTAACAAGAGACGGTGGTAATAAT------------AACACGAGT------------------------GACACC---GAGATCTTCAGACCTGGAGGAGGAGATATGAGGGACAATTGGAGA---AGTGAATTATATAAATATAAAGTAGTAAAAATTGAACCA---TTAGGAATAGCACCC---ACCAAGGCAAAGAGAAGAGTGGTGCAGAGAGAA---AAAAGAGCAGTG---GGAACAATAGGA---GCTATG---TTCCTT---GGG---------TTCTTGGGA---GCAGCAGGAAGCACTATGGGCGCAGCATCATTA---ACGCTGACGGTACAGGCCAGACTATTATTGTCTGGTATAGTGCAACAGCAGAACAATCTGCTGAGGGCTATTGAGGCGCAACAGCATCTGTTGCAACTCACGGTCTGGGGCATCAAACAGCTCCAGGCAAGA---GTCCTGGCTGTGGAAAGATACCTAAGGGATCAACAGCTCCTGGGGATTTGGGGTTGCTCTGGAAAACTCATTTGCACCACTACTGTGCCTTGGAATGCTAGTTGGAGT---------------------------AATAAATCTTTGAATTATATTTGGGAT---AACATGACCTGGATGCAGTGGGATAGAGAAATTAAC------AATTACACAACCCTCATATACACCTTACTTGAAGATGCGCAGAACCAGCAAGAAAAGAATGAACACGAATTATTAGAATTGGATAAGTGGGCAAGTTTGTGGAATTGGTTTGACATAACACATTGGCTGTGGTATATAAAAATATTCATAATGATAGTAGGTGGTCTGATAGGTTTAAGAATAGTTTTTGCTGTGCTTTCTATAGTGAATAGAGTTAGGCAGGGATACTCACCATTATCATTTCAGACCCAC---CTCCCAGCCAGGAGGGAA------CCCGACAGGCCCGAAGGAATCGAAGGAGAAGGTGGAGAGAAAGACAAAGACAGATCAGTTCGATTAGTGCATGGATTCTTAGCACTCATCTGGGACGACCTACGGAGCCTGTGCCTCTTCAGCTACCACCTCTTGAGAGACTTACTCTTGATTGTAACGAGGATTGTGGAAACTCTGGGACGCAGG---------------GGGTGGGAAGTCCTCAAGTATTGGTGG---AATCTCCTTCAGTATTGG---------------------------------------------------AGTCAGGAACTAAAGAATAGTGCTGTTAGCTTGTTTGATGCCATAGCTATCGCAGCAGCTGAGGGGACAGATAGGGCTATAGAAGTAGTGCGAAGA------------------CTTTTTAGAGCTTTTCTCCACATACCTACAAGAATCAGACAGGGATTGGAAAGGGCTTTGTTATAA

2.SC05.Trinidad.EU576782 ATGAGAGTGAAGGAGACAAGGAGGAACTGGCAGCACTTG---------TGGAAATGGGGC------------------------ACGATGCTCCTTGGGATGTTGATGATC------------TGTAGTGCTGCA---------GGAAATTTGTGGGTCACAGTCTATTATGGGGTACCTGTGTGGAAAGAAGCGACCACCACTCTATTTTGTGCATCAGATGCTAAAGCATATGATACAGAGGTACATAAT---GTCTGGGCCACACATGCCTGTGTACCCACAGACCCCAACCCACAAGAAGTAGTATTG---GGAAATGTGACAGAAAATTTTAACATGTGGAAAAATAACATGGTAGAACAGATGCATGAGGATATAATCAGTTTATGGGATCAAAGCCTAAAGCCATGTGTAAAATTAACCCCACTCTGTGTTACTTTAAATTGTACTGATGTGAATGGTACTAGTGCTAATGTCACTAGT------------------------------------------------------------------------------------------------------ATAGAAAAAGGAGAAATAAAAAACTGCTCTTTTAATATCACC---ACAACAATAAGAGAT------AAGGTGCAGAAAGCATATGCAACTTTTTATAGCCTTGATGTAGTACCAATAGATAATGATCAAGAT------------------------AATAGTAGC---------------------AGTAGCTATAGGTTGACAAATTGTAATACCTCAGTCATTACACAGGCCTGTCCAAAGGTGTCCTTTGAACCAATTCCCATACATTATTGTGCCCCGGCTGGTTTTGCGCTTCTAAAATGT---AATGATAAAAAGTTCAATGGAACAGGACCATGTACAAATGTCAGCACAGTACAATGTACACATGGAATTAGACCAGTAGTGTCAACTCAACTGTTGTTAAATGGCAGTCTAGCAGAAGAA---GAGGTAGTAATTAGATCTGAAAATTTCACAGACAATGCTAAAACCATAATAGTACAGCTGAATAACTCTGTAGTAATTAATTGTACAAGACCCAACAATAATACAAGAAAAAGTATACCTATAGGA------------CCAGGGAGT---GTATTTTAT---ACAGGAGAAATAATAGGAGATATAAGACAAGCACATTGTAACCTT------AGTAATACAGATTGGAATAACACTTTAAAACAGGTAGTTATAAAATTAAGA---GAACAATTTGAG------------AATAAAACA---ATAGTCTTTAATCAA---------TCCGCAGGAGGGGATCCAGAAATTGTATTGCACAGTTTTAATTGTGGAGGGGAATTTTTCTACTGTAATGCAACACAACTGTTTAATAGTACTTGGAATATT---------AATGGTACTTGGAATGGTACG---------------------AATGGTACTACAGAGTCA------------AATGACACT------------------------------ATCACACTCCCATGCAGAATAAAACAAATTATAAACATGTGGCAGGAAGTAGGCAAAGCAATGTATGCCCCTCCCATTAGAGGACAGATTAGATGTTCATCAAATATTACAGGATTACTATTAACAAGAGACGGTGGTAATAAT------------AACACGAGT------------------------GACACC---GAGATCTTCAGACCTGGAGGAGGAGATATGAGGGACAATTGGAGA---AGTGAATTATATAAATATAAAGTAGTAAAAATTGAACCA---TTAGGAATAGCACCC---ACCAAGGCAAAGAGAAGAGTGGTGCAGAGAGAA---AAAAGAGCAGTG---GGAACAATAGGA---GCTATG---TTCCTT---GGG---------TTCTTGGGA---GCAGCAGGAAGCACTATGGGCGCAGCATCATTA---ACGCTGACGGTACAGGCCAGACTATTATTGTCTGGTATAGTGCAACAGCAGAACAATCTGCTGAGGGCTATTGAGGCGCAACAGCATCTGTTGCAACTCACGGTCTGGGGCATCAAACAGCTCCAGGCAAGA---GTCCTGGCTGTGGAAAGATACCTAAGGGATCAACAGCTCCTGGGGATTTGGGGTTGCTCTGGAAAACTCATTTGCACCACTACTGTGCCTTGGAATGCTAGTTGGAGT---------------------------AATAAATCTTTGAATTATATTTGGGAT---AACATGACCTGGATGCAGTGGGATAGAGAAATTAAC------AATTACACAACCCTCATATACACCTTACTTGAAGATGCGCAGAACCAGCAAGAAAAGAATGAACACGAATTATTAGAATTGGATAAGTGGGCAAGTTTGTGGAATTGGTTTGACATAACACATTGGCTGTGGTATATAAAAATATTCATAATGATAGTAGGTGGTCTGATAGGTTTAAGAATAGTTTTTGCTGTGCTTTCTATAGTGAATAGAGTTAGGCAGGGATACTCACCATTATCATTTCAGACCCAC---CTCCCAGCCAGGAGGGAA------CCCGACAGGCCCGAAGGAATCGAAGGAGAAGGTGGAGAGAAAGACAAAGACAGATCAGTTCGATTAGTGCATGGATTCTTAGCACTCATCTGGGACGACCTACGGAGCCTGTGCCTCTTCAGCTACCACCTCTTGAGAGACTTACTCTTGATTGTAACGAGGATTGTGGAAACTCTGGGACGCAGG---------------GGGTGGGAAGTCCTCAAGTATTGGTGG---AATCTCCTTCAGTATTGG---------------------------------------------------AGTCAGGAACTAAAGAATAGTGCTGTTAGCTTGTTTGATGCCATAGCTATCGCAGCAGCTGAGGGGACAGATAGGGCTATAGAAGTAGTGCGAAGA------------------CTTTTTAGAGCTTTTCTCCACATACCTACAAGAATCAGACAGGGATTGGAAAGGGCTTTGTTATAA

2.SC05.Trinidad.EU576761 ATGAGAGTGAAGGAGACAAGGAGGAATTGGCAGCACTTG---------TGGAAATGGGGC------------------------ACGATGCTCCTTGGGATGTTGATGATC------------TGTAGTGCTGCA---------GGAAATTTGTGGGTCACAGTCTATTATGGGGTACCTGTGTGGAAAGAAGCGACCACCACTCTATTTTGTGCATCAGATGCTAAAGCATATGATACAGAGGTACATAAT---GTCTGGGCCACACATGCCTGTGTACCCACAGACCCCAACCCACAAGAAGTAGTATTG---GGAAATGTGACAGAAAATTTTAACATGTGGAAAAATAACATGGTAGAACAGATGCATGAGGATATAATCAGTTTATGGGATCAAAGCCTAAAGCCATGTGTAAAATTAACCCCACTCTGTGTTACTTTAAATTGTACTGATGTGAATGGTACTAGTGCTAATGTCACTAGT------------------------------------------------------------------------------------------------------ATAGAAAAAGGAGAAATAAAAAACTGCTCTTTTAATATCACC---ACAACAATAAGAGAT------AAGGTGCAGAAAGCATATGCAACTTTTTATAGCCTTGATGTAGTACCAATAGATAATGATCAAGAT------------------------AATAGTAGC---------------------AGTAGCTATAGGTTGACAAATTGTAATACCTCAGTCATTACACAGGCCTGTCCAAAGGTGTCCTTTGAACCAATTCCCATACATTATTGTGCCCCGGCTGGTTTTGCGCTTCTAAAATGT---AATGATAAAAAGTTCAATGGAACAGGACCATGTACAAATGTCAGCACAGTACAATGTACACATGGAATTAGACCAGTAGTGTCAACTCAACTGTTGTTAAATGGCAGTCTAGCAGAAGAA---GAGGTAGTAATTAGATCTGAAAATTTCACAGACAATGCTAAAACCATAATAGTACAGCTGAATAACTCTGTAGTAATTAATTGTACAAGACCCAACAATAATACAAGAAAAAGTATACCTATAGGA------------CCAGGGAGT---GTATTTTAT---ACAGGAGAAATAATAGGAGATATAAGACAAGCACATTGTAACCTT------AGTAATACAGATTGGAATAACACTTTAAAACAGGTAGTTATAAAATTAAGA---GAACAATTTGAG------------AATAAAACA---ATAGTCTTTAATCAA---------TCCGCAGGAGGGGATCCAGAAATTGTATTGCACAGTTTTAATTGTGGAGGGGAATTTTTCTACTGTAATGCAACACAACTGTTTAATAGTACTTGGAATATT---------AATGGTACTTGGAATGGTACG---------------------AATGGTACTACAGAGTCA------------AATGACACT------------------------------ATCACACTCCCATGCAGAATAAAACAAATTATAAACATGTGGCAGGAAGTAGGCAAAGCAATGTATGCCCCTCCCATTAGAGGACAGATTAGATGTTCATCAAATATTACAGGATTACTATTAACAAGAGACGGTGGTAATAAT------------AACACGAGT------------------------GACACC---GAGATCTTCAGACCTGGAGGAGGAGATATGAGGGACAATTGGAGA---AGTGAATTATATAAATATAAAGTAGTAAAAATTGAACCA---TTAGGAATAGCACCC---ACCAAGGCAAAGAGAAGAGTGGTGCAGAGAGAA---AAAAGAGCAGTG---GGAACAATAGGA---GCTATG---TTCCTT---GGG---------TTCTTGGGA---GCAGCAGGAAGCACTATGGGCGCAGCATCATTA---ACGCTGACGGTACAGGCCAGACTATTATTGTCTGGTATAGTGCAACAGCAGAACAATCTGCTGAGGGCTATTGAGGCGCAACAGCATCTGTTGCAACTCACGGTCTGGGGCATCAAACAGCTCCAGGCAAGA---GTCCTGGCTGTGGAAAGATACCTAAGGGATCAACAGCTCCTGGGGATTTGGGGTTGCTCTGGAAAACTCATTTGCACCACTACTGTGCCTTGGAATGCTAGTTGGAGT---------------------------AATAAATCTTTGAATTATATTTGGGAT---AACATGACCTGGATGCAGTGGGATAGAGAAATTAAC------AATTACACAACCCTCATATACACCTTACTTGAAGATGCGCAGAACCAGCAAGAAAAGAATGAACACGAATTATTAGAATTGGATAAGTGGGCAAGTTTGTGGAATTGGTTTGACATAACACATTGGCTGTGGTATATAAAAATATTCATAATGATAGTAGGTGGTCTGATAGGTTTAAGAATAGTTTTTGCTGTGCTTTCTATAGTGAATAGAGTTAGGCAGGGATACTCACCATTATCATTTCAGACCCAC---CTCCCAGCCAGGAGGGAA------CCCGACAGGCCCGAAGGAATCGAAGGAGAAGGTGGAGAGAAAGACAAAGACAGATCAGTTCGATTAGTGCATGGATTCTTAGCACTCATCTGGGACGACCTACGGAGCCTGTGCCTCTTCAGCTACCACCTCTTGAGAGACTTACTCTTGATTGTAACGAGGATTGTGGAAACTCTGGGACGCAGG---------------GGGTGGGAAGTCCTCAAGTATTGGTGG---AATCTCCTTCAGTATTGG---------------------------------------------------AGTCAGGAACTAAAGAATAGTGCTGTTAGCTTGTTTGATGCCATAGCTATCGCAGCAGCTGAGGGGACAGATAGGGCTATAGAAGTAGTGCGAAGA------------------CTTTTTAGAGCTTTTCTCCACATACCTACAAGAATCAGACAGGGATTGGAAAGGGCTTTGTTATAA

2.SC05.Trinidad.EU576770 ATGAGAGTGAAGGAGACAAGGAGGAATTGGCAGCACTTG---------TGGAAATGGGGC------------------------ACGATGCTCCTTGGGATGTTGATGATC------------TGTAGTGCTGCA---------GGAAATTTGTGGGTCACAGTCTATTATGGGGTACCTGTGTGGAAAGAAGCGACCACCACTCTATTTTGTGCATCAGATGCTAAAGCATATGATACAGAGGTACATAAT---GTCTGGGCCACACATGCCTGTGTACCCACAGACCCCAACCCACAAGAAGTAGTATTG---GGAAATGTGACAGAAAATTTTAACATGTGGAAAAATAACATGGTAGAACAGATGCATGAGGATATAATCAGTTTATGGGATCAAAGCCTAAAGCCATGTGTAAAATTAACCCCACTCTGTGTTACTTTAAATTGTACTGATGTGAATGGTACTAGTGCTAATGTCACTAGT------------------------------------------------------------------------------------------------------ATAGAAAAAGGAGAAATAAAAAACTGCTCTTTTAATATCACC---ACAACAATAAGAGAT------AAGGTGCAGAAAGCATATGCAACTTTTTATAGCCTTGATGTAGTACCAATAGATAATGATCAAGAT------------------------AATAGTAGC---------------------AGTAGCTATAGGTTGACAAATTGTAATACCTCAGTCATTACACAGGCCTGTCCAAAGGTGTCCTTTGAACCAATTCCCATACATTATTGTGCCCCGGCTGGTTTTGCGCTTCTAAAATGT---AATGATAAAAAGTTCAATGGAACAGGACCATGTACAAATGTCAGCACAGTACAATGTACACATGGAATTAGACCAGTAGTGTCAACTCAACTGTTGTTAAATGGCAGTCTAGCAGAAGAA---GAGGTAGTAATTAGATCTGAAAATTTCACAGACAATGCTAAAACCATAATAGTACAGCTGAATAACTCTGTAGTAATTAATTGTACAAGACCCAACAATAATACAAGAAAAAGTATACCTATAGGA------------CCAGGGAGT---GTATTTTAT---ACAGGAGAAATAATAGGAGATATAAGACAAGCACATTGTAACCTT------AGTAATACAGATTGGAATAACACTTTAAAACAGGTAGTTATAAAATTAAGA---GAACAATTTGAG------------AATAAAACA---ATAGTCTTTAATCAA---------TCCGCAGGAGGGGATCCAGAAATTGTATTGCACAGTTTTAATTGTGGAGGGGAATTTTTCTACTGTAATGCAACACAACTGTTTAATAGTACTTGGAATATT---------AATGGTACTTGGAATGGTACG---------------------AATGGTACTACAGAGTCA------------AATGACACT------------------------------ATCACACTCCCATGCAGAATAAAACAAATTATAAACATGTGGCAGGAAGTAGGCAAAGCAATGTATGCCCCTCCCATTAGAGGACAGATTAGATGTTCATCAAATATTACAGGATTACTATTAACAAGAGACGGTGGTAATAAT------------AACACGAGT------------------------GACACC---GAGATCTTCAGACCTGGAGGAGGAGATATGAGGGACAATTGGAGA---AGTGAATTATATAAATATAAAGTAGTAAAAATTGAACCA---TTAGGAATAGCACCC---ACCAAGGCAAAGAGAAGAGTGGTGCAGAGAGAAAAAAAAAGAGCAGTG---GGAACAATAGGA---GCTATG---TTCCTT---GGG---------TTCTTGGGA---GCAGCAGGAAGCACTATGGGCGCAGCATCATTA---ACGCTGACGGTACAGGCCAGACTATTATTGTCTGGTATAGTGCAACAGCAGAACAATCTGCTGAGGGCTATTGAGGCGCAACAGCATCTGTTGCAACTCACGGTCTGGGGCATCAAACAGCTCCAGGCAAGA---GTCCTGGCTGTGGAAAGATACCTAAGGGATCAACAGCTCCTGGGGATTTGGGGTTGCTCTGGAAAACTCATTTGCACCACTACTGTGCCTTGGAATGCTAGTTGGAGT---------------------------AATAAATCTTTGAATTATATTTGGGAT---AACATGACCTGGATGCAGTGGGATAGAGAAATTAAC------AATTACACAACCCTCATATACACCTTACTTGAAGATGCGCAGAACCAGCAAGAAAAGAATGAACACGAATTATTAGAATTGGATAAGTGGGCAAGTTTGTGGAATTGGTTTGACATAACACATTGGCTGTGGTATATAAAAATATTCATAATGATAGTAGGTGGTCTGATAGGTTTAAGAATAGTTTTTGCTGTGCTTTCTATAGTGAATAGAGTTAGGCAGGGATACTCACCATTATCATTTCAGACCCAC---CTCCCAGCCAGGAGGGAA------CCCGACAGGCCCGAAGGAATCGAAGGAGAAGGTGGAGAGAAAGACAAAGACAGATCAGTTCGATTAGTGCATGGATTCTTAGCACTCATCTGGGACGACCTACGGAGCCTGTGCCTCTTCAGCTACCACCTCTTGAGAGACTTACTCTTGATTGTAACGAGGATTGTGGAAACTCTGGGACGCAGG---------------GGGTGGGAAGTCCTCAAGTATTGGTGG---AATCTCCTTCAGTATTGG---------------------------------------------------AGTCAGGAACTAAAGAATAGTGCTGTTAGCTTGTTTGATGCCATAGCTATCGCAGCAGCTGAGGGGACAGATAGGGCTATAGAAGTAGTGCGAAGA------------------CTTTTTAGAGCTTTTCTCCACATACCTACAAGAATCAGACAGGGATTGGAAAGGGCTTTGTTATAA

2.SC05.Trinidad.EU576769 ATGAGAGTGAAGGAGACAAGGAGGAATTGGCAGCACTTG---------TGGAAATGGGGC------------------------ACGATGCTCCTTGGGATGTTGATGATC------------TGTAGTGCTGCA---------GGAAATTTGTGGGTCACAGTCTATTATGGGGTACCTGTGTGGAAAGAAGCGACCACCACTCTATTTTGTGCATCAGATGCTAAAGCATATGATACAGAGGTACATAAT---GTCTGGGCCACACATGCCTGTGTACCCACAGACCCCAACCCACAAGAAGTAGTATTG---GGAAATGTGACAGAAAATTTTAACATGTGGAAAAATAACATGGTAGAACAGATGCATGAGGATATAATCAGTTTATGGGATCAAAGCCTAAAGCCATGTGTAAAATTAACCCCACTCTGTGTTACTTTAAATTGTACTGATGTGAATGGTACTAGTGCTAATGTCACTAGT------------------------------------------------------------------------------------------------------ATAGAAAAAGGAGAAATAAAAAACTGCTCTTTTAATATCACC---ACAACAATAAGAGAT------AAGGTGCAGAAAGCATATGCAACTTTTTATAGCCTTGATGTAGTACCAATAGATAATGATCAAGAT------------------------AATAGTAGC---------------------AGTAGCTATAGGTTGACAAATTGTAATACCTCAGTCATTACACAGGCCTGTCCAAAGGTGTCCTTTGAACCAATTCCCATACATTATTGTGCCCCGGCTGGTTTTGCGCTTCTAAAATGT---AATGATAAAAAGTTCAATGGAACAGGACCATGTACAAATGTCAGCACAGTACAATGTACACATGGAATTAGACCAGTAGTGTCAACTCAACTGTTGTTAAATGGCAGTCTAGCAGAAGAA---GAGGTAGTAATTAGATCTGAAAATTTCACAGACAATGCTAAAACCATAATAGTACAGCTGAATAACTCTGTAGTAATTAATTGTACAAGACCCAACAATAATACAAGAAAAAGTATACCTATAGGA------------CCAGGGAGT---GTATTTTAT---ACAGGAGAAATAATAGGAGATATAAGACAAGCACATTGTAACCTT------AGTAATACAGATTGGAATAACACTTTAAAACAGGTAGTTATAAAATTAAGA---GAACAATTTGAG------------AATAAAACA---ATAGTCTTTAATCAA---------TCCGCAGGAGGGGATCCAGAAATTGTATTGCACAGTTTTAATTGTGGAGGGGAATTTTTCTACTGTAATGCAACACAACTGTTTAATAGTACTTGGAATATT---------AATGGTACTTGGAATGGTACG---------------------AATGGTACTACAGAGTCA------------AATGACACT------------------------------ATCACACTCCCATGCAGAATAAAACAAATTATAAACATGTGGCAGGAAGTAGGCAAAGCAATGTATGCCCCTCCCATTAGAGGACAGATTAGATGTTCATCAAATATTACAGGATTACTATTAACAAGAGACGGTGGTAATAAT------------AACACGAGT------------------------GACACC---GAGATCTTCAGACCTGGAGGAGGAGATATGAGGGACAATTGGAGA---AGTGAATTATATAAATATAAAGTAGTAAAAATTGAACCA---TTAGGAATAGCACCC---ACCAAGGCAAAGAGAAGAGTGGTGCAGAGAGAA---AAAAGAGCAGTG---GGAACAATAGGA---GCTATG---TTCCTT---GGG---------TTCTTGGGA---GCAGCAGGAAGCACTATGGGCGCAGCATCATTA---ACGCTGACGGTACAGGCCAGACTATTATTGTCTGGTATAGTGCAACAGCAGAACAATCTGCTGAGGGCTATTGAGGCGCAACAGCATCTGTTGCAACTCACGGTCTGGGGCATCAAACAGCTCCAGGCAAGA---GTCCTGGCTGTGGAAAGATACCTAAGGGATCAACAGCTCCTGGGGATTTGGGGTTGCTCTGGAAAACTCATTTGCACCACTACTGTGCCTTGGAATGCTAGTTGGAGT---------------------------AATAAATCTTTGAATTATATTTGGGAT---AACATGACCTGGATGCAGTGGGATAGAGAAATTAAC------AATTACACAACCCTCATATACACCTTACTTGAAGATGCGCAGAACCAGCAAGAAAAGAATGAACACGAATTATTAGAATTGGATAAGTGGGCAAGTTTGTGGAATTGGTTTGACATAACACATTGGCTGTGGTATATAAAAATATTCATAATGATAGTAGGTGGTCTGATAGGTTTAAGAATAGTTTTTGCTGTGCTTTCTATAGTGAATAGAGTTAGGCAGGGATACTCACCATTATCATTTCAGACCCAC---CTCCCAGCCAGGAGGGAA------CCCGACAGGCCCGAAGGAATCGAAGGAGAAGGTGGAGAGAAAGACAAAGACAGATCAGTTCGATTAGTGCATGGATTCTTAGCACTCATCTGGGACGACCTACGGAGCCTGTGCCTCTTCAGCTACCACCTCTTGAGAGACTTACTCTTGATTGTAACGAGGATTGTGGAAACTCTGGGACGCAGG---------------GGGTGGGAAGTCCTCAAGTATTGGTGG---AATCTCCTTCAGTATTGG---------------------------------------------------AGTCAGGAACTAAAGAATAGTGCTGTTAGCTTGTTTGATGCCATAGCTATCGCAGCAGCTGAGGGGACAGATAGGGCTATAGAAGTAGTGCGAAGA------------------CTTTTTAGAGCTTTTCTCCACATACCTACAAGAATCAGACAGGGATTGGAAAGGGCTTTGTTATAA

2.SC05.Trinidad.EU576783 ATGAGAGTGAAGGAGACAAGGAGGAATTGGCAGCACTTG---------TGGAAATGGGGC------------------------ACGATGCTCCTTGGGATGTTGATGATC------------TGTAGTGCTGCA---------GGAAATTTGTGGGTCACAGTCTATTATGGGGTACCTGTGTGGAAAGAAGCGACCACCACTCTATTTTGTGCATCAGATGCTAAAGCATATGATACAGAGGTACATAAT---GTCTGGGCCACACATGCCTGTGTACCCACAGACCCCAACCCACAAGAAGTAGTATTG---GGAAATGTGACAGAAAATTTTAACATGTGGAAAAATAACATGGTAGAACAGATGCATGAGGATATAATCAGTTTATGGGATCAAAGCCTAAAGCCATGTGTAAAATTAACCCCACTCTGTGTTACTTTAAATTGTACTGATGTGAATGGTACTAGTGCTAATGTCACTAGT------------------------------------------------------------------------------------------------------ATAGAAAAAGGAGAAATAAAAAACTGCTCTTTTAATATCACC---ACAACAATAAGAGAT------AAGGTGCAGAAAGCATATGCAACTTTTTATAGCCTTGATGTAGTACCAATAGATAATGATCAAGAT------------------------AATAGTAGC---------------------AGTAGCTATAGGTTGACAAATTGTAATACCTCAGTCATTACACAGGCCTGTCCAAAGGTGTCCTTTGAACCAATTCCCATACATTATTGTGCCCCGGCTGGTTTTGCGCTTCTAAAATGT---AATGATAAAAAGTTCAATGGAACAGGACCATGTACAAATGTCAGCACAGTACAATGTACACATGGAATTAGACCAGTAGTGTCAACTCAACTGTTGTTAAATGGCAGTCTAGCAGAAGAA---GAGGTAGTAATTAGATCTGAAAATTTCACAGACAATGCTAAAACCATAATAGTACAGCTGAATAACTCTGTAGTAATTAATTGTACAAGACCCAACAATAATACAAGAAAAAGTATACCTATAGGA------------CCAGGGAGT---GTATTTTAT---ACAGGAGAAATAATAGGAGATATAAGACAAGCACATTGTAACCTT------AGTAATACAGATTGGAATAACACTTTAAAACAGGTAGTTATAAAATTAAGA---GAACAATTTGAG------------AATAAAACA---ATAGTCTTTAATCAA---------TCCGCAGGAGGGGATCCAGAAATTGTATTGCACAGTTTTAATTGTGGAGGGGAATTTTTCTACTGTAATGCAACACAACTGTTTAATAGTACTTGGAATATT---------AATGGTACTTGGAATGGTACG---------------------AATGGTACTACAGAGTCA------------AATGACACT------------------------------ATCACACTCCCATGCAGAATAAAACAAATTATAAACATGTGGCAGGAAGTAGGCAAAGCAATGTATGCCCCTCCCATTAGAGGACAGATTAGATGTTCATCAAATATTACAGGATTACTATTAACAAGAGACGGTGGTAATAAT------------AACACGAGT------------------------GACACC---GAGATCTTCAGACCTGGAGGAGGAGATATGAGGGACAATTGGAGA---AGTGAATTATATAAATATAAAGTAGTAAAAATTGAACCA---TTAGGAATAGCACCC---ACCAAGGCAAAGAGAAGAGTGGTGCAGAGAGAA---AAAAGAGCAGTG---GGAACAATAGGA---GCTATG---TTCCTT---GGG---------TTCTTGGGA---GCAGCAGGAAGCACTATGGGCGCAGCATCATTA---ACGCTGACGGTACAGGCCAGACTATTATTGTCTGGTATAGTGCAACAGCAGAACAATCTGCTGAGGGCTATTGAGGCGCAACAGCATCTGTTGCAACTCACGGTCTGGGGCATCAAACAGCTCCAGGCAAGA---GTCCTGGCTGTGGAAAGATACCTAAGGGATCAACAGCTCCTGGGGATTTGGGGTTGCTCTGGAAAACTCATTTGCACCACTACTGTGCCTTGGAATGCTAGTTGGAGT---------------------------AATAAATCTTTGAATTATATTTGGGAT---AACATGACCTGGATGCAGTGGGATAGAGAAATTAAC------AATTACACAACCCTCATATACACCTTACTTGAAGATGCGCAGAACCAGCAAGAAAAGAATGAACACGAATTATTAGAATTGGATAAGTGGGCAAGTTTGTGGAATTGGTTTGACATAACACATTGGCTGTGGTATATAAAAATATTCATAATGATAGTAGGTGGTCTGATAGGTTTAAGAATAGTTTTTGCTGTGCTTTCTATAGTGAATAGAGTTAGGCAGGGATACTCACCATTATCATTTCAGACCCAC---CTCCCAGCCAGGAGGGAA------CCCGACAGGCCCGAAGGAATCGAAGGAGAAGGTGGAGAGAAAGACAAAGACAGATCAGTTCGATTAGTGCATGGATTCTTAGCACTCATCTGGGACGACCTACGGAGCCTGTGCCTCTTCAGCTACCACCTCTTGAGAGACTTACTCTTGATTGTAACGAGGATTGTGGAAACTCTGGGACGCAGG---------------GGGTGGGAAGTCCTCAAGTATTGGTGG---AATCTCCTTCAGTATTGG---------------------------------------------------AGTCAGGAACTAAAGAATAGTGCTGTTAGCTTGTTTGATGCCATAGCTATCGCAGCAGCTGAGGGGACAGATAGGGCTATAGAAGTAGTGCGAAGA------------------CTTTTTAGAGCTTTTCTCCACATACCTACAAGAATCAGACAGGGATTGGAAAGGGCTTTGTTATAA

2.SC05.Trinidad.EU576771 ATGAGAGTGAAGGAGACAAGGAGGAATTGGCAGCACTTG---------TGGAAATGGGGC------------------------ACGATGCTCCTTGGGATGTTGATGATC------------TGTAGTGCTGCA---------GGAAATTTGTGGGTCACAGTCTATTATGGGGTACCTGTGTGGAAAGAAGCGACCACCACTCTATTTTGTGCATCAGATGCTAAAGCATATGATACAGAGGTACATAAT---GTCTGGGCCACACATGCCTGTGTACCCACAGACCCCAACCCACAAGAAGTAGTATTG---GGAAATGTGACAGAAAATTTTAACATGTGGAAAAATAACATGGTAGAACAGATGCATGAGGATATAATCAGTTTATGGGATCAAAGCCTAAAGCCATGTGTAAAATTAACCCCACTCTGTGTTACTTTAAATTGTACTGATGTGAATGGTACTAGTGCTAATGTCACTAGT------------------------------------------------------------------------------------------------------ATAGAAAAAGGAGAAATAAAAAACTGCTCTTTTAATATCACC---ACAACAATAAGAGAT------AAGGTGCAGAAAGCATATGCAACTTTTTATAGCCTTGATGTAGTACCAATAGATAATGATCAAGAT------------------------AATAGTAGC---------------------AGTAGCTATAGGTTGACAAATTGTAATACCTCAGTCATTACACAGGCCTGTCCAAAGGTGTCCTTTGAACCAATTCCCATACATTATTGTGCCCCGGCTGGTTTTGCGCTTCTAAAATGT---AATGATAAAAAGTTCAATGGAACAGGACCATGTACAAATGTCAGCACAGTACAATGTACACATGGAATTAGACCAGTAGTGTCAACTCAACTGTTGTTAAATGGCAGTCTAGCAGAAGAA---GAGGTAGTAATTAGATCTGAAAATTTCACAGACAATGCTAAAACCATAATAGTACAGCTGAATAACTCTGTAGTAATTAATTGTACAAGACCCAACAATAATACAAGAAAAAGTATACCTATAGGA------------CCAGGGAGT---GTATTTTAT---ACAGGAGAAATAATAGGAGATATAAGACAAGCACATTGTAACCTT------AGTAATACAGATTGGAATAACACTTTAAAACAGGTAGTTATAAAATTAAGA---GAACAATTTGAG------------AATAAAACA---ATAGTCTTTAATCAA---------TCCGCAGGAGGGGATCCAGAAATTGTATTGCACAGTTTTAATTGTGGAGGGGAATTTTTCTACTGTAATGCAACACAACTGTTTAATAGTACTTGGAATATT---------AATGGTACTTGGAATGGTACG---------------------AATGGTACTACAGAGTCA------------AATGACACT------------------------------ATCACACTCCCATGCAGAATAAAACAAATTATAAACATGTGGCAGGAAGTAGGCAAAGCAATGTATGCCCCTCCCATTAGAGGACAGATTAGATGTTCATCAAATATTACAGGATTACTATTAACAAGAGACGGTGGTAATAAT------------AACACGAGT------------------------GACACC---GAGATCTTCAGACCTGGAGGAGGAGATATGAGGGACAATTGGAGA---AGTGAATTATATAAATATAAAGTAGTAAAAATTGAACCA---TTAGGAATAGCACCC---ACCAAGGCAAAGAGAAGAGTGGTGCAGAGAGAA---AAAAGAGCAGTG---GGAACAATAGGA---GCTATG---TTCCTT---GGG---------TTCTTGGGA---GCAGCAGGAAGCACTATGGGCGCAGCATCATTA---ACGCTGACGGTACAGGCCAGACTATTATTGTCTGGTATAGTGCAACAGCAGAACAATCTGCTGAGGGCTATTGAGGCGCAACAGCATCTGTTGCAACTCACGGTCTGGGGCATCAAACAGCTCCAGGCAAGA---GTCCTGGCTGTGGAAAGATACCTAAGGGATCAACAGCTCCTGGGGATTTGGGGTTGCTCTGGAAAACTCATTTGCACCACTACTGTGCCTTGGAATGCTAGTTGGAGT---------------------------AATAAATCTTTGAATTATATTTGGGAT---AACATGACCTGGATGCAGTGGGATAGAGAAATTAAC------AATTACACAACCCTCATATACACCTTACTTGAAGATGCGCAGAACCAGCAAGAAAAGAATGAACACGAATTATTAGAATTGGATAAGTGGGCAAGTTTGTGGAATTGGTTTGACATAACACATTGGCTGTGGTATATAAAAATATTCATAATGATAGTAGGTGGTCTGATAGGTTTAAGAATAGTTTTTGCTGTGCTTTCTATAGTGAATAGAGTTAGGCAGGGATACTCACCATTATCATTTCAGACCCAC---CTCCCAGCCAGGAGGGAA------CCCGACAGGCCCGAAGGAATCGAAGGAGAAGGTGGAGAGAAAGACAAAGACAGATCAGTTCGATTAGTGCATGGATTCTTAGCACTCATCTGGGACGACCTACGGAGCCTGTGCCTCTTCAGCTACCACCTCTTGAGAGACTTACTCTTGATTGTAACGAGGATTGTGGAAACTCTGGGACGCAGG---------------GGGTGGGAAGTCCTCAAGTATTGGTGG---AATCTCCTTCAGTATTGG---------------------------------------------------AGTCAGGAACTAAAGAATAGTGCTGTTAGCTTGTTTGATGCCATAGCTATCGCAGCAGCTGAGGGGACAGATAGGGCTATAGAAGTAGTGCGAAGA------------------CTTTTTAGAGCTTTTCTCCACATACCTACAAGAATCAGACAGGGATTGGAAAGGGCTTTGTTATAA

2.SC05.Trinidad.EU576778 ATGAGAGTGAAGGAGACAAGGAGGAATTGGCAGCACTTG---------TGGAAATGGGGC------------------------ACGATGCTCCTTGGGATGTTGATGATC------------TGTAGTGCTGCA---------GGAAATTTGTGGGTCACAGTCTATTATGGGGTACCTGTGTGGAAAGAAGCGACCACCACTCTATTTTGTGCATCAGATGCTAAAGCATATGATACAGAGGTACATAAT---GTCTGGGCCACACATGCCTGTGTACCCACAGACCCCAACCCACAAGAAGTAGTATTG---GGAAATGTGACAGAAAATTTTAACATGTGGAAAAATAACATGGTAGAACAGATGCATGAGGATATAATCAGTTTATGGGATCAAAGCCTAAAGCCATGTGTAAAATTAACCCCACTCTGTGTTACTTTAAATTGTACTGATGTGAATGGTACTAGTGCTAATGTCACTAGT------------------------------------------------------------------------------------------------------ATAGAAAAAGGAGAAATAAAAAACTGCTCTTTTAATATCACC---ACAACAATAAGAGAT------AAGGTGCAGAAAGCATATGCAACTTTTTATAGCCTTGATGTAGTACCAATAGATAATGATCAAGAT------------------------AATAGTAGC---------------------AGTAGCTATAGGTTGACAAATTGTAATACCTCAGTCATTACACAGGCCTGTCCAAAGGTGTCCTTTGAACCAATTCCCATACATTATTGTGCCCCGGCTGGTTTTGCGCTTCTAAAATGT---AATGATAAAAAGTTCAATGGAACAGGACCATGTACAAATGTCAGCACAGTACAATGTACACATGGAATTAGACCAGTAGTGTCAACTCAACTGTTGTTAAATGGCAGTCTAGCAGAAGAA---GAGGTAGTAATTAGATCTGAAAATTTCACAGACAATGCTAAAACCATAATAGTACAGCTGAATAACTCTGTAGTAATTAATTGTACAAGACCCAACAATAATACAAGAAAAAGTATACCTATAGGA------------CCAGGGAGT---GTATTTTAT---ACAGGAGAAATAATAGGAGATATAAGACAAGCACATTGTAACCTT------AGTAATACAGATTGGAATAACACTTTAAAACAGGTAGTTATAAAATTAAGA---GAACAATTTGAG------------AATAAAACA---ATAGTCTTTAATCAA---------TCCGCAGGAGGGGATCCAGAAATTGTATTGCACAGTTTTAATTGTGGAGGGGAATTTTTCTACTGTAATGCAACACAACTGTTTAATAGTACTTGGAATATT---------AATGGTACTTGGAATGGTACG---------------------AATGGTACTACAGAGTCA------------AATGACACT------------------------------ATCACACTCCCATGCAGAATAAAACAAATTATAAACATGTGGCAGGAAGTAGGCA-AGCAATGTATGCCCCTCCCATTAGAGGACAGATTAGATGTTCATCAAATATTACAGGATTACTATTAACAAGAGACGGTGGTAATAAT------------AACACGAGT------------------------GACACC---GAGATCTTCAGACCTGGAGGAGGAGATATGAGGGACAATTGGAGA---AGTGAATTATATAAATATAAAGTAGTAAAAATTGAACCA---TTAGGAATAGCACCC---ACCAAGGCAAAGAGAAGAGTGGTGCAGAGAGAA---AAAAGAGCAGTG---GGAACAATAGGA---GCTATG---TTCCTT---GGG---------TTCTTGGGA---GCAGCAGGAAGCACTATGGGCGCAGCATCATTA---ACGCTGACGGTACAGGCCAGACTATTATTGTCTGGTATAGTGCAACAGCAGAACAATCTGCTGAGGGCTATTGAGGCGCAACAGCATCTGTTGCAACTCACGGTCTGGGGCATCAAACAGCTCCAGGCAAGA---GTCCTGGCTGTGGAAAGATACCTAAGGGATCAACAGCTCCTGGGGATTTGGGGTTGCTCTGGAAAACTCATTTGCACCACTACTGTGCCTTGGAATGCTAGTTGGAGT---------------------------AATAAATCTTTGAATTATATTTGGGAT---AACATGACCTGGATGCAGTGGGATAGAGAAATTAAC------AATTACACAACCCTCATATACACCTTACTTGAAGATGCGCAGAACCAGCAAGAAAAGAATGAACACGAATTATTAGAATTGGATAAGTGGGCAAGTTTGTGGAATTGGTTTGACATAACACATTGGCTGTGGTATATAAAAATATTCATAATGATAGTAGGTGGTCTGATAGGTTTAAGAATAGTTTTTGCTGTGCTTTCTATAGTGAATAGAGTTAGGCAGGGATACTCACCATTATCATTTCAGACCCAC---CTCCCAGCCAGGAGGGAA------CCCGACAGGCCCGAAGGAATCGAAGGAGAAGGTGGAGAGAAAGACAAAGACAGATCAGTTCGATTAGTGCATGGATTCTTAGCACTCATCTGGGACGACCTACGGAGCCTGTGCCTCTTCAGCTACCACCTCTTGAGAGACTTACTCTTGATTGTAACGAGGATTGTGGAAACTCTGGGACGCAGG---------------GGGTGGGAAGTCCTCAAGTATTGGTGG---AATCTCCTTCAGTATTGG---------------------------------------------------AGTCAGGAACTAAAGAATAGTGCTGTTAGCTTGTTTGATGCCATAGCTATCGCAGCAGCTGAGGGGACAGATAGGGCTATAGAAGTAGTGCGAAGA------------------CTTTTTAGAGCTTTTCTCCACATACCTACAAGAATCAGACAGGGATTGGAAAGGGCTTTGTTATAA

2.SC05.Trinidad.EU576773 ATGAGAGTGAAGGAGACAAGGAGGAATTGGCAGCACTTG---------TGGAAATGGGGC------------------------ACGATGCTCCTTGGGATGTTGATGATC------------TGTAGTGCTGCA---------GGAAATTTGTGGGTCACAGTCTATTATGGGGTACCTGTGTGGAAAGAAGCGACCACCACTCTATTTTGTGCATCAGATGCTAAAGCATATGATACAGAGGTACATAAT---GTCTGGGCCACACATGCCTGTGTACCCACAGACCCCAACCCACAAGAAGTAGTATTG---GGAAATGTGACAGAAAATTTTAACATGTGGAAAAATAACATGGTAGAACAGATGCATGAGGATATAATCAGTTTATGGGATCAAAGCCTAAAGCCATGTGTAAAATTAACCCCACTCTGTGTTACTTTAAATTGTACTGATGTGAATGGTACTAGTGCTAATGTCACTAGT------------------------------------------------------------------------------------------------------ATAGAAAAAGGAGAAATAAAAAACTGCTCTTTTAATATCACC---ACAACAATAAGAGAT------AAGGTGCAGAAAGCATATGCAACTTTTTATAGCCTTGATGTAGTACCAATAGATAATGATCAAGAT------------------------AATAGTAGC---------------------AGTAGCTATAGGTTGACAAATTGTAATACCTCAGTCATTACACAGGCCTGTCCAAAGGTGTCCTTTGAACCAATTCCCATACATTATTGTGCCCCGGCTGGTTTTGCGCTTCTAAAATGT---AATGATAAAAAGTTCAATGGAACAGGACCATGTACAAATGTCAGCACAGTACAATGTACACATGGAATTAGACCAGTAGTGTCAACTCAACTGTTGTTAAATGGCAGTCTAGCAGAAGAA---GAGGTAGTAATTAGATCTGAAAATTTCACAGACAATGCTAAAACCATAATAGTACAGCTGAATAACTCTGTAGTAATTAATTGTACAAGACCCAACAATAATACAAGAAAAAGTATACCTATAGGA------------CCAGGGAGT---GTATTTTAT---ACAGGAGAAATAATAGGAGATATAAGACAAGCACATTGTAACCTT------AGTAATACAGATTGGAATAACACTTTAAAACAGGTAGTTATAAAATTAAGA---GAACAATTTGAG------------AATAAAACA---ATAGTCTTTAATCAA---------TCCGCAGGAGGGGATCCAGAAATTGTATTGCACAGTTTTAATTGTGGAGGGGAATTTTTCTACTGTAATGCAACACAACTGTTTAATAGCACTTGGAATATT---------AATGGTACTTGGAATGGTACG---------------------AATGGTACTACAGAGTCA------------AATGACACT------------------------------ATCACACTCCCATGCAGAATAAAACAAATTATAAACATGTGGCAGGAAGTAGGCAAAGCAATGTATGCCCCTCCCATTAGAGGACAGATTAGATGTTCATCAAATATTACAGGATTACTATTAACAAGAGACGGTGGTAATAAT------------AACACGAGT------------------------GACACC---GAGATCTTCAGACCTGGAGGAGGAGATATGAGGGACAATTGGAGA---AGTGAATTATATAAATATAAAGTAGTAAAAATTGAACCA---TTAGGAATAGCACCC---ACCAAGGCAAAGAGAAGAGTGGTGCAGAGAGAA---AAAAGAGCAGTG---GGAACAATAGGA---GCTATG---TTCCTT---GGG---------TTCTTGGGA---GCAGCAGGAAGCACTATGGGCGCAGCATCATTA---ACGCTGACGGTACAGGCCAGACTATTATTGTCTGGTATAGTGCAACAGCAGAACAATCTGCTGAGGGCTATTGAGGCGCAACAGCATCTGTTGCAACTCACGGTCTGGGGCATCAAACAGCTCCAGGCAAGA---GTCCTGGCTGTGGAAAGATACCTAAGGGATCAACAGCTCCTGGGGATTTGGGGTTGCTCTGGAAAACTCATTTGCACCACTACTGTGCCTTGGAATGCTAGTTGGAGT---------------------------AATAAATCTTTGAATTATATTTGGGAT---AACATGACCTGGATGCAGTGGGATAGAGAAATTAAC------AATTACACAACCCTCATATACACCTTACTTGAAGATGCGCAGAACCAGCAAGAAAAGAATGAACACGAATTATTAGAATTGGATAAGTGGGCAAGTTTGTGGAATTGGTTTGACATAACACATTGGCTGTGGTATATAAAAATATTCATAATGATAGTAGGTGGTCTGATAGGTTTAAGAATAGTTTTTGCTGTGCTTTCTATAGTAAATAGAGTTAGGCAGGGATACTCACCATTATCATTTCAGACCCAC---CTCCCAGCCAGGAGGGAA------CCCGACAGGCCCGAAGGAATCGAAGGAGAAGGTGGAGAGAAAGACAAAGACAGATCAGTTCGATTAGTGCATGGATTCTTAGCACTCATCTGGGACGACCTACGGAGCCTGTGCCTCTTCAGCTACCACCTCTTGAGAGACTTACTCTTGATTGTAACGAGGATTGTGGAAACTCTGGGACGCAGG---------------GGGTGGGAAGTCCTCAAGTATTGGTGG---AATCTCCTTCAGTATTGG---------------------------------------------------AGTCAGGAACTAAAGAATAGTGCTGTTAGCTTGTTTGATGCCATAGCTATCGCAGCAGCTGAGGGGACAGATAGGGCTATAGAAGTAGTGCGAAGA------------------CTTTTTAGAGCTTTTCTCCACATACCTACAAGAATCAGACAGGGATTGGAAAGGGCTTTGTTATAA

2.SC05.Trinidad.EU576785 ATGAGAGTGAAGGAGACAAGGAGGAATTGGCAGCACTTG---------TGGAAATGGGGC------------------------ACGATGCTCCTTGGGATGTTGATGATC------------TGTAGTGCTGCA---------GGAAATTTGTGGGTCACAGTCTATTATGGGGTACCTGTGTGGAAAGAAGCGACCACCACTCTATTTTGTGCATCAGATGCTAAAGCATATGATACAGAGGTACATAAT---GTCTGGGCCACACATGCCTGTGTACCCACAGACCCCAACCCACAAGAAGTAGTATTG---GGAAATGTGACAGAAAATTTTAACATGTGGAAAAATAACATGGTAGAACAGATGCATGAGGATATAATCAGTTTATGGGATCAAAGCCTAAAGCCATGTGTAAAATTAACCCCACTCTGTGTTACTTTAAATTGTACTGATGTGAATGGTACTAGTGCTAATGTCACTAGT------------------------------------------------------------------------------------------------------ATAGAAAAAGGAGAAATAAAAAACTGCTCTTTTAATATCACC---ACAACAATAAGAGAT------AAGGTGCAGAAAGCATATGCAACTTTTTATAGCCTTGATGTAGTACCAATAGATAATGATCAAGAT------------------------AATAGTAGC---------------------AGTAGCTATAGGTTGACAAATTGTAATACCTCAGTCATTACACAGGCCTGTCCAAAGGTGTCCTTTGAACCAATTCCCATACATTATTGTGCCCCGGCTGGTTTTGCGCTTCTAAAATGT---AATGATAAAAAGTTCAATGGAACAGGACCATGTACAAATGTCAGCACAGTACAATGTACACATGGAATTAGACCAGTAGTGTCAACTCAACTGTTGTTAAATGGCAGTCTAGCAGAAGAA---GAGGTAGTAATTAGATCTGAAAATTTCACAGACAATGCTAAAACCATAATAGTACAGCTGAATAACTCTGTAGTAATTAATTGTACAAGACCCAACAATAATACAAGAAAAAGTATACCTATAGGA------------CCAGGGAGT---GTATTTTAT---ACAGGAGAAATAATAGGAGATATAAGACAAGCACATTGTAACCTT------AGTAATACAGATTGGAATAACACTTTAAAACAGGTAGTTATAAAATTAAGA---GAACAATTTGAG------------AATAAAACA---ATAGTCTTTAATCAA---------TCCGCAGGAGGGGATCCAGAAATTGTATTGCACAGTTTTAATTGTGGAGGGGAATTTTTCTACTGTAATGCAACACAACTGTTTAATAGTACTTGGAATATT---------AATGGTACTTGGAATGGTACG---------------------AATGGTACTACAGAGTCA------------AATGACACT------------------------------ATCACACTCCCATGCAGAATAAAACAAATTATAAACATGTGGCAGGAAGTAGGCAAAGCAATGTATGCCCCTCCCATTAGAGGACAGATTAGATGTTCATCAAATATTACAGGATTACTATTAACAAGAGACGGTGGTAATAAT------------AACACGAGT------------------------GACACC---GAGATCTTCAGACCTGGAGGAGGAGATATGAGGGACAATTGGAGA---AGTGAATTATATAAATATAAAGTAGTAAAAATTGAACCA---TTAGGAATAGCACCC---ACCAAGGCAAAGAGAAGAGTGGTGCAGAGAGAA---AAAAGAGCAGTG---GGAACAATAGGA---GCTATG---TTCCTT---GGG---------TTCTTGGGA---GCAGCAGGAAGCACTATGGGCGCAGCATCATTA---ACGCTGACGGTACAGGCCAGACTATTATTGTCTGGTATAGTGCAACAGCAGAACAATCTGCTGAGGGCTATTGAGGCGCAACAGCATCTGTTGCAACTCACGGTCTGGGGCATCAAACAGCTCCAGGCAAGA---GTCCTGGCTGTGGAAAGATACCTAAGGGATCAACAGCTCCTGGGGATTTGGGGTTGCTCTGGAAAACTCATTTGCACCACTACTGTGCCTTGGAATGCTAGTTGGAGT---------------------------AATAAATCTTTGAATTATATTTGGGAT---AACATGACCTGGATGCAGTGGGATAGAGAAATTAAC------AATTACACAACCCTCATATACACCTTACTTGAAGATGCGCAGAACCAGCAAGAAAAGAATGAACACGAATTATTAGAATTGGATAAGTGGGCAAGTTTGTGGAATTGGTTTGACATAACACATTGGCTGTGGTATATAAAAATATTCATAATGATAGTAGGTGGTCTGATAGGTTTAAGAATAGTTTTTGCTGTGCTTTCTATAGTAAATAGAGTTAGGCAGGGATACTCACCATTATCATTTCAGACCCAC---CTCCCAGCCAGGAGGGAA------CCCGACAGGCCCGAAGGAATCGAAGGAGAAGGTGGAGAGAAAGACAAAGACAGATCAGTTCGATTAGTGCATGGATTCTTAGCACTCATCTGGGACGACCTACGGAGCCTGTGCCTCTTCAGCTACCACCTCTTGAGAGACTTACTCTTGATTGTAACGAGGATTGTGGAAACTCTGGGACGCAGG---------------GGGTGGGAAGTCCTCAAGTATTGGTGG---AATCTCCTTCAGTATTGG---------------------------------------------------AGTCAGGAACTAAAGAATAGTGCTGTTAGCTTGTTTGATGCCATAGCTATCGCAGCAGCTGAGGGGACAGATAGGGCTATAGAAGTAGTGCGAAGA------------------CTTTTTAGAGCTTTTCTCCACATACCTACAAGAATCAGACAGGGATTGGAAAGGGCTTTGTTATAA

2.SC05.Trinidad.EU576764 ATGAGAGTGAAGGAGACAAGGAGGAATTGGCAGCACTTG---------TGGAAATGGGGC------------------------ACGATGCTCCTTGGGATGTTGATGATC------------TGTAGTGCTGCA---------GGAAATTTGTGGGTCACAGTCTATTATGGGGTACCTGTGTGGAAAGAAGCGACCACCACTCTATTTTGTGCATCAGATGCTAAAGCATATGATACAGAGGTACATAAT---GTCTGGGCCACACATGCCTGTGTACCCACAGACCCCAACCCACAAGAAGTAGTATTG---GGAAATGTGACAGAAAATTTTAACATGTGGAAAAATAACATGGTAGAACAGATGCATGAGGATATAATCAGTTTATGGGATCAAAGCCTAAAGCCATGTGTAAAATTAACCCCACTCTGTGTTACTTTAAATTGTACTGATGTGAATGGTACTAGTGCTAATGTCACTAGT------------------------------------------------------------------------------------------------------ATAGAAAAAGGAGAAATAAAAAACTGCTCTTTTAATATCACC---ACAACAATAAGAGAT------AAGGTGCAGAAAGCATATGCAACTTTTTATAGCCTTGATGTAGTACCAATAGATAATGATCAAGAT------------------------AATAGTAGC---------------------AGTAGCTATAGGTTGACAAATTGTAATACCTCAGTCATTACACAGGCCTGTCCAAAGGTGTCCTTTGAACCAATTCCCATACATTATTGTGCCCCGGCTGGTTTTGCGCTTCTAAAATGT---AATGATAAAAAGTTCAATGGAACAGGACCATGTACAAATGTCAGCACAGTACAATGTACACATGGAATTAGACCAGTAGTGTCAACTCAACTGTTGTTAAATGGCAGTCTAGCAGAAGAA---GAGGTAGTAATTAGATCTGAAAATTTCACAGACAATGCTAAAACCATAATAGTACAGCTGAATAACTCTGTAGTAATTAATTGTACAAGACCCAACAATAATACAAGAAAAAGTATACCTATAGGA------------CCAGGGAGT---GTATTTTAT---ACAGGAGAAATAATAGGAGATATAAGACAAGCACATTGTAACCTT------AGTAATACAGATTGGAATAACACTTTAAAACAGGTAGTTATAAAATTAAGA---GAACAATTTGAG------------AATAAAACA---ATAGTCTTTAATCAA---------TCCGCAGGAGGGGATCCAGAAATTGTATTGCACAGTTTTAATTGTGGAGGGGAATTTTTCTACTGTAATGCAACACAACTGTTTAATAGTACTTGGAATATT---------AATGGTACTTGGAATGGTACG---------------------AATGGTACTACAGAGTCA------------AATGACACT------------------------------ATCACACTCCCATGCAGAATAAAACAAATTATAAACATGTGGCAGGAAGTAGGCAAAGCAATGTATGCCCCTCCCATTAGAGGACAGATTAGATGTTCATCAAATATTACAGGATTACTATTAACAAGAGACGGTGGTAATAAT------------AACACGAGT------------------------GACACC---GAGATCTTCAGACCTGGAGGAGGAGATATGAGGGACAATTGGAGA---AGTGAATTATATAAATATAAAGTAGTAAAAATTGAACCA---TTAGGAATAGCACCC---ACCAAGGCAAAGAGAAGAGTGGTGCAGAGAGAA---AAAAGAGCAGTG---GGAACAATAGGA---GCTATG---TTCCTT---GGG---------TTCTTGGGA---GCAGCAGGAAGCACTATGGGCGCAGCATCATTA---ACGCTGACGGTACAGGCCAGACTATTATTGTCTGGTATAGTGCAACAGCAGAACAATCTGCTGAGGGCTATTGAGGCGCAACAGCATCTGTTGCAACTCACGGTCTGGGGCATCAAACAGCTCCAGGCAAGA---GTCCTGGCTGTGGAAAGATACCTAAGGGATCAACAGCTCCTGGGGATTTGGGGTTGCTCTGGAAAACTCATTTGCACCACTACTGTGCCTTGGAATGCTAGTTGGAGT---------------------------AATAAATCTTTGAATTATATTTGGGAT---AACATGACCTGGATGCAGTGGGATAGAGAAATTAAC------AATTACACAACCCTCATATACACCTTACTTGAAGATGCGCAGAACCAGCAAGAAAAGAATGAACACGAATTATTAGAATTGGATAAGTGGGCAAGTTTGTGGAATTGGTTTGACATAACACATTGGCTGTGGTATATAAAAATATTCATAATGATAGTAGGTGGTCTGATAGGTTTAAGAATAGTTTTTGCTGTGCTTTCTATAGTAAATAGAGTTAGGCAGGGATACTCACCATTATCATTTCAGACCCAC---CTCCCAGCCAGGAGGGAA------CCCGACAGGCCCGAAGGAATCGAAGGAGAAGGTGGAGAGAAAGACAAAGACAGATCAGTTCGATTAGTGCATGGATTCTTAGCACTCATCTGGGACGACCTACGGAGCCTGTGCCTCTTCAGCTACCACCTCTTGAGAGACTTACTCTTGATTGTAACGAGGATTGTGGAAACTCTGGGACGCAGG---------------GGGTGGGAAGTCCTCAAGTATTGGTGG---AATCTCCTTCAGTATTGG---------------------------------------------------AGTCAGGAACTAAAGAATAGTGCTGTTAGCTTGTTTGATGCCATAGCTATCGCAGCAGCTGAGGGGACAGATAGGGCTATAGAAGTAGTGCGAAGA------------------CTTTTTAGAGCTTTTCTCCACATACCTACAAGAATCAGACAGGGATTGGAAAGGGCTTTGTTATAA

2.SC05.Trinidad.EU576763 ATGAGAGTGAAGGAGACAAGGAGGAATTGGCAGCACTTG---------TGGAAATGGGGC------------------------ACGATGCTCCTTGGGATGTTGATGATC------------TGTAGTGCTGCA---------GGAAATTTGTGGGTCACAGTCTATTATGGGGTACCTGTGTGGAAAGAAGCGACCACCACTCTATTTTGTGCATCAGATGCTAAAGCATATGATACAGAGGTACATAAT---GTCTGGGCCACACATGCCTGTGTACCCACAGACCCCAACCCACAAGAAGTAGTATTG---GGAAAGGTGACAGAAAATTTTAACATGTGGAAAAATAACATGGTAGAACAGATGCATGAGGATATAATCAGTTTATGGGATCAAAGCCTAAAGCCATGTGTAAAATTAACCCCACTCTGTGTTACTTTAAATTGTACTGATGTGAATGGTACTAGTGCTAATGTCACTAGT------------------------------------------------------------------------------------------------------ATAGAAAAAGGAGAAATAAAAAACTGCTCTTTTAATATCACC---ACAACAATAAGAGAT------AAGGTGCAGAAAGCATATGCAACTTTTTATAGCCTTGATGTAGTACCAATAGATAATGATCAAGAT------------------------AATAGTAGC---------------------AGTAGCTATAGGTTGACAAATTGTAATACCTCAGTCATTACACAGGCCTGTCCAAAGGTGTCCTTTGAACCAATTCCCATACATTATTGTGCCCCGGCTGGTTTTGCGCTTCTAAAATGT---AATGATAAAAAGTTCAATGGAACAGGACCATGTACAAATGTCAGCACAGTACAATGTACACATGGAATTAGACCAGTAGTGTCAACTCAACTGTTGTTAAATGGCAGTCTAGCAGAAGAA---GAGGTAGTAATTAGATCTGAAAATTTCACAGACAATGCTAAAACCATAATAGTACAGCTGAATAACTCTGTAGTAATTAATTGTACAAGACCCAACAATAATACAAGAAAAAGTATACCTATAGGA------------CCAGGGAGT---GTATTTTAT---ACAGGAGAAATAATAGGAGATATAAGACAAGCACATTGTAACCTT------AGTAATACAGATTGGAATAACACTTTAAAACAGGTAGTTATAAAATTAAGA---GAACAATTTGAG------------AATAAAACA---ATAGTCTTTAATCAA---------TCCGCAGGAGGGGATCCAGAAATTGTATTGCACAGTTTTAATTGTGGAGGGGAATTTTTCTACTGTAATGCAACACAACTGTTTAATAGTACTTGGAATATT---------AATGGTACTTGGAATGGTACG---------------------AATGGTACTACAGAGTCA------------AATGACACT------------------------------ATCACACTCCCATGCAGAATAAAACAAATTATAAACATGTGGCAGGAAGTAGGCAAAGCAATGTATGCCCCTCCCATTAGAGGACAGATTAGATGTTCATCAAATATTACAGGATTACTATTAACAAGAGACGGTGGTAATAAT------------AACACGAGT------------------------GACACC---GAGATCTTCAGACCTGGAGGAGGAGATATGAGGGACAATTGGAGA---AGTGAATTATATAAATATAAAGTAGTAAAAATTGAACCA---TTAGGAATAGCACCC---ACCAAGGCAAAGAGAAGAGTGGTGCAGAGAGAA---AAAAGAGCAGTG---GGAACAATAGGA---GCTATG---TTCCTT---GGG---------TTCTTGGGA---GCAGCAGGAAGCACTATGGGCGCAGCATCATTA---ACGCTGACGGTACAGGCCAGACTATTATTGTCTGGTATAGTGCAACAGCAGAACAATCTGCTGAGGGCTATTGAGGCGCAACAGCATCTGTTGCAACTCACGGTCTGGGGCATCAAACAGCTCCAGGCAAGA---GTCCTGGCTGTGGAAAGATACCTAAGGGATCAACAGCTCCTGGGGATTTGGGGTTGCTCTGGAAAACTCATTTGCACCACTACTGTGCCTTGGAATGCTAGTTGGAGT---------------------------AATAAATCTTTGAATTATATTTGGGAT---AACATGACCTGGATGCAGTGGGATAGAGAAATTAAC------AATTACACAACCCTCATATACACCTTACTTGAAGATGCGCAGAACCAGCAAGAAAAGAATGAACACGAATTATTAGAATTGGATAAGTGGGCAAGTTTGTGGAATTGGTTTGACATAACACATTGGCTGTGGTATATAAAAATATTCATAATGATAGTAGGTGGTCTGATAGGTTTAAGAATAGTTTTTGCTGTGCTTTCTATAGTAAATAGAGTTAGGCAGGGATACTCACCATTATCATTTCAGACCCAC---CTCCCAGCCAGGAGGGAA------CCCGACAGGCCCGAAGGAATCGAAGGAGAAGGTGGAGAGAAAGACAAAGACAGATCAGTTCGATTAGTGCATGGATTCTTAGCACTCATCTGGGACGACCTACGGAGCCTGTGCCTCTTCAGCTACCACCTCTTGAGAGACTTACTCTTGATTGTAACGAGGATTGTGGAAACTCTGGGACGCAGG---------------GGGTGGGAAGTCCTCAAGTATTGGTGG---AATCTCCTTCAGTATTGG---------------------------------------------------AGTCAGGAACTAAAGAATAGTGCTGTTAGCTTGTTTGATGCCATAGCTATCGCAGCAGCTGAGGGGACAGATAGGGCTATAGAAGTAGTGCGAAGA------------------CTTTTTAGAGCTTTTCTCCACATACCTACAAGAATCAGACAGGGATTGGAAAGGGCTTTGTTATAA

2.SC05.Trinidad.EU576760 ATGAGAGTGAAGGAGACAAGGAGGAATTGGCAGCACTTG---------TGGAAATGGGGC------------------------ACGATGCTCCTTGGGATGTTGATGATC------------TGTAGTGCTGCA---------GGAAATTTGTGGGTCACAGTCTATTATGGGGTACCTGTGTGGAAAGAAGCGACCACCACTCTATTTTGTGCATCAGATGCTAAAGCATATGATACAGAGGTACATAAT---GTCTGGGCCACACATGCCTGTGTACCCACAGACCCCAACCCACAAGAAGTAGTATTG---GGAAATGTGACAGAAAATTTTAACATGTGGAAAAATAACATGGTAGAACAGATGCATGAGGATATAATCAGTTTATGGGATCAAAGCCTAAAGCCATGTGTAAAATTAACCCCACTCTGTGTTACTTTAAATTGTACTGATGTGAATGGTACTAGTGCTAATGTCACTAGT------------------------------------------------------------------------------------------------------ATAGAAAAAGGAGAAATAAAAAACTGCTCTTTTAATATCACC---ACAACAATAAGAGAT------AAGGTGCAGAAAGCATATGCAACTTTTTATAGCCTTGATGTAGTACCAATAGATAATGATCAAGAT------------------------AATAGTAGC---------------------AGTAGCTATAGGTTGACAAATTGTAATACCTCAGTCATTACACAGGCCTGTCCAAAGGTGTCCTTTGAACCAATTCCCATACATTATTGTGCCCCGGCTGGTTTTGCGCTTCTAAAATGT---AATGATAAAAAGTTCAATGGAACAGGACCATGTACAAATGTCAGCACAGTACAATGTACACATGGAATTAGACCAGTAGTGTCAACTCAACTGTTGTTAAATGGCAGTCTAGCAGAAGAA---GAGGTAGTAATTAGATCTGAAAATTTCACAGACAATGCTAAAACCATAATAGTACAGCTGAATAACTCTGTAGTAATTAATTGTACAAGACCCAACAATAATACAAGAAAAAGTATACCTATAGGA------------CCAGGGAGT---GTATTTTAT---ACAGGAGAAATAATAGGAGATATAAGACAAGCACATTGTAACCTT------AGTAATACAGATTGGAATAACACTTTAAAACAGGTAGTTATAAAATTAAGA---GAACAATTTGAG------------AATAAAACA---ATAGTCTTTAATCAA---------TCCGCAGGAGGGGATCCAGAAATTGTATTGCACAGTTTTAATTGTGGAGGGGAATTTTTCTACTGTAATGCAACACAACTGTTTAATAGTACTTGGAATATT---------AATGGTACTTGGAATGGTACG---------------------AATGGTACTACAGAGTCA------------AATGACACT------------------------------ATCACACTCCCATGCAGAATAAAACAAATTATAAACATGTGGCAGGAAGTAGGCAAAGCAATGTATGCCCCTCCCATTAGAGGACAGATTAGATGTTCATCAAATATTACAGGATTACTATTAACAAGAGACGGTGGTAATAAT------------AACACGAGT------------------------GACACC---GAGATCTTCAGACCTGGAGGAGGAGATATGAGGGACAATTGGAGA---AGTGAATTATATAAATATAAAGTAGTAAAAATTGAACCA---TTAGGAATAGCACCC---ACCAAGGCAAAGAGAAGAGTGGTGCAGAGAGAA---AAAAGAGCAGTG---GGAACAATAGGA---GCTATG---TTCCTT---GGG---------TTCTTGGGA---GCAGCAGGAAGCACTATGGGCGCAGCATCATTA---ACGCTGACGGTACAGGCCAGACTATTATTGTCTGGTATAGTGCAACAGCAGAACAATCTGCTGAGGGCTATTGAGGCGCAACAGCATCTGTTGCAACTCACGGTCTGGGGCATCAAACAGCTCCAGGCAAGA---GTCCTGGCTGTGGAAAGATACCTAAGGGATCAACAGCTCCTGGGGATTTGGGGTTGCTCTGGAAAACTCATTTGCACCACTACTGTGCCTTGGAATGCTAGTTGGAGT---------------------------AATAAATCTTTGAATTATATTTGGGAT---AACATGACCTGGATGCAGTGGGATAGAGAAATTAAC------AATTACACAACCCTCATATACACCTTACTTGAAGATGCGCAGAACCAGCAAGAAAAGAATGAACACGAATTATTAGAATTGGATAAGTGGGCAAGTTTGTGGAATTGGTTTGACATAACACATTGGCTGTGGTATATAAAAATATTCATAATGATAGTAGGTGGTCTGATAGGTTTAAGAATAGTTTTTGCTGTGCTTTCTATAGTGAATAGAGTTAGGCAGGGATACTCACCATTATCATTTCAGACCCAC---CTCCCAGCCAGGAGGGAA------CCCGACAGGCCCGAAGGAATCGAAGGAGAAGGTGGAGAGAAAGACAAAGACAGATCAGTTCGATTAGTGCATGGATTCTTAGCACTCATCTGGGACGACCTACGGAGCCTGTGCCTCTTCAGCTACCACCTCTTGAGAGACTTACTCTTGATTGTAACGAGGATTGTGGAAACTCTGGGACGCAGG---------------GGGTGGGAAGTCCTCAAGTATTGGTGG---AATCTCCTTCAGTATTGG---------------------------------------------------AGTCAGGAACTGAAGAATAGTGCTGTTAGCTTGTTTGATGCCATAGCTATCGCAGCAGCTGAGGGGACAGATAGGGCTATAGAAGTAGTGCGAAGA------------------CTTTTTAGAGCTTTTCTCCACATACCTACAAGAATCAGACAGGGATTGGAAAGGGCTTTGTTATAA

2.SC05.Trinidad.EU576786 ATGAGAGTGAAGGAGACAAGGAGGAATTGGCAGCACTTG---------TGGAAATGGGGC------------------------ACGATGCTCCTTGGGATGTTGATGATC------------TGTAGTGCTGCA---------GGAAATTTGTGGGTCACAGTCTATTATGGGGTACCTGTGTGGAAAGAAGCGACCACCACTCTATTTTGTGCATCAGATGCTAAAGCATATGATACAGAGGTACATAAT---GTCTGGGCCACACATGCCTGTGTACCCACAGACCCCAACCCACAAGAAGTAGTATTG---GGAAATGTGGCAGAAAATTTTAACATGTGGAAAAATAACATGGTAGAACAGATGCATGAGGATATAATCAGTTTATGGGATCAAAGCCTAAAGCCATGTGTAAAATTAACCCCACTCTGTGTTACTTTAAATTGTACTGATGTGAATGGTACTAGTGCTAATGTCACTAGT------------------------------------------------------------------------------------------------------ATAGAAAAAGGAGAAATAAAAAACTGCTCTTTTAATATCACC---ACAACAATAAGAGAT------AAGGTGCAGAAAGCATATGCAACTTTTTATAGCCTTGATGTAGTACCAATAGATAATGATCAAGAT------------------------AATAGTAGC---------------------AGTAGCTATAGGTTGACAAATTGTAATACCTCAGTCATTACACAGGCCTGTCCAAAGGTGTCCTTTGAACCAATTCCCATACATTATTGTGCCCCGGCTGGTTTTGCGCTTCTAAAATGT---AATGATAAAAAGTTCAATGGAACAGGACCATGTACAAATGTCAGCACAGTACAATGTACACATGGAATTAGACCAGTAGTGTCAACTCAACTGTTGTTAAATGGCAGTCTAGCAGAAGAA---GAGGTAGTAATTAGATCTGAAAATTTCACAGACAATGCTAAAACCATAATAGTACAGCTGAATAACTCTGTAGTAATTAATTGTACAAGACCCAACAATAATACAAGAAAAAGTATACCTATAGGA------------CCAGGGAGT---GTATTTTAT---ACAGGAGAAATAATAGGAGATATAAGACAAGCACATTGTAACCTT------AGTAATACAGATTGGAATAACACTTTAAAACAGGTAGTTATAAAATTAAGA---GAACAATTTGAG------------AATAAAACA---ATAGTCTTTAATCAA---------TCCGCAGGAGGGGATCCAGAAATTGTATTGCACAGTTTTAATTGTGGAGGGGAATTTTTCTACTGTAATGCAACACAACTGTTTAATAGTACTTGGAATATT---------AATGGTACTTGGAATGGTACG---------------------AATGGTACTACAGAGTCA------------AATGACACT------------------------------ATCACACTCCCATGCAGAATAAAACAAATTATAAACATGTGGCAGGAAGTAGGCAAAGCAATGTATGCCCCTCCCATTAGAGGACAGATTAGATGTTCATCAAATATTACAGGATTACTATTAACAAGAGACGGTGGTAATAAT------------AACACGAGT------------------------GACACC---GAGATCTTCAGACCTGGAGGAGGAGATATGAGGGACAATTGGAGA---AGTGAATTATATAAATATAAAGTAGTAAAAATTGAACCA---TTAGGAATAGCACCC---ACCAAGGCAAAGAGAAGAGTGGTGCAGAGAGAA---AAAAGAGCAGTG---GGAACAATAGGA---GCTATG---TTCCTT---GGG---------TTCTTGGGA---GCAGCAGGAAGCACTATGGGCGCAGCATCATTA---ACGCTGACGGTACAGGCCAGACTATTATTGTCTGGTATAGTGCAACAGCAGAACAATCTGCTGAGGGCTATTGAGGCGCAACAGCATCTGTTGCAACTCACGGTCTGGGGCATCAAACAGCTCCAGGCAAGA---GTCCTGGCTGTGGAAAGATACCTAAGGGATCAACAGCTCCTGGGGATTTGGGGTTGCTCTGGAAAACTCATTTGCACCACTACTGTGCCTTGGAATGCTAGTTGGAGT---------------------------AATAAATCTTTGAATTATATTTGGGAT---AACATGACCTGGATGCAGTGGGATAGAGAAATTAAC------AATTACACAACCCTCATATACACCTTACTTGAAGATGCGCAGAACCAGCAAGAAAAGAATGAACACGAATTATTAGAATTGGATAAGTGGGCAAGTTTGTGGAATTGGTTTGACATAACACATTGGCTGTGGTATATAAAAATATTCATAATGATAGTAGGTGGTCTGATAGGTTTAAGAATAGTTTTTGCTGTGCTTTCTATAGTGAATAGAGTTAGGCAGGGATACTCACCATTATCATTTCAGACCCAC---CTCCCAGCCAGGAGGGAA------CCCGACAGGCCCGAAGGAATCGAAGGAGAAGGTGGAGAGAAAGACAAAGACAGATCAGTTCGATTAGTGCATGGATTCTTAGCACTCATCTGGGACGACCTACGGAGCCTGTGCCTCTTCAGCTACCACCTCTTGAGAGACTTACTCTTGATTGTAACGAGGATTGTGGAAACTCTGGGACGCAGG---------------GGGTGGGAAGTCCTCAAGTATTGGTGG---AATCTCCTTCAGTATTGG---------------------------------------------------AGTCAGGAACTAAAGAATAGTGCTGTTAGCTTGTTTGATGCCATAGCTATCGCAGCAGCTGAGGGGACAGATAGGGCTATAGAAGTAGTGCGAAGA------------------CTTTTTAGAGCTTTTCTCCACATACCTACAAGAATCAGACAGGGATTGGAAAGGGCTTTGTTATAA

2.SC05.Trinidad.EU576762 ATGAGAGTGAAGGAGACAAGGAGGAATTGGCAGCACTTG---------TGGAAATGGGGC------------------------ACGATGCTCCTTGGGATGTTGATGATC------------TGTAGTGCTGCA---------GGAAATTTGTGGGTCACAGTCTATTATGGGGTACCTGTGTGGAAAGAAGCGACCACCACTCTATTTTGTGCATCAGATGCTAAAGCATATGATACAGAGGTACATAAT---GTCTGGGCCACACATGCCTGTGTACCCACAGACCCCAACCCACAAGAAGTAGTATTG---GGAAATGTGACAGAAAATTTTAACATGTGGAAAAATAACATGGTAGAACAGATGCATGAGGATATAATCAGTTTATGGGATCAAAGCCTAAAGCCATGTGTAAAATTAACCCCACTCTGTGTTACTTTAAATTGTACTGATGTGAATGGTACTAGTGCTAATGTCACTAGT------------------------------------------------------------------------------------------------------ATAGAAAAAGGAGAAATAAAAAACTGCTCTTTTAATATCACC---ACAACAATAAGAGAT------AAGGTGCAGAAAGCATATGCAACTTTTTATAGCCTTGATGTAGTACCAATAGATAATGATCAAGAT------------------------AATAGTAGC---------------------AGTAGCTATAGGTTGACAAATTGTAATACCTCAGTCATTACACAGGCCTGTCCAAAGGTGTCCTTTGAACCAATTCCCATACATTATTGTGCCCCGGCTGGTTTTGCGCTTCTAAAATGT---AATGATAAAAAGTTCAATGGAACAGGACCATGTACAAATGTCAGCACAGTACAATGTACACATGGAATTAGACCAGTAGTGTCAACTCAACTGTTGTTAAATGGCAGTCTAGCAGAAGAA---GAGGTAGTAATTAGATCTGAAAATTTCACAGACAATGCTAAAACCATAATAGTACAGCTGAATAACTCTGTAGTAATTAATTGTACAAGACCCAACAATAATACAAGAAAAAGTATACCTATAGGA------------CCAGGGAGT---GTATTTTAT---ACAGGAGAAATAATAGGAGATATAAGACAAGCACATTGTAACCTT------AGTAATACAGATTGGAATAACACTTTAAAACAGGTAGTTATAAAATTAAGA---GAACAATTTGAG------------AATAAAACA---ATAGTCTTTAATCAA---------TCCGCAGGAGGGGATCCAGAAATTGTATTGCACAGTTTTAATTGTGGAGGGGAATTTTTCTACTGTAATGCAACACAACTGTTTAATAGTACTTGGAATATT---------AATGGTACTTGGAATGGTACG---------------------AATGGTACTACAGAGTCA------------AATGACACT------------------------------ATCACACTCCCATGCAGAATAAAACAAATTATAAACATGTGGCAGGAAGTAGGCAAAGCAATGTATGCCCCTCCCATTAGAGGACAGATTAGATGTTCATCAAATATTACAGGATTACTATTAACAAGAGACGGTGGTAATAAT------------AACACGAGT------------------------GACACC---GAGATCTTCAGACCTGGAGGAGGAGATATGAGGGACAATTGGAGA---AGTGAATTATATAAATATAAAGTAGTAAAAATTGAACCA---TTAGGAATAGCACCC---ACCAAGGCAAAGAGAAGAGTGGTGCAGAGAGAA---AAAAGAGCAGTG---GGAACAATAGGA---GCTATG---TTCCTT---GGG---------TTCTTGGGA---GCAGCAGGAAGCACTATGGGCGCAGCATCATTA---ACGCTGACGGTACAGGCCAGACTATTATTGTCTGGTATAGTGCAACAGCAGAACAATCTGCTGAGGGCTATTGAGGCGCAACAGCATCTGTTGCAACTCACGGTCTGGGGCATCAAACAGCTCCAGGCAAGA---GTCCTGGCTGTGGAAAGATACCTAAGGGATCAACAGCTCCTGGGGATTTGGGGTTGCTCTGGAAAACTCATTTGCACCACTACTGTGCCTTGGAATGCTAGTTGGAGT---------------------------AATAAATCTTTGAATTATATTTGGGAT---AACATGACCTGGATGCAGTGGGATAGAGAAATTAAC------AATTACACAACCCTCATATACACCTTACTTGAAGATGCGCAGAACCAGCAAGAAAAGAATGAACACGAATTATTAGAATTGGATAAGTGGGCAAGTTTGTGGAATTGGTTTGACATAACACATTGGCTGTGGTATATAAAAATATTCATAATGATAGTAGGTGGTCTGATAGGTTTAAGAATAGTTTTTGCTGTGCTTTCTATAGTGAATAGAGTTAGGCAGGGATACTCACCATTATCATTTCAGACCCAC---CTCCCAGCCAGGAGGGAA------CCCGACAGGCCCGAAGGAATCGAAGGAGAAGGTGGAGAGAAAGACAAAGACAGATCAGTTCGATTAGTGCATGGATTCTTAGCACTCATCTGGGACGACCTACGGAGCCTGTGCCTCTTCAGCTACCACCTCTTGAGAGACTTACTCTTGATTGTAACGAGGATTGTGGAAACTCTGGGACGCAGG---------------GGGTGGGAAGTCCTCAAGTATTGGTGG---AATCTCCTTCAGTATTGG---------------------------------------------------AGTCAGGAACTAAAGAATAGTGCTGTTAGCTTGTTTGATGCCATAGCTATCGCAGCAGCTGAGGGGACAGATAGGGCTATAGAAGTAGTGCGAAGA------------------CTTTTTAGAGCTTTTCTCCACATACCTACAAGAATCAGACAGGGATTGGAAAGGGCTTTGTTATAA

2.SC05.Trinidad.EU576787 ATGAGAGTGAAGGAGACAAGGAGGAATTGGCAGCACTTG---------TGGAAATGGGGC------------------------ACGATGCTCCTTGGGATGTTGATGATC------------TGTAGTGCTGCA---------GGAAATTTGTGGGTCACAGTCTATTATGGGGTACCTGTGTGGAAAGAAGCGACCACCACTCTATTTTGTGCATCAGATGCTAAAGCATATGATACAGAGGTACATAAT---GTCTGGGCCACACATGCCTGTGTACCCACAGACCCCAACCCACAAGAAGTAGTATTG---GGAAATGTGACAGAAAATTTTAACATGTGGAAAAATAACATGGTAGAACAGATGCATGAGGATATAATCAGTTTATGGGATCAAAGCCTAAAGCCATGTGTAAAATTAACCCCACTCTGTGTTACTTTAAATTGTACTGATGTGAATGGTACTAGTGCTAATGTCACTAGT------------------------------------------------------------------------------------------------------ATAGAAAAAGGAGAAATAAAAAACTGCTCTTTTAATATCACC---ACAACAATAAGAGAT------AAGGTGCAGAAAGCATATGCAACTTTTTATAGCCTTGATGTAGTACCAATAGATAATGATCAAGAT------------------------AATAGTAGC---------------------AGTAGCTATAGGTTGACAAATTGTAATACCTCAGTCATTACACAGGCCTGTCCAAAGGTGTCCTTTGAACCAATTCCCATACATTATTGTGCCCCGGCTGGTTTTGCGCTTCTAAAATGT---AATGATAAAAAGTTCAATGGAACAGGACCATGTACAAATGTCAGCACAGTACAATGTACACATGGAATTAGACCAGTAGTGTCAACTCAACTGTTGTTAAATGGCAGTCTAGCAGAAGAA---GAGGTAGTAATTAGATCTGAAAATTTCACAGACAATGCTAAAACCATAATAGTACAGCTGAATAACTCTGTAGTAATTAATTGTACAAGACCCAACAATAATACAAGAAAAAGTATACCTATAGGA------------CCAGGGAGT---GTATTTTAT---ACAGGAGAAATAATAGGAGATATAAGACAAGCACATTGTAACCTT------AGTAATACAGATTGGAATAACACTTTAAAACAGGTAGTTATAAAATTAAGA---GAACAATTTGAG------------AATAAAACA---ATAGTCTTTAATCAA---------TCCGCAGGAGGGGATCCAGAAATTGTATTGCACAGTTTTAATTGTGGAGGGGAATTTTTCTACTGTAATGCAACACAACTGTTTAATAGTACTTGGAATATT---------AATGGTACTTGGAATGGTACG---------------------AATGGTACTACAGAGTCA------------AATGACACT------------------------------ATCACACTCCCATGCAGAATAAAACAAATTATAAACATGTGGCAGGAAGTAGGCAAAGCAATGTATGCCCCTCCCATTAGAGGACAGATTAGATGTTCATCAAATATTACAGGATTACTATTAACAAGAGACGGTGGTAATAAT------------AACACGAGT------------------------GACACC---GAGATCTTCAGACCTGGAGGAGGAGATATGAGGGACAATTGGAGA---AGTGAATTATATAAATATAAAGTAGTAAAAATTGAACCA---TTAGGAATAGCACCC---ACCAAGGCAAAGAGAAGAGTGGTGCAGAGAGAA---AAAAGAGCAGTG---GGAACAATAGGA---GCTATG---TTCCTT---GGG---------TTCTTGGGA---GCAGCAGGAAGCACTATGGGCGCAGCATCATTA---ACGCTGACGGTACAGGCCAGACTATTATTGTCTGGTATAGTGCAACAGCAGAACAATCTGCTGAGGGCTATTGAGGCGCAACAGCATCTGTTGCAACTCACGGTCTGGGGCATCAAACAGCTCCAGGCAAGA---GTCCTGGCTGTGGAAAGATACCTAAGGGATCAACAGCTCCTGGGGATTTGGGGTTGCTCTGGAAAACTCATTTGCACCACTACTGTGCCTTGGAATGCTAGTTGGAGT---------------------------AATAAATCTTTGAATTATATTTGGGAT---AACATGACCTGGATGCAGTGGGATAGAGAAATTAAC------AATTACACAACCCTCATATACACCTTACTTGAAGATGCGCAGAACCAGCAAGAAAAGAATGAACACGAATTATTAGAATTGGATAAGTGGGCAAGTTTGTGGAATTGGTTTGACATAACACATTGGCTGTGGTATATAAAAATATTCATAATGATAGTAGGTGGTCTGATAGGTTTAAGAATAGTTTTTGCTGTGCTTTCTATAGTGAATAGAGTTAGGCAGGGATACTCACCATTATCATTTCAGACCCAC---CTCCCAGCCAGGAGGGAA------CCCGACAGGCCCGAAGGAATCGAAGGAGAAGGTGGAGAGAAAGACAAAGACAGATCAGTTCGATTAGTGCATGGATTCTTAGCACTCATCTGGGACGACCTACGGAGCCTGTGCCTCTTCAGCTACCACCTCTTGAGAGACTTACTCTTGATTGTAACGAGGATTGTGGAAACTCTGGGACGCAGG---------------GGGTGGGAAGTCCTCAAGTATTGGTGG---AATCTCCTTCAGTATTGG---------------------------------------------------AGTCAGGAACTAAAGAATAGTGCTGTTAGCTTGTTTGATGCCATAGCTATCGCAGCAGCTGAGGGGACAGATAGGGCTATAGAAGTAGTGCGAAGA------------------CTTTTTAGAGCTTTTCTCCACATACCTACAAGAATCAGACAGGGATTGGAAAGGGCTTTGTTATAA

2.SC05.Trinidad.EU576779 ATGAGAGTGAAGGAGACAAGGAGGAATTGGCAGCACTTG---------TGGAAATGGGGC------------------------ACGATGCTCCTTGGGATGTTGATGATC------------TGTAGTGCTGCA---------GGAAATTTGTGGGTCACAGTCTATTATGGGGTACCTGTGTGGAAAGAAGCGACCACCACTCTATTTTGTGCATCAGATGCTAAAGCATATGATACAGAGGTACATAAT---GTCTGGGCCACACATGCCTGTGTACCCACAGACCCCAACCCACAAGAAGTAGTATTG---GGAAATGTGACAGAAAATTTTAACATGTGGAAAAATAACATGGTAGAACAGATGCATGAGGATATAATCAGTTTATGGGATCAAAGCCTAAAGCCATGTGTAAAATTAACCCCACTCTGTGTTACTTTAAATTGTACTGATGTGAATGGTACTAGTGCTAATGTCACTAGT------------------------------------------------------------------------------------------------------ATAGAAAAAGGAGAAATAAAAAACTGCTCTTTTAATATCACC---ACAACAATAAGAGAT------AAGGTGCAGAAAGCATATGCAACTTTTTATAGCCTTGATGTAGTACCAATAGATAATGATCAAGAT------------------------AATAGTAGC---------------------AGTAGCTATAGGTTGACAAATTGTAATACCTCAGTCATTACACAGGCCTGTCCAAAGGTGTCCTTTGAACCACTTCCCATACATTATTGTGCCCCGGCTGGTTTTGCGCTTCTAAAATGT---AATGATAAAAAGTTCAATGGAACAGGACCATGTACAAATGTCAGCACAGTACAATGTACACATGGAATTAGACCAGTAGTGTCAACTCAACTGTTGTTAAATGGCAGTCTAGCAGAAGAA---GAGGTAGTAATTAGATCTGAAAATTTCACAGACAATGCTAAAACCATAATAGTACAGCTGAATAACTCTGTAGTAATTAATTGTACAAGACCCAACAATAATACAAGAAAAAGTATACCTATAGGA------------CCAGGGAGT---GTATTTTAT---ACAGGAGAAATAATAGGAGATATAAGACAAGCACATTGTAACCTT------AGTAATACAGATTGGAATAACACTTTAAAACAGGTAGTTATAAAATTAAGA---GAACAATTTGAG------------AATAAAACA---ATAGTCTTTAATCAA---------TCCGCAGGAGGGGATCCAGAAATTGTATTGCACAGTTTTAATTGTGGAGGGGAATTTTTCTACTGTAATGCAACACAACTGTTTAATAGTACTTGGAATATT---------AATGGTACTTGGAATGGTACG---------------------AATGGTACTACAGAGTCA------------AATGACACT------------------------------ATCACACTCCCATGCAGAATAAAACAAATTATAAACATGTGGCAGGAAGTAGGCAAAGCAATGTATGCCCCTCCCATTAGAGGACAGATTAGATGTTCATCAAATATTACAGGATTACTATTAACAAGAGACGGTGGTAATAAT------------AACACGAGT------------------------GACACC---GAGATCTTCAGACCTGGAGGAGGAGATATGAGGGACAATTGGAGA---AGTGAATTATATAAATATAAAGTAGTAAAAATTGAACCA---TTAGGAATAGCACCC---ACCAAGGCAAAGAGAAGAGTGGTGCAGAGAGAA---AAAAGAGCAGTG---GGAACAATAGGA---GCTATG---TTCCTT---GGG---------TTCTTGGGA---GCAGCAGGAAGCACTATGGGCGCAGCATCATTA---ACGCTGACGGTACAGGCCAGACTATTATTGTCTGGTATAGTGCAACAGCAGAACAATCTGCTGAGGGCTATTGAGGCGCAACAGCATCTGTTGCAACTCACGGTCTGGGGCATCAAACAGCTCCAGGCAAGA---GTCCTGGCTGTGGAAAGATACCTAAGGGATCAACAGCTCCTGGGGATTTGGGGTTGCTCTGGAAAACTCATTTGCACCACTACTGTGCCTTGGAATGCTAGTTGGAGT---------------------------AATAAATCTTTGAATTATATTTGGGAT---AACATGACCTGGATGCAGTGGGATAGAGAAATTAAC------AATTACACAACCCTCATATACACCTTACTTGAAGATGCGCAGAACCAGCAAGAAAAGAATGAACACGAATTATTAGAATTGGATAAGTGGGCAAGTTTGTGGAATTGGTTTGACATAACACATTGGCTGTGGTATATAAAAATATTCATAATGATAGTAGGTGGTCTAATAGGTTTAAGAATAGTTTTTGCTGTGCTTTCTATAGTGAATAGAGTTAGGCAGGGATACTCACCATTATCATTTCAGACCCAC---CTCCCAGCCAGGAGGGAA------CCCGACAGGCCCGAAGGAATCGAAGGAGAAGGTGGAGAGAAAGACAAAGACAGATCAGTTCGATTAGTGCATGGATTCTTAGCACTCATCTGGGACGACCTACGGAGCCTGTGCCTCTTCAGCTACCACCTCTTGAGAGACTTACTCTTGATTGTAACGAGGATTGTGGAAACTCTGGGACGCAGG---------------GGGTGGGAAGTCCTCAAGTATTGGTGG---AATCTCCTTCAGTATTGG---------------------------------------------------AGTCAGGAACTAAAGAATAGTGCTGTTAGCTTGTTTGATGCCATAGCTATCGCAGCAGCTGAGGGGACAGATAGGGCTATAGAAGTAGTGCGAAGA------------------CTTTTTAGAGCTTTTCTCCACATACCTACAAGAATCAGACAGGGATTGGAAAGGGCTTTGTTATAA

2.SC05.Trinidad.EU576772 ATGAGAGTGAAGGAGACAAGGAGGAATTGGCAGCACTTG---------TGGAAATGGGGC------------------------ACGATGCTCCTTGGGATGTTGATGATC------------TGTAGTGCTGCA---------GGAAATTTGTGGGTCACAGTCTATTATGGGGTACCTGTGTGGAAAGAAGCGACCACCACTCTATTTTGTGCATCAGATGCTAAAGCATATGATACAGAGGTACATAAT---GTCTGGGCCACACATGCCTGTGTACCCACAGACCCCAACCCACAAGAAGTAGTATTG---GGAAATGTGACAGAAAATTTTAACATGTGGAAAAATAACATGGTAGAACAGATGCATGAGGATATAATCAGTTTATGGGATCAAAGCCTAAAGCCATGTGTAAAATTAACCCCACTCTGTGTTACTTTAAATTGTACTGATGTGAATGGTACTAGTGCTAATGTCACTAGT------------------------------------------------------------------------------------------------------ATAGAAAAAGGAGAAATAAAAAACTGCTCTTTTAATATCACC---ACAACAATAAGAGAT------AAGGTGCAGAAAGCATATGCAACTTTTTATAGCCTTGATGTAGTACCAATAGATAATGATCAAGAT------------------------AATAGTAGC---------------------AGTAGCTATAGGTTGACAAATTGTAATACCTCAGTCATTACACAGGCCTGTCCAAAGGTGTCCTTTGAACCAATTCCCATACATTATTGTGCCCCGGCTGGTTTTGCGCTTCTAAAATGT---AATGATAAAAAGTTCAATGGAACAGGACCATGTACAAATGTCAGCACAGTACAATGTACACATGGAATTAGACCAGTAGTGTCAACTCAACTGTTGTTAAATGGCAGTCTAGCAGAAGAA---GAGGTAGTAATTAGATCTGAAAATTTCACAGACAATGCTAAAACCATAATAGTACAGCTGAATAACTCTGTAGTAATTAATTGTACAAGACCCAACAATAATACAAGAAAAAGTATACCTATAGGA------------CCAGGGAGT---GTATTTTAT---ACAGGAGAAATAATAGGAGATATAAGACAAGCACATTGTAACCTT------AGTAATACAGATTGGAATAACACTTTAAAACAGGTAGTTATAAAATTAAGA---GAACAATTTGAG------------AATAAAACA---ATAGTCTTTAATCAA---------TCCGCAGGAGGGGATCCAGAAATTGTATTGCACAGTTTTAATTGTGGAGGGGAATTTTTCTACTGTAATGCAACACAACTGTTTAATAGTACTTGGAATATT---------AATGGTACTTGGAATGGTACG---------------------AATGGTACTACAGAGTCA------------AATGACACT------------------------------ATCACACTCCCATGCAGAATAAAACAAATTATAAACATGTGGCAGGAAGTAGGCAAAGCAATGTATGCCCCTCCCATTAGAGGACAGATTAGATGTTCATCAAATATTACAGGATTACTATTAACAAGAGACGGTGGTAATAAT------------AACACGAGT------------------------GACACC---GAGATCTTCAGACCTGGAGGAGGAGATATGAGGGACAATTGGAGA---AGTGAATTATATAAATATAAAGTAGTAAAAATTGAACCA---TTAGGAATAGCACCC---ACCAAGGCAAAGAGAAGAGTGGTGCAGAGAGAA---AAAAGAGCAGTG---GGAACAATAGGA---GCTATG---TTCCTT---GGG---------TTCTTGGGA---GCAGCAGGAAGCACTATGGGCGCAGCATCATTA---ACGCTGACGGTACAGGCCAGACTATTATTGTCTGGTATAGTGCAACAGCAGAACAATCTGCTGAGGGCTATTGAGGCGCAACAGCATCTGTTGCAACTCACGGTCTGGGGCATCAAACAGCTCCAGGCAAGA---GTCCTGGCTGTGGAAAGATACCTAAGGGATCAACAGCTCCTGGGGATTTGGGGTTGCTCTGGAAAACTCATTTGCACCACTACTGTGCCTTGGAATGCTAGTTGGAGT---------------------------AATAAATCTTTGAATTATATTTGGGAT---AACATGACCTGGATGCAGTGGGATAGAGAAATTAAC------AATTACACAACCCTCATATACACCTTACTTGAAGATGCGCAGAACCAGCAAGAAAAGAATGAACACGAATTATTAGAATTGGATAAGTGGGCAAGTTTGTGGAATTGGTTTGACATAACACATTGGCTGTGGTATATAAAAATATTCATAATGATAGTAGGTGGTCTGATAGGTTTAAGAATAGTTTTTGCTGTGCTTTCTATAGTGAATAGAGTTAGGCAGGGATACTCACCATTATCATTTCAGACCCAC---CTCCCAGCCAGGAGGGAA------CCCGACAGGCCCGAAGGAATCGAAGGAGAAGGTGGAGAGAAAGACAAAGACAGATCAGTTCGATTAGTGCATGGATTCTTAGCACTCATCTGGGACGACCTACGGAGCCTGTGCCTCTTCAGCTACCACCTCTTGAGAGACTTACTCTTGATTGTAACGAGGATTGTGGAAACTCTGGGACGCAGG---------------GGGTGGGAAGTCCTCAAGTATTGGTGG---AATCTCCTTCAGTATTGG---------------------------------------------------AGTCAGGAACTGAAGAATAGTGCTGTTAGCTTGTTTGATGCCATAGCTATCGCAGCAGCTGAGGGGACAGATAGGGCTATAGAAGTAGTGCGAAGA------------------CTTTTTAGAGCTTTTCTCCACATACCTACAAGAATCAGACAGGGATTGGAAAGGGCTTTGTTATAA

2.SC05.Trinidad.EU576768 ATGAGAGTGAAGGAGACAAGGAGGAATTGGCAGCACTTG---------TGGAAATGGGGC------------------------ACGATGCTCCTTGGGATGTTGATGATC------------TGTAGTGCTGCA---------GGAAATTTGTGGGTCACAGTCTATTATGGGGTACCTGTGTGGAAAGAAGCGACCACCACTCTATTTTGTGCATCAGATGCTAAAGCATATGATACAGAGGTACATAAT---GTCTGGGCCACACATGCCTGTGTACCCACAGACCCCAACCCACAAGAAGTAGTATTG---GGAAATGTGACAGAAAATTTTAACATGTGGAAAAATAACATGGTAGAACAGATGCATGAGGATATAATCAGTTTATGGGATCAAAGCCTAAAGCCATGTGTAAAATTAACCCCACTCTGTGTTACTTTAAATTGTACTGATGTGAATGGTACTAGTGCTAATGTCACTAGT------------------------------------------------------------------------------------------------------ATAGAAAAAGGAGAAATAAAAAACTGCTCTTTTAATATCACC---ACAACAATAAGAGAT------AAGGTGCAGAAAGCATATGCAACTTTTTATAGCCTTGATGTAGTACCAATAGATAATGATCAAGAT------------------------AATAGTAGC---------------------AGTAGCTATAGGTTGACAAATTGTAATACCTCAGTCATTACACAGGCCTGTCCAAAGGTGTCCTTTGAACCAATTCCCATACATTATTGTGCCCCGGCTGGTTTTGCGCTTCTAAAATGT---AATGATAAAAAGTTCAATGGAACAGGACCATGTACAAATGTCAGCACAGTACAATGTACACATGGAATTAGACCAGTAGTGTCAACTCAACTGTTGTTAAATGGCAGTCTAGCAGAAGAA---GAGGTAGTAATTAGATCTGAAAATTTCACAGACAATGCTAAAACCATAATAGTACAGCTGAATAACTCTGTAGTAATTAATTGTACAAGACCCAACAATAATACAAGAAAAAGTATACCTATAGGA------------CCAGGGAGT---GTATTTTAT---ACAGGAGAAATAATAGGAGATATAAGACAAGCACATTGTAACCTT------AGTAATACAGATTGGAATAACACTTTAAAACAGGTAGTTATAAAATTAAGA---GAACAATTTGAG------------AATAAAACA---ATAGTCTTTAATCAA---------TCCGCAGGAGGGGATCCAGAAATTGTATTGCACAGTTTTAATTGTGGAGGGGAATTTTTCTACTGTAATGCAACACAACTGTTTAATAGTACTTGGAATATT---------AATGGTACTTGGAATGGTACG---------------------AATGGTACTACAGAGTCA------------AATGACACT------------------------------ATCACACTCCCATGCAGAATAAAACAAATTATAAACATGTGGCAGGAAGTAGGCAAAGCAATGTATGCCCCTCCCATTAGAGGACAGATTAGATGTTCATCAAATATTACAGGATTACTATTAACAAGAGACGGTGGTAATAAT------------AACACGAGT------------------------GACACC---GAGATCTTCAGACCTGGAGGAGGAGATATGAGGGACAATTGGAGA---AGTGAATTATATAAATATAAAGTAGTAAAAATTGAACCA---TTAGGAATAGCACCC---ACCAAGGCAAAGAGAAGAGTGGTGCAGAGAGAA---AAAAGAGCAGTG---GGAACAATAGGA---GCTATG---TTCCTT---GGG---------TTCTTGGGA---GCAGCAGGAAGCACTATGGGCGCAGCATCATTA---ACGCTGACGGTACAGGCCAGACTATTATTGTCTGGTATAGTGCAACAGCAGAACAATCTGCTGAGGGCTATTGAGGCGCAACAGCATCTGTTGCAACTCACGGTCTGGGGCATCAAACAGCTCCAGGCAAGA---GTCCTGGCTGTGGAAAGATACCTAAGGGATCAACAGCTCCTGGGGATTTGGGGTTGCTCTGGAAAACTCATTTGCACCACTACTGTGCCTTGGAATGCTAGTTGGAGT---------------------------AATAAATCTTTGAATTATATTTGGGAT---AACATGACCTGGATGCAGTGGGATAGAGAAATTAAC------AATTACACAACCCTCATATACACCTTACTTGAAGATGCGCAGAACCAGCAAGAAAAGAATGAACACGAATTATTAGAATTGGATAAGTGGGCAAGTTTGTGGAATTGGTTTGACATAACACATTGGCTGTGGTATATAAAAATATTCATAATGATAGTAGGTGGTCTGATAGGTTTAAGAATAGTTTTTGCTGTGCTTTCTATAGTGAATAGAGTTAGGCAGGGATACTCACCATTATCATTTCAGACCCAC---CTCCCAGCCAGGAGGGAA------CCCGACAGGCCCGAAGGAATCGAAGGAGAAGGTGGAGAGAAAGACAAAGACAGATCAGTTCGATTAGTGCATGGATTCTTAGCACTCATCTGGGACGACCTACGGAGCCTGTGCCTCTTCAGCTACCACCTCTTGAGAGACTTACTCTTGATTGTAACGAGGATTGTGGAAACTCTGGGACGCAGG---------------GGGTGGGAAGTCCTCAAGTATTGGTGG---AATCTCCTTCAGTATTGG---------------------------------------------------AGTCAGGAACTAAAGAATAGTGCTGTTAGCTTGTTTGATGCCATAGCTATCGCAGCAGCTGAGGGGACAGATAGGGCTATAGAAGTAGTGCGAAGA------------------CTTTTTAGAGCTTTTCTCCACATACCTACAAGAATCAGACAGGGATTGGAAAGGGCTTTGTTATAA

2.SC05.Trinidad.EU576781 ATGAGAGTGAAGGAGACAAGGAGGAATTGGCAGCACTTG---------TGGAAATGGGGC------------------------ACGATGCTCCTTGGGATGTTGATGATC------------TGTAGTGCTGCA---------GGAAATTTGTGGGTCACAGTCTATTATGGGGTACCTGTGTGGAAAGAAGCGACCACCACTCTATTTTGTGCATCAGATGCTAAAGCATATGATACAGAGGTACATAAT---GTCTGGGCCACACATGCCTGTGTACCCACAGACCCCAACCCACAAGAAGTAGTATTG---GGAAATGTGACAGAAAATTTTAACATGTGGAAAAATAACATGGTAGAACAGATGCATGAGGATATAATCAGTTTATGGGATCAAAGCCTAAAGCCATGTGTAAAATTAACCCCACTCTGTGTTACTTTAAATTGTACTGATGTGAATGGTACTAGTGCTAATGTCACTAGT------------------------------------------------------------------------------------------------------ATAGAAAAAGGAGAAATAAAAAACTGCTCTTTTAATATCACC---ACAACAATAAGAGAT------AAGGTGCAGAAAGCATATGCAACTTTTTATAGCCTTGATGTAGTACCAATAGATAATGATCAAGAT------------------------AATAGTAGC---------------------AGTAGCTATAGGTTGACAAATTGTAATACCTCAGTCATTACACAGGCCTGTCCAAAGGTGTCCTTTGAACCAATTCCCATACATTATTGTGCCCCGGCTGGTTTTGCGCTTCTAAAATGT---AATGATAAAAAGTTCAATGGAACAGGACCATGTACAAATGTCAGCACAGTACAATGTACACATGGAATTAGACCAGTAGTGTCAACTCAACTGTTGTTAAATGGCAGTCTAGCAGAAGAA---GAGGTAGTAATTAGATCTGAAAATTTCACAGACAATGCTAAAACCATAATAGTACAGCTGAATAACTCTGTAGTAATTAATTGTACAAGACCCAACAATAATACAAGAAAAAGTATACCTATAGGA------------CCAGGGAGT---GTATTTTAT---ACAGGAGAAATAATAGGAGATATAAGACAAGCACATTGTAACCTT------AGTAATACAGATTGGAATAACACTTTAAAACAGGTAGTTATAAAATTAAGA---GAACAATTTGAG------------AATAAAACA---ATAGTCTTTAATCAA---------TCCGCAGGAGGGGATCCAGAAATTGTATTGCACAGTTTTAATTGTGGAGGGGAATTTTTCTACTGTAATGCAACACAACTGTTTAATAGTACTTGGAATATT---------AATGGTACTTGGAATGGTACG---------------------AATGGTACTACAGAGTCA------------AATGACACT------------------------------ATCACACTCCCATGCAGAATAAAACAAATTATAAACATGTGGCAGGAAGTAGGCAAAGCAATGTATGCCCCTCCCATTAGAGGACAGATTAGATGTTCATCAAATATTACAGGATTACTATTAACAAGAGACGGTGGTAATAAT------------AACACGAGT------------------------GACACC---GAGATCTTCAGACCTGGAGGAGGAGATATGAGGGACAATTGGAGA---AGTGAATTATATAAATATAAAGTAGTAAAAATTGAACCA---TTAGGAATAGCACCC---ACCAAGGCAAAGAGAAGAGTGGTGCAGAGAGAA---AAAAGAGCAGTG---GGAACAATAGGA---GCTATG---TTCCTT---GGG---------TTCTTGGGA---GCAGCAGGAAGCACTATGGGCGCAGCATCATTA---ACGCTGACGGTACAGGCCAGACTATTATTGTCTGGTATAGTGCAACAGCAGAACAATCTGCTGAGGGCTATTGAGGCGCAACAGCATCTGTTGCAACTCACGGTCTGGGGCATCAAACAGCTCCAGGCAAGA---GTCCTGGCTGTGGAAAGATACCTAAGGGATCAACAGCTCCTGGGGATTTGGGGTTGCTCTGGAAAACTCATTTGCACCACTACTGTGCCTTGGAATGCTAGTTGGAGT---------------------------AATAAATCTTTGAATTATATTTGGGAT---AACATGACCTGGATGCAGTGGGATAGAGAAATTAAC------AATTACACAACCCTCATATACACCTTACTTGAAGATGCGCAGAACCAGCAAGAAAAGAATGAACACGAATTATTAGAATTGGATAAGTGGGCAAGTTTGTGGAATTGGTTTGACATAACACATTGGCTGTGGTATATAAAAATATTCATAATGATAGTAGGTGGTCTGATAGGTTTAAGAATAGTTTTTGCTGTGCTGTCTATAGTGAATAGAGTTAGGCAGGGATACTCACCATTATCATTTCAGACCCAC---CTCCCAGCCAGGAGGGAA------CCCGACAGGCCCGAAGGAATCGAAGGAGAAGGTGGAGAGAAAGACAAAGACAGATCAGTTCGATTAGTGCATGGATTCTTAGCACTCATCTGGGACGACCTACGGAGCCTGTGCCTCTTCAGCTACCACCTCTTGAGAGACTTACTCTTGATTGTAACGAGGATTGTGGAAACTCTGGGACGCAGG---------------GGGTGGGAAGTCCTCAAGTATTGGTGG---AATCTCCTTCAGTATTGG---------------------------------------------------AGTCAGGAACTAAAGAATAGTGCTGTTAGCTTGTTTGATGCCATAGCTATCGCAGCAGCTGAGGGGACAGATAGGGCTATAGAAGTAGTGCGAAGA------------------CTTTTTAGAGCTTTTCTCCACATACCTACAAGAATCAGACAGGGATTGGAAAGGGCTTTGTTATAA

2.SC05.Trinidad.EU576774 ATGAGAGTGAAGGAGACAAGGAGGAATTGGCAGCACTTG---------TGGAAATGGGGC------------------------ACGATGCTCCTTGGGATGTTGATGATC------------TGTAGTGCTGCA---------GGAAATTTGTGGGTCACAGTCTATTATGGGGTACCTGTGTGGAAAGAAGCGACCACCACTCTATTTTGTGCATCAGATGCTAAAGCATATGATACAGAGGTACATAAT---GTCTGGGCCACACATGCCTGTGTACCCACAGACCCCAACCCACAAGAAGTAGTATTG---GGAAATGTGACAGAAAATTTTAACATGTGGAAAAATAACATGGTAGAACAGATGCATGAGGATATAATCAGTTTATGGGATCAAAGCCTAAAGCCATGTGTAAAATTAACCCCACTCTGTGTTACTTTAAATTGTACTGATGTGAATGGTACTAGTGCTAATGTCACTAGT------------------------------------------------------------------------------------------------------ATAGAAAAAGGAGAAATAAAAAACTGCTCTTTTAATATCACC---ACAACAATAAGAGAT------AAGGTGCAGAAAGCATATGCAACTTTTTATAGCCTTGATGTAGTACCAATAGATAATGATCAAGAT------------------------AATAGTAGC---------------------AGTAGCTATAGGTTGACAAATTGTAATACCTCAGTCATTACACAGGCCTGTCCAAAGGTGTCCTTTGAACCAATTCCCATACATTATTGTGCCCCGGCTGGTTTTGCGCTTCTAAAATGT---AATGATAAAAAGTTCAATGGAACAGGACCATGTACAAATGTCAGCACAGTACAATGTACACATGGAATTAGACCAGTAGTGTCAACTCAACTGTTGTTAAATGGCAGTCTAGCAGAAGAA---GAGGTAGTAATTAGATCTGAAAATTTCACAGACAATGCTAAAACCATAATAGTACAGCTGAATAACTCTGTAGTAATTAATTGTACAAGACCCAACAATAATACAAGAAAAAGTATACCTATAGGA------------CCAGGGAGT---GTATTTTAT---ACAGGAGAAATAATAGGAGATATAAGACAAGCACATTGTAACCTT------AGTAATACAGATTGGAATAACACTTTAAAACAGGTAGTTATAAAATTAAGA---GAACAATTTGAG------------AATAAAACA---ATAGTCTTTAATCAA---------TCCGCAGGAGGGGATCCAGAAATTGTATTGCACAGTTTTAATTGTGGAGGGGAATTTTTCTACTGTAATGCAACACAACTGTTTAATAGTACTTGGAATATT---------AATGGTACTTGGAATGGTACG---------------------AATGGTACTACAGAGTCA------------AATGACACT------------------------------ATCACACTCCCATGCAGAATAAAACAAATTATAAACATGTGGCAGGAAGTAGGCAAAGCAATGTATGCCCCTCCCATTAGAGGACAGATTAGATGTTCATCAAATATTACAGGATTACTATTAACAAGAGACGGTGGTAATAAT------------AACACGAGT------------------------GACACC---GAGATCTTCAGACCTGGAGGAGGAGATATGAGGGACAATTGGAGA---AGTGAATTATATAAATATAAAGTAGTAAAAATTGAACCA---TTAGGAATAGCACCC---ACCAAGGCAAAGAGAAGAGTGGTGCAGAGAGAA---AAAAGAGCAGTG---GGAACAATAGGA---GCTATG---TTCCTT---GGG---------TTCTTGGGA---GCAGCAGGAAGCACTATGGGCGCAGCATCATTA---ACGCTGACGGTACAGGCCAGACTATTATTGTCTGGTATAGTGCAACAGCAGAACAATCTGCTGAGGGCTATTGAGGCGCAACAGCATCTGTTGCAACTCACGGTCTGGGGCATCAAACAGCTCCAGGCAAGA---GTCCTGGCTGTGGAAAGATACCTAAGGGATCAACAGCTCCTGGGGATTTGGGGTTGCTCTGGAAAACTCATTTGCACCACTACTGTGCCTTGGAATGCTAGTTGGAGT---------------------------AATAAATCTTTGAATTATATTTGGGAT---AACATGACCTGGATGCAGTGGGATAGAGAAATTAAC------AATTACACAACCCTCATATACACCTTACTTGAAGATGCGCAGAACCAGCAAGAAAAGAATGAACACGAATTATTAGAATTGGATAAGTGGGCAAGTTTGTGGAATTGGTTTGACATAACACATTGGCTGTGGTATATAAAAATATTCATAATGATAGTAGGTGGTCTGATAGGTTTAAGAATAGTTTTTGCTGTGCTTTCTATAGTGAATAGAGTTAGGCAGGGATACTCACCATTATCATTTCAGACCCAC---CTCCCAGCCAGGAGGGAA------CCCGACAGGCCCGAAGGAATCGAAGGAGAAGGTGGAGAGAAAGACAAAGACAGATCAGTTCGATTAGTGCATGGATTCTTAGCACTCATCTGGGACGACCTACGGAGCCTGTGCCTCTTCAGCTACCACCTCTTGAGAGACTTACTCTTGATTGTAACGAGGATTGTGGAAACTCTGGGACGCAGG---------------GGGTGGGAAGTCCTCAAGTATTGGTGG---AATCTCCTTCAGTATTGG---------------------------------------------------AGTCAGGAACTAAAGAATAGTGCTGTTAGCTTGTTTGATGCCATAGCTATCGCAGCAGCTGAGGGGACAGATAGGGCTATAGAAGTAGTGCGAAGA------------------CTTTTTAGAGCTTTTCTCCACATACCTACAAGAATCAGACAGGGATTGGAAAGGGCTTTGTTATAA

2.SC05.Trinidad.EU576777 ATGAGAGTGAAGGAGACAAGGAGGAATTGGCAGCACTTG---------TGGAAATGGGGC------------------------ACGATGCTCCTTGGGATGTTGATGATC------------TGTAGTGCTGCA---------GGAAATTTGTGGGTCACAGTCTATTATGGGGTACCTGTGTGGAAAGAAGCGACCACCACTCTATTTTGTGCATCAGATGCTAAAGCATATGATACAGAGGTACATAAT---GTCTGGGCCACACATGCCTGTGTACCCACAGACCCCAACCCACAAGAAGTAGTATTG---GGAAATGTGACAGAAAATTTTAACATGTGGAAAAATAACATGGTAGAACAGATGCATGAGGATATAATCAGTTTATGGGATCAAAGCCTAAAGCCATGTGTAAAATTAACCCCACTCTGTGTTACTTTAAATTGTACTGATGTGAATGGTACTAGTGCTAATGTCACTAGT------------------------------------------------------------------------------------------------------ATAGAAAAAGGAGAAATAAAAAACTGCTCTTTTAATATCACC---ACAACAATAAGAGAT------AAGGTGCAGAAAGCATATGCAACTTTTTATAGCCTTGATGTAGTACCAATAGATAATGATCAAGAT------------------------AATAGTAGC---------------------AGTAGCTATAGGTTGACAAATTGTAATACCTCAGTCATTACACAGGCCTGTCCAAAGGTGTCCTTTGAACCAATTCCCATACATTATTGTGCCCCGGCTGGTTTTGCGCTTCTAAAATGT---AATGATAAAAAGTTCAATGGAACAGGACCATGTACAAATGTCAGCACAGTACAATGTACACATGGAATTAGACCAGTAGTGTCAACTCAACTGTTGTTAAATGGCAGTCTAGCAGAAGAA---GAGGTAGTAATTAGATCTGAAAATTTCACAGACAATGCTAAAACCATAATAGTACAGCTGAATAACTCTGTAGTAATTAATTGTACAAGACCCAACAATAATACAAGAAAAAGTATACCTATAGGA------------CCAGGGAGT---GTATTTTAT---ACAGGAGAAATAATAGGAGATATAAGACAAGCACATTGTAACCTT------AGTAATACAGATTGGAATAACACTTTAAAACAGGTAGTTATAAAATTAAGA---GAACAATTTGAG------------AATAAAACA---ATAGTCTTTAATCAA---------TCCGCAGGAGGGGATCCAGAAATTGTATTGCACAGTTTTAATTGTGGAGGGGAATTTTTCTACTGTAATGCAACACAACTGTTTAATAGTACTTGGAATATT---------AATGGTACTTGGAATGGTACG---------------------AATGGTACTACAGAGTCA------------AATGACACT------------------------------ATCACACTCCCATGCAGAATAAAACAAATTATAAACATGTGGCAGGAAGTAGGCAAAGCAATGTATGCCCCTCCCATTAGAGGACAGATTAGATGTTCATCAAATATTACAGGATTACTATTAACAAGAGACGGTGGTAATAAT------------AACACGAGT------------------------GACACC---GAGATCTTCAGACCTGGAGGAGGAGATATGAGGGACAATTGGAGA---AGTGAATTATATAAATATAAAGTAGTAAAAATTGAACCA---TTAGGAATAGCACCC---ACCAAGGCAAAGAGAAGAGTGGTGCAGAGAGAA---AAAAGAGCAGTG---GGAACAATAGGA---GCTATG---TTCCTT---GGG---------TTCTTGGGA---GCAGCAGGAAGCACTATGGGCGCAGCATCATTA---ACGCTGACGGTACAGGCCAGACTATTATTGTCTGGTATAGTGCAACAGCAGAACAATCTGCTGAGGGCTATTGAGGCGCAACAGCATCTGTTGCAACTCACGGTCTGGGGCATCAAACAGCTCCAGGCAAGA---GTCCTGGCTGTGGAAAGATACCTAAGGGATCAACAGCTCCTGGGGATTTGGGGTTGCTCTGGAAAACTCATTTGCACCACTACTGTGCCTTGGAATGCTAGTTGGAGT---------------------------AATAAATCTTTGAATTATATTTGGGAT---AACATGACCTGGATGCAGTGGGATAGAGAAATTAAC------AATTACACAACCCTCATATACACCTTACTTGAAGATGCGCAGAACCAGCAAGAAAAGAATGAACACGAATTATTAGAATTGGATAAGTGGGCAAGTTTGTGGAATTGGTTTGACATAACACATTGGCTGTGGTATATAAAAATATTCATAATGATAGTAGGTGGTCTGATAGGTTTAAGAATAGTTTTTGCTGTGCTTTCTATAGTGAATAGAGTTAGGCAGGGATACTCACCATTATCATTTCAGACCCAC---CTCCCAGCCAGGAGGGAA------CCCGACAGGCCCGAAGGAATCGAAGGAGAAGGTGGAGAGAAAGACAAAGACAGATCAGTTCGATTAGTGCATGGATTCTTAGCACTCATCTGGGACGACCTACGGAGCCTGTGCCTCTTCAGCTACCACCTCTTGAGAGACTTACTCTTGATTGTAACGAGGATTGTGGAAACTCTGGGACGCAGG---------------GGGTGGGAAGTCCTCAAGTATTGGTGG---AATCTCCTTCAGTATTGG---------------------------------------------------AGTCAGGAACTAAAGAATAGTGCTGTTAGCTTGTTTGATGCCATAGCTATCGCAGCAGCTGAGGGGACAGATAGGGCTATAGAAGTAGTGCGAAGA------------------CTTTTTAGAGCTTTTCTCCACATACCTACAAGAATCAGACAGGGATTGGAAAGGGCTTTGTTATAA

2.SC05.Trinidad.EU576765 ATGAGAGTGAAGGAGACAAGGAGGAATTGGCAGCACTTG---------TGGAAATGGGGC------------------------ACGATGCTCCTTGGGATGTTGATGATC------------TGTAGTGCTGCA---------GGAAATTTGTGGGTCACAGTCTATTATGGGGTACCTGTGTGGAAAGAAGCGACCACCACTCTATTTTGTGCATCAGATGCTAAAGCATATGATACAGAGGTACATAAT---GTCTGGGCCACACATGCCTGTGTACCCACAGACCCCAACCCACAAGAAGTAGTATTG---GGAAATGTGACAGAAAATTTTAACATGTGGAAAAATAACATGGTAGAACAGATGCATGAGGATATAATCAGTTTATGGGATCAAAGCCTAAAGCCATGTGTAAAATTAACCCCACTCTGTGTTACTTTAAATTGTACTGATGTGAATGGTACTAGTGCTAATGTCACTAGT------------------------------------------------------------------------------------------------------ATAGAAAAAGGAGAAATAAAAAACTGCTCTTTTAATATCACC---ACAACAATAAGAGAT------AAGGTGCAGAAAGCATATGCAACTTTTTATAGCCTTGATGTAGTACCAATAGATAATGATCAAGAT------------------------AATAGTAGC---------------------AGTAGCTATAGGTTGACAAATTGTAATACCTCAGTCATTACACAGGCCTGTCCAAAGGTGTCCTTTGAACCAATTCCCATACATTATTGTGCCCCGGCTGGTTTTGCGCTTCTAAAATGT---AATGATAAAAAGTTCAATGGAACAGGACCATGTACAAATGTCAGCACAGTACAATGTACACATGGAATTAGACCAGTAGTGTCAACTCAACTGTTGTTAAATGGCAGTCTAGCAGAAGAA---GAGGTAGTAATTAGATCTGAAAATTTCACAGACAATGCTAAAACCATAATAGTACAGCTGAATAACTCTGTAGTAATTAATTGTACAAGACCCAACAATAATACAAGAAAAAGTATACCTATAGGA------------CCAGGGAGT---GTATTTTAT---ACAGGAGAAATAATAGGAGATATAAGACAAGCACATTGTAACCTT------AGTAATACAGATTGGAATAACACTTTAAAACAGGTAGTTATAAAATTAAGA---GAACAATTTGAG------------AATAAAACA---ATAGTCTTTAATCAA---------TCCGCAGGAGGGGATCCAGAAATTGTATTGCACAGTTTTAATTGTGGAGGGGAATTTTTCTACTGTAATGCAACACAACTGTTTAATAGTACTTGGAATATT---------AATGGTACTTGGAATGGTACG---------------------AATGGTACTACAGAGTCA------------AATGACACT------------------------------ATCACACTCCCATGCAGAATAAAACAAATTATAAACATGTGGCAGGAAGTAGGCAAAGCAATGTATGCCCCTCCCATTAGAGGACAGATTAGATGTTCATCAAATATTACAGGATTACTATTAACAAGAGACGGTGGTAATAAT------------AACACGAGT------------------------GACACC---GAGATCTTCAGACCTGGAGGAGGAGATATGAGGGACAATTGGAGA---AGTGAATTATATAAATATAAAGTAGTAAAAATTGAACCA---TTAGGAATAGCACCC---ACCAAGGCAAAGAGAAGAGTGGTGCAGAGAGAA---AAAAGAGCAGTG---GGAACAATAGGA---GCTATG---TTCCTT---GGG---------TTCTTGGGA---GCAGCAGGAAGCACTATGGGCGCAGCATCATTA---ACGCTGACGGTACAGGCCAGACTATTATTGTCTGGTATAGTGCAACAGCAGAACAATCTGCTGAGGGCTATTGAGGCGCAACAGCATCTGTTGCAACTCACGGTCTGGGGCATCAAACAGCTCCAGGCAAGA---GTCCTGGCTGTGGAAAGATACCTAAGGGATCAACAGCTCCTGGGGATTTGGGGTTGCTCTGGAAAACTCATTTGCACCACTACTGTGCCTTGGAATGCTAGTTGGAGT---------------------------AATAAATCTTTGAATTATATTTGGGAT---AACATGACCTGGATGCAGTGGGATAGAGAAATTAAC------AATTACACAACCCTCATATACACCTTACTTGAAGATGCGCAGAACCAGCAAGAAAAGAATGAACACGAATTATTAGAATTGGATAAGTGGGCAAGTTTGTGGAATTGGTTTGACATAACACATTGGCTGTGGTATATAAAAATATTCATAATGATAGTAGGTGGTCTGATAGGTTTAAGAATAGTTTTTGCTGTGCTTTCTATAGTAAATAGAGTTAGGCAGGGATACTCACCATTATCATTTCAGACCCAC---CTCCCAGCCAGGAGGGAA------CCCGACAGGCCCGAAGGAATCGAAGGAGAAGGTGGAGAGAAAGACAAAGACAGATCAGTTCGATTAGTGCATGGATTCTTAGCACTCATCTGGGACGACCTACGGAGCCTGTGCCTCTTCAGCTACCACCTCTTGAGAGACTTACTCTTGATTGTAACGAGGATTGTGGAAACTCTGGGACGCAGG---------------GGGTGGGAAGTCCTCAAGTATTGGTGG---AATCTCCTTCAGTATTGG---------------------------------------------------AGTCAGGAACTAAAGAATAGTGCTGTTAGCTTGTTTGATGCCATAGCTATCGCAGCAGCTGAGGGGACAGATAGGGCTATAGAAGTAGTGCGAAGA------------------CTTTTTAGAGCTTTTCTCCACATACCTACAAGAATCAGACAGGGATTGGAAAGGGCTTTGTTATAA

2.SC05.Trinidad.EU576776 ATGAGAGTGAAGGAGACAAGGAGGAATTGGCAGCACTTG---------TGGAAATGGGGC------------------------ACGATGCTCCTTGGGATGTTGATGATC------------TGTAGTGCTGCA---------GGAAATTTGTGGGTCACAGTCTATTATGGGGTACCTGTGTGGAAAGAAGCGACCACCACTCTATTTTGTGCATCAGATGCTAAAGCATATGATACAGAGGTACATAAT---GTCTGGGCCACACATGCCTGTGTACCCACAGACCCCAACCCACAAGAAGTAGTATTG---GGAAATGTGACAGAAAATTTTAACATGTGGAAAAATAACATGGTAGAACAGATGCATGAGGATATAATCAGTTTATGGGATCAAAGCCTAAAGCCATGTGTAAAATTAACCCCACTCTGTGTTACTTTAAATTGTACTGATGTGAATGGTACTAGTGCTAATGTCACTAGT------------------------------------------------------------------------------------------------------ATAGAAAAAGGAGAAATAAAAAACTGCTCTTTTAATATCACC---ACAACAATAAGAGAT------AAGGTGCAGAAAGCATATGCAACTTTTTATAGCCTTGATGTAGTACCAATAGATAATGATCAAGAT------------------------AATAGTAGC---------------------AGTAGCTATAGGTTGACAAATTGTAATACCTCAGTCATTACACAGGCCTGTCCAAAGGTGTCCTTTGAACCAATTCCCATACATTATTGTGCCCCGGCTGGTTTTGCGCTTCTAAAATGT---AATGATAAAAAGTTCAATGGAACAGGACCATGTACAAATGTCAGCACAGTACAATGTACACATGGAATTAGACCAGTAGTGTCAACTCAACTGTTGTTAAATGGCAGTCTAGCAGAAGAA---GAGGTAGTAATTAGATCTGAAAATTTCACAGACAATGCTAAAACCATAATAGTACAGCTGAATAACTCTGTAGTAATTAATTGTACAAGACCCAACAATAATACAAGAAAAAGTATACCTATAGGA------------CCAGGGAGT---GTATTTTAT---ACAGGAGAAATAATAGGAGATATAAGACAAGCACATTGTAACCTT------AGTAATACAGATTGGAATAACACTTTAAAACAGGTAGTTATAAAATTAAGA---GAACAATTTGAG------------AATAAAACA---ATAGTCTTTAATCAA---------TCCGCAGGAGGGGATCCAGAAATTGTATTGCACAGTTTTAATTGTGGAGGGGAATTTTTCTACTGTAATGCAACACAACTGTTTAATAGTACTTGGAATATT---------AATGGTACTTGGAATGGTACG---------------------AATGGTACTACAGAGTCA------------AATGACACT------------------------------ATCACACTCCCATGCAGAATAAAACAAATTATAAACATGTGGCAGGAAGTAGGCAAAGCAATGTATGCCCCTCCCATTAGAGGACAGATTAGATGTTCATCAAATATTACAGGATTACTATTAACAAGAGACGGTGGTAATAAT------------AACACGAGT------------------------GACACC---GAGATCTTCAGACCTGGAGGAGGAGATATGAGGGACAATTGGAGA---AGTGAATTATATAAATATAAAGTAGTAAAAATTGAACCA---TTAGGAATAGCACCC---ACCAAGGCAAAGAGAAGAGTGGTGCAGAGAGAA---AAAAGAGCAGTG---GGAACAATAGGA---GCTATG---TTCCTT---GGG---------TTCTTGGGA---GCAGCAGGAAGCACTATGGGCGCAGCATCATTA---ACGCTGACGGTACAGGCCAGACTATTATTGTCTGGTATAGTGCAACAGCAGAACAATCTGCTGAGGGCTATTGAGGCGCAACAGCATCTGTTGCAACTCACGGTCTGGGGCATCAAACAGCTCCAGGCAAGA---GTCCTGGCTGTGGAAAGATACCTAAGGGATCAACAGCTCCTGGGGATTTGGGGTTGCTCTGGAAAACTCATTTGCACCACTACTGTGCCTTGGAATGCTAGTTGGAGT---------------------------AATAAATCTTTGAATTATATTTGGGAT---AACATGACCTGGATGCAGTGGGATAGAGAAATTAAC------AATTACACAACCCTCATATACACCTTACTTGAAGATGCGCAGAACCAGCAAGAAAAGAATGAACACGAATTATTAGAATTGGATAAGTGGGCAAGTTTGTGGAATTGGTTTGACATAGCACATTGGCTGTGGTATATAAAAATATTCATAATGATAGTAGGTGGTCTGATAGGTTTAAGAATAGTTTTTGCTGTGCTTTCTATAGTGAATAGAGTTAGGCAGGGATACTCACCATTATCATTTCAGACCCAC---CTCCCAGCCAGGAGGGAA------CCCGACAGGCCCGAAGGAATCGAAGGAGAAGGTGGAGAGAAAGACAAAGACAGATCAGTTCGATTAGTGCATGGATTCTTAGCACTCATCTGGGACGACCTACGGAGCCTGTGCCTCTTCAGCTACCACCTCTTGAGAGACTTACTCTTGATTGTAACGAGGATTGTGGAAACTCTGGGACGCAGG---------------GGGTGGGAAGTCCTCAAGTATTGGTGG---AATCTCCTTCAGTATTGG---------------------------------------------------AGTCAGGAACTAAAGAATAGTGCTGTTAGCTTGTTTGATGCCATAGCTATCGCAGCAGCTGAGGGGACAGATAGGGCTATAGAAGTAGTGCGAAGA------------------CTTTTTAGAGCTTTTCTCCACATACCTACAAGAATCAGACAGGGATTGGAAAGGGCTTTGTTATAA

2.SC05.Trinidad.EU576784 ATGAGAGTGAAGGAGACAAGGAGGAATTGGCAGCACTTG---------TGGAAATGGGGC------------------------ACGATGCTCCTTGGGATGTTGATGATC------------TGTAGTGCTGCA---------GGAAATTTGTGGGTCACAGTCTATTATGGGGTACCTGTGTGGAAAGAAGCGACCACCACTCTATTTTGTGCATCAGATGCTAAAGCATATGATACAGAGGTACATAAT---GTCTGGGCCACACATGCCTGTGTACCCACAGACCCCAACCCACAAGAAGTAGTATTG---GGAAATGTGACAGAAAATTTTAACATGTGGAAAAATAACATGGTAGAACAGATGCATGAGGATATAATCAGTTTATGGGATCAAAGCCTAAAGCCATGTGTAAAATTAACCCCACTCTGTGTTACTTTAAATTGTACTGATGTGAATGGTACTAGTGCTAATGTCACTAGT------------------------------------------------------------------------------------------------------ATAGAAAAAGGAGAAATAAAAAACTGCTCTTTTAATATCACC---ACAACAATAAGAGAT------AAGGTGCAGAAAGCATATGCAACTTTTTATAGCCTTGATGTAGTACCAATAGATAATGATCAAGAT------------------------AATAGTAGC---------------------AGTAGCTATAGGTTGACAAATTGTAATACCTCAGTCATTACACAGGCCTGTCCAAAGGTGTCCTTTGAACCAATTCCCATACATTATTGTGCCCCGGCTGGTTTTGCGCTTCTAAAATGT---AATGATAAAAAGTTCAATGGAACAGGACCATGTACAAATGTCAGCACAGTACAATGTACACATGGAATTAGACCAGTAGTGTCAACTCAACTGTTGTTAAATGGCAGTCTAGCAGAAGAA---GAGGTAGTAATTAGATCTGAAAATTTCACAGACAATGCTAAAACCATAATAGTACAGCTGAATAACTCTGTAGTAATTAATTGTACAAGACCCAACAATAATACAAGAAAAAGTATACCTATAGGA------------CCAGGGAGT---GTATTTTAT---ACAGGAGAAATAATAGGAGATATAAGACAAGCACATTGTAACCTT------AGTAATACAGATTGGAATAACACTTTAAAACAGGTAGTTATAAAATTAAGA---GAACAATTTGAG------------AATAAAACA---ATAGTCTTTAATCAA---------TCCGCAGGAGGGGATCCAGAAATTGTATTGCACAGTTTTAATTGTGGAGGGGAATTTTTCTACTGTAATGCAACACAACTGTTTAATAGTACTTGGAATATT---------AATGGTACTTGGAATGGTACG---------------------AATGGTACTACAGAGTCA------------AATGACACT------------------------------ATCACACTCCCATGCAGAATAAAACAAATTATAAACATGTGGCAGGAAGTAGGCAAAGCAATGTATGCCCCTCCCATTAGAGGACAGATTAGATGTTCATCAAATATTACAGGATTACTATTAACAAGAGACGGTGGTAATAAT------------AACACGAGT------------------------GACACC---GAGATCTTCAGACCTGGAGGAGGAGATATGAGGGACAATTGGAGA---AGTGAATTATATAAATATAAAGTAGTAAAAATTGAACCA---TTAGGAATAGCACCC---ACCAAGGCAAAGAGAAGAGTGGTGCAGAGAGAA---AAAAGAGCAGTG---GGAACAATAGGA---GCTATG---TTCCTT---GGG---------TTCTTGGGA---GCAGCAGGAAGCACTATGGGCGCAGCATCATTA---ACGCTGACGGTACAGGCCAGACTATTATTGTCTGGTATAGTGCAACAGCAGAACAATCTGCTGAGGGCTATTGAGGCGCAACAGCATCTGTTGCAACTCACGGTCTGGGGCATCAAACAGCTCCAGGCAAGA---GTCCTGGCTGTGGAAAGATACCTAAGGGATCAACAGCTCCTGGGGATTTGGGGTTGCTCTGGAAAACTCATTTGCACCACTACTGTGCCTTGGAATGCTAGTTGGAGT---------------------------AATAAATCTTTGAATTATATTTGGGAT---AACATGACCTGGATGCAGTGGGATAGAGAAATTAAC------AATTACACAACCCTCATATACACCTTACTTGAAGATGCGCAGAACCAGCAAGAAAAGAATGAACACGAATTATTAGAATTGGATAAGTGGGCAAGTTTGTGGAATTGGTTTGACATAACACATTGGCTGTGGTATATAAAAATATTCATAATGATAGTAGGTGGTCTGATAGGTTTAAGAATAGTTTTTGCTGTGCTTTCTATAGTGAATAGAGTTAGGCAGGGATACTCACCATTATCATTTCAGACCCAC---CTCCCAGCCAGGAGGGAA------CCCGACAGGCCCGAAGGAATCGAAGGAGAAGGTGGAGAGAAAGACAAAGACAGATCAGTTCGATTAGTGCATGGATTCTTAGCACTCATCTGGGACGACCTACGGAGCCTGTGCCTCTTCAGCTACCACCTCTTGAGAGACTTACTCTTGATTGTAACGAGGATTGTGGAAACTCTGGGACGCAGG---------------GGGTGGGAAGTCCTCAAGTATTGGTGG---AATCTCCTTCAGTATTGG---------------------------------------------------AGTCAGGAACTAAAGAATAGTGCTGTTAGCTTGTTTGATGCCATAGCTATCGCAGCAGCTGAGGGGACAGATAGGGCTATAGAAGTAGTGCGAAGA------------------CTTTTTAGAGCTTTTCTCCACATACCTACAAGAATCAGACAGGGATTGGAAAGGGCTTTGTTATAA

2.SC05.Trinidad.EU576766 ATGAGAGTGAAGGAGACAAGGAGGAATTGGCAGCACTTG---------TGGAAATGGGGC------------------------ACGATGCTCCTTGGGATGTTGATGATC------------TGTAGTGCTGCA---------GGAAATTTGTGGGTCACAGTCTATTATGGGGTACCTGTGTGGAAAGAAGCGACCACCACTCTATTTTGTGCATCAGATGCTAAAGCATATGATACAGAGGTACATAAT---GTCTGGGCCACACATGCCTGTGTACCCACAGACCCCAACCCACAAGAAGTAGTATTG---GGAAATGTGACAGAAAATTTTAACATGTGGAAAAATAACATGGTAGAACAGATGCATGAGGATATAATCAGTTTATGGGATCAAAGCCTAAAGCCATGTGTAAAATTAACCCCACTCTGTGTTACTTTAAATTGTACTGATGTGAATGGTACTAGTGCTAATGTCACTAGT------------------------------------------------------------------------------------------------------ATAGAAAAAGGAGAAATAAAAAACTGCTCTTTTAATATCACC---ACAACAATAAGAGAT------AAGGTGCAGAAAGCATATGCAACTTTTTATAGCCTTGATGTAGTACCAATAGATAATGATCAAGAT------------------------AATAGTAGC---------------------AGTAGCTATAGGTTGACAAATTGTAATACCTCAGTCATTACACAGGCCTGTCCAAAGGTGTCCTTTGAACCAATTCCCATATATTATTGTGCCCCGGCTGGTTTTGCGCTTCTAAAATGT---AATGATAAAAAGTTCAATGGAACAGGACCATGTACAAATGTCAGCACAGTACAATGTACACATGGAATTAGACCAGTAGTGTCAACTCAACTGTTGTTAAATGGCAGTCTAGCAGAAGAA---GAGGTAGTAATTAGATCTGAAAATTTCACAGACAATGCTAAAACCATAATAGTACAGCTGAATAACTCTGTAGTAATTAATTGTACAAGACCCAACAATAATACAAGAAAAAGTATACCTATAGGA------------CCAGGGAGT---GTATTTTAT---ACAGGAGAAATAATAGGAGATATAAGACAAGCACATTGTAACCTT------AGTAATACAGATTGGAATAACACTTTAAAACAGGTAGTTATAAAATTAAGA---GAACAATTTGAG------------AATAAAACA---ATAGTCTTTAATCAA---------TCCGCAGGAGGGGATCCAGAAATTGTATTGCACAGTTTTAATTGTGGAGGGGAATTTTTCTACTGTAATGCAACACAACTGTTTAATAGTACTTGGAATATT---------AATGGTACTTGGAATGGTACG---------------------AATGGTACTACAGAGTCA------------AATGACACT------------------------------ATCACACTCCCATGCAGAATAAAACAAATTATAAACATGTGGCAGGAAGTAGGCAAAGCAATGTATGCCCCTCCCATTAGAGGACAGATTAGATGTTCATCAAATATTACAGGATTACTATTAACAAGAGACGGTGGTAATAAT------------AACACGAGT------------------------GACACC---GAGATCTTCAGACCTGGAGGAGGAGATATGAGGGACAATTGGAGA---AGTGAATTATATAAATATAAAGTAGTAAAAATTGAACCA---TTAGGAATAGCACCC---ACCAAGGCAAAGAGAAGAGTGGTGCAGAGAGAA---AAAAGAGCAGTG---GGAACAATAGGA---GCTATG---TTCCTT---GGG---------TTCTTGGGA---GCAGCAGGAAGCACTATGGGCGCAGCATCATTA---ACGCTGACGGTACAGGCCAGACTATTATTGTCTGGTATAGTGCAACAGCAGAACAATCTGCTGAGGGCTATTGAGGCGCAACAGCATCTATTGCAACTCACGGTCTGGGGCATCAAACAGCTCCAGGCAAGA---GTCCTGGCTGTGGAAAGATACCTAAGGGATCAACAGCTCCTGGGGATTTGGGGTTGCTCTGGAAAACTCATTTGCACCACTACTGTGCCTTGGAATGCTAGTTGGAGT---------------------------AATAAATCTTTGAATTATATTTGGGAT---AACATGACCTGGATGCAGTGGGATAGAGAAATTAAC------AATTACACAACCCTCATATACACCTTACTTGAAGATGCGCAGAACCAGCAAGAAAAGAATGAACACGAATTATTAGAATTGGATAAGTGGGCAAGTTTGTGGAATTGGTTTGACATAACACATTGGCTGTGGTATATAAAAATATTCATAATGATAGTAGGTGGTCTGATAGGTTTAAGAATAGTTTTTGCTGTGCTTTCTATAGTGAATAGAGTTAGGCAGGGATACTCACCATTATCATTTCAGACCCAC---CTCCCAGCCAGGAGGGAA------CCCGACAGGCCCGAAGGAATCGAAGGAGAAGGTGGAGAGAAAGACAAAGACAGATCAGTTCGATTAGTGCATGGATTCTTAGCACTCATCTGGGACGACCTACGGAGCCTGTGCCTCTTCAGCTACCACCTCTTGAGAGACTTACTCTTGATTGTAACGAGGATTGTGGAAACTCTGGGACGCAGG---------------GGGTGGGAAGTCCTCAAGTATTGGTGG---AATCTCCTTCAGTATTGG---------------------------------------------------AGTCAGGAACTAAAGAATAGTGCTGTTAGCTTGTTTGATGCCATAGCTATCGCAGCAGCTGAGGGGACAGATAGGGCTATAGAAGTAGTGCGAAGA------------------CTTTTTAGAGCTTTTCTCCACATACCTACAAGAATCAGACAGGGATTGGAAAGGGCTTTGTTATAA

2.SC05.Trinidad.EU576759 ATGAGAGTGAAGGAGACAAGGAGGAATTGGCAGCACTTG---------TGGAAATGGGGC------------------------ACGATGCTCCTTGGGATGTTGATGATC------------TGTAGTGCTGCA---------GGAAATTTGTGGGTCACAGTCTATTATGGGGTACCTGTGTGGAAAGAAGCGACCACCACTCTATTTTGTGCATCAGATGCTAAAGCATATGATACAGAGGTACATAAT---GTCTGGGCCACACATGCCTGTGTACCCACAGACCCCAACCCACAAGAAGTAGTATTG---GGAAATGTGACAGAAAATTTTAACATGTGGAAAAATAACATGGTAGAACAGATGCATGAGGATATAATCAGTTTATGGGATCAAAGCCTAAAGCCATGTGTAAAATTAACCCCACTCTGTGTTACTTTAAATTGTACTGATGTGAATGGTACTAGTGCTAATGTCACTAGT------------------------------------------------------------------------------------------------------ATAGAAAAAGGAGAAATAAAAAACTGCTCTTTTAATATCACC---ACAACAATAAGAGAT------AAGGTGCAGAAAGCATATGCAACTTTTTATAGCCTTGATGTAGTACCAATAGATAATGATCAAGAT------------------------AATAGTAGC---------------------AGTAGCTATAGGTTGACAAATTGTAATACCTCAGTCATTACACAGGCCTGTCCAAAGGTGTCCTTTGAACCAATTCCCATACATTATTGTGCCCCGGCTGGTTTTGCGCTTCTAAAATGT---AATGATAAAAAGTTCAATGGAACAGGACCATGTACAAATGTCAGCACAGTACAATGTACACATGGAATTAGACCAGTAGTGTCAACTCAACTGTTGTTAAATGGCAGTCTAGCAGAAGAA---GAGGTAGTAATTAGATCTGAAAATTTCACAGACAATGCTAAAACCATAATAGTACAGCTGAATAACTCTGTAGTAATTAATTGTACAAGACCCAACAATAATACAAGAAAAAGTATACCTATAGGA------------CCAGGGAGT---GTATTTTAT---ACAGGAGAAATAATAGGAGATATAAGACAAGCACATTGTAACCTT------AGTAATACAGATTGGAATAACACTTTAAAACAGGTAGTTATAAAATTAAGA---GAACAATTTGAG------------AATAAAACA---ATAGTCTTTAATCAA---------TCCGCAGGAGGGGATCCAGAAATTGTATTGCACAGTTTTAATTGTGGAGGGGAATTTTTCTACTGTAATGCAACACAACTGTTTAATAGTACTTGGAATATT---------AATGGTACTTGGAATGGTACG---------------------AATGGTACTACAGAGTCA------------AATGACACT------------------------------ATCACACTCCCATGCAGAATAAAACAAATTATAAACATGTGGCAGGAAGTAGGCAAAGCAATGTATGCCCCTCCCATTAGAGGACAGATTAGATGTTCATCAAATATTACAGGATTACTATTAACAAGAGACGGTGGTAATAAT------------AACACGAGT------------------------GACACC---GAGATCTTCAGACCTGGAGGAGGAGATATGAGGGACAATTGGAGA---AGTGAATTATATAAATATAAAGTAGTAAAAATTGAACCA---TTAGGAATAGCACCC---ACCAAGGCAAAGAGAAGAGTGGTGCAGAGAGAA---AAAAGAGCAGTG---GGAACAATAGGA---GCTATG---TTCCTT---GGG---------TTCTTGGGA---GCAGCAGGAAGCACTATGGGCGCAGCATCATTA---ACGCTGACGGTACAGGCCAGACTATTATTGTCTGGTATAGTGCAACAGCAGAACAATCTGCTGAGGGCTATTGAGGCGCAACAGCATCTGTTGCAACTCACGGTCTGGGGCATCAAACAGCTCCAGGCAAGA---GTCCTGGCTGTGGAAAGATACCTAAGGGATCAACAGCTCCTGGGGATTTGGGGTTGCTCTGGAAAACTCATTTGCACCACTACTGTGCCTTGGAATGCTAGTTGGAGT---------------------------AATAAATCTTTGAATTATATTTGGGAT---AACATGACCTGGATGCAGTGGGATAGAGAAATTAAC------AATTACACAACCCTCATATACACCTTACTTGAAGATGCGCAGAACCAGCAAGAAAAGAATGAACACGAATTATTAGAATTGGATAAGTGGGCAAGTTTGTGGAATTGGTTTGACATAACACATTGGCTGTGGTATATAAAAATATTCATAATGATAGTAGGTGGTCTGATAGGTTTAAGAATAGTTTTTGCTGTGCTTTCTATAGTGAATAGAGTTAGGCAGGGATACTCACCATTATCATTTCAGACCCAC---CTCCCAGCCAGGAGGGAA------CCCGACAGGCCCGAAGGAATCGAAGGAGAAGGTGGAGAGAAAGACAAAGACAGATCAGTTCGATTAGTGCATGGATTCTTAGCACTCATCTGGGACGACCTACGGAGCCTGTGCCTCTTCAGCTACCACCTCTTGAGAGACTTACTCTTGATTGTAACGAGGATTGTGGAAACTCTGGGACGCAGG---------------GGGTGGGAAGTCCTCAAGTATTGGTGG---AATCTCCTTCAGTATTGG---------------------------------------------------AGTCAGGAACTAAAGAATAGTGCTGTTAGCTTGTTTGATGCCATAGCTATCGCAGCAGCTGAGGGGACAGATAGGGCTATAGAAGTAGTGCGAAGA------------------CTTTTTAGAGCTTTTCTCCACATACCTACAAGAATCAGACAGGGATTGGAAAGGGCTTTGTTATAA

2.SC11.Trinidad.EU576806 ATGACAGTGATGGAGATCAGGAGGAATTAC------TTG---------TGGAGATGGGGC------------------------ATCATGCTCCTTGGAATGTTGATGATC------------TATAGTGCTGCAGAACAA---GAACAAGGGTGGGTCACAGTCTATTATGGGGTACCTGTGTGGAAAGAAGCAACCACCACTCTATTCTGTGCATCAGATGCCAAAGCATATGACACAGAGGTACATAAT---GTCTGGGCCACACATGCCTGTGTACCCACAGATCCCAACCCACAAGAAGTAATGCTG---GAAAATGTGACAGAAAATTTTAACATGTGGAAAAATAACATGGTAGAACAGATGCATGAAGATATAATCAGTTTATGGGATCAAAGCTTAAAGCCATGTGTAAAATTAACCCCACTCTGTGTTACTTTAAATTGCAGTGATACCTGGAGAAATGATACTAATGCCACTGCC---------------------------------------------------------------------------------------AGTAGTTGGGAAAAGGGAGAGAAAGGAGAGATAAAAAACTGCTCTTTCAATGTCACC---GCAGGCATAAGAGAT------AAGGTGCAGAAAGAATATGCACTTTTTTATAAACTTGATGTAATACCAATAGATAATAATAATGGTAGTGAA------------------AATAGTAGC------------------TATGGTAGCTATAGATTGATAAATTGTAATACCTCAGTCATTACACAGGCTTGTCCAAAGGTATCCTTTGAGCCAATTCCCATACACTATTGTGCCCCGGCTGGTTTTGCGATTCTAAAGTGT---AATAATAAGAAGTTCAATGGAACAGGACCATGTACAAATGTTAGCACAGTACAATGTACACATGGAATTAGACCAGTAGTGTCAACTCAACTGCTGTTAAATGGCAGTCTAGCAGAAGAA---GAGGTAGTAATTAGATCTGAAAATTTCACGAACAATGCTAAAATTATAATAGTACATCTGAATAAATCTGTAGTAATTAATTGTACAAGACCCAACAACAATACAAGAAGAAGTATACATATAGGA------------CCAGGGAAA---GTATTTTAT---ACAGGGGAAATAATAGGAGATATAAGACAAGCACATTGTAACCTT------AGTAGAGCGGCATGGAATAACACTTTAGAACAGATAGTTATAAAATTAAGA---GAACAATTTGGG------------AATAAAACA---ATAGTCTTTAAGCAA---------TCCTCAGGAGGGGACCCAGAAATTGTAATGCACACTTTTAATTGTGGAGGGGAATTTTTCTACTGTAATGCAACACCACTGTTTAATAGTAGCTGGTATGTT---------AATGGTACTAAC------------------------------ACTACAGGGTCAGATGGAGAA---------------------------------------------AATATCACACTCCTATGCAGAATAAAACAAATTGTAAACATGTGGCAGGAAGTAGGCAAAGCAATGTATGCCCCTCCCATTAGAGGACAAATTACATGCTCATCAAAGATCACAGGGTTGCTATTAACAAGAGATGGTGGTAACATG------------AACGGGACC---------------------AACGACACC---GAGACCTTCAGACCTGGAGGAGGAGATATGAGGGACAATTGGAGA---AGTGAATTATATAAATATAAAGTAGTAAAAATTGAACCA---TTAGGAGTAGCACCC---ACCAAGGCAAAGAGAAGAGTGGTGCAGAGAGAA---AAAAGAGCAGTG---GGAACAATAGGA---GCTATG---TTCCTT---GGA---------TTCTTGGGA---GCAGCAGGAAGCACTATGGGCGCAGCGTCACTG---ACGCTGACGGTACAGGCCAGACTATTATTGTCTGGTATAGTGCAACAGCAGAACAATCTGCTGAGGGCTATTGAGGCGCAACAACATCTGTTGCAACTCACGGTCTGGGGCATTAAACAGCTCCAGGCAAGA---GTCCTGGCTGTGGAAAGATATCTAAGGGATCAACAGCTCCTAGGGATTTGGGGTTGCTCTGGAAAACTCATCTGCACCACTACTGTGCCTTGGAACAGTAGTTGGAGT---------------------------AATAAATCTCTGGATGAAATTTGGAAT---AATATGACCTGGATGCAGTGGGATAGAGAAATTGAC------AATTACACAAACTTAATATACAGCTTACTTGAAGAATCGCAGAACCAGCAAGAGAAGAATGAAAAAGATTTATTAGAACTGGATAAGTGGGCAAGTTTGTGGAATTGGTTTGATATAACAAGCTGGCTGTGGTATATAAAAATATTCATAATGATAGTAGGTGGCTTGATAGGTTTAAGAATAATTTTTGCTGTACTTTCTATAGTGAATAGGGTTAGGCAGGGATACTCACCATTATCATTTCAGACCCTC---CTCCCAGCTCCGAGGGGA------CCCGACAGGCCCGAAGGAACCGAAGAAGAAGGTGGAGAGAGAGACAGAGGCAGATCAGTTCGATTAGTGCATGGATTCTCAGCACTCATCTGGGACGACCTGCGGAGCCTGTGCCTTTTCAGCTACCACCGCTTGAGAGACTTACTCTTGATTGCAGCGAGGATTGTAGAGATTCTGGGACGCAGG---------------GGGTGGGAAGCCCTCAAGTATTGGTGG---AATCTCCTGCAGTATTGG---------------------------------------------------AGTCAGGAACTAAAGAATAGTGCTATTAGCTTGCTTAATGCCACAGCTATCGCAGTAGCTGAGGGAACAGATAGGATTATAGGAGTAGTACAAAGA------------------ATTTGTAGAGCTATTATCCACATACCTACAAGAATAAGACAGGGCTTAGAAAGGGCTTTGCTATAA

2.SC11.Trinidad.EU576798 ATGACAGTGATGGAGATCAGGAGGAATTAC------TTG---------TGGAGATGGGGC------------------------ATCATGCTCCTTGGAATGTTGATGATC------------TATAGTGCTGCAGAACAA---GAACAAGGGTGGGTCACAGTCTATTATGGGGTACCTGTGTGGAAAGAAGCAACCACCACTCTATTCTGTGCATCAGATGCCAAAGCATATGACACAGAGGTACATAAT---GTCTGGGCCACACATGCCTGTGTACCCACAGATCCCAACCCACAAGAAGTAATGCTG---GAAAATGTGACAGAAAATTTTAACATGTGGAAAAATAACATGGTAGAACAGATGCATGAAGATATAATCAGTTTATGGGATCAAAGCTTAAAGCCATGTGTAAAATTAACCCCACTCTGTGTTACTTTAAATTGCAGTGATACCTGGAGAAATGATACTAATGCCACTGCC---------------------------------------------------------------------------------------AGTAGTTGGGAAAAGGGAGAGAAAGGAGAGATAAAAAACTGCTCTTTCAATGTCACC---GCAGGCATAAGAGAT------AAGGTGCAGAAAGAATATGCACTTTTTTATAAACTTGATGTAATACCAATAGATAATAATAATGGTAGTGAA------------------AATAGTAGC------------------TATGGTAGCTATAGATTGATAAATTGTAATACCTCAGTCATTACACAGGCTTGTCCAAAGGTATCCTTTGAGCCAATTCCCATACACTATTGTGCCCCGGCTGGTTTTGCGATTCTAAAGTGT---AATAATAAGAAGTTCAATGGAACAGGACCATGTACAAATGTTAGCACAGTACAATGTACACATGGAATTAGACCAGTAGTGTCAACTCAACTGCTGTTAAATGGCAGTCTAGCAGAAGAA---GAGGTAGTAATTAGATCTGAAAATTTCACGAACAATGCTAAAATTATAATAGTACATCTGAATAAATCTGTAGTAATTAATTGTACAAGACCCAACAACAATACAAGAAGAAGTATACATATAGGA------------CCAGGGAAA---GTATTTTAT---ACAGGGGAAATAATAGGAGATATAAGACAAGCACATTGTAACCTT------AGTAGAGCGGCATGGAATAACACTTTAGAACAGATAGTTATAAAATTAAGA---GAACAATTTGGG------------AATAAAACA---ATAGTCTTTAAGCAA---------TCCTCAGGAGGGGACCCAGAAATTGTAATGCACACTTTTAATTGTGGAGGGGAATTTTTCTACTGTAATGCAACACCACTGTTTAATAGTAGCTGGTATGTT---------AATGGTACTAAC------------------------------ACTACAGGGTCAGATGGAGAA---------------------------------------------AATATCACACTCCTATGCAGAATAAAACAAATTGTAAACATGTGGCAGGAAGTAGGCAAAGCAATGTATGCCCCTCCCATTAGAGGACAAATTACATGCTCATCAAAGATCACAGGGTTGCTATTAACAAGAGATGGTGGTAACATG------------AACGGGACC---------------------AACGACACC---GAGACCTTCAGACCTGGAGGAGGAGATATGAGGGACAATTGGAGA---AGTGAATTATATAAATATAAAGTAGTAAAAATTGAACCA---TTAGGAGTAGCACCC---ACCAAGGCAAAGAGAAGAGTGGTGCAGAGAGAA---AAAAGAGCAGTG---GGAACAATAGGA---GCTATG---TTCCTT---GGA---------TTCTTGGGA---GCAGCAGGAAGCACTATGGGCGCAGCGTCACTG---ACGCTGACGGTACAGGCCAGACTATTATTGTCTGGTATAGTGCAACAGCAGAACAATCTGCTGAGGGCTATTGAGGCGCAACAACATCTGTTGCAACTCACGGTCTGGGGCATTAAACAGCTCCAGGCAAGA---GTCCTGGCTGTGGAAAGATATCTAAGGGATCAACAGCTCCTAGGGATTTGGGGTTGCTCTGGAAAACTCATCTGCACCACTACTGTGCCTTGGAACAGTAGTTGGAGT---------------------------AATAAATCTCTGGATGAAATTTGGAAT---AATATGACCTGGATGCAGTGGGATAGAGAAATTGAC------AATTACACAAACTTAATATACAGCTTACTTGAAGAATCGCAGAACCAGCAAGAGAAGAATGAAAAAGATTTATTAGAACTGGATAAGTGGGCAAGTTTGTGGAATTGGTTTGATATAACAAGCTGGCTGTGGTATATAAAAATATTCATAATGATAGTAGGTGGCTTGATAGGTTTAAGAATAATTTTTGCTGTACTTTCTATAGTGAATAGGGTTAGGCAGGGATACTCACCATTATCATTTCAGACCCTC---CTCCCAGCTCCGAGGGGA------CCCGACAGGCCCGAAGGAACCGAAGAAGAAGGTGGAGAGAGAGACAGAGGCAGATCAGTTCGATTAGTGCATGGATTCTCAGCACTCATCTGGGACGACCTGCGGAGCCTGTGCCTTTTCAGCTACCACCGCTTGAGAGACTTACTCTTGATTGCAGCGAGGATTGTAGAGATTCTGGGACGCAGG---------------GGGTGGGAAGCCCTCAAGTATTGGTGG---AATCTCCTGCAGTATTGG---------------------------------------------------AGTCAGGAACTAAAGAATAGTGCTATTAGCTTGCTTAATGCCACAGCTATCGCAGTAGCTGAGGGAACAGATAGGATTATAGGAGTAGTACAAAGA------------------ATTTGTAGAGCTATTATCCACATACCTACAAGAATAAGACAGGGCTTAGAAAGGGCTTTGCTATAA

2.SC11.Trinidad.EU576801 ATGACAGTGATGGAGATCAGGAGGAATTAC------TTG---------TGGAGATGGGGC------------------------ATCATGCTCCTTGGAATGTTGATGATC------------TATAGTGCTGCAGAACAA---GAACAAGGGTGGGTCACAGTCTATTATGGGGTACCTGTGTGGAAAGAAGCAACCACCACTCTATTCTGTGCATCAGATGCCAAAGCATATGACACAGAGGTACATAAT---GTCTGGGCCACACATGCCTGTGTACCCACAGATCCCAACCCACAAGAAGTAATGCTG---GAAAATGTGACAGAAAATTTTAACATGTGGAAAAATAACATGGTAGAACAGATGCATGAAGATATAATCAGTTTATGGGATCAAAGCTTAAAGCCATGTGTAAAATTAACCCCACTCTGTGTTACTTTAAATTGCAGTGATACCTGGAGAAATGATACTAATGCCACTGCC---------------------------------------------------------------------------------------AGTAGTTGGGAAAAGGGAGAGAAAGGAGAGATAAAAAACTGCTCTTTCAATGTCACC---GCAGGCATAAGAGAT------AAGGTGCAGAAAGAATATGCACTTTTTTATAAACTTGATGTAATACCAATAGATAATAATAATGGTAGTGAA------------------AATAGTAGC------------------TATGGTAGCTATAGATTGATAAATTGTAATACCTCAGTCATTACACAGGCTTGTCCAAAGGTATCCTTTGAGCCAATTCCCATACACTATTGTGCCCCGGCTGGTTTTGCGATTCTAAAGTGT---AATAATAAGAAGTTCAATGGAACAGGACCATGTACAAATGTTAGCACAGTACAATGTACACATGGAATTAGACCAGTAGTGTCAACTCAACTGCTGTTAAATGGCAGTCTAGCAGAAGAA---GAGGTAGTAATTAGATCTGAAAATTTCACGAACAATGCTAAAATTATAATAGTACATCTGAATAAATCTGTAGTAATTAATTGTACAAGACCCAACAACAATACAAGAAGAAGTATACATATAGGA------------CCAGGGAAA---GTATTTTAT---ACAGGGGAAATAATAGGAGATATAAGACAAGCACATTGTAACCTT------AGTAGAGCGGCATGGAATAACACTTTAGAACAGATAGTTATAAAATTAAGA---GAACAATTTGGG------------AATAAAACA---ATAGTCTTTAAGCAA---------TCCTCAGGAGGGGACCCAGAAATTGTAATGCACACTTTTAATTGTGGAGGGGAATTTTTCTACTGTAATGCAACACCACTGTTTAATAGTAGCTGGTATGTT---------AATGGTACTAAC------------------------------ACTACAGGGTCAGATGGAGAA---------------------------------------------AATATCACACTCCTATGCAGAATAAAACAAATTGTAAACATGTGGCAGGAAGTAGGCAAAGCAATGTATGCCCCTCCCATTAGAGGACAAATTACATGCTCATCAAAGATCACAGGGTTGCTATTAACAAGAGATGGTGGTAACATG------------AACGGGACC---------------------AACGACACC---GAGACCTTCAGACCTGGAGGAGGAGATATGAGGGACAATTGGAGA---AGTGAATTATATAAATATAAAGTAGTAAAAATTGAACCA---TTAGGAGTAGCACCC---ACCAAGGCAAAGAGAAGAGTGGTGCAGAGAGAA---AAAAGAGCAGTG---GGAACAATAGGA---GCTATG---TTCCTT---GGA---------TTCTTGGGA---GCAGCAGGAAGCACTATGGGCGCAGCGTCACTG---ACGCTGACGGTACAGGCCAGACTATTATTGTCTGGTATAGTGCAACAGCAGAACAATCTGCTGAGGGCTATTGAGGCGCAACAACATCTGTTGCAACTCACGGTCTGGGGCATTAAACAGCTCCAGGCAAGA---GTCCTGGCTGTGGAAAGATATCTAAGGGATCAACAGCTCCTAGGGATTTGGGGTTGCTCTGGAAAACTCATCTGCACCACTACTGTGCCTTGGAACAGTAGTTGGAGT---------------------------AATAAATCTCTGGATGAAATTTGGAAT---AATATGACCTGGATGCAGTGGGATAGAGAAATTGAC------AATTACACAAACTTAATATACAGCTTACTTGAAGAATCGCAGAACCAGCAAGAGAAGAATGAAAAAGATTTATTAGAACTGGATAAGTGGGCAAGTTTGTGGAATTGGTTTGATATAACAAGCTGGCTGTGGTATATAAAAATATTCATAATGATAGTAGGTGGCTTGATAGGTTTAAGAATAATTTTTGCTGTACTTTCTATAGTGAATAGGGTTAGGCAGGGATACTCACCATTATCATTTCAGACCCTC---CTCCCAGCTCCGAGGGGA------CCCGACAGGCCCGAAGGAACCGAAGAAGAAGGTGGAGAGAGAGACAGAGGCAGATCAGTTCGATTAGTGCATGGATTCTCAGCACTCATCTGGGACGACCTGCGGAGCCTGTGCCTTTTCAGCTACCACCGCTTGAGAGACTTACTCTTGATTGCAGCGAGGATTGTAGAGATTCTGGGACGCAGG---------------GGGTGGGAAGCCCTCAAGTATTGGTGG---AATCTCCTGCAGTATTGG---------------------------------------------------AGTCAGGAACTAAAGAATAGTGCTATTAGCTTGCTTAATGCCACAGCTATCGCAGTAGCTGAGGGAACAGATAGGATTATAGGAGTAGTACAAAGA------------------ATTTGTAGAGCTATTATCCACATACCTACAAGAATAAGACAGGGCTTAGAAAGGGCTTTGCTATAA

2.SC11.Trinidad.EU576795 ATGACAGTGATGGAGATCAGGAGGAATTAC------TTG---------TGGAGATGGGGC------------------------ATCATGCTCCTTGGAATGTTGATGATC------------TATAGTGCTGCAGAACAA---GAACAAGGGTGGGTCACAGTCTATTATGGGGTACCTGTGTGGAAAGAAGCAACCACCACTCTATTCTGTGCATCAGATGCCAAAGCATATGACACAGAGGTACATAAT---GTCTGGGCCACACATGCCTGTGTACCCACAGATCCCAACCCACAAGAAGTAATGCTG---GAAAATGTGACAGAAAATTTTAACATGTGGAAAAATAACATGGTAGAACAGATGCATGAAGATATAATCAGTTTATGGGATCAAAGCTTAAAGCCATGTGTAAAATTAACCCCACTCTGTGTTACTTTAAATTGCAGTGATACCTGGAGAAATGATACTAATGCCACTGCC---------------------------------------------------------------------------------------AGTAGTTGGGAAAAGGGAGAGAAAGGAGAGATAAAAAACTGCTCTTTCAATGTCACC---GCAGGCATAAGAGAT------AAGGTGCAGAAAGAATATGCACTTTTTTATAAACTTGATGTAATACCAATAGATAATAATAATGGTAGTGAA------------------AATAGTAGC------------------TATGGTAGCTATAGATTGATAAATTGTAATACCTCAGTCATTACACAGGCTTGTCCAAAGGTATCCTTTGAGCCAATTCCCATACACTATTGTGCCCCGGCTGGTTTTGCGATTCTAAAGTGT---AATAATAAGAAGTTCAATGGAACAGGACCATGTACAAATGTTAGCACAGTACAATGTACACATGGAATTAGACCAGTAGTGTCAACTCAACTGCTGTTAAATGGCAGTCTAGCAGAAGAA---GAGGTAGTAATTAGATCTGAAAATTTCACGAACAATGCTAAAATTATAATAGTACATCTGAATAAATCTGTAGTAATTAATTGTACAAGACCCAACAACAATACAAGAAGAAGTATACATATAGGA------------CCAGGGAAA---GTATTTTAT---ACAGGGGAAATAATAGGAGATATAAGACAAGCACATTGTAACCTT------AGTAGAGCGGCATGGAATAACACTTTAGAACAGATAGTTATAAAATTAAGA---GAACAATTTGGG------------AATAAAACA---ATAGTCTTTAAGCAA---------TCCTCAGGAGGGGACCCAGAAATTGTAATGCACACTTTTAATTGTGGAGGGGAATTTTTCTACTGTAATGCAACACCACTGTTTAATAGTAGCTGGTATGTT---------AATGGTACTAAC------------------------------ACTACAGGGTCAGATGGAGAA---------------------------------------------AATATCACACTCCTATGCAGAATAAAACAAATTGTAAACATGTGGCAGGAAGTAGGCAAAGCAATGTATGCCCCTCCCATTAGAGGACAAATTACATGCTCATCAAAGATCACAGGGTTGCTATTAACAAGAGATGGTGGTAACATG------------AACGGGACC---------------------AACGACACC---GAGACCTTCAGACCTGGAGGAGGAGATATGAGGGACAATTGGAGA---AGTGAATTATATAAATATAAAGTAGTAAAAATTGAACCA---TTAGGAGTAGCACCC---ACCAAGGCAAAGAGAAGAGTGGTGCAGAGAGAA---AAAAGAGCAGTG---GGAACAATAGGA---GCTATG---TTCCTT---GGA---------TTCTTGGGA---GCAGCAGGAAGCACTATGGGCGCAGCGTCACTG---ACGCTGACGGTACAGGCCAGACTATTATTGTCTGGTATAGTGCAACAGCAGAACAATCTGCTGAGGGCTATTGAGGCGCAACAACATCTGTTGCAACTCACGGTCTGGGGCATTAAACAGCTCCAGGCAAGA---GTCCTGGCTGTGGAAAGATATCTAAGGGATCAACAGCTCCTAGGGATTTGGGGTTGCTCTGGAAAACTCATCTGCACCACTACTGTGCCTTGGAACAGTAGTTGGAGT---------------------------AATAAATCTCTGGATGAAATTTGGAAT---AATATGACCTGGATGCAGTGGGATAGAGAAATTGAC------AATTACACAAACTTAATATACAGCTTACTTGAAGAATCGCAGAACCAGCAAGAGAAGAATGAAAAAGATTTATTAGAACTGGATAAGTGGGCAAGTTTGTGGAATTGGTTTGATATAACAAGCTGGCTGTGGTATATAAAAATATTCATAATGATAGTAGGTGGCTTGATAGGTTTAAGAATAATTTTTGCTGTACTTTCTATAGTGAATAGGGTTAGGCAGGGATACTCACCATTATCATTTCAGACCCTC---CTCCCAGCTCCGAGGGGA------CCCGACAGGCCCGAAGGAACCGAAGAAGAAGGTGGAGAGAGAGACAGAGGCAGATCAGTTCGATTAGTGCATGGATTCTCAGCACTCATCTGGGACGACCTGCGGAGCCTGTGCCTTTTCAGCTACCACCGCTTGAGAGACTTACTCTTGATTGCAGCGAGGATTGTAGAGATTCTGGGACGCAGG---------------GGGTGGGAAGCCCTCAAGTATTGGTGG---AATCTCCTGCAGTATTGG---------------------------------------------------AGTCAGGAACTAAAGAATAGTGCTATTAGCTTGCTTAATGCCACAGCTATCGCAGTAGCTGAGGGAACAGATAGGATTATAGGAGTAGTACAAAGA------------------ATTTGTAGAGCTATTATCCACATACCTACAAGAATAAGACAGGGCTTAGAAAGGGCTTTGCTATAA

2.SC11.Trinidad.EU576792 ATGACAGTGATGGAGATCAGGAGGAATTAC------TTG---------TGGAGATGGGGC------------------------ATCATGCTCCTTGGAATGTTGATGATC------------TATAGTGCTGCAGAACAA---GAACAAGGGTGGGTCACAGTCTATTATGGGGTACCTGTGTGGAAAGAAGCAACCACCACTCTATTCTGTGCATCAGATGCCAAAGCATATGACACAGAGGTACATAAT---GTCTGGGCCACACATGCCTGTGTACCCACAGATCCCAACCCACAAGAAGTAATGCTG---GAAAATGTGACAGAAAATTTTAACATGTGGAAAAATAACATGGTAGAACAGATGCATGAAGATATAATCAGTTTATGGGATCAAAGCTTAAAGCCATGTGTAAAATTAACCCCACTCTGTGTTACTTTAAATTGCAGTGATACCTGGAGAAATGATACTAATGCCACTGCC---------------------------------------------------------------------------------------AGTAGTTGGGAAAAGGGAGAGAAAGGAGAGATAAAAAACTGCTCTTTCAATGTCACC---GCAGGCATAAGAGAT------AAGGTGCAGAAAGAATATGCACTTTTTTATAAACTTGATGTAATACCAATAGATAATAATAATGGTAGTGAA------------------AATAGTAGC------------------TATGGTAGCTATAGATTGATAAATTGTAATACCTCAGTCATTACACAGGCTTGTCCAAAGGTATCCTTTGAGCCAATTCCCATACACTATTGTGCCCCGGCTGGTTTTGCGATTCTAAAGTGT---AATAATAAGAAGTTCAATGGAACAGGACCATGTACAAATGTTAGCACAGTACAATGTACACATGGAATTAGACCAGTAGTGTCAACTCAACTGCTGTTAAATGGCAGTCTAGCAGAAGAA---GAGGTAGTAATTAGATCTGAAAATTTCACGAACAATGCTAAAATTATAATAGTACATCTGAATAAATCTGTAGTAATTAATTGTACAAGACCCAACAACAATACAAGAAGAAGTATACATATAGGA------------CCAGGGAAA---GTATTTTAT---ACAGGGGAAATAATAGGAGATATAAGACAAGCACATTGTAACCTT------AGTAGAGCGGCATGGAATAACACTTTAGAACAGATAGTTATAAAATTAAGA---GAACAATTTGGG------------AATAAAACA---ATAGTCTTTAAGCAA---------TCCTCAGGAGGGGACCCAGAAATTGTAATGCACACTTTTAATTGTGGAGGGGAATTTTTCTACTGTAATGCAACACCACTGTTTAATAGTAGCTGGTATGTT---------AATGGTACTAAC------------------------------ACTACAGGGTCAGATGGAGAA---------------------------------------------AATATCACACTCCTATGCAGAATAAAACAAATTGTAAACATGTGGCAGGAAGTAGGCAAAGCAATGTATGCCCCTCCCATTAGAGGACAAATTACATGCTCATCAAAGATCACAGGGTTGCTATTAACAAGAGATGGTGGTAACATG------------AACGGGACC---------------------AACGACACC---GAGACCTTCAGACCTGGAGGAGGAGATATGAGGGACAATTGGAGA---AGTGAATTATATAAATATAAAGTAGTAAAAATTGAACCA---TTAGGAGTAGCACCC---ACCAAGGCAAAGAGAAGAGTGGTGCAGAGAGAA---AAAAGAGCAGTG---GGAACAATAGGA---GCTATG---TTCCTT---GGA---------TTCTTGGGA---GCAGCAGGAAGCACTATGGGCGCAGCGTCACTG---ACGCTGACGGTACAGGCCAGACTATTATTGTCTGGTATAGTGCAACAGCAGAACAATCTGCTGAGGGCTATTGAGGCGCAACAACATCTGTTGCAACTCACGGTCTGGGGCATTAAACAGCTCCAGGCAAGA---GTCCTGGCTGTGGAAAGATATCTAAGGGATCAACAGCTCCTAGGGATTTGGGGTTGCTCTGGAAAACTCATCTGCACCACTACTGTGCCTTGGAACAGTAGTTGGAGT---------------------------AATAAATCTCTGGATGAAATTTGGAAT---AATATGACCTGGATGCAGTGGGATAGAGAAATTGAC------AATTACACAAACTTAATATACAGCTTACTTGAAGAATCGCAGAACCAGCAAGAGAAGAATGAAAAAGATTTATTAGAACTGGATAAGTGGGCAAGTTTGTGGAATTGGTTTGATATAACAAGCTGGCTGTGGTATATAAAAATATTCATAATGATAGTAGGTGGCTTGATAGGTTTAAGAATAATTTTTGCTGTACTTTCTATAGTGAATAGGGTTAGGCAGGGATACTCACCATTATCATTTCAGACCCTC---CTCCCAGCTCCGAGGGGA------CCCGACAGGCCCGAAGGAACCGAAGAAGAAGGTGGAGAGAGAGACAGAGGCAGATCAGTTCGATTAGTGCATGGATTCTCAGCACTCATCTGGGACGACCTGCGGAGCCTGTGCCTTTTCAGCTACCACCGCTTGAGAGACTTACTCTTGATTGCAGCGAGGATTGTAGAGATTCTGGGACGCAGG---------------GGGTGGGAAGCCCTCAAGTATTGGTGG---AATCTCCTGCAGTATTGG---------------------------------------------------AGTCAGGAACTAAAGAATAGTGCTATTAGCTTGCTTAATGCCACAGCTATCGCAGTAGCTGAGGGAACAGATAGGATTATAGGAGTAGTACAAAGA------------------ATTTGTAGAGCTATTATCCACATACCTACAAGAATAAGACAGGGCTTAGAAAGGGCTTTGCTATAA

2.SC11.Trinidad.EU576789 ATGACAGTGATGGAGATCAGGAGGAATTAC------TTG---------TGGAGATGGGGC------------------------ATCATGCTCCTTGGAATGTTGATGATC------------TATAGTGCTGCAGAACAA---GAACAAGGGTGGGTCACAGTCTATTATGGGGTACCTGTGTGGAAAGAAGCAACCACCACTCTATTCTGTGCATCAGATGCCAAAGCATATGACACAGAGGTACATAAT---GTCTGGGCCACACATGCCTGTGTACCCACAGATCCCAACCCACAAGAAGTAATGCTG---GAAAATGTGACAGAAAATTTTAACATGTGGAAAAATAACATGGTAGAACAGATGCATGAAGATATAATCAGTTTATGGGATCAAAGCTTAAAGCCATGTGTAAAATTAACCCCACTCTGTGTTACTTTAAATTGCAGTGATACCTGGAGAAATGATACTAATGCCACTGCC---------------------------------------------------------------------------------------AGTAGTTGGGAAAAGGGAGAGAAAGGAGAGATAAAAAACTGCTCTTTCAATGTCACC---GCAGGCATAAGAGAT------AAGGTGCAGAAAGAATATGCACTTTTTTATAAACTTGATGTAATACCAATAGATAATAATAATGGTAGTGAA------------------AATAGTAGC------------------TATGGTAGCTATAGATTGATAAATTGTAATACCTCAGTCATTACACAGGCTTGTCCAAAGGTATCCTTTGAGCCAATTCCCATACACTATTGTGCCCCGGCTGGTTTTGCGATTCTAAAGTGT---AATAATAAGAAGTTCAATGGAACAGGACCATGTACAAATGTTAGCACAGTACAATGTACACATGGAATTAGACCAGTAGTGTCAACTCAACTGCTGTTAAATGGCAGTCTAGCAGAAGAA---GAGGTAGTAATTAGATCTGAAAATTTCACGAACAATGCTAAAATTATAATAGTACATCTGAATAAATCTGTAGTAATTAATTGTACAAGACCCAACAACAATACAAGAAGAAGTATACATATAGGA------------CCAGGGAAA---GTATTTTAT---ACAGGGGAAATAATAGGAGATATAAGACAAGCACATTGTAACCTT------AGTAGAGCGGCATGGAATAACACTTTAGAACAGATAGTTATAAAATTAAGA---GAACAATTTGGG------------AATAAAACA---ATAGTCTTTAAGCAA---------TCCTCAGGAGGGGACCCAGAAATTGTAATGCACACTTTTAATTGTGGAGGGGAATTTTTCTACTGTAATGCAACACCACTGTTTAATAGTAGCTGGTATGTT---------AATGGTACTAAC------------------------------ACTACAGGGTCAGATGGAGAA---------------------------------------------AATATCACACTCCTATGCAGAATAAAACAAATTGTAAACATGTGGCAGGAAGTAGGCAAAGCAATGTATGCCCCTCCCATTAGAGGACAAATTACATGCTCATCAAAGATCACAGGGTTGCTATTAACAAGAGATGGTGGTAACATG------------AACGGGACC---------------------AACGACACC---GAGACCTTCAGACCTGGAGGAGGAGATATGAGGGACAATTGGAGA---AGTGAATTATATAAATATAAAGTAGTAAAAATTGAACCA---TTAGGAGTAGCACCC---ACCAAGGCAAAGAGAAGAGTGGTGCAGAGAGAA---AAAAGAGCAGTG---GGAACAATAGGA---GCTATG---TTCCTT---GGA---------TTCTTGGGA---GCAGCAGGAAGCACTATGGGCGCAGCGTCACTG---ACGCTGACGGTACAGGCCAGACTATTATTGTCTGGTATAGTGCAACAGCAGAACAATCTGCTGAGGGCTATTGAGGCGCAACAACATCTGTTGCAACTCACGGTCTGGGGCATTAAACAGCTCCAGGCAAGA---GTCCTGGCTGTGGAAAGATATCTAAGGGATCAACAGCTCCTAGGGATTTGGGGTTGCTCTGGAAAACTCATCTGCACCACTACTGTGCCTTGGAACAGTAGTTGGAGT---------------------------AATAAATCTCTGGATGAAATTTGGAAT---AATATGACCTGGATGCAGTGGGATAGAGAAATTGAC------AATTACACAAACTTAATATACAGCTTACTTGAAGAATCGCAGAACCAGCAAGAGAAGAATGAAAAAGATTTATTAGAACTGGATAAGTGGGCAAGTTTGTGGAATTGGTTTGATATAACAAGCTGGCTGTGGTATATAAAAATATTCATAATGATAGTAGGTGGCTTGATAGGTTTAAGAATAATTTTTGCTGTACTTTCTATAGTGAATAGGGTTAGGCAGGGATACTCACCATTATCATTTCAGACCCTC---CTCCCAGCTCCGAGGGGA------CCCGACAGGCCCGAAGGAACCGAAGAAGAAGGTGGAGAGAGAGACAGAGGCAGATCAGTTCGATTAGTGCATGGATTCTCAGCACTCATCTGGGACGACCTGCGGAGCCTGTGCCTTTTCAGCTACCACCGCTTGAGAGACTTACTCTTGATTGCAGCGAGGATTGTAGAGATTCTGGGACGCAGG---------------GGGTGGGAAGCCCTCAAGTATTGGTGG---AATCTCCTGCAGTATTGG---------------------------------------------------AGTCAGGAACTAAAGAATAGTGCTATTAGCTTGCTTAATGCCACAGCTATCGCAGTAGCTGAGGGAACAGATAGGATTATAGGAGTAGTACAAAGA------------------ATTTGTAGAGCTATTATCCACATACCTACAAGAATAAGACAGGGCTTAGAAAGGGCTTTGCTATAA

2.SC11.Trinidad.EU576793 ATGACAGTGATGGAGATCAGGAGGAATTAC------TTG---------TGGAGATGGGGC------------------------ATCATGCTCCTTGGAATGTTGATGATC------------TATAGTGCTGCAGAACAA---GAACAAGGGTGGGTCACAGTCTATTATGGGGTACCTGTGTGGAAAGAAGCAACCACCACTCTATTCTGTGCATCAGATGCCAAAGCATATGACACAGAGGTACATAAT---GTCTGGGCCACACATGCCTGTGTACCCACAGATCCCAACCCACAAGAAGTAATGCTG---GAAAATGTGACAGAAAATTTTAACATGTGGAAAAATAACATGGTAGAACAGATGCATGAAGATATAATCAGTTTATGGGATCAAAGCTTAAAGCCATGTGTAAAATTAACCCCACTCTGTGTTACTTTAAATTGCAGTGATACCTGGAGAAATGATACTAATGCCACTGCC---------------------------------------------------------------------------------------AGTAGTTGGGAAAAGGGAGAGAAAGGAGAGATAAAAAACTGCTCTTTCAATGTCACC---GCAGGCATAAGAGAT------AAGGTGCAGAAAGAATATGCACTTTTTTATAAACTTGATGTAATACCAATAGATAATAATAATGGTAGTGAA------------------AATAGTAGC------------------TATGGTAGCTATAGATTGATAAATTGTAATACCTCAGTCATTACACAGGCTTGTCCAAAGGTATCCTTTGAGCCAATTCCCATACACTATTGTGCCCCGGCTGGTTTTGCGATTCTAAAGTGT---AATAATAAGAAGTTCAATGGAACAGGACCATGTACAAATGTTAGCACAGTACAATGTACACATGGAATTAGACCAGTAGTGTCAACTCAACTGCTGTTAAATGGCAGTCTAGCAGAAGAA---GAGGTAGTAATTAGATCTGAAAATTTCACGAACAATGCTAAAATTATAATAGTACATCTGAATAAATCTGTAGTAATTAATTGTACAAGACCCAACAACAATACAAGAAGAAGTATACATATAGGA------------CCAGGGAAA---GTATTTTAT---ACAGGGGAAATAATAGGAGATATAAGACAAGCACATTGTAACCTT------AGTAGAGCGGCATGGAATAACACTTTAGAACAGATAGTTATAAAATTAAGA---GAACAATTTGGG------------AATAAAACA---ATAGTCTTTAAGCAA---------TCCTCAGGAGGGGACCCAGAAATTGTAATGCACACTTTTAATTGTGGAGGGGAATTTTTCTACTGTAATGCAACACCACTGTTTAATAGTAGCTGGTATGTT---------AATGGTACTAAC------------------------------ACTACAGGGTCAGATGGAGAA---------------------------------------------AATATCACACTCCTATGCAGAATAAAACAAATTGTAAACATGTGGCAGGAAGTAGGCAAAGCAATGTATGCCCCTCCCATTAGAGGACAAATTACATGCTCATCAAAGATCACAGGGTTGCTATTAACAAGAGATGGTGGTAACATG------------AACGGGACC---------------------AACGACACC---GAGACCTTCAGACCTGGAGGAGGAGATATGAGGGACAATTGGAGA---AGTGAATTATATAAATATAAAGTAGTAAAAATTGAACCA---TTAGGAGTAGCACCC---ACCAAGGCAAAGAGAAGAGTGGTGCAGAGAGAA---AAAAGAGCAGTG---GGAACAATAGGA---GCTATG---TTCCTT---GGA---------TTCTTGGGA---GCAGCAGGAAGCACTATGGGCGCAGCGTCACTG---ACGCTGACGGTACAGGCCAGACTATTATTGTCTGGTATAGTGCAACAGCAGAACAATCTGCTGAGGGCTATTGAGGCGCAACAACATCTGTTGCAACTCACGGTCTGGGGCATTAAACAGCTCCAGGCAAGA---GTCCTGGCTGTGGAAAGATATCTAAGGGATCAACAGCTCCTAGGGATTTGGGGTTGCTCTGGAAAACTCATCTGCACCACTACTGTGCCTTGGAACAGTAGTTGGAGT---------------------------AATAAATCTCTGGATGAAATTTGGAAT---AATATGACCTGGATGCAGTGGGATAGAGAAATTGAC------AATTACACAAACTTAATATACAGCTTACTTGAAGAATCGCAGAACCAGCAAGAGAAGAATGAAAAAGATTTATTAGAACTGGATAAGTGGGCAAGTTTGTGGAATTGGTTTGATATAACAAGCTGGCTGTGGTATATAAAAATATTCATAATGATAGTAGGTGGCTTGATAGGTTTAAGAATAATTTTTGCTGTACTTTCTATAGTGAATAGGGTTAGGCAGGGATACTCACCATTATCATTTCAGACCCTC---CTCCCAGCTCCGAGGGGA------CCCGACAGGCCCGAAGGAACCGAAGAAGAAGGTGGAGAGAGAGACAGAGGCAGATCAGTTCGATTAGTGCATGGATTCTCAGCACTCATCTGGGACGACCTGCGGAGCCTGTGCCTTTTCAGCTACCACCGCTTGAGAGACTTACTCTTGATTGCAGCGAGGATTGTAGAGATTCTGGGACGCAGG---------------GGGTGGGAAGCCCTCAAGTATTGGTGG---AATCTCCTGCAGTATTGG---------------------------------------------------AGTCAGGAACTAAAGAATAGTGCTATTAGCTTGCTTAATGCCACAGCTATCGCAGTAGCTGAGGGAACAGATAGGATTATAGGAGTAGTACAAAGA------------------ATTTGTAGAGCTATTATCCACATACCTACAAGAATAAGACAGGGCTTAGAAAGGGCTTTGCTATAA

2.SC11.Trinidad.EU576803 ATGACAGTGATGGAGATCAGGAGGAATTAC------TTG---------TGGAGATGGGGC------------------------ATCATGCTCCTTGGAATGTTGATGATC------------TATAGTGCTGCAGAACAA---GAACAAGGGTGGGTCACAGTCTATTATGGGGTACCTGTGTGGAAAGAAGCAACCACCACTCTATTCTGTGCATCAGATGCCAAAGCATATGACACAGAGGTACATAAT---GTCTGGGCCACACATGCCTGTGTACCCACAGATCCCAACCCACAAGAAGTAATGCTG---GAAAATGTGACAGAAAATTTTAACATGTGGAAAAATAACATGGTAGAACAGATGCATGAAGATATAATCAGTTTATGGGATCAAAGCTTAAAGCCATGTGTAAAATTAACCCCACTCTGTGTTACTTTAAATTGCAGTGATACCTGGAGAAATGATACTAATGCCACTGCC---------------------------------------------------------------------------------------AGTAGTTGGGAAAAGGGAGAGAAAGGAGAGATAAAAAACTGCTCTTTCAATGTCACC---GCAGGCATAAGAGAT------AAGGTGCAGAAAGAATATGCACTTTTTTATAAACTTGATGTAATACCAATAGATAATAATAATGGTAGTGAA------------------AATAGTAGC------------------TATGGTAGCTATAGATTGATAAATTGTAATACCTCAGTCATTACACAGGCTTGTCCAAAGGTATCCTTTGAGCCAATTCCCATACACTATTGTGCCCCGGCTGGTTTTGCGATTCTAAAGTGT---AATAATAAGAAGTTCAATGGAACAGGACCATGTACAAATGTTAGCACAGTACAATGTACACATGGAATTAGACCAGTAGTGTCAACTCAACTGCTGTTAAATGGCAGTCTAGCAGAAGAA---GAGGTAGTAATTAGATCTGAAAATTTCACGAACAATGCTAAAATTATAATAGTACATCTGAATAAATCTGTAGTAATTAATTGTACAAGACCCAACAACAATACAAGAAGAAGTATACATATAGGA------------CCAGGGAAA---GTATTTTAT---ACAGGGGAAATAATAGGAGATATAAGACAAGCACATTGTAACCTT------AGTAGAGCGGCATGGAATAACACTTTAGAACAGATAGTTATAAAATTAAGA---GAACAATTTGGG------------AATAAAACA---ATAGTCTTTAAGCAA---------TCCTCAGGAGGGGACCCAGAAATTGTAATGCACACTTTTAATTGTGGAGGGGAATTTTTCTACTGTAATGCAACACCACTGTTTAATAGTAGCTGGTATGTT---------AATGGTACTAAC------------------------------ACTACAGGGTCAGATGGAGAA---------------------------------------------AATATCACACTCCTATGCAGAATAAAACAAATTGTAAACATGTGGCAGGAAGTAGGCAAAGCAATGTATGCCCCTCCCATTAGAGGACAAATTACATGCTCATCAAAGATCACAGGGTTGCTATTAACAAGAGATGGTGGTAACATG------------AACGGGACC---------------------AACGACACC---GAGACCTTCAGACCTGGAGGAGGAGATATGAGGGACAATTGGAGA---AGTGAATTATATAAATATAAAGTAGTAAAAATTGAACCA---TTAGGAGTAGCACCC---ACCAAGGCAAAGAGAAGAGTGGTGCAGAGAGAA---AAAAGAGCAGTG---GGAACAATAGGA---GCTATG---TTCCTT---GGA---------TTCTTGGGA---GCAGCAGGAAGCACTATGGGCGCAGCGTCACTG---ACGCTGACGGTACAGGCCAGACTATTATTGTCTGGTATAGTGCAACAGCAGAACAATCTGCTGAGGGCTATTGAGGCGCAACAACATCTGTTGCAACTCACGGTCTGGGGCATTAAACAGCTCCAGGCAAGA---GTCCTGGCTGTGGAAAGATATCTAAGGGATCAACAGCTCCTAGGGATTTGGGGTTGCTCTGGAAAACTCATCTGCACCACTACTGTGCCTTGGAACAGTAGTTGGAGT---------------------------AATAAATCTCTGGATGAAATTTGGAAT---AATATGACCTGGATGCAGTGGGATAGAGAAATTGAC------AATTACACAAACTTAATATACAGCTTACTTGAAGAATCGCAGAACCAGCAAGAGAAGAATGAAAAAGATTTATTAGAACTGGATAAGTGGGCAAGTTTGTGGAATTGGTTTGATATAACAAGCTGGCTGTGGTATATAAAAATATTCATAATGATAGTAGGTGGCTTGATAGGTTTAAGAATAATTTTTGCTGTACTTTCTATAGTGAATAGGGTTAGGCAGGGATACTCACCATTATCATTTCAGACCCTC---CTCCCAGCTCCGAGGGGA------CCCGACAGGCCCGAAGGAACCGAAGAAGAAGGTGGAGAGAGAGACAGAGGCAGATCAGTTCGATTAGTGCATGGATTCTCAGCACTCATCTGGGACGACCTGCGGAGCCTGTGCCTTTTCAGCTACCACCGCTTGAGAGACTTACTCTTGATTGCAGCGAGGATTGTAGAGATTCTGGGACGCAGG---------------GGGTGGGAAGCCCTCAAGTATTGGTGG---AATCTCCTGCAGTATTGG---------------------------------------------------AGTCAGGAACTAAAGAATAGTGCTATTAGCTTGCTTAATGCCACAGCTATCGCAGTAGCTGAGGGAACAGATAGGATTATAGGAGTAGTACAAAGA------------------ATTTGTAGAGCTATTATCCACATACCTACAAGAATAAGACAGGGCTTAGAAAGGGCTTTGCTATAA

2.SC11.Trinidad.EU576799 ATGACAGTGATGGAGATCAGGAGGAATTAC------TTG---------TGGAGATGGGGC------------------------ATCATGCTCCTTGGAATGTTGATGATC------------TATAGTGCTGCAGAACAA---GAACAAGGGTGGGTCACAGTCTATTATGGGGTACCTGTGTGGAAAGAAGCAACCACCACTCTATTCTGTGCATCAGATGCCAAAGCATATGACACAGAGGTACATAAT---GTCTGGGCCACACATGCCTGTGTACCCACAGATCCCAACCCACAAGAAGTAATGCTG---GAAAATGTGACAGAAAATTTTAACATGTGGAAAAATAACATGGTAGAACAGATGCATGAAGATATAATCAGTTTATGGGATCAAAGCTTAAAGCCATGTGTAAAATTAACCCCACTCTGTGTTACTTTAAATTGCAGTGATACCTGGAGAAATGATACTAATGCCACTGCC---------------------------------------------------------------------------------------AGTAGTTGGGAAAAGGGAGAGAAAGGAGAGATAAAAAACTGCTCTTTCAATGTCACC---GCAGGCATAAGAGAT------AAGGTGCAGAAAGAATATGCACTTTTTTATAAACTTGATGTAATACCAATAGATAATAATAATGGTAGTGAA------------------AATAGTAGC------------------TATGGTAGCTATAGATTGATAAATTGTAATACCTCAGTCATTACACAGGCTTGTCCAAAGGTATCCTTTGAGCCAATTCCCATACACTATTGTGCCCCGGCTGGTTTTGCGATTCTAAAGTGT---AATAATAAGAAGTTCAATGGAACAGGACCATGTACAAATGTTAGCACAGTACAATGTACACATGGAATTAGACCAGTAGTGTCAACTCAACTGCTGTTAAATGGCAGTCTAGCAGAAGAA---GAGGTAGTAATTAGATCTGAAAATTTCACGAACAATGCTAAAATTATAATAGTACATCTGAATAAATCTGTAGTAATTAATTGTACAAGACCCAACAACAATACAAGAAGAAGTATACATATAGGA------------CCAGGGAAA---GTATTTTAT---ACAGGGGAAATAATAGGAGATATAAGACAAGCACATTGTAACCTT------AGTAGAGCGGCATGGAATAACACTTTAGAACAGATAGTTATAAAATTAAGA---GAACAATTTGGG------------AATAAAACA---ATAGTCTTTAAGCAA---------TCCTCAGGAGGGGACCCAGAAATTGTAATGCACACTTTTAATTGTGGAGGGGAATTTTTCTACTGTAATGCAACACCACTGTTTAATAGTAGCTGGTATGTT---------AATGGTACTAAC------------------------------ACTACAGGGTCAGATGGAGAA---------------------------------------------AATATCACACTCCTATGCAGAATAAAACAAATTGTAAACATGTGGCAGGAAGTAGGCAAAGCAATGTATGCCCCTCCCATTAGAGGACAAATTACATGCTCATCAAAGATCACAGGGTTGCTATTAACAAGAGATGGTGGTAACATG------------AACGGGACC---------------------AACGACACC---GAGACCTTCAGACCTGGAGGAGGAGATATGAGGGACAATTGGAGA---AGTGAATTATATAAATATAAAGTAGTAAAAATTGAACCA---TTAGGAGTAGCACCC---ACCAAGGCAAAGAGAAGAGTGGTGCAGAGAGAA---AAAAGAGCAGTG---GGAACAATAGGA---GCTATG---TTCCTT---GGA---------TTCTTGGGA---GCAGCAGGAAGCACTATGGGCGCAGCGTCACTG---ACGCTGACGGTACAGGCCAGACTATTATTGTCTGGTATAGTGCAACAGCAGAACAATCTGCTGAGGGCTATTGAGGCGCAACAACATCTGTTGCAACTCACGGTCTGGGGCATTAAACAGCTCCAGGCAAGA---GTCCTGGCTGTGGAAAGATATCTAAGAGATCAACAGCTCCTAGGGATTTGGGGTTGCTCTGGAAAACTCATCTGCACCACTACTGTGCCTTGGAACAGTAGTTGGAGT---------------------------AATAAATCTCTGGATGAAATTTGGAAT---AATATGACCTGGATGCAGTGGGATAGAGAAATTGAC------AATTACACAAACTTAATATACAGCTTACTTGAAGAATCGCAGAACCAGCAAGAGAAGAATGAAAAAGATTTATTAGAACTGGATAAGTGGGCAAGTTTGTGGAATTGGTTTGATATAACAAGCTGGCTGTGGTATATAAAAATATTCATAATGATAGTAGGTGGCTTGATAGGTTTAAGAATAATTTTTGCTGTACTTTCTATAGTGAATAGGGTTAGGCAGGGATACTCACCATTATCATTTCAGACCCTC---CTCCCAGCTCCGAGGGGA------CCCGACAGGCCCGAAGGAACCGAAGAAGAAGGTGGAGAGAGAGACAGAGGCAGATCAGTTCGATTAGTGCATGGATTCTCAGCACTCATCTGGGACGACCTGCGGAGCCTGTGCCTTTTCAGCTACCACCGCTTGAGAGACTTACTCTTGATTGCAGCGAGGATTGTAGAGATTCTGGGACGCAGG---------------GGGTGGGAAGCCCTCAAGTATTGGTGG---AATCTCCTGCAGTATTGG---------------------------------------------------AGTCAGGAACTAAAGAATAGTGCTATTAGCTTGCTTAATGCCACAGCTATCGCAGTAGCTGAGGGAACAGATAGGATTATAGGAGTAGTACAAAGA------------------ATTTGTAGAGCTATTATCCACATACCTACAAGAATAAGACAGGGCTTAGAAAGGGCTTTGCTATAA

2.SC11.Trinidad.EU576804 ATGACAGTGATGGAGATCAGGAGGAATTAC------TTG---------TGGAGATGGGGC------------------------ATCATGCTCCTTGGAATGTTGATGATC------------TATAGTGCTGCAGAACAA---GAACAAGGGTGGGTCACAGTCTATTATGGGGTACCTGTGTGGAAAGAAGCAACCACCACTCTATTCTGTGCATCAGATGCCAAAGCATATGACACAGAGGTACATAAT---GTCTGGGCCACACATGCCTGTGTACCCACAGATCCCAACCCACAAGAAGTAATGCTG---GAAAATGTGACAGAAAATTTTAACATGTGGAAAAATAACATGGTAGAACAGATGCATGAAGATATAATCAGTTTATGGGATCAAAGCTTAAAGCCATGTGTAAAATTAACCCCACTCTGTGTTACTTTAAATTGCAGTGATACCTGGAGAAATGATACTAATGCCACTGCC---------------------------------------------------------------------------------------AGTAGTTGGGAAAAGGGAGAGAAAGGAGAGATAAAAAACTGCTCTTTCAATGTCACC---GCAGGCATAAGAGAT------AAGGTGCAGAAAGAATATGCACTTTTTTATAAACTTGATGTAATACCAATAGATAATAATAATGGTAGTGAA------------------AATAGTAGC------------------TATGGTAGCTATAGATTGATAAATTGTAATACCTCAGTCATTACACAGGCTTGTCCAAAGGTATCCTTTGAGCCAATTCCCATACACTATTGTGCCCCGGCTGGTTTTGCGATTCTAAAGTGT---AATAATAAGAAGTTCAATGGAACAGGACCATGTACAAATGTTAGCACAGTACAATGTACACATGGAATTAGACCAGTAGTGTCAACTCAACTGCTGTTAAATGGCAGTCTAGCAGAAGAA---GAGGTAGTAATTAGATCTGAAAATTTCACGAACAATGCTAAAATTATAATAGTACATCTGAATAAATCTGTAGTAATTAATTGTACAAGACCCAACAACAATACAAGAAGAAGTATACATATAGGA------------CCAGGGAAA---GTATTTTAT---ACAGGGGAAATAATAGGAGATATAAGACAAGCACATTGTAACCTT------AGTAGAGCGGCATGGAATAACACTTTAGAACAGATAGTTATAAAATTAAGA---GAACAATTTGGG------------AATAAAACA---ATAGTCTTTAAGCAA---------TCCTCAGGAGGGGACCCAGAAATTGTAATGCACACTTTTAATTGTGGAGGGGAATTTTTCTACTGTAATGCAACACCACTGTTTAATAGTAGCTGGTATGTT---------AATGGTACTAAC------------------------------ACTACAGGGTCAGATGGAGAA---------------------------------------------AATATCACACTCCTATGCAGAATAAAACAAATTGTAAACATGTGGCAGGAAGTAGGCAAAGCAATGTATGCCCCTCCCATTAGAGGACAAATTACATGCTCATCAAAGATCACAGGGTTGCTATTAACAAGAGATGGTGGTAACATG------------AACGGGACC---------------------AACGACACC---GAGACCTTCAGACCTGGAGGAGGAGATATGAGGGACAATTGGAGA---AGTGAATTATATAAATATAAAGTAGTAAAAATTGAACCA---TTAGGAGTAGCACCC---ACCAAGGCAAAGAGAAGAGTGGTGCAGAGAGAA---AAAAGAGCAGTG---GGAACAATAGGA---GCTATG---TTCCTT---GGA---------TTCTTGGGA---GCAGCAGGAAGCACTATGGGCGCAGCGTCACTG---ACGCTGACGGTACAGGCCAGACTATTATTGTCTGGTATAGTGCAACAGCAGAACAATCTGCTGAGGGCTATTGAGGCGCAACAACATCTGTTGCAACTCACGGTCTGGGGCATTAAACAGCTCCAGGCAAGA---GTCCTGGCTGTGGAAAGATATCTAAGGGATCAACAGCTCCTAGGGATTTGGGGTTGCTCTGGAAAACTCATCTGCACCACTACTGTGCCTTGGAACAGTAGTTGGAGT---------------------------AATAAATCTCTGGATGAAATTTGGAAT---AATATGACCTGGATGCAGTGGGATAGAGAAATTGAC------AATTACACAAACTTAATATACAGCTTACTTGAAGAATCGCAGAACCAGCAAGAGAAGAATGAAAAAGATTTATTAGAACTGGATAAGTGGGCAAGTTTGTGGAATTGGTTTGATATAACAAGCTGGCTGTGGTATATAAAAATATTCATAATGATAGTAGGTGGCTTGATAGGTTTAAGAATAATTTTTGCTGTACTTTCTATAGTGAATAGGGTTAGGCAGGGATACTCACCATTATCATTTCAGACCCTC---CTCCCAGCTCCGAGGGGA------CCCGACAGGCCCGAAGGAACCGAAGAAGAAGGTGGAGAGAGAGACAGAGGCAGATCAGTTCGATTAGTGCATGGATTCTCAGCACTCATCTGGGACGACCTGCGGAGCCTGTGCCTTTTCAGCTACCACCGCTTGAGAGACTTACTCTTGATTGCAGCGAGGATTGTAGAGATTCTGGGACGCAGG---------------GGGTGGGAAGCCCTCAAGTATTGGTGG---AATCTCCTGCAGTATTGG---------------------------------------------------AGTCAGGAACTAAAGAATAGTGCTATTAGCTTGCTTAATGCCACAGCTATCGCAGTAGCTGAGGGAACAGATAGGATTATAGGAGTAGTACAAAGA------------------ATTTGTAGAGCTATTATCCACATACCTACAAGAATAAGACAGGGCTTAGAAAGGGCTTTGCTATAA

2.SC11.Trinidad.EU576794 ATGACAGTGATGGAGATCAGGAGGAATTAC------TTG---------TGGAGATGGGGC------------------------ATCATGCTCCTTGGAATGTTGATGATC------------TATAGTGCTGCAGAACAA---GAACAAGGGTGGGTCACAGTCTATTATGGGGTACCTGTGTGGAAAGAAGCAACCACCACTCTATTCTGTGCATCAGATGCCAAAGCATATGACACAGAGGTACATAAT---GTCTGGGCCACACATGCCTGTGTACCCACAGATCCCAACCCACAAGAAGTAATGCTG---GAAAATGTGACAGAAAATTTTAACATGTGGAAAAATAACATGGTAGAACAGATGCATGAAGATATAATCAGTTTATGGGATCAAAGCTTAAAGCCATGTGTAAAATTAACCCCACTCTGTGTTACTTTAAATTGCAGTGATACCTGGAGAAATGATACTAATGCCACTGCC---------------------------------------------------------------------------------------AGTAGTTGGGAAAAGGGAGAGAAAGGAGAGATAAAAAACTGCTCTTTCAATGTCACC---GCAGGCATAAGAGAT------AAGGTGCAGAAAGAATATGCACTTTTTTATAAACTTGATGTAATACCAATAGATAATAATAATGGTAGTGAA------------------AATAGTAGC------------------TATGGTAGCTATAGATTGATAAATTGTAATACCTCAGTCATTACACAGGCTTGTCCAAAGGTATCCTTTGAGCCAATTCCCATACACTATTGTGCCCCGGCTGGTTTTGCGATTCTAAAGTGT---AATAATAAGAAGTTCAATGGAACAGGACCATGTACAAATGTTAGCACAGTACAATGTACACATGGAATTAGACCAGTAGTGTCAACTCAACTGCTGTTAAATGGCAGTCTAGCAGAAGAA---GAGGTAGTAATTAGATCTGAAAATTTCACGAACAATGCTAAAATTATAATAGTACATCTGAATAAATCTGTAGTAATTAATTGTACAAGACCCAACAACAATACAAGAAGAAGTATACATATAGGA------------CCAGGGAAA---GTATTTTAT---ACAGGGGAAATAATAGGAGATATAAGACAAGCACATTGTAACCTT------AGTAGAGCGGCATGGAATAACACTTTAGAACAGATAGTTATAAAATTAAGA---GAACAATTTGGG------------AATAAAACA---ATAGTCTTTAAGCAA---------TCCTCAGGAGGGGACCCAGAAATTGTAATGCACACTTTTAATTGTGGAGGGGAATTTTTCTACTGTAATGCAACACCACTGTTTAATAGTAGCTGGTATGTT---------AATGGTACTAAC------------------------------ACTACAGGGTCAGATGGAGAA---------------------------------------------AATATCACACTCCTATGCAGAATAAAACAAATTGTAAACATGTGGCAGGAAGTAGGCAAAGCAATGTATGCCCCTCCCATTAGAGGACAAATTACATGCTCATCAAAGATCACAGGGTTGCTATTAACAAGAGATGGTGGTAACATG------------AACGGGACC---------------------AACGACACC---GAGACCTTCAGACCTGGAGGAGGAGATATGAGGGACAATTGGAGA---AGTGAATTATATAAATATAAAGTAGTAAAAATTGAACCA---TTAGGAGTAGCACCC---ACCAAGGCAAAGAGAAGAGTGGTGCAGAGAGAA---AAAAGAGCAGTG---GGAACAATAGGA---GCTATG---TTCCTT---GGA---------TTCTTGGGA---GCAGCAGGAAGCACTATGGGCGCAGCGTCACTG---ACGCTGACGGTACAGGCCAGACTATTATTGTCTGGTATAGTGCAACAGCAGAACAATCTGCTGAGGGCTATTGAGGCGCAACAACATCTGTTGCAACTCACGGTCTGGGGCATTAAACAGCTCCAGGCAAGA---GTCCTGGCTGTGGAAAGATATCTAAGGGATCAACAGCTCCTAGGGATTTGGGGTTGCTCTGGAAAACTCATCTGCACCACTACTGTGCCTTGGAACAGTAGTTGGAGT---------------------------AATAAATCTCTGGATGAAATTTGGAAT---AATATGACCTGGATGCAGTGGGATAGAGAAATTGAC------AATTACACAAACTTAATATACAGCTTACTTGAAGAATCGCAGAACCAGCAAGAGAAGAATGAAAAAGATTTATTAGAACTGGATAAGTGGGCAAGTTTGTGGAATTGGTTTGATATAACAAGCTGGCTGTGGTATATAAAAATATTCATAATGATAGTAGGTGGCTTGATAGGTTTAAGAATAATTTTTGCTGTACTTTCTATAGTGAATAGGGTTAGGCAGGGATACTCACCATTATCATTTCAGACCCTC---CTCCCAGCTCCGAGGGGA------CCCGACAGGCCCGAAGGAACCGAAGAAGAAGGTGGAGAGAGAGACAGAGGCAGATCAGTTCGATTAGTGCATGGATTCTCAGCACTCATCTGGGACGACCTGCGGAGCCTGTGCCTTTTCAGCTACCACCGCTTGAGAGACTTACTCTTGATTGCAGCGAGGATTGTAGAGATTCTGGGACGCAGG---------------GGGTGGGAAGCCCTCAAGTATTGGTGG---AATCTCCTGCAGTATTGG---------------------------------------------------AGTCAGGAACTAAAGAATAGTGCTATTAGCTTGCTTAATGCCACAGCTATCGCAGTAGCTGAGGGAACAGATAGGATTATAGGAGTAGTACAAAGA------------------ATTTGTAGAGCTATTATCCACATACCTACAAGAATAAGACAGGGCTTAGAAAGGGCTTTGCTATAA

2.SC11.Trinidad.EU576796 ATGACAGTGATGGAGATCAGGAGGAATTAC------TTG---------TGGAGATGGGGC------------------------ATCATGCTCCTTGGAATGTTGATGATC------------TATAGTGCTGCAGAACAA---GAACAAGGGTGGGTCACAGTCTATTATGGGGTACCTGTGTGGAAAGAAGCAACCACCACTCTATTCTGTGCATCAGATGCCAAAGCATATGACACAGAGGTACATAAT---GTCTGGGCCACACATGCCTGTGTACCCACAGATCCCAACCCACAAGAAGTAATGCTG---GAAAATGTGACAGAAAATTTTAACATGTGGAAAAATAACATGGTAGAACAGATGCATGAAGATATAATCAGTTTATGGGATCAAAGCTTAAAGCCATGTGTAAAATTAACCCCACTCTGTGTTACTTTAAATTGCAGTGATACCTGGAGAAATGATACTAATGCCACTGCC---------------------------------------------------------------------------------------AGTAGTTGGGAAAAGGGAGAGAAAGGAGAGATAAAAAACTGCTCTTTCAATGTCACC---GCAGGCATAAGAGAT------AAGGTGCAGAAAGAATATGCACTTTTTTATAAACTTGATGTAATACCAATAGATAATAATAATGGTAGTGAA------------------AATAGTAGC------------------TATGGTAGCTATAGATTGATAAATTGTAATACCTCAGTCATTACACAGGCTTGTCCAAAGGTATCCTTTGAGCCAATTCCCATACACTATTGTGCCCCGGCTGGTTTTGCGATTCTAAAGTGT---AATAATAAGAAGTTCAATGGAACAGGACCATGTACAAATGTTAGCACAGTACAATGTACACATGGAATTAGACCAGTAGTGTCAACTCAACTGCTGTTAAATGGCAGTCTAGCAGAAGAA---GAGGTAGTAATTAGATCTGAAAATTTCACGAACAATGCTAAAATTATAATAGTACATCTGAATAAATCTGTAGTAATTAATTGTACAAGACCCAACAACAATACAAGAAGAAGTATACATATAGGA------------CCAGGGAAA---GTATTTTAT---ACAGGGGAAATAATAGGAGATATAAGACAAGCACATTGTAACCTT------AGTAGAGCGGCATGGAATAACACTTTAGAACAGATAGTTATAAAATTAAGA---GAACAATTTGGG------------AATAAAACA---ATAGTCTTTAAGCAA---------TCCTCAGGAGGGGACCCAGAAATTGTAATGCACACTTTTAATTGTGGAGGGGAATTTTTCTACTGTAATGCAACACCACTGTTTAATAGTAGCTGGTATGTT---------AATGGTACTAAC------------------------------ACTACAGGGTCAGATGGAGAA---------------------------------------------AATATCACACTCCTATGCAGAATAAAACAAATTGTAAACATGTGGCAGGAAGTAGGCAAAGCAATGTATGCCCCTCCCATTAGAGGACAAATTACATGCTCATCAAAGATCACAGGGTTGCTATTAACAAGAGATGGTGGTAACATG------------AACGGGACC---------------------AACGACACC---GAGACCTTCAGACCTGGAGGAGGAGATATGAGGGACAATTGGAGA---AGTGAATTATATAAATATAAAGTAGTAAAAATTGAACCA---TTAGGAGTAGCACCC---ACCAAGGCAAAGAGAAGAGTGGTGCAGAGAGAA---AAAAGAGCAGTG---GGAACAATAGGA---GCTATG---TTCCTT---GGA---------TTCTTGGGA---GCAGCAGGAAGCACTATGGGCGCAGCGTCACTG---ACGCTGACGGTACAGGCCAGACTATTATTGTCTGGTATAGTGCAACAGCAGAACAATCTGCTGAGGGCTATTGAGGCGCAACAACATCTGTTGCAACTCACGGTCTGGGGCATTAAACAGCTCCAGGCAAGA---GTCCTGGCTGTGGAAAGATATCTAAGGGATCAACAGCTCCTAGGGATTTGGGGTTGCTCTGGAAAACTCATCTGCACCACTACTGTGCCTTGGAACAGTAGTTGGAGT---------------------------AATAAATCTCTGGATGAAATTTGGAAT---AATATGACCTGGATGCAGTGGGATAGAGAAATTGAC------AATTACACAAACTTAATATACAGCTTACTTGAAGAATCGCAGAACCAGCAAGAGAAGAATGAAAAAGATTTATTAGAACTGGATAAGTGGGCAAGTTTGTGGAATTGGTTTGATATAACAAGCTGGCTGTGGTATATAAAAATATTCATAATGATAGTAGGTGGCTTGATAGGTTTAAGAATAATTTTTGCTGTACTTTCTATAGTGAATAGGGTTAGGCAGGGATACTCACCATTATCATTTCAGACCCTC---CTCCCAGCTCCGAGGGGA------CCCGACAGGCCCGAAGGAACCGAAGAAGAAGGTGGAGAGAGAGACAGAGGCAGATCAGTTCGATTAGTGCATGGATTCTCAGCACTCATCTGGGACGACCTGCGGAGCCTGTGCCTTTTCAGCTACCACCGCTTGAGAGACTTACTCTTGATTGCAGCGAGGATTGTAGAGATTCTGGGACGCAGG---------------GGGTGGGAAGCCCTCAAGTATTGGTGG---AATCTCCTGCAGTATTGG---------------------------------------------------AGTCAGGAACTAAAGAATAGTGCTATTAGCTTGCTTAATGCCACAGCTATCGCAGTAGCTGAGGGAACAGATAGGATTATAGGAGTAGTACAAAGA------------------ATTTGTAGAGCTATTATCCACATACCTACAAGAATAAGACAGGGCTTAGAAAGGGCTTTGCTATAA

2.SC11.Trinidad.EU576807 ATGACAGTGATGGAGATCAGGAGGAATTAC------TTG---------TGGAGATGGGGC------------------------ATCATGCTCCTTGGAATGTTGATGATC------------TATAGTGCTGCAGAACAA---GAACAAGGGTGGGTCACAGTCTATTATGGGGTACCTGTGTGGAAAGAAGCAACCACCACTCTATTCTGTGCATCAGATGCCAAAGCATATGACACAGAGGTACATAAT---GTCTGGGCCACACATGCCTGTGTACCCACAGATCCCAACCCACAAGAAGTAATGCTG---GAAAATGTGACAGAAAATTTTAACATGTGGAAAAATAACATGGTAGAACAGATGCATGAAGATATAATCAGTTTATGGGATCAAAGCTTAAAGCCATGTGTAAAATTAACCCCACTCTGTGTTACTTTAAATTGCAGTGATACCTGGAGAAATGATACTAATGCCACTGCC---------------------------------------------------------------------------------------AGTAGTTGGGAAAAGGGAGAGAAAGGAGAGATAAAAAACTGCTCTTTCAATGTCACC---GCAGGCATAAGAGAT------AAGGTGCAGAAAGAATATGCACTTTTTTATAAACTTGATGTAATACCAATAGATAATAATAATGGTAGTGAA------------------AATAGTAGC------------------TATGGTAGCTATAGATTGATAAATTGTAATACCTCAGTCATTACACAGGCTTGTCCAAAGGTATCCTTTGAGCCAATTCCCATACACTATTGTGCCCCGGCTGGTTTTGCGATTCTAAAGTGT---AATAATAAGAAGTTCAATGGAACAGGACCATGTACAAATGTTAGCACAGTACAATGTACACATGGAATTAGACCAGTAGTGTCAACTCAACTGCTGTTAAATGGCAGTCTAGCAGAAGAA---GAGGTAGTAATTAGATCTGAAAATTTCACGAACAATGCTAAAATTATAATAGTACATCTGAATAAATCTGTAGTAATTAATTGTACAAGACCCAACAACAATACAAGAAGAAGTATACATATAGGA------------CCAGGGAAA---GTATTTTAT---ACAGGGGAAATAATAGGAGATATAAGACAAGCACATTGTAACCTT------AGTAGAGCGGCATGGAATAACACTTTAGAACAGATAGTTATAAAATTAAGA---GAACAATTTGGG------------AATAAAACA---ATAGTCTTTAAGCAA---------TCCTCAGGAGGGGACCCAGAAATTGTAATGCACACTTTTAATTGTGGAGGGGAATTTTTCTACTGTAATGCAACACCACTGTTTAATAGTAGCTGGTATGTT---------AATGGTACTAAC------------------------------ACTACAGGGTCAGATGGAGAA---------------------------------------------AATATCACACTCCTATGCAGAATAAAACAAATTGTAAACATGTGGCAGGAAGTAGGCAAAGCAATGTATGCCCCTCCCATTAGAGGACAAATTACATGCTCATCAAAGATCACAGGGTTGCTATTAACAAGAGATGGTGGTAACATG------------AACGGGACC---------------------AACGACACC---GAGACCTTCAGACCTGGAGGAGGAGATATGAGGGACAATTGGAGA---AGTGAATTATATAAATATAAAGTAGTAAAAATTGAACCA---TTAGGAGTAGCACCC---ACCAAGGCAAAGAGAAGAGTGGTGCAGAGAGA----AAAAGAGCAGTG---GGAACAATAGGA---GCTATG---TTCCTT---GGA---------TTCTTGGGA---GCAGCAGGAAGCACTATGGGCGCAGCGTCACTG---ACGCTGACGGTACAGGCCAGACTATTATTGTCTGGTATAGTGCAACAGCAGAACAATCTGCTGAGGGCTATTGAGGCGCAACAACATCTGTTGCAACTCACGGTCTGGGGCATTAAACAGCTCCAGGCAAGA---GTCCTGGCTGTGGAAAGATATCTAAGGGATCAACAGCTCCTAGGGATTTGGGGTTGCTCTGGAAAACTCATCTGCACCACTACTGTGCCTTGGAACAGTAGTTGGAGT---------------------------AATAAATCTCTGGATGAAATTTGGAAT---AATATGACCTGGATGCAGTGGGATAGAGAAATTGAC------AATTACACAAACTTAATATACAGCTTACTTGAAGAATCGCAGAACCAGCAAGAGAAGAATGAAAAAGATTTATTAGAACTGGATAAGTGGGCAAGTTTGTGGAATTGGTTTGATATAACAAGCTGGCTGTGGTATATAAAAATATTCATAATGATAGTAGGTGGCTTGATAGGTTTAAGAATAATTTTTGCTGTACTTTCTATAGTGAATAGGGTTAGGCAGGGATACTCACCATTATCATTTCAGACCCTC---CTCCCAGCTCCGAGGGGA------CCCGACAGGCCCGAAGGAACCGAAGAAGAAGGTGGAGAGAGAGACAGAGGCAGATCAGTTCGATTAGTGCATGGATTCTCAGCACTCATCTGGGACGACCTGCGGAGCCTGTGCCTTTTCAGCTACCACCGCTTGAGAGACTTACTCTTGATTGCAGCGAGGATTGTAGAGATTCTGGGACGCAGG---------------GGGTGGGAAGCCCTCAAGTATTGGTGG---AATCTCCTGCAGTATTGG---------------------------------------------------AGTCAGGAACTAAAGAATAGTGCTATTAGCTTGCTTAATGCCACAGCTATCGCAGTAGCTGAGGGAACAGATAGGATTATAGGAGTAGTACAAAGA------------------ATTTGTAGAGCTATTATCCACATACCTACAAGAATAAGACAGGGCTTAGAAAGGGCTTTGCTATAA

2.SC11.Trinidad.EU576805 ATGACAGTGATGGAGATCAGGAGGAATTAC------TTG---------TGGAGATGGGGC------------------------ATCATGCTCCTTGGAATGTTGATGATC------------TATAGTGCTGCAGAACAA---GAACAAGGGTGGGTCACAGTCTATTATGGGGTACCTGTGTGGAAAGAAGCAACCACCACTCTATTCTGTGCATCAGATGCCAAAGCATATGACACAGAGGTACATAAT---GTCTGGGCCACACATGCCTGTGTACCCACAGATCCCAACCCACAAGAAGTAATGCTG---GAAAATGTGACAGAAAATTTTAACATGTGGAAAAATAACATGGTAGAACAGATGCATGAAGATATAATCAGTTTATGGGATCAAAGCTTAAAGCCATGTGTAAAATTAACCCCACTCTGTGTTACTTTAAATTGCAGTGATACCTGGAGAAATGATACTAATGCCACTGCC---------------------------------------------------------------------------------------AGTAGTTGGGAAAAGGGAGAGAAAGGAGAGATAAAAAACTGCTCTTTCAATGTCACC---GCAGGCATAAGAGAT------AAGGTGCAGAAAGAATATGCACTTTTTTATAAACTTGATGTAATACCAATAGATAATAATAATGGTAGTGAA------------------AATAGTAGC------------------TATGGTAGCTATAGATTGATAAATTGTAATACCTCAGTCATTACACAGGCTTGTCCAAAGGTATCCTTTGAGCCAATTCCCATACACTATTGTGCCCCGGCTGGTTTTGCGATTCTAAAGTGT---AATAATAAGAAGTTCAATGGAACAGGACCATGTACAAATGTTAGCACAGTACAATGTACACATGGAATTAGACCAGTAGTGTCAACTCAACTGCTGTTAAATGGCAGTCTAGCAGAAGAA---GAGGTAGTAATTAGATCTGAAAATTTCACGAACAATGCTAAAATTATAATAGTACATCTGAATAAATCTGTAGTAATTAATTGTACAAGACCCAACAACAATACAAGAAGAAGTATACATATAGGA------------CCAGGGAAA---GTATTTTAT---ACAGGGGAAATAATAGGAGATATAAGACAAGCACATTGTAACCTT------AGTAGAGCGGCATGGAATAACACTTTAGAACAGATAGTTATAAAATTAAGA---GAACAATTTGGG------------AATAAAACA---ATAGTCTTTAAGCAA---------TCCTCAGGAGGGGACCCAGAAATTGTAATGCACACTTTTAATTGTGGAGGGGAATTTTTCTACTGTAATGCAACACCACTGTTTAATAGTAGCTGGTATGTT---------AATGGTACTAAC------------------------------ACTACAGGGTCAGATGGAGAA---------------------------------------------AATATCACACTCCTATGCAGAATAAAACAAATTGTAAACATGTGGCAGGAAGTAGGCAAAGCAATGTATGCCCCTCCCATTAGAGGACAAATTACATGCTCATCAAAGATCACAGGGTTGCTATTAACAAGAGATGGTGGTAACATG------------AACGGGACC---------------------AACGACACC---GAGACCTTCAGACCTGGAGGAGGAGATATGAGGGACAATTGGAGA---AGTGAATTATATAAATATAAAGTAGTAAAAATTGAACCA---TTAGGAGTAGCACCC---ACCAAGGCAAAGAGAAGAGTGGTGCAGAGAGAA---AAAAGAGCAGTG---GGAACAATAGGA---GCTATG---TTCCTT---GGA---------TTCTTGGGA---GCAGCAGGAAGCACTATGGGCGCAGCGTCACTG---ACGCTGACGGTACAGGCCAGACTATTATTGTCTGGTATAGTGCAACAGCAGAACAATCTGCTGAGGGCTATTGAGGCGCAACAACATCTGTTGCAACTCACGGTCTGGGGCATTAAACAGCTCCAGGCAAGA---GTCCTGGCTGTGGAAAGATATCTAAGGGATCAACAGCTCCTAGGGATTTGGGGTTGCTCTGGAAAACTCATCTGCACCACTACTGTGCCTTGGAACAGTAGTTGGAGT---------------------------AATAAATCTCTGGATGAAATTTGGAAT---AATATGACCTGGATGCAGTGGGATAGAGAAATTGAC------AATTACACAAACTTAATATACAGCTTACTTGAAGAATCGCAGAACCAGCAAGAGAAGAATGAAAAAGATTTATTAGAACTGGATAAGTGGGCAAGTTTGTGGAATTGGTTTGATATAACAAGCTGGCTGTGGTATATAAAAATATTCATAATGATAGTAGGTGGCTTGATAGGTTTAAGAATAATTTTTGCTGTACTTTCTATAGTGAATAGGGTTAGGCAGGGATACTCACCATTATCATTTCAGACCCTC---CTCCCAGCTCCGAGGGGA------CCCGACAGGCCCGAAGGAACCGAAGAAGAAGGTGGAGAGAGAGACAGAGGCAGATCAGTTCGATTAGTGCATGGATTCTCAGCACTCATCTGGGACGACCTGCGGAGCCTGTGCCTTTTCAGCTACCACCGCTTGAGAGACTTACTCTTGATTGCAGCGAGGATTGTAGAGATTCTGGGACGCAGG---------------GGGTGGGAAGCCCTCAAGTATTGGTGG---AATCTCCTGCAGTATTGG---------------------------------------------------AGTCAGGAACTAAAGAATAGTGCTATTAGCTTGCTTAATGCCACAGCTATCGCAGTAGCTGAGGGAACAGATAGGATTATAGGAGTAGTACAAAGA------------------ATTTGTAGAGCTATTATCCACATACCTACAAGAATAAGACAGGGCTTAGAAAGGGCTTTGCTATAA

2.SC11.Trinidad.EU576797 ATGACAGTGATGGAGATCAGGAGGAATTAC------TTG---------TGGAGATGGGGC------------------------ATCATGCTCCTTGGAATGTTGATGATC------------TATAGTGCTGCAGAACAA---GAACAAGGGTGGGTCACAGTCTATTATGGGGTACCTGTGTGGAAAGAAGCAACCACCACTCTATTCTGTGCATCAGATGCCAAAGCATATGACACAGAGGTACATAAT---GTCTGGGCCACACATGCCTGTGTACCCACAGATCCCAACCCACAAGAAGTAATGCTG---GAAAATGTGACAGAAAATTTTAACATGTGGAAAAATAACATGGTAGAACAGATGCATGAAGATATAATCAGTTTATGGGATCAAAGCTTAAAGCCATGTGTAAAATTAACCCCACTCTGTGTTACTTTAAATTGCAGTGATACCTGGAGAAATGATACTAATGCCACTGCC---------------------------------------------------------------------------------------AGTAGTTGGGAAAAGGGAGAGAAAGGAGAGATAAAAAACTGCTCTTTCAATGTCACC---GCAGGCATAAGAGAT------AAGGTGCAGAAAGAATATGCACTTTTTTATAAACTTGATGTAATACCAATAGATAATAATAATGGTAGTGAA------------------AATAGTAGC------------------TATGGTAGCTATAGATTGATAAATTGTAATACCTCAGTCATTACACAGGCTTGTCCAAAGGTATCCTTTGAGCCAATTCCCATACACTATTGTGCCCCGGCTGGTTTTGCGATTCTAAAGTGT---AATAATAAGAAGTTCAATGGAACAGGACCATGTACAAATGTTAGCACAGTACAATGTACACATGGAATTAGACCAGTAGTGTCAACTCAACTGCTGTTAAATGGCAGTCTAGCAGAAGAA---GAGGTAGTAATTAGATCTGAAAATTTCACGAACAATGCTAAAATTATAATAGTACATCTGAATAAATCTGTAGTAATTAATTGTACAAGACCCAACAACAATACAAGAAGAAGTATACATATAGGA------------CCAGGGAAA---GTATTTTAT---ACAGGGGAAATAATAGGAGATATAAGACAAGCACATTGTAACCTT------AGTAGAGCGGCATGGAATAACACTTTAGAACAGATAGTTATAAAATTAAGA---GAACAATTTGGG------------AATAAAACA---ATAGTCTTTAAGCAA---------TCCTCAGGAGGGGACCCAGAAATTGTAATGCACACTTTTAATTGTGGAGGGGAATTTTTCTACTGTAATGCAACACCACTGTTTAATAGTAGCTGGTATGTT---------AATGGTACTAAC------------------------------ACTACAGGGTCAGATGGAGAA---------------------------------------------AATATCACACTCCTATGCAGAATAAAACAAATTGTAAACATGTGGCAGGAAGTAGGCAAAGCAATGTATGCCCCTCCCATTAGAGGACAAATTACATGCTCATCAAAGATCACAGGGTTGCTATTAACAAGAGATGGTGGTAACATG------------AACGGGACC---------------------AACGACACC---GAGACCTTCAGACCTGGAGGAGGAGATATGAGGGACAATTGGAGA---AGTGAATTATATAAATATAAAGTAGTAAAAATTGAACCA---TTAGGAGTAGCACCC---ACCAAGGCAAAGAGAAGAGTGGTGCAGAGAGAA---AAAAGAGCAGTG---GGAACAATAGGA---GCTATG---TTCCTT---GGA---------TTCTTGGGA---GCAGCAGGAAGCACTATGGGCGCAGCGTCACTG---ACGCTGACGGTACAGGCCAGACTATTATTGTCTGGTATAGTGCAACAGCAGAACAATCTGCTGAGGGCTATTGAGGCGCAACAACATCTGTTGCAACTCACGGTCTGGGGCATTAAACAGCTCCAGGCAAGA---GTCCTGGCTGTGGAAAGATATCTAAGGGATCAACAGCTCCTAGGGATTTGGGGTTGCTCTGGAAAACTCATCTGCACCACTACTGTGCCTTGGAACAGTAGTTGGAGT---------------------------AATAAATCTCTGGATGAAATTTGGAAT---AATATGACCTGGATGCAGTGGGATAGAGAAATTGAC------AATTACACAAACTTAATATACAGCTTACTTGAAGAATCGCAGAACCAGCAAGAGAAGAATGAAAAAGATTTATTAGAACTGGATAAGTGGGCAAGTTTGTGGAATTGGTTTGATATAACAAGCTGGCTGTGGTATATAAAAATATTCATAATGATAGTAGGTGGCTTGATAGGTTTAAGAATAATTTTTGCTGTACTTTCTATAGTGAATAGGGTTAGGCAGGGATACTCACCATTATCATTTCAGACCCTC---CTCCCAGCTCCGAGGGGA------CCCGACAGGCCCGAAGGAACCGAAGAAGAAGGTGGAGAGAGAGACAGAGGCAGATCAGTTCGATTAGTGCATGGATTCTCAGCACTCATCTGGGACGACCTGCGGAGCCTGTGCCTTTTCAGCTACCACCGCTTGAGAGACTTACTCTTGATTGCAGCGAGGATTGTAGAGATTCTGGGACGCAGG---------------GGGTGGGAAGCCCTCAAGTATTGGTGG---AATCTCCTGCAGTATTGG---------------------------------------------------AGTCAGGAACTAAAGAATAGTGCTATTAGCTTGCTTAATGCCACAGCTATCGCAGTAGCTGAGGGAACAGATAGGATTATAGGAGTAGTACAAAGA------------------ATTTGTAGAGCTATTATCCACATACCTACAAGAATAAGACAGGGCTTAGAAAGGGCTTTGCTATAA

2.SC11.Trinidad.EU576802 ATGACAGTGATGGAGATCAGGAGGAATTAC------TTG---------TGGAGATGGGGC------------------------ATCATGCTCCTTGGAATGTTGATGATC------------TATAGTGCTGCAGAACAA---GAACAAGGGTGGGTCACAGTCTATTATGGGGTACCTGTGTGGAAAGAAGCAACCACCACTCTATTCTGTGCATCAGATGCCAAAGCATATGACACAGAGGTACATAAT---GTCTGGGCCACACATGCCTGTGTACCCACAGATCCCAACCCACAAGAAGTAATGCTG---GAAAATGTGACAGAAAATTTTAACATGTGGAAAAATAACATGGTAGAACAGATGCATGAAGATATAATCAGTTTATGGGATCAAAGCTTAAAGCCATGTGTAAAATTAACCCCACTCTGTGTTACTTTAAATTGCAGTGATACCTGGAGAAATGATACTAATGCCACTGCC---------------------------------------------------------------------------------------AGTAGTTGGGAAAAGGGAGAGAAAGGAGAGATAAAAAACTGCTCTTTCAATGTCACC---GCAGGCATAAGAGAT------AAGGTGCAGAAAGAATATGCACTTTTTTATAAACTTGATGTAATACCAATAGATAATAATAATGGTAGTGAA------------------AATAGTAGC------------------TATGGTAGCTATAGATTGATAAATTGTAATACCTCAGTCATTACACAGGCTTGTCCAAAGGTATCCTTTGAGCCAATTCCCATACACTATTGTGCCCCGGCTGGTTTTGCGATTCTAAAGTGT---AATAATAAGAAGTTCAATGGAACAGGACCATGTACAAATGTTAGCACAGTACAATGTACACATGGAATTAGACCAGTAGTGTCAACTCAACTGCTGTTAAATGGCAGTCTAGCAGAAGAA---GAGGTAGTAATTAGATCTGAAAATTTCACGAACAATGCTAAAATTATAATAGTACATCTGAATAAATCTGTAGTAATTAATTGTACAAGACCCAACAACAATACAAGAAGAAGTATACATATAGGA------------CCAGGGAAA---GTATTTTAT---ACAGGGGAAATAATAGGAGATATAAGACAAGCACATTGTAACCTT------AGTAGAGCGGCATGGAATAACACTTTAGAACAGATAGTTATAAAATTAAGA---GAACAATTTGGG------------AATAAAACA---ATAGTCTTTAAGCAA---------TCCTCAGGAGGGGACCCAGAAATTGTAATGCACACTTTTAATTGTGGAGGGGAATTTTTCTACTGTAATGCAACACCACTGTTTAATAGTAGCTGGTATGTT---------AATGGTACTAAC------------------------------ACTACAGGGTCAGATGGAGAA---------------------------------------------AATATCACACTCCTATGCAGAATAAAACAAATTGTAAACATGTGGCAGGAAGTAGGCAAAGCAATGTATGCCCCTCCCATTAGAGGACAAATTACATGCTCATCAAAGATCACAGGGTTGCTATTAACAAGAGATGGTGGTAACATG------------AACGGGACC---------------------AACGACACC---GAGACCTTCAGACCTGGAGGAGGAGATATGAGGGACAATTGGAGA---AGTGAATTATATAAATATAAAGTAGTAAAAATTGAACCA---TTAGGAGTGGCACCC---ACCAAGGCAAAGAGAAGAGTGGTGCAGAGAGAA---AAAAGAGCAGTG---GGAACAATAGGA---GCTATG---TTCCTT---GGA---------TTCTTGGGA---GCAGCAGGAAGCACTATGGGCGCAGCGTCACTG---ACGCTGACGGTACAGGCCAGACTATTATTGTCTGGTATAGTGCAACAGCAGAACAATCTGCTGAGGGCTATTGAGGCGCAACAACATCTGTTGCAACTCACGGTCTGGGGCATTAAACAGCTCCAGGCAAGA---GTCCTGGCTGTGGAAAGATATCTAAGGGATCAACAGCTCCTAGGGATTTGGGGTTGCTCTGGAAAACTCATCTGCACCACTACTGTGCCTTGGAACAGTAGTTGGAGT---------------------------AATAAATCTCTGGATGAAATTTGGAAT---AATATGACCTGGATGCAGTGGGATAGAGAAATTGAC------AATTACACAAACTTAATATACAGCTTACTTGAAGAATCGCAGAACCAGCAAGAGAAGAATGAAAAAGATTTATTAGAACTGGATAAGTGGGCAAGTTTGTGGAATTGGTTTGATATAACAAGCTGGCTGTGGTATATAAAAATATTCATAATGATAGTAGGTGGCTTGATAGGTTTAAGAATAATTTTTGCTGTACTTTCTATAGTGAATAGGGTTAGGCAGGGATACTCACCATTATCATTTCAGACCCTC---CTCCCAGCTCCGAGGGGA------CCCGACAGGCCCGAAGGAACCGAAGAAGAAGGTGGAGAGAGAGACAGAGGCAGATCAGTTCGATTAGTGCATGGATTCTCAGCACTCATCTGGGACGACCTGCGGAGCCTGTGCCTTTTCAGCTACCACCGCTTGAGAGACTTACTCTTGATTGCAGCGAGGATTGTAGAGATTCTGGGACGCAGG---------------GGGTGGGAAGCCCTCAAGTATTGGTGG---AATCTCCTGCAGTATTGG---------------------------------------------------AGTCAGGAACTAAAGAATAGTGCTATTAGCTTGCTTAATGCCACAGCTATCGCAGTAGCTGAGGGAACAGATAGGATTATAGGAGTAGTACAAAGA------------------ATTTGTAGAGCTATTATCCACATACCTACAAGAATAAGACAGGGCTTAGAAAGGGCTTTGCTATAA

2.SC11.Trinidad.EU576790 ATGACAGTGATGGAGATCAGGAGGAATTAC------TTG---------TGGAGATGGGGC------------------------ATCATGCTCCTTGGAATGTTGATGATC------------TATAGTGCTGCAGAACAA---GAACAAGGGTGGGTCACAGTCTATTATGGGGTACCTGTGTGGAAAGAAGCAACCACCACTCTATTCTGTGCATCAGATGCCAAAGCATATGACACAGAGGTACATAAT---GTCTGGGCCACACATGCCTGTGTACCCACAGATCCCAACCCACAAGAAGTAATGCTG---GAAAATGTGACAGAAAATTTTAACATGTGGAAAAATAACATGGTAGAACAGATGCATGAAGATATAATCAGTTTATGGGATCAAAGCTTAAAGCCATGTGTAAAATTAACCCCACTCTGTGTTACTTTAAATTGCAGTGATACCTGGAGAAATGATACTAATGCCACTGCC---------------------------------------------------------------------------------------AGTAGTTGGGAAAAGGGAGAGAAAGGAGAGATAAAAAACTGCTCTTTCAATGTCACC---GCAGGCATAAGAGAT------AAGGTGCAGAAAGAATATGCACTTTTTTATAAACTTGATGTAATACCAATAGATAATAATAATGGTAGTGAA------------------AATAGTAGC------------------TATGGTAGCTATAGATTGATAAATTGTAATACCTCAGTCATTACACAGGCTTGTCCAAAGGTATCCTTTGAGCCAATTCCCATACACTATTGTGCCCCGGCTGGTTTTGCGATTCTAAAGTGT---AATAATAAGAAGTTCAATGGAACAGGACCATGTACAAATGTTAGCACAGTACAATGTACACATGGAATTAGACCAGTAGTGTCAACTCAACTGCTGTTAAATGGCAGTCTAGCAGAAGAA---GAGGTAGTAATTAGATCTGAAAATTTCACGAACAATGCTAAAATTATAATAGTACATCTGAATAAATCTGTAGTAATTAATTGTACAAGACCCAACAACAATACAAGAAGAAGTATACATATAGGA------------CCAGGGAAA---GTATTTTAT---ACAGGGGAAATAATAGGAGATATAAGACAAGCACATTGTAACCTT------AGTAGAGCGGCATGGAATAACACTTTAGAACAGATAGTTATAAAATTAAGA---GAACAATTTGGG------------AATAAAACA---ATAGTCTTTAAGCAA---------TCCTCAGGAGGGGACCCAGAAATTGTAATGCACACTTTTAATTGTGGAGGGGAATTTTTCTACTGTAATGCAACACCACTGTTTAATAGTAGCTGGTATGTT---------AATGGTACTAAC------------------------------ACTACAGGGTCAGATGGAGAA---------------------------------------------AATATCACACTCCTATGCAGAATAAAACAAATTGTAAACATGTGGCAGGAAGTAGGCAAAGCAATGTATGCCCCTCCCATTAGAGGACAAATTACATGCTCATCAAAGATCACAGGGTTGCTATTAACAAGAGATGGTGGTAACATG------------AACGGGACC---------------------AACGACACC---GAGACCTTCAGACCTGGAGGAGGAGATATGAGGGACAATTGGAGA---AGTGAATTATATAAATATAAAGTAGTAAAAATTGAACCA---TTAGGAGTAGCACCC---ACCAAGGCAAAGAGAAGAGTGGTGCAGAGAGAA---AAAAGAGCAGTG---GGAACAATAGGA---GCTATG---TTCCTT---GGA---------TTCTTGGGA---GCAGCAGGAAGCACTATGGGCGCAGCGTCACTG---ACGCTGACGGTACAGGCCAGACTATTATTGTCTGGTATAGTGCAACAGCAGAACAATCTGCTGAGGGCTATTGAGGCGCAACAACATCTGTTGCAACTCACGGTCTGGGGCATTAAACAGCTCCAGGCAAGA---GTCCTGGCTGTGGAAAGATATCTAAGGGATCAACAGCTCCTAGGGATTTGGGGTTGCTCTGGAAAACTCATCTGCACCACTACTGTGCCTTGGAACAGTAGTTGGAGT---------------------------AATAAATCTCTGGATGAAATTTGGAAT---AATATGACCTGGATGCAGTGGGATAGAGAAATTGAC------AATTACACAAACTTAATATACAGCTTACTTGAAGAATCGCAGAACCAGCAAGAGAAGAATGAAAAAGATTTATTAGAACTGGATAAGTGGGCAAGTTTGTGGAATTGGTTTGATATAACAAGCTGGCTGTGGTATATAAAAATATTCATAATGATAGTAGGTGGCTTGATAGGTTTAAGAATAATTTTTGCTGTACTTTCTATAGTGAATAGGGTTAGGCAGGGATACTCACCATTATCATTTCAGACCCTC---CTCCCAGCTCCGAGGGGA------CCCGACAGGCCCGAAGGAACCGAAGAAGAAGGTGGAGAGAGAGACAGAGGCAGATCAGTTCGATTAGTGCATGGATTCTCAGCACTCATCTGGGACGACCTGCGGAGCCTGTGCCTTTTCAGCTACCACCGCTTGAGAGACTTACTCTTGATTGCAGCGAGGATTGTAGAGATTCTGGGACGCAGG---------------GGGTGGGAAGCCCTCAAGTATTGGTGG---AATCTCCTGCAGTATTGG---------------------------------------------------AGTCAGGAACTAAAGAATAGTGCTATTAGCTTGCTTAATGCCACAGCTATCGCAGTAGCTGAGGGAACAGATAGGATTATAGGAGTAGTACAAAGA------------------ATTTGTAGAGCTATTATCCACATACCTACAAGAATAAGACAGGGCTTAGAAAGGGCTTTGCTATAA

2.SC11.Trinidad.EU576791 ATGACAGTGATGGAGATCAGGAGGAATTAC------TTG---------TGGAGATGGGGC------------------------ATCATGCTCCTTGGAATGTTGATGATC------------TATAGTGCTGCAGAACAA---GAACAAGGGTGGGTCACAGTCTATTATGGGGTACCTGTGTGGAAAGAAGCAACCACCACTCTATTCTGTGCATCAGATGCCAAAGCATATGACACAGAGGTACATAAT---GTCTGGGCCACACATGCCTGTGTACCCACAGATCCCAACCCACAAGAAGTAATGCTG---GAAAATGTGACAGAAAATTTTAACATGTGGAAAAATAACATGGTAGAACAGATGCATGAAGATATAATCAGTTTATGGGATCAAAGCTTAAAGCCATGTGTAAAATTAACCCCACTCTGTGTTACTTTAAATTGCAGTGATACCTGGAGAAATGATACTAATGCCACTGCC---------------------------------------------------------------------------------------AGTAGTTGGGAAAAGGGAGAGAAAGGAGAGATAAAAAACTGCTCTTTCAATGTCACC---GCAGGCATAAGAGAT------AAGGTGCAGAAAGAATATGCACTTTTTTATAAACTTGATGTAATACCAATAGATAATAATAATGGTAGTGAA------------------AATAGTAGC------------------TATGGTAGCTATAGATTGATAAATTGTAATACCTCAGTCATTACACAGGCTTGTCCAAAGGTATCCTTTGAGCCAATTCCCATACACTATTGTGCCCCGGCTGGTTTTGCGATTCTAAAGTGT---AATAATAAGAAGTTCAATGGAACAGGACCATGTACAAATGTTAGCACAGTACAATGTACACATGGAATTAGACCAGTAGTGTCAACTCAACTGCTGTTAAATGGCAGTCTAGCAGAAGAA---GAGGTAGTAATTAGATCTGAAAATTTCACGAACAATGCTAAAATTATAATAGTACATCTGAATAAATCTGTAGTAATTAATTGTACAAGACCCAACAACAATACAAGAAGAAGTATACATATAGGA------------CCAGGGAAA---GTATTTTAT---ACAGGGGAAATAATAGGAGATATAAGACAAGCACATTGTAACCTT------AGTAGAGCGGCATGGAATAACACTTTAGAACAGATAGTTATAAAATTAAGA---GAACAATTTGGG------------AATAAAACA---ATAGTCTTTAAGCAA---------TCCTCAGGAGGGGACCCAGAAATTGTAATGCACACTTTTAATTGTGGAGGGGAATTTTTCTACTGTAATGCAACACCACTGTTTAATAGTAGCTGGTATGTT---------AATGGTACTAAC------------------------------ACTACAGGGTCAGATGGAGAA---------------------------------------------AATATCACACTCCTATGCAGAATAAAACAAATTGTAAACATGTGGCAGGAAGTAGGCAAAGCAATGTATGCCCCTCCCATTAGAGGACAAATTACATGCTCATCAAAGATCACAGGGTTGCTATTAACAAGAGATGGTGGTAACATG------------AACGGGACC---------------------AACGACACC---GAGACCTTCAGACCTGGAGGAGGAGATATGAGGGACAATTGGAGA---AGTGAATTATATAAATATAAAGTAGTAAAAATTGAACCA---TTAGGAGTAGCACCC---ACCAAGGCAAAGAGAAGAGTGGTGCAGAGAGAA---AAAAGAGCAGTG---GGAACAATAGGA---GCTATG---TTCCTT---GGA---------TTCTTGGGA---GCAGCAGGAAGCACTATGGGCGCAGCGTCACTG---ACGCTGACGGTACAGGCCAGACTATTATTGTCTGGTATAGTGCAACAGCAGAACAATCTGCTGAGGGCTATTGAGGCGCAACAACATCTGTTGCAACTCACGGTCTGGGGCATTAAACAGCTCCAGGCAAGA---GTCCTGGCTGTGGAAAGATATCTAAGGGATCAACAGCTCCTAGGGATTTGGGGTTGCTCTGGAAAACTCATCTGCACCACTACTGTGCCTTGGAACAGTAGTTGGAGT---------------------------AATAAATCTCTGGATGAAATTTGGAAT---AATATGACCTGGATGCAGTGGGATAGAGAAATTGAC------AATTACACAAACTTAATATACAGCTTACTTGAAGAATCGCAGAACCAGCAAGAGAAGAATGAAAAAGATTTATTAGAACTGGATAAGTGGGCAAGTTTGTGGAATTGGTTTGATATAACAAGCTGGCTGTGGTATATAAAAATATTCATAATGATAGTAGGTGGCTTGATAGGTTTAAGAATAATTTTTGCTGTACTTTCTATAGTGAATAGGGTTAGGCAGGGATACTCACCATTATCATTTCAGACCCTC---CTCCCAGCTCCGAGGGGA------CCCGACAGGCCCGAAGGAACCGAAGAAGAAGGTGGAGAGAGAGACAGAGGCAGATCAGTTCGATTAGTGCATGGATTCTCAGCACTCATCTGGGACGACCTGCGGAGCCTGTGCCTTTTCAGCTACCACCGCTTGAGAGACTTACTCTTGATTGCAGCGAGGATTGTAGAGATTCTGGGACGCAGG---------------GGGTGGGAAGCCCTCAAGTATTGGTGG---AATCTCCTGCAGTATTGG---------------------------------------------------AGTCAGGAACTAAAGAATAGTGCTATTAGCTTGCTTAATGCCACAGCTATCGCAGTAGCTGAGGGAACAGATAGGATTATAGGAGTAGTACAAAGA------------------ATTTGTAGAGCTATTATCCACATACCTACAAGAATAAGACAGGGCTTAGAAAGGGCTTTGCTATAA

2.SC11.Trinidad.EU576788 ATGACAGTGATGGAGATCAGGAGGAATTAC------TTG---------TGGAGATGGGGC------------------------ATCATGCTCCTTGGAATGTTGATGATC------------TATAGTGCTGCAGAACAA---GAACAAGGGTGGGTCACAGTCTATTATGGGGTACCTGTGTGGAAAGAAGCAACCACCACTCTATTCTGTGCATCAGATGCCAAAGCATATGACACAGAGGTACATAAT---GTCTGGGCCACACATGCCTGTGTACCCACAGATCCCAACCCACAAGAAGTAATGCTG---GAAAATGTGACAGAAAATTTTAACATGTGGAAAAATAACATGGTAGAACAGATGCATGAAGATATAATCAGTTTATGGGATCAAAGCTTAAAGCCATGTGTAAAATTAACCCCACTCTGTGTTACTTTAAATTGCAGTGATACCTGGAGAAATGATACTAATGCCACTGCC---------------------------------------------------------------------------------------AGTAGTTGGGAAAAGGGAGAGAAAGGAGAGATAAAAAACTGCTCTTTCAATGTCACC---GCAGGCATAAGAGAT------AAGGTGCAGAAAGAATATGCACTTTTTTATAAACTTGATGTAATACCAATAGATAATAATAATGGTAGTGAA------------------AATAGTAGC------------------TATGGTAGCTATAGATTGATAAATTGTAATACCTCAGTCATTACACAGGCTTGTCCAAAGGTATCCTTTGAGCCAATTCCCATACACTATTGTGCCCCGGCTGGTTTTGCGATTCTAAAGTGT---AATAATAAGAAGTTCAATGGAACAGGACCATGTACAAATGTTAGCACAGTACAATGTACACATGGAATTAGACCAGTAGTGTCAACTCAACTGCTGTTAAATGGCAGTCTAGCAGAAGAA---GAGGTAGTAATTAGATCTGAAAATTTCACGAACAATGCTAAAATTATAATAGTACATCTGAATAAATCTGTAGTAATTAATTGTACAAGACCCAACAACAATACAAGAAGAAGTATACATATAGGA------------CCAGGGAAA---GTATTTTAT---ACAGGGGAAATAATAGGAGATATAAGACAAGCACATTGTAACCTT------AGTAGAGCGGCATGGAATAACACTTTAGAACAGATAGTTATAAAATTAAGA---GAACAATTTGGG------------AATAAAACA---ATAGTCTTTAAGCAA---------TCCTCAGGAGGGGACCCAGAAATTGTAATGCACACTTTTAATTGTGGAGGGGAATTTTTCTACTGTAATGCAACACCACTGTTTAATAGTAGCTGGTATGTT---------AATGGTACTAAC------------------------------ACTACAGGGTCAGATGGAGAA---------------------------------------------AATATCACACTCCTATGCAGAATAAAACAAATTGTAAACATGTGGCAGGAAGTAGGCAAAGCAATGTATGCCCCTCCCATTAGAGGACAAATTACATGCTCATCAAAGATCACAGGGTTGCTATTAACAAGAGATGGTGGTAACATG------------AACGGGACC---------------------AACGACACC---GAGACCTTCAGACCTGGAGGAGGAGATATGAGGGACAATTGGAGA---AGTGAATTATATAAATATAAAGTAGTAAAAATTGAACCA---TTAGGAGTAGCACCC---ACCAAGGCAAAGAGAAGAGTGGTGCAGAGAGAA---AAAAGAGCAGTG---GGAACAATAGGA---GCTATG---TTCCTT---GGA---------TTCTTGGGA---GCAGCAGGAAGCACTATGGGCGCAGCGTCACTG---ACGCTGACGGTACAGGCCAGACTATTATTGTCTGGTATAGTGCAACAGCAGAACAATCTGCTGAGGGCTATTGAGGCGCAACAACATCTGTTGCAACTCACGGTCTGGGGCATTAAACAGCTCCAGGCAAGA---GTCCTGGCTGTGGAAAGATATCTAAGGGATCAACAGCTCCTAGGGATTTGGGGTTGCTCTGGAAAACTCATCTGCACCACTACTGTGCCTTGGAACAGTAGTTGGAGT---------------------------AATAAATCTCTGGATGAAATTTGGAAT---AATATGACCTGGATGCAGTGGGATAGAGAAATTGAC------AATTACACAAACTTAATATACAGCTTACTTGAAGAATCGCAGAACCAGCAAGAGAAGAATGAAAAAGATTTATTAGAACTGGATAAGTGGGCAAGTTTGTGGAATTGGTTTGATATAACAAGCTGGCTGTGGTATATAAAAATATTCATAATGATAGTAGGTGGCTTGATAGGTTTAAGAATAATTTTTGCTGTACTTTCTATAGTGAATAGGGTTAGGCAGGGATACTCACCATTATCATTTCAGACCCTC---CTCCCAGCTCCGAGGGGA------CCCGACAGGCCCGAAGGAACCGAAGAAGAAGGTGGAGAGAGAGACAGAGGCAGATCAGTTCGATTAGTGCATGGATTCTCAGCACTCATCTGGGACGACCTGCGGAGCCTGTGCCTTTTCAGCTACCACCGCTTGAGAGACTTACTCTTGATTGCAGCGAGGATTGTAGAGATTCTGGGACGCAGG---------------GGGTGGGAAGCCCTCAAGTATTGGTGG---AATCTCCTGCAGTATTGG---------------------------------------------------AGTCAGGAACTAAAGAATAGTGCTATTAGCTTGCTTAATGCCACAGCTATCGCAGTAGCTGAGGGAACAGATAGGATTATAGGAGTAGTACAAAGA------------------ATTTGTAGAGCTATTATCCACATACCTACAAGAATAAGACAGGGCTTAGAAAGGGCTTTGCTATAA

2.SC22.Trinidad.EU576885 ATGAGAGTGAAGGAGATCAAGAGGAATTGTCAGCGCTTG---------TGGAGATGGGGC------------------------ACCATGCTCCTTGGGATGTTGATGATC------------TGTAGTGCTACA---------CAACAATTGTGGGTTACAGTCTATTATGGGGTACCTGTGTGGAAAGAAGCAACCACCACTCTATTTTGTGCATCAGATGCTAAAGCATATGATACAGAGGCACATAAT---GTCTGGGCCACACATGCCTGTGTACCCACAGACCCCAACCCACAAGAAGTAGTATTG---GGAAATGTGACAGAAAATTTTAACATGTGGAAAAATAACATGGTAGAACAAATGCATGAGGATATAATCAGTTTATGGGATCAAAGCCTAAAGCCATGTGTAAAATTAACCCCACTCTGTGTTACTTTAAACTGCACTGATTATGTGAAAAATGATACTAGTAGCACT---------------------------------------------------------------------------------------------AACAGTAGCAGCTGGGAGAAAGGAGAAATAAAAAACTGCTCTTTCAATATCACC---ACAAGCATAAAAGAT------AAGATGCAGAGGGAATATGCACTTTTTTATAAACTTGATGTAATACCAATAGATAGTGAGAATAATAGTAATATAAATAGTGGGAATAATAGTAATAGTAATTTTAGT------AGCTATAGTAATTTTAGATTGATAAATTGTAATACCTCAGTCATTACACAGGCCTGTCCAAAGGTATCCTTTGAACCAATTCCCATACATTATTGTGCCCCGGCTGGGTTTGCGATTCTAAAGTGT---AATGATAAAAAGTTCAATGGATCAGGACCATGTACAAATGTCAGCACAGTACAATGTACACATGGAATTAGACCAGTAGTGTCAACTCAACTGTTGTTAAATGGCAGTCTAGCAGAAGAG---GAGGTAGTAATTAGATCTGAAAATTTCACGAACAATGTTAAAACCATAATAGTACATCTGAATAAATCTGTAGTAATTAATTGTACAAGACCCAACAACAATACAAGAAGAGGTATACATATGGGA------------CCAGGGAAA---GCATATTTT---ACAGGAGAAATAATAGGAGATATAAGACAAGCACATTGTAACATT------AGTAGAAAAGAATGGAATGACACTTTAAAACAGATAGTTATAAAATTAGGA---GAACAATTTAGA------------AATAAAACA---ATAGCCTTTAATCAA---------TCCTCAGGAGGGGACATTGAAATTGTAACACACAGTTTTAATTGTGGAGGGGAATTTTTCTACTGTAATTCAACACAACTGTTTAATAGTACTTGGAATAAT---------AATGGTACTAGG------------------------------AATGATACTAGGGAGTCA------------AATGACACT---------------------------ATCATCACACTACCATGCAGAATCAAACAAATTATAAACATGTGGCAGCAAGTAGGCAAAGCAATGTATGCCCCTCCCATCAGAGGACAAATTAGATGTTCATCAAATATCACAGGGCTGCTGTTAACAAGAGATGGTGGT------------------AACGAGAGCGAC---------------------------GCTGAGACCTTCAGACCTGGAGGAGGAGATATGAGGGACAATTGGAGA---AGTGAATTATACAAATATAAAGTAGTAAAAATTGAACCA---TTAGGAGTAGCACCC---TCCAAGGCAAAGAGAAAAGTGGTGCAGAGAGAA---AAAAGAGCAGTG---GGAACAATAGGA---GCTATG---TTCCTT---GGG---------TTCTTGGGA---GCAGCAGGAAGCACTATGGGCGCAGCGTCACTG---ACGCTGACGGTACAGGCCAGACTATTATTGTCTGGTATAGTGCAACAGCAGAACAATCTGCTGAGGGCTATTGAGGCGCAACAACATATTTTGCAACTCACGGTCTGGGGCATTAAACAGCTCCAGGCAAGA---GTCCTGGCTGTGGAAAGATACCTAAGGGATCAACAGCTCCTAGGGATTTGGGGTTGCTCTGGAAAACTCATCTGCACCACTGCTGTGCCTTGGAACGCTAGTTGGAGT---------------------------AATAAATCTCAAGAGGAGATTTGGGAG---AACATGACCTGGATGCAGTGGGATAGAGAAATTAAC------AATTACACAAACCTCATATATACCTTACTTGAAGAATCGCAGAACCAGCAAGAAAAGAATGAACAAGACTTATTAGCATTGGATGAATGGGCAAATTTGTGGAATTGGTTTGCCATATCAAAGTGGCTGTGGTATATAAAAATATTCATAATGATAGTAGGCGGCTTGATAGGTTTAAGAATAGTTTTTGCTGTACTTTCTATAGTGAATAGAGTTAGGCAGGGATACTCACCATTATCGTTTCAGACCCAC---CTCCCAGCCAGGAGGGGA------CCCGACAGGCCCGAAGGAATCGAAGAAGAAGGTGGAGAGAGAGACAGAGACAGATCAGTTCGCTTAGTACATGGATTCTTAGCACTCATCTGGGACGACCTACGGAGCCTGACCCTCTTCAGCTACCACCGCTTGAGAGACTTACTCTTGATTATAGCGAGGATTGTGGAAACTCTGGGACGCAGG---------------GGGTGGGAAGCCCTCAAGTATTGGTGG---AATCTCCTGCAATATTGG---------------------------------------------------AGTCAGGAACTAAAGAATAGTGCTGTTAGCTTGCTTGACGCCATAGCTATAGCAGTAGCTGAGGGAACAGATAGGGTTATAAAAGTAGTACGAAGA------------------GCCTTTAGAGCTATTCTCCACATACCTGTAAGAATAAGACAGGGCTTAGAAAGGGCTTTGCTATAA

2.SC22.Trinidad.EU576869 ATGAGAGTGAAGGAGATCAAGAGGAATTGTCAGCGCTTG---------TGGAGATGGGGC------------------------ACCATGCTCCTTGGGATGTTGATGATC------------TGTAGTGCTACA---------CAACAATTGTGGGTTACAGTCTATTATGGGGTACCTGTGTGGAAAGAAGCAACCACCACTCTATTTTGTGCATCAGATGCTAAAGCATATGATACAGAGGCACATAAT---GTCTGGGCCACACATGCCTGTGTACCCACAGACCCCAACCCACAAGAAGTAGTATTG---GGAAATGTGACAGAAAATTTTAACATGTGGAAAAATAACATGGTAGAACAAATGCATGAGGATATAATCAGTTTATGGGATCAAAGCCTAAAGCCATGTGTAAAATTAACCCCACTCTGTGTTACTTTAAACTGCACTGATTATGTGAAAAATGATACTAGTAGCACT---------------------------------------------------------------------------------------------AACAGTAGCAGCTGGGAGAAAGGAGAAATAAAAAACTGCTCTTTCAATATCACC---ACAAGCATAAAAGAT------AAGATGCAGAGGGAATATGCACTTTTTTATAAACTTGATGTAATACCAATAGATAGTGAGAATAATAGTAATATAAATAGTGGGAATAATAGTAATAGTAATTTTAGT------AGCTATAGTAATTTTAGATTGATAAATTGTAATACCTCAGTCATTACACAGGCCTGTCCAAAGGTATCCTTTGAACCAATTCCCATACATTATTGTGCCCCGGCTGGGTTTGCGATTCTAAAGTGT---AATGATAAAAAGTTCAATGGATCAGGACCATGTACAAATGTCAGCACAGTACAATGTACACATGGAATTAGACCAGTAGTGTCAACTCAACTGTTGTTAAATGGCAGTCTAGCAGAAGAG---GAGGTAGTAATTAGATCTGAAAATTTCACGAACAATGTTAAAACCATAATAGTACATCTGAATAAATCTGTAGTAATTAATTGTACAAGACCCAACAACAATACAAGAAGAGGTATACATATGGGA------------CCAGGGAAA---GCATATTTT---ACAGGAGAAATAATAGGAGATATAAGACAAGCACATTGTAACATT------AGTAGAAAAGAATGGAATGACACTTTAAAACAGATAGTTATAAAATTAGGA---GAACAATTTAGA------------AATAAAACA---ATAGCCTTTAATCAA---------TCCTCAGGAGGGGACATTGAAATTGTAACACACAGTTTTAATTGTGGAGGGGAATTTTTCTACTGTAATTCAACACAACTGTTTAATAGTACTTGGAATAAT---------AATGGTACTAGG------------------------------AATGATACTAGGGAGTCA------------AATGACACT---------------------------ATCATCACACTACCATGCAGAATCAAACAAATTATAAACATGTGGCAGCAAGTAGGCAAAGCAATGTATGCCCCTCCCATCAGAGGACAAATTAGATGTTCATCAAATATCACAGGGCTGCTGTTAACAAGAGATGGTGGT------------------AACGAGAGCGAC---------------------------GCTGAGACCTTCAGACCTGGAGGAGGAGATATGAGGGACAATTGGAGA---AGTGAATTATACAAATATAAAGTAGTAAAAATTGAACCA---TTAGGAGTAGCACCC---TCCAAGGCAAAGAGAAGAGTGGTGCAGAGAGAA---AAAAGAGCAGTG---GGAACAATAGGA---GCTATG---TTCCTT---GGG---------TTCTTGGGA---GCAGCAGGAAGCACTATGGGCGCAGCGTCACTG---ACGCTGACGGTACAGGCCAGACTATTATTGTCTGGTATAGTGCAACAGCAGAACAATCTGCTGAGGGCTATTGAGGCGCAACAACATATTTTGCAACTCACGGTCTGGGGCATTAAACAGCTCCAGGCAAGA---GTCCTGGCTGTGGAAAGATACCTAAGGGATCAACAGCTCCTAGGGATTTGGGGTTGCTCTGGAAAACTCATCTGCACCACTGCTGTGCCTTGGAACGCTAGTTGGAGT---------------------------AATAAATCTCAAGAGGAGATTTGGGAG---AACATGACCTGGATGCAGTGGGATAGAGAAATTAAC------AATTACACAAACCTCATATATACCTTACTTGAAGAATCGCAGAACCAGCAAGAAAAGAATGAACAAGACTTATTAGCATTGGATGAATGGGCAAATTTGTGGAATTGGTTTGCCATATCAAAGTGGCTGTGGTATATAAAAATATTCATAATGATAGTAGGCGGCTTGATAGGTTTAAGAATAGTTTTTGCTGTACTTTCTATAGTGAATAGAGTTAGGCAGGGATACTCACCATTATCGTTTCAGACCCAC---CTCCCAGCCAGGAGGGGA------CCCGACAGGCCCGAAGGAATCGAAGAAGAAGGTGGAGAGAGAGACAGAGACAGATCAGTTCGCTTAGTACATGGATTCTTAGCACTCATCTGGGACGACCTACGGAGCCTGACCCTCTTCAGCTACCACCGCTTGAGAGACTTACTCTTGATTATAGCGAGGATTGTGGAAACTCTGGGACGCAGG---------------GGGTGGGAAGCCCTCAAGTATTGGTGG---AATCTCCTGCAATATTGG---------------------------------------------------AGTCAGGAACTAAAGAATAGTGCTGTTAGCTTGCTTGACGCCATAGCTATAGCAGTAGCTGAGGGAACAGATAGGGTTATAAAAGTAGTACGAAGA------------------GCCTTTAGAGCTATTCTCCACATACCTGTAAGAATAAGACAGGGCTTAGAAAGGGCTTTGCTATAA

2.SC22.Trinidad.EU576873 ATGAGAGTGAAGGAGATCAAGAGGAATTGTCAGCGCTTG---------TGGAGATGGGGC------------------------ACCATGCTCCTTGGGATGTTGATGATC------------TGTAGTGCTACA---------CAACAATTGTGGGTTACAGTCTATTATGGGGTACCTGTGTGGAAAGAAGCAACCACCACTCTATTTTGTGCATCAGATGCTAAAGCATATGATACAGAGGCACATAAT---GTCTGGGTCACACATGCCTGTGTACCCACAGACCCCAACCCACAAGAAGTAGTATTG---GGAAATGTGACAGAAAATTTTAACATGTGGAAAAATAACATGGTAGAACAAATGCATGAGGATATAATCAGTTTATGGGATCAAAGCCTAAAGCCATGTGTAAAATTAACCCCACTCTGTGTTACTTTAAACTGCACTGATTATGTGAAAAATGATACTAGTAGCACT---------------------------------------------------------------------------------------------AACAGTAGCAGCTGGGAGAAAGGAGAAATAAAAAACTGCTCTTTCAATATCACC---ACAAGCATAAAAGAT------AAGATGCAGAGGGAATATGCACTTTTTTATAAACTTGATGTAATACCAATAGATAGTGAGAATAATAGTAATATAAATAGTGGGAATAATAGTAATAGTAATTTTAGT------AGCTATAGTAATTTTAGATTGATAAATTGTAATACCTCAGTCATTACACAGGCCTGTCCAAAGGTATCCTTTGAACCAATTCCCATACATTATTGTGCCCCGGCTGGGTTTGCGATTCTAAAGTGT---AATGATAAAAAGTTCAATGGATCAGGACCATGTACAAATGTCAGCACAGTACAATGTACACATGGAATTAGACCAGTAGTGTCAACTCAACTGTTGTTAAATGGCAGTCTAGCAGAAGAG---GAGGTAGTAATTAGATCTGAAAATTTCACGAACAATGTTAAAACCATAATAGTACATCTGAATAAATCTGTAGTAATTAATTGTACAAGACCCAACAACAATACAAGAAGAGGTATACATATGGGA------------CCAGGGAAA---GCATATTTT---ACAGGAGAAATAATAGGAGATATAAGACAAGCACATTGTAACATT------AGTAGAAAAGAATGGAATGACACTTTAAAACAGATAGTTATAAAATTAGGA---GAACAATTTAGA------------AATAAAACA---ATAGCCTTTAATCAA---------TCCTCAGGAGGGGACATTGAAATTGTAACACACAGTTTTAATTGTGGAGGGGAATTTTTCTACTGTAATTCAACACAACTGTTTAATAGTACTTGGAATAAT---------AATGGTACTAGG------------------------------AATGATACTAGGGAGTCA------------AATGACACT---------------------------ATCATCACACTACCATGCAGAATCAAACAAATTATAAACATGTGGCAGCAAGTAGGCAAAGCAATGTATGCCCCTCCCATCAGAGGACAAATTAGATGTTCATCAAATATCACAGGGCTGCTGTTAACAAGAGATGGTGGT------------------AACGAGAGCGAC---------------------------GCTGAGACCTTCAGACCTGGAGGAGGAGATATGAGGGACAATTGGAGA---AGTGAATTATACAAATATAAAGTAGTAAAAATTGAACCA---TTAGGAGTAGCACCC---TCCAAGGCAAAGAGAAGAGTGGTGCAGAGAGAA---AAAAGAGCAGTG---GGAACAATAGGA---GCTATG---TTCCTT---GGG---------TTCTTGGGA---GCAGCAGGAAGCACTATGGGCGCAGCGTCACTG---ACGCTGACGGTACAGGCCAGACTATTATTGTCTGGTATAGTGCAACAGCAGAACAATCTGCTGAGGGCTATTGAGGCGCAACAACATATTTTGCAACTCACGGTCTGGGGCATTAAACAGCTCCAGGCAAGA---GTCCTGGCTGTGGAAAGATACCTAAGGGATCAACAGCTCCTAGGGATTTGGGGTTGCTCTGGAAAACTCATCTGCACCACTGCTGTGCCTTGGAACGCTAGTTGGAGT---------------------------AATAAATCTCAAGAGGAGATTTGGGAG---AACATGACCTGGATGCAGTGGGATAGAGAAATTAAC------AATTACACAAACCTCATATATACCTTACTTGAAGAATCGCAGAACCAGCAAGAAAAGAATGAACAAGACTTATTAGCATTGGATGAATGGGCAAATTTGTGGAATTGGTTTGCCATATCAAAGTGGCTGTGGTATATAAAAATATTCATAATGATAGTAGGCGGCTTGATAGGTTTAAGAATAGTTTTTGCTGTACTTTCTATAGTGAATAGAGTTAGGCAGGGATACTCACCATTATCGTTTCAGACCCAC---CTCCCAGCCAGGAGGGGA------CCCGACAGGCCCGAAGGAATCGAAGAAGAAGGTGGAGAGAGAGACAGAGACAGATCAGTTCGCTTAGTACATGGATTCTTAGCACTCATCTGGGACGACCTACGGAGCCTGACCCTCTTCAGCTACCACCGCTTGAGAGACTTACTCTTGATTATAGCGAGGATTGTGGAAACTCTGGGACGCAGG---------------GGGTGGGAAGCCCTCAAGTATTGGTGG---AATCTCCTGCAATATTGG---------------------------------------------------AGTCAGGAACTAAAGAATAGTGCTGTTAGCTTGCTTGACGCCATAGCTATAGCAGTAGCTGAGGGAACAGATAGGGTTATAAAAGTAGTACGAAGA------------------GCCTTTAGAGCTATTCTCCACATACCTGTAAGAATAAGACAGGGCTTAGAAAGGGCTTTGCTATAA

2.SC22.Trinidad.EU576868 ATGAGAGTGAAGGAGATCAAGAGGAATTGTCAGCGCTTG---------TGGAGATGGGGC------------------------ACCATGCTCCTTGGGATGTTGATGATC------------TGTAGTGCTACA---------CAACAATTGTGGGTTACAGTCTATTATGGGGTACCTGTGTGGAAAGAAGCAACCACCACTCTATTTTGTGCATCAGATGCTAAAGCATATGATACAGAGGCACATAAT---GTCTGGGCCACACATGCCTGTGTACCCACAGACCCCAACCCACAAGAAGTAGTATTG---GGAAATGTGACAGAAAATTTTAACATGTGGAAAAATAACATGGTAGAACAAATGCATGAGGATATAATCAGTTTATGGGATCAAAGCCTAAAGCCATGTGTAAAATTAACCCCACTCTGTGTTACTTTAAACTGCACTGATTATGTGAAAAATGATACTAGTAGCACT---------------------------------------------------------------------------------------------AACAGTAGCAGCTGGGAGAAAGGAGAAATAAAAAACTGCTCTTTCAATATCACC---ACAAGCATAAAAGAT------AAGATGCAGAGGGAATATGCACTTTTTTATAAACTTGATGTAATACCAATAGATAGTGAGAATAATAGTAATATAAATAGTGGGAATAATAGTAATAGTAATTTTAGT------AGCTATAGTAATTTTAGATTGATAAATTGTAATACCTCAGTCATTACACAGGCCTGTCCAAAGGTATCCTTTGAACCAATTCCCATACATTATTGTGCCCCGGCTGGGTTTGCGATTCTAAAGTGT---AATGATAAAAAGTTCAATGGATCAGGACCATGTACAAATGTCAGCACAGTACAATGTACACATGGAATTAGACCAGTAGTGTCAACTCAACTGTTGTTAAATGGCAGTCTAGCAGAAGAG---GAGGTAGTAATTAGATCTGAAAATTTCACGAACAATGTTAAAACCATAATAGTACATCTGAATAAATCTGTAGTAATTAATTGTACAAGACCCAACAACAATACAAGAAGAGGTATACATATGGGA------------CCAGGGAAA---GCATATTTT---ACAGGAGAAATAATAGGAGATATAAGACAAGCACATTGTAACATT------AGTAGAAAAGAATGGAATGACACTTTAAAACAGATAGTTATAAAATTAGGA---GAACAATTTAGA------------AATAAAACA---ATAGCCTTTAATCAA---------TCCTCAGGAGGGGACATTGAAATTGTAACACACAGTTTTAATTGTGGAGGGGAATTTTTCTACTGTAATTCAACACAACTGTTTAATAGTACTTGGAATAAT---------AATGGTACTAGG------------------------------AATGATACTAGGGAGTCA------------AATGACACT---------------------------ATCATCACACTACCATGCAGAATCAAACAAATTATAAACATGTGGCAGCAAGTAGGCAAAGCAATGTATGCCCCTCCCATCAGAGGACAAATTAGATGTTCATCAAATATCACAGGGCTGCTGTTAACAAGAGATGGTGGT------------------AACGAGAGCGAC---------------------------GCTGAGACCTTCAGACCTGGAGGAGGAGATATGAGGGACAATTGGAGA---AGTGAATTATACAAATATAAAGTAGTAAAAATTGAACCA---TTAGGAGTAGCACCC---TCCAAGGCAAAGAGAAGAGTGGTGCAGAGAGAA---AAAAGAGCAGTG---GGAACAATAGGA---GCTATG---TTCCTT---GGG---------TTCTTGGGA---GCAGCAGGAAGCACTATGGGCGCAGCGTCACTG---ACGCTGACGGTACAGGCCAGACTATTATTGTCTGGTATAGTGCAACAGCAGAACAATCTGCTGAGGGCTATTGAGGCGCAACAACATATTTTGCAACTCACGGTCTGGGGCATTAAACAGCTCCAGGCAAGA---GTCCTGGCTGTGGAAAGATACCTAAGGGACCAACAGCTCCTAGGGATTTGGGGTTGCTCTGGAAAACTCATCTGCACCACTGCTGTGCCTTGGAACGCTAGTTGGAGT---------------------------AATAAATCTCAAGAGGAGATTTGGGAG---AACATGACCTGGATGCAGTGGGATAGAGAAATTAAC------AATTACACAAACCTCATATATACCTTACTTGAAGAATCGCAGAACCAGCAAGAAAAGAATGAACAAGACTTATTAGCATTGGATGAATGGGCAAATTTGTGGAATTGGTTTGCCATATCAAAGTGGCTGTGGTATATAAAAATATTCATAATGATAGTAGGCGGCTTGATAGGTTTAAGAATAGTTTTTGCTGTACTTTCTATAGTGAATAGAGTTAGGCAGGGATACTCACCATTATCGTTTCAGACCCAC---CTCCCAGCCAGGAGGGGA------CCCGACAGGCCCGAAGGAATCGAAGAAGAAGGTGGAGAGAGAGACAGAGACAGATCAGTTCGCTTAGTACATGGATTCTTAGCACTCATCTGGGACGACCTACGGAGCCTGACCCTCTTCAGCTACCACCGCTTGAGAGACTTACTCTTGATTATAGCGAGGATTGTGGAAACTCTGGGACGCAGG---------------GGGTGGGAAGCCCTCAAGTATTGGTGG---AATCTCCTGCAATATTGG---------------------------------------------------AGTCAGGAACTAAAGAATAGTGCTGTTAGCTTGCTTGACGCCATAGCTATAGCAGTAGCTGAGGGAACAGATAGGGTTATAAAAGTAGTACGAAGA------------------GCCTTTAGAGCTATTCTCCACATACCTGTAAGAATAAGACAGGGCTTAGAAAGGGCTTTGCTATAA

2.SC22.Trinidad.EU576860 ATGAGAGTGAAGGAGATCAAGAAGAATTGTCAGCGCTTG---------TGGAGATGGGGC------------------------ACCATGCTCCTTGGGATGTTGATGATC------------TGTAGTGCTACA---------CAACAATTGTGGGTTACAGTCTATTATGGGGTACCTGTGTGGAAAGAAGCAACCACCACTCTATTTTGTGCATCAGATGCTAAAGCATATGATACAGAGGCACATAAT---GTCTGGGCCACACATGCCTGTGTACCCACAGACCCCAACCCACAAGAAGTAGTATTG---GGAAATGTGACAGAAAATTTTAACATGTGGAAAAATAACATGGTAGAACAAATGCATGAGGATATAATCAGTTTATGGGATCAAAGCCTAAAGCCATGTGTAAAATTAACCCCACTCTGTGTTACTTTAAACTGCACTGATTATGTGAAAAATGATACTAGTAGCACT---------------------------------------------------------------------------------------------AACAGTAGCAGCTGGGAGAAAGGAGAAATAAAAAACTGCTCTTTCAATATCACC---ACAAGCATAAAAGAT------AAGATGCAGAGGGAATATGCACTTTTTTATAAACTTGATGTAATACCAATAGATAGTGAGAATAATAGTAATATAAATAGTGGGAATAATAGTAATAGTAATTTTAGT------AGCTATAGTAATTTTAGATTGATAAATTGTAATACCTCAGTCATTACACAGGCCTGTCCAAAGGTATCCTTTGAACCAATTCCCATACATTATTGTGCCCCGGCTGGGTTTGCGATTCTAAAGTGT---AATGATAAAAAGTTCAATGGATCAGGACCATGTACAAATGTCAGCACAGTACAATGTACACATGGAATTAGACCAGTAGTGTCAACTCAACTGTTGTTAAATGGCAGTCTAGCAGAAGAG---GAGGTAGTAATTAGATCTGAAAATTTCACGAACAATGTTAAAACCATAATAGTACATCTGAATAAATCTGTAGTAATTAATTGTACAAGACCCAACAACAATACAAGAAGAGGTATACATATGGGA------------CCAGGGAAA---GCATATTTT---ACAGGAGAAATAATAGGAGATATAAGACAAGCACATTGTAACATT------AGTAGAAAAGAATGGAATGACACTTTAAAACAGATAGTTATAAAATTAGGA---GAACAATTTAGA------------AATAAAACA---ATAGCCTTTAATCAA---------TCCTCAGGAGGGGACATTGAAATTGTAACACACAGTTTTAATTGTGGAGGGGAATTTTTCTACTGTAATTCAACACAACTGTTTAATAGTACTTGGAATAAT---------AATGGTACTAGG------------------------------AATGATACTAGGGAGTCA------------AATGACACT---------------------------ATCATCACACTACCATGCAGAATCAAACAAATTATAAACATGTGGCAGCAAGTAGGCAAAGCAATGTATGCCCCTCCCATCAGAGGACAAATTAGATGTTCATCAAATATCACAGGGCTGCTGTTAACAAGAGATGGTGGT------------------AACGAGAGCGAC---------------------------GCTGAGACCTTCAGACCTGGAGGAGGAGATATGAGGGACAATTGGAGA---AGTGAATTATACAAATATAAAGTAGTAAAAATTGAACCA---TTAGGAGTAGCACCC---TCCAAGGCAAAGAGAAGAGTGGTGCAGAGAGAA---AAAAGAGCAGTG---GGAACAATAGGA---GCTATG---TTCCTT---GGG---------TTCTTGGGA---GCAGCAGGAAGCACTATGGGCGCAGCGTCACTG---ACGCTGACGGTACAGGCCAGACTATTATTGTCTGGTATAGTGCAACAGCAGAACAATCTGCTGAGGGCTATTGAGGCGCAACAACATATTTTGCAACTCACGGTCTGGGGCATTAAACAGCTCCAGGCAAGA---GTCCTGGCTGTGGAAAGATACCTAAGGGATCAACAGCTCCTAGGGATTTGGGGTTGCTCTGGAAAACTCATCTGCACCACTGCTGTGCCTTGGAACGCTAGTTGGAGT---------------------------AATAAATCTCAAGAGGAGATTTGGGAG---AACATGACCTGGATGCAGTGGGATAGAGAAATTAAC------AATTACACAAACCTCATATATACCTTACTTGAAGAATCGCAGAACCAGCAAGAAAAGAATGAACAAGACTTATTAGCATTGGATGAATGGGCAAATTTGTGGAATTGGTTTGCCATATCAAAGTGGCTGTGGTATATAAAAATATTCATAATGATAGTAGGCGGCTTGATAGGTTTAAGAATAGTTTTTGCTGTACTTTCTATAGTGAATAGAGTTAGGCAGGGATACTCACCATTATCGTTTCAGACCCAC---CTCCCAGCCAGGAGGGGA------CCCGACAGGCCCGAAGGAATCGAAGAAGAAGGTGGAGAGAGAGACAGAGACAGATCAGTTCGCTTAGTACATGGATTCTTAGCACTCATCTGGGACGACCTACGGAGCCTGACCCTCTTCAGCTACCACCGCTTGAGAGACTTACTCTTGATTATAGCGAGGATTGTGGAAACTCTGGGACGCAGG---------------GGGTGGGAAGCCCTCAAGTATTGGTGG---AATCTCCTGCAATATTGG---------------------------------------------------AGTCAGGAACTAAAGAATAGTGCTGTTAGCTTGCTTGACGCCATAGCTATAGCAGTAGCTGAGGGAACAGATAGGGTTATAAAAGTAGTACGAAGA------------------GCCTTTAGAGCTATTCTCCACATACCTGTAAGAATAAGACAGGGCTTAGAAAGGGCTTTGCTATAA

2.SC22.Trinidad.EU576880 ATGAGAGTGAAGGAGATCAAGAGGAATTGTCAGCGCTTG---------TGGAGATGGGGC------------------------ACCATGCTCCTTGGGATGTTGATGATC------------TGTAGTGCTACA---------CAACAATTGTGGGTTACAGTCTATTATGGGGTACCTGTGTGGAAAGAAGCAACCACCACTCTATTTTGTGCATCAGATGCTAAAGCATATGATACAGAGGCACATAAT---GTCTGGGCCACACATGCCTGTGTACCCACAGACCCCAACCCACAAGAAGTAGTATTG---GGAAATGTGACAGAAAATTTTAACATGTGGAAAAATAACATGGTAGAACAAATGCATGAGGATATAATCAGTTTATGGGATCAAAGCCTAAAGCCATGTGTAAAATTAACCCCACTCTGTGTTACTTTAAACTGCACTGATTATGTGAAAAATGATACTAGTAGCACT---------------------------------------------------------------------------------------------AACAGTAGCAGCTGGGAGAAAGGAGAAATAAAAAACTGCTCTTTCAATATCACC---ACAAGCATAAAAGAT------AAGATGCAGAGGGAATATGCACTTTTTTATAAACTTGATGTAATACCAATAGATAGTGAGAATAATAGTAATATAAATAGTGGGAATAATAGTAATAGTAATTTTAGT------AGCTATAGTAATTTTAGATTGATAAATTGTAATACCTCAGTCATTACACAGGCCTGTCCAAAGGTATCCTTTGAACCAATTCCCATACATTATTGTGCCCCGGCTGGGTTTGCGATTCTAAAGTGT---AATGATAAAAAGTTCAATGGATCAGGACCATGTACAAATGTCAGCACAGTACAATGTACACATGGAATTAGACCAGTAGTGTCAACTCAACTGTTGTTAAATGGCAGTCTAGCAGAAGAG---GAGGTAGTAATTAGATCTGAAAATTTCACGAACAATGTTAAAACCATAATAGTACATCTGAATAAATCTGTAGTAATTAATTGTACAAGACCCAACAACAATACAAGAAGAGGTATACATATGGGA------------CCAGGGAAA---GCATATTTT---ACAGGAGAAATAATAGGAGATATAAGACAAGCACATTGTAACATT------AGTAGAAAAGAATGGAATGACACTTTAAAACAGATAGTTATAAAATTAGGA---GAACAATTTAGA------------AATAAAACA---ATAGCCTTTAATCAA---------TCCTCAGGAGGGGACATTGAAATTGTAACACACAGTTTTAATTGTGGAGGGGAATTTTTCTACTGTAATTCAACACAACTGTTTAATAGTACTTGGAATAAT---------AATGGTACTAGG------------------------------AATGATACTAGGGAGTCA------------AATGACACT---------------------------ATCATCACACTACCATGCAGAATCAAACAAATTATAAACATGTGGCAGCAAGTAGGCAAAGCAATGTATGCCCCTCCCATCAGAGGACAAATTAGATGTTCATCAAATATCACAGGGCTGCTGTTAACAAGAGATGGTGGT------------------AACGAGAGCGAC---------------------------GCTGAGACCTTCAGACCTGGAGGAGGAGATATGAGGGACAATTGGAGA---AGTGAATTATACAAATATAAAGTAGTAAAAATTGAACCA---TTAGGAGTAGCACCC---TCCAAGGCAAAGAGAAGAGTGGTGCAGAGAGAA---AAAAGAGCAGTG---GGAACAATAGGA---GCTATG---TTCCTT---GGG---------TTCTTGGGA---GCAGCAGGAAGCACTATGGGCGCAGCGTCACTG---ACGCTGACGGTACAGGCCAGACTATTATTGTCTGGTATAGTGCAACAGCAGAACAATCTGCTGAGGGCTATTGAGGCGCAACAACATATTTTGCAACTCACGGTCTGGGGCATTAAACAGCTCCAGGCAAGA---GTCCTGGCTGTGGAAAGATACCTAAGGGATCAACAGCTCCTAGGGATTTGGGGTTGCTCTGGAAAACTCATCTGCACCACTGCTGTGCCTTGGAACGCTAGTTGGAGT---------------------------AATAAATCTCAAGAGGAGATTTGGGAG---AACATGACCTGGATGCAGTGGGATAGAGAAATTAAC------AATTACACAAACCTCATATATACCTTACTTGAAGAATCGCAGAACCAGCAAGAAAAGAATGAACAAGACTTATTAGCATTGGATGAATGGGCAAATTTGTGGAATTGGTTTGCCATATCAAAGTGGCTGTGGTATATAAAAATATTCATAATGATAGTAGGCGGCTTGATAGGTTTAAGAATAGTTTTTGCTGTACTTTCTATAGTGAATAGAGTTAGGCAGGGATACTCACCATTATCGTTTCAGACCCAC---CTCCCAGCCAGGAGGGGA------CCCGACAGGCCCGAAGGAATCGAAGAAGAAGGTGGAGAGAGAGACAGAGACAGATCAGTTCGCTTAGTACATGGATTCTTAGCACTCATCTGGGACGACCTACGGAGCCTGACCCTCTTCAGCTACCACCGCTTGAGAGACTTACTCTTGATTATAGCGAGGATTGTGGAAACTCTGGGACGCAGG---------------GGGTGGGAAGCCCTCAAGTATTGGTGG---AATCTCCTGCAATATTGG---------------------------------------------------AGTCAGGAACTAAAGAATAGTGCTGTTAGCTTGCTTGACGCCATAGCTATAGCAGTAGCTGAGGGAACAGATAGGGTTATAAAAGTAGTACGAAGA------------------GCCTTTAGAGCTATTCTCCACATACCTGTAAGAATAAGACAGGGCTTAGAAAGGGCTTTGCTATAA

2.SC22.Trinidad.EU576878 ATGAGAGTGAAGGAGATCAAGAGGAATTGTCAGCGCTTG---------TGGAGATGGGGC------------------------ACCATGCTCCTTGGGATGTTGATGATC------------TGTAGTGCTACA---------CAACAATTGTGGGTTACAGTCTATTATGGGGTACCTGTGTGGAAAGAAGCAACCACCACTCTATTTTGTGCATCAGATGCTAAAGCATATGATACAGAGGCACATAAT---GTCTGGGCCACACATGCCTGTGTACCCACAGACCCCAACCCACAAGAAGTAGTATTG---GGAAATGTGACAGAAAATTTTAACATGTGGAAAAATAACATGGTAGAACAAATGCATGAGGATATAATCAGTTTATGGGATCAAAGCCTAAAGCCATGTGTAAAATTAACCCCACTCTGTGTTACTTTAAACTGCACTGATTATGTGAAAAATGATACTAGTAGCACT---------------------------------------------------------------------------------------------AACAGTAGCAGCTGGGAGAAAGGAGAAATAAAAAACTGCTCTTTCAATATCACC---ACAAGCATAAAAGAT------AAGATGCAGAGGGAATATGCACTTTTTTATAAACTTGATGTAATACCAATAGATAGTGAGAATAATAGTAATATAAATAGTGGGAATAATAGTAATAGTAATTTTAGT------AGCTATAGTAATTTTAGATTGATAAATTGTAATACCTCAGTCATTACACAGGCCTGTCCAAAGGTATCCTTTGAACCAATTCCCATACATTATTGTGCCCCGGCTGGGTTTGCGATTCTAAAGTGT---AATGATAAAAAGTTCAATGGATCAGGACCATGTACAAATGTCAGCACAGTACAATGTACACATGGAATTAGACCAGTAGTGTCAACTCAACTGTTGTTAAATGGCAGTCTAGCAGAAGAG---GAGGTAGTAATTAGATCTGAAAATTTCACGAACAATGTTAAAACCATAATAGTACATCTGAATAAATCTGTAGTAATTAATTGTACAAGACCCAACAACAATACAAGAAGAGGTATACATATGGGA------------CCAGGGAAA---GCATATTTT---ACAGGAGAAATAATAGGAGATATAAGACAAGCACATTGTAACATT------AGTAGAAAAGAATGGAATGACACTTTAAAACAGATAGTTATAAAATTAGGA---GAACAATTTAGA------------AATAAAACA---ATAGCCTTTAATCAA---------TCCTCAGGAGGGGACATTGAAATTGTAACACACAGTTTTAATTGTGGAGGGGAATTTTTCTACTGTAATTCAACACAACTGTTTAATAGTACTTGGAATAAT---------AATGGTACTAGG------------------------------AATGATACTAGGGAGTCA------------AATGACACT---------------------------ATCATCACACTACCATGCAGAATCAAACAAATTATAAACATGTGGCAGCAAGTAGGCAAAGCAATGTATGCCCCTCCCATCAGAGGACAAATTAGATGTTCATCAAATATCACAGGGCTGCTGTTAACAAGAGATGGTGGT------------------AACGAGAGCGAC---------------------------GCTGAGACCTTCAGACCTGGAGGAGGAGATATGAGGGACAATTGGAGA---AGTGAATTATACAAATATAAAGTAGTAAAAATTGAACCA---TTAGGAGTAGCACCC---TCCAAGGCAAAGAGAAGAGTGGTGCAGAGAGAA---AAAAGAGCAGTG---GGAACAATAGGA---GCTATG---TTCCTT---GGG---------TTCTTGGGA---GCAGCAGGAAGCACTATGGGCGCAGCGTCACTG---ACGCTGACGGTACAGGCCAGACTATTATTGTCTGGTATAGTGCAACAGCAGAACAATCTGCTGAGGGCTATTGAGGCGCAACAACATATTTTGCAACTCACGGTCTGGGGCATTAAACAGCTCCAGGCAAGA---GTCCTGGCTGTGGAAAGATACCTAAGGGATCAACAGCTCCTAGGGATTTGGGGTTGCTCTGGAAAACTCATCTGCACCACTGCTGTGCCTTGGAACGCTAGTTGGAGT---------------------------AATAAATCTCAAGAGGAGATTTGGGAG---AACATGACCTGGATGCAGTGGGATAGAGAAATTAAC------AATTACACAAACCTCATATATACCTTACTTGAAGAATCGCAGAACCAGCAAGAAAAGAATGAACAAGACTTATTAGCATTGGATGAATGGGCAAATTTGTGGAATTGGTTTGCCATATCAAAGTGGCTGTGGTATATAAAAATATTCATAATGATAGTAGGCGGCTTGATAGGTTTAAGAATAGTTTTTGCTGTACTTTCTATAGTGAATAGAGTTAGGCAGGGATACTCACCATTATCGTTTCAGACCCAC---CTCCCAGCCAGGAGGGGA------CCCGACAGGCCCGAAGGAATCGAAGAAGAAGGTGGAGAGAGAGACAGAGACAGATCAGTTCGCTTAGTACATGGATTCTTAGCACTCATCTGGGACGACCTACGGAGCCTGACCCTCTTCAGCTACCACCGCTTGAGAGACTTACTCTTGATTATAGCGAGGATTGTGGAAACTCTGGGACGCAGG---------------GGGTGGGAAGCCCTCAAGTATTGGTGG---AATCTCCTGCAATATTGG---------------------------------------------------AGTCAGGAACTAAAGAATAGTGCTGTTAGCTTGCTTGACGCCATAGCTATAGCAGTAGCTGAGGGAACAGATAGGGTTATAAAAGTAGTACGAAGA------------------GCCTTTAGAGCTATTCTCCACATACCTGTAAGAATAAGACAGGGCTTAGAAAGGGCTTTGCTATAA

2.SC22.Trinidad.EU576867 ATGAGAGTGAAGGAGATCAAGAGGAATTGTCAGCGCTTG---------TGGAGATGGGGC------------------------ACCATGCTCCTTGGGATGTTGATGATC------------TGTAGTGCTACA---------CAACAATTGTGGGTTACAGTCTATTATGGGGTACCTGTGTGGAAAGAAGCAACCACCACTCTATTTTGTGCATCAGATGCTAAAGCATATGATACAGAGGCACATAAT---GTCTGGGCCACACATGCCTGTGTACCCACAGACCCCAACCCACAAGAAGTAGTATTG---GGAAATGTGACAGAAAATTTTAACATGTGGAAAAATAACATGGTAGAACAAATGCATGAGGATATAATCAGTTTATGGGATCAAAGCCTAAAGCCATGTGTAAAATTAACCCCACTCTGTGTTACTTTAAACTGCACTGATTATGTGAAAAATGATACTAGTAGCACT---------------------------------------------------------------------------------------------AACAGTAGCAGCTGGGAGAAAGGAGAAATAAAAAACTGCTCTTTCAATATCACC---ACAAGCATAAAAGAT------AAGATGCAGAGGGAATATGCACTTTTTTATAAACTTGATGTAATACCAATAGATAGTGAGAATAATAGTAATATAAATAGTGGGAATAATAGTAATAGTAATTTTAGT------AGCTATAGTAATTTTAGATTGATAAATTGTAATACCTCAGTCATTACACAGGCCTGTCCAAAGGTATCCTTTGAACCAATTCCCATACATTATTGTGCCCCGGCTGGGTTTGCGATTCTAAAGTGT---AATGATAAAAAGTTCAATGGATCAGGACCATGTACAAATGTCAGCACAGTACAATGTACACATGGAATTAGACCAGTAGTGTCAACTCAACTGTTGTTAAATGGCAGTCTAGCAGAAGAG---GAGGTAGTAATTAGATCTGAAAATTTCACGAACAATGTTAAAACCATAATAGTACATCTGAATAAATCTGTAGTAATTAATTGTACAAGACCCAACAACAATACAAGAAGAGGTATACATATGGGA------------CCAGGGAAA---GCATATTTT---ACAGGAGAAATAATAGGAGATATAAGACAAGCACATTGTAACATT------AGTAGAAAAGAATGGAATGACACTTTAAAACAGATAGTTATAAAATTAGGA---GAACAATTTAGA------------AATAAAACA---ATAGCCTTTAATCAA---------TCCTCAGGAGGGGACATTGAAATTGTAACACACAGTTTTAATTGTGGAGGGGAATTTTTCTACTGTAATTCAACACAACTGTTTAATAGTACTTGGAATAAT---------AATGGTACTAGG------------------------------AATGATACTAGGGAGTCA------------AATGACACT---------------------------ATCATCACACTACCATGCAGAATCAAACAAATTATAAACATGTGGCAGCAAGTAGGCAAAGCAATGTATGCCCCTCCCATCAGAGGACAAATTAGATGTTCATCAAATATCACAGGGCTGCTGTTAACAAGAGATGGTGGT------------------AACGAGAGCGAC---------------------------GCTGAGACCTTCAGACCTGGAGGAGGAGATATGAGGGACAATTGGAGA---AGTGAATTATACAAATATAAAGTAGTAAAAATTGAACCA---TTAGGAGTAGCACCC---TCCAAGGCAAAGAGAAGAGTGGTGCAGAGAGAA---AAAAGAGCAGTG---GGAACAATAGGA---GCTATG---TTCCTT---GGG---------TTCTTGGGA---GCAGCAGGAAGCACTATGGGCGCAGCGTCACTG---ACGCTGACGGTACAGGCCAGACTATTATTGTCTGGTATAGTGCAACAGCAGAACAATCTGCTGAGGGCTATTGAGGCGCAACAACATATTTTGCAACTCACGGTCTGGGGCATTAAACAGCTCCAGGCAAGA---GTCCTGGCTGTGGAAAGATACCTAAGGGATCAACAGCTCCTAGGGATTTGGGGTTGCTCTGGAAAACTCATCTGCACCACTGCTGTGCCTTGGAACGCTAGTTGGAGT---------------------------AATAAATCTCAAGAGGAGATTTGGGAG---AACATGACCTGGATGCAGTGGGATAGAGAAATTAAC------AATTACACAAACCTCATATATACCTTACTTGAAGAATCGCAGAACCAGCAAGAAAAGAATGAACAAGACTTATTAGCATTGGATGAATGGGCAAATTTGTGGAATTGGTTTGCCATATCAAAGTGGCTGTGGTATATAAAAATATTCATAATGATAGTAGGCGGCTTGATAGGTTTAAGAATAGTTTTTGCTGTACTTTCTATAGTGAATAGAGTTAGGCAGGGATACTCACCATTATCGTTTCAGACCCAC---CTCCCAGCCAGGAGGGGA------CCCGACAGGCCCGAAGGAATCGAAGAAGAAGGTGGAGAGAGAGACAGAGACAGATCAGTTCGCTTAGTACATGGATTCTTAGCACTCATCTGGGACGACCTACGGAGCCTGACCCTCTTCAGCTACCACCGCTTGAGAGACTTACTCTTGATTATAGCGAGGATTGTGGAAACTCTGGGACGCAGG---------------GGGTGGGAAGCCCTCAAGTATTGGTGG---AATCTCCTGCAATATTGG---------------------------------------------------AGTCAGGAACTAAAGAATAGTGCTGTTAGCTTGCTTGACGCCATAGCTATAGCAGTAGCTGAGGGAACAGATAGGGTTATAAAAGTAGTACGAAGA------------------GCCTTTAGAGCTATTCTCCACATACCTGTAAGAATAAGACAGGGCTTAGAAAGGGCTTTGCTATAA

2.SC22.Trinidad.EU576872 ATGAGAGTGAAGGAGATCAAGAGGAATTGTCAGCGCTTG---------TGGAGATGGGGC------------------------ACCATGCTCCTTGGGATGTTGATGATC------------TGTAGTGCTACA---------CAACAATTGTGGGTTACAGTCTATTATGGGGTACCTGTGTGGAAAGAAGCAACCACCACTCTATTTTGTGCATCAGATGCTAAAGCATATGATACAGAGGCACATAAT---GTCTGGGCCACACATGCCTGTGTACCCACAGACCCCAACCCACAAGAAGTAGTATTG---GGAAATGTGACAGAAAATTTTAACATGTGGAAAAATAACATGGTAGAACAAATGCATGAGGATATAATCAGTTTATGGGATCAAAGCCTAAAGCCATGTGTAAAATTAACCCCACTCTGTGTTACTTTAAACTGCACTGATTATGTGAAAAATGATACTAGTAGCACT---------------------------------------------------------------------------------------------AACAGTAGCAGCTGGGAGAAAGGAGAAATAAAAAACTGCTCTTTCAATATCACC---ACAAGCATAAAAGAT------AAGATGCAGAGGGAATATGCACTTTTTTATAAACTTGATGTAATACCAATAGATAGTGAGAATAATAGTAATATAAATAGTGGGAATAATAGTAATAGTAATTTTAGT------AGCTATAGTAATTTTAGATTGATAAATTGTAATACCTCAGTCATTACACAGGCCTGTCCAAAGGTATCCTTTGAACCAATTCCCATACATTATTGTGCCCCGGCTGGGTTTGCGATTCTAAAGTGT---AATGATAAAAAGTTCAATGGATCAGGACCATGTACAAATGTCAGCACAGTACAATGTACACATGGAATTAGACCAGTAGTGTCAACTCAACTGTTGTTAAATGGCAGTCTAGCAGAAGAG---GAGGTAGTAATTAGATCTGAAAATTTCACGAACAATGTTAAAACCATAATAGTACATCTGAATAAATCTGTAGTAATTAATTGTACAAGACCCAACAACAATACAAGAAGAGGTATACATATGGGA------------CCAGGGAAA---GCATATTTT---ACAGGAGAAATAATAGGAGATATAAGACAAGCACATTGTAACATT------AGTAGAAAAGAATGGAATGACACTTTAAAACAGATAGTTATAAAATTAGGA---GAACAATTTAGA------------AATAAAACA---ATAGCCTTTAATCAA---------TCCTCAGGAGGGGACATTGAAATTGTAACACACAGTTTTAATTGTGGAGGGGAATTTTTCTACTGTAATTCAACACAACTGTTTAATAGTACTTGGAATAAT---------AATGGTACTAGG------------------------------AATGATACTAGGGAGTCA------------AATGACACT---------------------------ATCATCACACTACCATGCAGAATCAAACAAATTATAAACATGTGGCAGCAAGTAGGCAAAGCAATGTATGCCCCTCCCATCAGAGGACAAATTAGATGTTCATCAAATATCACAGGGCTGCTGTTAACAAGAGATGGTGGT------------------AACGAGAGCGAC---------------------------GCTGAGACCTTCAGACCTGGAGGAGGAGATATGAGGGACAATTGGAGA---AGTGAATTATACAAATATAAAGTAGTAAAAATTGAACCA---TTAGGAGTAGCACCC---TCCAAGGCAAAGAGAAGAGTGGTGCAGAGAGA----AAAAGAGCAGTG---GGAACAATAGGA---GCTATG---TTCCTT---GGG---------TTCTTGGGA---GCAGCAGGAAGCACTATGGGCGCAGCGTCACTG---ACGCTGACGGTACAGGCCAGACTATTATTGTCTGGTATAGTGCAACAGCAGAACAATCTGCTGAGGGCTATTGAGGCGCAACAACATATTTTGCAACTCACGGTCTGGGGCATTAAACAGCTCCAGGCAAGA---GTCCTGGCTGTGGAAAGATACCTAAGGGATCAACAGCTCCTAGGGATTTGGGGTTGCTCTGGAAAACTCATCTGCACCACTGCTGTGCCTTGGAACGCTAGTTGGAGT---------------------------AATAAATCTCAAGAGGAGATTTGGGAG---AACATGACCTGGATGCAGTGGGATAGAGAAATTAAC------AATTACACAAACCTCATATATACCTTACTTGAAGAATCGCAGAACCAGCAAGAAAAGAATGAACAAGACTTATTAGCATTGGATGAATGGGCAAATTTGTGGAATTGGTTTGCCATATCAAAGTGGCTGTGGTATATAAAAATATTCATAATGATAGTAGGCGGCTTGATAGGTTTAAGAATAGTTTTTGCTGTACTTTCTATAGTGAATAGAGTTAGGCAGGGATACTCACCATTATCGTTTCAGACCCAC---CTCCCAGCCAGGAGGGGA------CCCGACAGGCCCGAAGGAATCGAAGAAGAAGGTGGAGAGAGAGACAGAGACAGATCAGTTCGCTTAGTACATGGATTCTTAGCACTCATCTGGGACGACCTACGGAGCCTGACCCTCTTCAGCTACCACCGCTTGAGAGACTTACTCTTGATTATAGCGAGGATTGTGGAAACTCTGGGACGCAGG---------------GGGTGGGAAGCCCTCAAGTATTGGTGG---AATCTCCTGCAATATTGG---------------------------------------------------AGTCAGGAACTAAAGAATAGTGCTGTTAGCTTGCTTGACGCCATAGCTATAGCAGTAGCTGAGGGAACAGATAGGGTTATAAAAGTAGTACGAAGA------------------GCCTTTAGAGCTATTCTCCACATACCTGTAAGAATAAGACAGGGCTTAGAAAGGGCTTTGCTATAA

2.SC22.Trinidad.EU576886 ATGAGAGTGAAGGAGATCAAGAGGAATTGTCAGCGCTTG---------TGGAGATGGGGC------------------------ACCATGCTCCTTGGGATGTTGATGATC------------TGTAGTGCTACA---------CAACAATTGTGGGTTACAGTCTATTATGGGGTACCTGTGTGGAAAGAAGCAACCACCACTCTATTTTGTGCATCAGATGCTAAAGCATATGATACAGAGGCACATAAT---GTCTGGGCCACACATGCCTGTGTACCCACAGACCCCAACCCACAAGAAGTAGTATTG---GGAAATGTGACAGAAAATTTTAACATGTGGAAAAATAACATGGTAGAACAAATGCATGAGGATATAATCAGTTTATGGGATCAAAGCCTAAAGCCATGTGTAAAATTAACCCCACTCTGTGTTACTTTAAACTGCACTGATTATGTGAAAAATGATACTAGTAGCACT---------------------------------------------------------------------------------------------AACAGTAGCAGCTGGGAGAAAGGAGAAATAAAAAACTGCTCTTTCAATATCACC---ACAAGCATAAAAGAT------AAGATGCAGAGGGAATATGCACTTTTTTATAAACTTGATGTAATACCAATAGATAGTGAGAATAATAGTAATATAAATAGTGGGAATAATAGTAATAGTAATTTTAGT------AGCTATAGTAATTTTAGATTGATAAATTGTAATACCTCAGTCATTACACAGGCCTGTCCAAAGGTATCCTTTGAACCAATTCCCATACATTATTGTGCCCCGGCTGGGTTTGCGATTCTAAAGTGT---AATGATAAAAAGTTCAATGGATCAGGACCATGTACAAATGTCAGCACAGTACAATGTACACATGGAATTAGACCAGTAGTGTCAACTCAACTGTTGTTAAATGGCAGTCTAGCAGAAGAG---GAGGTAGTAATTAGATCTGAAAATTTCACGAACAATGTTAAAACCATAATAGTACATCTGAATAAATCTGTAGTAATTAATTGTACAAGACCCAACAACAATACAAGAAGAGGTATACATATGGGA------------CCAGGGAAA---GCATATTTT---ACAGGAGAAATAATAGGAGATATAAGACAAGCACATTGTAACATT------AGTAGAAAAGAATGGAATGACACTTTAAAACAGATAGTTATAAAATTAGGA---GAACAATTTAGA------------AATAAAACA---ATAGCCTTTAATCAA---------TCCTCAGGAGGGGACATTGAAATTGTAACACACAGTTTTAATTGTGGAGGGGAATTTTTCTACTGTAATTCAACACAACTGTTTAATAGTACTTGGAATAAT---------AATGGTACTAGG------------------------------AATGATACTAGGGAGTCA------------AATGACACT---------------------------ATCATCACACTACCATGCAGAATCAAACAAATTATAAACATGTGGCAGCAAGTAGGCAAAGCAATGTATGCCCCTCCCATCAGAGGACAAATTAGATGTTCATCAAATATCACAGGGCTGCTGTTAACAAGAGATGGTGGT------------------AACGAGAGCGAC---------------------------GCTGAGACCTTCAGACCTGGAGGAGGAGATATGAGGGACAATTGGAGA---AGTGAATTATACAAATATAAAGTAGTAAAAATTGAACCA---TTAGGAGTAGCACCC---TCCAAGGCAAAGAGAAGAGTGGTGCAGAGAGAA---AAAAGAGCAGTG---GGAACAATAGGA---GCTATG---TTCCTT---GGG---------TTCTTGGGA---GCAGCAGGAAGCACTATGGGCGCAGCGTCACTG---ACGCTGACGGTACAGGCCAGACTATTATTGTCTGGTATAGTGCAACAGCAGAACAATCTGCTGAGGGCTATTGAGGCGCAACAACATATTTTGCAACTCACGGTCTGGGGCATTAAACAGCTCCAGGCAAGA---GTCCTGGCTGTGGAAAGATACCTAAGGGATCAACAGCTCCTAGGGATTTGGGGTTGCTCTGGAAAACTCATCTGCACCACTGCTGTGCCTTGGAACGCTAGTTGGAGT---------------------------AATAAATCTCAAGAGGAGATTTGGGAG---AACATGACCTGGATGCAGTGGGATAGAGAAATTAAC------AATTACACAAACCTCATATATACCTTACTTGAAGAATCGCAGAACCAGCAAGAAAAGAATGAACAAGACTTATTAGCATTGGATGAATGGGCAAATTTGTGGAATTGGTTTGCCATATCAAAGTGGCTGTGGTATATAAAAATATTCATAATGATAGTAGGCGGCTTGATAGGTTTAAGAATAGTTTTTGCTGTACTTTCTATAGTGAATAGAGTTAGGCAGGGATACTCACCATTATCGTTTCAGACCCAC---CTCCCAGCCAGGAGGGGA------CCCGACAGGCCCGAAGGAATCGAAGAAGAAGGTGGAGAGAGAGACAGAGACAGATCAGTTCGCTTAGTACATGGATTCTTAGCACTCATCTGGGACGACCTACGGAGCCTGACCCTCTTCAGCTACCACCGCTTGAGAGACTTACTCTTGATTATAGCGAGGATTGTGGAAACTCTGGGACGCAGG---------------GGGTGGGAAGCCCTCAAGTATTGGTGG---AATCTCCTGCAATATTGG---------------------------------------------------AGTCAGGAACTAAAGAATAGTGCTGTTAGCTTGCTTGACGCCATAGCTATAGCAGTAGCTGAGGGAACAGATAGGGTTATAAAAGTAGTACGAAGA------------------GCCTTTAGAGCTATTCTCCACATACCTGTAAGAATAAGACAGGGCTTAGAAAGGGCTTTGCTATAA

2.SC22.Trinidad.EU576858 ATGAGAGTGAAGGAGATCAAGAGGAATTGTCAGCGCTTG---------TGGAGATGGGGC------------------------ACCATGCTCCTTGGGATGTTGATGATC------------TGTAGTGCTACA---------CAACAATTGTGGGTTACAGTCTATTATGGGGTACCTGTGTGGAAAGAAGCAACCACCACTCTATTTTGTGCATCAGATGCTAAAGCATATGATACAGAGGCACATAAT---GTCTGGGCCACACATGCCTGTGTACCCACAGACCCCAACCCACAAGAAGTAGTATTG---GGAAATGTGACAGAAAATTTTAACATGTGGAAAAATAACATGGTAGAACAAATGCATGAGGATATAATCAGTTTATGGGATCAAAGCCTAAAGCCATGTGTAAAATTAACCCCACTCTGTGTTACTTTAAACTGCACTGATTATGTGAAAAATGATACTAGTAGCACT---------------------------------------------------------------------------------------------AACAGTAGCAGCTGGGAGAAAGGAGAAATAAAAAACTGCTCTTTCAATATCACC---ACAAGCATAAAAGAT------AAGATGCAGAGGGAATATGCACTTTTTTATAAACTTGATGTAATACCAATAGATAGTGAGAATAATAGTAATATAAATAGTGGGAATAATAGTAATAGTAATTTTAGT------AGCTATAGTAATTTTAGATTGATAAATTGTAATACCTCAGTCATTACACAGGCCTGTCCAAAGGTATCCTTTGAACCAATTCCCATACATTATTGTGCCCCGGCTGGGTTTGCGATTCTAAAGTGT---AATGATAAAAAGTTCAATGGATCAGGACCATGTACAAATGTCAGCACAGTACAATGTACACATGGAATTAGACCAGTAGTGTCAACTCAACTGTTGTTAAATGGCAGTCTAGCAGAAGAG---GAGGTAGTAATTAGATCTGAAAATTTCACGAACAATGTTAAAACCATAATAGTACATCTGAATAAATCTGTAGTAATTAATTGTACAAGACCCAACAACAATACAAGAAGAGGTATACATATGGGA------------CCAGGGAAA---GCATATTTT---ACAGGAGAAATAATAGGAGATATAAGACAAGCACATTGTAACATT------AGTAAAAAAGAATGGAATGACACTTTAAAACAGATAGTTATAAAATTAGGA---GAACAATTTAGA------------AATAAAACA---ATAGCCTTTAATCAA---------TCCTCAGGAGGGGACATTGAAATTGTAACACACAGTTTTAATTGTGGAGGGGAATTTTTCTACTGTAATTCAACACAACTGTTTAATAGTACTTGGAATAAT---------AATGGTACTAGG------------------------------AATGATACTAGGGAGTCA------------AATGACACT---------------------------ATCATCACACTACCATGCAGAATCAAACAAATTATAAACATGTGGCAGCAAGTAGGCAAAGCAATGTATGCCCCTCCCATCAGAGGACAAATTAGATGTTCATCAAATATCACAGGGCTGCTGTTAACAAGAGATGGTGGT------------------AACGAGAGCGAC---------------------------GCTGAGACCTTCAGACCTGGAGGAGGAGATATGAGGGACAATTGGAGA---AGTGAATTATACAAATATAAAGTAGTAAAAATTGAACCA---TTAGGAGTAGCACCC---TCCAAGGCAAAGAGAAGAGTGGTGCAGAGAGAA---AAAAGAGCAGTG---GGAACAATAGGA---GCTATG---TTCCTT---GGG---------TTCTTGGGA---GCAGCAGGAAGCACTATGGGCGCAGCGTCACTG---ACGCTGACGGTACAGGCCAGACTATTATTGTCTGGTATAGTGCAACAGCAGAACAATCTGCTGAGGGCTATTGAGGCGCAACAACATATTTTGCAACTCACGGTCTGGGGCATTAAACAGCTCCAGGCAAGA---GTCCTGGCTGTGGAAAGATACCTAAGGGATCAACAGCTCCTAGGGATTTGGGGTTGCTCTGGAAAACTCATCTGCACCACTGCTGTGCCTTGGAACGCTAGTTGGAGT---------------------------AATAAATCTCAAGAGGAGATTTGGGAG---AACATGACCTGGATGCAGTGGGATAGAGAAATTAAC------AATTACACAAACCTCATATATACCTTACTTGAAGAATCGCAGAACCAGCAAGAAAAGAATGAACAAGACTTATTAGCATTGGATGAATGGGCAAATTTGTGGAATTGGTTTGCCATATCAAAGTGGCTGTGGTATATAAAAATATTCATAATGATAGTAGGCGGCTTGATAGGTTTAAGAATAGTTTTTGCTGTACTTTCTATAGTGAATAGAGTTAGGCAGGGATACTCACCATTATCGTTTCAGACCCAC---CTCCCAGCCAGGAGGGGA------CCCGACAGGCCCGAAGGAATCGAAGAAGAAGGTGGAGAGAGAGACAGAGACAGATCAGTTCGCTTAGTACATGGATTCTTAGCACTCATCTGGGACGACCTACGGAGCCTGACCCTCTTCAGCTACCACCGCTTGAGAGACTTACTCTTGATTATAGCGAGGATTGTGGAAACTCTGGGACGCAGG---------------GGGTGGGAAGCCCTCAAGTATTGGTGG---AATCTCCTGCAATATTGG---------------------------------------------------AGTCAGGAACTAAAGAATAGTGCTGTTAGCCTGCTTGACGCCATAGCTATAGCAGTAGCTGAGGGAACAGATAGGGTTATAAAAGTAGTACGAAGA------------------GCCTTTAGAGCTATTCTCCACATACCTGTAAGAATAAGACAGGGCTTAGAAAGGGCTTTGCTATAA

2.SC22.Trinidad.EU576870 ATGAGAGTGAAGGAGATCAAGAGGAATTGTCAGCGCTTG---------TGGAGATGGGGC------------------------ACCATGCTCCTTGGGATGTTGATGATC------------TGTAGTGCTACA---------CAACAATTGTGGGTTACAGTCTATTATGGGGTACCTGTGTGGAAAGAAGCAACCACCACTCTATTTTGTGCATCAGATGCTAAAGCATATGATACAGAGGCACATAAT---GTCTGGGCCACACATGCCTGTGTACCCACAGACCCCAACCCACAAGAAGTAGTATTG---GGAAATGTGACAGAAAATTTTAACATGTGGAAAAATAACATGGTAGAACAAATGCATGAGGATATAATCAGTTTATGGGATCAAAGCCTAAAGCCATGTGTAAAATTAACCCCACTCTGTGTTACTTTAAACTGCACTGATTATGTGAAAAATGATACTAGTAGCACT---------------------------------------------------------------------------------------------AACAGTAGCAGCTGGGAGAAAGGAGAAATAAAAAACTGCTCTTTCAATATCACC---ACAAGCATAAAAGAT------AAGATGCAGAGGGAATATGCACTTTTTTATAAACTTGATGTAATACCAATAGATAGTGAGAATAATAGTAATATAAATAGTGGGAATAATAGTAATAGTAATTTTAGT------AGCTATAGTAATTTTAGATTGATAAATTGTAATACCTCAGTCATTACACAGGCCTGTCCAAAGGTATCCTTTGAACCAATTCCCATACATTATTGTGCCCCGGCTGGGTTTGCGATTCTAAAGTGT---AATGATAAAAAGTTCAATGGATCAGGACCATGTACAAATGTCAGCACAGTACAATGTACACATGGAATTAGACCAGTAGTGTCAACTCAACTGTTGTTAAATGGCAGTCTAGCAGAAGAG---GAGGTAGTAATTAGATCTGAAAATTTCACGAACAATGTTAAAACCATAATAGTACATCTGAATAAATCTGTAGTAATTAATTGTACAAGACCCAACAACAATACAAGAAGAGGTATACATATGGGA------------CCAGGGAAA---GCATATTTT---ACAGGAGAAATAATAGGAGATATAAGACAAGCACATTGTAACATT------AGTAGAAAAGAATGGAATGACACTTTAAAACAGATAGTTATAAAATTAGGA---GAACAATTTAGA------------AATAAAACA---ATAGCCTTTAATCAA---------TCCTCAGGAGGGGACATTGAAATTGTAACACACAGTTTTAATTGTGGAGGGGAATTTTTCTACTGTAATTCAACACAACTGTTTAATAGTACTTGGAATAAT---------AATGGTACTAGG------------------------------AATGATACTAGGGAGTCA------------AATGACACT---------------------------ATCATCACACTACCATGCAGAATCAAACAAATTATAAACATGTGGCAGCAAGTAGGCAAAGCAATGTATGCCCCTCCCATCAGAGGACAAATTAGATGTTCATCAAATATCACAGGGCTGCTGTTAACAAGAGATGGTGGT------------------AACGAGAGCGAC---------------------------GCTGAGACCTTCAGACCTGGAGGAGGAGATATGAGGGACAATTGGAGA---AGTGAATTATACAAATATAAAGTAGTAAAAATTGAACCA---TTAGGAGTAGCACCC---TCCAAGGCAAAGAGAAGAGTGGTGCAGAGAGAA---AAAAGAGCAGTG---GGAACAATAGGA---GCTATG---TTCCTT---GGG---------TTCTTGGGA---GCAGCAGGAAGCACTATGGGCGCAGCGTCACTG---ACGCTGACGGTACAGGCCAGACTATTATTGTCTGGTATAGTGCAACAGCAGAACAATCTGCTGAGGGCTATTGAGGCGCAACAACATATTTTGCAACTCACGGTCTGGGGCATTAAACAGCTCCAGGCAAGA---GTCCTGGCTGTGGAAAGATACCTAAGGGATCAACAGCTCCTAGGGATTTGGGGTTGCTCTGGAAAACTCATCTGCACCACTGCTGTGCCTTGGAACGCTAGTTGGAGT---------------------------AATAAATCTCAAGAGGAGATTTGGGAG---AACATGACCTGGATGCAGTGGGATAGAGAAATTAAC------AATTACACAAACCTCATATATACCTTACTTGAAGAATCGCAGAACCAGCAAGAAAAGAATGAACAAGACTTATTAGCATTGGATGAATGGGCAAATTTGTGGAATTGGTTTGCCATATCAAAGTGGCTGTGGTATATAAAAATATTCATAATGATAGTAGGCGGCTTGATAGGTTTAAGAATAGTTTTTGCTGTACTTTCTATAGTGAATAGAGTTAGGCAGGGATACTCACCATTATCGTTTCAGACCCAC---CTCCCAGCCAGGAGGGGA------CCCGACAGGCCCGAAGGAATCGAAGAAGAAGGTGGAGAGAGAGACAGAGACAGATCAGTTCGCTTAGTACATGGATTCTTAGCACTCATCTGGGACGACCTACGGAGCCTGACCCTCTTCAGCTACCACCGCTTGAGAGACTTACTCTTGATTATAGCGAGGATTGTGGAAACTCTGGGGCGCAGG---------------GGGTGGGAAGCCCTCAAGTATTGGTGG---AATCTCCTGCAATATTGG---------------------------------------------------AGTCAGGAACTAAAGAATAGTGCTGTTAGCTTGCTTGACGCCATAGCTATAGCAGTAGCTGAGGGAACAGATAGGGTTATAAAAGTAGTACGAAGA------------------GCCTTTAGAGCTATTCTCCACATACCTGTAAGAATAAGACAGGGCTTAGAAAGGGCTTTGCTATAA

2.SC22.Trinidad.EU576865 ATGAGAGTGAAGGAGATCAAGAGGAATTGTCAGCGCTTG---------TGGAGATGGGGC------------------------ACCATGCTCCTTGGGATGTTGATGATC------------TGTAGTGCTACA---------CAACAATTGTGGGTTACAGTCTATTATGGGGTACCTGTGTGGAAAGAAGCAACCACCACTCTATTTTGTGCATCAGATGCTAAAGCATATGATACAGAGGCACATAAT---GTCTGGGCCACACATGCCTGTGTACCCACAGACCCCAACCCACAAGAAGTAGTATTG---GGAAATGTGACAGAAAATTTTAACATGTGGAAAAATAACATGGTAGAACAAATGCATGAGGATATAATCAGTTTATGGGATCAAAGCCTAAAGCCATGTGTAAAATTAACCCCACTCTGTGTTACTTTAAACTGCACTGATTATGTGAAAAATGATACTAGTAGCACT---------------------------------------------------------------------------------------------AACAGTAGCAGCTGGGAGAAAGGAGAAATAAAAAACTGCTCTTTCAATATCACC---ACAAGCATAAAAGAT------AAGATGCAGAGGGAATATGCACTTTTTTATAAACTTGATGTAATACCAATAGATAGTGAGAATAATAGTAATATAAATAGTGGGAATAATAGTAATAGTAATTTTAGT------AGCTATAGTAATTTTAGATTGATAAATTGTAATACCTCAGTCATTACACAGGCCTGTCCAAAGGTATCCTTTGAACCAATTCCCATACATTATTGTGCCCCGGCTGGGTTTGCGATTCTAAAGTGT---AATGATAAAAAGTTCAATGGATCAGGACCATGTACAAATGTCAGCACAGTACAATGTACACATGGAATTAGACCAGTAGTGTCAACTCAACTGTTGTTAAATGGCAGTCTAGCAGAAGAG---GAGGTAGTAATTAGATCTGAAAATTTCACGAACAATGTTAAAACCATAATAGTACATCTGAATAAATCTGTAGTAATTAATTGTACAAGACCCAACAACAATACAAGAAGAGGTATACATATGGGA------------CCAGGGAAA---GCATATTTT---ACAGGAGAAATAATAGGAGATATAAGACAAGCACATTGTAACATT------AGTAGAAAAGAATGGAATGACACTTTAAAACAGATAGTTATAAAATTAGGA---GAACAATTTAGA------------AATAAAACA---ATAGCCTTTAATCAA---------TCCTCAGGAGGGGACATTGAAATTGTAACACACAGTTTTAATTGTGGAGGGGAATTTTTCTACTGTAATTCAACACAACTGTTTAATAGTACTTGGAATAAT---------AATGGTACTAGG------------------------------AATGATACTAGGGAGTCA------------AATGACACT---------------------------ATCATCACACTACCATGCAGAATCAAACAAATTATAAACATGTGGCAGCAAGTAGGCAAAGCAATGTATGCCCCTCCCATCAGAGGACAAATTAGATGTTCATCAAATATCACAGGGCTGCTGTTAACAAGAGATGGTGGT------------------AACGAGAGCGAC---------------------------GCTGAGACCTTCAGACCTGGAGGAGGAGATATGAGGGACAATTGGAGA---AGTGAATTATACAAATATAAAGTAGTAAAAATTGAACCA---TTAGGAGTAGCACCC---TCCAAGGCAAAGAGAAGAGTGGTGCAGAGAGAA---AAAAGAGCAGTG---GGAACAATAGGA---GCTATG---TTCCTT---GGG---------TTCTTGGGA---GCAGCAGGAAGCACTATGGGCGCAGCGTCACTG---ACGCTGACGGTACAGGCCAGACTATTATTGTCTGGTATAGTGCAACAGCAGAACAATCTGCTGAGGGCTATTGAGGCGCAACAACATATTTTGCAACTCACGGTCTGGGGCATTAAACAGCTCCAGGCAAGA---GTCCTGGCTGTGGAAAGATACCTAAGGGATCAACAGCTCCTAGGGATTTGGGGTTGCTCTGGAAAACTCATCTGCACCACTGCTGTGCCTTGGAACGCTAGTTGGAGT---------------------------AATAAATCTCAAGAGGAGATTTGGGAG---AACATGACCTGGATGCAGTGGGATAGAGAAATTAAC------AATTACACAAACCTCATATATACCTTACTTGAAGAATCGCAGAACCAGCAAGAAAAGAATGAACAAGACTTATTAGCATTGGATGAATGGGCAAATTTGTGGAATTGGTTTGCCATATCAAAGTGGCTGTGGTATATAAAAATATTCATAATGATAGTAGGCGGCTTGATAGGTTTAAGAATAGTTTTTGCTGTACTTTCTATAGTGAATAGAGTTAGGCAGGGATACTCACCATTATCGTTTCAGACCCAC---CTCCCAGCCAGGAGGGGA------CCCGACAGGCCCGAAGGAATCGAAGAAGAAGGTGGAGAGAGAGACAGAGACAGATCAGTTCGCTTAGTACATGGATTCTTAGCACTCATCTGGGACGACCTACGGAGCCTGACCCTCTTCAGCTACCACCGCTTGAGAGACTTACTCTTGATTATAGCGAGGATTGTGGAAACTCTGGGACGCAGG---------------GGGTGGGAAGCCCTCAAGTATTGGTGG---AATCTCCTGCAATATTGG---------------------------------------------------AGTCAGGAACTAAAGAATAGTGCTGTTAGCTTGCTTGACGCCATAGCTATAGCAGTAGCTGAGGGAACAGATAGGGTTATAAAAGTAGTACGAAGA------------------GCCTTTAGAGCTATTCTCCACATACCTGTAAGAATAAGACAGGGCTTAGAAAGGGCTTTGCTATAA

2.SC22.Trinidad.EU576864 ATGAGAGTGAAGGAGATCAAGAGGAATTGTCAGCGCTTG---------TGGAGATGGGGC------------------------ACCATGCTCCTTGGGATGTTGATGATC------------TGTAGTGCTACA---------CAACAATTGTGGGTTACAGTCTATTATGGGGTACCTGTGTGGAAAGAAGCAACCACCACTCTATTTTGTGCATCAGATGCTAAAGCATATGATACAGAGGCACATAAT---GTCTGGGCCACACATGCCTGTGTACCCACAGACCCCAACCCACAAGAAGTAGTATTG---GGAAATGTGACAGAAAATTTTAACATGTGGAAAAATAACATGGTAGAACAAATGCATGAGGATATAATCAGTTTATGGGATCAAAGCCTAAAGCCATGTGTAAAATTAACCCCACTCTGTGTTACTTTAAACTGCACTGATTATGTGAAAAATGATACTAGTAGCACT---------------------------------------------------------------------------------------------AACAGTAGCAGCTGGGAGAAAGGAGAAATAAAAAACTGCTCTTTCAATATCACC---ACAAGCATAAAAGAT------AAGATGCAGAGGGAATATGCACTTTTTTATAAACTTGATGTAATACCAATAGATAGTGAGAATAATAGTAATATAAATAGTGGGAATAATAGTAATAGTAATTTTAGT------AGCTATAGTAATTTTAGATTGATAAATTGTAATACCTCAGTCATTACACAGGCCTGTCCAAAGGTATCCTTTGAACCAATTCCCATACATTATTGTGCCCCGGCTGGGTTTGCGATTCTAAAGTGT---AATGATAAAAAGTTCAATGGATCAGGACCATGTACAAATGTCAGCACAGTACAATGTACACATGGAATTAGACCAGTAGTGTCAACTCAACTGTTGTTAAATGGCAGTCTAGCAGAAGAG---GAGGTAGTAATTAGATCTGAAAATTTCACGAACAATGTTAAAACCATAATAGTACATCTGAATAAATCTGTAGTAATTAATTGTACAAGACCCAACAACAATACAAGAAGAGGTATACATATGGGA------------CCAGGGAAA---GCATATTTT---ACAGGAGAAATAATAGGAGATATAAGACAAGCACATTGTAACATT------AGTAGAAAAGAATGGAATGACACTTTAAAACAGATAGTTATAAAATTAGGA---GAACAATTTAGA------------AATAAAACA---ATAGCCTTTAATCAA---------TCCTCAGGAGGGGACATTGAAATTGTAACACACAGTTTTAATTGTGGAGGGGAATTTTTCTACTGTAATTCAACACAACTGTTTAATAGTACTTGGAATAAT---------AATGGTACTAGG------------------------------AATGATACTAGGGAGTCA------------AATGACACT---------------------------ATCATCACACTACCATGCAGAATCAAACAAATTATAAACATGTGGCAGCAAGTAGGCAAAGCAATGTATGCCCCTCCCATCAGAGGACAAATTAGATGTTCATCAAATATCACAGGGCTGCTGTTAACAAGAGATGGTGGT------------------AACGAGAGCGAC---------------------------GCTGAGACCTTCAGACCTGGAGGAGGAGATATGAGGGACAATTGGAGA---AGTGAATTATACAAATATAAAGTAGTAAAAATTGAACCA---TTAGGAGTAGCACCC---TCCAAGGCAAAGAGAAGAGTGGTGCAGAGAGAA---AAAAGAGCAGTG---GGAACAATAGGA---GCTATG---TTCCTT---GGG---------TTCTTGGGA---GCAGCAGGAAGCACTATGGGCGCAGCGTCACTG---ACGCTGACGGTACAGGCCAGACTATTATTGTCTGGTATAGTGCAACAGCAGAACAATCTGCTGAGGGCTATTGAGGCGCAACAACATATTTTGCAACTCACGGTCTGGGGCATTAAACAGCTCCAGGCAAGA---GTCCTGGCTGTGGAAAGATACCTAAGGGATCAACAGCTCCTAGGGATTTGGGGTTGCTCTGGAAAACTCATCTGCACCACTGCTGTGCCTTGGAACGCTAGTTGGAGT---------------------------AATAAATCTCAAGAGGAGATTTGGGAG---AACATGACCTGGATGCAGTGGGATAGAGAAATTAAC------AATTACACAAACCTCATATATACCTTACTTGAAGAATCGCAGAACCAGCAAGAAAAGAATGAACAAGACTTATTAGCATTGGATGAATGGGCAAATTTGTGGAATTGGTTTGCCATATCAAAGTGGCTGTGGTATATAAAAATATTCATAATGATAGTAGGCGGCTTGATAGGTTTAAGAATAGTTTTTGCTGTACTTTCTATAGTGAATAGAGTTAGGCAGGGATACTCACCATTATCGTTTCAGACCCAC---CTCCCAGCCAGGAGGGGA------CCCGACAGGCCCGAAGGAATCGAAGAAGAAGGTGGAGAGAGAGACAGAGACAGATCAGTTCGCTTAGTACATGGATTCTTAGCACTCATCTGGGACGACCTACGGAGCCTGACCCTCTTCAGCTACCACCGCTTGAGAGACTTACTCTTGATTATAGCGAGGATTGTGGAAACTCTGGGACGCAGG---------------GGGTGGGAAGCCCTCAAGTATTGGTGG---AATCTCCTGCAATATTGG---------------------------------------------------AGTCAGGAACTAAAGAATAGTGCTGTTAGCTTGCTTGACGCCATAGCTATAGCAGTAGCTGAGGGAACAGATAGGGTTATAAAAGTAGTACGAAGA------------------GCCTTTAGAGCTATTCTCCACATACCTGTAAGAATAAGACAGGGCTTAGAAAGGGCTTTGCTATAA

2.SC22.Trinidad.EU576876 ATGAGAGTGAAGGAGATCAAGAGGAATTGTCAGCGCTTG---------TGGAGATGGGGC------------------------ACCATGCTCCTTGGGATGTTGATGATC------------TGTAGTGCTACA---------CAACAATTGTGGGTTACAGTCTATTATGGGGTACCTGTGTGGAAAGAAGCAACCACCACTCTATTTTGTGCATCAGATGCTAAAGCATATGATACAGAGGCACATAAT---GTCTGGGCCACACATGCCTGTGTACCCACAGACCCCAACCCACAAGAAGTAGTATTG---GGAAATGTGACAGAAAATTTTAACATGTGGAAAAATAACATGGTAGAACAAATGCATGAGGATATAATCAGTTTATGGGATCAAAGCCTAAAGCCATGTGTAAAATTAACCCCACTCTGTGTTACTTTAAACTGCACTGATTATGTGAAAAATGATACTAGTAGCACT---------------------------------------------------------------------------------------------AACAGTAGCAGCTGGGAGAAAGGAGAAATAAAAAACTGCTCTTTCAATATCACC---ACAAGCATAAAAGAT------AAGATGCAGAGGGAATATGCACTTTTTTATAAACTTGATGTAATACCAATAGATAGTGAGAATAATAGTAATATAAATAGTGGGAATAATAGTAATAGTAATTTTAGT------AGCTATAGTAATTTTAGATTGATAAATTGTAATACCTCAGTCATTACACAGGCCTGTCCAAAGGTATCCTTTGAACCAATTCCCATACATTATTGTGCCCCGGCTGGGTTTGCGATTCTAAAGTGT---AATGATAAAAAGTTCAATGGATCAGGACCATGTACAAATGTCAGCACAGTACAATGTACACATGGAATTAGACCAGTAGTGTCAACTCAACTGTTGTTAAATGGCAGTCTAGCAGAAGAG---GAGGTAGTAATTAGATCTGAAAATTTCACGAACAATGTTAAAACCATAATAGTACATCTGAATAAATCTGTAGTAATTAATTGTACAAGACCCAACAACAATACAAGAAGAGGTATACATATGGGA------------CCAGGGAAA---GCATATTTT---ACAGGAGAAATAATAGGAGATATAAGACAAGCACATTGTAACATT------AGTAGAAAAGAATGGAATGACACTTTAAAACAGATAGTTATAAAATTAGGA---GAACAATTTAGA------------AATAAAACA---ATAGCCTTTAATCAA---------TCCTCAGGAGGGGACATTGAAATTGTAACACACAGTTTTAATTGTGGAGGGGAATTTTTCTACTGTAATTCAACACAACTGTTTAATAGTACTTGGAATAAT---------AATGGTACTAGG------------------------------AATGATACTAGGGAGTCA------------AATGACACT---------------------------ATCATCACACTACCATGCAGAATCAAACAAATTATAAACATGTGGCAGCAAGTAGGCAAAGCAATGTATGCCCCTCCCATCAGAGGACAAATTAGATGTTCATCAAATATCACAGGGCTGCTGTTAACAAGAGATGGTGGT------------------AACGAGAGCGAC---------------------------GCTGAGACCTTCAGACCTGGAGGAGGAGATATGAGGGACAATTGGAGA---AGTGAATTATACAAATATAAAGTAGTAAAAATTGAACCA---TTAGGAGTAGCACCC---TCCAAGGCAAAGAGAAGAGTGGTGCAGAGAGAA---AAAAGAGCAGTG---GGAACAATAGGA---GCTATG---TTCCTT---GGG---------TTCTTGGGA---GCAGCAGGAAGCACTATGGGCGCAGCGTCACTG---ACGCTGACGGTACAGGCCAGACTATTATTGTCTGGTATAGTGCAACAGCAGAACAATCTGCTGAGGGCTATTGAGGCGCAACAACATATTTTGCAACTCACGGTCTGGGGCATTAAACAGCTCCAGGCAAGA---GTCCTGGCTGTGGAAAGATACCTAAGGGATCAACAGCTCCTAGGAATTTGGGGTTGCTCTGGAAAACTCATCTGCACCACTGCTGTGCCTTGGAACGCTAGTTGGAGT---------------------------AATAAATCTCAAGAGGAGATTTGGGAG---AACATGACCTGGATGCAGTGGGATAGAGAAATTAAC------AATTACACAAACCTCATATATACCTTACTTGAAGAATCGCAGAACCAGCAAGAAAAGAATGAACAAGACTTATTAGCATTGGATGAATGGGCAAATTTGTGGAATTGGTTTGCCATATCAAAGTGGCTGTGGTATATAAAAATATTCATAATGATAGTAGGCGGCTTGATAGGTTTAAGAATAGTTTTTGCTGTACTTTCTATAGTGAATAGAGTTAGGCAGGGATACTCACCATTATCGTTTCAGACCCAC---CTCCCAGCCAGGAGGGGA------CCCGACAGGCCCGAAGGAATCGAAGAAGAAGGTGGAGAGAGAGACAGAGACAGATCAGTTCGCTTAGTACATGGATTCTTAGCACTCATCTGGGACGACCTACGGAGCCTGACCCTCTTCAGCTACCACCGCTTGAGAGACTTACTCTTGATTATAGCGAGGATTGTGGAAACTCTGGGACGCAGG---------------GGGTGGGAAGCCCTCAAGTATTGGTGG---AATCTCCTGCAATATTGG---------------------------------------------------AGTCAGGAACTAAAGAATAGTGCTGTTAGCTTGCTTGACGCCATAGCTATAGCAGTAGCTGAGGGAACAGATAGGGTTATAAAAGTAGTACGAAGA------------------GCCTTTAGAGCTATTCTCCACATACCTGTAAGAATAAGACAGGGCTTAGAAAGGGCTTTGCTATAA

2.SC22.Trinidad.EU576852 ATGAGAGTGAAGGAGATCAAGAGGAATTGTCAGCGCTTG---------TGGAGATGGGGC------------------------ACCATGCTCCTTGGGATGTTGATGATC------------TGTAGTGCTACA---------CAACAATTGTGGGTTACAGTCTATTATGGGGTACCTGTGTGGAAAGAAGCAACCACCACTCTATTTTGTGCATCAGATGCTAAAGCATATGATACAGAGGCACATAAT---GTCTGGGCCACGCATGCCTGTGTACCCACAGACCCCAACCCACAAGAAGTAGTATTG---GGAAATGTGACAGAAAATTTTAACATGTGGAAAAATAACATGGTAGAACAAATGCATGAGGATATAATCAGTTTATGGGATCAAAGCCTAAAGCCATGTGTAAAATTAACCCCACTCTGTGTTACTTTAAACTGCACTGATTATGTGAAAAATGATACTAGTAGCACT---------------------------------------------------------------------------------------------AACAGTAGCAGCTGGGAGAAAGGAGAAATAAAAAACTGCTCTTTCAATATCACC---ACAAGCATAAAAGAT------AAGATGCAGAGGGAATATGCACTTTTTTATAAACTTGATGTAATACCAATAGATAGTGAGAATAATAGTAATATAAATAGTGGGAATAATAGTAATAGTAATTTTAGT------AGCTATAGTAATTTTAGATTGATAAATTGTAATACCTCAGTCATTACACAGGCCTGTCCAAAGGTATCCTTTGAACCAATTCCCATACATTATTGTGCCCCGGCTGGGTTTGCGATTCTAAAGTGT---AATGATAAAAAGTTCAATGGATCAGGACCATGTACAAATGTCAGCACAGTACAATGTACACATGGAATTAGACCAGTAGTGTCAACTCAACTGTTGTTAAATGGCAGTCTAGCAGAAGAG---GAGGTAGTAATTAGATCTGAAAATTTCACGAACAATGTTAAAACCATAATAGTACATCTGAATAAATCTGTAGTAATTAATTGTACAAGACCCAACAACAATACAAGAAGAGGTATACATATGGGA------------CCAGGGAAA---GCATATTTT---ACAGGAGAAATAATAGGAGATATAAGACAAGCACATTGTAACATT------AGTAGAAAAGAATGGAATGACACTTTAAAACAGATAGTTATAAAATTAGGA---GAACAATTTAGA------------AATAAAACA---ATAGCCTTTAATCAA---------TCCTCAGGAGGGGACATTGAAATTGTAACACACAGTTTTAATTGTGGAGGGGAATTTTTCTACTGTAATTCAACACAACTGTTTAATAGTACTTGGAATAAT---------AATGGTACTAGG------------------------------AATGATACTAGGGAGTCA------------AATGACACT---------------------------ATCATCACACTACCATGCAGAATCAAACAAATTATAAACATGTGGCAGCAAGTAGGCAAAGCAATGTATGCCCCTCCCATCAGAGGACAAATTAGATGTTCATCAAATATCACAGGGCTGCTGTTAACAAGAGATGGTGGT------------------AACGAGAGCGAC---------------------------GCTGAGACCTTCAGACCTGGAGGAGGAGATATGAGGGACAATTGGAGA---AGTGAATTATACAAATATAAAGTAGTAAAAATTGAACCA---TTAGGAGTAGCACCC---TCCAAGGCAAAGAGAAGAGTGGTGCAGAGAGAA---AAAAGAGCAGTG---GGAACAATAGGA---GCTATG---TTCCTT---GGG---------TTCTTGGGA---GCAGCAGGAAGCACTATGGGCGCAGCGTCACTG---ACGCTGACGGTACAGGCCAGACTATTATTGTCTGGTATAGTGCAACAGCAGAACAATCTGCTGAGGGCTATTGAGGCGCAACAACATATTTTGCAACTCACGGTCTGGGGCATTAAACAGCTCCAGGCAAGA---GTCCTGGCTGTGGAAAGATACCTAAGGGATCAACAGCTCCTAGGGATTTGGGGTTGCTCTGGAAAACTCATCTGCACCACTGCTGTGCCTTGGAACGCTAGTTGGAGT---------------------------AATAAATCTCAAGAGGAGATTTGGGAG---AACATGACCTGGATGCAGTGGGATAGAGAAATTAAC------AATTACACAAACCTCATATATACCTTACTTGAAGAATCGCAGAACCAGCAAGAAAAGAATGAACAAGACTTATTAGCATTGGATGAATGGGCAAATTTGTGGAATTGGTTTGCCATATCAAAGTGGCTGTGGTATATAAAAATATTCATAATGATAGTAGGCGGCTTGATAGGTTTAAGAATAGTTTTTGCTGTACTTTCTATAGTGAATAGAGTTAGGCAGGGATACTCACCATTATCGTTTCAGACCCAC---CTCCCAGCCAGGAGGGGA------CCCGACAGGCCCGAAGGAATCGAAGAAGAAGGTGGAGAGAGAGACAGAGACAGATCAGTTCGCTTAGTACATGGATTCTTAGCACTCATCTGGGACGACCTACGGAGCCTGACCCTCTTCAGCTACCACCGCTTGAGAGACTTACTCTTGATTATAGCGAGGATTGTGGAAACTCTGGGACGCAGG---------------GGGTGGGAAGCCCTCAAGTATTGGTGG---AATCTCCTGCAATATTGG---------------------------------------------------AGTCAGGAACTAAAGAATAGTGCTGTTAGCTTGCTTGACGCCATAGCTATAGCAGTAGCTGAGGGAACAGATAGGGTTATAAAAGTAGTACGAAGA------------------GCCTTTAGAGCTATTCTCCACATACCTGTAAGAATAAGACAGGGCTTAGAAAGGGCTTTGCTATAA

2.SC22.Trinidad.EU576854 ATGAGAGTGAAGGAGATCAAGAGGAATTGTCAGCGCTTG---------TGGAGATGGGGC------------------------ACCATGCTCCTTGGGATGTTGATGATC------------TGTAGTGCTACA---------CAACAATTGTGGGTTACAGTCTATTATGGGGTACCTGTGTGGAAAGAAGCAACCACCACTCTATTTTGTGCATCAGATGCTAAAGCATATGATACAGAGGCACATAAT---GTCTGGGCCACACATGCCTGTGTACCCACAGACCCCAACCCACAAGAAGTAGTATTG---GGAAATGTGACAGAAAATTTTAACATGTGGAAAAATAACATGGTAGACCAAATGCATGAGGATATAATCAGTTTATGGGATCAAAGCCTAAAGCCATGTGTAAAATTAACCCCACTCTGTGTTACTTTAAACTGCACTGATTATGTGAAAAATGATACTAGTAGCACT---------------------------------------------------------------------------------------------AACAGTAGCAGCTGGGAGAAAGGAGAAATAAAAAACTGCTCTTTCAATATCACC---ACAAGCATAAAAGAT------AAGATGCAGAGGGAATATGCACTTTTTTATAAACTTGATGTAATACCAATAGATAGTGAGAATAATAGTAATATAAATAGTGGGAATAATAGTAATAGTAATTTTAGT------AGCTATAGTAATTTTAGATTGATAAATTGTAATACCTCAGTCATTACACAGGCCTGTCCAAAGGTATCCTTTGAACCAATTCCCATACATTATTGTGCCCCGGCTGGGTTTGCGATTCTAAAGTGT---AATGATAAAAAGTTCAATGGATCAGGACCATGTACAAATGTCAGCACAGTACAATGTACACATGGAATTAGACCAGTAGTGTCAACTCAACTGTTGTTAAATGGCAGTCTAGCAGAAGAG---GAGGTAGTAATTAGATCTGAAAATTTCACGAACAATGTTAAAACCATAATAGTACATCTGAATAAATCTGTAGTAATTAATTGTACAAGACCCAACAACAATACAAGAAGAGGTATACATATGGGA------------CCAGGGAAA---GCATATTTT---ACAGGAGAAATAATAGGAGATATAAGACAAGCACATTGTAACATT------AGTAGAAAAGAATGGAATGACACTTTAAAACAGATAGTTATAAAATTAGGA---GAACAATTTAGA------------AATAAAACA---ATAGCCTTTAATCAA---------TCCTCAGGAGGGGACATTGAAATTGTAACACACAGTTTTAATTGTGGAGGGGAATTTTTCTACTGTAATTCAACACAACTGTTTAATAGTACTTGGAATAAT---------AATGGTACTAGG------------------------------AATGATACTAGGGAGTCA------------AATGACACT---------------------------ATCATCACACTACCATGCAGAATCAAACAAATTATAAACATGTGGCAGCAAGTAGGCAAAGCAATGTATGCCCCTCCCATCAGAGGACAAATTAGATGTTCATCAAATATCACAGGGCTGCTGTTAACAAGAGATGGTGGT------------------AACGAGAGCGAC---------------------------GCTGAGACCTTCAGACCTGGAGGAGGAGATATGAGGGACAATTGGAGA---AGTGAATTATACAAATATAAAGTAGTAAAAATTGAACCA---TTAGGAGTAGCACCC---TCCAAGGCAAAGAGAAGAGTGGTGCAGAGAGAA---AAAAGAGCAGTG---GGAACAATAGGA---GCTATG---TTCCTT---GGG---------TTCTTGGGA---GCAGCAGGAAGCACTATGGGCGCAGCGTCACTG---ACGCTGACGGTACAGGCCAGACTATTATTGTCTGGTATAGTGCAACAGCAGAACAATCTGCTGAGGGCTATTGAGGCGCAACAACATATTTTGCAACTCACGGTCTGGGGCATTAAACAGCTCCAGGCAAGA---GTCCTGGCTGTGGAAAGATACCTAAGGGATCAACAGCTCCTAGGGATTTGGGGTTGCTCTGGAAAACTCATCTGCACCACTGCTGTGCCTTGGAACGCTAGTTGGAGT---------------------------AATAAATCTCAAGAGGAGATTTGGGAG---AACATGACCTGGATGCAGTGGGATAGAGAAATTAAC------AATTACACAAACCTCATATATACCTTACTTGAAGAATCGCAGAACCAGCAAGAAAAGAATGAACAAGACTTATTAGCATTGGATGAATGGGCAAATTTGTGGAATTGGTTTGCCATATCAAAGTGGCTGTGGTATATAAAAATATTCATAATGATAGTAGGCGGCTTGATAGGTTTAAGAATAGTTTTTGCTGTACTTTCTATAGTGAATAGAGTTAGGCAGGGATACTCACCATTATCGTTTCAGACCCAC---CTCCCAGCCAGGAGGGGA------CCCGACAGGCCCGAAGGAATCGAAGAAGAAGGTGGAGAGAGAGACAGAGACAGATCAGTTCGCTTAGTACATGGATTCTTAGCACTCATCTGGGACGACCTACGGAGCCTGACCCTCTTCAGCTACCACCGCTTGAGAGACTTACTCTTGATTATAGCGAGGATTGTGGAAACTCTGGGACGCAGG---------------GGGTGGGAAGCCCTCAAGTATTGGTGG---AATCTCCTGCAATATTGG---------------------------------------------------AGTCAGGAACTAAAGAATAGTGCTGTTAGCTTGCTTGACGCCATAGCTATAGCAGTAGCTGAGGGAACAGATAGGGTTATAAAAGTAGTACGAAGA------------------GCCTTTAGAGCTATTCTCCACATACCTGTAAGAATAAGACAGGGCTTAGAAAGGGCTTTGCTATAA

2.SC22.Trinidad.EU576882 ATGAGAGTGAAGGAGATCAAGAGGAATTGTCAGCGCTTG---------TGGAGATGGGGC------------------------ACCATGCTCCTTGGGATGTTGATGATC------------TGTAGTGCTACA---------CAACAATTGTGGGTTACAGTCTATTATGGGGTACCTGTGTGGAAAGAAGCAACCACCACTCTATTTTGTGCATCAGATGCTAAAGCATATGATACAGAGGCACATAAT---GTCTGGGCCACACATGCCTGTGTACCCACAGACCCCAACCCACAAGAAGTAGTATTG---GGAAATGTGACAGAAAATTTTAACATGTGGAAAAATAACATGGTAGAACAAATGCATGAGGATATAATCAGTTTATGGGATCAAAGCCTAAAGCCATGTGTAAAATTAACCCCACTCTGTGTTACTTTAAACTGCACTGATTATGTGAAAAATGATACTAGTAGCACT---------------------------------------------------------------------------------------------AACAGTAGCAGCTGGGAGAAAGGAGAAATAAAAAACTGCTCTTTCAATATCACC---ACAAGCATAAAAGAT------AAGATGCAGAGGGAATATGCACTTTTTTATAAACTTGATGTAATACCAATAGATAGTGAGAATAATAGTAATATAAATAGTGGGAATAATAGTAATAGTAATTTTAGT------AGCTATAGTAATTTTAGATTGATAAATTGTAATACCTCAGTCATTACACAGGCCTGTCCAAAGGTATCCTTTGAACCAATTCCCATACATTATTGTGCCCCGGCTGGGTTTGCGATTCTAAAGTGT---AATGATAAAAAGTTCAATGGATCAGGACCATGTACAAATGTCAGCACAGTACAATGTACACATGGAATTAGACCAGTAGTGTCAACTCAACTGTTGTTAAATGGCAGTCTAGCAGAAGAG---GAGGTAGTAATTAGATCTGAAAATTTCACGAACAATGTTAAAACCATAATAGTACATCTGAATAAATCTGTAGTAATTAATTGTACAAGACCCAACAACAATACAAGAAGAGGTATACATATGGGA------------CCAGGGAAA---GCATATTTT---ACAGGAGAAATAATAGGAGATATAAGACAAGCACATTGTAACATT------AGTAGAAAAGAATGGAATGACACTTTAAAACAGATAGTTATAAAATTAGGA---GAACAATTTAGA------------AATAAAACA---ATAGCCTTTAATCAA---------TCCTCAGGAGGGGACATTGAAATTGTAACACACAGTTTTAATTGTGGAGGGGAATTTTTCTACTGTAATTCAACACAACTGTTTAATAGTACTTGGAATAAT---------AATGGTACTAGG------------------------------AATGATACTAGGGAGTCA------------AATGACACT---------------------------ATCATCACACTACCATGCAGAATCAAACAAATTATAAACATGTGGCAGCAAGTAGGCAAAGCAATGTATGCCCCTCCCATCAGAGGACAAATTAGATGTTCATCAAATATCACAGGGCTGCTGTTAACAAGAGATGGTGGT------------------AACGAGAGCGAC---------------------------GCTGAGACCTTCAGACCTGGAGGAGGAGATATGAGGGACAATTGGAGA---AGTGAATTATACAAATATAAAGTAGTAAAAATTGAACCA---TTAGGAGTAGCACCC---TCCAAGGCAAAGAGAAGAGTGGTGCAGAGAGAA---AAAAGAGCAGTG---GGAACAATAGGA---GCTATG---TTCCTT---GGG---------TTCTTGGGA---GCAGCAGGAAGCACTATGGGCGCAGCGTCACTG---ACGCTGACGGTACAGGCCAGACTATTATTGTCTGGTATAGTGCAACAGCAGAACAATCTGCTGAGGGCTATTGAGGCGCAACAACATATTTTGCAACTCACGGTCTGGGGCATTAAACAGCTCCAGGCAAGA---GTCCTGGCTGTGGAAAGATACCTAAGGGATCAACAGCTCCTAGGGATTTGGGGTTGCTCTGGAAAACTCATCTGCACCACTGCTGTGCCTTGGAACGCTAGTTGGAGT---------------------------AATAAATCTCAAGAGGAGATTTGGGAG---AACATGACCTGGATGCAGTGGGATAGAGAAATTAAC------AATTACACAAACCTCATATATACCTTACTTGAAGAATCGCAGAACCAGCAAGAAAAGAATGAACAAGACTTATTAGCATTGGATGAATGGGCAAATTTGTGGAATTGGTTTGCCATATCAAAGTGGCTGTGGTATATAAAAATATTCATAATGATAGTAGGCGGCTTGATAGGTTTAAGAATAGTTTTTGCTGTACTTTCTATAGTGAATAGAGTTAGGCAGGGATACTCACCATTATCGTTTCAGACCCAC---CTCCCAGCCAGGAGGGGA------CCCGACAGGCCCGAAGGAATCGAAGAAGAAGGTGGAGAGAGAGACAGAGACAGATCAGTTCGCTTAGTACATGGATTCTTAGCACTCATCTGGGACGACCTACGGAGCCTGACCCTCTTCAGCTACCACCGCTTGAGAGACTTACTCTTGATTATAGCGAGGATTGTGGAAACTCTGGGACGCAGG---------------GGGTGGGAAGCCCTCAAGTATTGGTGG---AATCTCCTGCAATATTGG---------------------------------------------------AGTCAGGAACTAAAGAATAGTGCTGTTAGCTTGCTTGACGCCATAGCTATAGCAGTAGCTGAGGGAACAGATAGGGTTATAAAAGTAGTACGAAGA------------------GCCTTTAGAGCTATTCTCCACATACCTGTAAGAATAAGACAGGGCTTAGAAAGGGCTTTGCTATAA

2.SC22.Trinidad.EU576857 ATGAGAGTGAAGGAGATCAAGAGGAATTGTCAGCGCTTG---------TGGAGATGGGGC------------------------ACCATGCTCCTTGGGATGTTGATGATC------------TGTAGTGCTACA---------CAACAATTGTGGGTTACAGTCTATTATGGGGTACCTGTGTGGAAAGAAGCAACCACCACTCTATTTTGTGCATCAGATGCTAAAGCATATGATACAGAGGCACATAAT---GTCTGGGCCACACATGCCTGTGTACCCACAGACCCCAACCCACAAGAAGTAGTATTG---GGAAATGTGACAGAAAATTTTAACATGTGGAAAAATAACATGGTAGAACAAATGCATGAGGATATAATCAGTTTATGGGATCAAAGCCTAAAGCCATGTGTAAAATTAACCCCACTCTGTGTTACTTTAAACTGCACTGATTATGTGAAAAATGATACTAGTAGCACT---------------------------------------------------------------------------------------------AACAGTAGCAGCTGGGAGAAAGGAGAAATAAAAAACTGCTCTTTCAATATCACC---ACAAGCATAAAAGAT------AAGATGCAGAGGGAATATGCACTTTTTTATAAACTTGATGTAATACCAATAGATAGTGAGAATAATAGTAATATAAATAGTGGGAATAATAGTAATAGTAATTTTAGT------AGCTATAGTAATTTTAGATTGATAAATTGTAATACCTCAGTCATTACACAGGCCTGTCCAAAGGTATCCTTTGAACCAATTCCCATACATTATTGTGCCCCGGCTGGGTTTGCGATTCTAAAGTGT---AATGATAAAAAGTTCAATGGATCAGGACCATGTACAAATGTCAGCACAGTACAATGTACACATGGAATTAGACCAGTAGTGTCAACTCAACTGTTGTTAAATGGCAGTCTAGCAGAAGAG---GAGGTAGTAATTAGATCTGAAAATTTCACGAACAATGTTAAAACCATAATAGTACATCTGAATAAATCTGTAGTAATTAATTGTACAAGACCCAACAACAATACAAGAAGAGGTATACATATGGGA------------CCAGGGAAA---GCATATTTT---ACAGGAGAAATAATAGGAGATATAAGACAAGCACATTGTAACATT------AGTAGAAAAGAATGGAATGACACTTTAAAACAGATAGTTATAAAATTAGGA---GAACAATTTAGA------------AATAAAACA---ATAGCCTTTAATCAA---------TCCTCAGGAGGGGACATTGAAATTGTAACACACAGTTTTAATTGTGGAGGGGAATTTTTCTACTGTAATTCAACACAACTGTTTAATAGTACTTGGAATAAT---------AATGGTACTAGG------------------------------AATGATACTAGGGAGTCA------------AATGACACT---------------------------ATCATCACACTACCATGCAGAATCAAACAAATTATAAACATGTGGCAGCAAGTAGGCAAAGCAATGTATGCCCCTCCCATCAGAGGACAAATTAGATGTTCATCAAATATCACAGGGCTGCTGTTAACAAGAGATGGTGGT------------------AACGAGAGCGAC---------------------------GCTGAGACCTTCAGACCTGGAGGAGGAGATATGAGGGACAATTGGAGA---AGTGAATTATACAAATATAAAGTAGTAAAAATTGAACCA---TTAGGAGTAGCACCC---TCCAAGGCAAAGAGAAGAGTGGTGCAGAGAGAA---AAAAGAGCAGTG---GGAACAATAGGA---GCTATG---TTCCTT---GGG---------TTCTTGGGA---GCAGCAGGAAGCACTATGGGCGCAGCGTCACTG---ACGCTGACGGTACAGGCCAGACTATTATTGTCTGGTATAGTGCAACAGCAGAACAATCTGCTGAGGGCTATTGAGGCGCAACAACATATTTTGCAACTCACGGTCTGGGGCATTAAACAGCTCCAGGCAAGA---GTCCTGGCTGTGGAAAGATACCTAAGGGATCAACAGCTCCTAGGGATTTGGGGTTGCTCTGGAAAACTCATCTGCACCACTGCTGTGCCTTGGAACGCTAGTTGGAGT---------------------------AATAAATCTCAAGAGGAGATTTGGGAG---AACATGACCTGGATGCAGTGGGATAGAGAAATTAAC------AATTACACAAACCTCATATATACCTTACTTGAAGAATCGCAGAACCAGCAAGAAAAGAATGAACAAGACTTATTAGCATTGGATGAATGGGCAAATTTGTGGAATTGGTTTGCCATATCAAAGTGGCTGTGGTATATAAAAATATTCATAATGATAGTAGGCGGCTTGATAGGTTTAAGAATAGTTTTTGCTGTACTTTCTATAGTGAATAGAGTTAGGCAGGGATACTCACCATTATCGTTTCAGACCCAC---CTCCCAGCCAGGAGGGGA------CCCGACAGGCCCGAAGGAATCGAAGAAGAAGGTGGAGAGAGAGACAGAGACAGATCAGTTCGCTTAGTACATGGATTCTTAGCACTCATCTGGGACGACCTACGGAGCCTGACCCTCTTCAGCTACCACCGCTTGAGAGACTTACTCTTGATTATAGCGAGGATTGTGGAAACTCTGGGACGCAGG---------------GGGTGGGAAGCCCTCAAGTATTGGTGG---AATCTCCTGCAATATTGG---------------------------------------------------AGTCAGGAACTAAAGAATAGTGCTGTTAGCTTGCTTGACGCCATAGCTATAGCAGTAGCTGAGGGAACAGATAGGGTTATAAAAGTAGTACGAAGA------------------GCCTTTAGAGCTATTCTCCACATACCTGTAAGAATAAGACAGGGCTTAGAAAGGGCTTTGCTATAA

2.SC22.Trinidad.EU576879 ATGAGAGTGAAGGAGATCAAGAGGAATTGTCAGCGCTTG---------TGGAGATGGGGC------------------------ACCATGCTCCTTGGGATGTTGATGATC------------TGTAGTGCTACA---------CAACAATTGTGGGTTACAGTCTATTATGGGGTACCTGTGTGGAAAGAAGCAACCACCACTCTATTTTGTGCATCAGATGCTAAAGCATATGATACAGAGGCACATAAT---GTCTGGGCCACACATGCCTGTGTACCCACAGACCCCAACCCACAAGAAGTAGTATTG---GGAAATGTGACAGAAAATTTTAACATGTGGAAAAATAACATGGTAGAACAAATGCATGAGGATATAATCAGTTTATGGGATCAAAGCCTAAAGCCATGTGTAAAATTAACCCCACTCTGTGTTACTTTAAACTGCACTGATTATGTGAAAAATGATACTAGTAGCACT---------------------------------------------------------------------------------------------AACAGTAGCAGCTGGGAGAAAGGAGAAATAAAAAACTGCTCTTTCAATATCACC---ACAAGCATAAAAGAT------AAGATGCAGAGGGAATATGCACTTTTTTATAAACTTGATGTAATACCAATAGATAGTGAGAATAATAGTAATATAAATAGTGGGAATAATAGTAATAGTAATTTTAGT------AGCTATAGTAATTTTAGATTGATAAATTGTAATACCTCAGTCATTACACAGGCCTGTCCAAAGGTATCCTTTGAACCAATTCCCATACATTATTGTGCCCCGGCTGGGTTTGCGATTCTAAAGTGT---AATGATAAAAAGTTCAATGGATCAGGACCATGTACAAATGTCAGCACAGTACAATGTACACATGGAATTAGACCAGTAGTGTCAACTCAACTGTTGTTAAATGGCAGTCTAGCAGAAGAG---GAGGTAGTAATTAGATCTGAAAATTTCACGAACAATGTTAAAACCATAATAGTACATCTGAATAAATCTGTAGTAATTAATTGTACAAGACCCAACAACAATACAAGAAGAGGTATACATATGGGA------------CCAGGGAAA---GCATATTTT---ACAGGAGAAATAATAGGAGATATAAGACAAGCACATTGTAACATT------AGTAGAAAAGAATGGAATGACACTTTAAAACAGATAGTTATAAAATTAGGA---GAACAATTTAGA------------AATAAAACA---ATAGCCTTTAATCAA---------TCCTCAGGAGGGGACATTGAAATTGTAACACACAGTTTTAATTGTGGAGGGGAATTTTTCTACTGTAATTCAACACAACTGTTTAATAGTACTTGGAATAAT---------AATGGTACTAGG------------------------------AATGATACTAGGGAGTCA------------AATGACACT---------------------------ATCATCACACTACCATGCAGAATCAAACAAATTATAAACATGTGGCAGCAAGTAGGCAAAGCAATGTATGCCCCTCCCATCAGAGGACAAATTAGATGTTCATCAAATATCACAGGGCTGCTGTTAACAAGAGATGGTGGT------------------AACGAGAGCGAC---------------------------GCTGAGACCTTCAGACCTGGAGGAGGAGATATGAGGGACAATTGGAGA---AGTGAATTATACAAATATAAAGTAGTAAAAATTGAACCA---TTAGGAGTAGCACCC---TCCAAGGCAAAGAGAAGAGTGGTGCAGAGAGAA---AAAAGAGCAGTG---GGAACAATAGGA---GCTATG---TTCCTT---GGG---------TTCTTGGGA---GCAGCAGGAAGCACTATGGGCGCAGCGTCACTG---ACGCTGACGGTACAGGCCAGACTATTATTGTCTGGTATAGTGCAACAGCAGAACAATCTGCTGAGGGCTATTGAGGCGCAACAACATATTTTGCAACTCACGGTCTGGGGCATTAAACAGCTCCAGGCAAGA---GTCCTGGCTGTGGAAAGATACCTAAGGGATCAACAGCTCCTAGGGATTTGGGGTTGCTCTGGAAAACTCATCTGCACCACTGCTGTGCCTTGGAACGCTAGTTGGAGT---------------------------AATAAATCTCAAGAGGAGATTTGGGAG---AACATGACCTGGATGCAGTGGGATAGAGAAATTAAC------AATTACACAAACCTCATATATACCTTACTTGAAGAATCGCAGAACCAGCAAGAAAAGAATGAACAAGACTTATTAGCATTGGATGAATGGGCAAATTTGTGGAATTGGTTTGCCATATCAAAGTGGCTGTGGTATATAAAAATATTCATAATGATAGTAGGCGGCTTGATAGGTTTAAGAATAGTTTTTGCTGTACTTTCTATAGTGAATAGAGTTAGGCAGGGATACTCACCATTATCGTTTCAGACCCAC---CTCCCAGCCAGGAGGGGA------CCCGACAGGCCCGAAGGAATCGAAGAAGAAGGTGGAGAGAGAGACAGAGACAGATCAGTTCGCTTAGTACATGGATTCTTAGCACTCATCTGGGACGACCTACGGAGCCTGACCCTCTTCAGCTACCACCGCTTGAGAGACTTACTCTTGATTATAGCGAGGATTGTGGAAACTCTGGGACGCAGG---------------GGGTGGGAAGCCCTCAAGTATTGGTGG---AATCTCCTGCAATATTGG---------------------------------------------------AGTCAGGAACTAAAGAATAGTGCTGTTAGCTTGCTTGACGCCATAGCTATAGCAGTAGCTGAGGGAACAGATAGGGTTATAAAAGTAGTACGAAGA------------------GCCTTTAGAGCTATTCTCCACATACCTGTAAGAATAAGACAGGGCTTAGAAAGGGCTTTGCTATAA

2.SC22.Trinidad.EU576874 ATGAGAGTGAAGGAGATCAAGAGGAATTGTCAGCGCTTG---------TGGAGATGGGGC------------------------ACCATGCTCCTTGGGATGTTGATGATC------------TGTAGTGCTACA---------CAACAATTGTGGGTTACAGTCTATTATGGGGTACCTGTGTGGAAAGAAGCAACCACCACTCTATTTTGTGCATCAGATGCTAAAGCATATGATACAGAGGCACATAAT---GTCTGGGCCACACATGCCTGTGTACCCACAGACCCCAACCCACAAGAAGTAGTATTG---GGAAATGTGACAGAAAATTTTAACATGTGGAAAAATAACATGGTAGAACAAATGCATGAGGATATAATCAGTTTATGGGATCAAAGCCTAAAGCCATGTGTAAAATTAACCCCACTCTGTGTTACTTTAAACTGCACTGATTATGTGAAAAATGATACTAGTAGCACT---------------------------------------------------------------------------------------------AACAGTAGCAGCTGGGAGAAAGGAGAAATAAAAAATTGCTCTTTCAATATCACC---ACAAGCATAAAAGAT------AAGATGCAGAGGGAATATGCACTTTTTTATAAACTTGATGTAATACCAATAGATAGTGAGAATAATAGTAATATAAATAGTGGGAATAATAGTAATAGTAATTTTAGT------AGCTATAGTAATTTTAGATTGATAAATTGTAATACCTCAGTCATTACACAGGCCTGTCCAAAGGTATCCTTTGAACCAATTCCCATACATTATTGTGCCCCGGCTGGGTTTGCGATTCTAAAGTGT---AATGATAAAAAGTTCAATGGATCAGGACCATGTACAAATGTCAGCACAGTACAATGTACACATGGAATTAGACCAGTAGTGTCAACTCAACTGTTGTTAAATGGCAGTCTAGCAGAAGAG---GAGGTAGTAATTAGATCTGAAAATTTCACGAACAATGTTAAAACCATAATAGTACATCTGAATAAATCTGTAGTAATTAATTGTACAAGACCCAACAACAATACAAGAAGAGGTATACATATGGGA------------CCAGGGAAA---GCATATTTT---ACAGGAGAAATAATAGGAGATATAAGACAAGCACATTGTAACATT------AGTAGAAAAGAATGGAATGACACTTTAAAACAGATAGTTATAAAATTAGGA---GAACAATTTAGA------------AATAAAACA---ATAGCCTTTAATCAA---------TCCTCAGGAGGGGACATTGAAATTGTAACACACAGTTTTAATTGTGGAGGGGAATTTTTCTACTGTAATTCAACACAACTGTTTAATAGTACTTGGAATAAT---------AATGGTACTAGG------------------------------AATGATACTAGGGAGTCA------------AATGACACT---------------------------ATCATCACACTACCATGCAGAATCAAACAAATTATAAACATGTGGCAGCAAGTAGGCAAAGCAATGTATGCCCCTCCCATCAGAGGACAAATTAGATGTTCATCAAATATCACAGGGCTGCTGTTAACAAGAGATGGTGGT------------------AACGAGAGCGAC---------------------------GCTGAGACCTTCAGACCTGGAGGAGGAGATATGAGGGACAATTGGAGA---AGTGAATTATACAAATATAAAGTAGTAAAAATTGAACCA---TTAGGAGTAGCACCC---TCCAAGGCAAAGAGAAGAGTGGTGCAGAGAGAA---AAAAGAGCAGTG---GGAACAATAGGA---GCTATG---TTCCTT---GGG---------TTCTTGGGA---GCAGCAGGAAGCACTATGGGCGCAGCGTCACTG---ACGCTGACGGTACAGGCCAGACTATTATTGTCTGGTATAGTGCAACAGCAGAACAATCTGCTGAGGGCTATTGAGGCGCAACAACATATTTTGCAACTCACGGTCTGGGGCATTAAACAGCTCCAGGCAAGA---GTCCTGGCTGTGGAAAGATACCTAAGGGATCAACAGCTCCTAGGGATTTGGGGTTGCTCTGGAAAACTCATCTGCACCACTGCTGTGCCTTGGAACGCTAGTTGGAGT---------------------------AATAAATCTCAAGAGGAGATTTGGGAG---AACATGACCTGGATGCAGTGGGATAGAGAAATTAAC------AATTACACAAACCTCATATATACCTTACTTGAAGAATCGCAGAACCAGCAAGAAAAGAATGAACAAGACTTATTAGCATTGGATGAATGGGCAAATTTGTGGAATTGGTTTGCCATATCAAAGTGGCTGTGGTATATAAAAATATTCATAATGATAGTAGGCGGCTTGATAGGTTTAAGAATAGTTTTTGCTGTACTTTCTATAGTGAATAGAGTTAGGCAGGGATACTCACCATTATCGTTTCAGACCCAC---CTCCCAGCCAGGAGGGGA------CCCGACAGGCCCGAAGGAATCGAAGAAGAAGGTGGAGAGAGAGACAGAGACAGATCGGTTCGATTAGTACATGGATTCTTAGCACTCATCTGGGACGACCTACGGAGCCTGACCCTCTTCAGCTACCACCGCTTGAGAGACTTACTCTTGATTATAGCGAGGATTGTGGAAACTCTGGGACGCAGG---------------GGGTGGGAAGCCCTCAAGTATTGGTGG---AATCTCCTGCAATATTGG---------------------------------------------------AGTCAGGAACTAAAGAATAGTGCTGTTAGCTTGCTTGACGCCATAGCTATAGCAGTAGCTGAGGGAACAGATAGGGTTATAAAAGTAGTACGAAGA------------------GCCTTTAGAGCTATTCTCCACATACCTGTAAGAATAAGACAGGGCTTAGAAAGGGCTTTGCTATAA

2.SC22.Trinidad.EU576853 ATGAGAGTGAAGGAGATCAAGAGGAATTGTCAGCGCTTG---------TGGAGATGGGGC------------------------ACCATGCTCCTTGGGATGTTGATGATC------------TGTAGTGCTACA---------CAACAATTGTGGGTTACAGTCTATTATGGGGTACCTGTGTGGAAAGAAGCAACCACCACTCTATTTTGTGCATCAGATGCTAAAGCATATGATACAGAGGCACATAAT---GTCTGGGCCACACATGCCTGTGTACCCACAGACCCCAACCCACAAGAAGTAGTATTG---GGAAATGTGACAGAAAATTTTAACATGTGGAAAAATAACATGGTAGAACAAATGCATGAGGATATAATCAGTTTATGGGATCAAAGCCTAAAGCCATGTGTAAAATTAACCCCACTCTGTGTTACTTTAAACTGCACTGATTATGTGAAAATTGATACTAGTAGCACT---------------------------------------------------------------------------------------------AACAGTAGCAGCTGGGAGAAAGGAGAAATAAAAAACTGCTCTTTCAATATCACC---ACAAGCATAAAAGAT------AAGATGCAGAGGGAATATGCACTTTTTTATAAACTTGATGTAATACCAATAGATAGTGAGAATAATAGTAATATAAATAGTGGGAATAATAGTAATAGTAATTTTAGT------AGCTATAGTAATTTTAGATTGATAAATTGTAATACCTCAGTCATTACACAGGCCTGTCCAAAGGTATCCTTTGAACCAATTCCCATACATTATTGTGCCCCGGCTGGGTTTGCGATTCTAAAGTGT---AATGATAAAAAGTTCAATGGATCAGGACCATGTACAAATGTCAGCACAGTACAATGTACACATGGAATTAGACCAGTAGTGTCAACTCAACTGTTGTTAAATGGCAGTCTAGCAGAAGAG---GAGGTAGTAATTAGATCTGAAAATTTCACGAACAATGTTAAAACCATAATAGTACATCTGAATAAATCTGTAGTAATTAATTGTACAAGACCCAACAACAATACAAGAAGAGGTATACATATGGGA------------CCAGGGAAA---GCATATTTT---ACAGGAGAAATAATAGGAGATATAAGACAAGCACATTGTAACATT------AGTAGAAAAGAATGGAATGACACTTTAAAACAGATAGTTATAAAATTAGGA---GAACAATTTAGA------------AATAAAACA---ATAGCCTTTAATCAA---------TCCTCAGGAGGGGACATTGAAATTGTAACACACAGTTTTAATTGTGGAGGGGAATTTTTCTACTGTAATTCAACACAACTGTTTAATAGTACTTGGAATAAT---------AATGGTACTAGG------------------------------AATGATACTAGGGAGTCA------------AATGACACT---------------------------ATCATCACACTACCATGCAGAATCAAACAAATTATAAACATGTGGCAGCAAGTAGGCAAAGCAATGTATGCCCCTCCCATCAGAGGACAAATTAGATGTTCATCAAATATCACAGGGCTGCTGTTAACAAGAGATGGTGGT------------------AACGAGAGCGAC---------------------------GCTGAGACCTTCAGACCTGGAGGAGGAGATATGAGGGACAATTGGAGA---AGTGAATTATACAAATATAAAGTAGTAAAAATTGAACCA---TTAGGAGTAGCACCC---TCCAAGGCAAAGAGAAGAGTGGTGCAGAGAGAA---AAAAGAGCAGTG---GGAACAATAGGA---GCTATG---TTCCTT---GGG---------TTCTTGGGA---GCAGCAGGAAGCACTATGGGCGCAGCGTCACTG---ACGCTGACGGTACAGGCCAGACTATTATTGTCTGGTATAGTGCAACAGCAGAACAATCTGCTGAGGGCTATTGAGGCGCAACAACATATTTTGCAACTCACGGTCTGGGGCATTAAACAGCTCCAGGCAAGA---GTCCTGGCTGTGGAAAGATACCTAAGGGATCAACAGCTCCTAGGGATTTGGGGTTGCTCTGGAAAACTCATCTGCACCACTGCTGTGCCTTGGAACGCTAGTTGGAGT---------------------------AATAAATCTCAAGAGGAGATTTGGGAG---AACATGACCTGGATGCAGTGGGATAGAGAAATTAAC------AATTACACAAACCTCATATATACCTTACTTGAAGAATCGCAGAACCAGCAAGAAAAGAATGAACAAGACTTATTAGCATTGGATGAATGGGCAAATTTGTGGAATTGGTTTGCCATATCAAAGTGGCTGTGGTATATAAAAATATTCATAATGATAGTAGGCGGCTTGATAGGTTTAAGAATAGTTTTTGCTGTACTTTCTATAGTGAATAGAGTTAGGCAGGGATACTCACCATTATCGTTTCAGACCCAC---CTCCCAGCCAGGAGGGGA------CCCGACAGGCCCGAAGGAATCGAAGAAGAAGGTGGAGAGAGAGACAGAGACAGATCGGTTCGATTAGTACATGGATTCTTAGCACTCATCTGGGACGACCTACGGAGCCTGACCCTCTTCAGCTACCACCGCTTGAGAGACTTACTCTTGATTATAGCGAGGATTGTGGAAACTCTGGGACGCAGG---------------GGGTGGGAAGCCCTCAAGTATTGGTGG---AATCTCCTGCAATATTGG---------------------------------------------------AGTCAGGAACTAAAGAATAGTGCTGTTAGCTTGCTTGACGCCATAGCTATAGCAGTAGCTGAGGGAACAGATAGGGTTATAAAAGTAGTACGAAGA------------------GCCTTTAGAGCTATTCTCCACATACCTGTAAGAATAAGACAGGGCTTAGAAAGGGCTTTGCTATAA

2.SC22.Trinidad.EU576851 ATGAGAGTGAAGGAGATCAAGAGGAATTGTCAGCGCTTG---------TGGAGATGGGGC------------------------ACCATGCTCCTTGGGATGTTGATGATC------------TGTAGTGCTACA---------CAACAATTGTGGGTTACAGTCTATTATGGGGTACCTGTGTGGAAAGAAGCAACCACCACTCTATTTTGTGCATCAGATGCTAAAGCATATGATACAGAGGCACATAAT---GTCTGGGCCACACATGCCTGTGTACCCACAGACCCCAACCCACAAGAAGTAGTATTG---GGAAATGTGACAGAAAATTTTAACATGTGGAAAAATAACATGGTAGAACAAATGCATGAGGATATAATCAGTTTATGGGATCAAAGCCTAAAGCCATGTGTAAAATTAACCCCACTCTGTGTTACTTTAAACTGCACTGATTATGTGAAAAATGATACTAGTAGCACT---------------------------------------------------------------------------------------------AACAGTAGCAGCTGGGAGAAAGGAGAAATAAAAAACTGCTCTTTCAATATCACC---ACAAGCATAAAAGAT------AAGATGCAGAGGGAATATGCACTTTTTTATAAACTTGATGTAATACCAATAGATAGTGAGAATAATAGTAATATAAATAGTGGGAATAATAGTAATAGTAATTTTAGT------AGCTATAGTAATTTTAGATTGATAAATTGTAATACCTCAGTCATTACACAGGCCTGTCCAAAGGTATCCTTTGAACCAATTCCCATACATTATTGTGCCCCGGCTGGGTTTGCGATTCTAAAGTGT---AATGATAAAAAGTTCAATGGATCAGGACCATGTACAAATGTCAGCACAGTACAATGTACACATGGAATTAGACCAGTAGTGTCAACTCAACTGTTGTTAAATGGCAGTCTAGCAGAAGAG---GAGGTAGTAATTAGATCTGAAAATTTCACGAACAATGTTAAAACCATAATAGTACATCTGAATAAATCTGTAGTAATTAATTGTACAAGACCCAACAACAATACAAGAAGAGGTATACATATGGGA------------CCAGGGAAA---GCATATTTT---ACAGGAGAAATAATAGGAGATATAAGACAAGCACATTGTAACATT------AGTAGAAAAGAATGGAATGACACTTTAAAACAGATAGTTATAAAATTAGGA---GAACAATTTAGA------------AATAAAACA---ATAGCCTTTAATCAA---------TCCTCAGGAGGGGACATTGAAATTGTAACACACAGTTTTAATTGTGGAGGGGAATTTTTCTACTGTAATTCAACACAACTGTTTAATAGTACTTGGAATAAT---------AATGGTACTAGG------------------------------AATGATACTAGGGAGTCA------------AATGACACT---------------------------ATCATCACACTACCATGCAGAATCAAACAAATTATAAACATGTGGCAGCAAGTAGGCAAAGCAATGTATGCCCCTCCCATCAGAGGACAAATTAGATGTTCATCAAATATCACAGGGCTGCTGTTAACAAGAGATGGTGGT------------------AACGAGAGCGAC---------------------------GCTGAGACCTTCAGACCTGGAGGAGGAGATATGAGGGACAATTGGAGA---AGTGAATTATACAAATATAAAGTAGTAAAAATTGAACCA---TTAGGAGTAGCACCC---TCCAAGGCAAAGAGAAGAGTGGTGCAGAGAGAA---AAAAGAGCAGTG---GGAACAATAGGA---GCTATG---TTCCTT---GGG---------TTCTTGGGA---GCAGCAGGAAGCACTATGGGCGCAGCGTCACTG---ACGCTGACGGTACAGGCCAGACTATTATTGTCTGGTATAGTGCAACAGCAGAACAATCTGCTGAGGGCTATTGAGGCGCAACAACATATTTTGCAACTCACGGTCTGGGGCATTAAACAGCTCCAGGCAAGA---GTCCTGGCTGTGGAAAGATACCTAAGGGATCAACAGCTCCTAGGGATTTGGGGTTGCTCTGGAAAACTCATCTGCACCACTGCTGTGCCTTGGAACGCTAGTTGGAGT---------------------------AATAAATCTCAAGAGGAGATTTGGGAG---AACATGACCTGGATGCAGTGGGATAGAGAAATTAAC------AATTACACAAACCTCATATATACCTTACTTGAAGAATCGCAGAACCAGCAAGAAAAGAATGAACAAGACTTATTAGCATTGGATGAATGGGCAAATTTGTGGAATTGGTTTGCCATATCAAAGTGGCTGTGGTATATAAAAATATTCATAATGATAGTAGGCGGCTTGATAGGTTTAAGAATAGTTTTTGCTGTACTTTCTATAGTGAATAGAGTTAGGCAGGGATACTCACCATTATCGTTTCAGACCCAC---CTCCCAGCCAGGAGGGGA------CCCGACAGGCCCGAAGGAATCGAAGAAGAAGGTGGAGAGAGAGACAGAGACAGATCAGTTCGCTTAGTACATGGATTCTTAGCACTCATCTGGGACGACCTACGGAGCCTGACCCTCTTCAGCTACCACCGCTTGAGAGACTTACTCTTGATTATAGCGAGGATTGTGGAAACTCTGGGACGCAGG---------------GGGTGGGAAGCCCTCAAGTATTGGTGG---AATCTCCTGCAATATTGG---------------------------------------------------AGTCAGGAACTAAAGAATAGTGCTGTTAGCTTGCTTGACGCCATAGCTATAGCAGTAGCTGAGGGAACAGATAGGGTTATAAAAGTAGTACGAAGA------------------GCCTTTAGAGCTATTCTCCACATACCTGTAAGAATAAGACAGGGCTTAGAAAGGGCTTTGCTATAA

2.SC22.Trinidad.EU576863 ATGAGAGTGAAGGAGATCAAGAGGAATTGTCAGCGCTTG---------TGGAGATGGGGC------------------------ACCATGCTCCTTGGGATGTTGATGATC------------TGTAGTGCTACA---------CAACAATTGTGGGTTACAGTCTATTATGGGGTACCTGTGTGGAAAGAAGCAACCACCACTCTATTTTGTGCATCAGATGCTAAAGCATATGATACAGAGGCACATAAT---GTCTGGGCCACACATGCCTGTGTACCCACAGACCCCAACCCACAAGAAGTAGTATTG---GGAAATGTGACAGAAAATTTTAACATGTGGAAAAATAACATGGTAGAACAAATGCATGAGGATATAATCAGTTTATGGGATCAAAGCCTAAAGCCATGTGTAAAATTAACCCCACTCTGTGTTACTTTAAACTGCACTGATTATGTGAAAAATGATACTAGTAGCACT---------------------------------------------------------------------------------------------AACAGTAGCAGCTGGGAGAAAGGAGAAATAAAAAACTGCTCTTTCAATATCACC---ACAAGCATAAAAGAT------AAGATGCAGAGGGAATATGCACTTTTTTATAAACTTGATGTAATACCAATAGATAGTGAGAATAATAGTAATATAAATAGTGGGAATAATAGTAATAGTAATTTTAGT------AGCTATAGTAATTTTAGATTGATAAATTGTAATACCTCAGTCATTACACAGGCCTGTCCAAAGGTATCCTTTGAACCAATTCCCATACATTATTGTGCCCCGGCTGGGTTTGCGATTCTAAAGTGT---AATGATAAAAAGTTCAATGGATCAGGACCATGTACAAATGTCAGCACAGTACAATGTACACATGGAATTAGACCAGTAGTGTCAACTCAACTGTTGTTAAATGGCAGTCTAGCAGAAGAG---GAGGTAGTAATTAGATCTGAAAATTTCACGAACAATGTTAAAACCATAATAGTACATCTGAATAAATCTGTAGTAATTAATTGTACAAGACCCAACAACAATACAAGAAGAGGTATACATATGGGA------------CCAGGGAAA---GCATATTTT---ACAGGAGAAATAATAGGAGATATAAGACAAGCACATTGTAACATT------AGTAGAAAAGAATGGAATGACACTTTAAAACAGATAGTTATAAAATTAGGA---GAACAATTTAGA------------AATAAAACA---ATAGCCTTTAATCAA---------TCCTCAGGAGGGGACATTGAAATTGTAACACACAGTTTTAATTGTGGAGGGGAATTTTTCTACTGTAATTCAACACAACTGTTTAATAGTACTTGGAATAAT---------AATGGTACTAGG------------------------------AATGATACTAGGGAGTCA------------AATGACACT---------------------------ATCATCACACTACCATGCAGAATCAAACAAATTATAAACATGTGGCAGCAAGTAGGCAAAGCAATGTATGCCCCTCCCATCAGAGGACAAATTAGATGTTCATCAAATATCACAGGGCTGCTGTTAACAAGAGATGGTGGT------------------AACGAGAGCGAC---------------------------GCTGAGACCTTCAGACCTGGAGGAGGAGATATGAGGGACAATTGGAGA---AGTGAATTATACAAATATAAAGTAGTAAAAATTGAACCA---TTAGGAGTAGCACCC---TCCAAGGCAAAGAGAAGAGTGGTGCAGAGAGAA---AAAAGAGCAGTG---GGAACAATAGGA---GCTATG---TTCCTT---GGG---------TTCTTGGGA---GCAGCAGGAAGCACTATGGGCGCAGCGTCACTG---ACGCTGACGGTACAGGCCAGACTATTATTGTCTGGTATAGTGCAACAGCAGAACAATCTGCTGAGGGCTATTGAGGCGCAACAACATATTTTGCAACTCACGGTCTGGGGCATTAAACAGCTCCAGGCAAGA---GTCCTGGCTGTGGAAAGATACCTAAGGGATCAACAGCTCCTAGGGATTTGGGGTTGCTCTGGAAAACTCATCTGCACCACTGCTGTGCCTTGGAACGCTAGTTGGAGT---------------------------AATAAATCTCAAGAGGAGATTTGGGAG---AACATGACCTGGATGCAGTGGGATAGAGAAATTAAC------AATTACACAAACCTCATATATACCTTACTTGAAGAATCGCAGAACCAGCAAGAAAAGAATGAACAAGACTTATTAGCATTGGATGAATGGGCAAATTTGTGGAATTGGTTTGCCATATCAAAGTGGCTGTGGTATATAAAAATATTCATAATGATAGTAGGCGGCTTGATAGGTTTAAGAATAGTTTTTGCTGTACTTTCTATAGTGAATAGAGTTAGGCAGGGATACTCACCATTATCGTTTCAGACCCAC---CTCCCAGCCAGGAGGGGA------CCCGACAGGCCCGAAGGAATCGAAGAAGAAGGTGGAGAGAGAGACAGAGACAGATCAGTTCGCTTAGTACATGGATTCTTAGCACTCATCTGGGACGACCTACGGAGCCTGACCCTCTTCAGCTACCACCGCTTGAGAGACTTACTCTTGATTATAGCGAGGATTGTGGAAACTCTGGGACGCAGG---------------GGGTGGGAAGCCCTCAAGTATTGGTGG---AATCTCCTGCAATATTGG---------------------------------------------------AGTCAGGAACTAAAGAATAGTGCTGTTAGCTTGCTTGACGCCATAGCTATAGCAGTAGCTGAGGGAACAGATAGGGTTATAAAAGTAGTACGAAGA------------------GCCTTTAGAGCTATTCTCCACATACCTGTAAGAATAAGACAGGGCTTAGAAAGGGCTTTGCTATAA

2.SC22.Trinidad.EU576888 ATGAGAGTGAAGGAGATCAAGAGGAATTGTCAGCGCTTG---------TGGAGATGGGGC------------------------ACCATGCTCCTTGGGATGTTGATGATC------------TGTAGTGCTACA---------CAACAATTGTGGGTTACAGTCTATTATGGGGTACCTGTGTGGAAAGAAGCAACCACCACTCTATTTTGTGCATCAGATGCTAAAGCATATGATACAGAGGCACATAAT---GTCTGGGCCACACATGCCTGTGTACCCACAGACCCCAACCCACAAGAAGTAGTATTG---GGAAATGTGACAGAAAATTTTAACATGTGGAAAAATAACATGGTAGAACAAATGCATGAGGATATAATCAGTTTATGGGATCAAAGCCTAAAGCCATGTGTAAAATTAACCCCACTCTGTGTTACTTTAAACTGCACTGATTATGTGAAAAATGATACTAGTAGCACT---------------------------------------------------------------------------------------------AACAGTAGCAGCTGGGAGAAAGGAGAAATAAAAAACTGCTCTTTCAATATCACC---ACAAGCATAAAAGAT------AAGATGCAGAGGGAATATGCACTTTTTTATAAACTTGATGTAATACCAATAGATAGTGAGAATAATAGTAATATAAATAGTGGGAATAATAGTAATAGTAATTTTAGT------AGCTATAGTAATTTTAGATTGATAAATTGTAATACCTCAGTCATTACACAGGCCTGTCCAAAGGTATCCTTTGAACCAATTCCCATACATTATTGTGCCCCGGCTGGGTTTGCGATTCTAAAGTGT---AATGATAAAAAGTTCAATGGATCAGGACAATGTACAAATGTCAGCACAGTACAATGTACACATGGAATTAGACCAGTAGTGTCAACTCAACTGTTGTTAAATGGCAGTCTAGCAGAAGAG---GAGGTAGTAATTAGATCTGAAAATTTCACGAACAATGTTAAAACCATAATAGTACATCTGAATAAATCTGTAGTAATTAATTGTACAAGACCCAACAACAATACAAGAAGAGGTATACATATGGGA------------CCAGGGAAA---GCATATTTT---ACAGGAGAAATAATAGGAGATATAAGACAAGCACATTGTAACATT------AGTAGAAAAGAATGGAATGACACTTTAAAACAGATAGTTATAAAATTAGGA---GAACAATTTAGA------------AATAAAACA---ATAGCCTTTAATCAA---------TCCTCAGGAGGGGACATTGAAATTGTAACACACAGTTTTAATTGTGGAGGGGAATTTTTCTACTGTAATTCAACACAACTGTTTAATAGTACTTGGAATAAT---------AATGGTACTAGG------------------------------AATGATACTAGGGAGTCA------------AATGACACT---------------------------ATCATCACACTACCATGCAGAATCAAACAAATTATAAACATGTGGCAGCAAGTAGGCAAAGCAATGTATGCCCCTCCCATCAGAGGACAAATTAGATGTTCATCAAATATCACAGGGCTGCTGTTAACAAGAGATGGTGGT------------------AACGAGAGCGAC---------------------------GCTGAGACCTTCAGACCTGGAGGAGGAGATATGAGGGACAATTGGAGA---AGTGAATTATACAAATATAAAGTAGTAAAAATTGAACCA---TTAGGAGTAGCACCC---TCCAAGGCAAAGAGAAGAGTGGTGCAGAGAGAA---AAAAGAGCAGTG---GGAACAATAGGA---GCTGTG---TTCCTT---GGG---------TTCTTGGGA---GCAGCAGGAAGCACTATGGGCGCAGCGTCACTG---ACGCTGACGGTACAGGCCAGACTATTATTGTCTGGTATAGTGCAACAGCAGAACAATCTGCTGAGGGCTATTGAGGCGCAACAACATATTTTGCAACTCACGGTCTGGGGCATTAAACAGCTCCAGGCAAGA---GTCCTGGCTGTGGAAAGATACCTAAGGGATCAACAGCTCCTAGGGATTTGGGGTTGCTCTGGAAAACTCATCTGCACCACTGCTGTGCCTTGGAACGCTAGTTGGAGT---------------------------AATAAATCTCAAGAGGAGATTTGGGAA---AACATGACCTGGATGCAGTGGGATAGAGAAATTAAC------AATTACACAAACCTCATATATACCTTACTTGAAGAATCGCAGAACCAGCAAGAAAAGAATGAACAAGACTTATTAGCATTGGATGAATGGGCAAATTTGTGGAATTGGTTTGCCATATCAAAGTGGCTGTGGTATATAAAAATATTCATAATGATAGTAGGCGGCTTGATAGGTTTAAGAATAGTTTTTGCTGTACTTTCTATAGTGAATAGAGTTAGGCAGGGATACTCACCATTATCGTTTCAGACCCAC---CTCCCAGCCAGGAGGGGA------CCCGACAGGCCCGAAGGAATCGAAGAAGAAGGTGGAGAGAGAGACAGAGACAGATCAGTTCGCTTAGTACATGGATTCTTAGCACTCATCTGGGACGACCTACGGAGCCTGACCCTCTTCAGCTACCACCGCTTGAGAGACTTACTCTTGATTATAGCGAGGATTGTGGAAACTCTGGGACGCAGG---------------GGGTGGGAAGCCCTCAAGTATTGGTGG---AATCTCCTGCAATATTGG---------------------------------------------------AGTCAGGAACTAAAGAATAGTGCTGTTAGCTTGCTTGACGCCATAGCTATAGCAGTAGCTGAGGGAACAGATAGGGTTATAAAAGTAGTACGAAGA------------------GCCTTTAGAGCTATTCTCCACATACCTGTAAGAATAAGACAGGGCTTAGAAAGGGCTTTGCTATAA

2.SC22.Trinidad.EU576875 ATGAGAGTGAAGGAGATCAAGAGGAATTGTCAGCGCTTG---------TGGAGATGGGGC------------------------ACCATGCTCCTTGGGATGTTGATGATC------------TGTAGTGCTACA---------CAACAATTGTGGGTTACAGTCTATTATGGGGTACCTGTGTGGAAAGAAGCAACCACCACTCTATTTTGTGCATCAGATGCTAAAGCATATGATACAGAGGCACATAAT---GTCTGGGCCACACATGCCTGCGTACCCACAGACCCCAACCCACAAGAAGTAGTATTG---GGAAATGTGACAGAAAATTTTAACATGTGGAAAAATAACATGGTAGAACAAATGCATGAGGATATAATCAGTTTATGGGATCAAAGCCTAAAGCCATGTGTAAAATTAACCCCACTCTGTGTTACTTTAAACTGCACTGATTATGTGAAAAATGATACTAGTAGCACT---------------------------------------------------------------------------------------------AACAGTAGCAGCTGGGAGAAAGGAGAAATAAAAAACTGCTCTTTCAATATCACC---ACAAGCATAAAAGAT------AAGATGCAGAGGGAATATGCACTTTTTTATAAACTTGATGTAATACCAATAGATAGTGAGAATAATAGTAATATAAATAGTGGGAATAATAGTAATAGTAATTTTAGT------AGCTATAGTAATTTTAGATTGATAAATTGTAATACCTCAGTCATTACACAGGCCTGTCCAAAGGTATCCTTTGAACCAATTCCCATACATTATTGTGCCCCGGCTGGGTTTGCGATTCTAAAGTGT---AATGATAAAAGGTTCAATGGATCAGGACCATGTACAAATGTCAGCACAGTACAATGTACACATGGAATTAGACCAGTAGTGTCAACTCAACTGTTGTTAAATGGCAGTCTAGCAGAAGAG---GAGGTAGTAATTAGATCTGAAAATTTCACGAACAATGTTAAAACCATAATAGTACATCTGAATAAATCTGTAGTAATTAATTGTACAAGACCCAACAACAATACAAGAAGAGGTATACATATGGGA------------CCAGGGAAA---GCATATTTT---ACAGGAGAAATAATAGGAGATATAAGACAAGCACATTGTAACATT------AGTAAAAAAGAATGGAATGACACTTTAAAACAGATAGTTATAAAATTAGGA---GAACAATTTAGA------------AATAAAACA---ATAGCCTTTAATCAA---------TCCTCAGGAGGGGACATTGAAATTGTAACACACAGTTTTAATTGTGGAGGGGAATTTTTCTACTGTAATTCAACACAACTGTTTAATAGTACTTGGAATAAT---------AATGGTACTAGG------------------------------AATGATACTAGGGAGTCA------------AATGACACT---------------------------ATCATCACACTACCATGCAGAATCAAACAAATTATAAACATGTGGCAGCAAGTAGGCAAAGCAATGTATGCCCCTCCCATCAGAGGACAAATTAGATGTTCATCAAATATCACAGGGCTGCTGTTAACAAGAGATGGTGGT------------------AACGAGAGCGAC---------------------------GCTGAGACCTTCAGACCTGGAGGAGGAGATATGAGGGACAATTGGAGA---AGTGAATTATACAAATATAAAGTAGTAAAAATTGAACCA---TTAGGAGTAGCACCC---TCCAAGGCAAAGAGAAGAGTGGTGCAGAGAGAA---AAAAGAGCAGTG---GGAACAATAGGA---GCTATG---TTCCTT---GGG---------TTCTTGGGA---GCAGCAGGAAGCACTATGGGCGCAGCGTCACTG---ACGCTGACGGTACAGGCCAGACTATTATTGTCTGGTATAGTGCAACAGCAGAACAATCTGCTGAGGGCTATTGAGGCGCAACAACATATTTTGCAACTCACGGTCTGGGGCATTAAACAGCTCCAGGCAAGA---GTCCTGGCTGTGGAAAGATACCTAAGGGATCAACAGCTCCTAGGGATTTGGGGTTGCTCTGGAAAACTCATCTGCACCACTGCTGTGCCTTGGAACGCTAGTTGGAGT---------------------------AATAAATCTCAAGAGGAGATTTGGGAG---AACATGACCTGGATGCAGTGGGATAGAGAAATTAAC------AATTACACAAACCTCATATATACCTTACTTGAAGAATCGCAGAACCAGCAAGAAAAGAATGAACAAGACTTATTAGCATTGGATGAATGGGCAAATTTGTGGAATTGGTTTGCCATATCAAAGTGGCTGTGGTATATAAAAATATTCATAATGATAGTAGGCGGCTTGATAGGTTTAAGAATAGTTTTTGCTGTACTTTCTATAGTGAATAGAGTTAGGCAGGGATACTCACCATTATCGTTTCAGACCCAC---CTCCCAGCCAGGAGGGGA------CCCGACAGGCCCGAAGGAATCGAAGAAGAAGGTGGAGAGAGAGACAGAGACAGATCAGTTCGCTTAGTACATGGATTCTTAGCACTCATCTGGGACGACCTACGGAGCCTGACCCTCTTCAGCTACCACCGCTTGAGAGACTTACTCTTGATTATAGCGAGGATTGTGGAAACTCTGGGACGCAGG---------------GGGTGGGAAGCCCTCAAGTATTGGTGG---AATCTCCTGCAATATTGG---------------------------------------------------AGTCAGGAACTAAAGAATAGTGCTGTTAGCTTGCTTGACGCCATAGCTATAGCAGTAGCTGAGGGAACAGATAGGGTTATAAAAGTAGTACGAAGA------------------GCCTTTAGAGCTATTCTCCACATACCTGTAAGAATAAGACAGGGCTTAGAAAGGGCTTTGCTATAA

2.SC22.Trinidad.EU576883 ATGAGAGTGAAGGAGATCAAGAGGAATTGTCAGCGCTTG---------TGGAGATGGGGC------------------------ACCATGCTCCTTGGGATGTTGATGATC------------TGTAGTGCTACA---------CAACAATTGTGGGTTACAGTCTATTATGGGGTACCTGTGTGGAAAGAAGCAACCACCACTCTATTTTGTGCATCAGATGCTAAAGCATATGATACAGAGGCACATAAT---GTCTGGGCCACACATGCCTGTGTACCCACAGACCCCAACCCACAAGAAGTAGTATTG---GGAAATGTGACAGAAAATTTTAACATGTGGAAAAATAACATGGTAGAACAAATGCATGAGGATATAATCAGTTTATGGGATCAAAGCCTAAAGCCATGTGTAAAATTAACCCCACTCTGTGTTACTTTAAACTGCACTGATTATGTGAAAAATGATACTAGTAGCACT---------------------------------------------------------------------------------------------AACAGTAGCAGCTGGGAGAAAGGAGAAATAAAAAACTGCTCTTTCAATATCACC---ACAAGCATAAAAGAT------AAGATGCAGAGGGAATATGCACTTTTTTATAAACTTGATGTAATACCAATAGATAGTGAGAATAATAGTAATATAAATAGTGGGAATAATAGTAATAGTAATTTTAGT------AGCTATAGTAATTTTAGATTGATAAATTGTAATACCTCAGTCATTACACAGGCCTGTCCAAAGGTATCCTTTGAACCAATTCCCATACATTATTGTGCCCCGGCTGGGTTTGCGATTCTAAAGTGT---AATGATAAAAAGTTCAATGGATCAGGACCATGTACAAATGTCAGCACAGTACAATGTACACATGGAATTAGACCAGTAGTGTCAACTCAACTGTTGTTAAATGGCAGTCTAGCAGAAGAG---GAGGTAGTAATTAGATCTGAAAATTTCACGAACAATGTTAAAACCATAATAGTACATCTGAATAAATCTGTAGTAATTAATTGTACAAGACCCAACAACAATACAAGAAGAGGTATACATATGGGA------------CCAGGGAAA---GCATATTTT---ACAGGAGAAATAATAGGAGATATAAGACAAGCACATTGTAACATT------AGTAGAAAAGAATGGAATGACACTTTAAAACAGATAGTTATAAAATTAGGA---GAACAATTTAGA------------AATAAAACA---ATAGCCTTTAATCAA---------TCCTCAGGAGGGGACATTGAAATTGTAACACACAGTTTTAATTGTGGAGGGGAATTTTTCTACTGTAATTCAACACAACTGTTTAATAGTACTTGGAATAAT---------AATGGTACTAGG------------------------------AATGATACTAGGGAGTCA------------AATGACACT---------------------------ATCATCACACTACCATGCAGAATCAAACAAATTATAAACATGTGGCAGCAAGTAGGCAAAGCAATGTATGCCCCTCCCATCAGAGGACAAATTAGATGTTCATCAAATATCACAGGGCTGCTGTTAACAAGAGATGGTGGT------------------AACGAGAGCGAC---------------------------GCTGAGACCTTCAGACCTGGAGGAGGAGATATGAGGGACAATTGGAGA---AGTGAATTATACAAATATAAAGTAGTAAAAATTGAACCA---TTAGGAGTAGCACCC---TCCAAGGCAAAGAGAAGAGTGGTGCAGAGAGAA---AAAAGAGCAGTG---GGAACAATAGGA---GCTATG---TTCCTT---GGG---------TTCTTGGGA---GCAGCAGGAAGCACTATGGGCGCAGCGTCACTG---ACGCTGACGGTACAGGCCAGACTATTATTGTCTGGTATAGTGCAACAGCAGAACAATCTGCTGAGGGCTATTGAGGCGCAACAACATATTTTGCAACTCACGGTCTGGGGCATTAAACAGCTCCAGGCAAGA---GTCCTGGCTGTGGAAAGATACCTAAGGGATCAACAGCTCCTAGGGATTTGGGGTTGCTCTGGAAAACTCATCTGCACCACTGCTGTGCCTTGGAACGCTAGTTGGAGT---------------------------AATAAATCTCAAGAGGAGATTTGGGAG---AACATGACCTGGATGCAGTGGGATAGAGAAATTAAC------AATTACACAAACCTCATATATACCTTACTTGAAGAATCGCAGAACCAGCAAGAAAAGAATGAACAAGACTTATTAGCATTGGATGAATGGGCAAATTTGTGGAATTGGTTTGCCATATCAAAGTGGCTGTGGTATATAAAAATATTCATAATGATAGTAGGCGGCTTGATAGGTTTAAGAATAGTTTTTGCTGTACTTTCTATAGTGAATAGAGTTAGGCAGGGATACTCACCATTATCGTTTCAGACCCAC---CTCCCAGCCAGGAGGGGA------CCCGACAGGCCCGAAGGAATCGAAGAAGAAGGTGGAGAGAGAGACAGAGACAGATCAGTTCGCTTAGTACATGGATTCTTAGCACTCATCTGGGACGACCTACGGAGCCTGACCCTCTTCAGCTACCACCGCTTGAGAGACTTACTCTTGATTATAGCGAGGATTGTGGAAACTCTGGGACGCAGG---------------GGGTGGGAAGCCCTCAAGTATTGGTGG---AATCTCCTGCAATATTGG---------------------------------------------------AGTCAGGAACTAAAGAATAGTGCTGTTAGCTTGCTTGACGCCATAGCTATAGCAGTAGCTGAGGGAACAGATAGGGTTATAAAAGTAGTACGAAGA------------------GCCTTTAGAGCTATTCTCCACATACCTGTAAGAATAAGACAGGGCTTAGAAAGGGCTTTGCTATAA

2.SC22.Trinidad.EU576855 ATGAGAGTGAAGGAGATCAAGAGGAATTGTCAGCGCTTG---------TGGAGATGGGGC------------------------ACCATGCTCCTTGGGATGTTGATGATC------------TGTAGTGCTACA---------CAACAATTGTGGGTTACAGTCTATTATGGGGTACCTGTGTGGAAAGAAGCAACCACCACTCTATTTTGTGCATCAGATGCTAAAGCATATGATACAGAGGCACATAAT---GTCTGGGCCACACATGCCTGTGTACCCACAGACCCCAACCCACAAGAAGTAGTATTG---GGAAATGTGACAGAAAATTTTAACATGTGGAAAAATAACATGGTAGAACAAATGCATGAGGATATAATCAGTTTATGGGATCAAAGCCTAAAGCCATGTGTAAAATTAACCCCACTCTGTGTTACTTTAAACTGCACTGATTATGTGAAAAATGATACTAGTAGCACT---------------------------------------------------------------------------------------------AACAGTAGCAGCTGGGAGAAAGGAGAAATAAAAAACTGCTCTTTCAATATCACC---ACAAGCATAAAAGAT------AAGATGCAGAGGGAATATGCACTTTTTTATAAACTTGATGTAATACCAATAGATAGTGAGAATAATAGTAATATAAATAGTGGGAATAATAGTAATAGTAATTTTAGT------AGCTATAGTAATTTTAGATTGATAAATTGTAATACCTCAGTCATTACACAGGCCTGTCCAAAGGTATCCTTTGAACCAATTCCCATACATTATTGTGCCCCGGCTGGGTTTGCGATTCTAAAGTGT---AATGATAAAAAGTTCAATGGATCAGGACCATGTACAAATGTCAGCACAGTACAATGTACACATGGAATTAGACCAGTAGTGTCAACTCAACTGTTGTTAAATGGCAGTCTAGCAGAAGAG---GAGGTAGTAATTAGATCTGAAAATTTCACGAACAATGTTAAAACCATAATAGTACATCTGAATAAATCTGTAGTAATTAATTGTACAAGACCCAACAACAATACAAGAAGAGGTATACATATGGGA------------CCAGGGAAA---GCATATTTT---ACAGGAGAAATAATAGGAGATATAAGACAAGCACATTGTAACATT------AGTAGAAAAGAATGGAATGACACTTTAAAACAGATAGTTATAAAATTAGGA---GAACAATTTAGA------------AATAAAACA---ATAGCCTTTAATCAA---------TCCTCAGGAGGGGACATTGAAATTGTAACACACAGTTTTAATTGTGGAGGGGAATTTTTCTACTGTAATTCAACACAACTGTTTAATAGTACTTGGAATAAT---------AATGGTACTAGG------------------------------AATGATACTAGGGAGTCA------------AATGACACT---------------------------ATCATCACACTACCATGCAGAATCAAACAAATTATAAACATGTGGCAGCAAGTAGGCAAAGCAATGTATGCCCCTCCCATCAGAGGACAAATTAGATGTTCATCAAATATCACAGGGCTGCTGTTAACAAGAGATGGTGGT------------------AACGAGAGCGAC---------------------------GCTGAGACCTTCAGACCTGGAGGAGGAGATATGAGGGACAATTGGAGA---AGTGAATTATACAAATATAAAGTAGTAAAAATTGAACCA---TTAGGAGTAGCACCC---TCCAAGGCAAAGAGAAGAGTGGTGCAGAGAGAA---AAAAGAGCAGTG---GGAACAATAGGA---GCTATG---TTCCTT---GGG---------TTCTTGGGA---GCAGCAGGAAGCACTATGGGCGCAGCGTCACTG---ACGCTGACGGTACAGGCCAGACTATTATTGTCTGGTATAGTGCAACAGCAGAACAATCTGCTGAGGGCTATTGAGGCGCAACAACATATTTTGCAACTCACGGTCTGGGGCATTAAACAGCTCCAGGCAAGA---GTCCTGGCTGTGGAAAGATACCTAAGGGATCAACAGCTCCTAGGGATTTGGGGTTGCTCTGGAAAACTCATCTGCACCACTGCTGTGCCTTGGAACGCTAGTTGGAGT---------------------------AATAAATCTCAAGAGGAGATTTGGGAG---AACATGACCTGGATGCAGTGGGATAGAGAAATTAAC------AATTACACAAACCTCATATATACCTTACTTGAAGAATCGCAGAACCAGCAAGAAAAGAATGAACAAGACTTATTAGCATTGGATGAATGGGCAAATTTGTGGAATTGGTTTGCCATATCAAAGTGGCTGTGGTATATAAAAATATTCATAATGATAGTAGGCGGCTTGATAGGTTTAAGAATAGTTTTTGCTGTACTTTCTATAGTGAATAGAGTTAGGCAGGGATACTCACCATTATCGTTTCAGACCCAC---CTCCCAGCCAGGAGGGGA------CCCGACAGGCCCGAAGGAATCGAAGAAGAAGGTGGAGAGAGAGACAGAGACAGATCAGTTCGCTTAGTACATGGATTCTTAGCACTCATCTGGGACGACCTACGGAGCCTGACCCTCTTCAGCTACCACCGCTTGAGAGACTTACTCTTGATTATAGCGAGGATTGTGGAAACTCTGGGACGCAGG---------------GGGTGGGAAGCCCTCAAGTATTGGTGG---AATCTCCTGCAATATTGG---------------------------------------------------AGTCAGGAACTAAAGAATAGTGCTGTTAGCTTGCTTGACGCCATAGCTATAGCAGTAGCTGAGGGAACAGATAGGGTTATAAAAGTAGTACGAAGA------------------GCCTTTAGAGCTATTCTCCACATACCTGTAAGAATAAGACAGGGCTTAGAAAGGGCTTTGCTATAA

2.SC22.Trinidad.EU576856 ATGAGAGTGAAGGAGATCAAGAGGAATTGTCAGCGCTTG---------TGGAGATGGGGC------------------------ACCATGCTCCTTGGGATGTTGATGATC------------TGTAGTGCTACA---------CAACAATTGTGGGTTACAGTCTATTATGGGGTACCTGTGTGGAAAGAAGCAACCACCACTCTATTTTGTGCATCAGATGCTAAAGCATATGATACAGAGGCACATAAT---GTCTGGGCCACACATGCCTGTGTACCCACAGACCCCAACCCACAAGAAGTAGTATTG---GGAAATGTGACAGAAAATTTTAACATGTGGAAAAATAACATGGTAGAACAAATGCATGAGGATATAATCAGTTTATGGGATCAAAGCCTAAAGCCATGTGTAAAATTAACCCCACTCTGTGTTACTTTAAACTGCACTGATTATGTGAAAAATGATACTAGTAGCACT---------------------------------------------------------------------------------------------AACAGTAGCAGCTGGGAGAAAGGAGAAATAAAAAACTGCTCTTTCAATATCACC---ACAAGCATAAAAGAT------AAGATGCAGAGGGAATATGCACTTTTTTATAAACTTGATGTAATACCAATAGATAGTGAGAATAATAGTAATATAAATAGTGGGAATAATAGTAATAGTAATTTTAGT------AGCTATAGTAATTTTAGATTGATAAATTGTAATACCTCAGTCATTACACAGGCCTGTCCAAAGGTATCCTTTGAACCAATTCCCATACATTATTGTGCCCCGGCTGGGTTTGCGATTCTAAAGTGT---AATGATAAAAAGTTCAATGGATCAGGACCATGTACAAATGTCAGCACAGTACAATGTACACATGGAATTAGACCAGTAGTGTCAACTCAACTGTTGTTAAATGGCAGTCTAGCAGAAGAG---GAGGTAGTAATTAGATCTGAAAATTTCACGAACAATGTTAAAACCATAATAGTACATCTGAATAAATCTGTAGTAATTAATTGTACAAGACCCAACAACAATACAAGAAGAGGTATACATATGGGA------------CCAGGGAAA---GCATATTTT---ACAGGAGAAATAATAGGAGATATAAGACAAGCACATTGTAACATT------AGTAGAAAAGAATGGAATGACACTTTAAAACAGATAGTTATAAAATTAGGA---GAACAATTTAGA------------AATAAAACA---ATAGCCTTTAATCAA---------TCCTCAGGAGGGGACATTGAAATTGTAACACACAGTTTTAATTGTGGAGGGGAATTTTTCTACTGTAATTCAACACAACTGTTTAATAGTACTTGGAATAAT---------AATGGTACTAGG------------------------------AATGATACTAGGGAGTCA------------AATGACACT---------------------------ATCATCACACTACCATGCAGAATCAAACAAATTATAAACATGTGGCAGCAAGTAGGCAAAGCAATGTATGCCCCTCCCATCAGAGGACAAATTAGATGTTCATCAAATATAACAGGGCTGCTGTTAACAAGAGATGGTGGT------------------AACGAGAGCGAC---------------------------GCTGAGACCTTCAGACCTGGAGGAGGAGATATGAGGGACAATTGGAGA---AGTGAATTATACAAATATAAAGTAGTAAAAATTGAACCA---TTAGGAGTAGCACCC---TCCAAGGCAAAGAGAAGAGTGGTGCAGAGAGAA---AAAAGAGCAGTG---GGAACAATAGGA---GCTATG---TTCCTT---GGG---------TTCTTGGGA---GCAGCAGGAAGCACTATGGGCGCAGCGTCACTG---ACGCTGACGGTACAGGCCAGACTATTATTGTCTGGTATAGTGCAACAGCAGAACAATCTGCTGAGGGCTATTGAGGCGCAACAACATATTTTGCAACTCACGGTCTGGGGCATTAAACAGCTCCAGGCAAGA---GTCCTGGCTGTGGAAAGATACCTAAGGGATCAACAGCTCCTAGGGATTTGGGGTTGCTCTGGAAAACTCATCTGCACCACTGCTGTGCCTTGGAACGCTAGTTGGAGT---------------------------AATAAATCTCAAGAGGAGATTTGGGAG---AACATGACCTGGATGCAGTGGGATAGAGAAATTAAC------AATTACACAAACCTCATATATACCTTACTTGAAGAATCGCAGAACCAGCAAGAAAAGAATGAACAAGACTTATTAGCATTGGATGAATGGGCAAATTTGTGGAATTGGTTTGCCATATCAAAGTGGCTGTGGTATATAAAAATATTCATAATGATAGTAGGCGGCTTGATAGGTTTAAGAATAGTTTTTGCTGTACTTTCTATAGTGAATAGAGTTAGGCAGGGATACTCACCATTATCGTTTCAGACCCAC---CTCCCAGCCAGGAGGGGA------CCCGACAGGCCCGAAGGAATCGAAGAAGAAGGTGGAGAGAGAGACAGAGACAGATCGGTTCGATTAGTACATGGATTCTTAGCACTCATCTGGGACGACCTACGGAGCCTGACCCTCTTCAGCTACCACCGCTTGAGAGACTTACTCTTGATTATAGCGAGGATTGTGGAAACTCTGGGACGCAGG---------------GGGTGGGAAGCCCTCAAGTATTGGTGG---AATCTCCTGCAATATTGG---------------------------------------------------AGTCAGGAACTAAAGAATAGTGCTGTTAGCTTGCTTGACGCCATAGCTATAGCAGTAGCTGAGGGAACAGATAGGGTTATAAAAGTAGTACGAAGA------------------GCCTTTAGAGCTATTCTCCACATACCTGTAAGAATAAGACAGGGCTTAGAAAGGGCTTTGCTATAA

2.SC22.Trinidad.EU576877 ATGAGAGTGAAGGAGATCAAGAGGAATTGTCAGCGCTTG---------TGGAGATGGGGC------------------------ACCATGCTCCTTGGGATGTTGATGATC------------TGTAGTGCTACA---------CAACAATTGTGGGTTACAGTCTATTATGGGGTACCTGTGTGGAAAGAAGCAACCACCACTCTATTTTGTGCATCAGATGCTAAAGCATATGATACAGAGGCACATAAT---GTCTGGGCCACACATGCCTGTGTACCCACAGACCCCAACCCACAAGAAGTAGTATTG---GGAAATGTGACAGAAAATTTTAACATGTGGAAAAATAACATGGTAGAACAAATGCATGAGGATATAATCAGTTTATGGGATCAAAGCCTAAAGCCATGTGTAAAATTAACCCCACTCTGTGTTACTTTAAACTGCACTGATTATGTGAAAAATGATACTAGTAGCACT---------------------------------------------------------------------------------------------AACAGTAGCAGCTGGGAGAAAGGAGAAATAAAAAACTGCTCTTTCAATATCACC---ACAAGCATAAAAGAT------AAGATGCAGAGGGAATATGCACTTTTTTATAAACTTGATGTAATACCAATAGATAGTGAGAATAATAGTAATATAAATAGTGGGAATAATAGTAATAGTAATTTTAGT------AGCTATAGTAATTTTAGATTGATAAATTGTAATACCTCAGTCATTACACAGGCCTGTCCAAAGGTATCCTTTGAACCAATTCCCATACATTATTGTGCCCCGGCTGGGTTTGCGATTCTAAAGTGT---AATGATAAAAAGTTCAATGGATCAGGACCATGTACAAATGTCAGCACAGTACAATGTACACATGGAATTAGACCAGTAGTGTCAACTCAACTGTTGTTAAATGGCAGTCTAGCAGAAGAG---GAGGTAGTAATTAGATCTGAAAATTTCACGAACAATGTTAAAACCATAATAGTACATCTGAATAAATCTGTAGTAATTAATTGTACAAGACCCAACAACAATACAAGAAGAGGTATACATATGGGA------------CCAGGGAAA---GCATATTTT---ACAGGAGAAATAATAGGAGATATAAGACAAGCACATTGTAACATT------AGTAGAAAAGAATGGAATGACACTTTAAAACAGATAGTTATAAAATTAGGA---GAACAATTTAGA------------AATAAAACA---ATAGCCTTTAATCAA---------TCCTCAGGAGGGGACATTGAAATTGTAACACACAGTTTTAATTGTGGAGGGGAATTTTTCTACTGTAATTCAACACAACTGTTTAATAGTACTTGGAATAAT---------AATGGTACTAGG------------------------------AATGATACTAGGGAGTCA------------AATGACACT---------------------------ATCATCACACTACCATGCAGAATCAAACAAATTATAAACATGTGGCAGCAAGTAGGCAAAGCAATGTATGCCCCTCCCATCAGAGGACAAATTAGATGTTCATCAAATATCACAGGGCTGCTGTTAACAAGAGATGGTGGT------------------AACGAGAGCGAC---------------------------GCTGAGACCTTCAGACCTGGAGGAGGAGATATGAGGGACAATTGGAGA---AGTGAATTATACAAATATAAAGTAGTAAAAATTGAACCA---TTAGGAGTAGCACCC---TCCAAGGCAAAGAGAAAAGTGGTGCAGAGAGAA---AAAAGAGCAGTG---GGAACAATAGGA---GCTATG---TTCCTT---GGG---------TTCTTGGGA---GCAGCAGGAAGCACTATGGGCGCAGCGTCACTG---ACGCTGACGGTACAGGCCAGACTATTATTGTCTGGTATAGTGCAACAGCAGAACAATCTGCTGAGGGCTATTGAGGCGCAACAACATATTTTGCAACTCACGGTCTGGGGCATTAAACAGCTCCAGGCAAGA---GTCCTGGCTGTGGAAAGATACCTAAGGGATCAACAGCTCCTAGGGATTTGGGGTTGCTCTGGAAAACTCATCTGCACCACTGCTGTGCCTTGGAACGCTAGTTGGAGT---------------------------AATAAATCTCAAGAGGAGATTTGGGAG---AACATGACCTGGATGCAGTGGGATAGAGAAATTAAC------AATTACACAAACCTCATATATACCTTACTTGAAGAATCGCAGAACCAGCAAGAAAAGAATGAACAAGACTTATTAGCATTGGATGAATGGGCAAATTTGTGGAATTGGTTTGCCATATCAAAGTGGCTGTGGTATATAAAAATATTCATAATGATAGTAGGCGGCTTGATAGGTTTAAGAATAGTTTTTGCTGTACTTTCTATAGTGAATAGAGTTAGGCAGGGATACTCACCATTATCGTTTCAGACCCAC---CTCCCAGCCAGGAGGGGA------CCCGACAGGCCCGAAGGAATCGAAGAAGAAGGTGGAGAGAGAGACAGAGACAGATCAGTTCGCTTAGTACATGGATTCTTAGCACTCATCTGGGACGACCTACGGAGCCTGACCCTCTTCAGCTACCACCGCTTGAGAGACTTACTCTTGATTATAGCGAGGATTGTGGAAACTCTGGGACGCAGG---------------GGGTGGGAAGCCCTCAAGTATTGGTGG---AATCTCCTGCAATATTGG---------------------------------------------------AGTCAGGAACTAAAGAATAGTGCTGTTAGCTTGCTTGACGCCATAGCTATAGCAGTAGCTGAGGGAACAGATAGGGTTATAAAAGTAGTACGAAGA------------------GCCTTTAGAGCTATTCTCCACATACCTGTAAGAATAAGACAGGGCTTAGAAAGGGCTTTGCTATAA

2.SC22.Trinidad.EU576881 ATGAGAGTGAAGGAGATCAAGAGGAATTGTCAGCGCTTG---------TGGAGATGGGGC------------------------ACCATGCTCCTTGGGATGTTGATGATC------------TGTAGTGCTACA---------CAACAATTGTGGGTTACAGTCTATTATGGGGTACCTGTGTGGAAAGAAGCAACCACCACTCTATTTTGTGCATCAGATGCTAAAGCATATGATACAGAGGCACATAAT---GTCTGGGCCACACATGCCTGTGTACCCACAGACCCCAACCCACAAGAAGTAGTATTG---GGAAATGTGACAGAAAATTTTAACATGTGGAAAAATAACATGGTAGAACAAATGCATGAGGATATAATCAGTTTATGGGATCAAAGCCTAAAGCCATGTGTAAAATTAACCCCACTCTGTGTTACTTTAAACTGCACTGATTATGTGAAAAATGATACTAGTAGCACT---------------------------------------------------------------------------------------------AACAGTAGCAGCTGGGAGAAAGGAGAAATAAAAAACTGCTCTTTCAATATCACC---ACAAGCATAAAAGAT------AAGATGCAGAGGGAATATGCACTTTTTTATAAACTTGATGTAATACCAATAGATAGTGAGAATAATAGTAATATAAATAGTGGGAATAATAGTAATAGTAATTTTAGT------AGCTATAGTAATTTTAGATTGATAAATTGTAATACCTCAGTCATTACACAGGCCTGTCCAAAGGTATCCTTTGAACCAATTCCCATACATTATTGTGCCCCGGCTGGGTTTGCGATTCTAAAGTGT---AATGATAAAAAGTTCAATGGATCAGGACCATGTACAAATGTCAGCACAGTACAATGTACACATGGAATTAGACCAGTAGTGTCAACTCAACTGTTGTTAAATGGCAGTCTAGCAGAAGAG---GAGGTAGTAATTAGATCTGAAAATTTCACGAACAATGTTAAAACCATAATAGTACATCTGAATAAATCTGTAGTAATTAATTGTACAAGACCCAACAACAATACAAGAAGAGGTATACATATGGGA------------CCAGGGAAA---GCATATTTT---ACAGGAGAAATAATAGGAGATATAAGACAAGCACATTGTAACATT------AGTAGAAAAGAATGGAATGACACTTTAAAACAGATAGTTATAAAATTAGGA---GAACAATTTAGA------------AATAAAACA---ATAGCCTTTAATCAA---------TCCTCAGGAGGGGACATTGAAATTGTAACACACAGTTTTAATTGTGGAGGGGAATTTTTCTACTGTAATTCAACACAACTGTTTAATAGTACTTGGAATAAT---------AATGGTACTAGG------------------------------AATGATACTAGGGAGTCA------------AATGACACT---------------------------ATCATCACACTACCATGCAGAATCAAACAAATTATAAACATGTGGCAGCAAGTAGGCAAAGCAATGTATGCCCCTCCCATCAGAGGACAAATTAGATGTTCATCAAATATCACAGGGCTGCTGTTAACAAGAGATGGTGGT------------------AACGAGAGCGAC---------------------------GCTGAGACCTTCAGACCTGGAGGAGGAGATATGAGGGACAATTGGAGA---AGTGAATTATACAAATATAAAGTAGTAAAAATTGAACCA---TTAGGAGTAGCACCC---TCCAAGGCAAAGAGAAGAGTGGTGCAGAGAGAA---AAAAGAGCAGTG---GGAACAATAGGA---GCTATG---TTCCTT---GGG---------TTCTTGGGA---GCAGCAGGAAGCACTATGGGCGCAGCGTCACTG---ACGCTGACGGTACAGGCCAGACTATTATTGTCTGGTATAGTGCAACAGCAGAACAATCTGCTGAGGGCTATTGAGGCGCAACAACATATTTTGCAACTCACGGTCTGGGGCATTAAACAGCTCCAGGCAAGA---GTCCTGGCTGTGGAAAGATACCTAAGGGATCAACAGCTCCTAGGAATTTGGGGTTGCTCTGGAAAACTCATCTGCACCACTGCTGTGCCTTGGAACGCTAGTTGGAGT---------------------------AATAAATCTCAAGAGGAGATTTGGGAG---AACATGACCTGGATGCAGTGGGATAGAGAAATTAAC------AATTACACAAACCTCATATATACCTTACTTGAAGAATCGCAGAACCAGCAAGAAAAGAATGAACAAGACTTATTAGCATTGGATGAATGGGCAAATTTGTGGAATTGGTTTGCCATATCAAAGTGGCTGTGGTATATAAAAATATTCATAATGATAGTAGGCGGCTTGATAGGTTTAAGAATAGTTTTTGCTGTACTTTCTATAGTGAATAGAGTTAGGCAGGGATACTCACCATTATCGTTTCAGACCCAC---CTCCCAGCCAGGAGGGGA------CCCGACAGGCCCGAAGGAATCGAAGAAGAAGGTGGAGAGAGAGACAGAGACAGATCAGTTCGCTTAGTACATGGATTCTTAGCACTCATCTGGGACGACCTACGGAGCCTGACCCTCTTCAGCTACCACCGCTTGAGAGACTTACTCTTGATTATAGCGAGGATTGTGGAAACTCTGGGACGCAGG---------------GGGTGGGAAGCCCTCAAGTATTGGTGG---AATCTCCTGCAATATTGG---------------------------------------------------AGTCAGGAACTAAAGAATAGTGCTGTTAGCTTGCTTGACGCCATAGCTATAGCAGTAGCTGAGGGAACAGATAGGGTTATAAAAGTAGTACGAAGA------------------GCCTTTAGAGCTATTCTCCACATACCTGTAAGAATAAGACAGGGCTTAGAAAGGGCTTTGCTATAA

2.SC22.Trinidad.EU576887 ATGAGAGTGAAGGAGATCAAGAGGAATTGTCAGCGCTTG---------TGGAGATGGGGC------------------------ACCATGCTCCTTGGGATGTTGATGATC------------TGTAGTGCTACA---------CAACAATTGTGGGTTACAGTCTATTATGGGGTACCTGTGTGGAAAGAAGCAACCACCACTCTATTTTGTGCATCAGATGCTAAAGCATATGATACAGAGGCACATAAT---GTCTGGGCCACACATGCCTGTGTACCCACAGACCCCAACCCACAAGAAGTAGTATTG---GGAAATGTGACAGAAAATTTTAACATGTGGAAAAATAACATGGTAGAACAAATGCATGAGGATATAATCAGTTTATGGGATCAAAGCCTAAAGCCATGTGTAAAATTAACCCCACTCTGTGTTACTTTAAACTGCACTGATTATGTGAAAAATGATACTAGTAGCACT---------------------------------------------------------------------------------------------AACAGTAGCAGCTGGGAGAAAGGAGAAATAAAAAACTGCTCTTTCAATATCACC---ACAAGCATAAAAGAT------AAGATGCAGAGGGAATATGCACTTTTTTATAAACTTGATGTAATACCAATAGATAGTGAGAATAATAGTAATATAAATAGTGGGAATAATAGTAATAGTAATTTTAGT------AGCTATAGTAATTTTAGATTGATAAATTGTAATACCTCAGTCATTACACAGGCCTGTCCAAAGGTATCCTTTGAACCAATTCCCATACATTATTGTGCCCCGGCTGGGTTTGCGATTCTAAAGTGT---AATGATAAAAAGTTCAATGGATCAGGACCATGTACAAATGTCAGCACAGTACAATGTACACATGGAATTAGACCAGTAGTGTCAACTCAACTGTTGTTAAATGGCAGTCTAGCAGAAGAG---GAGGTAGTAATTAGATCTGAAAATTTCACGAACAATGTTAAAACCATAATAGTACATCTGAATAAATCTGTAGTAATTAATTGTACAAGACCCAACAACAATACAAGAAGAGGTATACATATGGGA------------CCAGGGAAA---GCATATTTT---ACAGGAGAAATAATAGGAGATATAAGACAAGCACATTGTAACATT------AGTAGAAAAGAATGGAATGACACTTTAAAACAGATAGTTATAAAATTAGGA---GAACAATTTAGA------------AATAAAACA---ATAGCCTTTAATCAA---------TCCTCAGGAGGGGACATTGAAATTGTAACACACAGTTTTAATTGTGGAGGGGAATTTTTCTACTGTAATTCAACACAACTGTTTAATAGTACTTGGAATAAT---------AATGGTACTAGG------------------------------AATGATACTAGGGAGTCA------------AATGACACT---------------------------ATCATCACACTACCATGCAGAATCAAACAAATTATAAACATGTGGCAGCAAGTAGGCAAAGCAATGTATGCCCCTCCCATCAGAGGACAAATTAGATGTTCATCAAATATCACAGGGCTGCTGTTAACAAGAGATGGTGGT------------------AACGAGAGCGAC---------------------------GCTGAGACCTTCAGACCTGGAGGAGGAGATATGAGGGACAATTGGAGA---AGTGAATTATACAAATATAAAGTAGTAAAAATTGAACCA---TTAGGAGTAGCACCC---TCCAAGGCAAAGAGAAGAGTGGTGCAGAGAGAA---AAAAGAGCAGTG---GGAACAATAGGA---GCTATG---TTCCTT---GGG---------TTCTTGGGA---GCAGCAGGAAGCACTATGGGCGCAGCGTCACTG---ACGCTGACGGTACAGGCCAGACTATTATTGTCTGGTATAGTGCAACAGCAGAACAATCTGCTGAGGGCTATTGAGGCGCAACAACATATTTTGCAACTCACGGTCTGGGGCATTAAACAGCTCCAGGCAAGA---GTCCTGGCTGTGGAAAGATACCTAAGGGATCAACAGCTCCTAGGGATTTGGGGTTGCTCTGGAAAACTCATCTGCACCACTGCTGTGCCTTGGAACGCTAGTTGGAGT---------------------------AATAAATCTCAAGAGGAGATTTGGGAG---AACATGACCTGGATGCAGTGGGATAGAGAAATTAAC------AATTACACAAACCTCATATATACCTTACTTGAAGAATCGCAGAACCAGCAAGAAAAGAATGAACAAGACTTATTAGCATTGGATGAATGGGCAAATTTGTGGAATTGGTTTGCCATATCAAAGTGGCTGTGGTATATAAAAATATTCATAATGATAGTAGGCGGCTTGATAGGTTTAAGAATAGTTTTTGCTGTACTTTCTATAGTGAATAGAGTTAGGCAGGGATACTCACCATTATCGTTTCAGACCCAC---CTCCCAGCCAGGAGGGGA------CCCGACAGGCCCGAAGGAATCGAAGAAGAAGGTGGAGAGAGAGACAGAGACAGATCGATTCGATTAGTACATGGATTCTTAGCACTCATCTGGGACGACCTACGGAGCCTGACCCTCTTCAGCTACCACCGCTTGAGAGACTTACTCTTGATTATAGCGAGGATTGTGGAAACTCTGGGACGCAGG---------------GGGTGGGAAGCCCTCAAGTATTGGTGG---AATCTCCTGCAATATTGG---------------------------------------------------AGTCAGGAACTAAAGAATAGTGCTGTTAGCTTGCTTGACGCCATAGCTATAGCAGTAGCTGAGGGAACAGATAGGGTTATAAAAGTAGTACGAAGA------------------GCCTTTAGAGCTATTCTCCACATACCTGTAAGAATAAGACAGGGCTTAGAAAGGGCTTTGCTATAA

2.SC22.Trinidad.EU576859 ATGAGAGTGAAGGAGATCAAGAGGAATTGTCAGCGCTTG---------TGGAGATGGGGC------------------------ACCATGCTCCTTGGGATGTTGATGATC------------TGTAGTGCTACA---------CAACAATTGTGGGTTACAGTCTATTATGGGGTACCTGTGTGGAAAGAAGCAACCACCACTCTATTTTGTGCATCAGATGCTAAAGCATATGATACAGAGGCACATAAT---GTCTGGGCCACACATGCCTGTGTACCCACAGACCCCAACCCACAAGAAGTAGTATTG---GGAAATGTGACAGAAAATTTTAACATGTGGAAAAATAACATGGTAGAACAAATGCATGAGGATATAATCAGTTTATGGGATCAAAGCCTAAAGCCATGTGTAAAATTAACCCCACTCTGTGTTACTTTAAACTGCACTGATTATGTGAAAAATGATACTAGTAGCACT---------------------------------------------------------------------------------------------AACAGTAGCAGCTGGGAGAAAGGAGAAATAAAAAACTGCTCTTTCAATATCACC---ACAAGCATAAAAGAT------AAGATGCAGAGGGAATATGCACTTTTTTATAAACTTGATGTAATACCAATAGATAGTGAGAATAATAGTAATATAAATAGTGGGAATAATAGTAATAGTAATTTTAGT------AGCTATAGTAATTTTAGATTGATAAATTGTAATACCTCAGTCATTACACAGGCCTGTCCAAAGGTATCCTTTGAACCAATTCCCATACATTATTGTGCCCCGGCTGGGTTTGCGATTCTAAAGTGT---AATGATAAAAAGTTCAATGGATCAGGACCATGTACAAATGTCAGCACAGTACAATGTACACATGGAATTAGACCAGTAGTGTCAACTCAACTGTTGTTAAATGGCAGTCTAGCAGAAGAG---GAGGTAGTAATTAGATCTGAAAATTTCACGAACAATGTTAAAACCATAATAGTACATCTGAATAAATCTGTAGTAATTAATTGTACAAGACCCAACAACAATACAAGAAGAGGTATACATATGGGA------------CCAGGGAAA---GCATATTTT---ACAGGAGAAATAATAGGAGATATAAGACAAGCACATTGTAACATT------AGTAGAAAAGAATGGAATGACACTTTAAAACAGATAGTTATAAAATTAGGA---GAACAATTTAGA------------AATAAAACA---ATAGCCTTTAATCAA---------TCCTCAGGAGGGGACATTGAAATTGTAACACACAGTTTTAATTGTGGAGGGGAATTTTTCTACTGTAATTCAACACAACTGTTTAATAGTACTTGGAATAAT---------AATGGTACTAGG------------------------------AATGATACTAGGGAGTCA------------AATGACACT---------------------------ATCATCACACTACCATGCAGAATCAAACAAATTATAAACATGTGGCAGCAAGTAGGCAAAGCAATGTATGCCCCTCCCATCAGAGGACAAATTAGATGTTCATCAAATATCACAGGGCTGCTGTTAACAAGAGATGGTGGT------------------AACGAGAGCGAC---------------------------GCTGAGACCTTCAGACCTGGAGGAGGAGATATGAGGGACAATTGGAGA---AGTGAATTATACAAATATAAAGTAGTAAAAATTGAACCA---TTAGGAGTAGCACCC---TCCAAGGCAAAGAGAAGAGTGGTGCAGAGAGAA---AAAAGAGCAGTG---GGAACAATAGGA---GCTATG---TTCCTT---GGG---------TTCTTGGGA---GCAGCAGGAAGCACTATGGGCGCAGCGTCACTG---ACGCTGACGGTACAGGCCAGACTATTATTGTCTGGTATAGTGCAACAGCAGAACAATCTGCTGAGGGCTATTGAGGCGCAACAACATATTTTGCAACTCACGGTCTGGGGCATTAAACAGCTCCAGGCAAGA---GTCCTGGCTGTGGAAAGATACCTAAGGGATCAACAGCTCCTAGGGATTTGGGGTTGCTCTGGAAAACTCATCTGCACCACTGCTGTGCCTTGGAACGCTAGTTGGAGT---------------------------AATAAATCTCAAGAGGAGATTTGGGAG---AACATGACCTGGATGCAGTGGGATAGAGAAATTAAC------AATTACACAAACCTCATATATACCTTACTTGAAGAATCGCAGAACCAGCAAGAAAAGAATGAACAAGACTTATTAGCATTGGATGAATGGGCAAATTTGTGGAATTGGTTTGCCATATCAAAGTGGCTGTGGTATATAAAAATATTCATAATGATAGTAGGCGGCTTGATAGGTTTAAGAATAGTTTTTGCTGTACTTTCTATAGTGAATAGAGTTAGGCAGGGATACTCACCATTATCGTTTCAGACCCAC---CTCCCAGCCAGGAGGGGA------CCCGACAGGCCCGAAGGAATCGAAGAAGAAGGTGGAGAGAGAGACAGAGACAGATCAGTTCGCTTAGTACATGGATTCTTAGCACTCATCTGGGACGACCTACGGAGCCTGACCCTCTTCAGCTACCACCACTTGAGAGACTTACTCTTGATTATAGCGAGGATTGTGGAAACTCTGGGACGCAGG---------------GGGTGGGAAGCCCTCAAGTATTGGTGG---AATCTCCTGCAATATTGG---------------------------------------------------AGTCAGGAACTAAAGAATAGTGCTGTTAGCTTGCTTGACGCCATAGCTATAGCAGTAGCTGAGGGAACAGATAGGGTTATAAAAGTAGTACGAAGA------------------GCCTTTAGAGCTATTCTCCACATACCTGTAAGAATAAGACAGGGCTTAGAAAGGGCTTTGCTATAA

2.SC22.Trinidad.EU576871 ATGAGAGTGAAGGAGATCAAGAGGAATTGTCAGCGCTTG---------TGGAGATGGGGC------------------------ACCATGCTCCTTGGGATGTTGATGATC------------TGTAGTGCTACA---------CAACAATTGTGGGTTACAGTCTATTATGGGGTACCTGTGTGGAAAGAAGCAACCACCACTCTATTTTGTGCATCAGATGCTAAAGCATATGATACAGAGGCACATAAT---GTCTGGGCCACACATGCCTGTGTACCCACAGACCCCAACCCACAAGAAGTAGTATTG---GGAAATGTGACAGAAAATTTTAACATGTGGAAAAATAACATGGTAGAACAAATGCATGAGGATATAATCAGTTTATGGGATCAAAGCCTAAAGCCATGTGTAAAATTAACCCCACTCTGTGTTACTTTAAACTGCACTGATTATGTGAAAAATGATACTAGTAGCACT---------------------------------------------------------------------------------------------AACAGTAGCAGCTGGGAGAAAGGAGAAATAAAAAACTGCTCTTTCAATATCACC---ACAAGCATAAAAGAT------AAGATGCAGAGGGAATATGCACTTTTTTATAAACTTGATGTAATACCAATAGATAGTGAGAATAATAGTAATATAAATAGTGGGAATAATAGTAATAGTAATTTTAGT------AGCTATAGTAATTTTAGATTGATAAATTGTAATACCTCAGTCATTACACAGGCCTGTCCAAAGGTATCCTTTGAACCAATTCCCATACATTATTGTGCCCCGGCTGGGTTTGCGATTCTAAAGTGT---AATGATAAAAAGTTCAATGGATCAGGACCATGTACAAATGTCAGCACAGTACAATGTACACATGGAATTAGACCAGTAGTGTCAACTCAACTGTTGTTAAATGGCAGTCTAGCAGAAGAG---GAGGTAGTAATTAGATCTGAAAATTTCACGAACAATGTTAAAACCATAATAGTACATCTGAATAAATCTGTAGTAATTAATTGTACAAGACCCAACAACAATACAAGAAGAGGTATACATATGGGA------------CCAGGGAAA---GCATATTTT---ACAGGAGAAATAATAGGAGATATAAGACAAGCACATTGTAACATT------AGTAGAAAAGAATGGAATGACACTTTAAAACAGATAGTTATAAAATTAGGA---GAACAATTTAGA------------AATAAAACA---ATAGCCTTTAATCAA---------TCCTCAGGAGGGGACATTGAAATTGTAACACACAGTTTTAATTGTGGAGGGGAATTTTTCTACTGTAATTCAACACAACTGTTTAATAGTACTTGGAATAAT---------AATGGTACTAGG------------------------------AATGATACTAGGGAGTCA------------AATGACACT---------------------------ATCATCACACTACCATGCAGAATCAAACAAATTATAAACATGTGGCAGCAAGTAGGCAAAGCAATGTATGCCCCTCCCATCAGAGGACAAATTAGATGTTCATCAAATATCACAGGGCTGCTGTTAACAAGAGATGGTGGT------------------AACGAGAGCGAC---------------------------GCTGAGACCTTCAGACCTGGAGGAGGAGATATGAGGGACAATTGGAGA---AGTGAATTATACAAATATAAAGTAGTAAAAATTGAACCA---TTAGGAGTAGCACCC---TCCAAGGCAAAGAGAAGAGTGGTGCAGAGAGAA---AAAAGAGCAGTG---GGAACAATAGGA---GCTATG---TTCCTT---GGG---------TTCTTGGGA---GCAGCAGGAAGCACTATGGGCGCAGCGTCACTG---ACGCTGACGGTACAGGCCAGACTATTATTGTCTGGTATAGTGCAACAGCAGAACAATCTGCTGAGGGCTATTGAGGCGCAACAACATATTTTGCAACTCACGGTCTGGGGCATTAAACAGCTCCAGGCAAGA---GTCCTGGCTGTGGAAAGATACCTAAGGGATCAACAGCTCCTAGGGATTTGGGGTTGCTCTGGAAAACTCATCTGCACCACTGCTGTGCCTTGGAACGCTAGTTGGAGT---------------------------AATAAATCTCAAGAGGAGATTTGGGAG---AACATGACCTGGATGCAGTGGGATAGAGAAATTAAC------AATTACACAAACCTCATATATACCTTACTTGAAGAATCGCAGAACCAGCAAGAAAAGAATGAACAAGACTTATTAGCATTGGATGAATGGGCAAATTTGTGGAATTGGTTTGCCATATCAAAGTGGCTGTGGTATATAAAAATATTCATAATGATAGTAGGCGGCTTGATAGGTTTAAGAATAGTTTTTGCTGTACTTTCTATAGTGAATAGAGTTAGGCAGGGATACTCACCATTATCGTTTCAGACCCAC---CTCCCAGCCAGGAGGGGA------CCCGACAGGCCCGAAGGAATCGAAGAAGAAGGTGGAGAGAGAGACAGAGACAGATCAGTTCGCTTAGTACATGGATTCTTAGCACTCATCTGGGACGACCTACGGAGCCTGACCCTCTTCAGCTACCACCGCTTGAGAGACTTACTCTTGATTATAGCGAGGATTGTGGAAACTCTGGGACGCAGG---------------GGGTGGGAAGCCCTCAAGTATTGGTGG---AATCTCCTGCAATATTGG---------------------------------------------------AGTCAGGAACTAAAGAATAGTGCTGTTAGCTTGCTTGACGCCATAGCTATAGCAGTAGCTGAGGGAACAGATAGGGTTATAAAAGTAGTACGAAGA------------------GCCTTTAGAGCTATTCTCCACATACCTGTAAGAATAAGACAGGGCTTAGAAAGGGCTTTGCTATAA

2.SC22.Trinidad.EU576866 ATGAGAGTGAAGGAGATCAAGAGGAATTGTCAGCGCTTG---------TGGAGATGGGGC------------------------ACCATGCTCCTTGGGATGTTGATGATC------------TGTAGTGCTACA---------CAACAATTGTGGGTTACAGTCTATTATGGGGTACCTGTGTGGAAAGAAGCAACCACCACTCTATTTTGTGCATCAGATGCTAAAGCATATGATACAGAGGCACATAAT---GTCTGGGCCACACATGCCTGTGTACCCACAGACCCCAACCCACAAGAAGTAGTATTG---GGAAATGTGACAGAAAATTTTAACATGTGGAAAAATAACATGGTAGAACAAATGCATGAGGATATAATCAGTTTATGGGATCAAAGCCTAAAGCCATGTGTAAAATTAACCCCACTCTGTGTTACTTTAAACTGCACTGATTATGTGAAAAATGATACTAGTAGCACT---------------------------------------------------------------------------------------------AACAGTAGCAGCTGGGAGAAAGGAGAAATAAAAAACTGCTCTTTCAATATCACC---ACAAGCATAAAAGAT------AAGATGCAGAGGGAATATGCACTTTTTTATAAACTTGATGTAATACCAATAGATAGTGAGAATAATAGTAATATAAATAGTGGGAATAATAGTAATAGTAATTTTAGT------AGCTATAGTAATTTTAGATTGATAAATTGTAATACCTCAGTCATTACACAGGCCTGTCCAAAGGTATCCTTTGAACCAATTCCCATACATTATTGTGCCCCGGCTGGGTTTGCGATTCTAAAGTGT---AATGATAAAAAGTTCAATGGATCAGGACCATGTACAAATGTCAGCACAGTACAATGTACACATGGAATTAGACCAGTAGTGTCAACTCAACTGTTGTTAAATGGCAGTCTAGCAGAAGAG---GAGGTAGTAATTAGATCTGAAAATTTCACGAACAATGTTAAAACCATAATAGTACATCTGAATAAATCTGTAGTAATTAATTGTACAAGACCCAACAACAATACAAGAAGAGGTATACATATGGGA------------CCAGGGAAA---GCATATTTT---ACAGGAGAAATAATAGGAGATATAAGACAAGCACATTGTAACATT------AGTAGAAAAGAATGGAATGACACTTTAAAACAGATAGTTATAAAATTAGGA---GAACAATTTAGA------------AATAAAACA---ATAGCCTTTAATCAA---------TCCTCAGGAGGGGACATTGAAATTGTAACACACAGTTTTAATTGTGGAGGGGAATTTTTCTACTGTAATTCAACACAACTGTTTAATAGTACTTGGAATAAT---------AATGGTACTAGG------------------------------AATGATACTAGGGAGTCA------------AATGACACT---------------------------ATCATCACACTACCATGCAGAATCAAACAAATTATAAACATGTGGCAGCAAGTAGGCAAAGCAATGTATGCCCCTCCCATCAGAGGACAAATTAGATGTTCATCAAATATCACAGGGCTGCTGTTAACAAGAGATGGTGGT------------------AACGAGAGCGAC---------------------------GCTGAGACCTTCAGACCTGGAGGAGGAGATATGAGGGACAATTGGAGA---AGTGAATTATACAAATATAAAGTAGTAAAAATTGAACCA---TTAGGAGTAGCACCC---TCCAAGGCAAAGAGAAGAGTGGTGCAGAGAGAA---AAAAGAGCAGTG---GGAACAATAGGA---GCTATG---TTCCTT---GGG---------TTCTTGGGA---GCAGCAGGAAGCACTATGGGCGCAGCGTCACTG---ACGCTGACGGTACAGGCCAGACTATTATTGTCTGGTATAGTGCAACAGCAGAACAATCTGCTGAGGGCTATTGAGGCGCAACAACATATTTTGCAACTCACGGTCTGGGGCATTAAACAGCTCCAGGCAAGA---GTCCTGGCTGTGGAAAGATACCTAAGGGATCAACAGCTCCTAGGGATTTGGGGTTGCTCTGGAAAACTCATCTGCACCACTGCTGTGCCTTGGAACGCTAGTTGGAGT---------------------------AATAAATCTCAAGAGGAGATTTGGGAG---AACATGACCTGGATGCAGTGGGATAGAGAAATTAAC------AATTACACAAACCTCATATATACCTTACTTGAAGAATCGCAGAACCAGCAAGAAAAGAATGAACAAGACTTATTAGCATTGGATGAATGGGCAAATTTGTGGAATTGGTTTGCCATATCAAAGTGGCTGTGGTATATAAAAATATTCATAATGATAGTAGGCGGCTTGATAGGTTTAAGAATAGTTTTTGCTGTACTTTCTATAGTGAATAGAGTTAGGCAGGGATACTCACCATTATCGTTTCAGACCCAC---CTCCCAGCCAGGAGGGGA------CCCGACAGGCCCGAAGGAATCGAAGAAGAAGGTGGAGAGAGAGACAGAGACAGATCAGTTCGCTTAGTACATGGATTCTTAGCACTCATCTGGGACGACCTACGGAGCCTGACCCTCTTCAGCTACCACCGCTTGAGAGACTTACTCTTGATTATAGCGAGGATTGTGGAAACTCTGGGACGCAGG---------------GGGTGGGAAGCCCTCAAGTATTGGTGG---AATCTCCTGCAATATTGG---------------------------------------------------AGTCAGGAACTAAAGAATAGTGCTGTTAGCTTGCTTGACGCCATAGCTATAGCAGTAGCTGAGGGAACAGATAGGGTTATAAAAGTAGTACGAAGA------------------GCCTTTAGAGCTATTCTCCACATACCTGTAAGAATAAGACAGGGCTTAGAAAGGGCTTTGCTATAA

2.SC22.Trinidad.EU576862 ATGAGAGTGAAGGAGATCAAGAGGAATTGTCAGCGCTTG---------TGGAGATGGGGC------------------------ACCATGCTCCTTGGGATGTTGATGATC------------TGTAGTGCTACA---------CAACAATTGTGGGTTACAGTCTATTATGGGGTACCTGTGTGGAAAGAAGCAACCACCACTCTATTTTGTGCATCAGATGCTAAAGCATATGATACAGAGGCACATAAT---GTCTGGGCCACACATGCCTGTGTACCCACAGACCCCAACCCACAAGAAGTAGTATTG---GGAAATGTGACAGAAAATTTTAACATGTGGAAAAATAACATGGTAGAACAAATGCATGAGGATATAATCAGTTTATGGGATCAAAGCCTAAAGCCATGTGTAAAATTAACCCCACTCTGTGTTACTTTAAACTGCACTGATTATGTGAAAAATGATACTAGTAGCACT---------------------------------------------------------------------------------------------AACAGTAGCAGCTGGGAGAAAGGAGAAATAAAAAACTGCTCTTTCAATATCACC---ACAAGCATAAAAGAT------AAGATGCAGAGGGAATATGCACTTTTTTATAAACTTGATGTAATACCAATAGATAGTGAGAATAATAGTAATATAAATAGTGGGAATAATAGTAATAGTAATTTTAGT------AGCTATAGTAATTTTAGATTGATAAATTGTAATACCTCAGTCATTACACAGGCCTGTCCAAAGGTATCCTTTGAACCAATTCCCATACATTATTGTGCCCCGGCTGGGTTTGCGATTCTAAAGTGT---AATGATAAAAAGTTCAATGGATCAGGACCATGTACAAATGTCAGCACAGTACAATGTACACATGGAATTAGACCAGTAGTGTCAACTCAACTGTTGTTAAATGGCAGTCTAGCAGAAGAG---GAGGTAGTAATTAGATCTGAAAATTTCACGAACAATGTTAAAACCATAATAGTACATCTGAATAAATCTGTAGTAATTAATTGTACAAGACCCAACAACAATACAAGAAGAGGTATACATATGGGA------------CCAGGGAAA---GCATATTTT---ACAGGAGAAATAATAGGAGATATAAGACAAGCACATTGTAACATT------AGTAGAAAAGAATGGAATGACACTTTAAAACAGATAGTTATAAAATTAGGA---GAACAATTTAGA------------AATAAAACA---ATAGCCTTTAATCAA---------TCCTCAGGAGGGGACATTGAAATTGTAACACACAGTTTTAATTGTGGAGGGGAATTTTTCTACTGTAATTCAACACAACTGTTTAATAGTACTTGGAATAAT---------AATGGTACTAGG------------------------------AATGATACTAGGGAGTCA------------AATGACACT---------------------------ATCATCACACTACCATGCAGAATCAAACAAATTATAAACATGTGGCAGCAAGTAGGCAAAGCAATGTATGCCCCTCCCATCAGAGGACAAATTAGATGTTCATCAAATATCACAGGGCTGCTGTTAACAAGAGATGGTGGT------------------AACGAGAGCGAC---------------------------GCTGAGACCTTCAGACCTGGAGGAGGAGATATGAGGGACAATTGGAGA---AGTGAATTATACAAATATAAAGTAGTAAAAATTGAACCA---TTAGGAGTAGCACCC---TCCAAGGCAAAGAGAAGAGTGGTGCAGAGAGAA---AAAAGAGCAGTG---GGAACAATAGGA---GCTATG---TTCCTT---GGG---------TTCTTGGGA---GCAGCAGGAAGCACTATGGGCGCAGCGTCACTG---ACGCTGACGGTACAGGCCAGACTATTATTGTCTGGTATAGTGCAACAGCAGAACAATCTGCTGAGGGCTATTGAGGCGCAACAACATATTTTGCAACTCACGGTCTGGGGCATTAAACAGCTCCAGGCAAGA---GTCCTGGCTGTGGAAAGATACCTAAGGGATCAACAGCTCCTAGGGATTTGGGGTTGCTCTGGAAAACTCATCTGCACCACTGCTGTGCCTTGGAACGCTAGTTGGAGT---------------------------AATAAATCTCAAGAGGAGATTTGGGAG---AACATGACCTGGATGCAGTGGGATAGAGAAATTAAC------AATTACACAAACCTCATATATACCTTACTTGAAGAATCGCAGAACCAGCAAGAAAAGAATGAACAAGACTTATTAGCATTGGATGAATGGGCAAATTTGTGGAATTGGTTTGCCATATCAAAGTGGCTGTGGTATATAAAAATATTCATAATGATAGTAGGCGGCTTGATAGGTTTAAGAATAGTTTTTGCTGTACTTTCTATAGTGAATAGAGTTAGGCAGGGATACTCACCATTATCGTTTCAGACCCAC---CTCCCAGCCAGGAGGGGA------CCCGACAGGCCCGAAGGAATCGAAGAAGAAGGTGGAGAGAGAGACAGAGACAGATCAGTTCGCTTAGTACATGGATTCTTAGCACTCATCTGGGACGACCTACGGAGCCTGACCCTCTTCAGCTACCACCGCTTGAGAGACTTACTCTTGATTATAGCGAGGATTGTGGAAACTCTGGGACGCAGG---------------GGGTGGGAAGCCCTCAAGTATTGGTGG---AATCTCCTGCAATATTGG---------------------------------------------------AGTCAGGAACTAAAGAATAGTGCTGTTAGCTTGCTTGACGCCATAGCTATAGCAGTAGCTGAGGGAACAGATAGGGTTATAAAAGTAGTACGAAGA------------------GCCTTTAGAGCTATTCTCCACATACCTGTAAGAATAAGACAGGGCTTAGAAAGGGCTTTGCTATAA

2.SC22.Trinidad.EU576884 ATGAGAGTGAAGGAGATCAAGAGGAATTGTCAGCGCTTG---------TGGAGATGGGGC------------------------ACCATGCTCCTTGGGATGTTGATGATC------------TGTAGTGCTACA---------CAACAATTGTGGGTTACAGTCTATTATGGGGTACCTGTGTGGAAAGAAGCAACCACCACTCTATTTTGTGCATCAGATGCTAAAGCATATGATACAGAGGCACATAAT---GTCTGGGCCACACATGCCTGTGTACCCACAGACCCCAACCCACAAGAAGTAGTATTG---GGAAATGTGACAGAAAATTTTAACATGTGGAAAAATAACATGGTAGAACAAATGCATGAGGATATAATCAGTTTATGGGATCAAAGCCTAAAGCCATGTGTAAAATTAACCCCACTCTGTGTTACTTTAAACTGCACTGATTATGTGAAAAATGATACTAGTAGCACT---------------------------------------------------------------------------------------------AACAGTAGCAGCTGGGAGAAAGGAGAAATAAAAAACTGCTCTTTCAATATCACC---ACAAGCATAAAAGAT------AAGATGCAGAGGGAATATGCACTTTTTTATAAACTTGATGTAATACCAATAGATAGTGAGAATAATAGTAATATAAATAGTGGGAATAATAGTAATAGTAATTTTAGT------AGCTATAGTAATTTTAGATTGATAAATTGTAATACCTCAGTCATTACACAGGCCTGTCCAAAGGTATCCTTTGAACCAATTCCCATACATTATTGTGCCCCGGCTGGGTTTGCGATTCTAAAGTGT---AATGATAAAAAGTTCAATGGATCAGGACCATGTACAAATGTCAGCACAGTACAATGTACACATGGAATTAGACCAGTAGTGTCAACTCAACTGTTGTTAAATGGCAGTCTAGCAGAAGAG---GAGGTAGTAATTAGATCTGAAAATTTCACGAACAATGTTAAAACCATAATAGTACATCTGAATAAATCTGTAGTAATTAATTGTACAAGACCCAACAACAATACAAGAAGAGGTATACATATGGGA------------CCAGGGAAA---GCATATTTT---ACAGGAGAAATAATAGGAGATATAAGACAAGCACATTGTAACATT------AGTAGAAAAGAATGGAATGACACTTTAAAACAGATAGTTATAAAATTAGGA---GAACAATTTAGA------------AATAAAACA---ATAGCCTTTAATCAA---------TCCTCAGGAGGGGACATTGAAATTGTAACACACAGTTTTAATTGTGGAGGGGAATTTTTCTACTGTAATTCAACACAACTGTTTAATAGTACTTGGAATAAT---------AATGGTACTAGG------------------------------AATGATACTAGGGAGTCA------------AATGACACT---------------------------ATCATCACACTACCATGCAGAATCAAACAAATTATAAACATGTGGCAGCAAGCAGGCAAAGCAATGTATGCCCCTCCCATCAGAGGACAAATTAGATGTTCATCAAATATCACAGGGCTGCTGTTAACAAGAGATGGTGGT------------------AACGAGAGCGAC---------------------------GCTGAGACCTTCAGACCTGGAGGAGGAGATATGAGGGACAATTGGAGA---AGTGAATTATACAAATATAAAGTAGTAAAAATTGAACCA---TTAGGAGTAGCACCC---TCCAAGGCAAAGAGAAGAGTGGTGCAGAGAGAA---AAAAGAGCAGTG---GGAACAATAGGA---GCTATG---TTCCTT---GGG---------TTCTTGGGA---GCAGCAGGAAGCACTATGGGCGCAGCGTCACTG---ACGCTGACGGTACAGGCCAGACTATTATTGTCTGGTATAGTGCAACAGCAGAACAATCTGCTGAGGGCTATTGAGGCGCAACAACATATTTTGCAACTCACGGTCTGGGGCATTAAACAGCTCCAGGCAAGA---GTCCTGGCTGTGGAAAGATACCTAAGGGATCAACAGCTCCTAGGGATTTGGGGTTGCTCTGGAAAACTCATCTGCACCACTGCTGTGCCTTGGAACGCTAGTTGGAGT---------------------------AATAAATCTCAAGAGGAGATTTGGGAG---AACATGACCTGGATGCAGTGGGATAGAGAAATTAAC------AATTACACAAACCTCATATATACCTTACTTGAAGAATCGCAGAACCAGCAAGAAAAGAATGAACAAGACTTATTAGCATTGGATGAATGGGCAAATTTGTGGAATTGGTTTGCCATATCAAAGTGGCTGTGGTATATAAAAATATTCATAATGATAGTAGGCGGCTTGATAGGTTTAAGAATAGTTTTTGCTGTACTTTCTATAGTGAATAGAGTTAGGCAGGGATACTCACCATTATCGTTTCAGACCCAC---CTCCCAGCCAGGAGGGGA------CCCGACAGGCCCGAAGGAATCGAAGAAGAAGGTGGAGAGAGAGACAGAGACAGATCGGTTCGATTAGTACATGGATTCTTAGCACTCATCTGGGACGACCTACGGAGCCTGACCCTCTTCAGCTACCACCGCTTGAGAGACTTACTCTTGATTATAGCGAGGATTGTGGAAACTCTGGGACGCAGG---------------GGGTGGGAAGCCCTCAAGTATTGGTGG---AATCTCCTGCAATATTGG---------------------------------------------------AGTCAGGAACTAAAGAATAGTGCTGTTAGCTTGCTTGACGCCATAGCTATAGCAGTAGCTGAGGGAACAGATAGGGTTATAAAAGTAGTACGAAGA------------------GCCTTTAGAGCTATTCTCCACATACCTGTAAGAATAAGACAGGGCTTAGAAAGGGCTTTGCTATAA

2.SC22.Trinidad.EU576861 ATGAGAGTGAAGGAGATCAAGAGGAATTGTCAGCGCTTG---------TGGAGATGGGGC------------------------ACCATGCTCCTTGGGATGTTGATGATC------------TGTAGTGCTACA---------CAACAATTGTGGGTTACAGTCTATTATGGGGTACCTGTGTGGAAAGAAGCAACCACCACTCTATTTTGTGCATCAGATGCTAAAGCATATGATACAGAGGCACATAAT---GTCTGGGCCACACATGCCTGTGTACCCACAGACCCCAACCCACAAGAAGTAGTATTG---GGAAATGTGACAGAAAATTTTAACATGTGGAAAAATAACATGGTAGAACAAATGCATGAGGATATAATCAGTTTATGGGATCAAAGCCTAAAGCCATGTGTAAAATTAACCCCACTCTGTGTTACTTTAAACTGCACTGATTATGTGAAAAATGATACTAGTAGCACT---------------------------------------------------------------------------------------------AACAGTAGCAGCTGGGAGAAAGGAGAAATAAAAAACTGCTCTTTCAATATCACC---ACAAGCATAAAAGAT------AAGATGCAGAGGGAATATGCACTTTTTTATAAACTTGATGTAATACCAATAGATAGTGAGAATAATAGTAATATAAATAGTGGGAATAATAGTAATAGTAATTTTAGT------AGCTATAGTAATTTTAGATTGATAAATTGTAATACCTCAGTCATTACACAGGCCTGTCCAAAGGTATCCTTTGAACCAATTCCCATACATTATTGTGCCCCGGCTGGGTTTGCGATTCTAAAGTGT---AATGATAAAAAGTTCAATGGATCAGGACCATGTACAAATGTCAGCACAGTACAATGTACACATGGAATTAGACCAGTAGTGTCAACTCAACTGTTGTTAAATGGCAGTCTAGCAGAAGAG---GAGGTAGTAATTAGATCTGAAAATTTCACGAACAATGTTAAAACCATAATAGTACATCTGAATAAATCTGTAGTAATTAATTGTACAAGACCCAACAACAATACAAGAAGAGGTATACATATGGGA------------CCAGGGAAA---GCATATTTT---ACAGGAGAAATAATAGGAGATATAAGACAAGCACATTGTAACATT------AGTAGAAAAGAATGGAATGACACTTTAAAACAGATAGTTATAAAATTAGGA---GAACAATTTAGA------------AATAAAACA---ATAGCCTTTAATCAA---------TCCTCAGGAGGGGACATTGAAATTGTAACACACAGTTTTAATTGTGGAGGGGAATTTTTCTACTGTAATTCAACACAACTGTTTAATAGTACTTGGAATAAT---------AATGGTACTAGG------------------------------AATGATACTAGGGAGTCA------------AATGACACT---------------------------ATCATCACACTACCATGCAGAATCAAACAAATTATAAACATGTGGCAGCAAGTAGGCAAAGCAATGTATGCCCCTCCCATCAGAGGACAAATTAGATGTTCATCAAATATCACAGGGCTGCTGTTAACAAGAGATGGTGGT------------------AACGAGAGCGAC---------------------------GCTGAGACCTTCAGACCTGGAGGAGGAGATATGAGGGACAATTGGAGA---AGTGAATTATACAAATATAAAGTAGTAAAAATTGAACCA---TTAGGAGTAGCACCC---TCCAAGGCAAAGAGAAGAGTGGTGCAGAGAGAA---AAAAGAGCAGTG---GGAACAATAGGA---GCTATG---TTCCTT---GGG---------TTCTTGGGA---GCAGCAGGAAGCACTATGGGCGCAGCGTCACTG---ACGCTGACGGTACAGGCCAGACTATTATTGTCTGGTATAGTGCAACAGCAGAACAATCTGCTGAGGGCTATTGAGGCGCAACAACATATTTTGCAACTCACGGTCTGGGGCATTAAACAGCTCCAGGCAAGA---GTCCTGGCTGTGGAAAGATACCTAAGGGATCAACAGCTCCTAGGGATTTGGGGTTGCTCTGGAAAACTCATCTGCACCACTGCTGTGCCTTGGAACGCTAGTTGGAGT---------------------------AATAAATCTCAAGAGGAGATTTGGGAG---AACATGACCTGGATGCAGTGGGATAGAGAAATTAAC------AATTACACAAACCTCATATATACCTTACTTGAAGAATCGCAGAACCAGCAAGAAAAGAATGAACAAGACTTATTAGCATTGGATGAATGGGCAAATTTGTGGAATTGGTTTGCCATATCAAAGTGGCTGTGGTATATAAAAATATTCATAATGATAGTAGGCGGCTTGATAGGTTTAAAAATAGTTTTTGCTGTACTTTCTATAGTGAATAGAGTTAGGCAGGGATACTCACCATTATCGTTTCAGACCCAC---CTCCCAGCCAGGAGGGGA------CCCGACAGGCCCGAAGGAATCGAAGAAGAAGGTGGAGAGAGAGACAGAGACAGATCAGTTCGCTTAGTACATGGATTCTTAGCACTCATCTGGGACGACCTACGGAGCCTGACCCTCTTCAGCTACCACCGCTTGAGAGACTTACTCTTGATTATAGCGAGGATTGTGGAAACTCTGGGACGCAGG---------------GGGTGGGAAGCCCTCAAGTATTGGTGG---AATCTCCTGCAATATTGG---------------------------------------------------AGTCAGGAACTAAAGAATAGTGCTGTTAGCTTGCTTGACGCCATAGCTATAGCAGTAGCTGAGGGAACAGATAGGGTTATAAAAGTAGTACGAAGA------------------GCCTTTAGAGCTATTCTCCACATACCTGTAAGAATAAGACAGGGCTTAGAAAGGGCTTTGCTATAA

2.SC33.Trinidad.EU576941 ATGAGAGTGAAGGGGATCAGGAGGAATTGGCAGGGCTTG---------TGGAGATGGGGC------------------------ACGATGCTCCTTGGAATGTTGATGATC------------TGTAGGGCTGCA---------GAAAACTTGTGGGTCACAGTCTATTATGGGGTACCTGTGTGGAAAGAAGCAACCACCACTTTATTTTGTGCATCAGATGCTAAAGCATATGAGACAGAGAAACATAAT---GTCTGGGCCACACATGCCTGTGTACCCACAGACCCCAACCCACAAGAAGTAGTATTG---GGAAATGTGACAGAAAATTTTAACATGTGGAAAAATAACATGGTAGAACAGATGCATGAGGATATAATCAGTTTATGGGATGAAAGCCTAAAGCCATGTGTAAAATTAACCCCACTCTGTGTTACTTTGAACTGCACTGATGAAGTGAAGACTAGTTATGCTAATAAGACTTCT---------------------------------------------------------AATGAGACTTATAAGACTTCTAATGAGACTTTTGGAGAA------------------ATAAAAAACTGCTCTTTCAGTGTCCCC---ACAGGCATAAAAGAT------AAGGTGCAGAATGTATATGCACTTTTTTATAAACTTGATGTAATACCAATAGATGATAATAAT---------------------------AATAGTAGCAAGAATAATAACGGTAGCTATAGTAGCTATAGATTGATAAATTGTAATACCTCAGTCATTACACAGGCCTGTCCAAAGGTGTCCTTTGAACCAATTCCCATACATTATTGTGCCCCGGCTGGTTTTGCAATTCTAAAATGT---AATAATAAGACATTCAATGGAACAGGACCATGTACAAATGTCAGCACAGTACAATGTACACATGGGATTAGACCAGTAGTGTCAACTCAACTGCTGTTAAATGGCAGTCTAGCAGAAGAA---GAGGTAGTAATTAGATCTGAAAATTTCACGAACAATGCTAAAACCATAATAGTACATCTGAAGAAATCTGTAGAAATTAATTGTACAAGGCCCGGCAACAATACAAGAAAAAGCATACATATAGGA------------CCAGGGAGA---GCATTTTATGCTACAGGAGACATAATAGGAGATATAAGACAAGCACATTGTAACCTT------AGTAGTGTACAATGGAATGACACTTTAAAACAGATAGTTATAAAATTAGGA---GAACAATTTGGG---------ACTAATAAAACA---ATAGCCTTTAATCAA---------TCCTCAGGAGGGGACCCAGAAATTGTAATGCACAGTTTTAATTGTGGAGGAGAATTTTTCTACTGTAATACAACACAACTATTTAATAGTACTTGGGAGTTT---------CATGGTAATTGGACAAGG------------------------TCAAATTTCACAGAATCA------------AATAGCACAACT---------------------------ATCACACTCCCATGTAGAATAAAACAAATTGTAAACATGTGGCAGGAAGTAGGCAAAGCAATGTATGCCCCTCCCATCAGAGGACAAATTAGATGTTCATCAAATATCACGGGGCTGCTATTAACAAGAGATGGTGGCGTG---------------AACGGGACCCGC------------------------------GAGACCTTTAGACCTGGAGGAGGAGATATGAGGGACAATTGGAGA---AGTGAATTATATAAATATAAAGTGGTAAAAATTGAGCCA---TTAGGAGTAGCACCC---ACCAAGGCAAAGAGAAGAGTGGTGCAGAGAGAA---AAAAGAGCAGTG---GGAACAATAGGA---GCTATG---TTCCTT---GGG---------TTCTTGGGA---GCAGCAGGAAGCACTATGGGCGCAGCGTCAATA---ACGCTGACGGTACAGGCCAGACAATTATTGTCTGGTATAGTGCAACAGCAAAACAATTTGCTGAGAGCTATTGAGGCGCAACAGCATATGTTGCAACTCACGGTCTGGGGCATCAAACAGCTCCAAGCAAGA---GTCCTGGCTGTGGAAAGATACCTAAGGGACCAACAGCTCCTAGGGATTTGGGGTTGCTCTGGAAAACTCATTTGCACCACTGCTGTGCCTTGGAATGCTAGTTGGAGT---------------------------AATAAATCTCAAGATTACATTTGGAAT---AACATGACCTGGATGCAGTGGGATAAAGAGATTAAC------AATTACACAAACTTAATATACTCCTTACTTGAAGACTCGCAAAACCAGCAAGAAAAGAATGAACATGAATTATTAGAATTGGACAAATGGGCAAGTTTGTGGAATTGGTTTGACATAACAAGATGGCTGTGGTATATAAAAATATTCATAATGATAGTAGGAGGCTTGATAGGTTTAAGAATAGTTATTGCTGTAGTTTCCATAGTGAATAGGGTTAGGCAGGGATACTCACCAATATCATTACAGACCCAC---TTCCCAGCACCGCGGGGA------CCCGACAGGCCCGAAGGAATCGAAGAAGGAGGTGGAGACAGAGACAGAGACAGATCACTGCGATTAGTGCATGGATCCTTAGCACTCATCTGGGACGATCTGAGGAGCCTGTGCATCTTCAGCTACCACCGCTTGAGAGACTTACTCTTGATTGTAGCGAGGATTGTGGAAATTCTGGGACGCAGG---------------GGGTGGGAAGCCCTCAAGTATTGGTGG---AATCTCCTGCAGTATTGG---------------------------------------------------AGTCAGGAACTGAAGAATAGTGCTGTTAGCTTGCTTGATGCCACAGCTATTGCAGTAGCTGAGGGGACAGATAGGATTATAGAAATAATACGAAGA------------------GCTTTTAGAGCTATCCTCCACATACCTACAAGAATAAGACAGGGCTTGGAAAGGGCTTTGCTATAA

2.SC33.Trinidad.EU576948 ATGAGAGTGAAGGGGATCAGGAGGAATTGGCAGGGCTTG---------TGGAGATGGGGC------------------------ACGATGCTCCTTGGAATGTTGATGATC------------TGTAGGGCTGCA---------GAAAACTTGTGGGTCACAGTCTATTATGGGGTACCTGTGTGGAAAGAAGCAACCACCACTTTATTTTGTGCATCAGATGCTAAAGCATATGAGACAGAGAAACATAAT---GTCTGGGCCACACATGCCTGTGTACCCACAGACCCCAACCCACAAGAAGTAGTATTG---GGAAATGTGACAGAAAATTTTAACATGTGGAAAAATAACATGGTAGAACAGATGCATGAGGATATAATCAGTTTATGGGATGAAAGCCTAAAGCCATGTGTAAAATTAACCCCACTCTGTGTTACTTTGAACTGCACTGATGAAGTGAAGACTAGTTATGCTAATAAGACTTCT---------------------------------------------------------AATGAGACTTATAAGACTTCTAATGAGACTTTTGGAGAA------------------ATAAAAAACTGCTCTTTCAGTGTCCCC---ACAGGCATAAAAGAT------AAGGTGCAGAATGTATATGCACTTTTTTATAAACTTGATGTAATACCAATAGATGATAATAAT---------------------------AATAGTAGCAAGAATAATAACGGTAGCTATAGTAGCTATAGATTGATAAATTGTAATACCTCAGTCATTACACAGGCCTGTCCAAAGGTGTCCTTTGAACCAATTCCCATACATTATTGTGCCCCGGCTGGTTTTGCAATTCTAAAATGT---AATAATAAGACATTCAATGGAACAGGACCATGTACAAATGTCAGCACAGTACAATGTACACATGGGATTAGACCAGTAGTGTCAACTCAACTGCTGTTAAATGGCAGTCTAGCAGAAGAA---GAGGTAGTAATTAGATCTGAAAATTTCACGAACAATGCTAAAACCATAATAGTACATCTGAAGAAATCTGTAGAAATTAATTGTACAAGGCCCGGCAACAATACAAGAAAAAGCATACATATAGGA------------CCAGGGAGA---GCATTTTATGCTACAGGAGACATAATAGGAGATATAAGACAAGCACATTGTAACCTT------AGTAGTGTACAATGGAATGACACTTTAAAACAGATAGTTATAAAATTAGGA---GAACAATTTGGG---------ACTAATAAAACA---ATAGCCTTTAATCAA---------TCCTCAGGAGGGGACCCAGAAATTGTAATGCACAGTTTTAATTGTGGAGGAGAATTTTTCTACTGTAATACAACACAACTATTTAATAGTACTTGGGAGTTT---------CATGGTAATTGGACAAGG------------------------TCAAATTTCACAGAATCA------------AATAGCACAACT---------------------------ATCACACTCCCATGTAGAATAAAACAAATTGTAAACATGTGGCAGGAAGTAGGCAAAGCAATGTATGCCCCTCCCATCAGAGGACAAATTAGATGTTCATCAAATATCACGGGGCTGCTATTAACAAGAGATGGTGGCGTG---------------AACGGGACCCGC------------------------------GAGACCTTTAGACCTGGAGGAGGAGATATGAGGGACAATTGGAGA---AGTGAATTATATAAATATAAAGTGGTAAAAATTGAGCCA---TTAGGAGTAGCACCC---ACCAAGGCAAAGAGAAGAGTGGTGCAGAGAGAA---AAAAGAGCAGTG---GGAACAATAGGA---GCTATG---TTCCTT---GGG---------TTCTTGGGA---GCAGCAGGAAGCACTATGGGCGCAGCGTCAATA---ACGCTGACGGTACAGGCCAGACAATTATTGTCTGGTATAGTGCAACAGCAAAACAATTTGCTGAGAGCTATTGAGGCGCAACAGCATATGTTGCAACTCACGGTCTGGGGCATCAAACAGCTCCAAGCAAGA---GTCCTGGCTGTGGAAAGATACCTAAGGGACCAACAGCTCCTAGGGATTTGGGGTTGCTCTGGAAAACTCATTTGCACCACTGCTGTGCCTTGGAATGCTAGTTGGAGT---------------------------AATAAATCTCAAGATTACATTTGGAAT---AACATGACCTGGATGCAGTGGGATAAAGAGATTAAC------AATTACACAAACTTAATATACTCCTTACTTGAAGACTCGCAAAACCAGCAAGAAAAGAATGAACATGAATTATTAGAATTGGACAAATGGGCAAGTTTGTGGAATTGGTTTGACATAACAAGATGGCTGTGGTATATAAAAATATTCATAATGATAGTAGGAGGCTTGATAGGTTTAAGAATAGTTATTGCTGTAGTTTCCATAGTGAATAGGGTTAGGCAGGGATACTCACCAATATCATTACAGACCCAC---TTCCCAGCACCGCGGGGA------CCCGACAGGCCCGAAGGAATCGAAGAAGGAGGTGGAGACAGAGACAGAGACAGATCACTGCGATTAGTGCATGGATCCTTAGCACTCATCTGGGACGATCTGAGGAGCCTGTGCATCTTCAGCTACCACCGCTTGAGAGACTTACTCTTGATTGTAGCGAGGATTGTGGAAATTCTGGGACGCAGG---------------GGGTGGGAAGCCCTCAAGTATTGGTGG---AATCTCCTGCAGTATTGG---------------------------------------------------AGTCAGGAACTGAAGAATAGTGCTGTTAGCTTGCTTGATGCCACAGCTATTGCAGTAGCTGAGGGGACAGATAGGATTATAGAAATAATACGAAGA------------------GCTTTTAGAGCTATCCTCCACATACCTACAAGAATAAGACAGGGCTTGGAAAGGGCTTTGCTATAA

2.SC33.Trinidad.EU576943 ATGAGAGTGAAGGGGATCAGGAGGAATTGGCAGGGCTTG---------TGGAGATGGGGC------------------------ACGATGCTCCTTGGAATGTTGATGATC------------TGTAGGGCTGCA---------GAAAACTTGTGGGTCACAGTCTATTATGGGGTACCTGTGTGGAAAGAAGCAACCACCACTTTATTTTGTGCATCAGATGCTAAAGCATATGAGACAGAGAAACATAAT---GTCTGGGCCACACATGCCTGTGTACCCACAGACCCCAACCCACAAGAAGTAGTATTG---GGAAATGTGACAGAAAATTTTAACATGTGGAAAAATAACATGGTAGAACAGATGCATGAGGATATAATCAGTTTATGGGATGAAAGCCTAAAGCCATGTGTAAAATTAACCCCACTCTGTGTTACTTTGAACTGCACTGATGAAGTGAAGACTAGTTATGCTAATAAGACTTCT---------------------------------------------------------AATGAGACTTATAAGACTTCTAATGAGACTTTTGGAGAA------------------ATAAAAAACTGCTCTTTCAGTGTCCCC---ACAGGCATAAAAGAT------AAGGTGCAGAATGTATATGCACTTTTTTATAAACTTGATGTAATACCAATAGATGATAATAAT---------------------------AATAGTAGCAAGAATAATAACGGTAGCTATAGTAGCTATAGATTGATAAATTGTAATACCTCAGTCATTACACAGGCCTGTCCAAAGGTGTCCTTTGAACCAATTCCCATACATTATTGTGCCCCGGCTGGTTTTGCAATTCTAAAATGT---AATAATAAGACATTCAATGGAACAGGACCATGTACAAATGTCAGCACAGTACAATGTACACATGGGATTAGACCAGTAGTGTCAACTCAACTGCTGTTAAATGGCAGTCTAGCAGAAGAA---GAGGTAGTAATTAGATCTGAAAATTTCACGAACAATGCTAAAACCATAATAGTACATCTGAAGAAATCTGTAGAAATTAATTGTACAAGGCCCGGCAACAATACAAGAAAAAGCATACATATAGGA------------CCAGGGAGA---GCATTTTATGCTACAGGAGACATAATAGGAGATATAAGACAAGCACATTGTAACCTT------AGTAGTGTACAATGGAATGACACTTTAAAACAGATAGTTATAAAATTAGGA---GAACAATTTGGG---------ACTAATAAAACA---ATAGCCTTTAATCAA---------TCCTCAGGAGGGGACCCAGAAATTGTAATGCACAGTTTTAATTGTGGAGGAGAATTTTTCTACTGTAATACAACACAACTATTTAATAGTACTTGGGAGTTT---------CATGGTAATTGGACAAGG------------------------TCAAATTTCACAGAATCA------------AATAGCACAACT---------------------------ATCACACTCCCATGTAGAATAAAACAAATTGTAAACATGTGGCAGGAAGTAGGCAAAGCAATGTATGCCCCTCCCATCAGAGGACAAATTAGATGTTCATCAAATATCACGGGGCTGCTATTAACAAGAGATGGTGGCGTG---------------AACGGGACCCGC------------------------------GAGACCTTTAGACCTGGAGGAGGAGATATGAGGGACAATTGGAGA---AGTGAATTATATAAATATAAAGTGGTAAAAATTGAGCCA---TTAGGAGTAGCACCC---ACCAAGGCAAAGAGAAGAGTGGTGCAGAGAGAA---AAAAGAGCAGTG---GGAACAATAGGA---GCTATG---TTCCTT---GGG---------TTCTTGGGA---GCAGCAGGAAGCACTATGGGCGCAGCGTCAATA---ACGCTGACGGTACAGGCCAGACAATTATTGTCTGGTATAGTGCAACAGCAAAACAATTTGCTGAGAGCTATTGAGGCGCAACAGCATATGTTGCAACTCACGGTCTGGGGCATCAAACAGCTCCAAGCAAGA---GTCCTGGCTGTGGAAAGATACCTAAGGGACCAACAGCTCCTAGGGATTTGGGGTTGCTCTGGAAAACTCATTTGCACCACTGCTGTGCCTTGGAATGCTAGTTGGAGT---------------------------AATAAATCTCAAGATTACATTTGGAAT---AACATGACCTGGATGCAGTGGGATAAAGAGATTAAC------AATTACACAAACTTAATATACTCCTTACTTGAAGACTCGCAAAACCAGCAAGAAAAGAATGAACATGAATTATTAGAATTGGACAAATGGGCAAGTTTGTGGAATTGGTTTGACATAACAAGATGGCTGTGGTATATAAAAATATTCATAATGATAGTAGGAGGCTTGATAGGTTTAAGAATAGTTATTGCTGTAGTTTCCATAGTGAATAGGGTTAGGCAGGGATACTCACCAATATCATTACAGACCCAC---TTCCCAGCACCGCGGGGA------CCCGACAGGCCCGAAGGAATCGAAGAAGGAGGTGGAGACAGAGACAGAGACAGATCACTGCGATTAGTGCATGGATCCTTAGCACTCATCTGGGACGATCTGAGGAGCCTGTGCATCTTCAGCTACCACCGCTTGAGAGACTTACTCTTGATTGTAGCGAGGATTGTGGAAATTCTGGGACGCAGG---------------GGGTGGGAAGCCCTCAAGTATTGGTGG---AATCTCCTGCAGTATTGG---------------------------------------------------AGTCAGGAACTGAAGAATAGTGCTGTTAGCTTGCTTGATGCCACAGCTATTGCAGTAGCTGAGGGGACAGATAGGATTATAGAAATAATACGAAGA------------------GCTTTTAGAGCTATCCTCCACATACCTACAAGAATAAGACAGGGCTTGGAAAGGGCTTTGCTATAA
[truncated: 6,622,274 more chars]
