# Supplementary material for: Recurrent Signature Patterns in HIV-1 B Clade Envelope Glycoproteins Associated with either Early or Chronic Infections
Source: PLoS Pathog. 2011 Sep 29;7(9):e1002209. doi: 10.1371/journal.ppat.1002209 (PMC3182927; doi:10.1371/journal.ppat.1002209)
Supplement: Table S6 — All sequences generated for this study under Holdout Set , aligned. The first character of each sequence name indicates either the Fiebig stage at time of sampling, or C for chronic infection (see material and methods for details). The GenBank numbers are all included in the name of each sequence in the files; as there are thousands of sequences, and the numbers are not continuous, this seemed the most parsimonious presentation. (DOC) [file ppat.1002209.s013.doc]

2.04013226.ADARC.GU330363 ATGAAAGTGAAGGTGATCAGGAAGAATTGTCAGCACTTG---------TGGACATGGGGC------------------------ACGATGCTCCTTGGGATGTTAATGATC------------TGTAGTGCTGAA---------GATCAATTGTGGGTCACAGTTTATTATGGGGTACCTGTGTGGAAAGAAGCAACCACCACTCTATTTTGTGCATCAGATGCCAAAGGATATGATACAGAGGTACATAAT---GTTTGGGCCACACATGCCTGTGTACCCACAGACCCCAACCCACAAGAAGTGTTATTG---GCAAATGTGACAGAAAATTTTAACATGTGGAAAAATAACATGGTAGACCAGATGCATGAGGATGTAATCAGTTTATGGGATCAAAGTCTAAAGCCATGTGTAAAATTGACACCACTCTGTGTTACTTTATCTTGCACTAATGTGACTAATAATAATAATACTGCCAGT------------------------------------------------------------------------------------AATAGTTCTGATTGGGAAAAGATGGAG---GGAGAAATAAAAAATTGCTCTTTCAATGTCACC---CCAAGCATAAGAGAT------AGGGTGCGCAAAGAATATGCACTCTTTTATAGCCTTGATGTAGTACCTATAAAGGATACT------------------------------AATGATAGT---------------------AGAACCTATAGATTAATAAATTGTAACACCTCAGTCATTACACAGGCCTGTCCAAAGGTATCCTTTGAGCCAATTCCCATACATTATTGTGCCCCGGCTGGTTATGCGATTCTAAAATGT---AATAATAAGACTTTCAATGGAACAGGACCATGTACAAATGTCAGCACAGTACAATGTACACATGGAATTAGGCCTGTAGTATCAACTCAACTGTTGTTAAATGGCAGCCTAGCAGAAGAA---GACATAGTAATCAGATCTGAAAATCTCACAGACAATGCTAAAACCATAATAGTACATCTGAATGAATCTGTAGAAATTAATTGTACAAGACCCAACAATAATACAAGAAAAAGTATAAATATAGGA------------CCAGGGGCA---GCGATGTATGCAACAGGAGCCATAATAGGAGATATAAGACAAGCACATTGTAACATT------AGTGGAGCAAGATGGAATGACACTTTAAAAAAGGTAGTCAAAAAATTAAGA---GAAAAATTTGGA------------AATAAAACA---ATAATCTTTGATCAA---------CACTCAGGAGGGGACCCAGAAATTGTAATGCACAGTTTTAATTGTGGAGGGGAATTTTTCTACTGTAATACAACAAAACTATTCAATAGTACTTGG---------------AATAGTACTAGG------------------------------AATGATACTGAAAGGAAT---------AGTAATGAAACT------------------------GACACAATCACACTCCCATGCAGAATAAAACAAATTATAAACATGTGGCAGGGAGTAGGAAAAGCAATGTATGCCCCTCCTATCAGAGGACTAATTAGATGTTCATCAAATATTACAGGGCTGTTATTAACAAGGGATGGTGGTAAGACT------------AACAGGAGC---------------GGGAGCAACGGGTCT---GAGACCTTCAGACCTGGAGGAGGAAATATGAAGGACAATTGGAGA---AGTGAATTATATAAATATAAAGTAGTAAAACTTGAACCA---TTAGGAATAGCACCC---ACCAAGGCAAGGAGAAGAGTGGTGCAGAGAGAA---AAAAGAGCAGTA---GGA---CTAGGA---GCGTTG---TTCATT---GGG---------TTCTTGGGA---GCAGCAGGAAGCACTATGGGCGCAGCGTCAGTA---ACGCTGACGGTACAGGCCAGACAATTATTGTCTGGTATAGTGCAACAGCAGAACAATCTGCTGATGGCTATTGATGCACAACAGCATCTGTTGCAACTCACAGTCTGGGGCGTCAAGCAGCTCCGGGCAAGA---ATCCTGGCTGTGGAAAGATACCTAAAGGATCAACAGCTCCTAGGGATTTGGGGTTGCTCTGGAAAACTCATCTGCACCACTAATGTGCCTTGGAATGCTAGTTGGAGT---------------------------AATAAATCTCAAGAGCAAATTTGGGAA---AACATGACCTGGATGCAGTGGGAAAGAGAAATTGAC------AATTACACAGGCCTTATATACTCTTTAATTGAAAAATCGCAAAACCAACAAGAAAAGAATGAACAAGAATTATTGGCATTGGATAAGTGGGCAAGTTTGTGGAATTGGTTTGACATAACAAAATGGCTGTGGTATATAAAAATATTCATAATAATAGTAGGAGGCTTGATAGGATTAAGAATAGTTTTTGCTGTATTTTCTATAGTAAATAGAGTTAGGCAGGGATACTCACCACTATCGTTTCAGACCCAC---CTCCCAGTTCCGAGGGGA------CCCGACAGGCCCGAAGGAATCGACGAAGAAGGTGGAGAGCAAGGCAGAGACAGATCAATTCGATTAGTGGATGGATTCTTGGCACTTTTCTGGGACGACCTGAGGAGCCTGTGTCTTTTCAGCTACCACCACTTGAGAGACTTACTCTTGATTGCAGCGCGGATTGTGCAACTTCTGGGACAGAGG---------------GGGTGGGAGATCCTCAAATATTGGTGG---AATCTCCTGCTGTATTGG---------------------------------------------------AGTCAGGAACTAAAGAATAGTGCTGTCAGCTTGCTCAACACCACTGCTATAGTAGTAGCTGAGGGGACAGACAGGGTTATAGAAGCATTGCAAAGA------------------GCCTTTAGGGCTATTCTCCACATCCCTACAAGAATAAGACAAGGCTTGGAAAGGGCTTTACTATAA

2.04013226.ADARC.GU330364 ATGAAAGTGAAGGTGATCAGGAAGAATTGTCAGCACTTG---------TGGACATGGGGC------------------------ACGATGCTCCTTGGGATGTTAATGATC------------TGTAGTGCTGAA---------GATCAATTGTGGGTCACAGTTTATTATGGGGTACCTGTGTGGAAAGAAGCAACCACCACTCTATTTTGTGCATCAGATGCCAAAGGATATGATACAGAGGTACATAAT---GTTTGGGCCACACATGCCTGTGTACCCACAGACCCCAACCCACAAGAAGTGTTATTG---GCAAATGTGACAGAAAATTTTAACATGTGGAAAAATAACATGGTAGACCAGATGCATGAGGATGTAATCAGTTTATGGGATCAAAGTCTAAAGCCATGTGTAAAATTGACACCACTCTGTGTTACTTTATCTTGCACTAATGTGACTAATAATAATAATACTGCCAGT------------------------------------------------------------------------------------AATAGTTCTGATTGGGAAAAGATGGAG---GGAGAAATAAAAAACTGCTCTTTCAATGTCACC---CCAAGCATAAGAGAT------AGGGTGCGCAAAGAATATGCACTCTTTTATAGCCTTGATGTAGTACCTATAAAGGATACT------------------------------AATGATAGT---------------------AGAACCTATAGATTAATAAATTGTAACACCTCAGTCATTACACAGGCCTGTCCAAAGGTATCCTTTGAGCCAATTCCCATACATTATTGTGCCCCGGCTGGTTATGCGATTCTAAAATGT---AATAATAAGACTTTCAATGGAACAGGACCATGTACAAATGTCAGCACAGTACAATGTACACATGGAATTAGGCCTGTAGTATCAACTCAACTGTTGTTAAATGGCAGCCTAGCAGAAGAA---GACATAGTAATCAGATCTGAAAATCTCACAGACAATGCTAGAACCATAATAGTACATCTGAATGAATCTGTAGAAATTAATTGTACAAGACCCAACAATAATACAAGAAAAAGTATAAATATAGGA------------CCAGGGGCA---GCGATGTATGCAACAGGAGCCATAATAGGAGATATAAGACAAGCACATTGTAACATT------AGTGGAGCAAGATGGAATGACACTTTAAAAAAGGTAGTCAAAAAATTAAGA---GAAAAATTTGGA------------AATAAAACA---ATAATCTTTGATCAA---------CACTCAGGAGGGGACCCAGAAATTGTAATGCACAGTTTTAATTGTGGAGGGGAATTTTTCTACTGTAATACAACAAAACTATTCAATAGTACTTGG---------------AATAGTACTAGG------------------------------AATGATACTGAAAGGAAT---------AGTAATGAAACT------------------------GACACAATCACACTCCCATGCAGAATAAAACAAATTATAAACATGTGGCAGGGAGTAGGAAAAGCAATGTATGCCCCTCCTATCAGAGGACTAATTAGATGTTCATCAAATATTACAGGGCTGTTATTAACAAGGGATGGTGGTAATACT------------AACAGGAGC---------------GGGAGCAACGGGTCT---GAGACCTTCAGACCTGGAGGAGGAAATATGAAGGACAATTGGAGA---AGTGAATTATATAAATATAAAGTAGTAAAACTTGAACCA---TTAGGAATAGCACCC---ACCAAGGCAAGGAGAAGAGTGGTGCAGAGAGAA---AAAAGAGCAGTA---GGA---CTAGGA---GCGTTG---TTCATT---GGG---------TTCTTGGGA---GCAGCAGGAAGCACTATGGGCGCAGCGTCAGTA---ACGCTGACGGTACAGGCCAGACAATTATTGTCTGGTATAGTGCAACAGCAGAACAATCTGCTGATGGCTATTGATGCACAACAGCATCTGTTGCAACTCACAGTCTGGGGCGTCAAGCAGCTCCGGGCAAGA---ATCCTGGCTGTGGAAAGATACCTAAAGGATCAACAGCTCCTAGGGATTTGGGGTTGCTCTGGAAAACTCATCTGCACCACTAATGTGCCTTGGAATGCTAGTTGGAGT---------------------------AATAAATCTCAAGAGCAAATTTGGGAA---AACATGACCTGGATGCAGTGGGAAAGAGAAATTGAC------AATTACACAGGCCTTATATACTCTTTAATTGAAAAATCGCAAAACCAACAAGAAAAGAATGAACAAGAATTATTGGCATTGGATAAGTGGGCAAGTTTGTGGAATTGGTTTGACATAACAAAATGGCTGTGGTATATAAAAATATTCATAATAATAGTAGGAGGCTTGATAGGATTAAGAATAGTTTTTGCTGTATTTTCTATAGTAAATAGAGTTAGGCAGGGATACTCACCACTATCGTTTCAGACCCAC---CTCCCAGTTCCGAGGGGA------CCCGACAGGCCCGAAGGAATCGACGAAGAAGGTGGAGAGCAAGGCAGAGACAGATCAATTCGATTAGTGGATGGATTCTTGGCACTTTTCTGGGACGACCTGAGGAGCCTGTGTCTTTTCAGCTACCACCACTTGAGAGACTTACTCTTGATTGCAGCGCGGATTGTGCAACTTCTGGGACAGAGG---------------GGGTGGGAGATCCTCAAATATTGGTGG---AATCTCCTGCTGTATTGG---------------------------------------------------AGTCAGGAACTAAAGAATAGTGCTGTCAGCTTGCTCAACACCACTGCTATAGTAGTAGCTGAGGGGACAGACAGGGTTATAGAAGCATTGCAAAGA------------------GCCTTTAGGGCTATTCTCCACATCCCTACAAGAATAAGACAAGGCTTGGAAAGGGCTTTACTATAA

2.04013226.ADARC.GU330365 ATGAAAGTGAAGGTGATCAGGAAGAATTGTCAGCACTTG---------TGGACATGGGGC------------------------ACGATGCTCCTTGGGATGTTAATGATC------------TGTAGTGCTGAA---------GATCAATTGTGGGTCACAGTTTATTATGGGGTACCTGTGTGGAAAGAAGCAACCACCACTCTATTTTGTGCATCAGATGCCAAAGGATATGATACAGAGGTACATAAT---GTTTGGGCCACACATGCCTGTGTACCCACAGACCCCAACCCACAAGAAGTGTTATTG---GCAAATGTGACAGAAAATTTTAACATGTGGAAAAATAACATGGTAGACCAGATGCATGAGGATGTAATCAGTTTATGGGATCAAAGTCTAAAGCCATGTGTAAAATTGACACCACTCTGTGTTACTTTATCTTGCACTAATGTGACTAATAATAATAATACTGCCAGT------------------------------------------------------------------------------------AATAGTTCTGATTGGGAAAAGATGGAG---GGAGAAATAAAAAACTGCTCTTTCAATGTCACC---CCAAGCATAAGAGAT------AGGGTGCGCAAAGAATATGCACTCTTTTATAGCCTTGATGTAGTACCTATAAAGGATACT------------------------------AATGATAGT---------------------AGAACCTATAGATTAATAAATTGTAACACCTCAGTCATTACACAGGCCTGTCCAAAGGTATCCTTTGAGCCAATTCCCATACATTATTGTGCCCCGGCTGGTTATGCGATTCTAAAATGT---AATAATAAGACTTTCAATGGAACAGGACCATGTACAAATGTCAGCACAGTACAATGTACACATGGAATTAGGCCTGTAGTATCAACTCAACTGTTGTTAAATGGCAGCCTAGCAGAAGAA---GACATAGTAATCAGATCTGAAAATCTCACAGACAATGCTAAAACCATAATAGTACATCTGAATGAATCTGTAGAAATTAATTGTACAAGACCCAACAATAATACAAGAAAAAGTATAAATATAGGA------------CCAGGGGCA---GCGATGTATGCAACAGGAGCCATAATAGGAGATATAAGACAAGCACATTGTAACATT------AGTGGAGCAAGATGGAATGACACTTTAAAAAAGGTAGTCAAAAAATTAAGA---GAAAAATTTGGA------------AATAAAACA---ATAATCTTTGATCAA---------CACTCAGGAGGGGACCCAGAAATTGTAATGCACAGTTTTAATTGTGGAGGGGAATTTTTCTACTGTAATACAACAAAACTATTCAATAGTACTTGG---------------AATAGTACTAGG------------------------------AATGATACTGAAAGGAAT---------AGTAATGAAACT------------------------GACACAATCACACTCCCATGCAGAATAAAACAAATTATAAACATGTGGCAGGGAGTAGGAAAAGCAATGTATGCCCCTCCTATCAGAGGACTAATTAGATGTTCATCAAATATTACAGGGCTGTTATTAACAAGGGATGGTGGTAATACT------------AACAGGAGC---------------GGGAGCAACGGGTCT---GAGACCTTCAGACCTGGAGGAGGAAATATGAAGGACAATTGGAGA---AGTGAATTATATAAATATAAAGTAGTAAAACTTGAACCA---TTAGGAATAGCACCC---ACCAAGGCAAGGAGAAGAGTGGTGCAGAGAGAA---AAAAGAGCAGTA---GGA---CTAGGA---GCGTTG---TTCATT---GGG---------TTCTTGGGA---GCAGCAGGAAGCACTATGGGCGCAGCGTCAGTA---ACGCTGACGGTACAGGCCAGACAATTATTGTCTGGTATAGTGCAACAGCAGAACAATCTGCTGATGGCTATTGATGCACAACAGCATCTGTTGCAACTCACAGTCTGGGGCGTCAAGCAGCTCCGGGCAAGA---ATCCTGGCTGTGGAAAGATACCTAAAGGATCAACAGCTCCTAGGGATTTGGGGTTGCTCTGGAAAACTCATCTGCACCACTAATGTGCCTTGGAATGCTAGTTGGAGT---------------------------AATAAATCTCAAGAGCAAATTTGGGAA---AACATGACCTGGATGCAGTGGGAAAGAGAAATTGAC------AATTACACAGGCCTTATATACTCTTTAATTGAAAAATCGCAAAACCAACAAGAAAAGAATGAACAAGAATTATTGGCATTGGATAAGTGGGCAAGTTTGTGGAATTGGTTTGACATAACAAAATGGCTGTGGTATATAAAAATATTCATAATAATAGTAGGAGGCTTGATAGGATTAAGAATAGTTTTTGCTGTATTTTCTATAGTAAATAGAGTTAGGCAGGGATACTCACCACTATCGTTTCAGACCCAC---CTCCCAGTTCCGAGGGGA------CCCGACAGGCCCGAAGGAATCGACGAAGAAGGTGGAGAGCAAGGCAGAGACAGATCAATTCGATTAGTGGATGGATTCTTGGCACTTTTCTGGGACGACCTGAGGAGCCTGTGTCTTTTCAGCTACCACCACTTGAGAGACTTACTCTTGATTGCAGCGCGGATTGTGCAACTTCTGGGACAGAGG---------------GGGTGGGAGATCCTCAAATATTGGTGG---AATCTCCTGCTGTATTGG---------------------------------------------------AGTCAGGAACTAAAGAATAGTGCTGTCAGCTTGCTCAACACCACTGCTATAGTAGTAGCTGAGGGGACAGACAGGGTTATAGAAGCATTGCAAAGA------------------GCCTTTAGGGCTATTCTCCACATCCCTACAAGAATAAGACAAGGCTTGGAAAGGGCTTTACTATAA

2.04013226.ADARC.GU330366 ATGAAAGTGAAGGTGATCAGGAAGAATTGTCAGCACTTG---------TGGACATGGGGC------------------------ACGATGCTCCTTGGGATGTTAATGATC------------TGTAGTGCTGAA---------GATCAATTGTGGGTCACAGTTTATTATGGGGTACCTGTGTGGAAAGAAGCAACCACCACTCTATTTTGTGCATCAGATGCCAAAGGATATGATACAGAGGTACATAAT---GTTTGGGCCACACATGCCTGTGTACCCACAGACCCCAACCCACAAGAAGTGTTATTG---GCAAATGTGACAGAAAATTTTAACATGTGGAAAAATAACATGGTAGACCAGATGCATGAGGATGTAATCAGTTTATGGGATCAAAGTCTAAAGCCATGTGTAAAATTGACACCACTCTGTGTTACTTTATCTTGCACTAATGTGACTAATAATAATAATACTGCCAGT------------------------------------------------------------------------------------AATAGTTCTGATTGGGAAAAGATGGAG---GGAGAAATAAAAAACTGCTCTTTCAATGTCACC---CCAAGCATAAGAGAT------AGGGTGCGCAAAGAATATGCACTCTTTTATAGCCTTGATGTAGTACCTATAAAGGATACT------------------------------AATGATAGT---------------------AGAACCTATAGATTAATAAATTGTAACACCTCAGTCATTACACAGGCCTGTCCAAAGGTATCCTTTGAGCCAATTCCCATACATTATTGTGCCCCGGCTGGTTATGCGATTCTAAAATGT---AATAATAAGACTTTCAATGGAACAGGACCATGTACAAATGTCAGCACAGTACAATGTACACATGGAATTAGGCCTGTAGTATCAACTCAACTGTTGTTAAATGGCAGCCTAGCAGAAGAA---GACATAGTAATCAGATCTGAAAATCTCACAGACAATGCTAAAACCATAATAGTACATCTGAATGAATCTGTAGAAATTAATTGTACAAGACCCAACAATAATACAAGAAAAAGTATAAATATAGGA------------CCAGGGGCA---GCGATGTATGCAACAGGAGCCATAATAGGAGATATAAGACAAGCACATTGTAACATT------AGTGGAGCAAGATGGAATGACACTTTAAATAAGGTAGTCAAAAAATTAAGA---GAAAAATTTGGA------------AATAAAACA---ATAATCTTTGATCAA---------CACTCAGGAGGGGACCCAGAAATTGTAATGCACAGTTTTAATTGTGGAGGGGAATTTTTCTACTGTAATACAACAAAACTATTCAATAGTACTTGG---------------AATAGTACTAGG------------------------------AATGATACTGAAAGGAAT---------AGTAATGAAACT------------------------GACACAATCACACTCCCATGCAGAATAAAACAAATTATAAACATGTGGCAGGGAGTAGGAAAAGCAATGTATGCCCCTCCTATCAGAGGACTAATTAGATGTTCATCAAATATTACAGGGCTGTTATTAACAAGGGATGGTGGTAATACT------------AACAGGAGC---------------GGGAGCAACGGGTCT---GAGACCTTCAGACCTGGAGGAGGAAATATGAAGGACAATTGGAGA---AGTGAATTATATAAATATAAAGTAGTAAAACTTGAACCA---TTAGGAATAGCACCC---ACCAAGGCAAGGAGAAGAGTGGTGCAGAGAGAA---AAAAGAGCAGTA---GGA---CTAGGA---GCGTTG---TTCATT---GGG---------TTCTTGGGA---GCAGCAGGAAGCACTATGGGCGCAGCGTCAGTA---ACGCTGACGGTACAGGCCAGACAATTATTGTCTGGTATAGTGCAACAGCAGAACAATCTGCTGATGGCTATTGATGCACAACAGCATCTGTTGCAACTCACAGTCTGGGGCGTCAAGCAGCTCCGGGCAAGA---ATCCTGGCTGTGGAAAGATACCTAAAGGATCAACAGCTCCTAGGGATTTGGGGTTGCTCTGGAAAACTCATCTGCACCACTAATGTGCCTTGGAATGCTAGTTGGAGT---------------------------AATAAATCTCAAGAGCAAATTTGGGAA---AACATGACCTGGATGCAGTGGGAAAGAGAAATTGAC------AATTACACAGGCCTTATATACTCTTTAATTGAAAAATCGCAAAACCAACAAGAAAAGAATGAACAAGAATTATTGGCATTGGATAAGTGGGCAAGTTTGTGGAATTGGTTTGACATAACAAAATGGCTGTGGTATATAAAAATATTCATAATAATAGTAGGAGGCTTGATAGGATTAAGAATAGTTTTTGCTGTATTTTCTATAGTAAATAGAGTTAGGCAGGGATACTCACCACTATCGTTTCAGACCCAC---CTCCCAGTTCCGAGGGGA------CCCGACAGGCCCGAAGGAATCGACGAAGAAGGTGGAGAGCAAGGCAGAGACAGATCAATTCGATTAGTGGATGGATTCTTGGCACTTTTCTGGGACGACCTGAGGAGCCTGTGTCTTTTCAGCTACCACCACTTGAGAGACTTACTCTTGATTGCAGCGCGGATTGTGCAACTTCTGGGACAGAGG---------------GGGTGGGAGATCCTCAAATATTGGTGG---AATCTCCTGCTGTATTGG---------------------------------------------------AGTCAGGAACTAAAGAATAGTGCTGTCAGCTTGCTCAACACCACTGCTATAGTAGTAGCTGAGGGGACAGACAGGGTTATAGAAGCATTGCAAAGA------------------GCCTTTAGGGCTATTCTCCACATCCCTACAAGAATAAGACAAGGCTTGGAAAGGGCTTTACTATAA

2.04013226.ADARC.GU330367 ATGAAAGTGAAGGTGATCAGGAAGAATTGTCAGCACTTG---------TGGACATGGGGC------------------------ACGATGCTCCTTGGGATGTTAATGATC------------TGTAGTGCTGAA---------GATCAATTGTGGGTCACAGTTTATTATGGGGTACCTGTGTGGAAAGAAGCAACCACCACTCTATTTTGTGCATCAGATGCCAAAGGATATGATACAGAGGTACATAAT---GTTTGGGCCACACATGCCTGTGTACCCACAGACCCCAACCCACAAGAAGTGTTATTG---GCAAATGTGACAGAAAATTTTAACATGTGGAAAAATAACATGGTAGACCAGATGCATGAGGATGTAATCAGTTTATGGGATCAAAGTCTAAAGCCATGTGTAAAATTGACACCACTCTGTGTTACTTTATCTTGCACTAATGTGACTAATAATAATAATACTGCCAGT------------------------------------------------------------------------------------AATAGTTCTGATTGGGAAAAGATGGAG---GGAGAAATAAAAAACTGCTCTTTCAATGTCACC---CCAAGCATAAGAGAT------AGGGTGCGCAAAGAATATGCACTCTTTTATAGCCTTGATGTAGTACCTATAAAGGATACT------------------------------AATGATAGT---------------------AGAACCTATAGATTAATAAATTGTAACACCTCAGTCATTACACAGGCCTGTCCAAAGGTATCCTTTGAGCCAATTCCCATACATTATTGTGCCCCGGCTGGTTATGCGATTCTAAAATGT---AATAATAAGACTTTCAATGGAACAGGACCATGTACAAATGTCAGCACAGTACAATGTACACATGGAATTAGGCCTGTAGTATCAACTCAACTGTTGTTAAATGGCAGCCTAGCAGAAGAA---GACATAGTAATCAGATCTGAAAATCTCACAGACAATGCTAAAACCATAATAGTACATCTGAATGAATCTGTAGAAATTAATTGTACAAGACCCAACAATAATACAAGAAAAAGTATAAATATAGGA------------CCAGGGGCA---GCGATGTATGCAACAGGAGCCATAATAGGAGATATAAGACAAGCACATTGTAACATT------AGTGGAGCAAGATGGAATGACACTTTAAAAAAGGTAGTCAAAAAATTAAGA---GAAAAATTTGGA------------AATAAAACA---ATAATCTTTGATCAA---------CACTCAGGAGGGGACCCAGAAATTGTAATGCACAGTTTTAATTGTGGAGGGGAATTTTTCTACTGTAATACAACAAAACTATTCAATAGTACTTGG---------------AATAGTACTAGG------------------------------AATGATACTGAAAGGAAT---------AGTAATGAAACT------------------------GACACAATCACACTCCCATGCAGAATAAAACAAATTATAAACATGTGGCAGGGAGTAGGAAAAGCAATGTATGCCCCTCCTATCAGAGGACTAATTAGATGTTCATCAAATATTACAGGGCTGTTATTAACAAGGGATGGTGGTAATACT------------AACAGGAGC---------------GGGAGCAACGGGTCT---GAGACCTTCAGACCTGGAGGAGGAAATATGAAGGACAATTGGAGA---AGTGAATTATATAAATATAAAGTAGTAAAACTTGAACCA---TTAGGAATAGCACCC---ACCAAGGCAAGGAGAAGAGTGGTGCAGAGAGAA---AAAAGAGCAGTA---GGA---CTAGGA---GCGTTG---TTCATT---GGG---------TTCTTGGGA---GCAGCAGGAAGCACTATGGGCGCAGCGTCAGTA---ACGCTGACGGTACAGGCCAGACAATTATTGTCTGGTATAGTGCAACAGCAGAACAATCTGCTGATGGCTATTGATGCACAACAGCATCTGTTGCAACTCACAGTCTGGGGCGTCAAGCAGCTCCGGGCAAGA---ATCCTGGCTGTGGAAAGATACCTAAAGGATCAACAGCTCCTAGGGATTTGGGGTTGCTCTGGAAAACTCATCTGCACCACTAATGTGCCTTGGAATGCTAGTTGGAGT---------------------------AATAAATCTCAAGAGCAAATTTGGGAA---AACATGACCTGGATGCAGTGGGAAAGAGAAATTGAC------AATTACACAGGCCTTATATACTCTTTAATTGAAAAATCGCAAAACCAACAAGAAAAGAATGAACAAGAATTATTGGCATTGGATAAGTGGGCAAGTTTGTGGAATTGGTTTGACATAACAAAATGGCTGTGGTATATAAAAATATTCATAATAATAGTAGGAGGCTTGATAGGATTAAGAATAGTTTTTGCTGTATTTTCTATAGTAAATAGAGTTAGGCAGGGATACTCACCACTATCGTTTCAGACCCAC---CTCCCAGTTCCGAGGGGA------CCCGACAGGCCCGAAGGAATCGACGAAGAAGGTGGAGAGCAAGGCAGAGACAGATCAATTCGATTAGTGGATGGATTCTTGGCACTTTTCTGGGACGACCTGAGGAGCCTGTGTCTTTTCAGCTACCACCACTTGAGAGACTTACTCTTGATTGCAGCGCGGATTGTGCAACTTCTGGGACAGAGG---------------GGGTGGGAGATCCTCAAATATTGGTGG---AATCTCCTGCTGTATTGG---------------------------------------------------AGTCAGGAACTAAAGAATAGTGCTGTCAGCTTGCTCAACACCACTGCTATAGTAGTAGCTGAGGGGACAGACAGGGTTATAGAAGCATTGCAAAGA------------------GCCTTTAGGGCTATTCTCCACATCCCTACAAGAATAAGACAAGGCTTGGAAAGGGCTTTACTATAA

2.04013226.ADARC.GU330368 ATGAAAGTGAAGGTGATCAGGAAGAATTGTCAGCACTTG---------TGGACATGGGGC------------------------ACGATGCTCCTTGGGATGTTAATGATC------------TGTAGTGCTGAA---------GATCAATTGTGGGTCACAGTTTATTATGGGGTACCTGTGTGGAAAGAAGCAACCACCACTCTATTTTGTGCATCAGATGCCAAAGGATATGATACAGAGGTACATAAT---GTTTGGGCCACACATGCCTGTGTACCCACAGACCCCAACCCACAAGAAGTGTTATTG---GCAAATGTGACAGAAAATTTTAACATGTGGAAAAATAACATGGTAGACCAGATGCATGAGGATGTAATCAGTTTATGGGATCAAAGTCTAAAGCCATGTGTAAAATTGACACCACTCTGTGTTACTTTATCTTGCACTAATGTGACTAATAATAATAATACTGCCAGT------------------------------------------------------------------------------------AATAGTTCTGATTGGGAAAAGATGGAG---GGAGAAATAAAAAACTGCTCTTTCAATGTCACC---CCAAGCATAAGAGAT------AGGGTGCGCAAAGAATATGCACTCTTTTATAGCCTTGATGTAGTACCTATAAAGGATACT------------------------------AATGATAGT---------------------AGAACCTATAGATTAATAAATTGTAACACCTCAGTCATTACACAGGCCTGTCCAAAGGTATCCTTTGAGCCAATTCCCATACATTATTGTGCCCCGGCTGGTTATGCGATTCTAAAATGT---AATAATAAGACTTTCAATGGAACAGGACCATGTACAAATGTCAGCACAGTACAATGTACACATGGAATTAGGCCTGTAGTATCAACTCAACTGTTGTTAAATGGCAGCCTAGCAGAAGAA---GACATAGTAATCAGATCTGAAAATCTCACAGACAATGCTAAAACCATAATAGTACATCTGAATGAATCTGTAGAAATTAATTGTACAAGACCCAACAATAATACAAGAAAAAGTATAAATATAGGA------------CCAGGGGCA---GCGATGTATGCAACAGGAGCCATAATAGGAGATATAAGACAAGCACATTGTAACATT------AGTGGAGCAAGATGGAATGACACTTTAAAAAAGGTAGTCAAAAAATTAAGA---GAAAAATTTGGA------------AATAAAACA---ATAATCTTTGATCAA---------CACTCAGGAGGGGACCCAGAAATTGTAATGCACAGTTTTAATTGTGGAGGGGAATTTTTCTACTGTAATACAACAAAACTATTCAATAGTACTTGG---------------AATAGTACTAGG------------------------------AATAATACTGAAAGGAAT---------AGTAATGAAACT------------------------GACACAATCACACTCCCATGCAGAATAAAACAAATTATAAACATGTGGCAGGGAGTAGGAAAAGCAATGTATGCCCCTCCTATCAGAGGACTAATTAGATGTTCATCAAATATTACAGGGCTGTTATTAACAAGGGATGGTGGTAATACT------------AACAGGAGC---------------GGGAGCAACGGGTCT---GAGACCTTCAGACCTGGAGGAGGAAATATGAAGGACAATTGGAGA---AGTGAATTATATAAATATAAAGTAGTAAAACTTGAACCA---TTAGGAATAGCACCC---ACCAAGGCAAGGAGAAGAGTGGTGCAGAGAGAA---AAAAGAGCAGTA---GGA---CTAGGA---GCGTTG---TTCATT---GGG---------TTCTTGGGA---GCAGCAGGAAGCACTATGGGCGCAGCGTCAGTA---ACGCTGACGGTACAGGCCAGACAATTATTGTCTGGTATAGTGCAACAGCAGAACAATCTGCTGATGGCTATTGATGCACAACAGCATCTGTTGCAACTCACAGTCTGGGGCGTCAAGCAGCTCCGGGCAAGA---ATCCTGGCTGTGGAAAGATACCTAAAGGATCAACAGCTCCTAGGGATTTGGGGTTGCTCTGGAAAACTCATCTGCACCACTAATGTGCCTTGGAATGCTAGTTGGAGT---------------------------AATAAATCTCAAGAGCAAATTTGGGAA---AACATGACCTGGATGCAGTGGGAAAGAGAAATTGAC------AATTACACAGGCCTTATATACTCTTTAATTGAAAAATCGCAAAACCAACAAGAAAAGAATGAACAAGAATTATTGGCATTGGATAAGTGGGCAAGTTTGTGGAATTGGTTTGACATAACAAAATGGCTGTGGTATATAAAAATATTCATAATAATAGTAGGAGGCTTGATAGGATTAAGAATAGTTTTTGCTGTATTTTCTATAGTAAATAGAGTTAGGCAGGGATACTCACCACTATCGTTTCAGACCCAC---CTCCCAGTTCCGAGGGGA------CCCGACAGGCCCGAAGGAATCGACGAAGAAGGTGGAGAGCAAGGCAGAGACAGATCAATTCGATTAGTGGATGGATTCTTGGCACTTTTCTGGGACGACCTGAGGAGCCTGTGTCTTTTCAGCTACCACCACTTGAGAGACTTACTCTTGATTGCAGCGCGGATTGTGCAACTTCTGGGACAGAGG---------------GGGTGGGAGATCCTCAAATATTGGTGG---AATCTCCTGCTGTATTGG---------------------------------------------------AGTCAGGAACTAAAGAATAGTGCTGTCAGCTTGCTCAACACCACTGCTATAGTAGTAGCTGAGGGGACAGACAGGGTTATAGAAGCATTGCAAAGA------------------GCCTTTAGGGCTATTCTCCACATCCCTACAAGAATAAGACAAGGCTTGGAAAGGGCTTTACTATAA

2.04013226.ADARC.GU330369 ATGAAAGTGAAGGTGATCAGGAAGAATTGTCAGCACTTG---------TGGACATGGGGC------------------------ACGATGCTCCTTGGGATGTTAATGATC------------TGTAGTGCTGAA---------GATCAATTGTGGGTCACAGTTTATTATGGGGTACCTGTGTGGAAAGAAGCAACCACCACTCTATTTTGTGCATCAGATGCCAAAGGATATGATACAGAGGTACATAAT---GTTTGGGCCACACATGCCTGTGTACCCACAGACCCCAACCCACAAGAAGTGTTATTG---GCAAATGTGACAGAAAATTTTAACATGTGGAAAAATAACATGGTAGACCAGATGCATGAGGATGTAATCAGTTTATGGGATCAAAGTCTAAAGCCATGTGTAAAATTGACACCACTCTGTGTTACTTTATCTTGCACTAATGTGACTAATAATAATAATACTGCCAGT------------------------------------------------------------------------------------AATAGTTCTGATTGGGAAAAGATGGAG---GGAGAAATAAAAAACTGCTCTTTCAATGTCACC---CCAAGCATAAGAGAT------AGGGTGCGCAAAGAATATGCACTCTTTTATAGCCTTGATGTAGTACCTATAAAGGATACT------------------------------AATGATAGT---------------------AGAACCTATAGATTAATAAATTGTAACACCTCAGTCATTACACAGGCCTGTCCAAAGGTATCCTTTGAGCCAATTCCCATACATTATTGTGCCCCGGCTGGTTATGCGATTCTAAAATGT---AATAATAAGACTTTCAATGGAACAGGACCATGTACAAATGTCAGCACAGTACAATGTACACATGGAATTAGGCCTGTAGTATCAACTCAACTGTTGTTAAATGGCAGCCTAGCAGAAGAA---GACATAGTAATCAGATCTGAAAATCTCACAGACAATGCTAAAACCATAATAGTACATCTGAATGAATCTGTAGAAATTAATTGTACAAGACCCAACAATAATACAAGAAAAAGTATAAATATAGGA------------CCAGGGGCA---GCGATGTATGCAACAGGAGCCATAATAGGAGATATAAGACAAGCACATTGTAACATT------AGTGGAGCAAGATGGAATGACACTTTAAAAAAGGTAGTCAAAAAATTAAGA---GAAAAATTTGGA------------AATAAAACA---ATAATCTTTGATCAA---------CACTCAGGAGGGGACCCAGAAATTGTAATGCACAGTTTTAATTGTGGAGGGGAATTTTTCTACTGTAATACAACAAAACTATTCAATAGTACTTGG---------------AATAGTACTAGG------------------------------AATGATACTGAAAGGAAT---------AGTAATGAAACT------------------------GACACAATCACACTCCCATGCAGAATAAAACAAATTATAAACATGTGGCAGGGAGTAGGAAAAGCAATGTATGCCCCTCCTATCAGAGGACTAATTAGATGTTCATCAAATATTACAGGGCTGTTATTAACAAGGGATGGTGGTAATACT------------AACAGGAGC---------------GGGAGCAACGGGTCT---GAGACCTTCAGACCTGGAGGAGGAAATATGAAGGACAATTGGAGA---AGTGAATTATATAAATATAAAGTAGTAAAACTTGAACCA---TTAGGAATAGCACCC---ACCAAGGCAAGGAGAAGAGTGGTGCAGAGAGAA---AAAAGAGCAGTA---GGA---CTAGGA---GCGTTG---TTCATT---GGG---------TTCTTGGGA---GCAGCAGGAAGCACTATGGGCGCAGCGTCAGTA---ACGCTGACGGTACAGGCCAGACAATTATTGTCTGGTATAGTGCAACAGCAGAACAATCTGCTGATGGCTATTGATGCACAACAGCATCTGTTGCAACTCACAGTCTGGGGCGTCAAGCAGCTCCGGGCAAGA---ATCCTGGCTGTGGAAAGATACCTAAAGGATCAACAGCTCCTAGGGATTTGGGGTTGCTCTGGAAAACTCATCTGCACCACTAATGTGCCTTGGAATGCTAGTTGGAGT---------------------------AATAAATCTCAAGAGCAAATTTGGGAA---AACATGACCTGGATGCAGTGGGAAAGAGAAATTGAC------AATTACACAGGCCTTATATACTCTTTAATTGAAAAATCGCAAAACCAACAAGAAAAGAATGAACAAGAATTATTGGCATTGGATAAGTGGGCAAGTTTGTGGAATTGGTTTGACATAACAAAATGGCTGTGGTATATAAAAATATTCATAATAATAGTAGGAGGCTTGATAGGATTAAGAATAGTTTTTGCTGTATTTTCTATAGTAAATAGAGTTAGGCAGGGATACTCACCACTATCGTTTCAGACCCAC---CTCCCAGTTCCGAGGGGA------CCCGACAGGCCCGAAGGAATCGACGAAGAAGGTGGAGAGCAAGGCAGAGACAGATCAATTCGATTAGTGGATGGATTCTTGGCACTTTTCTGGGACGACCTGAGGAGCCTGTGTCTTTTCAGCTACCACCACTTGAGAGACTTACTCTTGATTGCAGCGCGGATTGTGCAACTTCTGGGACAGAGG---------------GGGTGGGAGATCCTCAAATATTGGTGG---AATCTCCTGCTGTATTGG---------------------------------------------------AGTCAGGAACTAAAGAATAGTGCTGTCAGCTTGCTCAACACCACTGCTATAGTAGTAGCTGAGGGGACAGACAGGGTTATAGAAGCATTGCAAAGA------------------GCCTTTAGGGCTATTCTCCACATCCCTACAAGAATAAGACAAGGCTTGGAAAGGGCTTTACTATAA

2.04013226.ADARC.GU330370 ATGAAAGTGAAGGTGATCAGGAAGAATTGTCAGCACTTG---------TGGACATGGGGC------------------------ACGATGCTCCTTGGGATGTTAATGATC------------TGTAGTGCTGAA---------GATCAATTGTGGGTCACAGTTTATTATGGGGTACCTGTGTGGAAAGAAGCAACCACCACTCTATTTTGTGCATCAGATGCCAAAGGATATGATACAGAGGTACATAAT---GTTTGGGCCACACATGCCTGTGTACCCACAGACCCCAACCCACAAGAAGTGTTATTG---GCAAATGTGACAGAAAATTTTAACATGTGGAAAAATAACATGGTAGACCAGATGCATGAGGATGTAATCAGTTTATGGGATCAAAGTCTAAAGCCATGTGTAAAATTGACACCACTCTGTGTTACTTTATCTTGCACTAATGTGACTAATAATAATAATACTGCCAGT------------------------------------------------------------------------------------AATAGTTCTGATTGGGAAAAGATGGAG---GGAGAAATAAAAAACTGCTCTTTCAATGTCACC---CCAAGCATAAGAGAT------AGGGTGCGCAAAGAATATGCACTCTTTTATAGCCTTGATGTAGTACCTATAAAGGATACT------------------------------AATGATAGT---------------------AGAACCTATAGATTAATAAATTGTAACACCTCAGTCATTACACAGGCCTGTCCAAAGGTATCCTTTGAGCCAATTCCCATACATTATTGTGCCCCGGCTGGTTATGCGATTCTAAAATGT---AATAATAAGACTTTCAATGGAACAGGACCATGTACAAATGTCAGCACAGTACAATGTACACATGGAATTAGGCCTGTAGTATCAACTCAACTGTTGTTAAATGGCAGCCTAGCAGAAGAA---GACATAGTAATCAGATCTGAAAATCTCACAGACAATGCTAAAACCATAATAGTACATCTGAATGAATCTGTAGAAATTAATTGTACAAGACCCAACAATAATACAAGAAAAAGTATAAATATAGGA------------CCAGGGGCA---GCGATGTATGCAACAGGAGCCATAATAGGAGATATAAGACAAGCACATTGTAACATT------AGTGGAGCAAGATGGAATGACACTTTAAAAAAGGTAGTCAAAAAATTAAGA---GAAAAATTTGGA------------AATAAAACA---ATAATCTTTGATCAA---------CACTCAGGAGGGGACCCAGAAATTGTAATGCACAGTTTTAATTGTGGAGGGGAATTTTTCTACTGTAATACAACAAAACTATTCAATAGTACTTGG---------------AATAGTACTAGG------------------------------AATGATACTGAAAGGAAT---------AGTAATGAAACT------------------------GACACAATCACACTCCCATGCAGAATAAAACAAATTATAAACATGTGGCAGGGAGTAGGAAAAGCAATGTATGCCCCTCCTATCAGAGGACTAATTAGATGTTCATCAAATATTACAGGGCTGTTATTAACAAGGGATGGTGGTAATACT------------AACAGGAGC---------------GGGAGCAACGGGTCT---GAGACCTTCAGACCTGGAGGAGGAAATATGAAGGACAATTGGAGA---AGTGAATTATATAAATATAAAGTAGTAAAACTTGAACCA---TTAGGAATAGCACCC---ACCAAGGCAAGGAGAAGAGTGGTGCAGAGAGAA---AAAAGAGCAGTA---GGA---CTAGGA---GCGTTG---TTCATT---GGG---------TTCTTGGGA---GCAGCAGGAAGCACTATGGGCGCAGCGTCAGTA---ACGCTGACGGTACAGGCCAGACAATTATTGTCTGGTATAGTGCAACAGCAGAACAATCTGCTGATGGCTATTGATGCACAACAGCATCTGTTGCAACTCACAGTCTGGGGCGTCAAGCAGCTCCGGGCAAGA---ATCCTGGCTGTGGAAAGATACCTAAAGGATCAACAGCTCCTAGGGATTTGGGGTTGCTCTGGAAAACTCATCTGCACCACTAATGTGCCTTGGAATGCTAGTTGGAGT---------------------------AATAAATCTCAAGAGCAAATTTGGGAA---AACATGACCTGGATGCAGTGGGAAAGAGAAATTGAC------AATTACACAGGCCTTATATACTCTTTAATTGAAAAATCGCAAAACCAACAAGAAAAGAATGAACAAGAATTATTGGCATTGGATAAGTGGGCAAGTTTGTGGAATTGGTTTGACATAACAAAATGGCTGTGGTATATAAAAATATTCATAATAATAGTAGGAGGCTTGATAGGATTAAGAATAGTTTTTGCTGTATTTTCTATAGTAAATAGAGTTAGGCAGGGATACTCACCACTATCGTTTCAGACCCAC---CTCCCAGTTCCGAGGGGA------CCCGACAGGCCCGAAGGAATCGACGAAGAAGGTGGAGAGCAAGGCAGAGACAGATCAATTCGATTAGTGGATGGATTCTTGGCACTTTTCTGGGACGACCTGAGGAGCCTGTGTCTTTTCAGCTACCACCACTTGAGAGACTTACTCTTGATTGCAGCGCGGATTGTGCAACTTCTGGGACAGAGG---------------GGGTGGGAGATCCTCAAATATTGGTGG---AATCTCCTGCTGTATTGG---------------------------------------------------AGTCAGGAACTAAAGAATAGTGCTGTCAGCTTGCTCAACACCACTGCTATAGTAGTAGCTGAGGGGACAGACAGGGTTATAGAAGCATTGCAAAGA------------------GCCTTTAGGGCTATTCTCCACATCCCTACAAGAATAAGACAAGGCTTGGAAAGGGCTTTACTATAA

2.04013226.ADARC.GU330371 ATGAAAGTGAAGGTGATCAGGAAGAATTGTCAGCACTTG---------TGGACATGGGGC------------------------ACGATGCTCCTTGGGATGTTAATGATC------------TGTAGTGCTGAA---------GATCAATTGTGGGTCACAGTTTATTATGGGGTACCTGTGTGGAAAGAAGCAACCACCACTCTATTTTGTGCATCAGATGCCAAAGGATATGATACAGAGGTACATAAT---GTTTGGGCCACACATGCCTGTGTACCCACAGACCCCAACCCACAAGAAGTGTTATTG---GCAAATGTGACAGAAAATTTTAACATGTGGAAAAATAACATGGTAGACCAGATGCATGAGGATGTAATCAGTTTATGGGATCAAAGTCTAAAGCCATGTGTAAAATTGACACCACTCTGTGTTACTTTATCTTGCACTAATGTGACTAATAATAATAATACTGCCAGT------------------------------------------------------------------------------------AATAGTTCTGATTGGGAAAAGATGGAG---GGAGAAATAAAAAACTGCTCTTTCAATGTCACC---CCAAGCATAAGAGAT------AGGGTGCGCAAAGAATATGCACTCTTTTATAGCCTTGATGTAGTACCTATAAAGGATACT------------------------------AATGATAGT---------------------AGAACCTATAGATTAATAAATTGTAACACCTCAGTCATTACACAGGCCTGTCCAAAGGTATCCTTTGAGCCAATTCCCATACATTATTGTGCCCCGGCTGGTTATGCGATTCTAAAATGT---AATAATAAGACTTTCAATGGAACAGGACCATGTACAAATGTCAGCACAGTACAATGTACACATGGAATTAGGCCTGTAGTATCAACTCAACTGTTGTTAAATGGCAGCCTAGCAGAAGAA---GACATAGTAATCAGATCTGAAAATCTCACAGACAATGCTAAAACCATAATAGTACATCTGAATGAATCTGTAGAAATTAATTGTACAAGACCCAACAATAATACAAGAAAAAGTATAAATATAGGA------------CCAGGGGCA---GCGATGTATGCAACAGGAGCCATAATAGGAGATATAAGACAAGCACATTGTAACATT------AGTGGAGCAAGATGGAATGACACTTTAAAAAAGGTAGTCAAAAAATTAAGA---GAAAAATTTGGA------------AATAAAACA---ATAATCTTTGATCAA---------CACTCAGGAGGGGACCCAGAAGTTGTAATGCACAGTTTTAATTGTGGAGGGGAATTTTTCTACTGTAATACAACAAAACTATTCAATAGTACTTGG---------------AATAGTACTAGG------------------------------AATGATACTGAAAGGAAT---------AGTAATGAAACT------------------------GACACAATCACACTCCCATGCAGAATAAAACAAATTATAAACATGTGGCAGGGAGTAGGAAAAGCAATGTATGCCCCTCCTATCAGAGGACTAATTAGATGTTCATCAAATATTACAGGGCTGTTATTAACAAGGGATGGTGGTAATACT------------AACAGGAGC---------------GGGAGCAACGGGTCT---GAGACCTTCAGACCTGGAGGAGGAAATATGAAGGACAATTGGAGA---AGTGAATTATATAAATATAAAGTAGTAAAACTTGAACCA---TTAGGAATAGCACCC---ACCAAGGCAAGGAGAAGAGTGGTGCAGAGAGAA---AAAAGAGCAGTA---GGA---CTAGGA---GCGTTG---TTCATT---GGG---------TTCTTGGGA---GCAGCAGGAAGCACTATGGGCGCAGCGTCAGTA---ACGCTGACGGTACAGGCCAGACAATTATTGTCTGGTATAGTGCAACAGCAGAACAATCTGCTGATGGCTATTGATGCACAACAGCATCTGTTGCAACTCACAGTCTGGGGCGTCAAGCAGCTCCGGGCAAGA---ATCCTGGCTGTGGAAAGATACCTAAAGGATCAACAGCTCCTAGGGATTTGGGGTTGCTCTGGAAAACTCATCTGCACCACTAATGTGCCTTGGAATGCTAGTTGGAGT---------------------------AATAAATCTCAAGAGCAAATTTGGGAA---AACATGACCTGGATGCAGTGGGAAAGAGAAATTGAC------AATTACACAGGCCTTATATACTCTTTAATTGAAAAATCGCAAAACCAACAAGAAAAGAATGAACAAGAATTATTGGCATTGGATAAGTGGGCAAGTTTGTGGAATTGGTTTGACATAACAAAATGGCTGTGGTATATAAAAATATTCATAATAATAGTAGGAGGCTTGATAGGATTAAGAATAGTTTTTGCTGTATTTTCTATAGTAAATAGAGTTAGGCAGGGATACTCACCACTATCGTTTCAGACCCAC---CTCCCAGTTCCGAGGGGA------CCCGACAGGCCCGAAGGAATCGACGAAGAAGGTGGAGAGCAAGGCAGAGACAGATCAATTCGATTAGTGGATGGATTCTTGGCACTTTTCTGGGACGACCTGAGGAGCCTGTGTCTTTTCAGCTACCACCACTTGAGAGACTTACTCTTGATTGCAGCGCGGATTGTGCAACTTCTGGGACAGAGG---------------GGGTGGGAGATCCTCAAATATTGGTGG---AATCTCCTGCTGTATTGG---------------------------------------------------AGTCAGGAACTAAAGAATAGTGCTGTCAGCTTGCTCAACACCACTGCTATAGTAGTAGCTGAGGGGACAGACAGGGTTATAGAAGCATTGCAAAGA------------------GCCTTTAGGGCTATTCTCCACATCCCTACAAGAATAAGACAAGGCTTGGAAAGGGCTTTACTATAA

2.04013226.ADARC.GU330372 ATGAAAGTGAAGGTGATCAGGAAGAATTGTCAGCACTTG---------TGGACATGGGGC------------------------ACGATGCTCCTTGGGATGTTAATGATC------------TGTAGTGCTGAA---------GATCAATTGTGGGTCACAGTTTATTATGGGGTACCTGTGTGGAAAGAAGCAACCACCACTCTATTTTGTGCATCAGATGCCAAAGGATATGATACAGAGGTACATAAT---GTTTGGGCCACACATGCCTGTGTACCCACAGACCCCAACCCACAAGAAGTGTTATTG---GCAAATGTGACAGAAAATTTTAACATGTGGAAAAATAACATGGTAGACCAGATGCATGAGGATGTAATCAGTTTATGGGATCAAAGTCTAAAGCCATGTGTAAAATTGACACCACTCTGTGTTACTTTATCTTGCACTAATGTGACTAATAATAATAATACTGCCAGT------------------------------------------------------------------------------------AATAGTTCTGATTGGGAAAAGATGGAG---GGAGAAATAAAAAACTGCTCTTTCAATGTCACC---CCAAGCATAAGAGAT------AGGGTGCGCAAAGAATATGCACTCTTTTATAGCCTTGATGTAGTACCTATAAAGGATACT------------------------------AATGATAGT---------------------AGAACCTATAGATTAATAAATTGTAACACCTCAGTCATTACACAGGCCTGTCCAAAGGTATCCTTTGAGCCAATTCCCATACATTATTGTGCCCCGGCTGGTTATGCGATTCTAAAATGT---AATAATAAGACTTTCAATGGAACAGGACCATGTACAAATGTCAGCACAGTACAATGTACACATGGAATTAGGCCTGTAGTATCAACTCAACTGTTGTTAAATGGCAGCCTAGCAGAAGAA---GACATAGTAATCAGATCTGAAAATCTCACAGACAATGCTAAAACCATAATAGTACATCTGAATGAATCTGTAGAAATTAATTGTACAAGACCCAACAATAATACAAGAAAAAGTATAAATATAGGA------------CCAGGGGCA---GCGATGTATGCAACAGGAGCCATAATAGGAGATATAAGACAAGCACATTGTAACATT------AGTGGAGCAAGATGGAATGACACTTTAAAAAAGGTAGTCAAAAAATTAAGA---GAAAAATTTGGA------------AATAAAACA---ATAATCTTTGATCAA---------CACTCAGGAGGGGACCCAGAAATTGTAATGCACAGTTTTAATTGTGGAGGGGAATTTTTCTACTGTAATACAACAAAACTATTCAATAGTACTTGG---------------AATAGTACTAGG------------------------------AATGATACTGAAAGGAAT---------AGTAATGAAACT------------------------GACACAATCACACTCCCATGCAGAATAAAACAAATTATAAACATGTGGCAGGGAGTAGGAAAAGCAATGTATGCCCCTCCTATCAGAGGACTAATTAGATGTTCATCAAATATTACAGGGCTGTTATTAACAAGGGATGGTGGTAATACT------------AACAGGAGC---------------GGGAGCAACGGGTCT---GAGACCTTCAGACCTGGAGGAGGAAATATGAAGGACAATTGGAGA---AGTGAATTATATAAATATAAAGTAGTAAAACTTGAACCA---TTAGGAATAGCACCC---ACCAAGGCAAGGAGAAGAGTGGTGCAAAGAGAA---AAAAGAGCAGTA---GGA---CTAGGA---GCGTTG---TTCATT---GGG---------TTCTTGGGA---GCAGCAGGAAGCACTATGGGCGCAGCGTCAGTA---ACGCTGACGGTACAGGCCAGACAATTATTGTCTGGTATAGTGCAACAGCAGAACAATCTGCTGATGGCTATTGATGCACAACAGCATCTGTTGCAACTCACAGTCTGGGGCGTCAAGCAGCTCCGGGCAAGA---ATCCTGGCTGTGGAAAGATACCTAAAGGATCAACAGCTCCTAGGGATTTGGGGTTGCTCTGGAAAACTCATCTGCACCACTAATGTGCCTTGGAATGCTAGTTGGAGT---------------------------AATAAATCTCAAGAGCAAATTTGGGAA---AACATGACCTGGATGCAGTGGGAAAGAGAAATTGAC------AATTACACAGGCCTTATATACTCTTTAATTGAAAAATCGCAAAACCAACAAGAAAAGAATGAACAAGAATTATTGGCATTGGATAAGTGGGCAAGTTTGTGGAATTGGTTTGACATAACAAAATGGCTGTGGTATATAAAAATATTCATAATAATAGTAGGAGGCTTGATAGGATTAAGAATAGTTTTTGCTGTATTTTCTATAGTAAATAGAGTTAGGCAGGGATACTCACCACTATCGTTTCAGACCCAC---CTCCCAGTTCCGAGGGGA------CCCGACAGGCCCGAAGGAATCGACGAAGAAGGTGGAGAGCAAGGCAGAGACAGATCAATTCGATTAGTGGATGGATTCTTGGCACTTTTCTGGGACGACCTGAGGAGCCTGTGTCTTTTCAGCTACCACCACTTGAGAGACTTACTCTTGATTGCAGCGCGGATTGTGCAACTTCTGGGACAGAGG---------------GGGTGGGAGATCCTCAAATATTGGTGG---AATCTCCTGCTGTATTGG---------------------------------------------------AGTCAGGAACTAAAGAATAGTGCTGTCAGCTTGCTCAACACCACTGCTATAGTAGTAGCTGAGGGGACAGACAGGGTTATAGAAGCATTGCAAAGA------------------GCCTTTAGGGCTATTCTCCACATCCCTACAAGAATAAGACAAGGCTTGGAAAGGGCTTTACTATAA

2.04013226.ADARC.GU330373 ATGAAAGTGAAGGTGATCAGGAAGAATTGTCAGCACTTG---------TGGACATGGGGC------------------------ACGATGCTCCTTGGGATGTTAATGATC------------TGTAGTGCTGAA---------GATCAATTGTGGGTCACAGTTTATTATGGGGTACCTGTGTGGAAAGAAGCAACCACCACTCTATTTTGTGCATCAGATGCCAAAGGATATGATACAGAGGTACATAAT---GTTTGGGCCACACATGCCTGTGTACCCACAGACCCCAACCCACAAGAAGTGTTATTG---GCAAATGTGACAGAAAATTTTAACATGTGGAAAAATAACATGGTAGACCAGATGCATGAGGATGTAATCAGTTTATGGGATCAAAGTCTAAAGCCATGTGTAAAATTGACACCACTCTGTGTTACTTTATCTTGCACTAATGTGACTAATAATAATAATACTGCCAGT------------------------------------------------------------------------------------AATAGTTCTGATTGGGAAAAGATGGAG---GGAGAAATAAAAAACTGCTCTTTCAATGTCACC---CCAAGCATAAGAGAT------AGGGTGCGCAAAGAATATGCACTCTTTTATAGCCTTGATGTAGTACCTATAAAGGATACT------------------------------AATGATAGT---------------------AGAACCTATAGATTAATAAATTGTAACACCTCAGTCATTACACAGGCCTGTCCAAAGGTATCCTTTGAGCCAATTCCCATACATTATTGTGCCCCGGCTGGTTATGCGATTCTAAAATGT---AATAATAAGACTTTCAATGGAACAGGAACATGTACAAATGTCAGCACAGTACAATGTACACATGGAATTAGGCCTGTAGTATCAACTCAACTGTTGTTAAATGGCAGCCTAGCAGAAGAA---GACATAGTAATCAGATCTGAAAATCTCACAGACAATGCTAGAACCATAATAGTACATCTGAATGAATCTGTAGAAATTAATTGTACAAGACCCAACAATAATACAAGAAAAAGTATAAATATAGGA------------CCAGGGGCA---GCGATGTATGCAACAGGAGCCATAATAGGAGATATAAGACAAGCACATTGTAACATT------AGTGGAGCAAGATGGAATGACACTTTAAAAAAGGTAGTCAAAAAATTAAGA---GAAAAATTTGGA------------AATAAAACA---ATAATCTTTGATCAA---------CACTCAGGAGGGGACCCAGAAATTGTAATGCACAGTTTTAATTGTGGAGGGGAATTTTTCTACTGTAATACAACAAAACTATTCAATAGTACTTGG---------------AATAGTACTAGG------------------------------AATGATACTGAAAGGAAT---------AGTAATGAAACT------------------------GACACAATCACACTCCCATGCAGAATAAAACAAATTATAAACATGTGGCAGGGAGTAGGAAAAGCAATGTATGCCCCTCCTATCAGAGGACTAATTAGATGTTCATCAAATATTACAGGGCTGTTATTAACAAGGGATGGTGGTAATACT------------AACAGGAGC---------------GGGAGCAACGGGTCT---GAGACCTTCAGACCTGGAGGAGGAAATATGAAGGACAATTGGAGA---AGTGAATTATATAAATATAAAGTAGTAAAACTTGAACCA---TTAGGAATAGCACCC---ACCAAGGCAAGGAGAAGAGTGGTGCAGAGAGAA---AAAAGAGCAGTA---GGA---CTAGGA---GCGTTG---TTCATT---GGG---------TTCTTGGGA---GCAGCAGGAAGCACTATGGGCGCAGCGTCAGTA---ACGCTGACGGTACAGGCCAGACAATTATTGTCTGGTATAGTGCAACAGCAGAACAATCTGCTGATGGCTATTGATGCACAACAGCATCTGTTGCAACTCACAGTCTGGGGCGTCAAGCAGCTCCGGGCAAGA---ATCCTGGCTGTGGAAAGATACCTAAAGGATCAACAGCTCCTAGGGATTTGGGGTTGCTCTGGAAAACTCATCTGCACCACTAATGTGCCTTGGAATGCTAGTTGGAGT---------------------------AATAAATCTCAAGAGCAAATTTGGGAA---AACATGACCTGGATGCAGTGGGAAAGAGAAATTGAC------AATTACACAGGCCTTATATACTCTTTAATTGAAAAATCGCAAAACCAACAAGAAAAGAATGAACAAGAATTATTGGCATTGGATAAGTGGGCAAGTTTGTGGAATTGGTTTGACATAACAAAATGGCTGTGGTATATAAAAATATTCATAATAATAGTAGGAGGCTTGATAGGATTAAGAATAGTTTTTGCTGTATTTTCTATAGTAAATAGAGTTAGGCAGGGATACTCACCACTATCGTTTCAGACCCAC---CTCCCAGTTCCGAGGGGA------CCCGACAGGCCCGAAGGAATCGACGAAGAAGGTGGAGAGCAAGGCAGAGACAGATCAATTCGATTAGTGGATGGATTCTTGGCACTTTTCTGGGACGACCTGAGGAGCCTGTGTCTTTTCAGCTACCACCACTTGAGAGACTTACTCTTGATTGCAGCGCGGATTGTGCAACTTCTGGGACAGAGG---------------GGGTGGGAGATCCTCAAATATTGGTGG---AATCTCCTGCTGTATTGG---------------------------------------------------AGTCAGGAACTAAAGAATAGTGCTGTCAGCTTGCTCAACACCACTGCTATAGTAGTAGCTGAGGGGACAGACAGGGTTATAGAAGCATTGCAAAGA------------------GCCTTTAGGGCTATTCTCCACATCCCTACAAGAATAAGACAAGGCTTGGAAAGGGCTTTACTATAA

2.04013226.ADARC.GU330374 ATGAAAGTGAAGGTGATCAGGAAGAATTGTCAGCACTTG---------TGGACATGGGGC------------------------ACGATGCTCCTTGGGATGTTAATGATC------------TGTAGTGCTGAA---------GATCAATTGTGGGTCACAGTTTATTATGGGGTACCTGTGTGGAAAGAAGCAACCACCACTCTATTTTGTGCATCAGATGCCAAAGGATATGATACAGAGGTACATAAT---GTTTGGGCCACACATGCCTGTGTACCCACAGACCCCAACCCACAAGAAGTGTTATTG---GCAAATGTGACAGAAAATTTTAACATGTGGAAAAATAACATGGTAGACCAGATGCATGAGGATGTAATCAGTTTATGGGATCAAAGTCTAAAGCCATGTGTAAAATTGACACCACTCTGTGTTACTTTATCTTGCACTAATGTGACTAATAATAATAATACTGCCAGT------------------------------------------------------------------------------------AATAGTTCTGATTGGGAAAAGATGGAG---GGAGAAATAAAAAACTGCTCTTTCAATGTCACC---CCAAGCATAAGAGAT------AGGGTGCGCAAAGAATATGCACTCTTTTATAGCCTTGATGTAGTACCTATAAAGGATACT------------------------------AATGATAGT---------------------AGAACCTATAGATTAATAAATTGTAACACCTCAGTCATTACACAGGCCTGTCCAAAGGTATCCTTTGAGCCAATTCCCATACATTATTGTGCCCCGGCTGGTTATGCGATTCTAAAATGT---AATAATAAGACTTTCAATGGAACAGGACCATGTACAAATGTCAGCACAGTACAATGTACACATGGAATTAGGCCTGTAGTATCAACTCAACTGTTGTTAAATGGCAGCCTAGCAGAAGAA---GACATAGTAATCAGATCTGAAAATCTCACAGACAATGCTAAAACCATAATAGTACATCTGAATGAATCTGTAGAAATTAATTGTACAAGACCCAACAATAATACAAGAAAAAGTATAAATATAGGA------------CCAGGGGCA---GCGATGTATGCAACAGGAGCCATAATAGGAGATATAAGACAAGCACATTGTAACATT------AGTGGAGCAAGATGGAATGACACTTTAAAAAAGGTAGTCAAAAAATTAAGA---GAAAAATTTGGA------------AATAAAACA---ATAATCTTTGATCAA---------CACTCAGGAGGGGACCCAGAAATTGTAATGCACAGTTTTAATTGTGGAGGGGAATTTTTCTACTGTAATACAACAAAACTATTCAATAGTACTTGG---------------AATAGTACTAGG------------------------------AATGATACTGAAAGGAAT---------AGTAATGAAACT------------------------GACACAATCACACTCCCATGCAGAATAAAACAAATTATAAACATGTGGCAGGGAGTAGGAAAAGCAATGTATGCCCCTCCTATCAGAGGACTAATTAGATGTTCATCAAATATTACAGGGCTGTTATTAACAAGGGATGGTGGTAATACT------------AACAGGAGC---------------GGGAGCAACGGGTCT---GAGACCTTCAGACCTGGAGGAGGAAATATGAAGGACAATTGGAGA---AGTGAATTATATAAATATAAAGTAGTAAAACTTGAACCA---TTAGGAATAGCACCC---ACCAAGGCAAGGAGAAGAGTGGTGCAGAGAGAA---AAAAGAGCAGTA---GGA---CTAGGA---GCGTTG---TTCATT---GGG---------TTCTTGGGA---GCAGCAGGAAGCACTATGGGCGCAGCGTCAGTA---ACGCTGACGGTACAGGCCAGACAATTATTGTCTGGTATAGTGCAACAGCAGAACAATCTGCTGATGGCTATTGATGCACAACAGCATCTGTTGCAACTCACAGTCTGGGGCGTCAAGCAGCTCCGGGCAAGA---ATCCTGGCTGTGGAAAGATACCTAAAGGATCAACAGCTCCTAGGGATTTGGGGTTGCTCTGGAAAACTCATCTGCACCACTAATGTGCCTTGGAATGCTAGTTGGAGT---------------------------AATAAATCTCAAGAGCAAATTTGGGAA---AACATGACCTGGATGCAGTGGGAAAGAGAAATTGAC------AATTACACAGGCCTTATATACTCTTTAATTGAAAAATCGCAAAACCAACAAGAAAAGAATGAACAAGAATTATTGGCATTGGATAAGTGGGCAAGTTTGTGGAATTGGTTTGACATAACAAAATGGCTGTGGTATATAAAAATATTCATAATAATAGTAGGAGGCTTGATAGGATTAAGAATAGTTTTTGCTGTATTTTCTATAGTAAATAGAGTTAGGCAGGGATACTCACCACTATCGTTTCAGACCCAC---CTCCCAGTTCCGAGGGGA------CCCGACAGGCCCGAAGGAATCGACGAAGAAGGTGGAGAGCAAGGCAGAGACAGATCAATTCGATTAGTGGATGGATTCTTGGCACTTTTCTGGGACGACCTGAGGAGCCTGTGTCTTTTCAGCTACCACCACTTGAGAGACTTACTCTTGATTGCAGCGCGGATTGTGCAACTTCTGGGACAGAGG---------------GGGTGGGAGATCCTCAAATATTGGTGG---AATCTCCTGCTGTATTGG---------------------------------------------------AGTCAGGAACTAAAGAATAGTGCTGTCAGCTTGCTCAACACCACTGCTATAGTAGTAGCTGAGGGGACAGACAGGGTTATAGAAGCATTGCAAAGA------------------GCCTTTAGGGCTATTCTCCACATCCCTACAAGAATAAGACAAGGCTTGGAAAGGGCTTTACTATAA

2.04013226.ADARC.GU330375 ATGAAAGTGAAGGTGATCAGGAAGAATTGTCAGCACTTG---------TGGACATGGGGC------------------------ACGATGCTCCTTGGGATGTTAATGATC------------TGTAGTGCTGAA---------GATCAATTGTGAGTCACAGTTTATTATGGGGTACCTGTGTGGAAAGAAGCAACCACCACTCTATTTTGTGCATCAGATGCCAAAGGATATGATACAGAGGTACATAAT---GTTTGGGCCACACATGCCTGTGTACCCACAGACCCCAACCCACAAGAAGTGTTATTG---GCAAATGTGACAGAAAATTTTAACATGTGGAAAAATAACATGGTAGACCAGATGCATGAGGATGTAATCAGTTTATGGGATCAAAGTCTAAAGCCATGTGTAAAATTGACACCACTCTGTGTTACTTTATCTTGCACTAATGTGACTAATAATAATAATACTGCCAGT------------------------------------------------------------------------------------AATAGTTCTGATTGGGAAAAGATGGAG---GGAGAAATAAAAAACTGCTCTTTCAATGTCACC---CCAAGCATAAGAGAT------AGGGTGCGCAAAGAATATGCACTCTTTTATAGCCTTGATGTAGTACCTATAAAGGATACT------------------------------AATGATAGT---------------------AGAACCTATAGATTAATAAATTGTAACACCTCAGTCATTACACAGGCCTGTCCAAAGGTATCCTTTGAGCCAATTCCCATACATTATTGTGCCCCGGCTGGTTATGCGATTCTAAAATGT---AATAATAAGACTTTCAATGGAACAGGACCATGTACAAATGTCAGCACAGTACAATGTACACATGGAATTAGGCCTGTAGTATCAACTCAACTGTTGTTAAATGGCAGCCTAGCAGAAGAA---GACATAGTAATCAGATCTGAAAATCTCACAGACAATGCTAAAACCATAATAGTACATCTGAATGAATCTGTAGAAATTAATTGTACAAGACCCAACAATAATACAAGAAAAAGTATAAATATAGGA------------CCAGGGGCA---GCGATGTATGCAACAGGAGCCATAATAGGAGATATAAGACAAGCACATTGTAACATT------AGTGGAGCAAGATGGAATGACACTTTAAAAAAGGTAGTCAAAAAATTAAGA---GAAAAATTTGGA------------AATAAAACA---ATAATCTTTGATCAA---------CACTCAGGAGGGGACCCAGAAATTGTAATGCACAGTTTTAATTGTGGAGGGGAATTTTTCTACTGTAATACAACAAAACTATTCAATAGTACTTGG---------------AATAGTACTAGG------------------------------AATGATACTGAAAGGAAT---------AGTAATGAAACT------------------------GACACAATCACACTCCCATGCAGAATAAAACAAATTATAAACATGTGGCAGGGAGTAGGAAAAGCAATGTATGCCCCTCCTATCAGAGGACTAATTAGATGTTCATCAAATATTACAGGGCTGTTATTAACAAGGGATGGTGGTAATACT------------AACAGGAGC---------------GGGAGCAACGGGTCT---GAGACCTTCAGACCTGGAGGAGGAAATATGAAGGACAATTGGAGA---AGTGAATTATATAAATATAAAGTAGTAAAACTTGAACCA---TTAGGAATAGCACCC---ACCAAGGCAAGGAGAAGAGTGGTGCAGAGAGAA---AAAAGAGCAGTA---GGA---CTAGGA---GCGTTG---TTCATT---GGG---------TTCTTGGGA---GCAGCAGGAAGCACTATGGGCGCAGCGTCAGTA---ACGCTGACGGTACAGGCCAGACAATTATTGTCTGGTATAGTGCAACAGCAGAACAATCTGCTGATGGCTATTGATGCACAACAGCATCTGTTGCAACTCACAGTCTGGGGCGTCAAGCAGCTCCGGGCAAGA---ATCCTGGCTGTGGAAAGATACCTAAAGGATCAACAGCTCCTAGGGATTTGGGGTTGCTCTGGAAAACTCATCTGCACCACTAATGTGCCTTGGAATGCTAGTTGGAGT---------------------------AATAAATCTCAAGAGCAAATTTGGGAA---AACATGACCTGGATGCAGTGGGAAAGAGAAATTGAC------AATTACACAGGCCTTATATACTCTTTAATTGAAAAATCGCAAAACCAACAAGAAAAGAATGAACAAGAATTATTGGCATTGGATAAGTGGGCAAGTTTGTGGAATTGGTTTGACATAACAAAATGGCTGTGGTATATAAAAATATTCATAATAATAGTAGGAGGCTTGATAGGATTAAGAATAGTTTTTGCTGTATTTTCTATAGTAAATAGAGTTAGGCAGGGATACTCACCACTATCGTTTCAGACCCAC---CTCCCAGTTCCGAGGGGA------CCCGACAGGCCCGAAGGAATCGACGAAGAAGGTGGAGAGCAAGGCAGAGACAGATCAATTCGATTAGTGGATGGATTCTTGGCACTTTTCTGGGACGACCTGAGGAGCCTGTGTCTTTTCAGCTACCACCACTTGAGAGACTTACTCTTGATTGCAGCGCGGATTGTGCAACTTCTGGGACAGAGG---------------GGGTGGGAGATCCTCAAATATTGGTGG---AATCTCCTGCTGTATTGG---------------------------------------------------AGTCAGGAACTAAAGAATAGTGCTGTCAGCTTGCTCAACACCACTGCTATAGTAGTAGCTGAGGGGACAGACAGGGTTATAGAAGCATTGCAAAGA------------------GCCTTTAGGGCTATTCTCCACATCCCTACAAGAATAAGACAAGGCTTGGAAAGGGCTTTACTATAA

2.04013226.ADARC.GU330376 ATGAAAGTGAAGGTGATCAGGAAGAATTGTCAGCACTTG---------TGGACATGGGGC------------------------ACGATGCTCCTTGGGATGTTAATGATC------------TGTAGTGCTGAA---------GATCAATTGTGGGTCACAGTTTATTATGGGGTACCTGTGTGGAAAGAAGCAACCACCACTCTATTTTGTGCATCAGATGCCAAAGGATATGATACAGAGGTACATAAT---GTTTGGGCCACACATGCCTGTGTACCCACAGACCCCAACCCACAAGAAGTGTTATTG---GCAAATGTGACAGAAAATTTTAACATGTGGAAAAATAACATGGTAGACCAGATGCATGAGGATGTAATCAGTTTATGGGATCAAAGTCTAAAGCCATGTGTAAAATTGACACCACTCTGTGTTACTTTATCTTGCACTAATGTGACTAATAATAATAATACTGCCAGT------------------------------------------------------------------------------------AATAGTTCTGATTGGGAAAAGATGGAG---GGAGAAATAAAAAACTGCTCTTTCAATGTCACC---CCAAGCATAAGAGAT------AGGGTGCGCAAAGAATATGCACTCTTTTATAGCCTTGATGTAGTACCTATAAAGGATACT------------------------------AATGATAGT---------------------AGAACCTATAGATTAATAAATTGTAACACCTCAGTCATTACACAGGCCTGTCCAAAGGTATCCTTTGAGCCAATTCCCATACATTATTGTGCCCCGGCTGGTTATGCGATTCTAAAATGT---AATAATAAGACTTTCAATGGAACAGGACCATGTACAAATGTCAGCACAGTACAATGTACACATGGAATTAGGCCTGTAGTATCAACTCAACTGTTGTTAAATGGCAGCCTAGCAGAAGAA---GACATAGTAATCAGATCTGAAAATCTCACAGACAATGCTAAAACCATAATAGTACATCTGAATGAATCTGTAGAAATTAATTGTACAAGACCCAACAATAATACAAGAAAAAGTATAAATATAGGA------------CCAGGGGCA---GCGATGTATGCAACAGGAGCCATAATAGGAGATATAAGACAAGCACATTGTAACATT------AGTGGAGCAAGATGGAATGACACTTTAAAAAAGGTAGTCAAAAAATTAAGA---GAAAAATTTGGA------------AATAAAACA---ATAATCTTTGATCAA---------CACTCAGGAGGGGACCCAGAAATTGTAATGCACAGTTTTAATTGTGGAGGGGAATTTTTCTACTGTAATACAACAAAACTATTCAATAGTACTTGG---------------AATAGTACTAGG------------------------------AATGATACTGAAAGGAAT---------AGTAATGAAACT------------------------GACACAATCACACTCCCATGCAGAATAAAACAAATTATAAACATGTGGCAGGGAGTAGGAAAAGCAATGTATGCCCCTCCTATCAGAGGACTAATTAGATGTTCATCAAATATTACAGGGCTGTTATTAACAAGGGATGGTGGTAATACT------------AACAGGAGC---------------GGGAGCAACGGGTCT---GAGACCTTCAGACCTGGAGGAGGAAATATGAAGGACAATTGGAGA---AGTGAATTATATAAATATAAAGTAGTAAAACTTGAACCA---TTAGGAATAGCACCC---ACCAAGGCAAGGAGAAGAGTGGTGCAGAGAGAA---AAAAGAGCAGTA---GGA---CTAGGA---GCGTTG---TTCATT---GGG---------TTCTTGGGA---GCAGCAGGAAGCACTATGGGCGCAGCGTCAGTA---ACGCTGACGGTACAGGCCAGACAATTATTGTCTGGTATAGTGCAACAGCAGAACAATCTGCTGATGGCTATTGATGCACAACAGCATCTGTTGCAACTCACAGTCTGGGGCGTCAAGCAGCTCCGGGCAAGA---ATCCTGGCTGTGGAAAGATACCTAAAGGATCAACAGCTCCTAGGGATTTGGGGTTGCTCTGGAAAACTCATCTGCACCACTAATGTGCCTTGGAATGCTAGTTGGAGT---------------------------AATAAATCTCAAGAGCAAATTTGGGAA---AACATGACCTGGATGCAGTGGGAAAGAGAAATTGAC------AATTACACAGGCCTTATATACTCTTTAATTGAAAAATCGCAAAACCAACAAGAAAAGAATGAACAAGAATTATTGGCATTGGATAAGTGGGCAAGTTTGTGGAATTGGTTTGACATAACAAAATGGCTGTGGTATATAAAAATATTCATAATAATAGTAGGAGGCTTGATAGGATTAAGAATAGTTTTTGCTGTATTTTCTATAGTAAATAGAGTTAGGCAGGGATACTCACCACTATCGTTTCAGACCCAC---CTCCCAGTTCCGAGGGGA------CCCGACAGGCCCGAAGGAATCGACGAAGAAGGTGGAGAGCAAGGCAGAGACAGATCAATTCGATTAGTGGATGGATTCTTGGCACTTTTCTGGGACGACCTGAGGAGCCTGTGTCTTTTCAGCTACCACCACTTGAGAGACTTACTCTTGATTGCAGCGCGGATTGTGCAACTTCTGGGACAGAGG---------------GGGTGGGAGATCCTCAAATATTGGTGG---AATCTCCTGCTGTATTGG---------------------------------------------------AGTCAGGAACTAAAGAATAGTGCTGTCAGCTTGCTCAACACCACTGCTATAGTAGTAGCTGAGGGGACAGACAGGGTTATAGAAGCATTGCAAAGA------------------GCCTTTAGGGCTATTCTCCACATCCCTACAAGAAT-AGACAAGGCTTGGAAAGGGCTTTACTATAA

2.04013226.ADARC.GU330377 ATGAAAGTGAAGGTGATCAGGAAGAATTGTCAGCACTTG---------TGGACATGGGGC------------------------ACGATGCTCCTTGGGATGTTAATGATC------------TGTAGTGCTGAA---------GATCAATTGTGGGTCACAGTTTATTATGGGGTACCTGTGTGGAAAGAAGCAACCACCACTCTATTTTGTGCATCAGATGCCAAAGGATATGATACAGAGGTACATAAT---GTTTGGGCCACACATGCCTGTGTACCCACAGACCCCAACCCACAAGAAGTGTTATTG---GCAAATGTGACAGAAAATTTTAACATGTGGAAAAATAACATGGTAGACCAGATGCATGAGGATGTAATCAGTTTATGGGATCAAAGTCTAAAGCCATGTGTAAAATTGACACCACTCTGTGTTACTTTATCTTGCACTAATGTGACTAATAATAATAATACTGCCAGT------------------------------------------------------------------------------------AATAGTTCTGATTGGGAAAAGATGGAG---GGAGAAATAAAAAACTGCTCTTTCAATGTCACC---CCAAGCATAAGAGAT------AGGGTGCGCAAAGAATATGCACTCTTTTATAGCCTTGATGTAGTACCTATAAAGGATACT------------------------------AATGATAGT---------------------AGAACCTATAGATTAATAAATTGTAACACCTCAGTCATTACACAGGCCTGTCCAAAGGTATCCTTTGAGCCAATTCCCATACATTATTGTGCCCCGGCTGGTTATGCGATTCTAAAATGT---AATAATAAGACTTTCAATGGAACAGGACCATGTACAAATGTCAGCACAGTACAATGTACACATGGAATTAGGCCTGTAGTATCAACTCAACTGTTGTTAAATGGCAGCCTAGCAGAAGAA---GACATAGTAATCAGATCTGAAAATCTCACAGACAATGCTAAAACCATAATAGTACATCTGAATGAATCTGTAGAAATTAATTGTACAAGACCCAACAATAATACAAGAAAAAGTATAAATATAGGA------------CCAGGGGCA---GCGATGTATGCAACAGGAGCCATAATAGGAGATATAAGACAAGCACATTGTAACATT------AGTGGAGCAAGATGGAATGACACTTTAAAAAAGGTAGTCAAAAAATTAAGA---GAAAAATTTGGA------------AATAAAACA---ATAATCTTTGATCAA---------CACTCAGGAGGGGACCCAGAAATTGTAATGCACAGTTTTAATTGTGGAGGGGAATTTTTCTACTGTAATACAACAAAACTATTCAATAGTACTTGG---------------AATAGTACTAGG------------------------------AATGATACTGAAAGGAAT---------AGTAATGAAACT------------------------GACACAATCACACTCCCATGCAGAATAAAACAAATTATAAACATGTGGCAGGGAGTAGGAAAAGCAATGTATGCCCCTCCTATCAGAGGACTAATTAGATGTTCATCAAATATTACAGGGCTGTTATTAACAAGGGATGGTGGTAATACT------------AACAGGAGC---------------GGGAGCAACGGGTCT---GAGACCTTCAGACCTGGAGGAGGAAATATGAAGGACAATTGGAGA---AGTAAATTATATAAATATAAAGTAGTAAAACTTGAACCA---TTAGGAATAGCACCC---ACCAAGGCAAGGAGAAGAGTGGTGCAGAAAGAA---AAAAGAGCAGTA---GGA---CTAGGA---GCGTTG---TTCATT---GGG---------TTCTTGGGA---GCAGCAGGAAGCACTATGGGCGCAGCGTCAGTA---ACGCTGACGGTACAGGCCAGACAATTATTGTCTGGTATAGTGCAACAGCAGAACAATCTGCTGATGGCTATTGATGCACAACAGCATCTGTTGCAACTCACAGTCTGGGGCGTCAAGCAGCTCCGGGCAAGA---ATCCTGGCTGTGGAAAGATACCTAAAGGATCAACAGCTCCTAGGGATTTGGGGTTGCTCTGGAAAACTCATCTGCACCACTAATGTGCCTTGGAATGCTAGTTGGAGT---------------------------AATAAATCTCAAGAGCAAATTTGGGAA---AACATGACCTGGATGCAGTGGAAAAGAGAAATTGAC------AATTACACAGGCCTTATATACTCTTTAATTGAAAAATCGCAAAACCAACAAGAAAAGAATGAACAAGAATTATTGGCATTGGATAAGTGGGCAAGTTTGTGGAATTGGTTTGACATAACAAAATGGCTGTGGTATATAAAAATATTCATAATAATAGTAGGAGGCTTGATAGGATTAAGAATAGTTTTTGCTGTATTTTCTATAGTAAATAGAGTTAGGCAGGGATACTCACCACTATCGTTTCAGACCCAC---CTCCCAGTTCCGAGGGGA------CCCGACAGGCCCGAAGGAATCGACGAAGAAGGTGGAGAGCAAGGCAGAGACAGATCAATTCGATTAGTGGATGGATTCTTGGCACTTTTCTGGGACGACCTGAGGAGCCTGTGTCTTTTCAGCTACCACCACTTGAGAGACTTACTCTTGATTGCAGCGCGGATTGTGCAACTTCTGGGACAGAGG---------------GGGTGGGAGATCCTCAAATATTGGTGG---AATCTCCTGCTGTATTGG---------------------------------------------------AGTCAGGAACTAAAGAATAGTGCTGTCAGCTTGCTCAACACCACTGCTATAGTAGTAGCTGAGGGGACAGACAGGGTTATAGAAGCATTGCAAAGA------------------GCCTTTAGGGCTATTCTCCACATCCCTACAAGAATAAGACAAGGCTTGGAAAGGGCTTTACTATAA

2.04013226.ADARC.GU330378 ATGAAAGTGAAGGTGATCAGGAAGAATTGTCAGCACTTG---------TGGACATGGGGC------------------------ACGATGCTCCTTGGGATGTTAATGATC------------TGTAGTGCTGAA---------GATCAATTGTGGGTCACAGTTTATTATGGGGTACCTGTGTGGAAAGAAGCAACCACCACTCTATTTTGTGCATCAGATGCCAAAGGATATGATACAGAGGTACATAAT---GTTTGGGCCACACATGCCTGTGTACCCACAGACCCCAACCCACAAGAAGTGTTATTG---GCAAATGTGACAGAAAATTTTAACATGTGGAAAAATAACATGGTAGACCAGATGCATGAGGATGTAATCAGTTTATGGGATCAAAGTCTAAAGCCATGTGTAAAATTGACACCACTCTGTGTTACTTTATCTTGCACTAATGTGACTAATAATAATAATACTGCCAGT------------------------------------------------------------------------------------AATAGTTCTGATTGGGAAAAGATGGAG---GGAGAAATAAAAAACTGCTCTTTCAATGTCACC---CCAAGCATAAGAGAT------AGGGTGCGCAAAGAATATGCACTCTTTTATAGCCTTGATGTAGTACCTATAAAGGATACT------------------------------AATGATAGT---------------------AGAACCTATAGATTAATAAATTGTAACACCTCAGTCATTACACAGGCCTGTCCAAAGGTATCCTTTGAGCCAATTCCCATACATTATTGTGCCCCGGCTGGTTATGCGATTCTAAAATGT---AATAATAAGACTTTCAATGGAACAGGACCATGTACAAATGTCAGCACAGTACAATGTACACATGGAATTAGGCCTGTAGTATCAACTCAACTGTTGTTAAATGGCAGCCTAGCAGAAGAA---GACATAGTAATCAGATCTGAAAATCTCACAGACAATGCTAAAACCATAATAGTACATCTGAATGAATCTGTAGAAATTAATTGTACAAGACCCAACAATAATACAAGAAAAAGTATAAATATAGGA------------CCAGGGGCA---GCGATGTATGCAACAGGAGCCATAATAGGAGATATAAGACAAGCACATTGTAACATT------AGTGGAGCAAGATGGAATGACACTTTAAAAAAGGTAGTCAAAAAATTAAGA---GAAAAATTTGGA------------AATAAAACA---ATAATCTTTGATCAA---------CACTCAGGAGGGGACCCAGAAATTGTAATGCACAGTTTTAATTGTGGAGGGGAATTTTTCTACTGTAATACAACAAAACTATTCAATAGTACTTGG---------------AATAGTACTAGG------------------------------AATGATACTGAAAGGAAT---------AGTAATGAAACT------------------------GACACAATCACACTCCCATGCAGAATAAAACAAATTATAAACATGTGGCAGGGAGTAGGAAAAGCAATGTATGCCCCTCCTATCAGAGGACTAATTAGATGTTCATCAAATATTACAGGGCTGTTATTAACAAGGGATGGTGGTAATACT------------AACAGGAGC---------------GGGAGCAACGGGTCT---GAGACCTTCAGACCTGGAGGAGGAAATATGAAGGACAATTGGAGA---AGTGAATTATATAAATATAAAGTAGTAAAACTTGAACCA---TTAGGAATAGCACCC---ACCAAGGCAAGGAGAAGAGTGGTGCAGAGAGAA---AAAAGAGCAGTA---GGA---CTAGGA---GCGTTG---TTCATT---GGG---------TTCTTGGGA---GCAGCAGGAAGCACTATGGGCGCAGCGTCAGTA---ACGCTGACGGTACAGGCCAGACAATTATTGTCTGGTATAGTGCAACAGCAGAACAATCTGCTGATGGCTATTGATGCACAACAGCATCTGTTGCAACTCACAGTCTGGGGCGTCAAGCAGCTCCGGGCAAGA---ATCCTGGCTGTGGAAAGATACCTAAAGGATCAACAGCTCCTAGGGATTTGGGGTTGCTCTGGAAAACTCATCTGCACCACTAATGTGCCTTGGAATGCTAGTTGGAGT---------------------------AATAAATCTCAAGAGCAAATTTGGGAA---AACATGACCTGGATGCAGTGGGAAAGAGAAATTGAC------AATTACACAGGCCTTATATACTCTTTAATTGAAAAATCGCAAAACCAACAAGAAAAGAATGAACAAGAATTATTGGCATTGGATAAGTGGGCAAGTTTGTGGAATTGGTTTGACATAACAAAATGGCTGTGGTATATAAAAATATTCATAATAATAGTAGGAGGCTTGATAGGATTAAGAATAGTTTTTGCTGTATTTTCTATAGTAAATAGAGTTAGGCAGGGATACTCACCACTATCGTTTCAGACCCAC---CTCCCAGTTCCGAGGGGA------CCCGACAGGCCCGAAGGAATCGACGAAGAAGGTGGAGAGCAAGGCAGAGACAGATCAATTCGATTAGTGGATGGATTCTTGGCACTTTTCTGGGACGACCTGAGGAGCCTGTGTCTTTTCAGCTACCACCACTTGAGAGACTTACTCTTGATTGCAGCGCGGATTGTGCAACTTCTGGGACAGAGG---------------GGGTGGGAGATCCTCAAATATTGGTGG---AATCTCCTGCTGTATTGG---------------------------------------------------AGTCAGGAACTAAAGAATAGTGCTGTCAGCTTGCTCAACACCACTGCTATAGTAGTAGCTGAGGGGACAGACAGGGTTATAGAAGCATTGCAAAGA------------------GCCTTTAGGGCTATTCTCCACATCCCTACAAGAATAAGACAAGGCTTGGAAAGGGCTTTACTATAA

2.04013226.ADARC.GU330379 ATGAAAGTGAAGGTGATCAGGAAGAATTGTCAGCACTTG---------TGGACATGGGGC------------------------ACGATGCTCCTTGGGATGTTAATGATC------------TGTAGTGCTGAA---------GATCAATTGTGGGTCACAGTTTATTATGGGGTACCTGTGTGGAAAGAAGCAACCACCACTCTATTTTGTGCATCAGATGCCAAAGGATATGATACAGAGGTACATAAT---GTTTGGGCCACACATGCCTGTGTACCCACAGACCCCAACCCACAAGAAGTGTTATTG---GCAAATGTGACAGAAAATTTTAACATGTGGAAAAATAACATGGTAGACCAGATGCATGAGGATGTAATCAGTTTATGGGATCAAAGTCTAAAGCCATGTGTAAAATTGACACCACTCTGTGTTACTTTATCTTGCACTAATGTGACTAATAATAATAATACTGCCAGT------------------------------------------------------------------------------------AATAGTTCTGATTGGGAAAAGATGGAG---GGAGAAATAAAAAACTGCTCTTTCAATGTCACC---CCAAGCATAAGAGAT------AGGGTGCGCAAAGAATATGCACTCTTTTATAGCCTTGATGTAGTACCTATAAAGGATACT------------------------------AATGATAGT---------------------AGAACCTATAGATTAATAAATTGTAACACCTCAGTCATTACACAGGCCTGTCCAAAGGTATCCTTTGAGCCAATTCCCATACATTATTGTGCCCCGGCTGGTTATGCGATTCTAAAATGT---AATAATAAGACTTTCAATGGAACAGGACCATGTACAAATGTCAGCACAGTACAATGTACACATGGAATTAGGCCTGTAGTATCAACTCAACTGTTGTTAAATGGCAGCCTAGCAGAAGAA---GACATAGTAATCAGATCTGAAAATCTCACAGACAATGCTAGAACCATAATAGTACATCTGAATGAATCTGTAGAAATTAATTGTACAAGACCCAACAATAATACAAGAAAAAGTATAAATATAGGA------------CCAGGGGCA---GCGATGTATGCAACAGGAGCCATAATAGGAGATATAAGACAAGCACATTGTAACATT------AGTGGAGCAAGATGGAATGACACTTTAAAAAAGGTAGTCAAAAAATTAAGA---GAAAAATTTGGA------------AATAAAACA---ATAATCTTTGATCAA---------CACTCAGGAGGGGACCCAGAAATTGTAATGCACAGTTTTAATTGTGGAGGGGAATTTTTCTACTGTAATACAACAAAACTATTCAATAGTACTTGG---------------AATAGTACTAGG------------------------------AATGATACTGAAAGGAAT---------AGTAATGAAACT------------------------GACACAATCACACTCCCATGCAGAATAAAACAAATTATAAACATGTGGCAGGGAGTAGGAAAAGCAATGTATGCCCCTCCTATCAGAGGACTAATTAGATGTTCATCAAATATTACAGGGCTGTTATTAACAAGGGATGGTGGTAATACT------------AACAGGAGC---------------GGGAGCAACGGGTCT---GAGACCTTCAGACCTGGAGGAGGAAATATGAAGGACAATTGGAGA---AGTGAATTATATAAATATAAAGTAGTAAAACTTGAACCA---TTAGGAATAGCACCC---ACCAAGGCAAGGAGAAGAGTGGTGCAGAGAGAA---AAAAGAGCAGTA---GGA---CTAGGA---GCGTTG---TTCATT---GGG---------TTCTTGGGA---GCAGCAGGAAGCACTATGGGCGCAGCGTCAGTA---ACGCTGACGGTACAGGCCAGACAATTATTGTCTGGTATAGTGCAACAGCAGAACAATCTGCTGATGGCTATTGATGCACAACAGCATCTGTTGCAACTCACAGTCTGGGGCGTCAAGCAGCTCCGGGCAAGA---ATCCTGGCTGTGGAAAGATACCTAAAGGATCAACAGCTCCTAGGGATTTGGGGTTGCTCTGGAAAACTCATCTGCACCACTAATGTGCCTTGGAATGCTAGTTGGAGT---------------------------AATAAATCTCAAGAGCAAATTTGGGAA---AACATGACCTGGATGCAGTGGGAAAGAGAAATTGAC------AATTACACAGGCCTTATATACTCTTTAATTGAAAAATCGCAAAACCAACAAGAAAAGAATGAACAAGAATTATTGGCATTGGATAAGTGGGCAAGTTTGTGGAATTGGTTTGACATAACAAAATGGCTGTGGTATATAAAAATATTCATAATAATAGTAGGAGGCTTGATAGGATTAAGAATAGTTTTTGCTGTATTTTCTATAGTAAATAGAGTTAGGCAGGGATACTCACCACTATCGTTTCAGACCCAC---CTCCCAGTTCCGAGGGGA------CCCGACAGGCCCGAAGGAATCGACGAAGAAGGTGGAGAGCAAGGCAGAGACAGATCAATTCGATTAGTGGATGGATTCTTGGCACTTTTCTGGGACGACCTGAGGAGCCTGTGTCTTTTCAGCTACCACCACTTGAGAGACTTACTCTTGATTGCAGCGCGGATTGTGCAACTTCTGGGACAGAGG---------------GGGTGGGAGATCCTCAAATATTGGTGG---AATCTCCTGCTGTATTGG---------------------------------------------------AGTCAGGAACTAAAGAATAGTGCTGTCAGCTTGCTCAACACCACTGCTATAGTAGTAGCTGAGGGGACAGACAGGGTTATAGAAGCATTGCAAAGA------------------GCCTTTAGGGCTATTCTCCACATCCCTACAAGAATAAGACAAGGCTTGGAAAGGGCTTTACTATAA

2.04013226.ADARC.GU330380 ATGAAAGTGAAGGTGATCAGGAAGAATTGTCAGCACTTG---------TGGACATGGGGC------------------------ACGATGCTCCTTGGGATGTTAATGATC------------TGTAGTGCTGAA---------GATCAATTGTGGGTCACAGTTTATTATGGGGTACCTGTGTGGAAAGAAGCAACCACCACTCTATTTTGTGCATCAGATGCCAAAGGATATGATACAGAGGTACATAAT---GTTTGGGCCACACATGCCTGTGTACCCACAGACCCCAACCCACAAGAAGTGTTATTG---GCAAATGTGACAGAAAATTTTAACATGTGGAAAAATAACATGGTAGACCAGATGCATGAGGATGTAATCAGTTTATGGGATCAAAGTCTAAAGCCATGTGTAAAATTGACACCACTCTGTGTTACTTTATCTTGCACTAATGTGACTAATAATAATAATACTGCCAGT------------------------------------------------------------------------------------AATAGTTCTGATTGGGAAAAGATGGAG---GGAGAAATAAAAAACTGCTCTTTCAATGTCACC---CCAAGCATAAGAGAT------AGGGTGCGCAAAGAATATGCACTCTTTTATAGCCTTGATGTAGTACCTATAAAGGATACT------------------------------AATGATAGT---------------------AGAACCTATAGATTAATAAATTGTAACACCTCAGTCATTACACAGGCCTGTCCAAAGGTATCCTTTGAGCCAATTCCCATACATTATTGTGCCCCGGCTGGTTATGCGATTCTAAAATGT---AATAATAAGACTTTCAATGGAACAGGACCATGTACAAATGTCAGCACAGTACAATGTACACATGGAATTAGGCCTGTAGTATCAACTCAACTGTTGTTAAATGGCAGCCTAGCAGAAGAA---GACATAGTAATCAGATCTGAAAATCTCACAGACAATGCTAAAACCATAATAGTACATCTGAATGAATCTGTAGAAATTAATTGTACAAGACCCAACAATAATACAAGAAAAAGTATAAATATAGGA------------CCAGGGGCA---GCGATGTATGCAACAGGAGCCATAATAGGAGATATAAGACAAGCACATTGTAACATT------AGTGGAGCAAGATGGAATGACACTTTAAAAAAGGTAGTCAAAAAATTAAGA---GAAAAATTTGGA------------AATAAAACA---ATAATCTTTGATCAA---------CACTCAGGAGGGGACCCAGAAATTGTAATGCACAGTTTTAATTGTGGAGGGGAATTTTTCTACTGTAATACAACAAAACTATTCAATAGTACTTGG---------------AATAGTACTAGG------------------------------AATGATACTGAAAGGAAT---------AGTAATGAAACT------------------------GACACAATCACACTCCCATGCAGAATAAAACAAATTATAAACATGTGGCAGGGAGTAGGAAAAGCAATGTATGCCCCTCCTATCAGAGGACTAATTAGATGTTCATCAAATATTACAGGGCTGTTATTAACAAGGGATGGTGGTAATACT------------AACAGGAGC---------------GGGAGCAACGGGTCT---GAGACCTTCAGACCTGGAGGAGGAAATATGAAGGACAATTGGAGA---AGTGAATTATATAAATATAAAGTAGTAAAACTTGAACCA---TTAGGAATAGCACCC---ACCAAGGCAAGGAGAAGAGTGGTGCAGAGAGAA---AAAAGAGCAGTA---GGA---CTAGGA---GCGTTG---TTCATT---GGG---------TTCTTGGGA---GCAGCAGGAAGCACTATGGGCGCAGCGTCAGTA---ACGCTGACGGTACAGGCCAGACAATTATTGTCTGGTATAGTGCAACAGCAGAACAATCTGCTGATGGCTATTGATGCACAACAGCATCTGTTGCAACTCACAGTCTGGGGCGTCAAGCAGCTCCGGGCAAGA---ATCCTGGCTGTGGAAAGATACCTAAAGGATCAACAGCTCCTAGGGATTTGGGGTTGCTCTGGAAAACTCATCTGCACCACTAATGTGCCTTGGAATGCTAGTTGGAGT---------------------------AATAAATCTCAAGAGCAAATTTGGGAA---AACATGACCTGGATGCAGTGGGAAAGAGAAATTGAC------AATTACACAGGCCTTATATACTCTTTAATTGAAAAATCGCAAAACCAACAAGAAAAGAATGAACAAGAATTATTGGCATTGGATAAGTGGGCAAGTTTGTGGAATTGGTTTGACATAACAAAATGGCTGTGGTATATAAAAATATTCATAATAATAGTAGGAGGCTTGATAGGATTAAGAATAGTTTTTGCTGTATTTTCTATAGTAAATAGAGTTAGGCAGGGATACTCACCACTATCGTTTCAGACCCAC---CTCCCAGTTCCGAGGGGA------CCCGACAGGCCCGAAGGAATCGACGAAGAAGGTGGAGAGCAAGGCAGAGACAGATCAATTCGATTAGTGGATGGATTCTTGGCACTTTTCTGGGACGACCTGAGGAGCCTGTGTCTTTTCAGCTACCACCACTTGAGAGACTTACTCTTGATTGCAGCGCGGATTGTGCAACTTCTGGGACAGAGG---------------GGGTGGGAGATCCTCAAATATTGGTGG---AATCTCCTGCTGTATTGG---------------------------------------------------AGTCAGGAACTAAAGAATAGTGCTGTCAGCTTGCTCAACACCACTGCTATAGTAGTAGCTGAGGGGACAGACAGGGTTATAGAAGCATTGCAAAGA------------------GCCTTTAGGGCTATTCTCCACATCCCTACAAGAATAAGACAAGGCTTGGAAAGGGCTTTACTATAA

2.04013226.ADARC.GU330381 ATGAAAGTGAAGGTGATCAGGAAGAATTGTCAGCACTTG---------TGGACATGGGGC------------------------ACGATGCTCCTTGGGATGTTAATGATC------------TGTAGTGCTGAA---------GATCAATTGTGGGTCACAGTTTATTATGGGGTACCTGTGTGGAAAGAAGCAACCACCACTCTATTTTGTGCATCAGATGCCAAAGGATATGATACAGAGGTACATAAT---GTTTGGGCCACACATGCCTGTGTACCCACAGACCCCAACCCACAAGAAGTGTTATTG---GCAAATGTGACAGAAAATTTTAACATGTGGAAAAATAACATGGTAGACCAGATGCATGAGGATGTAATCAGTTTATGGGATCAAAGTCTAAAGCCATGTGTAAAATTGACACCACTCTGTGTTACTTTATCTTGCACTAATGTGACTAATAATAATAATACTGCCAGT------------------------------------------------------------------------------------AATAGTTCTGATTGGGAAAAGATGGAG---GGAGAAATAAAAAACTGCTCTTTCAATGTCACC---CCAAGCATAAGAGAT------AGGGTGCGCAAAGAATATGCACTCTTTTATAGCCTTGATGTAGTACCTATAAAGGATACT------------------------------AATGATAGT---------------------AGAACCTATAGATTAATAAATTGTAACACCTCAGTCATTACACAGGCCTGTCCAAAGGTATCCTTTGAGCCAATTCCCATACATTATTGTGCCCCGGCTGGTTATGCGATTCTAAAATGT---AATAATAAGACTTTCAATGGAACAGGACCATGTACAAATGTCAGCACAGTACAATGTACACATGGAATTAGGCCTGTAGTATCAACTCAACTGTTGTTAAATGGCAGCCTAGCAGAAGAA---GACATAGTAATCAGATCTGAAAATCTCACAGACAATGCTAGAACCATAATAGTACATCTGAATGAATCTGTAGAAATTAATTGTACAAGACCCAACAATAATACAAGAAAAAGTATAAATATAGGA------------CCAGGGGCA---GCGATGTATGCAACAGGAGCCATAATAGGAGATATAAGACAAGCACATTGTAACATT------AGTGGAGCAAGATGGAATGACACTTTAAAAAAGGTAGTCAAAAAATTAAGA---GAAAAATTTGGA------------AATAAAACA---ATAATCTTTGATCAA---------CACTCAGGAGGGGACCCAGAAATTGTAATGCACAGTTTTAATTGTGGAGGGGAATTTTTCTACTGTAATACAACAAAACTATTCAATAGTACTTGG---------------AATAGTACTAGG------------------------------AATGATACTGAAAGGAAT---------AGTAATGAAACT------------------------GACACAATCACACTCCCATGCAGAATAAAACAAATTATAAACATGTGGCAGGGAGTAGGAAAAGCAATGTATGCCCCTCCTATCAGAGGACTAATTAGATGTTCATCAAATATTACAGGGCTGTTATTAACAAGGGATGGTGGTAATACT------------AACAGGAGC---------------GGGAGCAACGGGTCT---GAGACCTTCAGACCTGGAGGAGGAAATATGAAGGACAATTGGAGA---AGTGAATTATATAAATATAAAGTAGTAAAACTTGAACCA---TTAGGAATAGCACCC---ACCAAGGCAAGGAGAAGAGTGGTGCAGAGAGAA---AAAAGAGCAGTA---GGA---CTAGGA---GCGTTG---TTCATT---GGG---------TTCTTGGGA---GCAGCAGGAAGCACTATGGGCGCAGCGTCAGTA---ACGCTGACGGTACAGGCCAGACAATTATTGTCTGGTATAGTGCAACAGCAGAACAATCTGCTGATGGCTATTGATGCACAACAGCATCTGTTGCAACTCACAGTCTGGGGCGTCAAGCAGCTCCGGGCAAGA---ATCCTGGCTGTGGAAAGATACCTAAAGGATCAACAGCTCCTAGGGATTTGGGGTTGCTCTGGAAAACTCATCTGCACCACTAATGTGCCTTGGAATGCTAGTTGGAGT---------------------------AATAAATCTCAAGAGCAAATTTGGGAA---AACATGACCTGGATGCAGTGGGAAAGAGAAATTGAC------AATTACACAGGCCTTATATACTCTTTAATTGAAAAATCGCAAAACCAACAAGAAAAGAATGAACAAGAATTATTGGCATTGGATAAGTGGGCAAGTTTGTGGAATTGGTTTGACATAACAAAATGGCTGTGGTATATAAAAATATTCATAATAATAGTAGGAGGCTTGATAGGATTAAGAATAGTTTTTGCTGTATTTTCTATAGTAAATAGAGTTAGGCAGGGATACTCACCACTATCGTTTCAGACCCAC---CTCCCAGTTCCGAGGGGA------CCCGACAGGCCCGAAGGAATCGACGAAGAAGGTGGAGAGCAAGGCAGAGACAGATCAATTCGATTAGTGGATGGATTCTTGGCACTTTTCTGGGACGACCTGAGGAGCCTGTGTCTTTTCAGCTACCACCACTTGAGAGACTTACTCTTGATTGCAGCGCGGATTGTGCAACTTCTGGGACAGAGG---------------GGGTGGGAGATCCTCAAATATTGGTGG---AATCTCCTGCTGTATTGG---------------------------------------------------AGTCAGGAACTAAAGAATAGTGCTGTCAGCTTGCTCAACACCACTGCTATAGTAGTAGCTGAGGGGACAGACAGGGTTATAGAAGCATTGCAAAGA------------------GCCTTTAGGGCTATTCTCCACATCCCTACAAGAATAAGACAAGGCTTGGAAAGGGCTTTACTATAA

2.04013226.ADARC.GU330382 ATGAAAGTGAAGGTGATCAGGAAGAATTGTCAGCACTTG---------TGGACATGGGGC------------------------ACGATGCTCCTTGGGATGTTAATGATC------------TGTAGTGCTGAA---------GATCAATTGTGGGTCACAGTTTATTATGGGGTACCTGTGTGGAAAGAAGCAACCACCACTCTATTTTGTGCATCAGATGCCAAAGGATATGATACAGAGGTACATAAT---GTTTGGGCCACACATGCCTGTGTACCCACAGACCCCAACCCACAAGAAGTGTTATTG---GCAAATGTGACAGAAAATTTTAACATGTGGAAAAATAACATGGTAGACCAGATGCATGAGGATGTAATCAGTTTATGGGATCAAAGTCTAAAGCCATGTGTAAAATTGACACCACTCTGTGTTACTTTATCTTGCACTAATGTGACTAATAATAATAATACTGCCAGT------------------------------------------------------------------------------------AATAGTTCTGATTGGGAAAAGATGGAG---GGAGAAATAAAAAACTGCTCTTTCAATGTCACC---CCAAGCATAAGAGAT------AGGGTGCGCAAAGAATATGCACTCTTTTATAGCCTTGATGTAGTACCTATAAAGGATACT------------------------------AATGATAGT---------------------AGAACCTATAGATTAATAAATTGTAACACCTCAGTCATTACACAGGCCTGTCCAAAGGTATCCTTTGAGCCAATTCCCATACATTATTGTGCCCCGGCTGGTTATGCGATTCTAAAATGT---AATAATAAGACTTTCAATGGAACAGGACCATGTACAAATGTCAGCACAGTACAATGTACACATGGAATTAGGCCTGTAGTATCAACTCAACTGTTGTTAAATGGCAGCCTAGCAGAAGAA---GACATAGTAATCAGATCTGAAAATCTCACAGACAATGCTAAAACCATAATAGTACATCTGAATGAATCTGTAGAAATTAATTGTACAAGACCCAACAATAATACAAGAAAAAGTATAAATATAGGA------------CCAGGGGCA---GCGATGTATGCAACAGGAGCCATAATAGGAGATATAAGACAAGCACATTGTAACATT------AGTGGAGCAAGATGGAATGACACTTTAAAAAAGGTAGTCAAAAAATTAAGA---GAAAAATTTGGA------------AATAAAACA---ATAATCTTTGATCAA---------CACTCAGGAGGGGACCCAGAAATTGTAATGCACAGTTTTAATTGTGGAGGGGAATTTTTCTACTGTAATACAACAAAACTATTCAATAGTACTTGG---------------AATAGTACTAGG------------------------------AATGATACTGAAAGGAAT---------AGTAATGAAACT------------------------GACACAATCACACTCCCATGCAGAATAAAACAAATTATAAACATGTGGCAGGGAGTAGGAAAAGCAATGTATGCCCCTCCTATCAGAGGACTAATTAGATGTTCATCAAATATTACAGGGCTGTTATTAACAAGGGATGGTGGTAATACT------------AACAGGAGC---------------GGGAGCAACGGGTCT---GAGACCTTCAGACCTGGAGGAGGAAATATGAAGGACAATTGGAGA---AGTGAATTATATAAATATAAAGTAGTAAAACTTGAACCA---TTAGGAATAGCACCC---ACCAAGGCAAGGAGAAGAGTGGTGCAGAGAGAA---AAAAGAGCAGTA---GGA---CTAGGA---GCGTTG---TTCATT---GGG---------TTCTTGGGA---GCAGCAGGAAGCACTATGGGCGCAGCGTCAGTA---ACGCTGACGGTACAGGCCAGACAATTATTGTCTGGTATAGTGCAACAGCAGAACAATCTGCTGATGGCTATTGATGCACAACAGCATCTGTTGCAACTCACAGTCTGGGGCGTCAAGCAGCTCCGGGCAAGA---ATCCTGGCTGTGGAAAGATACCTAAAGGATCAACAGCTCCTAGGGATTTGGGGTTGCTCTGGAAAACTCATCTGCACCACTAATGTGCCTTGGAATGCTAGTTGGAGT---------------------------AATAAATCTCAAGAGCAAATTTGGGAA---AACATGACCTGGATGCAGTGGGAAAGAGAAATTGAC------AATTACACAGGCCTTATATACTCTTTAATTGAAAAATCGCAAAACCAACAAGAAAAGAATGAACAAGAATTATTGGCATTGGATAAGTGGGCAAGTTTGTGGAATTGGTTTGACATAACAAAATGGCTGTGGTATATAAAAATATTCATAATAATAGTAGGAGGCTTGATAGGATTAAGAATAGTTTTTGCTGTATTTTCTATAGTAAATAGAGTTAGGCAGGGATACTCACCACTATCGTTTCAGACCCAC---CTCCCAGTTCCGAGGGGA------CCCGACAGGCCCGAAGGAATCGACGAAGAAGGTGGAGAGCAAGGCAGAGACAGATCAATTCGATTAGTGGATGGATTCTTGGCACTTTTCTGGGACGACCTGAGGAGCCTGTGTCTTTTCAGCTACCACCACTTGAGAGACTTACTCTTGATTGCAGCGCGGATTGTGCAACTTCTGGGACAGAGG---------------GGGTGGGAGATCCTCAAATATTGGTGG---AATCTCCTGCTGTATTGG---------------------------------------------------AGTCAGGAACTAAAGAATAGTGCTGTCAGCTTGCTCAACACCACTGCTATAGTAGTAGCTGAGGGGACAGACAGGGTTATAGAAGCATTGCAAAGA------------------GCCTTTAGGGCTATTCTCCACATCCCTACAAGAATAAGACAAGGCTTGGAAAGGGCTTTACTATAA

2.04013226.ADARC.GU330383 ATGAAAGTGAAGGTGATCAGGAAGAATTGTCAGCACTTG---------TGGACATGGGGC------------------------ACGATGCTCCTTGGGATGTTAATGATC------------TGTAGTGCTGAA---------GATCAATTGTGGGTCACAGTTTATTATGGGGTACCTGTGTGGAAAGAAGCAACCACCACTCTATTTTGTGCATCAGATGCCAAAGGATATGATACAGAGGTACATAAT---GTTTGGGCCACACATGCCTGTGTACCCACAGACCCCAACCCACAAGAAGTGTTATTG---GCAAATGTGACAGAAAATTTTAACATGTGGAAAAATAACATGGTAGACCAGATGCATGAGGATGTAATCAGTTTATGGGATCAAAGTCTAAAGCCATGTGTAAAATTGACACCACTCTGTGTTACTTTATCTTGCACTAATGTGACTAATAATAATAATACTGCCAGT------------------------------------------------------------------------------------AATAGTTCTGATTGGGAAAAGATGGAG---GGAGAAATAAAAAACTGCTCTTTCAATGTCACC---CCAAGCATAAGAGAT------AGGGTGCGCAAAGAATATGCACTCTTTTATAGCCTTGATGTAGTACCTATAAAGGATACT------------------------------AATGATAGT---------------------AGAACCTATAGATTAATAAATTGTAACACCTCAGTCATTACACAGGCCTGTCCAAAGGTATCCTTTGAGCCAATTCCCATACATTATTGTGCCCCGGCTGGTTATGCGATTCTAAAATGT---AATAATAAGACTTTCAATGGAACAGGACCATGTACAAATGTCAGCACAGTACAATGTACACATGGAATTAGGCCTGTAGTATCAACTCAACTGTTGTTAAATGGCAGCCTAGCAGAAGAA---GACATAGTAATCAGATCTGAAAATCTCACAGACAATGCTAAAACCATAATAGTACATCTGAATGAATCTGTAGAAATTAATTGTACAAGACCCAACAATAATACAAGAAAAAGTATAAATATAGGA------------CCAGGGGCA---GCGATGTATGCAACAGGAGCCATAATAGGAGATATAAGACAAGCACATTGTAACATT------AGTGGAGCAAGATGGAATGACACTTTAAAAAAGGTAGTCAAAAAATTAAGA---GAAAAATTTGGA------------AATAAAACA---ATAATCTTTGATCAA---------CACTCAGGAGGGGACCCAGAAATTGTAATGCACAGTTTTAATTGTGGAGGGGAATTTTTCTACTGTAATACAACAAAACTATTCAATAGTACTTGG---------------AATAGTACTAGG------------------------------AATGATACTGAAAGGAAT---------AGTAATGAAACT------------------------GACACAATCACACTCCCATGCAGAATAAAACAAATTATAAACATGTGGCAGGGAGTAGGAAAAGCAATGTATGCCCCTCCTATCAGAGGACTAATTAGATGTTCATCAAATATTACAGGGCTGTTATTAACAAGGGATGGTGGTAAGACT------------AACAGGAGC---------------GGGAGCAACGGGTCT---GAGACCTTCAGACCTGGAGGAGGAAATATGAAGGACAATTGGAGA---AGTGAATTATATAAATATAAAGTAGTAAAACTTGAACCA---TTAGGAATAGCACCC---ACCAAGGCAAGGAGAAGAGTGGTGCAGAGAGAA---AAAAGAGCAGTA---GGA---CTAGGA---GCGTTG---TTCATT---GGG---------TTCTTGGGA---GCAGCAGGAAGCACTATGGGCGCAGCGTCAGTA---ACGCTGACGGTACAGGCCAGACAATTATTGTCTGGTATAGTGCAACAGCAGAACAATCTGCTGATGGCTATTGATGCACAACAGCATCTGTTGCAACTCACAGTCTGGGGCGTCAAGCAGCTCCGGGCAAGA---ATCCTGGCTGTGGAAAGATACCTAAAGGATCAACAGCTCCTAGGGATTTGGGGTTGCTCTGGAAAACTCATCTGCACCACTAATGTGCCTTGGAATGCTAGTTGGAGT---------------------------AATAAATCTCAAGAGCAAATTTGGGAA---AACATGACCTGGATGCAGTGGGAAAGAGAAATTGAC------AATTACACAGGCCTTATATACTCTTTAATTGAAAAATCGCAAAACCAACAAGAAAAGAATGAACAAGAATTATTGGCATTGGATAAGTGGGCAAGTTTGTGGAATTGGTTTGACATAACAAAATGGCTGTGGTATATAAAAATATTCATAATAATAGTAGGAGGCTTGATAGGATTAAGAATAGTTTTTGCTGTATTTTCTATAGTAAATAGAGTTAGGCAGGGATACTCACCACTATCGTTTCAGACCCAC---CTCCCAGTTCCGAGGGGA------CCCGACAGGCCCGAAGGAATCGACGAAGAAGGTGGAGAGCAAGGCAGAGACAGATCAATTCGATTAGTGGATGGATTCTTGGCACTTTTCTGGGACGACCTGAGGAGCCTGTGTCTTTTCAGCTACCACCACTTGAGAGACTTACTCTTGATTGCAGCGCGGATTGTGCAACTTCTGGGACAGAGG---------------GGGTGGGAGATCCTCAAATATTGGTGG---AATCTCCTGCTGTATTGG---------------------------------------------------AGTCAGGAACTAAAGAATAGTGCTGTCAGCTTGCTCAACACCACTGCTATAGTAGTAGCTGAGGGGACAGACAGGGTTATAGAAGCATTGCAAAGA------------------GCCTTTAGGGCTATTCTCCACATCCCTACAAGAATAAGACAAGGCTTGGAAAGGGCTTTACTATAA

2.04013226.ADARC.GU330384 ATGAAAGTGAAGGTGATCAGGAAGAATTGTCAGCACTTG---------TGGACATGGGGC------------------------ACGATGCTCCTTGGGATGTTAATGATC------------TGTAGTGCTGAA---------GATCAATTGTGGGTCACAGTTTATTATGGGGTACCTGTGTGGAAAGAAGCAACCACCACTCTATTTTGTGCATCAGATGCCAAAGGATATGATACAGAGGTACATAAT---GTTTGGGCCACACATGCCTGTGTACCCACAGACCCCAACCCACAAGAAGTGTTATTG---GCAAATGTGACAGAAAATTTTAACATGTGGAAAAATAACATGGTAGACCAGATGCATGAGGATGTAATCAGTTTATGGGATCAAAGTCTAAAGCCATGTGTAAAATTGACACCACTCTGTGTTACTTTATCTTGCACTAATGTGACTAATAATAATAATACTGCCAGT------------------------------------------------------------------------------------AATAGTTCTGATTGGGAAAAGATGGAG---GGAGAAATAAAAAACTGCTCTTTCAATGTCACC---CCAAGCATAAGAGAT------AGGGTGCGCAAAGAATATGCACTCTTTTATAGCCTTGATGTAGTACCTATAAAGGATACT------------------------------AATGATAGT---------------------AGAACCTATAGATTAATAAATTGTAACACCTCAGTCATTACACAGGCCTGTCCAAAGGTATCCTTTGAGCCAATTCCCATACATTATTGTGCCCCGGCTGGTTATGCGATTCTAAAATGT---AATAATAAGACTTTCAATGGAACAGGACCATGTACAAATGTCAGCACAGTACAATGTACACATGGAATTAGGCCTGTAGTATCAACTCAACTGTTGTTAAATGGCAGCCTAGCAGAAGAA---GACATAGTAATCAGATCTGAAAATCTCACAGACAATGCTAAAACCATAATAGTACATCTGAATGAATCTGTAGAAATTAATTGTACAAGACCCAACAATAATACAAGAAAAAGTATAAATATAGGA------------CCAGGGGCA---GCGATGTATGCAACAGGAGCCATAATAGGAGATATAAGACAAGCACATTGTAACATT------AGTGGAGCAAGATGGAATGACACTTTAAAAAAGGTAGTCAAAAAATTAAGA---GAAAAATTTGGA------------AATAAAACA---ATAATCTTTGATCAA---------CACTCAGGAGGGGACCCAGAAATTGTAATGCACAGTTTTAATTGTGGAGGGGAATTTTTCTACTGTAATACAACAAAACTATTCAATAGTACTTGG---------------AATAGTACTAGG------------------------------AATGATACTGAAAGGAAT---------AGTAATGAAACT------------------------GACACAATCACACTCCCATGCAGAATAAAACAAATTATAAACATGTGGCAGGGAGTAGGAAAAGCAATGTATGCCCCTCCTATCAGAGGACTAATTAGATGTTCATCAAATATTACAGGGCTGTTATTAACAAGGGATGGTGGTAAGACT------------AACAGGAGC---------------GGGAGCAACGGGTCT---GAGACCTTCAGACCTGGAGGAGGAAATATGAAGGACAATTGGAGA---AGTGAATTATATAAATATAAAGTAGTAAAACTTGAACCA---TTAGGAATAGCACCC---ACCAAGGCAAGGAGAAGAGTGGTGCAGAGAGAA---AAAAGAGCAGTA---GGA---CTAGGA---GCGTTG---TTCATT---GGG---------TTCTTGGGA---GCAGCAGGAAGCACTATGGGCGCAGCGTCAGTA---ACGCTGACGGTACAGGCCAGACAATTATTGTCTGGTATAGTGCAACAGCAGAACAATCTGCTGATGGCTATTGATGCACAACAGCATCTGTTGCAACTCACAGTCTGGGGCGTCAAGCAGCTCCGGGCAAGA---ATCCTGGCTGTGGAAAGATACCTAAAGGATCAACAGCTCCTAGGGATTTGGGGTTGCTCTGGAAAACTCATCTGCACCACTAATGTGCCTTGGAATGCTAGTTGGAGT---------------------------AATAAATCTCAAGAGCAAATTTGGGAA---AACATGACCTGGATGCAGTGGGAAAGAGAAATTGAC------AATTACACAGGCCTTATATACTCTTTAATTGAAAAATCGCAAAACCAACAAGAAAAGAATGAACAAGAATTATTGGCATTGGATAAGTGGGCAAGTTTGTGGAATTGGTTTGACATAACAAAATGGCTGTGGTATATAAAAATATTCATAATAATAGTAGGAGGCTTGATAGGATTAAGAATAGTTTTTGCTGTATTTTCTATAGTAAATAGAGTTAGGCAGGGATACTCACCACTATCGTTTCAGACCCAC---CTCCCAGTTCCGAGGGGA------CCCGACAGGCCCGAAGGAATCGACGAAGAAGGTGGAGAGCAAGGCAGAGACAGATCAATTCGATTAGTGGATGGATTCTTGGCACTTTTCTGGGACGACCTGAGGAGCCTGTGTCTTTTCAGCTACCACCACTTGAGAGACTTACTCTTGATTGCAGCGCGGATTGTGCAACTTCTGGGACAGAGG---------------GGGTGGGAGATCCTCAAATATTGGTGG---AATCTCCTGCTGTATTGG---------------------------------------------------AGTCAGGAACTAAAGAATAGTGCTGTCAGCTTGCTCAACACCACTGCTATAGTAGTAGCTGAGGGGACAGACAGGGTTATAGAAGCATTGCAAAGA------------------GCCTTTAGGGCTATTCTCCACATCCCTACAAGAATAAGACAAGGCTTGGAAAGGGCTTTACTATAA

2.04013226.ADARC.GU330385 ATGAAAGTGAAGGTGATCAGGAAGAATTGTCAGCACTTG---------TGGACATGGGGT------------------------ACGATGCTCCTTGGGATGTTAATGATC------------TGTAGTGCTGAA---------GATCAATTGTGGGTCACAGTTTATTATGGGGTACCTGTGTGGAAAGAAGCAACCACCACTCTATTTTGTGCATCAGATGCCAAAGGATATGATACAGAGGTACATAAT---GTTTGGGCCACACATGCCTGTGTACCCACAGACCCCAACCCACAAGAAGTGTTATTG---GCAAATGTGACAGAAAATTTTAACATGTGGAAAAATAACATGGTAGACCAGATGCATGAGGATGTAATCAGTTTATGGGATCAAAGTCTAAAGCCATGTGTAAAATTGACACCACTCTGTGTTACTTTATCTTGCACTAATGTGACTAATAATAATAATACTGCCAGT------------------------------------------------------------------------------------AATAGTTCTGATTGGGAAAAGATGGAG---GGAGAAATAAAAAACTGCTCTTTCAATGTCACC---CCAAGCATAAGAGAT------AGGGTGCGCAAAGAATATGCACTCTTTTATAGCCTTGATGTAGTACCTATAAAGGATACT------------------------------AATGATAGT---------------------AGAACCTATAGATTAATAAATTGTAACACCTCAGTCATTACACAGGCCTGTCCAAAGGTATCCTTTGAGCCAATTCCCATACATTATTGTGCCCCGGCTGGTTATGCGATTCTAAAATGT---AATAATAAGACTTTCAATGGAACAGGACCATGTACAAATGTCAGCACAGTACAATGTACACATGGAATTAGGCCTGTAGTATCAACTCAACTGTTGTTAAATGGCAGCCTAGCAGAAGAA---GACATAGTAATCAGATCTGAAAATCTCACAGACAATGCTAAAACCATAATAGTACATCTGAATGAATCTGTAGAAATTAATTGTACAAGACCCAACAATAATACAAGAAAAAGTATAAATATAGGA------------CCAGGGGCA---GCGATGTATGCAACAGGAGCCATAATAGGAGATATAAGACAAGCACATTGTAACATT------AGTGGTGCAAGATGGAATGACACTTTAAAAAAGGTAGTCAAAAAATTAAGA---AAAAAATTTGGA------------AATAAAACA---ATAATCTTTGATCAA---------CACTCAGGAGGGGACCCAGAAATTGTAATGCACAGTTTTAATTGTGGAGGGGAATTTTTCTACTGTAATACAACAAAACTATTCAATAGTACTTGG---------------AATAGTACTAGG------------------------------AATGATACTGAAAGGAAT---------AGTAATGAAACT------------------------GACACAATCACACTCCCATGCAGAATAAAACAAATTATAAACATGTGGCAGGGAGTAGGAAAAGCAATGTATGCCCCTCCTATCAGAGGACTAATTAGATGTTCATCAAATATTACAGGGCTGTTATTAACAAGGGATGGTGGTAATACT------------AACAGGAGC---------------GGGAGCAACGGGTCT---GAGACCTTCAGACCTGGAGGAGGAAATATGAAGGACAATTGGAGA---AGTGAATTATATAAATATAAAGTAGTAAAACTTGAACCA---TTAGAAATAGCACCC---ACCAAGGCAAGGAGAAGAGTGGTGCAGAGAGAA---AAAAGAGCAGTA---GGA---CTAGGA---GCGTTG---TTCATT---GGG---------TTCTTGGGA---GCAGCAGGAAGCACTATGGGCGCAGCGTCAGTA---ACGCTAACGGTACAGGCCAGACAATTATTGTCTGGTATAGTGCAACAGCAGAACAATCTGCTGATGGCTATTGATGCACAACAGCATCTGTTGCAACTCACAGTCTGGGGCGTCAAGCAGCTCCGGGCAAGA---ATCCTGGCTGTGGAAAAATACCTAAAGGATCAACAGCTCCTAGGGATTTGGGGTTGCTCTGGAAAACTCATCTGCACCACTAATGTGCCTTGGAATGCTAGTTGGAGT---------------------------AATAAATCTCAAGAGCAAATTTGGGAA---AACATGACCTGGATGCAGTGGGAAAGAGAAATTGAC------AATTACACAGGCCTTATATACTCTTTAATTGAAAAATCGCAAAACCAACAAGAAAAGAATGAACAAGAATTATTGGCATTGGATAAGTGGGCAAGTTTGTGGAATTGGTTTGACATAACAAAATGGCTGTGGTATATAAAAATATTCATAATAATAGTAGGAGGCTTGATAGGATTAAGAATAGTTTTTGCTGTATTTTCTATAGTAAATAGAGTTAGGCAGGGATACTCACCACTATCGTTTCAGACCCAC---CTCCCAGTTCCGAGGGGA------CCCGACAGGCCCGAAGGAATCGACGAAGAAGGTGGAGAGCAAGGCAGAGACAGATCAATTCGATTAGTGGATGGATTCTTGGCACTTTTCTGGGACGACCTGAGGAGCCTGTGTCTTTTCAGCTACCACCACTTGAGAGACTTACTCTTGATTGCAGCGCGGATTGTGCAACTTCTGGGACAGAGG---------------GGGTGGGAGATCCTCAAATATTGGTGG---AATCTCCTGCTGTATTGG---------------------------------------------------AGTCAGGAACTAAAGAATAGTGCTGTCAGCTTGCTCAACACCACTGCTATAGTAGTAGCTGAGGGGACAGACAGGGTTATAGAAGCATTGCAAAGA------------------GCCTTTAGGGCTATTCTCCACATCCCTACAAGAATAAGACAAGGCTTGGAAAGGGCTTTACTATAA

2.04013226.ADARC.GU330386 ATGAAAGTGAAGGTGATCAGGAAGAATTGTCAGCACTTG---------TGGACATGGGGC------------------------ACGATGCTCCTTGGGATGTTAATGATC------------TGTAGTGCTGAA---------GATCAATTGTGGGTCACAGTTTATTATGGGGTACCTGTGTGGAAAGAAGCAACCACCACTCTATTTTGTGCATCAGATGCCAAAGGATATGATACAGAGGTACATAAT---GTTTGGGCCACACATGCCTGTGTACCCACAGACCCCAACCCACAAGAAGTGTTATTG---GCAAATGTGACAGAAAATTTTAACATGTGGAAAAATAACATGGTAGACCAGATGCATGAGGATGTAATCAGTTTATGGGATCAAAGTCTAAAGCCATGTGTAAAATTGACACCACTCTGTGTTACTTTATCTTGCACTAATGTGACTAATAATAATAATACTGCCAGT------------------------------------------------------------------------------------AATAGTTCTGATTGGGAAAAGATGGAG---GGAGAAATAAAAAACTGCTCTTTCAATGTCACC---CCAAGCATAAGAGAT------AGGGTGCGCAAAGAATATGCACTCTTTTATAGCCTTGATGTAGTACCTATAAAGGATACT------------------------------AATGATAGT---------------------AGAACCTATAGATTAATAAATTGTAACACCTCAGTCATTACACAGGCCTGTCCAAAGGTATCCTTTGAGCCAATTCCCATACATTATTGTGCCCCGGCTGGTTATGCGATTCTAAAATGT---AATAATAAGACTTTCAATGGAACAGGACCATGTACAAATGTCAGCACAGTACAATGTACACATGGAATTAGGCCTGTAGTATCAACTCAACTGTTGTTAAATGGCAGCCTAGCAGAAGAA---GACATAGTAATCAGATCTGAAAATCTCACAGACAATGCTAAAACCATAATAGTACATCTGAATGAATCTGTAGAAATTAATTGTACAAGACCCAACAATAATACAAGAAAAAGTATAAATATAGGA------------CCAGGGGCA---GCGATGTATGCAACAGGAGCCATAATAGGAGATATAAGACAAGCACATTGTAACATT------AGTGGAGCAAGATGGAATGACACTTTAAAAAAGGTAGTCAAAAAATTAAGA---GAAAAATTTGAA------------AATAAAACA---ATAATCTTTGATCAA---------CACTCAGGAGGGGACCCAGAAATTGTAATGCACAGTTTTAATTGTGGAGGGGAATTTTTCTACTGTAATACAACAAAACTATTCAATAGTACTTGG---------------AATAGTACTAGG------------------------------AATGATACTGAAAGGAAT---------AGTAATGAAACT------------------------GACACAATCACACTCCCATGCAGAATAAAACAAATTATAAACATGTGGCAGGGAGTAGGAAAAGCAATGTATGCCCCTCCTATCAGAGGACTAATTAGATGTTCATCAAATATTACAGGGCTGTTATTAACAAGGGATGGTGGTAATACT------------AACAGGAGC---------------GGGAGCAACGGGTCT---GAGACCTTCAGACCTGGAGGAGGAAATATGAAGGACAATTGGAGA---AGTGAATTATATAAATATAAAGTAGTAAAACTTGAACCA---TTAGGAATAGCACCC---ACCAAGGCAAGGAGAAGAGTGGTGCAGAGAGAA---AAAAGAGCAGTA---GGA---CTAGGA---GCGTTG---TTCATT---GGG---------TTCTTGGGA---GCAGCAGGAAGCACTATGGGCGCAGCGTCAGTA---ACGCTGACGGTACAGGCCAGACAATTATTGTCTGGTATAGTGCAACAGCAGAACAATCTGCTGATGGCTATTGATGCACAACAGCATCTGTTGCAACTCACAGTCTGGGGCGTCAAGCAGCTCCGGGCAAGA---ATCCTGGCTGTGGAAAGATACCTAAAGGATCAACAGCTCCTAGGGATTTGGGGTTGCTCTGGAAAACTCATCTGCACCACTAATGTGCCTTGGAATGCTAGTTGGAGT---------------------------AATAAATCTCAAGAGCAAATTTGGGAA---AACATGACCTGGATGCAGTGGGAAAGAGAAATTGAC------AATTACACAGGCCTTATATACTCTTTAATTGAAAAATCGCAAAACCAACAAGAAAAGAATGAACAAGAATTATTGGCATTGGATAAGTGGGCAAGTTTGTGGAATTGGTTTGACATAACAAAATGGCTGTGGTATATAAAAATATTCATAATAATAGTAGGAGGCTTGATAGGATTAAGAATAGTTTTTGCTGTATTTTCTATAGTAAATAGAGTTAGGCAGGGATACTCACCACTATCGTTTCAGACCCAC---CTCCCAGTTCCGAGGGGA------CCCGACAGGCCCGAAGGAATCGACGAAGAAGGTGGAGAGCAAGGCAGAGACAGATCAATTCGATTAGTGGATGGATTCTTGGCACTTTTCTGGGACGACCTGAGGAGCCTGTGTCTTTTCAGCTACCACCACTTGAGAGACTTACTCTTGATTGCAGCGCGGATTGTGCAACTTCTGGGACAGAGG---------------GGGTGGGAGATCCTCAAATATTGGTGG---AATCTCCTGCTGTATTGG---------------------------------------------------AGTCAGGAACTAAAGAATAGTGCTGTCAGCTTGCTCAACACCACTGCTATAGTAGTAGCTGAGGGGACAGACAGGGTTATAGAAGCATTGCAAAGA------------------GCCTTTAGGGCTATTCTCCACATCCCTACAAGAATAAGACAAGGCTTGGAAAGGGCTTTACTATAA

2.04013226.ADARC.GU330387 ATGAAAGTGAAGGTGATCAGGAAGAATTGTCAGCACTTG---------TGGACATGGGGC------------------------ACGATGCTCCTTGGGATGTTAATGATC------------TGTAGTGCTGAA---------GATCAATTGTGGGTCACAGTTTATTATGGGGTACCTGTGTGGAAAGAAGCAACCACCACTCTATTTTGTGCATCAGATGCCAAAGGATATGATACAGAGGTACATAAT---GTTTGGGCCACACATGCCTGTGTACCCACAGACCCCAACCCACAAGAAGTGTTATTG---GCAAATGTGACAGAAAATTTTAACATGTGGAAAAATAACATGGTAGACCAGATGCATGAGGATGTAATCAGTTTATGGGATCAAAGTCTAAAGCCATGTGTAAAATTGACACCACTCTGTGTTACTTTATCTTGCACTAATGTGACTAATAATAATAATACTGCCAGT------------------------------------------------------------------------------------AATAGTTCTGATTGGGAAAAGATGGAG---GGAGAAATAAAAAACTGCTCTTTCAATGTCACC---CCAAGCATAAGAGAT------AGGGTGCGCAAAGAATATGCACTCTTTTATAGCCTTGATGTAGTACCTATAAAGGATACT------------------------------AATGATAGT---------------------AGAACCTATAGATTAATAAATTGTAACACCTCAGTCATTACACAGGCCTGTCCAAAGGTATCCTTTGAGCCAATTCCCATACATTATTGTGCCCCGGCTGGTTATGCGATTCTAAAATGT---AATAATAAGACTTTCAATGGAACAGGACCATGTACAAATGTCAGCACAGTACAATGTACACATGGAATTAGGCCTGTAGTATCAACTCAACTGTTGTTAAATGGCAGCCTAGCAGAAGAA---GACATAGTAATCAGATCTGAAAATCTCACAGACAATGCTAAAACCATAATAGTACATCTGAATGAATCTGTAGAAATTAATTGTACAAGACCCAACAATAATACAAGAAAAAGTATAAATATAGGA------------CCAGGGGCA---GCGATGTATGCAACAGGAGCCATAATAGGAGATATAAGACAAGCACATTGTAACATT------AGTGGAGCAAGATGGAATGACACTTTAAAAAAGGTAGTCAAAAAATTAAGA---GAAAAATTTGGA------------AATAAAACA---ATAATCTTTGATCAA---------CACTCAGGAGGGGACCCAGAAATTGTAATGCACAGTTTTAATTGTGGAGGGGAATTTTTCTACTGTAATACAACAAAACTATTCAATAGTACTTGG---------------AATAGTACTAGG------------------------------AATGATACTGAAAGGAAT---------AGTAATGAAACT------------------------GACACAATCACACTCCCATGCAGAATAAAACAAATTATAAACATGTGGCAGGGAGTAGGAAAAGCAATGTATGCCCCTCCTATCAGAGGACTAATTAGATGTTCATCAAATATTACAGGGCTGTTATTAACAAGGGATGGTGGTAATACT------------AACAGGAGC---------------GGGAGCAACGGGTCT---GAGACCTTCAGACCTGGAGGAGGAAATATGAAGGACAATTGGAGA---AGTGAATTATATAAATATAAAGTAGTAAAACTTGAACCA---TTAGGAATAGCACCC---ACCAAGGCAAGGAGAAGAGTGGTGCAGAGAGAA---AAAAGAGCAGTA---GGA---CTAGGA---GCGTTG---TTCATT---GGG---------TTCTTGGGA---GCAGCAGGAAGCACTATGGGCGCAGCGTCAGTA---ACGCTGACGGTACAGGCCAGACAATTATTGTCTGGTATAGTGCAACAGCAGAACAATCTGCTGATGGCTATTGATGCACAACAGCATCTGTTGCAACTCACAGTCTGGGGCGTCAAGCAGCTCCGGGCAAGA---ATCCTGGCTGTGGAAAGATACCTAAAGGATCAACAGCTCCTAGGGATTTGGGGTTGCTCTGGAAAACTCATCTGCACCACTAATGTGCCTTGGAATGCTAGTTGGAGT---------------------------AATAAATCTCAAGAGCAAATTTGGGAA---AACATGACCTGGATGCAGTGGGAAAGAGAAATTGAC------AATTACACAGGCCTTATATACTCTTTAATTGAAAAATCGCAAAACCAACAAGAAAAGAATGAACAAGAATTATTGGCATTGGATAAGTGGGCAAGTTTGTGGAATTGGTTTGACATAACAAAATGGCTGTGGTATATAAAAATATTCATAATAATAGTAGGAGGCTTGATAGGATTAAGAATAGTTTTTGCTGTATTTTCTATAGTAAATAGAGTTAGGCAGGGATACTCACCACTATCGTTTCAGACCCAC---CTCCCAGTTCCGAGGGGA------CCCGACAGGCCCGAAGGAATCGACGAAGAAGGTGGAGAGCAAGGCAGAGACAGATCAATTCGATTAGTGGATGGATTCTTGGCACTTTTCTGGGACGACCTGAGGAGCCTGTGTCTTTTCAGCTACCACCACTTGAGAGACTTACTCTTGATTGCAGCGCGGATTGTGCAACTTCTGGGACAGAGG---------------GGGTGGGAGATCCTCAAATATTGGTGG---AATCTCCTGCTGTATTGG---------------------------------------------------AGTCAGGAACTAAAGAATAGTGCTGTCAGCTTGCTCAACACCACTGCTATAGTAGTAGCTGAGGGGACAGACAGGGTTATAGAAGCATTGCAAAGA------------------GCCTTTAGGGCTATTCTCCACATCCCTACAAGAATAAGACAAGGCTTGGAAAGGGCTTTACTATAA

2.04013226.ADARC.GU330388 ATGAAAGTGAAGGTGATCAGGAAGAATTGTCAGCACTTG---------TGGACATGGGGC------------------------ACGATGCTCCTTGGGATGTTAATGATC------------TGTAGTGCTGAA---------GATCAATTGTGGGTCACAGTTTATTATGGGGTACCTGTGTGGAAAGAAGCAACCACCACTCTATTTTGTGCATCAGATGCCAAAGGATATGATACAGAGGTACATAAT---GTTTGGGCCACACATGCCTGTGTACCCACAGACCCCAACCCACAAGAAGTGTTATTG---GCAAATGTGACAGAAAATTTTAACATGTGGAAAAATAACATGGTAGACCAGATGCATGAGGATGTAATCAGTTTATGGGATCAAAGTCTAAAGCCATGTGTAAAATTGACACCACTCTGTGTTACTTTATCTTGCACTAATGTGACTAATAATAATAATACTGCCAGT------------------------------------------------------------------------------------AATAGTTCTGATTGGGAAAAGATGGAG---GGAGAAATAAAAAACTGCTCTTTCAATGTCACC---CCAAGCATAAGAGAT------AGGGTGCGCAAAGAATATGCACTCTTTTATAGCCTTGATGTAGTACCTATAAAGGATACT------------------------------AATGATAGT---------------------AGAACCTATAGATTAATAAATTGTAACACCTCAGTCATTACACAGGCCTGTCCAAAGGTATCCTTTGAGCCAATTCCCATACATTATTGTGCCCCGGCTGGTTATGCGATTCTAAAATGT---AATAATAAGACTTTCAATGGAACAGGACCATGTACAAATGTCAGCACAGTACAATGTACACATGGAATTAGGCCTGTAGTATCAACTCAACTGTTGTTAAATGGCAGCCTAGCAGAAGAA---GACATAGTAATCAGATCTGAAAATCTCACAGACAATGCTAAAACCATAATAGTACATCTGAATGAATCTGTAGAAATTAATTGTACAAGACCCAACAATAATACAAGAAAAAGTATAAATATAGGA------------CCAGGGGCA---GCGATGTATGCAACAGGAGCCATAATAGGAGATATAAGACAAGCACATTGTAACATT------AGTGGAGCAAGATGGAATGACACTTTAAAAAAGGTAGTCAAAAAATTAAGA---GAAAAATTTGGA------------AATAAAACA---ATAATCTTTGATCAA---------CACTCAGGAGGGGACCCAGAAATTGTAATGCACAGTTTTAATTGTGGAGGGGAATTTTTCTACTGTAATACAACAAAACTATTCAATAGTACTTGG---------------AATAGTACTAGG------------------------------AATGATACTGAAAGGAAT---------AGTAATGAAACT------------------------GACACAATCACACTCCCATGCAGAATAAAACAAATTATAAACATGTGGCAGGGAGTAGGAAAAGCAATGTATGCCCCTCCTATCAGAGGACTAATTAGATGTTCATCAAATATTACAGGGCTGTTATTAACAAGGGATGGTGGTAATACT------------AACAGGAGC---------------GGGAGCAACGGGTCT---GAGACCTTCAGACCTGGAGGAGGAAATATGAAGGACAATTGGAGA---AGTGAATTATATAAATATAAAGTAGTAAAACTTGAACCA---TTAGGAATAGCACCC---ACCAAGGCAAGGAGAAGAGTGGTGCAGAGAGAA---AAAAGAGCAGTA---GGA---CTAGGA---GCGTTG---TTCATT---GGG---------TTCTTGGGA---GCAGCAGGAAGCACTATGGGCGCAGCGTCAGTA---ACGCTGACGGTACAGGCCAGACAATTATTGTCTGGTATAGTGCAACAGCAGAACAATCTGCTGATGGCTATTGATGCACAACAGCATCTGTTGCAACTCACAGTCTGGGGCGTCAAGCAGCTCCGGGCAAGA---ATCCTGGCTGTGGAAAGATACCTAAAGGATCAACAGCTCCTAGGGATTTGGGGTTGCTCTGGAAAACTCATCTGCACCACTAATGTGCCTTGGAATGCTAGTTGGAGT---------------------------AATAAATCTCAAGAGCAAATTTGGGAA---AACATGACCTGGATGCAGTGGGAAAGAGAAATTGAC------AATTACACAGGCCTTATATACTCTTTAATTGAAAAATCGCAAAACCAACAAGAAAAGAATGAACAAGAATTATTGGCATTGGATAAGTGGGCAAGTTTGTGGAATTGGTTTGACATAACAAAATGGCTGTGGTATATAAAAATATTCATAATAATAGTAGGAGGCTTGATAGGATTAAGAATAGTTTTTGCTGTATTTTCTATAGTAAATAGAGTTAGGCAGGGATACTCACCACTATCGTTTCAGACCCAC---CTCCCAGTTCCGAGGGGA------CCCGACAGGCCCGAAGGAATCGACGAAGAAGGTGGAGAGCAAGGCAGAGACAGATCAATTCGATTAGTGGATGGATTCTTGGCACTTTTCTGGGACGACCTGAGGAGCCTGTGTCTTTTCAGCTACCACCACTTGAGAGACTTACTCTTGATTGCAGCGCGGATTGTGCAACTTCTGGGACAGAGG---------------GGGTGGGAGATCCTCAAATATTGGTGG---AATCTCCTGCTGTATTGG---------------------------------------------------AGTCAGGAACTAAAGAATAGTGCTGTCAGCTTGCTCAACACCACTGCTATAGTAGTAGCTGAGGGGACAGACAGGGTTATAGAAGCATTGCAAAGA------------------GCCTTTAGGGCTATTCTCCACATCCCTACAAGAATAAGACAAGGCTTGGAAAGGGCTTTACTATAA

2.04013226.ADARC.GU330389 ATGAAAGTGAAGGTGATCAGGAAGAATTGTCAGCACTTG---------TGGACATGGGGC------------------------ACGATGCTCCTTGGGATGTTAATGATC------------TGTAGTGCTGAA---------GATCAATTGTGGGTCACAGTTTATTATGGGGTACCTGTGTGGAAAGAAGCAACCACCACTCTATTTTGTGCATCAGATGCCAAAGGATATGATACAGAGGTACATAAT---GTTTGGGCCACACATGCCTGTGTACCCACAGACCCCAACCCACAAGAAGTGTTATTG---GCAAATGTGACAGAAAATTTTAACATGTGGAAAAATAACATGGTAGACCAGATGCATGAGGATGTAATCAGTTTATGGGATCAAAGTCTAAAGCCATGTGTAAAATTGACACCACTCTGTGTTACTTTATCTTGCACTAATGTGACTAATAATAATAATACTGCCAGT------------------------------------------------------------------------------------AATAGTTCTGATTGGGAAAAGATGGAG---GGAGAAATAAAAAACTGCTCTTTCAATGTCACC---CCAAGCATAAGAGAT------AGGGTGCGCAAAGAATATGCACTCTTTTATAGCCTTGATGTAGTACCTATAAAGGATACT------------------------------AATGATAGT---------------------AGAACCTATAGATTAATAAATTGTAACACCTCAGTCATTACACAGGCCTGTCCAAAGGTATCCTTTGAGCCAATTCCCATACATTATTGTGCCCCGGCTGGTTATGCGATTCTAAAATGTAATAATAATAAGACTTTCAATGGAACAGGACCATGTACAAATGTCAGCACAGTACAATGTACACATGGAATTAGGCCTGTAGTATCAACTCAACTGTTGTTAAATGGCAGCCTAGCAGAAGAA---GACATAGTAATCAGATCTGAAAATCTCACAGACAATGCTAAAACCATAATAGTACATCTGAATGAATCTGTAGAAATTAATTGTACAAGACCCAACAATAATACAAGAAAAAGTATAAATATAGGA------------CCAGGGGCA---GCGATGTATGCAACAGGAGCCATAATAGGAGATATAAGACAAGCACATTGTAACATT------AGTGGAGCAAGATGGAATGACACTTTAAAAAAGGTAGTCAAAAAATTAAGA---GAAAAATTTGGA------------AATAAAACA---ATAATCTTTGATCAA---------CACTCAGGAGGGGACCCAGAAATTGTAATGCACAGTTTTAATTGTGGAGGGGAATTTTTCTACTGTAATACAACAAAACTATTCAATAGTACTTGG---------------AATAGTACTAGG------------------------------AATGATACTGAAAGGAAT---------AGTAATGAAACT------------------------GACACAATCACACTCCCATGCAGAATAAAACAAATTATAAACATGTGGCAGGGAGTAGGAAAAGCAATGTATGCCCCTCCTATCAGAGGACTAATTAGATGTTCATCAAATATTACAGGGCTGTTATTAACAAGGGATGGTGGTAATACT------------AACAGGAGC---------------GGGAGCAACGGGTCT---GAGACCTTCAGACCTGGAGGAGGAAATATGAAGGACAATTGGAGA---AGTGAATTATATAAATATAAAGTAGTAAAACTTGAACCA---TTAGGAATAGCACCC---ACCAAGGCAAGGAGAAGAGTGGTGCAGAGAGAA---AAAAGAGCAGTA---GGA---CTAGGA---GCGTTG---TTCATT---GGG---------TTCTTGGGA---GCAGCAGGAAGCACTATGGGCGCAGCGTCAGTA---ACGCTGACGGTACAGGCCAGACAATTATTGTCTGGTATAGTGCAACAGCAGAACAATCTGCTGATGGCTATTGATGCACAACAGCATCTGTTGCAACTCACAGTCTGGGGCGTCAAGCAGCTCCGGGCAAGA---ATCCTGGCTGTGGAAAGATACCTAAAGGATCAACAGCTCCTAGGGATTTGGGGTTGCTCTGGAAAACTCATCTGCACCACTAATGTGCCTTGGAATGCTAGTTGGAGT---------------------------AATAAATCTCAAGAGCAAATTTGGGAA---AACATGACCTGGATGCAGTGGGAAAGAGAAATTGAC------AATTACACAGGCCTTATATACTCTTTAATTGAAAAATCGCAAAACCAACAAGAAAAGAATGAACAAGAATTATTGGCATTGGATAAGTGGGCAAGTTTGTGGAATTGGTTTGACATAACAAAATGGCTGTGGTATATAAAAATATTCATAATAATAGTAGGAGGCTTGATAGGATTAAGAATAGTTTTTGCTGTATTTTCTATAGTAAATAGAGTTAGGCAGGGATACTCACCACTATCGTTTCAGACCCAC---CTCCCAGTTCCGAGGGGA------CCCGACAGGCCCGAAGGAATCGACGAAGAAGGTGGAGAGCAAGGCAGAGACAGATCAATTCGATTAGTGGATGGATTCTTGGCACTTTTCTGGGACGACCTGAGGAGCCTGTGTCTTTTCAGCTACCACCACTTGAGAGACTTACTCTTGATTGCAGCGCGGATTGTGCAACTTCTGGGACAGAGG---------------GGGTGGGAGATCCTCAAATATTGGTGG---AATCTCCTGCTGTATTGG---------------------------------------------------AGTCAGGAACTAAAGAATAGTGCTGTCAGCTTGCTCAACACCACTGCTATAGTAGTAGCTGAGGGGACAGACAGGGTTATAGAAGCATTGCAAAGA------------------GCCTTTAGGGCTATTCTCCACATCCCTACAAGAATAAGACAAGGCTTGGAAAGGGCTTTACTATAA

2.04013226.ADARC.GU330390 ATGAAAGTGAAGGTGATCAGGAAGAATTGTCAGCACTTG---------TGGACATGGGGC------------------------ACGATGCTCCTTGGGATGTTAATGATC------------TGTAGTGCTGAA---------GATCAATTGTGGGTCACAGTTTATTATGGGGTACCTGTGTGGAAAGAAGCAACCACCACTCTATTTTGTGCATCAGATGCCAAAGGATATGATACAGAGGTACATAAT---GTTTGGGCCACACATGCCTGTGTACCCACAGACCCCAACCCACAAGAAGTGTTATTG---GCAAATGTGACAGAAAATTTTAACATGTGGAAAAATAACATGGTAGACCAGATGCATGAGGATGTAATCAGTTTATGGGATCAAAGTCTAAAGCCATGTGTAAAATTGACACCACTCTGTGTTACTTTATCTTGCACTAATGTGACTAATAATAATAATACTGCCAGT------------------------------------------------------------------------------------AATAGTTCTGATTGGGAAAAGATGGAG---GGAGAAATAAAAAACTGCTCTTTCAATGTCACC---CCAAGCATAAGAGAT------AGGGTGCGCAAAGAATATGCACTCTTTTATAGCCTTGATGTAGTACCTATAAAGGATACT------------------------------AATGATAGT---------------------AGAACCTATAGATTAATAAATTGTAACACCTCAGTCATTACACAGGCCTGTCCAAAGGTATCCTTTGAGCCAATTCCCATACATTATTGTGCCCCGGCTGGTTATGCGATTCTAAAATGT---AATAATAAGACTTTCAATGGAACAGGACCATGTACAAATGTCAGCACAGTACAATGTACACATGGAATTAGGCCTGTAGTATCAACTCAACTGTTGTTAAATGGCAGCCTAGCAGAAGAA---GACATAGTAATCAGATCTGAAAATCTCACAGACAATGCTAAAACCATAATAGTACATCTGAATGAATCTGTAGAAATTAATTGTACAAGACCCAACAATAATACAAGAAAAAGTATAAATATAGGA------------CCAGGGGCA---GCGATGTATGCAACAGGAGCCATAATAGGAGATATAAGACAAGCACATTGTAACATT------AGTGGAGCAAGATGGAATGACACTTTAAAAAAGGTAGTCAAAAAATTAAGA---GAAAAATTTGGA------------AATAAAACA---ATAATCTTTGATCAA---------CACTCAGGAGGGGACCCAGAAATTGTAATGCACAGTTTTAATTGTGGAGGGGAATTTTTCTACTGTAATACAACAAAACTATTCAATAGTACTTGG---------------AATAGTACTAGG------------------------------AATGATACTGAAAGGAAT---------AGTAATGAAACT------------------------GACACAATCACACTCCCATGCAGAATAAAACAAATTATAAACATGTGGCAGGGAGTAGGAAAAGCAATGTATGCCCCTCCTATCAGAGGACTAATTAGATGTTCATCAAATATTACAGGGCTGTTATTAACAAGGGATGGTGGTAATACT------------AACAGGAGC---------------GGGAGCAACGGGTCT---GAGACCTTCAGACCTGGAGGAGGAAATATGAAGGACAATTGGAGA---AGTGAATTATATAAATATAAAGTAGTAAAACTTGAACCA---TTAGGAATAGCACCC---ACCAAGGCAAGGAGAAGAGTGGTGCAGAGAGAA---AAAAGAGCAGTA---GGA---CTAGGA---GCGTTG---TTCATT---GGG---------TTCTTGGGA---GCAGCAGGAAGCACTATGGGCGCAGCGTCAGTA---ACGCTGACGGTACAGGCCAGACAATTATTGTCTGGTATAGTGCAACAGCAGAACAATCTGCTGATGGCTATTGATGCACAACAGCATCTGTTGCAACTCACAGTCTGGGGCGTCAAGCAGCTCCGGGCAAGA---ATCCTGGCTGTGGAAAGATACCTAAAGGATCAACAGCTCCTAGGGATTTGGGGTTGCTCTGGAAAACTCATCTGCACCACTAATGTGCCTTGGAATGCTAGTTGGAGT---------------------------AATAAATCTCAAGAGCAAATTTGGGAA---AACATGACCTGGATGCAGTGGGAAAGAGAAATTGAC------AATTACACAGGCCTTATATACTCTTTAATTGAAAAATCGCAAAACCAACAAGAAAAGAATGAACAAGAATTATTGGCATTGGATAAGTGGGCAAGTTTGTGGAATTGGTTTGACATAACAAAATGGCTGTGGTATATAAAAATATTCATAATAATAGTAGGAGGCTTGATAGGATTAAGAATAGTTTTTGCTGTATTTTCTATAGTAAATAGAGTTAGGCAGGGATACTCACCACTATCGTTTCAGACCCAC---CTCCCAGTTCCGAGGGGA------CCCGACAGGCCCGAAGGAATCGACGAAGAAGGTGGAGAGCAAGGCAGAGACAGATCAATTCGATTAGTGGATGGATTCTTGGCACTTTTCTGGGACGACCTGAGGAGCCTGTGTCTTTTCAGCTACCACCACTTGAGAGACTTACTCTTGATTGCAGCGCGGATTGTGCAACTTCTGGGACAGAGG---------------GGGTGGGAGATCCTCAAATATTGGTGG---AATCTCCTGCTGTATTGG---------------------------------------------------AGTCAGGAACTAAAGAATAGTGCTGTCAGCTTGCTCAACACCACTGCTATAGTAGTAGCTGAGGGGACAGACAGGGTTATAGAAGCATTGCAAAGA------------------GCCTTTAGGGCTATTCTCCACATCCCTACAAGAATAAGACAAGGCTTGGAAAGGGCTTTACTATAA

2.04013226.ADARC.GU330391 ATGAAAGTGAAGGTGATCAGGAAGAATTGTCAGCACTTG---------TGGACATGGGGC------------------------ACGATGCTCCTTGGGATGTTAATGATC------------TGTAGTGCTGAA---------GATCAATTGTGGGTCACAGTTTATTATGGGGTACCTGTGTGGAAAGAAGCAACCACCACTCTATTTTGTGCATCAGATGCCAAAGGATATGATACAGAGGTACATAAT---GTTTGGGCCACACATGCCTGTGTACCCACAGACCCCAACCCACAAGAAGTGTTATTG---GCAAATGTGACAGAAAATTTTAACATGTGGAAAAATAACATGGTAGACCAGATGCATGAGGATGTAATCAGTTTATGGGATCAAAGTCTAAAGCCATGTGTAAAATTGACACCACTCTGTGTTACTTTATCTTGCACTAATGTGACTAATAATAATAATACTGCCAGT------------------------------------------------------------------------------------AATAGTTCTGATTGGGAAAAGATGGAG---GGAGAAATAAAAAACTGCTCTTTCAATGTCACC---CCAAGCATAAGAGAT------AGGGTGCGCAAAGAATATGCACTCTTTTATAGCCTTGATGTAGTACCTATAAAGGATACT------------------------------AATGATAGT---------------------AGAACCTATAGATTAATAAATTGTAACACCTCAGTCATTACACAGGCCTGTCCAAAGGTATCCTTTGAGCCAATTCCCATACATTATTGTGCCCCGGCTGGTTATGCGATTCTAAAATGT---AATAATAAGACTTTCAATGGAACAGGACCATGTACAAATGTCAGCACAGTACAATGTACACATGGAATTAGGCCTGTAGTATCAACTCAACTGTTGTTAAATGGCAGCCTAGCAGAAGAA---GACATAGTAATCAGATCTGAAAATCTCACAGACAATGCTAAAACCATAATAGTACATCTGAATGAATCTGTAGAAATTAATTGTACAAGACCCAACAATAATACAAGAAAAAGTATAAATATAGGA------------CCAGGGGCA---GCGATGTATGCAACAGGAGCCATAATAGGAGATATAAGACAAGCACATTGTAACATT------AGTGGAGCAAGATGGAATGACACTTTAAAAAAGGTAGTCAAAAAATTAAGA---GAAAAATTTGGA------------AATAAAACA---ATAATCTTTGATCAA---------CACTCAGGAGGGGACCCAGAAATTGTAATGCACAGTTTTAATTGTGGAGGGGAATTTTTCTACTGTAATACAACAAAACTATTCAATAGTACTTGG---------------AATAGTACTAGG------------------------------AATGATACTGAAAGGAAT---------AGTAATGAAACT------------------------GACACAATCACACTCCCATGCAGAATAAAACAAATTATAAACATGTGGCAGGGAGTAGGAAAAGCAATGTATGCCCCTCCTATCAGAGGACTAATTAGATGTTCATCAAATATTACAGGGCTGTTATTAACAAGGGATGGTGGTAATACT------------AACAGGAGC---------------GGGAGCAACGGGTCT---GAGACCTTCAGACCTGGAGGAGGAAATATGAAGGACAATTGGAGA---AGTGAATTATATAAATATAAAGTAGTAAAACTTGAACCA---TTAGGAATAGCACCC---ACCAAGGCAAGGAGAAGAGTGGTGCAGAGAGAA---AAAAGAGCAGTA---GGA---CTAGGA---GCGTTG---TTCATT---GGG---------TTCTTGGGA---GCAGCAGGAAGCACTATGGGCGCAGCGTCAGTA---ACGCTGACGGTACAGGCCAGACAATTATTGTCTGGTATAGTGCAACAGCAGAACAATCTGCTGATGGCTATTGATGCACAACAGCATCTGTTGCAACTCACAGTCTGGGGCGTCAAGCAGCTCCGGGCAAGA---ATCCTGGCTGTGGAAAGATACCTAAAGGATCAACAGCTCCTAGGGATTTGGGGTTGCTCTGGAAAACTCATCTGCACCACTAATGTGCCTTGGAATGCTAGTTGGAGT---------------------------AATAAATCTCAAGAGCAAATTTGGGAA---AACATGACCTGGATGCAGTGGGAAAGAGAAATTGAC------AATTACACAGGCCTTATATACTCTTTAATTGAAAAATCGCAAAACCAACAAGAAAAGAATGAACAAGAATTATTGGCATTGGATAAGTGGGCAAGTTTGTGGAATTGGTTTGACATAACAAAATGGCTGTGGTATATAAAAATATTCATAATAATAGTAGGAGGCTTGATAGGATTAAGAATAGTTTTTGCTGTATTTTCTATAGTAAATAGAGTTAGGCAGGGATACTCACCACTATCGTTTCAGACCCAC---CTCCCAGTTCCGAGGGGA------CCCGACAGGCCCGAAGGAATCGACGAAGAAGGTGGAGAGCAAGGCAGAGACAGATCAATTCGATTAGTGGATGGATTCTTGGCACTTTTCTGGGACGACCTGAGGAGCCTGTGTCTTTTCAGCTACCACCACTTGAGAGACTTACTCTTGATTGCAGCGCGGATTGTGCAACTTCTGGGACAGAGG---------------GGGTGGGAGATCCTCAAATATTGGTGG---AATCTCCTGCTGTATTGG---------------------------------------------------AGTCAGGAACTAAAGAATAGTGCTGTCAGCTTGCTCAACACCACTGCTATAGTAGTAGCTGAGGGGACAGACAGGGTTATAGAAGCATTGCAAAGA------------------GCCTTTAGGGCTATTCTCCACATCCCTACAAGAATAAGACAAGGCTTGGAAAGGGCTTTACTATAA

2.04013226.ADARC.GU330392 ATGAAAGTGAAGGTGATCAGGAAGAATTGTCAGCACTTG---------TGGACATGGGGC------------------------ACGATGCTCCTTGGGATGTTAATGATC------------TGTAGTGCTGAA---------GATCAATTGTGGGTCACAGTTTATTATGGGGTACCTGTGTGGAAAGAAGCAACCACCACTCTATTTTGTGCATCAGATGCCAAAGGATATGATACAGAGGTACATAAT---GTTTGGGCCACACATGCCTGTGTACCCACAGACCCCAACCCACAAGAAGTGTTATTG---GCAAATGTGACAGAAAATTTTAACATGTGGAAAAATAACATGGTAGACCAGATGCATGAGGATGTAATCAGTTTATGGGATCAAAGTCTAAAGCCATGTGTAAAATTGACACCACTCTGTGTTACTTTATCTTGCACTAATGTGACTAATAATAATAATACTGCCAGT------------------------------------------------------------------------------------AATAGTTCTGATTGGGAAAAGATGGAG---GGAGAAATAAAAAACTGCTCTTTCAATGTCACC---CCAAGCATAAGAGAT------AGGGTGCGCAAAGAATATGCACTCTTTTATAGCCTTGATGTAGTACCTATAAAGGATACT------------------------------AATGATAGT---------------------AGAACCTATAGATTAATAAATTGTAACACCTCAGTCATTACACAGGCCTGTCCAAAGGTATCCTTTGAGCCAATTCCCATACATTATTGTGCCCCGGCTGGTTATGCGATTCTAAAATGT---AATAATAAGACTTTCAATGGAACAGGACCATGTACAAATGTCAGCACAGTACAATGTACACATGGAATTAGGCCTGTAGTATCAACTCAACTGTTGTTAAATGGCAGCCTAGCAGAAGAA---GACATAGTAATCAGATCTGAAAATCTCACAGACAATGCTAAAACCATAATAGTACATCTGAATGAATCTGTAGAAATTAATTGTACAAGACCCAACAATAATACAAGAAAAAGTATAAATATAGGA------------CCAGGGGCA---GCGATGTATGCAACAGGAGCCATAATAGGAGATATAAGACAAGCACATTGTAACATT------AGTGGAGCAAGATGGAATGACACTTTAAAAAAGGTAGTCAAAAAATTAAGA---GAAAAATTTGGA------------AATAAAACA---ATAATCTTTGATCAA---------CACTCAGGAGGGGACCCAGAAATTGTAATGCACAGTTTTAATTGTGGAGGGGAATTTTTCTACTGTAATACAACAAAACTATTCAATAGTACTTGG---------------AATAGTACTAGG------------------------------AATGATACTGAAAGGAAT---------AGTAATGAAACT------------------------GACACAATCACACTCCCATGCAGAATAAAACAAATTATAAACATGTGGCAGGGAGTAGGAAAAGCAATGTATGCCCCTCCTATCAGAGGACTAATTAGATGTTCATCAAATATTACAGGGCTGTTATTAACAAGGGATGGTGGTAATACT------------AACAGGAGC---------------GGGAGCAACGGGTCT---GAGACCTTCAGACCTGGAGGAGGAAATATGAAGGACAATTGGAGA---AGTGAATTATATAAATATAAAGTAGTAAAACTTGAACCA---TTAGGAATAGCACCC---ACCAAGGCAAGGAGAAGAGTGGTGCAGAGAGAA---AAAAGAGCAGTA---GGA---CTAGGA---GCGTTG---TTCATT---GGG---------TTCTTGGGA---ACAGCAGGAAGCACTATGGGCGCAGCGTCAGTA---ACGCTGACGGTACAGGCCAGACAATTATTGTCTGGTATAGTGCAACAGCAGAACAATCTGCTGATGGCTATTGATGCACAACAGCATCTGTTGCAACTCACAGTCTGGGGCGTCAAGCAGCTCCGGGCAAGA---ATCCTGGCTGTGGAAAGATACCTAAAGGATCAACAGCTCCTAGGGATTTGGGGTTGCTCTGGAAAACTCATCTGCACCACTAATGTGCCTTGGAATGCTAGTTGGAGT---------------------------AATAAATCTCAAGAGCAAATTTGGGAA---AACATGACCTGGATGCAGTGGGAAAGAGAAATTGAC------AATTACACAGGCCTTATATACTCTTTAATTGAAAAATCGCAAAACCAACAAGAAAAGAATGAACAAGAATTATTGGCATTGGATAAGTGGGCAAGTTTGTGGAATTGGTTTGACATAACAAAATGGCTGTGGTATATAAAAATATTCATAATAATAGTAGGAGGCTTGATAGGATTAAGAATAGTTTTTGCTGTATTTTCTATAGTAAATAGAGTTAGGCAGGGATACTCACCACTATCGTTTCAGACCCAC---CTCCCAGTTCCGAGGGGA------CCCGACAGGCCCGAAGGAATCGACGAAGAAGGTGGAGAGCAAGGCAGAGACAGATCAATTCGATTAGTGGATGGATTCTTGGCACTTTTCTGGGACGACCTGAGGAGCCTGTGTCTTTTCAGCTACCACCACTTGAGAGACTTACTCTTGATTGCAGCGCGGATTGTGCAACTTCTGGGACAGAGG---------------GGGTGGGAGATCCTCAAATATTGGTGG---AATCTCCTGCTGTATTGG---------------------------------------------------AGTCAGGAACTAAAGAATAGTGCTGTCAGCTTGCTCAACACCACTGCTATAGTAGTAGCTGAGGGGACAGACAGGGTTATAGAAGCATTGCAAAGA------------------GCCTTTAGGGCTATTCTCCACATCCCTACAAGAATAAGACAAGGCTTGGAAAGGGCTTTACTATAA

2.04013226.ADARC.GU330393 ATGAAAGTGAAGGTGATCAGGAAGAATTGTCAGCACTTG---------TGGACATGGGGC------------------------ACGATGCTCCTTGGGATGTTAATGATC------------TGTAGTGCTGAA---------GATCAATTGTGGGTCACAGTTTATTATGGGGTACCTGTGTGGAAAGAAGCAACCACCACTCTATTTTGTGCATCAGATGCCAAAGGATATGATACAGAGGTACATAAT---GTTTGGGCCACACATGCCTGTGTACCCACAGACCCCAACCCACAAGAAGTGTTATTG---GCAAATGTGACAGAAAATTTTAACATGTGGAAAAATAACATGGTAGACCAGATGCATGAGGATGTAATCAGTTTATGGGATCAAAGTCTAAAGCCATGTGTAAAATTGACACCACTCTGTGTTACTTTATCTTGCACTAATGTGACTAATAATAATAATACTGCCAGT------------------------------------------------------------------------------------AATAGTTCTGATTGGGAAAAGATGGAG---GGAGAAATAAAAAACTGCTCTTTCAATGTCACC---CCAAGCATAAGAGAT------AGGGTGCGCAAAGAATATGCACTCTTTTATAGCCTTGATGTAGTACCTATAAAGGATACT------------------------------AATGATAGT---------------------AGAACCTATAGATTAATAAATTGTAACACCTCAGTCATTACACAGGCCTGTCCAAAGGTATCCTTTGAGCCAATTCCCATACATTATTGTGCCCCGGCTGGTTATGCGATTCTAAAATGT---AATAATAAGACTTTCAATGGAACAGGACCATGTACAAATGTCAGCACAGTACAATGTACACATGGAATTAGGCCTGTAGTATCAACTCAACTGTTGTTAAATGGCAGCCTAGCAGAAGAA---GACATAGTAATCAGATCTGAAAATCTCACAGACAATGCTAAAACCATAATAGTACATCTGAATGAATCTGTAGAAATTAATTGTACAAGACCCAACAATAATACAAGAAAAAGTATAAATATAGGA------------CCAGGGGCA---GCGATGTATGCAACAGGAGCCATAATAGGAGATATAAGACAAGCACATTGTAACATT------AGTGGAGCAAGATGGAATGACACTTTAAAAAAGGTAGTCAAAAAATTAAGA---GAAAAATTTGGA------------AATAAAACA---ATAATCTTTGATCAA---------CACTCAGGAGGGGACCCAGAAATTGTAATGCACAGTTTTAATTGTGGAGGGGAATTTTTCTACTGTAATACAACAAAACTATTCAATAGTACTTGG---------------AATAGTACTAGG------------------------------AATGATACTGAAAGGAAT---------AGTAATGAAACT------------------------GACACAATCACACTCCCATGCAGAATAAAACAAATTATAAACATGTGGCAGGGAGTAGGAAAAGCAATGTATGCCCCTCCTATCAGAGGACTAATTAGATGTTCATCAAATATTACAGGGCTGTTATTAACAAGGGATGGTGGTAATACT------------AACAGGAGC---------------GGGAGCAACGGGTCT---GAGACCTTCAGACCTGGAGGAGGAAATATGAAGGACAATTGGAGA---AGTGAATTATATAAATATAAAGTAGTAAAACTTGAACCA---TTAGGAATAGCACCC---ACCAAGGCAAGGAGAAGAGTGGTGCAGAGAGAA---AAAAGAGCAGTA---GGA---CTAGGA---GCGTTG---TTCATT---GGG---------TTCTTGGGA---GCAGCAGGAAGCACTATGGGCGCAGCGTCAGTA---ACGCTGACGGTACAGGCCAGACAATTATTGTCTGGTATAGTGCAACAGCAGAACAATCTGCTGATGGCTATTGATGCACAACAGCATCTGTTGCAACTCACAGTCTGGGGCGTCAAGCAGCTTCGGGCAAGA---ATCCTGGCTGTGGAAAGATACCTAAAGGATCAACAGCTCCTAGGGATTTGGGGTTGCTCTGGAAAACTCATCTGCACCACTAATGTGCCTTGGAATGCTAGTTGGAGT---------------------------AATAAATCTCAAGAGCAAATTTGGGAA---AACATGACCTGGATGCAGTGGGAAAGAGAAATTGAC------AATTACACAGGCCTTATATACTCTTTAATTGAAAAATCGCAAAACCAACAAGAAAAGAATGAACAAGAATTATTGGCATTGGATAAGTGGGCAAGTTTGTGGAATTGGTTTGACATAACAAAATGGCTGTGGTATATAAAAATATTCATAATAATAGTAGGAGGCTTGATAGGATTAAGAATAGTTTTTGCTGTATTTTCTATAGTAAATAGAGTTAGGCAGGGATACTCACCACTATCGTTTCAGACCCAC---CTCCCAGTTCCGAGGGGA------CCCGACAGGCCCGAAGGAATCGACGAAGAAGGTGGAGAGCAAGGCAGAGACAGATCAATTCGATTAGTGGATGGATTCTTGGCACTTTTCTGGGACGACCTGAGGAGCCTGTGTCTTTTCAGCTACCACCACTTGAGAGACTTACTCTTGATTGCAGCGCGGATTGTGCAACTTCTGGGACAGAGG---------------GGGTGGGAGATCCTCAAATATTGGTGG---AATCTCCTGCTGTATTGG---------------------------------------------------AGTCAGGAACTAAAGAATAGTGCTGTCAGCTTGCTCAACACCACTGCTATAGTAGTAGCTGAGGGGACAGACAGGGTTATAGAAGCATTGCAAAGA------------------GCCTTTAGGGCTATTCTCCACATCCCTACAAGAATAAGACAAGGCTTGGAAAGGGCTTTACTATAA

2.04013226.ADARC.GU330394 ATGAAAGTGAAGGTGATCAGGAAGAATTGTCAGCACTTG---------TGGACATGGGGC------------------------ACGATGCTCCTTGGGATGTTAATGATC------------TGTAGTGCTGAA---------GATCAATTGTGGGTCACAGTTTATTATGGGGTACCTGTGTGGAAAGAAGCAACCACCACTCTATTTTGTGCATCAGATGCCAAAGGATATGATACAGAGGTACATAAT---GTTTGGGCCACACATGCCTGTGTACCCACAGACCCCAACCCACAAGAAGTGTTATTG---GCAAATGTGACAGAAAATTTTAACATGTGGAAAAATAACATGGTAGACCAGATGCATGAGGATGTAATCAGTTTATGGGATCAAAGTCTAAAGCCATGTGTAAAATTGACACCACTCTGTGTTACTTTATCTTGCACTAATGTGACTAATAATAATAATACTGCCAGT------------------------------------------------------------------------------------AATAGTTCTGATTGGGAAAAGATGGAG---GGAGAAATAAAAAACTGCTCTTTCAATGTCACC---CCAAGCATAAGAGAT------AGGGTGCGCAAAGAATATGCACTCTTTTATAGCCTTGATGTAGTACCTATAAAGGATACT------------------------------AATGATAGT---------------------AGAACCTATAGATTAATAAATTGTAACACCTCAGTCATTACACAGGCCTGTCCAAAGGTATCCTTTGAGCCAATTCCCATACATTATTGTGCCCCGGCTGGTTATGCGATTCTAAAATGT---AATAATAAGACTTTCAATGGAACAGGACCATGTACAAATGTCAGCACAGTACAATGTACACATGGAATTAGGCCTGTAGTATCAACTCAACTGTTGTTAAATGGCAGCCTAGCAGAAGAA---GACATAGTAATCAGATCTGAAAATCTCACAGACAATGCTAAAACCATAATAGTACATCTGAATGAATCTGTAGAAATTAATTGTACAAGACCCAACAATAATACAAGAAAAAGTATAAATATAGGA------------CCAGGGGCA---GCGATGTATGCAACAGGAGCCATAATAGGAGATATAAGACAAGCACATTGTAACATT------AGTGGAGCAAGATGGAATGACACTTTAAAAAAGGTAGTCAAAAAATTAAGA---GAAAAATTTGGA------------AATAAAACA---ATAATCTTTGATCAA---------CACTCAGGAGGGGACCCAGAAATTGTAATGCACAGTTTTAATTGTGGAGGGGAATTTTTCTACTGTAATACAACAAAACTATTCAATAGTACTTGG---------------AATAGTACTAGG------------------------------AATGATACTGAAAGGAAT---------AGTAATGAAACT------------------------GACACAATCACACTCCCATGCAGAATAAAACAAATTATAAACATGTGGCAGGGAGTAGGAAAAGCAATGTATGCCCCTCCTATCAGAGGACTAATTAGATGTTCATCAAATATTACAGGGCTGTTATTAACAAGGGATGGTGGTAATACT------------AACAGGAGC---------------GGGAGCAACGGGTCT---GAGACCTTCAGACCTGGAGGAGGAAATATGAAGGACAATTGGAGA---AGTGAATTATATAAATATAAAGTAGTAAAACTTGAACCA---TTAGGAATAGCACCC---ACCAAGGCAAGGAGAAGAGTGGTGCAGAGAGAA---AAAAGAGCAGTA---GGA---CTAGGA---GCGTTG---TTCATT---GGG---------TTCTTGGGA---GCAGCAGGAAGCACTATGGGCGCAGCGTCAGTA---ACGCTGACGGTACAGGCCAGACAATTATTGTCTGGTATAGTGCAACAGCAGAACAATCTGCTGATGGCTATTGATGCACAACAGCATCTGTTGCAACTCACAGTCTGGGGCGTCAAGCAGCTCCGGGCAAGA---ATCCTGGCTGTGGAAAGATACCTAAAGGATCAACAGCTCCTAGGGATTTGGGGTTGCTCTGGAAAACTCATCTGCACCACTAATGTGCCTTGGAATGCTAGTTGGAGT---------------------------AATAAATCTCAAGAGCAAATTTGGGAA---AACATGACCTGGATGCAGTGGGAAAGAGAAATTGAC------AATTACACAGGCCTTATATACTCTTTAATTGAAAAATCGCAAAACCAACAAGAAAAGAATGAACAAGAATTATTGGCATTGGATAAGTGGGCAAGTTTGTGGAATTGGTTTGACATAACAAAATGGCTGTGGTATATAAAAATATTCATAATAATAGTAGGAGGCTTGATAGGATTAAGAATAGTTTTTGCTGTATTTTCTATAGTAAATAGAGTTAGGCAGGGATACTCACCACTATCGTTTCAGACCCAC---CTCCCAGTTCCGAGGGGA------CCCGACAGGCCCGAAGGAATCGACGAAGAAGGTGGAGAGCAAGGCAGAGACAGATCAATTCGATTAGTGGATGGATTCTTGGCACTTTTCTGGGACGACCTGAGGAGCCTGTGTCTTTTCAGCTACCACCACTTGAGAGACTTACTCTTGATTGCAGCGCGGATTGTGCAACTTCTGGGACAGAGG---------------GGGTGGGAGATCCTCAAATATTGGTGG---AATCTCCTGCTGTATTGG---------------------------------------------------AGTCAGGAACTAAAGAATAGTGCTGTCAGCTTGCTCAACACCACTGCTATAGTAGTAGCTGAGGGGACAGACAGGGTTATAGAAGCATTGCAAAGA------------------GCCTTTAGGGCTATTCTCCACATCCCTACAAGAATAAGACAAGGCTTGGAAAGGGCTTTACTATAA

2.04013226.ADARC.GU330395 ATGAAAGTGAAGGTGATCAGGAAGAATTGTCAGCACTTG---------TGGACATGGGGC------------------------ACGATGCTCCTTGGGATGTTAATGATC------------TGTAGTGCTGAA---------GATCAATTGTGGGTCACAGTTTATTATGGGGTACCTGTGTGGAAAGAAGCAACCACCACTCTATTTTGTGCATCAGATGCCAAAGGATATGATACAGAGGTACATAAT---GTTTGGGCCACACATGCCTGTGTACCCACAGACCCCAACCCACAAGAAGTGTTATTG---GCAAATGTGACAGAAAATTTTAACATGTGGAAAAATAACATGGTAGACCAGATGCATGAGGATGTAATCAGTTTATGGGATCAAAGTCTAAAGCCATGTGTAAAATTGACACCACTCTGTGTTACTTTATCTTGCACTAATGTGACTAATAATAATAATACTGCCAGT------------------------------------------------------------------------------------AATAGTTCTGATTGGGAAAAGATGGAG---GGAGAAATAAAAAACTGCTCTTTCAATGTCACC---CCAAGCATAAGAGAT------AGGGTGCGCAAAGAATATGCACTCTTTTATAGCCTTGATGTAGTACCTATAAAGGATACT------------------------------AATGATAGT---------------------AGAACCTATAGATTAATAAATTGTAACACCTCAGTCATTACACAGGCCTGTCCAAAGGTATCCTTTGAGCCAATTCCCATACATTATTGTGCCCCGGCTGGTTATGCGATTCTAAAATGT---AATAATAAGACTTTCAATGGAACAGGACCATGTACAAATGTCAGCACAGTACAATGTACACATGGAATTAGGCCTGTAGTATCAACTCAACTGTTGTTAAATGGCAGCCTAGCAGAAGAA---GACATAGTAATCAGATCTGAAAATCTCACAGACAATGCTAAAACCATAATAGTACATCTGAATGAATCTGTAGAAATTAATTGTACAAGACCCAACAATAATACAAGAAAAAGTATAAATATAGGA------------CCAGGGGCA---GCGATGTATGCAACAGGAGCCATAATAGGAGATATAAGACAAGCACATTGTAACATT------AGTGGAGCAAGATGGAATGACACTTTAAAAAAGGTAGTCAAAAAATTAAGA---GAAAAATTTGGA------------AATAAAACA---ATAATCTTTGATCAA---------CACTCAGGAGGGGACCCAGAAATTGTAATGCACAGTTTTAATTGTGGAGGGGAATTTTTCTACTGTAATACAACAAAACTATTCAATAGTACTTGG---------------AATAGTACTAGG------------------------------AATGATACTGAAAGGAAT---------AGTAATGAAACT------------------------GACACAATCACACTCCCATGCAGAATAAAACAAATTATAAACATGTGGCAGGGAGTAGGAAAAGCAATGTATGCCCCTCCTATCAGAGGACTAATTAGATGTTCATCAAATATTACAGGGCTGTTATTAACAAGGGATGGTGGTAATACT------------AACAGGAGC---------------GGGAGCAACGGGTCT---GAGACCTTCAGACCTGGAGGAGGAAATATGAAGGACAATTGGAGA---AGTGAATTATATAAATATAAAGTAGTAAAACTTGAACCA---TTAGGAATAGCACCC---ACCAAGGCAAGGAGAAGAGTGGTGCAGAGAGAA---AAAAGAGCAGTA---GGA---CTAGGA---GCGTTG---TTCATT---GGG---------TTCTTGGGA---GCAGCAGGAAGCACTATGGGCGCAGCGTCAGTA---ACGCTGACGGTACAGGCCAGACAATTATTGTCTGGTATAGTGCAACAGCAGAACAATCTGCTGATGGCTATTGATGCACAACAGCATCTGTTGCAACTCACAGTCTGGGGCGTCAAGCAGCTCCGGGCAAGA---ATCCTGGCTGTGGAAAGATACCTAAAGGATCAACAGCTCCTAGGGATTTGGGGTTGCTCTGGAAAACTCATCTGCACCACTAATGTGCCTTGGAATGCTAGTTGGAGT---------------------------AATAAATCTCAAGAGCAAATTTGGGAA---AACATGACCTGGATGCAGTGGGAAAGAGAAATTGAC------AATTACACAGGCCTTATATACTCTTTAATTGAAAAATCGCAAAACCAACAAGAAAAGAATGAACAAGAATTATTGGCATTGGATAAGTGGGCAAGTTTGTGGAATTGGTTTGACATAACAAAATGGCTGTGGTATATAAAAATATTCATAATAATAGTAGGAGGCTTGATAGGATTAAGAATAGTTTTTGCTGTATTTTCTATAGTAAATAGAGTTAGGCAGGGATACTCACCACTATCGTTTCAGACCCAC---CTCCCAGTTCCGAGGGGA------CCCGACAGGCCCGAAGGAATCGACGAAGAAGGTGGAGAGCAAGGCAGAGACAGATCAATTCGATTAGTGGATGGATTCTTGGCACTTTTCTGGGACGACCTGAGGAGCCTGTGTCTTTTCAGCTACCACCACTTGAGAGACTTACTCTTGATTGCAGCGCGGATTGTGCAACTTCTGGGACAGAGG---------------GGGTGGGAGATCCTCAAATATTGGTGG---AATCTCCTGCTGTATTGG---------------------------------------------------AGTCAGGAACTAAAGAATAGTGCTGTCAGCTTGCTCAACACCACTGCTATAGTAGTAGCTGAGGGGACAGACAGGGTTATAGAAGCATTGCAAAGA------------------GCCTTTAGGGCTATTCTCCACATCCCTACAAGAATAAGACAAGGCTTGGAAAGGGCTTTACTATAA

2.04013240.ADARC.GU330396 ATGAGAGTGAAGGGGATCATGAGGAATTATCAGCACTTA---------TGGAGATGGGGC------------------------ATGATGCTCCTTGGGATAATCATGATC------------TGTAGTGCTGCA---------GAACAATTGTGGGTCACAATCTATTATGGGGTACCTGTGTGGAAAGAAGCAACCACCACTCTATTTTGTGCATCAAATGCTAAAGCATATGATACAGAGGTACATAAT---GTCTGGGCCACACATGCCTGTGTACCCACAGACCCCAACCCACAAGAAGTAAGATTG---GAAAATGTGACAGAAAATTTTAACATGTGGAAAAATAATATGGTAGAACAGATGCATGAGGATATAATTAGCCTATGGGATCAAAGCCTAAAACCATGTGTGAAATTAACCCCACTCTGTGTTACTTTAAACTGCACTGATGCTAATACCACTAATACTAATGCCAAT------------------------------------------------------------------------------AGTACTAATAACAGTAGCTTGGGAACAATGGAGAAAGGAGAAATAAAAAACTGCTCTTTCAACATCACC---ACAAACCTAAGAGAT------AAGGTGCAGAAAGAATATGCACTTTTTTATAACCTTGATGTAGTGCCAATAAAGGGTGAGGAT---------------------------AATACTAGC---------------------------TATAGGTTGATAAGTTGTAATACCTCAGTCATTACACAGGCCTGTCCAAAGGTATCCTTTGAGCCAATTCCCATACATTATTGTACTCCAGCTGGTTTTGCGATTCTACAATGT---AATGATAAGAAATTCAATGGATCAGGACCATGTACAAATGTCAGCACAGTACAATGTACACATGGAATTAGGCCAGTAGTATCAACTCAACTGCTGTTAAATGGCAGTCTAGCAGAAAAA---GAGGTAGTAATTAGGTCTGAGAATTTCACAAATAATGCTAAAACCATAATAGTACAGCTAAATGAATCTGTAGTAATTAATTGTACAAGACCCAACAACAATACAAGAAAAAGTATACCTATAGGA------------CCAGGAAGA---GCATTTTATGCAACAGGAGAAATAATAGGAGATATAAGACAAGCACATTGTAACGTT------AGTACAAAAGCCTGGAAGGAAGCTTTACAACAGGTAGCTATAAAACTATCA---GAACAATTTGGG------------AATAAAACA---ATAGTCTTTAATCAA---------TCCTCAGGAGGAGACCCAGAAGTTGTAATGCACAGTTTTAATTGTAGAGGGGAATTTTTCTACTGTAATACAACAGGACTGTTTAATAATACTTGGGGGTTT---------AATAGTACTTGG---------------------GATGCTATTAATGTGCCAGAA------------------AATGACACA------------------------------ATCACACTCCCATGCAGAATAAAACAAATTGTAAACATGTGGCAGGAAGTAGGAAAAGCAATGTATGCCCCTCCCATCAGAGGACAAATTAATTGTTCATCAAATATTACAGGGCTGCTATTAACAAGAGATGGTGGT------------------AATACCACA---------------------AATAACACT---GAGGTCTTCAGACCTGGAGGAGGAGATATGAGAGACAATTGGAGA---AGTGAATTATATAAATATAAAGTAGTAAAAATTGAACCA---TTAGGAATAGCACCC---ACCAAGGCAAAGAGAAGAGTGGTGCAGAGAGAA---AAAAGAGCAGTG---GGA---ATAGGA---GCTTTG---TTCCTT---GGG---------TTCTTGGGA---GCAGCAGGAAGCACTATGGGCGCAGCGTCGATG---ACGCTGACGGTACAGGCCAGACTATTATTGTCTGGTATAGTGCAACAGCAGAACAATTTGCTGAGAGCTATTGAGGCGCAACAGCATCTGTTGCAACTCACAGTCTGGGGCATCAAGCAGCTCCAGGCAAGA---GTCCTGGCTGTGGAAAGATACCTAAGGGATCAACAGCTCCTGGGGATTTGGGGATGCTCTGGAAAACTCATTTGCACCACTGCTGTGCCTTGGAATGTTAGTTGGAGT---------------------------AATAAATCCATGAATGACATTTGGAAT---AACATGACCTGGATGGAGTGGGAAAGAGAGATTGAC------AATTATACAAACATAATATACACCTTACTTGAAGAATCGCAGAACCAACAAGATAAGAATGAACAGGAATTATTGGAATTGGATAAATGGGCAAGTTTGTGGAATTGGTTTAGCATAACAAATTGGCTGTGGTACATAAAAATATTCATAATGATAGTAGGAGGCTTAATAGGTTTAAGAATAGTTTTTACTGTATTTTCTATAGTGAATAGAGTCAGGCAGGGATATTCACCATTATCGTTTCAGACCCGC---CTCCCAACTTCGAGGGGA------CTCGACAGGCCCGAAGGAATCGAAGAAGAAGGTGGAGACAGAGACAGAGACAGATCCAGGCCATTAGTGGATGGATTCTTAGCAATTATCTGGGTCGACCTGCGGAGCCTGTGCCTCTTCAGCTACCATCGCTTGAGAGACTTACTCTTGATTGTAGCGAGGATTGTGGAACTTCTGGGACGCAGG---------------GGGTGGGAAGCCCTCAAATATTGGTGG---AATCTCCTGCGGTATTGG---------------------------------------------------AGCCAGGAACTAAGGAATAGTGCTATTAGCTTGCTTAATGCCACAGCCATAGCAGTAGCTGAGGGAACAGATAGGGTGTTAGAAGTATTACAAAGA------------------GCTTTTAGAGCTGTTATACACATACCTAGAAGAATAAGACAGGGCTTAGAAAGGTTTTTGCTATAA

2.04013240.ADARC.GU330397 ATGAGAGTGAAGGGGATCATGAGGAATTATCAGCACTTA---------TGGAGATGGGGC------------------------ATGATGCTCCTTGGGATAATCATGATC------------TGTAGTGCTGCA---------GAACAATTGTGGGTCACAATCTATTATGGGGTACCTGTGTGGAAAGAAGCAACCACCACTCTATTTTGTGCATCAAATGCTAAAGCATATGATACAGAGGTACATAAT---GTCTGGGCCACACATGCCTGTGTACCCACAGACCCCAACCCACAAGAAGTAAGATTG---GAAAATGTGACAGAAAATTTTAACATGTGGAAAAATAATATGGTAGAACAGATGCATGAGGATATAATTAGCCTATGGGATCAAAGCCTAAAACCATGTGTGAAATTAACCCCACTCTGTGTTACTTTAAACTGCACTGATGCTAATACCACTAATACTAATGCCAAT------------------------------------------------------------------------------AGTACTAATAACAGTAGCTTGGGAACAATGGAGAAAGGAGAAATAAAAAACTGCTCTTTCAACATCACC---ACAAACCTAAGAGAT------AAGGTGCAGAAAGAATATGCACTTTTTTATAACCTTGATGTAGTGCCAATAAAGGGTGAGGAT---------------------------AATACTAGC---------------------------TATAGGTTGATAAGTTGTAATACCTCAGTCATTACACAGGCCTGTCCAAAGGTATCCTTTGAGCCAATTCCCATACATTATTGTACTCCAGCTGGTTTTGCGATTCTACAATGT---AATGATAAGAAATTCAATGGATCAGGACCATGTACAAATGTCAGCACAGTACAATGTACACATGGAATTAGGCCAGTAGTATCAACTCAACTGCTGTTAAATGGCAGTCTAGCAGAAAAA---GAGGTAGTAATTAGGTCTGAGAATTTCACAAATAATGCTAAAACCATAATAGTACAGCTAAATGAATCTGTAGTAATTAATTGTACAAGACCCAACAACAATACAAGAAAAAGTATACCTATAGGA------------CCAGGAAGA---GCATTTTATGCAACAGGAGAAATAATAGGAGATATAAGACAAGCACATTGTAACGTT------AGTACAAAAGCCTGGAAGGAAGCTTTACAACAGGTAGCTATAAAACTATCA---GAACAATTTGGG------------AATAAAACA---ATAGTCTTTAATCAA---------TCCTCAGGAGGAGACCCAGAAGTTGTAATGCACAGTTTTAATTGTAGAGGGGAATTTTTCTACTGTAATACAACAGGACTGTTTAATAATACTTGGGGGTTT---------AATAGTACTTGG---------------------GATGCTATTAATGTGCCAGAA------------------AATGACACA------------------------------ATCACACTCCCATGCAGAATAAAACAAATTGTAAACATGTGGCAGGAAGTAGGAAAAGCAATGTATGCCCCTCCCATCAGAGGACGACTTAATTGTTCATCAAATATTACAGGGCTGCTATTAACAAGAGATGGTGGT------------------AATACCACA---------------------AATAACACT---GAGGTCTTCAGACCTGGAGGAGGAGATATGAGAGACAATTGGAGA---AGTGAATTATATAAATATAAAGTAGTAAAAATTGAACCA---TTAGGAATAGCACCC---ACCAAGGCAAAGAGAAGAGTGGTGCAGAGAGAA---AAAAGAGCAGTG---GGA---ATAGGA---GCTTTG---TTCCTT---GGG---------TTCTTGGGA---GCAGCAGGAAGCACTATGGGCGCAGCGTCGATG---ACGCTGACGGTACAGGCCAGACTATTATTGTCTGGTATAGTGCAACAGCAGAACAATTTGCTGAGAGCTATTGAGGCGCAACAGCATCTGTTGCAACTCACAGTCTGGGGCATCAAGCAGCTCCAGGCAAGA---GTCCTGGCTGTGGAAAGATACCTAAGAGATCAACAGCTCCTGGGGATTTGGGGATGCTCTGGAAAACTCATTTGCACCACTACTGTGCCTTGGAATGTTAGTTGGAGT---------------------------AATAAATCCATGAATGACATTTGGAAT---AACATGACCTGGATGGAGTGGGAAAGAGAGATTGAC------AATTATACAAACATAATATACACCTTACTTGAAGAATCGCAGAACCAACAAGATAAGAATGAACAGGAATTATTGGAATTGGATAAATGGGCAAGTTTGTGGAATTGGTTTAGCATAACAAATTGGCTGTGGTACATAAAAATATTCATAATGATAGTAGGAGGCTTAATAGGTTTAAGAATAGTTTTTACTGTATTTTCTATAGTGAATAGAGTTAGGCAGGGATATTCACCATTATCGTTTCAGACCCGC---TTCCCAACCTCGAGGGGA------CTCGACAGGCCCGAAGGAATCGAAGAAGAAGGTGGAGACAGAGACAGAGACAGATCCAGGCCATTAGTGGATGGATTCTTAGCAATTATCTGGGTCGACCTGCGGAGCCTGTGCCTCTTCAGCTACCATCGCTTGAGAGACTTACTCTTGATTGTAGCGAGGATTGTGGAACTTCTGGGACGCAGG---------------GGGTGGGAAGCCCTCAAATATTGGTGG---AATCTCCTGCGGTATTGG---------------------------------------------------AGCCAGGAACTAAGGAATAGTGCTATTAGCTTGCTTAATGCCACAGCCATAGCAGTAGCTGAGGGAACAGATAGGGTGTTAGAAGTATTACAAAGA------------------GCTTTTAGAGCTGTTATACACATACCTAGAAGAATAAGACAGGGCTTAGAAAGGTTTTTGCTATAA

2.04013240.ADARC.GU330398 ATGAGAGTGAAGGGGATCATGAGGAATTATCAGCACTTA---------TGGAGATGGGGC------------------------ATGATGCTCCTTGGGATAATCATGATC------------TGTAGTGCTGCA---------GAACAATTGTGGGTCACAATCTATTATGGGGTACCTGTGTGGAAAGAAGCAACCACCACTCTATTTTGTGCATCAAATGCTAAAGCATATGATACAGAGGTACATAAT---GTCTGGGCCACACATGCCTGTGTACCCACAGACCCCAACCCACAAGAAGTAAGATTG---GAAAATGTGACAGAAAATTTTAACATGTGGAAAAATAATATGGTAGAACAGATGCATGAGGATATAATTAGCCTATGGGATCAAAGCCTAAAACCATGTGTGAAATTAACCCCACTCTGTGTTACTTTAAACTGCACTGATGCTAATACCACTAATACTAATGCCAAT------------------------------------------------------------------------------AGTACTAATAACAGTAGCTTGGGAACAATGGAGAAAGGAGAAATAAAAAACTGCTCTTTCAACATCACC---ACAAACCTAAGAGAT------AAGGTGCAGAAAGAATATGCACTTTTTTATAACCTTGATGTAGTGCCAATAAAGGGTGAGGAT---------------------------AATACTAGC---------------------------TATAGGTTGATAAGTTGTAATACCTCAGTCATTACACAGGCCTGTCCAAAGGTATCCTTTGAGCCAATTCCCATACATTATTGTACTCCAGCTGGTTTTGCGATTCTACAATGT---AATGATAAGAAATTCAATGGATCAGGACCATGTACAAATGTCAGCACAGTACAATGTACACATGGAATTAGGCCAGTAGTATCAACTCAACTGCTGTTAAATGGCAGTCTAGCAGAAAAA---GAGGTAGTAATTAGGTCTGAGAATTTCACAAATAATGCTAAAACCATAATAGTACAGCTAAATGAATCTGTAGTAATTAATTGTACAAGACCCAACAACAATACAAGAAAAAGTATACCTATAGGA------------CCAGGAAGA---GCATTTTATGCAACAGGAGAAATAATAGGAGATATAAGACAAGCACATTGTAACGTT------AGTACAAAAGCCTGGAAGGAAGCTTTACAACAGGTAGCTATAAAACTATCA---GAACAATTTGGG------------AATAAAACA---ATAGTCTTTAATCAA---------TCCTCAGGAGGAGACCCAGAAGTTGTAATGCACAGTTTTAATTGTAGAGGGGAATTTTTCTACTGTAATACAACAGGACTGTTTAATAATACTTGGGGGTTT---------AATAGTACTTGG---------------------GATGCTATTAATGTGCCAGAA------------------AATGACACA------------------------------ATCACACTCCCATGCAGAATAAAACAAATTGTAAACATGTGGCAGGAAGTAGGAAAAGCAATGTATGCCCCTCCCATCAGAGGACGACTTAATTGTTCATCAAATATTACAGGGCTGCTATTAACAAGAGATGGTGGT------------------AATACCACA---------------------AATAACACT---GAGGTCTTCAGACCTGGAGGAGGAGATATGAGAGACAATTGGAGA---AGTGAATTATATAAATATAAAGTAGTAAAAATTGAACCA---TTAGGAATAGCACCC---ACCAAGGCAAAGAGAAGAGTGGTGCAGAGAGAA---AAAAGAGCAGTG---GGA---ATAGGA---GCTTTG---TTCCTT---GGG---------TTCTTGGGA---GCAGCAGGAAGCACTATGGGCGCAGCGTCGATG---ACGCTGACGGTACAGGCCAGACTATTATTGTCTGGTATAGTGCAACAGCAGAACAATTTGCTGAGAGCTATTGAGGCGCAACAGCATCTGTTGCAACTCACAGTCTGGGGCATCAAGCAGCTCCAGGCAAGA---GTCCTGGCTGTGGAAAGATACCTAAGAGATCAACAGCTCCTGGGGATTTGGGGATGCTCTGGAAAACTCATTTGCACCACTACTGTGCCTTGGAATGTTAGTTGGAGT---------------------------AATAAATCCATGAATGACATTTGGAAT---AACATGACCTGGATGGAGTGGGAAAGAGAGATTGAC------AATTATACAAACATAATATACACCTTACTTGAAGAATCGCAGAACCAACAAGATAAGAATGAACAGGAATTATTGGAATTGGATAAATGGGCAAGTTTGTGGAATTGGTTTAGCATAACAAATTGGCTGTGGTACATAAAAATATTCATAATGATAGTAGGAGGCTTAATAGGTTTAAGAATAGTTTTTACTGTATTTTCTATAGTGAATAGAGTTAGGCAGGGATATTCACCATTATCGTTTCAGACCCGC---TTCCCAACCTCGAGGGGA------CTCGACAGGCCCGAAGGAATCGAAGAAGAAGGTGGAGACAGAGACAGAGACAGATCCAGGCCATTAGTGGATGGATTCTTAGCAATTATCTGGGTCGACCTGCGGAGCCTGTGCCTCTTCAGCTACCATCGCTTGAGAGACTTACTCTTGATTGTAGCGAGGATTGTGGAACTTCTGGGACGCAGG---------------GGGTGGGAAGCCCTCAAATATTGGTGG---AATCTCCTGCGGTATTGG---------------------------------------------------AGCCAGGAACTAAGGAATAGTGCTATTAGCTTGCTTAATGCCACAGCCATAGCAGTAGCTGAGGGAACAGATAGGGTGTTAGAAGTATTACAAAGA------------------GCTTTTAGAGCTGTTATACACATACCTAGAAGAATAAGACAGGGCTTAGAAAGGTTTTTGCTATAA

2.04013240.ADARC.GU330399 ATGAGAGTGAAGGGGATCATGAGGAATTATCAGCACTTA---------TGGAGATGGGGC------------------------ATGATGCTCCTTGGGATAATCATGATC------------TGTAGTGCTGCA---------GAACAATTGTGGGTCACAATCTATTATGGGGTACCTGTGTGGAAAGAAGCAACCACCACTCTATTTTGTGCATCAAATGCTAAAGCATATGATACAGAGGTACATAAT---GTCTGGGCCACACATGCCTGTGTACCCACAGACCCCAACCCACAAGAAGTAAGATTG---GAAAATGTGACAGAAAATTTTAACATGTGGAAAAATAATATGGTAGAACAGATGCATGAGGATATAATTAGCCTATGGGATCAAAGCCTAAAACCATGTGTGAAATTAACCCCACTCTGTGTTACTTTAAACTGCACTGATGCTAATACCACTAATACTAATGCCAAT------------------------------------------------------------------------------AGTACTAATAACAGTAGCTTGGGAACAATGGAGAAAGGAGAAATAAAAAACTGCTCTTTCAACATCACC---ACAAACCTAAGAGAT------AAGGTGCAGAAAGAATATGCACTTTTTTATAACCTTGATGTAGTGCCAATAAAGGGTGAGGAT---------------------------AATACTAGC---------------------------TATAGGTTGATAAGTTGTAATACCTCAGTCATTACACAGGCCTGTCCAAAGGTATCCTTTGAGCCAATTCCCATACATTATTGTACTCCAGCTGGTTTTGCGATTCTACAATGT---AATGATAAGAAATTCAATGGATCAGGACCATGTACAAATGTCAGCACAGTACAATGTACACATGGAATTAGGCCAGTAGTATCAACTCAACTGCTGTTAAATGGCAGTCTAGCAGAAAAA---GAGGTAGTAATTAGGTCTGAGAATTTCACAAATAATGCTAAAACCATAATAGTACAGCTAAATGAATCTGTAGTAATTAATTGTACAAGACCCAACAACAATACAAGAAAAAGTATACCTATAGGA------------CCAGGAAGA---GCATTTTATGCAACAGGAGAAATAATAGGAGATATAAGACAAGCACATTGTAACGTT------AGTACAAAAGCCTGGAAGGAAGCTTTACAACAGGTAGCTATAAAACTATCA---GAACAATTTGGG------------AATAAAACA---ATAGTCTTTAATCAA---------TCCTCAGGAGGAGACCCAGAAGTTGTAATGCACAGTTTTAATTGTAGAGGGGAATTTTTCTACTGTAATACAACAGGACTGTTTAATAATACTTGGGGGTTT---------AATAGTACTTGG---------------------GATGCTATTAATGTGCCAGAA------------------AATGACACA------------------------------ATCACACTCCCATGCAGAATAAAACAAATTGTAAACATGTGGCAGGAAGTAGGAAAAGCAATGTATGCCCCTCCCATCAGAGGACGACTTAATTGTTCATCAAATATTACAGGGCTGCTATTAACAAGAGATGGTGGT------------------AATACCACA---------------------AATAACACT---GAGGTCTTCAGACCTGGAGGAGGAGATATGAGAGACAATTGGAGA---AGTGAATTATATAAATATAAAGTAGTAAAAATTGAACCA---TTAGGAATAGCACCC---ACCAAGGCAAAGAGAAGAGTGGTGCAGAGAGAA---AAAAGAGCAGTG---GGA---ATAGGA---GCTTTG---TTCCTT---GGG---------TTCTTGGGA---GCAGCAGGAAGCACTATGGGCGCAGCGTCGATG---ACGCTGACGGTACAGGCCAGACTATTATTGTCTGGTATAGTGCAACAGCAGAACAATTTGCTGAGAGCTATTGAGGCGCAACAGCATCTGTTGCAACTCACAGTCTGGGGCATCAAGCAGCTCCAGGCAAGA---GTCCTGGCTGTGGAAAGATACCTAAGAGATCAACAGCTCCTGGGGATTTGGGGATGCTCTGGAAAACTCATTTGCACCACTACTGTGCCTTGGAATGTTAGTTGGAGT---------------------------AATAAATCCATGAATGACATTTGGAAT---AACATGACCTGGATGGAGTGGGAAAGAGAGATTGAC------AATTATACAAACATAATATACACCTTACTTGAAGAATCGCAGAACCAACAAGATAAGAATGAACAGGAATTATTGGAATTGGATAAATGGGCAAGTTTGTGGAATTGGTTTAGCATAACAAATTGGCTGTGGTACATAAAAATATTCATAATGATAGTAGGAGGCTTAATAGGTTTAAGAATAGTTTTTACTGTATTTTCTATAGTGAATAGAGTTAGGCAGGGATATTCACCATTATCGTTTCAGACCCGC---TTCCCAACCTCGAGGGGA------CTCGACAGGCCCGAAGGAATCGAAGAAGAAGGTGGAGACAGAGACAGAGACAGATCCAGGCCATTAGTGGATGGATTCTTAGCAATTATCTGGGTCGACCTGCGGAGCCTGTGCCTCTTCAGCTACCATCGCTTGAGAGACTTACTCTTGATTGTAGCGAGGATTGTGGAACTTCTGGGACGCAGG---------------GGGTGGGAAGCCCTCAAATATTGGTGG---AATCTCCTGCGGTATTGG---------------------------------------------------AGCCAGGAACTAAGGAATAGTGCTATTAGCTTGCTTAATGCCACAGCCATAGCAGTAGCTGAGGGAACAGATAGGGTGTTAGAAGTATTACAAAGA------------------GCTTTTAGAGCTGTTATACACATACCTAGAAGAATAAGACAGGGCTTAGAAAGGTTTTTGCTATAA

2.04013240.ADARC.GU330400 ATGAGAGTGAAGGGGATCATGAGGAATTATCAGCACTTA---------TGGAGATGGGGC------------------------ATGATGCTCCTTGGGATAATCATGATC------------TGTAGTGCTGCA---------GAACAATTGTGGGTCACAATCTATTATGGGGTACCTGTGTGGAAAGAAGCAAACACCACTCTATTTTGTGCATCAAATGCTAAAGCATATGATACAGAGGTACATAAT---GTCTGGGCCACACATGCCTGTGTACCCACAGACCCCAACCCACAAGAAGTAAGATTG---GAAAATGTGACAGAAAATTTTAACATGTGGAAAAATAATATGGTAGAACAGATGCATGAGGATATAATTAGCCTATGGGATCAAAGCCTAAAACCATGTGTGAAATTAACCCCACTCTGTGTTACTTTAAACTGCACTGATGCTAATACCACTAATACTAATGCCAAT------------------------------------------------------------------------------AGTACTAATAACAGTAGCTTGGGAACAATGGAGAAAGGAGAAATAAAAAACTGCTCTTTCAACATCACC---ACAAACCTAAGAGAT------AAGGTGCAGAAAGAATATGCACTTTTTTATAACCTTGATGTAGTGCCAATAAAGGGTGAGGAT---------------------------AATACTAGC---------------------------TATAGGTTGATAAGTTGTAATACCTCAGTCATTACACAGGCCTGTCCAAAGGTATCCTTTGAGCCAATTCCCATACATTATTGTACTCCAGCTGGTTTTGCGATTCTACAATGT---AATGATAAGAAATTCAATGGATCAGGACCATGTACAAATGTCAGCACAGTACAATGTACACATGGAATTAGGCCAGTAGTATCAACTCAACTGCTGTTAAATGGCAGTCTAGCAGAAAAA---GAGGTAGTAATTAGGTCTGAGAATTTCACAAATAATGCTAAAACCATAATAGTACAGCTAAATGAATCTGTAGTAATTAATTGTACAAGACCCAACAACAATACAAGAAAAAGTATACCTATAGGA------------CCAGGAAGA---GCATTTTATGCAACAGGAGAAATAATAGGAGATATAAGACAAGCACATTGTAACGTT------AGTACAAAAGCCTGGAAGGAAGCTTTACAACAGGTAGCTATAAAACTATCA---GAACAATTTGGG------------AATAAAACA---ATAGTCTTTAATCAA---------TCCTCAGGAGGAGACCCAGAAGTTGTAATGCACAGTTTTAATTGTAGAGGGGAATTTTTCTACTGTAATACAACAGGACTGTTTAATAATACTTGGGGGTTT---------AATAGTACTTGG---------------------GATGCTATTAATGTGCCAGAA------------------AATGACACA------------------------------ATCACACTCCCATGCAGAATAAAACAAATTGTAAACATGTGGCAGGAAGTAGGAAAAGCAATGTATGCCCCTCCCATCAGAGGACGACTTAATTGTTCATCAAATATTACAGGGCTGCTATTAACAAGAGATGGTGGT------------------AATACCACA---------------------AATAACACT---GAGGTCTTCAGACCTGGAGGAGGAGATATGAGAGACAATTGGAGA---AGTGAATTATATAAATATAAAGTAGTAAAAATTGAACCA---TTAGGAATAGCACCC---ACCAAGGCAAAGAGAAGAGTGGTGCAGAGAGAA---AAAAGAGCAGTG---GGA---ATAGGA---GCTTTG---TTCCTT---GGG---------TTCTTGGGA---GCAGCAGGAAGCACTATGGGCGCAGCGTCGATG---ACGCTGACGGTACAGGCCAGACTATTATTGTCTGGTATAGTGCAACAGCAGAACAATTTGCTGAGAGCTATTGAGGCGCAACAGCATCTGTTGCAACTCACAGTCTGGGGCATCAAGCAGCTCCAGGCAAGA---GTCCTGGCTGTGGAAAGATACCTAAGAGATCAACAGCTCCTGGGGATTTGGGGATGCTCTGGAAAACTCATTTGCACCACTACTGTGCCTTGGAATGTTAGTTGGAGT---------------------------AATAAATCCATGAATGACATTTGGAAT---AACATGACCTGGATGGAGTGGGAAAGAGAGATTGAC------AATTATACAAACATAATATACACCTTACTTGAAGAATCGCAGAACCAACAAGATAAGAATGAACAGGAATTATTGGAATTGGATAAATGGGCAAGTTTGTGGAATTGGTTTAGCATAACAAATTGGCTGTGGTACATAAAAATATTCATAATGATAGTAGGAGGCTTAATAGGTTTAAGAATAGTTTTTACTGTATTTTCTATAGTGAATAGAGTTAGGCAGGGATATTCACCATTATCGTTTCAGACCCGC---CTCCCAACTTCGAGGGGA------CTCGACAGGCCCGAAGGAATCGAAGAAGAAGGTGGAGACAGAGACAGAGACAGATCCAGGCCATTAGTGGATGGATTCTTAGCAATTATCTGGGTCGACCTGCGGAGCCTGTGCCTCTTCAGCTACCATCGCTTGAGAGACTTACTCTTGATTGTAGCGAGGATTGTGGAACTTCTGGGACGCAGG---------------GGGTGGGAAGCCCTCAAATATTGGTGG---AATCTCCTGCGGTATTGG---------------------------------------------------AGCCAGGAACTAAGGAATAGTGCTATTAGCTTGCTTAATGCCACAGCCATAGCAGTAGCTGAGGGAACAGATAGGGTGTTAGAAGTATTACAAAGA------------------GCTTTTAGAGCTGTTATACACATACCTAGAAGAATAAGACAGGGCTTAGAAAGGTTTTTGCTATAA

2.04013240.ADARC.GU330401 ATGAGAGTGAAGGGGATCATGAGGAATTATCAGCACTTA---------TGGAGATGGGGC------------------------ATGATGCTCCTTGGGATAATCATGATC------------TGTAGTGCTGCA---------GAACAATTGTGGGTCACAATCTATTATGGGGTACCTGTGTGGAAAGAAGCAACCACCACTCTATTTTGTGCATCAAATGCTAAAGCATATGATACAGAGGTACATAAT---GTCTGGGCCACACATGCCTGTGTACCCACAGACCCCAACCCACAAGAAGTAAGATTG---GAAAATGTGACAGAAAATTTTAACATGTGGAAAAATAATATGGTAGAACAGATGCATGAGGATATAATTAGCCTATGGGATCAAAGCCTAAAACCATGTGTGAAATTAACCCCACTCTGTGTTACTTTAAACTGCACTGATGCTAATACCACTAATACTAATGCCAAT------------------------------------------------------------------------------AGTACTAATAACAGTAGCTTGGGAACAATGGAGAAAGGAGAAATAAAAAACTGCTCTTTCAACATCACC---ACAAACCTAAGAGAT------AAGGTGCAGAAAGAATATGCACTTTTTTATAACCTTGATGTAGTGCCAATAAAGGGTGAGGAT---------------------------AATACTAGC---------------------------TATAGGTTGATAAGTTGTAATACCTCAGTCATTACACAGGCCTGTCCAAAGGTATCCTTTGAGCCAATTCCCATACATTATTGTACTCCAGCTGGTTTTGCGATTCTACAATGT---AATGATAAGAAATTCAATGGATCAGGACCATGTACAAATGTCAGCACAGTACAATGTACACATGGAATTAGGCCAGTAGTATCAACTCAACTGCTGTTAAATGGCAGTCTAGCAGAAAAA---GAGGTAGTAATTAGGTCTGAGAATTTCACAAATAATGCTAAAACCATAATAGTACAGCTAAATGAATCTGTAGTAATTAATTGTACAAGACCCAACAACAATACAAGAAAAAGTATACCTATAGGA------------CCAGGAAGA---GCATTTTATGCAACAGGAGAAATAATAGGAGATATAAGACAAGCACATTGTAACGTT------AGTACAAAAGCCTGGAAGGAAGCTTTACAACAGGTAGCTATAAAACTATCA---GAACAATTTGGG------------AATAAAACA---ATAGTCTTTAATCAA---------TCCTCAGGAGGAGACCCAGAAGTTGTAATGCACAGTTTTAATTGTAGAGGGGAATTTTTCTACTGTAATACAACAGGACTGTTTAATAATACTTGGGGGTTT---------AATAGTACTTGG---------------------GATGCTATTAATGTGCCAGAA------------------AATGACACA------------------------------ATCACACTCCCATGCAGAATAAAACAAATTGTAAACATGTGGCAGGAAGTAGGAAAAGCAATGTATGCCCCTCCCATCAGAGGACGACTTAATTGTTCATCAAATATTACAGGGCTGCTATTAACAAGAGATGGTGGT------------------AATACCACA---------------------AATAACACT---GAGGTCTTCAGACCTGGAGGAGGAGATATGAGAGACAATTGGAGA---AGTGAATTATATAAATATAAAGTAGTAAAAATTGAACCA---TTAGGAATAGCACCC---ACCAAGGCAAAGAGAAGAGTGGTGCAGAGAGAA---AAAAGAGCAGTG---GGA---ATAGGA---GCTTTG---TTCCTT---GGG---------TTCTTGGGA---GCAGCAGGAAGCACTATGGGCGCAGCGTCGATG---ACGCTGACGGTACAGGCCAGACTATTATTGTCTGGTATAGTGCAACAGCAGAACAATTTGCTGAGAGCTATTGAGGCGCAACAGCATCTGTTGCAACTCACAGTCTGGGGCATCAAGCAGCTCCAGGCAAGA---GTCCTGGCTGTGGAAAGATACCTAAGAGATCAACAGCTCCTGGGGATTTGGGGATGCTCTGGAAAACTCATTTGCACCACTACTGTGCCTTGGAATGTTAGTTGGAGT---------------------------AATAAATCCATGAATGACATTTGGAAT---AACATGACCTGGATGGAGTGGGAAAGAGAGATTGAC------AATTATACAAACATAATATACACCTTACTTGAAGAATCGCAGAACCAACAGGATAAGAATGAACAGGAATTATTGGAATTGGATAAATGGGCAAGTTTGTGGAATTGGTTTAGCATAACAAATTGGCTGTGGTACATAAAAATATTCATAATGATAGTAGGAGGCTTAATAGGTTTAAGAATAGTTTTTACTGTATTTTTTATAGTGAATAGAGTTAGGCAGGGATATTCACCATTATCGTTTCAGACCCGC---TTCCCAACCTCGAGGGGA------CTCGACAGGCCCGAAGGAATCGAAGAAGAAGGTGGAGACAGAGACAGAGACAGATCCAGGCCATTAGTGGATGGATTCTTAGCAATTATCTGGGTCGACCTGCGGAGCCTGTGCCTCTTCAGCTACCATCGCTTGAGAGACTTACTCTTGATTGTAGCGAGGATTGTGGAACTTCTGGGACGCAGG---------------GGGTGGGAAGCCCTCAAATATTGGTGG---AATCTCCTGCGGTATTGG---------------------------------------------------AGCCAGGAACTAAGGAATAGTGCTATTAGCTTGCTTAATGCCACAGCCATAGCAGTAGCTGAGGGAACAGATAGGGTGGTAGAAGTATTACAAAGA------------------GCTTTTAGAGCTGTTATACACATACCTAGAAGAATAAGACAGGGCTTAGAAAGGTTTTTGCTATAA

2.04013240.ADARC.GU330402 ATGAGAGTGAAGGGGATCATGAGGAATTATCAGCACTTA---------TGGAGATGGGGC------------------------ATGATGCTCCTTGGGATAATCATGATC------------TGTAGTGCTGCA---------GAACAATTGTGGGTCACAATCTATTATGGGGTACCTGTGTGGAAAGAAGCAACCACCACTCTATTTTGTGCATCAAATGCTAAAGCATATGATACAGAGGTACATAAT---GTCTGGGCCACACATGCCTGTGTACCCACAGACCCCAACCCACAAGAAGTAAGATTG---GAAAATGTGACAGAAAATTTTAACATGTGGAAAAATAATATGGTAGAACAGATGCATGAGGATATAATTAGCCTATGGGATCAAAGCCTAAAACCATGTGTGAAATTAACCCCACTCTGTGTTACTTTAAACTGCACTGATGCTAATACCACTAATACTAATGCCAAT------------------------------------------------------------------------------AGTACTAATAACAGTAGCTTGGGAACAATGGAGAAAGGAGAAATAAAAAACTGCTCTTTCAACATCACC---ACAAACCTAAGAGAT------AAGGTGCAGAAAGAATATGCACTTTTTTATAACCTTGATGTAGTGCCAATAAAGGGTGAGGAT---------------------------AATACTAGC---------------------------TATAGGTTGATAAGTTGTAATACCTCAGTCATTACACAGGCCTGTCCAAAGGTATCCTTTGAGCCAATTCCCATACATTATTGTACTCCAGCTGGTTTTGCGATTCTACAATGT---AATGATAAGAAATTCAATGGATCAGGACCATGTACAAATGTCAGCACAGTACAATGTACACATGGAATTAGGCCAGTAGTATCAACTCAACTGCTGTTAAATGGCAGTCTAGCAGAAAAA---GAGGTAGTAATTAGGTCTGAGAATTTCACAAATAATGCTAAAACCATAATAGTACAGCTAAATGAATCTGTTGTAATTAATTGTACAAGACCCAACAACAATACAAGAAAAAGTATACCTATAGGA------------CCAGGAAGA---GCATTTTATGCAACAGGAGAAATAATAGGAGATATAAGACAAGCACATTGTAACGTT------AGTACAAAAGCCTGGAAGGAAGCTTTACAACAGGTAGCTATAAAACTATCA---GAACAATTTGGG------------AATAAAACA---ATAGTCTTTAATCAA---------TCCTCAGGAGGAGACCCAGAAGTTGTAATGCACAGTTTTAATTGTAGAGGGGAATTTTTCTACTGTAATACAACAGGACTGTTTAATAATACTTGGGGGTTT---------AATAGTACTTGG---------------------GATGCTATTAATGTGCCAGAA------------------AATGACACA------------------------------ATCACACTCCCATGCAGAATAAAACAAATTGTAAACATGTGGCAGGAAGTAGGAAAAGCAATGTATGCCCCTCCCATCAGAGGACAAATTAATTGTTCATCAAATATTACAGGGCTGCTATTAACAAGAGATGGTGGT------------------AATACCACA---------------------AATAACACT---GAGGTCTTCAGACCTGGAGGAGGAGATATGAGAGACAATTGGAGA---AGTGAATTATATAAATATAAAGTAGTAAAAATTGAACCA---TTAGGAATAGCACCC---ACCAAGGCAAAGAGAAGAGTGGTGCAGAGAGAA---AAAAGAGCAGTG---GGA---ATAGGA---GCTTTG---TTCCTT---GGG---------TTCTTGGGA---GCAGCAGGAAGCACTATGGGCGCAGCGTCGATG---ACGCTGACGGTACAGGCCAGACTATTATTGTCTGGTATAGTGCAACAGCAGAACAATTTGCTGAGAGCTATTGAGGCGCAACAGCATCTGTTGCAACTCACAGTCTGGGGCATCAAGCAGCTCCAGGCAAGA---GTCCTGGCTGTGGAAAGATACCTAAGGGATCAACAGCTCCTGGGGATTTGGGGATGCTCTGGAAAACTCATTTGCACCACTGCTGTGCCTTGGAATGTTAGTTGGAGT---------------------------AATAAATCCATGAATGACATTTGGAAT---AACATGACCTGGATGGAGTGGGAAAGAGAGATTGAC------AATTATACAAACATAATATACACCTTACTTGAAGAATCGCAGAACCAACAAGATAAGAATGAACAGGAATTATTGGAATTGGATAAATGGGCAAGTTTGTGGAATTGGTTTAGCATAACAAATTGGCTGTGGTACATAAAAATATTCATAATGATAGTAGGAGGCTTAATAGGTTTAAGAATAGTTTTTACTGTATTTTCTATAGTGAATAGAGTTAGGCAGGGATATTCACCATTATCGTTTCAGACCCGC---CTCCCAACTTCGAGGGGA------CTCGACAGGCCCGAAGGAATCGAAGAAGAAGGTGGAGACAGAGACAGAGACAGATCCAGGCCATTAGTGGATGGATTCTTAGCAATTATCTGGGTCGACCTGCGGAGCCTGTGCCTCTTCAGCTACCATCGCTTGAGAGACTTACTCTTGATTGTAGCGAGGATTGTGGAACTTCTGGGACGCAGG---------------GGGTGGGAAGCCCTCAAATATTGGTGG---AATCTCCTGCGGTATTGG---------------------------------------------------AGCCAGGAACTAAGGAATAGTGCTATTAGCTTGCTTAATGCCACAGCCATAGCAGTAGCTGAGGGAACAGATAGGGTGTTAGAAGTATTACAAAGA------------------GCTTTTAGAGCTGTTATACACATACCTAGAAGAATAAGACAGGGCTTAGAAAGGTTTTTGCTATAA

2.04013240.ADARC.GU330403 ATGAGAGTGAAGGGGATCATGAGGAATTATCAGCACTTA---------TGGAGATGGGGC------------------------ATGATGCTCCTTGGGATAATCATGATC------------TGTAGTGCTGCA---------GAACAATTGTGGGTCACAATCTATTATGGGGTACCTGTGTGGAAAGAAGCAACCACCACTCTATTTTGTGCATCAAATGCTAAAGCATATGATACAGAGGTACATAAT---GTCTGGGCCACACATGCCTGTGTACCCACAGACCCCAACCCACAAGAAGTAAGATTG---GAAAATGTGACAGAAAATTTTAACATGTGGAAAAATAATATGGTAGAACAGATGCATGAGGATATAATTAGCCTATGGGATCAAAGCCTAAAACCATGTGTGAAATTAACCCCACTCTGTGTTACTTTAAACTGCACTGATGCTAATACCACTAATACTAATGCCAAT------------------------------------------------------------------------------AGTACTAATAACAGTAGCTTGGGAACAATGGAGAAAGGAGAAATAAAAAACTGCTCTTTCAACATCACC---ACAAACCTAAGAGAT------AAGGTGCAGAAAGAATATGCACTTTTTTATAACCTTGATGTAGTGCCAATAAAGGGTGAGGAT---------------------------AATACTAGC---------------------------TATAGGTTGATAAGTTGTAATACCTCAGTCATTACACAGGCCTGTCCAAAGGTATCCTTTGAGCCAATTCCCATACATTATTGTACTCCAGCTGGTTTTGCGATTCTACAATGT---AATGATAAGAAATTCAATGGATCAGGACCATGTACAAATGTCAGCACAGTACAATGTACACATGGAATTAGGCCAGTAGTATCAACTCAACTGCTGTTAAATGGCAGTCTAGCAGAAAAA---GAGGTAGTAATTAGGTCTGAGAATTTCACAAATAATGCTAAAACCATAATAGTACAGCTAAATGAATCTGTAGTAATTAATTGTACAAGACCCAACAACAATACAAGAAAAAGTATACCTATAGGA------------CCAGGAAGA---GCATTTTATGCAACAGGAGAAATAATAGGAGATATAAGACAAGCACATTGTAACGTT------AGTACAAAAGCCTGGAAGGAAGCTTTACAACAGGTAGCTATAAAACTATCA---GAACAATTTGGG------------AATAAAACA---ATAGTCTTTAATCAA---------TCCTCAGGAGGAGACCCAGAAGTTGTAATGCACAGTTTTAATTGTAGAGGGGAATTTTTCTACTGTAATACAACAGGACTGTTTAATAATACTTGGGGGTTT---------AATAGTACTTGG---------------------GATGCTATTAATGTGCCAGAA------------------AATGACACA------------------------------ATCACACTCCCATGCAGAATAAAACAAATTGTAAACATGTGGCAGGAAGTAGGAAAAGCAATGTATGCCCCTCCCATCAGAGGACGACTTAATTGTTCATCAAATATTACAGGGCTGCTATTAACAAGAGATGGTGGT------------------AATACCACA---------------------AATAACACT---GAGGTCTTCAGACCTGGAGGAGGAGATATGAGAGACAATTGGAGA---AGTGAATTATATAAATATAAAGTAGTAAAAATTGAACCA---TTAGGAATAGCACCC---ACCAAGGCAAAGAGAAGAGTGGTGCAGAGAGAA---AAAAGAGCAGTG---GGA---ATAGGA---GCTTTG---TTCCTT---GGG---------TTCTTGGGA---GCAGCAGGAAGCACTATGGGCGCAGCGTCGATG---ACGCTGACGGTACAGGCCAGACTATTATTGTCTGGTATAGTGCAACAGCAGAACAATTTGCTGAGAGCTATTGAGGCGCAACAGCATCTGTTGCAACTCACAGTCTGGGGCATCAAGCAGCTCCAGGCAAGA---GTCCTGGCTGTGGAAAGATACCTAAGAGATCAACAGCTCCTGGGGATTTGGGGATGCTCTGGAAAACTCATTTGCACCACTACTGTGCCTTGGAATGTTAGTTGGAGT---------------------------AATAAATCCATGAATGACATTTGGAAT---AACATGACCTGGATGGAGTGGGAAAGAGAGATTGAC------AATTATACAAACATAATATACACCTTACTTGAAGAATCGCAGAACCAACAAGATAAGAATGAACAGGAATTATTGGAATTGGATAAATGGGCAAGTTTGTGGAATTGGTTTAGCATAACAAATTGGCTGTGGTACATAAAAATATTCATAATGATAGTAGGAGGCTTAATAGGTTTAAGAATAGTTTTTACTGTATTTTCTATAGTGAATAGAGTTAGGCAGGGATATTCACCATTATCGTTTCAGACCCGC---TTCCCAACCTCGAGGGGA------CTCGACAGGCCCGAAGGAATCGAAGAAGAAGGTGGAGACAGAGACAGAGACAGATCCAGGCCATTAGTGGATGGATTCTTAGCAATTATCTGGGTCGACCTGCGGAGCCTGTGCCTCTTCAGCTACCATCGCTTGAGAGACTTACTCTTGATTGTAGCGAGGATTGTGGAACTTCTGGGACGCAGG---------------GGGTGGGAAGCCCTCAAATATTGGTGG---AATCTCCTGCGGTATTGG---------------------------------------------------AGCCAGGAACTAAGGAATAGTGCTATTAGCTTGCTTAATGCCACAGCCATAGCAGTAGCTGAGGGAACAGATAGGGTGTTAGAAGTATTACAAAGA------------------GCTTTTAGAGCTGTTATACACATACCTAGAAGAATAAGACAGGGCTTAGAAAGGTTTTTGCTATAA

2.04013240.ADARC.GU330404 ATGAGAGTGAAGGGGATCATGAGGAATTATCAGCACTTA---------TGGAGATGGGGC------------------------ATGATGCTCCTTGGGATAATCATGATC------------TGTAGTGCTGCA---------GAACAATTGTGGGTCACAATCTATTATGGGGTACCTGTGTGGAAAGAAGCAACCACCACTCTATTTTGTGCATCAAATGCTAAAGCATATGATACAGAGGTACATAAT---GTCTGGGCCACACATGCCTGTGTACCCACAGACCCCAACCCACAAGAAGTAAGATTG---GAAAATGTGACAGAAAATTTTAACATGTGGAAAAATAATATGGTAGAACAGATGCATGAGGATATAATTAGCCTATGGGATCAAAGCCTAAAACCATGTGTGAAATTAACCCCACTCTGTGTTACTTTAAACTGCACTGATGCTAATACCACTAATACTAATGCCAAT------------------------------------------------------------------------------AGTACTAATAACAGTAGCTTGGGAACAATGGAGAAAGGAGAAATAAAAAACTGCTCTTTCAACATCACC---ACAAACCTAAGAGAT------AAGGTGCAGAAAGAATATGCACTTTTTTATAACCTTGATGTAGTGCCAATAAAGGGTGAGGAT---------------------------AATACTAGC---------------------------TATAGGTTGATAAGTTGTAATACCTCAGTCATTACACAGGCCTGTCCAAAGGTATCCTTTGAGCCAATTCCCATACATTATTGTACTCCAGCTGGTTTTGCGATTCTACAATGT---AATGATAAGAAATTCAATGGATCAGGACCATGTACAAATGTCAGCACAGTACAATGTACACATGGAATTAGGCCAGTAGTATCAACTCAACTGCTGTTAAATGGCAGTCTAGCAGAAAAA---GAGGTAGTAATTAGGTCTGAGAATTTCACAAATAATGCTAAAACCATAATAGTACAGCTAAATGAATCTGTAGTAATTAATTGTACAAGACCCAACAACAATACAAGAAAAAGTATACCTATAGGA------------CCAGGAAGA---GCATTTTATGCAACAGGAGAAATAATAGGAGATATAAGACAAGCACATTGTAACGTT------AGTACAAAAGCCTGGAAGGAAGCTTTACAACAGGTAGCTATAAAACTATCA---GAACAATTTGGG------------AATAAAACA---ATAGTCTTTAATCAA---------TCCTCAGGAGGAGACCCAGAAGTTGTAATGCACAGTTTTAATTGTAGAGGGGAATTTTTCTACTGTAATACAACAGGACTGTTTAATAATACTTGGGGGTTT---------AATAGTACTTGG---------------------GATGCTATTAATGTGCCAGAA------------------AATGACACA------------------------------ATCACACTCCCATGCAGAATAAAACAAATTGTAAACATGTGGCAGGAAGTAGGAAAAGCAATGTATGCCCCTCCCATCAGAGGACGACTTAATTGTTCATCAAATATTACAGGGCTGCTATTAACAAGAGATGGTGGT------------------AATACCACA---------------------AATAACACT---GAGGTCTTCAGACCTGGAGGAGGAGATATGAGAGACAATTGGAGA---AGTGAATTATATAAATATAAAGTAGTAAAAATTGAACCA---TTAGGAATAGCACCC---ACCAAGGCAAAGAGAAGAGTGGTGCAGAGAGAA---AAAAGAGCAGTG---GGA---ATAGGA---GCTTTG---TTCCTT---GGG---------TTCTTGGGA---GCAGCAGGAAGCACTATGGGCGCAGCGTCGATG---ACGCTGACGGTACAGGCCAGACTATTATTGTCTGGTATAGTGCAACAGCAGAACAATTTGCTGAGAGCTATTGAGGCGCAACAGCATCTGTTGCAACTCACAGTCTGGGGCATCAAGCAGCTCCAGGCAAGA---GTCCTGGCTGTGGAAAGATACCTAAGAGATCAACAGCTCCTGGGGATTTGGGGATGCTCTGGAAAACTCATTTGCACCACTACTGTGCCTTGGAATGTTAGTTGGAGT---------------------------AATAAATCCATGAATGACATTTGGAAT---AACATGACCTGGATGGAGTGGGAAAGAGAGATTGAC------AATTATACAAACATAATATACACCTTACTTGAAGAATCGCAGAACCAACAAGATAAGAATGAACAGGAATTATTGGAATTGGATAAATGGGCAAGTTTGTGGAATTGGTTTAGCATAACAAATTGGCTGTGGTACATAAAAATATTCATAATGATAGTAGGAGGCTTAATAGGTTTAAGAATAGTTTTTACTGTATTTTCTATAGTGAATAGAGTTAGGCAGGGATATTCACCATTATCGTTTCAGACCCGC---TTCCCAACCTCGAGGGGA------CTCGACAGGCCCGAAGGAATCGAAGAAGAAGGTGGAGACAGAGACAGAGACAGATCCAGGCCATTAGTGGATGGATTCTTAGCAATTATCTGGGTCGACCTGCGGAGCCTGTGCCTCTTCAGCTACCATCGCTTGAGAGACTTACTCTTGATTGTAGCGAGGATTGTGGAACTTCTGGGACGCAGG---------------GGGTGGGAAGCCCTCAAATATTGGTGG---AATCTCCTGCGGTATTGG---------------------------------------------------AGCCAGGAACTAAGGAATAGTGCTATTAGCTTGCTTAATGCCACAGCCATAGCAGTAGCTGAGGGAACAGATAGGGTGTTAGAAGTATTACAAAGA------------------GCTTTTAGAGCTGTTATACACATACCTAGAAGAATAAGACAGGGCTTAGAAAGGTTTTTGCTATAA

2.04013240.ADARC.GU330405 ATGAGAGTGAAGGGGATCATGAGGAATTATCAGCACTTA---------TGGAGATGGGGC------------------------ATGATGCTCCTTGGGATAATCATGATC------------TGTAGTGCTGCA---------GAACAATTGTGGGTCACAATCTATTATGGGGTACCTGTGTGGAAAGAAGCGACCACCACTCTATTTTGTGCATCAAATGCTAAAGCATATGATACAGAGGTACATAAT---GTCTGGGCCACACATGCCTGTGTACCCACAGACCCCAACCCACAAGAAGTAAGATTG---GAAAATGTGACAGAAAATTTTAACATGTGGAAAAATAATATGGTAGAACAGATGCATGAGGATATAATTAGCCTATGGGATCAAAGCCTAAAACCATGTGTGAAATTAACCCCACTCTGTGTTACTTTAAACTGCACTGATGCTAATACCACTAATACTAATGCCAAT------------------------------------------------------------------------------AGTACTAATAACAGTAGCTTGGGAACAATGGAGAAAGGAGAAATAAAAAACTGCTCTTTCAACATCACC---ACAAACCTAAGAGAT------AAGGTGCAGAAAGAATATGCACTTTTTTATAACCTTGATGTAGTGCCAATAAAGGGTGAGGAT---------------------------AATACTAGC---------------------------TATAGGTTGATAAGTTGTAATACCTCAGTCATTACACAGGCCTGTCCAAAGGTATCCTTTGAGCCAATTCCCATACATTATTGTACTCCAGCTGGTTTTGCGATTCTACAATGT---AATGATAAGAAATTCAATGGATCAGGACCATGTACAAATGTCAGCACAGTACAATGTACACATGGAATTAGGCCAGTAGTATCAACTCAACTGCTGTTAAATGGCAGTCTAGCAGAAAAA---GAGGTAGTAATTAGGTCTGAGAATTTCACAAATAATGCTAAAACCATAATAGTACAGCTAAATGAATCTGTAGTAATTAATTGTACAAGACCCAACAACAATACAAGAAAAAGTATACCTATAGGA------------CCAGGAAGA---GCATTTTATGCAACAGGAGAAATAATAGGAGATATAAGACAAGCACATTGTAACGTT------AGTACAAAAGCCTGGAAGGAAGCTTTACAACAGGTAGCTATAAAACTATCA---GAACAATTTGGG------------AATAAAACA---ATAGTCTTTAATCAA---------TCCTCAGGAGGAGACCCAGAAGTTGTAATGCACAGTTTTAATTGTAGAGGGGAATTTTTCTACTGTAATACAACAGGACTGTTTAATAATACTTGGGGGTTT---------AATAGTACTTGG---------------------GATGCTATTAATGTGCCAGAA------------------AATGACACA------------------------------ATCACACTCCCATGCAGAATAAAACAAATTGTAAACATGTGGCAGGAAGTAGGAAAAGCAATGTATGCCCCTCCCATCAGAGGACGACTTAATTGTTCATCAAATATTACAGGGCTGCTATTAACAAGAGATGGTGGT------------------AATACCACA---------------------AATAACACT---GAGGTCTTCAGACCTGGAGGAGGAGATATGAGAGACAATTGGAGA---AGTGAATTATATAAATATAAAGTAGTAAAAATTGAACCA---TTAGGAATAGCACCC---ACCAAGGCAAAGAGAAGAGTGGTGCAGAGAGAA---AAAAGAGCAGTG---GGA---ATAGGA---GCTTTG---TTCCTT---GGG---------TTCTTGGGA---GCAGCAGGAAGCACTATGGGCGCAGCGTCGATG---ACGCTGACGGTACAGGCCAGACTATTATTGTCTGGTATAGTGCAACAGCAGAACAATTTGCTGAGAGCTATTGAGGCGCAACAGCATCTGTTGCAACTCACAGTCTGGGGCATCAAGCAGCTCCAGGCAAGA---GTCCTGGCTGTGGAAAGATACCTAAGAGATCAACAGCTCCTGGGGATTTGGGGATGCTCTGGAAAACTCATTTGCACCACTACTGTGCCTTGGAATGTTAGTTGGAGT---------------------------AATAAATCCATGAATGACATTTGGAAT---AACATGACCTGGATGGAGTGGGAAAGAGAGATTGAC------AATTATACAAACATAATATACACCTTACTTGAAGAATCGCAGAACCAACAAGATAAGAATGAACAGGAATTATTGGAATTGGATAAATGGGCAAGTTTGTGGAATTGGTTTAGCATAACAAATTGGCTGTGGTACATAAAAATATTCATAATGATAGTAGGAGGCTTAATAGGTTTAAGAATAGTTTTTACTGTATTTTCTATAGTGAATAGAGTTAGGCAGGGATATTCACCATTATCGTTTCAGACCCGC---TTCCCAACCTCGAGGGGA------CTCGACAGGCCCGAAGGAATCGAAGAAGAAGGTGGAGACAGAGACAGAGACAGATCCAGGCCATTAGTGGATGGATTCTTAGCAATTATCTGGGTCGACCTGCGGAGCCTGTGCCTCTTCAGCTACCATCGCTTGAGAGACTTACTCTTGATTGTAGCGAGGATTGTGGAACTTCTGGGACGCAGG---------------GGGTGGGAAGCCCTCAAATATTGGTGG---AATCTCCTGCGGTATTGG---------------------------------------------------AGCCAGGAACTAAGGAATAGTGCTATTAGCTTGCTTAATGCCACAGCCATAGCAGTAGTTGAGGGAACAGATAGGGTGGTAGAAGTATTACAAAGA------------------GCTTTTAGAGCTGTTATACACATACCTAGAAGAATAAGACAGGGCTTAGAAAGGTTTTTGCTATAA

2.04013240.ADARC.GU330406 ATGAGAGTGAAGGGGATCATGAGGAATTATCAGCACTTA---------TGGAGATGGGGC------------------------ATGATGCTCCTTGGGATAATCATGATC------------TGTAGTGCTGCA---------GAACAATTGTGGGTCACAATCTATTATGGGGTACCTGTGTGGAAAGAAGCAACCACCACTCTATTTTGTGCATCAAATGCTAAAGCATATGATACAGAGGTACATAAT---GTCTGGGCCACACATGCCTGTGTACCCACAGACCCCAACCCACAAGAAGTAAGATTG---GAAAATGTGACAGAAAATTTTAACATGTGGAAAAATAATATGGTAGAACAGATGCATGAGGATATAATTAGCCTATGGGATCAAAGCCTAAAACCATGTGTGAAATTAACCCCACTCTGTGTTACTTTAAACTGCACTGATGCTAATACCACTAATACTAATGCCAAT------------------------------------------------------------------------------AGTACTAATAACAGTAGCTTGGGAACAATGGAGAAAGGAGAAATAAAAAACTGCTCTTTCAACATCACC---ACAAACCTAAGAGAT------AAGGTGCAGAAAGAATATGCACTTTTTTATAACCTTGATGTAGTGCCAATAAAGGGTGAGGAT---------------------------AATACTAGC---------------------------TATAGGTTGATAAGTTGTAATACCTCAGTCATTACACAGGCCTGTCCAAAGGTATCCTTTGAGCCAATTCCCATACATTATTGTACTCCAGCTGGTTTTGCGATTCTACAATGT---AATGATAAGAAATTCAATGGATCAGGACCATGTACAAATGTCAGCACAGTACAATGTACACATGGAATTAGGCCAGTAGTATCAACTCAACTGCTGTTAAATGGCAGTCTAGCAGAAAAA---GAGGTAGTAATTAGGTCTGAGAATTTCACAAATAATGCTAAAACCATAATAGTACAGCTAAATGAATCTGTAGTAATTAATTGTACAAGACCCAACAACAATACAAGAAAAAGTATACCTATAGGA------------CCAGGAAGA---GCATTTTATGCAACAGGAGAAATAATAGGAGATATAAGACAAGCACATTGTAACGTT------AGTACAAAAGCCTGGAAGGAAGCTTTACAACAGGTAGCTATAAAACTATCA---GAACAATTTGGG------------AATAAAACA---ATAGTCTTTAATCAA---------TCCTCAGGAGGAGACCCAGAAGTTGTAATGCACAGTTTTAATTGTAGAGGGGAATTTTTCTACTGTAATACAACAGGACTGTTTAATAATACTTGGGGGTTT---------AATAGTACTTGG---------------------GATGCTATTAATGTGCCAGAA------------------AATGACACA------------------------------ATCACACTCCCATGCAGAATAAAACAAATTGTAAACATGTGGCAGGAAGTAGGAAAAGCAATGTATGCCCCTCCCATCAGAGGACGACTTAATTGTTCATCAAATATTACAGGGCTGCTATTAACAAGAGATGGTGGT------------------AATACCACA---------------------AATAACACT---GAGGTCTTCAGACCTGGAGGAGGAGATATGAGAGACAATTGGAGA---AGTGAATTATATAAATATAAAGTAGTAAAAATTGAACCA---TTAGGAATAGCACCC---ACCAAGGCAAAGAGAAGAGTGGTGCAGAGAGAA---AAAAGAGCAGTG---GGA---ATAGGA---GCTTTG---TTCCTT---GGG---------TTCTTGGGA---GCAGCAGGAAGCACTATGGGCGCAGCGTCGATG---ACGCTGACGGTACAGGCCAGACTATTATTGTCTGGTATAGTGCAACAGCAGAACAATTTGCTGAGAGCTATTGAGGCGCAACAGCATCTGTTGCAACTCACAGTCTGGGGCATCAAGCAGCTCCAGGCAAGA---GTCCTGGCTGTGGAAAGATACCTAAGAGATCAACAGCTCCTGGGGATTTGGGGATGCTCTGGAAAACTCATTTGCACCACTACTGTGCCTTGGAATGTTAGTTGGAGT---------------------------AATAAATCCATGAATGACATTTGGAAT---AACATGACCTGGATGGAGTGGGAAAGAGAGATTGAC------AATTATACAAACATAATATACACCTTACTTGAAGAATCGCAGAACCAACAAGATAAGAATGAACAGGAATTATTGGAATTGGATAAATGGGCAAGTTTGTGGAATTGGTTTAGCATAACAAATTGGCTGTGGTACATAAAAATATTCATAATGATAGTAGGAGGCTTAATAGGTTTAAGAATAGTTTTTACTGTATTTTCTATAGTGAATAGAGTTAGGCAGGGATATTCACCATTATCGTTTCAGACCCGC---TTCCCAACCTCGAGGGGA------CTCGACAGGCCCGAAGGAATCGAAGAAGAAGGTGGAGACAGAGACAGAGACAGATCCAGGCCATTAGTGGATGGATTCTTAGCAATTATCTGGGTCGACCTGCGGAGCCTGTGCCTCTTCAGCTACCATCGCTTGAGAGACTTACTCTTGATTGTAGCGAGGATTGTGGAACTTCTGGGACGCAGG---------------GGGTGGGAAGCCCTCAAATATTGGTGG---AATCTCCTGCGGTATTGG---------------------------------------------------AGCCAGGAACTAAGGAATAGTGCTATTAGCTTGCTTAATGCCACAGCCATAGCAGTAGCTGAGGGAACAGATAGGGTGTTAGAAGTATTACAAAGA------------------GCTTTTAGAGCTGTTATACACATACCTAGAAGAATAAGACAGGGCTTAGAAAGGTTTTTGCTATAA

2.04013240.ADARC.GU330407 ATGAGAGTGAAGGGGATCATGAGGAATTATCAGCACTTA---------TGGAGATGGGGC------------------------ATGATGCTCCTTGGGATAATCATGATC------------TGTAGTGCTGCA---------GAACAATTGTGGGTCACAATCTATTATGGGGTACCTGTGTGGAAAGAAGCAACCACCACTCTATTTTGTGCATCAAATGCTAAAGCATATGATACAGAGGTACATAAT---GTCTGGGCCACACATGCCTGTGTACCCACAGACCCCAACCCACAAGAAGTAAGATTG---GAAAATGTGACAGAAAATTTTAACATGTGGAAAAATAATATGGTAGAACAGATGCATGAGGATATAATTAGCCTATGGGATCAAAGCCTAAAACCATGTGTGAAATTAACCCCACTCTGTGTTACTTTAAACTGCACTGATGCTAATACCACTAATACTAATGCCAAT------------------------------------------------------------------------------AGTACTAATAACAGTAGCTTGGGAACAATGGAGAAAGGAGAAATAAAAAACTGCTCTTTCAACATCACC---ACAAACCTAAGAGAT------AAGGTGCAGAAAGAATATGCACTTTTTTATAACCTTGATGTAGTGCCAATAAAGGGTGAGGAT---------------------------AATACTAGC---------------------------TATAGGTTGATAAGTTGTAATACCTCAGTCATTACACAGGCCTGTCCAAAGGTATCCTTTGAGCCAATTCCCATACATTATTGTACTCCAGCTGGTTTTGCGATTCTACAATGT---AATGATAAGAAATTCAATGGATCAGGACCATGTACAAATGTCAGCACAGTACAATGTACACATGGAATTAGGCCAGTAGTATCAACTCAACTGCTGTTAAATGGCAGTCTAGCAGAAAAA---GAGGTAGTAATTAGGTCTGAGAATTTCACAAATAATGCTAAAACCATAATAGTACAGCTAAATGAATCTGTAGTAATTAATTGTACAAGACCCAACAACAATACAAGAAAAAGTATACCTATAGGA------------CCAGGAAGA---GCATTTTATGCAACAGGAGAAATAATAGGAGATATAAGACAAGCACATTGTAACGTT------AGTACAAAAGCCTGGAAGGAAGCTTTACAACAGGTAGCTATAAAACTATCA---GAACAATTTGGG------------AATAAAACA---ATAGTCTTTAATCAA---------TCCTCAGGAGGAGACCCAGAAGTTGTAATGCACAGTTTTAATTGTAGAGGGGAATTTTTCTACTGTAATACAACAGGACTGTTTAATAATACTTGGGGGTTT---------AATAGTACTTGG---------------------GATGCTATTAATGTGCCAGAA------------------AATGACACA------------------------------ATCACACTCCCATGCAGAATAAAACAAATTGTAAACATGTGGCAGGAAGTAGGAAAAGCAATGTATGCCCCTCCCATCAGAGGACAAATTAATTGTTCATCAAATATTACAGGGCTGCTATTAACAAGAGATGGTGGT------------------AATACCACA---------------------AATAACACT---GAGGTCTTCAGACCTGGAGGAGGAGATATGAGAGACAATTGGAGA---AGTGAATTATATAAATATAAAGTAGTAAAAATTGAACCA---TTAGGAATAGCACCC---ACCAAGGCAAAGAGAAGAGTGGTGCAGAGAGAA---AAAAGAGCAGTG---GGA---ATAGGA---GCTTTG---TTCCTT---GGG---------TTCTTGGGA---GCAGCAGGAAGCACTATGGGCGCAGCGTCGATG---ACGCTGACGGTACAGGCCAGACTATTATTGTCTGGTATAGTGCAACAGCAGAACAATTTGCTGAGAGCTATTGAGGCGCAACAGCATCTGTTGCAACTCACAGTCTGGGGCATCAAGCAGCTCCAGGCAAGA---GTCCTGGCTGTGGAAAGATACCTAAGGGATCAACAGCTCCTGGGGATTTGGGGATGCTCTGGAAAACTCATTTGCACCACTGCTGTGCCTTGGAATGTTAGTTGGAGT---------------------------AATAAATCCATGAATGACATTTGGAAT---AACATGACCTGGATGGAGTGGGAAAGAGAGATTGAC------AATTATACAAACATAATATACACCTTACTTGAAGAATCGCAGAACCAACAAGATAAGAATGAACAGGAATTATTGGAATTGGATAAATGGGCAAGTTTGTGGAATTGGTTTAGCATAACAAATTGGCTGTGGTACATAAAAATATTCATAATGATAGTAGGAGGCTTAATAGGTTTAAGAATAGTTTTTACTGTATTTTCTATAGTGAATAGAGTTAGGCAGGGATATTCACCATTATCGTTTCAGACCCGC---TTCCCAACCTCGAGGGGA------CTCGACAGGCCCGAAGGAATCGAAGAAGAAGGTGGAGACAGAGACAGAGACAGATCCAGGCCATTAGTGGATGGATTCTTAGCAATTATCTGGGTCGACCTGCGGAGCCTGTGCCTCTTCAGCTACCATCGCTTGAGAGACTTACTCTTGATTGTAGCGAGGATTGTGGAACTTCTGGGACGCAGG---------------GGGTGGGAAGCCCTCAAATATTGGTGG---AATCTCCTGCGGTATTGG---------------------------------------------------AGCCAGGAACTAAGGAATAGTGCTATTAGCTTGCTTAATGCCACAGCCATAGCAGTAGCTGAGGGAACAGATAGGGTGTTAGAAGTATTACAAAGA------------------GCTTTTAGAGCTGTTATACACATACCTAGAAGAATAAGACAGGGCTTAGAAAGGTTTTTGCTATAA

2.04013240.ADARC.GU330408 ATGAGAGTGAAGGGGATCATGAGGAATTATCAGCACTTA---------TGGAGATGGGGC------------------------ATGATGATCCTTGGGATAATCATGATC------------TGTAGTGCTGCA---------GAACAATTGTGGGTCACAATCTATTATGGGGTACCTGTGTGGAAAAAAGCAACCACCACTCTATTTTGTGCATCAAATGCTAAAGCATATGATACAGAGGTACATAAT---GTCTGGGCCACACATGCCTGTGTACCCACAGACCCCAACCCACAAGAAGTAAGATTG---GAAAATGTGACAGAAAATTTTAACATGTGGAAAAATAATATGGTAGAACAGATGCATGAGGATATAATTAGCCTATGGGATCAAAGCCTAAAACCATGTGTGAAATTAACCCCACTCTGTGTTACTTTAAACTGCACTGATGCTAATACCACTAATACTAATGCCAAT------------------------------------------------------------------------------AGTACTAATAACAGTAGCTTGGGAACAATGGAGAAAGGAGAAATAAAAAACTGCTCTTTCAACATCACC---ACAAACCTAAGAGAT------AAGGTGCAGAAAGAATATGCACTTTTTTATAACCTTGATGTAGTGCCAATAAAGGGTGAGGAT---------------------------AATACTAGC---------------------------TATAGGTTGATAAGTTGTAATACCTCAGTCATTACACAGGCCTGTCCAAAGGTATCCTTTGAGCCAATTCCCATACATTATTGTACTCCAGCTGGTTTTGCGATTCTACAATGT---AATGATAAGAAATTCAATGGATCAGGACCATGTACAAATGTCAGCACAGTACAATGTACACATGGAATTAGGCCAGTAGTATCAACTCAACTGCTGTTAAATGGCAGTCTAGCAGAAAAA---GAGGTAGTAATTAGGTCTGAGAATTTCACAAATAATGCTAAAACCATAATAGTACAGCTAAATGAATCTGTAGTAATTAATTGTACAAGACCCAACAACAATACAAGAAAAAGTATACCTATAGGA------------CCAGGAAGA---GCATTTTATGCAACAGGAGAAATAATAGGAGATATAAGACAAGCACATTGTAACGTT------AGTACAAAAGCCTGGAAGGAAGCTTTACAACAGGTAGCTATAAAACTATCA---GAACAATTTGGG------------AATAAAACA---ATAGTCTTTAATCAA---------TCCTCAGGAGGAGACCCAGAAGTTGTAATGCACAGTTTTAATTGTAGAGGGGAATTTTTCTACTGTAATACAACAGGACTGTTTAATAATACTTGGGGGTTT---------AATAGTACTTGG---------------------GATGCTATTAATGTGCCAGAA------------------AATGACACA------------------------------ATCACACTCCCATGCAGAATAAAACAAATTGTAAACATGTGGCAGGAAGTAGGAAAAGCAATGTATGCCCCTCCCATCAGAGGACGACTTAATTGTTCATCAAATATTACAGGGCTGCTATTAACAAGAGATGGTGGT------------------AATACCACA---------------------AATAACACT---GAGGTCTTCAGACCTGGAGGAGGAGATATGAGAGACAATTGGAGA---AGTGAATTATATAAATATAAAGTAGTAAAAATTGAACCA---TTAGGAATAGCACCC---ACCAAGGCAAAGAGAAGAGTGGTGCAGAGAGAA---AAAAGAGCAGTG---GGA---ATAGGA---GCTTTG---TTCCTT---GGG---------TTCTTGGGA---GCAGCAGGAAGCACTATGGGCGCAGCGTCGATG---ACGCTGACGGTACAGGCCAGACTATTATTGTCTGGTATAGTGCAACAGCAGAACAATTTGCTGAGAGCTATTGAGGCGCAACAGCATCTGTTGCAACTCACAGTCTGGGGCATCAAGCAGCTCCAGGCAAGA---GTCCTGGCTGTGGAAAGATACCTAAGAGATCAACAGCTCCTGGGGATTTGGGGATGCTCTGGAAAACTCATTTGCACCACTACTGTGCCTTGGAATGTTAGTTGGAGT---------------------------AATAAATCCATGAATGACATTTGGAAT---AACATGACCTGGATGGAGTGGGAAAGAGAGATTGAC------AATTATACAAACATAATATACACCTTACTTGAAGAATCGCAGAACCAACAAGATAAGAATGAACAGGAATTATTGGAATTGGATAAATGGGCAAGTTTGTGGAATTGGTTTAGCATAACAAATTGGCTGTGGTACATAAAAATATTCATAATGATAGTAGGAGGCTTAATAGGTTTAAGAATAGTTTTTACTGTATTTTCTATAGTGAATAGAGTTAGGCAGGGATATTCACCATTATCGTTTCAGACCCGC---TTCCCAACCTCGAGGGGA------CTCGACAGGCCCGAAGGAATCGAAGAAGAAGGTGGAGACAGAGACAGAGACAGATCCAGGCCATTAGTGGATGGGTTCTTAGCAATTATCTGGGTCGACCTGCGGAGCCTGTGCCTCTTCAGCTACCATCGCTTGAGAGACTTACTCTTGATTGTAGCGAGGATTGTGGAACTTCTGGGACGCAGG---------------GGGTGGGAAGCCCTCAAATATTGGTGG---AATCTCCTGCGGTATTGG---------------------------------------------------AGCCAGGAACTAAGGAATAGTGCTATTAGCTTGCTTAATGCCACAGCCATAGCAGTAGCTGAGGGAACAGATAGGGTGGTAGAAGTATTACAAAGA------------------GCTTTTAGAGCTGTTATACGCATACCTAGAAGAATAAGACAGGGCTTAGAAAGGTTTTTGCTATAA

2.04013240.ADARC.GU330409 ATGAGAGTGAAGGGGATCATGAGGAATTATCAGCACTTA---------TGGAGATGGGGC------------------------ATGATGCTCCTTGGGATAATCATGATC------------TGTAGTGCTGCA---------GAACAATTGTGGGTCACAATCTATTATGGGGTACCTGTGTGGAAAGAAGCAACCACCACTCTATTTTGTGCATCAAATGCTAAAGCATATGATACAGAGGTACATAAT---GTCTGGGCCACACATGCCTGTGTACCCACAGACCCCAACCCACAAGAAGTAAGATTG---GAAAATGTGACAGAAAATTTTAACATGTGGAAAAATAATATGGTAGAACAGATGCATGAGGATATAATTAGCCTATGGGATCAAAGCCTAAAACCATGTGTGAAATTAACCCCACTCTGTGTTACTTTAAACTGCACTGATGCTAATACCACTAATACTAATGCCAAT------------------------------------------------------------------------------AGTACTAATAACAGTAGCTTGGGAACAATGGAGAAAGGAGAAATAAAAAACTGCTCTTTCAACATCACC---ACAAACCTAAGAGAT------AAGGTGCAGAAAGAATATGCACTTTTTTATAACCTTGATGTAGTGCCAATAAAGGGTGAGGAT---------------------------AATACTAGC---------------------------TATAGGTTGATAAGTTGTAATACCTCAGTCATTACACAGGCCTGTCCAAAGGTATCCTTTGAGCCAATTCCCATACATTATTGTACTCCAGCTGGTTTTGCGATTCTACAATGT---AATGATAAGAAATTCAATGGATCAGGACCATGTACAAATGTCAGCACAGTACAATGTACACATGGAATTAGGCCAGTAGTATCAACTCAACTGCTGTTAAATGGCAGTCTAGCAGAAAAA---GAGGTAGTAATTAGGTCTGAGAATTTCACAAATAATGCTAAAACCATAATAGTACAGCTAAATGAATCTGTAGTAATTAATTGTACAAGACCCAACAACAATACAAGAAAAAGTATACCTATAGGA------------CCAGGAAGA---GCATTTTATGCAACAGGAGAAATAATAGGAGATATAAGACAAGCACATTGTAACGTT------AGTACAAAAGCCTGGAAGGAAGCTTTACAACAGGTAGCTATAAAACTATCA---GAACAATTTGGG------------AATAAAACA---ATAGTCTTTAATCAA---------TCCTCAGGAGGAGACCCAGAAGTTGTAATGCACAGTTTTAATTGTAGAGGGGAATTTTTCTACTGTAATACAACAGGACTGTTTAATAATACTTGGGGGTTT---------AATAGTACTTGG---------------------GATGCTATTAATGTGCCAGAA------------------AATGACACA------------------------------ATCACACTCCCATGCAGAATAAAACAAATTGTAAACATGTGGCAGGAAGTAGGAAAAGCAATGTATGCCCCTCCCATCAGAGGACGACTTAATTGTTCATCAAATATTACAGGGCTGCTATTAACAAGAGATGGTGGT------------------AATACCACA---------------------AATAACACT---GAGGTCTTCAGACCTGGAGGAGGAGATATGAGAGACAATTGGAGA---AGTGAATTATATAAATATAAAGTAGTAAAAATTGAACCA---TTAGGAATAGCACCC---ACCAAGGCAAAGAGAAGAGTGGTGCAGAGAGAA---AAAAGAGCAGTG---GGA---ATAGGA---GCTTTG---TTCCTT---GGG---------TTCTTGGGA---GCAGCAGGAAGCACTATGGGCGCAGCGTCGATG---ACGCTGACGGTACAGGCCAGACTATTATTGTCTGGTATAGTGCAACAGCAGAACAATTTGCTGAGAGCTATTGAGGCGCAACAGCATCTGTTGCAACTCACAGTCTGGGGCATCAAGCAGCTCCAGGCAAGA---GTCCTGGCTGTGGAAAGATACCTAAGAGATCAACAGCTCCTGGGGATTTGGGGATGCTCTGGAAAACTCATTTGCACCACTACTGTGCCTTGGAATGTTAGTTGGAGT---------------------------AATAAATCCATGAATGACATTTGGAAT---AACATGACCTGGATGGAGTGGGAAAGAGAGATTGAC------AATTATACAAACATAATATACACCTTACTTGAAGAATCGCAGAACCAACAAGATAAGAATGAACAGGAATTATTGGAATTGGATAAATGGGCAAGTTTGTGGAATTGGTTTAGCATAACAAATTGGCTGTGGTACATAAAAATATTCATAATGATAGTAGGAGGCTTAATAGGTTTAAGAATAGTTTTTACTGTATTTTCTATAGTGAATAGAGTTAGGCAGGGATATTCACCATTATCGTTTCAGACCCGC---TTCCCAACCTCGAGGGGA------CTCGACAGGCCCGAAGGAATCGAAGAAGAAGGTGGAGACAGAGACAGAGACAGATCCAGGCCATTAGTGGATGGATTCTTAGCAATTATCTGGGTCGACCTGCGGAGCCTGTGCCTCTTCAGCTACCATCGCTTGAGAGACTTACTCTTGATTGTAGCGAGGATTGTGGAACTTCTGGGACGCAGG---------------GGGTGGGAAGCCCTCAAATATTGGTGG---AATCTCCTGCGGTATTGG---------------------------------------------------AGCCAGGAACTAAGGAATAGTGCTATTAGCTTGCTTAATGCCACAGCCATAGCAGTAGCTGAGGGAACAGATAGGGTGTTAGAAGTATTACAAAGA------------------GCTTTTAGAGCTGTTATACACATACCTAGAAGAATAAGACAGGGCTTAGAAAGGTTTTTGCTATAA

2.04013240.ADARC.GU330410 ATGAGAGTGAAGGGGATCATGAGGAATTATCAGCACTTA---------TGGAGATGGGGC------------------------ATGATGCTCCTTGGGATAATCATGATC------------TGTAGTGCTGCA---------GAACAATTGTGGGTCACAATCTATTATGGGGTACCTGTGTGGAAAGAAGCAACCACCACTCTATTTTGTGCATCAAATGCTAAAGCATATGATACAGAGGTACATAAT---GTCTGGGCCACACATGCCTGTGTACCCACAGACCCCAACCCACAAGAAGTAAGATTG---GAAAATGTGACAGAAAATTTTAACATGTGGAAAAATAATATGGTAGAACAGATGCATGAGGATATAATTAGCCTATGGGATCAAAGCCTAAAACCATGTGTGAAATTAACCCCACTCTGTGTTACTTTAAACTGCACTGATGCTAATACCACTAATACTAATGCCAAT------------------------------------------------------------------------------AGTACTAATAACAGTAGCTTGGGAACAATGGAGAAAGGAGAAATAAAAAACTGCTCTTTCAACATCACC---ACAAACCTAAGAGAT------AAGGTGCAGAAAGAATATGCACTTTTTTATAACCTTGATGTAGTGCCAATAAAGGGTGAGGAT---------------------------AATACTAGC---------------------------TATAGGTTGATAAGTTGTAATACCTCAGTCATTACACAGGCCTGTCCAAAGGTATCCTTTGAGCCAATTCCCATACATTATTGTACTCCAGCTGGTTTTGCGATTCTACAATGT---AATGATAAGAAATTCAATGGATCAGGACCATGTACAAATGTCAGCACAGTACAATGTACACATGGAATTAGGCCAGTAGTATCAACTCAACTGCTGTTAAATGGCAGTCTAGCAGAAAAA---GAGGTAGTAATTAGGTCTGAGAATTTCACAAATAATGCTAAAACCATAATAGTACAGCTAAATGAATCTGTAGTAATTAATTGTACAAGACCCAACAACAATACAAGAAAAAGTATACCTATAGGA------------CCAGGAAGA---GCATTTTATGCAACAGGAGAAATAATAGGAGATATAAGACAAGCACATTGTAACGTT------AGTACAAAAGCCTGGAAGGAAGCTTTACAACAGGTAGCTATAAAACTATCA---GAACAATTTGGG------------AATAAAACA---ATAGTCTTTAATCAA---------TCCTCAGGAGGAGACCCAGAAGTTGTAATGCACAGTTTTAATTGTAGAGGGGAATTTTTCTACTGTAATACAACAGGACTGTTTAATAATACTTGGGGGTTT---------AATAGTACTTGG---------------------GATGCTATTAATGTGCCAGAA------------------AATGACACA------------------------------ATCACACTCCCATGCAGAATAAAACAAATTGTAAACATGTGGCAGGAAGTAGGAAAAGCAATGTATGCCCCTCCCATCAGAGGACGACTTAATTGTTCATCAAATATTACAGGGCTGCTATTAACAAGAGATGGTGGT------------------AATACCACA---------------------AATAACACT---GAGGTCTTCAGACCTGGAGGAGGAGATATGAGAGACAATTGGAGA---AGTGAATTATATAAATATAAAGTAGTAAAAATTGAACCA---TTAGGAATAGCACCC---ACCAAGGCAAAGAGAAGAGTGGTGCAGAGAGAA---AAAAGAGCAGTG---GGA---ATAGGA---GCTTTG---TTCCTT---GGG---------TTCTTGGGA---GCAGCAGGAAGCACTATGGGCGCAGCGTCGATG---ACGCTGACGGTACAGGCCAGACTATTATTGTCTGGTATAGTGCAACAGCAGAACAATTTGCTGAGAGCTATTGAGGCGCAACAGCATCTGTTGCAACTCACAGTCTGGGGCATCAAGCAGCTCCAGGCAAGA---GTCCTGGCTGTGGAAAGATACCTAAGAGATCAACAGCTCCTGGGGATTTGGGGATGCTCTGGAAAACTCATTTGCACCACTACTGTGCCTTGGAATGTTAGTTGGAGT---------------------------AATAAATCCATGAATGACATTTGGAAT---AACATGACCTGGATGGAGTGGGAAAGAGAGATTGAC------AATTATACAAACATAATATACACCTTACTTGAAGAATCGCAGAACCAACAAGATAAGAATGAACAGGAATTATTGGAATTGGATAAATGGGCAAGTTTGTGGAATTGGTTTAGCATAACAAATTGGCTGTGGTACATAAAAATATTCATAATGATAGTAGGAGGCTTAATAGGTTTAAGAATAGTTTTTACTGTATTTTCTATAGTGAATAGAGTTAGGCAGGGATATTCACCATTATCGTTTCAGACCCGC---TTCCCAACCTCGAGGGGA------CTCGACAGGCCCGAAGGAATCGAAGAAGAAGGTGGAGACAGAGACAGAGACAGATCCAGGCCATTAGTGGATGGATTCTTAGCAATTATCTGGGTCGACCTGCGGAGCCTGTGCCTCTTCAGCTACCATCGCTTGAGAGACTTACTCTTGATTGTAGCGAGGATTGTGGAACTTCTGGGACGCAGG---------------GGGTGGGAAGCCCTCAAATATTGGTGG---AATCTCCTGCGGTATTGG---------------------------------------------------AGCCAGGAACTAAGGAATAGTGCTATTAGCTTGCTTAATGCCACAGCCATAGCAGTAGCTGAGGGAACAGATAGGGTGTTAGAAGTATTACAAAGA------------------GCTTTTAGAGCTGTTATACACATACCTAGAAGAATAAGACAGGGCTTAGAAAGGTTTTTGCTATAA

2.04013240.ADARC.GU330411 ATGAGAGTGAAGGGGATCATGAGGAATTATCAGCACTTA---------TGGAGATGGGGC------------------------ATGATGCTCCTTGGGATAATCATGATC------------TGTAGTGCTGCA---------GAACAATTGTGGGTCACAATCTATTATGGGGTACCTGTGTGGAAAGAAGCAACCACCACTCTATTTTGTGCATCAAATGCTAAAGCATATGATACAGAGGTACATAAT---GTCTGGGCCACACATGCCTGTGTACCCACAGACCCCAACCCACAAGAAGTAAGATTG---GAAAATGTGACAGAAAATTTTAACATGTGGAAAAATAATATGGTAGAACAGATGCATGAGGATATAATTAGCCTATGGGATCAAAGCCTAAAACCATGTGTGAAATTAACCCCACTCTGTGTTACTTTAAACTGCACTGATGCTAATACCACTAATACTAATGCCAAT------------------------------------------------------------------------------AGTACTAATAACAGTAGCTTGGGAACAATGGAGAAAGGAGAAATAAAAAACTGCTCTTTCAACATCACC---ACAAACCTAAGAGAT------AAGGTGCAGAAAGAATATGCACTTTTTTATAACCTTGATGTAGTGCCAATAAAGGGTGAGGAT---------------------------AATACTAGC---------------------------TATAGGTTGATAAGTTGTAATACCTCAGTCATTACACAGGCCTGTCCAAAGGTATCCTTTGAGCCAATTCCCATACATTATTGTACTCCAGCTGGTTTTGCGATTCTACAATGT---AATGATAAGAAATTCAATGGATCAGGACCATGTACAAATGTCAGCACAGTACAATGTACACATGGAATTAGGCCAGTAGTATCAACTCAACTGCTGTTAAATGGCAGTCTAGCAGGAAAA---GAGGTAGTAATTAGGTCTGAGAATTTCACAAATAATGCTAAAACCATAATAGTACAGCTAAATGAATCTGTAGTAATTAATTGTACAAGACCCAACAACAATACAAGAAAAAGTATACCTATAGGA------------CCAGGAAGA---GCATTTTATGCAACAGGAGAAATAATAGGAGATATAAGACAAGCACATTGTAACGTT------AGTACAAAAGCCTGGAAGGAAGCTTTACAACAGGTAGCTATAAAACTATCA---GAACAATTTGGG------------AATAAAACA---ATAGTCTTTAATCAA---------TCCTCAGGAGGAGACCCAGAAGTTGTAATGCACAGTTTTAATTGTAGAGGGGAATTTTTCTACTGTAATACAACAGGACTGTTTAATAATACTTGGGGGTTT---------AATAGTACTTGG---------------------GATGCTATTAATGTGCCAGAA------------------AATGACACA------------------------------ATCACACTCCCATGCAGAATAAAACAAATTGTAAACATGTGGCAGGAAGTAGGAAAAGCAATGTATGCCCCTCCCATCAGAGGACGACTTAATTGTTCATCAAATATTACAGGGCTGCTATTAACAAGAGATGGTGGT------------------AATACCACA---------------------AATAACACT---GAGGTCTTCAGACCTGGAGGAGGAGATATGAGAGACAATTGGAGA---AGTGAATTATATAAATATAAAGTAGTAAAAATTGAACCA---TTAGGAATAGCACCC---ACCAAGGCAAAGAGAAGAGTGGTGCAGAGAGAA---AAAAGAGCAGTG---GGA---ATAGGA---GCTTTG---TTCCTT---GGG---------TTCTTGGGA---GCAGCAGGAAGCACTATGGGCGCAGCGTCGATG---ACGCTGACGGTACAGGCCAGACTATTATTGTCTGGTATAGTGCAACAGCAGAACAATTTGCTGAGAGCTATTGAGGCGCAACAGCATCTGTTGCAACTCACAGTCTGGGGCATCAAGCAGCTCCAGGCAAGA---GTCCTGGCTGTGGAAAGATACCTAAGAGATCAACAGCTCCTGGGGATTTGGGGATGCTCTGGAAAACTCATTTGCACCACTACTGTGCCTTGGAATGTTAGTTGGAGT---------------------------AATAAATCCATGAATGACATTTGGAAT---AACATGACCTGGATGGAGTGGGAAAGAGAGATTGAC------AATTATACAAACATAATATACACCTTACTTGAAGAATCGCAGAACCAACAAGATAAGAATGAACAGGAATTATTGGAATTGGATAAATGGGCAAGTTTGTGGAATTGGTTTAGCATAACAAATTGGCTGTGGTACATAAAAATATTCATAATGATAGTAGGAGGCTTAATAGGTTTAAGAATAGTTTTTACTGTATTTTCTATAGTGAATAGAGTTAGGCAGGGATATTCACCATTATCGTTTCAGACCCGC---TTCCCAACCTCGAGGGGA------CTCGACAGGCCCGAAGGAATCGAAGAAGAAGGTGGAGACAGAGACAGAGACAGATCCAGGCCATTAGTGGATGGATTCTTAGCAATTATCTGGGTCGACCTGCGGAGCCTGTGCCTCTTCAGCTACCATCGCTTGAGAGACTTACTCTTGATTGTAGCGAGGATTGTGGAACTTCTGGGACGCAGG---------------GGGTGGGAAGCCCTCAAATATTGGTGG---AATCTCCTGCGGTATTGG---------------------------------------------------AGCCAGGAACTAAGGAATAGTGCTATTAGCTTGCTTAATGCCACAGCCATAGCAGTAGCTGAGGGAACAGATAGGGTGGTAGAAGTATTACAAAGA------------------GCTTTTAGAGCTGTTATACACATACCTAGAAGAATAAGACAGGGCTTAGAAAGGTTTTTGCTATAA

2.04013240.ADARC.GU330412 ATGAGAGTGAAGGGGATCATGAGGAATTATCAGCACTTA---------TGGAGATGGGGC------------------------ATGATGCTCCTTGGGATAATCATGATC------------TGTAGTGCTGCA---------GAACAATTGTGGGTCACAATCTATTATGGGGTACCTGTGTGGAAAGAAGCAACCACCACTCTATTTTGTGCATCAAATGCTAAAGCATATGATACAGAGGTACATAAT---GTCTGGGCCACACATGCCTGTGTACCCACAGACCCCAACCCACAAGAAGTAAGATTG---GAAAATGTGACAGAAAATTTTAACATGTGGAAAAATAATATGGTAGAACAGATGCATGAGGATATAATTAGCCTATGGGATCAAAGCCTAAAACCATGTGTGAAATTAACCCCACTCTGTGTTACTTTAAACTGCACTGATGCTAATACCACTAATACTAATGCCAAT------------------------------------------------------------------------------AGTACTAATAACAGTAGCTTGGGAACAATGGAGAAAGGAGAAATAAAAAACTGCTCTTTCAACATCACC---ACAAACCTAAGAGAT------AAGGTGCAGAAAGAATATGCACTTTTTTATAACCTTGATGTAGTGCCAATAAAGGGTGAGGAT---------------------------AATACTAGC---------------------------TATAGGTTGATAAGTTGTAATACCTCAGTCATTACACAGGCCTGTCCAAAGGTATCCTTTGAGCCAATTCCCATACATTATTGTACTCCAGCTGGTTTTGCGATTCTACAATGT---AATGATAAGAAATTCAATGGATCAGGACCATGTACAAATGTCAGCACAGTACAATGTACACATGGAATTAGGCCAGTAGTATCAACTCAACTGCTGTTAAATGGCAGTCTAGCAGAAAAA---GAGGTAGTAATTAGGTCTGAGAATTTCACAAATAATGCTAAAACCATAATAGTACAGCTAAATGAATCTGTAGTAATTAATTGTACAAGACCCAACAACAATACAAGAAAAAGTATACCTATAGGA------------CCAGGAAGA---GCATTTTATGCAACAGGAGAAATAATAGGAGATATAAGACAAGCACATTGTAACGTT------AGTACAAAAGACTGGAAGGAAGCTTTACAACAGGTAGCTATAAAACTATCA---GAACAATTTGGG------------AATAAAACA---ATAGTCTTTAATCAA---------TCCTCAGGAGGAGACCCAGAAGTTGTAATGCACAGTTTTAATTGTAGAGGGGAATTTTTCTACTGTAATACAACAGGACTGTTTAATAATACTTGGGGGTTT---------AATAGTACTTGG---------------------GATGCTATTAATGTGCCAGAA------------------AATGACACA------------------------------ATCACACTCCCATGCAGAATAAAACAAATTGTAAACATGTGGCAGGAAGTAGGAAAAGCAATGTATGCCCCTCCCATCAGAGGACGACTTAATTGTTCATCAAATATTACAGGGCTGCTATTAACAAGAGATGGTGGT------------------AATACCACA---------------------AATAACACT---GAGGTCTTCAGACCTGGAGGAGGAGATATGAGAGACAATTGGAGA---AGTGAATTATATAAATATAAAGTAGTAAAAATTGAACCA---TTAGGAATAGCACCC---ACCAAGGCAAAGAGAAGAGTGGTGCAGAGAGAA---AAAAGAGCAGTG---GGA---ATAGGA---GCTTTG---TTCCTT---GGG---------TTCTTGGGA---GCAGCAGGAAGCACTATGGGCGCAGCGTCGATG---ACGCTGACGGTACAGGCCAGACTATTATTGTCTGGTATAGTGCAACAGCAGAACAATTTGCTGAGAGCTATTGAGGCGCAACAGCATCTGTTGCAACTCACAGTCTGGGGCATCAAGCAGCTCCAGGCAAGA---GTCCTGGCTGTGGAAAGATACCTAAGAGATCAACAGCTCCTGGGGATTTGGGGATGCTCTGGAAAACTCATTTGCACCACTACTGTGCCTTGGAATGTTAGTTGGAGT---------------------------AATAAATCCATGAATGACATTTGGAAT---AACATGACCTGGATGGAGTGGGAAAGAGAGATTGAC------AATTATACAAACATAATATACACCTTACTTGAAGAATCGCAGAACCAACAAGATAAGAATGAACAGGAATTATTGGAATTGGATAAATGGGCAAGTTTGTGGAATTGGTTTAGCATAACAAATTGGCTGTGGTACATAAAAATATTCATAATGATAGTAGGAGGCTTAATAGGTTTAAGAATAGTTTTTACTGTATTTTCTATAGTGAATAGAGTTAGGCAGGGATATTCACCATTATCGTTTCAGACCCGC---TTCCCAACCTCGAGGGGA------CTCGACAGGCCCGAAGGAATCGAAGAAGAAGGTGGAGACAGAGACAGAGACAGATCCAGGCCATTAGTGGATGGATTCTTAGCAATTATCTGGGTCGACCTGCGGAGCCTGTGCCTCTTCAGCTACCATCGCTTGAGAGACTTACTCTTGATTGTAGCGAGGATTGTGGAACTTCTGGGACGCAGG---------------GGGTGGGAAGCCCTCAAATATTGGTGG---AATCTCCTGCGGTATTGG---------------------------------------------------AGCCAGGAACTAAGGAATAGTGCTATTAGCTTGCTTAATGCCACAGCCATAGCAGTAGCTGAGGGAACAGATAGGGTGGTAGAAGTATTACAAAGA------------------GCTTTTAGAGCTGTTATACACATACCTAGAAGAATAAGACAGGGCTTAGAAAGGTTTTTGCTATAA

2.04013240.ADARC.GU330413 ATGAGAGTGAAGGGGATCATGAGGAATTATCAGCACTTA---------TGGAGATGGGGC------------------------ATGATGCTCCTTGGGATAATCATGATC------------TGTAGTGCTGCA---------GAACAATTGTGGGTCACAATCTATTATGGGGTACCTGTGTGGAAAGAAGCAACCACCACTCTATTTTGTGCATCAAATGCTAAAGCATATGATACAGAGGTACATAAT---GTCTGGGCCACACATGCCTGTGTACCCACAGACCCCAACCCACAAGAAGTAAGATTG---GAAAATGTGACAGAAAATTTTAACATGTGGAAAAATAATATGGTAGAACAGATGCATGAGGATATAATTAGCCTATGGGATCAAAGCCTAAAACCATGTGTGAAATTAACCCCACTCTGTGTTACTTTAAACTGCACTGATGCTAATACCACTAATACTAATGCCAAT------------------------------------------------------------------------------AGTACTAATAACAGTAGCTTGGGAACAATGGAGAAAGGAGAAATAAAAAACTGCTCTTTCAACATCACC---ACAAACCTAAGAGAT------AAGGTGCAGAAAGAATATGCACTTTTTTATAACCTTGATGTAGTGCCAATAAAGGGTGAGGAT---------------------------AATACTAGC---------------------------TATAGGTTGATAAGTTGTAATACCTCAGTCATTACACAGGCCTGTCCAAAGGTATCCTTTGAGCCAATTCCCATACATTATTGTACTCCAGCTGGTTTTGCGATTCTACAATGT---AATGATAAGAAATTCAATGGATCAGGACCATGTACAAATGTCAGCACAGTACAATGTACACATGGAATTAGGCCAGTAGTATCAACTCAACTGCTGTTAAATGGCAGTCTAGCAGAAAAA---GAGGTAGTAATTAGGTCTGAGAATTTCACAAATAATGCTAAAACCATAATAGTACAGCTAAATGAATCTGTAGTAATTAATTGTACAAGACCCAACAACAATACAAGAAAAAGTATACCTATAGGA------------CCAGGAAGA---GCATTTTATGCAACAGGAGAAATAATAGGAGATATAAGACAAGCACATTGTAACGTT------AGTACAAAAGCCTGGAAGGAAGCTTTACAACAGGTAGCTATAAAACTATCA---GAACAATTTGGG------------AATAAAACA---ATAGTCTTTAATCAA---------TCCTCAGGAGGAGACCCAGAAGTTGTAATGCACAGTTTTAATTGTAGAGGGGAATTTTTCTACTGTAATACAACAGGACTGTTTAATAATACTTGGGGGTTT---------AATAGTACTTGG---------------------GATGCTATTAATGTGCCAGAA------------------AATGACACA------------------------------ATCACACTCCCATGCAGAATAAAACAAATTGTAAACATGTGGCAGGAAGTAGGAAAAGCAATGTATGCCCCTCCCATCAGAGGACGACTTAATTGTTCATCAAATATTACAGGGCTGCTATTAACAAGAGATGGTGGT------------------AATACCACA---------------------AATAACACT---GAGGTCTTCAGACCTGGAGGAGGAGATATGAGAGACAATTGGAGA---AGTGAATTATATAAATATAAAGTAGTAAAAATTGAACCA---TTAGGAATAGCACCC---ACCAAGGCAAAGAGAAGAGTGGTGCAGAGAGAA---AAAAGAGCAGTG---GGA---ATAGGA---GCTTTG---TTCCTT---GGG---------TTCTTGGGA---GCAGCAGGAAGCACTATGGGCGCAGCGTCGATG---ACGCTGACGGTACAGGCCAGACTATTATTGTCTGGTATAGTGCAACAGCAGAACAATTTGCTGAGAGCTATTGAGGCGCAACAGCATCTGTTGCAACTCACAGTCTGGGGCATCAAGCAGCTCCAGGCAAGA---GTCCTGGCTGTGGAAAGATACCTAAGAGATCAACAGCTCCTGGGGATTTGGGGATGCTCTGGAAAACTCATTTGCACCACTACTGTGCCTTGGAATGTTAGTTGGAGT---------------------------AATAAATCCATGAATGACATTTGGAAT---AACATGACCTGGATGGAGTGGGAAAGAGAGATTGAC------AATTATACAAACATAATATACACCTTACTTGAAGAATCGCAGAACCAACAAGATAAGAATGAACAGGAATTATTGGAATTGGATAAATGGGCAAGTTTGTGGAATTGGTTTAGCATAACAAATTGGCTGTGGTACATAAAAATATTCATAATGATAGTAGGAGGCTTAATAGGTTTAAGAATAGTTTTTACTGTATTTTCTATAGTGAATAGAGTTAGGCAGGGATATTCACCATTATCGTTTCAGACCCGC---TTCCCAACCTCGAGGGGA------CTCGACAGGCCCGAAGGAATCGAAGAAGAAGGTGGAGACAGAGACAGAGACAGATCCAGGCCATTAGTGGATGGATTCTTAGCAATTATCTGGGTCGACCTGCGGAGCCTGTGCCTCTTCAGCTACCATCGCTTGAGAGACTTACTCTTGATTGTAGCGAGGATTGTGGAACTTCTGGGACGCAGG---------------GGGTGGGAAGCCCTCAAATATTGGTGG---AATCTCCTGCGGTATTGG---------------------------------------------------AGCCAGGAACTAAGGAATAGTGCTATTAGCTTGCTTAATGCCACAGCCATAGCAGTAGCTGAGGGAACAGATAGGGTGTTAGAAGTATTACAAAGA------------------GCTTTTAGAGCTGTTATACACATACCTAGAAGAATAAGACAGGGCTTAGAAAGGTTTTTGCTATAA

2.04013240.ADARC.GU330414 ATGAGAGTGAAGGGGATCATGAGGAATTATCAGCACTTA---------TGGAGATGGGGC------------------------ATGATGCTCCTTGGGATAATCATGATC------------TGTAGTGCTGCA---------GAACAATTGTGGGTCACAATCTATTATGGGGTACCTGTGTGGAAAGAAGCAACCACCACTCTATTTTGTGCATCAAATGCTAAAGCATATGATACAGAGGTACATAAT---GTCTGGGCCACACATGCCTGTGTACCCACAGACCCCAACCCACAAGAAGTAAGATTG---GAAAATGTGACAGAAAATTTTAACATGTGGAAAAATAATATGGTAGAACAGATGCATGAGGATATAATTAGCCTATGGGATCAAAGCCTAAAACCATGTGTGAAATTAACCCCACTCTGTGTTACTTTAAACTGCACTGATGCTAATACCACTAATACTAATGCCAAT------------------------------------------------------------------------------AGTACTAATAACAGTAGCTTGGGAACAATGGAGAAAGGAGAAATAAAAAACTGCTCTTTCAACATCACC---ACAAACCTAAGAGAT------AAGGTGCAGAAAGAATATGCACTTTTTTATAACCTTGATGTAGTGCCAATAAAGGGTGAGGAT---------------------------AATACTAGC---------------------------TATAGGTTGATAAGTTGTAATACCTCAGTCATTACACAGGCCTGTCCAAAGGTATCCTTTGAGCCAATTCCCATACATTATTGTACTCCAGCTGGTTTTGCGATTCTACAATGT---AATGATAAGAAATTCAATGGATCAGGACCATGTACAAATGTCAGCACAGTACAATGTACACATGGAATTAGGCCAGTAGTATCAACTCAACTGCTGTTAAATGGCAGTCTAGCAGAAAAA---GAGGTAGTAATTAGGTCTGAGAATTTCACAAATAATGCTAAAACCATAATAGTACAGCTAAATGAATCTGTAGTAATTAATTGTACAAGACCCAACAACAATACAAGAAAAAGTATACCTATAGGA------------CCAGGAAGA---GCATTTTATGCAACAGGAGAAATAATAGGAGATATAAGACAAGCACATTGTAACGTT------AGTACAAAAGCCTGGAAGGAAGCTTTACAACAGGTAGCTATAAAACTATCA---GAACAATTTGGG------------AATAAAACA---ATAGTCTTTAATCAA---------TCCTCAGGAGGAGACCCAGAAGTTGTAATGCACAGTTTTAATTGTAGAGGGGAATTTTTCTACTGTAATACAACAGGACTGTTTAATAATACTTGGGGGTTT---------AATAGTACTTGG---------------------GATGCTATTAATGTACCAGAA------------------AATGACACA------------------------------ATCACACTCCCATGCAGAATAAAACAAATTGTAAACATGTGGCAGGAAGTAGGAAAAGCAATGTATGCCCCTCCCATCAGAGGACGACTTAATTGTTCATCAAATATTACAGGGCTGCTATTAACAAGAGATGGTGGT------------------AATACCACA---------------------AATAACACT---GAGGTCTTCAGACCTGGAGGAGGAGATATGAGAGACAATTGGAGA---AGTGAATTATATAAATATAAAGTAGTAAAAATTGAACCA---TTAGGAATAGCACCC---ACCAAGGCAAAGAGAAGAGTGGTGCAGAGAGAA---AAAAGAGCAGTG---GGA---ATAGGA---GCTTTG---TTCCTT---GGG---------TTCTTGGGA---GCAGCAGGAAGCACTATGGGCGCAGCGTCGATG---ACGCTGACGGTACAGGCCAGACTATTATTGTCTGGTATAGTGCAACAGCAGAACAATTTGCTGAGAGCTATTGAGGCGCAACAGCATCTGTTGCAACTCACAGTCTGGGGCATCAAGCAGCTCCAGGCAAGA---GTCCTGGCTGTGGAAAGATACCTAAGAGATCAACAGCTCCTGGGGATTTGGGGATGCTCTGGAAAACTCATTTGCACCACTACTGTGCCTTGGAATGTTAGTTGGAGT---------------------------AATAAATCCATGAATGACATTTGGAAT---AACATGACCTGGATGGAGTGGGAAAGAGAGATTGAC------AATTATACAAACATAATATACACCTTACTTGAAGAATCGCAGAACCAACAAGATAAGAATGAACAGGAATTATTGGAATTGGATAAATGGGCAAGTTTGTGGAATTGGTTTAGCATAACAAATTGGCTGTGGTACATAAAAATATTCATAATGATAGTAGGAGGCTTAATAGGTTTAAGAATAGTTTTTACTGTATTTTCTATAGTGAATAGAGTTAGGCAGGGATATTCACCATTATCGTTTCAGACCCGC---TTCCCAACCTCGAGGGGA------CTCGACAGGCCCGAAGGAATCGAAGAAGAAGGTGGAGACAGAGACAGAGACAGATCCAGGCCATTAGTGGATGGATTCTTAGCAATTATCTGGGTCGACCTGCGGAGCCTGTGCCTCTTCAGCTACCATCGCTTGAGAGACTTACTCTTGATTGTAGCGAGGATTGTGGAACTTCTGGGACGCAGG---------------GGGTGGGAAGCCCTCAAATATTGGTGG---AATCTCCTGCGGTATTGG---------------------------------------------------AGCCAGGAACTAAGGAATAGTGCTATTAGCTTGCTTAATGCCACAGCCATAGCAGTAGCTGAGGGAACAGATAGGGTGTTAGAAGTATTACAAAGA------------------GCTTTTAGAGCTGTTATACACATACCTAGAAGAATAAGACAGGGCTTAGAAAGGTTTTTGCTATAA

2.04013240.ADARC.GU330415 ATGAGAGTGAAGGGGATCATGAGGAATTATCAGCACTTA---------TGGAGATGGGGC------------------------ATGATGCTCCTTGGGATAATCATGATC------------TGTAGTGCTGCA---------GAACAATTGTGGGTCACAATCTATTATGGGGTACCTGTGTGGAAAGAAGCAACCACCACTCTATTTTGTGCATCAAATGCTAAAGCATATGATACAGAGGTACATAAT---GTCTGGGCCACACATGCCTGTGTACCCACAGACCCCAACCCACAAGAAGTAAGATTG---GAAAATGTGACAGAAAATTTTAACATGTGGAAAAATAATATGGTAGAACAGATGCATGAGGATATAATTAGCCTATGGGATCAAAGCCTAAAACCATGTGTGAAATTAACCCCACTCTGTGTTACTTTAAACTGCACTGATGCTAATACCACTAATACTAATGCCAAT------------------------------------------------------------------------------AGTACTAATAACAGTAGCTTGGGAACAATGGAGAAAGGAGAAATAAAAAACTGCTCTTTCAACATCACC---ACAAACCTAAGAGAT------AAGGTGCAGAAAGAATATGCACTTTTTTATAACCTTGATGTAGTGCCAATAAAGGGTGAGGAT---------------------------AATACTAGC---------------------------TATAGGTTGATAAGTTGTAATACCTCAGTCATTACACAGGCCTGTCCAAAGGTATCCTTTGAGCCAATTCCCATACATTATTGTACTCCAGCTGGTTTTGCGATTCTACAATGT---AATGATAAGAAATTCAATGGATCAGGACCATGTACAAATGTCAGCACAGTACAATGTACACATGGAATTAGGCCAGTAGTATCAACTCAACTGCTGTTAAATGGCAGTCTAGCAGAAAAA---GAGGTAGTAATTAGGTCTGAGAATTTCACAAATAATGCTAAAACCATAATAGTACAGCTAAATGAATCTGTAGTAATTAATTGTACAAGACCCAACAACAATACAAGAAAAAGTATACCTATAGGA------------CCAGGAAGA---GCATTTTATGCAACAGGAGAAATAATAGGAGATATAAGACAAGCACATTGTAACGTT------AGTACAAAAGCCTGGAAGGAAGCTTTACAACAGGTAGCTATAAAACTATCA---GAACAATTTGGG------------AATAAAACA---ATAGTCTTTAATCAA---------TCCTCAGGAGGAGACCCAGAAGTTGTAATGCACAGTTTTAATTGTAGAGGGGAATTTTTCTACTGTAATACAACAGGACTGTTTAATAATACTTGGGGGTTT---------AATAGTACTTGG---------------------GATGCTATTAATGTGCCAGAA------------------AATGACACA------------------------------ATCACACTCCCATGCAGAATAAAACAAATTGTAAACATGTGGCAGGAAGTAGGAAAAGCAATGTATGCCCCTCCCATCAGAGGACAACTTAATTGTTCATCAAATATTACAGGGCTGCTATTAACAAGAGATGGTGGT------------------AATACCACA---------------------AATAACACT---GAGGTCTTCAGACCTGGAGGAGGAGATATGAGAGACAATTGGAGA---AGTGAATTATATAAATATAAAGTAGTAAAAATTGAACCA---TTAGGAATAGCACCC---ACCAAGGCAAAGAGAAGAGTGGTGCAGAGAGAA---AAAAGAGCAGTG---GGA---ATAGGA---GCTTTG---TTCCTT---GGG---------TTCTTGGGA---GCAGCAGGAAGCACTATGGGCGCAGCGTCGATG---ACGCTGACGGTACAAGCCAGACTATTATTGTCTGGTATAGTGCAACAGCAGAACAATTTGCTGAGAGCTATTGAGGCGCAACAGCATCTGTTGCAACTCACAGTCTGGGGCATCAAGCAGCTCCAGGCAAGA---GTCCTGGCTGTGGAAAGATACCTAAGGGATCAACAGCTCCTGGGGATTTGGGGATGCTCTGGAAAACTCATTTGCACCACTGCTGTGCCTTGGAATGTTAGTTGGAGT---------------------------AATAAATCCATGAATGACATTTGGAAT---AACATGACCTGGATGGAGTGGGAAAGAGAGATTGAC------AATTATACAAACATAATATACACCTTACTTGAAGAATCGCAGAACCAACAAGATAAGAATGAACAGGAATTATTGGAATTGGATAAATGGGCAAGTTTGTGGAATTGGTTTAGCATAACAAATTGGCTGTGGTACATAAAAATATTCATAATGATAGTAGGAGGCTTAATAGGTTTAAGAATAGTTTTTACTGTATTTTTTATAGTGAATAGAGTTAGGCAGGGATATTCACCATTATCGTTTCAGACCCGC---TTCCCAACCTCGAGGGGA------CTCGACAGGCCCGAAGGAATCGAAGAAGAAGGTGGAGACAGAGACAGAGACAGATCCAGGCCATTAGTGGATGGATTCTTAGCAATTATCTGGGTCGACCTGCGGAGCCTGTGCCTCTTCAGCTACCATCGCTTGAGAGACTTACTCTTGATTGTAGCGAGGATTGTGGAACTTCTGGGACGCAGG---------------GGGTGGGAAGCCCTCAAATATTGGTGG---AATCTCCTGCGGTATTGG---------------------------------------------------AGCCAGGAACTAAGGAATAGTGCTATTAGCTTGCTTAATGCCACAGCCATAGCAGTAGCTGAGGGAACAGATAGGGTGTTAGAAGTATTACAAAGA------------------GCTTTTAGAGCTGTTATACACATACCTAGAAGAATAAGACAGGGCTTAGAAAGGTTTTTGCTATAA

2.04013240.ADARC.GU330416 ATGAGAGTGAAGGGGATCATGAGGAATTATCAGCACTTA---------TGGAGATGGGGC------------------------ATGATGCTCCTTGGGATAATCATGATC------------TGTAGTGCTGCA---------GAACAATTGTGGGTCACAATCTATTATGGGGTACCTGTGTGGAAAGAAGCAACCACCACTCTATTTTGTGCATCAAATGCTAAAGCATATGATACAGAGGTACATAAT---GTCTGGGCCACACATGCCTGTGTACCCACAGACCCCAACCCACAAGAAGTAAGATTG---GAAAATGTGACAGAAAATTTTAACATGTGGAAAAATAATATGGTAGAACAGATGCATGAGGATATAATTAGCCTATGGGATCAAAGCCTAAAACCATGTGTGAAATTAACCCCACTCTGTGTTACTTTAAACTGCACTGATGCTAATACCACTAATACTAATGCCAAT------------------------------------------------------------------------------AGTACTAATAACAGTAGCTTGGAAACAATGGAGAAAGGAGAAATAAAAAACTGCTCTTTCAACATCACC---ACAAACCTAAGAGAT------AAGGTGCAGAAAGAATATGCACTTTTTTATAACCTTGATGTAGTGCCAATAAAGGGTGAGGAT---------------------------AATACTAGC---------------------------TATAGGTTGATAAGTTGTAATACCTCAGTCATTACACAGGCCTGTCCAAAGGTATCCTTTGAGCCAATTCCCATACATTATTGTACTCCAGCTGGTTTTGCGATTCTACAATGT---AATGATAAGAAATTCAATGGATCAGGACCATGTACAAATGTCAGCACAGTACAATGTACACATGGAATTAGGCCAGTAGTATCAACTCAACTGCTGTTAAATGGCAGTCTAGCAGAAAAA---GAGGTAGTAATTAGGTCTGAGAATTTCACAAATAATGCTAAAACCATAATAGTACAGCTAAATGAATCTGTAGTAATTAATTGTACAAGACCCAACAACAATACAAGAAAAAGTATACCTATAGGA------------CCAGGAAGA---GCATTTTATGCAACAGGAGAAATAATAGGAGATATAAGACAAGCACATTGTAACGTT------AGTACAAAAGCCTGGAAGGAAGCTTTACAACAGGTAGCTATAAAACTATCA---GAACAATTTGGG------------AATAAAACA---ATAGTCTTTAATCAA---------TCCTCAGGAGGAGACCCAGAAGTTGTAATGCACAGTTTTAATTGTAGAGGGGAATTTTTCTACTGTAATACAACAGGACTGTTTAATAATACTTGGGGGTTT---------AATAGTACTTGG---------------------GATGCTATTAATGTGCCAGAA------------------AATGACACA------------------------------ATCACACTCCCATGCAGAATAAAACAAATTGTAAACATGTGGCAGGAAGTAGGAAAAGCAATGTATGCCCCTCCCATCAGAGGACAACTTAATTGTTCATCAAATATTACAGGGCTGCTATTAACAAGAGATGGTGGT------------------AATACCACA---------------------AATAACACT---GAGGTCTTCAGACCTGGAGGAGGAGATATGAGAGACAATTGGAGA---AGTGAATTATATAAATATAAAGTAGTAAAAATTGAACCA---TTAGGAATAGCACCC---ACCAAGGCAAAGAGAAGAGTGGTGCAGAGAGAA---AAAAGAGCAGTG---GGA---ATAGGA---GCTTTG---TTCCTT---GGG---------TTCTTGGGA---GCAGCAGGAAGCACTATGGGCGCAGCGTCGATG---ACGCTGACGGTACAAGCCAGACTATTATTGTCTGGTATAGTGCAACAGCAGAACAATTTGCTGAGAGCTATTGAGGCGCAACAGCATCTGTTGCAACTCACAGTCTGGGGCATCAAGCAGCTCCAGGCAAGA---GTCCTGGCTGTGGAAAGATACCTAAGGGATCAACAGCTCCTGGGGATTTGGGGATGCTCTGGAAAACTCATTTGCACCACTGCTGTGCCTTGGAATGTTAGTTGGAGT---------------------------AATAAATCCATGAATGACATTTGGAAT---AACATGACCTGGATGGAGTGGGAAAGAGAGATTGAC------AATTATACAAACATAATATACACCTTACTTGAAGAATCGCAGAACCAACAAGATAAGAATGAACAGGAATTATTGGAATTGGATAAATGGGCAAGTTTGTGGAATTGGTTTAGCATAACAAATTGGCTGTGGTACATAAAAATATTCATAATGATAGTAGGAGGCTTAATAGGTTTAAGAATAGTTTTTACTGTATTTTTTATAGTGAATAGAGTTAGGCAGGGATATTCACCATTATCGTTTCAGACCCGC---TTCCCAACCTCGAGGGGA------CTCGACAGGCCCGAAGGAATCGAAGAAGAAGGTGGAGACAGAGACAGAGACAGATCCAGGCCATTAGTGGATGGATTCTTAGCAATTATCTGGGTCGACCTGCGGAGCCTGTGCCTCTTCAGCTACCATCGCTTGAGAGACTTACTCTTGATTGTAGCGAGGATTGTGGAACTTCTGGGACGCAGG---------------GGGTGGGAAGCCCTCAAATATTGGTGG---AATCTCCTGCGGTATTGG---------------------------------------------------AGCCAGGAACTAAGGAATAGTGCTATTAGCTTGCTTAATGCCACAGCCATAGCAGTAGCTGAGGGAACAGATAGGGTGTTAGAAGTATTACAAAGA------------------GCTTTTAGAGCTGTTATACACATACCTAGAAGAATAAGACAGGGCTTAGAAAGGTTTTTGCTATAA

2.04013240.ADARC.GU330417 ATGAGAGTGAAGGGGATCATGAGGAATTATCAGCACTTA---------TGGAGATGGGGC------------------------ATGATGCTCCTTGGGATAATCATGATC------------TGTAGTGCTGCA---------GAACAATTGTGGGTCACAATCTATTATGGGGTACCTGTGTGGAAAGAAGCAACCACCACTCTATTTTGTGCATCAAATGCTAAAGCATATGATACAGAGGTACATAAT---GTCTGGGCCACACATGCCTGTGTACCCACAGACCCCAACCCACAAGAAGTAAGATTG---GAAAATGTGACAGAAAATTTTAACATGTGGAAAAATAATATGGTAGAACAGATGCATGAGGATATAATTAGCCTATGGGATCAAAGCCTAAAACCATGTGTGAAATTAACCCCACTCTGTGTTACTTTAAACTGCACTGATGCTAATACCACTAATACTAATGCCAAT------------------------------------------------------------------------------AGTACTAATAACAGTAGCTTGGGAACAATGGAGAAAGGAGAAATAAAAAACTGCTCTTTCAACATCACC---ACAAACCTAAGAGAT------AAGGTGCAGAAAGAATATGCACTTTTTTATAACCTTGATGTAGTGCCAATAAAGGGTGAGGAT---------------------------AATACTAGC---------------------------TATAGGTTGATAAGTTGTAATACCTCAGTCATTACACAGGCCTGTCCAAAGGTATCCTTTGAGCCAATTCCCATACATTATTGTACTCCAGCTGGTTTTGCGATTCTACAATGT---AATGATAAGAAATTCAATGGATCAGGACCATGTACAAATGTCAGCACAGTACAATGTACACATGGAATTAGGCCAGTAGTATCAACTCAACTGCTGTTAAATGGCAGTCTAGCAGAAAAA---GAGGTAGTAATTAGGTCTGAGAATTTCACAAATAATGCTAAAACCATAATAGTACAGCTAAATGAATCTGTAGTAATTAATTGTACAAGACCCAACAACAATACAAGAAAAAGTATACCTATAGGA------------CCAGGAAGA---GCATTTTATGCAACAGGAGAAATAATAGGAGATATAAGACAAGCACATTGTAACGTT------AGTACAAAAGCCTGGAAGGAAGCTTTACAACAGGTAGCTATAAAACTATCA---GAACAATTTGGG------------AATAAAACA---ATAGTCTTTAATCAA---------TCCTCAGGAGGAGACCCAGAAGTTGTAATGCACAGTTTTAATTGTAGAGGGGAATTTTTCTACTGTAATACAACAGGACTGTTTAATAATACTTGGGGGTTT---------AATAGTACTTGG---------------------GATGCTATTAATGTGCCAGAA------------------AATGACACA------------------------------ATCACACTCCCATGCAGAATAAAACAAATTGTAAACATGTGGCAGGAAGTAGGAAAAGCAATGTATGCCCCTCCCATCAGAGGACAACTTAATTGTTCATCAAATATTACAGGGCTGCTATTAACAAGAGATGGTGGT------------------AATACCACA---------------------AATAACACT---GAGGTCTTCAGACCTGGAGGAGGAGATATGAGAGACAATTGGAGA---AGTGAATTATATAAATATAAAGTAGTAAAAATTGAACCA---TTAGGAATAGCACCC---ACCAAGGCAAAGAGAAGAGTGGTGCAGAGAGAA---AAAAGAGCAGTG---GGA---ATAGGA---GCTTTG---TTCCTT---GGG---------TTCTTGGGA---GCAGCAGGAAGCACTATGGGCGCAGCGTCGATG---ACGCTGACGGTACAAGCCAGACTATTATTGTCTGGTATAGTGCAACAGCAGAACAATTTGCTGAGAGCTATTGAGGCGCAACAGCATCTGTTGCAACTCACAGTCTGGGGCATCAAGCAGCTCCAGGCAAGA---GTCCTGGCTGTGGAAAGATACCTAAGGGATCAACAGCTCCTGGGGATTTGGGGATGCTCTGGAAAACTCATTTGCACCACTGCTGTGCCTTGGAATGTTAGTTGGAGT---------------------------AATAAATCCATGAATGACATTTGGAAT---AACATGACCTGGATGGAGTGGGAAAGAGAGATTGAC------AATTATACAAACATAATATACACCTTACTTGAAGAATCGCAGAACCAACAAGATAAGAATGAACAGGAATTATTGGAATTGGATAAATGGGCAAGTTTGTGGAATTGGTTTAGCATAACAAATTGGCTGTGGTACATAAAAATATTCATAATGATAGTAGGAGGCTTAATAGGTTTAAGAATAGTTTTTACTGTATTTTTTATAGTGAATAGAGTTAGGCAGGGATATTCACCATTATCGTTTCAGACCCGC---TTCCCAACCTCGAGGGGA------CTCGACAGGCCCGAAGGAATCGAAGAAGAAGGTGGAGACAGAGACAGAGACAGATCCAGGCCATTAGTGGATGGATTCTTAGCAATTATCTGGGTCGACCTGCGGAGCCTGTGCCTCTTCAGCTACCATCGCTTGAGAGACTTACTCTTGATTGTAGCGAGGATTGTGGAACTTCTGGGACGCAGG---------------GGGTGGGAAGCCCTCAAATATTGGTGG---AATCTCCTGCGGTATTGG---------------------------------------------------AGCCAGGAACTAAGGAATAGTGCTATTAGCTTGCTTAATGCCACAGCCATAGCAGTAGCTGAGGGAACAGATAGGGTGTTAGAAGTATTACAAAGA------------------GCTTTTAGAGCTGTTATACACATACCTAGAAGAATAAGACAGGGCTTAGAAAGGTTTTTGCTATAA

2.04013240.ADARC.GU330418 ATGAGAGTGAAGGGGATCATGAGGAATTATCAGCACTTA---------TGGAGATGGGGC------------------------ATGATGCTCCTTGGGATAATCATGATC------------TGTAGTGCTGCA---------GAACAATTGTGGGTCACAATCTATTATGGGGTACCTGTGTGGAAAGAAGCAACCACCACTCTATTTTGTGCATCAAATGCTAAAGCATATGATACAGAGGTACATAAT---GTCTGGGCCACACATGCCTGTGTACCCACAGACCCCAACCCACAAGAAGTAAGATTG---GAAAATGTGACAGAAAATTTTAACATGTGGAAAAATAATATGGTAGAACAGATGCATGAGGATATAATTAGCCTATGGGATCAAAGCCTAAAACCATGTGTGAAATTAACCCCACTCTGTGTTACTTTAAACTGCACTGATGCTAATACCACTAATACTAATGCCAAT------------------------------------------------------------------------------AGTACTAATAACAGTAGCTTGGGAACAATGGAGAAAGGAGAAATAAAAAACTGCTCTTTCAACATCACC---ACAAACCTAAGAGAT------AAGGTGCAGAAAGAATATGCACTTTTTTATAACCTTGATGTAGTGCCAATAAAGGGTGAGGAT---------------------------AATACTAGC---------------------------TATAGGTTGATAAGTTGTAATACCTCAGTCATTACACAGGCCTGTCCAAAGGTATCCTTTGAGCCAATTCCCATACATTATTGTACTCCAGCTGGTTTTGCGATTCTACAATGT---AATGATAAGAAATTCAATGGATCAGGACCATGTACAAATGTCAGCACAGTACAATGTACACATGGAATTAGGCCAGTAGTATCAACTCAACTGCTGTTAAATGGCAGTCTAGCAGAAAAA---GAGGTAGTAATTAGGTCTGAGAATTTCACAAATAATGCTAAAACCATAATAGTACAGCTAAATGAATCTGTAGTAATTAATTGTACAAGACCCAACAACAATACAAGAAAAAGTATACCTATAGGA------------CCAGGAAGA---GCATTTTATGCAACAGGAGAAATAATAGGAGATATAAGACAAGCACATTGTAACGTT------AGTACAAAAGCCTGGAAGGAAGCTTTACAACAGGTAGCTATAAAACTATCA---GAACAATTTGGG------------AATAAAACA---ATAGTCTTTAATCAA---------TCCTCAGGAGGAGACCCAGAAGTTGTAATGCACAGTTTTAATTGTAGAGGGGAATTTTTCTACTGTAATACAACAGGACTGTTTAATAATACTTGGGGGTTT---------AATAGTACTTGG---------------------GATGCTATTAATGTGCCAGAA------------------AATGACACA------------------------------ATCACACTCCCATGCAGAATAAAACAAATTGTAAACATGTGGCAGGAAGTAGGAAAAGCAATGTATGCCCCTCCCATCAGAGGACAAATTAATTGTTCATCAAATATTACAGGGCTGCTATTAACAAGAGATGGTGGT------------------AATACCACA---------------------AATAACACT---GAGGTCTTCAGACCTGGAGGAGGAGATATGAGAGACAATTGGAGA---AGTGAATTATATAAATATAAAGTAGTAAAAATTGAACCA---TTAGGAATAGCACCC---ACCAGGGCAAAGAGAAGAGTGGTGCAGAGAGAA---AAAAGAGCAGTG---GGA---ATAGGA---GCTTTG---TTCCTT---GGG---------TTCTTGGGA---GCAGCAGGAAGCACTATGGGCGCAGCGTCGATG---ACGCTGACGGTACAGGCCAGACTATTATTGTCTGGTATAGTGCAACAGCAGAACAATTTGCTGAGAGCTATTGAGGCGCAACAGCATCTGTTGCAACTCACAGTCTGGGGCATCAAGCAGCTCCAGGCAAGA---GTCCTGGCTGTGGAAAGATACCTAAGGGATCAACAGCTCCTGGGGATTTGGGGATGCTCTGGAAAACTCATTTGCACCACTGCTGTGCCTTGGAATGTTAGTTGGAGT---------------------------AATAAATCCATGAATGACATTTGGAAT---AACATGACCTGGATGGAGTGGGAAAGAGAGATTGAC------AATTATACAAACATAATATACACCTTACTTGAAGAATCGCAGAACCAACAAGATAAGAATGAACAGGAATTATTGGAATTGGATAAATGGGCAAGTTTGTGGAATTGGTTTAGCATAACAAATTGGCTGTGGTACATAAAAATATTCATAATGATAGTAGGAGGCTTAATAGGTTTAAGAATAGTTTTTACTGTATTTTCTATAGTGAATAGAGTTAGGCAGGGATATTCACCATTATCGTTTCAGACCCGC---CTCCCAACTTCGAGGGGA------CTCGACAGGCCCGAAGGAATCGAAGAAGAAGGTGGAGACAGAGACAGAGACAGATCCAGGCCATTAGTGGATGGATTCTTAGCAATTATCTGGGTCGACCTGCGGAGCCTGTGCCTCTTCAGCTACCATCGCTTGAGAGACTTACTCTTGATTGTAGCGAGGATTGTGGAACTTCTGGGACGCAGG---------------GGGTGGGAAGCCCTCAAATATTGGTGG---AATCTCCTGCGGTATTGG---------------------------------------------------AGCCAGGAACTAAGGAATAGTGCTATTAGCTTGCTTAATGCCACAGCCATAGCAGTAGCTGAGGGAACAGATAGGGTGTTAGAAGTATTACAAAGA------------------GCTTTTAGAGCTGTTATACACATACCTAGAAGAATAAGACAGGGCTTAGAAAGGTTTTTGCTATAA

2.04013240.ADARC.GU330419 ATGAGAGTGAAGGGGATCATGAGGAATTATCAGCACTTA---------TGGAGATGGGGC------------------------ATGATGCTCCTTGGGATAATCATGATC------------TGTAGTGCTGCA---------GAACAATTGTGGGTCACAATCTATTATGGGGTACCTGTGTGGAAAGAAGCAACCACCACTCTATTTTGTGCATCAAATGCTAAAGCATATGATACAGAGGTACATAAT---GTCTGGGCCACACATGCCTGTGTACCCACAGACCCCAACCCACAAGAAGTAAGATTG---GAAAATGTGACAGAAAATTTTAACATGTGGAAAAATAATATGGTAGAACAGATGCATGAGGATATAATTAGCCTATGGGATCAAAGCCTAAAACCATGTGTGAAATTAACCCCACTCTGTGTTACTTTAAACTGCACTGATGCTAATACCACTAATACTAATGCCAAT------------------------------------------------------------------------------AGTACTAATAACAGTAGCTTGGGAACAATGGAGAAAGGAGAAATAAAAAACTGCTCTTTCAACATCACC---ACAAACCTAAGAGAT------AAGGTGCAGAAAGAATATGCACTTTTTTATAACCTTGATGTAGTGCCAATAAAGGGTGAGGAT---------------------------AATACTAGC---------------------------TATAGGTTGATAAGTTGTAATACCTCAGTCATTACACAGGCCTGTCCAAAGGTATCCTTTGAGCCAATTCCCATACATTATTGTACTCCAGCTGGTTTTGCGATTCTACAATGT---AATGATAAGAAATTCAATGGATCAGGACCATGTACAAATGTCAGCACAGTACAATGTACACATGGAATTAGGCCAGTAGTATCAACTCAACTGCTGTTAAATGGCAGTCTAGCAGAAAAA---GAGGTAGTAATTAGGTCTGAGAATTTCACAAATAATGCTAAAACCATAGTAGTACAGCTAAATGAATCTGTAGTAATTAATTGTACAAGACCCAACAACAATACAAGAAAAAGTATACCTATAGGA------------CCAGGAAGA---GCATTTTATGCAACAGGAGAAATAATAGGAGATATAAGACAAGCACATTGTAACGTT------AGTACAAAAGCCTGGAAGGAAGCTTTACAACAGGTAGCTATAAAACTATCA---GAACAATTTGGG------------AATAAAACA---ATAGTCTTTAATCAA---------TCCTCAGGAGGAGACCCAGAAGTTGTAATGCACAGTTTTAATTGTAGAGGGGAATTTTTCTACTGTAATACAACAGGACTGTTTAATAATACTTGGGGGTTT---------AATAGTACTTGG---------------------GATGCTATTAATGTGCCAGAA------------------AATGACACA------------------------------ATCACACTCCCATGCAGAATAAAACAAATTGTAAACATGTGGCAGGAAGTAGGAAAAGCAATGTATGCCCCTCCCATCAGAGGACGACTTAATTGTTCATCAAATATTACAGGGCTGCTATTAACAAGAGATGGTGGT------------------AATACCACA---------------------AATAACACT---GAGGTCTTCAGACCTGGAGGAGGAGATATGAGAGACAATTGGAGA---AGTGAATTATATAAATATAAAGTAGTAAAAATTGAACCA---TTAGGAATAGCACCC---ACCAAGGCAAAGAGAAGAGTGGTGCAGAGAGAA---AAAAGAGCAGTG---GGA---ATAGGA---GCTTTG---TTCCTT---GGG---------TTCTTGGGA---GCAGCAGGAAGCACTATGGGCGCAGCGTCGATG---ACGCTGACGGTACAGGCCAGACTATTATTGTCTGGTATAGTGCAACAGCAGAACAATTTGCTGAGAGCTATTGAGGCGCAACAGCATCTGTTGCAACTCACAGTCTGGGGCATCAAGCAGCTCCAGGCAAGA---GTCCTGGCTGTGGAAAGATACCTAAGAGATCAACAGCTCCTGGGGATTTGGGGATGCTCTGGAAAACTCATTTGCACCACTACTGTGCCTTGGAATGTTAGTTGGAGT---------------------------AATAAATCCATGAATGACATTTGGAAT---AACATGACCTGGATGGAGTGGGAAAGAGAGATTGAC------AATTATACAAACATAATATACACCTTACTTGAAGAATCGCAGAACCAACAAGATAAGAATGAACAGGAATTATTGGAATTGGATAAATGGGCAAGTTTGTGGAATTGGTTTAGCATAACAAATTGGCTGTGGTACATAAAAATATTCATAATGATAGTAGGAGGCTTAATAGGTTTAAGAATAGTTTTTACTGTATTTTCTATAGTGAATAGAGTTAGGCAGGGATATTCATCATTATCGTTTCAGACCCGC---TTCCCAACCTCGAGGGGA------CTCGACAGGCCCGAAGGAATCGAAGAAGAAGGTGGAGACAGAGACAGAGACAGATCCAGGCCATTAGTGGATGGATTCTTAGCAATTATCTGGGTCGACCTGCGGAGCCTGTGCCTCTTCAGCTACCATCGCTTGAGAGACTTACTCTTGATTGTAGCGAGGATTGTGGAACTTCTGGGACGCAGG---------------GGGTGGGAAGCCCTCAAATATTGGTGG---AATCTCCTGCGGTATTGG---------------------------------------------------AGCCAGGAACTAAGGAATAGTGCTATTAGCTTGCTTAATGCCACAGCCATAGCAGTAGCTGAGGGAACAGATAGGGTGGTAGAAGTATTACAAAGA------------------GCTTTTAGAGCTGTTATACACATACCTAGAAGAATAAGACAGGGCTTAGAAAGGTTTTTGCTATAA

2.04013240.ADARC.GU330420 ATGAGAGTGAAGGGGATCATGAGGAATTATCAGCACTTA---------TGGAGATGGGGC------------------------ATGATGCTCCTTGGGATAATCATGATC------------TGTAGTGCTGCA---------GAACAATTGTGGGTCACAATCTATTATGGGGTACCTGTGTGGAAAGAAGCAACCACCACTCTATTTTGTGCATCAAATGCTAAAGCATATGATACAGAGGTACATAAT---GTCTGGGCCACACATGCCTGTGTACCCACAGACCCCAACCCACAAGAAGTAAGATTG---GAAAATGTGACAGAAAATTTTAACATGTGGAAAAATAATATGGTAGAACAGATGCATGAGGATATAATTAGCCTATGGGATCAAAGCCTAAAACCATGTGTGAAATTAACCCCACTCTGTGTTACTTTAAACTGCACTGATGCTAATACCACTAATACTAATGCCAAT------------------------------------------------------------------------------AGTACTAATAACAGTAGCTTGGGAACAATGGAGAAAGGAGAAATAAAAAACTGCTCTTTCAACATCACC---ACAAACCTAAGAGAT------AAGGTGCAGAAAGAATATGCACTTTTTTATAACCTTGATGTAGTGCCAATAAAGGGTGAGGAT---------------------------AATACTAGC---------------------------TATAGGTTGATAAGTTGTAATACCTCAGTCATTACACAGGCCTGTCCAAAGGTATCCTTTGAGCCAATTCCCATACATTATTGTACTCCAGCTGGTTTTGCGATTCTACAATGT---AATGATAAGAAATTCAATGGATCAGGACCATGTACAAATGTCAGCACAGTACAATGTACACATGGAATTAGGCCAGTAGTATCAACTCAACTGCTGTTAAATGGCAGTCTAGCAGAAAAA---GAGGTAGTAATTAGGTCTGAGAATTTCACAAATAATGCTAAAACCATAATAGTACAGCTAAATGAATCTGTAGTAATTAATTGTACAAGACCCAACAACAATACAAGAAAAAGTATACCTATAGGA------------CCAGGAAGA---GCATTTTATGCAACAGGAGAAATAATAGGAGATATAAGACAAGCACATTGTAACGTT------AGTACAAAAGCCTGGAAGGAAGCTTTACAACAGGTAGCTATAAAACTATCA---GAACAATTTGGG------------AATAAAACA---ATAGTCTTTAATCAA---------TCCTCAGGAGGAGACCCAGAAGTTGTAATGCACAGTTTTAATTGTAGAGGGGAATTTTTCTACTGTAATACAACAGGACTGTTTAATAATACTTGGGGGTTT---------AATAGTACTTGG---------------------GATGCTATTAATGTGCCAGAA------------------AATGACACA------------------------------ATCACACTCCCATGCAGAATAAAACAAATTGTAAACATGTGGCAGGAAGTAGGAAAAGCAATGTATGCCCCTCCCATCAGAGGACGACTTAATTGTTCATCAAATATTACAGGGCTGCTATTAACAAGAGATGGTGGT------------------AATACCACA---------------------AATAACACT---GAGGTCTTCAGACCTGGAGGAGGAGATATGAGAGACAATTGGAGA---AGTGAATTATATAAATATAAAGTAGTAAAAATTAAACCA---TTAGGAATAGCACCC---ACCAAGGCAAAGAGAAGAGTGGTGCAGAGAGAA---AAAAGAGCAGTG---GGA---ATAGGA---GCTTTG---TTCCTT---GGG---------TTCTTGGGA---GCAGCAGGAAGCACTATGGGCGCAGCGTCGATG---ACGCTGACGGTACAGGCCAGACTATTATTGTCTGGTATAGTGCAACAGCAGAACAATTTGCTGAGAGCTATTGAGGCGCAACAGCATCTGTTGCAACTCACAGTCTGGGGCATCAAGCAGCTCCAGGCAAGA---GTCCTGGCTGTGGAAAGATACCTAAGGGATCAACAGCTCCTGGGGATTTGGGGATGCTCTGGAAAACTCATTTGCACCACTACTGTGCCTTGGAATGTTAGTTGGAGT---------------------------AATAAATCCATGAATGACATTTGGAAT---AACATGACCTGGATGGAGTGGGAAAGAGAGATTGAC------AATTATACAAACATAATATACACCTTACTTGAAGAATCGCAGAACCAACAAGATAAGAATGAACAGGAATTATTGGAATTGGATAAATGGGCAAGTTTGTGGAATTGGTTTAGCATAACAAATTGGCTGTGGTACATAAAAATATTCATAATGATAGTAGGAGGCTTAATAGGTTTAAGAATAGTTTTTACTGTATTTTCTATAGTGAATAGAGTTAGGCAGGGATATTCACCATTATCGTTTCAGACCCGC---CTCCCAACTTCGAGGGGA------CTCGACAGGCCCGAAGGAATCGAAGAAGAAGGTGGAGACAGAGACAGAGACAGATCCAGGCCATTAGTGGATGGATTCTTAGCAATTATCTGGGTCGACCTGCGGAGCCTGTGCCTCTTCAGCTACCATCGCTTGAGAGACTTACTCTTGATTGTAGCGAGGATTGTGGAACTTCTGGGACGCAGG---------------GGGTGGGAAGCCCTCAAATATTGGTGG---AATCTCCTGCGGTATTGG---------------------------------------------------AGCCAGGAACTAAGGAATAGTGCTATTAGCTTGCTTAATGCCACAGCCATAGCAGTAGCTGAGGGAACAGATAGGGTGTTAGAAGTATTACAAAGA------------------GCTTTTAGAGCTGTTATACACATACCTAGAAGAATAAGACAGGGCTTAGAAAGGTTTTTGCTATAA

2.04013240.ADARC.GU330421 ATGAGAGTGAAGGGGATCATGAGGAATTATCAGCACTTA---------TGGAGATGGGGC------------------------ATGATGCTCCTTGGGATAATCATGATC------------TGTAGTGCTGCA---------GAACAATTGTGGGTCACAATCTATTATGGGGTACCTGTGTGGAAAGAAGCAACCACCACTCTATTTTGTGCATCAAATGCTAAAGCATATGATACAGAGGTACATAAT---GTCTGGGCCACACATGCCTGTGTACCCACAGACCCCAACCCACAAGAAGTAAGATTG---GAAAATGTGACAGAAAATTTTAACATGTGGAAAAATAATATGGTAGAACAGATGCATGAGGATATAATTAGCCTATGGGATCAAAGCCTAAAACCATGTGTGAAATTAACCCCACTCTGTGTTACTTTAAACTGCACTGATGCTAATACCACTAATACTAATGCCAAT------------------------------------------------------------------------------AGTACTAATAACAGTAGCTTGGGAACAATGGAGAAAGGAGAAATAAAAAACTGCTCTTTCAACATCACC---ACAAACCTAAGAGAT------AAGGTGCAGAAAGAATATGCACTTTTTTATAACCTTGATGTAGTGCCAATAAAGGGTGAGGAT---------------------------AATACTAGC---------------------------TATAGGTTGATAAGTTGTAATACCTCAGTCATTACACAGGCCTGTCCAAAGGTATCCTTTGAGCCAATTCCCATACATTATTGTACTCCAGCTGGTTTTGCGATTCTACAATGT---AATGATAAGAAATTCAATGGATCAGGACCATGTACAAATGTCAGCACAGTACAATGTACACATGGAATTAGGCCAGTAGTATCAACTCAACTGCTGTTAAATGGCAGTCTAGCAGAAAAA---GAGGTAGTAATTAGGTCTGAGAATTTCACAAATAATGCTAAAACCATAATAGTACAGCTAAATGAATCTGTAGTAATTAATTGTACAAGACCCAACAACAATACAAGAAAAAGTATACCTATAGGA------------CCAGGAAGA---GCATTTTATGCAACAGGAGAAATAATAGGAGATATAAGACAAGCACATTGTAACGTT------AGTACAAAAGCCTGGAAGGAAGCTTTACAACAGGTAGCTATAAAACTATCA---GAACAATTTGGG------------AATAAAACA---ATAGTCTTTAATCAA---------TCCTCAGGAGGAGACCCAGAAGTTGTAATGCACAGTTTTAATTGTAGAGGGGAATTTTTCTACTGTAATACAACAGGACTGTTTAATAATACTTGGGGGTTT---------AATAGTACTTGG---------------------GATGCTATTAATGTGCCAGAA------------------AATGACACA------------------------------ATCACACTCCCATGCAGAATAAAACAAATTGTAAACATGTGGCAGGAAGTAGGAAAAGCAATGTATGCCCCTCCCATCAGAGGACGACTTAATTGTTCATCAAATATTACAGGGCTGCTATTAACAAGAGATGGTGGT------------------AATACCACA---------------------AATAACACT---GAGGTCTTCAGACCTGGAGGAGGAGATATGAGAGACAATTGGAGA---AGTGAATTATATAAATATAAAGTAGTAAAAATTGAACCA---TTAGGAATAGCACCC---ACCAAGGCAAAGAGAAGAGTGGTGCAGAGAGAA---AAAAGAGCAGTG---GGA---ATAGGA---GCTTTG---TTCCTT---GGG---------TTCTTGGGA---GCAGCAGGAAGCACTATGGGCGCAGCGTCGATG---ACGCTGACGGTACAGGCCAGACTATTATTGTCTGGTATAGTGCAACAGCAGAACAATTTGCTGAGAGCTATTGAGGCGCAACAGCATCTGTTGCAACTCACAGTCTGGGGCATCAAGCAGCTCCAGGCAAGA---GTCCTGGCTGTGGAAAGATACCTAAGAGATCAACAGCTCCTGGGGATTTGGGGATGCTCTGGAAAACTCATTTGCACCACTACTGTGCCTTGGAATGTTAGTTGGAGT---------------------------AATAAATCCATGAATGACATTTGGAAT---AACATGACCTGGATGGAGTGGGAAAGAGAGATTGAC------AATTATACAAACATAATATACACCTTACTTGAAGAATCGCAGAACCAACAAGATAAGAATGAACAGGAATTATTGGAATTGGATAAATGGGCAAGTTTGTGGAATTGGTTTAGCATAACAAATTGGCTGTGGTACATAAAAATATTCATAATGATAGTAGGAGGCTTAATAGGTTTAAGAATAGTTTTTACTGTATTTTCTATAGTGAATAGAGTTAGGCAGGGATATTCACCATTATCGTTTCAGACCCGC---TTCCCAACCTCGAGGGGA------CTCGACAGGCCCGAAGGAATCGAAGAAGAAGGTGGAGACAGAGACAGAGACAGATCCAGGCCATTAGTGGATGGATTCTTAGCAATTATCTGGGTCGACCTGCGGAGCCTGTGCCTCTTCAGCTACCATCGCTTGAGAGACTTACTCTTGATTGTAGCGAGGATTGTGGAACTTCTGGGACGCAGG---------------GGGTGGGAAGCCCTCAAATATTGGTGG---AATCTCCTGCGGTATTGG---------------------------------------------------AGCCAGGAACTAAGGAATAGTGCTATTAGCTTGCTTAATGCCACAGCCATAGCAGTAGCTGAGGGAACAGATAGGGTGGTAGAAGTATTACAAAGA------------------GCTTTTAGAGCTGTTATACACATACCTAGAAGAATAAGACAGGGCTTAGAAAGGTTTTTGCTATAA

2.04013240.ADARC.GU330422 ATGAGAGTGAAGGGGATCATGAGGAATTATCAGCACTTA---------TGGAGATGGGGC------------------------ATGATGCTCCTTGGGATAATCATGATC------------TGTAGTGCTGCA---------GAACAATTGTGGGTCACAATCTATTATGGGGTACCTGTGTGGAAAGAAGCAACCACCACTCTATTTTGTGCATCAAATGCTAAAGCATATGATACAGAGGTACATAAT---GTCTGGGCCACACATGCCTGTGTACCCACAGACCCCAACCCACAAGAAGTAAGATTG---GAAAATGTGACAGAAAATTTTAACATGTGGAAAAATAATATGGTAGAACAGATGCATGAGGATATAATTAGCCTATGGGATCAAAGCCTAAAACCATGTGTGAAATTAACCCCACTCTGTGTTACTTTAAACTGCACTGATGCTAATACCACTAATACTAATGCCAAT------------------------------------------------------------------------------AGTACTAATAACAGTAGCTTGGGAACAATGGAGAAAGGAGAAATAAAAAACTGCTCTTTCAACATCACC---ACAAACCTAAGAGAT------AAGGTGCAGAAAGAATATGCACTTTTTTATAACCTTGATGTAGTGCCAATAAAGGGTGAGGAT---------------------------AATACTAGC---------------------------TATAGGTTGATAAGTTGTAATACCTCAGTCATTACACAGGCCTGTCCAAAGGTATCCTTTGAGCCAATTCCCATACATTATTGTACTCCAGCTGGTTTTGCGATTCTACAATGT---AATGATAAGAAATTCAATGGATCAGGACCATGTACAAATGTCAGCACAGTACAATGTACACATGGAATTAGGCCAGTAGTATCAACTCAACTGCTGTTAAATGGCAGTCTAGCAGAAAAA---GAGGTAGTAATTAGGTCTGAGAATTTCACAAATAATGCTAAAACCATAATAGTACAGCTAAATGAATCTGTAGTAATTAATTGTACAAGACCCAACAACAATACAAGAAAAAGTATACCTATAGGA------------CCAGGAAGA---GCATTTTATGCAACAGGAGAAATACTAGGAGATATAAGACAAGCACATTGTAACGTT------AGTACAAAAGCCTGGAAGGAAGCTTTACAACAGGTAGCTATAAAACTATCA---GAACAATTTGGG------------AATAAAACA---ATAGTCTTTAATCAA---------TCCTCAGGAGGAGACCCAGAAGTTGTAATGCACAGTTTTAATTGTAGAGGGGAATTTTTCTACTGTAAGACAACAGGACTGTTTAATAATACTTGGGGGTTT---------AATAGTACTTGG---------------------GATGCTATTAATGTGCCAGAA------------------AATGACACA------------------------------ATCACACTCCCATGCAGAATAAAACAAATTGTAAACATGTGGCAGGAAGTAGGAAAAGCAATGTATGCCCCTCCCATCAGAGGACGACTTAATTGTTCATCAAATATTACAGGGCTGCTATTAACAAGAGATGGTGGT------------------AATACCACA---------------------AATAACACT---GAGGTCTTCAGACCTGGAGGAGGAGATATGAGAGACAATTGGAGA---AGTGAATTATATAAATATAAAGTAGTAAAAATTGAACCA---TTAGGAATAGCACCC---ACCAAGGCAAAGAGAAGAGTGGTGCAGAGAGAA---AAAAGAGCAGTG---GGA---ATAGGA---GCTTTG---TTCCTT---GGG---------TTCTTGGGA---GCAGCAGGAAGCACTATGGGCGCAGCGTCGATG---ACGCTGACGGTACAGGCCAGACTATTATTGTCTGGTATAGTGCAACAGCAGAACAATTTGCTGAGAGCTATTGAGGCGCAACAGCATCTGTTGCAACTCACAGTCTGGGGCATCAAGCAGCTCCAGGCAAGA---GTCCTGGCTGTGGAAAGATACCTAAGAGATCAACAGCTCCTGGGGATTTGGGGATGCTCTGGAAAACTCATTTGCACCACTACTGTGCCTTGGAATGTTAGTTGGAGT---------------------------AATAAATCCATGAATGACATTTGGAAT---AACATGACCTGGATGGAGTGGGAAAGAGAGATTGAC------AATTATACAAACATAATATACACCTTACTTGAAGAATCGCAGAACCAACAAGATAAGAATGAACAGGAATTATTGGAATTGGATAAATGGGCAAGTTTGTGGAATTGGTTTAGCATAACAAATTGGCTGTGGTACATAAAAATATTCATAATGATAGTAGGAGGCTTAATAGGTTTAAGAATAGTTTTTACTGTATTTTCTATAGTGAATAGAGTTAGGCAGGGATATTCACCATTATCGTTTCAGACCCGC---TTCCCAACCTCGAGGGGA------CTCGACAGGCCCGAAGGAATCGAAGAAGAAGGTGGAGACAGAGACAGAGACAGATCCAGGCCATTAGTGGATGGATTCTTAGCAATTATCTGGGTCGACCTGCGGAGCCTGTGCCTCTTCAGCTACCATCGCTTGAGAGACTTACTCTTGATTGTAGCGAGGATTGTGGAACTTCTGGGACGCAGG---------------GGGTGGGAAGCCCTCAAATATTGGTGG---AATCTCCTGCGGTATTGG---------------------------------------------------AGCCAGGAACTAAGGAATAGTGCTATTAGCTTGCTTAATGCCACAGCCATAGCAGTAGCTGAGGGAACAGATAGGGTGGTAGAAGTATTACAAAGA------------------GCTTTTAGAGCTGTTATACACATACCTAGAAGAATAAGACAGGGCTTAGAAAGGTTTTTGCTATAA

2.04013240.ADARC.GU330423 ATGAGAGTGAAGGGGATCATGAGGAATTATCAGCACTTA---------TGGAGATGGGGC------------------------ATGATGCTCCTTGGGATAATCATGATC------------TGTAGTGCTGCA---------GAACAATTGTGGGTCACAATCTATTATGGGGTACCTGTGTGGAAAGAAGCAACCACCACTCTATTTTGTGCATCAAATGCTAAAGCATATGATACAGAGGTACATAAT---GTCTGGGCCACACATGCCTGTGTACCCACAGACCCCAACCCACAAGAAGTAAGATTG---GAAAATGTGACAGAAAATTTTAACATGTGGAAAAATAATATGGTAGAACAGATGCATGAGGATATAATTAGCCTATGGGATCAAAGCCTAAAACCATGTGTGAAATTAACCCCACTCTGTGTTACTTTAAACTGCACTGATGCTAATACCACTAATACTAATGCCAAT------------------------------------------------------------------------------AGTACTAATAACAGTAGCTTGGGAACAATGGAGAAAGGAGAAATAAAAAACTGCTCTTTCAACATCACC---ACAAACCTAAGAGAT------AAGGTGCAGAAAGAATATGCACTTTTTTATAACCTTGATGTAGTGCCAATAAAGGGTGAGGAT---------------------------AATACTAGC---------------------------TATAGGTTGATAAGTTGTAATACCTCAGTCATTACACAGGCCTGTCCAAAGGTATCCTTTGAGCCAATTCCCATACATTATTGTACTCCAGCTGGTTTTGCGATTCTACAATGT---AATGATAAGAAATTCAATGGATCAGGACCATGTACAAATGTCAGCACAGTACAATGTACACATGGAATTAGGCCAGTAGTATCAACTCAACTGCTGTTAAATGGCAGTCTAGCAGAAAAA---GAGGTAGTAATTAGGTCTGAGAATTTCACAAATAATGCTAAAACCATAATAGTACAGCTAAATGAATCTGTAGTAATTAATTGTACAAGACCCAACAACAATACAAGAAAAAGTATACCTATAGGA------------CCAGGAAGA---GCATTTTATGCAACAGGAGAAATAATAGGAGATATAAGACAAGCACATTGTAACGTT------AGTACAAAAGCCTGGAAGGAAGCTTTACAACAGGTAGCTATAAAACTATCA---GAACAATTTGGG------------AATAAAACA---ATAGTCTTTAATCAA---------TCCTCAGGAGGAGACCCAGAAGTTGTAATGCACAGTTTTAATTGTAGAGGGGAATTTTTCTACTGTAATACAACAGGACTGTTTAATAATACTTGGGGGTTT---------AATAGTACTTGG---------------------GATGCTATTAATGTGCCAGAA------------------AATGACACA------------------------------ATCACACTCCCATGCAGAATAAAACAAATTGTAAACATGTGGCAGGAAGTAGGAAAAGCAATGTATGCCCCTCCCATCAGAGGACAAATTAATTGTTCATCAAATATTACAGGGCTGCTATTAACAAGAGATGGTGGT------------------AATACCACA---------------------AATAACACT---GAGGTCTTCAGACCTGGAGGAGGAGATATGAGAGACAATTGGAGA---AGTGAATTATATAAATATAAAGTAGTAAAAATTGAACCA---TTAGGAATAGCACCC---ACCAAGGCAAAGAGAAGAGTGGTGCAGAGAGAA---AAAAGAGCAGTG---GGA---ATAGGA---GCTTTG---TTCCTT---GGG---------TTCTTGGGA---GCAGCAGGAAGCACTATGGGCGCAGCGTCGATG---ACGCTGACGGTACAGGCCAGACTATTATTGTCTGGTATAGTGCAACAGCAGAACAATTTGCTGAGAGCTATTGAGGCGCAACAGCATCTGTTGCAACTCACAGTCTGGGGCATCAAGCAGCTCCAGGCAAGA---GTCCTGGCTGTGGAAAGATACCTAAGGGATCAACAGCTCCTGGGGATTTGGGGATGCTCTGGAAAACTCATTTGCACCACTGCTGTGCCTTGGAATGTTAGTTGGAGT---------------------------AATAAATCCATGAATGACATTTGGAAT---AACATGACCTGGATGGAGTGGGAAAGAGAGATTGAC------AATTATACAAACATAATATACACCTTACTTGAAGAATCGCAGAACCAACAAGATAAGAATGAACAGGAATTATTGGAATTGGATAAATGGGCAAGTTTGTGGAATTGGTTTAGCATAACAAATTGGCTGTGGTACATAAAAATATTCATAATGATAGTAGGAGGCTTAATAGGTTTAAGAATAGTTTTTACTGTATTTTCTATAGTGAATAGAGTTAGGCAGGGATATTCACCATTATCGTTTCAGACCCGC---TTCCCAACCTCGAGGGGA------CTCGACAGGCCCGAAGGAATCGAAGAAGAAGGTGGAGACAGAGACAGAGACAGATCCAGGCCATTAGTGGATGGATTCTTAGCAATTATCTGGGTCGACCTGCGGAGCCTGTGCCTCTTCAGCTACCATCGCTTGAGAGACTTACTCTTGATTGTAGCGAGGATTGTGGAACTTCTGGGACGCAGG---------------GGGTGGGAAGCCCTCAAATATTGGTGG---AATCTCCTGCGGTATTGG---------------------------------------------------AGCCAGGAACTAAGGAATAGTGCTATTAGCTTGCTTAATGCCACAGCCATAGCAGTAGCTGAGGGAACAGATAGGGTGTTAGAAGTATTACAAAGA------------------GCTTTTAGAGCTGTTATACACATACCTAGAAGAATAAGACAGGGCTTAGAAAGGTTTTTGCTATAA

2.04013240.ADARC.GU330424 ATGAGAGTGAAGGGGATCATGAGGAATTATCAGCACTTA---------TGGAGATGGGGC------------------------ATGATGCTCCTTGGGATAATCATGATC------------TGTAGTGCTGCA---------GAACAATTGTGGGTCACAATCTATTATGGGGTACCTGTGTGGAAAGAAGCAACCACCACTCTATTTTGTGCATCAAATGCTAAAGCATATGATACAGAGGTACATAAT---GTCTGGGCCACACATGCCTGTGTACCCACAGACCCCAACCCACAAGAAGTAAGATTG---GAAAATGTGACAGAAAATTTTAACATGTGGAAAAATAATATGGTAGAACAGATGCATGAGGATATAATTAGCCTATGGGATCAAAGCCTAAAACCATGTGTGAAATTAACCCCACTCTGTGTTACTTTAAACTGCACTGATGCTAATACCACTAATACTAATGCCAAT------------------------------------------------------------------------------AGTACTAATAACAGTAGCTTGGGAACAATGGAGAAAGGAGAAATAAAAAACTGCTCTTTCAACATCACC---ACAAACCTAAGAGAT------AAGGTGCAGAAAGAATATGCACTTTTTTATAACCTTGATGTAGTGCCAATAAAGGGTGAGGAT---------------------------AATACTAGC---------------------------TATAGGTTGATAAGTTGTAATACCTCAGTCATTACACAGGCCTGTCCAAAGGTATCCTTTGAGCCAATTCCCATACATTATTGTACTCCAGCTGGTTTTGCGATTCTACAATGT---AATGATAAGAAATTCAATGGATCAGGACCATGTACAAATGTCAGCACAGTACAATGTACACATGGAATTAGGCCAGTAGTATCAACTCAACTGCTGTTAAATGGCAGTCTAGCAGAAAAA---GAGGTAGTAATTAGGTCTGAGAATTTCACAAATAATGCTAAAACCATAATAGTACAGCTAAATGAATCTGTAGTAATTAATTGTACAAGACCCAACAACAATACAAGAAAAAGTATACCTATAGGA------------CCAGGAAGA---GCATTTTATGCAACAGGAGAAATAATAGGAGATATAAGACAAGCACATTGTAACGTT------AGTACAAAAGCCTGGAAGGAAGCTTTACAACAGGTAGCTATAAAACTATCA---GAACAATTTGGG------------AATAAAACA---ATAGTCTTTAATCAA---------TCCTCAGGAGGAGACCCAGAAGTTGTAATGCACAGTTTTAATTGTAGAGGGGAATTTTTCTACTGTAATACAACAGGACTGTTTAATAATACTTGGGGGTTT---------AATAGTACTTGG---------------------GATGCTATTAATGTGCCAGAA------------------AATGACACA------------------------------ATCACACTCCCATGCAGAATAAAACAAATTGTAAACATGTGGCAGGAAGTAGGAAAAGCAATGTATGCCCCTCCCATCAGAGGACGACTTAATTGTTCATCAAATATTACAGGGCTGCTATTAACAAGAGATGGTGGT------------------AATACCACA---------------------AATAACACT---GAGGTCTTCAGACCTGGAGGAGGAGATATGAGAGACAATTGGAGA---AGTGAATTATATAAATATAAAGTAGTAAAAATTGAACCA---TTAGGAATAGCACCC---ACCAAGGCAAAGAGAAGAGTGGTGCAGAGAGAA---AAAAGAGCAGTG---GGA---ATAGGA---GCTTTG---TTCCTT---GGG---------TTCTTGGGA---GCAGCAGGAAGCACTATGGGCGCAGCGTCGATG---ACGCTGACGGTACAGGCCAGACTATTATTGTCTGGTATAGTGCAACAGCAGAACAATTTGCTGAGAGCTATTGAGGCGCAACAGCATCTGTTGCAACTCACAGTCTGGGGCATCAAGCAGCTCCAGGCAAGA---GTCCTGGCTGTGGAAAGATACCTAAGAGATCAACAGCTCCTGGGGATTTGGGGATGCTCTGGAAAACTCATTTGCACCACTACTGTGCCTTGGAATGTTAGTTGGAGT---------------------------AATAAATCCATGAATGACATTTGGAAT---AACATGACCTGGATGGAGTGGGAAAGAGAGATTGAC------AATTATACAAACATAATATACACCTTACTTGAAGAATCGCAGAACCAACAAGATAAGAATGAACAGGAATTATTGGAATTGGATAAATGGGCAAGTTTGTGGAATTGGTTTAGCATAACAAATTGGCTGTGGTACATAAAAATATTCATAATGATAGTAGGAGGCTTAATAGGTTTAAGAATAGTTTTTACTGTATTTTCTATAGTGAATAGAGTTAGGCAGGGATATTCACCATTATCGTTTCAGACCCGC---TTCCCAACCTCGAGGGGA------CTCGACAGGCCCGAAGGAATCGAAGAAGAAGGTGGAGACAGAGACAGAGACAGATCCAGGCCATTAGTGGATGGATTCTTAGCAATTATCTGGGTCGACCTGCGGAGCCTGTGCCTCTTCAGCTACCATCGCTTGAGAGACTTACTCTTGATTGTAGCGAGGATTGTGGAACTTCTGGGACGCAGG---------------GGGTGGGAAGCCCTCAAATATTGGTGG---AATCTCCTGCGGTATTGG---------------------------------------------------AGCCAGGAACTAAGGAATAGTGCTATTAGCTTGCTTAATGCCACAGCCATAGCAGTAGCTGAGGGAACAGATAGGGTGTTAGAAGTATTACAAAGA------------------GCTTTTAGAGCTGTTATACACATACCTAGAAGAATAAGACAGGGCTTAGAAAGGTTTTTGCTATAA

2.04013240.ADARC.GU330425 ATGAGAGTGAAGGGGATCATGAGGAATTATCAGCACTTA---------TGGAGATGGGGC------------------------ATGATGCTCCTTGGGATAATCATGATC------------TGTAGTGCTGCA---------GAACAATTGTGGGTCACAATCTATTATGGGGTACCTGTGTGGAAAGAAGCAACCACCACTCTATTTTGTGCATCAAATGCTAAAGCATATGATACAGAGGTACATAAT---GTCTGGGCCACACATGCCTGTGTACCCACAGACCCCAACCCACAAGAAGTAAGATTG---GAAAATGTGACAGAAAATTTTAACATGTGGAAAAATAATATGGTAGAACAGATGCATGAGGATATAATTAGCCTATGGGATCAAAGCCTAAAACCATGTGTGAAATTAACCCCACTCTGTGTTACTTTAAACTGCACTGATGCTAATACCACTAATACTAATGCCAAT------------------------------------------------------------------------------AGTACTAATAACAGTAGCTTGGGAACAATGGAGAAAGGAGAAATAAAAAACTGCTCTTTCAACATCACC---ACAAACCTAAGAGAT------AAGGTGCAGAAAGAATATGCACTTTTTTATAACCTTGATGTAGTGCCAATAAAGGGTGAGGAT---------------------------AATACTAGC---------------------------TATAGGTTGATAAGTTGTAATACCTCAGTCATTACACAGGCCTGTCCAAAGGTATCCTTTGAGCCAATTCCCATACATTATTGTACTCCAGCTGGTTTTGCGATTCTACAATGT---AATGATAAGAAATTCAATGGATCAGGACCATGTACAAATGTCAGCACAGTACAATGTACACATGGAATTAGGCCAGTAGTATCAACTCAACTGCTGTTAAATGGCAGTCTAGCAGAAAAA---GAGGTAGTAATTAGGTCTGAGAATTTCACAAATAATGCTAAAACCATAATAGTACAGCTAAATGAATCTGTAGTAATTAATTGTACAAGACCCAACAACAATACAAGAAAAAGTATACCTATAGGA------------CCAGGAAGA---GCATTTTATGCAACAGGAGAAATAATAGGAGATATAAGACAAGCACATTGTAACGTT------AGTACAAAAGCCTGGAAGGAAGCTTTACAACAGGTAGCTATAAAACTATCA---GAACAATTTGGG------------AATAAAACA---ATAGTCTTTAATCAA---------TCCTCAGGAGGAGACCCAGAAGTTGTAATGCACAGTTTTAATTGTAGAGGGGAATTTTTCTACTGTAATACAACAGGACTGTTTAATAATACTTGGGGGTTT---------AATAGTACTTGG---------------------GATGCTATTAATGTGCCAGAA------------------AATGACACA------------------------------ATCACACTCCCATGCAGAATAAAACAAATTGTAAACATGTGGCAGGAAGTAGGAAAAGCAATGTATGCCCCTCCCATCAGAGGACGACTTAATTGTTCATCAAATATTACAGGGCTGCTATTAACAAGAGATGGTGGT------------------AATACCACA---------------------AATAACACT---GAGGTCTTCAGACCTGGAGGAGGAGATATGAGAGACAATTGGAGA---AGTGAATTATATAAATATAAAGTAGTAAAAATTGAACCA---TTAGGAATAGCACCC---ACCAAGGCAAAGAGAAGAGTGGTGCAGAGAGAA---AAAAGAGCAGTG---GGA---ATAGGA---GCTTTG---TTCCTT---GGG---------TTCTTGGGA---GCAGCAGGAAGCACTATGGGCGCAGCGTCGATG---ACGCTGACGGTACAGGCCAGACTATTATTGTCTGGTATAGTGCAACAGCAGAACAATTTGCTGAGAGCTATTGAGGCGCAACAGCATCTGTTGCAACTCACAGTCTGGGGCATCAAGCAGCTCCAGGCAAGA---GTCCTGGCTGTGGAAAGATACCTAAGAGATCAACAGCTCCTGGGGATTTGGGGATGCTCTGGAAAACTCATTTGCACCACTACTGTGCCTTGGAATGTTAGTTGGAGT---------------------------AATAAATCCATGAATGACATTTGGAAT---AACATGACCTGGATGGAGTGGGAAAGAGAGATTGAC------AATTATACAAACATAATATACACCTTACTTGAAGAATCGCAGAACCAACAAGATAAGAATGAACAGGAATTATTGGAATTGGATAAATGGGCAAGTTTGTGGAATTGGTTTAGCATAACAAATTGGCTGTGGTACATAAAAATATTCATAATGATAGTAGGAGGCTTAATAGGTTTAAGAATAGTTTTTACTGTATTTTCTATAGTGAATAGAGTTAGGCAGGGATATTCACCATTATCGTTTCAGACCCGC---TTCCCAACCTCGAGGGGA------CTCGACAGGCCCGAAGGAATCGAAGAAGAAGGTGGAGACAGAGACAGAGACAGATCCAGGCCATTAGTGGATGGATTCTTAGCAATTATCTGGGTCGACCTGCGGAGCCTGTGCCTCTTCAGCTACCATCGCTTGAGAGACTTACTCTTGATTGTAGCGAGGATTGTGGAACTTCTGGGACGCAGG---------------GGGTGGGAAGCCCTCAAATATTGGTGG---AATCTCCTGCGGTATTGG---------------------------------------------------AGCCAGGAACTAAGGAATAGTGCTATTAGCTTGCTTAATGCCACAGCCATAGCAGTAGCTGAGGGAACAGATAGGGTGTTAGAAGTATTACAAAGA------------------GCTTTTAGAGCTGTTATACACATACCTAGAAGAATAAGACAGGGCTTAGAAAGGTTTTTGCTATAA

2.04013240.ADARC.GU330426 ATGAGAGTGAAGGGGATCATGAGGAATTATCAGCACTTA---------TGGAGATGGGGC------------------------ATGATGCTCCTTGGGATAATCATGATC------------TGTAGTGCTGCA---------GAACAATTGTGGGTCACAATCTATTATGGGGTACCTGTGTGGAAAGAAGCAAACACCACTCTATTTTGTGCATCAAATGCTAAAGCATATGATACAGAGGTACATAAT---GTCTGGGCCACACATGCCTGTGTACCCACAGACCCCAACCCACAAGAAGTAAGATTG---GAAAATGTGACAGAAAATTTTAACATGTGGAAAAATAATATGGTAGAACAGATGCATGAGGATATAATTAGCCTATGGGATCAAAGCCTAAAACCATGTGTGAAATTAACCCCACTCTGTGTTACTTTAAACTGCACTGATGCTAATACCACTAATACTAATGCCAAT------------------------------------------------------------------------------AGTACTAATAACAGTAGCTTGGGAACAATGGAGAAAGGAGAAATAAAAAACTGCTCTTTCAACATCACC---ACAAACCTAAGAGAT------AAGGTGCAGAAAGAATATGCACTTTTTTATAACCTTGATGTAGTGCCAATAAAGGGTGAGGAT---------------------------AATACTAGC---------------------------TATAGGTTGATAAGTTGTAATACCTCAGTCATTACACAGGCCTGTCCAAAGGTATCCTTTGAGCCAATTCCCATACATTATTGTACTCCAGCTGGTTTTGCGATTCTACAATGT---AATGATAAGAAATTCAATGGATCAGGACCATGTACAAATGTCAGCACAGTACAATGTACACATGGAATTAGGCCAGTAGTATCAACTCAACTGCTGTTAAATGGCAGTCTAGCAGAAAAA---GAGGTAGTAATTAGGTCTGAGAATTTCACAAATAATGCTAAAACCATAATAGTACAGCTAAATGAATCTGTAGTAATTAATTGTACAAGACCCAACAACAATACAAGAAAAAGTATACCTATAGGA------------CCAGGAAGA---GCATTTTATGCAACAGGAGAAATAATAGGAGATATAAGACAAGCACATTGTAACGTT------AGTACAAAAGCCTGGAAGGAAGCTTTACAACAGGTAGCTATAAAACTATCA---GAACAATTTGGG------------AATAAAACA---ATAGTCTTTAATCAA---------TCCTCAGGAGGAGACCCAGAAGTTGTAATGCACAGTTTTAATTGTAGAGGGGAATTTTTCTACTGTAATACAACAGGACTGTTTAATAATACTTGGGGGTTT---------AATAGTACTTGG---------------------GATGCTATTAATGTGCCAGAA------------------AATGACACA------------------------------ATCACACTCCCATGCAGAATAAAACAAATTGTAAACATGTGGCAGGAAGTAGGAAAAGCAATGTATGCCCCTCCCATCAGAGGACAACTTAATTGTTCATCAAATATTACAGGGCTGCTATTAACAAGAGATGGTGGT------------------AATACCACA---------------------AATAACACT---GAGGTCTTCAGACCTGGAGGAGGAGATATGAGAGACAATTGGAGA---AGTGAATTATATAAATATAAAGTAGTAAAAATTGAACCA---TTAGGAATAGCACCC---ACCAAGGCAAAGAGAAGAGTGGTGCAGAGAGAA---AAAAGAGCAGTG---GGA---ATAGGA---GCTTTG---TTCCTT---GGG---------TTCTTGGGA---GCAGCAGGAAGCACTATGGGCGCAGCGTCGATG---ACGCTGACGGTACAGGCCAGACTATTATTGTCTGGTATAGTGCAACAGCAGAACAATTTGCTGAGAGCTATTGAGGCGCAACAGCATCTGTTGCAACTCACAGTCTGGGGCATCAAGCAGCTCCAGGCAAGA---GTCCTGGCTGTGGAAAGATACCTAAGAGATCAACAGCTCCTGGGGATTTGGGGATGCTCTGGAAAACTCATTTGCACCACTGCTGTGCCTTGGAATGTTAGTTGGAGT---------------------------AATAAATCCATGAATGACATTTGGAAT---AACATGACCTGGATGGAGTGGGAAAGAGAGATTGAC------AATTATACAAACATAATATACACCTTACTTGAAGAATCGCAGAACCAACAAGATAAGAATGAACAGGAATTATTGGAATTGGATAAATGGGCAAGTTTGTGGAATTGGTTTAGCATAACAAATTGGCTGTGGTACATAAAAATATTCATAATGATAGTAGGAGGCTTAATAGGTTTAAGAATAGTTTTTACTGTATTTTCTATAGTGAATAGAGTTAGGCAGGGATATTCACCATTATCGTTTCAGACCCGC---TTCCCAACCTCGAGGGGA------CTCGACAGGCCCGAAGGAATCGAAGAAGAAGGTGGAGACAGAGACAGAGACAGATCCAGGCCATTAGTGGATGGATTCTTAGCAATTATCTGGGTCGACCTGCGGAGCCTGTGCCTCTTCAGCTACCATCGCTTGAGAGACTTACTCTTGATTGTAGCGAGGATTGTGGAACTTCTGGGACGCAGG---------------GGGTGGGAAGCCCTCAAATATTGGTGG---AATCTCCTGCGGTATTGG---------------------------------------------------AGCCAGGAACTAAGGAATAGTGCTATTAGCTTGCTTAATGCCACAGCCATAGCAGTAGCTGAGGGAACAGATAGGGCGTTAGAAGTATTACAAAGA------------------GCTTTTAGAGCTGTTATACACATACCTAGAAGAATAAGACAGGGCTTAGAAAGGTTTTTGCTATAA

2.04013240.ADARC.GU330427 ATGAGAGTGAAGGGGATCATGAGGAATTATCAGCACTTA---------TGGAGATGGGGC------------------------ATGATGCTCCTTGGGATAATCATGATC------------TGTAGTGCTGCA---------GAACAATTGTGGGTCACAATCTATTATGGGGTACCTGTGTGGAAAGAAGCAACCACCACTCTATTTTGTGCATCAAATGCTAAAGCATATGATACAGAGGTACATAAT---GTCTGGGCCACACATGCCTGTGTACCCACAGACCCCAACCCACAAGAAGTAAGATTG---GAAAATGTGACAGAAAATTTTAACATGTGGAAAAATAATATGGTAGAACAGATGCATGAGGATATAATTAGCCTATGGGATCAAAGCCTAAAACCATGTGTGAAATTAACCCCACTCTGTGTTACTTTAAACTGCACTGATGCTAATACCACTAATACTAATGCCAAT------------------------------------------------------------------------------AGTACTAATAACAGTAGCTTGGGAACAATGGAGAAAGGAGAAATAAAAAACTGCTCTTTCAACATCACC---ACAAACCTAAGAGAT------AAGGTGCAGAAAGAATATGCACTTTTTTATAACCTTGATGTAGTGCCAATAAAGGGTGAGGAT---------------------------AATACTAGC---------------------------TATAGGTTGATAAGTTGTAATACCTCAGTCATTACACAGGCCTGTCCAAAGGTATCCTTTGAGCCAATTCCCATACATTATTGTACTCCAGCTGGTTTTGCGATTCTACAATGT---AATGATAAGAAATTCAATGGATCAGGACCATGTACAAATGTCAGCACAGTACAATGTACACATGGAATTAGGCCAGTAGTATCAACTCAACTGCTGTTAAATGGCAGTCTAGCAGAAAAA---GAGGTAGTAATTAGGTCTGAGAATTTCACAAATAATGCTAAAACCATAATAGTACAGCTAAATGAATCTGTAGTAATTAATTGTACAAGACCCAACAACAATACAAGAAAAAGTATACCTATAGGA------------CCAGGAAGA---GCATTTTATGCAACAGGAGAAATAATAGGAGATATAAGACAAGCACATTGTAACGTT------AGTACAAAAGCCTGGAAGGAAGCTTTACAACAGGTAGCTATAAAACTATCA---GAACAATTTGGG------------AATAAAACA---ATAGTCTTTAATCAA---------TCCTCAGGAGGAGACCCAGAAGTTGTAATGCACAGTTTTAATTGTAGAGGGGAATTTTTCTACTGTAATACAACAGGACTGTTTAATAATACTTGGGGGTTT---------AATAGTACTTGG---------------------GATGCTATTAATGTGCCAGAA------------------AATGACACA------------------------------ATCACACTCCCATGCAGAATAAAACAAATTGTAAACATGTGGCAGGAAGTAGGAAAAGCAATGTATGCCCCTCCCATCAGAGGACGACTTAATTGTTCATCAAATATTACAGGGCTGCTATTAACAAGAGATGGTGGT------------------AATACCACA---------------------AATAACACT---GAGGTCTTCAGACCTGGAGGAGGAGATATGAGAGACAATTGGAGA---AGTGAATTATATAAATATAAAGTAGTAAAAATTGAACCA---TTAGGAATAGCACCC---ACCAAGGCAAAGAGAAGAGTGGTGCAGAGAGAA---AAAAGAGCAGTG---GGA---ATAGGA---GCTTTG---TTCCTT---GGG---------TTCTTGGGA---GCAGCAGGAAGCACTATGGGCGCAGCGTCGATG---ACGCTGACGGTACAGGCCAGACTATTATTGTCTGGTATAGTGCAACAGCAGAACAATTTGCTGAGAGCTATTGAGGCGCAACAGCATCTGTTGCAACTCACAGTCTGGGGCATCAAGCAGCTCCAGGCAAGA---GTCCTGGCTGTGGAAAGATACCTAAGGGATCAACAGCTCCTGGGGATTTGGGGATGCTCTGGAAAACTCATTTGCACCACTGCTGTGCCTTGGAATGTTAGTTGGAGT---------------------------AATAAATCCATGAATGACATTTGGAAT---AACATGACCTGGATGGAGTGGGAAAGAGAGATTGAC------AATTATACAAACATAATATACACCTTACTTGAAGAATCGCAGAACCAACAAGATAAGAATGAACAGGAATTATTGGAATTGGATAAATGGGCAAGTTTGTGGAATTGGTTTAGCATAACAAATTGGCTGTGGTACATAAAAATATTCATAATGATAGTAGGAGGCTTAATAGGTTTAAGAATAGTTTTTACTGTATTTTCTATAGTGAATAGAGTTAGGCAGGGATATTCACCATTATCGTTTCAGACCCGC---TTCCCAACCTCGAGGGGA------CTCGACAGGCCCGAAGGAATCGAAGAAGAAGGTGGAGACAGAGACAGAGACAGATCCAGGCCATTAGTGGATGGATTCTTAGCAATTATCTGGGTCGACCTGCGGAGCCTGTGCCTCTTCAGCTACCATCGCTTGAGAGACTTACTCTTGATTGTAGCGAGGATTGTGGAACTTCTGGGACGCAGG---------------GGGTGGGAAGCCCTCAAATATTGGTGG---AATCTCCTGCGGTATTGG---------------------------------------------------AGCCAGGAACTAAGGAATAGTGCTATTAGCTTGCTTAATGCCACAGCCATAGCAGTAGCTGAGGGAACAGATAGGGTGGTAGAAGTATTACAAAGA------------------GCTTTTAGAGCTGTTATACACATACCTAGAAGAATAAGACAGGGCTTAGAAAGGTTTTTGCTATAA

2.04013240.ADARC.GU330428 ATGAGAGTGAAGGGGATCATGAGGAATTATCAGCACTTA---------TGGAGATGGGGC------------------------ATGATGCTCCTTGGGATAATCATGATC------------TGTAGTGCTGCA---------GAACAATTGTGGGTCACAATCTATTATGGGGTACCTGTGTGGAAAGAAGCAACCACCACTCTATTTTGTGCATCAAATGCTAAAGCATATGATACAGAGGTACATAAT---GTCTGGGCCACACATGCCTGTGTACCCACAGACCCCAACCCACAAGAAGTAAGATTG---GAAAATGTGACAGAAAATTTTAACATGTGGAAAAATAATATGGTAGAACAGATGCATGAGGATATAATTAGCCTATGGGATCAAAGCCTAAAACCATGTGTGAAATTAACCCCACTCTGTGTTACTTTAAACTGCACTGATGCTAATACCACTAATACTAATGCCAAT------------------------------------------------------------------------------AGTACTAATAACAGTAGCTTGGGAACAATGGAGAAAGGAGAAATAAAAAACTGCTCTTTCAACATCACC---ACAAACCTAAGAGAT------AAGGTGCAGAAAGAATATGCACTTTTTTATAACCTTGATGTAGTGCCAATAAAGGGTGAGGAT---------------------------AATACTAGC---------------------------TATAGGTTGATAAGTTGTAATACCTCAGTCATTACACAGGCCTGTCCAAAGGTATCCTTTGAGCCAATTCCCATACATTATTGTACTCCAGCTGGTTTTGCGATTCTACAATGT---AATGATAAGAAATTCAATGGATCAGGACCATGTACAAATGTCAGCACAGTACAATGTACACATGGAATTAGGCCAGTAGTATCAACTCAACTGCTGTTAAATGGCAGTCTAGCAGAAAAA---GAGGTAGTAATTAGGTCTGAGAATTTCACAAATAATGCTAAAACCATAATAGTACAGCTAAATGAATCTGTAGTAATTAATTGTACAAGACCCAACAACAATACAAGAAAAAGTATACCTATAGGA------------CCAGGAAGA---GCATTTTATGCAACAGGAGAAATAATAGGAGATATAAGACAAGCACATTGTAACGTT------AGTACAAAAGCCTGGAAGGAAGCTTTACAACAGGTAGCTATAAAACTATCA---GAACAATTTGGG------------AATAAAACA---ATAGTCTTTAATCAA---------TCCTCAGGAGGAGACCCAGAAGTTGTAATGCACAGTTTTAATTGTAGAGGGGAATTTTTCTACTGTAATACAACAGGACTGTTTAATAATACTTGGGGGTTT---------AATAGTACTTGG---------------------GATGCTATTAATGTGCCAGAA------------------AATGACACA------------------------------ATCACACTCCCATGCAGAATAAAACAAATTGTAAACATGTGGCAGGAAGTAGGAAAAGCAATGTATGCCCCTCCCATCAGAGGACGACTTAATTGTTCATCAAATATTACAGGGCTGCTATTAACAAGAGATGGTGGT------------------AATACCACA---------------------AATAACACT---GAGGTCTTCAGACCTGGAGGAGGAGATATGAGAGACAATTGGAGA---AGTGAATTATATAAATATAAAGTAGTAAAAATTGAACCA---TTAGGAATAGCACCC---ACCAAGGCAAAGAGAAGAGTGGTGCAGAGAGAA---AAAAGAGCAGTG---GGA---ATAGGA---GCTTTG---TTCCTT---GGG---------TTCTTGGGA---GCAGCAGGAAGCACTATGGGCGCAGCGTCGATG---ACGCTGACGGTACAGGCCAGACTATTATTGTCTGGTATAGTGCAACAGCAGAACAATTTGCTGAGAGCTATTGAGGCGCAACAGCATCTGTTGCAACTCACAGTCTGGGGCATCAAGCAGCTCCAGGCAAGA---GTCCTGGCTGTGGAAAGATACCTAAGAGATCAACAGCTCCTGGGGATTTGGGGATGCTCTGGAAAACTCATTTGCACCACTACTGTGCCTTGGAATGTTAGTTGGAGT---------------------------AATAAATCCATGAATGACATTTGGAAT---AACATGACCTGGATGGAGTGGGAAAGAGAGATTGAC------AATTATACAAACATAATATACACCTTACTTGAAGAATCGCAGAACCAACAAGATAAGAATGAACAGGAATTATTGGAATTGGATAAATGGGCAAGTTTGTGGAATTGGTTTAGCATAACAAATTGGCTGTGGTACATAAAAATATTCATAATGATAGTAGGAGGCTTAATAGGTTTAAGAATAGTTTTTACTGTATTTTCTATAGTGAATAGAGTTAGGCAGGGATATTCACCATTATCGTTTCAGACCCGC---TTCCCAACCTCGAGGGGA------CTCGACAGGCCCGAAGGAATCGAAGAAGAAGGTGGAGACAGAGACAGAGACAGATCCAGGCCATTAGTGGATGGATTCTTAGCAATTATCTGGGTCGACCTGCGGAGCCTGTGCCTCTTCAGCTACCATCGCTTGAGAGACTTACTCTTGATTGTAGCGAGGATTGTGGAACTTCTGGGACGCAGG---------------GGGTGGGAAGCCCTCAAATATTGGTGG---AATCTCCTGCGGTATTGG---------------------------------------------------AGCCAGGAACTAAGGAATAGTGCTATTAGCTTGCTTAATGCCACAGCCATAGCAGTAGCTGAGGGAACAGATAGGGTGTTAGAAGTATTACAAAGA------------------GCTTTTAGAGCTGTTATACACATACCTAGAAGAATAAGACAGGGCTTAGAAAGGTTTTTGCTATAA

2.04013240.ADARC.GU330429 ATGAGAGTGAAGGGGATCATGAGGAATTATCAGCACTTA---------TGGAGATGGGGC------------------------ATGATGCTCCTTGGGATAATCATGATC------------TGTAGTGCTGCA---------GAACAATTGTGGGTCACAATCTATTATGGGGTACCTGTGTGGAAAGAAGCAACCACCACTCTATTTTGTGCATCAAATGCTAAAGCATATGATACAGAGGTACATAAT---GTCTGGGCCACACATGCCTGTGTACCCACAGACCCCAACCCACAAGAAGTAAGATTG---GAAAATGTGACAGAAAATTTTAACATGTGGAAAAATAATATGGTAGAACAGATGCATGAGGATATAATTAGCCTATGGGATCAAAGCCTAAAACCATGTGTGAAATTAACCCCACTCTGTGTTACTTTAAACTGCACTGATGCTAATACCACTAATACTAATGCCAAT------------------------------------------------------------------------------AGTACTAATAACAGTAGCTTGGGAACAATGGAGAAAGGAGAAATAAAAAACTGCTCTTTCAACATCACC---ACAAACCTAAGAGAT------AAGGTGCAGAAAGAATATGCACTTTTTTATAACCTTGATGTAGTGCCAATAAAGGGTGAGGAT---------------------------AATACTAGC---------------------------TATAGGTTGATAAGTTGTAATACCTCAGTCATTACACAGGCCTGTCCAAAGGTATCCTTTGAGCCAATTCCCATACATTATTGTACTCCAGCTGGTTTTGCGATTCTACAATGT---AATGATAAGAAATTCAATGGATCAGGACCATGTACAAATGTCAGCACAGTACAATGTACACATGGAATTAGGCCAGTAGTATCAACTCAACTGCTGTTAAATGGCAGTCTAGCAGAAAAA---GAGGTAGTAATTAGGTCTGAGAATTTCACAAATAATGCTAAAACCATAATAGTACAGCTAAATGAATCTGTAGTAATTAATTGTACAAGACCCAACAACAATACAAGAAAAAGTATACCTATAGGA------------CCAGGAAGA---GCATTTTATGCAACAGGAGAAATAATAGGAGATATAAGACAAGCACATTGTAACGTT------AGTACAAAAGCCTGGAAGGAAGCTTTACAACAGGTAGCTATAAAACTATCA---GAACAATTTGGG------------AATAAAACA---ATAGTCTTTAATCAA---------TCCTCAGGAGGAGACCCAGAAGTTGTAATGCACAGTTTTAATTGTAGAGGGGAATTTTTCTACTGTAATACAACAGGACTGTTTAATAATACTTGGGGGTTT---------AATAGTACTTGG---------------------GATGCTATTAATGTGCCAGAA------------------AATGACACA------------------------------ATCACACTCCCATGCAGAATAAAACAAATTGTAAACATGTGGCAGGAAGTAGGAAAAGCAATGTATGCCCCTCCCATCAGAGGACGACTTAATTGTTCATCAAATATTACAGGGCTGCTATTAACAAGAGATGGTGGT------------------AATACCACA---------------------AATAACACT---GAGGTCTTCAGACCTGGAGGAGGAGATATGAGAGACAATTGGAGA---AGTGAATTATATAAATATAAAGTAGTAAAAATTGAACCA---TTAGGAATAGCACCC---ACCAAGGCAAAGAGAAGAGTGGTGCAGAGAGAA---AAAAGAGCAGTG---GGA---ATAGGA---GCTTTG---TTCCTT---GGG---------TTCTTGGGA---GCAGCAGGAAGCACTATGGGCGCAGCGTCGATG---ACGCTGACGGTACAGGCCAGACTATTATTGTCTGGTATAGTGCAACAGCAGAACAATTTGCTGAGAGCTATTGAGGCGCAACAGCATCTGTTGCAACTCACAGTCTGGGGCATCAAGCAGCTCCAGGCAAGA---GTCCTGGCTGTGGAAAGATACCTAAGAGATCAACAGCTCCTGGGGATTTGGGGATGCTCTGGAAAACTCATTTGCACCACTACTGTGCCTTGGAATGTTAGTTGGAGT---------------------------AATAAATCCATGAATGACATTTGGAAT---AACATGACCTGGATGGAGTGGGAAAGAGAGATTGAC------AATTATACAAACATAATATACACCTTACTTGAAGAATCGCAGAACCAACAAGATAAGAATGAACAGGAATTATTGGAATTGGATAAATGGGCAAGTTTGTGGAATTGGTTTAGCATAACAAATTGGCTGTGGTACATAAAAATATTCATAATGATAGTAGGAGGCTTAATAGGTTTAAGAATAGTTTTTACTGTATTTTCTATAGTGAATAGAGTTAGGCAGGGATATTCACCATTATCGTTTCAGACCCGC---TTCCCAACCTCGAGGGGA------CTCGACAGGCCCGAAGGAATCGAAGAAGAAGGTGGAGACAGAGACAGAGACAGATCCAGGCCATTAGTGGATGGATTCTTAGCAATTATCTGGGTCGACCTGCGGAGCCTGTGCCTCTTCAGCTACCATCGCTTGAGAGACTTACTCTTGATTGTAGCGAGGATTGTGGAACTTCTGGGACGCAGG---------------GGGTGGGAAGCCCTCAAATATTGGTGG---AATCTCCTGCGGTATTGG---------------------------------------------------AGCCAGGAACTAAGGAATAGTGCTATTAGCTTGCTTAATGCCACAGCCATAGCAGTAGCTGAGGGAACAGATAGGGTGTTAGAAGTATTACAAAGA------------------GCTTTTAGAGCTGTTATACACATACCTAGAAGAATAAGACAGGGCTTAGAAAGGTTTTTGCTATAA

2.04013240.ADARC.GU330430 ATGAGAGTGAAGGGGATCATGAGGAATTATCAGCACTTA---------TGGAGATGGGGC------------------------ATGATGCTCCTTGGGATAATCATGATC------------TGTAGTGCTGCA---------GAACAATTGTGGGTCACAATCTATTATGGGGTACCTGTGTGGAAAGAAGCAACCACCACTCTATTTTGTGCATCAAATGCTAAAGCATATGATACAGAGGTACATAAT---GTCTGGGCCACACATGCCTGTGTACCCACAGACCCCAACCCACAAGAAGTAAGATTG---GAAAATGTGACAGAAAATTTTAACATGTGGAAAAATAATATGGTAGAACAGATGCATGAGGATATAATTAGCCTATGGGATCAAAGCCTAAAACCATGTGTGAAATTAACCCCACTCTGTGTTACTTTAAACTGCACTGATGCTAATACCACTAATACTAATGCCAAT------------------------------------------------------------------------------AGTACTAATAACAGTAGCTTGGGAACAATGGAGAAAGGAGAAATAAAAAACTGCTCTTTCAACATCACC---ACAAACCTAAGAGAT------AAGGTGCAGAAAGAATATGCACTTTTTTATAACCTTGATGTAGTGCCAATAAAGGGTGAGGAT---------------------------AATACTAGC---------------------------TATAGGTTGATAAGTTGTAATACCTCAGTCATTACACAGGCCTGTCCAAAGGTATCCTTTGAGCCAATTCCCATACATTATTGTACTCCAGCTGGTTTTGCGATTCTACAATGT---AATGATAAGAAATTCAATGGATCAGGACCATGTACAAATGTCAGCACAGTACAATGTACACATGGAATTAGGCCAGTAGTATCAACTCAACTGCTGTTAAATGGCAGTCTAGCAGAAAAA---GAGGTAGTAATTAGGTCTGAGAATTTCACAAATAATGCTAAAACCATAATAGTACAGCTAAATGAATCTGTAGTAATTAATTGTACAAGACCCAACAACAATACAAGAAAAAGTATACCTATAGGA------------CCAGGAAGA---GCATTTTATGCAACAGGAGAAATAATAGGAGATATAAGACAAGCACATTGTAACGTT------AGTACAAAAGCCTGGAAGGAAGCTTTACAACAGGTAGCTATAAAACTATCA---GAACAATTTGGG------------AATAAAACA---ATAGTCTTTAATCAA---------TCCTCAGGAGGAGACCCAGAAGTTGTAATGCACAGTTTTAATTGTAGAGGGGAATTTTTCTACTGTAATACAACAGGACTGTTTAATAATACTTGGGGGTTT---------AATAGTACTTGG---------------------GATGCTATTAATGTGCCAGAA------------------AATGACACA------------------------------ATCACACTCCCATGCAGAATAAAACAAATTGTAAACATGTGGCAGGAAGTAGGAAAAGCAATGTATGCCCCTCCCATCAGAGGACGACTTAATTGTTCATCAAATATTACAGGGCTGCTATTAACAAGAGATGGTGGT------------------AATACCACA---------------------AATAACACT---GAGGTCTTCAGACCTGGAGGAGGAGATATGAGAGACAATTGGAGA---AGTGAATTATATAAATATAAAGTAGTAAAAATTGAACCA---TTAGGAATAGCACCC---ACCAAGGCAAAGAGAAGAGTGGTGCAGAGAGAA---AAAAGAGCAGTG---GGA---ATAGGA---GCTTTG---TTCCTT---GGG---------TTCTTGGGA---GCAGCAGGAAGCACTATGGGCGCAGCGTCGATG---ACGCTGACGGTACAGGCCAGACTATTATTGTCTGGTATAGTGCAACAGCAGAACAATTTGCTGAGAGCTATTGAGGCGCAACAGCATCTGTTGCAACTCACAGTCTGGGGCATCAAGCAGCTCCAGGCAAGA---GTCCTGGCTGTGGAAAGATACCTAAGAGATCAACAGCTCCTGGGGATTTGGGGATGCTCTGGAAAACTCATTTGCACCACTACTGTGCCTTGGAATGTTAGTTGGAGT---------------------------AATAAATCCATGAATGACATTTGGAAT---AACATGACCTGGATGGAGTGGGAAAGAGAGATTGAC------AATTATACAAACATAATATACACCTTACTTGAAGAATCGCAGAACCAACAAGATAAGAATGAACAGGAATTATTGGAATTGGATAAATGGGCAAGTTTGTGGAATTGGTTTAGCATAACAAATTGGCTGTGGTACATAAAAATATTCATAATGATAGTAGGAGGCTTAATAGGTTTAAGAATAGTTTTTACTGTATTTTCTATAGTGAATAGAGTTAGGCAGGGATATTCACCATTATCGTTTCAGACCCGC---TTCCCAACCTCGAGGGGA------CTCGACAGGCCCGAAGGAATCGAAGAAGAAGGTGGAGACAGAGACAGAGACAGATCCAGGCCATTAGTGGATGGATTCTTAGCAATTATCTGGGTCGACCTGCGGAGCCTGTGCCTCTTCAGCTACCATCGCTTGAGAGACTTACTCTTGATTGTAGCGAGGATTGTGGAACTTCTGGGACGCAGG---------------GGGTGGGAAGCCCTCAAATATTGGTGG---AATCTCCTGCGGTATTGG---------------------------------------------------AGCCAGGAACTAAGGAATAGTGCTATTAGCTTGCTTAATGCCACAGCCATAGCAGTAGCTGAGGGAACAGATAGGGTGTTAGAAGTATTACAAAGA------------------GCTTTTAGAGCTGTTATACACATACCTAGAAGAATAAGACAGGGCTTAGAAAGGTTTTTGCTATAA

2.04013240.ADARC.GU330431 ATGAGAGTGAAGGGGATCATGAGGAATTATCAGCACTTA---------TGGAGATGGGGC------------------------ATGATGCTCCTTGGGATAATCATGATC------------TGTAGTGCTGCA---------GAACAATTGTGGGTCACAATCTATTATGGGGTACCTGTGTGGAAAGAAGCAACCACCACTCTATTTTGTGCATCAAATGCTAAAGCATATGATACAGAGGTACATAAT---GTCTGGGCCACACATGCCTGTGTACCCACAGACCCCAACCCACAAGAAGTAAGATTG---GAAAATGTGACAGAAAATTTTAACATGTGGAAAAATAATATAGTAGAACAGATGCATGAGGATATAATTAGCCTATGGGATCAAAGCCTAAAACCATGTGTGAAATTAACCCCACTCTGTGTTACTTTAAACTGCACTGATGCTAATACCACTAATACTAATGCCAAT------------------------------------------------------------------------------AGTACTAATAACAGTAGCTTGGGAACAATGGAGAAAGGAGAAATAAAAAACTGCTCTTTCAACATCACC---ACAAACCTAAGAGAT------AAGGTGCAGAAAGAATATGCACTTTTTTATAACCTTGATGTAGTGCCAATAAAGGGTGAGGAT---------------------------AATACTAGC---------------------------TATAGGTTGATAAGTTGTAATACCTCAGTCATTACACAGGCCTGTCCAAAGGTATCCTTTGAGCCAATTCCCATACATTATTGTACTCCAGCTGGTTTTGCGATTCTACAATGT---AATGATAAGAAATTCAATGGATCAGGACCATGTACAAATGTCAGCACAGTACAATGTACACATGGAATTAGGCCAGTAGTATCAACTCAACTGCTGTTAAATGGCAGTCTAGCAGAAAAA---GAGGTAGTAATTAGGTCTGAGAATTTCACAAATAATGCTAAAACCATAATAGTACAGCTAATTGAATCTGTAGTAATTAATTGTACAAGACCCAACAACAATACAAGAAAAAGTATACCTATAGGA------------CCAGGAAGA---GCATTTTATGCAACAGGAGAAATAATAGGAGATATAAGACAAGCACATTGTAACGTT------AGTACAAAAGCCTGGAAGGAAGCTTTACAACAGGTAGCTATAAAACTATCA---GAACAATTTGGG------------AATAAAACA---ATAGTCTTTAATCAA---------TCCTCAGGAGGAGACCCAGAAGTTGTAATGCACAGTTTTAATTGTAGAGGGGAATTTTTCTACTGTAATACAACAGGACTGTTTAATAATACTTGGGGGTTT---------AATAGTACTTGG---------------------GATGCTATTAATGTGCCAGAA------------------AATGACACA------------------------------ATCACACTCCCATGCAGAATAAAACAAATTGTAAACATGTGGCAGGAAGTAGGAAAAGCAATGTATGCCCCTCCCATCAGAGGACAAATTAATTGTTCATCAAATATTACAGGGCTGCTATTAACAAGAGATGGTGGT------------------AATACCACA---------------------AATAACACT---GAGGTCTTCAGACCTGGAGGAGGAGATATGAGAGACAATTGGAGA---AGTGAATTATATAAATATAAAGTAGTAAAAATTGAACCA---TTAGGAATAGCACCC---ACCAAGGCAAAGAGAAGAGTGGTGCAGAGAGAA---AAAAGAGCAGTG---GGA---ATAGGA---GCTTTG---TTCCTT---GGG---------TTCTTGGGA---GCAGCAGGAAGCACTATGGGCGCAGCGTCGATG---ACGCTGACGGTACAGGCCAGACTATTATTGTCTGGTATAGTGCAACAGCAGAACAATTTGCTGAGAGCTATTGAGGCGCAACAGCATCTGTTGCAACTCACAGTCTGGGGCATCAAGCAGCTCCAGGCAAGA---GTCCTGGCTGTGGAAAGATACCTAAGGGATCAACAGCTCCTGGGGATTTGGGGATGCTCTGGAAAACTCATTTGCACCACTGCTGTGCCTTGGAATGTTAGTTGGAGT---------------------------AATAAATCCATGAATGACATTTGGAAT---AACATGACCTGGATGGAGTGGGAAAGAGAGATTGAC------AATTATACAAACATAATATACACCTTACTTGAAGAATCGCAGAACCAACAAGATAAGAATGAACAGGAATTATTGGAATTGGATAAATGGGCAAGTTTGTGGAATTGGTTTAGCATAACAAATTGGCTGTGGTACATAAAAATATTCATAATGATAGTAGGAGGCTTAATAGGTTTAAGAATAGTTTTTACTGTATTTTCTATAGTGAATAGAGTTAGGCAGGGATATTCACCATTATCGTTTCAGACCCGC---CTCCCAACTTCGAGGGGA------CTCGACAGGCCCGAAGGAATCGAAGAAGAAGGTGGAGACAGAGACAGAGACAGATCCAGGCCATTAGTGGATGGATTCTTAGCAATTATCTGGGTCGACCTGCGGAGCCTGTGCCTCTTCAGCTACCATCGCTTGAGAGACTTACTCTTGATTGTAGCGAGGATTGTGGAACTTCTGGGACGCAGG---------------GGGTGGGAAGCTCTCAAATATTGGTGG---AATCTCCTGCGGTATTGG---------------------------------------------------AGCCAGGAACTAAGGAATAGTGCTATTAGCTTGCTTAATGCCACAGCCATAGCAGTAGCTGAGGGAACAGATAGGGTGTTAGAAGTATTACAAAGA------------------GCTTTTAGAGCTGTTATACACATACCTAGAAGAATAAGACAGGGCTTAGAAAGGTTTTTGCTATAA

2.04013240.ADARC.GU330432 ATGAGAGTGAAGGGGATCATGAGGAATTATCAGCACTTA---------TGGAGATGGGGC------------------------ATGATGCTCCTTGGGATAATCATGATC------------TGTAGTGCTGCA---------GAACAATTGTGGGTCACAATCTATTATGGGGTACCTGTGTGGAAAGAAGCAACCACCACTCTATTTTGTGCATCAAATGCTAAAGCATATGATACAGAGGTACATAAT---GTCTGGGCCACACATGCCTGTGTACCCACAGACCCCAACCCACAAGAAGTAAGATTG---GAAAATGTGACAGAAAATTTTAACATGTGGAAAAATAATATGGTAGAACAGATGCATGAGGATATAATTAGCCTATGGGATCAAAGCCTAAAACCATGTGTGAAATTAACCCCACTCTGTGTTACTTTAAACTGCACTGATGCTAATACCACTAATACTAATGCCAAT------------------------------------------------------------------------------AGTACTAATAACAGTAGCTTGGGAACAATGGAGAAAGGAGAAATAAAAAACTGCTCTTTCAACATCACC---ACAAACCTAAGAGAT------AAGGTGCAGAAAGAATATGCACTTTTTTATAACCTTGATGTAGTGCCAATAAAGGGTGAGGAT---------------------------AATACTAGC---------------------------TATAGGTTGATAAGTTGTAATACCTCAGTCATTACACAGGCCTGTCCAAAGGTATCCTTTGAGCCAATTCCCATACATTATTGTACTCCAGCTGGTTTTGCGATTCTACAATGT---AATGATAAGAAATTCAATGGATCAGGACCATGTACAAATGTCAGCACAGTACAATGTACACATGGAATTAGGCCAGTAGTATCAACTCAACTGCTGTTAAATGGCAGTCTAGCAGAAAAA---GAGGTAGTAATTAGGTCTGAGAATTTCACAAATAATGCTAAAACCATAATAGTACAGCTAAATGAATCTGTAGTAATTAATTGTACAAGACCCAACAACAATACAAGAAAAAGTATACCTATAGGA------------CCAGGAAGA---GCATTTTATGCAACAGGAGAAATAATAGGAGATATAAGACAAGCACATTGTAACGTT------AGTACAAAAGCCTGGAAGGAAGCTTTACAACAGGTAGCTATAAAACTATCA---GAACAATTTGGG------------AATAAAACA---ATAGTCTTTAATCAA---------TCCTCAGGAGGAGACCCAGAAGTTGTAATGCACAGTTTTAATTGTAGAGGGGAATTTTTCTACTGTAATACAACAGGACTGTTTAATAATACTTGGGGGTTT---------AATAGTACTTGG---------------------GATGCTATTAATGTGCCAGAA------------------AATGACACA------------------------------ATCACACTCCCATGCAGAATAAAACAAATTGTAAACATGTGGCAGGAAGTAGGAAAAGCAATGTATGCCCCTCCCATCAGAGGACAAATTAATTGTTCATCAAATATTACAGGGCTGCTATTAACAAGAGATGGTGGT------------------AATACCACA---------------------AATAACACT---GAGGTCTTCAGACCTGGAGGAGGAGATATGAGAGACAATTGGAGA---AGTGAATTATATAAATATAAAGTAGTAAAAATTGAACCA---TTAGGAATAGCACCC---ACCAAGGCAAAGAGAAGAGTGGTGCAGAGAGAA---AAAAGAGCAGTG---GGA---ATAGGA---GCTTTG---TTCCTT---GGG---------TTCTTGGGA---GCAGCAGGAAGCACTATGGGCGCAGCGTCGATG---ACGCTGACGGTACAGGCCAGACTATTATTGTCTGGTATAGTGCAACAGCAGAACAATTTGCTGAGAGCTATTGAGGCGCAACAGCATCTGTTGCAACTCACAGTCTGGGGCATCAAGCAGCTCCAGGCAAGA---GTCCTGGCTGTGGAAAGATACCTAAGGGATCAACAGCTCCTGGGGATTTGGGGATGCTCTGGAAAACTCATTTGCACCACTGCTGTGCCTTGGAATGTTAGTTGGAGT---------------------------AATAAATCCATGAATGACATTTGGAAT---AACATGACCTGGATGGAGTGGGAAAGAGAGATTGAC------AATTATACAAACATAATATACACCTTACTTGAAGAATCGCAGAACCAACAAGATAAGAATGAACAGGAATTATTGGAATTGGATAAATGGGCAAGTTTGTGGAATTGGTTTAGCATAACAAATTGGCTGTGGTACATAAAAATATTCATAATGATAGTAGGAGGCTTAATAGGTTTAAGAATAGTTTTTACTGTATTTTCTATAGTGAATAGAGTTAGGCAGGGATATTCACCATTATCGTTTCAGACCCGC---CTCCCAACTTCGAGGGGA------CTCGACAGGCCCGAAGGAATCGAAGAAGAAGGTGGAGACAGAGACAGAGACAGATCCAGGCCATTAGTGGATGGATTCTTAGCAATTATCTGGGTCGACCTGCGGAGCCTGTGCCTCTTCAGCTACCATCGCTTGAGAGACTTACTCTTGATTGTAGCGAGGATTGTGGAACTTCTGGGACGCAGG---------------GGGTGGGAAGCCCTCAAATATTGGTGG---AATCTCCTGCGGTATTGG---------------------------------------------------AGCCAGGAACTAAGGAATAGTGCTATTAGCTTGCTTAATGCCACAGCCATAGCAGTAGCTGAGGGAACAGATAGGGTGTTAGAAGTATTACAAAGA------------------GCTTTTAGAGCTGTTATACACATACCTAGAAGAATAAGACAGGGCTTAGAAAGGTTTTTGCTATAA

2.04013240.ADARC.GU330433 ATGAGAGTGAAGGGGATCATGAGGAATTATCAGCACTTA---------TGGAGATGGGGC------------------------ATGATGCTCCTTGGGATAATCATGATC------------TGTAGTGCTGCA---------GAACAATTGTGGGTCACAATCTATTATGGGGTACCTGTGTGGAAAGAAGCAAACACCACTCTATTTTGTGCATCAAATGCTAAAGCATATGATACAGAGGTACATAAT---GTCTGGGCCACACATGCCTGTGTACCCACAGACCCCAACCCACAAGAAGTAAGATTG---GAAAATGTGACAGAAAATTTTAACATGTGGAAAAATAATATGGTAGAACAGATGCATGAGGATATAATTAGCCTATGGGATCAAAGCCTAAAACCATGTGTGAAATTAACCCCACTCTGTGTTACTTTAAACTGCACTGATGCTAATACCACTAATACTAATGCCAAT------------------------------------------------------------------------------AGTACTAATAACAGTAGCTTGGGAACAATGGAGAAAGGAGAAATAAAAAACTGCTCTTTCAACATCACC---ACAAACCTAAGAGAT------AAGGTGCAGAAAGAATATGCACTTTTTTATAACCTTGATGTAGTGCCAATAAAGGGTGAGGAT---------------------------AATACTAGC---------------------------TATAGGTTGATAAGTTGTAATACCTCAGTCATTACACAGGCCTGTCCAAAGGTATCCTTTGAGCCAATTCCCATACATTATTGTACTCCAGCTGGTTTTGCGATTCTACAATGT---AATGATAAGAAATTCAATGGATCAGGACCATGTACAAATGTCAGCACAGTACAATGTACACATGGAATTAGGCCAGTAGTATCAACTCAACTGCTGTTAAATGGCAGTCTAGCAGAAAAA---GAGGTAGTAATTAGGTCTGAGAATTTCACAAATAATGCTAAAACCATAATAGTACAGCTAAATGAATCTGTAGTAATTAATTGTACAAGACCCAACAACAATACAAGAAAAAGTATACCTATAGGA------------CCAGGAAGA---GCATTTTATGCAACAGGAGAAATAATAGGAGATATAAGACAAGCACATTGTAACGTT------AGTACAAAAGCCTGGAAGGAAGCTTTACAACAGGTAGCTATAAAACTATCA---GAACAATTTGGG------------AATAAAACA---ATAGTCTTTAATCAA---------TCCTCAGGAGGAGACCCAGAAGTTGTAATGCACAGTTTTAATTGTAGAGGGGAATTTTTCTACTGTAATACAACAGGACTGTTTAATAATACTTGGGGGTTT---------AATAGTACTTGG---------------------GATGCTATTAATGTGCCAGAA------------------AATGACACA------------------------------ATCACACTCCCATGCAGAATAAAACAAATTGTAAACATGTGGCAGGAAGTAGGAAAAGCAATGTATGCCCCTCCCATCAGAGGACGACTTAATTGTTCATCAAATATTACAGGGCTGCTATTAACAAGAGATGGTGGT------------------AATACCACA---------------------AATAACACT---GAGGTCTTCAGACCTGGAGGAGGAGATATGAGAGACAATTGGAGA---AGTGAATTATATAAATATAAAGTAGTAAAAATTGAACCA---TTAGGAATAGCACCC---ACCAAGGCAAAGAGAAGAGTGGTGCAGAGAGAA---AAAAGAGCAGTG---GGA---ATAGGA---GCTTTG---TTCCTT---GGG---------TTCTTGGGA---GCAGCAGGAAGCACTATGGGCGCAGCGTCGATG---ACGCTGACGGTACAGGCCAGACTATTATTGTCTGGTATAGTGCAACAGCAGAACAATTTGCTGAGAGCTATTGAGGCGCAACAGCATCTGTTGCAACTCACAGTCTGGGGCATCAAGCAGCTCCAGGCAAGA---GTCCTGGCTGTGGAAAGATACCTAAGAGATCAACAGCTCCTGGGGATTTGGGGATGCTCTGGAAAACTCATTTGCACCACTACTGTGCCTTGGAATGTTAGTTGGAGT---------------------------AATAAATCCATGAATGACATTTGGAAT---AACATGACCTGGATGGAGTGGGAAAGAGAGATTGAC------AATTATACAAACATAATATACACCTTACTTGAAGAATCGCAGAACCAACAAGATAAGAATGAACAGGAATTATTGGAATTGGATAAATGGGCAAGTTTGTGGAATTGGTTTAGCATAACAAATTGGCTGTGGTACATAAAAATATTCATAATGATAGTAGGAGGCTTAATAGGTTTAAGAATAGTTTTTACTGTATTTTCTATAGTGAATAGAGTTAGGCAGGGATATTCACCATTATCGTTTCAGACCCGC---TTCCCAACCTCGAGGGGA------CTCGACAGGCCCGAAGGAATCGAAGAAGAAGGTGGAGACAGAGACAGAGACAGATCCAGGCCATTAGTGGATGGATTCTTAGCAATTATCTGGGTCGACCTGCGGAGCCTGTGCCTCTTCAGCTACCATCGCTTGAGAGACTTACTCTTGATTGTAGCGAGGATTGTGGAACTTCTGGGACGCAGG---------------GGGTGGGAAGCCCTCAAATATTGGTGG---AATCTCCTGCGGTATTGG---------------------------------------------------AGCCAGGAACTAAGGAATAGTGCTATTAGCTTGCTTAATGCCACAGCCATAGCAGTAGCTGAGGGAACAGATAGGGTGTTAGAAGTATTACAAAGA------------------GCTTTTAGAGCTGTTATACACATACCTAGAAGAATAAGACAGGGCTTAGAAAGGTTTTTGCTATAA

2.04013240.ADARC.GU330434 ATGAGAGTGAAGGGGATCATGAGGAATTATCAGCACTTA---------TGGAGATGGGGC------------------------ATGATGCTCCTTGGGATAATCATGATC------------TGTAGTGCTGCA---------GAACAATTGTGGGTCACAATCTATTATGGGGTACCTGTGTGGAAAGAAGCAACCACCACTCTATTTTGTGCATCAAATGCTAAAGCATATGATACAGAGGTACATAAT---GTCTGGGCCACACATGCCTGTGTACCCACAGACCCCAACCCACAAGAAGTAAGATTG---GAAAATGTGACAGAAAATTTTAACATGTGGAAAAATAATATGGTAGAACAGATGCATGAGGATATAATTAGCCTATGGGATCAAAGCCTAAAACCATGTGTGAAATTAACCCCACTCTGTGTTACTTTAAACTGCACTGATGCTAATACCACTAATACTAATGCCAAT------------------------------------------------------------------------------AGTACTAATAACAGTAGCTTGGGAACAATGGAGAAAGGAGAAATAAAAAACTGCTCTTTCAACATCACC---ACAAACCTAAGAGAT------AAGGTGCAGAAAGAATATGCACTTTTTTATAACCTTGATGTAGTGCCAATAAAGGGTGAGGAT---------------------------AATACTAGC---------------------------TATAGGTTGATAAGTTGTAATACCTCAGTCATTACACAGGCCTGTCCAAAGGTATCCTTTGAGCCAATTCCCATACATTATTGTACTCCAGCTGGTTTTGCGATTCTACAATGT---AATGATAAGAAATTCAATGGATCAGGACCATGTACAAATGTCAGCACAGTACAATGTACACATGGAATTAGGCCAGTAGTATCAACTCAACTGCTGTTAAATGGCAGTCTAGCAGAAAAA---GAGGTAGTAATTAGGTCTGAGAATTTCACAAATAATGCTAAAACCATAATAGTACAGCTAAATGAATCTGTAGTAATTAATTGTACAAGACCCAACAACAATACAAGAAAAAGTATACCTATAGGA------------CCAGGAAGA---GCATTTTATGCAACAGGAGAAATAATAGGAGATATAAGACAAGCACATTGTAACGTT------AGTACAAAAGCCTGGAAGGAAGCTTTACAACAGGTAGCTATAAAACTATCA---GAACAATTTGGG------------AATAAAACA---ATAGTCTTTAATCAA---------TCCTCAGGAGGAGACCCAGAAGTTGTAATGCACAGTTTTAATTGTAGAGGGGAATTTTTCTACTGTAATACAACAGGACTGTTTAATAATACTTGGGGGTTT---------AATAGTACTTGG---------------------GATGCTATTAATGTGCCAGAA------------------AATGACACA------------------------------ATCACACTCCCATGCAGAATAAAACAAATTGTAAACATGTGGCAGGAAGTAGGAAAAGCAATGTATGCCCCTCCCATCAGAGGACGACTTAATTGTTCATCAAATATTACAGGGCTGCTATTAACAAGAGATGGTGGT------------------AATACCACA---------------------AATAACACT---GAGGTCTTCAGACCTGGAGGAGGAGATATGAGAGACAATTGGAGA---AGTGAATTATATAAATATAAAGTAGTAAAAATTGAACCA---TTAGGAATAGCACCC---ACCAAGGCAAAGAGAAGAGTGGTGCAGAGAGAA---AAAAGAGCAGTG---GGA---ATAGGA---GCTTTG---TTCCTT---GGG---------TTCTTGGGA---GCAGCAGGAAGCACTATGGGCGCAGCGTCGATG---ACGCTGACGGTACAGGCCAGACTATTATTGTCTGGTATAGTGCAACAGCAGAACAATTTGCTGAGAGCTATTGAGGCGCAACAGCATCTGTTGCAACTCACAGTCTGGGGCATCAAGCAGCTCCAGGCAAGA---GTCCTGGCTGTGGAAAGATACCTAAGAGATCAACAGCTCCTGGGGATTTGGGGATGCTCTGGAAAACTCATTTGCACCACTACTGTGCCTTGGAATGTTAGTTGGAGT---------------------------AATAAATCCATGAATGACATTTGGAAT---AACATGACCTGGATGGAGTGGGAAAGAGAGATTGAC------AATTATACAAACATAATATACACCTTACTTGAAGAATCGCAGAACCAACAAGATAAGAATGAACAGGAATTATTGGAATTGGATAAATGGGCAAGTTTGTGGAATTGGTTTAGCATAACAAATTGGCTGTGGTACATAAAAATATTCATAATGATAGTAGGAGGCTTAATAGGTTTAAGAATAGTTTTTACTGTATTTTCTATAGTGAATAGAGTTAGGCAGGGATATTCACCATTATCGTTTCAGACCCGC---TTCCCAACCTCGAGGGGA------CTCGACAGGCCCGAAGGAATCGAAGAAGAAGGTGGAGACAGAGACAGAGACAGATCCAGGCCATTAGTGGATGGATTCTTAGCAATTATCTGGGTCGACCTGCGGAGCCTGTGCCTCTTCAGCTACCATCGCTTGAGAGACTTACTCTTGATTGTAGCGAGGATTGTGGAACTTCTGGGACGCAGG---------------GGGTGGGAAGCCCTCAAATATTGGTGG---AATCTCCTGCGGTATTGG---------------------------------------------------AGCCAGGAACTAAGGAATAGTGCTATTAGCTTGCTTAATGCCACAGCCATAGCAGTAGCTGAGGGAACAGATAGGGTGTTAGAAGTATTACAAAGA------------------GCTTTTAGAGCTGTTATACACATACCTAGAAGAATAAGACAGGGCTTAGAAAGGTTTTTGCTATAA

2.04013240.ADARC.GU330435 ATGAGAGTGAAGGGGATCATGAGGAATTATCAGCACTTA---------TGGAGATGGGGC------------------------ATGATGCTCCTTGGGATAATCATGATC------------TGTAGTGCTGCA---------GAACAATTGTGGGTCACAATCTATTATGGGGTACCTGTGTGGAAAGAAGCAACCACCACTCTATTTTGTGCATCAAATGCTAAAGCATATGATACAGAGGTACATAAT---GTCTGGGCCACACATGCCTGTGTACCCACAGACCCCAACCCACAAGAAGTAAGATTG---GAAAATGTGACAGAAAATTTTAACATGTGGAAAAATAATATGGTAGAACAGATGCATGAGGATATAATTAGCCTATGGGATCAAAGCCTAAAACCATGTGTGAAATTAACCCCACTCTGTGTTACTTTAAACTGCACTGATGCTAATACCACTAATACTAATGCCAAT------------------------------------------------------------------------------AGTACTAATAACAGTAGCTTGGGAACAATGGAGAAAGGAGAAATAAAAAACTGCTCTTTCAACATCACC---ACAAACCTAAGAGAT------AAGGTGCAGAAAGAATATGCACTTTTTTATAACCTTGATGTAGTGCCAATAAAGGGTGAGGAT---------------------------AATACTAGC---------------------------TATAGGTTGATAAGTTGTAATACCTCAGTCATTACACAGGCCTGTCCAAAGGTATCCTTTGAGCCAATTCCCATACATTATTGTACTCCAGCTGGTTTTGCGATTCTACAATGT---AATGATAAGAAATTCAATGGATCAGGACCATGTACAAATGTCAGCACAGTACAATGTACACATGGAATTAGGCCAGTAGTATCAACTCAACTGCTGTTAAATGGCAGTCTAGCAGAAAAA---GAGGTAGTAATTAGGTCTGAGAATTTCACAAATAATGCTAAAACCATAATAGTACAGCTAAATGAATCTGTAGTAATTAATTGTACAAGACCCAACAACAATACAAGAAAAAGTATACCTATAGGA------------CCAGGAAGA---GCATTTTATGCAACAGGAGAAATAATAGGAGATATAAGACAAGCACATTGTAACGTT------AGTACAAAAGCCTGGAAGGAAGCTTTACAACAGGTAGCTATAAAACTATCA---GAACAATTTGGG------------AATAAAACA---ATAGTCTTTAATCAA---------TCCTCAGGAGGAGACCCAGAAGTTGTAATGCACAGTTTTAATTGTAGAGGGGAATTTTTCTACTGTAATACAACAGGACTGTTTAATAATACTTGGGGGTTT---------AATAGTACTTGG---------------------GATGCTATTAATGTGCCAGAA------------------AATGACACA------------------------------ATCACACTCCCATGCAGAATAAAACAAATTGTAAACATGTGGCAGGAAGTAGGAAAAGCAATGTATGCCCCTCCCATCAGAGGACGACTTAATTGTTCATCAAATATTACAGGGCTGCTATTAACAAGAGATGGTGGT------------------AATACCACA---------------------AATAACACT---GAGGTCTTCAGACCTGGAGGAGGAGATATGAGAGACAATTGGAGA---AGTGAATTATATAAATATAAAGTAGTAAAAATTGAACCA---TTAGGAATAGCACCC---ACCAAGGCAAAGAGAAGAGTGGTGCAGAGAGAA---AAAAGAGCAGTG---GGA---ATAGGA---GCTTTG---TTCCTT---GGG---------TTCTTGGGA---GCAGCAGGAAGCACTATGGGCGCAGCGTCGATG---ACGCTGACGGTACAGGCCAGACTATTATTGTCTGGTATAGTGCAACAGCAGAACAATTTGCTGAGAGCTATTGAGGCGCAACAGCATCTGTTGCAACTCACAGTCTGGGGCATCAAGCAGCTCCAGGCAAGA---GTCCTGGCTGTGGAAAGATACCTAAGAGATCAACAGCTCCTGGGGATTTGGGGATGCTCTGGAAAACTCATTTGCACCACTACTGTGCCTTGGAATGTTAGTTGGAGT---------------------------AATAAATCCATGAATGACATTTGGAAT---AACATGACCTGGATGGAGTGGGAAAGAGAGATTGAC------AATTATACAAACATAATATACACCTTACTTGAAGAATCGCAGAACCAACAAGATAAGAATGAACAGGAATTATTGGAATTGGATAAATGGGCAAGTTTGTGGAATTGGTTTAGCATAACAAATTGGCTGTGGTACATAAAAATATTCATAATGATAGTAGGAGGCTTAATAGGTTTAAGAATAGTTTTTACTGTATTTTCTATAGTGAATAGAGTTAGGCAGGGATATTCACCATTATCGTTTCAGACCCGC---TTCCCAACCTCGAGGGGA------CTCGACAGGCCCGAAGGAATCGAAGAAGAAGGTGGAGACAGAGACAGAGACAGATCCAGGCCATTAGTGGATGGATTCTTAGCAATTATCTGGGTCGACCTGCGGAGCCTGTGCCTCTTCAGCTACCATCGCTTGAGAGACTTACTCTTGATTGTAGCGAGGATTGTGGAACTTCTGGGACGCAGG---------------GGGTGGGAAGCCCTCAAATATTGGTGG---AATCTCCTGCGGTATTGG---------------------------------------------------AGCCAGGAACTAAGGAATAGTGCTATTAGCTTGCTTAATGCCACAGCCATAGCAGTAGCTGAGGGAACAGATAGGGTGTTAGAGGTATTACAAAGA------------------GCTTTTAGAGCTGTTATACACATACCTAGAAGAATAAGACAGGGCTTAGAAAGGTTTTTGCTATAA

2.04013240.ADARC.GU330436 ATGAGAGTGAAGGGGATCATGAGGAATTATCAGCACTTA---------TGGAGATGGGGC------------------------ATGATGCTCCTTGGGATAATCATGATC------------TGTAGTGCTGCA---------GAACAATTGTGGGTCACAATCTATTATGGGGTACCTGTGTGGAAAGAAGCAACCACCACTCTATTTTGTGCATCAAATGCTAAAGCATATGATACAGAGGTACATAAT---GTCTGGGCCACACATGCCTGTGTACCCACAGACCCCAACCCACAAGAAGTAAGATTG---GAAAATGTGACAGAAAATTTTAACATGTGGAAAAATAATATGGTAGAACAGATGCATGAGGATATAATTAGCCTATGGGATCAAAGCCTAAAACCATGTGTGAAATTAACCCCACTCTGTGTTACTTTAAACTGCACTGATGCTAATACCACTAATACTAATGCCAAT------------------------------------------------------------------------------AGTACTAATAACAGTAGCTTGGGAACAATGGAGAAAGGAGAAATAAAAAACTGCTCTTTCAACATCACC---ACAAACCTAAGAGAT------AAGGTGCAGAAAGAATATGCACTTTTTTATAACCTTGATGTAGTGCCAATAAAGGGTGAGGAT---------------------------AATACTAGC---------------------------TATAGGTTGATAAGTTGTAATACCTCAGTCATTACACAGGCCTGTCCAAAGGTATCCTTTGAGCCAATTCCCATACATTATTGTACTCCAGCTGGTTTTGCGATTCTACAATGT---AATGATAAGAAATTCAATGGATCAGGACCATGTACAAATGTCAGCACAGTACAATGTACACATGGAATTAGGCCAGTAGTATCAACTCAACTGCTGTTAAATGGCAGTCTAGCAGAAAAA---GAGGTAGTAATTAGGTCTGAGAATTTCACAAATAATGCTAAAACCATAATAGTACAGCTAAATGAATCTGTAGTAATTAATTGTACAAGACCCAACAACAATACAAGAAAAAGTATACCTATAGGA------------CCAGGAAGA---GCATTTTATGCAACAGGAGAAATAATAGGAGATATAAGACAAGCACATTGTAACGTT------AGTACAAAAGCCTGGAAGGAAGCTTTACAACAGGTAGCTATAAAACTATCA---GAACAATTTGGG------------AATAAAACA---ATAGTCTTTAATCAA---------TCCTCAGGAGGAGACCCAGAAGTTGTAATGCACAGTTTTAATTGTAGAGGGGAATTTTTCTACTGTAATACAACAGGACTGTTTAATAATACTTGGGGGTTT---------AATAGTACTTGG---------------------GATGCTATTAATGTGCCAGAA------------------AATGACACA------------------------------ATCACACTCCCATGCAGAATAAAACAAATTGTAAACATGTGGCAGGAAGTAGGAAAAGCAATGTATGCCCCTCCCATCAGAGGACGACTTAATTGTTCATCAAATATTACAGGGCTGCTATTAACAAGAGATGGTGGT------------------AATACCACA---------------------AATAACACT---GAGGTCTTCAGACCTGGAGGAGGAGATATGAGAGACAATTGGAGA---AGTGAATTATATAAATATAAAGTAGTAAAAATTGAACCA---TTAGGAATAGCACCC---ACCAAGGCAAAGAGAAGAGTGGTGCAGAGAGAA---AAAAGAGCAGTG---GGA---ATAGGA---GCTTTG---TTCCTT---GGG---------TTCTTGGGA---GCAGCAGGAAGCACTATGGGCGCAGCGTCGATG---ACGCTGACGGTACAGGCCAGACTATTATTGTCTGGTATAGTGCAACAGCAGAACAATTTGCTGAGAGCTATTGAGGCGCAACAGCATCTGTTGCAACTCACAGTCTGGGGCATCAAGCAGCTCCAGGCAAGA---GTCCTGGCTGTGGAAAGATACCTAAGAGATCAACAGCTCCTGGGGATTTGGGGATGCTCTGGAAAACTCATTTGCACCACTACTGTGCCTTGGAATGTTAGTTGGAGT---------------------------AATAAATCCATGAATGACATTTGGAAT---AACATGACCTGGATGGAGTGGGAAAGAGAGATTGAC------AATTATACAAACATAATATACACCTTACTTGAAGAATCGCAGAACCAACAAGATAAGAATGAACAGGAATTATTGGAATTGGATAAATGGGCAAGTTTGTGGAATTGGTTTAGCATAACAAATTGGCTGTGGTACATAAAAATATTCATAATGATAGTAGGAGGCTTAATAGGTTTAAGAATAGTTTTTACTGTATTTTCTATAGTGAATAGAGTTAGGCAGGGATATTCACCATTATCGTTTCAGACCCGC---TTCCCAACCTCGAGGGGA------CTCGACAGGCCCGAAGGAATCGAAGAAGAAGGTGGAGACAGAGACAGAGACAGATCCAGGCCATTAGTGGATGGATTCTTAGCAATTATCTGGGTCGACCTGCGGAGCCTGTGCCTCTTCAGCTACCATCGCTTGAGAGACTTACTCTTGATTGTAGCGAGGATTGTGGAACTTCTGGGACGCAGG---------------GGGTGGGAAGCCCTCAAATATTGGTGG---AATCTCCTGCGGTATTGG---------------------------------------------------AGCCAGGAACTAAGGAATAGTGCTATTAGCTTGCTTAATGCCACAGCCATAGCAGTAGCTGAGGGAACAGATAGGGTGTTAGAAGTATTACAAAGA------------------GCTTTTAGAGCTGTTATACACATACCTAGAAGAATAAGACAGGGCTTAGAAAGGTTTTTGCTATAA

2.04013240.ADARC.GU330437 ATGAGAGTGAAGGGGATCATGAGGAATTATCAGCACTTA---------TGGAGATGGGGC------------------------ATGATGCTCCTTGGGATAATCATGATC------------TGTAGTGCTGCA---------GAACAATTGTGGGTCACAATCTATTATGGGGTACCTGTGTGGAAAGAAGCAACCACCACTCTATTTTGTGCATCAAATGCTAAAGCATATGATACAGAGGTACATAAT---GTCTGGGCCACACATGCCTGTGTACCCACAGACCCCAACCCACAAGAAGTAAGATTG---GAAAATGTGACAGAAAATTTTAACATGTGGAAAAATAATATGGTAGAACAGATGCATGAGGATATAATTAGCCTATGGGATCAAAGCCTAAAACCATGTGTGAAATTAACCCCACTCTGTGTTACTTTAAACTGCACTGATGCTAATACCACTAATACTAATGCCAAT------------------------------------------------------------------------------AGTACTAATAACAGTAGCTTGGGAACAATGGAGAAAGGAGAAATAAAAAACTGCTCTTTCAACATCACC---ACAAACCTAAGAGAT------AAGGTGCAGAAAGAATATGCACTTTTTTATAACCTTGATGTAGTGCCAATAAAGGGTGAGGAT---------------------------AATACTAGC---------------------------TATAGGTTGATAAGTTGTAATACCTCAGTCATTACACAGGCCTGTCCAAAGGTATCCTTTGAGCCAATTCCCATACATTATTGTACTCCAGCTGGTTTTGCGATTCTACAATGT---AATGATAAGAAATTCAATGGATCAGGACCATGTACAAATGTCAGCACAGTACAATGTACACATGGAATTAGGCCAGTAGTATCAACTCAACTGCTGTTAAATGGCAGTCTAGCAGAAAAA---GAGGTAGTAATTAGGTCTGAGAATTTCACAAATAATGCTAAAACCATAATAGTACAGCTAAATGAATCTGTAGTAATTAATTGTACAAGACCCAACAACAATACAAGAAAAAGTATACCTATAGGA------------CCAGGAAGA---GCATTTTATGCAACAGGAGAAATAATAGGAGATATAAGACAAGCACATTGTAACGTT------AGTACAAAAGCCTGGAAGGAAGCTTTACAACAGGTAGCTATAAAACTATCA---GAACAATTTGGG------------AATAAAACA---ATAGTCTTTAATCAA---------TCCTCAGGAGGAGACCCAGAAGTTGTAATGCACAGTTTTAATTGTAGAGGGGAATTTTTCTACTGTAATACAACAGGACTGTTTAATAATACTTGGGGGTTT---------AATAGTACTTGG---------------------GATGCTATTAATGTGCCAGAA------------------AATGACACA------------------------------ATCACACTCCCATGCAGAATAAAACAAATTGTAAACATGTGGCAGGAAGTAGGAAAAGCAATGTATGCCCCTCCCATCAGAGGACAATTTAATTGTTCATCAAATATTACAGGGCTGCTATTAACAAGAGATGGTGGT------------------AATACCACA---------------------AATAACACT---GAGGTCTTCAGACCTGGAGGAGGAGATATGAGAGACAATTGGAGA---AGTGAATTATATAAATATAAAGTAGTAAAAATTGAACCA---TTAGGAATAGCACCC---ACCAAGGCAAAGAGAAGAGTGGTGCAGAGAGAA---AAAAGAGCAGTG---GGA---ATAGGA---GCTTTG---TTCCTT---GGG---------TTCTTGGGA---GCAGCAGGAAGCACTATGGGCGCAGCGTCGATG---ACGCTGACGGTACAAGCCAGACTATTATTGTCTGGTATAGTGCAACAGCAGAACAATTTGCTGAGAGCTATTGAGGCGCAACAGCATCTGTTGCAACTCACAGTCTGGGGCATCAAGCAGCTCCAGGCAAGA---GTCCTGGCTGTGGAAAGATACCTAAGGGATCAACAGCTCCTGGGGATTTGGGGATGCTCTGGAAAACTCATTTGCACCACTGCTGTGCCTTGGAATGTTAGTTGGAGT---------------------------AATAAATCCATGAATGACATTTGGAAT---AACATGACCTGGATGGAGTGGGAAAGAGAGATTGAC------AATTATACAAACATAATATACACCTTACTTGAAGAATCGCAGAACCAACAAGATAAGAATGAACAGGAATTATTGGAATTGGATAAATGGGCAAGTTTGTGGAATTGGTTTAGCATAACAAATTGGCTGTGGTACATAAAAATATTCATAATGATAGTAGGAGGCTTAATAGGTTTAAGAATAGTTTTTACTGTATTTTTTATAGTGAATAGAGTTAGGCAGGGATATTCACCATTATCGTTTCAGACCCGC---TTCCCAACCTCGAGGGGA------CTCGACAGGCCCGAAGGAATCGAAGAAGAAGGTGGAGACAGAGACAGAGACAGATCCAGGCCATTAGTGGATGGATTCTTAGCAATTATCTGGGTCGACCTGCGGAGCCTGTGCCTCTTCAGCTACCATCGCTTGAGAGACTTACTCTTGATTGTAGCGAGGATTGTGGAACTTCTGGGACGCAGG---------------GGGTGGGAAGCCCTCAAATATTGGTGG---AATCTCCTGCGGTATTGG---------------------------------------------------AGCCAGGAACTAAGGAATAGTGCTATTAGCTTGCTTAATGCCACAGCCATAGCAGTAGCTGAGGGAACAGATAGGGTGTTAGAAGTATTACAAAGA------------------GCTTTTAGAGCTGTTATACACATACCTAGAAGAATAAGACAGGGCTTAGAAAGGTTTTTGCTATAA

2.04013240.ADARC.GU330438 ATGAGAGTGAAGGGGATCATGAGGAATTATCAGCACTTA---------TGGAGATGGGGC------------------------ATGATGCTCCTTGGGATAATCATGATC------------TGTAGTGCTGCA---------GAACAATTGTGGGTCACAATCTATTATGGGGTACCTGTGTGGAAAGAAGCAACCACCACTCTATTTTGTGCATCAAATGCTAAAGCATATGATACAGAGGTACATAAT---GTCTGGGCCACACATGCCTGTGTACCCACAGACCCCAACCCACAAGAAGTAAGATTG---GAAAATGTGACAGAAAATTTTAACATGTGGAAAAATAATATGGTAGAACAGATGCATGAGGATATAATTAGCCTATGGGATCAAAGCCTAAAACCATGTGTGAAATTAACCCCACTCTGTGTTACTTTAAACTGCACTGATGCTAATACCACTAATACTAATGCCAAT------------------------------------------------------------------------------AGTACTAATAACAGTAGCTTGGGAACAATGGAGAAAGGAGAAATAAAAAACTGCTCTTTCAACATCACC---ACAAACCTAAGAGAT------AAGGTGCAGAAAGAATATGCACTTTTTTATAACCTTGATGTAGTGCCAATAAAGGGTGAGGAT---------------------------AATACTAGC---------------------------TATAGGTTGATAAGTTGTAATACCTCAGTCATTACACAGGCCTGTCCAAAGGTATCCTTTGAGCCAATTCCCATACATTATTGTACTCCAGCTGGTTTTGCGATTCTACAATGT---AATGATAAGAAATTCAATGGATCAGGACCATGTACAAATGTCAGCACAGTACAATGTACACATGGAATTAGGCCAGTAGTATCAACTCAACTGCTGTTAAATGGCAGTCTAGCAGAAAAA---GAGGTAGTAATTAGGTCTGAGAATTTCACAAATAATGCTAAAACCATAATAGTACAGCTAAATGAATCTGTAGTAATTAATTGTACAAGACCCAACAACAATACAAGAAAAAGTATACCTATAGGA------------CCAGGAAGA---GCATTTTATGCAACAGGAGAAATAATAGGAGATATAAGACAAGCACATTGTAACGTT------AGTACAAAAGCCTGGAAGGAAGCTTTACAACAGGTAGCTATAAAACTATCA---GAACAATTTGGG------------AATAAAACA---ATAGTCTTTAATCAA---------TCCTCAGGAGGAGACCCAGAAGTTGTAATGCACAGTTTTAATTGTAGAGGGGAATTTTTCTACTGTAATACAACAGGACTGTTTAATAATACTTGGGGGTTT---------AATAGTACTTGG---------------------GATGCTATTAATGTGCCAGAA------------------AATGACACA------------------------------ATCACACTCCCATGCAGAATAAAACAAATTGTAAACATGTGGCAGGAAGTAGGAAAAGCAATGTATGCCCCTCCCATCAGAGGACAACTTAATTGTTCATCAAATATTACAGGGCTGCTATTAACAAGAGATGGTGGT------------------AATACCACA---------------------AATAACACT---GAGGTCTTCAGACCTGGAGGAGGAGATATGAGAGACAATTGGAGA---AGTGAATTATATAAATATAAAGTAGTAAAAATTGAACCA---TTAGGAATAGCACCC---ACCAAGGCAAAGAGAAGAGTGGTGCAGAGAGAA---AAAAGAGCAGTG---GGA---ATAGGA---GCTTTG---TTCCTT---GGG---------TTCTTGGGA---GCAGCAGGAAGCACTATGGGCGCAGCGTCGATG---ACGCTGACGGTACAAGCCAGACTATTATTGTCTGGTATAGTGCAACAGCAGAACAATTTGCTGAGAGCTATTGAGGCGCAACAGCATCTGTTGCAACTCACAGTCTGGGGCATCAAGCAGCTCCAGGCAAGA---GTCCTGGCTGTGGAAAGATACCTAAGGGATCAACAGCTCCTGGGGATTTGGGGATGCTCTGGAAAACTCATTTGCACCACTGCTGTGCCTTGGAATGTTAGTTGGAGT---------------------------AATAAATCCATGAATGACATTTGGAAT---AACATGACCTGGATGGAGTGGGAAAGAGAGATTGAC------AATTATACAAACATAATATACACCTTACTTGAAGAATCGCAGAACCAACAAGATAAGAATGAACAGGAATTATTGGAATTGGATAAATGGGCAAGTTTGTGGAATTGGTTTAGCATAACAAATTGGCTGTGGTACATAAAAATATTCATAATGATAGTAGGAGGCTTAATAGGTTTAAGAATAGTTTTTACTGTATTTTTTATAGTGAATAGAGTTAGGCAGGGATATTCACCATTATCGTTTCAGACCCGC---TTCCCAACCTCGAGGGGA------CTCGACAGGCCCGAAGGAATCGAAGAAGAAGGTGGAGACAGAGACAGAGACAGATCCAGGCCATTAGTGGATGGATTCTTAGCAATTATCTGGGTCGACCTGCGGAGCCTGTGCCTCTTCAGCTACCATCGCTTGAGAGACTTACTCTTGATTGTAGCGAGGATTGTGGAACTTCTGGGACGCAGG---------------GGGTGGGAAGCCCTCAAATATTGGTGG---AATCTCCTGCGGTATTGG---------------------------------------------------AGCCAGGAACTAAGGAATAGTGCTATTAGCTTGCTTAATGCCACAGCCATAGCAGTAGCTGAGGGAACAGATAGGGTGTTAGAAGTATTACAAAGA------------------GCTTTTAGAGCTGTTATACACATACCTAGAAGAATAAGACAGGGCTTAGAAAGGTTTTTGCTATAA

2.04013240.ADARC.GU330439 ATGAGAGTGAAGGGGATCATGAGGAATTATCAGCACTTA---------TGGAGATGGGGC------------------------ATGATGCTCCTTGGGATAATCATGATC------------TGTAGTGCTGCA---------GAACAATTGTGGGTCACAATCTATTATGGGGTACCTGTGTGGAAAGAAGCAACCACCACTCTATTTTGTGCATCAAATGCTAAAGCATATGATACAGAGGTACATAAT---GTCTGGGCCACACATGCCTGTGTACCCACAGACCCCAACCCACAAGAAGTAAGATTG---GAAAATGTGACAGAAAATTTTAACATGTGGAAAAATAATATGGTAGAACAGATGCATGAGGATATAATTAGCCTATGGGATCAAAGCCTAAAACCATGTGTGAAATTAACCCCACTCTGTGTTACTTTAAACTGCACTGATGCTAATACCACTAATACTAATGCCAAT------------------------------------------------------------------------------AGTACTAATAACAGTAGCTTGGGAACAATGGAGAAAGGAGAAATAAAAAACTGCTCTTTCAACATCACC---ACAAACCTAAGAGAT------AAGGTGCAGAAAGAATATGCACTTTTTTATAACCTTGATGTAGTGCCAATAAAGGGTGAGGAT---------------------------AATACTAGC---------------------------TATAGGTTGATAAGTTGTAATACCTCAGTCATTACACAGGCCTGTCCAAAGGTATCCTTTGAGCCAATTCCCATACATTATTGTACTCCAGCTGGTTTTGCGATTCTACAATGT---AATGATAAGAAATTCAATGGATCAGGACCATGTACAAATGTCAGCACAGTACAATGTACACATGGAATTAGGCCAGTAGTATCAACTCAACTGCTGTTAAATGGCAGTCTAGCAGAAAAA---GAGGTAGTAATTAGGTCTGAGAATTTCACAAATAATGCTAAAACCATAATAGTACAGCTAAATGAATCTGTAGTAATTAATTGTACAAGACCCAACAACAATACAAGAAAAAGTATACCTATAGGA------------CCAGGAAGA---GCATTTTATGCAACAGGAGAAATAATAGGAGATATAAGACAAGCACATTGTAACGTT------AGTACAAAAGCCTGGAAGGAAGCTTTACAACAGGTAGCTATAAAACTATCA---GAACAATTTGGG------------AATAAAACA---ATAGTCTTTAATCAA---------TCCTCAGGAGGAGACCCAGAAGTTGTAATGCACAGTTTTAATTGTAGAGGGGAATTTTTCTACTGTAATACAACAGGACTGTTTAATAATACTTGGGGGTTT---------AATAGTACTTGG---------------------GATGCTATTAATGTGCCAGAA------------------AATGACACA------------------------------ATCACACTCCCATGCAGAATAAAACAAATTGTAAACATGTGGCAGGAAGTAGGAAAAGCAATGTATGCCCCTCCCATCAGAGGACGACTTAATTGTTCATCAAATATTACAGGGCTGCTATTAACAAGAGATGGTGGT------------------AATACCACA---------------------AATAACACT---GAGGTCTTCAGACCTGGAGGAGGAGATATGAGAGACAATTGGAGA---AGTGAATTATATAAATATAAAGTAGTAAAAATTGAACCA---TTAGGAATAGCACCC---ACCAAGGCAAAGAGAAGAGTGGTGCAGAGAGAA---AAAAGAGCAGTG---GGA---ATAGGA---GCTTTG---TTCCTT---GGG---------TTCTTGGGA---GCAGCAGGAAGCACTATGGGCGCAGCGTCGATG---ACGCTGACGGTACAGGCCAGACTATTATTGTCTGGTATAGTGCAACAGCAGAACAATTTGCTGAGAGCTATTGAGGCGCAACAGCATCTGTTGCAACTCACAGTCTGGGGCATCAAGCAGCTCCAGGCAAGA---GTCCTGGCTGTGGAAAGATACCTAAGAGATCAACAGCTCCTGGGGATTTGGGGATGCTCTGGAAAACTCATTTGCACCACTACTGTGCCTTGGAATGTTAGTTGGAGT---------------------------AATAAATCCATGAATGACATTTGGAAT---AACATGACCTGGATGGAGTGGGAAAGAGAGATTGAC------AATTATACAAACATAATATACACCTTACTTGAAGAATCGCAGAACCAACAAGATAAGAATGAACAGGAATTATTGGAATTGGATAAATGGGCAGGTTTGTGGAATTGGTTTAGCATAACAAATTGGCTGTGGTACATAAAAATATTCATAATGATAGTAGGAGGCTTAATAGGTTTAAGAATAGTTTTTACTGTATTTTCTATAGTGAATAGAGTTAGGCAGGGATATTCACCATTATCGTTTCAGACCCGC---TTCCCAACCTCGAGGGGA------CTCGACAGGCCCGAAGGAATCGAAGAAGAAGGTGGAGACAGAGACAGAGACAGATCCAGGCCATTAGTGGATGGATTCTTAGCAATTATCTGGGTCGACCTGCGGAGCCTGTGCCTCTTCAGCTACCATCGCTTGAGAGACTTACTCTTGATTGTAGCGAGGATTGTGGAACTTCTGGGACGCAGG---------------GGGTGGGAAGCCTTCAAATATTGGTGG---AATCTCCTGCGGTATTGG---------------------------------------------------AGCCAGGAACTAAGGAATAGTGCTATTAGCTTGCTTAATGCCACAGCCATAGCAGTAGCTGAGGGAACAGATAGGGTGGTAGAAGTATTACAAAGA------------------GCTTTTAGAGCTGTTATACACATACCTAGAAGAATAAGACAGGGCTTAGAAAGGTTTTTGCTATAA

2.04013240.ADARC.GU330440 ATGAGAGTGAAGGGGATCATGAGGAATTATCAGCACTTA---------TGGAGATGGGGC------------------------ATGATGCTCCTTGGGATAATCATGATC------------TGTAGTGCTGCA---------GAACAATTGTGGGTCACAATCTATTATGGGGTACCTGTGTGGAAAGAAGCAACCACCACTCTATTTTGTGCATCAAATGCTAAAGCATATGATACAGAGGTACATAAT---GTCTGGGCCACACATGCCTGTGTACCCACAGACCCCAACCCACAAGAAGTAAGATTG---GAAAATGTGACAGAAAATTTTAACATGTGGAAAAATAATATGGTAGAACAGATGCATGAGGATATAATTAGCCTATGGGATCAAAGCCTAAAACCATGTGTGAAATTAACCCCACTCTGTGTTACTTTAAACTGCACTGATGCTAATACCACTAATACTAATGCCAAT------------------------------------------------------------------------------AGTACTAATAACAGTAGCTTGGGAACAATGGAGAAAGGAGAAATAAAAAACTGCTCTTTCAACATCACC---ACAAACCTAAGAGAT------AAGGTGCAGAAAGAATATGCACTTTTTTATAACCTTGATGTAGTGCCAATAAAGGGTGAGGAT---------------------------AATACTAGC---------------------------TATAGGTTGATAAGTTGTAATACCTCAGTCATTACACAGGCCTGTCCAAAGGTATCCTTTGAGCCAATTCCCATACATTATTGTACTCCAGCTGGTTTTGCGATTCTACAATGT---AATGATAAGAAATTCAATGGATCAGGACCATGTACAAATGTCAGCACAGTACAATGTACACATGGAATTAGGCCAGTAGTATCAACTCAACTGCTGTTAAATGGCAGTCTAGCAGAAAAA---GAGGTAGTAATTAGGTCTGAGAATTTCACAAATAATGCTAAAACCATAATAGTACAGCTAAATGAATCTGTAGTAATTAATTGTACAAGACCCAACAACAATACAAGAAAAAGTATACCTATAGGA------------CCAGGAAGA---GCATTTTATGCAACAGGAGAAATAATAGGAGATATAAGACAAGCACATTGTAACGTT------AGTACAAAAGCCTGGAAGGAAGCTTTACAACAGGTAGCTATAAAACTATCA---GAACAATTTGGG------------AATAAAACA---ATAGTCTTTAATCAA---------TCCTCAGGAGGAGACCCAGAAGTTGTAATGCACAGTTTTAATTGTAGAGGGGAATTTTTCTACTGTAATACAACAGGACTGTTTAATAATACTTGGGGGTTT---------AATAGTACTTGG---------------------GATGCTATTAATGTGCCAGAA------------------AATGACACA------------------------------ATCACACTCCCATGCAGAATAAAACAAATTGTAAACATGTGGCAGGAAGTAGGAAAAGCAATGTATGCCCCTCCCATCAGAGGACAAATTAATTGTTCATCAAATATTACAGGGCTGCTATTAACAAGAGATGGTGGT------------------AATACCACA---------------------AATAACACT---GAGGTCTTCAGACCTGGAGGAGGAGATATGAGAGACAATTGGAGA---AGTGAATTATATAAATATAAAGTAGTAAAAATTGAACCA---TTAGGAATAGCACCC---ACCAAGGCAAAGAGAAGAGTGGTGCAGAGAGAA---AAAAGAGCAGTG---GGA---ATAGGA---GCTTTG---TTCCTT---GGG---------TTCTTGGGA---GCAGCAGGAAGCACTATGGGCGCAGCGTCGATG---ACGCTGACGGTACAGGCCAGACTATTATTGTCTGGTATAGTGCAACAGCAGAACAATTTGCTGAGAGCTATTGAGGCGCAACAGCATCTGTTGCAACTCACAGTCTGGGGCATCAAGCAGCTCCAGGCAAGA---GTCCTGGCTGTGGAAAGATACCTAAGGGATCAACAGCTCCTGGGGATTTGGGGATGCTCTGGAAAACTCATTTGCACCACTGCTGTGCCTTGGAATGTTAGTTGGAGT---------------------------AATAAATCCATGAATGACATTTGGAAT---AACATGACCTGGATGGAGTGGGAAAGAGAGATTGAC------AATTATACAAACATAATATACACCTTACTTGAAGAATCGCAGAACCAACAAGATAAGAATGAACAGGAATTATTGGAATTGGATAAATGGGCAAGTTTGTGGAATTGGTTTAGCATAACAAATTGGCTGTGGTACATAAAAATATTCATAATGATAGTAGGAGGCTTAATAGGTTTAAGAATAGTTTTTACTGTATTTTCTATAGTGAATAGAGTTAGGCAGGGATATTCACCATTATCGTTTCAGACCCGC---CTCCCAACTTCGAGGGGA------CTCGACAGGCCCGAAGGAATCGAAGAAGAAGGTGGAGACAGAGACAGAGACAGATCCAGGCCATTAGTGGATGGATTCTTAGCAATTATCTGGGTCGACCTGCGGAGCCTGTGCCTCTTCAGCTACCATCGCTTGAGAGACTTACTCTTGATTGTAGCGAGGATTGTGGAACTTCTGGGACGCAGG---------------GGGTGGGAAGCCCTCAAATATTGGTGG---AATCTCCTGCGGTATTGG---------------------------------------------------AGCCAGGAACTAAGGAATAGTGCTATTAGCTTGCTTAATGCCACAGCCATAGCAGTAGCTGAGGGAACAGATAGGGTGTTAGAAGTATTACAAAGA------------------GCTTTTAGAGCTGTTATACACATACCTAGAAGAATAAGACAGGGCTTAGAAAGGTTTTTGCTATAA

2.04013240.ADARC.GU330441 ATGAGAGTGAAGGGGATCATGAGGAATTATCAGCACTTA---------TGGAGATGGGGC------------------------ATGATGCTCCTTGGGATAATCATGATC------------TGTAGTGCTGCA---------GAACAATTGTGGGTCACAATCTATTATGGGGTACCTGTGTGGAAAGAAGCAACCACCACTCTATTTTGTGCATCAAATGCTAAAGCATATGATACAGAGGTACATAAT---GTCTGGGCCACACATGCCTGTGTACCCACAGACCCCAACCCACAAGAAGTAAGATTG---GAAAATGTGACAGAAAATTTTAACATGTGGAAAAATAATATGGTAGAACAGATGCATGAGGATATAATTAGCCTATGGGATCAAAGCCTAAAACCATGTGTGAAATTAACCCCACTCTGTGTTACTTTAAACTGCACTGATGCTAATACCACTAATACTAATGCCAAT------------------------------------------------------------------------------AGTACTAATAACAGTAGCTTGGGAACAATGGAGAAAGGAGAAATAAAAAACTGCTCTTTCAACATCACC---ACAAACCTAAGAGAT------AAGGTGCAGAAAGAATATGCACTTTTTTATAACCTTGATGTAGTGCCAATAAAGGGTGAGGAT---------------------------AATACTAGC---------------------------TATAGGTTGATAAGTTGTAATACCTCAGTCATTACACAGGCCTGTCCAAAGGTATCCTTTGAGCCAATTCCCATACATTATTGTACTCCAGCTGGTTTTGCGATTCTACAATGT---AATGATAAGAAATTCAATGGATCAGGACCATGTACAAATGTCAGCACAGTACAATGTACACATGGAATTAGGCCAGTAGTATCAACTCAACTGCTGTTAAATGGCAGTCTAGCAGAAAAA---GAGGTAGTAATTAGGTCTGAGAATTTCACAAATAATGCTAAAACCATAATAGTACAGCTAAATGAATCTGTAGTAATTAATTGTACAAGACCCAACAACAATACAAGAAAAAGTATACCTATAGGA------------CCAGGAAGA---GCATTTTATGCAACAGGAGAAATAATAGGAGATATAAGACAAGCACATTGTAACGTT------AGTACAAAAGCCTGGAAGGAAGCTTTACAACAGGTAGCTATAAAACTATCA---GAACAATTTGGG------------AATAAAACA---ATAGTCTTTAATCAA---------TCCTCAGGAGGAGACCCAGAAGTTGTAATGCACAGTTTTAATTGTAGAGGGGAATTTTTCTACTGTAATACAACAGGACTGTTTAATAATACTTGGGGGTTT---------AATAGTACTTGG---------------------GATGCTATTAATGTGCCAGAA------------------AATGACACA------------------------------ATCACACTCCCATGCAGAATAAAACAAATTGTAAACATGTGGCAGGAAGTAGGAAAAGCAATGTATGCCCCTCCCATCAGAGGACAAATTAATTGTTCATCAAATATTACAGGGCTGCTATTAACAAGAGATGGTGGT------------------AATACCACA---------------------AATAACACT---GAGGTCTTCAGACCTGGAGGAGGAGATATGAGAGACAATTGGAGA---AGTGAATTATATAAATATAAAGTAGTAAAAATTGAACCA---TTAGGAATAGCACCC---ACCAAGGCAAAGAGAAGAGTGGTGCAGAGAGAA---AAAAGAGCAGTG---GGA---ATAGGA---GCTTTG---TTCCTT---GGG---------TTCTTGGGA---GCAGCAGGAAGCACTATGGGCGCAGCGTCGATG---ACGCTGACGGTACAGGCCAGACTATTATTGTCTGGTATAGTGCAACAGCAGAACAATTTGCTGAGAGCTATTGAGGCGCAACAGCATCTGTTGCAACTCACAGTCTGGGGCATCAAGCAGCTCCAGGCAAGA---GTCCTGGCTGTGGAAAGATACCTAAGGGATCAACAGCTCCTGGGGATTTGGGGATGCTCTGGAAAACTCATTTGCACCACTGCTGTGCCTTGGAATGTTAGTTGGAGT---------------------------AATAAATCCATGAATGACATTTGGAAT---AACATGACCTGGATGGAGTGGGAAAGAGAGATTGAC------AATTATACAAACATAATATACACCTTACTTGAAGAATCGCAGAACCAACAAGATAAGAATGAACAGGAATTATTGGAATTGGATAAATGGGCAAGTTTGTGGAATTGGTTTAGCATAACAAATTGGCTGTGGTACATAAAAATATTCATAATGATAGTAGGAGGCTTAATAGGTTTAAGAATAGTTTTTACTGTATTTTCTATAGTGAATAGAGTTAGGCAGGGATATTCACCATTATCGTTTCAGACCCGC---CTCCCAACTTCGAGGGGA------CTCGACAGGCCCGAAAGAATCGAAGAAGAAGGTGGAGACAGAGACAGAGACAGATCCAGGCCATTAGTGGATGGATTCTTAGCAATTATCTGGGTCGACCTGCGGAGCCTGTGCCTCTTCAGCTACCATCGCTTGAGAGACTTACTCTTGATTGTAGCGAGGATTGTGGAACTTCTGGGACGCAGG---------------GGGTGGGAAGCCCTCAAATATTGGTGG---AATCTCCTGCGGTATTGG---------------------------------------------------AGCCAGGAACTAAGGAATAGTGCTATTAGCTTGCTTAATGCCACAGCCATAGCAGTAGCTGAGGGAACAGATAGGGTGTTAGAAGTATTACAAAGA------------------GCTTTTAGAGCTGTTATACACATACCTAGAAGAATAAGACAGGGCTTAGAAAGGTTTTTGCTATAA

2.04013240.ADARC.GU330442 ATGAGAGTGAAGGGGATCATGAGGAATTATCAGTACTTA---------TGGAGATGGGGC------------------------ATGATGCTCCTTGGGATAATCATGATC------------TGTAGTGCTGCA---------GAACAATTGTGGGTCACAATCTATTATGGGGTACCTGTGTGGAAAGAAGCAACCACCACTCTATTTTGTGCATCAAATGCTAAAGCATATGATACAGAGGTACATAAT---GTCTGGGCCACACATGCCTGTGTACCCACAGACCCCAACCCACAAGAAGTAAGATTG---GAAAATGTGACAGAAAATTTTAACATGTGGAAAAATAATATGGTAGAACAGATGCATGAGGATATAATTAGCCTATGGGATCAAAGCCTAAAACCATGTGTGAAATTAACCCCACTCTGTGTTACTTTAAACTGCACTGATGCTAATACCACTAATACTAATGCCAAT------------------------------------------------------------------------------AGTACTAATAACAGTAGCTTGGGAACAATGGAGAAAGGAGAAATAAAAAACTGCTCTTTCAACATCACC---ACAAACCTAAGAGAT------AAGGTGCAGAAAGAATATGCACTTTTTTATAACCTTGATGTAGTGCCAATAAAGGGTGAGGAT---------------------------AATACTAGC---------------------------TATAGGTTGATAAGTTGTAATACCTCAGTCATTACACAGGCCTGTCCAAAGGTATCCTTTGAGCCAATTCCCATACATTATTGTACTCCAGCTGGTTTTGCGATTCTACAATGT---AATGATAAGAAATTCAATGGATCAGGACCATGTACAAATGTCAGCACAGTACAATGTACACATGGAATTAGGCCAGTAGTATCAACTCAACTGCTGTTAAATGGCAGTCTAGCAGAAAAA---GAGGTAGTAATTAGGTCTGAGAATTTCACAAATAATGCTAAAACCATAATAGTACAGCTAAATGAATCTGTAGTAATTAATTGTACAAGACCCAACAACAATACAAGAAAAAGTATACCTATAGGA------------CCAGGAAGA---GCATTTTATGCAACAGGAGAAATAATAGGAGATATAAGACAAGCACATTGTAACGTT------AGTACAAAAGCCTGGAAGGAAGCTTTACAACAGGTAGCTATAAAACTATCA---GAACAATTTGGG------------AATAAAACA---ATAGTCTTTAATCAA---------TCCTCAGGAGGAGACCCAGAAGTTGTAATGCATAGTTTTAATTGTAGAGGGGAATTTTTCTACTGTAATACAACAGGACTGTTTAATAATACTTGGGGGTTT---------AATAGTACTTGG---------------------GATGCTATTAATGTGCCAGAA------------------AATGACACA------------------------------ATCACACTCCCATGCAGAATAAAACAAATTGTAAACATGTGGCAGGAAGTAGGAAAAGCAATGTATGCCCCTCCCATCAGAGGACGACTTAATTGTTCATCAAATATTACAGGGCTGCTATTAACAAGAGATGGTGGT------------------AATACCACA---------------------AATAACACT---GAGGTCTTCAGACCTGGAGGAGGAGATATGAGAGACAATTGGAGA---AGTGAATTATATAAATATAAAGTAGTAAAAATTGAACCA---TTAGGAATAGCACCC---ACCAAGGCAAAGAGAAGAGTGGTGCAGAGAGAA---AAAAGAGCAGTG---GGA---ATAGGA---GCTTTG---TTCCTT---GGG---------TTCTTGGGA---GCAGCAGGAAGCACTATGGGCGCAGCGTCGATG---ACGCTGACGGTACAGGCCAGACTATTATTGTCTGGTATAGTGCAACAGCAGAACAATTTGCTGAGAGCTATTGAGGCGCAACAGCATCTGTTGCAACTCACAGTCTGGGGCATCAAGCAGCTCCAGGCAAGA---GTCCTGGCTGTGGAAAGATACCTAAGAGATCAACAGCTCCTGGGGATTTGGGGATGCTCTGGAAAACTCATTTGCACCACTACTGTGCCTTGGAATGTTAGTTGGAGT---------------------------AATAAATCCATGAATGACATTTGGAAT---AACATGACCTGGATGGAGTGGGAAAGAGAGATTGAC------AATTATACAAACATAATATACACCTTACTTGAAGAATCGCAGAACCAACAAGATAAGAATGAACAGGAATTATTGGAATTGGATAAATGGGCAAGTTTGTGGAATTGGTTTAGCATAACAAATTGGCTGTGGTACATAAAAATATTCATAATGATAGTAGGAGGCTTAATAGGTTTAAGAATAGTTTTTACTGTATTTTCTATAGTGAATAGAGTTAGGCAGGGATATTCACCATTATCGTTTCAGACCCGC---TTCCCAACCTCGAGGGGA------CTCGACAGGCCCGAAGGAATCGAAGAAGAAGGTGGAGACAGAGACAGAGACAGATCCAGGCCATTAGTGGATGGATTCTTAGCAATTATCTGGGTCGACCTGCGGAGCCTGTGCCTCTTCAGCTACCATCGCTTGAGAGACTTACTCTTGATTGTAGCGAGGATTGTGGAACTTCTGGGACGCAGG---------------GGGTGGGAAGCCCTCAAATATTGGTGG---AATCTCCTGCGGTATTGG---------------------------------------------------AGCCAGGAACTAAGGAATAGTGCTATTAGCTTGCTTAATGCCACAGCCATAGCAGTAGCTGAGGGAACAGATAGGGTGTTAGAAGTATTACAAAGA------------------GCTTTTAGAGCTGTTATACACATACCTAGAAGAATAAGACAGGGCTTAGAAAGGTTTTTGCTATAA

2.04013240.ADARC.GU330443 ATGAGAGTGAAGGGGATCATGAGGAATTATCAGCACTTA---------TGGAGATGGGGC------------------------ATGATGCTCCTTGGGATAATCATGATC------------TGTAGTGCTGCA---------GAACAATTGTGGGTCACAATCTATTATGGGGTACCTGTGTGGAAAGAAGCAACCACCACTCTATTTTGTGCATCAAATGCTAAAGCATATGATACAGAGGTACATAAT---GTCTGGGCCACACATGCCTGTGTACCCACAGACCCCAACCCACAAGAAGTAAGATTG---GAAAATGTGACAGAAAATTTTAACATGTGGAAAAATAATATGGTAGAACAGATGCATGAGGATATAATTAGCCTATGGGATCAAAGCCTAAAACCATGTGTGAAATTAACCCCACTCTGTGTTACTTTAAACTGCACTGATGCTAATACCACTAATACTAATGCCAAT------------------------------------------------------------------------------AGTACTAATAACAGTAGCTTGGGAACAATGGAGAAAGGAGAAATAAAAAACTGCTCTTTCAACATCACC---ACAAACCTAAGAGAT------AAGGTGCAGAAAGAATATGCACTTTTTTATAACCTTGATGTAGTGCCAATAAAGGGTGAGGAT---------------------------AATACTAGC---------------------------TATAGGTTGATAAGTTGTAATACCTCAGTCATTACACAGGCCTGTCCAAAGGTATCCTTTGAGCCAATTCCCATACATTATTGTACTCCAGCTGGTTTTGCGATTCTACAATGT---AATGATAAGAAATTCAATGGATCAGGACCATGTACAAATGTCAGCACAGTACAATGTACACATGGAATTAGGCCAGTAGTATCAACTCAACTGCTGTTAAATGGCAGTCTAGCAGAAAAA---GAGGTAGTAATTAGGTCTGAGAATTTCACAAATAATGCTAAAACCATAATAGTACAGCTAAATGAATCTGTAGTAATTAATTGTACAAGACCCAACAACAATACAAGAAAAAGTATACCTATAGGA------------CCAGGAAGA---GCATTTTATGCAACAGGAGAAATAATAGGAGATATAAGACAAGCACATTGTAACGTT------AGTACAAAAGCCTGGAAGGAAGCTTTACAACAGGTAGCTATAAAACTATCA---GAACAATTTGGG------------AATAAAACA---ATAGTCTTTAATCAA---------TCCTCAGGAGGAGACCCAGAAGTTGTAATGCACAGTTTTAATTGTAGAGGGGAATTTTTCTACTGTAATACAACAGGACTGTTTAATAATACTTGGGGGTTT---------AATAGTACTTGG---------------------GATGCTATTAATGTGCCAGAA------------------AATGACACA------------------------------ATCACACTCCCATGCAGAATAAAACAAATTGTAAACATGTGGCAGGAAGTAGGAAAAGCAATGTATGCCCCTCCCATCAGAGGACGACTTAATTGTTCATCAAATATTACAGGGCTGCTATTAACAAGAGATGGTGGT------------------AATACCACA---------------------AATAACACT---GAGGTCTTCAGACCTGGAGGAGGAGATATGAGAGACAATTGGAGA---AGTGAATTATATAAATATAAAGTAGTAAAAATTGAACCA---TTAGGAATAGCACCC---ACCAAGGCAAAGAGAAGAGTGGTGCAGAGAGAA---AAAAGAGCAGTG---GGA---ATAGGA---GCTTTG---TTCCTT---GGG---------TTCTTGGGA---GCAGCAGGAAGCACTATGGGCGCAGCGTCGATG---ACGCTGACGGTACAGGCCAGACTATTATTGTCTGGTATAGTGCAACAGCAGAACAATTTGCTGAGAGCTATTGAGGCGCAACAGCATCTGTTGCAACTCACAGTCTGGGGCATCAAGCAGCTCCAGGCAAGA---GTCCTGGCTGTGGAAAGATACCTAAGAGATCAACAGCTCCTGGGGATTTGGGGATGCTCTGGAAAACTCATTTGCACCACTACTGTGCCTTGGAATGTTAGTTGGAGT---------------------------AATAAATCCATGAATGACATTTGGAAT---AACATGACCTGGATGGAGTGGGAAAGAGAGATTGAC------AATTATACAAACATAATATACACCTTACTTGAAGAATCGCAGAACCAACAAGATAAGAATGAACAGGAATTATTGGAATTGGATAAATGGGCAAGTTTGTGGAATTGGTTTAGCATAACAAATTGGCTGTGGTACATAAAAATATTCATAATGATAGTAGGAGGCTTAATAGGTTTAAGAATAGTTTTTACTGTATTTTCTATAGTGAATAGAGTTAGGCAGGGATATTCACCATTATCGTTTCAGACCCGC---TTCCCAACCTCGAGGGGA------CTCGACAGGCCCGAAGGAATCGAAGAAGAAGGTGGAGACAGAGACAGAGACAGATCCAGGCCATTAGTGGATGGATTCTTAGCAATTATCTGGGTCGACCTGCGGAGCCTGTGCCTCTTCAGCTACCATCGCTTGAGAGACTTACTCTTGATTGTAGCGAGGATTGTGGAACTTCTGGGACGCAGG---------------GGGTGGGAAGCCCTCAAATATTGGTGG---AATCTCCTGCGGTATTGG---------------------------------------------------AGCCAGGAACTAAGGAATAGTGCTATTAGCTTGCTTAATGCCACAGCCATAGCAGTAGCTGAGGGAACAGATAGGGTGGTAGAAGTATTACAAAGA------------------GCTTTTAGAGCTGTTATACACATACCTAGAAGAATAAGACAGGGCTTAGAAAGGTTTTTGCTATAA

2.04013240.ADARC.GU330444 ATGAGAGTGAAGGGGATCATGAGGAATTATCAGCACTTA---------TGGAGATGGGGC------------------------ATGATGCTCCTTGGGATAATCATGATC------------TGTAGTGCTGCA---------GAACAATTGTGGGTCACAATCTATTATGGGGTACCTGTGTGGAAAGAAGCAACCACCACTCTATTTTGTGCATCAAATGCTAAAGCATATGATACAGAGGTACATAAT---GTCTGGGCCACACATGCCTGTGTACCCACAGACCCCAACCCACAAGAAGTAAGATTG---GAAAATGTGACAGAAAATTTTAACATGTGGAAAAATAATATGGTAGAACAGATGCATGAGGATATAATTAGCCTATGGGATCAAAGCCTAAAACCATGTGTGAAATTAACCCCACTCTGTGTTACTTTAAACTGCACTGATGCTAATACCACTAATACTAATGCCAAT------------------------------------------------------------------------------AGTACTAATAACAGTAGCTTGGGAACAATGGAGAAAGGAGAAATAAAAAACTGCTCTTTCAACATCACC---ACAAACCTAAGAGAT------AAGGTGCAGAAAGAATATGCACTTTTTTATAACCTTGATGTAGTGCCAATAAAGGGTGAGGAT---------------------------AATACTAGC---------------------------TATAGGTTGATAAGTTGTAATACCTCAGTCATTACACAGGCCTGTCCAAAGGTATCCTTTGAGCCAATTCCCATACATTATTGTACTCCAGCTGGTTTTGCGATTCTACAATGT---AATGATAAGAAATTCAATGGATCAGGACCATGTACAAATGTCAGCACAGTACAATGTACACATGGAATTAGGCCAGTAGTATCAACTCAACTGCTGTTAAATGGCAGTCTAGCAGAAAAA---GAGGTAGTAATTAGGTCTGAGAATTTCACAAATAATGCTAAAACCATAATAGTACAGCTAAATGAATCTGTAGTAATTAATTGTACAAGACCCAACAACAATACAAGAAAAAGTATACCTATAGGA------------CCAGGAAGA---GCATTTTATGCAACAGGAGAAATAATAGGAGATATAAGACAAGCACATTGTAACGTT------AGTACAAAAGCCTGGAAGGAAGCTTTACAACAGGTAGCTATAAAACTATCA---GAACAATTTGGG------------AATAAAACA---ATAGTCTTTAATCAA---------TCCTCAGGAGGAGACCCAGAAGTTGTAATGCACAGTTTTAATTGTAGAGGGGAATTTTTCTACTGTAATACAACAGGACTGTTTAATAATACTTGGGGGTTT---------AATAGTACTTGG---------------------GATGCTATTAATGTGCCAGAA------------------AATGACACA------------------------------ATCACACTCCCATGCAGAATAAAACAAATTGTAAACATGTGGCAGGAAGTAGGAAAAGCAATGTATGCCCCTCCCATCAGAGGACGACTTAATTGTTCATCAAATATTACAGGGCTGCTATTAACAAGAGATGGTGGT------------------AATACCACA---------------------AATAACACT---GAGGTCTTCAGACCTGGAGGAGGAGATATGAGAGACAATTGGAGA---AGTGAATTATATAAATATAAAGTAGTAAAAATTGAACCA---TTAGGAATAGCACCC---ACCAAGGCAAAGAGAAGAGTGGTGCAGAGAGAA---AAAAGAGCAGTG---GGA---ATAGGA---GCTTTG---TTCCTT---GGG---------TTCTTGGGA---GCAGCAGGAAGCACTATGGGCGCAGCGTCGATG---ACGCTGACGGTACAGGCCAGACTATTATTGTCTGGTATAGTGCAACAGCAGAACAATTTGCTGAGAGCTATTGAGGCGCAACAGCATCTGTTGCAACTCACAGTCTGGGGCATCAAGCAGCTCCAGGCAAGA---GTCCTGGCTGTGGAAAGATACCTAAGAGATCAACAGCTCCTGGGGATTTGGGGATGCTCTGGAAAACTCATTTGCACCACTACTGTGCCTTGGAATGTTAGTTGGAGT---------------------------AATAAATCCATGAATGACATTTGGAAT---AACATGACCTGGATGGAGTGGGAAAGAGAGATTGAC------AATTATACAAACATAATATACACCTTACTTGAAGAATCGCAGAACCAACAAGATAAGAATGAACAGGAATTATTGGAATTGGATAAATGGGCAAGTTTGTGGAATTGGTTTAGCATAACAAATTGGCTGTGGTACATAAAAATATTCATAATGATAGTAGGAGGCTTAATAGGTTTAAGAATAGTTTTTACTGTATTTTCTATAGTGAATAGAGTTAGGCAGGGATATTCACCATTATCGTTTCAGACCCGC---TTCCCAACCTCGAGGGGA------CTCGACAGGCCCGAAGGAATCGAAGAAGAAGGTGGAGACAGAGACAGAGACAGATCCAGGCCATTAGTGGATGGATTCTTAGCAATTATCTGGGTCGACCTGCGGAGCCTGTGCCTCTTCAGCTACCATCGCTTGAGAGACTTACTCTTGATTGTAGCGAGGATTGTGGAACTTCTGGGACGCAGG---------------GGGTGGGAAGCCCTCAAATATTGGTGG---AATCTCCTGCGGTATTGG---------------------------------------------------AGCCAGGAACTAAGGAATAGTGCTATTAGCTTGCTTAATGCCACAGCCATAGCAGTAGCTGAGGGAACAGATAGGGTGGTAGAAGTATTACAAAGA------------------GCTTTTAGAGCTGTTATACACATACCTAGAAGAATAAGACAGGGCTTAGAAAGGTTTTTGCTATAA

2.04013240.ADARC.GU330445 ATGAGAGTGAAGGGGATCATGAGGAATTATCAGCACTTA---------TGGAGATGGGGC------------------------ATGATGCTCCTTGGGATAATCATGATC------------TGTAGTGCTGCA---------GAACAATTGTGGGTCACAATCTATTATGGGGTACCTGTGTGGAAAGAAGCAACCACCACTCTATTTTGTGCATCAAATGCTAAAGCATATGATACAGAGGTACATAAT---GTCTGGGCCACACATGCCTGTGTACCCACAGACCCCAACCCACAAGAAGTAAGATTG---GAAAATGTGACAGAAAATTTTAACATGTGGAAAAATAATATGGTAGAGCAGATGCATGAGGATATAATTAGCCTATGGGATCAAAGCCTAAAACCATGTGTGAAATTAACCCCACTCTGTGTTACTTTAAACTGCACTGATGCTAATACCACTAATACTAATGCCAAT------------------------------------------------------------------------------AGTACTAATAACAGTAGCTTGGGAACAATGGAGAAAGGAGAAATAAAAAACTGCTCTTTCAACATCACC---ACAAACCTAAGAGAT------AAGGTGCAGAAAGAATATGCACTTTTTTATAACCTTGATGTAGTGCCAATAAAGGGTGAGGAT---------------------------AATACTAGC---------------------------TATAGGTTGATAAGTTGTAATACCTCAGTCATTACACAGGCCTGTCCAAAGGTATCCTTTGAGCCAATTCCCATACATTATTGTACTCCAGCTGGTTTTGCGATTCTACAATGT---AATGATAAGAAATTCAATGGATCAGGACCATGTACAAATGTCAGCACAGTACAATGTACACATGGAATTAGGCCAGTAGTATCAACTCAACTGCTGTTAAATGGCAGTCTAGCAGAAAAA---GAGGTAGTAATTAGGTCTGAGAATTTCACAAATAATGCTAAAACCATAATAGTACAGCTAAATGAATCTGTAGTAATTAATTGTACAAGACCCAACAACAATACAAGAAAAAGTATACCTATAGGA------------CCAGGAAGA---GCATTTTATGCAACAGGAGAAATAATAGGAGATATAAGACAAGCACATTGTAACGTT------AGTACAAAAGCCTGGAAGGAAGCTTTACAACAGGTAGCTATAAAACTATCA---GAACAATTTGGG------------AATAAAACA---ATAGTCTTTAATCAA---------TCCTCAGGAGGAGACCCAGAAGTTGTAATGCACAGTTTTAATTGTAGAGGGGAATTTTTCTACTGTAATACAACAGGACTGTTTAATAATACTTGGGGGTTT---------AATAGTACTTGG---------------------GATGCTATTAATGTGCCAGAA------------------AATGACACA------------------------------ATCACACTCCCATGCAGAATAAAACAAATTGTAAACATGTGGCAGGAAGTAGGAAAAGCAATGTATGCCCCTCCCATCAGAGGACGACTTAATTGTTCATCAAATATTACAGGGCTGCTATTAACAAGAGATGGTGGT------------------AATACCACA---------------------AATAACACT---GAGGTCTTCAGACCTGGAGGAGGAGATATGAGAGACAATTGGAGA---AGTGAATTATATAAATATAAAGTAGTAAAAATTGAACCA---TTAGGAATAGCACCC---ACCAAGGCAAAGAGAAGAGTGGTGCAGAGAGAA---AAAAGAGCAGTG---GGA---ATAGGA---GCTTTG---TTCCTT---GGG---------TTCTTGGGA---GCAGCAGGAAGCACTATGGGCGCAGCGTCGATG---ACGCTGACGGTACAGGCCAGACTATTATTGTCTGGTATAGTGCAACAGCAGAACAATTTGCTGAGAGCTATTGAGGCGCAACAGCATCTGTTGCAACTCACAGTCTGGGGCATCAAGCAGCTCCAGGCAAGA---GTCCTGGCTGTGGAAAGATACCTAAGAGATCAACAGCTCCTGGGGATTTGGGGATGCTCTGGAAAACTCATTTGCACCACTACTGTGCCTTGGAATGTTAGTTGGAGT---------------------------AATAAATCCATGAATGACATTTGGAAT---AACATGACCTGGATGGAGTGGGAAAGAGAGATTGAC------AATTATACAAACATAATATACACCTTACTTGAAGAATCGCAGAACCAACAAGATAAGAATGAACAGGAATTATTGGAATTGGATAAATGGGCAAGTTTGTGGAATTGGTTTAGCATAACAAATTGGCTGTGGTACATAAAAATATTCATAATGATAGTAGGAGGCTTAATAGGTTTAAGAATAGTTTTTACTGTATTTTCTATAGTGAATAGAGTTAGGCAGGGATATTCACCATTATCGTTTCAGACCCGC---TTCCCAACCTCGAGGGGA------CTCGACAGGCCCGAAGGAATCGAAGAAGAAGGTGGAGACAGAGACAGAGACAGATCCAGGCCATTAGTGGATGGATTCTTAGCAATTATCTGGGTCGACCTGCGGAGCCTGTGCCTCTTCAGCTACCATCGCTTGAGAGACTTACTCTTGATTGTAGCGAGGATTGTGGAACTTCTGGGACGCAGG---------------GGGTGGGAAGCCCTCAAATATTGGTGG---AATCTCCTGCGGTATTGG---------------------------------------------------AGCCAGGAACTAAGGAATAGTGCTATTAGCTTGCTTAATGCCACAGCCATAGCAGTAGCTGAGGGAACAGATAGGGTGTTAGAAGTATTACAAAGA------------------GCTTTTAGAGCTGTTATACACATACCTAGAAGAATAAGACAGGGCTTAGAAAGGTTTTTGCTATAA

2.04013240.ADARC.GU330446 ATGAGAGTGAAGGGGATCATGAGGAATTATCAGCACTTA---------TGGAGATGGGGC------------------------ATGATGCTCCTTGGGATAATCATGATC------------TGTAGTGCTGCA---------GAACAATTGTGGGTCACAATCTATTATGGGGTACCTGTGTGGAAAGAAGCAACCACCACTCTATTTTGTGCATCAAATGCTAAAGCATATGATACAGAGGTACATAAT---GTCTGGGCCACACATGCCTGTGTACCCACAGACCCCAACCCACAAGAAGTAAGATTG---GAAAATGTGACAGAAAATTTTAACATGTGGAAAAATAATATGGTAGAACAGATGCATGAGGATATAATTAGCCTATGGGATCAAAGCCTAAAACCATGTGTGAAATTAACCCCACTCTGTGTTACTTTAAACTGCACTGATGCTAATACCACTAATACTAATGCCAAT------------------------------------------------------------------------------AGTACTAATAACAGTAGCTTGGGAACAATGGAGAAAGGAGAAATAAAAAACTGCTCTTTCAACATCACC---ACAAACCTAAGAGAT------AAGGTGCAGAAAGAATATGCACTTTTTTATAACCTTGATGTAGTGCCAATAAAGGGTGAGGAT---------------------------AATACTAGC---------------------------TATAGGTTGATAAGTTGTAATACCTCAGTCATTACACAGGCCTGTCCAAAGGTATCCTTTGAGCCAATTCCCATACATTATTGTACTCCAGCTGGTTTTGCGATTCTACAATGT---AATGATAAGAAATTCAATGGATCAGGACCATGTACAAATGTCAGCACAGTACAATGTACACATGGAATTAGGCCAGTAGTATCAACTCAACTGCTGTTAAATGGCAGTCTAGCAGAAAAA---GAGGTAGTAATTAGGTCTGAGAATTTCACAAATAATGCTAAAACCATAATAGTACAGCTAAATGAATCTGTAGTAATTAATTGTACAAGACCCAACAACAATACAAGAAAAAGTATACCTATAGGA------------CCAGGAAGA---GCATTTTATGCAACAGGAGAAATAATAGGAGATATAAGACAAGCACATTGTAACGTT------AGTACAAAAGCCTGGAAGGAAGCTTTACAACAGGTAGCTATAAAACTATCA---GAACAATTTGGG------------AATAAAACA---ATAGTCTTTAATCAA---------TCCTCAGGAGGAGACCCAGAAGTTGTAATGCACAGTTTTAATTGTAGAGGGGAATTTTTCTACTGTAATACAACAGGACTGTTTAATAATACTTGGGGGTTT---------AATAGTACTTGG---------------------GATGCTATTAATGTGCCAGAA------------------AATGACACA------------------------------ATCACACTCCCATGCAGAATAAAACAAATTGTAAACATGTGGCAGGAAGTAGGAAAAGCAATGTATGCCCCTCCCATCAGAGGACAACTTAATTGTTCATCAAATATTACAGGGCTGCTATTAACAAGAGATGGTGGT------------------AATACCACA---------------------AATAACACT---GAGGTCTTCAGACCTGGAGGAGGAGATATGAGAGACAATTGGAGA---AGTGAATTATATAAATATAAAGTAGTAAAAATTGAACCA---TTAGGAATAGCACCC---ACCAAGGCAAAGAGAAGAGTGGTGCAGAGAGAA---AAAAGAGCAGTG---GGA---ATAGGA---GCTTTG---TTCCTT---GGG---------TTCTTGGGA---GCAGCAGGAAGCACTATGGGCGCAGCGTCGATG---ACGCTGACGGTACAGGCCAGACTATTATTGTCTGGTATAGTGCAACAGCAGAACAATTTGCTGAGAGCTATTGAGGCGCAACAGCATCTGTTGCAACTCACAGTCTGGGGCATCAAGCAGCTCCAGGCAAGA---GTCCTGGCTGTGGAAAGATACCTAAGGGATCAACAGCTCCTGGGGATTTGGGGATGCTCTGGAAAACTCATTTGCACCACTGCTGTGCCTTGGAATGTTAGTTGGAGT---------------------------AATAAATCCATGAATGACATTTGGAAT---AACATGACCTGGATGGAGTGGGAAAGAGAGATTGAC------AATTATACAAACATAATATACACCTTACTTGAAGAATCGCAGAACCAACAAGATAAGAATGAACAGGAATTATTGGAATTGGATAAATGGGCAAGTTTGTGGAATTGGTTTAGCATAACAAATTGGCTGTGGTACATAAAAATATTCATAATGATAGTAGGAGGCTTAATAGGTTTAAGAATAGTTTTTACTGTATTTTCTATAGTGAATAGAGTTAGGCAGGGATATTCACCATTATCGTTTCAGACCCGC---CTCCCAACTTCGAGGGGA------CTCGACAGGCCCGAAGGAATCGAAGAAGAAGGTGGAGACAGAGACAGAGACAGATCCAGGCCATTAGTGGATGGATTCTTAGCAATTATCTGGGTCGACCTGCGGAGCCTGTGCCTCTTCAGCTACCATCGCTTGAGAGACTTACTCTTGATTGTAGCGAGGATTGTGGAACTTCTGGGACGCAGG---------------GGGTGGGAAGCCCTCAAATATTGGTGG---AATCTCCTGCGGTATTGG---------------------------------------------------AGCCAGGAACTAAGGAATAGTGCTATTAGCTTGCTTAATGCCACAGCCATAGCAGTAGCTGAGGGAACAGATAGGGTGTTAGAAGTATTACAAAGA------------------GCTTTTAGAGCTGTTATACACATACCTAGAAGAATAAGACAGGGCTTAGAAAGGTTTTTGCTATAA

2.04013240.ADARC.GU330447 ATGAGAGTGAAGGGGATCATGAGGAATTATCAGCACTTA---------TGGAGATGGGGC------------------------ATGATGCTCCTTGGGATAATCATGATC------------TGTAGTGCTGCA---------GAACAATTGTGGGTCACAATCTATTATGGGGTACCTGTGTGGAAAGAAGCAACCACCACTCTATTTTGTGCATCAAATGCTAAAGCATATGATACAGAGGTACATAAT---GTCTGGGCCACACATGCCTGTGTACCCACAGACCCCAACCCACAAGAAGTAAGATTG---GAAAATGTGACAGAAAATTTTAACATGTGGAAAAATAATATGGTAGAACAGATGCATGAGGATATAATTAGCCTATGGGATCAAAGCCTAAAACCATGTGTGAAATTAACCCCACTCTGTGTTACTTTAAACTGCACTGATGCTAATACCACTAATACTAATGCCAAT------------------------------------------------------------------------------AGTACTAATAACAGTAGCTTGGGAACAATGGAGAAAGGAGAAATAAAAAACTGCTCTTTCAACATCACC---ACAAACCTAAGAGAT------AAGGTGCAGAAAGAATATGCACTTTTTTATAACCTTGATGTAGTGCCAATAAAGGGTGAGGAT---------------------------AATACTAGC---------------------------TATAGGTTGATAAGTTGTAATACCTCAGTCATTACACAGGCCTGTCCAAAGGTATCCTTTGAGCCAATTCCCATACATTATTGTACTCCAGCTGGTTTTGCGATTCTACAATGT---AATGATAAGAAATTCAATGGATCAGGACCATGTACAAATGTCAGCACAGTACAATGTACACATGGAATTAGGCCAGTAGTATCAACTCAACTGCTGTTAAATGGCAGTCTAGCAGAAAAA---GAGGTAGTAATTAGGTCTGAGAATTTCACAAATAATGCTAAAACCATAATAGTACAGCTAAATGAATCTGTAGTAATTAATTGTACAAGACCCAACAACAATACAAGAAAAAGTATACCTATAGGA------------CCAGGAAGA---GCATTTTATGCAACAGGAGAAATAATAGGAGATATAAGACAAGCACATTGTAACGTT------AGTACAAAAGCCTGGAAGGAAGCTTTACAACAGGTAGCTATAAAACTATCA---GAACAATTTGGG------------AATAAAACA---ATAGTCTTTAATCAA---------TCCTCAGGAGGAGACCCAGAAGTTGTAATGCACAGTTTTAATTGTAGAGGGGAATTTTTCTACTGTAATACAACAGGACTGTTTAATAATACTTGGGGGTTT---------AATAGTACTTGG---------------------GATGCTATTAATGTGCCAGAA------------------AATGACACA------------------------------ATCACACTCCCATGCAGAATAAAACAAATTGTAAACATGTGGCAGGAAGTAGGAAAAGCAATGTATGCCCCTCCCATCAGAGAACGACTTAATTGTTCATCAAATATTACAGGGCTGCTATTAACAAGAGATGGTGGT------------------AATACCACA---------------------AATAACACT---GAGGTCTTCAGACCTGGAGGAGGAGATATGAGAGACAATTGGAGA---AGTGAATTATATAAATATAAAGTAGTAAAAATTGAACCA---TTAGGAATAGCACCC---ACCAAGGCAAAGAGAAGAGTGGTGCAGAGAGAA---AAAAGAGCAGTG---GGA---ATAGGA---GCTTTG---TTCCTT---GGG---------TTCTTGGGA---GCAGCAGGAAGCACTATGGGCGCAGCGTCGATG---ACGCTGACGGTACAGGCCAGACTATTATTGTCTGGTATAGTGCAACAGCAGAACAATTTGCTGAGAGCTATTGAGGCGCAACAGCATCTGTTGCAACTCACAGTCTGGGGCATCAAGCAGCTCCAGGCAAGA---GTCCTGGCTGTGGAAAGATACCTAAGAGATCAACAGCTCCTGGGGATTTGGGGATGCTCTGGAAAACTCATTTGCACCACTACTGTGCCTTGGAATGTTAGTTGGAGT---------------------------AATAAATCCATGAATGACATTTGGAAT---AACATGACCTGGATGGAGTGGGAAAGAGAGATTGAC------AATTATACAAACATAATATACACCTTACTTGAAGAATCGCAGAACCAACAAGATAAGAATGAACAGGAATTATTGGAATTGGATAAATGGGCAAGTTTGTGGAATTGGTTTAGCATAACAAATTGGCTGTGGTACATAAAAATATTCATAATGATAGTAGGAGGCTTAATAGGTTTAAGAATAGTTTTTACTGTATTTTCTATAGTGAATAGAGTTAGGCAGGGATATTCACCATTATCGTTTCAGACCCGC---TTCCCAACCTCGAGGGGA------CTCGACAGGCCCGAAGGAATCGAAGAAGAAGGTGGAGACAGAGACAGAGACAGATCCAGGCCATTAGTGGATGGATTCTTAGCAATTATCTGGGTCGACCTGCGGAGCCTGTGCCTCTTCAGCTACCATCGCTTGAGAGACTTACTCTTGATTGTAGCGAGGATTGTGGAACTTCTGGGACGCAGG---------------GGGTGGGAAGCCCTCAAATATTGGTGG---AATCTCCTGCGGTATTGG---------------------------------------------------AGCCAGGAACTAAGGAATAGTGCTATTAGCTTGCTTAATGCCACAGCCATAGCAGTAGCTGAGGGAACAGATAGGGTGTTAGAAGTATTACAAAGA------------------GCTTTTAGAGCTGTTATACACATACCTAGAAGAATAAGACAGGGCTTAGAAAGGTTTTTGCTATAA

2.04013240.ADARC.GU330448 ATGAGAGTGAAGGGGATCATGAGGAATTATCAGCACTTA---------TGGAGATGGGGC------------------------ATGATGCTCCTTGGGATAATCATGATC------------TGTAGTGCTGCA---------GAACAATTGTGGGTCACAATCTATTATGGGGTACCTGTGTGGAAAGAAGCAACCACCACTCTATTTTGTGCATCAAATGCTAAAGCATATGATACAGAGGTACATAAT---GTCTGGGCCACACATGCCTGTGTACCCACAGACCCCAACCCACAAGAAGTAAGATTG---GAAAATGTGACAGAAAATTTTAACATGTGGAAAAATAATATGGTAGAACAGATGCATGAGGATATAATTAGCCTATGGGATCAAAGCCTAAAACCATGTGTGAAATTAACCCCACTCTGTGTTACTTTAAACTGCACTGATGCTAATACCACTAATACTAATGCCAAT------------------------------------------------------------------------------AGTACTAATAACAGTAGCTTGGGAACAATGGAGAAAGGAGAAATAAAAAACTGCTCTTTCAACATCACC---ACAAACCTAAGAGAT------AAGGTGCAGAAAGAATATGCACTTTTTTATAACCTTGATGTAGTGCCAATAAAGGGTGAGGAT---------------------------AATACTAGC---------------------------TATAGGTTGATAAGTTGTAATACCTCAGTCATTACACAGGCCTGTCCAAAGGTATCCTTTGAGCCAATTCCCATACATTATTGTACTCCAGCTGGTTTTGCGATTCTACAATGT---AATGATAAGAAATTCAATGGATCAGGACCATGTACAAATGTCAGCACAGTACAATGTACACATGGAATTAGGCCAGTAGTATCAACTCAACTGCTGTTAAATGGCAGTCTAGCAGAAAAA---GAGGTAGTAATTAGGTCTGAGAATTTCACAAATAATGCTAAAACCATAATAGTACAGCTAAATGAATCTGTAGTAATTAATTGTACAAGACCCAACAACAATACAAGAAAAAGTATACCTATAGGA------------CCAGGAAGA---GCATTTTATGCAACAGGAGAAATAATAGGAGATATAAGACAAGCACATTGTAACGTT------AGTACAAAAGCCTGGAAGGAAGCTTTACAACAGGTAGCTATAAAACTATCA---GAACAATTTGGG------------AATAAAACA---ATAGTCTTTAATCAA---------TCCTCAGGAGGAGACCCAGAAGTTGTAATGCACAGTTTTAATTGTAGAGGGGAATTTTTCTACTGTAATACAACAGGACTGTTTAATAATACTTGGGGGTTT---------AATAGTACTTGG---------------------GATGCTATTAATGTGCCAGAA------------------AATGACACA------------------------------ATCACACTCCCATGCAGAATAAAACAAATTGTAAACATGTGGCAGGAAGTAGGAAAAGCAATGTATGCCCCTCCCATCAGAGGACGACTTAATTGTTCATCAAATATTACAGGGCTGCTATTAACAAGAGATGGTGGT------------------AATACCACA---------------------AATAACACT---GAGGTCTTCAGACCTGGAGGAGGAGATATGAGAGACAATTGGAGA---AGTGAATTATATAAATATAAAGTAGTAAAAATTGAACCA---TTAGGAATAGCACCC---ACCAAGGCAAAGAGAAGAGTGGTGCAGAGAGAA---AAAAGAGCAGTG---GGA---ATAGGA---GCTTTG---TTCCTT---GGG---------TTCTTGGGA---GCAGCAGGAAGCACTATGGGCGCAGCGTCGATG---ACGCTGACGGTACAGGCCAGACTATTATTGTCTGGTATAGTGCAACAGCAGAACAATTTGCTGAGAGCTATTGAGGCGCAACAGCATCTGTTGCAACTCACAGTCTGGGGCATCAAGCAGCTCCAGGCAAGA---GTCCTGGCTGTGGAAAGATACCTAAGAGATCAACAGCTCCTGGGGATTTGGGGATGCTCTGGAAAACTCATTTGCACCACTACTGTGCCTTGGAATGTTAGTTGGAGT---------------------------AATAAATCCATGAATGACATTTGGAAT---AACATGACCTGGATGGAGTGGGAAAGAGAGATTGAC------AATTATACAAACATAATATACACCTTACTTGAAGAATCGCAGAACCAACAAGATAAGAATGAACAGGAATTATTGGAATTGGATAAATGGGCAAGTTTGTGGAATTGGTTTAGCATAACAAATTGGCTGTGGTACATAAAAATATTCATAATGATAGTAGGAGGCTTAATAGGTTTAAGAATAGTTTTTACTGTATTTTCTATAGTGAATAGAGTTAGGCAGGGATATTCACCATTATCGTTTCAGACCCGC---TTCCCAACCTCGAGGGGA------CTCGACAGGCCCGAAGGAATCGAAGAAGAAGGTGGAGACAGAGACAGAGACAGATCCAGGCCATTAGTGGATGGATTCTTAGCAATTATCTGGGTCGACCTGCGGAGCCTGTGCCTCTTCAGCTACCATCGCTTGAGAGACTTACTCTTGATTGTAGCGAGGATTGTGGAACTTCTGGGACGCAGG---------------GGGTGGGAAGCCCTCAAATATTGGTGG---AATCTCCTGCGGTATTGG---------------------------------------------------AGCCAGGAACTAAGGAATAGTGCTATTAGCTTGCTTAATGCCACAGCCATAGCAGTAGCTGAGGGAACAGATAGGGTGTTAGAAGTATTACAAAGA------------------GCTTTTAGAGCTGTTATACACATACCTAGAAGAATAAGACAGGGCTTAGAAAGGTTTTTGCTATAA

2.04013240.ADARC.GU330449 ATGAGAGTGAAGGGGATCATGAGGAATTATCAGCACTTA---------TGGAGATGGGGC------------------------ATGATGCTCCTTGGGATAATCATGATC------------TGTAGTGCTGCA---------GAACAATTGTGGGTCACAATCTATTATGGGGTACCTGTGTGGAAAGAAGCAACCACCACTCTATTTTGTGCATCAAATGCTAAAGCATATGATACAGAGGTACATAAT---GTCTGGGCCACACATGCCTGTGTACCCACAGACCCCAACCCACAAGAAGTAAGATTG---GAAAATGTGACAGAAAATTTTAACATGTGGAAAAATAATATGGTAGAACAGATGCATGAGGATATAATTAGCCTATGGGATCAAAGCCTAAAACCATGTGTGAAATTAACCCCACTCTGTGTTACTTTAAACTGCACTGATGCTAATACCACTAATACTAATGCCAAT------------------------------------------------------------------------------AGTACTAATAACAGTAGCTTGGGAACAATGGAGAAAGGAGAAATAAAAAACTGCTCTTTCAACATCACC---ACAAACCTAAGAGAT------AAGGTGCAGAAAGAATATGCACTTTTTTATAACCTTGATGTAGTGCCAATAAAGGGTGAGGAT---------------------------AATACTAGC---------------------------TATAGGTTGATAAGTTGTAATACCTCAGTCATTACACAGGCCTGTCCAAAGGTATCCTTTGAGCCAATTCCCATACATTATTGTACTCCAGCTGGTTTTGCGATTCTACAATGT---AATGATAAGAAATTCAATGGATCAGGACCATGTACAAATGTCAGCACAGTACAATGTACACATGGAATTAGGCCAGTAGTATCAACTCAACTGCTGTTAAATGGCAGTCTAGCAGAAAAA---GAGGTAGTAATTAGGTCTGAGAATTTCACAAATAATGCTAAAACCATAATAGTACAGCTAAATGAATCTGTAGTAATTAATTGTACAAGACCCAACAACAATACAAGAAAAAGTATACCTATAGGA------------CCAGGAAGA---GCATTTTATGCAACAGGAGAAATAATAGGAGATATAAGACAAGCACATTGTAACGTT------AGTACAAAAGCCTGGAAGGAAGCTTTACAACAGGTAGCTATAAAACTATCA---GAACAATTTGGG------------AATAAAACA---ATAGTCTTTAATCAA---------TCCTCAGGAGGAGACCCAGAAGTTGTAATGCACAGTTTTAATTGTAGAGGGGAATTTTTCTACTGTAATACAACAGGACTGTTTAATAATACTTGGGGGTTT---------AATAGTACTTGG---------------------GATGCTATTAATGTGCCAGAA------------------AATGACACA------------------------------ATCACACTCCCATGCAGAATAAAACAAATTGTAAACATGTGGCAGGAAGTAGGAAAAGCAATGTATGCCCCTCCCATCAGAGGACGACTTAATTGTTCATCAAATATTACAGGGCTGCTATTAACAAGAGATGGTGGT------------------AATACCACA---------------------AATAACACT---GAGGTCTTCAGACCTGGAGGAGGAGATATGAGAGACAATTGGAGA---AGTGAATTATATAAATATAAAGTAGTAAAAATTGAACCA---TTAGGAATAGCACCC---ACCAAGGCAAAGAGAAGAGTGGTGCAGAGAGAA---AAAAGAGCAGTG---GGA---ATAGGA---GCTTTG---TTCCTT---GGG---------TTCTTGGGA---GCAGCAGGAAGCACTATGGGCGCAGCGTCGATG---ACGCTGACGGTACAGGCCAGACTATTATTGTCTGGTATAGTGCAACAGCAGAACAATTTGCTGAGAGCTATTGAGGCGCAACAGCATCTGTTGCAACTCACAGTCTGGGGCATCAAGCAGCTCCAGGCAAGA---GTCCTGGCTGTGGAAAGATACCTAAGAGATCAACAGCTCCTGGGGATTTGGGGATGCTCTGGAAAACTCATTTGCACCACTACTGTGCCTTGGAATGTTAGTTGGAGT---------------------------AATAAATCCATGAATGACATTTGGAAT---AACATGACCTGGATGGAGTGGGAAAGAGAGATTGAC------AATTATACAAACATAATATACACCTTACTTGAAGAATCGCAGAACCAACAAGATAAGAATGAACAGGAATTATTGGAATTGGATAAATGGGCAAGTTTGTGGAATTGGTTTAGCATAACAAATTGGCTGTGGTACATAAAAATATTCATAATGATAGTAGGAGGCTTAATAGGTTTAAGAATAGTTTTTACTGTATTTTCTATAGTGAATAGAGTTAGGCAGGGATATTCACCATTATCGTTTCAGACCCGC---TTCCCAACCTCGAGGGGA------CTCGACAGGCCCGAAGGAATCGAAGAAGAAGGTGGAGACAGAGACAGAGACAGATCCAGGCCATTAGTGGATGGATTCTTAGCAATTATCTGGGTCGACCTGCGGAGCCTGTGCCTCTTCAGCTACCATCGCTTGAGAGACTTACTCTTGATTGTAGCGAGGATTGTGGAACTTCTGGGACGCAGG---------------GGGTGGGAAGCCCTCAAATATTGGTGG---AATCTCCTGCGGTATTGG---------------------------------------------------AGCCAGGAACTAAGGAATAGTGCTATTAGCTTGCTTAATGCCACAGCCATAGCAGTAGCTGAGGGAACAGATAGGGTGTTAGAAGTATTACAAAGA------------------GCTTTTAGAGCTGTTATACACATACCTAGAAGAATAAGACAGGGCTTAGAAAGGTTTTTGCTATAA

2.04013240.ADARC.GU330450 ATGAGAGTGAAGGGGATCATGAGGAATTATCAGCACTTA---------TGGAGATGGGGC------------------------ATGATGCTCCTTGGGATAATCATGATC------------TGTAGTGCTGCA---------GAACAATTGTGGGTCACAATCTATTATGGGGTACCTGTGTGGAAAGAAGCAACCACCACTCTATTTTGTGCATCAAATGCTAAAGCATATGATACAGAGGTACATAAT---GTCTGGGCCACACATGCCTGTGTACCCACAGACCCCAACCCACAAGAAGTAAGATTG---GAAAATGTGACAGAAAATTTTAACATGTGGAAAAATAATATGGTAGAACAGATGCATGAGGATATAATTAGCCTATGGGATCAAAGCCTAAAACCATGTGTGAAATTAACCCCACTCTGTGTTACTTTAAACTGCACTGATGCTAATACCACTAATACTAATGCCAAT------------------------------------------------------------------------------AGTACTAATAACAGTAGCTTGGGAACAATGGAGAAAGGAGAAATAAAAAACTGCTCTTTCAACATCACC---ACAAACCTAAGAGAT------AAGGTGCAGAAAGAATATGCACTTTTTTATAACCTTGATGTAGTGCCAATAAAGGGTGAGGAT---------------------------AATACTAGC---------------------------TATAGGTTGATAAGTTGTAATACCTCAGTCATTACACAGGCCTGTCCAAAGGTATCCTTTGAGCCAATTCCCATACATTATTGTACTCCAGCTGGTTTTGCGATTCTACAATGT---AATGATAAGAAATTCAATGGATCAGGACCATGTACAAATGTCAGCACAGTACAATGTACACATGGAATTAGGCCAGTAGTATCAACTCAACTGCTGTTAAATGGCAGTCTAGCAGAAAAA---GAGGTAGTAATTAGGTCTGAGAATTTCACAAATAATGCTAAAACCATAATAGTACAGCTAAATGAATCTGTAGTAATTAATTGTACAAGACCCAACAACAATACAAGAAAAAGTATACCTATAGGA------------CCAGGAAGA---GCATTTTATGCAACAGGAGAAATAATAGGAGATATAAGACAAGCACATTGTAACGTT------AGTACAAAAGCCTGGAAGGAAGCTTTACAACAGGTAGCTATAAAACTATCA---GAACAATTTGGG------------AATAAAACA---ATAGTCTTTAATCAA---------TCCTCAGGAGGAGACCCAGAAGTTGTAATGCACAGTTTTAATTGTAGAGGGGAATTTTTCTACTGTAATACAACAGGACTGTTTAATAATACTTGGGGGTTT---------AATAGTACTTGG---------------------GATGCTATTAATGTGCCAGAA------------------AATGACACA------------------------------ATCACACTCCCATGCAGAATAAAACAAATTGTAAACATGTGGCAGGAAGTAGGAAAAGCAATGTATGCCCCTCCCATCAGAGGACGACTTAATTGTTCATCAAATATTACAGGGCTGCTATTAACAAGAGATGGTGGT------------------AATACCACA---------------------AATAACACT---GAGGTCTTCAGACCTGGAGGAGGAGATATGAGAGACAATTGGAGA---AGTGAATTATATAAATATAAAGTAGTAAAAATTGAACCA---TTAGGAATAGCACCC---ACCAAGGCAAAGAGAAGAGTGGTGCAGAGAGAA---AAAAGAGCAGTG---GGA---ATAGGA---GCTTTG---TTCCTT---GGG---------TTCTTGGGA---GCAGCAGGAAGCACTATGGGCGCAGCGTCGATG---ACGCTGACGGTACAGGCCAGACTATTATTGTCTGGTATAGTGCAACAGCAGAACAATTTGCTGAGAGCTATTGAGGCGCAACAGCATCTGTTGCAACTCACAGTCTGGGGCATCAAGCAGCTCCAGGCAAGA---GTCCTGGCTGTGGAAAGATACCTAAGAGATCAACAGCTCCTGGGGATTTGGGGATGCTCTGGAAAACTCATTTGCACCACTACTGTGCCTTGGAATGTTAGTTGGAGT---------------------------AATAAATCCATGAATGACATTTGGAAT---AACATGACCTGGATGGAGTGGGAAAGAGAGATTGAC------AATTATACAAACATAATATACACCTTACTTGAAGAATCGCAGAACCAACAAGATAAGAATGAACAGGAATTATTGGAATTGGATAAATGGGCAAGTTTGTGGAATTGGTTTAGCATAACAAATTGGCTGTGGTACATAAAAATATTCATAATGATAGTAGGAGGCTTAATAGGTTTAAGAATAGTTTTTACTGTATTTTCTATAGTGAATAGAGTTAGGCAGGGATATTCACCATTATCGTTTCAGACCCGC---TTCCCAACCTCGAGGGGA------CTCGACAGGCCCGAAGGAATCGAAGAAGAAGGTGGAGACAGAGACAGAGACAGATCCAGGCCATTAGTGGATGGATTCTTAGCAATTATCTGGGTCGACCTGCGGAGCCTGTGCCTCTTCAGCTACCATCGCTTGAGAGACTTACTCTTGATTGTAGCGAGGATTGTGGAACTTCTGGGACGCAGG---------------GGGTGGGAAGCCCTCAAATATTGGTGG---AATCTCCTGCGGTATTGG---------------------------------------------------AGCCAGGAACTAAGGAATAGTGCTATTAGCTTGCTTAATGCCACAGCCATAGCAGTAGCTGAGGGAACAGATAGGGTGTTAGAAGTATTACAAAGA------------------GCTTTTAGAGCTGTTATACACATACCTAGAAGAATAAGACAGGGCTTAGAAAGGTTTTTGCTATAA

2.04013240.ADARC.GU330451 ATGAGAGTGAAGGGGATCATGAGGAATTATCAGCACTTA---------TGGAGATGGGGC------------------------ATGATGCTCCTTGGGATAATCATGATC------------TGTAGTGCTGCA---------GAACAATTGTGGGTCACAATCTATTATGGGGTACCTGTGTGGAAAGAAGCAACCACCACTCTATTTTGTGCATCAAATGCTAAAGCATATGATACAGAGGTACATAAT---GTCTGGGCCACACATGCCTGTGTACCCACAGACCCCAACCCACAAGAAGTAAGATTG---GAAAATGTGACAGAAAATTTTAACATGTGGAAAAATAATATGGTAGAACAGATGCATGAGGATATAATTAGCCTATGGGATCAAAGCCTAAAACCATGTGTGAAATTAACCCCACTCTGTGTTACTTTAAACTGCACTGATGCTAATACCACTAATACTAATGCCAAT------------------------------------------------------------------------------AGTACTAATAACAGTAGCTTGGGAACAATGGAGAAAGGAGAAATAAAAAACTGCTCTTTCAACATCACC---ACAAACCTAAGAGAT------AAGGTGCAGAAAGAATATGCACTTTTTTATAACCTTGATGTAGTGCCAATAAAGGGTGAGGAT---------------------------AATACTAGC---------------------------TATAGGTTGATAAGTTGTAATACCTCAGTCATTACACAGGCCTGTCCAAAGGTATCCTTTGAGCCAATTCCCATACATTATTGTACTCCAGCTGGTTTTGCGATTCTACAATGT---AATGATAAGAAATTCAATGGATCAGGACCATGTACAAATGTCAGCACAGTACAATGTACACATGGAATTAGGCCAGTAGTATCAACTCAACTGCTGTTAAATGGCAGTCTAGCAGAAAAA---GAGGTAGTAATTAGGTCTGAGAATTTCACAAATAATGCTAAAACCATAATAGTACAGCTAAATGAATCTGTAGTAATTAATTGTACAAGACCCAACAACAATACAAGAAAAAGTATACCTATAGGA------------CCAGGAAGA---GCATTTTATGCAACAGGAGAAATAATAGGAGATATAAGACAAGCACATTGTAACGTT------AGTACAAAAGCCTGGAAGGAAGCTTTACAACAGGTAGCTATAAAACTATCA---GAACAATTTGGG------------AATAAAACA---ATAGTCTTTAATCAA---------TCCTCAGGAGGAGACCCAGAAGTTGTAATGCACAGTTTTAATTGTAGAGGGGAATTTTTCTACTGTAATACAACAGGACTGTTTAATAATACTTGGGGGTTT---------AATAGTACTTGG---------------------GATGCTATTAATGTGCCAGAA------------------AATGACACA------------------------------ATCACACTCCCATGCAGAATAAAACAAATTGTAAACATGTGGCAGGAAGTAGGAAAAGCAATGTATGCCCCTCCCATCAGAGGACGACTTAATTGTTCATCAAATATTACAGGGCTGCTATTAACAAGAGATGGTGGT------------------AATACCACA---------------------AATAACACT---GAGGTCTTCAGACCTGGAGGAGGAGATATGAGAGACAATTGGAGA---AGTGAATTATATAAATATAAAGTAGTAAAAATTGAACCA---TTAGGAATAGCACCC---ACCAAGGCAAAGAGAAGAGTGGTGCAGAGAGAA---AAAAGAGCAGTG---GGA---ATAGGA---GCTTTG---TTCCTT---GGG---------TTCTTGGGA---GCAGCAGGAAGCACTATGGGCGCAGCGTCGATG---ACGCTGACGGTACAGGCCAGACTATTATTGTCTGGTATAGTGCAACAGCAGAACAATTTGCTGAGAGCTATTGAGGCGCAACAGCATCTGTTGCAACTCACAGTCTGGGGCATCAAGCAGCTCCAGGCAAGA---GTCCTGGCTGTGGAAAGATACCTAAGAGATCAACAGCTCCTGGGGATTTGGGGATGCTCTGGAAAACTCATTTGCACCACTACTGTGCCTTGGAATGTTAGTTGGAGT---------------------------AATAAATCCATGAATGACATTTGGAAT---AACATGACCTGGATGGAGTGGGAAAGAGAGATTGAC------AATTATACAAACATAATATACACCTTACTTGAAGAATCGCAGAACCAACAAGATAAGAATGAACAGGAATTATTGGAATTGGATAAATGGGCAAGTTTGTGGAATTGGTTTAGCATAACAAATTGGCTGTGGTACATAAAAACATTCATAATGATAGTAGGAGGCTTAATAGGTTTAAGAATAGTTTTTACTGTATTTTCTATAGTGAATAGAGTTAGGCAGGGATATTCACCATTATCGTTTCAGACCCGC---TTCCCAACCTCGAGGGGA------CTCGACAGGCCCGAAGGAATCGAAGAAGAAGGTGGAGACAGAGACAGAGACAGATCCAGGCCATTAGTGGATGGATTCTTAGCAATTATCTGGGTCGACCTGCGGAGCCTGTGCCTCTTCAGCTACCATCGCTTGAGAGACTTACTCTTGATTGTAGCGAGGATTGTGGAACTTCTGGGACGCAGG---------------GGGTGGGAAGCCCTCAAATATTGGTGG---AATCTCCTGCGGTATTGG---------------------------------------------------AGCCAGGAACTAAGGAATAGTGCTATTAGCTTGCTTAATGCCACAGCCATAGCAGTAGCTGAGGGAACAGATAGGGTGTTAGAAGTATTACAAAGA------------------GCTTTTAGAGCTGTTATACACATACCTAGAAGAATAAGACAGGGCTTAGAAAGGTTTTTGCTATAA

2.04013240.ADARC.GU330452 ATGAGAGTGAAGGGGATCATGAGGAATTATCAGCACTTA---------TGGAGATGGGGC------------------------ATGATGCTCCTTGGGATAATCATGATC------------TGTAGTGCTGCA---------GAACAATTGTGGGTCACAATCTATTATGGGGTACCTGTGTGGAAAGAAGCAACCACCACTCTATTTTGTGCATCAAATGCTAAAGCATATGATACAGAGGTACATAAT---GTCTGGGCCACACATGCCTGTGTACCCACAGACCCCAACCCACAAGAAGTAAGATTG---GAAAATGTGACAGAAAATTTTAACATGTGGAAAAATAATATGGTAGAACAGATGCATGAGGATATAATTAGCCTATGGGATCAAAGCCTAAAACCATGTGTGAAATTAACCCCACTCTGTGTTACTTTAAACTGCACTGATGCTAATACCACTAATACTAATGCCAAT------------------------------------------------------------------------------AGTACTAATAACAGTAGCTTGGGAACAATGGAGAAAGGAGAAATAAAAAACTGCTCTTTCAACATCACC---ACAAACCTAAGAGAT------AAGGTGCAGAAAGAATATGCACTTTTTTATAACCTTGATGTAGTGCCAATAAAGGGTGAGGAT---------------------------AATACTAGC---------------------------TATAGGTTGATAAGTTGTAATACCTCAGTCATTACACAGGCCTGTCCAAAGGTATCCTTTGAGCCAATTCCCATACATTATTGTACTCCAGCTGGTTTTGCGATTCTACAATGT---AATGATAAGAAATTCAATGGATCAGGACCATGTACAAATGTCAGCACAGTACAATGTACACATGGAATTAGGCCAGTAGTATCAACTCAACTGCTGTTAAATGGCAGTCTAGCAGAAAAA---GAGGTAGTAATTAGGTCTGAGAATTTCACAAATAATGCTAAAACCATAATAGTACAGCTAAATGAATCTGTAGTAATTAATTGTACAAGACCCAACAACAATACAAGAAAAAGTATACCTATAGGA------------CCAGGAAGA---GCATTTTATGCAACAGGAGAAATAATAGGAGATATAAGACAAGCACATTGTAACGTT------AGTACAAAAGCCTGGAAGGAAGCTTTACAACAGGTAGCTATAAAACTATCA---GAACAATTTGGG------------AATAAAACA---ATAGTCTTTAATCAA---------TCCTCAGGAGGAGACCCAGAAGTTGTAATGCACAGTTTTAATTGTAGAGGGGAATTTTTCTACTGTAATACAACAGGACTGTTTAATAATACTTGGGGGTTT---------AATAGTACTTGG---------------------GATGCTATTAATGTGCCAGAA------------------AATGACACA------------------------------ATCACACTCCCATGCAGAATAAAACAAATTGTAAACATGTGGCAGGAAGTAGGAAAAGCAATGTATGCCCCTCCCATCAGAGGACAAATTAATTGTTCATCAAATATTACAGGGCTGCTATTAACAAGAGATGGTGGT------------------AATACCACA---------------------AATAACACT---GAGGTCTTCAGACCTGGAGGAGGAGATATGAGAGACAATTGGAGA---AGTGAATTATATAAATATAAAGTAGTAAAAATTGAACCA---TTAGGAATAGCACCC---ACCAAGGCAAAGAGAAGAGTGGTGCAGAGAGAA---AAAAGAGCAGTG---GGA---ATAGGA---GCTTTG---TTCCTT---GGG---------TTCTTGGGA---GCAGCAGGAAGCACTATGGGCGCAGCGTCGATG---ACGCTGACGGTACAGGCCAGACTATTATTGTCTGGTATAGTGCAACAGCAGAACAATTTGCTGAGAGCTATTGAGGCGCAACAGCATCTGTTGCAACTCACAGTCTGGGGCATCAAGCAGCTCCAGGCAAGA---GTCCTGGCTGTGGAAAGATACCTAAGGGATCAACAGCTCCTGGGGATTTGGGGATGCTCTGGAAAACTCATTTGCACCACTGCTGTGCCTTGGAATGTTAGTTGGAGT---------------------------AATAAATCCATGAATGACATTTGGAAT---AACATGACCTGGATGGAGTGGGAAAGAGAGATTGAC------AATTATACAAACATAATATACACCTTACTTGAAGAATCGCAGAACCAACAAGATAAGAATGAACAGGAATTATTGGAATTGGATAAATGGGCAAGTTTGTGGAATTGGTTTAGCATAACAAATTGGCTGTGGTACATAAAAATATTCATAATGATAGTAGGAGGCTTAATAGGTTTAAGAATAGTTTTTACTGTATTTTCTATAATGAATAGAGTTAGGCAGGGATATTCACCATTATCGTTTCAGACCCGC---CTCCCAACTTCGAGGGGA------CTCGACAGGCCCGAAGGAATCGAAGAAGAAGGTGGAGACAGAGACAGAGACAGATCCAGGCCATTAGTGGATGGATTCTTAGCAATTATCTGGGTCGACCTGCGGAGCCTGTGCCTCTTCAGCTACCATCGCTTGAGAGACTTACTCTTGATTGTAGCGAGGATTGTGGAACTTCTGGGACGCAGG---------------GGGTGGGAAGCCCTCAAATATTGGTGG---AATCTCCTGCGGTATTGG---------------------------------------------------AGCCAGGAACTAAGGAATAGTGCTATTAGCTTGCTTAATGCCACAGCCATAGCAGTAGCTGAGGGAACAGATAGGGTGTTAGAAGTATTACAAAGA------------------GCTTTTAGAGCTGTTATACACATACCTAGAAGAATAAGACAGGGCTTAGAAAGGTTTTTGCTATAA

2.04013240.ADARC.GU330453 ATGAGAGTGAAGGGGATCATGAGGAATTATCAGCACTTA---------TGGAGATGGGGC------------------------ATGATGCTCCTTGGGATAATCATGATC------------TGTAGTGCTGCA---------GAACAATTGTGGGTCACAATCTATTATGGGGTACCTGTGTGGAAAGAAGCAACCACCACTCTATTTTGTGCATCAAATGCTAAAGCATATGATACAGAGGTACATAAT---GTCTGGGCCACACATGCCTGTGTACCCACAGACCCCAACCCACAAGAAGTAAGATTG---GAAAATGTGACAGAAAATTTTAACATGTGGAAAAATAATATGGTAGAACAGATGCATGAGGATATAATTAGCCTATGGGATCAAAGCCTAAAACCATGTGTGAAATTAACCCCACTCTGTGTTACTTTAAACTGCACTGATGCTAATACCACTAATACTAATGCCAAT------------------------------------------------------------------------------AGTACTAATAACAGTAGCTTGGGAACAATGGAGAAAGGAGAAATAAAAAACTGCTCTTTCAACATCACC---ACAAACCTAAGAGAT------AAGGTGCAGAAAGAATATGCACTTTTTTATAACCTTGATGTAGTGCCAATAAAGGGTGAGGAT---------------------------AATACTAGC---------------------------TATAGGTTGATAAGTTGTAATACCTCAGTCATTACACAGGCCTGTCCAAAGGTATCCTTTGAGCCAATTCCCATACATTATTGTACTCCAGCTGGTTTTGCGATTCTACAATGT---AATGATAAGAAATTCAATGGATCAGGACCATGTACAAATGTCAGCACAGTACAATGTACACATGGAATTAGGCCAGTAGTATCAACTCAACTGCTGTTAAATGGCAGTCTAGCAGAAAAA---GAGGTAGTAATTAGGTCTGAGAATTTCACAAATAATGCTAAAACCATAATAGTACAGCTAAATGAATCTGTAGTAATTAATTGTACAAGACCCAACAACAATACAAGAAAAAGTATACCTATAGGA------------CCAGGAAGA---GCATTTTATGCAACAGGAGAAATAATAGGAGATATAAGACAAGCACATTGTAACGTT------AGTACAAAAGCCTGGAAGGAAGCTTTACAACAGGTAGCTATAAAACTATCA---GAACAATTTGGG------------AATAAAACA---ATAGTCTTTAATCAA---------TCCTCAGGAGGAGACCCAGAAGTTGTAATGCACAGTTTTAATTGTAGAGGGGAATTTTTCTACTGTAATACAACAGGACTGTTTAATAATACTTGGGGGTTT---------AATAGTACTTGG---------------------GATGCTATTAATGTGCCAGAA------------------AATGACACA------------------------------ATCACACTCCCATGCAGAATAAAACAAATTGTAAACATGTGGCAGGAAGTAGGAAAAGCAATGTATGCCCCTCCCATCAGAGGACGACTTAATTGTTCATCAAATATTACAGGGCTGCTATTAACAAGAGATGGTGGT------------------AATACCACA---------------------AATAACACT---GAGGTCTTCAGACCTGGAGGAGGAGATATGAGAGACAATTGGAGA---AGTGAATTATATAAATATAAAGTAGTAAAAATTGAACCA---TTAGGAATAGCACCC---ACCAAGGCAAAGAGAAGAGTGGTGCAGAGAGAA---AAAAGAGCAGTG---GGA---ATAGGA---GCTTTG---TTCCTT---GGG---------TTCTTGGGA---GCAGCAGGAAGCACTATGGGCGCAGCGTCGATG---ACGCTGACGGTACAGGCCAGACTATTATTGTCTGGTATAGTGCAACAGCAGAACAATTTGCTGAGAGCTATTGAGGCGCAACAGCATCTGTTGCAACTCACAGTCTGGGGCATCAAGCAGCTCCAGGCAAGA---GTCCTGGCTGTGGAAAGATACCTAAGAGATCAACAGCTCCTGGGGATTTGGGGATGCTCTGGAAAACTCATTTGCACCACTACTGTGCCTTGGAATGTTAGTTGGAGT---------------------------AATAAATCCATGAATGACATTTGGAAT---AACATGACCTGGATGGAGTGGGAAAGAGAGATTGAC------AATTATACAAACATAATATACACCTTACTTGAAGAATCGCAGAACCAACAAGATAAGAATGAACAGGAATTATTGGAATTGGATAAATGGGCAAGTTTGTGGAATTGGTTTAGCATAACAAATTGGCTGTGGTACATAAAAATATTCATAATGATAGTAGGAGGCTTAATAGGTTTAAGAATAGTTTTTACTGTATTTTCTATAGTGAATAGAGTTAGGCAGGGATATTCACCATTATCGTTTCAGACCCGC---TTCCCAACCTCGAGGGGA------CTCGACAGGCCCGAAGGAATCGAAGAAGAAGGTGGAGACAGAGACAGAGACAGATCCAGGCCATTAGTGGATGGATTCTTAGCAATTATCTGGGTCGACCTGCGGAGCCTGTGCCTCTTCAGCTACCATCGCTTGAGAGACTTACTCTTGATTGTAGCGAGGATTGTGGAACTTCTGGGACGCAGG---------------GGGTGGGAAGCCATCAAATATTGGTGG---AATCTCCTGCGGTATTGG---------------------------------------------------AGCCAGGAACTAAGGAATAGTGCTATTAGCTTGCTTAATGCCACAGCCATAGCAGTAGCTGAGGGAACAGATAGGGTGTTAGAAGTATTACAAAGA------------------GCTTTTAGAGCTGTTATACACATACCTAGAAGAATAAGACAGGGCTTAGAAAGGTTTTTGCTATAA

2.04013240.ADARC.GU330454 ATGAGAGTGAAGGGGATCATGAGGAATTATCAGCACTTA---------TGGAGATGGGGC------------------------ATGATGCTCCTTGGGATAATCATGATC------------TGTAGTGCTGCA---------GAACAATTGTGGGTCACAATCTATTATGGGGTACCTGTGTGGAAAGAAGCAACCACCACTCTATTTTGTGCATCAAATGCTAAAGCATATGATACAGAGGTACATAAT---GTCTGGGCCACACATGCCTGTGTACCCACAGACCCCAACCCACAAGAAGTAAGATTG---GAAAATGTGACAGAAAATTTTAACATGTGGAAAAATAATATGGTAGAACAGATGCATGAGGATATAATTAGCTTATGGGATCAAAGCCTAAAACCATGTGTGAAATTAACCCCACTCTGTGTTACTTTAAACTGCACTGATGCTAATACCACTAATACTAATGCCAAT------------------------------------------------------------------------------AGTACTAATAACAGTAGCTTGGGAACAATGGAGAAAGGAGAAATAAAAAACTGCTCTTTCAACATCACC---ACAAACCTAAGAGAT------AAGGTGCAGAAAGAATATGCACTTTTTTATAACCTTGATGTAGTGCCAATAAAGGGTGAGGAT---------------------------AATACTAGC---------------------------TATAGGTTGATAAGTTGTAATACCTCAGTCATTACACAGGCCTGTCCAAAGGTATCCTTTGAGCCAATTCCCATACATTATTGTACTCCAGCTGGTTTTGCGATTCTACAATGT---AATGATAAGAAATTCAATGGATCAGGACCATGTACAAATGTCAGCACAGTACAATGTACACATGGAATTAGGCCAGTAGTATCAACTCAACTGCTGTTAAATGGCAGTCTAGCAGAAAAA---GAGGTAGTAATTAGGTCTGAGAATTTCACAAATAATGCTAAAACCATAATAGTACAGCTAAATGAATCTGTAGTAATTAATTGTACAAGACCCAACAACAATACAAGAAAAAGTATACCTATAGGA------------CCAGGAAGA---GCATTTTATGCAACAGGAGAAATAATAGGAGATATAAGACAAGCACATTGTAACGTT------AGTACAAAAGCCTGGAAGGAAGCTTTACAACAGGTAGCTATAAAACTATCA---GAACAATTTGGG------------AATAAAACA---ATAGTCTTTAATCAA---------TCCTCAGGAGGAGACCCAGAAGTTGTAATGCACAGTTTTAATTGTAGAGGGGAATTTTTCTACTGTAATACAACAGGACTGTTTAATAATACTTGGGGGTTT---------AATAGTACTTGG---------------------GATGCTATTAATGTGCCAGAA------------------AATGACACA------------------------------ATCACACTCCCATGCAGAATAAAACAAATTGTAAACATGTGGCAGGAAGTAGGAAAAGCAATGTATGCCCCTCCCATCAGAGGACGACTTAATTGTTCATCAAATATTACAGGGCTGCTATTAACAAGAGATGGTGGT------------------AATACCACA---------------------AATAACACT---GAGGTCTTCAGACCTGGAGGAGGAGATATGAGAGACAATTGGAGA---AGTGAATTATATAAATATAAAGTAGTAAAAATTGAACCA---TTAGGAATAGCACCC---ACCAAGGCAAAGAGAAGAGTGGTGCAGAGAGAA---AAAAGAGCAGTG---GGA---ATAGGA---GCTTTG---TTCCTT---GGG---------TTCTTGGGA---GCAGCAGGAAGCACTATGGGCGCAGCGTCGATG---ACGCTGACGGTACAGGCCAGACTATTATTGTCTGGTATAGTGCAACAGCAGAACAATTTGCTGAGAGCTATTGAGGCGCAACAGCATCTGTTGCAACTCACAGTCTGGGGCATCAAGCAGCTCCAGGCAAGA---GTCCTGGCTGTGGAAAGATACCTAAGAGATCAACAGCTCCTGGGGATTTGGGGATGCTCTGGAAAACTCATTTGCACCACTACTGTGCCTTGGAATGTTAGTTGGAGT---------------------------AATAAATCCATGAATGACATTTGGAAT---AACATGACCTGGATGGAGTGGGAAAGAGAGATTGAC------AATTATACAAACATAATATACACCTTACTTGAAGAATCGCAGAACCAACAAGATAAGAATGAACAGGAATTATTGGAATTGGATAAATGGGCAAGTTTGTGGAATTGGTTTAGCATAACAAATTGGCTGTGGTACATAAAAATATTCATAATGATAGTAGGAGGCTTAATAGGTTTAAGAATAGTTTTTACTGTATTTTCTATAGTGAATAGAGTTAGGCAGGGATATTCACCATTATCGTTTCAGACCCGC---TTCCCAACCTCGAGGGGA------CTCGACAGGCCCGAAGGAATCGAAGAAGAAGGTGGAGACAGAGACAGAGACAGATCCAGGCCATTAGTGGATGGATTCTTAGCAATTATCTGGGTCGACCTGCGGAGCCTGTGCCTCTTCAGCTACCATCGCTTGAGAGACTTACTCTTGATTGTAGCGAGGATTGTGGAACTTCTGGGACGCAGG---------------GGGTGGGAAGCCCTCAAATATTGGTGG---AATCTCCTGCGGTATTGG---------------------------------------------------AGCCAGGAACTAAGGAATAGTGCTATTAGCTTGCTTAATGCCACAGCCATAGCAGTAGCTGAGGGAACAGATAGGGTGTTAGAAGTATTACAAAGA------------------GCTTTTAGAGCTGTTATACACATACCTAGAAGAATAAGACAGGGCTTAGAAAGGTTTTTGCTATAA

2.04013240.ADARC.GU330455 ATGAGAGTGAAGGGGATCATGAGGAATTATCAGCACTTA---------TGGAGATGGGGC------------------------ATGATGCTCCTTGGGATAATCATGATC------------TGTAGTGCTGCA---------GAACAATTGTGGGTCACAATCTATTATGGGGTACCTGTGTGGAAAGAAGCAACCACCACTCTATTTTGTGCATCAAATGCTAAAGCATATGATACAGAGGTACATAAT---GTCTGGGCCACACATGCCTGTGTACCCACAGACCCCAACCCACAAGAAGTAAGATTG---GAAAATGTGACAGAAAATTTTAACATGTGGAAAAATAATATGGTAGAACAGATGCATGAGGATATAATTAGCCTATGGGATCAAAGCCTAAAACCATGTGTGAAATTAACCCCACTCTGTGTTACTTTAAACTGCACTGATGCTAATACCACTAATACTAATGCCAAT------------------------------------------------------------------------------AGTACTAATAACAGTAGCTTGGGAACAATGGAGAAAGGAGAAATAAAAAACTGCTCTTTCAACATCACC---ACAAACCTAAGAGAT------AAGGTGCAGAAAGAATATGCACTTTTTTATAACCTTGATGTAGTGCCAATAAAGGGTGAGGAT---------------------------AATACTAGC---------------------------TATAGGTTGATAAGTTGTAATACCTCAGTCATTACACAGGCCTGTCCAAAGGTATCCTTTGAGCCAATTCCCATACATTATTGTACTCCAGCTGGTTTTGCGATTCTACAATGT---AATGATAAGAAATTCAATGGATCAGGACCATGTACAAATGTCAGCACAGTACAATGTACACATGGAATTAGGCCAGTAGTATCAACTCAACTGCTGTTAAATGGCAGTCTAGCAGAAAAA---GAGGTAGTAATTAGGTCTGAGAATTTCACAAATAATGCTAAAACCATAATAGTACAGCTAAATGAATCTGTAGTAATTAATTGTACAAGACCCAACAACAATACAAGAAAAAGTATACCTATAGGA------------CCAGGAAGA---GCATTTTATGCAACAGGAGAAATAATAGGAGATATAAGACAAGCACATTGTAACGTT------AGTACAAAAGCCTGGAAGGAAGCTTTACAACAGGTAGCTATAAAACTATCA---GAACAATTTGGG------------AATAAAACA---ATAGTCTTTAATCAA---------TCCTCAGGAGGAGACCCAGAAGTTGTAATGCACAGTTTTAATTGTAGAGGGGAATTTTTCTACTGTAATACAACAGGACTGTTTAATAATACTTGGGGGTTT---------AATAGTACTTGG---------------------GATGCTATTAATGTGCCAGAA------------------AATGACACA------------------------------ATCACACTCCCATGCAGAATAAAACAAATTGTAAACATGTGGCAGGAAGTAGGAAAAGCAATGTATGCCCCTCCCATCAGAGGACGACTTAATTGTTCATCAAATATTACAGGGCTGCTATTAACAAGAGATGGTGGT------------------AATACCACA---------------------AATAACACT---GAGGTCTTCAGACCTGGAGGAGGAGATATGAGAGACAATTGGAGA---AGTGAATTATATAAATATAAAGTAGTAAAAATTGAACCA---TTAGGAATAGCACCC---ACCAAGGCAAAGAGAAGAGTGGTGCAGAGAGAA---AAAAGAGCAGTG---GGA---ATAGGA---GCTTTG---TTCCTT---GGG---------TTCTTGGGA---GCAGCAGGAAGCACTATGGGCGCAGCGTCGATG---ACGCTGACGGTACAGGCCAGACTATTATTGTCTGGTATAGTGCAACAGCAGAACAATTTGCTGAGAGCTATTGAGGCGCAACAGCATCTGTTGCAACTCACAGTCTGGGGCATCAAGCAGCTCCAGGCAAGA---GTCCTGGCTGTGGAAAGATACCTAAGAGATCAACAGCTCCTGGGGATTTGGGGATGCTCTGGAAAACTCATTTGCACCACTACTGTGCCTTGGAATGTTAGTTGGAGT---------------------------AATAAATCCATGAATGACATTTGGAAT---AACATGACCTGGATGGAGTGGGAAAGAGAGATTGAC------AATTATACAAACATAATATACACCTTACTTGAAGAATCGCAGAACCAACAAGATAAGAATGAACAGGAATTATTGGAATTGGATAAATGGGCAAGTTTGTGGAATTGGTTTAGCATAACAAATTGGCTGTGGTACATAAAAATATTCATAATGATAGTAGGAGGCTTAATAGGTTTAAGAATAGTTTTTACTGTATTTTCTATAGTGAATAGAGTTAGGCAGGGATATTCACCATTATCGTTTCAGACCCGC---TTCCCAACCTCGAGGGGA------CTCGACAGGCCCGAAGGAATCGAAGAAGAAGGTGGAGACAGAGACAGAGACAGATCCAGGCCATTAGTGGATGGATTCTTAGCAATTATCTGGGTCGACCTGCGGAGCCTGTGCCTCTTCAGCTACCATCGCTTGAGAGACTTACTCTTGATTGTAGCGAGGATTGTGGAACTTCTGGGACGCAGG---------------GGGTGGGAAGCCCTCAAATATTGGTGG---AATCTCCTGCGGTATTGG---------------------------------------------------AGCCAGGAACTAAGGAATAGTGCTATTAGCTTGCTTAATGCCACAGCCATAGCAGTAGCTGAGGGAACAGATAGGGTGGTAGAAGTATTACAAAGA------------------GCTTTTAGAGCTGTTATACACATACCTAGAAGAATAAGACAGGGCTTAGAAAGGTTTTTGCTATAA

2.04013240.ADARC.GU330456 ATGAGAGTGAAGGGGATCATGAGGAATTATCAGCACTTA---------TGGAGATGGGGC------------------------ATGATGCTCCTTGGGATAATCATGATC------------TGTAGTGCTGCA---------GAACAATTGTGGGTCACAATCTATTATGGGGTACCTGTGTGGAAAGAAGCAACCACCACTCTATTTTGTGCATCAAATGCTAAAGCATATGATACAGAGGTACATAAT---GGCTGGGCCACACATGCCTGTGTACCCACAGACCCCAACCCACAAGAAGTAAGATTG---GAAAATGTGACAGAAAATTTTAACATGTGGAAAAATAATATGGTAGAACAGATGCATGAGGATATAATTAGCCTATGGGATCAAAGCCTAAAACCATGTGTGAAATTAACCCCACTCTGTGTTACTTTAAACTGCACTGATGCTAATGCCACTAATACTAATGCCAAT------------------------------------------------------------------------------AGTACTAATAACAGTAGCTTGGGAACAATGGAGAAAGGAGAAATAAAAAACTGCTCTTTCAACATCACC---ACAAACCTAAGAGAT------AAGGTGCAGAAAGAATATGCACTTTTTTATAACCTTGATGTAGTGCCAATAAAGGGTGAGGAT---------------------------AATACTAGC---------------------------TATAGGTTGATAAGTTGTAATACCTCAGTCATTACACAGGCCTGTCCAAAGGTATCCTTTGAGCCAATTCCCATACATTATTGTACTCCAGCTGGTTTTGCGATTATACAATGT---AATGATAAGAAATTCAATGGATCAGGACCATGTACAAATGTCAGCACAGTACAATGTACACATGGAATTAGGCCAGTAGTATCAACTCAACTGCTGTTAAATGGCAGTCTAGCAGAAAAA---GAGGTAGTAATTAGGTCTGAGAATTTCACAAATAATGCTAAAACCATAATAGTACAGCTAAATGAATCTGTAGTAATTAATTGTACAAGACCCAACAACAATACAAGAAAAAGTATACCTATAGGA------------CCAGGAAGA---GCATTTTATGCAACAGGAGAAATAATAGGAGATATAAGACAAGCACATTGTAACGTT------AGTACAAAAGCCTGGAAGGAAGCTTTACAACAGGTAGCTATAAAACTATCA---GAACAATTTGGG------------AATAAAACA---ATAGTCTTTAATCAA---------TCCTCAGGAGGAGACCCAGAAGTTGTAATGCACAGTTTTAATTGTAGAGGGGAATTTTTCTACTGTAATACAACAGGACTGTTTAATAATACTTGGGGGTTT---------AATAGTACTTGG---------------------GATGCTATTAATGTGCCAGAA------------------AATGACACA------------------------------ATCACACTCCCATGCAGAATAAAACAAATTGTAAACATGTGGCAGGAAGTAGGAAAAGCAATGTATGCCCCTCCCATCAGAGGACGACTTAATTGTTCATCAAATATTACAGGGCTGCTATTAACAAGAGATGGTGGT------------------AATACCACA---------------------AATAACACT---GAGGTCTTCAGACCTGGAGGAGGAGATATGAGAGACAATTGGAGA---AGTGAATTATATAAATATAAAGTAGTAAAAATTGAACCA---TTAGGAATAGCACCC---ACCAAGGCAAAGAGAAGAGTGGTGCAGAGTGAA---AAAAGAGCAGTG---GGA---ATAGGA---GCTTTG---TTCCTT---GGG---------TTCTTGGGA---GCAGCAGGAAGCACTATGGGCGCAGCGTCGATG---ACGCTGACGGTACAGGCCAGACTATTATTGTCTGGTATAGTGCAACAGCAGAACAATTTGCTGAGAGCTATTGAGGCGCAACAGCATCTGTTGCAACTCACAGTCTGGGGCATCAAGCAGCTCCAGGCAAGA---GTCCTGGCTGTGGAAAGATACCTAAGAGATCAACAGCTCCTGGGGATTTGGGGATGCTCTGGAAAACTCATTTGCACCACTACTGTGCCTTGGAATGTTAGTTGGAGT---------------------------AATAAATCCATGAATGACATTTGGAAT---AACATGACCTGGATGGAGTGGGAAAGAGAGATTGAC------AATTATACAAACATAATATACACCTTACTTGAAGAATCGCAGAACCAACAAGATAAGAATGAACAGGAATTACTGGAATTGGATAAATGGGCAAGTTTGTGGAATTGGTTTAGCATAACAAATTGGCTGTGGTACATAAAAATATTCATAATGATAGTAGGAGGCTTAATAGGTTTAAGAATAGTTTTTACTGTATTTTCTATAGTGAATAGAGTTAGGCAGGGATATTCACCATTATCGTTTCAGACCCGC---TTCCCAACCTCGAGGGGA------CTCGACAGGCCCGAAGGAATCGAAGAAGAAGGTGGAGACAGAGACAGAGACAGATCCAGGCCATTAGTGGATGGATTCTTAGCAATTATCTGGGTCGACCTGCGGAGCCTGTGCCTCTTCAGCTACCATCGCTTGAGAGACTTACTCTTGATTGTAGCGAGGATTGTGGAACTTCTGGGACGCAGG---------------GGGTGGGAAGCCCTCAAATATTGGTGG---AATCTCCTGCGGTATTGG---------------------------------------------------AGCCAGGAACTAAGGAATAGTGCTATTAGCTTGCTTAATGCCACAGCCATAGCAGTAGCTGAGGGAACAGATAGGGTGTTAGAAGTATTACAAAGA------------------GCTTTTAGAGCTGTTATACACATACCTAGAAGAATAAGACAGGGCTTAGAAAGGTTTTTGCTATAA

2.04013240.ADARC.GU330457 ATGAGAGTGAAGGGGATCATGAGGAATTATCAGCACTTA---------TGGAGATGGGGC------------------------ATGATGCTCCTTGGGATAATCATGATC------------TGTAGTGCTGCA---------GAACAATTGTGGGTCACAATCTATTATGGGGTACCTGTGTGGAAAGAAGCAACCACCACTCTATTTTGTGCATCAAATGCTAAAGCATATGATACAGAGGTACATAAT---GTCTGGGCCACACATGCCTGTGTACCCACAGACCCCAACCCACAAGAAGTAAGATTG---GAAAATGTGACAGAAAATTTTAACATGTGGAAAAATAATATGGTAGAACAGATGCATGAGGATATAATTAGCCTATGGGATCAAAGCCTAAAACCATGTGTGAAATTAACCCCACTCTGTGTTACTTTAAACTGCACTGATGCTAATACCACTAATACTAATGCCAAT------------------------------------------------------------------------------AGTACTAATAACAGTAGCTTGGGAACAATGGAGAAAGGAGAAATAAAAAACTGCTCTTTCAACATCACC---ACAAACCTAAGAGAT------AAGGTGCAGAAAGAATATGCACTTTTTTATAACCTTGATGTAGTGCCAATAAAGGGTGAGGAT---------------------------AATACTAGC---------------------------TATAGGTTGATAAGTTGTAATACCTCAGTCATTACACAGGCCTGTCCAAAGGTATCCTTTGAGCCAATTCCCATACATTATTGTACTCCAGCTGGTTTTGCGATTCTACAATGT---AATGATAAGAAATTCAATGGATCAGGACCATGTACAAATGTCAGCACAGTACAATGTACACATGGAATTAGGCCAGTAGTATCAACTCAACTGCTGTTAAATGGCAGTCTAGCAGAAAAA---GAGGTAGTAATTAGGTCTGAGAATTTCACAAATAATGCTAAAACCATAATAGTACAGCTAAATGAATCTGTAGTAATTAATTGTACAAGACCCAACAACAATACAAGAAAAAGTATACCTATAGGA------------CCAGGAAGA---GCATTTTATGCAACAGGAGAAATAATAGGAGATATAAGACAAGCACATTGTAACGTT------AGTACAAAAGCCTGGAAGGAAGCTTTACAACAGGTAGCTATAAAACTATCA---GAACAATTTGGG------------AATAAAACA---ATAGTCTTTAATCAA---------TCCTCAGGAGGAGACCCAGAAGTTGTAATGCACAGTTTTAATTGTAGAGGGGAATTTTTCTACTGTAATACAACAGGACTGTTTAATAATACTTGGGGGTTT---------AATAGTACTTGG---------------------GATGCTATTAATGTGCCAGAA------------------AATGACACA------------------------------ATCACACTCCCATGCAGAATAAAACAAATTGTAAACATGTGGCAGGAAGTAGGAAAAGCAATGTATGCCCCTCCCATCAGAGGACGACTTAATTGTTCATCAAATATTACAGGGCTGCTATTAACAAGAGATGGTGGT------------------AATACCACA---------------------AATAACACT---GAGGTCTTCAGACCTGGAGGAGGAGATATGAGAGACAATTGGAGA---AGTGAATTATATAAATATAAAGTAGTAAAAATTGAACCA---TTAGGAATAGCACCC---ACCAAGGCAAAGAGAAGAGTGGTGCAGAGAGAA---AAAAGAGCAGTG---GGA---ATAGGA---GCTTTG---TTCCTT---GGG---------TTCTTGGGA---GCAGCAGGAAGCACTATGGGCGCAGCGTCGATG---ACGCTGACGGTACAGGCCAGACTATTATTGTCTGGTATAGTGCAACAGCAGAACAATTTGCTGAGAGCTATTGAGGCGCAACAGCATCTGTTGCAACTCACAGTCTGGGGCATCAAGCAGCTCCAGGCAAGA---GTCCTGGCTGTGGAAAGATACCTAAGAGATCAACAGCTCCTGGGGATTTGGGGATGCTCTGGAAAACTCATTTGCACCACTACTGTGCCTTGGAATGTTAGTTGGAGT---------------------------AATAAATCCATGAATGACATTTGGAAT---AACATGACCTGGATGGAGTGGGAAAGAGAGATTGAC------AATTATACAAACATAATATACACCTTACTTGAAGAATCGCAGAACCAACAAGATAAGAATGAACAGGAATTATTGGAATTGGATAAATGGGCAAGTTTGTGGAATTGGTTTAGCATAACAAATTGGCTGTGGTACATAAAAATATTCATAATGATAGTAGGAGGCTTAATAGGTTTAAGAATAGTTTTTACTGTATTTTCTATAGTGAATAGAGTTAGGCAGGGATATTCACCATTATCGTTTCAGACCCGC---TTCCCAACCTCGAGGGGA------CTCGACAGGCCCGAAGGAATCGAAGAAGAAGGTGGAGACAGAGACAGAGACAGATCCAGGCCATTAGTGGATGGATTCTTAGCAATTATCTGGGTCGACCTGCGGAGCCTGTGCCTCTTCAGCTACCATCGCTTGAGAGACTTACTCTTGATTGTAGCGAGGATTGTGGAACTTCTGGGACGCAGG---------------GGGTGGGAAGCCCTCAAATATTGGTGG---AATCTCCTGCGGTATTGG---------------------------------------------------AGCCAGGAACTAAGGAATAGTGCTATTAGCTTGCTTAATGCCACAGCCATAGCAGTAGCTGAGGGAACAGATAGGGTGTTAGAAGTATTACAAAGA------------------GCTTTTAGAGCTGTTATACACATACCTAGAAGAATAAGACAGGGCTTAGAAAGGTTTTTGCTATAA

2.04013240.ADARC.GU330458 ATGAGAGTGAAGGGGATCATGAGGAATTATCAGCACTTA---------TGGAGATGGGGC------------------------ATGATGCTCCTTGGGATAATCATGATC------------TGTAGTGCTGCA---------GAACAATTGTGGGTCACAATCTATTATGGGGTACCTGTGTGGAAAGAAGCAACCACCACTCTATTTTGTGCATCAAATGCTAAAGCATATGATACAGAGGTACATAAT---GTCTGGGCCACACATGCCTGTGTACCCACAGACCCCAACCCACAAGAAGTAAGATTG---GAAAATGTGACAGAAAATTTTAACATGTGGAAAAATAATATGGTAGAACAGATGCATGAGGATATAATTAGCCTATGGGATCAAAGCCTAAAACCATGTGTGAAATTAACCCCACTCTGTGTTACTTTAAACTGCACTGATGCTAATACCACTAATACTAATGCCAAT------------------------------------------------------------------------------AGTACTAATAACAGTAGCTTGGGAACAATGGAGAAAGGAGAAATAAAAAACTGCTCTTTCAACATCACC---ACAAACCTAAGAGAT------AAGGTGCAGAAAGAATATGCACTTTTTTATAACCTTGATGTAGTGCCAATAAAGGGTGAGGAT---------------------------AATACTAGC---------------------------TATAGGTTGATAAGTTGTAATACCTCAGTCATTACACAGGCCTGTCCAAAGGTATCCTTTGAGCCAATTCCCATACATTATTGTACTCCAGCTGGTTTTGCGATTCTACAATGT---AATGATAAGAAATTCAATGGATCAGGACCATGTACAAATGTCAGCACAGTACAATGTACACATGGAATTAGGCCAGTAGTATCAACTCAACTGCTGTTAAATGGCAGTCTAGCAGAAAAA---GAGGTAGTAATTAGGTCTGAGAATTTCACAAATAATGCTAAAACCATAATAGTACAGCTAAATGAATCTGTAGTAATTAATTGTACAAGACCCAACAACAATACAAGAAAAAGTATACCTATAGGA------------CCAGGAAGA---GCATTTTATGCAACAGGAGAAATAATAGGAGATATAAGACAAGCACATTGTAACGTT------AGTACAAAAGCCTGGAAGGAAGCTTTACAACAGGTAGCTATAAAACTATCA---GAACAATTTGGG------------AATAAAACA---ATAGTCTTTAATCAA---------TCCTCAGGAGGAGACCCAGAAGTTGTAATGCACAGTTTTAATTGTAGAGGGGAATTTTTCTACTGTAATACAACAGGACTGTTTAATAATACTTGGGGGTTT---------AATAGTACTTGG---------------------GATGCTATTAATGTGCCAGAA------------------AATGACACA------------------------------ATCACACTCCCATGCAGAATAAAACAAATTGTAAACATGTGGCAGGAAGTAGGAAAAGCAATGTATGCCCCTCCCATCAGAGGACGACTTAATTGTTCATCAAATATTACAGGGCTGCTATTAACAAGAGATGGTGGT------------------AATACCACA---------------------AATAACACT---GAGGTCTTCAGACCTGGAGGAGGAGATATGAGAGACAATTGGAGA---AGTGAATTATATAAATATAAAGTAGTAAAAATTGAACCA---TTAGGAATAGCACCC---ACCAAGGCAAAGAGAAGAGTGGTGCAGAGAGAA---AAAAGAGCAGTG---GGA---ATAGGA---GCTTTG---TTCCTT---GGG---------TTCTTGGGA---GCAGCAGGAAGCACTATGGGCGCAGCGTCGATG---ACGCTGACGGTACAGGCCAGACTATTATTGTCTGGTATAGTGCAACAGCAGAACAATTTGCTGAGAGCTATTGAGGCGCAACAGCATCTATTGCAACTCACAGTCTGGGGCATCAAGCAGCTCCAGGCAAGA---GTCCTGGCTGTGGAAAGATACCTAAGAGATCAACAGCTCCTGGGGATTTGGGGATGCTCTGGAAAACTCATTTGCACCACTACTGTGCCTTGGAATGTTAGTTGGAGT---------------------------AATAAATCCATGAATGACATTTGGAAT---AACATGACCTGGATGGAGTGGGAAAGAGAGATTGAC------AATTATACAAACATAATATACACCTTACTTGAAGAATCGCAGAACCAACAAGATAAGAATGAACAGGAATTATTGGAATTGGATAAATGGGCAAGTTTGTGGAATTGGTTTAGCATAACAAATTGGCTGTGGTACATAAAAATATTCATAATGATAGTAGGAGGCTTAATAGGTTTAAGAATAGTTTTTACTGTATTTTCTATAGTGAATAGAGTTAGGCAGGGATATTCACCATTATCGTTTCAGACCCGC---TTCCCAACCTCGAGGGGA------CTCGACAGGCCCGAAGGAATCGAAGAAGAAGGTGGAGACAGAGACAGAGACAGATCCAGGCCATTAGTGGATGGATTCTTAGCAATTATCTGGGTCGACCTGCGGAGCCTGTGCCTCTTCAGCTACCATCGCTTGAGAGACTTACTCTTGATTGTAGCGAGGATTGTGGAACTTCTGGGACGCAGG---------------GGGTGGGAAGCCCTCAAATATTGGTGG---AATCTCCTGCGGTATTGG---------------------------------------------------AGCCAGGAACTAAGGAATAGTGCTATTAGCTTGCTTAATGCCACAGCCATAGCAGTAGCTGAGGGAACAGATAGGGTGGTAGAAGTATTACAAAGA------------------GCTTTTAGAGCTGTTATACACATACCTAGAAGAATAAGACAGGGCTTAGAAAGGTTTTTGCTATAA

2.04013240.ADARC.GU330459 ATGAGAGTGAAGGGGATCATGAGGAATTATCAGCACTTA---------TGGAGATGGGGC------------------------ATGATGCTCCTTGGGATAATCATGATC------------TGTAGTGCTGCA---------GAACAATTGTGGGTCACAATCTATTATGGGGTACCTGTGTGGAAAGAAGCAACCACCACTCTATTTTGTGCATCAAATGCTAAAGCATATGATACAGAGGTACATAAT---GTCTGGGCCACACATGCCTGTGTACCCACAGACCCCAACCCACAAGAAGTAAGATTG---GAAAATGTGACAGAAAATTTTAACATGTGGAAAAATAATATGGTAGAACAGATGCATGAGGATATAATTAGCCTATGGGATCAAAGCCTAAAACCATGTGTGAAATTAACCCCACTCTGTGTTACTTTAAACTGCACTGATGCTAATACCACTAATACTAATGCCAAT------------------------------------------------------------------------------AGTACTAATAACAGTAGCTTGGGAACAATGGAGAAAGGAGAAATAAAAAACTGCTCTTTCAACATCACC---ACAAACCTAAGAGAT------AAGGTGCAGAAAGAATATGCACTTTTTTATAACCTTGATGTAGTGCCAATAAAGGGTGAGGAT---------------------------AATACTAGC---------------------------TATAGGTTGATAAGTTGTAATACCTCAGTCATTACACAGGCCTGTCCAAAGGTATCCTTTGAGCCAATTCCCATACATTATTGTACTCCAGCTGGTTTTGCGATTCTACAATGT---AATGATAAGAAATTCAATGGATCAGGACCATGTACAAATGTCAGCACAGTACAATGTACACATGGAATTAGGCCAGTAGTATCAACTCAACTGCTGTTAAATGGCAGTCTAGCAGAAAAA---GAGGTAGTAATTAGGTCTGAGAATTTCACAAATAATGCTAAAACCATAATAGTACAGCTAAATGAATCTGTAGTAATTAATTGTACAAGACCCAACAACAATACAAGAAAAAGTATACCTATAGGA------------CCAGGAAGA---GCATTTTATGCAACAGGAGAAATAATAGGAGATATAAGACAAGCACATTGTAACGTT------AGTACAAAAGCCTGGAAGGAAGCTTTACAACAGGTAGCTATAAAACTATCA---GAACAATTTGGG------------AATAAAACA---ATAGTCTTTAATCAA---------TCCTCAGGAGGAGACCCAGAAGTTGTAATGCACAGTTTTAATTGTAGAGGGGAATTTTTCTACTGTAATACAACAGGACTGTTTAATAATACTTGGGGGTTT---------AATAGTACTTGG---------------------GATGCTATTAATGTGCCAGAA------------------AATGACACA------------------------------ATCACACTCCCATGCAGAATAAAACAAATTGTAAACATGTGGCAGGAAGTAGGAAAAGCAATGTATGCCCCTCCCATCAGAGGACGACTTAATTGTTCATCAAATATTACAGGGCTGCTATTAACAAGAGATGGTGGT------------------AATACCACA---------------------AATAACACT---GAGGTCTTCAGACCTGGAGGAGGAGATATGAGAGACAATTGGAGA---AGTGAATTATATAAATATAAAGTAGTAAAAATTGAACCA---TTAGGAATAGCACCC---ACCAAGGCAAAGAGAAGAGTGGTGCAGAGAGAA---AAAAGAGCAGTG---GGA---ATAGGA---GCTTTG---TTCCTT---GGG---------TTCTTGGGA---GCAGCAGGAAGCACTATGGGCGCAGCGTCGATG---ACGCTGACGGTACAGGCCAGACTATTATTGTCTGGTATAGTGCAACAGCAGAACAATTTGCTGAGAGCTATTGAGGCGCAACAGCATCTGTTGCAACTCACAGTCTGGGGCATCAAGCAGCTCCAGGCAAGA---GTCCTGGCTGTGGAAAGATACCTAAGAGATCAACAGCTCCTGGGGATTTGGGGATGCTCTGGAAAACTCATTTGCACCACTACTGTGCCTTGGAATGTTAGTTGGAGT---------------------------AATAAATCCATGAATGACATTTGGAAT---AACATGACCTGGATGGAGTGGGAAAGAGAGATTGAC------AATTATACAAACATAATATACACCTTACTTGAAGAATCGCAGAACCAACAAGATAAGAATGAACAGGAATTATTGGAATTGGATAAATGGGCAAGTTTGTGGAATTGGTTTAGCATAACAAATTGGCTGTGGTACATAAAAATATTCATAATGATAGTAGGAGGCTTAATAGGTTTAAGAATAGTTTTTACTGTATTTTCTATAGTGAATAGAGTTAGGCAGGGATATTCACCATTATCGTTTCAGACCCGC---TTCCCAACCTCGAGGGGA------CTCGACAGGCCCGAAGGAATCGAAGAAGAAGGTGGAGACAGAGACAGAGACAGATCCAGGCCATTAGTGGATGGATTCTTAGCAATTATCTGGGTCGACCTGCGGAGCCTGTGCCTCTTCAGCTACCATCGCTTGAGAGACTTACTCTTGATTGTAGCGAGGATTGTGGAACTTCTGGGACGCAGG---------------GGGTGGGAAGCCCTCAAATATTGGTGG---AATCTCCTGCGGTATTGG---------------------------------------------------AGCCAGGAACTAAGGAATAGTGCTATTAGCTTGCTTAATGCCACAGCCATAGCAGTAGCTGAGGGAACAGATAGGGTGTTAGAAGTATTACAAAGA------------------GCTTTTAGAGCTGTTATACACATACCTAGAAGAATAAGACAGGGCTTAGAAAGGTTTTTGCTATAA

2.04013240.ADARC.GU330460 ATGAGAGTGAAGGGGATCATGAGGAATTATCAGCACTTA---------TGGAGATGGGGC------------------------ATGATGCTCCTTGGGATAATCATGATC------------TGTAGTGCTGCA---------GAACAATTGTGGGTCACAATCTATTATGGGGTACCTGTGTGGAAAGAAGCAACCACCACTCTATTTTGTGCATCAAATGCTAAAGCATATGATACAGAGGTACATAAT---GTCTGGGCCACACATGCCTGTGTACCCACAGACCCCAACCCACAAGAAGTAAGATTG---GAAAATGTGACAGAAAATTTTAACATGTGGAAAAATAATATGGTAGAACAGATGCATGAGGATATAATTAGCCTATGGGATCAAAGCCTAAAACCATGTGTGAAATTAACCCCACTCTGTGTTACTTTAAACTGCACTGATGCTAATACCACTAATACTAATGCCAAT------------------------------------------------------------------------------AGTACTAATAACAGTAGCTTGGGAACAATGGAGAAAGGAGAAATAAAAAACTGCTCTTTCAACATCACC---ACAAACCTAAGAGAT------AAGGTGCAGAAAGAATATGCACTTTTTTATAACCTTGATGTAGTGCCAATAAAGGGTGAGGAT---------------------------AATACTAGC---------------------------TATAGGTTGATAAGTTGTAATACCTCAGTCATTACACAGGCCTGTCCAAAGGTATCCTTTGAGCCAATTCCCATACATTATTGTACTCCAGCTGGTTTTGCGATTCTACAATGT---AATGATAAGAAATTCAATGGATCAGGACCATGTACAAATGTCAGCACAGTACAATGTACACATGGAATTAGGCCAGTAGTATCAACTCAACTGCTGTTAAATGGCAGTCTAGCAGAAAAA---GAGGTAGTAATTAGGTCTGAGAATTTCACAAATAATGCTAAAACCATAATAGTACAGCTAAATGAATCTGTAGTAATTAATTGTACAAGACCCAACAACAATACAAGAAAAAGTATACCTATAGGA------------CCAGGAAGA---GCATTTTATGCAACAGGAGAAATAATAGGAGATATAAGACAAGCACATTGTAACGTT------AGTACAAAAGCCTGGAAGGAAGCTTTACAACAGGTAGCTATAAAACTATCA---GAACAATTTGGG------------AATAAAACA---ATAGTCTTTAATCAA---------TCCTCAGGAGGAGACCCAGAAGTTGTAATGCACAGTTTTAATTGTAGAGGGGAATTTTTCTACTGTAATACAACAGGACTGTTTAATAATACTTGGGGGTTT---------AATAGTACTTGG---------------------GATGCTATTAATGTGCCAGAA------------------AATGACACA------------------------------ATCACACTCCCATGCAGAATAAAACAAATTGTAAACATGTGGCAGGAAGTAGGAAAAGCAATGTATGCCCCTCCCATCAGAGGACGACTTAATTGTTCATCAAATATTACAGGGCTGCTATTAACAAGAGATGGTGGT------------------AATACCACA---------------------AATAACACT---GAGGTCTTCAGACCTGGAGGAGGAGATATGAGAGACAATTGGAGA---AGTGAATTATATAAATATAAAGTAGTAAAAATTGAACCA---TTAGGAATAGCACCC---ACCAAGGCAAAGAGAAGAGTGGTGCAGAGAGAA---AAAAGAGCAGTG---GGA---ATAGGA---GCTTTG---TTCCTT---GGG---------TTCTTGGGA---GCAGCAGGAAGCACTATGGGCGCAGCGTCGATG---ACGCTGACGGTACAGGCCAGACTATTATTGTCTGGTATAGTGCAACAGCAGAACAATTTGCTGAGAGCTATTGAGGCGCAACAGCATCTGTTGCAACTCACAGTCTGGGGCATCAAGCAGCTCCAGGCAAGA---GTCCTGGCTGTGGAAAGATACCTAAGAGATCAACAGCTCCTGGGGATTTGGGGATGCTCTGGAAAACTCATTTGCACCACTACTGTGCCTTGGAATGTTAGTTGGAGT---------------------------AATAAATCCATGAATGACATTTGGAAT---AACATGACCTGGATGGAGTGGGAAAGAGAGATTGAC------AATTATACAAACATAATATACACCTTACTTGAAGAATCGCAGAACCAACAAGATAAGAATGAACAGGAATTATTGGAATTGGATAAATGGGCAAGTTTGTGGAATTGGTTTAGCATAACAAATTGGCTGTGGTACATAAAAATATTCATAATGATAGTAGGAGGCTTAATAGGTTTAAGAATAGTTTTTACTGTATTTTCTATAGTGAATAGAGTTAGGCAGGGATATTCACCATTATCGTTTCAGACCCGC---TTCCCAACCTCGAGGGGA------CTCGACAGGCCCGAAGGAATCGAAGAAGAAGGTGGAGACAGAGACAGAGACAGATCCAGGCCATTAGTGGATGGATTCTTAGCAATTATCTGGGTCGACCTGCGGAGCCTGTGCCTCTTCAGCTACCATCGCTTGAGAGACTTACTCTTGATTGTAGCGAGGATTGTGGAACTTCTGGGACGCAGG---------------GGGTGGGAAGCCCTCAAATATTGGTGG---AATCTCCTGCGGTATTGG---------------------------------------------------AGCCAGGAACTAAGGAATAGTGCTATTAGCTTGCTTAATGCCACAGCCATAGCAGTAGCTGAGGGAACAGATAGGGTGGTAGAAGTATTACAAAGA------------------GCTTTTAGAGCTGTTATACACATACCTAGAAGAATAAGACAGGGCTTAGAAAGGTTTTTGCTATAA

2.04013240.ADARC.GU330461 ATGAGAGTGAAGGGGATCATGAGGAATTATCAGCACTTA---------TGGAGATGGGGC------------------------ATGATGCTCCTTGGGATAATCATGATC------------TGTAGTGCTGCA---------GAACAATTGTGGGTCACAATCTATTATGGGGTACCTGTGTGGAAAGAAGCAACCACCACTCTATTTTGTGCATCAAATGCTAAAGCATATGATACAGAGGTACATAAT---GTCTGGGCCACACATGCCTGTGTACCCACAGACCCCAACCCACAAGAAGTAAGATTG---GAAAATGTGACAGAAAATTTTAACATGTGGAAAAATAATATGGTAGAACAGATGCATGAGGATATAATTAGCCTATGGGATCAAAGCCTAAAACCATGTGTGAAATTAACCCCACTCTGTGTTACTTTAAACTGCACTGATGCTAATACCACTAATACTAATGCCAAT------------------------------------------------------------------------------AGTACTAATAACAGTAGCTTGGGAACAATGGAGAAAGGAGAAATAAAAAACTGCTCTTTCAACATCACC---ACAAACCTAAGAGAT------AAGGTGCAGAAAGAATATGCACTTTTTTATAACCTTGATGTAGTGCCAATAAAGGGTGAGGAT---------------------------AATACTAGC---------------------------TATAGGTTGATAAGTTGTAATACCTCAGTCATTACACAGGCCTGTCCAAAGGTATCCTTTGAGCCAATTCCCATACATTATTGTACTCCAGCTGGTTTTGCGATTCTACAATGT---AATGATAAGAAATTCAATGGATCAGGACCATGTACAAATGTCAGCACAGTACAATGTACACATGGAATTAGGCCAGTAGTATCAACTCAACTGCTGTTAAATGGCAGTCTAGCAGAAAAA---GAGGTAGTAATTAGGTCTGAGAATTTCACAAATAATGCTAAAACCATAATAGTACAGCTAAATGAATCTGTAGTAATTAATTGTACAAGACCCAACAACAATACAAGAAAAAGTATACCTATAGGA------------CCAGGAAGA---GCATTTTATGCAACAGGAGAAATAATAGGAGATATAAGACAAGCACATTGTAACGTT------AGTACAAAAGCCTGGAAGGAAGCTTTACAACAGGTAGCTATAAAACTATCA---GAACAATTTGGG------------AATAAAACA---ATAGTCTTTAATCAA---------TCCTCAGGAGGAGACCCAGAAGTTGTAATGCACAGTTTTAATTGTAGAGGGGAATTTTTCTACTGTAATACAACAGGACTGTTTAATAATACTTGGGGGTTT---------AATAGTACTTGG---------------------GATGCTATTAATGTGCCAGAA------------------AATGACACA------------------------------ATCACACTCCCATGCAGAATAAAACAAATTGTAAACATGTGGCAGGAAGTAGGAAAAGCAATGTATGCCCCTCCCATCAGAGGACGACTTAATTGTTCATCAAATATTACAGGGCTGCTATTAACAAGAGATGGTGGT------------------AATACCACA---------------------AATAACACT---GAGGTCTTCAGACCTGGAGGAGGAGATATGAGAGACAATTGGAGA---AGTGAATTATATAAATATAAAGTAGTAAAAATTGAACCA---TTAGGAATAGCACCC---ACCAAGGCAAAGAGAAGAGTGGTGCAGAGAGAA---AAAAGAGCAGTG---GGA---ATAGGA---GCTTTG---TTCCTT---GGG---------TTCTTGGGA---GCAGCAGGAAGCACTATGGGCGCAGCGTCGATG---ACGCTGACGGTACAGGCCAGACTATTATTGTCTGGTATAGTGCAACAGCAGAACAATTTGCTGAGAGCTATTGAGGCGCAACAGCATCTGTTGCAACTCACAGTCTGGGGCATCAAGCAGCTCCAGGCAAGA---GTCCTGGCTGTGGAAAGATACCTAAGAGATCAACAGCTCCTGGGGATTTGGGGATGCTCTGGAAAACTCATTTGCACCACTACTGTGCCTTGGAATGTTAGTTGGAGT---------------------------AATAAATCCATGAATGACATTTGGAAT---AACATGACCTGGATGGAGTGGGAAAGAGAGATTGAC------AATTATACAAACATAATATACACCTTACTTGAAGAATCGCAGAACCAACAAGATAAGAATGAACAGGAATTATTGGAATTGGATAAATGGGCAAGTTTGTGGAATTGGTTTAGCATAACAAATTGGCTGTGGTACATAAAAATATTCATAATGATAGTAGGAGGCTTAATAGGTTTAAGAATAGTTTTTACTGTATTTTCTATAGTGAATAGAGTTAGGCAGGGATATTCACCATTATCGTTTCAGACCCGC---TTCCCAACCTCGAGGGGA------CTCGACAGGCCCGAAGGAATCGAAGAAGAAGGTGGAGACAGAGACAGAGACAGATCCAGGCCATTAGTGGATGGATTCTTAGCAATTATCTGGGTCGACCTGCGGAGCCTGTGCCTCTTCAGCTACCATCGCTTGAGAGACTTACTCTTGATTGTAGCGAGGATTGTGGAACTTCTGGGACGCAGG---------------GGGTGGGAAGCCCTCAAATATTGGTGG---AATCTCCTGCGGTATTGG---------------------------------------------------AGCCAGGAACTAAGGAATAGTGCTATTAGCTTGCTTAATGCCACAGCCATAGCAGTAGCTGAGGGAACAGATAGGGTGTTAGAAGTATTACAAAGA------------------GCTTTTAGAGCTGTTATACACATACCTAGAAGAATAAGACAGGGCTTAGAAAGGTTTTTGCTATAA

2.04013291.ADARC.GU330499 ATGAGAGTGAAGGAGATCAGGAAGAGTTATCAGCACTTG---------TGGAGATGGGGC------------------------ATCATGCTCCTTGGGATATTGATGATC------------TGTAGAGCTTCA---------GAAAATTTGTGGGTCACAGTCTATTATGGGGTACCTGTGTGGAAAGAAGCAACCACCACTCTGTTTTGTGCATCAGATGCTAAAGCTTATGAGATAGAGGTACATAAT---GTTTGGGCCACACATGCCTGCGTACCCACAGACCCCAACCCACAAGAAGTAGTATTG---GTAAATGTGACAGAAGATTTTAATATGTGGAAAAATAACATGGTAGACCAAATGCATGAGGATATAATCAGTTTATGGGATCAAAGCCTAAAACCATGTGTAAAACTAACCCCACTCTGTGTCACTCTAAATTGCACTGATTATGTGGGGAATGCCACCAATACCAACAAGACC---------------------------------------------------------------------------ACTACCCCTGCCCCTACTAATAGCTGGGACAAGGGAGAAATAAAAAACTGCTCTTTCAATATCACC---ACAAATATAAGAGAT------AAGAGGCAAAAAGAATATGCACTATTTTATAAACTAGATGTAGTACCAATAGAT------------------------------------AATACTAGT---------------------AATAGTTATAGGTTGATAAATTGTAACACCTCAGTCATTACACAGGCCTGTCCAAAGGTATCCTTTGAGCCAATTCCCATACATTATTGTGCCCCGGCTGGTTTTGCGATTTTAAAGTGT---AATGAAAAAGGGTTCATAGGAACAGGAACATGTAAAAATGTCAGCACAGTACAATGTACACATGGAATTAAGCCAGTAGTATCCACTCAACTGCTGTTGAATGGCAGTCTAGCAGAAGAA---GGGATAGTAATAAGATCTGAGAATTTCTCAGACAATGCTAAAACCATAATAGTACAGCTGAATGAATCTGTAGTTATTAATTGTACAAGACCCAACAACAATACAAGAAAAGGTATACATATAGGA------------CCAGGGGCG---GCATTTTATGCAACAGGAGATATAATAGGAGATATAAGACAAGCACATTGTAACCTT------AGTAGAGCACAATGGAATAAAACTTTAAAACAGGTAGCTATAAAATTAAAA---GAACAATTT---------------AATAAAACAGTAATAGTCTTTAATAGA---------TCCTCAGGAGGGGACCCAGAAATTGTAATGCATAGTTTTAATTGTGGAGGGGAATTTTTCTACTGTAATACAACTAAGCTGTTTAATAATACTTGG---------------AATGGTACTAATAATAAT---------------------TGGAATGGTACTGAAATC------------------------------------------------------ATAACACTCCCATGCAGAATAAAACAAATTATAAACATGTGGCAAGAAGTAGGAAAAGCAATGTATGCCCCTCCCATCAGAGGACAGATTAACTGCTCGTCACATATTACAGGGCTGCTATTAACAAGAGATGGTGGC------------------AACAACACAGAC------------------AACAACAACACAGAGGTCTTCAGACCTGGAGGGGGAAATATGAAGGACAATTGGAGA---AGTGAATTATATAAATACAAAGTAGTAAAAATTGAACCA---TTAGGAGTAGCACCC---ACCAAGGCAAAGAGGAGAGTGGTGCAGAGAGAA---AAAAGAGCAGCA---------ATAGGA---GCTTTG---TTCCTT---GGG---------TTCTTGGGA---GCAGCAGGAAGCACTATGGGCGCAGCGTCACTG---ACGCTGACGGTACAGGCCAGACTATTATTGTCTGGTATAGTGCAACAGCAGAACAATTTGCTGAGGGCTATTGAGGCGCAACAGCATCTGTTGCAACTCACAGTCTGGGGCATCAAGCAGCTCCAGGCAAGA---GTCCTGGCTGTGGAAAGATACCTAAGGGATCAACAGCTCCTAGGGATTTGGGGTTGCTCTGGAAAAATCATTTGCACCACTGCTGTGCCTTGGAATAATACTTGGAGT---------------------------AATAAAAATCTGAGCCAGATTTGGGAT---AACATGACCTGGATGGAGTGGGAGAAAGAAATTGAT------AATTACACAGAAATAATATATGACTTGCTTGAAAAATCGCAAAACCAACAAGAAAAGAATGAACAAGAGTTATTGGAATTGGATAAATGGGCAAGTTTGTGGAATTGGTTTAGCATAACAAACTGGCTGTGGTATATAAAAATATTCATAATGATAGTAGGAGGCTTGATAGGTTTAAGAATAGTTTTTACTGTACTTTCTATAGTGAATAGAGTTAGGCAGGGATACTCACCATTGTCATTGCAGACCCTC---CTCCCAGCTCCGAGGGGA------CCCGGCAGGCCCGAAGGAACAGAAGAAGAAGGTGGAGAGCGAGACAGAGGCAGATCCGATCCATTAGCGACAGGATTCTTAGCACTTTTCTGGGACGACCTGAGGACCCTGTGCCTCTTCAGCTACCACCGCTTGAGAGACTTACTCTTGATTGTGACGAGGATTGTGGAACTTCTGGGACGCAGG---------------GGGTGGGAACTGCTCAAGTATTGGTGG---AATCTCCTACAGTATTGG---------------------------------------------------AGTCAGGAACTAAAGAATAGTGCTGTTAACTTGTTTGATACTGTAGCCATAGCAGCAGCTGAGGGGACAGATAGGGTTATAGAAATAATACAAAGA------------------GCTGGTAGAGCTATCCTCCACATCCCTAGAAGAATAAGACAGGGCGCGGAAAGGGCTTTGATATAA

2.04013291.ADARC.GU330500 ATGAGAGTGAAGGAGATCAGGAAGAGTTATCAGCACTTG---------TGGAGATGGGGC------------------------ATCATGCTCCTTGGGATATTGATGATC------------TGTAGAGCTTCA---------GAAAATTTGTGGGTCACAGTCTATTATGGGGTACCTGTGTGGAAAGAAGCAACCACCACTCTGTTTTGTGCATCAGATGCTAAAGCTTATGAGACAGAGGTACATAAT---GTTTGGGCCACACATGCCTGCGTACCCACAGACCCCAACCCACAAGAAGTAGTATTG---GTAAATGTGACAGAAGATTTTAATATGTGGAAAAATAACATGGTAGACCAAATGCATGAGGATATAATCAGTTTATGGGATCAAAGCCTAAAACCATGTGTAAAACTAACCCCACTCTGTGTCACTCTAAATTGCACTGATTATGTGGGGAATGCCACCAATACCAACAAGACC---------------------------------------------------------------------------ACTACCCCTGCCCCTACTAATAGCTGGGACAAGGGAGAAATAAAAAACTGCTCTTTCAATATCACC---ACAAATATAAGAGAT------AAGAGGCAAAAAGAATATGCACTATTTTATAAACTAGATGTAGTACCAATAGAT------------------------------------AATACTAGT---------------------AATAGTTATAGGTTGATAAATTGTAACACCTCAGTCATTACACAGGCCTGTCCAAAGGTATCCTTTGAGCCAATTCCCATACATTATTGTGCCCCGGCTGGTTTTGCGATTTTAAAGTGT---AATGAAAAAGGGTTCATAGGAACAGGAACATGTAAAAATGTCAGCACAGTACAATGTACACATGGAATTAAGCCAGTAGTATCCACTCAACTGCTGTTGAATGGCAGTCTAGCAGAAGAA---GGGATAGTAATAAGATCTGAGAATTTCTCAGACAATGCTAAAACCATAATAGTACAGCTGAATGAATCTGTAGTTATTAATTGTACAAGACCCAACAACAATACAAGAAAAGGTATACATATAGGA------------CCAGGGGCG---GCATTTTATGCAACAGGAGATATAATAGGAGATATAAGACAAGCACATTGTAACCTT------AGTAGAGCACAATGGAATAAAACTTTAAAACAGGTAGCTATAAAATTAAAA---GAACAATTT---------------AATAAAACAGTAATAGTCTTTAATAGA---------TCCTCAGGAGGGGACCCAGAAATTGTAATGCATAGTTTTAATTGTGGAGGGGAATTTTTCTACTGTAATACAACTAAGCTGTTTAATAATACTTGG---------------AATGGTACTAATAATAAT---------------------TGGAATGGTACTGAAATC------------------------------------------------------ATAACACTCCCATGCAGAATAAAACAAATTATAAACATGTGGCAAGAAGTAGGAAAAGCAATGTATGCCCCTCCCATCAGAGGACAGATTAACTGCTCGTCACATATTACAGGGCTGCTATTAACAAGAGATGGTGGC------------------AACAACACAGAC------------------AACAACAACACAGAGGTCTTCAGACCTGGAGGGGGAAATATGAAGGACAATTGGAGA---AGTGAATTATATAAATACAAAGTAGTAAAAATTGAACCA---TTAGGAGTAGCACCC---ACCAAGGCAAAGAGGAGAGTGGTGCAGAGAGAA---AAAAGAGCAGCA---------ATAGGA---GCTTTG---TTCCTT---GGG---------TTCTTGGGA---GCAGCAGGAAGCACTATGGGCGCAGCGTCACTG---ACGCTGACGGTACAGGCCAGACTATTATTGTCTGGTATAGTGCAACAGCAGAACAATTTGCTGAGGGCTATTGAGGCGCAACAGCATCTGTTGCAACTCACAGTCTGGGGCATCAAGCAGCTCCAGGCAAGA---GTCCTGGCTGTGGAAAGATACCTAAGGGATCAACAGCTCCTAGGGATTTGGGGTTGCTCTGGAAAAATCATTTGCACCACTGCTGTGCCTTGGAATAATACTTGGAGT---------------------------AATAAAAATCTGAGCCAGATTTGGGAT---AACATGACCTGGATGGAGTGGGAGAAAGAAATTGAT------AATTACACAGAAATAATATATGACTTGCTTGAAAAATCGCAAAACCAACAAGAAAAGAATGAACAAGAGTTATTGGAATTGGATAAATGGGCAAGTTTGTGGAATTGGTTTAGCATAACAAACTGGCTGTGGTATATAAAAATATTCATAATGATAGTAGGAGGCTTGATAGGTTTAAGAATAGTTTTTACTGTACTTTCTATAGTGAATAGAGTTAGGCAGGGATACTCACCATTGTCATTGCAGACCCTC---CTCCCAGCTCCGAGGGGA------CCCGGCAGGCCCGAAGGAACAGAAGAAGAAGGTGGAGAGCGAGACAGAGGCAGATCCGATCCATTAGCGACAGGATTCTTAGCACTTTTCTGGGACGACCTGAGGACCCTGTGCCTCTTCAGCTACCACCGCTTGAGAGACTTACTCTTGATTGTGACGAGGATTGTGGAACTTCTGGGACGCAGG---------------GGGTGGGAACTGCTCAAGTATTGGTGG---AATCTCCTACAGTATTGG---------------------------------------------------AGTCAGGAACTAAAGAATAGTGCTGTTAACTTGTTTGATACTGTAGCCATAGTAGCAGCTGAGGGGACAGATAGGGTTATAGAAATAATACAAAGA------------------GCTGGTAGAGCTATCCTCCACATCCCTAGAAGAATAAGACAGGGCGCGGAAAGGGCTTTGATATAA

2.04013291.ADARC.GU330501 ATGAGAGTGAAGGAGATCAGGAAGAGTTATCAGCACTTG---------TGGAGATGGGGC------------------------ATCATGCTCCTTGGGATATTGATGATC------------TGTAGAGCTTCA---------AAAAATTTGTGGGTCACAGTCTATTATGGGGTACCTGTGTGGAAAGAAGCAACCACCACTCTGTTTTGTGCATCAGATGCTAAAGCTTATGAGACAGAGGTACATAAT---GTTTGGGCCACACATGCCTGCGTACCCACAGACCCCAACCCACAAGAAGTAGTATTG---GTAAATGTGACAGAAGATTTTAATATGTGGAAAAATAACATAGTAGACCAAATGCATGAGGATATAATCAGTTTATGGGATCAAAGCCTAAAACCATGTGTAAAACTAACCCCACTCTGTGTCACTCTAAATTGCACTGATTATGTGGGGAATGCCACCAATACCAACAAGACC---------------------------------------------------------------------------ACTACCCCTGCCCCTACTAATAGCTGGGACAAGGGAGAAATAAAAAACTGCTCTTTCAATATCACC---ACAAATATAAGAGAT------AAGAGGCAAAAAGAATATGCACTATTTTATAAACTAGATGTAGTACCAATAGAT------------------------------------AATACTAGT---------------------AATAGTTATAGGTTGATAAATTGTAACACCTCAGTCATTACACAGGCCTGTCCAAAGGTATCCTTTGAGCCAATTCCCATACATTATTGTGCCCCGGCTGGTTTTGCGATTTTAAAGTGT---AATGAAAAAGGGTTCATAGGAACAGGAACATGTAAAAATGTCAGCACAGTACAATGTACACATGGAATTAAGCCAGTAGTATCCACTCAACTGCTGTTGAATGGCAGTCTAGCAGAAGAA---GGGATAGTAATAAGATCTGAGAATTTCTCAGACAATGCTAAAACCATAATAGTACAGCTGAATGAATCTGTAGTTATTAATTGTACAAGACCCAACAACAATACAAGAAAAGGTATACATATAGGA------------CCAGGGGCG---GCATTTTATGCAACAGGAGATATAATAGGAGATATAAGACAAGCACATTGTAACCTT------AGTAGAGCACAATGGAATAAAACTTTAAAACAGGTAGCTATAAAATTAAAA---GAACAATTT---------------AATAAAGCAGTAATAGTCTTTAATAGA---------TCCTCAGGAGGGGACCCAGAAATTGTAATGCATAGTTTTAATTGTGGAGGGGAATTTTTCTACTGTAATACAACTAAGCTGTTTAATAATACTTGG---------------AATGGTACTAATAATAAT---------------------TGGAATGGTACTGAAATC------------------------------------------------------ATAACACTCCCATGCAGAATAAAACAAATTATAAACATGTGGCAAGAAGTAGGAAAAGCAATGTATGCCCCTCCCATCAGAGGACAGATTAACTGCTCGTCACATATTACAGGGCTGCTATTAACAAGAGATGGTGGC------------------AACAACACAGAC------------------AACAACAACACAGAGGTCTTCAGACCTGGAGGGGGAAATATGAAGGACAATTGGAGA---AGTGAATTATATAAATACAAAGTAGTAAAAATTGAACCA---TTAGGAGTAGCACCC---ACCAAGGCAAAGAGGAGAGTGGTGCAGAGAGAA---AAAAGAGCAGCA---------ATAGGA---GCTTTG---TTCCTT---GGG---------TTCTTGGGA---GCAGCAGGAAGCACTATGGGCGCAGCGTCACTG---ACGCTGACGGTACAGGCCAGACTATTATTGTCTGGTATAGGGCAACAGCAGAACAATTTGCTGAGGGCTATTGAGGCGCAACAGCATCTGTTGCAACTCACAGTCTGGGGCATCAAGCAGCTCCAGGCAAGA---GTCCTGGCTGTGGAAAGATACCTAAGGGATCAACAGCTCCTAGGGATTTGGGGTTGCTCTGGAAAAATCATTTGCACCACTGCTGTGCCTTGGAATAATACTTGGAGT---------------------------AATAAAAATCTGAGCCAGATTTGGGAT---AACATGACCTGGATGGAGTGGGAGAAAGAAATTGAT------AATTACACAGAAATAATATATGACTTGCTTGAAAAATCGCAAAACCAACAAGAAAAGAATGAACAAGAGTTATTGGAATTGGATAAATGGGCAAGTTTGTGGAATTGGTTTAGCATAACAAACTGGCTGTGGTATATAAAAATATTCATAATGATAGTAGGAGGCTTGATAGGTTTAAGAATAGTTTTTACTGTACTTTCTATAGTGAATAGAGTTAGGCAGGGATACTCACCATTGTCATTGCAGACCCTC---CTCCCAGCTCCGAGGGGA------CCCGGCAGGCCCGAAGGAACAGAAGAAGAAGGTGGAGAGCGAGACAGAGGCAGATCCGATCCATTAGCGACAGGATTCTTAGCACTTTTCTGGGACGACCTGAGGACCCTGTGCCTCTTCAGCTACCACCGCTTGAGAGACTTACTCTTGATTGTGACGAGGATTGTGGAACTTCTGGGACGCAGG---------------GGGTGGGAACTGCTCAAGTATTGGTGG---AATCTCCTACAGTATTGG---------------------------------------------------AGTCAGGAACTAAAGAATAGTGCTGTTAACTTGTTTGATACTGTAGCCATAGCAGCAGCTGAGGGGACAGATAGGGTTATAGAAATAATACAAAGA------------------GCTGGTAGAGCTATCCTCCACATCCCTAGAAGAATAAGACAGGGCGCGGAAAGGGCTTTGATATAA

2.04013291.ADARC.GU330502 ATGAGAGTGAAGGAGATCAGGAAGAGTTATCAGCACTTG---------TGGAGATGGGGC------------------------ATCATGCTCCTTGGGATATTGATGATC------------TGTAGAGCTTCA---------GAAAATTTGTGGGTCACAGTCTATTATGGGGTACCTGTGTGGAAAGAAGCAACCACCACTCTGTTTTGTGCATCAGATGCTAAAGCTTATGAGATAGAGGTACATAAT---GTTTGGGCCACACATGCCTGCGTACCCACAGACCCCAACCCACAAGAAGTAGTATTG---GTAAATGTGACAGAAGATTTTAATATGTGGAAAAATAACATGGTAGACCAAATGCATGAGGATATAATCAGTTTATGGGATCAAAGCCTAAAACCATGTGTAAAACTAACCCCACTCTGTGTCACTCTAAATTGCACTGATTATGTGGGGAATGCCACCAATACCAACAAGACC---------------------------------------------------------------------------ACTACCCCTGCCCCTACTAATAGCTGGGACAAGGGAGAAATAAAAAACTGCTCTTTCAATATCACC---ACAAATATAAGAGAT------AAGAGGCAAAAAGAATATGCACTATTTTATAAACTAGATGTAGTACCAATAGAT------------------------------------AATACTAGT---------------------AATAGTTATAGGTTGATAAATTGTAACACCTCAGTCATTACACAGGCCTGTCCAAAGGTATCCTTTGAGCCAATTCCCATACATTATTGTGCCCCGGCTGGTTTTGCGATTTTAAAGTGT---AATGAAAAAGGGTTCATAGGAACAGGAACATGTAAAAATGTCAGCACAGTACAATGTACACATGGAATTAAGCCAGTAGTATCCACTCAACTGCTGTTGAATGGCAGTCTAGCAGAAGAA---GGGATAGTAATAAGATCTGAGAATTTCTCAGACAATGCTAAAACCATAATAGTACAGCTGAATGAATCTGTAGTTATTAATTGTACAAGACCCAACAACAATACAAGAAAAGGTATACATATAGGA------------CCAGGGGCG---GCATTTTATGCAACAGGAGATATAATAGGAGATATAAGACAAGCACATTGTAACCTT------AGTAGAGCACAATGGAATAAAACTTTAAAACAGGTAGCTATAAAATTAAAA---GAACAAGTT---------------AATAAAACAGTAATAGTCTTTAATAGA---------TCCTCAGGAGGGGACCCAGAAATTGTAATGCATAGTTTTAATTGTGGAGGGGAATTTTTCTACTGTAATACAACTAAGCTGTTTAATAATACTTGG---------------AATGGTACTAATAATAAT---------------------TGGAATGGTACTGAAATC------------------------------------------------------ATAACACTCCCATGCAGAATAAAACAAATTATAAACATGTGGCAAGAAGTAGGAAAAGCAATGTATGCCCCTCCCATCAGAGGACAGATTAACTGCTCGTCACATATTACAGGGCTGCTATTAACAAGAGATGGTGGC------------------AACAACACAGAC------------------AACAACAACACAGAGGTCTTCAGACCTGGAGGGGGAAATATGAAGGACAATTGGAGA---AGTGAATTATATAAATACAAAGTAGTAAAAATTGAACCA---TTAGGAGTAGCACCC---ACCAAGGCAAAGAGGAGAGTGGTGCAGAGAGAA---AAAAGAGCAGCA---------ATAGGA---GCTTTG---TTCCTT---GGG---------TTCTTGGGA---GCAGCAGGAAGCACTATGGGCGCAGCGTCACTG---ACGCTGACGGTACAGGCCAGACTATTATTGTCTGGTATAGTGCAACAGCAGAACAATTTGCTGAGGGCTATTGAGGCGCAACAGCATCTGTTGCAACTCACAGTCTGGGGCATCAAGCAGCTCCAGGCAAGA---GTCCTGGCTGTGGAAAGATACCTAAGGGATCAACAGCTCCTAGGGATTTGGGGTTGCTCTGGAAAAATCATTTGCACCACTGCTGTGCCTTGGAATAATACTTGGAGT---------------------------AATAAAAATCTGAGCCAGATTTGGGAT---AACATGACCTGGATGGAGTGGGAGAAAGAAATTGAT------AATTACACAGAAATAATATATGACTTGCTTGAAAAATCGCAAAACCAACAAGAAAAGAATGAACAAGAGTTATTGGAATTGGATAAATGGGCAAGTTTGTGGAATTGGTTTAGCATAACAAACTGGCTGTGGTATATAAAAATATTCATAATGATAGTAGGAGGCTTGATAGGTTTAAGAATAGTTTTTACTGTACTTTCTATAGTGAATAGAGTTAGGCAGGGATACTCACCATTGTCATTGCAGACCCTC---CTCCCAGCTCCGAGGGGA------CCCGGCAGGCCCGAAGGAACAGAAGAAGAAGGTGGAGAGCGAGACAGAGGCAGATCCGATCCATTAGCGACAGGATTCTTAGCACTTTTCTGGGACGACCTGAGGACCCTGTGCCTCTTCAGCTACCACCGCTTGAGAGACTTACTCTTGATTGTGACGAGGATTGTGGAACTTCTGGGACGCAGG---------------GGGTGGGAACTGCTCAAGTATTGGTGG---AATCTCCTACAGTATTGG---------------------------------------------------AGTCAGGAACTAAAGAATAGTGCTGTTAACTTGTTTGATACTGTAGCCATAGCAGCAGCTGAGGGGACAGATAGGGTTATAGAAATAATACAAAGA------------------GCTGGTAGAGCTATCCTCCACATCCCTAGAAGAATAAGACAGGGCGCGGAAAGGGCTTTGATATAA

2.04013291.ADARC.GU330503 ATGAGAGTGAAGGAGATCAGGAAGAGTTATCAGCACTTG---------TGGAGATGGGGC------------------------ATCATGCTCCTTGGGATATTGATGATC------------TGTAGAGCTTCA---------GAAAATTTGTGGGTCACAGTCTATTATGGGGTACCTGTGTGGAAAGAAGCAACCACCACTCTGTTTTGTGCATCAGATGCTAAAGCTTATGAGACAGAGGTACATAAT---GTTTGGGCCACACATGCCTGCGTACCCACAGACCCCAACCCACAAGAAGTAGTATTG---GTAAATGTGACAGAAGATTTTAATATGTGGAAAAATAACATGGTAGACCAAATGCATGAGGATATAATCAGTTTATGGGATCAAAGCCTAAAACCATGTGTAAAACTAACCCCACTCTGTGTCACTCTAAATTGCACTGATTATGTGGGGAATGCCACCAATACCAACAAGACC---------------------------------------------------------------------------ACTACCCCTGCCCCTACTAATAGCTGGGACAAGGGAGAAATAAAAAACTGCTCTTTCAATATCACC---ACAAATATAAGAGAT------AAGAGGCAAAAAGAATATGCACTATTTTATAAACTAGATGTAGTACCAATAGAT------------------------------------AATACTAGT---------------------AATAGTTATAGGTTGATAAATTGTAACACCTCAGTCATTACACAGGCCTGTCCAAAGGTATCCTTTGAGCCAATTCCCATACATTATTGTGCCCCGGCTGGTTTTGCGATTTTAAAGTGT---AATGAAAAAGGGTTCATAGGAACAGGAACATGTAAAAATGTCAGCACAGTACAATGTACACATGGAATTAAGCCAGTAGTATCCACTCAACTGCTGTTGAATGGCAGTCTAGCAGAAGAA---GGGATAGTAATAAGATCTGAGAATTTCTCAGACAATGCTAAAACCATAATAGTACAGCTGAATGAATCTGTAGTTATTAATTGTACAAGACCCAACAACAATACAAGAAAAGGTATACATATAGGA------------CCAGGGGCG---GCATTTTATGCAACAGGAGATATAATAGGAGATATAAGACAAGCACATTGTAACCTT------AGTAGAGCACAATGGAATAAAACTTTAAAACAGGTAGCTATAAAATTAAAA---GAACAATTT---------------AATAAAACAGTAATAGTCTTTAATAGA---------TCCTCAGGAGGGGACCCAGAAATTGTAATGCATAGTTTTAATTGTGGAGGGGAATTTTTCTACTGTAATACAACTAAGCTGTTTAATAATACTTGG---------------AATGGTACTAATAATAAT---------------------TGGAATGGTACTGAAATC------------------------------------------------------ATAACACTCCCATGCAGAATAAAACAAATTATAAACATGTGGCAAGAAGTAGGAAAAGCAATGTATGCCCCTCCCATCAGAGGACAGATTAACTGCTCGTCACATATTACAGGGCTGCTATTAACAAGAGATGGTGGC------------------AACAACACAGAC------------------AACAACAACACAGAGGTCTTCAGACCTGGAGGGGGAAATATGAAGGACAATTGGAGA---AGTGAATTATATAAATACAAAGTAGTAAAAATTGAACCA---TTAGGAGTAGCACCC---ACCAAGGCAAAGAGGAGAGTGGTGCAGAGAGAA---AAAAGAGCAGCA---------ATAGGA---GCTTTG---TTCCTT---GGG---------TTCTTGGGA---GCAGCAGGAAGCACTATGGGCGCAGCGTCACTG---ACGCTGACGGTACAGGCCAGACTATTATTGTCTGGTATAGTGCAACAGCAGAACAATTTGCTGAGGGCTATTGAGGCGCAACAGCATCTGTTGCAACTCACAGTCTGGGGCATCAAGCAGCTCCAGGCAAGA---GTCCTGGCTGTGGAAAGATACCTAAGGGATCAACAGCTCCTAGGGATTTGGGGTTGCTCTGGAAAAATCATTTGCACCACTGCTGTGCCTTGGAATAATACTTGGAGT---------------------------AATAAAAATCTGAGCCAGATTTGGGAT---AACATGACCTGGATGGAGTGGGAGAAAGAAATTGAT------AATTACACAGAAATAATATATGACTTGCTTGAAAAATCGCAAAACCAACAAGAAAAGAATGAACAAGAGTTATTGGAATTGGATAAATGGGCAAGTTTGTGGAATTGGTTTAGCATAACAAACTGGCTGTGGTATATAAAAATATTCATAATGATAGTAGGAGGCTTGATAGGTTTAAGAATAGTTTTTACTGTACTTTCTATAGTGAATAGAGTTAGGCAGGGATACTCACCATTGTCATTGCAGACCCTC---CTCCCAGCTCCGAGGGGA------CCCGGCAGGCCCGAAGGAACAGAAGAAGAAGGTGGAGAGCGAGACAGAGGCAGATCCGATCCATTAGCGACAGGATTCTTAGCACTTTTCTGGGACGACCTGAGGACCCTGTGCCTCTTCAGCTACCACCGCTTGAGAGACTTACTCTTGATTGTGACGAGGATTGTGGAACTTCTGGGACGCAGG---------------GGGTGGGAACTGCTCAAGTATTGGTGG---AATCTCCTACAGTATTGG---------------------------------------------------AGTCAGGAACTAAAGAATAGTGCTGTTAACTTGTTTGATACTGTAGCCATAGCAGCAGCTGAGGGGACAGATAGGGTTATAGAAATAATACAAAGA------------------GCTGGTAGAGCTATCCTCCACATCCCTAGAAGAATAAGACAGGGCGCGGAAAGGGCTTTGATATAA

2.04013291.ADARC.GU330504 ATGAGAGTGAAGGAGATCAGGAAGAGTTATCAGCACTTG---------TGGAGATGGGGC------------------------ATCATGCTCCTTGGGATATTGATGATC------------TGTAGAGCTTCA---------GAAAATTTGTGGGTCACAGTCTATTATGGGGTACCTGTGTGGAAAGAAGCAACCACCACTCTGTTTTGTGCATCAGATGCTAAAGCTTATGAGACAGAGGTACATAAT---GTTTGGGCCACACATGCCTGCGTACCCACAGACCCCAACCCACAAGAAGTAGTATTG---GTAAATGTGACAGAAGATTTTAATATGTGGAAAAATAACATGGTAGACCAAATGCATGAGGATATAATCAGTTTATGGGATCAAAGCCTAAAACCATGTGTAAAACTAACCCCACTCTGTGTCACTCTAAATTGCACTGATTATGTGGGGAATGCCACCAATACCAACAAGACC---------------------------------------------------------------------------ACTACCCCTGCCCCTACTAATAGCTGGGACAAGGGAGAAATAAAAAACTGCTCTTTCAATATCACC---ACAAATATAAGAGAT------AAGAGGCAAAAAGAATATGCACTATTTTATAAACTAGATGTAGTACCAATAGAT------------------------------------AATACTAGT---------------------AATAGTTATAGGTTGATAAATTGTAACACCTCAGTCATTACACAGGCCTGTCCAAAGGTATCCTTTGAGCCAATTCCCATACATTATTGTGCCCCGGCTGGTTTTGCGATTTTAAAGTGT---AATGAAAAAGGGTTCATAGGAACAGGAACATGTAAAAATGTCAGCACAGTACAATGTACACATGGAATTAAGCCAGTAGTATCCACTCAACTGCTGTTGAATGGCAGTCTAGCAGAAGAA---GGGATAGTAATAAGATCTGAGAATTTCTCAGACAATGCTAAAACCATAATAGTACAGCTGAATGAATCTGTAGTTATTAATTGTACAAGACCCAACAACAATACAAGAAAAGGTATACATATAGGA------------CCAGGGGCG---GCATTTTATGCAACAGGAGATATAATAGGAGATATAAGACAAGCACATTGTAACCTT------AGTAGAGCACAATGGAATAAAACTTTAAAACAGGTAGCTATAAAATTAAAA---GAACAATTT---------------AATAAAAAAGTAATAGTCTTTAATAGA---------TCCTCAGGAGGGGACCCAGAAATTGTAATGCATAGTTTTAATTGTGGAGGGGAATTTTTCTACTGTAATACAACTAAGCTGTTTAATAATACTTGG---------------AATGGTACTAATAATAAT---------------------TGGAATGGTACTGAAATC------------------------------------------------------ATAACACTCCCATGCAGAATAAAACAAATTATAAACATGTGGCAAGAAGTAGGAAAAGCAATGTATGCCCCTCCCATCAGAGGACAGATTAACTGCTCGTCACATATTACAGGGCTGCTATTAACAAGAGATGGTGGC------------------AACAACACAGAC------------------AACAACAACACAGAGGTCTTCAGACCTGGAGGGGGAAATATGAAGGACAATTGGAGA---AGTGAATTATATAAATACAAAGTAGTAAAAATTGAACCA---TTAGGAGTAGCACCC---ACCAAGGCAAAGAGGAGAGTGGTGCAGAGAGAA---AAAAGAGCAGCA---------ATAGGA---GCTTTG---TTCCTT---GGG---------TTCTTGGGA---GCAGCAGGAAGCACTATGGGCGCAGCGTCACTG---ACGCTGACGGTACAGGCCAGACTATTATTGTCTGGTATAGTGCAACAGCAGAACAATTTGCTGAGGGCTATTGAGGCGCAACAGCATCTGTTGCAACTCACAGTCTGGGGCATCAAGCAGCTCCAGGCAAGA---GTCCTGGCTGTGGAAAGATACCTAAGGGATCAACAGCTCCTAGGGATTTGGGGTTGCTCTGGAAAAATCATTTGCACCACTGCTGTGCCTTGGAATAATACTTGGAGT---------------------------AATAAAAATCTGAGCCAGATTTGGGAT---AACATGACCTGGATGGAGTGGGAGAAAGAAATTGAT------AATTACACAGAAATAATATATGACTTGCTTGAAAAATCGCAAAACCAACAAGAAAAGAATGAACAAGAGTTATTGGAATTGGATAAATGGGCAAGTTTGTGGAATTGGTTTAGCATAACAAACTGGCTGTGGTATATAAAAATATTCATAATGATAGTAGGAGGCTTGATAGGTTTAAGAATAGTTTTTACTGTACTTTCTATAGTGAATAGAGTTAGGCAGGGATACTCACCATTGTCATTGCAGACCCTC---CTCCCAGCTCCGAGGGGA------CCCGGCAGGCCCGAAGGAACAGAAGAAGAAGGTGGAGAGCGAGACAGAGGCAGATCCGATCCATTAGCGACAGGATTCTTAGCACTTTTCTGGGACGACCTGAGGACCCTGTGCCTCTTCAGCTACCACCGCTTGAGAGACTTACTCTTGATTGTGACGAGGATTGTGGAACTTCTGGGACGCAGG---------------GGGTGGGAACTGCTCAAGTATTGGTGG---AATCTCCTACAGTATTGG---------------------------------------------------AGTCAGGAACTAAAGAATAGTGCTGTTAACTTGTTTGATACTGTAGCCATAGCAGCAGCTGAGGGGACAGATAGGGTTATAGAAATAATACAAAGA------------------GCTGGTAGAGCTATCCTCCACATCCCTAGAAGAATAAGACAGGGCGCGGAAAGGGCTTTGATATAA

2.04013291.ADARC.GU330505 ATGAGAGTGAAGGAGATCAGGAAGAGTTATCAGCACTTG---------TGGAGATGGGGC------------------------ATCATGCTCCTTGGGATATTGATGATC------------TGTAGAGCTTCA---------GAAAATTTGTGGGTCACAGTCTATTATGGGGTACCTGTGTGGAAAGAAGCAACCACCACTCTGTTTTGTGCATCAGATGCTAAAGCTTATGAGACAGAGGTACATAAT---GTTTGGGCCACACATGCCTGCGTACCCACAGACCCCAACCCACAAGAAGTAGTATTG---GTAAATGTGACAGAAGATTTTAATATGTGGAAAAATAACATGGTAGACCAAATGCATGAGGATATAATCAGTTTATGGGATCAAAGCCTAAAACCATGTGTAAAACTAACCCCACTCTGTGTCACTCTAAATTGCACTGATTATGTGGGGAATGCCACCAATACCAACAAGACC---------------------------------------------------------------------------ACTACCCCTGCCCCTACTAATAGCTGGGACAAGGGAGAAATAAAAAACTGCTCTTTCAATATCACC---ACAAATATAAGAGAT------AAGAGGCAAAAAGAATATGCACTATTTTATAAACTAGATGTAGTACCAATAGAT------------------------------------AATACTAGT---------------------AATAGTTATAGGTTGATAAATTGTAACACCTCAGTCATTACACAGGCCTGTCCAAAGGTATCCTTTGAGCCAATTCCCATACATTATTGTGCCCCGGCTGGTTTTGCGATTTTAAAGTGT---AATGAAAAAGGGTTCATAGGAACAGGAACATGTAAAAATGTCAGCACAGTACAATGTACACATGGAATTAAGCCAGTAGTATCCACTCAACTGCTGTTGAATGGCAGTCTAGCAGAAGAA---GGGATAGTAATAAGATCTGAGAATTTCTCAGACAATGCTAAAACCATAATAGTACAGCTGAATGAATCTGTAGTTATTAATTGTACAAGACCCAACAACAATACAAGAAAAGGTATACATATAGGA------------CCAGGGGCG---GCATTTTATGCAACAGGAGATATAATAGGAGATATAAGACAAGCACATTGTAACCTT------AGTAGAGCACAATGGAATAAAACTTTAAAACAGGTAGCTATAAAATTAAAA---GAACAATTT---------------AATAAAACAGTAATAGTCCTTAATAGA---------TCCTCAGGAGGGGACCCAGAAATTGTAATGCATAGTTTTAATTGTGGAGGGGAATTTTTCTACTGTAATACAACTAAGCTGTTTAATAATACTTGG---------------AATGGTACTAATAATAAT---------------------TGGAATGGTACTGAAATC------------------------------------------------------ATAACACTCCCATGCAGAATAAAACAAATTATAAACATGTGGCAAGAAGTAGGAAAAGCAATGTATGCCCCTCCCATCAGAGGACAGATTAACTGCTCGTCACATATTACAGGGCTGCTATTAACAAGAGATGGTGGC------------------AACAACACAGAC------------------AACAACAACACAGAGGTCTTCAGACCTGGAGGGGGAAATATGAAGGACAATTGGAGA---AGTGAATTATATAAATACAAAGTAGTAAAAATTGAACCA---TTAGGAGTAGCACCC---ACCAAGGCAAAGAGGAGAGTGGTGCAGAGAGAA---AAAAGAGCAGCA---------ATAGGA---GCTTTG---TTCCTT---GGG---------TTCTTGGGA---GCAGCAGGAAGCACTATGGGCGCAGCGTCACTG---ACGCTGACGGTACAGGCCAGACTATTATTGTCTGGTATAGTGCAACAGCAGAACAATTTGCTGAGGGCTATTGAGGCGCAACAGCATCTGTTGCAACTCACAGTCTGGGGCATCAAGCAGCTCCAGGCAAGA---GTCCTGGCTGTGGAAAGATACCTAAGGGATCAACAGCTCCTAGGGATTTGGGGTTGCTCTGGAAAAATCATTTGCACCACTGCTGTGCCTTGGAATAATACTTGGAGT---------------------------AATAAAAATCTGAGCCAGATTTGGGAT---AACATGACCTGGATGGAGTGGGAGAAAGAAATTGAT------AATTACACAGAAATAATATATGACTTGCTTGAAAAATCGCAAAACCAACAAGAAAAGAATGAACAAGAGTTATTGGAATTGGATAAATGGGCAAGTTTGTGGAATTGGTTTAGCATAACAAACTGGCTGTGGTATATAAAAATATTCATAATGATAGTAGGAGGCTTGATAGGTTTAAGAATAGTTTTTACTGTACTTTCTATAGTGAATAGAGTTAGGCAGGGATACTCACCATTGTCATTGCAGACCCTC---CTCCCAGCTCCGAGGGGA------CCCGGCAGGCCCGAAGGAACAGAAGAAGAAGGTGGAGAGCGAGACAGAGGCAGATCCGATCCATTAGCGACAGGATTCTTAGCACTTTTCTGGGACGACCTGAGGACCCTGTGCCTCTTCAGCTACCACCGCTTGAGAGACTTACTCTTGATTGTGACGAGGATTGTGGAACTTCTGGGACGCAGG---------------GGGTGGGAACTGCTCAAGTATTGGTGG---AATCTCCTACAGTATTGG---------------------------------------------------AGTCAGGAACTAAAGAATAGTGCTGTTAACTTGTTTGATACTGTAGCCATAGCAGCAGCTGAGGGGACAGATAGGGTTATAGAAATAATACAAAGA------------------GCTGGTAGAGCTATCCTCCACATCCCTAGAAGAATAAGACAGGGCGCGGAAAGGGCTTTGATATAA

2.04013291.ADARC.GU330506 ATGAGAGTGAAGGAGATCAGGAAGAGTTATCAGCACTTG---------TGGAGATGGGGC------------------------ATCATGCTCCTTGGGATATTGATGATC------------TGTAGAGCTTCA---------GAAAATTTGTGGGTCACAGTCTATTATGGGGTACCTGTGTGGAAAGAAGCAACCACCACTCTGTTTTGTGCATCAGATGCTAAAGCTTATGAGATAGAGGTACATAAT---GTTTGGGCCACACATGCCTGCGTACCCACAGACCCCAACCCACAAGAAGTAGTATTG---GTAAATGTGACAGAAGATTTTAATATGTGGAAAAATAACATGGTAGACCAAATGCATGAGGATATAATCAGTTTATGGGATCAAAGCCTAAAACCATGTGTAAAACTAACCCCACTCTGTGTCACTCTAAATTGCACTGATTATGTGGGGAATGCCACCAATACCAACAAGACC---------------------------------------------------------------------------ACTACCCCTGCCCCTACTAATAGCTGGGACAAGGGAGAAATAAAAAACTGCTCTTTCAATATCACC---ACAAATATAAGAGAT------AAGAGGCAAAAAGAATATGCACTATTTTATAAACTAGATGTAGTACCAATAGAT------------------------------------AATACTAGT---------------------AATAGTTATAGGTTGATAAATTGTAACACCTCAGTCATTACACAGGCCTGTCCAAAGGTATCCTTTGAGCCAATTCCCATACATTATTGTGCCCCGGCTGGTTTTGCGATTTTAAAGTGT---AATGAAAAAGGGTTCATAGGAACAGGAACATGTAAAAATGTCAGCACAGTACAATGTACACATGGAATTAAGCCAGTAGTATCCACTCAACTGCTGTTGAATGGCAGTCTAGCAGAAGAA---GGGATAGTAATAAGATCTGAGAATTTCTCAGACAATGCTAAAACCATAATAGTACAGCTGAATGAATCTGTAGTTATTAATTGTACAAGACCCAACAACAATACAAGAAAAGGTATACATATAGGA------------CCAGGGGCG---GCATTTTATGCAACAGGAGATATAATAGGAGATATAAGACAAGCACATTGTAACCTT------AGTAGAGCACAATGGAATAAAACTTTAAAACAGGTAGCTATAAAATTAAAA---GAACAATTT---------------AATAAAACAGTACTAGTCTTTAATAGA---------TCCTCAGGAGGGGACCCAGAAATTGTAATGCATAGTTTTAATTGTGGAGGGGAATTTTTCTACTGTAATACAACTAAGCTGTTTAATAATACTTGG---------------AATGGTACTAATAATAAT---------------------TGGAATGGTACTGAAATC------------------------------------------------------ATAACACTCCCATGCAGAATAAAACAAATTATAAACATGTGGCAAGAAGTAGGAAAAGCAATGTATGCCCCTCCCATCAGAGGACAGATTAACTGCTCGTCACATATTACAGGGCTGCTATTAACAAGAGATGGTGGC------------------AACAACACAGAC------------------AACAACAACACAGAGGTCTTCAGACCTGGAGGGGGAAATATGAAGGACAATTGGAGA---AGTGAATTATATAAATACAAAGTAGTAAAAATTGAACCA---TTAGGAGTAGCACCC---ACCAAGGCAAAGAGGAGAGTGGTGCAGAGAGAA---AAAAGAGCAGCA---------ATAGGA---GCTTTG---TTCCTT---GGG---------TTCTTGGGA---GCAGCAGGAAGCACTATGGGCGCAGCGTCACTG---ACGCTGACGGTACAGGCCAGACTATTATTGTCTGGTATAGTGCAACAGCAGAACAATTTGCTGAGGGCTATTGAGGCGCAACAGCATCTGTTGCAACTCACAGTCTGGGGCATCAAGCAGCTCCAGGCAAGA---GTCCTGGCTGTGGAAAGATACCTAAGGGATCAACAGCTCCTAGGGATTTGGGGTTGCTCTGGAAAAATCATTTGCACCACTGCTGTGCCTTGGAATAATACTTGGAGT---------------------------AATAAAAATCTGAGCCAGATTTGGGAT---AACATGACCTGGATGGAGTGGGAGAAAGAAATTGAT------AATTACACAGAAATAATATATGACTTGCTTGAAAAATCGCAAAACCAACAAGAAAAGAATGAACAAGAGTTATTGGAATTGGATAAATGGGCAAGTTTGTGGAATTGGTTTAGCATAACAAACTGGCTGTGGTATATAAAAATATTCATAATGATAGTAGGAGGCTTGATAGGTTTAAGAATAGTTTTTACTGTACTTTCTATAGTGAATAGAGTTAGGCAGGGATACTCACCATTGTCATTGCAGACCCTC---CTCCCAGCTCCGAGGGGA------CCCGGCAGGCCCGAAGGAACAGAAGAAGAAGGTGGAGAGCGAGACAGAGGCAGATCCGATCCATTAGCGACAGGATTCTTAGCACTTTTCTGGGACGACCTGAGGACCCTGTGCCTCTTCAGCTACCACCGCTTGAGAGACTTACTCTTGATTGTGACGAGGATTGTGGAACTTCTGGGACGCAGG---------------GGGTGGGAACTGCTCAAGTATTGGTGG---AATCTCCTACAGTATTGG---------------------------------------------------AGTCAGGAACTAAAGAATAGTGCTGTTAACTTGTTTGATACTGTAGCCATAGCAGCAGCTGAGGGGACAGATAGGGTTATAGAAATAATACAAAGA------------------GCTGGTAGAGCTATCCTCCACATCCCTAGAAGAATAAGACAGGGCGCGGAAAGGGCTTTGATATAA

2.04013291.ADARC.GU330507 ATGAGAGTGAAGGAGATCAGGAAGAGTTATCAGCACTTG---------TGGAGATGGGGC------------------------ATCATGCTCCTTGGGATATTGATGATC------------TGTAGAGCTTCA---------GAAAATTTGTGGGTCACAGTCTATTATGGGGTACCTGTGTGGAAAGAAGCAACCACCACTCTGTTTTGTGCATCAGATGCTAAAGCTTATGAGATAGAGGTACATAAT---GTTTGGGCCACACATGCCTGCGTACCCACAGACCCCAACCCACAAGAAGTAGTATTG---GTAAATGTGACAGAAGATTTTAATATGTGGAAAAATAACATGGTAGACCAAATGCATGAGGATATAATCAGTTTATGGGATCAAAGCCTAAAACCATGTGTAAAACTAACCCCACTCTGTGTCACTCTAAATTGCACTGATTATGTGGGGAATGCCACCAATACCAACAAGACC---------------------------------------------------------------------------ACTACCCCTGCCCCTACTAATAGCTGGGACAAGGGAGAAATAAAAAACTGCTCTTTCAATATCACC---ACAAATATAAGAGAT------AAGAGGCAAAAAGAATATGCACTATTTTATAAACTAGATGTAGTACCAATAGAT------------------------------------AATACTAGT---------------------AATAGTTATAGGTTGATAAATTGTAACACCTCAGTCATTACACAGGCCTGTCCAAAGGTATCCTTTGAGCCAATTCCCATACATTATTGTGCCCCGGCTGGTTTTGCGATTTTAAAGTGT---AATGAAAAAGGGTTCATAGGAACAGGAACATGTAAAAATGTCAGCACAGTACAATGTACACATGGAATTAAGCCAGTAGTATCCACTCAACTGCTGTTGAATGGCAGTCTAGCAGAAGAA---GGGATAGTAATAAGATCTGAGAATTTCTCAGACAATGCTAAAACCATAATAGTACAGCTGAATGAATCTGTAGTTATTAATTGTACAAGACCCAACAACAATACAAGAAAAGGTATACATATAGGA------------CCAGGGGCG---GCATTTTATGCAACAGGAGATATAATAGGAGATATAAGACAAGCACATTGTAACCTT------AGTAGAGCACAATGGAATAAAACTTTAAAACAGGTAGCTATAAAATTAAAA---GAACAATTT---------------AGTAAAACAGTAATAGTCTTTAATAGA---------TCCTCAGGAGGGGACCCAGAAATTGTAATGCATAGTTTTAATTGTGGAGGGGAATTTTTCTACTGTAATACAACTAAGCTGTTTAATAATACTTGG---------------AATGGTACTAATAATAAT---------------------TGGAATGGTACTGAAATC------------------------------------------------------ATAACACTCCCATGCAGAATAAAACAAATTATAAACATGTGGCAAGAAGTAGGAAAAGCAATGTATGCCCCTCCCATCAGAGGACAGATTAACTGCTCGTCACATATTACAGGGCTGCTATTAACAAGAGATGGTGGC------------------AACAACACAGAC------------------AACAACAACACAGAGGTCTTCAGACCTGGAGGGGGAAATATGAAGGACAATTGGAGA---AGTGAATTATATAAATACAAAGTAGTAAAAATTGAACCA---TTAGGAGTAGCACCC---ACCAAGGCAAAGAGGAGAGTGGTGCAGAGAGAA---AAAAGAGCAGCA---------ATAGGA---GCTTTG---TTCCTT---GGG---------TTCTTGGGA---GCAGCAGGAAGCACTATGGGCGCAGCGTCACTG---ACGCTGACGGTACAGGCCAGACTATTATTGTCTGGTATAGTGCAACAGCAGAACAATTTGCTGAGGGCTATTGAGGCGCAACAGCATCTGTTGCAACTCACAGTCTGGGGCATCAAGCAGCTCCAGGCAAGA---GTCCTGGCTGTGGAAAGATACCTAAGGGATCAACAGCTCCTAGGGATTTGGGGTTGCTCTGGAAAAATCATTTGCACCACTGCTGTGCCTTGGAATAATACTTGGAGT---------------------------AATAAAAATCTGAGCCAGATTTGGGAT---AACATGACCTGGATGGAGTGGGAGAAAGAAATTGAT------AATTACACAGAAATAATATATGACTTGCTTGAAAAATCGCAAAACCAACAAGAAAAGAATGAACAAGAGTTATTGGAATTGGATAAATGGGCAAGTTTGTGGAATTGGTTTAGCATAACAAACTGGCTGTGGTATATAAAAATATTCATAATGATAGTAGGAGGCTTGATAGGTTTAAGAATAGTTTTTACTGTACTTTCTATAGTGAATAGAGTTAGGCAGGGATACTCACCATTGTCATTGCAGACCCTC---CTCCCAGCTCCGAGGGGA------CCCGGCAGGCCCGAAGGAACAGAAGAAGAAGGTGGAGAGCGAGACAGAGGCAGATCCGATCCATTAGCGACAGGATTCTTAGCACTTTTCTGGGACGACCTGAGGACCCTGTGCCTCTTCAGCTACCACCGCTTGAGAGACTTACTCTTGATTGTGACGAGGATTGTGGAACTTCTGGGACGCAGG---------------GGGTGGGAACTGCTCAAGTATTGGTGG---AATCTCCTACAGTATTGG---------------------------------------------------AGTCAGGAACTAAAGAATAGTGCTGTTAACTTGTTTGATACTGTAGCCATAGCAGCAGCTGAGGGGACAGATAGGGTTATAGAAATAATACAAAGA------------------GCTGGTAGAGCTATCCTCCACATCCCTAGAAGAATAAGACAGGGCGCGGAAAGGGCTTTGATATAA

2.04013291.ADARC.GU330508 ATGAGAGTGAAGGAGATCAGGAAGAGTTATCAGCACTTG---------TGGAGATGGGGC------------------------ATCATGCTCCTTGGGATATTGATGATC------------TGTAGAGCTTCA---------GAAAATTTGTGGGTCACAGTCTATTATGGGGTACCTGTGTGGAAAGAAGCAACCACCACTCTGTTTTGTGCATCAGATGCTAAAGCTTATGAGACAGAGGTACATAAT---GTTTGGGCCACACATGCCTGCGTACCCACAGACCCCAACCCACAAGAAGTAGTATTG---GTAAATGTGACAGAAGATTTTAATATGTGGAAAAATAACATGGTAGACCAAATGCATGAGGATATAATCAGTTTATGGGATCAAAGCCTAAAACCATGTGTAAAACTAACCCCACTCTGTGTCACTCTAAATTGCACTGATTATGTGGGGAATGCCACCAATACCAACAAGACC---------------------------------------------------------------------------ACTACCCCTGCCCCTACTAATAGCTGGGACAAGGGAGAAATAAAAAACTGCTCTTTCAATATCACC---ACAAATATAAGAGAT------AAGAGGCAAAAAGAATATGCACTATTTTATAAACTAGATGTAGTACCAATAGAT------------------------------------AATACTAGT---------------------AATAGTTATAGGTTGATAAATTGTAACACCTCAGTCATTACACAGGCCTGTCCAAAGGTATCCTTTGAGCCAATTCCCATACATTATTGTGCCCCGGCTGGTTTTGCGATTTTAAAGTGT---AATGAAAAAGGGTTCATAGGAACAGGAACATGTAAAAATGTCAGCACAGTACAATGTACACATGGAATTAAGCCAGTAGTATCCACTCAACTGCTGTTGAATGGCAGTCTAGCAGAAGAA---GGGATAGTAATAAGATCTGAGAATTTCTCAGACAATGCTAAAACCATAATAGTACAGCTGAATGAATCTGTAGTTATTAATTGTACAAGACCCAACAACAATACAAGAAAAGGTATACATATAGGA------------CCAGGGGCG---GCATTTTATGCAACAGGAGATATAATAGGAGATATAAGACAAGCACATTGTAACCTT------AGTAGAGCACAATGGAATAAAACTTTAAAACAGGTAGCTGTAAAATTAAAA---GAACAATTT---------------AAGAAAACAGTAATAGTCTTTAATAGA---------TCCTCAGGAGGGGACCCAGAAATTGTAATGCATAGTTTTAATTGTGGAGGGGAATTTTTCTACTGTAATACAACTAAGCTGTTTAATAATACTTGG---------------AATGGTACTAATAATAAT---------------------TGGAATGGTACTGAAATC------------------------------------------------------ATAACACTCCCATGCAGAATAAAACAAATTATAAACATGTGGCAAGAAGTAGGAAAAGCAATGTATGCCCCTCCCATCAGAGGACAGATTATCTGCTCGTCACATATTACAGGGCTGCTATTAACAAGAGATGGTGGC------------------AACAACACAGAC------------------AACAACAACACAGAGGTCTTCAGACCTGGAGGGGGAAATATGAAGGACAATTGGAGA---AGTGAATTATATAAATACAAAGTAGTAAAAATTGAACCA---TTAGGAGTAGCACCC---ACCAAGGCAAAGAGGAGAGTGGTGCAGAGAGAA---AAAAGAGCAGCA---------ATAGGA---GCTTTG---TTCCTT---GGG---------TTCTTGGGA---GCAGCAGGAAGCACTATGGGCGCAGCGTCACTG---ACGCTGACGGTACAGGCCAGACTATTATTGTCTGGTATAGTGCAACAGCAGAACAATTTGCTGAGGGCTATTGAGGCGCAACAGCATCTGTTGCAACTCACAGTCTGGGGCATCAAGCAGCTCCAGGCAAGA---GTCCTGGCTGTGGAAAGATACCTAAGGGATCAACAGCTCCTAGGGATTTGGGGTTGCTCTGGAAAAATCATTTGCACCACTGCTGTGCCTTGGAATAATACTTGGAGT---------------------------AATAAAAATCTGAGCCAGATTTGGGAT---AACATGACCTGGATGGAGTGGGAGAAAGAAATTGAT------AATTACACAGAAATAATATATGACTTGCTTGAAAAATCGCAAAACCAACAAGAAAAGAATGAACAAGAGTTATTGGAATTGGATAAATGGGCAAGTTTGTGGAATTGGTTTAGCATAACAAACTGGCTGTGGTATATAAAAATATTCATAATGATAGTAGGAGGCTTGATAGGTTTAAGAATAGTTTTTACTGTACTTTCTATAGTGAATAGAGTTAGGCAGGGATACTCACCATTGTCATTGCAGACCCTC---CTCCCAGCTCCGAGGGGA------CCCGGCAGGCCCGAAGGAACAGAAGAAGAAGGTGGAGAGCGAGACAGAGGCAGATCCGATCCATTAGCGACAGGATTCTTAGCACTTTTCTGGGACGACCTGAGGACCCTGTGCCTCTTCAGCTACCACCGCTTGAGAGACTTACTCTTGATTGTGACGAGGATTGTGGAACTTCTGGGACGCAGG---------------GGGTGGGAACTGCTCAAGTATTGGTGG---AATCTCCTACAGTATTGG---------------------------------------------------AGTCAGGAACTAAAGAATAGTGCTGTTAACTTGTTTGATACTGTAGCCATAGCAGCAGCTGAGGGGACAGATAGGGTTATAGAAATAATACAAAGA------------------GCTGGTAGAGCTATCCTCCACATCCCTAGAAGAATAAGACAGGGCGCGGAAAGGGCTTTGATATAA

2.04013291.ADARC.GU330509 ATGAGAGTGAAGGAGATCAGGAAGAGTTATCAGCACTTG---------TGGAGATGGGGC------------------------ATCATGCTCCTTGGGATATTGATGATC------------TGTAGAGCTTCA---------GAAAATTTGTGGGTCACAGTCTATTATGGGGTACCTGTGTGGAAAGAAGCAACCACCACTCTGTTTTGTGCATCAGATGCTAAAGCTTATGAGACAGAGGTACATAAT---GTTTGGGCCACACATGCCTGCGTACCCACAGACCCCAACCCACAAGAAGTAGTATTG---GTAAATGTGACAGAAGATTTTAATATGTGGAAAAATAACATGGTAGACCAAATGCATGAGGATATAATCAGTTTATGGGATCAAAGCCTAAAACCATGTGTAAAACTAACCCCACTCTGTGTCACTCTAAATTGCACTGATTATGTGGGGAATGCCACCAATACCAACAAGACC---------------------------------------------------------------------------ACTACCCCTGCCCCTACTAATAGCTGGGACAAGGGAGAAATAAAAAACTGCTCTTTCAATATCACC---ACAAATATAAGAGAT------AAGAGGCAAAAAGAATATGCACTATTTTATAAACTAGATGTAGTACCAATAGAT------------------------------------AATACTAGT---------------------AATAGTTATAGGTTGATAAATTGTAACACCTCAGTCATTACACAGGCCTGTCCAAAGGTATCCTTTGAGCCAATTCCCATACATTATTGTGCCCCGGCTGGTTTTGCGATTTTAAAGTGT---AATGAAAAAGGGTTCATAGGAACAGGAACATGTAAAAATGTCAGCACAGTACAATGTACACATGGAATTAAGCCAGTAGTATCCACTCAACTGCTGTTGAATGGCAGTCTAGCAGAAGAA---GGGATAGTAATAAGATCTGAGAATTTCTCAGACAATGCTAAAACCATAATAGTACAGCTGAATGAATCTGTAGTTATTAATTGTACAAGACCCAACAACAATACAAGAAAAGGTATACATATAGGA------------CCAGGGGCG---GCATTTTATGCAACAGGAGATATAATAGGAGATATAAGACAAGCACATTGTAACCTT------AGTAGAGCACAATGGAATAAAACTTTAAAACAGGTAGCTATAAAATTAAAA---GAACAACTT---------------AATAAAACAGTAATAGTCTTTAATAGA---------TCCTCAGGAGGGGACCCAGAAATTGTAATGCATAGTTTTAATTGTGGAGGGGAATTTTTCTACTGTAATACAACTAAGCTGTTTAATAATACTTGG---------------AATGGTACCAATAATAAT---------------------TGGAATGGTACTGAAATC------------------------------------------------------ATAACACTCCCATGCAGAATAAAACAAATTATAAACATGTGGCAAGAAGTAGGAAAAGCAATGTATGCCCCTCCCATCAGAGGACAGATTAACTGCTCGTCACATATTACAGGGCTGCTATTAACAAGAGATGGTGGC------------------AACAACACAGAC------------------AACAACAACACAGAGGTCTTCAGACCTGGAGGGGGAAATATGAAGGACAATTGGAGA---AGTGAATTATATAAATACAAAGTAGTAAAAATTGAACCA---TTAGGAGTAGCACCC---ACCAAAGCAAAGAGGAGAGTGGTGCAGAGAGAA---AAAAGAGCAGCA---------ATAGGA---GCTTTG---TTCCTT---GGG---------TTCTTGGGA---GCAGCAGGAAGCACTATGGGCGCAGCGTCACTG---ACGCTGACGGTACAGGCCAGACTATTATTGTCTGGTATAGTGCAACAGCAGAACAATTTGCTGAGGGCTATTGAGGCGCAACAGCATCTGTTGCAACTCACAGTCTGGGGCATCAAGCAGCTCCAGGCAAGA---GTCCTGGCTGTGGAAAGATACCTAAGGGATCAACAGCTCCTAGGGATTTGGGGTTGCTCTGGAAAAATCATTTGCACCACTGCTGTGCCTTGGAATAATACTTGGAGT---------------------------AATAAAAATCTGAGCCAGATTTGGGAT---AACATGACCTGGATGGAGTGGGAGAAAGAAATTGAT------AATTACACAGAAATAATATATGACTTGCTTGAAAAATCGCAAAACCAACAAGAAAAGAATGAACAAGAGTTATTGGAATTGGATAAATGGGCAAGTTTGTGGAATTGGTTTAGCATAACAAACTGGCTGTGGTATATAAAAATATTCATAATGATAGTAGGAGGCTTGATAGGTTTAAGAATAGTTTTTACTGTACTTTCTATAGTGAATAGAGTTAGGCAGGGATACTCACCATTGTCATTGCAGACCCTC---CTCCCAGCTCCGAGGGGA------CCCGGCAGGCCCGAAGGAACAGAAGAAGAAGGTGGAGAGCGAGACAGAGGCAGATCCGATCCATTAGCGACAGGATTCTTAGCACTTTTCTGGGACGACCTGAGGACCCTGTGCCTCTTCAGCTACCACCGCTTGAGAGACTTACTCTTGATTGTGACGAGGATTGTGGAACTTCTGGGACGCAGG---------------GGGTGGGAACTGCTCAAGTATTGGTGG---AATCTCCTACAGTATTGG---------------------------------------------------AGTCAGGAACTAAAGAATAGTGCTGTTAACTTGTTTGATACTGTAGCCATAGCAGCAGCTGAGGGGACAGATAGGGTTATAGAAATAATACAAAGA------------------GCTGGTAGAGCTATCCTCCACATCCCTAGAAGAATAAGACAGGGCGCGGAAAGGGCTTTGATATAA

2.04013291.ADARC.GU330510 ATGAGAGTGAAGGAGATCAGGAAGAGTTATCAGCACTTG---------TGGAGATGGGGC------------------------ATCATGCTCCTTGGGATATTGATGATC------------TGTAGAGCTTCA---------GAAAATTTGTGGGTCACAGTCTATTATGGGGTACCTGTGTGGAAAGAAGCAACCACCACTCTGTTTTGTGCATCAGATGCTAAAGCTTATGAGATAGAGGTACATAAT---GTTTGGGCCACACATGCCTGCGTACCCACAGACCCCAACCCACAAGAAGTAGTATTG---GTAAATGTGACAGAAGATTTTAATATGTGGAAAAATAACATGGTAGACCAAATGCATGAGGATATAATCAGTTTATGGGATCAAAGCCTAAAACCATGTGTAAAACTAACCCCACTCTGTGTCACTCTAAATTGCACTGATTATGTGGGGAATGCCACCAATACCAACAAGACC---------------------------------------------------------------------------ACTACCCCTGCCCCTACTAATAGCTGGGACAAGGGAGAAATAAAAAACTGCTCTTTCAATATCACC---ACAAATATAAGAGAT------AAGAGGCAAAAAGAATATGCACTATTTTATAAACTAGATGTAGTACCAATAGAT------------------------------------AATACTAGT---------------------AATAGTTATAGGTTGATAAATTGTAACACCTCAGTCATTACACAGGCCTGTCCAAAGGTATCCTTTGAGCCAATTCCCATACATTATTGTGCCCCGGCTGGTTTTGCGATTTTAAAGTGT---AATGAAAAAGGGTTCATAGGAACAGGAACATGTAAAAATGTCAGCACAGTACAATGTACACATGGAATTAAGCCAGTAGTATCCACTCAACTGCTGTTGAATGGCAGTCTAGCAGAAGAA---GGGATAGTAATAAGATCTGAGAATTTCTCAGACAATGCTAAAACCATAATAGTACAGCTGAATGAATCTGTAGTTATTAATTGTACAAGACCCAACAACAATACAAGAAAAGGTATACATATAGGA------------CCAGGGGCG---GCATTTTATGCAACAGGAGATATAATAGGAGATATAAGACAAGCACATTGTAACCTT------AGTAGAGCACAATGGAATAAAACTTTAAAACAGGTAGCTATAAAATTAAAA---GAACAATTT---------------AATAAAACAGTATTAGTCTTTAATAGA---------TCCTCAGGAGGGGACCCAGAAATTGTAATGCATAGTTTTAATTGTGGAGGGGAATTTTTCTACTGTAATACAACTAAGCTGTTTAATAATACTTGG---------------AATGGTACTAATAATAAT---------------------TGGAATGGTACTGAAATC------------------------------------------------------ATAACACTCCCATGCAGAATAAAACAAATTATAAACATGTGGCAAGAAGTAGGAAAAGCAATGTATGCCCCTCCCATCAGAGGACAGATTAACTGCTCGTCACATATTACAGGGCTGCTATTAACAAGAGATGGTGGC------------------AACAACACAGAC------------------AACAACAACACAGAGGTCTTCAGACCTGGAGGGGGAAATATGAAGGACAATTGGAGA---AGTGAATTATATAAATACAAAGTAGTAAAAATTGAACCA---TTAGGAGTAGCACCC---ACCAAGGCAAAGAGGAGAGTGGTGCAGAGAGAA---AAAAGAGCAGCA---------ATAGGA---GCTTTG---TTCCTT---GGG---------TTCTTGGGA---GCAGCAGGAAGCACTATGGGCGCAGCGTCACTG---ACGCTGACGGTACAGGCCAGACTATTATTGTCTGGTATAGTGCAACAGCAGAACAATTTGCTGAGGGCTATTGAGGCGCAACAGCATCTGTTACAACTCACAGTCTGGGGCATCAAGCAGCTCCAGGCAAGA---GTCCTGGCTGTGGAAAGATACCTAAGGGATCAACAGCTCCTAGGGATTTGGGGTTGCTCTGGAAAAATCATTTGCACCACTGCTGTGCCTTGGAATAATACTTGGAGT---------------------------AATAAAAATCTGAGCCAGATTTGGGAT---AACATGACCTGGATGGAGTGGGAGAAAGAAATTGAT------AATTACACAGAAATAATATATGACTTGCTTGAAAAATCGCAAAACCAACAAGAAAAGAATGAACAAGAGTTATTGGAATTGGATAAATGGGCAAGTTTGTGGAATTGGTTTAGCATAACAAACTGGCTGTGGTATATAAAAATATTCATAATGATAGTAGGAGGCTTGATAGGTTTAAGAATAGTTTTTACTGTACTTTCTATAGTGAATAGAGTTAGGCAGGGATACTCACCATTGTCATTGCAGACCCTC---CTCCCAGCTCCGAGGGGA------CCCGGCAGGCCCGAAGGAACAGAAGAAGAAGGTGGAGAGCGAGACAGAGGCAGATCCGATCCATTAGCGACAGGATTCTTAGCACTTTTCTGGGACGACCTGAGGACCCTGTGCCTCTTCAGCTACCACCGCTTGAGAGACTTACTCTTGATTGTGACGAGGATTGTGGAACTTCTGGGACGCAGG---------------GGGTGGGAACTGCTCAAGTATTGGTGG---AATCTCCTACAGTATTGG---------------------------------------------------AGTCAGGAACTAAAGAATAGTGCTGTTAACTTGTTTGATACTGTAGCCATAGCAGCAGCTGAGGGGACAGATAGGGTTATAGAAATAATACAAAGA------------------GCTGGTAGAGCTATCCTCCACATCCCTAGAAGAATAAGACAGGGCGCGGAAAGGGCTTTGATATAA

2.04013291.ADARC.GU330511 ATGAGAGTGAAGGAGATCAGGAAGAGTTATCAGCACTTG---------TGGAGATGGGGC------------------------ATCATGCTCCTTGGGATATTGATGATC------------TGTAGAGCTTCA---------GAAAATTTGTGGGTCACAGTCTATTATGGGGTACCTGTGTGGAAAGAAGCAACCACCACTCTGTTTTGTGCATCAGATGCTAAAGCTTATGAGATAGAGGTACATAAT---GTTTGGGCCACACATGCCTGCGTACCCACAGACCCCAACCCACAAGAAGTAGTATTG---GTAAATGTGACAGAAGATTTTAATATGTGGAAAAATAACATGGTAGACCAAATGCATGAGGATATAATCAGTTTATGGGATCAAAGCCTAAAACCATGTGTAAAACTAACCCCACTCTGTGTCACTCTAAATTGCACTGATTATGTGGGGAATGCCACCAATACCAACAAGACC---------------------------------------------------------------------------ACTACCCCTGCCCCTACTAATAGCTGGGACAAGGGAGAAATAAAAAACTGCTCTTTCAATATCACC---ACAAATATAAGAGAT------AAGAGGCAAAAAGAATATGCACTATTTTATAAACTAGATGTAGTACCAATAGAT------------------------------------AATACTAGT---------------------AATAGTTATAGGTTGATAAATTGTAACACCTCAGTCATTACACAGGCCTGTCCAAAGGTATCCTTTGAGCCAATTCCCATACATTATTGTGCCCCGGCTGGTTTTGCGATTTTAAAGTGT---AATGAAAAAGGGTTCATAGGAACAGGAACATGTAAAAATGTCAGCACAGTACAATGTACACATGGAATTAAGCCAGTAGTATCCACTCAACTGCTGTTGAATGGCAGTCTAGCAGAAGAA---GGGATAGTAATAAGATCTGAGAATTTCTCAGACAATGCTAAAACCATAATAGTACAGCTGAATGAATCTGTAGTTATTAATTGTACAAGACCCAACAACAATACAAGAAAAGGTATACATATAGGA------------CCAGGGGCG---GCATTTTATGCAACAGGAGATATAATAGGAGATATAAGACAAGCACATTGTAACCTT------AGTAGAGCACAATGAAATAAAACTTTAAAACAGGTAGCTATAAAATTAAAA---GAACAATTT---------------AGTAAAACAGTAATAGTCTTTAATAGA---------TCCTCAGGAGGGGACCCAGAAATTGTAATGCATAGTTTTAATTGTGGAGGGGAATTTTTCTACTGTAATACAACTAAGCTGTTTAATAATACTTGG---------------AATGGTACTAATAATAAT---------------------TGGAATGGTACTGAAATC------------------------------------------------------ATAACACTCCCATGCAGAATAAAACAAATTATAAACATGTGGCAAGAAGTAGGAAAAGCAATGTATGCCCCTCCCATCAGAGGACAGATTAACTGCTCGTCACATATTACAGGGCTGCTATTAACAAGAGATGGTGGC------------------AACAACACAGAC------------------AACAACAACACAGAGGTCTTCAGACCTGGAGGGGGAAATATGAAGGACAATTGGAGA---AGTGAATTATATAAATACAAAGTAGTAAAAATTGAACCA---TTAGGAGTAGCACCC---ACCAAGGCAAAGAGGAGAGTGGTGCAGAGAGAA---AAAAGAGCAGCA---------ATAGGA---GCTTTG---TTCCTT---GGG---------TTCTTGGGA---GCAGCAGGAAGCACTATGGGCGCAGCGTCACTG---ACGCTGACGGTACAGGCCAGACTATTATTGTCTGGTATAGTGCAACAGCAGAACAATTTGCTGAGGGCTATTGAGGCGCAACAGCATCTGTTGCAACTCACAGTCTGGGGCATCAAGCAGCTCCAGGCAAGA---GTCCTGGCTGTGGAAAGATACCTAAGGGATCAACAGCTCCTAGGGATTTGGGGTTGCTCTGGAAAAATCATTTGCACCACTGCTGTGCCTTGGAATAATACTTGGAGT---------------------------AATAAAAATCTGAGCCAGATTTGGGAT---AACATGACCTGGATGGAGTGGGAGAAAGAAATTGAT------AATTACACAGAAATAATATATGACTTGCTTGAAAAATCGCAAAACCAACAAGAAAAGAATGAACAAGAGTTATTGGAATTGGATAAATGGGCAAGTTTGTGGAATTGGTTTAGCATAACAAACTGGCTGTGGTATATAAAAATATTCATAATGATAGTAGGAGGCTTGATAGGTTTAAGAATAGTTTTTACTGTACTTTCTATAGTGAATAGAGTTAGGCAGGGATACTCACCATTGTCATTGCAGACCCTC---CTCCCAGCTCCGAGGGGA------CCCGGCAGGCCCGAAGGAACAGAAGAAGAAGGTGGAGAGCGAGACAGAGGCAGATCCGATCCATTAGCGACAGGATTCTTAGCACTTTTCTGGGACGACCTGAGGACCCTGTGCCTCTTCAGCTACCACCGCTTGAGAGACTTACTCTTGATTGTGACGAGGATTGTGGAACTTCTGGGACGCAGG---------------GGGTGGGAACTGCTCAAGTATTGGTGG---AATCTCCTACAGTATTGG---------------------------------------------------AGTCAGGAACTAAAGAATAGTGCTGTTAACTTGTTTGATACTGTAGCCATAGCAGCAGCTGAGGGGACAGATAGGGTTATAGAAATAATACAAAGA------------------GCTGGTAGAGCTATCCTCCACATCCCTAGAAGAATAAGACAGGGCGCGGAAAGGGCTTTGATATAA

2.04013291.ADARC.GU330512 ATGAGAGTGAAGGAGATCAGGAAGAGTTATCAGCACTTG---------TGGAGATGGGGC------------------------ATCATGCTCCTTGGGATATTGATGATC------------TGTAGAGCTTCA---------GAAAATTTGTGGGTCACAGTCTATTATGGGGTACCTGTGTGGAAAGAAGCAACCACCACTCTGTTTTGTGCATCAGATGCTAAAGCTTATGAGATAGAGGTACATAAT---GTTTGGGCCACACATGCCTGCGTACCCACAGACCCCAACCCACAAGAAGTAGTATTG---GTAAATGTGACAGAAGATTTTAATATGTGGAAAAATAACATGGTAGACCAAATGCATGAGGATATAATCAGTTTATGGGATCAAAGCCTAAAACCATGTGTAAAACTAACCCCACTCTGTGTCACTCTAAATTGCACTGATTATGTGGGGAATGCCACCAATACCAACAAGACC---------------------------------------------------------------------------ACTACCCCTGCCCCTACTAATAGCTGGGACAAGGGAGAAATAAAAAACTGCTCTTTCAATATCACC---ACAAATATAAGAGAT------AAGAGGCAAAAAGAATATGCACTATTTTATAAACTAGATGTAGTACCAATAGAT------------------------------------AATACTAGT---------------------AATAGTTATAGGTTGATAAATTGTAACACCTCAGTCATTACACAGGCCTGTCCAAAAGTATCCTTTGAGCCAATTCCCATACATTATTGTGCCCCGGCTGGTTTTGCGATTTTAAAGTGT---AATGAAAAAGGGTTCATAGGAACAGGAACATGTAAAAATGTCAGCACAGTACAATGTACACATGGAATTAAGCCAGTAGTATCCACTCAACTGCTGTTGAATGGCAGTCTAGCAGAAGAA---GGGATAGTAATAAGATCTGAGAATTTCTCAGACAATGCTAAAACCATAATAGTACAGCTGAATGAATCTGTAGTTATTAATTGTACAAGACCCAACAACAATACAAGAAAAGGTATACATATAGGA------------CCAGGGGCG---GCATTTTATGCAACAGGAGATATAATAGGAGATATAAGACAAGCACATTGTAACCTT------AGTAGAGCACAATGGAATAAAACTTTAAAACAGGTAGCTATAAAATTAAAA---GAACAATTT---------------AATAAAACAGTAATAGTCTTTAATAGA---------TCCTCAGGAGGGGACCCAGAAATTGTAATGCATAGTTTTAATTGTGGAGGGGAATTTTTCTACTGTAATACAACTAAGCTGTTTAATAATACTTGG---------------AATGGTACTAATAATAAT---------------------TGGAATGGTACTGAAATC------------------------------------------------------ATAACACTCCCATGCAGAATAAAACAAATTATAAACATGTGGCAAGAAGTAGGAAAAGCAATGTATGCCCCTCCCATCAGAGGACAGATTAACTGCTCGTCACATATTACAGGGCTGCTATTAACAAGAGATGGTGGC------------------AACAACACAGAC------------------AACAACAACACAGAGGTCTTCAGACCTGGAGGGGGAAATATGAAGGACAATTGGAGA---AGTGAATTATATAAATACAAAGTAGTAAAAATTGAACCA---TTAGGAGTAGCACCC---ACCAAGGCAAAGAGGAGAGTGGTGCAGAGAGAA---AAAAGAGCAGCA---------ATAGGA---GCTTTG---TTCCTT---GGG---------TTCTTGGGA---GCAGCAGGAAGCACTATGGGCGCAGCGTCACTG---ACGCTGACGGTACAGGCCAGACTATTATTGTCTGGTATAGTGCAACAGCAGAACAATTTGCTGAGGGCTATTGAGGCGCAACAGCATCTGTTGCAACTCACAGTCTGGGGCATCAAGCAGCTCCAGGCAAGA---GTCCTGGCTGTGGAAAGATACCTAAGGGATCAACAGCTCCTAGGGATTTGGGGTTGCTCTGGAAAAATCATTTGCACCACTGCTGTGCCTTGGAATAATACTTGGAGT---------------------------AATAAAAATCTGAGCCAGATTTGGGAT---AACATGACCTGGATGGAGTGGGAGAAAGAAATTGAT------AATTACACAGAAATAATATATGACTTGCTTGAAAAATCGCAAAACCAACAAGAAAAGAATGAACAAGAGTTATTGGAATTGGATAAATGGGCAAGTTTGTGGAATTGGTTTAGCATAACAAACTGGCTGTGGTATATAAAAATATTCATAATGATAGTAGGAGGCTTGATAGGTTTAAGAATAGTTTTTACTGTACTTTCTATAGTGAATAGAGTTAGGCAGGGATACTCACCATTGTCATTGCAGACCCTC---CTCCCAGCTCCGAGGGGA------CCCGGCAGGCCCGAAGGAACAGAAGAAGAAGGTGGAGAGCGAGACAGAGGCAGATCCGATCCATTAGCGACAGGATTCTTAGCACTTTTCTGGGACGACCTGAGGACCCTGTGCCTCTTCAGCTACCACCGCTTGAGAGACTTACTCTTGATTGTGACGAGGATTGTGGAACTTCTGGGACGCAGG---------------GGGTGGGAACTGCTCAAGTATTGGTGG---AATCTCCTACAGTATTGG---------------------------------------------------AGTCAGGAACTAAAGAATAGTGCTGTTAACTTGTTTGATACTGTAGCCATAGCAGCAGCTGAGGGGACAGATAGGGTTATAGAAATAATACAAAGA------------------GCTGGTAGAGCTATCCTCCACATCCCTAGAAGAATAAGACAGGGCGCGGAAAGGGCTTTGATATAA

2.04013291.ADARC.GU330513 ATGAGAGTGAAGGAGATCAGGAAGAGTTATCAGCACTTG---------TGGAGATGGGGC------------------------ATCATGCTCCTTGGGATATTGATGATC------------TGTAGAGCTTCA---------GAAAATTTGTGGGTCACAGTCTATTATGGGGTACCTGTGTGGAAAGAAGCAACCACCACTCTGTTTTGTGCATCAGATGCTAAAGCTTATGAGATAGAGGTACATAAT---GTTTGGGCCACACATGCCTGCGTACCCACAGACCCCAACCCACAAGAAGTAGTATTG---GTAAATGTGACAGAAGATTTTAATATGTGGAAAAATAACATGGTAGACCAAATGCATGAGGATATAATCAGTTTATGGGATCAAAGCCTAAAACCATGTGTAAAACTAACCCCACTCTGTGTCACTCTAAATTGCACTGATTATGTGGGGAATGCCACCAATACCAACAAGACC---------------------------------------------------------------------------ACTACCCCTGCCCCTACTAATAGCTGGGACAAGGGAGAAATAAAAAACTGCTCTTTCAATATCACC---ACAAATATAAGAGAT------AAGAGGCAAAAAGAATATGCACTATTTTATAAACTAGATGTAGTACCAATAGAT------------------------------------AATACTAGT---------------------AATAGTTATAGGTTGATAAATTGTAACACCTCAGTCATTACACAGGCCTGTCCAAAGGTATCCTTTGAGCCAATTCCCATACATTATTGTGCCCCGGCTGGTTTTGCGATTTTAAAGTGT---AATGAAAAAGGGTTCATAGGAACAGGAACATGTAAAAATGTCAGCACAGTACAATGTACACATGGAATTAAGCCAGTAGTATCCACTCAACTGCTGTTGAATGGCAGTCTAGCAGAAGAA---GGGATAGTAATAAGATCTGAGAATTTCTCAGACAATGCTAAAACCATAATAGTACAGCTGAATGAATCTGTAGTTATTAATTGTACAAGACCCAACAACAATACAAGAAAAGGTATACATATAGGA------------CCAGGGGCG---GCATTTTATGCAACAGGAGATATAATAGGAGATATAAGACAAGCACATTGTAACCTT------AGTAGAGCACAATGGAATAAAACTTTAAAACAGGTAGCTATAAAATTAAAA---GAACAATTT---------------AAGAAAACAGTAATAGTCTTTAATAGA---------TCCTCAGGAGGGGACCCAGAAATTGTAATGCATAGTTTTAATTGTGGAGGGGAATTTTTCTACTGTAATACAACTAAGCTGTTTAATAATACTTGG---------------AATGGTACTAATAATAAT---------------------TGGAATGGTACTGAAATC------------------------------------------------------ATAACACTCCCATGCAGAATAAAACAAATTATAAACATGTGGCAAGAAGTAGGAAAAGCAATGTATGCCCCTCCCATCAGAGGACAGATTAACTGCTCGTCACATATTACAGGGCTGCTATTAACAAGAGATGGTGGC------------------AACAACACAGAC------------------AACAACAACACAGAGGTCTTCAGACCTGGAGGGGGAAATATGAAGGACAATTGGAGA---AGTGAATTATATAAATACAAAGTAGTAAAAATTGAACCA---TTAGGAGTAGCACCC---ACCAAGGCAAAGAGGAGAGTGGTGCAGAGAGAA---AAAAGAGCAGCA---------ATAGGA---GCTTTG---TTCCTT---GGG---------TTCTTGGGA---GCAGCAGGAAGCACTATGGGCGCAGCGTCACTG---ACGCTGACGGTACAGGCCAGACTATTATTGTCTGGTATAGTGCAACAGCAGAACAATTTGCTGAGGGCTATTGAGGCGCAACAGCATCTGTTGCAACTCACAGTCTGGGGCATCAAGCAGCTCCAGGCAAGA---GTCCTGGCTGTGGAAAGATACCTAAGGGATCAACAGCTCCTAGGGATTTGGGGTTGCTCTGGAAAAATCATTTGCACCACTGCTGTGCCTTGGAATAATACTTGGAGT---------------------------AATAAAAATCTGAGCCAGATTTGGGAT---AACATGACCTGGATGGAGTGGGAGAAAGAAATTGAT------AATTACACAGAAATAATATATGACTTGCTTGAAAAATCGCAAAACCAACAAGAAAAGAATGAACAAGAGTTATTGGAATTGGATAAATGGGCAAGTTTGTGGAATTGGTTTAGCATAACAAACTGGCTGTGGTATATAAAAATATTCATAATGATAGTAGGAGGCTTGATAGGTTTAAGAATAGTTTTTACTGTACTTTCTATAGTGAATAGAGTTAGGCAGGGATACTCACCATTGTCATTGCAGACCCTC---CTCCCAGCTCCGAGGGGA------CCCGGCAGGCCCGAAGGAACAGAAGAAGAAGGTGGAGAGCGAGACAGAGGCAGATCCGATCCATTAGCGACAGGATTCTTAGCACTTTTCTGGGACGACCTGAGGACCCTGTGCCTCTTCAGCTACCACCGCTTGAGAGACTTACTCTTGATTGTGACGAGGATTGTGGAACTTCTGGGACGCAGG---------------GGGTGGGAACTGCTCAAGTATTGGTGG---AATCTCCTACAGTATTGG---------------------------------------------------AGTCAGGAACTAAAGAATAGTGCTGTTAACTTGTTTGATACTGTAGCCATAGCAGCAGCTGAGGGGACAGATAGGGTTATAGAAATAATACAAAGA------------------GCTGGTAGAGCTATCCTCCACATCCCTAGAAGAATAAGACAGGGCGCGGAAAGGGCTTTGATATAA

2.04013291.ADARC.GU330514 ATGAGAGTGAAGGAGATCAGGAAGAGTTATCAGCACTTG---------TGGAGATGGGGC------------------------ATCATGCTCCTTGGGATATTGATGATC------------TGTAGAGCTTCA---------GAAAATTTGTGGGTCACAGTCTATTATGGGGTACCTGTGTGGAAAGAAGCAACCACCACTCTGTTTTGTGCATCAGATGCTAAAGCTTATGAGACAGAGGTACATAAT---GTTTGGGCCACACATGCCTGCGTACCCACAGACCCCAACCCACAAGAAGTAGTATTG---GTAAATGTGACAGAAGATTTTAATATGTGGAAAAATAACATGGTAGACCAAATGCATGAGGATATAATCAGTTTATGGGATCAAAGCCTAAAACCATGTGTAAAACTAACCCCACTCTGTGTCACTCTAAATTGCACTGATTATGTGGGGAATGCCACCAATACCCACAAGACC---------------------------------------------------------------------------ACTACCCCTGCCCCTACTAATAGCTGGGACAAGGGAGAAATAAAAAACTGCTCTTTCAATATCACC---ACAAATATAAGAGAT------AAGAGGCAAAAAGAATATGCACTATTTTATAAACTAGATGTAGTACCAATAGAT------------------------------------AATACTAGT---------------------AATAGTTATAGGTTGATAAATTGTAACACCTCAGTCATTACACAGGCCTGTCCAAAGGTATCCTTTGAGCCAATTCCCATACATTATTGTGCCCCGGCTGGTTTTGCGATTTTAAAGTGT---AATGAAAAAGGGTTCATAGGAACAGGAACATGTAAAAATGTCAGCACAGTACAATGTACACATGGAATTAAGCCAGTAGTATCCACTCAACTGCTGTTGAATGGCAGTCTAGCAGAAGAA---GGGATAGTAATAAGATCTGAGAATTTCTCAGACAATGCTAAAACCATAATAGTACAGCTGAATGAATCTGTAGTTATTAATTGTACAAGACCCAACAACAATACAAGAAAAGGTATACATATAGGA------------CCAGGGGCG---GCATTTTATGCAACAGGAGATATAATAGGAGATATAAGACAAGCACATTGTAACCTT------AGTAGAGCACAATGGAATAAAACTTTAAAACAGGTAGCTATAAAATTAAAA---GAACAACTT---------------AATAAAACAGTAATAGTCTTTAATAGA---------TCCTCAGGAGGGGACCCAGAAATTGTAATGCATAGTTTTAATTGTGGAGGGGAATTTTTCTACTGTAATACAACTAAGCTGTTTAATAATACTTGG---------------AATGGTACTAATAATAAT---------------------TGGAATGGTACTGAAATC------------------------------------------------------ATAACACTCCCATGCAGAATAAAACAAATTATAAACATGTGGCAAGAAGTAGGAAAAGCAATGTATGCCCCTCCCATCAGAGGACAGATTAACTGCTCGTCACATATTACAGGGCTGCTATTAACAAGAGATGGTGGC------------------AACAACACAGAC------------------AACAACAACACAGAGGTCTTCAGACCTGGAGGGGGAAATATGAAGGACAATTGGAGA---AGTGAATTATATAAATACAAAGTAGTAAAAATTGAACCA---TTAGGAGTAGCACCC---ACCAAGGCAAAGAGGAGAGTGGTGCAGAGAGAA---AAAAGAGCAGCA---------ATAGGA---GCTTTG---TTCCTT---GGG---------TTCTTGGGA---GCAGCAGGAAGCACTATGGGCGCAGCGTCACTG---ACGCTGACGGTACAGGCCAGACTATTATTGTCTGGTATAGTGCAACAGCAGAACAATTTGCTGAGGGCTATTGAGGCGCAACAGCATCTGTTGCAACTCACAGTCTGGGGCATCAAGCAGCTCCAGGCAAGA---GTCCTGGCTGTGGAAAGATACCTAAGGGATCAACAGCTCCTAGGGATTTGGGGTTGCTCTGGAAAAATCATTTGCACCACTGCTGTGCCTTGGAATAATACTTGGAGT---------------------------AATAAAAATCTGAGCCAGATTTGGGAT---AACATGACCTGGATGGAGTGGGAGAAAGAAATTGAT------AATTACACAGAAATAATATATGACTTGCTTGAAAAATCGCAAAACCAACAAGAAAAGAATGAACAAGAGTTATTGGAATTGGATAAATGGGCAAGTTTGTGGAATTGGTTTAGCATAACAAACTGGCTGTGGTATATAAAAATATTCATAATGATAGTAGGAGGCTTGATAGGTTTAAGAATAGTTTTTACTGTACTTTCTATAGTGAATAGAGTTAGGCAGGGATACTCACCATTGTCATTGCAGACCCTC---CTCCCAGCTCCGAGGGGA------CCCGGCAGGCCCGAAGGAACAGAAGAAGAAGGTGGAGAGCGAGACAGAGGCAGATCCGATCCATTAGCGACAGGATTCTTAGCACTTTTCTGGGACGACCTGAGGACCCTGTGCCTCTTCAGCTACCACCGCTTGAGAGACTTACTCTTGATTGTGACGAGGATTGTGGAACTTCTGGGACGCAGG---------------GGGTGGGAACTGCTCAAGTATTGGTGG---AATCTCCTACAGTATTGG---------------------------------------------------AGTCAGGAACTAAAGAATAGTGCTGTTAACTTGTTTGATACTGTAGCCATAGCAGCAGCTGAGGGGACAGATAGGGTTATAGAAATAATACAAAGA------------------GCTGGTAGAGCTATCCTCCACATCCCTAGAAGAATAAGACAGGGCGCGGAAAGGGCTTTGATATAA

2.04013291.ADARC.GU330515 ATGAGAGTGAAGGAGATCAGGAAGAGTTATCAGCACTTG---------TGGAGATGGGGC------------------------ATCATGCTCCTTGGGATATTGATGATC------------TGTAGAGCTTCA---------GAAAATTTGTGGGTCACAGTCTATTATGGGGTACCTGTGTGGAAAGAAGCAACCACCACTCTGTTTTGTGCATCAGATGCTAAAGCTTATGAGACAGAGGTACATAAT---GTTTGGGCCACACATGCCTGCGTACCCACAGACCCCAACCCACAAGAAGTAGTATTG---GTAAATGTGACAGAAGATTTTAATATGTGGAAAAATAACATGGTAGACCAAATGCATGAGGATATAATCAGTTTATGGGATCAAAGCCTAAAACCATGTGTAAAACTAACCCCACTCTGTGTCACTCTAAATTGCACTGATTATGTGGGGAATGCCACCAATACCAACAAGACC---------------------------------------------------------------------------ACTACCCCTGCCCCTACTAATAGCTGGGACAAGGGAGAAATAAAAAACTGCTCTTTCAATATCACC---ACAAATATAAGAGAT------AAGAGGCAAAAAGAATATGCACTATTTTATAAACTAGATGTAGTACCAATAGAT------------------------------------AATACTAGT---------------------AATAGTTATAGGTTGATAAATTGTAACACCTCAGTCATTACACAGGCCTGTCCAAAGGTATCCTTTGAGCCAATTCCCATACATTATTGTGCCCCGGCTGGTTTTGCGATTTTAAAGTGT---AATGAAAAAGGGTTCATAGGAACAGGAACATGTAAAAATGTCAGCACAGTACAATGTACACATGGAATTAAGCCAGTAGTATCCACTCAACTGCTGTTGAATGGCAGTCTAGCAGAAGAA---GGGATAGTAATAAGATCTGAGAATTTCTCAGACAATGCTAAAACCATAATAGTACAGCTGAATGAATCTGTAGTTATTAATTGTACAAGACCCAACAACAATACAAGAAAAGGTATACATATAGGA------------CCAGGGGCG---GCATTTTATGCAACAGGAGATATAATAGGAGATATAAGACAAGCACATTGTAACCTT------AGTAGAGCACAATGGAATAAAACTTTAAAACAGGTAGCTATAAAATTAAAA---GAACAATTT---------------AATAAAACAGTAATAGTCTTTAATAGA---------TCCTCAGGAGGGGACCCAGAAATTGTAATGCATAGTTTTAATTGTGGAGGGGAATTTTTCTACTGTAATACAACTAAGCTGTTTAATAATACTTGG---------------AATGGTACTAATAATAAT---------------------TGGAATGGTACTGAAATC------------------------------------------------------ATAACACTCCCATGCAGAATAAAACAAATTATAAACATGTGGCAAGAAGTAGGAAAAGCAATGTATGCCCCTCCCATCAGAGGACAGATTAACTGCTCGTCACATATTACGGGGCTGCTATTAACAAGAGATGGTGGC------------------AACAACACAGAC------------------AACAACAACACAGAGGTCTTCAGACCTGGAGGGGGAAATATGAAGGACAATTGGAGA---AGTGAATTATATAAATACAAAGTAGTAAAAATTGAACCA---TTAGGAGTAGCACCC---ACCAAGGCAAAGAGGAGAGTGGTGCAGAGAGAA---AAAAGAGCAGCA---------ATAGGA---GCTTTG---TTCCTT---GGG---------TTCTTGGGA---GCAGCAGGAAGCACTATGGGCGCAGCGTCACTG---ACGCTGACGGTACAGGCCAGACTATTATTGTCTGGTATAGTGCAACAGCAGAACAATTTGCTGAGAGCTATTGAGGCGCAACAGCATCTGTTGCAACTCACAGTCTGGGGCATCAAGCAGCTCCAGGCAAGA---GTCCTGGCTGTGGAAAGATACCTAAGGGATCAACAGCTCCTAGGGATTTGGGGTTGCTCTGGAAAAATCATTTGCACCACTGCTGTGCCTTGGAATAATACTTGGAGT---------------------------AATAAAAATCTGAGCCAGATTTGGGAT---AACATGACCTGGATGGAGTGGGAGAAAGAAATTGAT------AATTACACAGAAATAATATATGACTTGCTTGAAAAATCGCAAAACCAACAAGAAAAGAATGAACAAGAGTTATTGGAATTGGATAAATGGGCAAGTTTGTGGAATTGGTTTAGCATAACAAACTGGCTGTGGTATATAAAAATATTCATAATGATAGTAGGAGGCTTGATAGGTTTAAGAATAGTTTTTACTGTACTTTCTATAGTGAATAGAGTTAGGCAGGGATACTCACCATTGTCATTGCAGACCCTC---CTCCCAGCTCCGAGGGGA------CCCGGCAGGCCCGAAGGAACAGAAGAAGAAGGTGGAGAGCGAGACAGAGGCAGATCCGATCCATTAGCGACAGGATTCTTAGCACTTTTCTGGGACGACCTGAGGACCCTGTGCCTCTTCAGCTACCACCGCTTGAGAGACTTACTCTTGATTGTGACGAGGATTGTGGAACTTCTGGGACGCAGG---------------GGGTGGGAACTGCTCAAGTATTGGTGG---AATCTCCTACAGTATTGG---------------------------------------------------AGTCAGGAACTAAAGAATAGTGCTGTTAACTTGTTTGATACTGTAGCCATAGCAGCAGCTGAGGGGACAGATAGGGTTATAGAAATAATACAAAGA------------------GCTGGTAGAGCTATCCTCCACATCCCTAGAAGAATAAGACAGGGCGCGGAAAGGGCTTTGATATAA

2.04013291.ADARC.GU330516 ATGAGAGTGAAGGAGATCAGGAAGAGTTATCAGCACTTG---------TGGAGATGGGGC------------------------ATCATGCTCCTTGGGATATTGATGATC------------TGTAGAGCTTCA---------GAAAATTTGTGGGTCACAGTCTATTATGGGGTACCTGTGTGGAAAGAAGCAACCACCACTCTGTTTTGTGCATCAGATGCTAAAGCTTATGAGACAGAGGTACATAAT---GTTTGGGCCACACATGCCTGCGTACCCACAGACCCCAACCCACAAGAAGTAGTATTG---GTAAATGTGACAGAAGATTTTAATATGTGGAAAAATAACATGGTAGACCAAATGCATGAGGATATAATCAGTTTATGGGATCAAAGCCTAAAACCATGTGTAAAACTAACCCCACTCTGTGTCACTCTAAATTGCACTGATTATGTGGGGAATGCCACCAATACCAACAAGACC---------------------------------------------------------------------------ACTACCCCTGCCCCTACTAATAGCTGGGACAAGGGAGAAATAAAAAACTGCTCTTTCAATATCACC---ACAAATATAAGAGAT------AAGAGGCAAAAAGAATATGCACTATTTTATAAACTAGATGTAGTACCAATAGAT------------------------------------AATACTAGT---------------------AATAGTTATAGGTTGATAAATTGTAACACCTCAGTCATTACACAGGCCTGTCCAAAGGTATCCTTTGAGCCAATTCCCATACATTATTGTGCCCCGGCTGGTTTTGCGATTTTAAAGTGT---AATGAAAAAGGGTTCATAGGAACAGGAACATGTAAAAATGTCAGCACAGTACAATGTACACATGGAATTAAGCCAGTAGTATCCACTCAACTGCTGTTGAATGGCAGTCTAGCAGAAGAA---GGGATAGTAATAAGATCTGAGAATTTCTCAGACAATGCTAAAACCATAATAGTACAGCTGAATGAATCTGTAGTTATTAATTGTACAAGACCCAACAACAATACAAGAAAAGGTATACATATAGGA------------CCAGGGGCG---GCATTTTATGCAACAGGAGATATAATAGGAGATATAAGACAAGCACATTGTAACCTT------AGTAGAGCACAATGGAATAAAACTTTAAAACAGGTAGCTATAAAATTAAAA---GAACAAATT---------------AATAAAACAGTAATAGTCTTTAATAGA---------TCCTCAGGAGGGGACCCAGAAATTGTAATGCATAGTTTTAATTGTGGAGGGGAATTTTTCTACTGTAATACAACTAAGCTGTTTAATAATACTTGG---------------AATGGTACTAATAATAAT---------------------TGGAATGGTACTGAAATC------------------------------------------------------ATAACACTCCCATGCAGAATAAAACAAATTATAAACATGTGGCAAGAAGTAGGAAAAGCAATGTATGCCCCTCCCATCAGAGGACAGATTAACTGCTCGTCACATATTACAGGGCTGCTATTAACAAGAGATGGTGGC------------------AACAACACAGAC------------------AACAACAACACAGAGGTCTTCAGACCTGGAGGGGGAAATATGAAGGACAATTGGAGA---AGTGAATTATATAAATACAAAGTAGTAAAAATTGAACCA---TTAGGAGTAGCACCC---ACCAAGGCAAAGAGGAGAGTGGTGCAGAGAGAA---AAAAGAGCAGCA---------ATAGGA---GCTTTG---TTCCTT---GGG---------TTCTTGGGA---GCAGCAGGAAGCACTATGGGCGCAGCGTCACTG---ACGCTGACGGTACAGGCCAGACTGTTATTGTCTGGTATAGTGCAACAGCAGAACAATTTGCTGAGGGCTATTGAGGCGCAACAGCATCTGTTGCAACTCACAGTCTGGGGCATCAAGCAGCTCCAGGCAAGA---GTCCTGGCTGTGGAAAGATACCTAAGGGATCAACAGCTCCTAGGGATTTGGGGTTGCTCTGGAAAAATCATTTGCACCACTGCTGTGCCTTGGAATAATACTTGGAGT---------------------------AATAAAAATCTGAGCCAGATTTGGGAT---AACATGACCTGGATGGAGTGGGAGAAAGAAATTGAT------AATTACACAGAAATAATATATGACTTGCTTGAAAAATCGCAAAACCAACAAGAAAAGAATGAACAAGAGTTATTGGAATTGGATAAATGGGCAAGTTTGTGGAATTGGTTTAGCATAACAAACTGGCTGTGGTATATAAAAATATTCATAATGATAGTAGGAGGCTTGATAGGTTTAAGAATAGTTTTTACTGTACTTTCTATAGTGAATAGAGTTAGGCAGGGATACTCACCATTGTCATTGCAGACCCTC---CTCCCAGCTCCGAGGGGA------CCCGGCGGGCCCGAAGGAACAGAAGAAGAAGGTGGAGAGCGAGACAGAGGCAGATCCGATCCATTAGCGACAGGATTCTTAGCACTTTTCTGGGACGACCTGAGGACCCTGTGCCTCTTCAGCTACCACCGCTTGAGAGACTTACTCTTGATTGTGACGAGGATTGTGGAACTTCTGGGACGCAGG---------------GGGTGGGAACTGCTCAAGTATTGGTGG---AATCTCCTACAGTATTGG---------------------------------------------------AGTCAGGAACTAAAGAATAGTGCTGTTAACTTGTTTGATACTGTAGCCATAGCAGCAGCTGAGGGGACAGATAGGGTTATAGAAATAATACAAAGA------------------GCTGGTAGAGCTATCCTCCACATCCCTAGAAGAATAAGACAGGGCGCGGAAAGGGCTTTGATATAA

2.04013291.ADARC.GU330517 ATGAGAGTGAAGGAGATCAGGAAGAGTTATCAGCACTTG---------TGGAGATGGGGC------------------------ATCATGCTCCTTGGGATATTGATGATC------------TGTAGAGCTTCA---------GAAAATTTGTGGGTCACAGTCTATTATGGGGTACCTGTGTGGAAAGAAGCAACCACCACTCTGTTTTGTGCATCAGATGCTAAAGCTTATGAGACAGAGGTACATAAT---GTTTGGGCCACACATGCCTGCGTACCCACAGACCCCAACCCACAAGAAGTAGTATTG---GTAAATGTGACAGAAGATTTTAATATGTGGAAAAATAACATGGTAGACCAAATGCATGAGGATATAATCAGTTTATGGGATCAAAGCCTAAAACCATGTGTAAAACTAACCCCACTCTGTGTCACTCTAAATTGCACTGATTATGTGGGGAATGCCACCAATACCAACAAGACC---------------------------------------------------------------------------ACTACCCCTGCCCCTACTAATAGCTGGGACAAGGGAGAAATAAAAAACTGCTCTTTCAATATCACC---ACAAATATAAGAGAT------AAGAGGCAAAAAGAATATGCACTATTTTATAAACTAGATGTAGTACCAATAGAT------------------------------------AATACTAGT---------------------AATAGTTATAGGTTGATAAATTGTAACACCTCAGTCATTACACAGGCCTGTCCAAAGGTATCCTTTGAGCCAATTCCCATACATTATTGTGCCCCGGCTGGTTTTGCGATTTTAAAGTGT---AATGAAAAAGGGTTCATAGGAACAGGAACATGTAAAAATGTCAGCACAGTACAATGTACACATGGAATTAAGCCAGTAGTATCCACTCAACTGCTGTTGAATGGCAGTCTAGCAGAAGAA---GGGATAGTAATAAGATCTGAGAATTTCTCAGACAATGCTAAAACCATAATAGTACAGCTGAATGAATCTGTAGTTATTAATTGTACAAGACCCAACAACAATACAAGAAAAGGTATACATATAGGA------------CCAGGGGCG---GCATTTTATGCAACAGGAGATATAATAGGAGATATAAGACAAGCACATTGTAACCTT------AGTAGAGCACAATGGAATAAAACTTTAAAACAGGTAGCTATAAAATTAAAA---GAACAATTT---------------AATAAAAAAGTAATAGTCTTTAATAGA---------TCCTCAGGAGGGGACCCAGAAATTGTAATGCATAGTTTTAATTGTGGAGGGGAATTTTTCTACTGTAATACAACTAAGCTGTTTAATAATACTTGG---------------AATGGTACTAATAATAAT---------------------TGGAATGGTACTGAAATC------------------------------------------------------ATAACACTCCCATGCAGAATAAAACAAATTATAAACATGTGGCAAGAAGTAGGAAAAGCAATGTATGCCCCTCCCATCAGAGGACAGATTAACTGCTCGTCACATATTACAGGGCTGCTATTAACAAGAGATGGTGGC------------------AACAACACAGAC------------------AACAACAACACAGAGGTCTTCAGACCTGGAGGGGGAAATATGAAGGACAATTGGAGA---AGTGAATTATATAAATACAAAGTAGTAAAAATTGAACCA---TTAGGAGTAGCACCC---ACCAAGGCAAAGAGGAGAGTGGTGCAGAGAGA----AAAAGAGCAGCA---------ATAGGA---GCTTTG---TTCCTT---GGG---------TTCTTGGGA---GCAGCAGGAAGCACTATGGGCGCAGCGTCACTG---ACGCTGACGGTACAGGCCAGACTATTATTGTCTGGTATAGTGCAACAGCAGAACAATTTGCTGAGGGCTATTGAGGCGCAACAGCATCTGTTGCAACTCACAGTCTGGGGCATCAAGCAGCTCCAGGCAAGA---GTCCTGGCTGTGGAAAGATACCTAAGGGATCAACAGCTCCTAGGGATTTGGGGTTGCTCTGGAAAAATCATTTGCACCACTGCTGTGCCTTGGAATAATACTTGGAAT---------------------------AATAAAAATCTGAGCCAGATTTGGGAT---AACATGACCTGGATGGAGTGGGAGAAAGAAATTGAT------AATTACACAGAAATAATATATGACTTGCTTGAAAAATCGCAAAACCAACAAGAAAAGAATGAACAAGAGTTATTGGAATTGGATAAATGGGCAAGTTTGTGGAATTGGTTTAGCATAACAAACTGGCTGTGGTATATAAAAATATTCATAATGATAGTAGGAGGCTTGATAGGTTTAAGAATAGTTTTTACTGTACTTTCTATAGTGAATAGAGTTAGGCAGGGATACTCACCATTGTCATTGCAGACCCTC---CTCCCAGCTCCGAGGGGA------CCCGGCAGGCCCGAAGGAACAGAAGAAGAAGGTGGAGAGCGAGACAGAGGCAGATCCGATCCATTAGCGACAGGATTCTTAGCACTTTTCTGGGACGACCTGAGGACCCTGTGCCTCTTCAGCTACCACCGCTTGAGAGACTTACTCTTGATTGTGACGAGGATTGTGGAACTTCTGGGACGCAGG---------------GGGTGGGAACTGCTCAAGTATTGGTGG---AATCTCCTACAGTATTGG---------------------------------------------------AGTCAGGAACTAAAGAATAGTGCTGTTAACTTGTTTGATACTGTAGCCATAGCAGCAGCTGAGGGGACAGATAGGGTTATAGAAATAATACAAAGA------------------GCTGGTAGAGCTATCCTCCACATCCCTAGAAGAATAAGACAGGGCGCGGAAAGGGCTTTGATATAA

2.04013291.ADARC.GU330518 ATGAGAGTGAAGGAGATCAGGAAGAGTTATCAGCACTTG---------TGGAGATGGGGC------------------------ATCATGCTCCTTGGGATATTGATGATC------------TGTAGAGCTTCA---------GAAAATTTGTGGGTCACAGTCTATTATGGGGTACCTGTGTGGAAAGAAGCAACCACCACTCTGTTTTGTGCATCAGATGCTAAAGCTTATGAGATAGAGGTACATAAT---GTTTGGGCCACACATGCCTGCGTACCCACAGACCCCAACCCACAAGAAGTAGTATTG---GTAAATGTGACAGAAGATTTTAATATGTGGAAAAATAACATGGTAGACCAAATGCATGAGGATATAATCAGTTTATGGGATCAAAGCCTAAAACCATGTGTAAAACTAACCCCACTCTGTGTCACTCTAAATTGCACTGATTATGTGGGGAATGCCACCAATACCAACAAGACC---------------------------------------------------------------------------ACTACCCCTGCCCCTACTAATAGCTGGGACAAGGGAGAAATAAAAAACTGCTCTTTCAATATCACC---ACAAATATAAGAGAT------AAGAGGCAAAAAGAATATGCACTATTTTATAAACTAGATGTAGTACCAATAGAT------------------------------------AATACTAGT---------------------AATAGTTATAGGTTGATAAATTGTAACACCTCAGTCATTACACAGGCCTGTCCAAAGGTATCCTTTGAGCCAATTCCCATACATTATTGTGCCCCGGCTGGTTTTGCGATTTTAAAGTGT---AATGAAAAAGGGTTCATAGGAACAGGAACATGTAAAAATGTCAGCACAGTACAATGTACACATGGAATTAAGCCAGTAGTATCCACTCAACTGCTGTTGAATGGCAGTCTAGCAGAAGAA---GGGATAGTAATAAGATCTGAGAATTTCTCAGACAATGCTAAAACCATAATAGTACAGCTGAATGAATCTGTAGTTATTAATTGTACAAGACCCAACAACAATACAAGAAAAGGTATACATATAGGA------------CCAGGGGCG---GCATTTTATGCAACAGGAGATATAATAGGAGATATAAGACAAGCACATTGTAACCTT------AGTAGAGCACAATGGAATAAAACTTTAAAACAGGTAGCTATAAAATTAAAA---GAACAATCT---------------AATAAAACAGTAATAGTCTTTAATAGA---------TCCTCAGGAGGGGACCCAGAAATTGTAATGCATAGTTTTAATTGTGGAGGGGAATTTTTCTACTGTAATACAACTAAGCTGTTTAATAATACTTGG---------------AATGGTACTAACAATAAT---------------------TGGAATGGTACTGAAATC------------------------------------------------------ATAACACTCCCATGCAGAATAAAACAAATTATAAACATGTGGCAAGAAGTAGGAAAAGCAATGTATGCCCCTCCCATCAGAGGACAGATTAACTGCTCGTCACATATTACAGGGCTGCTATTAACAAGAGATGGTGGC------------------AACAACACAGAC------------------AACAACAACACAGAGGTCTTCAGACCTGGAGGGGGAAATATGAAGGACAATTGGAGA---AGTGAATTATATAAATACAAAGTAGTAAAAATTGAACCA---TTAGGAGTAGCACCC---ACCAAGGCAAAGAGGAGAGTGGTGCAGAGAGAA---AAAAGAGCAGCA---------ATAGGA---GCTTTG---TTCCTT---GGG---------TTCTTGGGA---GCAGCAGGAAGCACTATGGGCGCAGCGTCACTG---ACGCTGACGGTACAGGCCAGACTATTATTGTCTGGTATAGTGCAACAGCAGAACAATTTGCTGAGGGCTATTGAGGCGCAACAGCATCTGTTGCAACTCACAGTCTGGGGCATCAAGCAGCTCCAGGCAAGA---GTCCTGGCTGTGGAAAGATACCTAAGGGATCAACAGCTCCTAGGGATTTGGGGTTGCTCTGGAAAAATCATTTGCACCACTGCTGTGCCTTGGAATAATACTTGGAGT---------------------------AATAAAAATCTGAGCCAGATTTGGGAT---AACATGACCTGGATGGAGTGGGAGAAAGAAATTGAT------AATTACACAGAAATAATATATGACTTGCTTGAAAAATCGCAAAACCAACAAGAAAAGAATGAACAAGAGTTATTGGAATTGGATAAATGGGCAGGTTTGTGGAATTGGTTTAGCATAACAAACTGGCTGTGGTATATAAAAATATTCATAATGATAGTAGGAGGCTTGATAGGTTTAAGAATAGTTTTTACTGTACTTTCTATAGTGAATAGAGTTAGGCAGGGATACTCACCATTGTCATTGCAGACCCTC---CTCCCAGCTCCGAGGGGA------CCCGGCAGGCCCGAAGGAACAGAAGAAGAAGGTGGAGAGCGAGACAGAGGCAGATCCGATCCATTAGCGACAGGATTCTTAGCACTTTTCTGGGACGACCTGAGGACCCTGTGCCTCTTCAGCTACCACCGCTTGAGAGACTTACTCTTGATTGTGACGAGGATTGTGGAACTTCTGGGACGCAGG---------------GGGTGGGAACTGCTCAAGTATTGGTGG---AATCTCCTACAGTATTGG---------------------------------------------------AGTCAGGAACTAAAGAATAGTGCTGTTAACTTGTTTGATACTGTAGCCATAGCAGCAGCTGAGGGGACAGATAGGGTTATAGAAATAATACAAAGA------------------GCTGGTAGAGCTATCCTCCACATCCCTAGAAGAATAAGACAGGGCGCGGAAAGGGCTTTGATATAA

2.04013291.ADARC.GU330519 ATGAGAGTGAAGGAGATCAGGAAGAGTTATCAGCACTTG---------TGGAAATGGGGC------------------------ATCATGCTCCTTGGGATATTGATGATC------------TGTAGAGCTTCA---------GAAAATTTGTGGGTCACAGTCTATTATGGGGTACCTGTGTGGAAAGAAGCAACCACCACTCTGTTTTGTGCATCAGATGCTAAAGCTTATGAGACAGAGGTACATAAT---GTTTGGGCCACACATGCCTGCGTACCCACAGACCCCAACCCACAAGAAGTAGTATTG---GTAAATGTGACAGAAGATTTTAATATGTGGAAAAATAACATGGTAGACCAAATGCATGAGGATATAATCAGTTTATGGGATCAAAGCCTAAAACCATGTGTAAAACTAACCCCACTCTGTGTCACTCTAAATTGCACTGATTATGTGGGGAATGCCACCAATACCAACAAGACC---------------------------------------------------------------------------ACTACCCCTGCCCCTACTAATAGCTGGGACAAGGGAGAAATAAAAAACTGCTCTTTCAATATCACC---ACAAATATAAGAGAT------AAGAGGCAAAAAGAATATGCACTATTTTATAAACTAGATGTAGTACCAATAGAT------------------------------------AATACTAGT---------------------AATAGTTATAGGTTGATAAATTGTAACACCTCAGTCATTACACAGGCCTGTCCAAAGGTATCCTTTGAGCCAATTCCCATACATTATTGTGCCCCGGCTGGTTTTGCGATTTTAAAGTGT---AATGAAAAAGGGTTCATAGGAACAGGAACATGTAAAAATGTCAGCACAGTACAATGTACACATGGAATTAAGCCAGTAGTATCCACTCAACTGCTGTTGAATGGCAGTCTAGCAGAAGAA---GGGATAGTAATAAGATCTGAGAATTTCTCAGACAATGCTAAAACCATAATAGTACAGCTGAATGAATCTGTAGTTATTAATTGTACAAGACCCAACAACAATACAAGAAAAGGTATACATATAGGA------------CCAGGGGCG---GCATTTTATGCAACAGGAGATATAATAGGAG--ATAAGACAAGCACATTGTAACCTT------AGTAGAGCACAATGGAATAAAACTTTAAAACAGGTAGCTATAAAATTAAAA---GAACAATTT---------------AATAAAGCAGTAATAGTCTTTAATAGA---------TCCTCAGGAGGGGACCCAGAAATTGTAATGCATAGTTTTAATTGTGGAGGGGAATTTTTCTACTGTAATACAACTAAGCTGTTTAATAATACTTGG---------------AATGGTACTAATAATAAT---------------------TGGAATGGTACTGAAATC------------------------------------------------------ATAACACTCCCATGCAGAATAAAACAAATTATAAACATGTGGCAAGAAGTAGGAAAAGCAATGTATGCCCCTCCCATCAGAGGACAGATTAACTGCTCGTCACATATTACAGGGCTGCTATTAACAAGAGATGGTGGC------------------AACAACACAGAC------------------AACAACAACACAGAGGTCTTCAGACCTGGAGGGGGAAATATGAAGGACAATTGGAGA---AGTGAATTATATAAATACAAAGTAGTAAAAATTGAACCA---TTAGGAGTAGCACCC---ACCAAGGCAAAGAGGAGAGTGGTGCAGAGAGAA---AAAAGAGCAGCA---------ATAGGA---GCTTTG---TTCCTT---GGG---------TTCTTGGGA---GCAGCAGGAAGCACTATGGGCGCAGCGTCACTG---ACGCTGACGGTACAGGCCAGACTATTATTGTCTGGTATAGTGCAACAGCAGAACAATTTGCTGAGGGCTATTGAGGCGCAACAGCATCTGTTGCAACTCACAGTCTGGGGCATCAAGCAGCTCCAGGCAAGA---GTCCTGGCTGTGGAAAGATACCTAAGGGATCAACAGCTCCTAGGGATTTGGGGTTGCTCTGGAAAAATCATTTGCACCACTGCTGTGCCTTGGAATAATACTTGGAGT---------------------------AATAAAAATCTGAGCCAGATTTGGGAT---AACATGACCTGGATGGAGTGGGAGAAAGAAATTGAT------AATTACACAGAAATAATATATGACTTGCTTGAAAAATCGCAAAACCAACAAGAAAAGAATGAACAAGAGTTATTGGAATTGGATAAATGGGCAAGTTTGTGGAATTGGTTTAGCATAACAAACTGGCTGTGGTATATAAAAATATTCATAATGATAGTAGGAGGCTTGATAGGTTTAAGAATAGTTTTTACTGTACTTTCTATAGTGAATAGAGTTAGGCAGGGATACTCACCATTGTCATTGCAGACCCTC---CTCCCAGCTCCGAGGGGA------CCCGGCAGGCCCGAAGGAACAGAAGAAGAAGGTGGAGAGCGAGACAGAGGCAGATCCGATCCATTAGCGACAGGATTCTTAGCACTTTTCTGGGACGACCTGAGGACCCTGTGCCTCTTCAGCTACCACCGCTTGAGAGACTTACTCTTGATTGTGACGAGGATTGTGGAACTTCTGGGACGCAGG---------------GGGTGGGAACTGCTCAAGTATTGGTGG---AATCTCCTACAGTATTGG---------------------------------------------------AGTCAGGAACTAAAGAATAGTGCTGTTAACTTGTTTGATACTGTAGCCATAGCAGCAGCTGAGGGGACAGATAGGGTTATAGAAATAATACAAAGA------------------GCTGGTAGAGCTATCCTCCACATCCCTAGAAGAATAAGACAGGGCGCGGAAAGGGCTTTGATATAA

2.04013291.ADARC.GU330520 ATGAGAGTGAAGGAGATCAGGAAGAGTTATCAGCACTTG---------TGGAGATGGGGC------------------------ATCATGCTCCTTGGGATATTGATGATC------------TGTAGAGCTTCA---------GAAAATTTGTGGGTCACAGTCTATTATGGGGTACCTGTGTGGAAAGAAGCAACCACCACTCTGTTTTGTGCATCAGATGCTAAAGCTTATGAGACAGAGGTACATAAT---GTTTGGGCCACACATGCCTGCGTACCCACAGACCCCAACCCACAAGAAGTAGTATTG---GTAAATGTGACAGAAGATTTTAATATGTGGAAAAATAACATGGTAGACCAAATGCATGAGGATATAATCAGTTTATGGGATCAAAGCCTAAAACCATGTGTAAAACTAACCCCACTCTGTGTCACTCTAAATTGCACTGATTATGTGGGGAATGCCACCAATACCAACAAGACC---------------------------------------------------------------------------ACTACCCCTGCCCCTACTAATAGCTGGGACAAGGGAGAAATAAAAAACTGCTCTTTCAATATCACC---ACAAATATAAGAGAT------AAGAGGCAAAAAGAATATGCACTATTTTATAAACTAGATGTAGTACCAATAGAT------------------------------------AATACTAGT---------------------AATAGTTATAGGTTGATAAATTGTAACACCTCAGTCATTACACAGGCCTGTCCAAAGGTATCCTTTGAGCCAATTCCCATACATTATTGTGCCCCGGCTGGTTTTGCGATTTTAAAGTGT---AATGAAAAAGGGTTCATAGGAACAGGAACATGTAAAAATGTCAGCACAGTACAATGTACACATGGAATTAAGCCAGTAGTATCCACTCAACTGCTGTTGAATGGCAGTCTAGCAGAAGAA---GGGATAGTAATAAGATCTGAGAATTTCTCAGACAATGCTAAAACCATAATAGTACAGCTGAATGAATCTGTAGTTATTAATTGTACAAGACCCAACAACAATACAAGAAAAGGTATACATATAGGA------------CCAGGGGCG---GCATTTTATGCAACAGGAGATATAATAGGAGATATAAGACAAGCACATTGTAACCTT------AGTAGAGCACAATGGAATAAAACTTTAAAACAGGTAGCTATAAAATTAAAA---GAACAACTT---------------AATAAAACAGTAATAGTCTTTAATAGA---------TCCTCAGGAGGGGACCCAGAAATTGTAATGCATAGTTTTAATTGTGGAGGGGAATTTTTCTACTGTAATACAACTAAGCTGTTTAATAATACTTGG---------------AATGGTACTAATAATAAT---------------------TGGAATGGTACTGAAATC------------------------------------------------------ATAACACTCCCATGCAGAATAAAACAAATTATAAACATGTGGCAAGAAGTAGGAAAAGCAATGTATGCCCCTCCCATCAGAGGACAGATTAACTGCTCGTCACATATTACAGGGCTGCTATTAACAAGAGATGGTGGC------------------AACAACACAGAC------------------AACAACAACACAGAGGTCTTCAGACCTGGAGGGGGAAATATGAAGGACAATTGGAGA---AGTGAATTATATAAATACAAAGTAGTAAAAATTGAACCA---TTAGGAGTAGCACCC---ACCAAGGCAAAGAGGAGAGTGGTGCAGAGAGAA---AAAAGAGCAGCA---------ATAGGA---GCTTTG---TTCCTT---GGG---------TTCTTGGGA---GCAGCAGGAAGCACTATGGGCGCAGCGTCACTG---ACGCTGACGGTACAGGCCAGACTATTATTGTCTGGTATAGTGCAACAGCAGAACAATTTGCTGAGGGCTATTGAGGCGCAACAGCATCTGTTGCAACTCACAGTCTGGGGCATCAAGCAGCTCCAGGCAAGA---GTCCTGGCTGTGGAAAGATACCTAAGGGATCAACAGCTCCTAGGGATTTGGGGTTGCTCTGGAAAAATCATTTGCACCACTGCTGTGCCTTGGAATAATACTTGGAGT---------------------------AATAAAAATCTGAGCCAGATTTGGGAT---AACATGACCTGGATGGAGTGGGAGAAAGAAATTGAT------AATTACACAGAAATAATATATGACTTGCTTGAAAAATCGCAAAACCAACAAGAAAAGAATGAACAAGAGTTATTGGAATTGGATAAATGGGCAAGTTTGTGGAATTGGTTTAGCATAACAAACTGGCTGTGGTATATAAAAATATTCATAATGATAGTAGGAGGCTTGATAGGTTTAAGAATAGTTTTTACTGTACTTTCTATAGTGAATAGAGTTAGGCAGGGATACTCACCATTGTCATTGCAGACCCTC---CTCCCAGCTCCGAGGGGA------CCCGGCAGGCCCGAAGGAACAGAAGAAGAAGGTGGAGAGCGAGACAGAGGCAGATCCGATCCATTAGCGACAGGATTCTTAGCACTTTTCTGGGACGACCTGAGGACCCTGTGCCTCTTCAGCTACCACCGCTTGAGAGACTTACTCTTGATTGTGACGAGGATTGTGGAACTTCTGGGACGCAGG---------------GGGTGGGAACTGCTCAAGTATTGGTGG---AATCTCCTACAGTATTGG---------------------------------------------------AGTCAGGAACTAAAGAATAGTGCTGTTAACTTGTTTGATACTGTAGCCATAGCAGCAGCTGAGGGGACAGATAGGGTTATAGAAATAATACAAAGA------------------GCTGGTAGAGCTATCCTCCACATCCCTAGAAGAATAAGACAGGGCGCGGAAAGGGCTTTGATATAA

2.04013291.ADARC.GU330521 ATGAGAGTGAAGGAGATCAGGAAGAGTTATCAGCACTTG---------TGGAGATGGGGC------------------------ATCATGCTCCTTGGGATATTGATGATC------------TGTAGAGCTTCA---------GAAAATTTGTGGGTCACAGTCTATTATGGGGTACCTGTGTGGAAAGAAGCAACCACCACTCTGTTTTGTGCATCAGATGCTAAAGCTTATGAGATAGAGGTACATAAT---GTTTGGGCCACACATGCCTGCGTACCCACAGACCCCAACCCACAAGAAGTAGTATTG---GTAAATGTGACAGAAGATTTTAATATGTGGAAAAATAACATGGTAGACCAAATGCATGAGGATATAATCAGTTTATGGGATCAAAGCCTAAAACCATGTGTAAAACTAACCCCACTCTGTGTCACTCTAAATTGCACTGATTATGTGGGGAATGCCACCAATACCAACAAGACC---------------------------------------------------------------------------ACTACCCCTGCCCCTACTAATAGCTGGGACAAGGGAGAAATAAAAAACTGCTCTTTCAATATCACC---ACAAATATAAGAGAT------AAGAGGCAAAAAGAATATGCACTATTTTATAAACTAGATGTAGTACCAATAGAT------------------------------------AATACTAGT---------------------AATAGTTATAGGTTGATAAATTGTAACACCTCAGTCATTACACAGGCCTGTCCAAAGGTATCCTTTGAGCCAATTCCCATACATTATTGTGCCCCGGCTGGTTTTGCGATTTTAAAGTGT---AATGAAAAAGGGTTCATAGGAACAGGAACATGTAAAAATGTCAGCACAGTACAATGTACACATGGAATTAAGCCAGTAGTATCCACTCAACTGCTGTTGAATGGCAGTCTAGCAGAAGAA---GGGATAGTAATAAGATCTGAGAATTTCTCAGACAATGCTAAAACCATAATAGTACAGCTGAATGAATCTGTAGTTATTAATTGTACAAGACCCAACAACAATACAAGAAAAGGTATACATATAGGA------------CCAGGGGCG---GCATTTTATGCAACAGGAGATATAATAGGAGATATAAGACAAGCACATTGTAACCTT------AGTAGAGCACAATGGAATAAAACTTTAAAACAGGTAGCTATAAAATTAAAA---GAACAACTT---------------AATAAAACAGTAATAGTCTTTAATAGA---------TCCTCAGGAGGGGACCCAGAAATTGTAATGCATAGTTTTAATTGTGGAGGGGAATTTTTCTACTGTAATACAACTAAGCTGTTTAATAATACTTGG---------------AATGGTACTAATAATAAT---------------------TGGAATGGTACTGAAATC------------------------------------------------------ATAACACTCCCATGCAGAATAAAACAAATTATAAACATGTGGCAAGAAGTAGGAAAAGCAATGTATGCCCCTCCCATCAGAGGACAGATTAACTGCTCGTCACATATTACAGGGCTGCTATTAACAAGAGATGGTGGC------------------AACAACACAGAC------------------AACAACAACACAGAGGTCTTCAGACCTGGAGGGGGAAATATGAAGGACAATTGGAGA---AGTGAATTATATAAATACAAAGTAGTAAAAATTGAACCA---TTAGGAGTAGCACCC---ACCAAGGCAAAGAGGAGAGTGGTGCAGAGAGAA---AAAAGAGCAGCA---------ATAGGA---GCTTTG---TTCCTT---GGG---------TTCTTGGGA---GCAGCAGGAAGCACTATGGGCGCAGCGTCACTG---ACGCTGACGGTACAGGCCAGACTATTATTGTCTGGTATAGTGCAACAGCAGAACAATTTGCTGAGGGCTATTGAGGCGCAACAGCATCTGTTGCAACTCACAGTCTGGGGCATCAAGCAGCTCCAGGCAAGA---GTCCTGGCTGTGGAAAGATACCTAAGGGATCAACAGCTCCTAGGGATTTGGGGTTGCTCTGGAAAAATCATTTGCACCACTGCTGTGCCTTGGAATAATACTTGGAGT---------------------------AATAAAAATCTGAGCCAGATTTGGGAT---AACATGACCTGGATGGAGTGGGAGAAAGAAATTGAT------AATTACACAGAAATAATATATGACTTGCTTGAAAAATCGCAAAACCAACAAGAAAAGAATGAACAAGAGTTATTGGAATTGGATAAATGGGCAAGTTTGTGGAATTGGTTTAGCATAACAAACTGGCTGTGGTATATAAAAATATTCATAATGATAGTAGGAGGCTTGATAGGTTTAAGAATAGTTTTTACTGTACTTTCTATAGTGAATAGAGTTAGGCAGGGATACTCACCATTGTCATTGCAGACCCTC---CTCCCAGCTCCGAGGGGA------CCCGGCAGGCCCGAAGGAACAGAAGAAGAAGGTGGAGAGCGAGACAGAGGCAGATCCGATCCATTAGCGACAGGATTCTTAGCACTTTTCTGGGACGACCTGAGGACCCTGTGCCTCTTCAGCTACCACCGCTTGAGAGACTTACTCTTGATTGTGACGAGGATTGTGGAACTTCTGGGACGCAGG---------------GGGTGGGAACTGCTCAAGTATTGGTGG---AATCTCCTACAGTATTGG---------------------------------------------------AGTCAGGAACTAAAGAATAGTGCTGTTAACTTGTTTGATACTGTAGCCATAGCAGCAGCTGAGGGGACAGATAGGGTTATAGAAATAATACAAAGA------------------GCTGGTAGAGCTATCCTCCACATCCCTAGAAGAATAAGACAGGGCGCGGAAAGGGCTTTGATATAA

2.04013291.ADARC.GU330522 ATGAGAGTGAAGGAGATCAGGAAGAGTTATCAGCACTTG---------TGGAGATGGGGC------------------------ATCATGCTCCTTGGGATATTGATGATC------------TGTAGAGCTTCA---------GAAAATTTGTGGGTCACAGTCTATTATGGGGTACCTGTGTGGAAAGAAGCAACCACCACTCTGTTTTGTGCATCAGATGCTAAAGCTTATGAGACAGAGGTACATAAT---GTTTGGGCCACACATGCCTGCGTACCCACAGACCCCAACCCACAAGAAGTAGTATTG---GTAAATGTGACAGAAGATTTTAATATGTGGAAAAATAACATGGTAGACCAAATGCATGAGGATATAATCAGTTTATGGGATCAAAGCCTAAAACCATGTGTAAAACTAACCCCACTCTGTGTCACTCTAAATTGCACTGATTATGTGGGGAATGCCACCAATACCAACAAGACC---------------------------------------------------------------------------ACTACCCCTGCCCCTACTAATAGCTGGGACAAGGGAGAAATAAAAAACTGCTCTTTCAATATCACC---ACAAATATAAGAGAT------AAGAGGCAAAAAGAATATGCACTATTTTATAAACTAGATGTAGTACCAATAGAT------------------------------------AATACTAGT---------------------AATAGTTATAGGTTGATAAATTGTAACACCTCAGTCATTACACAGGCCTGTCCAAAGGTATCCTTTGAGCCAATTCCCATACATTATTGTGCCCCGGCTGGTTTTGCGATTTTAAAGTGT---AATGAAAAAGGGTTCATAGGAACAGGAACATGTAAAAATGTCAGCACAGTACAATGTACACATGGAATTAAGCCAGTAGTATCCACTCAACTGCTGTTGAATGGCAGTCTAGCAGAAGAA---GGGATAGTAATAAGATCTGAGAATTTCTCAGACAATGCTAAAACCATAATAGTACAGCTGAATGAATCTGTAGTTATTAATTGTACAAGACCCAACAACAATACAAGAAAAGGTATACATATAGGA------------CCAGGGGCG---GCATTTTATGCAACAGGAGATATAATAGGAGATATAAGACAAGCACATTGTAACCTT------AGTAGAGCACAATGGAATAAAACTTTAAAACAGGTAGCTATAAAATTAAAA---GAACAATTT---------------AATAAAACAGTACTAGTCTTTAATAGA---------TCCTCAGGAGGGGACCCAGAAATTGTAATGCATAGTTTTAATTGTGGAGGGGAATTTTTCTACTGTAATACAACTAAGCTGTTTAATAATACTTGG---------------AATGGTACTAATAATAAT---------------------TGGAATGGTACTGAAATC------------------------------------------------------ATAACACTCCCATGCAGAATAAAACAAATTATAAACATGTGGCAAGAAGTAGGAAAAGCAATGTATGCCCCTCCCATCAGAGGACAGATTAACTGCTCGTCACATATTACAGGGCTGCTATTAACAAGAGATGGTGGC------------------AACAACACAGAC------------------AACAACAACACAGAGGTCTTCAGACCTGGAGGGGGAAATATGAAGGACAATTGGAGA---AGTGAATTATATAAATACAAAGTAGTAAAAATTGAACCA---TTAGGAGTAGCACCC---ACCAAGGCAAAGAGGAGAGTGGTGCAGAGAGAA---AAAAGAGCAGCA---------ATAGGA---GCTTTG---TTCCTT---GGG---------TTCTTGGGA---GCAGCAGGAAGCACTATGGGCGCAGCGTCACTG---ACGCTGACGGTACAGGCCAGACTATTATTGTCTGGTATAGTGCAACAGCAGAACAATTTGCTGAGGGCTATTGAGGCGCAACAGCATCTGTTGCAACTCACAGTCTGGGGCATCAAGCAGCTCCAGGCAAGA---GTCCTGGCTGTGGAAAGATACCTAAGGGATCAACAGCTCCTAGGGATTTGGGGTTGCTCTGGAAAAATCATTTGCACCACTGCTGTGCCTTGGAATAATACTTGGAGT---------------------------AATAAAAATCTGAGCCAGATTTGGGAT---AACATGACCTGGATGGAGTGGGAGAAAGAAATTGAT------AATTACACAGAAATAATATATGACTTGCTTGAAAAATCGCAAAACCAACAAGAAAAGAATGAACAAGAGTTATTGGAATTGGATAAATGGGCAAGTTTGTGGAATTGGTTTAGCATAACAAACTGGCTGTGGTATATAAAAATATTCATAATGATAGTAGGAGGCTTGATAGGTTTAAGAATAGTTTTTACTGTACTTTCTATAGTGAATAGAGTTAGGCAGGGATACTCACCATTGTCATTGCAGACCCTC---CTCCCAGCTCCGAGGGGA------CCCGGCAGGCCCGAAGGAACAGAAGAAGAAGGTGGAGAGCGAGACAGAGGCAGATCCGATCCATTAGCGACAGGATTCTTAGCACTTTTCTGGGACGACCTGAGGACCCTGTGCCTCTTCAGCTACCACCGCTTGAGAGACTTACTCTTGATTGTGACGAGGATTGTGGAACTTCTGGGACGCAGG---------------GGGTGGGAACTGCTCAAGTATTGGTGG---AATCTCCTACAGTATTGG---------------------------------------------------AGTCAGGAACTAAAGAATAGTGCTGTTAACTTGTTTGATACTGTCGCCATAGCAGCAGCTGAGGGGACAGATAGGGTTATAGAAATAATACAAAGA------------------GCTGGTAGAGCTATCCTCCACATCCCTAGAAGAATAAGACAGGGCGCGGAAAGGGCTTTGATATAA

2.04013291.ADARC.GU330523 ATGAGAGTGAAGGAGATCAGGAAGAGTTATCAGCACTTG---------TGGAGATGGGGC------------------------ATCATGCTCCTTGGGATATTGATGATC------------TGTAGAGCTTCA---------GAAAATTTGTGGGTCACAGTCTATTATGGGGTACCTGTGTGGAAAGAAGCAACCACCACTCTGTTTTGTGCATCAGATGCTAAAGCTTATGAGACAGAGGTACATAAT---GTTTGGGCCACACATGCCTGCGTACCCACAGACCCCAACCCACAAGAAGTAGTATTG---GTAAATGTGACAGAAGATTTTAATATGTGGAAAAATAACATGGTAGACCAAATGCATGAGGATATAATCAGTTTATGGGATCAAAGCCTAAAACCATGTGTAAAACTAACCCCACTCTGTGTCACTCTAAATTGCACTGATTATGTGGGGAATGCCACCAATACCAACAAGACC---------------------------------------------------------------------------ACTACCCCTGCCCCTACTAATAGCTGGGACAAGGGAGAAATAAAAAACTGCTCTTTCAATATCACC---ACAAATATAAGAGAT------AAGAGGCAAAAAGAATATGCACTATTTTATAAACTAGATGTAGTACCAATAGAT------------------------------------AATACTAGT---------------------AATAGTTATAGGTTGATAAATTGTAACACCTCAGTCATTACACAGGCCTGTCCAAAGGTATCCTTTGAGCCAATTCCCATACATTATTGTGCCCCGGCTGGTTTTGCGATTTTAAAGTGT---AATGAAAAAGGGTTCATAGGAACAGGAACATGTAAAAATGTCAGCACAGTACAATGTACACATGGAATTAAGCCAGTAGTATCCACTCAACTGCTGTTGAATGGCAGTCTAGCAGAAGAA---GGGATAGTAATAAGATCTGAGAATTTCTCAGACAATGCTAAAACCATAATAGTACAGCTGAATGAATCTGTAGTTATTAATTGTACAAGACCCAACAACAATACAAGAAAAGGTATACATATAGGA------------CCAGGGGCG---GCATTTTATGCAACAGGAGATATAATAGGAGATATAAGACAAGCACATTGTAACCTT------AGTAGAGCACAATGGAATAAAACTTTAAAACAGGTAGCTATAAAATTAAAA---GAACAATCT---------------AATAAAACAGTAATAGTCTTTAATAGA---------TCCTCAGGAGGGGACCCAGAAATTGTAATGCATAGTTTTAATTGTGGAGGGGAATTTTTCTACTGTAATACAACTAAGCTGTTTAATAATACTTGG---------------AATGGTACTAATAATAAT---------------------TGGAATGGTACTGAAATC------------------------------------------------------ATAACACTCCCATGCAGAATAAAACAAATTATAAACATGTGGCAAGAAGTAGGAAAAGCAATGTATGCCCCTCCCATCAGAGGACAGATTAACTGCTCGTCACATATTACGGGGCTGCTATTAACAAGAGATGGTGGC------------------AACAACACAGAC------------------AACAACAACACAGAGGTCTTCAGACCTGGAGGGGGAAATATGAAGGACAATTGGAGA---AGTGAATTATATAAATACAAAGTAGTAAAAATTGAACCA---TTAGGAGTAGCACCC---ACCAAGGCAAAGAGGAGAGTGGTGCAGAGAGAA---AAAAGAGCAGCA---------ATAGGA---GCTTTG---TTCCTT---GGG---------TTCTTGGGA---GCAGCAGGAAGCACTATGGGCGCAGCGTCACTG---ACGCTGACGGTACAGGCCAGACTATTATTGTCTGGTATAGTGCAACAGCAGAACAATTTGCTGAGGGCTATTGAGGCGCAACAGCATCTGTTGCAACTCACAGTCTGGGGCATCAAGCAGCTCCAGGCAAGA---GTCCTGGCTGTGGAAAGATACCTAAGGGATCAACAGCTCCTAGGGATTTGGGGTTGCTCTGGAAAAATCATTTGCACCACTGCTGTGCCTTGGAATAATACTTGGAGT---------------------------AATAAAAATCTGAGCCAGATTTGGGAT---AACATGACCTGGATGGAGTGGGAGAAAGAAATTGAT------AATTACACAGAAATAATATATGACTTGCTTGAAAAATCGCAAAACCAACAAGAAAAGAATGAACAAGAGTTATTGGAATTGGATAAATGGGCAAGTTTGTGGAATTGGTTTAGCATAACAAACTGGCTGTGGTATATAAAAATATTCATAATGATAGTAGGAGGCTTGATAGGTTTAAGAATAGTTTTTACTGTACTTTCTATAGTGAATAGAGTTAGGCAGGGATACTCACCATTGTCATTGCAGACCCTC---CTCCCAGCTCCGAGGGGA------CCCGGCAGGCCCGAAGGAACAGAAGAAGAAGGTGGAGAGCGAGACAGAGGCAGATCCGATCCATTAGCGACAGGATTCTTAGCACTTTTCTGGGACGACCTGAGGACCCTGTGCCTCTTCAGCTACCACCGCTTGAGAGACTTACTCTTGATTGTGACGAGGATTGTGGAACTTCTGGGACGCAGG---------------GGGTGGGAACTGCTCAAGTATTGGTGG---AATCTCCTACAGTATTGG---------------------------------------------------AGTCAGGAACTAAAGAATAGTGCTGTTAACTTGTTTGATACTGTAGCCATAGCAGCAGCTGAGGGGACAGATAGGGTTATAGAAATAATACAAAGA------------------GCTGGTAGAGCTATCCTCCACATCCCTAGAAGAATAAGACAGGGCGCGGAAAGGGCTTTGATATAA

2.04013296.ADARC.GU330524 ATGAAAGCGAAGGAGACCAAGAGGAATTATCAGCACTTG---------TGGAGATGGGGC------------------------ATCATGCTCCTTGGGATGTTAATGATG---------ATCTGTAGTGCTGCA---------GAACAATTGTGGGTCACAGTCTATTATGGAGTACCTGTGTGGAAAGATGCAAACACCACTCTATTTTGCGCATCAGATGCTAAGGCATATGATACAGAGGTACATAAT---GTTTGGGCCACACATGCCTGTGTACCCACAGACCCCAACCCACAAGCAATAGAATTA---AAAAATGTGACAGAAGATTTTAACATGTGGAAAAATAACATGGTAGAACAGATGCATGAGGATATAATCAGTTTATGGGATCAAAGTCTAAAGCCATGTGTAAAATTAACCCCACTCTGTGTCACTTTAGATTGCAATAATACTGTGACTCCCACTAGGACCAATAGTAGTAATAATGCCACTAGTTCCACTAACAGTCCT---------------------ACCACTAGTACTACCACTAGTCCCACTACCAGTACTACCAATAATAGTAGTTGGGAAAAAGAAGAAATAAAAAACTGCTCTTTCAATATCACC---ACAAGCATAAGAGAT------AAAGTGCAGAGGGAATATGCACTTTTTTATAGCCTTGATGTAGTACAAATAGATAAGACT---------------------------------------------------------------AGCTATAGGTTAATAAATTGTAACACCTCAGTCATTACACAGGCCTGTCCAAAGGTATCCTTTGAACCAATTCCCATACATTATTGTGCCCCGGCTGGTTTTGCGATTCTAAGGTGT---AATGATAAGAAGTTCAGTGGAAAAGGAAAATGTGACAATGTCAGCACAGTACAGTGTACACATGGAATTAAGCCAGTAGTGTCAACTCAACTGCTGTTAAATGGCAGTCTAGCAGAAGGA---GATGTAGTGCTTAAATCTGACAATTTTTCAAACAATGCTAAAACCATAATAGTACAGCTAAACGAAACTGTAAGAATTAATTGTTCAAGACCCAACAACAACACAAGAAAAGGTATACATATAGGA------------CCAGGGAGA---GCATTTTATACAACAGGAGAAATAATAGGAGACATAAGAAAGGCACATTGTAACATT------AGTAGAACAAACTGGACTAACACTTTAGGGCAGATAGCAAAAAAATTAGGA---GAACAGTTTAAC------------ACTAGCACA---GTAAACTTTAGACCA---------TCCTCAGGAGGGGACCCAGAAATTGTAATGCACAGTTTTAATTGTAGAGGGGAATTTTTCTACTGTAATACAACAAAACTGTTTTATAGTAATTGGACTATT---------AATGGGACTATTGTGAGT------------------------AATTGGACATATGAAAATGACACTGGAACAAATGACACT------------------------------ATCATACTCCCATGTAGAATAAAACAAATTATAAACATGTGGCAGGAAGTAGGAAAAGCAATGTATGCCCCTCCCATCAAAGGACCAATTACATGTAACTCAAGTATTACAGGGTTGCTATTAACAAGAGATGGTGGTATT---------------AACAGGACCAAT------------------AATGAGACC---GAGATCTTCAGACCTGTAGGGGGAGATATGAGGGACAATTGGAGA---AGTGAATTATATAAATATAAAGTAGTAGAAATTCAACCA---TTAGGAATAGCACCC---ACCAAGGCAAAGAGAAGAGTGGTGCAGAGAGAA---AAGAGAGCGGTA------ACACTAGGA---GCTATG---TTCCTT---GGG---------TTCTTGGGA---GCAGCAGGAAGCACTATGGGCGCAGCGTCAGTG---GCGCTGACGGTACAGGCCAGACAATTATTGTCTGGTATAGTGCAACAGCAGAACAATCTGCTGAGGGCTATTGAGGCGCAACAGCACATGTTGCAACTCACAGTCTGGGGCATTAAGCAGCTCCAGGCAAGA---GTCCTTGCTGTGGAGAGATACCTACGGGATCAACAGCTCCTAGGGATGTGGGGTTGCTCTGGAAAACTCATTTGCACCACTACTGTGCCTTGGAATACTAGTTGGAGT---------------------------GGTAAAAATCTAACTGACATTTGGGAT---AACATGACCTGGATGGAGTGGGAAAAAGAAATTGAC------AATTACACAGACCTCATATACACCTTACTTGAAGCATCGCAAACCCAACAAGAGATAAATGAACAAGAATTATTGGCACTAGATAAGTGGGCAAGCTTGTGGAATTGGTTTGACATAACAAACTGGCTGTGGTATATAAGATTATTCATAATGATAGTAGGAGGCTTGATAGGTTTAAGAATTATTTTTGCTGTGCTTTCTATAGTGAATAGAGTTAGGCAGGGATACTCACCATTATCATTTCAGACCCGC---TTCCCAGCCCCGAGGGGA------CCCGACAGGCCCGAAGGAATCGAAGAAGAAGGTGGAGAGAGAGACAGAGACAGATCCGGTCGATTAGTGACTGGATTCTTAGCACTTATCTGGGACGACCTACGGAGCCTGTGCATCTTCAGCTACCGCCACTTGAGAGACTTACTCTTGATTGCAGCGAGGATTGTGGAACTTCTGGGACGCAGG---------------GGGTGGGGAATCCTCAAGTATTGGTGG---AGTCTCCTGCAGTATTGG---------------------------------------------------AGTCAGGAACTAAAGAATAGTGCTGTTAACTTGCTTAATGCCACAGCTATCGCAGTAGCTGAGGGGACAGATAGGATTATAGAAGTAGTACAAAGA------------------GCTTATAGAGCTTTTATCCACATACCTAGAAGAATAAGACAGGGTTTAGAAAGAGCTTTGCAATAA

2.04013296.ADARC.GU330525 ATGAAAGCGAAGGAGACCAAGAGGAATTATCAGCACTTG---------TGGAGATGGGGC------------------------ATCATGCTCCTTGGGATGTTAATGATG---------ATCTGTAGTGCTGCA---------GAACAATTGTGGGTCACAGTCTATTATGGAGTACCTGTGTGGAAAGATGCAAACACCACTCTATTTTGCGCATCAGATGCTAAGGCATATGATACAGAGGTACATAAT---GTTTGGGCCACACATGCCTGTGTACCCACAGACCCCAACCCACAAGCAATAGAATTA---AAAAATGTGACAGAAGATTTTAACATGTGGAAAAATAACATGGTAGAACAGATGCATGAGGATATAATCAGTTTATGGGATCAAAGTCTAAAGCCATGTGTAAAATTAACCCCACTCTGTGTCACTTTAGATTGCAATAATACTGTGACTCCCACTAGGACCAATAGTAGTAATAATGCCACTAGTTCCACTAACAGTCCT---------------------ACCACTAGTACTACCACTAGTCCCACTACCAGTACTACCAATAATAGTAGTTGGGAAAAAGAAGAAATAAAAAACTGCTCTTTCAATATCACC---ACAAGCATAAGAGAT------AAAGTGCAGAGGGAATATGCACTTTTTTATAGCCTTGATGTAGTACAAATAGATAAGACT---------------------------------------------------------------AGCTATAGGTTAATAAATTGTAACACCTCAGTCATTACACAGGCCTGTCCAAAGGTATCCTTTGAACCAATTCCCATACATTATTGTGCCCCGGCTGGTTTTGCGATTCTAAGGTGT---AATGATAAGAAGTTCAGTGGAAAAGGAAAATGTGACAATGTCAGCACAGTACAGTGTACACATGGAATTAAGCCAGTAGTGTCAACTCAACTGCTGTTAAATGGCAGTCTAGCAGAAGGA---GATGTAGTGCTTAAATCTGACAATTTTTCAAACAATGCTAAAACCATAATAGTACAGCTAAACGAAACTGTAAGAATTAATTGTTCAAGACCCAACAACAACACAAGAAAAGGTATACATATAGGA------------CCAGGGAGA---GCATTTTATACAACAGGAGAAATAATAGGAGACATAAGAAAGGCACATTGTAACATT------AGTAGAACAAACTGGACTAACACTTTAGGGCAGATAGCAAAAAAATTAGGA---GAACAGTTTAAC------------ACTAGCACA---GTAAACTTTAGACCA---------TCCTCAGGAGGGGACCCAGAAATTGTAATGCACAGTTTTAATTGTAGAGGGGAATTTTTCTACTGTAATACAACAAAACTGTTTTATAGTAATTGGACTATT---------AATGGGACTATTGTGAGT------------------------AATTGGACATATGAAAATGACACTGGAACAAATGACACT------------------------------ATCATACTCCCATGTAGAATAAAACAAATTATAAACATGTGGCAGGAAGTAGGAAAAGCAATGTATGCCCCTCCCATCAAAGGACCAATTACATGTAACTCAAGTATTACAGGGTTGCTATTAACAAGAGATGGTGGTATT---------------AACAGGACCAAT------------------AATGAGACC---GAGATCTTCAGACCTGTAGGGGGAGATATGAGGGACAATTGGAGA---AGTGAATTATATAAATATAAAGTAGTAGAAATTCAACCA---TTAGGAATAGCACCC---ACCAAGGCAAAGAGAAGAGTGGTGCAGAGAGAA---AAGAGAGCGGTA------ACACTAGGA---GCTATG---TTCCTT---GGG---------TTCTTGGGA---GCAGCAGGAAGCACTATGGGCGCAGCGTCAGTG---GCGCTGACGGTACAGGCCAGACAATTATTGTCTGGTATAGTGCAACAGCAGAACAATCTGCTGAGGGCTATTGAGGCGCAACAGCACATGTTGCAACTCACAGTCTGGGGCATTAAGCAGCTCCAGGCAAGA---GTCCTTGCTGTGGAGAGATACCTACGGGATCAACAGCTCCTAGGGATGTGGGGTTGCTCTGGAAAACTCATTTGCACCACTACTGTGCCTTGGAATACTAGTTGGAGT---------------------------GGTAAAAATCTAACTGACATTTGGGAT---AACATGACCTGGATGGAGTGGGAAAAAGAAATTGAC------AATTACACAGACCTCATATACACCTTACTTGAAGCATCGCAAACCCAACAAGAGATAAATGAACAAGAATTATTGGCACTAGATAAGTGGGCAAGCTTGTGGAATTGGTTTGACATAACAAACTGGCTGTGGTATATAAGATTATTCATAATGATAGTAGGAGGCTTGATAGGTTTAAGAATTATTTTTGCTGTGCTTTCTATAGTGAATAGAGTTAGGCAGGGATACTCACCATTATCATTTCAGACCCGC---TTCCCAGCCCCGAGGGGA------CCCGACAGGCCCGAAGGAATCGAAGAAGAAGGTGGAGAGAGAGACAGAGACAGATCCGGTCGATTAGTGACTGGATTCTTAGCACTTATCTGGGACGACCTACGGAGCCTGTGCATCTTCAGCTACCGCCACTTGAGAGACTTACTCTTGATTGCAGCGAGGATTGTGGAACTTCTGGGACGCAGG---------------GGGTGGAGAATCCTCAAGTATTGGTGG---AGTCTCCTGCAGTATTGG---------------------------------------------------AGTCAGGAACTAAAGAATAGTGCTGTTAACTTGCTTAATGCCACAGCTATCGCAGTAGCTGAGGGGACAGATAGGATTATAGAAGTAGTACAAAGA------------------GCTTATAGAGCTTTTATCCACATACCTAGAAGAATAAGACAGGGTTTAGAAAGAGCTTTGCAATAA

2.04013296.ADARC.GU330526 ATGAAAGCGAAGGAGACCAAGAGGAATTATCAGCACTTG---------TGGAGATGGGGC------------------------ATCATGCTCCTTGGGATGTTAATGATG---------ATCTGTAGTGCTGCA---------GAACAATTGTGGGTCACAGTCTATTATGGAGTACCTGTGTGGAAAGATGCAAACACCACTCTATTTTGCGCATCAGATGCTAAGGCATATGATACAGAGGTACATAAT---GTTTGGGCCACACATGCCTGTGTACCCACAGACCCCAACCCACAAGCAATAGAATTA---AAAAATGTGACAGAAGATTTTAACATGTGGAAAAATAACATGGTAGAACAGATGCATGAGGATATAATCAGTTTATGGGATCAAAGTCTAAAGCCATGTGTAAAATTAACCCCACTCTGTGTCACTTTAGATTGCAATAATACTGTGACTCCCACTAGGACCAATAGTAGTAATAATGCCACTAGTTCCACTAACAGTCCT---------------------ACCACTAGTACTACCACTAGTCCCACTACCAGTACTACCAATAATAGTAGTTGGGAAAAAGAAGAAATAAAAAACTGCTCTTTCAATATCACC---ACAAGCATAAGAGAT------AAAGTGCAGAGGGAATATGCACTTTTTTATAGCCTTGATGTAGTACAAATAGATAAGACT---------------------------------------------------------------AGCTATAGGTTAATAAATTGTAACACCTCAGTCATTACACAGGCCTGTCCAAAGGTATCCTTTGAACCAATTCCCATACATTATTGTGCCCCGGCTGGTTTTGCGATTCTAAGGTGT---AATGATAAGAAGTTCAGTGGAAAAGGAAAATGTGACAATGTCAGCACAGTACAGTGTACACATGGAATTAAGCCAGTAGTGTCAACTCAACTGCTGTTAAATGGCAGTCTAGCAGAAGGA---GATGTAGTGCTTAAATCTGACAATTTTTCAAACAATGCTAAAACCATAATAGTACAGCTAAACGAAACTGTAAGAATTAATTGTTCAAGACCCAACAACAACACAAGAAAAGGTATACATATAGGA------------CCAGGGAGA---GCATTTTATACAACAGGAGAAATAATAGGAGACATAAGAAAGGCACATTGTAACATT------AGTAGAACAAACTGGACTAACACTTTAGGGCAGATAGC-AAAAAATTAGGA---GAACAGTTTAAC------------ACTAGCACA---GTAAACTTTAGACCA---------TCCTCAGGAGGGGACCCAGAAATTGTAATGCACAGTTTTAATTGTAGAGGGGAATTTTTCTACTGTAATACAACAAAACTGTTTTATAGTAATTGGACTATT---------AATGGGACTATTGTGAGT------------------------AATTGGACATATGAAAATGACACTGGAACAAATGACACT------------------------------ATCATACTCCCATGTAGAATAAAACAAATTATAAACATGTGGCAGGAAGTAGGAAAAGCAATGTATGCCCCTCCCATCAAAGGACCAATTACATGTAACTCAAGTATTACAGGGTTGCTATTAACAAGAGATGGTGGTATT---------------AACAGGACCAAT------------------AATGAGACC---GAGATCTTCAGACCTGTAGGGGGAGATATGAGGGACAATTGGAGA---AGTGAATTATATAAATATAAAGTAGTAGAAATTCAACCA---TTAGGAATAGCACCC---ACCAAGGCAAAGAGAAGAGTGGTGCAGAGAGAA---AAGAGAGCGGTA------ACACTAGGA---GCTATG---TTCCTT---GGG---------TTCTTGGGA---GCAGCAGGAAGCACTATGGGCGCAGCGTCAGTG---GCGCTGACGGTACAGGCCAGACAATTATTGTCTGGTATAGTGCAACAGCAGAACAATCTGCTGAGGGCTATTGAGGCGCAACAGCACATGTTGCAACTCACAGTCTGGGGCATTAAGCAGCTCCAGGCAAGA---GTCCTTGCTGTGGAGAGATACCTACGGGATCAACAGCTCCTAGGGATGTGGGGTTGCTCTGGAAAACTCATTTGCACCACTACTGTGCCTTGGAATACTAGTTGGAGT---------------------------GGTAAAAATCTAACTGACATTTGGGAT---AACATGACCTGGATGGAGTGGGAAAAAGAAATTGAC------AATTACACAGACCTCATATACACCTTACTTGAAGCATCGCAAACCCAACAAGAGATAAATGAACAAGAATTATTGGCACTAGATAAGTGGGCAAGCTTGTGGAATTGGTTTGACATAACAAACTGGCTGTGGTATATAAGATTATTCATAATGATAGTAGGAGGCTTGATAGGTTTAAGAATTATTTTTGCTGTGCTTTCTATAGTGAATAGAGTTAGGCAGGGATACTCACCATTATCATTTCAGACCCGC---TTCCCAGCCCCGAGGGGA------CCCGACAGGCCCGAAGGAATCGAAGAAGAAGGTGGAGAGAGAGACAGAGACAGATCCGGTCGATTAGTGACTGGATTCTTAGCACTTATCTGGGACGACCTACGGAGCCTGTGCATCTTCAGCTACCGCCACTTGAGAGACTTACTCTTGATTGCAGCGAGGATTGTGGAACTTCTGGGACGCAGG---------------GGGTGGGGAATCCTCAAGTATTGGTGG---AGTCTCCTGCAGTATTGG---------------------------------------------------AGTCAGGAACTAAAGAATAGTGCTGTTAACTTGCTTAATGCCACAGCTATCGCAGTAGCTGAGGGGACAGATAGGATTATAGAAGTAGTACAAAGA------------------GCTTATAGAGCTTTTATCCACATACCTAGAAGAATAAGACAGGGTTTAGAAAGAGCTTTGCAATAA
[truncated: 7,792,814 more chars]
