# Supplementary material for: Recurrent Signature Patterns in HIV-1 B Clade Envelope Glycoproteins Associated with either Early or Chronic Infections
Source: PLoS Pathog. 2011 Sep 29;7(9):e1002209. doi: 10.1371/journal.ppat.1002209 (PMC3182927; doi:10.1371/journal.ppat.1002209)
Supplement: Table S7 — All sequences generated for this study under PlasmaDonors Set , aligned. The first character of each sequence name indicates either the Fiebig stage at time of sampling, or C for chronic infection (see material and methods for details). The GenBank numbers are all included in the name of each sequence in the files; as there are thousands of sequences, and the numbers are not continuous, this seemed the most parsimonious presentation. (DOC) [file ppat.1002209.s014.doc]

1.62995.SPD.EU575639 ATGAGAGCGAAGGGGATCAGGAAGAATTATCAGTACTTG---------TGGAGAGGGGGC------------------------ACCTTGCTCCTTGGGATATTGATGATC------------TGTAGTGCTGTA---------GAACAATTATGGGTCACAGTCTATTATGGGGTACCTGTGTGGAAAGAAGCAACCACCACTCTATTTTGTGCATCAGATGCTAAATCATATAGTACAGAGGTACATAAT---GTCTGGGCCACACATGCCTGTGTACCCACAGACCCTAGCCCACAAGAAGTAGTATTG---CAAAATGTGACAGAAAATTTTAACATGTGGAAAAATAACATGGTAGAACAAATGCATGAGGATATAATCAGTTTATGGGATCAAAGCCTAAAGCCATGTGTAAAATTAACCCCACTCTGTGTTACTCTAAATTGCTCTGATTTTAATGCTACTCAGGCCACTACTACTCAGGCC---------------------------------------------------------------------------------TATAATAGTAGCTGGAAGGTG------GAAGGAATGAAAAACTGCTCTTTCAATACCACC---TCAGGAATAAGAGAT------AAGGTGAAGAAAGAATATGCACTTCTTTATAAACTTGATATAGTAGAAATAAATGATGAT------------------------------AATAATACT---GGGGATAATACTAGCTATAGGTTCTATAGGTTGCTAAGTTGTAACACCTCGGTCATGACACAGGCCTGTCCAAAGGTATCCTTTGAGCCAATTCCCATACATTATTGTGCCCCAGCTGGTTTTGCGATTCTAAAGTGT---AATAATAAGACGTTCAATGGAACAGGACCGTGTACAAATGTCAGCACAGTACAATGTACACATGGAATTAGGCCAGTAGTATCAACTCAACTGCTGTTAAATGGCAGTCTAGCAGAAAAA---GAGATAGTAATTAGATCTGAAAATATCACGGACAATGCTAAGACCATAATAGTACAGTTGAATACAGCTGTAGAAATTAATTGTACAAGACCCAGCAACAATACAAGAAAAAGTATAAGTATAGGA------------CCAGGGAGA---GCATTTTATACAACAGGAGAGGTAATAGGAGATATAAGACAAGCACATTGCAACATT------AGTCAAGCAAAATGGAATAACACTCTAAGACAGGTGGCTATTAAATTAAGA---GAACAATTTCCG------------AATAAAACA---ATAATCTTTAATCAA---------TCCTCAGGAGGGGACCTAGAAATTGAAATGCACAGTTTTAATTGTGGAGGGGAATTTTTCTACTGCAATACAACAATACTGTTTAATAGTACTTGG---------------AATGAAACTAACCTGTTT------------------------AATGCCACTGAAGGA---------------AATAACACAGAA------------------------GTTATTACACTCCAATGCAGAATAAAACAAATTATAAACATGTGGCAGGAAGTAGGAAAAGCAATGTATGCCCCTCCCATCAGTGGACAAATTAACTGTTCATCAAACATTACAGGGCTGCTATTAACAAGAGATGGCGGTATTGGGAAC---------AATATCACC---------------------AATACCTCC---GAGATCTTCAGACCTATAGGAGGAAATATGAGGGACAATTGGAGA---AGTGAATTATATAAATATAAAGTAGTCAAAATTGAACCA---ATAGGAATAGCACCC---ACCAAGGCAAAGAGAAGAGTGGTGCAGAGAGAA---AAAAGAGCAGTG---GGA---ATAGGA---GCTATG---TTCCTT---GGG---------TTCTTGGGA---GCAGCAGGAAGCACTATGGGCGCAGCGTCAGTG---ACGCTGACGGTACAGGCCAGACTATTATTGTCTGGTATAGTGCAACAGCAGAACAATCTGCTGAGAGCTATTGAGGCGCAACAACATCTGTTGCAACTCACAGTCTGGGGCATCAAGCAGCTCCAGGCAAGA---GTCCTGGCTGTGGAAAGATACCTAAAGGATCAACAGCTCCTGGGGATTTGGGGTTGCTCTGGAAAACTCATTTGCACCACTGCTGTGCCTTGGAATACTAGTTGGAGT---------------------------AATAAATCTCTGGAAAAAATTTGGAAT---AACATGACCTGGATGGAGTGGGAAAGAGAAATAGAC------AATTATACAAACTTAATATATAACTTACTTGAAGACTCGCAAAACCAACAAGACAAGAATGAAAAAGAATTATTGGAATTAGATAAATGGGCAAATTTGTGGAATTGGTTTGACATAACAAAGTGGCTGTGGTATATAAAAATATTCATAATGATAGTAGGAGGCTTAGTAGGTTTACGAATAGTTTTTTCTGTACTTTCTATAGTGAATAGAGTTAGGCAGGGATACTCACCACTATCGTTTCAGACCCAC---TCCCCAGCCCCGAGGGGA------CCCGACAGGCCCGGAGGAACAGAAGAAGAAGGTGGAGAAAGAGACAGAGACAGATCCGGTCCATTTGTGAACGGATTCTTGACGCTCATCTGGGTCGACCTGAGGAGCCTGTGCCTCTTCTGTTACCACCGCTTGAGAGACTTACTCTTGATTCTAGCGAGGATTGTAGAACTTCTGGGACGCAGG---------------GGGTGGGAAATCCTCAAATATTGGTGG---AATATCCTACAGTATTGG---------------------------------------------------AGTCAGGAACTAAAGAATAGCGCTGTCAGCTTGCTCAACGCCATAGCCATAGCAGTAGCTGAGGGGACAGATAGGATTATAGAAATAGTACAAAGA------------------GGTTTTAGA---------------------AGAATAAGACAGGGCTTGGAAAGGGCTGTGCTATAA

1.62995.SPD.EU575627 ATGAGAGCGAAGGGGATCAGGAAGAATTATCAGTACTTG---------TGGAGAGGGGGC------------------------ACCTTGCTCCTTGGGATATTGATGATC------------TGTAGTGCTGTA---------GAACAATTATGGGTCACAGTCTATTATGGGGTACCTGTGTGGAAAGAAGCAACCACCACTCTATTTTGTGCATCAGATGCTAAATCATATAGTACAGAGGTACATAAT---GTCTGGGCCACACATGCCTGTGTACCCACAGACCCTAGCCCACAAGAAGTAGTATTG---CAAAATGTGACAGAAAATTTTAACATGTGGAAAAATAACATGGTAGAACAAATGCATGAGGATATAATCAGTTTATGGGATCAAAGCCTAAAGCCATGTGTAAAATTAACCCCACTCTGTGTTACTCTAAATTGCTCTGATTTTAATGCTACTCAGGCCACTACTACTCAGGCC---------------------------------------------------------------------------------TATAATAGTAGCTGGAAGGTG------GAAGGAATGAAAAACTGCTCTTTCAATACCACC---TCAGGAATAAGAGAT------AAGGTGAAGAAAGAATATGCACTTCTTTATAAACTTGATATAGTAGAAATAAATGATGAT------------------------------AATAATACT---GGGGATAATACTAGCTATAGGTTCTATAGGTTGCTAAGTTGTAACACCTCGGTCATGACACAGGCCTGTCCAAAGGTATCCTTTGAGCCAATTCCCATACATTATTGTGCCCCAGCTGGTTTTGCGATTCTAAAGTGT---AATAATAAGACGTTCAATGGAACAGGACCGTGTACAAATGTCAGCACAGTACAATGTACACATGGAATTAGGCCAGTAGTATCAACTCAACTGCTGTTAAATGGCAGTCTAGCAGAAAAA---GAGATAGTAATTAGATCTGAAAATATCACGGACAATGCTAAGACCATAATAGTACAGTTGAATACAGCTGTAGAAATTAATTGTACAAGACCCAGCAACAATACAAGAAAAAGTATAAGTATAGGA------------CCAGGGAGA---GCATTTTATACAACAGGAGAGGTAATAGGAGATATAAGACAAGCACATTGCAACATT------AGTCAAGCAAAATGGAATAACACTCTAAGACAGGTGGCTATTAAATTAAGA---GAACAATTTCCG------------AATAAAACA---ATAATCTTTAATCAA---------TCCTCAGGAGGGGACCTAGAAATTGAAATGCACAGTTTTAATTGTGGAGGGGAATTTTTCTACTGCAATACAACAATACTGTTTAATAGTACTTGG---------------AATGAAACTAACCTGTTT------------------------AATGCCACTGAAGGA---------------AATAACACAGAA------------------------GTTATTACACTCCAATGCAGAATAAAACAAATTATAAACATGTGGCAGGAAGTAGGAAAAGCAATGTATGCCCCTCCCATCAGTGGACAAATTAACTGTTCATCAAACATTACAGGGCTGCTATTAACAAGAGATGGCGGTATTGGGAAC---------AATATCACC---------------------AATACCTCC---GAGATCTTCAGACCTATAGGAGGAAATATGAGGGACAATTGGAGA---AGTGAATTATATAAATATAAAGTAGTCAAAATTGAACCA---ATAGGAATAGCACCC---ACCAAGGCAAAGAGAAGAGTGGTGCAGAGAGAA---AAAAGAGCAGTG---GGA---ATAGGA---GCTATG---TTCCTT---GGG---------TTCTTGGGA---GCAGCAGGAAGCACTATGGGCGCAGCGTCAGTG---ACGCTGACGGTACAGGCCAGACTATTATTGTCTGGTATAGTGCAACAGCAGAACAATCTGCTGAGAGCTATTGAGGCGCAACAACATCTGTTGCAACTCACAGTCTGGGGCATCAAGCAGCTCCAGGCAAGA---GTCCTGGCTGTGGAAAGATACCTAAAGGATCAACAGCTCCTGGGGATTTGGGGTTGCTCTGGAAAACTCATTTGCACCACTGCTGTGCCTTGGAATACTAGTTGGAGT---------------------------AATAAATCTCTGGAAAAAATTTGGAAT---AACATGACCTGGATGGAGTGGGAAAGAGAAATAGAC------AATTATACAAACTTAATATATAACTTACTTGAAGACTCGCAAAACCAACAAGACAAGAATGAAAAAGAATTATTGGAATTAGATAAATGGGCAAATTTGTGGAATTGGTTTGACATAACAAAGTGGCTGTGGTATATAAAAATATTCATAATGATAGTAGGAGGCTTAGTAGGTTTACGAATAGTTTTTTCTGTACTTTCTATAGTGAATAGAGTTAGGCAGGGATACTCACCACTATCGTTTCAGACCCAC---TCCCCAGCCCCGAGGGGA------CCCGACAGGCCCGGAGGAACAGAAGAAGAAGGTGGAGAAAGAGACAGAGACAGATCCGGTCCATTTGTGAACGGATTCTTGACGCTCATCTGGGTCGACCTGAGGAGCCTGTGCCTCTTCTGTTACCACCGCTTGAGAGACTTACTCTTGATTCTAGCGAGGATTGTAGAACTTCTGGGACGCAGG---------------GGGTGGGAAATCCTCAAATATTGGTGG---AATATCCTACAGTATTGG---------------------------------------------------AGTCAGGAACTAAAGAATAGCGCTGTCAGCTTGCTCAACGCCATAGCCATAGCAGTAGCTGAGGGGACAGATAGGATTATAGAAATAGTACAAAGA------------------GGTTTTAGA---------------------AGAATAAGACAGGGCTTGGAAAGGGCTGTGCTATAA

1.62995.SPD.EU575647 ATGAGAGCGAAGGGGATCAGGAAGAATTATCAGTACTTG---------TGGAGAGGGGGC------------------------ACCTTGCTCCTTGGGATATTGATGATC------------TGTAGTGCTGTA---------GAACAATTATGGGTCACAGTCTATTATGGGGTACCTGTGTGGAAAGAAGCAACCACCACTCTATTTTGTGCATCAGATGCTAAATCATATAGTACAGAGGTACATAAT---GTCTGGGCCACACATGCCTGTGTACCCACAGACCCTAGCCCACAAGAAGTAGTATTG---CAAAATGTGACAGAAAATTTTAACATGTGGAAAAATAACATGGTAGAACAAATGCATGAGGATATAATCAGTTTATGGGATCAAAGCCTAAAGCCATGTGTAAAATTAACCCCACTCTGTGTTACTCTAAATTGCTCTGATTTTAATGCTACTCAGGCCACTACTACTCAGGCC---------------------------------------------------------------------------------TATAATAGTAGCTGGAAGGTG------GAAGGAATGAAAAACTGCTCTTTCAATACCACC---TCAGGAATAAGAGAT------AAGGTGAAGAAAGAATATGCACTTCTTTATAAACTTGATATAGTAGAAATAAATGATGAT------------------------------AATAATACT---GGGGATAATACTAGCTATAGGTTCTATAGGTTGCTAAGTTGTAACACCTCGGTCATGACACAGGCCTGTCCAAAGGTATCCTTTGAGCCAATTCCCATACATTATTGTGCCCCAGCTGGTTTTGCGATTCTAAAGTGT---AATAATAAGACGTTCAATGGAACAGGACCGTGTACAAATGTCAGCACAGTACAATGTACACATGGAATTAGGCCAGTAGTATCAACTCAACTGCTGTTAAATGGCAGTCTAGCAGAAAAA---GAGATAGTAATTAGATCTGAAAATATCACGGACAATGCTAAGACCATAATAGTACAGTTGAATACAGCTGTAGAAATTAATTGTACAAGACCCAGCAACAATACAAGAAAAAGTATAAGTATAGGA------------CCAGGGAGA---GCATTTTATACAACAGGAGAGGTAATAGGAGATATAAGACAAGCACATTGCAACATT------AGTCAAGCAAAATGGAATAACACTCTAAGACAGGTGGCTATTAAATTAAGA---GAACAATTTCCG------------AATAAAACA---ATAATCTTTAATCAA---------TCCTCAGGAGGGGACCTAGAAATTGAAATGCACAGTTTTAATTGTGGAGGGGAATTTTTCTACTGCAATACAACAATACTGTTTAATAGTACTTGG---------------AATGAAACTAACCTGTTT------------------------AATGCCACTGAAGGA---------------AATAACACAGAA------------------------GTTATTACACTCCAATGCAGAATAAAACAAATTATAAACATGTGGCAGGAAGTAGGAAAAGCAATGTATGCCCCTCCCATCAGTGGACAAATTAACTGTTCATCAAACATTACAGGGCTGCTATTAACAAGAGATGGCGGTATTGGGAAC---------AATATCACC---------------------AATACCTCC---GAGATCTTCAGACCTATAGGAGGAAATATGAGGGACAATTGGAGA---AGTGAATTATATAAATATAAAGTAGTCAAAATTGAACCA---ATAGGAATAGCACCC---ACCAAGGCAAAGAGAAGAGTGGTGCAGAGAGAA---AAAAGAGCAGTG---GGA---ATAGGA---GCTATG---TTCCTT---GGG---------TTCTTGGGA---GCAGCAGGAAGCACTATGGGCGCAGCGTCAGTG---ACGCTGACGGTACAGGCCAGACTATTATTGTCTGGTATAGTGCAACAGCAGAACAATCTGCTGAGAGCTATTGAGGCGCAACAACATCTGTTGCAACTCACAGTCTGGGGCATCAAGCAGCTCCAGGCAAGA---GTCCTGGCTGTGGAAAGATACCTAAAGGATCAACAGCTCCTGGGGATTTGGGGTTGCTCTGGAAAACTCATTTGCACCACTGCTGTGCCTTGGAATACTAGTTGGAGT---------------------------AATAAATCTCTGGAAAAAATTTGGAAT---AACATGACCTGGATGGAGTGGGAAAGAGAAATAGAC------AATTATACAAACTTAATATATAACTTACTTGAAGACTCGCAAAACCAACAAGACAAGAATGAAAAAGAATTATTGGAATTAGATAAATGGGCAAATTTGTGGAATTGGTTTGACATAACAAAGTGGCTGTGGTATATAAAAATATTCATAATGATAGTAGGAGGCTTAGTAGGTTTACGAATAGTTTTTTCTGTACTTTCTATAGTGAATAGAGTTAGGCAGGGATACTCACCACTATCGTTTCAGACCCAC---TCCCCAGCCCCGAGGGGA------CCCGACAGGCCCGGAGGAACAGAAGAAGAAGGTGGAGAAAGAGACAGAGACAGATCCGGTCCATTTGTGAACGGATTCTTGACGCTCATCTGGGTCGACCTGAGGAGCCTGTGCCTCTTCTGTTACCACCGCTTGAGAGACTTACTCTTGATTCTAGCGAGGATTGTAGAACTTCTGGGACGCAGG---------------GGGTGGGAAATCCTCAAATATTGGTGG---AATATCCTACAGTATTGG---------------------------------------------------AGTCAGGAACTAAAGAATAGCGCTGTCAGCTTGCTCAACGCCATAGCCATAGCAGTAGCTGAGGGGACAGATAGGATTATAGAAATAGTACAAAGA------------------GGTTTTAGA---------------------AGAATAAGACAGGGCTTGGAAAGGGCTGTGCTATAA

1.62995.SPD.EU575630 ATGAGAGCGAAGGGGATCAGGAAGAATTATCAGTACTTG---------TGGAGAGGGGGC------------------------ACCTTGCTCCTTGGGATATTGATGATC------------TGTAGTGCTGTA---------GAACAATTATGGGTCACAGTCTATTATGGGGTACCTGTGTGGAAAGAAGCAACCACCACTCTATTTTGTGCATCAGATGCTAAATCATATAGTACAGAGGTACATAAT---GTCTGGGCCACACATGCCTGTGTACCCACAGACCCTAGCCCACAAGAAGTAGTATTG---CAAAATGTGACAGAAAATTTTAACATGTGGAAAAATAACATGGTAGAACAAATGCATGAGGATATAATCAGTTTATGGGATCAAAGCCTAAAGCCATGTGTAAAATTAACCCCACTCTGTGTTACTCTAAATTGCTCTGATTTTAATGCTACTCAGGCCACTACTACTCAGGCC---------------------------------------------------------------------------------TATAATAGTAGCTGGAAGGTG------GAAGGAATGAAAAACTGCTCTTTCAATACCACC---TCAGGAATAAGAGAT------AAGGTGAAGAAAGAATATGCACTTCTTTATAAACTTGATATAGTAGAAATAAATGATGAT------------------------------AATAATACT---GGGGATAATACTAGCTATAGGTTCTATAGGTTGCTAAGTTGTAACACCTCGGTCATGACACAGGCCTGTCCAAAGGTATCCTTTGAGCCAATTCCCATACATTATTGTGCCCCAGCTGGTTTTGCGATTCTAAAGTGT---AATAATAAGACGTTCAATGGAACAGGACCGTGTACAAATGTCAGCACAGTACAATGTACACATGGAATTAGGCCAGTAGTATCAACTCAACTGCTGTTAAATGGCAGTCTAGCAGAAAAA---GAGATAGTAATTAGATCTGAAAATATCACGGACAATGCTAAGACCATAATAGTACAGTTGAATACAGCTGTAGAAATTAATTGTACAAGACCCAGCAACAATACAAGAAAAAGTATAAGTATAGGA------------CCAGGGAGA---GCATTTTATACAACAGGAGAGGTAATAGGAGATATAAGACAAGCACATTGCAACATT------AGTCAAGCAAAATGGAATAACACTCTAAGACAGGTGGCTATTAAATTAAGA---GAACAATTTCCG------------AATAAAACA---ATAATCTTTAATCAA---------TCCTCAGGAGGGGACCTAGAAATTGAAATGCACAGTTTTAATTGTGGAGGGGAATTTTTCTACTGCAATACAACAATACTGTTTAATAGTACTTGG---------------AATGAAACTAACCTGTTT------------------------AATGCCACTGAAGGA---------------AATAACACAGAA------------------------GTTATTACACTCCAATGCAGAATAAAACAAATTATAAACATGTGGCAGGAAGTAGGAAAAGCAATGTATGCCCCTCCCATCAGTGGACAAATTAACTGTTCATCAAACATTACAGGGCTGCTATTAACAAGAGATGGCGGTATTGGGAAC---------AATATCACC---------------------AATACCTCC---GAGATCTTCAGACCTATAGGAGGAAATATGAGGGACAATTGGAGA---AGTGAATTATATAAATATAAAGTAGTCAAAATTGAACCA---ATAGGAATAGCACCC---ACCAAGGCAAAGAGAAGAGTGGTGCAGAGAGAA---AAAAGAGCAGTG---GGA---ATAGGA---GCTATG---TTCCTT---GGG---------TTCTTGGGA---GCAGCAGGAAGCACTATGGGCGCAGCGTCAGTG---ACGCTGACGGTACAGGCCAGACTATTATTGTCTGGTATAGTGCAACAGCAGAACAATCTGCTGAGAGCTATTGAGGCGCAACAACATCTGTTGCAACTCACAGTCTGGGGCATCAAGCAGCTCCAGGCAAGA---GTCCTGGCTGTGGAAAGATACCTAAAGGATCAACAGCTCCTGGGGATTTGGGGTTGCTCTGGAAAACTCATTTGCACCACTGCTGTGCCTTGGAATACTAGTTGGAGT---------------------------AATAAATCTCTGGAAAAAATTTGGAAT---AACATGACCTGGATGGAGTGGGAAAGAGAAATAGAC------AATTATACAAACTTAATATATAACTTACTTGAAGACTCGCAAAACCAACAAGACAAGAATGAAAAAGAATTATTGGAATTAGATAAATGGGCAAATTTGTGGAATTGGTTTGACATAACAAAGTGGCTGTGGTATATAAAAATATTCATAATGATAGTAGGAGGCTTAGTAGGTTTACGAATAGTTTTTTCTGTACTTTCTATAGTGAATAGAGTTAGGCAGGGATACTCACCACTATCGTTTCAGACCCAC---TCCCCAGCCCCGAGGGGA------CCCGACAGGCCCGGAGGAACAGAAGAAGAAGGTGGAGAAAGAGACAGAGACAGATCCGGTCCATTTGTGAACGGATTCTTGACGCTCATCTGGGTCGACCTGAGGAGCCTGTGCCTCTTCTGTTACCACCGCTTGAGAGACTTACTCTTGATTCTAGCGAGGATTGTAGAACTTCTGGGACGCAGG---------------GGGTGGGAAATCCTCAAATATTGGTGG---AATATCCTACAGTATTGG---------------------------------------------------AGTCAGGAACTAAAGAATAGCGCTGTCAGCTTGCTCAACGCCATAGCCATAGCAGTAGCTGAGGGGACAGATAGGATTATAGAAATAGTACAAAGA------------------GGTTTTAGA---------------------AGAATAAGACAGGGCTTGGAAAGGGCTGTGCTATAA

1.62995.SPD.EU575641 ATGAGAGCGAAGGGGATCAGGAAGAATTATCAGTACTTG---------TGGAGAGGGGGC------------------------ACCTTGCTCCTTGGGATATTGATGATC------------TGTAGTGCTGTA---------GAACAATTATGGGTCACAGTCTATTATGGGGTACCTGTGTGGAAAGAAGCAACCACCACTCTATTTTGTGCATCAGATGCTAAATCATATAGTACAGAGGTACATAAT---GTCTGGGCCACACATGCCTGTGTACCCACAGACCCTAGCCCACAAGAAGTAGTATTG---CAAAATGTGACAGAAAATTTTAACATGTGGAAAAATAACATGGTAGAACAAATGCATGAGGATATAATCAGTTTATGGGATCAAAGCCTAAAGCCATGTGTAAAATTAACCCCACTCTGTGTTACTCTAAATTGCTCTGATTTTAATGCTACTCAGGCCACTACTACTCAGGCC---------------------------------------------------------------------------------TATAATAGTAGCTGGAAGGTG------GAAGGAATGAAAAACTGCTCTTTCAATACCACC---TCAGGAATAAGAGAT------AAGGTGAAGAAAGAATATGCACTTCTTTATAAACTTGATATAGTAGAAATAAATGATGAT------------------------------AATAATACT---GGGGATAATACTAGCTATAGGTTCTATAGGTTGCTAAGTTGTAACACCTCGGTCATGACACAGGCCTGTCCAAAGGTATCCTTTGAGCCAATTCCCATACATTATTGTGCCCCAGCTGGTTTTGCGATTCTAAAGTGT---AATAATAAGACGTTCAATGGAACAGGACCGTGTACAAATGTCAGCACAGTACAATGTACACATGGAATTAGGCCAGTAGTATCAACTCAACTGCTGTTAAATGGCAGTCTAGCAGAAAAA---GAGATAGTAATTAGATCTGAAAATATCACGGACAATGCTAAGACCATAATAGTACAGTTGAATACAGCTGTAGAAATTAATTGTACAAGACCCAGCAACAATACAAGAAAAAGTATAAGTATAGGA------------CCAGGGAGA---GCATTTTATACAACAGGAGAGGTAATAGGAGATATAAGACAAGCACATTGCAACATT------AGTCAAGCAAAATGGAATAACACTCTAAGACAGGTGGCTATTAAATTAAGA---GAACAATTTCCG------------AATAAAACA---ATAATCTTTAATCAA---------TCCTCAGGAGGGGACCTAGAAATTGAAATGCACAGTTTTAATTGTGGAGGGGAATTTTTCTACTGCAATACAACAATACTGTTTAATAGTACTTGG---------------AATGAAACTAACCTGTTT------------------------AATGCCACTGAAGGA---------------AATAACACAGAA------------------------GTTATTACACTCCAATGCAGAATAAAACAAATTATAAACATGTGGCAGGAAGTAGGAAAAGCAATGTATGCCCCTCCCATCAGTGGACAAATTAACTGTTCATCAAACATTACAGGGCTGCTATTAACAAGAGATGGCGGTATTGGGAAC---------AATATCACC---------------------AATACCTCC---GAGATCTTCAGACCTATAGGAGGAAATATGAGGGACAATTGGAGA---AGTGAATTATATAAATATAAAGTAGTCAAAATTGAACCA---ATAGGAATAGCACCC---ACCAAGGCAAAGAGAAGAGTGGTGCAGAGAGAA---AAAAGAGCAGTG---GGA---ATAGGA---GCTATG---TTCCTT---GGG---------TTCTTGGGA---GCAGCAGGAAGCACTATGGGCGCAGCGTCAGTG---ACGCTGACGGTACAGGCCAGACTATTATTGTCTGGTATAGTGCAACAGCAGAACAATCTGCTGAGAGCTATTGAGGCGCAACAACATCTGTTGCAACTCACAGTCTGGGGCATCAAGCAGCTCCAGGCAAGA---GTCCTGGCTGTGGAAAGATACCTAAAGGATCAACAGCTCCTGGGGATTTGGGGTTGCTCTGGAAAACTCATTTGCACCACTGCTGTGCCTTGGAATACTAGTTGGAGT---------------------------AATAAATCTCTGGAAAAAATTTGGAAT---AACATGACCTGGATGGAGTGGGAAAGAGAAATAGAC------AATTATACAAACTTAATATATAACTTACTTGAAGACTCGCAAAACCAACAAGACAAGAATGAAAAAGAATTATTGGAATTAGATAAATGGGCAAATTTGTGGAATTGGTTTGACATAACAAAGTGGCTGTGGTATATAAAAATATTCATAATGATAGTAGGAGGCTTAGTAGGTTTACGAATAGTTTTTTCTGTACTTTCTATAGTGAATAGAGTTAGGCAGGGATACTCACCACTATCGTTTCAGACCCAC---TCCCCAGCCCCGAGGGGA------CCCGACAGGCCCGGAGGAACAGAAGAAGAAGGTGGAGAAAGAGACAGAGACAGATCCGGTCCATTTGTGAACGGATTCTTGACGCTCATCTGGGTCGACCTGAGGAGCCTGTGCCTCTTCTGTTACCACCGCTTGAGAGACTTACTCTTGATTCTAGCGAGGATTGTAGAACTTCTGGGACGCAGG---------------GGGTGGGAAATCCTCAAATATTGGTGG---AATATCCTACAGTATTGG---------------------------------------------------AGTCAGGAACTAAAGAATAGCGCTGTCAGCTTGCTCAACGCCATAGCCATAGCAGTAGCTGAGGGGACAGATAGGATTATAGAAATAGTACAAAGA------------------GGTTTTAGA---------------------AGAATAAGACAGGGCTTGGAAAGGGCTGTGCTATAA

1.62995.SPD.EU575633 ATGAGAGCGAAGGGGATCAGGAAGAATTATCAGTACTTG---------TGGAGAGGGGGC------------------------ACCTTGCTCCTTGGGATATTGATGATC------------TGTAGTGCTGTA---------GAACAATTATGGGTCACAGTCTATTATGGGGTACCTGTGTGGAAAGAAGCAACCACCACTCTATTTTGTGCATCAGATGCTAAATCATATAGTACAGAGGTACATAAT---GTCTGGGCCACACATGCCTGTGTACCCACAGACCCTAGCCCACAAGAAGTAGTATTG---CAAAATGTGACAGAAAATTTTAACATGTGGAAAAATAACATGGTAGAACAAATGCATGAGGATATAATCAGTTTATGGGATCAAAGCCTAAAGCCATGTGTAAAATTAACCCCACTCTGTGTTACTCTAAATTGCTCTGATTTTAATGCTACTCAGGCCACTACTACTCAGGCC---------------------------------------------------------------------------------TATAATAGTAGCTGGAAGGTG------GAAGGAATGAAAAACTGCTCTTTCAATACCACC---TCAGGAATAAGAGAT------AAGGTGAAGAAAGAATATGCACTTCTTTATAAACTTGATATAGTAGAAATAAATGATGAT------------------------------AATAATACT---GGGGATAATACTAGCTATAGGTTCTATAGGTTGCTAAGTTGTAACACCTCGGTCATGACACAGGCCTGTCCAAAGGTATCCTTTGAACCAATTCCCATACATTATTGTGCCCCAGCTGGTTTTGCGATTCTAAAGTGT---AATAATAAGACGTTCAATGGAACAGGACCGTGTACAAATGTCAGCACAGTACAATGTACACATGGAATTAGGCCAGTAGTATCAACTCAACTGCTGTTAAATGGCAGTCTAGCAGAAAAA---GAGATAGTAATTAGATCTGAAAATATCACGGACAATGCTAAGACCATAATAGTACAGTTGAATACAGCTGTAGAAATTAATTGTACAAGACCCAGCAACAATACAAGAAAAAGTATAAGTATAGGA------------CCAGGGAGA---GCATTTTATACAACAGGAGAGGTAATAGGAGATATAAGACAAGCACATTGCAACATT------AGTCAAGCAAAATGGAATAACACTCTAAGACAGGTGGCTATTAAATTAAGA---GAACAATTTCCG------------AATAAAACA---ATAATCTTTAATCAA---------TCCTCAGGAGGGGACCTAGAAATTGAAATGCACAGTTTTAATTGTGGAGGGGAATTTTTCTACTGCAATACAACAATACTGTTTAATAGTACTTGG---------------AATGAAACTAACCTGTTT------------------------AATGCCACTGAAGGA---------------AATAACACAGAA------------------------GTTATTACACTCCAATGCAGAATAAAACAAATTATAAACATGTGGCAGGAAGTAGGAAAAGCAATGTATGCCCCTCCCATCAGTGGACAAATTAACTGTTCATCAAACATTACAGGGCTGCTATTAACAAGAGATGGCGGTATTGGGAAC---------AATATCACC---------------------AATACCTCC---GAGATCTTCAGACCTATAGGAGGAAATATGAGGGACAATTGGAGA---AGTGAATTATATAAATATAAAGTAGTCAAAATTGAACCA---ATAGGAATAGCACCC---ACCAAGGCAAAGAGAAGAGTGGTGCAGAGAGAA---AAAAGAGCAGTG---GGA---ATAGGA---GCTATG---TTCCTT---GGG---------TTCTTGGGA---GCAGCAGGAAGCACTATGGGCGCAGCGTCAGTG---ACGCTGACGGTACAGGCCAGACTATTATTGTCTGGTATAGTGCAACAGCAGAACAATCTGCTGAGAGCTATTGAGGCGCAACAACATCTGTTGCAACTCACAGTCTGGGGCATCAAGCAGCTCCAGGCAAGA---GTCCTGGCTGTGGAAAGATACCTAAAGGATCAACAGCTCCTGGGGATTTGGGGTTGCTCTGGAAAACTCATTTGCACCACTGCTGTGCCTTGGAATACTAGTTGGAGT---------------------------AATAAATCTCTGGAAAAAATTTGGAAT---AACATGACCTGGATGGAGTGGGAAAGAGAAATAGAC------AATTATACAAACTTAATATATAACTTACTTGAAGACTCGCAAAACCAACAAGACAAGAATGAAAAAGAATTATTGGAATTAGATAAATGGGCAAATTTGTGGAATTGGTTTGACATAACAAAGTGGCTGTGGTATATAAAAATATTCATAATGATAGTAGGAGGCTTAGTAGGTTTACGAATAGTTTTTTCTGTACTTTCTATAGTGAATAGAGTTAGGCAGGGATACTCACCACTATCGTTTCAGACCCAC---TCCCCAGCCCCGAGGGGA------CCCGACAGGCCCGGAGGAACAGAAGAAGAAGGTGGAGAAAGAGACAGAGACAGATCCGGTCCATTTGTGAACGGATTCTTGACGCTCATCTGGGTCGACCTGAGGAGCCTGTGCCTCTTCTGTTACCACCGCTTGAGAGACTTACTCTTGATTCTAGCGAGGATTGTAGAACTTCTGGGACGCAGG---------------GGGTGGGAAATCCTCAAATATTGGTGG---AATATCCTACAGTATTGG---------------------------------------------------AGTCAGGAACTAAAGAATAGCGCTGTCAGCTTGCTCAACGCCATAGCCATAGCAGTAGCTGAGGGGACAGATAGGATTATAGAAATAGTACAAAGA------------------GGTTTTAGA---------------------AGAATAAGACAGGGCTTGGAAAGGGCTGTGCTATAA

1.62995.SPD.EU575632 ATGAGAGCGAAGGGGATCAGGAAGAATTATCAGTACTTG---------TGGAGAGGGGGC------------------------ACCTTGCTCCTTGGGATATTGATGATC------------TGTAGTGCTGTA---------GAACAATTATGGGTCACAGTCTATTATGGGGTACCTGTGTGGAAAGAAGCAACCACCACTCTATTTTGTGCATCAGATGCTAAATCATATAGTACAGAGGTACATAAT---GTCTGGGCCACACATGCCTGTGTACCCACAGACCCTAGCCCACAAGAAGTAGTATTG---CAAAATGTGACAGAAAATTTTAACATGTGGAAAAATAACATGGTAGAACAAATGCATGAGGATATAATCAGTTTATGGGATCAAAGCCTAAAGCCATGTGTAAAATTAACCCCACTCTGTGTTACTCTAAATTGCTCTGATTTTAATGCTACTCAGGCCACTACTACTCAGGCC---------------------------------------------------------------------------------TATAATAGTAGCTGGAAGGTG------GAAGGAATGAAAAACTGCTCTTTCAATACCACC---TCAGGAATAAGAGAT------AAGGTGAAGAAAGAATATGCACTTCTTTATAAACTTGATATAGTAGAAATAAATGATGAT------------------------------AATAATACT---GGGGATAATACTAGCTATAGGTTCTATAGGTTGCTAAGTTGTAACACCTCGGTCATGACACAGGCCTGTCCAAAGGTATCCTTTGAGCCAATTCCCATACATTATTGTGCCCCAGCTGGTTTTGCGATTCTAAAGTGT---AATAATAAGACGTTCAATGGAACAGGACCGTGTACAAATGTCAGCACAGTACAATGTACACATGGAATTAGGCCAGTAGTATCAACTCAACTGCTGTTAAATGGCAGTCTAGCAGAAAAA---GAGATAGTAATTAGATCTGAAAATATCACGGACAATGCTAAGACCATAATAGTACAGTTGAATACAGCTGTAGAAATTAATTGTACAAGACCCAGCAACAATACAAGAAAAAGTATAAGTATAGGA------------CCAGGGAGA---GCATTTTATACAACAGGAGAGGTAATAGGAGATATAAGACAAGCACATTGCAACATT------AGTCAAGCAAAATGGAATAACACTCTAAGACAGGTGGCTATTAAATTAAGA---GAACAATTTCCG------------AATAAAACA---ATAATCTTTAATCAA---------TCCTCAGGAGGGGACCTAGAAATTGAAATGCACAGTTTTAATTGTGGAGGGGAATTTTTCTACTGCAATACAACAATACTGTTTAATAGTACTTGG---------------AATGAAACTAACCTGTTT------------------------AATGCCACTGAAGGA---------------AATAACACAGAA------------------------GTTATTACACTCCAATGCAGAATAAAACAAATTATAAACATGTGGCAGGAAGTAGGAAAAGCAATGTATGCCCCTCCCATCAGTGGACAAATTAACTGTTCATCAAACATTACAGGGCTGCTATTAACAAGAGATGGCGGTATTGGGAAC---------AATATCACC---------------------AATACCTCC---GAGATCTTCAGACCTATAGGAGGAAATATGAGGGACAATTGGAGA---AGTGAATTATATAAATATAAAGTAGTCAAAATTGAACCA---ATAGGAATAGCACCC---ACCAAGGCAAAGAGAAGAGTGGTGCAGAGAGAA---AAAAGAGCAGTG---GGA---ATAGGA---GCTATG---TTCCTT---GGG---------TTCTTGGGA---GCAGCAGGAAGCACTATGGGCGCAGCGTCAGTG---ACGCTGACGGTACAGGCCAGACTATTATTGTCTGGTATAGTGCAACAGCAGAACAATCTGCTGAGAGCTATTGAGGCGCAACAACATCTGTTGCAACTCACAGTCTGGGGCATCAAGCAGCTCCAGGCAAGA---GTCCTGGCTGTGGAAAGATACCTAAAGGATCAACAGCTCCTGGGGATTTGGGGTTGCTCTGGAAAACTCATTTGCACCACTGCTGTGCCTTGGAATACTAGTTGGAGT---------------------------AATAAATCTCTGGAAAAAATTTGGAAT---AACATGACCTGGATGGAGTGGGAAAGAGAAATAGAC------AATTATACAAACTTAATATATAACTTACTTGAAGACTCGCAAAACCAACAAGACAAGAATGAAAAAGAATTATTGGAATTAGATAAATGGGCAAATTTGTGGAATTGGTTTGACATAACAAAGTGGCTGTGGTATATAAAAATATTCATAATGATAGTAGGAGGCTTAGTAGGTTTACGAATAGTTTTTTCTGTACTTTCTATAGTGAATAGAGTTAGGCAGGGATACTCACCACTATCGTTTCAGACCCAC---TCCCCAGCCCCGAGGGGA------CCCGACAGGCCCGGAGGAACAGAAGAAGAAGGTGGAGAAAGAGACAGAGACAGATCCGGTCCATTTGTGAACGGATTCTTGACGCTCATCTGGGTCGACCTGAGGAGCCTGTGCCTCTTCTGTTACCACCGCTTGAGAGACTTACTCTTGATTCTAGCGAGGATTGTAGAACTTCTGGGACGCAGG---------------GGGTGGGAAATCCTCAAATATTGGTGG---AATATCCTACAGTATTGG---------------------------------------------------AGTCAGGAACTAAAGAATAGCGCTGTCAGCTTGCTCAACGCCATAGCCATAGCAGTAGCTGAGGGGACAGATAGGATTATAGAAATAGTACAAAGA------------------GGTTTTAGA---------------------AGAATAAGACAGGGCTTGGAAAGGGCTGTGCTATAA

1.62995.SPD.EU575643 ATGAGAGCGAAGGGGATCAGGAAGAATTATCAGTACTTG---------TGGAGAGGGGGC------------------------ACCTTGCTCCTTGGGATATTGATGATC------------TGTAGTGCTGTA---------GAACAATTATGGGTCACAGTCTATTATGGGGTACCTGTGTGGAAAGAAGCAACCACCACTCTATTTTGTGCATCAGATGCTAAATCATATAGTACAGAGGTACATAAT---GTCTGGGCCACACATGCCTGTGTACCCACAGACCCTAGCCCACAAGAAGTAGTATTG---CAAAATGTGACAGAAAATTTTAACATGTGGAAAAATAACATGGTAGAACAAATGCATGAGGATATAATCAGTTTATGGGATCAAAGCCTAAAGCCATGTGTAAAATTAACCCCACTCTGTGTTACTCTAAATTGCTCTGATTTTAATGCTACTCAGGCCACTACTACTCAGGCC---------------------------------------------------------------------------------TATAATAGTAGCTGGAAGGTG------GAAGGAATGAAAAACTGCTCTTTCAATACCACC---TCAGGAATAAGAGAT------AAGGTGAAGAAAGAATATGCACTTCTTTATAAACTTGATATAGTAGAAATAAATGATGAT------------------------------AATAATACT---GGGGATAATACTAGCTATAGGTTCTATAGGTTGCTAAGTTGTAACACCTCGGTCATGACACAGGCCTGTCCAAAGGTATCCTTTGAGCCAATTCCCATACATTATTGTGCCCCAGCTGGTTTTGCGATTCTAAAGTGT---AATAATAAGACGTTCAATGGAACAGGACCGTGTACAAATGTCAGCACAGTACAATGTACACATGGAATTAGGCCAGTAGTATCAACTCAACTGCTGTTAAATGGCAGTCTAGCAGAAAAA---GAGATAGTAATTAGATCTGAAAATATCACGGACAATGCTAAGACCATAATAGTACAGTTGAATACAGCTGTAGAAATTAATTGTACAAGACCCAGCAACAATACAAGAAAAAGTATAAGTATAGGA------------CCAGGGAGA---GCATTTTATACAACAGGAGAGGTAATAGGAGATATAAGACAAGCACATTGCAACATT------AGTCAAGCAAAATGGAATAACACTCTAAGACAGGTGGCTATTAAATTAAGA---GAACAATTTCCG------------AATAAAACA---ATAATCTTTAATCAA---------TCCTCAGGAGGGGACCTAGAAATTGAAATGCACAGTTTTAATTGTGGAGGGGAATTTTTCTACTGCAATACAACAATACTGTTTAATAGTACTTGG---------------AATGAAACTAACCTGTTT------------------------AATGCCACTGAAGGA---------------AATAACACAGAA------------------------GTTATTACACTCCAATGCAGAATAAAACAAATTATAAACATGTGGCAGGAAGTAGGAAAAGCAATGTATGCCCCTCCCATCAGTGGACAAATTAACTGTTCATCAAACATTACAGGGCTGCTATTAACAAGAGATGGCGGTATTGGGAAC---------AATATCACC---------------------AATACCTCC---GAGATCTTCAGACCTATAGGAGGAAATATGAGGGACAATTGGAGA---AGTGAATTATATAAATATAAAGTAGTCAAAATTGAACCA---ATAGGAATAGCACCC---ACCAAGGCAAAGAGAAGAGTGGTGCAGAGAGAA---AAAAGAGCAGTG---GGA---ATAGGA---GCTATG---TTCCTT---GGG---------TTCTTGGGA---GCAGCAGGAAGCACTATGGGCGCAGCGTCAGTG---ACGCTGACGGTACAGGCCAGACTATTATTGTCTGGTATAGTGCAACAGCAGAACAATCTGCTGAGAGCTATTGAGGCGCAACAACATCTGTTGCAACTCACAGTCTGGGGCATCAAGCAGCTCCAGGCAAGA---GTCCTGGCTGTGGAAAGATACCTAAAGGATCAACAGCCCCTGGGGATTTGGGGTTGCTCTGGAAAACTCATTTGCACCACTGCTGTGCCTTGGAATACTAGTTGGAGT---------------------------AATAAATCTCTGGAAAAAATTTGGAAT---AACATGACCTGGATGGAGTGGGAAAGAGAAATAGAC------AATTATACAAACTTAATATATAACTTACTTGAAGACTCGCAAAACCAACAAGACAAGAATGAAAAAGAATTATTGGAATTAGATAAATGGGCAAATTTGTGGAATTGGTTTGACATAACAAAGTGGCTGTGGTATATAAAAATATTCATAATGATAGTAGGAGGCTTAGTAGGTTTACGAATAGTTTTTTCTGTACTTTCTATAGTGAATAGAGTTAGGCAGGGATACTCACCACTATCGTTTCAGACCCAC---TCCCCAGCCCCGAGGGGA------CCCGACAGGCCCGGAGGAACAGAAGAAGAAGGTGGAGAAAGAGACAGAGACAGATCCGGTCCATTTGTGAACGGATTCTTGACGCTCATCTGGGTCGACCTGAGGAGCCTGTGCCTCTTCTGTTACCACCGCTTGAGAGACTTACTCTTGATTCTAGCGAGGATTGTAGAACTTCTGGGACGCAGG---------------GGGTGGGAAATCCTCAAATATTGGTGG---AATATCCTACAGTATTGG---------------------------------------------------AGTCAGGAACTAAAGAATAGCGCTGTCAGCTTGCTCAACGCCATAGCCATAGCAGTAGCTGAGGGGACAGATAGGATTATAGAAATAGTACAAAGA------------------GGTTTTAGA---------------------AGAATAAGACAGGGCTTGGAAAGGGCTGTGCTATAA

1.62995.SPD.EU575634 ATGAGAGCGAAGGGGATCAGGAAGAATTATCAGTACTTG---------TGGAGAGGGGGC------------------------ACCTTGCTCCTTGGGATATTGATGATC------------TGTAGTGCTGTA---------GAACAATTATGGGTCACAGTCTATTATGGGGTACCTGTGTGGAAAGAAGCAACCACCACTCTATTTTGTGCATCAGATGCTAAATCATATAGTACAGAGGTACATAAT---GTCTGGGCCACACATGCCTGTGTACCCACAGACCCTAGCCCACAAGAAGTAGTATTG---CAAAATGTGACAGAAAATTTTAACATGTGGAAAAATAACATGGTAGAACAAATGCATGAGGATATAATCAGTTTATGGGATCAAAGCCTAAAGCCATGTGTAAAATTAACCCCACTCTGTGTTACTCTAAATTGCTCTGATTTTAATGCTACTCAGGCCACTACTACTCAGGCC---------------------------------------------------------------------------------TATAATAGTAGCTGGAAGGTG------GAAGGAATGAAAAACTGCTCTTTCAATACCACC---TCAGGAATAAGAGAT------AAGGTGAAGAAAGAATATGCACTTCTTTATAAACTTGATATAGTAGAAATAAATGATGAT------------------------------AATAATACT---GGGGATAATACTAGCTATAGGTTCTATAGGTTGCTAAGTTGTAACACCTCGGTCATGACACAGGCCTGTCCAAAGGTATCCTTTGAGCCAATTCCCATACATTATTGTGCCCCAGCTGGTTTTGCGATTCTAAAGTGT---AATAATAAGACGTTCAATGGAACAGGACCGTGTACAAATGTCAGCACAGTACAATGTACACATGGAATTAGGCCAGTAGTATCAACTCAACTGCTGTTAAATGGCAGTCTAGCAGAAAAA---GAGATAGTAATTAGATCTGAAAATATCACGGACAATGCTAAGACCATAATAGTACAGTTGAATACAGCTGTAGAAATTAATTGTACAAGACCCAGCAACAATACAAGAAAAAGTATAAGTATAGGA------------CCAGGGAGA---GCATTTTATACAACAGGAGAGGTAATAGGAGATATAAGACAAGCACATTGCAACATT------AGTCAAGCAAAATGGAATAACACTCTAAGACAGGTGGCTATTAAATTAAGA---GAACAATTTCCG------------AATAAAACA---ATAATCTTTAATCAA---------TCCTCAGGAGGGGACCTAGAAATTGAAATGCACAGTTTTAATTGTGGAGGGGAATTTTTCTACTGCAATACAACAATACTGTTTAATAGTACTTGG---------------AATGAAACTAACCTGTTT------------------------AATGCCACTGAAGGA---------------AATAACACAGAA------------------------GTTATTACACTCCAATGCAGAATAAAACAAATTATAAACATGTGGCAGGAAGTAGGAAAAGCAATGTATGCCCCTCCCATCAGTGGACAAATTAACTGTTCATCAAACATTACAGGGCTGCTATTAACAAGAGATGGCGGTATTGGGAAC---------AATATCACC---------------------AATACCTCC---GAGATCTTCAGACCTATAGGAGGAAATATGAGGGACAATTGGAGA---AGTGAATTATATAAATATAAAGTAGTCAAAATTGAACCA---ATAGGAATAGCACCC---ACCAAGGCAAAGAGAAGAGTGGTGCAGAGAGAA---AAAAGAGCAGTG---GGA---ATAGGA---GCTATG---TTCCTT---GGG---------TTCTTGGGA---GCAGCAGGAAGCACTATGGGCGCAGCGTCAGTG---ACGCTGACGGTACAGGCCAAACTATTATTGTCTGGTATAGTGCAACAGCAGAACAATCTGCTGAGAGCTATTGAGGCGCAACAACATCTGTTGCAACTCACAGTCTGGGGCATCAAGCAGCTCCAGGCAAGA---GTCCTGGCTGTGGAAAGATACCTAAAGGATCAACAGCTCCTGGGGATTTGGGGTTGCTCTGGAAAACTCATTTGCACCACTGCTGTGCCTTGGAATACTAGTTGGAGT---------------------------AATAAATCTCTGGAAAAAATTTGGAAT---AACATGACCTGGATGGAGTGGGAAAGAGAAATAGAC------AATTATACAAACTTAATATATAACTTACTTGAAGACTCGCAAAACCAACAAGACAAGAATGAAAAAGAATTATTGGAATTAGATAAATGGGCAAATTTGTGGAATTGGTTTGACATAACAAAGTGGCTGTGGTATATAAAAATATTCATAATGATAGTAGGAGGCTTAGTAGGTTTACGAATAGTTTTTTCTGTACTTTCTATAGTGAATAGAGTTAGGCAGGGATACTCACCACTATCGTTTCAGACCCAC---TCCCCAGCCCCGAGGGGA------CCCGACAGGCCCGGAGGAACAGAAGAAGAAGGTGGAGAAAGAGACAGAGACAGATCCGGTCCATTTGTGAACGGATTCTTGACGCTCATCTGGGTCGACCTGAGGAGCCTGTGCCTCTTCTGTTACCACCGCTTGAGAGACTTACTCTTGATTCTAGCGAGGATTGTAGAACTTCTGGGACGCAGG---------------GGGTGGGAAATCCTCAAATATTGGTGG---AATATCCTACAGTATTGG---------------------------------------------------AGTCAGGAACTAAAGAATAGCGCTGTCAGCTTGCTCAACGCCATAGCCATAGCAGTAGCTGAGGGGACAGATAGGATTATAGAAATAGTACAAAGA------------------GGTTTTAGA---------------------AGAATAAGACAGGGCTTGGAAAGGGCTGTGCTATAA

1.62995.SPD.EU575636 ATGAGAGCGAAGGGGATCAGGAAGAATTATCAGTACTTG---------TGGAGAGGGGGC------------------------ACCTTGCTCCTTGGGATATTGATGATC------------TGTAGTGCTGTA---------GAACAATTATGGGTCACAGTCTATTATGGGGTACCTGTGTGGAAAGAAGCAACCACCACTCTATTTTGTGCATCAGATGCTAAATCATATAGTACAGAGGTACATAAT---GTCTGGGCCACACATGCCTGTGTACCCACAGACCCTAGCCCACAAGAAGTAGTATTG---CAAAATGTGACAGAAAATTTTAACATGTGGAAAAATAACATGGTAGAACAAATGCATGAGGATATAATCAGTTTATGGGATCAAAGCCTAAAGCCATGTGTAAAATTAACCCCACTCTGTGTTACTCTAAATTGCTCTGATTTTAATGCTACTCAGGCCACTACTACTCAGGCC---------------------------------------------------------------------------------TATAATAGTAGCTGGAAGGTG------GAAGGAATGAAAAACTGCTCTTTCAATACCACC---TCAGGAATAAGAGAT------AAGGTGAAGAAAGAATATGCACTTCTTTATAAACTTGATATAGTAGAAATAAATGATGAT------------------------------AATAATACT---GGGGATAATACTAGCTATAGGTTCTATAGGTTGCTAAGTTGTAACACCTCGGTCATGACACAGGCCTGTCCAAAGGTATCCTTTGAGCCAATTCCCATACATTATTGTGCCCCAGCTGGTTTTGCGATTCTAAAGTGT---AATAATAAGACGTTCAATGGAACAGGACCGTGTACAAATGTCAGCACAGTACAATGTACACATGGAATTAGGCCAGTAGTATCAACTCAACTGCTGTTAAATGGCAGTCTAGCAGAAAAA---GAGATAGTAATTAGATCTGAAAATATCACGGACAATGCTAAGACCATAATAGTACAGTTGAATACAGCTGTAGAAATTAATTGTACAAGACCCAGCAACAATACAAGAAAAAGTATAAGTATAGGA------------CCAGGGAGA---GCATTTTATACAACAGGAGAGGTAATAGGAGATATAAGACAAGCACATTGCAACATT------AGTCAAGCAAAATGGAATAACACTCTAAGACAGGTGGCTATTAAATTAAGA---GAACAATTTCCG------------AATAAAACA---ATAATCTTTAATCAA---------TCCTCAGGAGGGGACCTAGAAATTGAAATGCACAGTTTTAATTGTGGAGGGGAATTTTTCTACTGCAATACAACAATACTGTTTAATAGTACTTGG---------------AATGAAACTAACCTGTTT------------------------AATGCCACTGAAGGA---------------AATAACACAGAA------------------------GTTATTACACTCCAATGCAGAATAAAACAAATTATAAACATGTGGCAGGAAGTAGGAAAAGCAATGTATGCCCCTCCCATCAGTGGACAAATTAACTGTTCATCAAACATTACAGGGCTGCTATTAACAAGAGATGGCGGTATTGGGAAC---------AATATCACC---------------------AATACCTCC---GAGATCTTCAGACCTATAGGAGGAAATATGAGGGACAATTGGAGA---AGTGAATTATATAAATATAAAGTAGTCAAAATTGAACCA---ATAGGAATAGCACCC---ACCAAGGCAAAGAGAAGAGTGGTGCAGAGAGAA---AAAAGAGCAGTG---GGA---ATAGGA---GCTATG---TTCCTT---GGG---------TTCTTGGGA---GCAGCAGGAAGCACTATGGGCGCAGCGTCAGTG---ACGCTGACGGTACAGGCCAGACTATTATTGTCTGGTATAGTGCAACAGCAGAACAATCTGCTGAGAGCTATTGAGGCGCAACAACATCTGTTGCAACTCACAGTCTGGGGCATCAAGCAGCTCCAGGCAAGA---GTCCTGGCTGTGGAAAGATACCTAAAGGATCAACAGCTCCTGGGGATTTGGGGTTGCTCTGGAAAACTCATTTGCACCACTGCTGTGCCTTGGAATACTAGTTGGAGT---------------------------AATAAATCTCTGGAAAAAATTTGGAAT---AACATGACCTGGATGGAGTGGGAAAGAGAAATAGAC------AATTATACAAACTTAATATATAACTTACTTGAAGACTCGCAAAACCAACAAGACAAGAATGAAAAAGAATTATTGGAATTAGATAAATGGGCAAATTTGTGGAATTGGTTTGACATAACAAAGTGGCTGTGGTATATAAAAATATTCATAATGATAGTAGGAGGCTTAGTAGGTTTACGAATAGTTTTTTCTGTACTTTCTATAGTGAATAGAGTTAGGCAGGGATACTCACCACTATCGTTTCAGACCCAC---TCCCCAGCCCCGAGGGGA------CCCGACAGGCCCGGAGGAACAGAAGAAGAAGGTGGAGAAAGAGACAGAGACAGATCCGGTCCATTTGTGAACGGATTCTTGACGCTCATCTGGGTCGACCTGAGGAGCCTGTGCCTCTTCTGTTACCACCGCTTGAGAGACTTACTCTTGATTCTAGCGAGGATTGTAGAACTTCTGGGACGCAGG---------------GGGTGGGAAATCCTCAAATATTGGTGG---AATATCCTACAGTATTGG---------------------------------------------------AGTCAGGAACTAAAGAATAGCGCTGTCAGCTTGCTCAACGCCATAGCCATAGCAGTAGCTGAGGGGACAGATAGGATTATAGAAATAGTACAAAGA------------------GGTTTTAGA---------------------AGAATAAGACAGGGCTTGGAAAGGGCTGTGCTATAA

1.62995.SPD.EU575631 ATGAGAGCGAAGGGGATCAGGAAGAATTATCAGTACTTG---------TGGAGAGGGGGC------------------------ACCTTGCTCCTTGGGATATTGATGATC------------TGTAGTGCTGTA---------GAACAATTATGGGTCACAGTCTATTATGGGGTACCTGTGTGGAAAGAAGCAACCACCACTCTATTTTGTGCATCAGATGCTAAATCATATAGTACAGAGGTACATAAT---GTCTGGGCCACACATGCCTGTGTACCCACAGACCCTAGCCCACAAGAAGTAGTATTG---CAAAATGTGACAGAAAATTTTAACATGTGGAAAAATAACATGGTAGAACAAATGCATGAGGATATAATCAGTTTATGGGATCAAAGCCTAAAGCCATGTGTAAAATTAACCCCACTCTGTGTTACTCTAAATTGCTCTGATTTTAATGCTACTCAGGCCACTACTACTCAGGCC---------------------------------------------------------------------------------TATAATAGTAGCTGGAAGGTG------GAAGGAATGAAAAACTGCTCTTTCAATACCACC---TCAGGAATAAGAGAT------AAGGTGAAGAAAGAATATGCACTTCTTTATAAACTTGATATAATAGAAATAAATGATGAT------------------------------AATAATACT---GGGGATAATACTAGCTATAGGTTCTATAGGTTGCTAAGTTGTAACACCTCGGTCATGACACAGGCCTGTCCAAAGGTATCCTTTGAGCCAATTCCCATACATTATTGTGCCCCAGCTGGTTTTGCGATTCTAAAGTGT---AATAATAAGACGTTCAATGGAACAGGACCGTGTACAAATGTCAGCACAGTACAATGTACACATGGAATTAGGCCAGTAGTATCAACTCAACTGCTGTTAAATGGCAGTCTAGCAGAAAAA---GAGATAGTAATTAGATCTGAAAATATCACGGACAATGCTAAGACCATAATAGTACAGTTGAATACAGCTGTAGAAATTAATTGTACAAGACCCGGCAACAATACAAGAAAAAGTATAAGTATAGGA------------CCAGGGAGA---GCATTTTATACAACAGGAGAGGTAATAGGAGATATAAGACAAGCACATTGCAACATT------AGTCAAGCAAAATGGAATAACACTCTAAGACAGGTGGCTATTAAATTAAGA---GAACAATTTCCG------------AATAAAACA---ATAATCTTTAATCAA---------TCCTCAGGAGGGGACCTAGAAATTGAAATGCACAGTTTTAATTGTGGAGGGGAATTTTTCTACTGCAATACAACAATACTGTTTAATAGTACTTGG---------------AATGAAACTAACCTGTTT------------------------AATGCCACTGAAGGA---------------AATAACACAGAA------------------------GTTATTACACTCCAATGCAGAATAAAACAAATTATAAACATGTGGCAGGAAGTAGGAAAAGCAATGTATGCCCCTCCCATCAGTGGACAAATTAACTGTTCATCAAACATTACAGGGCTGCTATTAACAAGAGATGGCGGTATTGGGAAC---------AATATCACC---------------------AATACCTCC---GAGATCTTCAGACCTATAGGAGGAAATATGAGGGACAATTGGAGA---AGTGAATTATATAAATATAAAGTAGTCAAAATTGAACCA---ATAGGAATAGCACCC---ACCAAGGCAAAGAGAAGAGTGGTGCAGAGAGAA---AAAAGAGCAGTG---GGA---ATAGGA---GCTATG---TTCCTT---GGG---------TTCTTGGGA---GCAGCAGGAAGCACTATGGGCGCAGCGTCAGTG---ACGCTGACGGTACAGGCCAGACTATTATTGTCTGGTATAGTGCAACAGCAGAACAATCTGCTGAGAGCTATTGAGGCGCAACAACATCTGTTGCAACTCACAGTCTGGGGCATCAAGCAGCTCCAGGCAAGA---GTCCTGGCTGTGGAAAGATACCTAAAGGATCAACAGCTCCTGGGGATTTGGGGTTGCTCTGGAAAACTCATTTGCACCACTGCTGTGCCTTGGAATACTAGTTGGAGT---------------------------AATAAATCTCTGGAAAAAATTTGGAAT---AACATGACCTGGATGGAGTGGGAAAGAGAAATAGAC------AATTATACAAACTTAATATATAACTTACTTGAAGACTCGCAAAACCAACAAGACAAGAATGAAAAAGAATTATTGGAATTAGATAAATGGGCAAATTTGTGGAATTGGTTTGACATAACAAAGTGGCTGTGGTATATAAAAATATTCATAATGATAGTAGGAGGCTTAGTAGGTTTACGAATAGTTTTTTCTGTACTTTCTATAGTGAATAGAGTTAGGCAGGGATACTCACCACTATCGTTTCAGACCCAC---TCCCCAGCCCCGAGGGGA------CCCGACAGGCCCGGAGGAACAGAAGAAGAAGGTGGAGAAAGAGACAGAGACAGATCCGGTCCATTTGTGAACGGATTCTTGACGCTCATCTGGGTCGACCTGAGGAGCCTGTGCCTCTTCTGTTACCACCGCTTGAGAGACTTACTCTTGATTCTAGCGAGGATTGTAGAACTTCTGGGACGCAGG---------------GGGTGGGAAATCCTCAAATATTGGTGG---AATATCCTACAGTATTGG---------------------------------------------------AGTCAGGAACTAAAGAATAGCGCTGTCAGCTTGCTCAACGCCATAGCCATAGCAGTAGCTGAGGGGACAGATAGGATTATAGAAATAGTACAAAGA------------------GGTTTTAGA---------------------AGAATAAGACAGGGCTTGGAAAGGGCTGTGCTATAA

1.62995.SPD.EU575638 ATGAGAGCGAAGGGGATCAGGAAGAATTATCAGTACTTG---------TGGAGAGGGGGC------------------------ACCTTGCTCCTTGGGATATTGATGATC------------TGTAGTGCTGTA---------GAACAATTATGGGTCACAGTCTATTATGGGGTACCTGTGTGGAAAGAAGCAACCACCACTCTATTTTGTGCATCAGATGCTAAATCATATAGTACAGAGGTACATAAT---GTCTGGGCCACACATGCCTGTGTACCCACAGACCCTAGCCCACAAGAAGTAGTATTG---CAAAATGTGACAGAAAATTTTAACATGTGGAAAAATAACATGGTAGAACAAATGCATGAGGATATAATCAGTTTATGGGATCAAAGCCTAAAGCCATGTGTAAAATTAACCCCACTCTGTGTTACTCTAAATTGCTCTGATTTTAATGCTACTCAGGCCACTACTACTCAGGCC---------------------------------------------------------------------------------TATAATAGTAGCTGGAAGGTG------GAAGGAATGAAAAACTGCTCTTTCAATACCACC---TCAGGAATAAGAGAT------AAGGTGAAGAAAGAATATGCACTTCTTTATAAACTTGATATAGTAGAAATAAATGATGAT------------------------------AATAATACT---GGGGATAATACTAGCTATAGGTTCTATAGGTTGCTAAGTTGTAACACCTCGGTCATGACACAGGCCTGTCCAAAGGTATCCTTTGAGCCAATTCCCATACATTATTGTGCCCCAGCTGGTTTTGCGATTCTAAAGTGT---AATAATAAGACGTTCAATGGAACAGGACCGTGTACAAATGTCAGCACAGTACAATGTACACATGGAATTAGGCCAGTAGTATCAACTCAACTGCTGTTAAATGGCAGTCTAGCAGAAAAA---GAGATAGTAATTAGATCTGAAAATATCACGGACAATGCTAAGACCATAATAGTACAGTTGAATACAGCTGTAGAAATTAATTGTACAAGACCCAGCAACAATACAAGAAAAAGTATAAGTATAGGA------------CCAGGGAGA---GCATTTTATACAACAGGAGAGGTAATAGGAGATATAAGACAAGCACATTGCAACATT------AGTCAAGCAAAATGGAATAACACTCTAAGACAGGTGGCTATTAAATTAAGA---GAACAATTTCCG------------AATAAAACA---ATAATCTTTAATCAA---------TCCTCAGGAGGGGACCTAGAAATTGAAATGCACAGTTTTAATTGTGGAGGGGAATTTTTCTACTGCAATACAACAATACTGTTTAATAGTACTTGG---------------AATGAAACTAACCTGTTT------------------------AATGCCACTGAAGGA---------------AATAACACAGAA------------------------GTTATTACACTCCAATGCAGAATAAAACAAATTATAAACATGTGGCAGGAAGTAGGAAAAGCAATGTATGCCCCTCCCATCAGTGGACAAATTAACTGTTCATCAAACATTACAGGGCTGCTATTAACAAGAGATGGCGGTATTGGGAAC---------AATATCACC---------------------AATACCTCC---GAGATCTTCAGACCTATAGGAGGAAATATGAGGGACAATTGGAGA---AGTGAATTATATAAATATAAAGTAGTCAAAATTGAACCA---ATAGGAATAGCACCC---ACCAAGGCAAAGAGAAGAGTGGTGCAGAGAGAA---AAAAGAGCAGTG---GGA---ATAGGA---GCTATG---TTCCTT---GGG---------TTCTTGGGA---GCAGCAGGAAGCACTATGGGCGCAGCGTCAGTG---ACGCTGACGGTACAGGCCAGACTATTATTGTCTGGTATAGTGCAACAGCAGAACAATCTGCTGAGAGCTATTGAGGCGCAACAACATCTGTTGCAACTCACAGTCTGGGGCATCAAGCAGCTCCAGGCAAGA---GTCCTGGCTGTGGAAAGATACCTAAAGGATCAACAGCTCCTGGGGATTTGGGGTTGCTCTGGAAAACTCATTTGCACCACTGCTGTGCCTTGGAATACTAGTTGGAGT---------------------------AATAAATCTCTGGAAAAAATTTGGAAT---AACATGACCTGGATGGAGTGGGAAAGAGAAATAGAC------AATTATACAAACTTAATATATAACTTACTTGAAGACTCGCAAAACCAACAAGACAAGAATGAAAAAGAATTATTGGAATTAGATAAATGGGCAAATTTGTGGAATTGGTTTGACATAACAAAGTGGCTGTGGTATATAAAAATATTCATAATGATAGTAGGAGGCTTAGTAGGTTTACGAATAGTTTTTTCTGTACTTTCTATAGTGAATAGAGTTAGGCAGGGATACTCACCACTATCGTTTCAGACCCAC---TCCCCAGCCCCGAGGGGA------CCCGACAGGCCCGGAGGAACAGAAGAAGAAGGTGGAGAAAGAGACAGAGACAGATCCGGTCCATTTGTGAACGGATTCTTGACGCTCATCTGGGTCGACCTGAGGAGCCTGTGCCTCTTCTGTTACCACCGCTTGAGAGACTTACTCTTGATTCTAGCGAGGATTGTAGAACTTCTGGGACGCAGG---------------GGGTGGGAAATCCTCAAATATTGGTGG---AATATCCTACAGTATTGG---------------------------------------------------AGTCAGGAACTAAAGAATAGCGCTGTCAGCTTGCTCAACGCCATAGCCATAGCAGTAGCTGAGGGGACAGATAGGATTATAGAAATAGTACAAAGA------------------GGTTTTAGA---------------------AGAATAAGACAGGGCTTGGAAAGGGCTGTGCTATAA

1.62995.SPD.EU575635 ATGAGAGCGAAGGGGATCAGGAAGAATTATCAGTACTTG---------TGGAGAGGGGGC------------------------ACCTTGCTCCTTGGGATATTGATGATC------------TGTAGTGCTGTA---------GAACAATTATGGGTCACAGTCTATTATGGGGTACCTGTGTGGAAAGAAGCAACCACCACTCTATTTTGTGCATCAGATGCTAAATCATATAGTACAGAGGTACATAAT---GTCTGGGCCACACATGCCTGTGTACCCACAGACCCTAGCCCACAAGAAGTAGTATTG---CAAAATGTGACAGAAAATTTTAACATGTGGAAAAATAACATGGTAGAACAAATGCATGAGGATATAATCAGTTTATGGGATCAAAGCCTAAAGCCATGTGTAAAATTAACCCCACTCTGTGTTACTCTAAATTGCTCTGATTTTAATGCTACTCAGGCCACTACTACTCAGGCC---------------------------------------------------------------------------------TATAATAGTAGCTGGAAGGTG------GAAGGAATGAAAAACTGCTCTTTCAATACCACC---TCAGGAATAAGAGAT------AAGGTGAAGAAAGAATATGCACTTCTTTATAAACTTGATATAGTAGAAATAAATGATGAT------------------------------AATAATACT---GGGGATAATACTAGCTATAGGTTCTATAGGTTGCTAAGTTGTAACACCTCGGTCATGACACAGGCCTGTCCAAAGGTATCCTTTGAGCCAATTCCCATACATTATTGTGCCCCAGCTGGTTTTGCGATTCTAAAGTGT---AATAATAAGACGTTCAATGGAACAGGACCGTGTACAAATGTCAGCACAGTACAATGTACACATGGAATTAGGCCAGTAGTATCAACTCAACTGCTGTTAAATGGCAGTCTAGCAGAAAAA---GAGATAGTAATTAGATCTGAAAATATCACGGACAATGCTAAGACCATAATAGTACAGTTGAATACAGCTGTAGAAATTAATTGTACAAGACCCAGCAACAATACAAGAAAAAGTATAAGTATAGGA------------CCAGGGAGA---GCATTTTATACAACAGGAGAGGTAATAGGAGATATAAGACAAGCACATTGCAACATT------AGTCAAGCAAAATGGAATAACACTCTAAGACAGGTGGCTATTAAATTAAGA---GAACAATTTCCG------------AATAAAACA---ATAATCTTTAATCAA---------TCCTCAGGAGGGGACCTAGAAATTGAAATGCACAGTTTTAATTGTGGAGGGGAATTTTTCTACTGCAATACAACAATACTGTTTAATAGTACTTGG---------------AATGAAACTAACCTGTTT------------------------AATGCCACTGAAGGA---------------AATAACACAGAA------------------------GTTATTACACTCCAATGCAGAATAAAACAAATTATAAACATGTGGCAGGAAGTAGGAAAAGCAATGTATGCCCCTCCCATCAGTGGACAAATTAACTGTTCATCAAACATTACAGGGCTGCTATTAACAAGAGATGGCGGTATTGGGAAC---------AATATCACC---------------------AATACCTCC---GAGATCTTCAGACCTATAGGAGGAAATATGAGGGACAATTGGAGA---AGTGAATTATATAAATATAAAGTAGTCAAAATTGAACCA---ATAGGAATAGCACCC---ACCAAGGCAAAGAGAAGAGTGGTGCAGAGAGAA---AAAAGAGCAGTG---GGA---ATAGGA---GCTATG---TTCCTT---GGG---------TTCTTGGGA---GCAGCAGGAAGCACTATGGGCGCAGCGTCAGTG---ACGCTGACGGTACAGGCCAGACTATTATTGTCTGGTATAGTGCAACAGCAGAACAATCTGCTGAGAGCTATTGAGGCGCAACAACATCTGTTGCAACTCACAGTCTGGGGCATCAAGCAGCTCCAGGCAAGA---GTCCTGGCTGTGGAAAGATACCTAAAGGATCAACAGCTCCTGGGGATTTGGGGTTGCTCTGGAAAACTCATTTGCACCACTGCTGTGCCTTGGAATACTAGTTGGAGT---------------------------AATAAATCTCTGGAAAAAATTTGGAAT---AACATGACCTGGATGGAGTGGGAAAGAGAAATAGAC------AATTATACAAACTTAATATATAACTTACTTGAAGACTCGCAAAACCAACAAGACAAGAATGAAAAAGAATTATTGGAATTAGATAAATGGGCAAATTTGTGGAATTGGTTTGACATAACAAAGTGGCTGTGGTATATAAAAATATTCATAATGATAGTAGGAGGCTTAGTAGGTTTACGAATAGTTTTTTCTGTACTTTCTATAGTGAATAGAGTTAGGCAGGGATACTCACCACTATCGTTTCAGACCCAC---TCCCCAGCCCCGAGGGGA------CCCGACAGGCCCGGAGGAACAGAAGAAGAAGGTGGAGAAAGAGACAGAGACAGATCCGGTCCATTTGTGAACGGATTCTTGACGCTCATCTGGGTCGACCTGAGGAGCCTGTGCCTCTTCTGTTACCACCGCTTGAGAGACTTACTCTTGATTCTAGCGAGGATTGTAGAACTTCTGGGACGCAGG---------------GGGTGGGAAATCCTCAAATATTGGTGG---AATATCCTACAGTATTGG---------------------------------------------------AGTCAGGAACTAAAGAATAGCGCTGTCAGCTTGCTCAACGCCATAGCCATAGCAGTAGCTGAGGGGACAGATAGGATTATAGAAATAGTACAAAGA------------------GGTTTTAGA---------------------AGAATAAGACAGGGCTTGGAAAGGGCTGTGCTATAA

1.62995.SPD.EU575622 ATGAGAGCGAAGGGGATCAGGAAGAATTATCAGTACTTG---------TGGAGAGGGGGC------------------------ACCTTGCTCCTTGGGATATTGATGATC------------TGTAGTGCTGTA---------GAACAATTATGGGTCACAGTCTATTATGGGGTACCTGTGTGGAAAGAAGCAACCACCACTCTATTTTGTGCATCAGATGCTAAATCATATAGTACAGAGGTACATAAT---GTCTGGGCCACACATGCCTGTGTACCCACAGACCCTAGCCCACAAGAAGTAGTATTG---CAAAATGTGACAGAAAATTTTAACATGTGGAAAAATAACATGGTAGAACAAATGCATGAGGATATAATCAGTTTATGGGATCAAAGCCTAAAGCCATGTGTAAAATTAACCCCACTCTGTGTTACTCTAAATTGCTCTGATTTTAATGCTACTCAGGCCACTACTACTCAGGCC---------------------------------------------------------------------------------TATAATAGTAGCTGGAAGGTG------GAAGGAATGAAAAACTGCTCTTTCAATACCACC---TCAGGAATAAGAGAT------AAGGTGAAGAAAGAATATGCACTTCTTTATAAACTTGATATAGTAGAAATAAATGATGAT------------------------------AATAATACT---GGGGATAATACTAGCTATAGGTTCTATAGGTTGCTAAGTTGTAACACCTCGGTCATGACACAGGCCTGTCCAAAGGTATCCTTTGAGCCAATTCCCATACATTATTGTGCCCCAGCTGGTTTTGCGATTCTAAAGTGT---AATAATAAGACGTTCAATGGAACAGGACCGTGTACAAATGTCAGCACAGTACAATGTACACATGGAATTAGGCCAGTAGTATCAACTCAACTGCTGTTAAATGGCAGTCTAGCAGAAAAA---GAGATAGTAATTAGATCTGAAAATATCACGGACAATGCTAAGACCATAATAGTACAGTTGAATACAGCTGTAGAAATTAATTGTACAAGACCCAGCAACAATACAAGAAAAAGTATAAGTATAGGA------------CCAGGGAGA---GCATTTTATACAACAGGAGAGGTAATAGGAGATATAAGACAAGCACATTGCAACATT------AGTCAAGCAAAATGGAATAACACTCTAAGACAGGTGGCTATTAAATTAAGA---GAACAATTTCCG------------AATAAAACA---ATAATCTTTAATCAA---------TCCTCAGGAGGGGACCTAGAAATTGAAATGCACAGTTTTAATTGTGGAGGGGAATTTTTCTACTGCAATACAACAATACTGTTTAATAGTACTTGG---------------AATGAAACTAACCTGTTT------------------------AATGCCACTGAAGGA---------------AATAACACAGAA------------------------GTTATTACACTCCAATGCAGAATAAAACAAATTATAAACATGTGGCAGGAAGTAGGAAAAGCAATGTATGCCCCTCCCATCAGTGGACAAATTAACTGTTCATCAAACATTACAGGGCTGCTATTAACAAGAGATGGCGGTATTGGGAAC---------AATATCACC---------------------AATACCTCC---GAGATCTTCAGACCTATAGGAGGAAATATGAGGGACAATTGGAGA---AGTGAATTATATAAATATAAAGTAGTCAAAATTGAACCA---ATAGGAATAGCACCC---ACCAAGGCAAAGAGAAGAGTGGTGCAGAGAGAA---AAAAGAGCAGTG---GGA---ATAGGA---GCTATG---TTCCTT---GGG---------TTCTTGGGA---GCAGCAGGAAGCACTATGGGCGCAGCGTCAGTG---ACGCTGACGGTACAGGCCAGACTATTATTGTCTGGTATAGTGCAACAGCAGAACAATCTGCTGAGAGCTATTGAGGCGCAACAACATCTGTTGCAACTCACAGTCTGGGGCATCAAGCAGCTCCAGGCAAGA---GTCCTGGCTGTGGAAAGATACCTAAAGGATCAACAGCTCCTGGGGATTTGGGGTTGCTCTGGAAAACTCATTTGCACCACTGCTGTGCCTTGGAATACTAGTTGGAGT---------------------------AATAAATCTCTGGAAAAAATTTGGAAT---AACATGACCTGGATGGAGTGGGAAAGAGAAATAGAC------AATTATACAAACTTAATATATAACTTACTTGAAGACTCGCAAAACCAACAAGACAAGAATGAAAAAGAATTATTGGAATTAGATAAATGGGCAAATTTGTGGAATTGGTTTGACATAACAAAGTGGCTGTGGTATATAAAAATATTCATAATGATAGTAGGAGGCTTAGTAGGTTTACGAATAGTTTTTTCTGTACTTTCTATAGTGAATAGAGTTAGGCAGGGATACTCACCACTATCGTTTCAGACCCAC---TCCCCAGCCCCGAGGGGA------CCCGACAGGCCCGGAGGAACAGAAGAAGAAGGTGGAGAAAGAGACAGAGACAGATCCGGTCCATTTGTGAACGGATTCTTGACGCTCATCTGGGTCGACCTGAGGAGCCTGTGCCTCTTCTGTTACCACCGCTTGAGAGACTTACTCTTGATTCTAGCGAGGATTGTAGAACTTCTGGGACGCAGG---------------GGGTGGGAAATCCTCAAATATTGGTGG---AATATCCTACAGTATTGG---------------------------------------------------AGTCAGGAACTAAAGAATAGCGCTGTCAGCTTGCTCAACGCCATAGCCATAGCAGTAGCTGAGGGGACAGATAGGATTATAGAAATAGTACAAAGA------------------GGTTTTAGA---------------------AGAATAAGACAGGGCTTGGAAAGGGCTGTGCTATAA

1.62995.SPD.EU575642 ATGAGAGCGAAGGGGATCAGGAAGAATTATCAGTACTTG---------TGGAGAGGGGGC------------------------ACCTTGCTCCTTGGGATATTGATGATC------------TGTAGTGCTGTA---------GAACAATTATGGGTCACAGTCTATTATGGGGTACCTGTGTGGAAAGAAGCAACCACCACTCTATTTTGTGCATCAGATGCTAAATCATATAGTACAGAGGTACATAAT---GTCTGGGCCACACATGCCTGTGTACCCACAGACCCTAGCCCACAAGAAGTAGTATTG---CAAAATGTGACAGAAAATTTTAACATGTGGAAAAATAACATGGTAGAACAAATGCATGAGGATATAATCAGTTTATGGGATCAAAGCCTAAAGCCATGTGTAAAATTAACCCCACTCTGTGTTACTCTAAATTGCTCTGATTTTAATGCTACTCAGGCCACTACTACTCAGGCC---------------------------------------------------------------------------------TATAATAGTAGCTGGAAGGTG------GAGGGAATGAAAAACTGCTCTTTCAATACCACC---TCAGGAATAAGAGAT------AAGGTGAAGAAAGAATATGCACTTCTTTATAAACTTGATATAGTAGAAATAAATGATGAT------------------------------AATAATACT---GGGGATAATACTAGCTATAGGTTCTATAGGTTGCTAAGTTGTAACACCTCGGTCATGACACAGGCCTGTCCAAAGGTATCCTTTGAGCCAATTCCCATACATTATTGTGCCCCAGCTGGTTTTGCGATTCTAAAGTGT---AATAATAAGACGTTCAATGGAACAGGACCGTGTACAAATGTCAGCACAGTACAATGTACACATGGAATTAGGCCAGTAGTATCAACTCAACTGCTGTTAAATGGCAGTCTAGCAGAAAAA---GAGATAGTAATTAGATCTGAAAATATCACGGACAATGCTAAGACCATAATAGTACAGTTGAATACAGCTGTAGAAATTAATTGTACAAGACCCAGCAACAATACAAGAAAAAGTATAAGTATAGGA------------CCAGGGAGA---GCATTTTATACAACAGGAGAGGTAATAGGAGATATAAGACAAGCACATTGCAACATT------AGTCAAGCAAAATGGAATAACACTCTAAGACAGGTGGCTATTAAATTAAGA---GAACAATTTCCG------------AATAAAACA---ATAATCTTTAATCAA---------TCCTCAGGAGGGGACCTAGAAATTGAAATGCACAGTTTTAATTGTGGAGGGGAATTTTTCTACTGCAATACAACAATACTGTTTAATAGTACTTGG---------------AATGAAACTAACCTGTTT------------------------AATGCCACTGAAGGA---------------AATAACACAGAA------------------------GTTATTACACTCCAATGCAGAATAAAACAAATTATAAACATGTGGCAGGAAGTAGGAAAAGCAATGTATGCCCCTCCCATCAGTGGACAAATTAACTGTTCATCAAACATTACAGGGCTGCTATTAACAAGAGATGGCGGTATTGGGAAC---------AATATCACC---------------------AATACCTCC---GAGATCTTCAGACCTATAGGAGGAAATATGAGGGACAATTGGAGA---AGTGAATTATATAAATATAAAGTAGTCAAAATTGAACCA---ATAGGAATAGCACCC---ACCAAGGCAAAGAGAAGAGTGGTGCAGAGAGAA---AAAAGAGCAGTG---GGA---ATAGGA---GCTATG---TTCCTT---GGG---------TTCTTGGGA---GCAGCAGGAAGCACTATGGGCGCAGCGTCAGTG---ACGCTGACGGTACAGGCCAGACTATTATTGTCTGGTATAGTGCAACAGCAGAACAATCTGCTGAGAGCTATTGAGGCGCAACAACATCTGTTGCAACTCACAGTCTGGGGCATCAAGCAGCTCCAGGCAAGA---GTCCTGGCTGTGGAAAGATACCTAAAGGATCAACAGCTCCTGGGGATTTGGGGTTGCTCTGGAAAACTCATTTGCACCACTGCTGTGCCTTGGAATACTAGTTGGAGT---------------------------AATAAATCTCTGGAAAAAATTTGGAAT---AACATGACCTGGATGGAGTGGGAAAGAGAAATAGAC------AATTATACAAACTTAATATATAACTTACTTGAAGACTCGCAAAACCAACAAGACAAGAATGAAAAAGAATTATTGGAATTAGATAAATGGGCAAATTTGTGGAATTGGTTTGACATAACAAAGTGGCTGTGGTATATAAAAATATTCATAATGATAGTAGGAGGCTTAGTAGGTTTACGAATAGTTTTTTCTGTACTTTCTATAGTGAATAGAGTTAGGCAGGGATACTCACCACTATCGTTTCAGACCCAC---TCCCCAGCCCCGAGGGGA------CCCGACAGGCCCGGAGGAACAGAAGAAGAAGGTGGAGAAAGAGACAGAGACAGATCCGGTCCATTTGTGAACGGATTCTTGACGCTCATCTGGGTCGACCTGAGGAGCCTGTGCCTCTTCTGTTACCACCGCTTGAGAGACTTACTCTTGATTCTAGCGAGGATTGTAGAACTTCTGGGACGCAGG---------------GGGTGGGAAATCCTCAAATATTGGTGG---AATATCCTACAGTATTGG---------------------------------------------------AGTCAGGAACTAAAGAATAGCGCTGTCAGCTTGCTCAACGCCATAGCCATAGCAGTAGCTGAGGGGACAGATAGGATTATAGAAATAGTACAAAGA------------------GGTTTTAGA---------------------AGAATAAGACAGGGCTTGGAAAGGGCTGTGCTATAA

1.62995.SPD.EU575646 ATGAGAGCGAAGGGGATCAGGAAGAATTATCAGTACTTG---------TGGAGAGGGGGC------------------------ACCTTGCTCCTTGGGATATTGATGATC------------TGTAGTGCTGTA---------GAACAATTATGGGTCACAGTCTATTATGGGGTACCTGTGTGGAAAGAAGCAACCACCACTCTATTTTGTGCATCAGATGCTAAATCATATAGTACAGAGGTACATAAT---GTCTGGGCCACACATGCCTGTGTACCCACAGACCCTAGCCCACAAGAAGTAGTATTG---CAAAATGTGACAGAAAATTTTAACATGTGGAAAAATAACATGGTAGAACAAATGCATGAGGATATAATCAGTTTATGGGATCAAAGCCTAAAGCCATGTGTAAAATTAACCCCACTCTGTGTTACTCTAAATTGCTCTGATTTTAATGCTACTCAGGCCACTACTACTCAGGCC---------------------------------------------------------------------------------TATAATAGTAGCTGGAAGGTG------GAAGGAATGAAAAACTGCTCTTTCAATACCACC---TCAGGAATAAGAGAT------AAGGTGAAGAAAGAATATGCACTTCTTTATAAACTTGATATAGTAGAAATAAATGATGAT------------------------------AATAATACT---GGGGATAATACTAGCTATAGGTTCTATAGGTTGCTAAGTTGTAACACCTCGGTCATGACACAGGCCTGTCCAAAGGTATCCTTTGAGCCAATTCCCATACATTATTGTGCCCCAGCTGGTTTTGCGATTCTAAAGTGT---AATAATAAGACGTTCAATGGAACAGGACCGTGTACAAATGTCAGCACAGTACAATGTACACATGGAATTAGGCCAGTAGTATCAACTCAACTGCTGTTAAATGGCAGTCTAGCAGAAAAA---GAGATAGTAATTAGATCTGAAAATATCACGGACAATGCTAAGACCATAATAGTACAGTTGAATACAGCTGTAGAAATTAATTGTACAAGACCCAGCAACAATACAAGAAAAAGTATAAGTATAGGA------------CCAGGGAGA---GCATTTTATACAACAGGAGAGGTAATAGGAGATATAAGACAAGCACATTGCAACATT------AGTCAAGCAAAATGGAATAACACTCTAAGACAGGTGGCTATTAAATTAAGA---GAACAATTTCCG------------AATAAAACA---ATAATCTTTAATCAA---------TCCTCAGGAGGGGACCTAGAAATTGAAATGCACAGTTTTAATTGTGGAGGGGAATTTTTCTACTGCAATACAACAATACTGTTTAATAGTACTTGG---------------AATGAAACTAACCTGTTT------------------------AATGCCACTGAAGGA---------------AATAACACAGAA------------------------GTTATTACACTCCAATGCAGAATAAAACAAATTATAAACATGTGGCAGGAAGTAGGAAAAGCAATGTATGCCCCTCCCATCAGTGGACAAATTAACTGTTCATCAAACATTACAGGGCTGCTATTAACAAGAGATGGCGGTATTGGGAAC---------AATATCACC---------------------AATACCTCC---GAGATCTTCAGACCTATAGGAGGAAATATGAGGGACAATTGGAGA---AGTGAATTATATAAATATAAAGTAGTCAAAATTGAACCA---ATAGGAATAGCACCC---ACCAAGGCAAAGAGAAGAGTGGTGCAGAGAGAA---AAAAGAGCAGTG---GGA---ATAGGA---GCTATG---TTCCTT---GGG---------TTCTTGGGA---GCAGCAGGAAGCACTATGGGCGCAGCGTCAGTG---ACGCTGACGGTACAGGCCAGACTATTATTGTCTGGTATAGTGCAACAGCAGAACAATCTGCTGAGAGCTATTGAGGCGCAACAACATCTGTTGCAACTCACAGTCTGGGGCATCAAGCAGCTCCAGGCAAGA---GTCCTGGCTGTGGAAAGATACCTAAAGGATCAACAGCTCCTGGGGATTTGGGGTTGCTCTGGAAAACTCATTTGCACCACTGCTGTGCCTTGGAATACTAGTTGGAGT---------------------------AATAAATCTCTGGAAAAAATTTGGAAT---AACATGACCTGGATGGAGTGGGAAAGAGAAATAGAC------AATTATACAAACTTAATATATAACTTACTTGAAGACTCGCAAAACCAACAAGACAAGAATGAAAAAGAATTATTGGAATTAGATAAATGGGCAAATTTGTGGAATTGGTTTGACATAACAAAGTGGCTGTGGTATATAAAAATATTCATAATGATAGTAGGAGGCTTAGTAGGTTTACGAATAGTTTTTTCTGTACTTTCTATAGTGAATAGAGTTAGGCAGGGATACTCACCACTATCGTTTCAGACCCAC---TCCCCAGCCCCGAGGGGA------CCCGACAGGCCCGGAGGAACAGAAGAAGAAGGTGGAGAAAGAGACAGAGACAGATCCGGTCCATTTGTGAACGGATTCTTGACGCTCATCTGGGTCGACCTGAGGAGCCTGTGCCTCTTCTGTTACCACCGCTTGAGAGACTTACTCTTGATTCTAGCGAGGATTGTAGAACTTCTGGGACGCAGG---------------GGGTGGGAAATCCTCAAATATTGGTGG---AATATCCTACAGTATTGG---------------------------------------------------AGTCAGGAACTAAAGAATAGCGCTGTCAGCTTGCTCAACGCCATAGCCATAGCAGTAGCTGAGGGGACAGATAGGATTATAGAAATAGTACAAAGA------------------GGTTTTAGA---------------------AGAATAAGACAGGGCTTGGAAAGGGCTGTGCTATAA

1.62995.SPD.EU575626 ATGAGAGCGAAGGGGATCAGGAAGAATTATCAGTACTTG---------TGGAGAGGGGGC------------------------ACCTTGCTCCTTGGGATATTGATGATC------------TGTAGTGCTGTA---------GAACAATTATGGGTCACAGTCTATTATGGGGTACCTGTGTGGAAAGAAGCAACCACCACTCTATTTTGTGCATCAGATGCTAAATCATATAGTACAGAGGTATATAAT---GTCTGGGCCACACATGCCTGTGTACCCACAGACCCTAGCCCACAAGAAGTAGTATTG---CAAAATGTGACAGAAAATTTTAACATGTGGAAAAATAACATGGTAGAACAAATGCATGAGGATATAATCAGTTTATGGGATCAAAGCCTAAAGCCATGTGTAAAATTAACCCCACTCTGTGTTACTCTAAATTGCTCTGATTTTAATGCTACTCAGGCCACTACTACTCAGGCC---------------------------------------------------------------------------------TATAATAGTAGCTGGAAGGTG------GAAGGAATGAAAAACTGCTCTTTCAATACCACC---TCAGGAATAAGAGAT------AAGGTGAAGAAAGAATATGCACTTCTTTATAAACTTGATATAGTAGAAATAAATGATGAT------------------------------AATAATACT---GGGGATAATACTAGCTATAGGTTCTATAGGTTGCTAAGTTGTAACACCTCGGTCATGACACAGGCCTGTCCAAAGGTATCCTTTGAGCCAATTCCCATACATTATTGTGCCCCAGCTGGTTTTGCGATTCTAAAGTGT---AATAATAAGACGTTCAATGGAACAGGACCGTGTACAAATGTCAGCACAGTACAATGTACACATGGAATTAGGCCAGTAGTATCAACTCAACTGCTGTTAAATGGCAGTCTAGCAGAAAAA---GAGATAGTAATTAGATCTGAAAATATCACGGACAATGCTAAGACCATAATAGTACAGTTGAATACAGCTGTAGAAATTAATTGTACAAGACCCAGCAACAATACAAGAAAAAGTATAAGTATAGGA------------CCAGGGAGA---GCATTTTATACAACAGGAGAGGTAATAGGAGATATAAGACAAGCACATTGCAACATT------AGTCAAGCAAAATGGAATAACACTCTAAGACAGGTGGCTATTAAATTAAGA---GAACAATTTCCG------------AATAAAACA---ATAATCTTTAATCAA---------TCCTCAGGAGGGGACCTAGAAATTGAAATGCACAGTTTTAATTGTGGAGGGGAATTTTTCTACTGCAATACAACAATACTGTTTAATAGTACTTGG---------------AATGAAACTAACCTGTTT------------------------AATGCCACTGAAGGA---------------AATAACACAGAA------------------------GTTATTACACTCCAATGCAGAATAAAACAAATTATAAACATGTGGCAGGAAGTAGGAAAAGCAATGTATGCCCCTCCCATCAGTGGACAAATTAACTGTTCATCAAACATTACAGGGCTGCTATTAACAAGAGATGGCGGTATTGGGAAC---------AATATCACC---------------------AATACCTCC---GAGATCTTCAGACCTATAGGAGGAAATATGAGGGACAATTGGAGA---AGTGAATTATATAAATATAAAGTAGTCAAAATTGAACCA---ATAGGAATAGCACCC---ACCAAGGCAAAGAGAAGAGTGGTGCAGAGAGAA---AAAAGAGCAGTG---GGA---ATAGGA---GCTATG---TTCCTT---GGG---------TTCTTGGGA---GCAGCAGGAAGCACTATGGGCGCAGCGTCAGTG---ACGCTGACGGTACAGGCCAGACTATTATTGTCTGGTATAGTGCAACAGCAGAACAATCTGCTGAGAGCTATTGAGGCGCAACAACATCTGTTGCAACTCACAGTCTGGGGCATCAAGCAGCTCCAGGCAAGA---GTCCTGGCTGTGGAAAGATACCTAAAGGATCAACAGCTCCTGGGGATTTGGGGTTGCTCTGGAAAACTCATTTGCACCACTGCTGTGCCTTGGAATACTAGTTGGAGT---------------------------AATAAATCTCTGGAAAAAATTTGGAAT---AACATGACCTGGATGGAGTGGGAAAGAGAAATAGAC------AATTATACAAACTTAATATATAACTTACTTGAAGACTCGCAAAACCAACAAGACAAGAATGAAAAAGAATTATTGGAATTAGATAAATGGGCAAATTTGTGGAATTGGTTTGACATAACAAAGTGGCTGTGGTATATAAAAATATTCATAATGATAGTAGGAGGCTTAGTAGGTTTACGAATAGTTTTTTCTGTACTTTCTATAGTGAATAGAGTTAGGCAGGGATACTCACCACTATCGTTTCAGACCCAC---TCCCCAGCCCCGAGGGGA------CCCGACAGGCCCGGAGGAACAGAAGAAGAAGGTGGAGAAAGAGACAGAGACAGATCCGGTCCATTTGTGAACGGATTCTTGACGCTCATCTGGGTCGACCTGAGGAGCCTGTGCCTCTTCTGTTACCACCGCTTGAGAGACTTACTCTTGATTCTAGCGAGGATTGTAGAACTTCTGGGACGCAGG---------------GGGTGGGAAATCCTCAAATATTGGTGG---AATATCCTACAGTATTGG---------------------------------------------------AGTCAGGAACTAAAGAATAGCGCTGTCAGCTTGCTCAACGCCATAGCCATAGCAGTAGCTGAGGGGACAGATAGGATTATAGAAATAGTACAAAGA------------------GGTTTTAGA---------------------AGAATAAGACAGGGCTTGGAAAGGGCTGTGCTATAA

1.62995.SPD.EU575625 ATGAGAGCGAAGGGGATCAGGAAGAATTATCAGTACTTG---------TGGAGAGGGGGC------------------------ACCTTGCTCCTTGGGATATTGATGATC------------TGTAGTGCTGTA---------GAACAATTATGGGTCACAGTCTATTATGGGGTACCTGTGTGGAAAGAAGCAACCACCACTCTATTTTGTGCATCAGATGCTAAATCATATAGTACAGAGGTACATAAT---GTCTGGGCCACACATGCCTGTGTACCCACAGACCCTAGCCCACAAGAAGTAGTATTG---CAAAATGTGACAGAAAATTTTAACATGTGGAAAAATAACATGGTAGAACAAATGCATGAGGATATAATCAGTTTATGGGATCAAAGCCTAAAGCCATGTGTAAAATTAACCCCACTCTGTGTTACTCTAAATTGCTCTGATTTTAATGCTACTCAGGCCACTACTACTCAGGCC---------------------------------------------------------------------------------TATAATAGTAGCTGGAAGGTG------GAAGGAATGAAAAACTGCTCTTTCAATACCACC---TCAGGAATAAGAGAT------AAGGTGAAGAAAGAATATGCACTTCTTTATAAACTTGATATAGTAGAAATAAATGATGAT------------------------------AATAATACT---GGGGATAATACTAGCTATAGGTTCTATAGGTTGCTAAGTTGTAACACCTCGGTCATGACACAGGCCTGTCCAAAGGTATCCTTTGAGCCAATTCCCATACATTATTGTGCCCCAGCTGGTTTTGCGATTCTAAAGTGT---AATAATAAGACGTTCAATGGAACAGGACCGTGTACAAATGTCAGCACAGTACAATGTACACATGGAATTAGGCCAGTAGTATCAACTCAACTGCTGTTAAATGGCAGTCTAGCAGAAAAA---GAGATAGTAATTAGATCTGAAAATATCACGGACAATGCTAAGACCATAATAGTACAGTTGAATACAGCTGTAGAAATTAATTGTACAAGACCCAGCAACAATACAAGAAAAAGTATAAGTATAGGA------------CCAGGGAGA---GCATTTTATACAACAGGAGAGGTAATAGGAGATATAAGACAAGCACATTGCAACATT------AGTCAAGCAAAATGGAATAACACTCTAAGACAGGTGGCTATTAAATTAAGA---GAACAATTTCCG------------AATAAAACA---ATAATCTTTAATCAA---------TCCTCAGGAGGGGACCTAGAAATTGAAATGCACAGTTTTAATTGTGGAGGGGAATTTTTCTACTGCAATACAACAATACTGTTTAATAGTACTTGG---------------AATGAAACTAACCTGTTT------------------------AATGCCACTGAAGGA---------------AATAACACAGAA------------------------GTTATTACACTCCAATGCAGAATAAAACAAATTATAAACATGTGGCAGGAAGTAGGAAAAGCAATGTATGCCCCTCCCATCAGTGGACAAATTAACTGTTCATCAAACATTACAGGGCTGCTATTAACAAGAGATGGCGGTATTGGGAAC---------AATATCACC---------------------AATACCTCC---GAGATCTTCAGACCTATAGGAGGAAATATGAGGGACAATTGGAGA---AGTGAATTATATAAATATAAAGTAGTCAAAATTGAACCA---ATAGGAATAGCACCC---ACCAAGGCAAAGAGAAGAGTGGTGCAGAGAGAA---AAAAGAGCAGTG---GGA---ATAGGA---GCTATG---TTCCTT---GGG---------TTCTTGGGA---GCAGCAGGAAGCACTATGGGCGCAGCGTCAGTG---ACGCTGACGGTACAGGCCAGACTATTATTGTCTGGTATAGTGCAACAGCAGAACAATCTGCTGAGAGCTATTGAGGCGCAACAACATCTGTTGCAACTCACAGTCTGGGGCATCAAGCAGCTCCAGGCAAGA---GTCCTGGCTGTGGAAAGATACCTAAAGGATCAACAGCTCCTGGGGATTTGGGGTTGCTCTGGAAAACTCATTTGCACCACTGCTGTGCCTTGGAATACTAGTTGGAGT---------------------------AATAAATCTCTGGAAAAAATTTGGAAT---AACATGACCTGGATGGAGTGGGAAAGAGAAATAGAC------AATTATACAAACTTAATATATAACTTACTTGAAGACTCGCAAAACCAACAAGACAAGAATGAAAAAGAATTATTGGAATTAGATAAATGGGCAAATTTGTGGAATTGGTTTGACATAACAAAGTGGCTGTGGTATATAAAAATATTCATAATGATAGTAGGAGGCTTAGTAGGTTTACGAATAGTTTTTTCTGTACTTTCTATAGTGAATAGAGTTAGGCAGGGATACTCACCACTATCGTTTCAGACCCAC---TCCCCAGCCCCGAGGGGA------CCCGACAGGCCCGGAGGAACAGAAGAAGAAGGTGGAGAAAGAGACAGAGACAGATCCGGTCCATTTGTGAACGGATTCTTGACGCTCATCTGGGTCGACCTGAGGAGCCTGTGCCTCTTCTGTTACCACCGCTTGAGAGACTTACTCTTGATTCTAGCGAGGATTGTAGAACTTCTGGGACGCAGG---------------GGGTGGGAAATCCTCAAATATTGGTGG---AATATCCTACAGTATTGG---------------------------------------------------AGTCAGGAACTAAAGAATAGCGCTGTCAGCTTGCTCAACGCCATAGCCATAGCAGTAGCTGAGGGGACAGATAGGATTATAGAAATAGTACAAAGA------------------GGTTTTAGA---------------------AGAATAAGACAGGGCTTGGAAAGGGCTGTGCTATAA

1.62995.SPD.EU575629 ATGAGAGCGAAGGGGATCAGGAAGAATTATCAGTACTTG---------TGGAGAGGGGGC------------------------ACCTTGCTCCTTGGGATATTGATGATC------------TGTAGTGCTGTA---------GAACAATTATGGGTCACAGTCTATTATGGGGTACCTGTGTGGAAAGAAGCAACCACCACTCTATTTTGTGCATCAGATGCTAAATCATATAGTACAGAGGTACATAAT---GTCTGGGCCACACATGCCTGTGTACCCACAGACCCTAGCCCACAAGAAGTAGTATTG---CAAAATGTGACAGAAAATTTTAACATGTGGAAAAATAACATGGTAGAACAAATGCATGAGGATATAATCAGTTTATGGGATCAAAGCCTAAAGCCATGTGTAAAATTAACCCCACTCTGTGTTACTCTAAATTGCTCTGATTTTAATGCTACTCAGGCCACTACTACTCAGGCC---------------------------------------------------------------------------------TATAATAGTAGCTGGAAGGTG------GAAGGAATGAAAAACTGCTCTTTCAATACCACC---TCAGGAATAAGAGAT------AAGGTGAAGAAAGAATATGCACTTCTTTATAAACTTGATATAGTAGAAATAAATGATGAT------------------------------AATAATACT---GGGGATAATACTAGCTATAGGTTCTATAGGTTGCTAAGTTGTAACACCTCGGTCATGACACAGGCCTGTCCAAAGGTATCCTTTGAGCCAATTCCCATACATTATTGTGCCCCAGCTGGTTTTGCGATTCTAAAGTGT---AATAATAAGACGTTCAATGGAACAGGACCGTGTACAAATGTCAGCACAGTACAATGTACACATGGAATTAGGCCAGTAGTATCAACTCAACTGCTGTTAAATGGCAGTATAGCAGAAAAA---GAGATAGTAATTAGATCTGAAAATATCACGGACAATGCTAAGACCATAATAGTACAGTTGAATACAGCTGTAGAAATTAATTGTACAAGACCCAGCAACAATACAAGAAAAAGTATAAGTATAGGA------------CCAGGGAGA---GCATTTTATACAACAGGAGAGGTAATAGGAGATATAAGACAAGCACATTGCAACATT------AGTCAAGCAAAATGGAATAACACTCTAAGACAGGTGGCTATTAAATTAAGA---GAACAATTTCCG------------AATAAAACA---ATAATCTTTAATCAA---------TCCTCAGGAGGGGACCTAGAAATTGAAATGCACAGTTTTAATTGTGGAGGGGAATTTTTCTACTGCAATACAACAATACTGTTTAATAGTACTTGG---------------AATGAAACTAACCTGTTT------------------------AATGCCACTGAAGGA---------------AATAACACAGAA------------------------GTTATTACACTCCAATGCAGAATAAAACAAATTATAAACATGTGGCAGGAAGTAGGAAAAGCAATGTATGCCCCTCCCATCAGTGGACAAATTAACTGTTCATCAAACATTACAGGGCTGCTATTAACAAGAGATGGCGGTATTGGGAAC---------AATATCACC---------------------AATACCTCC---GAGATCTTCAGACCTATAGGAGGAAATATGAGGGACAATTGGAGA---AGTGAATTATATAAATATAAAGTAGTCAAAATTGAACCA---ATAGGAATAGCACCC---ACCAAGGCAAAGAGAAGAGTGGTGCAGAGAGAA---AAAAGAGCAGTG---GGA---ATAGGA---GCTATG---TTCCTT---GGG---------TTCTTGGGA---GCAGCAGGAAGCACTATGGGCGCAGCGTCAGTG---ACGCTGACGGTACAGGCCAGACTATTATTGTCTGGTATAGTGCAACAGCAGAACAATCTGCTGAGAGCTATTGAGGCGCAACAACATCTGTTGCAACTCACAGTCTGGGGCATCAAGCAGCTCCAGGCAAGA---GTCCTGGCTGTGGAAAGATACCTAAAGGATCAACAGCTCCTGGGGATTTGGGGTTGCTCTGGAAAACTCATTTGCACCACTGCTGTGCCTTGGAATACTAGTTGGAGT---------------------------AATAAATCTCTGGAAAAAATTTGGAAT---AACATGACCTGGATGGAGTGGGAAAGAGAAATAGAC------AATTATACAAACTTAATATATAACTTACTTGAAGACTCGCAAAACCAACAAGACAAGAATGAAAAAGAATTATTGGAATTAGATAAATGGGCAAATTTGTGGAATTGGTTTGACATAACAAAGTGGCTGTGGTATATAAAAATATTCATAATGATAGTAGGAGGCTTAGTAGGTTTACGAATAGTTTTTTCTGTACTTTCTATAGTGAATAGAGTTAGGCAGGGATACTCACCACTATCGTTTCAGACCCAC---TCCCCAGCCCCGAGGGGA------CCCGACAGGCCCGGAGGAACAGAAGAAGAAGGTGGAGAAAGAGACAGAGACAGATCCGGTCCATTTGTGAACGGATTCTTGACGCTCATCTGGGTCGACCTGAGGAGCCTGTGCCTCTTCTGTTACCACCGCTTGAGAGACTTACTCTTGATTCTAGCGAGGATTGTAGAACTTCTGGGACGCAGG---------------GGGTGGGAAATCCTCAAATATTGGTGG---AATATCCTACAGTATTGG---------------------------------------------------AGTCAGGAACTAAAGAATAGCGCTGTCAGCTTGCTCAACGCCATAGCCATAGCAGTAGCTGAGGGGACAGATAGGATTATAGAAATAGTACAAAGA------------------GGTTTTAGA---------------------AGAATAAGACAGGGCTTGGAAAGGGCTGTGCTATAA

1.62995.SPD.EU575640 ATGAGAGCGAAGGGGATCAGGAAGAATTATCAGTACTTG---------TGGAGAGGGGGC------------------------ACCTTGCTCCTTGGGATATTGATGATC------------TGTAGTGCTGTA---------GAACAATTATGGGTCACAGTCTATTATGGGGTACCTGTGTGGAAAGAAGCAACCACCACTCTATTTTGTGCATCAGATGCTAAATCATATAGTACAGAGGTACATAAT---GTCTGGGCCACACATGCCTGTGTACCCACAGACCCTAGCCCACAAGAAGTAGTATTG---CAAAATGTGACAGAAAATTTTAACATGTGGAAAAATAACATGGTAGAACAAATGCATGAGGATATAATCAGTTTATGGGATCAAAGCCTAAAGCCATGTGTAAAATTAACCCCACTCTGTGTTACTCTAAATTGCTCTGATTTTAATGCTACTCAGGCCACTACTACTCAGGCC---------------------------------------------------------------------------------TATAATAGTAGCTGGAAGGTG------GAAGGAATGAAAAACTGCTCTTTCAATACCACC---TCAGGAATAAGAGAT------AAGGTGAAGAAAGAATATGCACTTCTTTATAAACTTGATATAGTAGAAATAAATGATGAT------------------------------AATAATACT---GGGGATAATACTAGCTATAGGTTCTATAGGTTGCTAAGTTGTAACACCTCGGTCATGACACAGGCCTGTCCAAAGGTATCCTTTGAGCCAATTCCCATACATTATTGTGCCCCAGCTGGTTTTGCGATTCTAAAGTGT---AATAATAAGACGTTCAATGGAACAGGACCGTGTACAAATGTCAGCACAGTACAATGTACACATGGAATTAGGCCAGTAGTATCAACTCAACTGCTGTTAAATGGCAGTCTAGCAGAAAAA---GAGATAGTAATTAGATCTGAAAATATCACGGACAATGCTAAGACCATAATAGTACAGTTGAATACAGCTGTAGAAATTAATTGTACAAGACCCAGCAACAATACAAGAAAAAGTATAAGTATAGGA------------CCAGGGAGA---GCATTTTATACAACAGGAGAGGTAATAGGAGATATAAGACAAGCACATTGCAACATT------AGTCAAGCAAAATGGAATAACACTCTAAGACAGGTGGCTATTAAATTAAGA---GAACAATTTCCG------------AATAAAACA---ATAATCTTTAATCAA---------TCCTCAGGAGGGGACCTAGAAATTGAAATGCACAGTTTTAATTGTGGAGGGGAATTTTTCTACTGCAATACAACAATACTGTTTAATAGTACTTGG---------------AATGAAACTAACCTGTTT------------------------AATGCCACTGAAGGA---------------AATAACACAGAA------------------------GTTATTACACTCCAATGCAGAATAAAACAAATTATAAACATGTGGCAGGAAGTAGGAAAAGCAATGTATGCCCCTCCCATCAGTGGACAAATTAACTGTTCATCAAACATTACAGGGCTGCTATTAACAAGAGATGGCGGTATTGGGAAC---------AATATCACC---------------------AATACCTCC---GAGATCTTCAGACCTATAGGAGGAAATATGAGGGACAATTGGAGA---AGTGAATTATATAAATATAAAGTAGTCAAAATTGAACCA---ATAGGAATAGCACCC---ACCAAGGCAAAGAGAAGAGTGGTGCAGAGAGAA---AAAAGAGCAGTG---GGA---ATAGGA---GCTATG---TTCCTT---GGG---------TTCTTGGGA---GCAGCAGGAAGCACTATGGGCGCAGCGTCAGTG---ACGCTGACGGTACAGGCCAGACTATTATTGTCTGGTATAGTGCAACAGCAGAACAATCTGCTGAGAGCTATTGAGGCGCAACAACATCTGTTGCAACTCACAGTCTGGGGCATCAAGCAGCTCCAGGCAAGA---GTCCTGGCTGTGGAAAGATACCTAAAGGATCAACAGCTCCTGGGGATTTGGGGTTGCTCTGGAAAACTCATTTGCACCACTGCTGTGCCTTGGAATACTAGTTGGAGT---------------------------AATAAATCTCTGGAAAAAATTTGGAAT---AACATGACCTGGATGGAGTGGGAAAGAGAAATAGAC------AATTATACAAACTTAATATATAACTTACTTGAAGACTCGCAAAACCAACAAGACAAGAATGAAAAAGAATTATTGGAATTAGATAAATGGGCAAATTTGTGGAATTGGTTTGACATAACAAAGTGGCTGTGGTATATAAAAATATTCATAATGATAGTAGGAGGCTTAGTAGGTTTACGAATAGTTTTTTCTGTACTTTCTATAGTGAATAGAGTTAGGCAGGGATACTCACCACTATCGTTTCAGACCCAC---TCCCCAGCCCCGAGGGGA------CCCGACAGGCCCGGAGGAACAGAAGAAGAAGGTGGAGAAAGAGACAGAGACAGATCCGGTCCATTTGTGAACGGATTCTTGACGCTCATCTGGGTCGACCTGAGGAGCCTGTGCCTCTTCTGTTACCACCGCTTGAGAGACTTACTCTTGATTCTAGCGAGGATTGTAGAACTTCTGGGACGCAGG---------------GGGTGGGAAATCCTCAAATATTGGTGG---AATATCCTACAGTATTGG---------------------------------------------------AGTCAGGAACTAAAGAATAGCGCTGTCAGCTTGCTCAACGCCATAGCCATAGCAGTAGCTGAGGGGACAGATAGGATTATAGAAATAGTACAAAGA------------------GGTTTTAGA---------------------AGAATAAGACAGGGCTTGGAAAGGGCTGTGCTATAA

1.62995.SPD.EU575621 ATGAGAGCGAAGGGGATCAGGAAGAATTATCAGTACTTG---------TGGAGAGGGGGC------------------------ACCTTGCTCCTTGGGATATTGATGATC------------TGTAGTGCTGTA---------GAACAATTATGGGTCACAGTCTATTATGGGGTACCTGTGTGGAAAGAAGCAACCACCACTCTATTTTGTGCATCAGATGCTAAATCATATAGTACAGAGGTACATAAT---GTCTGGGCCACACATGCCTGTGTACCCACAGACCCTAGCCCACAAGAAGTAGTATTG---CAAAATGTGACAGAAAATTTTAACATGTGGAAAAATAACATGGTAGAACAAATGCATGAGGATATAATCAGTTTATGGGATCAAAGCCTAAAGCCATGTGTAAAATTAACCCCACTCTGTGTTACTCTAAATTGCTCTGATTTTAATGCTACTCAGGCCACTACTACTCAGGCC---------------------------------------------------------------------------------TATAATAGTAGCTGGAAGGTG------GAAGGAATGAAAAACTGCTCTTTCAATACCACC---TCAGGAATAAGAGAT------AAGGTGAAGAAAGAATATGCACTTCTTTATAAACTTGATATAGTAGAAATAAATGATGAT------------------------------AATAATACT---GGGGATAATACTAGCTATAGGTTCTATAGGTTGCTAAGTTGTAACACCTCGGTCATGACACAGGCCTGTCCAAAGGTATCCTTTGAGCCAATTCCCATACATTATTGTGCCCCAGCTGGTTTTGCGATTCTAAAGTGT---AATAATAAGACGTTCAATGGAACAGGACCGTGTACAAATGTCAGCACAGTACAATGTACACATGGAATTAGGCCAGTAGTATCAACTCAACTGCTGTTAAATGGCAGTCTAGCAGAAAAA---GAGATAGTAATTAGATCTGAAAATATCACGGACAATGCTAAGACCATAATAGTACAGTTGAATACAGCTGTAGAAATTAATTGTACAAGACCCAGCAACAATACAAGAAAAAGTATAAGTATAGGA------------CCAGGGAGA---GCATTTTATACAACAGGAGAGGTAATAGGAGATATAAGACAAGCACATTGCAACATT------AGTCAAGCAAAATGGAATAACACTCTAAGACAGGTGGCTATTAAATTAAGA---GAACAATTTCCG------------AATAAAACA---ATAATCTTTAATCAA---------TCCTCAGGAGGGGACCTAGAAATTGAAATGCACAGTTTTAATTGTGGAGGGGAATTTTTCTACTGCAATACAACAATACTGTTTAATAGTACTTGG---------------AATGAAACTAACCTGTTT------------------------AATGCCACTGAAGGA---------------AATAACACAGAA------------------------GTTATTACACTCCAATGCAGAATAAAACAAATTATAAACATGTGGCAGGAAGTAGGAAAAGCAATGTATGCCCCTCCCATCAGTGGACAAATTAACTGTTCATCAAACATTACAGGGCTGCTATTAACAAGAGATGGCGGTATTGGGAAC---------AATATCACC---------------------AATACCTCC---GAGATCTTCAGACCTATAGGAGGAAATATGAGGGACAAGTGGAGA---AGTGAATTATATAAATATAAAGTAGTCAAAATTGAACCA---ATAGGAATAGCACCC---ACCAAGGCAAAGAGAAGAGTGGTGCAGAGAGAA---AAAAGAGCAGTG---GGA---ATAGGA---GCTATG---TTCCTT---GGG---------TTCTTGGGA---GCAGCAGGAAGCACTATGGGCGCAGCGTCAGTG---ACGCTGACGGTACAGGCCAGACTATTATTGTCTGGTATAGTGCAACAGCAGAACAATCTGCTGAGAGCTATTGAGGCGCAACAACATCTGTTGCAACTCACAGTCTGGGGCATCAAGCAGCTCCAGGCAAGA---GTCCTGGCTGTGGAAAGATACCTAAAGGATCAACAGCTCCTGGGGATTTGGGGTTGCTCTGGAAAACTCATTTGCACCACTGCTGTGCCTTGGAATACTAGTTGGAGT---------------------------AATAAATCTCTGGAAAAAATTTGGAAT---AACATGACCTGGATGGAGTGGGAAAGAGAAATAGAC------AATTATACAAACTTAATATATAACTTACTTGAAGACTCGCAAAACCAACAAGACAAGAATGAAAAAGAATTATTGGAATTAGATAAATGGGCAAATTTGTGGAATTGGTTTGACATAACAAAGTGGCTGTGGTATATAAAAATATTCATAATGATAGTAGGAGGCTTAGTAGGTTTACGAATAGTTTTTTCTGTACTTTCTATAGTGAATAGAGTTAGGCAGGGATACTCACCACTATCGTTTCAGACCCAC---TCCCCAGCCCCGAGGGGA------CCCGACAGGCCCGGAGGAACAGAAGAAGAAGGTGGAGAAAGAGACAGAGACAGATCCGGTCCATTTGTGAACGGATTCTTGACGCTCATCTGGGTCGACCTGAGGAGCCTGTGCCTCTTCTGTTACCACCGCTTGAGAGACTTACTCTTGATTCTAGCGAGGATTGTAGAACTTCTGGGACGCAGG---------------GGGTGGGAAATCCTCAAATATTGGTGG---AATATCCTACAGTATTGG---------------------------------------------------AGTCAGGAACTAAAGAATAGCGCTGTCAGCTTGCTCAACGCCATAGCCATAGCAGTAGCTGAGGGGACAGATAGGATTATAGAAATAGTACAAAGA------------------GGTTTTAGA---------------------AGAATAAGACAGGGCTTGGAAAGGGCTGTGCTATAA

1.62995.SPD.EU575624 ATGAGAGCGAAGGGGATCAGGAAGAATTATCAGTACTTG---------TGGAGAGGGGGC------------------------ACCTTGCTCCTTGGGATATTGATGATC------------TGTAGTGCTGTA---------GAACAATTATGGGTCACAGTCTATTATGGGGTACCTGTGTGGAAAGAAGCAACCACCACTCTATTTTGTGCATCAGATGCTAAATCATATAGTACAGAGGTACATAAT---GTCTGGGCCACACATGCCTGTGTACCCACAGACCCTAGCCCACAAGAAGTAGTATTG---CAAAATGTGACAGAAAATTTTAACATGTGGAAAAATAACATGGTAGAACAAATGCATGAGGATATAATCAGTTTATGGGATCAAAGCCTAAAGCCATGTGTAAAATTAACCCCACTCTGTGTTACTCTAAATTGCTCTGATTTTAATGCTACTCAGGCCACTACTACTCAGGCC---------------------------------------------------------------------------------TATAATAGTAGCTGGAAGGTG------GAAGGAATGAAAAACTGCTCTTTCAATACCACC---TCAGGAATAAGAGAT------AAGGTGAAGAAAGAATATGCACTTCTTTATAAACTTGATATAGTAGAAATAAATGATGAT------------------------------AATAATACT---GGGGATAATACTAGCTATAGGTTCTATAGGTTGCTAAGTTGTAACACCTCGGTCATGACACAGGCCTGTCCAAAGGTATCCTTTGAGCCAATTCCCATACATTATTGTGCCCCAGCTGGTTTTGCGATTCTAAAGTGT---AATAATAAGACGTTCAATGGAACAGGACCGTGTACAAATGTCAGCACAGTACAATGTACACATGGAATTAGGCCAGTAGTATCAACTCAACTGCTGTTAAATGGCAGTCTAGCAGAAAAA---GAGATAGTAATTAGATCTGAAAATATCACGGACAATGCTAAGACCATAATAGTACAGTTGAATACAGCTGTAGAAATTAATTGTACAAGACCCAGCAACAATACAAGAAAAAGTATAAGTATAGGA------------CCAGGGAGA---GCATTTTATACAACAGGAGAGGTAATAGGAGATATAAGACAAGCACATTGCAACATT------AGTCAAGCAAAATGGAATAACACTCTAAGACAGGTGGCTATTAAATTAAGA---GAACAATTTCCG------------AATAAAACA---ATAATCTTTAATCAA---------TCCTCAGGAGGGGACCTAGAAATTGAAATGCACAGTTTTAATTGTGGAGGGGAATTTTTCTACTGCAATACAACAATACTGTTTAATAGTACTTGG---------------AATGAAACTAACCTGTTT------------------------AATGCCACTGAAGGA---------------AATAACACAGAA------------------------GTTATTACACTCCAATGCAGAATAAAACAAATTATAAACATGTGGCAGGAAGTAGGAAAAGCAATGTATGCCCCTCCCATCAGTGGACAAATTAACTGTTCATCAAACATTACAGGGCTGCTATTAACAAGAGATGGCGGTATTGGGAAC---------AATATCACC---------------------AATACCTCC---GAGATCTTCAGACCTATAGGAGGAAATATGAGGGACAATTGGAGA---AGTGAATTATATAAATATAAAGTAGTCAAAATTGAACCA---ATAGGAATAGCACCC---ACCAAGGCAAAGAGAAGAGTGGTGCAGAGAGAA---AAAAGAGCAGGG---GGA---ATAGGA---GCTATG---TTCCTT---GGG---------TTCTTGGGA---GCAGCAGGAAGCACTATGGGCGCAGCGTCAGTG---ACGCTGACGGTACAGGCCAGACTATTATTGTCTGGTATAGTGCAACAGCAGAACAATCTGCTGAGAGCTATTGAGGCGCAACAACATCTGTTGCAACTCACAGTCTGGGGCATCAAGCAGCTCCAGGCAAGA---GTCCTGGCTGTGGAAAGATACCTAAAGGATCAACAGCTCCTGGGGATTTGGGGTTGCTCTGGAAAACTCATTTGCACCACTGCTGTGCCTTGGAATACTAGTTGGAGT---------------------------AATAAATCTCTGGAAAAAATTTGGAAT---AACATGACCTGGATGGAGTGGGAAAGAGAAATAGAC------AATTATACAAACTTAATATATAACTTACTTGAAGACTCGCAAAACCAACAAGACAAGAATGAAAAAGAATTATTGGAATTAGATAAATGGGCAAATTTGTGGAATTGGTTTGACATAACAAAGTGGCTGTGGTATATAAAAATATTCATAATGATAGTAGGAGGCTTAGTAGGTTTACGAATAGTTTTTTCTGTACTTTCTATAGTGAATAGAGTTAGGCAGGGATACTCACCACTATCGTTTCAGACCCAC---TCCCCAGCCCCGAGGGGA------CCCGACAGGCCCGGAGGAACAGAAGAAGAAGGTGGAGAAAGAGACAGAGACAGATCCGGTCCATTTGTGAACGGATTCTTGACGCTCATCTGGGTCGACCTGAGGAGCCTGTGCCTCTTCTGTTACCACCGCTTGAGAGACTTACTCTTGATTCTAGCGAGGATTGTAGAACTTCTGGGACGCAGG---------------GGGTGGGAAATCCTCAAATATTGGTGG---AATATCCTACAGTATTGG---------------------------------------------------AGTCAGGAACTAAAGAATAGCGCTGTCAGCTTGCTCAACGCCATAGCCATAGCAGTAGCTGAGGGGACAGATAGGATTATAGAAATAGTACAAAGA------------------GGTTTTAGA---------------------AGAATAAGACAGGGCTTGGAAAGGGCTGTGCTATAA

1.62995.SPD.EU575623 ATGAGAGCGAAGGGGATCAGGAAGAATTATCAGTACTTG---------TGGAGAGGGGGC------------------------ACCTTGCTCCTTGGGATATTGATGATC------------TGTAGTGCTGTA---------GAACAATTATGGGTCACAGTCTATTATGGGGTACCTGTGTGGAAAGAAGCAACCACCACTCTATTTTGTGCATCAGATGCTAAATCATATAGTACAGAGGTACATAAT---GTCTGGGCCACACATGCCTGTGTACCCACAGACCCTAGCCCACAAGAAGTAGTATTG---CAAAATGTGACAGAAAATTTTAACATGTGGAAAAATAACATGGTAGAACAAATGCATGAGGATATAATCAGTTTATGGGATCAAAGCCTAAAGCCATGTGTAAAATTAACCCCACTCTGTGTTACTCTAAATTGCTCTGATTTTAATGCTACTCAGGCCACTACTACTCAGGCC---------------------------------------------------------------------------------TATAATAGTAGCTGGAAGGTG------GAAGGAATGAAAAACTGCTCTTTCAATACCACC---TCAGGAATAAGAGAT------AAGGTGAAGAAAGAATATGCACTTCTTTATAAACTTGATATAGTAGAAATAAATGATGAT------------------------------AATAATACT---GGGGATAATACTAGCTATAGGTTCTATAGGTTGCTAAGTTGTAACACCTCGGTCATGACACAGGCCTGTCCAAAGGTATCCTTTGAGCCAATTCCCATACATTATTGTGCCCCAGCTGGTTTTGCGATTCTAAAGTGT---AATAATAAGACGTTCAATGGAACAGGACCGTGTACAAATGTCAGCACAGTACAATGTACACATGGAATTAGGCCAGTAGTATCAACTCAACTGCTGTTAAATGGCAGTCTAGCAGAAAAA---GAGATAGTAATTAGATCTGAAAATATCACGGACAATGCTAAGACCATAATAGTACAGTTGAATACAGCTGTAGAAATTAATTGTACAAGACCCAGCAACAATACAAGAAAAAGTATAAGTATAGGA------------CCAGGGAGA---GCATTTTATACAACAGGAGAGGTAATAGGAGATATAAGACAAGCACATTGCAACATT------AGTCAAGCAAAATGGAATAACACTCTAAGACAGGTGGCTATTAAATTAAGA---GAACAATTTCCG------------AATAAAACA---ATAATCTTTAATCAA---------TCCTCAGGAGGGGACCTAGAAATTGAAATGCACAGTTTTAATTGTGGAGGGGAATTTTTCTACTGCAATACAACAATACTGTTTAATAGTACTTGG---------------AATGAAACTAACCTGTTT------------------------AATGCCACTGAAGGA---------------AATAACACAGAA------------------------GTTATTACACTCCAATGCAGAATAAAACAAATTATAAACATGTGGCAGGAAGTAGGAAAAGCAATGTATGCCCCTCCCATCAGTGGACAAATTAACTGTTCATCAAACATTACAGGGCTGCTATTAACAAGAGATGGCGGTATTGGGAAC---------AATATCACC---------------------AATACCTCC---GAGATCTTCAGACCTATAGGAGGAAATATGAGGGACAATTGGAGA---AGTGAATTATATAAATATAAAGTAGTCAAAATTGAACCA---ATAGGAATAGCACCC---ACCAAGGCAAAGAGAAGAGTGGTGCAGAGAGAA---AAAAGAGCAGTG---GGA---ATAGGA---GCTATG---TTCCTT---GGG---------TTCTTGGGA---GCAGCAGGAAGCACTATGGGCGCAGCGTCAGTG---ACGCTGACGGTACAGGCCAGACTATTATTGTCTGGTATAGTGCAACAGCAGAACAATCTGCTGAGAGCTATTGAGGCGCAACAACATCTGTTGCAACTCACAGTCTGGGGCATCAAGCAGCTCCAGGCAAGA---GTCCTGGCTGTGGAAAGATACCTAAAGGATCAACAGCTCCTGGGGATTTGGGGTTGCTCTGGAAAACTCATTTGCACCACTGCTGTGCCTTGGAATACTAGTTGGAGT---------------------------AATAAATCTCTGGAAAAAATTTGGAAT---AACATGACCTGGATGGAGTGGGAAAGAGAAATAGAC------AATTATACAAACTTAATATATAACTTACTTGAAGACTCGCAAAACCAACAAGACAAGAATGAAAAAGAATTATTGGAATTAGATAAATGGGCAAATTTGTGGAATTGGTTTGACATAACAAAGTGGCTGTGGTATATAAAAATATTCATAATGATAGTAGGAGGCTTAGTAGGTTTACGAATAGTTTTTTCTGTACTTTCTATAGTGAATAGAGTTAGGCAGGGATACTCACCACTATCGTTTCAGACCCAC---TCCCCAGCCCCGAGGGGA------CCCGACAGGCCCGGAGGAACAGAAGAAGAAGGTGGAGAAAGAGACAGAGACAGATCCGGTCCATTTGTGAACGGATTCTTGACGCTCATCTGGGTCGACCTGAGGAGCCTGTGCCTCTTCTGTTACCACCGCTTGAGAGACTTACTCTTGATTCTAGCGAGGATTGTAGAACTTCTGGGACGCAGG---------------GGGTGGGAAATCCTCAAATATTGGTGG---AATATCCTACAGTATTGG---------------------------------------------------AGTCAGGAACTAAAGAATAGCGCTGTCAGCTTGCTCAACGCCATAGCCATAGCAGTAGCTGAGGGGACAGATAGGATTATAGAAATAGTACAAAGA------------------GGTTTTAGA---------------------AGAATAAGACAGGGCTTGGAAAGGGCTGTGCTATAA

1.62995.SPD.EU575628 ATGAGAGCGAAGGGGATCAGGAAGAATTATCAGTACTTG---------TGGAGAGGGGGC------------------------ACCTTGCTCCTTGGGATATTGATGATC------------TGTAGTGCTGTA---------GAACAATTATGGGTCACAGTCTATTATGGGGTACCTGTGTGGAAAGAAGCAACCACCACTCTATTTTGTGCATCAGATGCTAAATCATATAGTACAGAGGTACATAAT---GTCTGGGCCACACATGCCTGTGTACCCACAGACCCTAGCCCACAAGAAGTAGTATTG---CAAAATGTGACAGAAAATTTTAACATGTGGAAAAATAACATGGTAGAACAAATGCATGAGGATATAATCAGTTTATGGGATCAAAGCCTAAAGCCATGTGTAAAATTAACCCCACTCTGTGTTACTCTAAATTGCTCTGATTTTAATGCTACTCAGGCCACTACTACTCAGGCC---------------------------------------------------------------------------------TATAATAGTAGCTGGAAGGTG------GAAGGAATGAAAAACTGCTCTTTCAATACCACC---TCAGGAATAAGAGAT------AAGGTGAAGAAAGAATATGCACTTCTTTATAAACTTGATATAGTAGAAATAAATGATGAT------------------------------AATAATACT---GGGGATAATACTAGCTATAGGTTCTATAGGTTGCTAAGTTGTAACACCTCGGTCATGACACAGGCCTGTCCAAAGGTATCCTTTGAGCCAATTCCCATACATTATTGTGCCCCAGCTGGTTTTGCGATTCTAAAGTGT---AATAATAAGACGTTCAATGGAACAGGACCGTGTACAAATGTCAGCACAGTACAATGTACACATGGAATTAGGCCAGTAGTATCAACTCAACTGCTGTTAAATGGCAGTCTAGCAGAAAAA---GAGATAGTAATTAGATCTGAAAATATCACGGACAATGCTAAGACCATAATAGTACAGTTGAATACAGCTGTAGAAATTAATTGTACAAGACCCAGCAACAATACAAGAAAAAGTATAAGTATAGGA------------CCAGGGAGA---GCATTTTATACAACAGGAGAGGTAATAGGAGATATAAGACAAGCACATTGCAACATT------AGTCAAGCAAAATGGAATAACACTCTAAGACAGGTGGCTATTAAATTAAGA---GAACAATTTCCG------------AATAAAACA---ATAATCTTTAATCAA---------TCCTCAGGAGGGGACCTAGAAATTGAAATGCACAGTTTTAATTGTGGAGGGGAATTTTTCTACTGCAATACAACAATACTGTTTAATAGTACTTGG---------------AATGAAACTAACCTGTTT------------------------AATGCCACTGAAGGA---------------AATAACACAGAA------------------------GTTATTACACTCCAATGCAGAATAAAACAAATTATAAACATGTGGCAGGAAGTAGGAAAAGCAATGTATGCCCCTCCCATCAGTGGACAAATTAACTGTTCATCAAACATTACAGGGCTGCTATTAACAAGAGATGGCGGTATTGGGAAC---------AATATCACC---------------------AATACCTCC---GAGATCTTCAGACCTATAGGAGGAAATATGAGGGACAATTGGAGA---AGTGAATTATATAAATATAAAGTAGTCAAAATTGAACCA---ATAGGAATAGCACCC---ACCAAGGCAAAGAGAAGAGTGGTGCAGAGAGAA---AAAAGAGCAGTG---GGA---ATAGGA---GCTATG---TTCCTT---GGG---------TTCTTGGGA---GCAGCAGGAAGCACTATGGGCGCAGCGTCAGTG---ACGCTGACGGTACAGGCCAGACTATTATTGTCTGGTATAGTGCAACAGCAGAACAATCTGCTGAGAGCTATTGAGGCGCAACAACATCTGTTGCAACTCACAGTCTGGGGCATCAAGCAGCTCCAGGCAAGA---GTCCTGGCTGTGGAAAGATACCTAAAGGATCAACAGCTCCTGGGGATTTGGGGTTGCTCTGGAAAACTCATTTGCACCACTGCTGTGCCTTGGAATACTAGTTGGAGT---------------------------AATAAATCTCTGGAAAAAATTTGGAAT---AACATGACCTGGATGGAGTGGGAAAGAGAAATAGAC------AATTATACAAACTTAATATATAACTTACTTGAAGACTCGCAAAACCAACAAGACAAGAATGAAAAAGAATTATTGGAATTAGATAAATGGGCAAATTTGTGGAATTGGTTTGACATAACAAAGTGGCTGTGGTATATAAAAATATTCATAATGATAGTAGGAGGCTTAGTAGGTTTACGAATAGTTTTTTCTGTACTTTCTATAGTGAATAGAGTTAGGCAGGGATACTCACCACTATCGTTTCAGACCCAC---TCCCCAGCCCCGAGGGGA------CCCGACAGGCCCGGAGGAACAGAAGAAGAAGGTGGAGAAAGAGACAGAGACAGATCCGGTCCATTTGTGAACGGATTCTTGACGCTCATCTGGGTCGACCTGAGGAGCCTGTGCCTCTTCTGTTACCACCGCTTGAGAGACTTACTCTTGATTCTAGCGAGGATTGTAGAACTTCTGGGACGCAGG---------------GGGTGGGAAATCCTCAAATATTGGTGG---AATATCCTACAGTATTGG---------------------------------------------------AGTCAGGAACTAAAGAATAGCGCTGTCAGCTTGCTCAACGCCATAGCCATAGCAGTAGCTGAGGGGACAGATAGGATTATAGAAATAGTACAAAGA------------------GGTTTTAGA---------------------AGAATAAGACAGGGCTTGGAAAGGGCTGTGCTATAA

1.62995.SPD.EU575645 ATGAGAGCGAAGGGGATCAGGAAGAATTATCAGTACTTG---------TGGAGAGGGGGC------------------------ACCTTGCTCCTTGGGATATTGATGATC------------TGTAGTGCTGTA---------GAACAATTATGGGTCACAGTCTATTATGGGGTACCTGTGTGGAAAGAAGCAACCACCACTCTATTTTGTGCATCAGATGCTAAATCATATAGTACAGAGGTACATAAT---GTCTGGGCCACACATGCCTGTGTACCCACAGACCCTAGCCCACAAGAAGTAGTATTG---CAAAATGTGACAGAAAATTTTAACATGTGGAAAAATAACATGGTAGAACAAATGCATGAGGATATAATCAGTTTATGGGATCAAAGCCTAAAGCCATGTGTAAAATTAACCCCACTCTGTGTTACTCTAAATTGCTCTGATTTTAATGCTACTCAGGCCACTACTACTCAGGCC---------------------------------------------------------------------------------TATAATAGTAGCTGGAAGGTG------GAAGGAATGAAAAACTGCTCTTTCAATACCACC---TCAGGAATAAGAGAT------AAGGTGAAGAAAGAATATGCACTTCTTTATAAACTTGATATAGTAGAAATAAATGATGAT------------------------------AATAATACT---GGGGATAATACTAGCTATAGGTTCTATAGGTTGCTAAGTTGTAACACCTCGGTCATGACACAGGCCTGTCCAAAGGTATCCTTTGAGCCAATTCCCATACATTATTGTGCCCCAGCTGGTTTTGCGATTCTAAAGTGT---AATAATAAGACGTTCAATGGAACAGGACCGTGTACAAATGTCAGCACAGTACAATGTACACATGGAATTAGGCCAGTAGTATCAACTCAACTGCTGTTAAATGGCAGTCTAGCAGAAAAA---GAGATAGTAATTAGATCTGAAAATATCACGGACAATGCTAAGACCATAATAGTACAGTTGAATACAGCTGTAGAAATTAATTGTACAAGACCCAGCAACAATACAAGAAAAAGTATAAGTATAGGA------------CCAGGGAGA---GCATTTTATACAACAGGAGAGGTAATAGGAGATATAAGACAAGCACATTGCAACATT------AGTCAAGCAAAATGGAATAACACTCTAAGACAGGTGGCTATTAAATTAAGA---GAACAATTTCCG------------AATAAAACA---ATAATCTTTAATCAA---------TCCTCAGGAGGGGACCTAGAAATTGAAATGCACAGTTTTAATTGTGGAGGGGAATTTTTCTACTGCAATACAACAATACTGTTTAATAGTACTTGG---------------AATGAAACTAACCTGTTT------------------------AATGCCACTGAAGGA---------------AATAACACAGAA------------------------GTTATTACACTCCAATGCAGAATAAAACAAATTATAAACATGTGGCAGGAAGTAGGAAAAGCAATGTATGCCCCTCCCATCAGTGGACAAATTAACTGTTCATCAAACATTACAGGGCTGCTATTAACAAGAGATGGCGGTATTGGGAAC---------AATATCACC---------------------AATACCTCC---GAGATCTTCAGACCTATAGGAGGAAATATGAGGGACAATTGGAGA---AGTGAATTATATAAATATAAAGTAGTCAAAATTGAACCA---ATAGGAATAGCACCC---ACCAAGGCAAAGAGAAGAGTGGTGCAGAGAGAA---AAAAGAGCAGTG---GGA---ATAGGA---GCTATG---TTCCTT---GGG---------TTCTTGGGA---GCAGCAGGAAGCACTATGGGCGCAGCGTCAGTG---ACGCTGACGGTACAGGCCAGACTATTATTGTCTGGTATAGTGCAACAGCAGAACAATCTGCTGAGAGCTATTGAGGCGCAACAACATCTGTTGCAACTCACAGTCTGGGGCATCAAGCAGCTCCAGGCAAGA---GTCCTGGCTGTGGAAAGATACCTAAAGGATCAACAGCTCCTGGGGATTTGGGGTTGCTCTGGAAAACTCATTTGCACCACTGCTGTGCCTTGGAATACTAGTTGGAGT---------------------------AATAAATCTCTGGAAAAAATTTGGAAT---AACATGACCTGGATGGAGTGGGAAAGAGAAATAGAC------AATTATACAAACTTAATATATAACTTACTTGAAGACTCGCAAAACCAACAAGACAAGAATGAAAAAGAATTATTGGAATTAGATAAATGGGCAAATTTGTGGAATTGGTTTGACATAACAAAGTGGCTGTGGTATATAAAAATATTCATAATGATAGTAGGAGGCTTAGTAGGTTTACGAATAGTTTTTTCTGTACTTTCTATAGTGAATAGAGTTAGGCAGGGATACTCACCACTATCGTTTCAGACCCAC---TCCCCAGCCCCGAGGGGA------CCCGACAGGCCCGGAGGAACAGAAGAAGAAGGTGGAGAAAGAGACAGAGACAGATCCGGTCCATTTGTGAACGGATTCTTGACGCTCATCTGGGTCGACCTGAGGAGCCTGTGCCTCTTCTGTTACCACCGCTTGAGAGACTTACTCTTGATTCTAGCGAGGATTGTAGAACTTCTGGGACGCAGG---------------GGGTGGGAAATCCTCAAATATTGGTGG---AATATCCTACAGTATTGG---------------------------------------------------AGTCAGGAACTAAAGAATAGCGCTGTCAGCTTGCTCAACGCCATAGCCATAGCAGTAGCTGAGGGGACAGATAGGATTATAGAAATAGTACAAAGA------------------GGTTTTAGA---------------------AGAATAAGACAGGGCTTGGAAAGGGCTGTGCTATAA

1.62995.SPD.EU575644 ATGAGAGCGAAGGGGATCAGGAAGAATTATCAGTACTTG---------TGGAGAGGGGGC------------------------ACCTTGCTCCTTGGGATATTGATGATC------------TGTAGTGCTGTA---------GAACAATTATGGGTCACAGTCTATTATGGGGTACCTGTGTGGAAAGAAGCAACCACCACTCTATTTTGTGCATCAGATGCTAAATCATATAGTACAGAGGTACATAAT---GTCTGGGCCACACATGCCTGTGTACCCACAGACCCTAGCCCACAAGAAGTAGTATTG---CAAAATGTGACAGAAAATTTTAACATGTGGAAAAATAACATGGTAGAACAAATGCATGAGGATATAATCAGTTTATGGGATCAAAGCCTAAAGCCATGTGTAAAATTAACCCCACTCTGTGTTACTCTAAATTGCTCTGATTTTAATGCTACTCAGGCCACTACTACTCAGGCC---------------------------------------------------------------------------------TATAATAGTAGCTGGAAGGTG------GAAGGAATGAAAAACTGCTCTTTCAATACCACC---TCAGGAATAAGAGAT------AAGGTGAAGAAAGAATATGCACTTCTTTATAAACTTGATATAGTAGAAATAAATGATGAT------------------------------AATAATACT---GGGGATAATACTAGCTATAGGTTCTATAGGTTGCTAAGTTGTAACACCTCGGTCATGACACAGGCCTGTCCAAAGGTATCCTTTGAGCCAATTCCCATACATTATTGTGCCCCAGCTGGTTTTGCGATTCTAAAGTGT---AATAATAAGACGTTCAATGGAACAGGACCGTGTACAAATGTCAGCACAGTACAATGTACACATGGAATTAGGCCAGTAGTATCAACTCAACTGCTGTTAAATGGCAGTCTAGCAGAAAAA---GAGATAGTAATTAGATCTGAAAATATCACGGACAATGCTAAGACCATAATAGTACAGTTGAATACAGCTGTAGAAATTAATTGTACAAGACCCAGCAACAATACAAGAAAAAGTATAAGTATAGGA------------CCAGGGAGA---GCATTTTATACAACAGGAGAGGTAATAGGAGATATAAGACAAGCACATTGCAACATT------AGTCAAGCAAAATGGAATAACACTCTAAGACAGGTGGCTATTAAATTAAGA---GAACAATTTCCG------------AATAAAACA---ATAATCTTTAATCAA---------TCCTCAGGAGGGGACCTAGAAATTGAAATGCACAGTTTTAATTGTGGAGGGGAATTTTTCTACTGCAATACAACAATACTGTTTAATAGTACTTGG---------------AATGAAACTAACCTGTTT------------------------AATGCCACTGAAGGA---------------AATAACACAGAA------------------------GTTATTACACTCCAATGCAGAATAAAACAAATTATAAACATGTGGCAGGAAGTAGGAAAAGCAATGTATGCCCCTCCCATCAGTGGACAAATTAACTGTTCATCAAACATTACAGGGCTGCTATTAACAAGAGATGGCGGTATTGGGAAC---------AATATCACC---------------------AATACCTCC---GAGATCTTCAGACCTATAGGAGGAAATATGAGGGACAATTGGAGA---AGTGAATTATATAAATATAAAGTAGTCAAAATTGAACCA---ATAGGAATAGCACCC---ACCAAGGCAAAGAGAAGAGTGGTGCAGAGAGAA---AAAAGAGCAGTG---GGA---ATAGGA---GCTATG---TTCCTT---GGG---------TTCTTGGGA---GCAGCAGGAAGCACTATGGGCGCAGCGTCAGTG---ACGCTGACGGTACAGGCCAGACTATTATTGTCTGGTATAGTGCAACAGCAGAACAATCTGCTGAGAGCTATTGAGGCGCAACAACATCTGTTGCAACTCACAGTCTGGGGCATCAAGCAGCTCCAGGCAAGA---GTCCTGGCTGTGGAAAGATACCTAAAGGATCAACAGCTCCTGGGGATTTGGGGTTGCTCTGGAAAACTCATTTGCACCACTGCTGTGCCTTGGAATACTAGTTGGAGT---------------------------AATAAATCTCTGGAAAAAATTTGGAAT---AACATGACCTGGATGGAGTGGGAAAGAGAAATAGAC------AATTATACAAACTTAATATATAACTTACTTGAAGACTCGCAAAACCAACAAGACAAGAATGAAAAAGAATTATTGGAATTAGATAAATGGGCAAATTTGTGGAATTGGTTTGACATAACAAAGTGGCTGTGGTATATAAAAATATTCATAATGATAGTAGGAGGCTTAGTAGGTTTACGAATAGTTTTTTCTGTACTTTCTATAGTGAATAGAGTTAGGCAGGGATACTCACCACTATCGTTTCAGACCCAC---TCCCCAGCCCCGAGGGGA------CCCGACAGGCCCGGAGGAACAGAAGAAGAAGGTGGAGAAAGAGACAGAGACAGATCCGGTCCATTTGTGAACGGATTCTTGACGCTCATCTGGGTCGACCTGAGGAGCCTGTGCCTCTTCTGTTACCACCGCTTGAGAGACTTACTCTTGATTCTAGCGAGGATTGTAGAACTTCTGGGACGCAGG---------------GGGTGGGAAATCCTCAAATATTGGTGG---AATATCCTACAGTATTGG---------------------------------------------------AGTCAGGAACTAAAGAATAGCGCTGTCAGCTTGCTCAACGCCATAGCCATAGCAGTAGCTGAGGGGACAGATAGGATTATAGAAATAGTACAAAGA------------------GGTTTTAGA---------------------AGAATAAGACAGGGCTTGGAAAGGGCTGTGCTATAA

2.1054.SPD.EU575279 ATG---------GAGATCAGGAGGAATTATCAGCACTTG---------TGGAGATGGGGC------------------------ACCATGCTCCTTGGGTTATTGATGATC------------TGTAATGCTGCA---------GAACAGTTGTGGGTCACAGTATATTATGGGGTACCTGTGTGGAGAGAAGCAAACACCACTCTATTTTGTGCATCAGATGCTAAATCCTATGATACAGAGGTACATAAT---GTTTGGGCCACACATGCCTGTGTACCTACAGACCCTAACCCACAAGAAGTGGTAATG---GGAAATGTGACAGAAAGTTTTAACATGTGGAAAAATCACATGGTAGAACAGATGCATGAGGATATAATTAGTTTATGGGATCAAAGCCTAAAGCCATGTGTAAGATTAACCCCACTTTGTGTTACTTTAAATTGCAGTAATTATGCTGGAACTAATACCACTGCTATTAATACT---------------------------------------------------------------------------AATACCACTGTCTGGGGGGAAAAGATGGACCCAGGAGAAATAAAAAACTGCTCTTTCAATATCGCC---ACACCCATAAAAGAT------AAGAGGCATCAAGAATATGCATTGTTTTATAAAAGTGATGTAGTACCAATAGATGAGGAT------------------------------AATGATACT---------------------ACCAGTTATAGGTTGATAAGTTGTAACACCTCAGTCATTACACAGGCCTGCCCAAAGGTATCCTTTGAACCAATTCCAATACATTATTGTGCCCCAGCTGGTTTTGCGATTCTAAAGTGT---AATAATAAGACGTTCAATGGAAGTGGACCATGTACAAATGTCAGCACAGTACAATGTACACATGGAATTAAGCCAGTGGTATCAACTCAACTGCTGCTAAATGGCAGTCTAGCAGAGGAG---GAGGTAGTAATTAGATCTGCAAATTTCACGGACAATGCTAAAACTATAATGATACAGCTGAAAGACCCTGTAGAAATTAGTTGTACAAGACCCAATAACAATACAATAAAAGGTATACATATAGGA------------CCAGGGAGA---GCATTTTATACAACAGGACAAGTAATAGGAGATATAAGAAAAGCATATTGTAACATT------AGTAGAGCAAAATGGAATCACACTTTAAGTCAGGTAGTTGAAAAATTAAGA---TTACAATTTCAG------------AATAAAACA---ATAGTCTTTAATCAA---------TCCTCAGGAGGGGACCCAGAAATTGTAATGCACACTTTCAATTGTGGAGGGGAGTTTTTCTATTGCAACTCAACACCACTGTTTAATAGTACTTGG---------------AATGATACAAAAGGGTCA------------------------AATAACACAGTAGGA---------------AATGACACA------------------------------ATCATACTTCAATGCAGGATAAAACAAATTATAAACATGTGGCAGGAAGTAGGCAAAGCAATGTATGCCCCTCCCATCAAAGGAAACATTAGCTGTTCATCAAATATTACAGGGCTGCTATTAACAAGAGATGGTGGTATTGTGGACAAT------AACGATACC---------------------------------GAGACCTTCAGACCTGGAGGAGGAGATATGAGGGACAATTGGAGA---AGTGAATTATATAAATATAAAGTAGTAAAAATTGAACCA---TTAGGAATAGCACCC---ACCAAGGCAAAGAGAAGAGTGGTGCAGAGAGAA---AAAAGAGCAATA---GGA---ATAGGA---GCTATG---TTCCTT---GGG---------TTCTTAGGA---GCAGCAGGAAGCACTATGGGCGCAGCGTCAATG---ACGCTGACGGTACAGGCCAGACAATTATTGTCTGGTATAGTGCAACAGCAGAACAATTTGCTGAGGGCTATTGAGGCGCAACAGCATCTGTTGCAACTCACAGTCTGGGGCGTCAAGCAGCTCCAGGCAAGA---GTCCTGGCTGTGGAAAGATACCTAAAGGATCAACAGCTCCTGGGGATTTGGGGTTGCTCTGGAAAACTCATCTGCACCACTACTGTGCCTTGGAATGTTAGTTGGAGT---------------------------AATAAATCTCTGGATAATATTTGGAAT---GAAATGACCTGGATGGAGTGGGAGAGAGAAATTGAC------AATTACACAGACATAATATACTCCTTAATTGAAGAATCACAGAACCAACAAGACAAGAATGAAAAAGAATTGTTGGAATTGGATAAATGGGACAGTTTGTGGAATTGGTTTAGCATAACAAAGTGGCTGTGGTATATAAGAATATTCATAATGATAGTAGGAGGCTTGGTAGGTTTAAGAATAGTTTTTGCTGTACTTTCTATAGTGAATAGAGTTAGGCAGGGATACTCACCCTTATCGTTTCAGACCCGC---CTCCCAACCCCGAGGGGA------CCCGACAGGCCCGAAGGAATCGAAGAAGAAGGTGGAGACAGAGACAGAGACAGATCCACGACATTAGTGCAAGGATTCTTAGCACTTATCTGGGTCGACCTGAGGAGCCTGTGCATTTTCATCTACCACCGCTTGAGAGACTTACTCTTGATTGTAACGAGGATTGTGGAACTTCTGGGACGCCGG---------------GGGTGGGAACTCCTCAAATATTGGTGG---AATCTCCTACAATATTGG---------------------------------------------------AGTCAGGAACTAAAGAATAGTGCTGTTAGCTTGCTCAACACCACAGCCGTAGCAGTAGCTGAGGGGACAGATAGGGTCATAGAAGCATTACAAAGA------------------GTTGGTAGAGGTATCCTTCATATACCTACAAGAATAAGACAGGGCTTAGAAAGGGCTTTGCTATAA

2.1054.SPD.EU575258 ATG---------GAGATCAGGAGGAATTATCAGCACTTG---------TGGAGATGGGGC------------------------ACCATGCTCCTTGGGTTATTGATGATC------------TGTAATGCTGCA---------GAACAGTTGTGGGTCACAGTATATTATGGGGTACCTGTGTGGAGAGAAGCAAACACCACTCTATTTTGTGCATCAGATGCTAAATCCTATGATACAGAGGTACATAAT---GTTTGGGCCACACATGCCTGTGTACCTACAGACCCTAACCCACAAGAAGTGGTAATG---GGAAATGTGACAGAAAGTTTTAACATGTGGAAAAATCACATGGTAGAACAGATGCATGAGGATATAATTAGTTTATGGGATCAAAGCCTAAAGCCATGTGTAAGATTAACCCCACTTTGTGTTACTTTAAATTGCAGTAATTATGCTGGAACTAATACCACTGCTATTAATACT---------------------------------------------------------------------------AATACCACTGTCTGGGGGGAAAAGATGGACCCAGGAGAAATAAAAAACTGCTCTTTCAATATCGCC---ACACCCATAAAAGAT------AAGAGGCATCAAGAATATGCATTGTTTTATAAAAGTGATGTAGTACCAATAGATGAGGAT------------------------------AATGATACT---------------------ACCAGTTATAGGTTGATAAGTTGTAACACCTCAGTCATTACACAGGCCTGCCCAAAGGTATCCTTTGAACCAATTCCAATACATTATTGTGCCCCAGCTGGTTTTGCGATTCTAAAGTGT---AATAATAAGACGTTCAATGGAAGTGGACCATGTACAAATGTCAGCACAGTACAATGTACACATGGAATTAAGCCAGTGGTATCAACTCAACTGCTGCTAAATGGCAGTCTAGCAGAGGAG---GAGGTAGTAATTAGATCTGCAAATTTCACGGACAATGCTAAAACTATAATGATACAGCTGAAAGACCCTGTAGAAATTAGTTGTACAAGACCCAATAACAATACAATAAAAGGTATACATATAGGA------------CCAGGGAGA---GCATTTTATACAACAGGACAAGTAATAGGAGATATAAGAAAAGCATATTGTAACATT------AGTAGAGCAAAATGGAATCACACTTTAAGTCAGGTAGTTGAAAAATTAAGA---TTACAATTTCAG------------AATAAAACA---ATAGTCTTTAATCAA---------TCCTCAGGAGGGGACCCAGAAATTGTAATGCACACTTTCAATTGTGGAGGGGAGTTTTTCTATTGCAACTCAACACCACTGTTTAATAGTACTTGG---------------AATGATACAAAAGGGTCA------------------------AATAACACAGTAGGA---------------AATGACACA------------------------------ATCATACTTCAATGCAGGATAAAACAAATTATAAACATGTGGCAGGAAGTAGGCAAAGCAATGTATGCCCCTCCCATCAAAGGAAACATTAGCTGTTCATCAAATATTACAGGGCTGCTATTAACAAGAGATGGTGGTATTGTGGACAAT------AACGATACC---------------------------------GAGACCTTCAGACCTGGAGGAGGAGATATGAGGGACAATTGGAGA---AGTGAATTATATAAATATAAAGTAGTAAAAATTGAACCA---TTAGGAATAGCACCC---ACCAAGGCAAAGAGAAGAGTGGTGCAGAGAGAA---AAAAGAGCAATA---GGA---ATAGGA---GCTATG---TTCCTT---GGG---------TTCTTAGGA---GCAGCAGGAAGCACTATGGGCGCAGCGTCAATG---ACGCTGACGGTACAGGCCAGACAATTATTGTCTGGTATAGTGCAACAGCAGAACAATTTGCTGAGGGCTATTGAGGCGCAACAGCATCTGTTGCAACTCACAGTCTGGGGCGTCAAGCAGCTCCAGGCAAGA---GTCCTGGCTGTGGAAAGATACCTAAAGGATCAACAGCTCCTGGGGATTTGGGGTTGCTCTGGAAAACTCATCTGCACCACTACTGTGCCTTGGAATGTTAGTTGGAGT---------------------------AATAAATCTCTGGATAATATTTGGAAT---GAAATGACCTGGATGGAGTGGGAGAGAGAAATTGAC------AATTACACAGACATAATATACTCCTTAATTGAAGAATCACAGAACCAACAAGACAAGAATGAAAAAGAATTGTTGGAATTGGATAAATGGGACAGTTTGTGGAATTGGTTTAGCATAACAAAGTGGCTGTGGTATATAAGAATATTCATAATGATAGTAGGAGGCTTGGTAGGTTTAAGAATAGTTTTTGCTGTACTTTCTATAGTGAATAGAGTTAGGCAGGGATACTCACCCTTATCGTTTCAGACCCGC---CTCCCAACCCCGAGGGGA------CCCGACAGGCCCGAAGGAATCGAAGAAGAAGGTGGAGACAGAGACAGAGACAGATCCACGACATTAGTGCAAGGATTCTTAGCACTTATCTGGGTCGACCTGAGGAGCCTGTGCATTTTCATCTACCACCGCTTGAGAGACTTACTCTTGATTGTAACGAGGATTGTGGAACTTCTGGGACGCCGG---------------GGGTGGGAACTCCTCAAATATTGGTGG---AATCTCCTACAATATTGG---------------------------------------------------AGTCAGGAACTAAAGAATAGTGCTGTTAGCTTGCTCAACACCACAGCCGTAGCAGTAGCTGAGGGGACAGATAGGGTCATAGAAGCATTACAAAGA------------------GTTGGTAGAGGTATCCTTCATATACCTACAAGAATAAGACAGGGCTTAGAAAGGGCTTTGCTATAA

2.1054.SPD.EU575244 ATG---------GAGATCAGGAGGAATTATCAGCACTTG---------TGGAGATGGGGC------------------------ACCATGCTCCTTGGGTTATTGATGATC------------TGTAATGCTGCA---------GAACAGTTGTGGGTCACAGTATATTATGGGGTACCTGTGTGGAGAGAAGCAAACACCACTCTATTTTGTGCATCAGATGCTAAATCCTATGATACAGAGGTACATAAT---GTTTGGGCCACACATGCCTGTGTACCTACAGACCCTAACCCACAAGAAGTGGTAATG---GGAAATGTGACAGAAAGTTTTAACATGTGGAAAAATCACATGGTAGAACAGATGCATGAGGATATAATTAGTTTATGGGATCAAAGCCTAAAGCCATGTGTAAGATTAACCCCACTTTGTGTTACTTTAAATTGCAGTAATTATGCTGGAACTAATACCACTGCTATTAATACT---------------------------------------------------------------------------AATACCACTGTCTGGGGGGAAAAGATGGACCCAGGAGAAATAAAAAACTGCTCTTTCAATATCGCC---ACACCCATAAAAGAT------AAGAGGCATCAAGAATATGCATTGTTTTATAAAAGTGATGTAGTACCAATAGATGAGGAT------------------------------AATGATACT---------------------ACCAGTTATAGGTTGATAAGTTGTAACACCTCAGTCATTACACAGGCCTGCCCAAAGGTATCCTTTGAACCAATTCCAATACATTATTGTGCCCCAGCTGGTTTTGCGATTCTAAAGTGT---AATAATAAGACGTTCAATGGAAGTGGACCATGTACAAATGTCAGCACAGTACAATGTACACATGGAATTAAGCCAGTGGTATCAACTCAACTGCTGCTAAATGGCAGTCTAGCAGAGGAG---GAGGTAGTAATTAGATCTGCAAATTTCACGGACAATGCTAAAACTATAATGATACAGCTGAAAGACCCTGTAGAAATTAGTTGTACAAGACCCAATAACAATACAATAAAAGGTATACATATAGGA------------CCAGGGAGA---GCATTTTATACAACAGGACAAGTAATAGGAGATATAAGAAAAGCATATTGTAACATT------AGTAGAGCAAAATGGAATCACACTTTAAGTCAGGTAGTTGAAAAATTAAGA---TTACAATTTCAG------------AATAAAACA---ATAGTCTTTAATCAA---------TCCTCAGGAGGGGACCCAGAAATTGTAATGCACACTTTCAATTGTGGAGGGGAGTTTTTCTATTGCAACTCAACACCACTGTTTAATAGTACTTGG---------------AATGATACAAAAGGGTCA------------------------AATAACACAGTAGGA---------------AATGACACA------------------------------ATCATACTTCAATGCAGGATAAAACAAATTATAAACATGTGGCAGGAAGTAGGCAAAGCAATGTATGCCCCTCCCATCAAAGGAAACATTAGCTGTTCATCAAATATTACAGGGCTGCTATTAACAAGAGATGGTGGTATTGTGGACAAT------AACGATACC---------------------------------GAGACCTTCAGACCTGGAGGAGGAGATATGAGGGACAATTGGAGA---AGTGAATTATATAAATATAAAGTAGTAAAAATTGAACCA---TTAGGAATAGCACCC---ACCAAGGCAAAGAGAAGAGTGGTGCAGAGAGAA---AAAAGAGCAATA---GGA---ATAGGA---GCTATG---TTCCTT---GGG---------TTCTTAGGA---GCAGCAGGAAGCACTATGGGCGCAGCGTCAATG---ACGCTGACGGTACAGGCCAGACAATTATTGTCTGGTATAGTGCAACAGCAGAACAATTTGCTGAGGGCTATTGAGGCGCAACAGCATCTGTTGCAACTCACAGTCTGGGGCGTCAAGCAGCTCCAGGCAAGA---GTCCTGGCTGTGGAAAGATACCTAAAGGATCAACAGCTCCTGGGGATTTGGGGTTGCTCTGGAAAACTCATCTGCACCACTACTGTGCCTTGGAATGTTAGTTGGAGT---------------------------AATAAATCTCTGGATAATATTTGGAAT---GAAATGACCTGGATGGAGTGGGAGAGAGAAATTGAC------AATTACACAGACATAATATACTCCTTAATTGAAGAATCACAGAACCAACAAGACAAGAATGAAAAAGAATTGTTGGAATTGGATAAATGGGACAGTTTGTGGAATTGGTTTAGCATAACAAAGTGGCTGTGGTATATAAGAATATTCATAATGATAGTAGGAGGCTTGGTAGGTTTAAGAATAGTTTTTGCTGTACTTTCTATAGTGAATAGAGTTAGGCAGGGATACTCACCCTTATCGTTTCAGACCCGC---CTCCCAACCCCGAGGGGA------CCCGACAGGCCCGAAGGAATCGAAGAAGAAGGTGGAGACAGAGACAGAGACAGATCCACGACATTAGTGCAAGGATTCTTAGCACTTATCTGGGTCGACCTGAGGAGCCTGTGCATTTTCATCTACCACCGCTTGAGAGACTTACTCTTGATTGTAACGAGGATTGTGGAACTTCTGGGACGCCGG---------------GGGTGGGAACTCCTCAAATATTGGTGG---AATCTCCTACAATATTGG---------------------------------------------------AGTCAGGAACTAAAGAATAGTGCTGTTAGCTTGCTCAACACCACAGCCGTAGCAGTAGCTGAGGGGACAGATAGGGTCATAGAAGCATTACAAAGA------------------GTTGGTAGAGGTATCCTTCATATACCTACAAGAATAAGACAGGGCTTAGAAAGGGCTTTGCTATAA

2.1054.SPD.EU575272 ATG---------GAGATCAGGAGGAATTATCAGCACTTG---------TGGAGATGGGGC------------------------ACCATGCTCCTTGGGTTATTGATGATC------------TGTAATGCTGCA---------GAACAGTTGTGGGTCACAGTATATTATGGGGTACCTGTGTGGAGAGAAGCAAACACCACTCTATTTTGTGCATCAGATGCTAAATCCTATGATACAGAGGTACATAAT---GTTTGGGCCACACATGCCTGTGTACCTACAGACCCTAACCCACAAGAAGTGGTAATG---GGAAATGTGACAGAAAGTTTTAACATGTGGAAAAATCACATGGTAGAACAGATGCATGAGGATATAATTAGTTTATGGGATCAAAGCCTAAAGCCATGTGTAAGATTAACCCCACTTTGTGTTACTTTAAATTGCAGTAATTATGCTGGAACTAATACCACTGCTATTAATACT---------------------------------------------------------------------------AATACCACTGTCTGGGGGGAAAAGATGGACCCAGGAGAAATAAAAAACTGCTCTTTCAATATCGCC---ACACCCATAAAAGAT------AAGAGGCATCAAGAATATGCATTGTTTTATAAAAGTGATGTAGTACCAATAGATGAGGAT------------------------------AATGATACT---------------------ACCAGTTATAGGTTGATAAGTTGTAACACCTCAGTCATTACACAGGCCTGCCCAAAGGTATCCTTTGAACCAATTCCAATACATTATTGTGCCCCAGCTGGTTTTGCGATTCTAAAGTGT---AATAATAAGACGTTCAATGGAAGTGGACCATGTACAAATGTCAGCACAGTACAATGTACACATGGAATTAAGCCAGTGGTATCAACTCAACTGCTGCTAAATGGCAGTCTAGCAGAGGAG---GAGGTAGTAATTAGATCTGCAAATTTCACGGACAATGCTAAAACTATAATGATACAGCTGAAAGACCCTGTAGAAATTAGTTGTACAAGACCCAATAACAATACAATAAAAGGTATACATATAGGA------------CCAGGGAGA---GCATTTTATACAACAGGACAAGTAATAGGAGATATAAGAAAAGCATATTGTAACATT------AGTAGAGCAAAATGGAATCACACTTTAAGTCAGGTAGTTGAAAAATTAAGA---TTACAATTTCAG------------AATAAAACA---ATAGTCTTTAATCAA---------TCCTCAGGAGGGGACCCAGAAATTGTAATGCACACTTTCAATTGTGGAGGGGAGTTTTTCTATTGCAACTCAACACCACTGTTTAATAGTACTTGG---------------AATGATACAAAAGGGTCA------------------------AATAACACAGTAGGA---------------AATGACACA------------------------------ATCATACTTCAATGCAGGATAAAACAAATTATAAACATGTGGCAGGAAGTAGGCAAAGCAATGTATGCCCCTCCCATCAAAGGAAACATTAGCTGTTCATCAAATATTACAGGGCTGCTATTAACAAGAGATGGTGGTATTGTGGACAAT------AACGATACC---------------------------------GAGACCTTCAGACCTGGAGGAGGAGATATGAGGGACAATTGGAGA---AGTGAATTATATAAATATAAAGTAGTAAAAATTGAACCA---TTAGGAATAGCACCC---ACCAAGGCAAAGAGAAGAGTGGTGCAGAGAGAA---AAAAGAGCAATA---GGA---ATAGGA---GCTATG---TTCCTT---GGG---------TTCTTAGGA---GCAGCAGGAAGCACTATGGGCGCAGCGTCAATG---ACGCTGACGGTACAGGCCAGACAATTATTGTCTGGTATAGTGCAACAGCAGAACAATTTGCTGAGGGCTATTGAGGCGCAACAGCATCTGTTGCAACTCACAGTCTGGGGCGTCAAGCAGCTCCAGGCAAGA---GTCCTGGCTGTGGAAAGATACCTAAAGGATCAACAGCTCCTGGGGATTTGGGGTTGCTCTGGAAAACTCATCTGCACCACTACTGTGCCTTGGAATGTTAGTTGGAGT---------------------------AATAAATCTCTGGATAATATTTGGAAT---GAAATGACCTGGATGGAGTGGGAGAGAGAAATTGAC------AATTACACAGACATAATATACTCCTTAATTGAAGAATCACAGAACCAACAAGACAAGAATGAAAAAGAATTGTTGGAATTGGATAAATGGGACAGTTTGTGGAATTGATTTAGCATAACAAAGTGGCTGTGGTATATAAGAATATTCATAATGATAGTAGGAGGCTTGGTAGGTTTAAGAATAGTTTTTGCTGTACTTTCTATAGTGAATAGAGTTAGGCAGGGATACTCACCCTTATCGTTTCAGACCCGC---CTCCCAACCCCGAGGGGA------CCCGACAGGCCCGAAGGAATCGAAGAAGAAGGTGGAGACAGAGACAGAGACAGATCCACGACATTAGTGCAAGGATTCTTAGCACTTATCTGGGTCGACCTGAGGAGCCTGTGCATTTTCATCTACCACCGCTTGAGAGACTTACTCTTGATTGTAACGAGGATTGTGGAACTTCTGGGACGCCGG---------------GGGTGGGAACTCCTCAAATATTGGTGG---AATCTCCTACAATATTGG---------------------------------------------------AGTCAGGAACTAAAGAATAGTGCTGTTAGCTTGCTCAACACCACAGCCGTAGCAGTAGCTGAGGGGACAGATAGGGTCATAGAAGCATTACAAAGA------------------GTTGGTAGAGGTATCCTTCATATACCTACAAGAATAAGACAGGGCTTAGAAAGGGCTTTGCTATAA

2.1054.SPD.EU575245 ATG---------GAGATCAGGAGGAATTATCAGCACTTG---------TGGAGATGGGGC------------------------ACCATGCTCCTTGGGTTATTGATGATC------------TGTAATGCTGCA---------GAACAGTTGTGGGTCACAGTATATTATGGGGTACCTGTGTGGAGAGAAGCAAACACCACTCTATTTTGTGCATCAGATGCTAAATCCTATGATACAGAGGTACATAAT---GTTTGGGCCACACATGCCTGTGTACCTACAGACCCTAACCCACAAGAAGTGGTAATG---GGAAATGTGACAGAAAGTTTTAACATGTGGAAAAATCACATGGTAGAACAGATGCATGAGGATATAATTAGTTTATGGGATCAAAGCCTAAAGCCATGTGTAAGATTAACCCCACTTTGTGTTACTTTAAATTGCAGTAATTATGCTGGAACTAATACCACTGCTATTAATACT---------------------------------------------------------------------------AATACCACTGTCTGGGGGGAAAAGATGGACCCAGGAGAAATAAAAAACTGCTCTTTCAATATCGCC---ACACCCATAAAAGAT------AAGAGGCATCAAGAATATGCATTGTTTTATAAAAGTGATGTAGTACCAATAGATGAGGAT------------------------------AATGATACT---------------------ACCAGTTATAGGTTGATAAGTTGTAACACCTCAGTCATTACACAGGCCTGCCCAAAGGTATCCTTTGAACCAATTCCAATACATTATTGTGCCCCAGCTGGTTTTGCGATTCTAAAGTGT---AATAATAAGACGTTCAATGGAAGTGGACCATGTACAAATGTCAGCACAGTACAATGTACACATGGAATTAAGCCAGTGGTATCAACTCAACTGCTGCTAAATGGCAGTCTAGCAGAGGAG---GAGGTAGTAATTAGATCTGCAAATTTCACGGACAATGCTAAAACTATAATGATACAGCTGAAAGACCCTGTAGAAATTAGTTGTACAAGACCCAATAACAATACAATAAAAGGTATACATATAGGA------------CCAGGGAGA---GCATTTTATACAACAGGACAAGTAATAGGAGATATAAGAAAAGCATATTGTAACATT------AGTAGAGCAAAATGGAATCACACTTTAAGTCAGGTAGTTGAAAAATTAAGA---TTACAATTTCAG------------AATAAAACA---ATAGTCTTTAATCAA---------TCCTCAGGAGGGGACCCAGAAATTGTAATGCACACTTTCAATTGTGGAGGGGAGTTTTTCTGTTGCAACTCAACACCACTGTTTAATAGTACTTGG---------------AATGATACAAAAGGGTCA------------------------AATAACACAGTAGGA---------------AATGACACA------------------------------ATCATACTTCAATGCAGGATAAAACAAATTATAAACATGTGGCAGGAAGTAGGCAAAGCAATGTATGCCCCTCCCATCAAAGGAAACATTAGCTGTTCATCAAATATTACAGGGCTGCTATTAACAAGAGATGGTGGTATTGTGGACAAT------AACGATACC---------------------------------GAGACCTTCAGACCTGGAGGAGGAGATATGAGGGACAATTGGAGA---AGTGAATTATATAAATATAAAGTAGTAAAAATTGAACCA---TTAGGAATAGCACCC---ACCAAGGCAAAGAGAAGAGTGGTGCAGAGAGAA---AAAAGAGCAATA---GGA---ATAGGA---GCTATG---TTCCTT---GGG---------TTCTTAGGA---GCAGCAGGAAGCACTATGGGCGCAGCGTCAATG---ACGCTGACGGTACAGGCCAGACAATTATTGTCTGGTATAGTGCAACAGCAGAACAATTTGCTGAGGGCTATTGAGGCGCAACAGCATCTGTTGCAACTCACAGTCTGGGGCGTCAAGCAGCTCCAGGCAAGA---GTCCTGGCTGTGGAAAGATACCTAAAGGATCAACAGCTCCTGGGGATTTGGGGTTGCTCTGGAAAACTCATCTGCACCACTACTGTGCCTTGGAATGTTAGTTGGAGT---------------------------AATAAATCTCTGGATAATATTTGGAAT---GAAATGACCTGGATGGAGTGGGAGAGAGAAATTGAC------AATTACACAGACATAATATACTCCTTAATTGAAGAATCACAGAACCAACAAGACAAGAATGAAAAAGAATTGTTGGAATTGGATAAATGGGACAGTTTGTGGAATTGGTTTAGCATAACAAAGTGGCTGTGGTATATAAGAATATTCATAATGATAGTAGGAGGCTTGGTAGGTTTAAGAATAGTTTTTGCTGTACTTTCTATAGTGAATAGAGTTAGGCAGGGATACTCACCCTTATCGTTTCAGACCCGC---CTCCCAACCCCGAGGGGA------CCCGACAGGCCCGAAGGAATCGAAGAAGAAGGTGGAGACAGAGACAGAGACAGATCCACGACATTAGTGCAAGGATTCTTAGCACTTATCTGGGTCGACCTGAGGAGCCTGTGCATTTTCATCTACCACCGCTTGAGAGACTTACTCTTGATTGTAACGAGGATTGTGGAACTTCTGGGACGCCGG---------------GGGTGGGAACTCCTCAAATATTGGTGG---AATCTCCTACAATATTGG---------------------------------------------------AGTCAGGAACTAAAGAATAGTGCTGTTAGCTTGCTCAACACCACAGCCGTAGCAGTAGCTGAGGGGACAGATAGGGTCATAGAAGCATTACAAAGA------------------GTTGGTAGAGGTATCCTTCATATACCTACAAGAATAAGACAGGGCTTAGAAAGGGCTTTGCTATAA

2.1054.SPD.EU575257 ATG---------GAGATCAGGAGGAATTATCAGCACTTG---------TGGAGATGGGGC------------------------ACCATGCTCCTTGGGTTATTGATGATC------------TGTAATGCTGCA---------GAACAGTTGTGGGTCACAGTATATTATGGGGTACCTGTGTGGAGAGAAGCAAACACCACTCTATTTTGTGCATCAGATGCTAAATCCTATGATACAGAGGTACATAAT---GTTTGGGCCACACATGCCTGTGTACCTACAGACCCTAACCCACAAGAAGTGGTAATG---GGAAATGTGACAGAAAGTTTTAACATGTGGAAAAATCACATGGTAGAACAGATGCATGAGGATATAATTAGTTTATGGGATCAAAGCCTAAAGCCATGTGTAAGATTAACCCCACTTTGTGTTACTTTAAATTGCAGTAATTATGCTGGAACTAATACCACTGCTATTAATACT---------------------------------------------------------------------------AATACCACTGTCTGGGGGGAAAAGATGGACCCAGGAGAAATAAAAAACTGCTCTTTCAATATCGCC---ACACCCATAAAAGAT------AAGAGGCATCAAGAATATGCATTGTTTTATAAAAGTGATGTAGTACCAATAGATGAGGAT------------------------------AATGATACT---------------------ACCAGTTATAGGTTGATAAGTTGTAACACCTCAGTCATTACACAGGCCTGCCCAAAGGTATCCTTTGAACCAATTCCAATACATTATTGTGCCCCAGCTGGTTTTGCGATTCTAAAGTGT---AATAATAAGACGTTCAATGGAAGTGGACCATGTACAAATGTCAGCACAGTACAATGTACACATGGAATTAAGCCAGTGGTATCAACTCAACTGCTGCTAAATGGCAGTCTAGCAGAGGAG---GAGGGAGTAATTAGATCTGCAAATTTCACGGACAATGCTAAAACTATAATGATACAGCTGAAAGACCCTGTAGAAATTAGTTGTACAAGACCCAATAACAATACAATAAAAGGTATACATATAGGA------------CCAGGGAGA---GCATTTTATACAACAGGACAAGTAATAGGAGATATAAGAAAAGCATATTGTAACATT------AGTAGAGCAAAATGGAATCACACTTTAAGTCAGGTAGTTGAAAAATTAAGA---TTACAATTTCAG------------AATAAAACA---ATAGTCTTTAATCAA---------TCCTCAGGAGGGGACCCAGAAATTGTAATGCACACTTTCAATTGTGGAGGGGAGTTTTTCTATTGCAACTCAACACCACTGTTTAATAGTACTTGG---------------AATGATACAAAAGGGTCA------------------------AATAACACAGTAGGA---------------AATGACACA------------------------------ATCATACTTCAATGCAGGATAAAACAAATTATAAACATGTGGCAGGAAGTAGGCAAAGCAATGTATGCCCCTCCCATCAAAGGAAACATTAGCTGTTCATCAAATATTACAGGGCTGCTATTAACAAGAGATGGTGGTATTGTGGACAAT------AACGATACC---------------------------------GAGACCTTCAGACCTGGAGGAGGAGATATGAGGGACAATTGGAGA---AGTGAATTATATAAATATAAAGTAGTAAAAATTGAACCA---TTAGGAATAGCACCC---ACCAAGGCAAAGAGAAGAGTGGTGCAGAGAGAA---AAAAGAGCAATA---GGA---ATAGGA---GCTATG---TTCCTT---GGG---------TTCTTAGGA---GCAGCAGGAAGCACTATGGGCGCAGCGTCAATG---ACGCTGACGGTACAGGCCAGACAATTATTGTCTGGTATAGTGCAACAGCAGAACAATTTGCTGAGGGCTATTGAGGCGCAACAGCATCTGTTGCAACTCACAGTCTGGGGCGTCAAGCAGCTCCAGGCAAGA---GTCCTGGCTGTGGAAAGATACCTAAAGGATCAACAGCTCCTGGGGATTTGGGGTTGCTCTGGAAAACTCATCTGCACCACTACTGTGCCTTGGAATGTTAGTTGGAGT---------------------------AATAAATCTCTGGATAATATTTGGAAT---GAAATGACCTGGATGGAGTGGGAGAGAGAAATTGAC------AATTACACAGACATAATATACTCCTTAATTGAAGAATCACAGAACCAACAAGACAAGAATGAAAAAGAATTGTTGGAATTGGATAAATGGGACAGTTTGTGGAATTGGTTTAGCATAACAAAGTGGCTGTGGTATATAAGAATATTTATAATGATAGTAGGAGGCTTGGTAGGTTTAAGAATAGTTTTTGCTGTACTTTCTATAGTGAATAGAGTTAGGCAGGGATACTCACCCTTATCGTTTCAGACCCGC---CTCCCAACCCCGAGGGGA------CCCGACAGGCCCGAAGGAATCGAAGAAGAAGGTGGAGACAGAGACAGAGACAGATCCACGACATTAGTGCAAGGATTCTTAGCACTTATCTGGGTCGACCTGAGGAGCCTGTGCATTTTCATCTACCACCGCTTGAGAGACTTACTCTTGATTGTAACGAGGATTGTGGAACTTCTGGGACGCCGG---------------GGGTGGGAACTCCTCAAATATTGGTGG---AATCTCCTACAATATTGG---------------------------------------------------AGTCAGGAACTAAAGAATAGTGCTGTTAGCTTGCTCAACACCACAGCCGTAGCAGTAGCTGAGGGGACAGATAGGGTCATAGAAGCATTACAAAGA------------------GTTGGTAGAGGTATCCTTCATATACCTACAAGAATAAGACAGGGCTTAGAAAGGGCTTTGCTATAA

2.1054.SPD.EU575251 ATG---------GAGATCAGGAGGAATTATCAGCACTTG---------TGGAGATGGGGC------------------------ACCATGCTCCTTGGGTTATTGATGATC------------TGTAATGCTGCA---------GAACAGTTGTGGGTCACAGTATATTATGGGGTACCTGTGTGGAGAGAAGCAAACACCACTCTATTTTGTGCATCAGATGCTAAATCCTATGATACAGAGGTACATAAT---GTTTGGGCCACACATGCCTGTGTACCTACAGACCCTAACCCACAAGAAGTGGTAATG---GGAAATGTGACAGAAAGTTTTAACATGTGGAAAAATCACATGGTAGAACAGATGCATGAGGATATAATTAGTTTATGGGATCAAAGCCTAAAGCCATGTGTAAGATTAACCCCACTTTGTGTTACTTTAAATTGCAGTAATTATGCTGGAACTAATACCACTGCTATTAATACT---------------------------------------------------------------------------AATACCACTGTCTGGGGGGAAAAGATGGACCCAGGAGAAATAAAAAACTGCTCTTTCAATATCGCC---ACACCCATAAAAGAT------AAGAGGCATCAAGAATATGCATTGTTTTATAAAAGTGATGTAGTACCAATAGATGAGGAT------------------------------AATGATACT---------------------ACCAGTTATAGGTTGATAAGTTGTAACACCTCAGTCATTACACAGGCCTGCCCAAAGGTATCCTTTGAACCAATTCCAATACATTATTGTGCCCCAGCTGGTTTTGCGATTCTAAAGTGT---AATAATAAGACGTTCAATGGAAGTGGACCATGTACAAATGTCAGCACAGTACAATGTACACATGGAATTAAGCCAGTGGTATCAACTCAACTGCTGCTAAATGGCAGTCTAGCAGAGGAG---GAGGTAGTAATTAGATCTGCAAATTTCACGGACAATGCTAAAACTATAATGATACAGCTGAAAGACCCTGTAGAAATTAGTTGTACAAGACCCAATAACAATACAATAAAAGGTATACATATAGGA------------CCAGGGAGA---GCATTTTATACAACAGGACAAGTAATAGGAGATATAAGAAAAGCATATTGTAACATT------AGTAGAGCAAAATGGAATCACACTTTAAGTCAGGTAGTTGAAAAATTAAGA---TTACAATTTCAG------------AATAAAACA---ATAGTCTTTAATCAA---------TCCTCAGGAGGGGACCCAGAAATTGTAATGCACACTTTCAATTGTGGAGGGGAGTTTTTCTATTGCAACTCAACACCACTGTTTAATAGTACTTGG---------------AATGATACAAAAGGGTCA------------------------AATAACACAGTAGGA---------------AATGACACA------------------------------ATCATACTTCAATGCAGGATAAAACAAATTATAAACATGTGGCAGGAAGTAGGCAAAGCAATGTATGCCCCTCCCATCAAAGGAAACATTAGCTGTTCATCAAATATTACAGGGCTGCTATTAACAAGAGATGGTGGTATTGTGGACAAT------AACGATACC---------------------------------GAGACCTTCAGACCTGGAGGAGGAGATATGAGGGACAATTGGAGA---AGTGAATTATATAAATATAAAGTAGTAAAAATTGAACCA---TTAGGAATAGCACCC---ACCAAGGCAAAGAGAAGAGTGGTGCAGAGAGAA---AAAAGAGCAATA---GGA---ATAGGA---GCTATG---TTCCTT---GGG---------TTCTTAGGA---GCAGCAGGAAGCACTATGGGCGCAGCGTCAATG---ACGCTGACGGTACAGGCCAGACAATTATTGTCTGGTATAGTGCAACAGCAGAACAATTTGCTGAGGGCTATTGAGGCGCAACAGCATCTGTTGCAACTCACAGTCTGGGGCGTCAAGCAGCTCCAGGCAAGA---GTCCTGGCTGTGGAAAGATACCTAAAGGATCAACAGCTCCTGGGGATTTGGGGTTGCTCTGGAAAACTCATCTGCACCACTACTGTGCCTTGGAATGTTAGTTGGAGT---------------------------AATAAATCTCTGGATAATATTTGGAAT---GAAATGACCTGGATGGAGTGGGAGAGAGAAATTGAC------AATTACACAGACATAATATACTCCTTAATTGAAGAATCACAGAACCAACAAGACAAGAATGAAAAAGAATTGTTGGAATTGGATAAATGGGACAGTTTGTGGAATTGGTTTAGCATAACAAAGTGGCTGTGGTATATAAGAATATTCATAATGATAGTAGGAGGCTTGGTAGGTTTAAGAATAGTTTTTGCTGTACTTTCTATAGTGAATAGAGTTAGGCAGGGATACTCACCCTTATCGTTTCAGACCCGC---CTCCCAACCCCGAGGGGA------CCCGACAGGCCCGAAGGAATCGAAGAAGAAGGTGGAGACAGAGACAGAGACAGATCCACGACATTAGTGCAAGGATTCTTAGCACTTATCTGGGTCGACCTGAGGAGCCTGTGCATTTTCATCTACCACCGCTTGAGAGACTTACTCTTGATTGTAACGAGGATTGTGGAACTTCTGGGACGCCGG---------------GGGTGGGAACTCCTCAAATATTGGTGG---AATCTCCTACAATATTGG---------------------------------------------------AGTCAGGAACTAAAGAATAGTGCTGTTAGCTTGCTCAACACCACAGCCGTAGCAGTAGCTGAGGGGACAGATAGGGTCATAGAAGCATTACAAAGA------------------GTTGGTAGAGGTATCCTTCATATACCTACAAGAATAAGACAGGGCTTAGAAAGGGCTTTGCTATAA

2.1054.SPD.EU575281 ATG---------GAGATCAGGAGGAATTATCAGCACTTG---------TGGAGATGGGGC------------------------ACCATGCTCCTTGGGTTATTGATGATC------------TGTAATGCTGCA---------GAACAGTTGTGGGTCACAGTATATTATGGGGTACCTGTGTGGAGAGAAGCAAACACCACTCTATTTTGTGCATCAGATGCTAAATCCTATGATACAGAGGTACATAAT---GTTTGGGCCACACATGCCTGTGTACCTACAGACCCTAACCCACAAGAAGTGGTAATG---GGAAATGTGACAGAAAGTTTTAACATGTGGAA-AATCACATGGTAGAACAGATGCATGAGGATATAATTAGTTTATGGGATCAAAGCCTAAAGCCATGTGTAAGATTAACCCCACTTTGTGTTACTTTAAATTGCAGTAATTATGCTGGAACTAATACCACTGCTATTAATACT---------------------------------------------------------------------------AATACCACTGTCTGGGGGGAAACGATGGACCCAGGAGAAATAAAAAACTGCTCTTTCAATATCGCC---ACACCCATAAAAGAT------AAGAGGCATCAAGAATATGCATTGTTTTATAAAAGTGATGTAGTACCAATAGATGAGGAT------------------------------AATGATACT---------------------ACCAGTTATAGGTTGATAAGTTGTAACACCTCAGTCATTACACAGGCCTGCCCAAAGGTATCCTTTGAACCAATTCCAATACATTATTGTGCCCCAGCTGGTTTTGCGATTCTAAAGTGT---AATAATAAGACGTTCAATGGAAGTGGACCATGTACAAATGTCAGCACAGTACAATGTACACATGGAATTAAGCCAGTGGTATCAACTCAACTGCTGCTAAATGGCAGTCTAGCAGAGGAG---GAGGTAGTAATTAGATCTGCAAATTTCACGGACAATGCTAAAACTATAATGATACAGCTGAAAGACCCTGTAGAAATTAGTTGTACAAGACCCAATAACAATACAATAAAAGGTATACATATAGGA------------CCAGGGAGA---GCATTTTATACAACAGGACAAGTAATAGGAGATATAAGAAAAGCATATTGTAACATT------AGTAGAGCAAAATGGAATCACACTTTAAGTCAGGTAGTTGAAAAATTAAGA---TTACAATTTCAG------------AATAAAACA---ATAGTCTTTAATCAA---------TCCTCAGGAGGGGACCCAGAAATTGTAATGCACACTTTCAATTGTGGAGGGGAGTTTTTCTATTGCAACTCAACACCACTGTTTAATAGTACTTGG---------------AATGATACAAAAGGGTCA------------------------AATAACACAGTAGGA---------------AATGACACA------------------------------ATCATACTTCAATGCAGGATAAAACAAATTATAAACATGTGGCAGGAAGTAGGCAAAGCAATGTATGCCCCTCCCATCAAAGGAAACATTAGCTGTTCATCAAATATTACAGGGCTGCTATTAACAAGAGATGGTGGTATTGTGGACAAT------AACGATACC---------------------------------GAGACCTTCAGACCTGGAGGAGGAGATATGAGGGACAATTGGAGA---AGTGAATTATATAAATATAAAGTAGTAAAAATTGAACCA---TTAGGAATAGCACCC---ACCAAGGCAAAGAGAAGAGTGGTGCAGAGAGAA---AAAAGAGCAATA---GGA---ATAGGA---GCTATG---TTCCTT---GGG---------TTCTTAGGA---GCAGCAGGAAGCACTATGGGCGCAGCGTCAATG---ACGCTGACGGTACAGGCCAGACAATTATTGTCTGGTATAGTGCAACAGCAGAACAATTTGCTGAGGGCTATTGAGGCGCAACAGCATCTGTTGCAACTCACAGTCTGGGGCGTCAAGCAGCTCCAGGCAAGA---GTCCTGGCTGTGGAAAGATACCTAAAGGATCAACAGCTCCTGGGGATTTGGGGTTGCTCTGGAAAACTCATCTGCACCACTACTGTGCCTTGGAATGTTAGTTGGAGT---------------------------AATAAATCTCTGGATAATATTTGGAAT---GAAATGACCTGGATGGAGTGGGAGGGAGAAATTGAC------AATTACACAGACATAATATACTCCTTAATTGAAGAATCACAGAACCAACAAGACAAGAATGAAAAAGAATTGTTGGAATTGGATAAATGGGACAGTTTGTGGAATTGGTTTAGCATAACAAAGTGGCTGTGGTATATAAGAATATTCATAATGATAGTAGGAGGCTTGGTAGGTTTAAGAATAGTTTTTGCTGTACTTTCTATAGTGAATAGAGTTAGGCAGGGATACTCACCCTTATCGTTTCAGACCCGC---CTCCCAACCCCGAGGGGA------CCCGACAGGCCCGAAGGAATCGAAGAAGAAGGTGGAGACAGAGACAGAGACAGATCCACGACATTAGTGCAAGGATTCTTAGCACTTATCTGGGTCGACCTGAGGAGCCTGTGCATTTTCATCTACCACCGCTTGAGAGACTTACTCTTGATTGTAACGAGGATTGTGGAACTTCTGGGACGCCGG---------------GGGTGGGAACTCCTCAAATATTGGTGG---AATCTCCTACAATATTGG---------------------------------------------------AGTCAGGAACTAAAGAATAGTGCTGTTAGCTTGCTCAACACCACAGCCGTAGCAGTAGCTGAGGGGACAGATAGGGTCATAGAAGCATTACAAAGA------------------GTTGGTAGAGGTATCCTTCATATACCTACAAGAATAAGACAGGGCTTAGAAAGGGCTTTGCTATAA

2.1054.SPD.EU575253 ATG---------GAGATCAGGAGGAATTATCAGCACTTG---------TGGAGATGGGGC------------------------ACCATGCTCCTTGGGTTATTGATGATC------------TGTAATGCTGCA---------GAACAGTTGTGGGTCACAGTATATTATGGGGTACCTGTGTGGAGAGAAGCAAACACCACTCTATTTTGTGCATCAGATGCTAAATCCTATGATACAGAGGTACATAAT---GTTTGGGCCACACATGCCTGTGTACCTACAGACCCTAACCCACAAGAAGTGGTAATG---GGAAATGTGACAGAAAGTTTTAACATGTGGAAAAATCACATGGTAGAACAGATGCATGAGGATATAATTAGTTTATGGGATCAAAGCCTAAAGCCATGTGTAAGATTAACCCCACTTTGTGTTACTTTAAATTGCAGTAATTATGCTGGAACTAATACCACTGCTATTAATACT---------------------------------------------------------------------------AATACCACTGTCTGGGGGGAAAAGATGGACCCAGGAGAAATAAAAAACTGCTCTTTCAATATCGCC---ACACCCATAAAAGAT------AAGAGGCATCAAGAATATGCATTGTTTTATAAAAGTGATGTAGTACCAATAGATGAGGAT------------------------------AATGATACT---------------------ACCAGTTATAGGTTGATAAGTTGTAACACCTCAGTCATTACACAGGCCTGCCCAAAGGTATCCTTTGAACCAATTCCAATACATTATTGTGCCCCAGCTGGTTTTGCGATTCTAAAGTGT---AATAATAAGACGTTCAATGGAAGTGGACCATGTACAAATGTCAGCACAGTACAATGTACACATGGAATTAAGCCAGTGGTATCAACTCAACTGCTGCTAAATGGCAGTCTAGCAGAGGAG---GAGGTAGTAATTAGATCTGCAAATTTCACGGACAATGCTAAAACTATAATGATACAGCTGAAAGACCCTGTAGAAATTAGTTGTACAAGACCCAATAACAATACAATAAAAGGTATACATATAGGA------------CCAGGGAGA---GCATTTTATACAACAGGACAAGTAATAGGAGATATAAGAAAAGCATATTGTAACATT------AGTAGAGCAAAATGGAATCACACTTTAAGTCAGGTAGTTGAAAAATTAAGA---TTACAATTTCAG------------AATAAAACA---ATAGTCTTTAATCAA---------TCCTCAGGAGGGGACCCAGAAATTGTAATGCACACTTTCAATTGTGGAGGGGAGTTTTTCTATTGCAACTCAACACCACTGTTTAATAGTACTTGG---------------AATGATACAAAAGGGTCA------------------------AATAACACAGTAGGA---------------AATGACACA------------------------------ATCATACTTCAATGCAGGATAAAACAAATTATAAACATGTGGCAGGAAGTAGGCAAAGCAATGTATGCCCCTCCCATCAAAGGAAACATTAGCTGTTCATCAAATATTACAGGGCTGCTATTAACAAGAGATGGTGGTATTGTGGACAAT------AACGATACC---------------------------------GAGACCTTCAGACCTGGAGGAGGAGATATGAGGGACAATTGGAGA---AGTGAATTATATAAATATAAAGTAGTAAAAATTGAACCA---TTAGGAATAGCACCC---ACCAAGGCAAAGAGAAAAGTGGTGCAGAGAGAA---AAAAGAGCAATA---GGA---ATAGGA---GCTATG---TTCCTT---GGG---------TTCTTAGGA---GCAGCAGGAAGCACTATGGGCGCAGCGTCAATG---ACGCTGACGGTACAGGCCAGACAATTATTGTCTGGTATAGTGCAACAGCAGAACAATTTGCTGAGGGCTATTGAGGCGCAACAGCATCTGTTGCAACTCACAGTCTGGGGCGTCAAGCAGCTCCAGGCAAGA---GTCCTGGCTGTGGAAAGATACCTAAAGGATCAACAGCTCCTGGGGATTTGGGGTTGCTCTGGAAAACTCATCTGCACCACTACTGTGCCTTGGAATGTTAGTTGGAGT---------------------------AATAAATCTCTGGATAATATTTGGAAT---AAAATGACCTGGATGGAGTGGGAGAGAGAAATTGAC------AATTACACAGACATAATATACTCCTTAATTGAAGAATCACAGAACCAACAAGACAAGAATGAAAAAGAATTGTTGGAATTGGATAAATGGGACAGTTTGTGGAATTGGTTTAGCATAACAAAGTGGCTGTGGTATATAAGAATATTCATAATGATAGTAGGAGGCTTGGTAGGTTTAAGAATAGTTTTTGCTGTACTTTCTATAGTGAATAGAGTTAGGCAGGGATACTCACCCTTATCGTTTCAGACCCGC---CTCCCAACCCCGAGGGGA------CCCGACAGGCCCGAAGGAATCGAAGAAGAAGGTGGAGACAGAGACAGAGACAGATCCACGACATTAGTGCAAGGATTCTTAGCACTTATCTGGGTCGACCTGAGGAGCCTGTGCATTTTCATCTACCACCGCTTGAGAGACTTACTCTTGATTGTAACGAGGATTGTGGAACTTCTGGGACGCCGG---------------GGGTGGGAACTCCTCAAATATTGGTGG---AATCTCCTACAATATTGG---------------------------------------------------AGTCAGGAACTAAAGAATAGTGCTGTTAGCTTGCTCAACACCACAGCCGTAGCAGTAGCTGAGGGGACAGATAGGGTCATAGAAGCATTACAAAGA------------------GTTGGTAGAGGTATCCTTCATATACCTACAAGAATAAGACAGGGCTTAGAAAGGGCTTTGCTATAA

2.1054.SPD.EU575256 ATG---------GAGATCAGGAGGAATTATCAGCACTTG---------TGGAGATGGGGC------------------------ACCATGCTCCTTGGGTTATTGATGATC------------TGTAATGCTGCA---------GAACAGTTGTGGGTCACAGTATATTATGGGGTACCTGTGTGGAGAGAAGCAAACACCACTCTATTTTGTGCATCAGATGCTAAATCCTATGATACAGAGGTACATAAT---GTTTGGGCCACACATGCCTGTGTACCTACAGACCCTAACCCACAAGAAGTGGTAATG---GGAAATGTGACAGAAAGTTTTAACATGTGGAAAAATCACATGGTAGAACAGATGCATGAGGATATAATTAGTTTATGGGATCAAAGCCTAAAGCCATGTGTAAGATTAACCCCACTTTGTGTTACTTTAAATTGCAGTAATTATGCTGGAACTAATACCACTGCTATTAATACT---------------------------------------------------------------------------AATACCACTGTCTGGGGGGAAAAGATGGACCCAGGAGAAATAAAAAACTGCTCTTTCAATATCGCC---ACACCCATAAAAGAT------AAGAGGCATCAAGAATATGCATTGTTTTATAAAAGTGATGTAGTACCAATAGATGAGGAT------------------------------AATGATACT---------------------ACCAGTTATAGGTTGATAAGTTGTAACACCTCAGTCATTACACAGGCCTGCCCAAAGGTATCCTTTGAACCAATTCCAATACATTATTGTGCCCCAGCTGGTTTTGCGATTCTAAAGTGT---AATAATAAGACGTTCAATGGAAGTGGACCATGTACAAATGTCAGCACAGTACAATGTACACATGGAATTAAGCCAGTGGTATCAACTCAACTGCTGCTAAATGGCAGTCTAGCAGAGGAG---GAGGTAGTAATTAGATCTGCAAATTTCACGGACAATGCTAAAACTATAATGATACAGCTGAAAGACCCTGTAGAAATTAGTTGTACAAGACCCAATAACAATACAATAAAAGGTATACATATAGGA------------CCAGGGAGA---GCATTTTATACAACAGGACAAGTAATAGGAGATATAAGAAAAGCATATTGTAACATT------AGTAGAGCAAAATGGAATCACACTTTAAGTCAGGTAGTTGAAAAATTAAGA---TTACAATTTCAG------------AATAAAACA---ATAGTCTTTAATCAA---------TCCTCAGGAGGGGACCCAGAAATTGTAATGCACACTTTCAATTGTGGAGGGGAGTTTTTCTATTGCAACTCAACACCACTGTTTAATAGTACTTGG---------------AATGATACAAAAGGGTCA------------------------AATAACACAGTAGGA---------------AATGACACA------------------------------ATCATACTTCAATGCAGGATAAAACAAATTATAAACATGTGGCAGGAAGTAGGCAAAGCAATGTATGCCCCTCCCATCAAAGGAAACATTAGCTGTTCATCAAATATTACAGGGCTGCTATTAACAAGAGATGGTGGTATTGTGGACAAT------AACGATACC---------------------------------GAGACCTTCAGACCTGGAGGAGGAGATATGAGGGACAATTGGAGA---AGTGAATTATATAAATATAAAGTAGTAAAAATTGAACCA---TTAGGAATAGCACCC---ACCAAGGCAAAGAGAAGAGTGGTGCAGAGAGAA---AAAAGAGCAATA---GGA---ATAGGA---GCTATG---TTCCTT---GGG---------TTCTTAGGA---GCAGCAGGAAGCACTATGGGCGCAGCGTCAATG---ACGCTGACGGTACAGGCCAGACAATTATTGTCTGGTATAGTGCAACAGCAGAACAATTTGCTGAGGGCTATTGAGGCGCAACAGCATCTGTTGCAACTCACAGTCTGGGGCGTCAAGCAGCTCCAGGCAAGA---GTCCTGGCTGTGGAAAGATACCTAAAGGATCAACAGCTCCTGGGGATTTGGGGTTGCTCTGGAAAACTCATCTGCACCACTACTGTGCCTTGGAATGTTAGTTGGAGT---------------------------AATAAATCTCTGGATAATATTTGGAAT---GAAATGACCTGGATGGAGTGGGAGAGAGAAATTGAC------AATTACACAGACATAATATACTCCTTAATTGAAGAATCACAGAACCAACAAGACAAGAATGAAAAAGAATTGTTGGAATTGGATAAATGGGACAGTTTGTGGAATTGGTTTAGCATAACAAAGTGGCTGTGGTATATAAGAATATTCATAATGATAGTAGGAGGCTTGGTAGGTTTAAGAATAGTTTTTGCTGTACTTTCTATAGTGAATAGAGTTAGGCAGGGATACTCACCCTTATCGTTTCAGACCCGC---CTCCCAACCCCGAGGGGA------CCCGACAGGCCCGAAGGAATCGAAGAAGAAGGTGGAGACAGAGACAGAGACAGATCCACGACATTAGTGCAAGGATTCTTAGCACTTATCTGGGTCGACCTGAGGAGCCTGTGCATTTTCATCTACCACCGCTTGAGAGACTTACTCTTGATTGTAACGAGGATTGTGGAACTTCTGGGACGCCGG---------------GGGTGGGAACTCCTCAAATATTGGTGG---AATCTCCTACAATATTGG---------------------------------------------------AGTCAGGAACTAAAGAATAGTGCTGTTAGCTTGCTCAACACCACAGCCGTAGCAGTAGCTGAGGGGACAGATAGGGTCATAGAAGCATTACAAAGA------------------GTTGGTAGAGGTATCCTTCATATACCTACAAGAATAAGACAGGGCTTAGAAAGGGCTTTGCTATAA

2.1054.SPD.EU575255 ATG---------GAGATCAGGAGGAATTATCAGCACTTG---------TGGAGATGGGGC------------------------ACCATGCTCCTTGGGTTATTGATGATC------------TGTAATGCTGCA---------GAACAGTTGTGGGTCACAGTATATTATGGGGTACCTGTGTGGAGAGAAGCAAACACCACTCTATTTTGTGCATCAGATGCTAAATCCTATGATACAGAGGTACATAAT---GTTTGGGCCACACATGCCTGTGTACCTACAGACCCTAACCCACAAGAAGTGGTAATG---GGAAATGTGACAGAAAGTTTTAACATGTGGAAAAATCACATGGTAGAACAGATGCATGAGGATATAATTAGTTTATGGGATCAAAGCCTAAAGCCATGTGTAAGATTAACCCCACTTTGTGTTACTTTAAATTGCAGTAATTATGCTGGAACTAATACCACTGCTATTAATACT---------------------------------------------------------------------------AATACCACTGTCTGGGGGGAAAAGATGGACCCAGGAGAAATAAAAAACTGCTCT--------CGCC---ACACCCATAAAAGAT------AAGAGGCATCAAGAATATGCATTGTTTTATAAAAGTGATGTAGTACCAATAGATGAGGAT------------------------------AATGATACT---------------------ACCAGTTATAGGTTGATAAGTTGTAACACCTCAGTCATTACACAGGCCTGCCCAAAGGTATCCTTTGAACCAATTCCAATACATTATTGTGCCCCAGCTGGTTTTGCGATTCTAAAGTGT---AATAATAAGACGTTCAATGGAAGTGGACCATGTACAAATGTCAGCACAGTACAATGTACACATGGAATTAAGCCAGTGGTATCAACTCAACTGCTGCTAAATGGCAGTCTAGCAGAGGAG---GAGGTAGTAATTAGATCTGCAAATTTCACGGACAATGCTAAAACTATAATGATACAGCTGAAAGACCCTGTAGAAATTAGTTGTACAAGACCCAATAACAATACAATAAAAGGTATACATATAGGA------------CCAGGGAGA---GCATTTTATACAACAGGACAAGTAATAGGAGATATAAGAAAAGCATATTGTAACATT------AGTAGAGCAAAATGGAATCACACTTTAAGTCAGGTAGTTGAAAAATTAAGA---TTACAATTTCAG------------AATAAAACA---ATAGTCTTTAATCAA---------TCCTCAGGAGGGGACCCAGAAATTGTAATGCACACTTTCAATTGTGGAGGGGAGTTTTTCTATTGCAACTCAACACCACTGTTTAATAGTACTTGG---------------AATGATACAAAAGGGTCA------------------------AATAACACAGTAGGA---------------AATGACACA------------------------------ATCATACTTCAATGCAGGATAAAACAAATTATAAACATGTGGCAGGAAGTAGGCAAAGCAATGTATGCCCCTCCCATCAAAGGAAACATTAGCTGTTCATCAAATATTACAGGGCTGCTATTAACAAGAGATGGTGGTATTGTGGACAAT------AACGATACC---------------------------------GAGACCTTCAGACCTGGAGGAGGAGATATGAGGGACAATTGGAGA---AGTGAATTATATAAATATAAAGTAGTAAAAATTGAACCA---TTAGGAATAGCACCC---ACCAAGGCAAAGAGAAGAGTGGTGCAGAGAGAA---AAAAGAGCAATA---GGA---ATAGGA---GCTATG---TTCCTT---GGG---------TTCTTAGGA---GCAGCAGGAAGCACTATGGGCGCAGCGTCAATG---ACGCTGACGGTACAGGCCAGACAATTATTGTCTGGTATAGTGCAACAGCAGAACAATTTGCTGAGGGCTATTGAGGCGCAACAGCATCTGTTGCAACTCACAGTCTGGGGCGTCAAGCAGCTCCAGGCAAGA---GTCCTGGCTGTGGAAAGATACCTAAAGGATCAACAGCTCCTGGGGATTTGGGGTTGCTCTGGAAAACTCATCTGCACCACTACTGTGCCTTGGAATGTTAGTTGGAGT---------------------------AATAAATCTCTGGATAATATTTGGAAT---GAAATGACCTGGATGGAGTGGGAGAGAGAAATTGAC------AATTACACAGACATAATATACTCCTTAATTGAAGAATCACAGAACCAACAAGACAAGAATGAAAAAGAATTGTTGGAATTGGATAAATGGGACAGTTTGTGGAATTGGTTTAGCATAACAAAGTGGCTGTGGTATATAAGAATATTCATAATGATAGTAGGAGGCTTGGTAGGTTTAAGAATAGTTTTTGCTGTACTTTCTATAGTGAATAGAGTTAGGCAGGGATACTCACCCTTATCGTTTCAGACCCGC---CTCCCAACCCCGAGGGGA------CCCGACAGGCCCGAAGGAATCGAAGAAGAAGGTGGAGACAGAGACAGAGACAGATCCACGACATTAGTGCAAGGATTCTTAGCACTTATCTGGGTCGACCTGAGGAGCCTGTGCATTTTCATCTACCACCGCTTGAGAGACTTACTCTTGATTGTAACGAGGATTGTGGAACTTCTGGGACGCCGG---------------GGGTGGGAACTCCTCAAATATTGGTGG---AATCTCCTACAATATTGG---------------------------------------------------AGTCAGGAACTAAAGAATAGTGCTGTTAGCTTGCTCAACACCACAGCCGTAGCAGTAGCTGAGGGGACAGATAGGGTCATAGAAGCATTACAAAGA------------------GTTGGTAGAGGTATCCTTCATATACCTACAAGAATAAGACAGGGCTTAGAAAGGGCTTTGCTATAA

2.1054.SPD.EU575254 ATG---------GAGATCAGGAGGAATTATCAGCACTTG---------TGGAGATGGGGC------------------------ACCATGCTCCTTGGGTTATTGATGATC------------TGTAATGCTGCA---------GAACAGTTGTGGGTCACAGTATATTATGGGGTACCTGTGTGGAGAGAAGCAAACACCACTCTATTTTGTGCATCAGATGCTAAATCCTATGATACAGAGGTACATAAT---GTTTGGGCCACACATGCCTGTGTACCTACAGACCCTAACCCACAAGAAGTGGTAATG---GGAAATGTGACAGAAAGTTTTAACATGTGGAAAAATCACATGGTAGAACAGATGCATGAGGATATAATTAGTTTATGGGATCAAAGCCTAAAGCCATGTGTAAGATTAACCCCACTTTGTGTTACTTTAAATTGCAGTAATTATGCTGGAACTAATACCACTGCTATTAATACT---------------------------------------------------------------------------AATACCACTGTCTGGGGGGAAAAGATGGACCCAGGAGAAATAAAAAACTGCTCTTTCAATATCGCC---ACACCCATAAAAGAT------AAGAGGCATCAAGAATATGCATTGTTTTATAAAAGTGATGTAGTACCAATAGATGAGGAT------------------------------AATGATACT---------------------ACCAGTTATAGGTTGATAAGTTGTAACACCTCAGTCATTACACAGGCCTGCCCAAAGGTATCCTTTGAACCAATTCCAATACATTATTGTGCCCCAGCTGGTTTTGCGATTCTAAAGTGT---AATAATAAGACGTTCAATGGAAGTGGACCATGTACAAATGTCAGCACAGTACAATGTACACATGGAATTAAGCCAGTGGTATCAACTCAACTGCTGCTAAATGGCAGTCTAGCAGAGGAG---GAGGTAGTAATTAGATCTGCAAATTTCACGGACAATGCTAAAACTATAATGATACAGCTGAAAGACCCTGTAGAAATTAGTTGTACAAGACCCAATAACAATACAATAAAAGGTATACATATAGGA------------CCAGGGAGA---GCATTTTATACAACAGGACAAGTAATAGGAGATATAAGAAAAGCATATTGTAACATT------AGTAGAGCAAAATGGAATCACACTTTAAGTCAGGTAGTTGAAAAATTAAGA---TTACAATTTCAG------------AATAAAACA---ATAGTCTTTAATCAA---------TCCTCAGGAGGGGACCCAGAAATTGTAATGCACACTTTCAATTGTGGAGGGGAGTTTTTCTATTGCAACTCAACACCACTGTTTAATAGTACTTGG---------------AATGATACAAAAGGGTCA------------------------AATAACACAGTAGGA---------------AATGACACA------------------------------ATCATACTTCAATGCAGGATAAAACAAATTATAAACATGTGGCAGGAAGTAGGCAAAGCAATGTATGCCCCTCCCATCAAAGGAAACATTAGCTGTTCATCAAATATTACAGGGCTGCTATTAACAAGAGATGGTGGTATTGTGGACAAT------AACGATACC---------------------------------GAGACCTTCAGACCTGGAGGAGGAGATATGAGGGACAATTGGAGA---AGTGAATTATATAAATATAAAGTAGTAAAAATTGAACCA---TTAGGAATAGCACCC---ACCAAGGCAAAGAGAAGAGTGGTGCAGAGAGAA---AAAAGAGCAATA---GGA---ATAGGA---GCTATG---TTCCTT---GGG---------TTCTTAGGA---GCAGCAGGAAGCACTATGGGCGCAGCGTCAATG---ACGCTGACGGTACAGGCCAGACAATTATTGTCTGGTATAGTGCAACAGCAGAACAATTTGCTGAGGGCTATTGAGGCGCAACAGCATCTGTTGCAACTCACAGTCTGGGGCGTCAAGCAGCTCCAGGCAAGA---GTCCTGGCTGTGGAAAGATACCTAAAGGATCAACAGCTCCTGGGGATTTGGGGTTGCTCTGGAAAACTCATCTGCACCACTACTGTGCCTTGGAATGTTAGTTGGAGT---------------------------AATAAATCTCTGGATAATATTTGGAAT---GAAATGACCTGGATGGAGTGGGAGAGAGAAATTGAC------AATTACACAGACATAATATACTCCTTAATTGAAGAATCACAGAACCAACAAGACAAGAATGAAAAAGAATTGTTGGAATTGGATAAATGGGACAGTTTGTGGAATTGGTTTAGCATAACAAAGTGGCTGTGGTATATAAGAATATTCATAATGATAGTAGGAGGCTTGGTAGGTTTAAGAATAGTTTTTGCTGTACTTTCTATAGTGAATAGAGTTAGGCAGGGATACTCACCCTTATCGTTTCAGACCCGC---CTCCCAACCCCGAGGGGA------CCCGACAGGCCCGAAGGAATCGAAGAAGAAGGTGGAGACAGAGACAGAGACAGATCCACGACATTAGTGCAAGGATTCTTAGCACTTATCTGGGTCGACCTGAGGAGCCTGTGCATTTTCATCTACCACCGCTTGAGAGACTTACTCTTGATTGTAACGAGGATTGTGGAACTTCTGGGACGCCGG---------------GGGTGGGAACTCCTCAAATATTGGTGG---AATCTCCTACAATATTGG---------------------------------------------------AGTCAGGAACTAAAGAATAGTGCTGTTAGCTTGCTCAACACCACAGCCGTAGCAGTAGCTGAGGGGACAGATAGGGTCATAGAAGCATTACAAAGA------------------GTTGGTAGAGGTATCCTTCATATACCTACAAGAATAAGACAGGGCTTAGAAAGGGCTTTGCTATAA

2.1054.SPD.EU575263 ATG---------GAGATCAGGAGGAATTATCAGCACTTG---------TGGAGATGGGGC------------------------ACCATGCTCCTTGGGTTATTGATGATC------------TGTAATGCTGCA---------GAACAGTTGTGGGTCACAGTATATTATGGGGTACCTGTGTGGAGAGAAGCAAACACCACTCTATTTTGTGCATCAGATGCTAAATCCTATGATACAGAGGTACATAAT---GTTTGGGCCACACATGCCTGTGTACCTACAGACCCTAACCCACAAGAAGTGGTAATG---GGAAATGTGACAGAAAGTTTTAACATGTGGAAAAATCACATGGTAGAACAGATGCATGAGGATATAATTAGTTTATGGGATCAAAGCCTAAAGCCATGTGTAAGATTAACCCCACTTTGTGTTACTTTAAATTGCAGTAATTATGCTGGAACTAATACCACTGCTATTAATACT---------------------------------------------------------------------------AATACCACTGTCTGGGGGGAAAAGATGGACCCAGGAGAAATAAAAAACTGCTCTTTCAATATCGCC---ACACCCATAAAAGAT------AAGAGGCATCAAGAATATGCATTGTTTTATAAAAGTGATGTAGTACCAATAGATGAGGAT------------------------------AATGATACT---------------------ACCAGTTATAGGTTGATAAGTTGTAACACCTCAGTCATTACACAGGCCTGCCCAAAGGTATCCTTTGAACCAATTCCAATACATTATTGTGCCCCAGCTGGTTTTGCGATTCTAAAGTGT---AATAATAAGACGTTCAATGGAAGTGGACCATGTACAAATGTCAGCACAGTACAATGTACACATGGAATTAAGCCAGTGGTATCAACTCAACTGCTGCTAAATGGCAGTCTAGCAGAGGAG---GAGGTAGTAATTAGATCTGCAAATTTCACGGACAATGCTAAAACTATAATGATACAGCTGAAAGACCCTGTAGAAATTAGTTGTACAAGACCCAATAACAATACAATAAAAGGTATACATATAGGA------------CCAGGGAGA---GCATTTTATACAACAGGACAAGTAATAGGAGATATAAGAAAAGCATATTGTAACATT------AGTAGAGCAAAATGGAATCACACTTTAAGTCAGGTAGTTGAAAAATTAAGA---TTACAATTTCAG------------AATAAAACA---ATAGTCTTTAATCAA---------TCCTCAGGAGGGGACCCAGAAATTGTAATGCACACTTTCAATTGTGGAGGGGAGTTTTTCTATTGCAACTCAACACCACTGTTTAATAGTACTTGG---------------AATGATACAAAAGGGTCA------------------------AATAACACAGTAGGA---------------AATGACACA------------------------------ATCATACTTCAATGCAGGATAAAACAAATTATAAACATGTGGCAGGAAGTAGGCAAAGCAATGTATGCCCCTCCCATCAAAGGAAACATTAGCTGTTCATCAAATATTACAGGGCTGCTATTAACAAGAGATGGTGGTATTGTGGACAAT------AACGATACC---------------------------------GAGACCTTCAGACCTGGAGGAGGAGATATGAGGGACAATTGGAGA---AGTGAATTATATAAATATAAAGTAGTAAAAATTGAACCA---TTAGGAATAGCACCC---ACCAAGGCAAAGAGAAGAGTGGTGCAGAGAGAA---AAAAGAGCAATA---GGA---ATAGGA---GCTATG---TTCCTT---GGG---------TTCTTAGGA---GCAGCAGGAAGCACTATGGGCGCAGCGTCAATG---ACGCTGACGGTACAGGCCAGACAATTATTGTCTGGTATAGTGCAACAGCAGAACAATTTGCTGAGGGCTATTGAGGCGCAACAGCATCTGTTGCAACTCACAGTCTGGGGCGTCAAGCAGCTCCAGGCAAGA---GTCCTGGCTGTGGAAAGATACCTAAAGGATCAACAGCTCCTGGGGATTTGGGGTTGCTCTGGAAAACTCATCTGCACCACTACTGTGCCTTGGAATGTTAGTTGGAGT---------------------------AATAAATCTCTGGATAATATTTGGAAT---GAAATGACCTGGATGGAGTGGGAGAGAGAAATTGAC------AATTACACAGACATAATATACTCCTTAATTGAAGAATCACAGAACCAACAAGACAAGAATGAAAAAGAATTGTTGGAATTGGATAAATGGGACAGTTTGTGGAATTGGTTTAGCATAACAAAGTGGCTGTGGTATATAAGAATATTCATAATGATAGTAGGAGGCTTGGTAGGTTTAAGAATAGTTTTTGCTGTACTTTCTATAGTGAATAGAGTTAGGCAGGGATACTCACCCTTATCGTTTCAGACCCGC---CTCCCAACCCCGAGGGGA------CCCGACAGGCCCGAAGGAATCGAAGAAGAAGGTGGAGACAGAGACAGAGACAGATCCACGACATTAGTGCAAGGATTCTTAGCACTTATCTGGGTCGACCTGAGGAGCCTGTGCATTTTCATCTACCACCGCTTGAGAGACTTACTCTTGATTGTAACGAGGATTGTGGAACTTCTGGGACGCCGG---------------GGGTGGGAACTCCTCAAATATTGGTGG---AATCTCCTACAATATTGG---------------------------------------------------AGTCAGGAACTAAAGAATAGTGCTGTTAGCTTGCTCAACACCACAGCCGTAGCAGTAGCTGAGGGGACAGATAGGGTCATAGAAGCATTACAAAGA------------------GTTGGTAGAGGTATCCTTCATATACCTACAAGAATAAGACAGGGCTTAGAAAGGGCTTTGCTATAA

2.1054.SPD.EU575246 ATG---------GAGATCAGGAGGAATTATCAGCACTTG---------TGGAGATGGGGC------------------------ACCATGCTCCTTGGGTTATTGATGATC------------TGTAATGCTGCA---------GAACAGTTGTGGGTCACAGTATATTATGGGGTACCTGTGTGGAGAGAAGCAAACACCACTCTATTTTGTGCATCAGATGCTAAATCCTATGATACAGAGGTACATAAT---GTTTGGGCCACACATGCCTGTGTACCTACAGACCCTAACCCACAAGAAGTGGTAATG---GGAAATGTGACAGAAAGTTTTAACATGTGGAAAAATCACATGGTAGAACAGATGCATGAGGATATAATTAGTTTATGGGATCAAAGCCTAAAGCCATGTGTAAGATTAACCCCACTTTGTGTTACTTTAAATTGCAGTAATTATGCTGGAACTAATACCACTGCTATTAATACT---------------------------------------------------------------------------AATACCACTGTCTGGGGGGAAAAGATGGACCCAGGAGAAATAAAAAACTGCTCTTTCAATATCGCC---ACACCCATAAAAGAT------AAGAGGCATCAAGAATATGCATTGTTTTATAAAAGTGATGTAGTACCAATAGATGAGGAT------------------------------AATGATACT---------------------ACCAGTTATAGGTTGATAAGTTGTAACACCTCAGTCATTACACAGGCCTGCCCAAAGGTATCCTTTGAACCAATTCCAATACATTATTGTGCCCCAGCTGGTTTTGCGATTCTAAAGTGT---AATAATAAGACGTTCAATGGAAGTGGACCATGTACAAATGTCAGCACAGTACAATGTACACATGGAATTAAGCCAGTGGTATCAACTCAACTGCTGCTAAATGGCAGTCTAGCAGAGGAG---GAGGTAGTAATTAGATCTGCAAATTTCACGGACAATGCTAAAACTATAATGATACAGCTGAAAGACCCTGTAGAAATTAGTTGTACAAGACCCAATAACAATACAATAAAAGGTATACATATAGGA------------CCAGGGAGA---GCATTTTATACAACAGGACAAGTAATAGGAGATATAAGAAAAGCATATTGTAACATT------AGTAGAGCAAAATGGAATCACACTTTAAGTCAGGTAGTTGAAAAATTAAGA---TTACAATTTCAG------------AATAAAACA---ATAGTCTTTAATCAA---------TCCTCAGGAGGGGACCCAGAAATTGTAATGCACACTTTCAATTGTGGAGGGGAGTTTTTCTATTGCAACTCAACACCACTGTTTAATAGTACTTGG---------------AATGATACAAAAGGGTCA------------------------AATAACACAGTAGGA---------------AATGACACA------------------------------ATCATACTTCAATGCAGGATAAAACAAATTATAAACATGTGGCAGGAAGTAGGCAAAGCAATGTATGCCCCTCCCATCAAAGGAAACATTAGCTGTTCATCAAATATTACAGGGCTGCTATTAACAAGAGATGGTGGTATTGTGGACAAT------AACGATACC---------------------------------GAGACCTTCAGACCTGGAGGAGGAGATATGAGGGACAATTGGAGA---AGTGAATTATATAAATATAAAGTAGTAAAAATTGAACCA---TTAGGAATAGCACCC---ACCAAGGCAAAGAGAAGAGTGGTGCAGAGAGAA---AAAAGAGCAATA---GGA---ATAGGA---GCTATG---TTCCTT---GGG---------TTCTTAGGA---GCAGCAGGAAGCACTATGGGCGCAGCGTCAATG---ACGCTGACGGTACAGGCCAGACAATTATTGTCTGGTATAGTGCAACAGCAGAACAATTTGCTGAGGGCTATTGAGGCGCAACAGCATCTGTTGCAACTCACAGTCTGGGGCGTCAAGCAGCTCCAGGCAAGA---GTCCTGGCTGTGGAAAGATACCTAAAGGATCAACAGCTCCTGGGGATTTGGGGTTGCTCTGGAAAACTCATCTGCACCACTACTGTGCCTTGGAATGTTAGTTGGAGT---------------------------AATAAATCTCTGGATAATATTTGGAAT---GAAATGACCTGGATGGAGTGGGAGAGAGAAATTGAC------AATTACACAGACATAATATACTCCTTAATTGAAGAATCACAGAACCAACAAGACAAGAATGAAAAAGAATTGTTGGAATTGGATAAATGGGACAGTTTGTGGAATTGGTTTAGCATAACAAAGTGGCTGTGGTATATAAGAATATTCATAATGATAGTAGGAGGCTTGGTAGGTTTAAGAATAGTTTTTGCTGTACTTTCTATAGTGAATAGAGTTAGGCAGGGATACTCACCCTTATCGTTTCAGACCCGC---CTCCCAACCCCGAGGGGA------CCCGACAGGCCCGAAGGAATCGAAGAAGAAGGTGGAGACAGAGACAGAGACAGATCCACGACATTAGTGCAAGGATTCTTAGCACTTATCTGGGTCGACCTGAGGAGCCTGTGCATTTTCATCTACCACCGCTTGAGAGACTTACTCTTGATTGTAACGAGGATTGTGGAACTTCTGGGACGCCGG---------------GGGTGGGAACTCCTCAAATATTGGTGG---AATCTCCTACAATATTGG---------------------------------------------------AGTCAGGAACTAAAGAATAGTGCTGTTAGCTTGCTCAACACCACAGCCGTAGCAGTAGCTGAGGGGACAGATAGGGTCATAGAAGCATTACAAAGA------------------GTTGGTAGAGGTATCCTTCATATACCTACAAGAATAAGACAGGGCTTAGAAAGGGCTTTGCTATAA

2.1054.SPD.EU575265 ATG---------GAGATCAGGAGGAATTATCAGCACTTG---------TGGAGATGGGGC------------------------ACCATGCTCCTTGGGTTATTGATGATC------------TGTAATGCTGCA---------GAACAGTTGTGGGTCACAGTATATTATGGGGTACCTGTGTGGAGAGAAGCAAACACCACTCTATTTTGTGCATCAGATGCTAAATCCTATGATACAGAGGTACATAAT---GTTTGGGCCACACATGCCTGTGTACCTACAGACCCTAACCCACAAGAAGTGGTAATG---GGAAATGTGACAGAAAGTTTTAACATGTGGAAAAATCACATGGTAGAACAGATGCATGAGGATATAATTAGTTTATGGGATCAAAGCCTAAAGCCATGTGTAAGATTAACCCCACTTTGTGTTACTTTAAATTGCAGTAATTATGCTGGAACTAATACCACTGCTATTAATACT---------------------------------------------------------------------------AATACCACTGTCTGGGGGGAAAAGATGGACCCAGGAGAAATAAAAAACTGCTCTTTCAATATCGCC---ACACCCATAAAAGAT------AAGAGGCATCAAGAATATGCATTGTTTTATAAAAGTGATGTAGTACCAATAGATGAGGAT------------------------------AATGATACT---------------------ACCAGTTATAGGTTGATAAGTTGTAACACCTCAGTCATTACACAGGCCTGCCCAAAGGTATCCTTTGAACCAATTCCAATACATTATTGTGCCCCAGCTGGTTTTGCGATTCTAAAGTGT---AATAATAAGACGTTCAATGGAAGTGGACCATGTACAAATGTCAGCACAGTACAATGTACACATGGAATTAAGCCAGTGGTATCAACTCAACTGCTGCTAAATGGCAGTCTAGCAGAGGAG---GAGGTAGTAATTAGATCTGCAAATTTCACGGACAATGCTAAAACTATAATGATACAGCTGAAAGACCCTGTAGAAATTAGTTGTACAAGACCCAATAACAATACAATAAAAGGTATACATATAGGA------------CCAGGGAGA---GCATTTTATACAACAGGACAAGTAATAGGAGATATAAGAAAAGCATATTGTAACATT------AGTAGAGCAAAATGGAATCACACTTTAAGTCAGGTAGTTGAAAAATTAAGA---TTACAATTTCAG------------AATAAAACA---ATAGTCTTTAATCAA---------TCCTCAGGAGGGGACCCAGAAATTGTAATGCACACTTTCAATTGTGGAGGGGAGTTTTTCTATTGCAACTCAACACCACTGTTTAATAGTACTTGG---------------AATGATACAAAAGGGTCA------------------------AATAACACAGTAGGA---------------AATGACACA------------------------------ATCATACTTCAATGCAGGATAAAACAAATTATAAACATGTGGCAGGAAGTAGGCAAAGCAATGTATGCCCCTCCCATCAAAGGAAACATTAGCTGTTCATCAAATATTACAGGGCTGCTATTAACAAGAGATGGTGGTATTGTGGACAAT------AACGATACC---------------------------------GAGACCTTCAGACCTGGAGGAGGAGATATGAGGGACAATTGGAGA---AGTGAATTATATAAATATAAAGTAGTAAAAATTGAACCA---TTAGGAATAGCACCC---ACCAAGGCAAAGAGAAGAGTGGTGCAGAGAGAA---AAAAGAGCAATA---GGA---ATAGGA---GCTATG---TTCCTT---GGG---------TTCTTAGGA---GCAGCAGGAAGCACTATGGGCGCAGCGTCAATG---ACGCTGACGGTACAGGCCAGACAATTATTGTCTGGTATAGTGCAACAGCAGAACAATTTGCTGAGGGCTATTGAGGCGCAACAGCATCTGTTGCAACTCACAGTCTGGGGCGTCAAGCAGCTCCAGGCAAGA---GTCCTGGCTGTGGAAAGATACCTAAAGGATCAACAGCTCCTGGGGATTTGGGGTTGCTCTGGAAAACTCATCTGCACCACTACTGTGCCTTGGAATGTTAGTTGGAGT---------------------------AATAAATCTCTGGATAATATTTGGAAT---GAAATGACCTGGATGGAGTGGGAGAGAGAAATTGAC------AATTACACAGACATAATATACTCCTTAATTGAAGAATCACAGAACCAACAAGACAAGAATGAAAAAGAATTGTTGGAATTGGATAAATGGGACAGTTTGTGGAATTGGTTTAGCATAACAAAGTGGCTGTGGTATATAAGAATATTCATAATGATAGTAGGAGGCTTGGTAGGTTTAAGAATAGTTTTTGCTGTACTTTCTATAGTGAATAGAGTTAGGCAGGGATACTCACCCTTATCGTTTCAGACCCGC---CTCCCAACCCCGAGGGGA------CCCGACAGGCCCGAAGGAATCGAAGAAGAAGGTGGAGACAGAGACAGAGACAGATCCACGACATTAGTGCAAGGATTCTTAGCACTTATCTGGGTCGACCTGAGGAGCCTGTGCATTTTCATCTACCACCGCTTGAGAGACTTACTCTTGATTGTAACGAGGATTGTGGAACTTCTGGGACGCCGG---------------GGGTGGGAACTCCTCAAATATTGGTGG---AATCTCCTACAATATTGG---------------------------------------------------AGTCAGGAACTAAAGAATAGTGCTGTTAGCTTGCTCAACACCACAGCCGTAGCAGTAGCTGAGGGGACAGATAGGGTCATAGAAGCATTACAAAGA------------------GTTGGTAGAGGTATCCTTCATATACCTACAAGAATAAGACAGGGCTTAGAAAGGGCTTTGCTATAA

2.1054.SPD.EU575249 ATG---------GAGATCAGGAGGAATTATCAGCACTTG---------TGGAGATGGGGC------------------------ACCATGCTCCTTGGGTTATTGATGATC------------TGTAATGCTGCA---------GAACAGTTGTGGGTCACAGTATATTATGGGGTACCTGTGTGGAGAGAAGCAAACACCACTCTATTTTGTGCATCAGATGCTAAATCCTATGATACAGAGGTACATAAT---GTTTGGGCCACACATGCCTGTGTACCTACAGACCCTAACCCACAAGAAGTGGTAATG---GGAAATGTGACAGAAAGTTTTAACATGTGGAAAAATCACATGGTAGAACAGATGCATGAGGATATAATTAGTTTATGGGATCAAAGCCTAAAGCCATGTGTAAGATTAACCCCACTTTGTGTTACTTTAAATTGCAGTAATTATGCTGGAACTAATACCACTGCTATTAATACT---------------------------------------------------------------------------AATACCACTGTCTGGGGGGAAAAGATGGACCCAGGAGAAATAAAAAACTGCTCTTTCAATATCGCC---ACACCCATAAAAGAT------AAGAGGCATCAAGAATATGCATTGTTTTATAAAAGTGATGTAGTACCAATAGATGAGGAT------------------------------AATGATACT---------------------ACCAGTTATAGGTTGATAAGTTGTAACACCTCAGTCATTACACAGGCCTGCCCAAAGGTATCCTTTGAACCAATTCCAATACATTATTGTGCCCCAGCTGGTTTTGCGATTCTAAAGTGT---AATAATAAGACGTTCAATGGAAGTGGACCATGTACAAATGTCAGCACAGTACAATGTACACATGGAATTAAGCCAGTGGTATCAACTCAACTGCTGCTAAATGGCAGTCTAGCAGAGGAG---GAGGTAGTAATTAGATCTGCAAATTTCACGGACAATGCTAAAACTATAATGATACAGCTGAAAGACCCTGTAGAAATTAGTTGTACAAGACCCAATAACAATACAATAAAAGGTATACATATAGGA------------CCAGGGAGA---GCATTTTATACAACAGGACAAGTAATAGGAGATATAAGAAAAGCATATTGTAACATT------AGTAGAGCAAAATGGAATCACACTTTAAGTCAGGTAGTTGAAAAATTAAGA---TTACAATTTCAG------------AATAAAACA---ATAGTCTTTAATCAA---------TCCTCAGGAGGGGACCCAGAAATTGTAATGCACACTTTCAATTGTGGAGGGGAGTTTTTCTATTGCAACTCAACACCACTGTTTAATAGTACTTGG---------------AATGATACAAAAGGGTCA------------------------AATAACACAGTAGGA---------------AATGACACA------------------------------ATCATACTTCAATGCAGGATAAAACAAATTATAAACATGTGGCAGGAAGTAGGCAAAGCAATGTATGCCCCTCCCATCAAAGGAAACATTAGCTGTTCATCAAATATTACAGGGCTGCTATTAACAAGAGATGGTGGTATTGTGGACAAT------AACGATACC---------------------------------GAGACCTTCAGACCTGGAGGAGGAGATATGAGGGACAATTGGAGA---AGTGAATTATATAAATATAAAGTAGTAAAAATTGAACCA---TTAGGAATAGCACCC---ACCAAGGCAAAGAGAAGAGTGGTGCAGAGAGAA---AAAAGAGCAATA---GGA---ATAGGA---GCTATG---TTCCTT---GGG---------TTCTTAGGA---GCAGCAGGAAGCACTATGGGCGCAGCGTCAATG---ACGCTGACGGTACAGGCCAGACAATTATTGTCTGGTATAGTGCAACAGCAGAACAATTTGCTGAGGGCTATTGAGGCGCAACAGCATCTGTTGCAACTCACAGTCTGGGGCGTCAAGCAGCTCCAGGCAGGA---GTCCTGGCTGTGGAAAGATACCTAAAGGATCAACAGCTCCTGGGGATTTGGGGTTGCTCTGGAAAACTCATCTGCACCACTACTGTGCCTTGGAATGTTAGTTGGAGT---------------------------AATAAATCTCTGGATAATATTTGGAAT---GAAATGACCTGGATGGAGTGGGAGAGAGAAATTGAC------AATTACACAGACATAATATACTCCTTAATTGAAGAATCACAGAACCAACAAGACAAGAATGAAAAAGAATTGTTGGAATTGGATAAATGGGACAGTTTGTGGAATTGGTTTAGCATAACAAAGTGGCTGTGGTATATAAGAATATTCATAATGATAGTAGGAGGCTTGGTAGGTTTAAGAATAGTTTTTGCTGTACTTTCTATAGTGAATAGAGTTAGGCAGGGATACTCACCCTTATCGTTTCAGACCCGC---CTCCCAACCCCGAGGGGA------CCCGACAGGCCCGAAGGAATCGAAGAAGAAGGTGGAGACAGAGACAGAGACAGATCCACGACATTAGTGCAAGGATTCTTAGCACTTATCTGGGTCGACCTGAGGAGCCTGTGCATTTTCATCTACCACCGCTTGAGAGACTTACTCTTGATTGTAACGAGGATTGTGGAACTTCTGGGACGCCGG---------------GGGTGGGAACTCCTCAAATATTGGTGG---AATCTCCTACAATATTGG---------------------------------------------------AGTCAGGAACTAAAGAATAGTGCTGTTAGCTTGCTCAACACCACAGCCGTAGCAGTAGCTGAGGGGACAGATAGGGTCATAGAAGCATTACAAAGA------------------GTTGGTAGAGGTATCCTTCATATACCTACAAGAATAAGACAGGGCTTAGAAAGGGCTTTGCTATAA

2.1054.SPD.EU575274 ATG---------GAGATCAGGAGGAATTATCAGCACTTG---------TGGAGATGGGGC------------------------ACCATGCTCCTTGGGTTATTGATGATC------------TGTAATGCTGCA---------GAACAGTTGTGGGTCACAGTATATTATGGGGTACCTGTGTGGAGAGAAGCAAACACCACTCTATTTTGTGCATCAGATGCTAAATCCTATGATACAGAGGTACATAAT---GTTTGGGCCACACATGCCTGTGTACCTACAGACCCTAACCCACAAGAAGTGGTAATG---GGAAATGTGACAGAAAGTTTTAACATGTGGAAAAATCACATGGTAGAACAGATGCATGAGGATATAATTAGTTTATGGGATCAAAGCCTAAAGCCATGTGTAAGATTAACCCCACTTTGTGTTACTTTAAATTGCAGTAATTATGCTGGAACTAATACCACTGCTATTAATACT---------------------------------------------------------------------------AATACCACTGTCTGGGGGGAAAAGATGGACCCAGGAGAAATAAAAAACTGCTCTTTCAATATCGCC---ACACCCATAAAAGAT------AAGAGGCATCAAGAATATGCATTGTTTTATAAAAGTGATGTAGTACCAATAGATGAGGAT------------------------------AATGATACT---------------------ACCAGTTATAGGTTGATAAGTTGTAACACCTCAGTCATTACACAGGCCTGCCCAAAGGTATCCTTTGAACCAATTCCAATACATTATTGTGCCCCAGCTGGTTTTGCGATTCTAAAGTGT---AATAATAAGACGTTCAATGGAAGTGGACCATGTACAAATGTCAGCACAGTACAATGTACACATGGAATTAAGCCAGTGGTATCAACTCAACTGCTGCTAAATGGCAGTCTAGCAGAGGAG---GAGGTAGTAATTAGATCTGCAAATTTCACGGACAATGCTAAAACTATAATGATACAGCTGAAAGACCCTGTAGAAATTAGTTGTACAAGACCCAATAACAATACAATAAAAGGTATACATATAGGA------------CCAGGGAGA---GCATTTTATACAACAGGACAAGTAATAGGAGATATAAGAAAAGCATATTGTAACATT------AGTAGAGCAAAATGGAATCACACTTTAAGTCAGGTAGTTGAAAAATTAAGA---TTACAATTTCAG------------AATAAAACA---ATAGTCTTTAATCAA---------TCCTCAGGAGGGGACCCAGAAATTGTAATGCACACTTTCAATTGTGGAGGGGAGTTTTTCTATTGCAACTCAACACCACTGTTTAATAGTACTTGG---------------AATGATACAAAAGGGTCA------------------------AATAACACAGTAGGA---------------AATGACACA------------------------------ATCATACTTCAATGCAGGATAAAACAAATTATAAACATGTGGCAGGAAGTAGGCAAAGCAATGTATGCCCCTCCCATCAAAGGAAACATTAGCTGTTCATCAAATATTACAGGGCTGCTATTAACAAGAGATGGTGGTATTGTGGACAAT------AACGATACC---------------------------------GAGACCTTCAGACCTGGAGGAGGAGATATGAGGGACAATTGGAGA---AGTGAATTATATAAATATAAAGTAGTAAAAATTGAACCA---TTAGGAATAGCACCC---ACCAAGGCAAAGAGAAGAGTGGTGCAGAGAGAA---AAAAGAGCAATA---GGA---ATAGGA---GCTATG---TTCCTT---GGG---------TTCTTAGGA---GCAGCAGGAAGCACTATGGGCGCAGCGTCAATG---ACGCTGACGGTACAGGCCAGACAATTATTGTCTGGTATAGTGCAACAGCAGAACAATTTGCTGAGGGCTATTGAGGCGCAACAGCATCTGTTGCAACTCACAGTCTGGGGCGTCAAGCAGCTCCAGGCAAGA---GTCCTGGCTGTGGAAAGATACCTAAAGGATCAACAGCTCCTGGGGATTTGGGGTTGCTCTGGAAAACTCATCTGCACCACTACTGTGCCTTGGAATGTTAGTTGGAGT---------------------------AATAAATCTCTGGATAATATTTGGAAT---GAAATGACCTGGATGGAGTGGGAGAGAGAAATTGAC------AATTACACAGACATAATATACTCCTTAATTGAAGAATCACAGAACCAACAAGACAAGAATGAAAAAGAATTGTTGGAATTGGATAAATGGGACAGTTTGTGGAATTGGTTTAGCATAACAAAGTGGCTGTGGTATATAAGAATATTCATAATGATAGTAGGAGGCTTGGTAGGTTTAAGAATAGTTTTTGCTGTACTTTCTATAGTGAATAGAGTTAGGCAGGGATACTCACCCTTATCGTTTCAGACCCGC---CTCCCAACCCCGAGGGGA------CCCGACAGGCCCGAAGGAATCGAAGAAGAAGGTGGAGACAGAGACAGAGACAGATCCACGACATTAGTGCAAGGATTCTTAGCACTTATCTGGGTCGACCTGAGGAGCCTGTGCATTTTCATCTACCACCGCTTGAGAGACTTACTCTTGATTGTAACGAGGATTGTGGAACTTCTGGGACGCCGG---------------GGGTGGGAACTCCTCAAATATTGGTGG---AATCTCCTACAATATTGG---------------------------------------------------AGTCAGGAACTAAAGAATAGTGCTGTTAGCTTGCTCAACACCACAGCCGTAGCAGTAGCTGAGGGGACAGATAGGGTCATAGAAGCATTACAAAGA------------------GTTGGTAGAGGTATCCTTCATATACCTACAAGAATAAGACAGGGCTTAGAAAGGGCTTTGCTATAA

2.1054.SPD.EU575278 ATG---------GAGATCAGGAGGAATTATCAGCACTTG---------TGGAGATGGGGC------------------------ACCATGCTCCTTGGGTTATTGATGATC------------TGTAATGCTGCA---------GAACAGTTGTGGGTCACAGTATATTATGGGGTACCTGTGTGGAGAGAAGCAAACACCACTCTATTTTGTGCATCAGATGCTAAATCCTATGATACAGAGGTACATAAT---GTTTGGGCCACACATGCCTGTGTACCTACAGACCCTAACCCACAAGAAGTGGTAATG---GGAAATGTGACAGAAAGTTTTAACATGTGGAAAAATCACATGGTAGAACAGATGCATGAGGATATAATTAGTTTATGGGATCAAAGCCTAAAGCCATGTGTAAGATTAACCCCACTTTGTGTTACTTTAAATTGCAGTAATTATGCTGGAACTAATACCACTGCTATTAATACT---------------------------------------------------------------------------AATACCACTGTCTGGGGGGAAAAGATGGACCCAGGAGAAATAAAAAACTGCTCTTTCAATATCGCC---ACACCCATAAAAGAT------AAGAGGCATCAAGAATATGCATTGTTTTATAAAAGTGATGTAGTACCAATAGATGAGGAT------------------------------AATGATACT---------------------ACCAGTTATAGGTTGATAAGTTGTAACACCTCAGTCATTACACAGGCCTGCCCAAAGGTATCCTTTGAACCAATTCCAATACATTATTGTGCCCCAGCTGGTTTTGCGATTCTAAAGTGT---AATAATAAGACGTTCAATGGAAGTGGACCATGTACAAATGTCAGCACAGTACAATGTACACATGGAATTAAGCCAGTGGTATCAACTCAACTGCTGCTAAATGGCAGTCTAGCAGAGGAG---GAGGTAGTAATTAGATCTGCAAATTTCACGGACAATGCTAAAACTATAATGATACAGCTGAAAGACCCTGTAGAAATTAGTTGTACAAGACCCAATAACAATACAATAAAAGGTATACATATAGGA------------CCAGGGAGA---GCATTTTATACAACAGGACAAGTAATAGGAGATATAAGAAAAGCATATTGTAACATT------AGTAGAGCAAAATGGAATCACACTTTAAGTCAGGTAGTTGAAAAATTAAGA---TTACAATTTCAG------------AATAAAACA---ATAGTCTTTAATCAA---------TCCTCAGGAGGGGACCCAGAAATTGTAATGCACACTTTCAATTGTGGAGGGGAGTTTTTCTATTGCAACTCAACACCACTGTTTAATAGTACTTGG---------------AATGATACAAAAGGGTCA------------------------AATAACACAGTAGGA---------------AATGACACA------------------------------ATCATACTTCAATGCAGGATAAAACAAATTATAAACATGTGGCAGGAAGTAGGCAAAGCAATGTATGCCCCTCCCATCAAAGGAAACATTAGCTGTTCATCAAATATTACAGGGCTGCTATTAACAAGAGATGGTGGTATTGTGGACAAT------AACGATACC---------------------------------GAGACCTTCAGACCTGGAGGAGGAGATATGAGGGACAATTGGAGA---AGTGAATTATATAAATATAAAGTAGTAAAAATTGAACCA---TTAGGAATAGCACCC---ACCAAGGCAAAGAGAAGAGTGGTGCAGAGAGAA---AAAAGAGCAATA---GGA---ATAGGA---GCTATG---TTCCTT---GGG---------TTCTTAGGA---GCAGCAGGAAGCACTATGGGCGCAGCGTCAATG---ACGCTGACGGTACAGGCCAGACAATTATTGTCTGGTATAGTGCAACAGCAGAACAATTTGCTGAGGGCTATTGAGGCGCAACAGCATCTGTTGCAACTCACAGTCTGGGGCGTCAAGCAGCTCCAGGCAAGA---GTCCTGGCTGTGGAAAGATACCTAAAGGATCAACAGCTCCTGGGGATTTGGGGTTGCTCTGGAAAACTCATCTGCACCACTACTGTGCCTTGGAATGTTAGTTGGAGT---------------------------AATAAATCTCTGGATAATATTTGGAAT---GAAATGACCTGGATGGAGTGGGAGAGAGAAATTGAC------AATTACACAGACATAATATACTCCTTAATTGAAGAATCACAGAACCAACAAGACAAGAATGAAAAAGAATTGTTGGAATTGGATAAATGGGACAGTTTGTGGAATTGGTTTAGCATAACAAAGTGGCTGTGGTATATAAGAATATTCATAATGATAGTAGGAGGCTTGGTAGGTTTAAGAATAGTTTTTGCTGTACTTTCTATAGTGAATAGAGTTAGGCAGGGATACTCACCCTTATCGTTTCAGACCCGC---CTCCCAACCCCGAGGGGA------CCCGACAGGCCCGAAGGAATCGAAGAAGAAGGTGGAGACAGAGACAGAGACAGATCCACGACATTAGTGCAAGGATTCTTAGCACTTATCTGGGTCGACCTGAGGAGCCTGTGCATTTTCATCTACCACCGCTTGAGAGACTTACTCTTGATTGTAACGAGGATTGTGGAACTTCTGGGACGCCGG---------------GGGTGGGAACTCCTCAAATATTGGTGG---AATCTCCTACAATATTGG---------------------------------------------------AGTCAGGAACTAAAGAATAGTGCTGTTAGCTTGCTCAACACCACAGCCGTAGCAGTAGCTGAGGGGACAGATAGGGTCATAGAAGCATTACAAAGA------------------GTTGGTAGAGGTATCCTTCATATACCTACAAGAATAAGACAGGGCTTAGAAAGGGCTTTGCTATAA

2.1054.SPD.EU575260 ATG---------GAGATCAGGAGGAATTATCAGCACTTG---------TGGAGATGGGGC------------------------ACCATGCTCCTTGGGTTATTGATGATC------------TGTAATGCTGCA---------GAACAGTTGTGGGTCACAGTATATTATGGGGTACCTGTGTGGAGAGAAGCAAACACCACTCTATTTTGTGCATCAGATGCTAAATCCTATGATACAGAGGTACATAAT---GTTTGGGCCACACATGCCTGTGTACCTACAGACCCTAACCCACAAGAAGTGGTAATG---GGAAATGTGACAGAAAGTTTTAACATGTGGAAAAATCACATGGTAGAACAGATGCATGAGGATATAATTAGTTTATGGGATCAAAGCCTAAAGCCATGTGTAAGATTAACCCCACTTTGTGTTACTTTAAATTGCAGTAATTATGCTGGAACTAATACCACTGCTATTAATACT---------------------------------------------------------------------------AATACCACTGTCTGGGGGGAAAAGATGGACCCAGGAGAAATAAAAAACTGCTCTTTCAATATCGCC---ACACCCATAAAAGAT------AAGAGGCATCAAGAATATGCATTGTTTTATAAAAGTGATGTAGTACCAATAGATGAGGAT------------------------------AATGATACT---------------------ACCAGTTATAGGTTGATAAGTTGTAACACCTCAGTCATTACACAGGCCTGCCCAAAGGTATCCTTTGAACCAATTCCAATACATTATTGTGCCCCAGCTGGTTTTGCGATTCTAAAGTGT---AATAATAAGACGTTCAATGGAAGTGGACCATGTACAAATGTCAGCACAGTACAATGTACACATGGAATTAAGCCAGTGGTATCAACTCAACTGCTGCTAAATGGCAGTCTAGCAGAGGAG---GAGGTAGTAATTAGATCTGCAAATTTCACGGACAATGCTAAAACTATAATGATACAGCTGAAAGACCCTGTAGAAATTAGTTGTACAAGACCCAATAACAATACAATAAAAGGTATACATATAGGA------------CCAGGGAGA---GCATTTTATACAACAGGACAAGTAATAGGAGATATAAGAAAAGCATATTGTAACATT------AGTAGAGCAAAATGGAATCACACTTTAAGTCAGGTAGTTGAAAAATTAAGA---TTACAATTTCAG------------AATAAAACA---ATAGTCTTTAATCAA---------TCCTCAGGAGGGGACCCAGAAATTGTAATGCACACTTTCAATTGTGGAGGGGAGTTTTTCTATTGCAACTCAACACCACTGTTTAATAGTACTTGG---------------AATGATACAAAAGGGTCA------------------------AATAACACAGTAGGA---------------AATGACACA------------------------------ATCATACTTCAATGCAGGATAAAACAAATTATAAACATGTGGCAGGAAGTAGGCAAAGCAATGTATGCCCCTCCCATCAAAGGAAACATTAGCTGTTCATCAAATATTACAGGGCTGCTATTAACAAGAGATGGTGGTATTGTGGACAAT------AACGATACC---------------------------------GAGACCTTCAGACCTGGAGGAGGAGATATGAGGGACAATTGGAGA---AGTGAATTATATAAATATAAAGTAGTAAAAATTGAACCA---TTAGGAATAGCACCC---ACCAAGGCAAAGAGAAGAGTGGTGCAGAGAGAA---AAAAGAGCAATA---GGA---ATAGGA---GCTATG---TTCCTT---GGG---------TTCTTAGGA---GCAGCAGGAAGCACTATGGGCGCAGCGTCAATG---ACGCTGACGGTACAGGCCAGACAATTATTGTCTGGTATAGTGCAACAGCAGAACAATTTGCTGAGGGCTATTGAGGCGCAACAGCATCTGTTGCAACTCACAGTCTGGGGCGTCAAGCAGCTCCAGGCAAGA---GTCCTGGCTGTGGAAAGATACCTAAAGGATCAACAGCTCCTGGGGATTTGGGGTTGCTCTGGAAAACTCATCTGCACCACTACTGTGCCTTGGAATGTTAGTTGGAGT---------------------------AATAAATCTCTGGATAATATTTGGAAT---GAAATGACCTGGATGGAGTGGGAGAGAGAAATTGAC------AATTACACAGACATAATATACTCCTTAATTGAAGAATCACAGAACCAACAAGACAAGAATGAAAAAGAATTGTTGGAATTGGATAAATGGGACAGTTTGTGGAATTGGTTTAGCATAACAAAGTGGCTGTGGTATATAAGAATATTCATAATGATAGTAGGAGGCTTGGTAGGTTTAAGAATAGTTTTTGCTGTACTTTCTATAGTGAATAGAGTTAGGCAGGGATACTCACCCTTATCGTTTCAGACCCGC---CTCCCAACCCCGAGGGGA------CCCGACAGGCCCGAAGGAATCGAAGAAGAAGGTGGAGACAGAGACAGAGACAGATCCACGACATTAGTGCAAGGATTCTTAGCACTTATCTGGGTCGACCTGAGGAGCCTGTGCATTTTCATCTACCACCGCTTGAGAGACTTACTCTTGATTGTAACGAGGATTGTGGAACTTCTGGGACGCCGG---------------GGGTGGGAACTCCTCAAATATTGGTGG---AATCTCCTACAATATTGG---------------------------------------------------AGTCAGGAACTAAAGAATAGTGCTGTTAGCTTGCTCAACACCACAGCCGTAGCAGTAGCTGAGGGGACAGATAGGGTCATAGAAGCATTACAAAGA------------------GTTGGTAGAGGTATCCTTCATATACCTACAAGAATAAGACAGGGCTTAGAAAGGGCTTTGCTATAA

2.1054.SPD.EU575270 ATG---------GAGATCAGGAGGAATTATCAGCACTTG---------TGGAGATGGGGC------------------------ACCATGCTCCTTGGGTTATTGATGATC------------TGTAATGCTGCA---------GAACAGTTGTGGGTCACAGTATATTATGGGGTACCTGTGTGGAGAGAAGCAAACACCACTCTATTTTGTGCATCAGATGCTAAATCCTATGATACAGAGGTACATAAT---GTTTGGGCCACACATGCCTGTGTACCTACAGACCCTAACCCACAAGAAGTGGTAATG---GGAAATGTGACAGAAAGTTTTAACATGTGGAAAAATCACATGGTAGAACAGATGCATGAGGATATAATTAGTTTATGGGATCAAAGCCTAAAGCCATGTGTAAGATTAACCCCACTTTGTGTTACTTTAAATTGCAGTAATTATGCTGGAACTAATACCACTGCTATTAATACT---------------------------------------------------------------------------AATACCACTGTCTGGGGGGAAAAGATGGACCCAGGAGAAATAAAAAACTGCTCTTTCAATATCGCC---ACACCCATAAAAGAT------AAGAGGCATCAAGAATATGCATTGTTTTATAAAAGTGATGTAGTACCAATAGATGAGGAT------------------------------AATGATACT---------------------ACCAGTTATAGGTTGATAAGTTGTAACACCTCAGTCATTACACAGGCCTGCCCAAAGGTATCCTTTGAACCAATTCCAATACATTATTGTGCCCCAGCTGGTTTTGCGATTCTAAAGTGT---AATAATAAGACGTTCAATGGAAGTGGACCATGTACAAATGTCAGCACAGTACAATGTACACATGGAATTAAGCCAGTGGTATCAACTCAACTGCTGCTAAATGGCAGTCTAGCAGAGGAG---GAGGTAGTAATTAGATCTGCAAATTTCACGGACAATGCTAAAACTATAATGATACAGCTGAAAGACCCTGTAGAAATTAGTTGTACAAGACCCAATAACAATACAATAAAAGGTATACATATAGGA------------CCAGGGAGA---GCATTTTATACAACAGGACAAGTAATAGGAGATATAAGAAAAGCATATTGTAACATT------AGTAGAGCAAAATGGAATCACACTTTAAGTCAGGTAGTTGAAAAATTAAGA---TTACAATTTCAG------------AATAAAACA---ATAGTCTTTAATCAA---------TCCTCAGGAGGGGACCCAGAAATTGTAATGCACACTTTCAATTGTGGAGGGGAGTTTTTCTATTGCAACTCAACACCACTGTTTAATAGTACTTGG---------------AATGATACAAAAGGGTCA------------------------AATAACACAGTAGGA---------------AATGACACA------------------------------ATCATACTTCAATGCAGGATAAAACAAATTATAAACATGTGGCAGGAAGTAGGCAAAGCAATGTATGCCCCTCCCATCAAAGGAAACATTAGCTGTTCATCAAATATTACAGGGCTGCTATTAACAAGAGATGGTGGTATTGTGGACAAT------AACGATACC---------------------------------GAGACCTTCAGACCTGGAGGAGGAGATATGAGGGACAATTGGAGA---AGTGAATTATATAAATATAAAGTAGTAAAAATTGAACCA---TTAGGAATAGCACCC---ACCAAGGCAAAGAGAAGAGTGGTGCAGAGAGAA---AAAAGAGCAATA---GGA---ATAGGA---GCTATG---TTCCTT---GGG---------TTCTTAGGA---GCAGCAGGAAGCACTATGGGCGCAGCGTCAATG---ACGCTGACGGTACAGGCCAGACAATTATTGTCTGGTATAGTGCAACAGCAGAACAATTTGCTGAGGGCTATTGAGGCGCAACAGCATCTGTTGCAACTCACAGTCTGGGGCGTCAAGCAGCTCCAGGCAAGA---GTCCTGGCTGTGGAAAGATACCTAAAGGATCAACAGCTCCTGGGGATTTGGGGTTGCTCTGGAAAACTCATCTGCACCACTACTGTGCCTTGGAATGTTAGTTGGAGT---------------------------AATAAATCTCTGGATAATATTTGGAAT---GAAATGACCTGGATGGAGTGGGAGAGAGAAATTGAC------AATTACACAGACATAATATACTCCTTAATTGAAGAATCACAGAACCAACAAGACAAGAATGAAAAAGAATTGTTGGAATTGGATAAATGGGACAGTTTGTGGAATTGGTTTAGCATAACAAAGTGGCTGTGGTATATAAGAATATTCATAATGATAGTAGGAGGCTTGGTAGGTTTAAGAATAGTTTTTGCTGTACTTTCTATAGTGAATAGAGTTAGGCAGGGATACTCACCCTTATCGTTTCAGACCCGC---CTCCCAACCCCGAGGGGA------CCCGACAGGCCCGAAGGAATCGAAGAAGAAGGTGGAGACAGAGACAGAGACAGATCCACGACATTAGTGCAAGGATTCTTAGCACTTATCTGGGTCGACCTGAGGAGCCTGTGCATTTTCATCTACCACCGCTTGAGAGACTTACTCTTGATTGTAACGAGGATTGTGGAACTTCTGGGACGCCGG---------------GGGTGGGAACTCCTCAAATATTGGTGG---AATCTCCTACAATATTGG---------------------------------------------------AGTCAGGAACTAAAGAATAGTGCTGTTAGCTTGCTCAACACCACAGCCGTAGCAGTAGCTGAGGGGACAGATAGGGTCATAGAAGCATTACAAAGA------------------GTTGGTAGAGGTATCCTTCATATACCTACAAGAATAAGACAGGGCTTAGAAAGGGCTTTGCTATAA

2.1054.SPD.EU575248 ATG---------GAGATCAGGAGGAATTATCAGCACTTG---------TGGAGATGGGGC------------------------ACCATGCTCCTGGGGTTATTGATGATC------------TGTAATGCTGCA---------GAACAGTTGTGGGTCACAGTATATTATGGGGTACCTGTGTGGAGAGAAGCAAACACCACTCTATTTTGTGCATCAGATGCTAAATCCTATGATACAGAGGTACATAAT---GTTTGGGCCACACATGCCTGTGTACCTACAGACCCTAACCCACAAGAAGTGGTAATG---GGAAATGTGACAGAAAGTTTTAACATGTGGAAAAATCACATGGTAGAACAGATGCATGAGGATATAATTAGTTTATGGGATCAAAGCCTAAAGCCATGTGTAAGATTAACCCCACTTTGTGTTACTTTAAATTGCAGTAATTATGCTGGAACTAATACCACTGCTATTAATACT---------------------------------------------------------------------------AATACCACTGTCTGGGGGGAAAAGATGGACCCAGGAGAAATAAAAAACTGCTCTTTCAATATCGCC---ACACCCATAAAAGAT------AAGAGGCATCAAGAATATGCATTGTTTTATAAAAGTGATGTAGTACCAATAGATGAGGAT------------------------------AATGATACT---------------------ACCAGTTATAGGTTGATAAGTTGTAACACCTCAGTCATTACACAGGCCTGCCCAAAGGTATCCTTTGAACCAATTCCAATACATTATTGTGCCCCAGCTGGTTTTGCGATTCTAAAGTGT---AATAATAAGACGTTCAATGGAAGTGGACCATGTACAAATGTCAGCACAGTACAATGTACACATGGAATTAAGCCAGTGGTATCAACTCAACTGCTGCTAAATGGCAGTCTAGCAGAGGAG---GAGGTAGTAATTAGATCTGCAAATTTCACGGACAATGCTAAAACTATAATGATACAGCTGAAAGACCCTGTAGAAATTAGTTGTACAAGACCCAATAACAATACAATAAAAGGTATACATATAGGA------------CCAGGGAGA---GCATTTTATACAACAGGACAAGTAATAGGAGATATAAGAAAAGCATATTGTAACATT------AGTAGAGCAAAATGGAATCACACTTTAAGTCAGGTAGTTGAAAAATTAAGA---TTACAATTTCAG------------AATAAAACA---ATAGTCTTTAATCAA---------TCCTCAGGAGGGGACCCAGAAATTGTAATGCACACTTTCAATTGTGGAGGGGAGTTTTTCTATTGCAACTCAACACCACTGTTTAATAGTACTTGG---------------AATGATACAAAAGGGTCA------------------------AATAACACAGTAGGA---------------AATGACACA------------------------------ATCATACTTCAATGCAGGATAAAACAAATTATAAACATGTGGCAGGAAGTAGGCAAAGCAATGTATGCCCCTCCCATCAAAGGAAACATTAGCTGTTCATCAAATATTACAGGGCTGCTATTAACAAGAGATGGTGGTATTGTGGACAAT------AACGATACC---------------------------------GAGACCTTCAGACCTGGAGGAGGAGATATGAGGGACAATTGGAGA---AGTGAATTATATAAATATAAAGTAGTAAAAATTGAACCA---TTAGGAATAGCACCC---ACCAAGGCAAAGAGAAGAGTGGTGCAGAGAGAA---AAAAGAGCAATA---GGA---ATAGGA---GCTATG---TTCCTT---GGG---------TTCTTAGGA---GCAGCAGGAAGCACTATGGGCGCAGCGTCAATG---ACGCTGACGGTACAGGCCAGACAATTATTGTCTGGTATAGTGCAACAGCAGAACAATTTGCTGAGGGCTATTGAGGCGCAACAGCATCTGTTGCAACTCACAGTCTGGGGCGTCAAGCAGCTCCAGGCAAGA---GTCCTGGCTGTGGAAAGATACCTAAAGGATCAACAGCTCCTGGGGATTTGGGGTTGCTCTGGAAAACTCATCTGCACCACTACTGTGCCTTGGAATGTTAGTTGGAGT---------------------------AATAAATCTCTGGATAATATTTGGAAT---GAAATGACCTGGATGGAGTGGGAGAGAGAAATTGAC------AATTACACAGACATAATATACTCCTTAATTGAAGAATCACAGAACCAACAAGACAAGAATGAAAAAGAATTGTTGGAATTGGATAAATGGGACAGTTTGTGGAATTGGTTTAGCATAACAAAGTGGCTGTGGTATATAAGAATATTCATAATGATAGTAGGAGGCTTGGTAGGTTTAAGAATAGTTTTTGCTGTACTTTCTATTGTGAATAGAGTTAGGCAGGGATACTCACCCTTATCGTTTCAGACCCGC---CTCCCAACCCCGAGGGGA------CCCGACAGGCCCGAAGGAATCGAAGAAGAAGGTGGAGACAGAGACAGAGACAGATCCACGACATTAGTGCAAGGATTCTTAGCACTTATCTGGGTCGACCTGAGGAGCCTGTGCATTTTCATCTACCACCGCTTGAGAGACTTACTCTTGATTGTAACGAGGATTGTGGAACTTCTGGGACGCCGG---------------GGGTGGGAACTCCTCAAATATTGGTGG---AATCTCCTACAATATTGG---------------------------------------------------AGTCAGGAACTAAAGAATAGTGCTGTTAGCTTGCTCAACACCACAGCCGTAGCAGTAGCTGAGGGGACAGATAGGGTCATAGAAGCATTACAAAGA------------------GTTGGTAGAGGTATCCTTCATATACCTACAAGAATAAGACAGGGCTTAGAAAGGGCTTTGCTATAA

2.1054.SPD.EU575271 ATG---------GAGATCAGGAGGAATTATCAGCACTTG---------TGGAGATGGGGC------------------------ACCATGCTCCTTGGGTTATTGATGATC------------TGTAATGCTGCA---------GAACAGTTGTGGGTCACAGTATATTATGGGGTACCTGTGTGGAGAGAAGCAAACACCACTCTATTTTGTGCATCAGATGCTAAATCCTATGATACAGAGGTACATAAT---GTTTGGGCCACACATGCCTGTGTACCTACAGACCCTAACCCACAAGAAGTGGTAATG---GGAAATGTGACAGAAAGTTTTAACATGTGGAAAAATCACATGGTAGAACAGATGCATGAGGATATAATTAGTTTATGGGATCAAAGCCTAAAGCCATGTGTAAGATTAACCCCACTTTGTGTTACTTTAAATTGCAGTAATTATGCTGGAACTAATACCACTGCTATTAATACT---------------------------------------------------------------------------AATACCACTGTCTGGGGGGAAAAGATGGACCCAGGAGAAATAAAAAACTGCTCTTTCAATATCGCC---ACACCCATAAAAGAT------AAGAGGCATCAAGAATATGCATTGTTTTATAAAAGTGATGTAGTACCAATAGATGAGGAT------------------------------AATGATACT---------------------ACCAGTTATAGGTTGATAAGTTGTAACACCTCAGTCATTACACAGGCCTGCCCAAAGGTATCCTTTGAACCAATTCCAATACATTATTGTGCCCCAGCTGGTTTTGCGATTCTAAAGTGT---AATAATAAGACGTTCAATGGAAGTGGACCATGTACAAATGTCAGCACAGTACAATGTACACATGGAATTAAGCCAGTGGTATCAACTCAACTGCTGCTAAATGGCAGTCTAGCAGAGGAG---GAGGTAGTAATTAGATCTGCAAATTTCACGGACAATGCTAAAACTATAATGATACAGCTGAAAGACCCTGTAGAAATTAGTTGTACAAGACCCAATAACAATACAATAAAAGGTATACATATAGGA------------CCAGGGAGA---GCATTTTATACAACAGGACAAGTAATAGGAGATATAAGAAAAGCATATTGTAACATT------AGTAGAGCAAAATGGAATCACACTTTAAGTCAGGTAGTTGAAAAATTAAGA---TTACAATTTCAG------------AATAAAACA---ATAGTCTTTAATCAA---------TCCTCAGGAGGGGACCCAGAAATTGTAATGCACACTTTCAATTGTGGAGGGGAGTTTTTCTATTGCAACTCAACACCACTGTTTAATAGTACTTGG---------------AATGATACAAAAGGGTCA------------------------AATAACACAGTAGGA---------------AATGACACA------------------------------ATCATACTTCAATGCAGGATAAAACAAATTATAAACATGTGGCAGGAAGTAGGCAAAGCAATGTATGCCCCTCCCATCAAAGGAAACATTAGCTGTTCATCAAATATTACAGGGCTGCTATTAACAAGAGATGGTGGTATTGTGGACAAT------AACGATACC---------------------------------GAGACCTTCAGACCTGGAGGAGGAGATATGAGGGACAATTGGAGA---AGTGAATTATATAAATATAAAGTAGTAAAAATTGAACCA---TTAGGAATAGCACCC---ACCAAGGCAAAGAGAAGAGTGGTGCAGAGAGAA---AAAAGAGCAATA---GGA---ATAGGA---GCTATG---TTCCTT---GGG---------TTCTTAGGA---GCAGCAGGAAGCACTATGGGCGCAGCGTCAATG---ACGCTGACGGTACAGGCCAGACAATTATTGTCTGGTATAGTGCAACAGCAGAACAATTTGCTGAGGGCTATTGAGGCGCAACAGCATCTGTTGCAACTCACAGTCTGGGGCGTCAAGCAGCTCCAGGCAAGA---GTCCTGGCTGTGGAAAGATACCTAAAGGATCAACAGCTCCTGGGGATTTGGGGTTGCTCTGGAAAACTCATCTGCACCACTACTGTGCCTTGGAATGTTAGTTGGAGT---------------------------AATAAATCTCTGGATAATATTTGGAAT---GAAATGACCTGGATGGAGTGGGAGAGAGAAATTGAC------AATTACACAGACATAATATACTCCTTAATTGAAGAATCACAGAACCAACAAGACAAGAATGAAAAAGAATTGTTGGAATTGGATAAATGGGACAGTTTGTGGAATTGGTTTAGCATAACAAAGTGGCTGTGGTATATAAGAATATTCATAATGATAGTAGGAGGCTTGGTAGGTTTAAGAATAGTTTTTGCTGTACTTTCTATAGTGAATAGAGTTAGGCAGGGATACTCACCCTTATCGTTTCAGACCCGC---CTCCCAACCCCGAGGGGA------CCCGACAGGCCCGAAGGAATCGAAGAAGAAGGTGGAGACAGAGACAGAGACAGATCCACGACATTAGTGCAAGGATTCTTAGCACTTATCTGGGTCGACCTGAGGAGCCTGTGCATTTTCATCTACCACCGCTTGAGAGACTTACTCTTGATTGTAACGAGGATTGTGGAACTTCTGGGACGCCGG---------------GGGTGGGAACTCCTCAAATATTGGTGG---AATCTCCTACAATATTGG---------------------------------------------------AGTCAGGAACTAAAGAATAGTGCTGTTAGCTTGCTCAACACCACAGCCGTAGCAGTAGCTGAGGGGACAGATAGGGTCATAGAAGCATTACAAAGA------------------GTTGGTAGAGGTATCCTTCATATACCTACAAGAATAAGACAGGGCTTAGAAAGGGCTTTGCTATAA

2.1054.SPD.EU575275 ATG---------GAGATCAGGAGGAATTATCAGCACTTG---------TGGAGATGGGGC------------------------ACCATGCTCCTTGGGTTATTGATGATC------------TGTAATGCTGCA---------GAACAGTTGTGGGTCACAGTATATTATGGGGTACCTGTGTGGAGAGAAGCAAACACCACTCTATTTTGTGCATCAGATGCTAAATCCTATGATACAGAGGTACATAAT---GTTTGGGCCACACATGCCTGTGTACCTACAGACCCTAACCCACAAGAAGTGGTAATG---GGAAATGTGACAGAAAGTTTTAACATGTGGAAAAATCACATGGTAGAACAGATGCATGAGGATATAATTAGTTTATGGGATCAAAGCCTAAAGCCATGTGTAAGATTAACCCCACTTTGTGTTACTTTAAATTGCAGTAATTATGCTGGAACTAATACCACTGCTATTAATACT---------------------------------------------------------------------------AATACCACTGTCTGGGGGGAAAAGATGGACCCAGGAGAAATAAAAAACTGCTCTTTCAATATCGCC---ACACCCATAAAAGAT------AAGAGGCATCAAGAATATGCATTGTTTTATAAAAGTGATGTAGTACCAATAGATGAGGAT------------------------------AATGATACT---------------------ACCAGTTATAGGTTGATAAGTTGTAACACCTCAGTCATTACACAGGCCTGCCCAAAGGTATCCTTTGAACCAATTCCAATACATTATTGTGCCCCAGCTGGTTTTGCGATTCTAAAGTGT---AATAATAAGACGTTCAATGGAAGTGGACCATGTACAAATGTCAGCACAGTACAATGTACACATGGAATTAAGCCAGTGGTATCAACTCAACTGCTGCTAAATGGCAGTCTAGCAGAGGAG---GAGGTAGTAATTAGATCTGCAAATTTCACGGACAATGCTAAAACTATAATGATACAGCTGAAAGACCCTGTAGAAATTAGTTGTACAAGACCCAATAACAATACAATAAAAGGTATACATATAGGA------------CCAGGGAGA---GCATTTTATACAACAGGACAAGTAATAGGAGATATAAGAAAAGCATATTGTAACATT------AGTAGAGCAAAATGGAATCACACTTTAAGTCAGGTAGTTGAAAAATTAAGA---TTACAATTTCAG------------AATAAAACA---ATAGTCTTTAATCAA---------TCCTCAGGAGGGGACCCAGAAATTGTAATGCACACTTTCAATTGTGGAGGGGAGTTTTTCTATTGCAACTCAACACCACTGTTTAATAGTACTTGG---------------AATGATACAAAAGGGTCA------------------------AATAACACAGTAGGA---------------AATGACACA------------------------------ATCATACTTCAATGCAGGATAAAACAAATTATAAACATGTGGCAGGAAGTAGGCAAAGCAATGTATGCCCCTCCCATCAAAGGAAACATTAGCTGTTCATCAAATATTACAGGGCTGCTATTAACAAGAGATGGTGGTATTGTGGACAAT------AACGATACC---------------------------------GAGACCTTCAGACCTGGAGGAGGAGATATGAGGGACAATTGGAGA---AGTGAATTATATAAATATAAAGTAGTAAAAATTGAACCA---TTAGGAATAGCACCC---ACCAAGGCAAAGAGAAGAGTGGTGCAGAGAGAA---AAAAGAGCAATA---GGA---ATAGGA---GCTATG---TTCCTT---GGG---------TTCTTAGGA---GCAGCAGGAAGCACTATGGGCGCAGCGTCAATG---ACGCTGACGGTACAGGCCAGACAATTATTGTCTGGTATAGTGCAACAGCAGAACAATTTGCTGAGGGCTATTGAGGCGCAACAGCATCTGTTGCAACTCACAGTCTGGGGCGTCAAGCAGCTCCAGGCAAGA---GTCCTGGCTGTGGAAAGATACCTAAAGGATCAACAGCTCCTGGGGATTTGGGGTTGCTCTGGAAAACTCATCTGCACCACTACTGTGCCTTGGAATGTTAGTTGGAGT---------------------------AATAAATCTCTGGATAATATTTGGAAT---GAAATGACCTGGATGGAGTGGGAGAGAGAAATTGAC------AATTACACAGACATAATATACTCCTTAATTGAAGAATCACAGAACCAACAAGACAAGAATGAAAAAGAATTGTTGGAATTGGATAAATGGGACAGTTTGTGGAATTGGTTTAGCATAACAAAGTGGCTGTGGTATATAAGAATATTCATAATGATAGTAGGAGGCTTGGTAGGTTTAAGAATAGTTTTTGCTGTACTTTCTATAGTGAATAGAGTTAGGCAGGGATACTCACCCTTATCGTTTCAGACCCGC---CTCCCAACCCCGAGGGGA------CCCGACAGGCCCGAAGGAATCGAAGAAGAAGGTGGAGACAGAGACAGAGACAGATCCACGACATTAGTGCAAGGATTCTTAGCACTTATCTGGGTCGACCTGAGGAGCCTGTGCATTTTCATCTACCACCGCTTGAGAGACTTACTCTTGATTGTAACGAGGATTGTGGAACTTCTGGGACGCCGG---------------GGGTGGGAACTCCTCAAATATTGGTGG---AATCTCCTACAATATTGG---------------------------------------------------AGTCAGGAACTAAAGAATAGTGCTGTTAGCTTGCTCAACACCACAGCCGTAGCAGTAGCTGAGGGGACAGATAGGGTCATAGAAGCATTACAAAGA------------------GTTGGTAGAGGTATCCTTCATATACCTACAAGAATAAGACAGGGCTTAGAAAGGGCTTTGCTATAA

2.1054.SPD.EU575247 ATG---------GAGATCAGGAGGAATTATCAGCACTTG---------TGGAGATGGGGC------------------------ACCATGCTCCTTGGGTTATTGATGATC------------TGTAATGCTGCA---------GAACAGTTGTGGGTCACAGTATATTATGGGGTACCTGTGTGGAGAGAAGCAAACACCACTCTATTTTGTGCATCAGATGCTAAATCCTATGATACAGAGGTACATAAT---GTTTGGGCCACACATGCCTGTGTACCTACAGACCCTAACCCACAAGAAGTGGTAATG---GGAAATGTGACAGAAAGTTTTAACATGTGGAAAAATCACATGGTAGAACAGATGCATGAGGATATAATTAGTTTATGGGATCAAAGCCTAAAGCCATGTGTAAGATTAACCCCACTTTGTGTTACTTTAAATTGCAGTAATTATGCTGGAACTAATACCACTGCTATTAATACT---------------------------------------------------------------------------AATACCACTGTCTGGGGGGAAAAGATGGACCCAGGAGAAATAAAAAACTGCTCTTTCAATATCGCC---ACACCCATAAAAGAT------AAGAGGCATCAAGAATATGCATTGTTTTATAAAAGTGATGTAGTACCAATAGATGAGGAT------------------------------AATGATACT---------------------ACCAGTTATAGGTTGATAAGTTGTAACACCTCAGTCATTACACAGGCCTGCCCAAAGGTATCCTTTGAACCAATTCCAATACATTATTGTGCCCCAGCTGGTTTTGCGATTCTAAAGTGT---AATAATAAGACGTTCAATGGAAGTGGACCATGTACAAATGTCAGCACAGTACAATGTACACATGGAATTAAGCCAGTGGTATCAACTCAACTGCTGCTAAATGGCAGTCTAGCAGAGGAG---GAGGTAGTAATTAGATCTGCAAATTTCACGGACAATGCTAAAACTATAATGATACAGCTGAAAGACCCTGTAGAAATTAGTTGTACAAGACCCAATAACAATACAATAAAAGGTATACATATAGGA------------CCAGGGAGA---GCATTTTATACAACAGGACAAGTAATAGGAGATATAAGAAAAGCATATTGTAACATT------AGTAGAGCAAAATGGAATCACACTTTAAGTCAGGTAGTTGAAAAATTAAGA---TTACAATTTCAG------------AATAAAACA---ATAGTCTTTAATCAA---------TCCTCAGGAGGGGACCCAGAAATTGTAATGCACACTTTCAATTGTGGAGGGGAGTTTTTCTATTGCAACTCAACACCACTGTTTAATAGTACTTGG---------------AATGATACAAAAGGGTCA------------------------AATAACACAGTAGGA---------------AATGACACA------------------------------ATCATACTTCAATGCAGGATAAAACAAATTATAAACATGTGGCAGGAAGTAGGCAAAGCAATGTATGCCCCTCCCATCAAAGGAAACATTAGCTGTTCATCAAATATTACAGGGCTGCTATTAACAAGAGATGGTGGTATTGTGGACAAT------AACGATACC---------------------------------GAGACCTTCAGACCTGGAGGAGGAGATATGAGGGACAATTGGAGA---AGTGAATTATATAAATATAAAGTAGTAAAAATTGAACCA---TTAGGAATAGCACCC---ACCAAGGCAAAGAGAAGAGTGGTGCAGAGAGAA---AAAAGAGCAATA---GGA---ATAGGA---GCTATG---TTCCTT---GGG---------TTCTTAGGA---GCAGCAGGAAGCACTATGGGCGCAGCGTCAATG---ACGCTGACGGTACAGGCCAGACAATTATTGTCTGGTATAGTGCAACAGCAGAACAATTTGCTGAGGGCTATTGAGGCGCAACAGCATCTGTTGCAACTCACAGTCTGGGGCGTCAAGCAGCTCCAGGCAAGA---GTCCTGGCTGTGGAAAGATACCTAAAGGATCAACAGCTCCTGGGGATTTGGGGTTGCTCTGGAAAACTCATCTGCACCACTACTGTGCCTTGGAATGTTAGTTGGAGT---------------------------AATAAATCTCTGGATAATATTTGGAAT---GAAATGACCTGGATGGAGTGGGAGAGAGAAATTGAC------AATTACACAGACATAATATACTCCTTAATTGAAGAATCACAGAACCAACAAGACAAGAATGAAAAAGAATTGTTGGAATTGGATAAATGGGACAGTTTGTGGAATTGGTTTAGCATAACAAAGTGGCTGTGGTATATAAGAATATTCATAATGATAGTAGGAGGCTTGGTAGGTTTAAGAATAGTTTTTGCTGTACTTTCTATAGTGAATAGAGTTAGGCAGGGATACTCACCCTTATCGTTTCAGACCCGC---CTCCCAACCCCGAGGGGA------CCCGACAGGCCCGAAGGAATCGAAGAAGAAGGTGGAGACAGAGACAGAGACAGATCCACGACATTAGTGCAAGGATTCTTAGCACTTATCTGGGTCGACCTGAGGAGCCTGTGCATTTTCATCTACCACCGCTTGAGAGACTTACTCTTGATTGTAACGAGGATTGTGGAACTTCTGGGACGCCGG---------------GGGTGGGAACTCCTCAAATATTGGTGG---AATCTCCTACAATATTGG---------------------------------------------------AGTCAGGAACTAAAGAATAGTGCTGTTAGCTTGCTCAACACCACAGCCGTAGCAGTAGCTGAGGGGACAGATAGGGTCATAGAAGCATTACAAAGA------------------GTTGGTAGAGGTATCCTTCATATACCTACAAGAATAAGACAGGGCTTAGAAAGGGCTTTGCTATAA

2.1054.SPD.EU575250 ATG---------GAGATCAGGAGGAATTATCAGCACTTG---------TGGAGATGGGGC------------------------ACCATGCTCCTTGGGTTATTGATGATC------------TGTAATGCTGCA---------GAACAGTTGTGGGTCACAGTATATTATGGGGTACCTGTGTGGAGAGAAGCAAACACCACTCTATTTTGTGCATCAGATGCTAAATCCTATGATACAGAGGTACATAAT---GTTTGGGCCACACATGCCTGTGTACCTACAGACCCTAACCCACAAGAAGTGGTAATG---GGAAATGTGACAGAAAGTTTTAACATGTGGAAAAATCACATGGTAGAACAGATGCATGAGGATATAATTAGTTTATGGGATCAAAGCCTAAAGCCATGTGTAAGATTAACCCCACTTTGTGTTACTTTAAATTGCAGTAATTATGCTGGAACTAATACCACTGCTATTAATACT---------------------------------------------------------------------------AATACCACTGTCTGGGGGGAAAAGATGGACCCAGGAGAAATAAAAAACTGCTCTTTCAATATCGCC---ACACCCATAAAAGAT------AAGAGGCATCAAGAATATGCATTGTTTTATAAAAGTGATGTAGTACCAATAGATGAGGAT------------------------------AATGATACT---------------------ACCAGTTATAGGTTGATAAGTTGTAACACCTCAGTCATTACACAGGCCTGCCCAAAGGTATCCTTTGAACCAATTCCAATACATTATTGTGCCCCAGCTGGTTTTGCGATTCTAAAGTGT---AATAATAAGACGTTCAATGGAAGTGGACCATGTACAAATGTCAGCACAGTACAATGTACACATGGAATTAAGCCAGTGGTATCAACTCAACTGCTGCTAAATGGCAGTCTAGCAGAGGAG---GAGGTAGTAATTAGATCTGCAAATTTCACGGACAATGCTAAAACTATAATGATACAGCTGAAAGACCCTGTAGAAATTAGTTGTACAAGACCCAATAACAATACAATAAAAGGTATACATATAGGA------------CCAGGGAGA---GCATTTTATACAACAGGACAAGTAATAGGAGATATAAGAAAAGCATATTGTAACATT------AGTAGAGCAAAATGGAATCACACTTTAAGTCAGGTAGTTGAAAAATTAAGA---TTACAATTTCAG------------AATAAAACA---ATAGTCTTTAATCAA---------TCCTCAGGAGGGGACCCAGAAATTGTAATGCACACTTTCAATTGTGGAGGGGAGTTTTTCTATTGCAACTCAACACCACTGTTTAATAGTACTTGG---------------AATGATACAAAAGGGTCA------------------------AATAACACAGTAGGA---------------AATGACACA------------------------------ATCATACTTCAATGCAGGATAAAACAAATTATAAACATGTGGCAGGAAGTAGGCAAAGCAATGTATGCCCCTCCCATCAAAGGAAACATTAGCTGTTCATCAAATATTACAGGGCTGCTATTAACAAGAGATGGTGGTATTGTGGACAAT------AACGATACC---------------------------------GAGACCTTCAGACCTGGAGGAGGAGATATGAGGGACAATTGGAGA---AGTGAATTATATAAATATAAAGTAGTAAAAATTGAACCA---TTAGGAATAGCACCC---ACCAAGGCAAAGAGAAGAGTGGTGCAGAGAGAA---AAAAGAGCAATA---GGA---ATAGGA---GCTATG---TTCCTT---GGG---------TTCTTAGGA---GCAGCAGGAAGCACTATGGGCGCAGCGTCAATG---ACGCTGACGGTACAGGCCAGACAATTATTGTCTGGTATAGTGCAACAGCAGAACAATTTGCTGAGGGCTATTGAGGCGCAACAGCATCTGTTGCAACTCACAGTCTGGGGCGTCAAGCAGCTCCAGGCAAGA---GTCCTGGCTGTGGAAAGATACCTAAAGGATCAACAGCTCCTGGGGATTTGGGGTTGCTCTGGAAAACTCATCTGCACCACTACTGTGCCTTGGAATGTTAGTTGGAGT---------------------------AATAAATCTCTGGATAATATTTGGAAT---GAAATGACCTGGATGGAGTGGGAGAGAGAAATTGAC------AATTACACAGACATAATATACTCCTTAATTGAAGAATCACAGAACCAACAAGACAAGAATGAAAAAGAATTGTTGGAATTGGATAAATGGGACAGTTTGTGGAATTGGTTTAGCATAACAAAGTGGCTGTGGTATATAAGAATATTCATAATGATAGTAGGAGGCTTGGTAGGTTTAAGAATAGTTTTTGCTGTACTTTCTATAGTGAATAGAGTTAGGCAGGGATACTCACCCTTATCGTTTCAGACCCGC---CTCCCAACCCCGAGGGGA------CCCGACAGGCCCGAAGGAATCGAAGAAGAAGGTGGAGACAGAGACAGAGACAGATCCACGACATTAGTGCAAGGATTCTTAGCACTTATCTGGGTCGACCTGAGGAGCCTGTGCATTTTCATCTACCACCGCTTGAGAGACTTACTCTTGATTGTAACGAGGATTGTGGAACTTCTGGGACGCCGG---------------GGGTGGGAACTCCTCAAATATTGGTGG---AATCTCCTACAATATTGG---------------------------------------------------AGTCAGGAACTAAAGAATAGTGCTGTTAGCTTGCTCAACACCACAGCCGTAGCAGTAGCTGAGGGGACAGATAGGGTCATAGAAGCATTACAAAGA------------------GTTGGTAGAGGTATCCTTCATATACCTACAAGAATAAGACAGGGCTTAGAAAGGGCTTTGCTATAA

2.1054.SPD.EU575262 ATG---------GAGATCAGGAGGAATTATCAGCACTTG---------TGGAGATGGGGC------------------------ACCATGCTCCTTGGGTTATTGATGATC------------TGTAATGCTGCA---------GAACAGTTGTGGGTCACAGTATATTATGGGGTACCTGTGTGGAGAGAAGCAAACACCACTCTATTTTGTGCATCAGATGCTAAATCCTATGATACAGAGGTACATAAT---GTTTGGGCCACACATGCCTGTGTACCTACAGACCCTAACCCACAAGAAGTGGTAATG---GGAAATGTGACAGAAAGTTTTAACATGTGGAAAAATCACATGGTAGAACAGATGCATGAGGATATAATTAGTTTATGGGATCAAAGCCTAAAGCCATGTGTAAGATTAACCCCACTTTGTGTTACTTTAAATTGCAGTAATTATGCTGGAACTAATACCACTGCTATTAATACT---------------------------------------------------------------------------AATACCACTGTCTGGGGGGAAAAGATGGACCCAGGAGAAATAAAAAACTGCTCTTTCAATATCGCC---ACACCCATAAAAGAT------AAGAGGCATCAAGAATATGCATTGTTTTATAAAAGTGATGTAGTACCAATAGATGAGGAT------------------------------AATGATACT---------------------ACCAGTTATAGGTTGATAAGTTGTAACACCTCAGTCATTACACAGGCCTGCCCAAAGGTATCCTTTGAACCAATTCCAATACATTATTGTGCCCCAGCTGGTTTTGCGATTCTAAAGTGT---AATAATAAGACGTTCAATGGAAGTGGACCATGTACAAATGTCAGCACAGTACAATGTACACATGGAATTAAGCCAGTGGTATCAACTCAACTGCTGCTAAATGGCAGTCTAGCAGAGGAG---GAGGTAGTAATTAGATCTGCAAATTTCACGGACAATGCTAAAACTATAATGATACAGCTGAAAGACCCTGTAGAAATTAGTTGTACAAGACCCAATAACAATACAATAAAAGGTATACATATAGGA------------CCAGGGAGA---GCATTTTATACAACAGGACAAGTAATAGGAGATATAAGAAAAGCATATTGTAACATT------AGTAGAGCAAAATGGAATCACACTTTAAGTCAGGTAGTTGAAAAATTAAGA---TTACAATTTCAG------------AATGAAACA---ATAGTCTTTAATCAA---------TCCTCAGGAGGGGACCCAGAAATTGTAATGCACACTTTCAATTGTGGAGGGGAGTTTTTCTATTGCAACTCAACACCACTGTTTAATAGTACTTGG---------------AATGATACAAAAGGGTCA------------------------AATAACACAGTAGGA---------------AATGACACA------------------------------ATCATACTTCAATGCAGGATAAAACAAATTATAAACATGTGGCAGGAAGTAGGCAAAGCAATGTATGCCCCTCCCATCAAAGGAAACATTAGCTGTTCATCAAATATTACAGGGCTGCTATTAACAAGAGATGGTGGTATTGTGGACAAT------AACGATACC---------------------------------GAGACCTTCAGACCTGGAGGAGGAGATATGAGGGACAATTGGAGA---AGTGAATTATATAAATATAAAGTAGTAAAAATTGAACCA---TTAGGAATAGCACCC---ACCAAGGCAAAGAGAAGAGTGGTGCAGAGAGAA---AAAAGAGCAATA---GGA---ATAGGA---GCTATG---TTCCTT---GGG---------TTCTTAGGA---GCAGCAGGAAGCACTATGGGCGCAGCGTCAATG---ACGCTGACGGTACAGGCCAGACAATTATTGTCTGGTATAGTGCAACAGCAGAACAATTTGCTGAGGGCTATTGAGGCGCAACAGCATCTGTTGCAACTCACAGTCTGGGGCGTCAAGCAGCTCCAGGCAAGA---GTCCTGGCTGTGGAAAGATACCTAAAGGATCAACAGCTCCTGGGGATTTGGGGTTGCTCTGGAAAACTCATCTGCACCACTACTGTGCCTTGGAATGTTAGTTGGAGT---------------------------AATAAATCTCTGGATAATATTTGGAAT---GAAATGACCTGGATGGAGTGGGAGAGAGAAATTGAC------AATTACACAGACATAATATACTCCTTAATTGAAGAATCACAGAACCAACAAGACAAGAATGAAAAAGAATTGTTGGAATTGGATAAATGGGACAGTTTGTGGAATTGGTTTAGCATAACAAAGTGGCTGTGGTATATAAGAATATTCATAATGATAGTAGGAGGCTTGGTAGGTTTAAGAATAGTTTTTGCTGTACTTTCTATAGTGAATAGAGTTAGGCAGGGATACTCACCCTTATCGTTTCAGACCCGC---CTCCCAACCCCGAGGGGA------CCCGACAGGCCCGAAGGAATCGAAGAAGAAGGTGGAGACAGAGACAGAGACAGATCCACGACATTAGTGCAAGGATTCTTAGCACTTATCTGGGTCGACCTGAGGAGCCTGTGCATTTTCATCTACCACCGCTTGAGAGACTTACTCTTGATTGTAACGAGGATTGTGGAACTTCTGGGACGCCGG---------------GGGTGGGAACTCCTCAAATATTGGTGG---AATCTCCTACAATATTGG---------------------------------------------------AGTCAGGAACTAAAGAATAGTGCTGTTAGCTTGCTCAACACCACAGCCGTAGCAGTAGCTGAGGGGACAGATAGGGTCATAGAAGCATTACAAAGA------------------GTTGGTAGAGGTATCCTTCATATACCTACAAGAATAAGACAGGGCTTAGAAAGGGCTTTGCTATAA

2.1054.SPD.EU575259 ATG---------GAGATCAGGAGGAATTATCAGCACTTG---------TGGAGATGGGGC------------------------ACCATGCTCCTTGGGTTATTGATGATC------------TGTAATGCTGCA---------GAACAGTTGTGGGTCACAGTATATTATGGGGTACCTGTGTGGAGAGAAGCAAACACCACTCTATTTTGTGCATCAGATGCTAAATCCTATGATACAGAGGTACATAAT---GTTTGGGCCACACATGCCTGTGTACCTACAGACCCTAACCCACAAGAAGTGGTAATG---GGAAATGTGACAGAAAGTTTTAACATGTGGAAAAATCACATGGTAGAACAGATGCATGAGGATATAATTAGTTTATGGGATCAAAGCCTAAAGCCATGTGTAAGATTAACCCCACTTTGTGTTACTTTAAATTGCAGTAATTATGCTGGAACTAATACCACTGCTATTAATACT---------------------------------------------------------------------------AATACCACTGTCTGGGGGGAAAAGATGGACCCAGGAGAAATAAAAAACTGCTCTTTCAATATCGCC---ACACCCATAAAAGAT------AAGAGGCATCAAGAATATGCATTGTTTTATAAAAGTGATGTAGTACCAATAGATGAGGAT------------------------------AATGATACT---------------------ACCAGTTATAGGTTGATAAGTTGTAACACCTCAGTCATTACACAGGCCTGCCCAAAGGTATCCTTTGAACCAATTCCAATACATTATTGTGCCCCAGCTGGTTTTGCGATTCTAAAGTGT---AATAATAAGACGTTCAATGGAAGTGGACCATGTACAAATGTCAGCACAGTACAATGTACACATGGAATTAAGCCAGTGGTATCAACTCAACTGCTGCTAAATGGCAGTCTAGCAGAGGAG---GAGGTAGTAATTAGATCTGCAAATTTCACGGACAATGCTAAAACTATAATGATACAGCTGAAAGACCCTGTAGAAATTAGTTGTACAAGACCCAATAACAATACAATAAAAGGTATACATATAGGA------------CCAGGGAGA---GCATTTTATACAACAGGACAAGTAATAGGAGATATAAG-------------AACATT------AGTAGAGCAAAATGGAATCACACTTTAAGTCAGGTAGTTGAAAAATTAAGA---TTACAATTTCAG------------AATAAAACA---ATAGTCTTTAATCAA---------TCCTCAGGAGGGGACCCAGAAATTGTAATGCACACTTTCAATTGTGGAGGGGAGTTTTTCTATTGCAACTCAACACCACTGTTTAATAGTACTTGG---------------AATGATACAAAAGGGTCA------------------------AATAACACAGTAGGA---------------AATGACACA------------------------------ATCATACTTCAATGCAGGATAAAACAAATTATAAACATGTGGCAGGAAGTAGGCAAAGCAATGTATGCCCCTCCCATCAAAGGAAACATTAGCTGTTCATCAAATATTACAGGGCTGCTATTAACAAGAGATGGTGGTATTGTGGACAAT------AACGATACC---------------------------------GAGACCTTCAGACCTGGAGGAGGAGATATGAGGGACAATTGGAGA---AGTGAATTATATAAATATAAAGTAGTAAAAATTGAACCA---TTAGGAATAGCACCC---ACCAAGGCAAAGAGAAGAGTGGTGCAGAGAGAA---AAAAGAGCAATA---GGA---ATAGGA---GCTATG---TTCCTT---GGG---------TTCTTAGGA---GCAGCAGGAAGCACTATGGGCGCAGCGTCAATG---ACGCTGACGGTACAGGCCAGACAATTATTGTCTGGTATAGTGCAACAGCAGAACAATTTGCTGAGGGCTATTGAGGCGCAACAGCATCTGTTGCAACTCACAGTCTGGGGCGTCAAGCAGCTCCAGGCAAGA---GTCCTGGCTGTGGAAAGATACCTAAAGGATCAACAGCTCCTGGGGATTTGGGGTTGCTCTGGAAAACTCATCTGCACCACTACTGTGCCTTGGAATGTTAGTTGGAGT---------------------------AATAAATCTCTGGATAATATTTGGAAT---GAAATGACCTGGATGGAGTGGGAGAGAGAAATTGAC------AATTACACAGACATAATATACTCCTTAATTGAAGAATCACAGAACCAACAAGACAAGAATGAAAAAGAATTGTTGGAATTGGATAAATGGGACAGTTTGTGGAATTGGTTTAGCATAACAAAGTGGCTGTGGTATATAAGAATATTCATAATGATAGTAGGAGGCTTGGTAGGTTTAAGAATAGTTTTTGCTGTACTTTCTATAGTGAATAGAGTTAGGCAGGGATACTCACCCTTATCGTTTCAGACCCGC---CTCCCAACCCCGAGGGGA------CCCGACAGGCCCGAAGGAATCGAAGAAGAAGGTGGAGACAGAGACAGAGACAGATCCACGACATTAGTGCAAGGATTCTTAGCACTTATCTGGGTCGACCTGAGGAGCCTGTGCATTTTCATCTACCACCGCTTGAGAGACTTACTCTTGATTGTAACGAGGATTGTGGAACTTCTGGGACGCCGG---------------GGGTGGGAACTCCTCAAATATTGGTGG---AATCTCCTACAATATTGG---------------------------------------------------AGTCAGGAACTAAAGAATAGTGCTGTTAGCTTGCTCAACACCACAGCCGTAGCAGTAGCTGAGGGGACAGATAGGGTCATAGAAGCATTACAAAGA------------------GTTGGTAGAGGTATCCTTCATATACCTACAAGAATAAGACAGGGCTTAGAAAGGGCTTTGCTATAA

2.1054.SPD.EU575282 ATG---------GAGATCAGGAGGAATTATCAGCACTTG---------TGGAGATGGGGC------------------------ACCATGCTCCTTGGGTTATTGATGATC------------TGTAATGCTGCA---------GAACAGTTGTGGGTCACAGTATATTATGGGGTACCTGTGTGGAGAGAAGCAAACACCACTCTATTTTGTGCATCAGATGCTAAATCCTATGATACAGAGGTACATAAT---GTTTGGGCCACACATGCCTGTGTACCTACAGACCCTAACCCACAAGAAGTGGTAATG---GGAAATGTGACAGAAAGTTTTAACATGTGGAAAAATCACATGGTAGAACAGATGCATGAGGATATAATTAGTTTATGGGATCAAAGCCTAAAGCCATGTGTAAGATTAACCCCACTTTGTGTTACTTTAAATTGCAGTAATTATGCTGGAACTAATACCACTGCTATTAATACT---------------------------------------------------------------------------AATACCACTGTCTGGGGGGAAAAGATGGACCCAGGAGAAATAAAAAACTGCTCTTTCAATATCGCC---ACACCCATAAAAGAT------AAGAGGCATCAAGAATATGCATTGTTTTATAAAAGTGATGTAGTACCAATAGATGAGGAT------------------------------AATGATACT---------------------ACCAGTTATAGGTTGATAAGTTGTAACACCTCAGTCATTACACAAGCCTGCCCAAAGGTATCCTTTGAACCAATTCCAATACATTATTGTGCCCCAGCTGGTTTTGCGATTCTAAAGTGT---AATAATAAGACGTTCAATGGAAGTGGACCATGTACAAATGTCAGCACAGTACAATGTACACATGGAATTAAGCCAGTGGTATCAACTCAACTGCTGCTAAATGGCAGTCTAGCAGAGGAG---GAGGTAGTAATTAGATCTGCAAATTTCACGGACAATGCTAAAACTATAATGATACAGCTGAAAGACCCTGTAGAAATTAGTTGTACAAGACCCAATAACAATACAATAAAAGGTATACATATAGGA------------CCAGGGAGA---GCATTTTATACAACAGGACAAGTAATAGGAGATATAAGAAAAGCATATTGTAACATT------AGTAGAGCAAAATGGAATCACACTTTAAGTCAGGTAGTTGAAAAATTAAGA---TTACAATTTCAG------------AATAAAACA---ATAGTCTTTAATCAA---------TCCTCAGGAGGGGACCCAGAAATTGTAATGCACACTTTCAATTGTGGAGGGGAGTTTTTCTACTGCAACTCAACACCACTGTTTAATAGTACTTGG---------------AATGATACAAAAGGGTCA------------------------AATAACACAGTAGGA---------------AATGACACA------------------------------ATCATACTTCAATGCAGGATAAAACAAATTATAAACATGTGGCAGGAAGTAGGCAAAGCAATGTATGCCCCTCCCATCAAAGGAAACATTAGCTGTTCATCAAATATTACAGGGCTGCTATTAACAAGAGATGGTGGTATTGTGGACAAT------AACGATACC---------------------------------GAGACCTTCAGACCTGGAGGAGGAGATATGAGGGACAATTGGAGA---AGTGAATTATATAAATATAAAGTAGTAAAAATTGAACCA---TTAGGAATAGCACCC---ACCAAGGCAAAGAGAAGAGTGGTGCAGAGAGAA---AAAAGAGCAATA---GGA---ATAGGA---GCTATG---TTCCTT---GGG---------TTCTTAGGA---GCAGCAGGAAGCACTATGGGCGCAGCGTCAATG---ACGCTGACGGTACAGGCCAGACAATTATTGTCTGGTATAGTGCAACAGCAGAACAATTTGCTGAGGGCTATTGAGGCGCAACAGCATCTGTTGCAACTCACAGTCTGGGGCGTCAAGCAGCTCCAGGCAAAA---GTCCTGGCTGTGGAAAGATACCTAAAGGATCAACAGCTCCTGGGGATTTGGGGTTGCTCTGGAAAACTCATCTGCACCACTACTGTGCCTTGGAATGTTAGTTGGAGT---------------------------AATAAATCTCTGGATAATATTTGGAAT---GAAATGACCTGGATGGAGTGGGAGAGAGAAATTGAC------AATTACACAGACATAATATACTCCTTAATTGAAGAATCACAGAACCAACAAGACAAGAATGAAAAAGAATTGTTGGAATTGGATAAATGGGACAGTTTGTGGAATTGGTTTAGCATAACAAAGTGGCTGTGGTATATAAGAATATTCATAATGATAGTAGGAGGCTTGGTAGGTTTAAGAATAGTTTTTGCTGTACTTTCTATAGTGAATAGAGTTAGGCAGGGATACTCACCCTTATCGTTTCAGACCCGC---CTCCCAACCCCGAGGGGA------CCCGACAGGCCCGAAGGAATCGAAGAAGAAGGTGGAGACAGAGACAGAGACAGATCCACGACATTAGTGCAAGGATTCTTAGCACTTATCTGGGTCGACCTGAGGAGCCTGTGCATTTTCATCTACCACCGCTTGAGAGACTTACTCTTGATTGTAACGAGGATTGTGGAACTTCTGGGACGCCGG---------------GGGTGGGAACTCCTCAAATATTGGTGG---AATCTCCTACAATATTGG---------------------------------------------------AGTCAGGAACTAAAGAATAGTGCTGTTAGCTTGCTCAACACCACAGCCGTAGCAGTAGCTGAGGGGACAGATAGGGTCATAGAAGCATTACAAAGA------------------GTTGGTAGAGGTATCCTTCATATACCTACAAGAATAAGACAGGGCTTAGAAAGGGCTTTGCTATAA

2.1054.SPD.EU575268 ATG---------GAGATCAGGAGGAATTATCAGCACTTG---------TGGAGATGGGGC------------------------ACCATGCTCCTTGGGTTATTGATGATC------------TGTAATGCTGCA---------GAACAGTTGTGGGTCACAGTATATTATGGGGTACCTGTGTGGAGAGAAGCAAACACCACTCTATTTTGTGCATCAGATGCTAAATCCTATGATACAGAGGTACATAAT---GTTTGGGCCACACATGCCTGTGTACCTACAGACCCTAACCCACAAGAAGTGGTAATG---GGAAATGTGACAGAAAGTTTTAACATGTGGAAAAATCACATGGTAGAACAGATGCATGAGGATATAATTAGTTTATGGGATCAAAGCCTAAAGCCATGTGTAAGATTAACCCCACTTTGTGTTACTTTAAATTGCAGTAATTATGCTGGAACTAATACCACTGCTATTAATACT---------------------------------------------------------------------------AATACCACTGTCTGGGGGGAAAAGATGGACCCAGGAGAAATAAAAAACTGCTCTTTCAATATCGCC---ACACCCATAAAAGAT------AAGAGGCATCAAGAATATGCATTGTTTTATAAAAGTGATGTAGTACCAATAGATGAGGAT------------------------------AATGATACT---------------------ACCAGTTATAGGTTGATAAGTTGTAACACCTCAGTCATTACACAGGCCTGCCCAAAGGTATCCTTTGAACCAATTCCAATACATTATTGTGCCCCAGCTGGTTTTGCGATTCTAAAGTGT---AATAATAAGACGTTCAATGGAAGTGGACCATGTACAAATGTCAGCACAGTACAATGTACACATGGAATTAAGCCAGTGGTATCAACTCAACTGCTGCTAAATGGCAGTCTAGCAGAGGAG---GAGGTAGTAATTAGATCTGCAAATTTCACGGACAATGCTAAAACTATAATGATACAGCTGAAAGACCCTGTAGAAATTAGTTGTACAAGACCCAATAACAATACAATAAAAGGTATACATATAGGA------------CCAGGGAGA---GCATTTTATACAACAGGACAAGTAATAGGAGATATAAGAAAAGCATATTGTAACATT------AGTAGAGCAAAATGGAATCACACTTTAAGTCAGGTAGTTGAAAAATTAAGA---TTACAATTTCAG------------AATAAAACA---ATAGTCTTTAATCAA---------TCCTCAGGAGGGGACCCAGAAATTGTAATGCACACTTTCAATTGTGGAGGGGAGTTTTTCTATTGCAACTCAACACCACTGTTTAATAGTACTTGG---------------AATGATACAAAAGGGTCA------------------------AATAACACAGTAGGA---------------AATGACACA------------------------------ATCATACTTCAATGCAGGATAAAACAAATTATAAACATGTGGCAGGAAGTAGGCAAAGCAATGTATGCCCCTCCCATCAAAGGAAACATTAGCTGTTCATCAAATATTACAGGGCTGCTATTAACAAGAGATGGTGGTATTGTGGACAAT------AACGATACC---------------------------------GAGACCTTCAGACCTGGAGGAGGAGATATGAGGGACAATTGGAGA---AGTGAATTATATAAATATAAAGTAGTAAAAATTGAACCA---TTAGGAATAGCACCC---ACCAAGGCAAAGAGAAGAGTGGTGCAGAGAGAA---AAAAGAGCAATA---GGA---ATAGGA---GCTATG---TTCCTT---GGG---------TTCTTAGGA---GCAGCAGGAAGCACTATGGGCGCAGCGTCAATG---ACGCTGACGGTACAGGCCAGACAATTATTGTCTGGTATAGTGCAACAGCAGAACAATTTGCTGAGGGCTATTGAGGCGCAACAGCATCTGTTGCAACTCACAGTCTGGGGCGTCAAGCAGCTCCAGGCAAGA---GTCCTGGCTGTGGAAAGATACCTAAAGGATCAACAGCTCCTGGGGATTTGGGGTTGCTCTGGGAAACTCATCTGCACCACTACTGTGCCTTGGAATGTTAGTTGGAGT---------------------------AATAAATCTCTGGATAATATTTGGAAT---GAAATGACCTGGATGGAGTGGGAGAGAGAAATTGAC------AATTACACAGACATAATATACTCCTTAATTGAAGAATCACAGAACCAACAAGACAAGAATGAAAAAGAATTGTTGGAATTGGATAAATGGGACAGTTTGTGGAATTGGTTTAGCATAACAAAGTGGCTGTGGTATATAAGAATATTCATAATGATAGTAGGAGGCTTGGTAGGTTTAAGAATAGTTTTTGCTGTACTTTCTATAGTGAATAGAGTTAGGCAGGGATACTCACCCTTATCGTTTCAGACCCGC---CTCCCAACCCCGAGGGGA------CCCGACAGGCCCGAAGGAATCGAAGAAGAAGGTGGAGACAGAGACAGAGACAGATCCACGACATTAGTGCAAGGATTCTTAGCACTTATCTGGGTCGACCTGAGGAGCCTGTGCATTTTCATCTACCACCGCTTGAGAGACTTACTCTTGATTGTAACGAGGATTGTGGAACTTCTGGGACGCCGG---------------GGGTGGGAACTCCTCAAATATTGGTGG---AATCTCCTACAATATTGG---------------------------------------------------AGTCAGGAACTAAAGAATAGTGCTGTTAGCTTGCTCAACACCACAGCCGTAGCAGTAGCTGAGGGGACAGATAGGGTCATAGAAGCATTACAAAGA------------------GTTGGTAGAGGTATCCTTCATATACCTACAAGAATAAGACAGGGCTTAGAAAGGGCTTTGCTATAA

2.1054.SPD.EU575277 ATG---------GAGATCAGGAGGAATTATCAGCACTTG---------TGGAGATGGGGC------------------------ACCATGCTCCTTGGGTTATTGATGATC------------TGTAATGCTGCA---------GAACAGTTGTGGGTCACAGTATATTATGGGGTACCTGTGTGGAGAGAAGCAAACACCACTCTATTTTGTGCATCAGATGCTAAATCCTATGATACAGAGGTACATAAT---GTTTGGGCCACACATGCCTGTGTACCTACAGACCCTAACCCACAAGAAGTGGTAATG---GGAAATGTGACAGAAAGTTTTAACATGTGGAAAAATCACATGGTAGAACAGATGCATGAGGATATAATTAGTTTATGGGATCAAAGCCTAAAGCCATGTGTAAGATTAACCCCACTTTGTGTTACTTTAAATTGCAGTAATTATGCTGGAACTAATACCACTGCTATTAATACT---------------------------------------------------------------------------AATACCACTGTCTGGGGGGAAAAGATGGACCCAGGAGAAATAAAAAACTGCTCTTTCAATATCGCC---ACACCCATAAAAGAT------AAGAGGCATCAAGAATATGCATTGTTTTATAAAAGTGATGTAGTACCAATAGATGAGGAT------------------------------AATGATACT---------------------ACCAGTTATAGGTTGATAAGTTGTAACACCTCAGTCATTACACAGGCCTGCCCAAAGGTATCCTTTGAACCAATTCCAATACATTATTGTGCCCCAGCTGGTTTTGCGATTCTAAAGTGT---AATAATAAGACGTTCAATGGAAGTGGACCATGTACAAATGTCAGCACAGTACAATGTACACATGGAATTAAGCCAGTGGTATCAACTCAACTGCTGCTAAATGGCAGTCTAGCAGAGGAG---GAGGTAGTAATTAGATCTGCAAATTTCACGGACAATGCTAAAACTATAATGATACAGCTGAAAGACCCTGTAGAAATTAGTTGTACAAGACCCAATAACAATACAATAAAAGGTATACATATAGGA------------CCAGGGAGA---GCATTTTATACAACAGGACAAGTAATAGGAGATATAAGAAAAGCATATTGTAACATT------AGTAGAGCAAAATGGAATCACACTTTAAGTCAGGTAGTTGAAAAATTAAGA---TTACAATTTCAG------------AATAAAACA---ATAGTCTTTAATCAA---------TCCTCAGGAGGGGACCCAGAAATTGTAATGCACACTTTCAATTGTGGAGGGGAGTTTTTCTATTGCAACTCAACACCACTGTTTAATAGTACTTGG---------------AATGATACAAAAGGGTCA------------------------AATAACACAGTAGGA---------------AATGACACA------------------------------ATCATACTTCAATGCAGGATAAAACAAATTATAAACATGTGGCAGGAAGTAGGCAAAGCAATGTATGCCCCTCCCATCAAAGGAAACATTAGCTGTTCATCAAATATTACAGGGCTGCTATTAACAAGAGATGGTGGTATTGTGGACAAT------AACGATACC---------------------------------GAGACCTTCAGACCTGGAGGAGGAGATATGAGGGACAATTGGAGA---AGTGAATTATATAAATATAAAGTAGTAAAAATTGAACCA---TTAGGAATAGCACCC---ACCAAGGCAAAGAGAAGAGTGGTGCAGAGAGAA---AAAAGAGCAATA---GGA---ATAGGA---GCTATG---TTCCTT---GGG---------TTCTTAGGA---GCAGCAGGAAGCACTATGGGCGCAGCGTCAATG---ACGCTGACGGTACAGGCCAGACAATTATTGTCTGGTATAGTGCAACAGCAGAACAATTTGCTGAGGGCTATTGAGGCGCAACAGCATCTGTTGCAACTCACAGTCTGGGGCGTCAAGCAGCTCCAGGCAAGA---GTCCTGGCTGTGGAAAGATACCTAAAGGATCAACAGCTCCTGGGGATTTGGGGTTGCTCTGGAAAACTCATCTGCACCACTACTGTGCCTTGGAATGTTAGTTGGAGT---------------------------AATAAATCTCTGGATAATATTTGGAAT---GAAATGACCTGGATGGAGTGGGAGAGAGAAATTGAC------AATTACACAGACATAATATACTCCTTAATTGAAGAATCACAGAACCAACAAGACAAGAATGAAAAAGAATTGTTGGAATTGGATAAATGGGACAGTTTGTGGAATTGGTTTAGCATAACAAAGTGGCTGTGGTATATAAGAATATTCATAATGATAGTAGGAGGCTTGGTAGGTTTAAGAATAGTTTTTGCTGTACTTTCTATAGTGAATAGAGTTAGGCAGGGATACTCACCCTTATCGTTTCAGACCCGC---CTCCCAACCCCGAGGGGA------CCCGACAGGCCCGAAGGAATCGAAGAAGAAGGTGGAGACAGAGACAGAGACAGATCCACGACATTAGTGCAAGGATTCTTAGCACTTATCTGGGTCGACCTGAGGAGCCTGTGCATTTTCATCTACCACCGCTTGAGAGACTTACTCTTGATTGTAACGAGGATTGTGGAACTTCTGGGACGCCGG---------------GGGTGGGAACTCCTCAAATATTGGTGG---AATCTCCTACAATATTGG---------------------------------------------------AGTCAGGAACTAAAGAATAGTGCTGTTAGCTTGCTCAACACCACAGCCGTAGCAGTAGCTGAGGGGACAGATAGGGTCATAGAAGCATTACAAAGA------------------GTTGGTAGAGGTATCCTTCATATACCTACAAGAATAAGACAGGGCTTAGAAAGGGCTTTGCTATAA

2.1054.SPD.EU575267 ATG---------GAGATCAGGAGGAATTATCAGCACTTG---------TGGAGATGGGGC------------------------ACCATGCTCCTTGGGTTATTGATGATC------------TGTAATGCTGCA---------GAACAGTTGTGGGTCACAGTATATTATGGGGTACCTGTGTGGAGAGAAGCAAACACCACTCTATTTTGTGCATCAGATGCTAAATCCTATGATACAGAGGTACATAAT---GTTTGGGCCACACATGCCTGTGTACCTACAGACCCTAACCCACAAGAAGTGGTAATG---GGAAATGTGACAGAAAGTTTTAACATGTGGAAAAATCACATGGTAGAACAGATGCATGAGGATATAATTAGTTTATGGGATCAAAGCCTAAAGCCATGTGTAAGATTAACCCCACTTTGTGTTACTTTAAATTGCAGTAATTATGCTGGAACTAATACCACTGCTATTAATACT---------------------------------------------------------------------------AATACCACTGTCTGGGGGGAAAAGATGGACCCAGGAGAAATAAAAAACTGCTCTTTCAATATCGCC---ACACCCATAAAAGAT------AAGAGGCATCAAGAATATGCATTGTTTTATAAAAGTGATGTAGTACCAATAGATGAGGAT------------------------------AATGATACT---------------------ACCAGTTATAGGTTGATAAGTTGTAACACCTCAGTCATTACACAGGCCTGCCCAAAGGTATCCTTTGAACCAATTCCAATACATTATTGTGCCCCAGCTGGTTTTGCGATTCTAAAGTGT---AATAATAAGACGTTCAATGGAAGTGGACCATGTACAAATGTCAGCACAGTACAATGTACACATGGAATTAAGCCAGTGGTATCAACTCAACTGCTGCTAAATGGCAGTCTAGCAGAGGAG---GAGGTAGTAATTAGATCTGCAAATTTCACGGACAATGCTAAAACTATAATGATACAGCTGAAAGACCCTGTAGAAATTAGTTGTACAAGACCCAATAACAATACAATAAAAGGTATACATATAGGA------------CCAGGGAGA---GCATTTTATACAACAGGACAAGTAATAGGAGATATAAGAAAAGCATATTGTAACATT------AGTAGAGCAAAATGGAATCACACTTTAAGTCAGGTAGTTGAAAAATTAAGA---TTACAATTTCAG------------AATAAAACA---ATAGTCTTTAATCAA---------TCCTCAGGAGGGGACCCAGAAATTGTAATGCACACTTTCAATTGTGGAGGGGAGTTTTTCTATTGCAACTCAACACCACTGTTTAATAGTACTTGG---------------AATGATACAAAAGGGTCA------------------------AATAACACAGTAGGA---------------AATGACACA------------------------------ATCATACTTCAATGCAGGATAAAACAAATTATAAACATGTGGCAGGAAGTAGGCAAAGCAATGTATGCCCCTCCCATCAAAGGAAACATTAGCTGTTCATCAAATATTACAGGGCTGCTATTAACAAGAGATGGTGGTATTGTGGACAAT------AACGATACC---------------------------------GAGACCTTCAGACCTGGAGGAGGAGATATGAGGGACAATTGGAGA---AGTGAATTATATAAATATAAAGTAGTAAAAATTGAACCA---TTAGGAATAGCACCC---ACCAAGGCAAAGAGAAGAGTGGTGCAGAGAGAA---AAAAGAGCAATA---GGA---ATAGGA---GCTATG---TTCCTT---GGG---------TTCTTAGGA---GCAGCAGGAAGCACTATGGGCGCAGCGTCAATG---ACGCTGACGGTACAGGCCAGACAATTATTGTCTGGTATAGTGCAACAGCAGAACAATTTGCTGAGGGCTATTGAGGCGCAACAGCATCTGTTGCAACTCACAGTCTGGGGCGTCAAGCAGCTCCAGGCAAGA---GTCCTGGCTGTGGAAAGATACCTAAAGGATCAACAGCTCCTGGGGATTTGGGGTTGCTCTGGAAAACTCATCTGCACCACTACTGTGCCTTGGAATGTTAGTTGGAGT---------------------------AATAAATCTCTGGATAATATTTGGAAT---GAAATGACCTGGATGGAGTGGGAGAGAGAAATTGAC------AATTACACAGACATAATATACTCCTTAATTGAAGAATCACAGAACCAACAAGACAAGAATGAAAAAGAATTGTTGGAATTGGATAAATGGGACAGTTTGTGGAATTGGTTTAGCATAACAAAGTGGCTGTGGTATATAAGAATATTCATAATGATAGTAGGAGGCTTGGTAGGTTTAAGAATAGTTTTTGCTGTACTTTCTATAGTGAATAGAGTTAGGCAGGGATACTCACCCTTATCGTTTCAGACCCGC---CTCCCAACCCCGAGGGGA------CCCGACAGGCCCGAAGGAATCGAAGAAGAAGGTGGAGACAGAGACAGAGACAGATCCACGACATTAGTGCAAGGATTCTTAGCACTTATCTGGGTCGACCTGAGGAGCCTGTGCATTTTCATCTACCACCGCTTGAGAGACTTACTCTTGATTGTAACGAGGATTGTGGAACTTCTGGGACGCCGG---------------GGGTGGGAACTCCTCAAATATTGGTGG---AATCTCCTACAATATTGG---------------------------------------------------AGTCAGGAACTAAAGAATAGTGCTGTTAGCTTGCTCAACACCACAGCCGTAGCAGTAGCTGAGGGGACAGATAGGGTCATAGAAGCATTACAAAGA------------------GTTGGTAGAGGTATCCTTCATATACCTACAAGAATAAGACAGGGCTTAGAAAGGGCTTTGCTATAA

2.1054.SPD.EU575264 ATG---------GAGATCAGGAGGAATTATCAGCACTTG---------TGGAGATGGGGC------------------------ACCATGCTCCTTGGGTTATTGATGATC------------TGTAATGCTGCA---------GAACAGTTGTGGGTCACAGTATATTATGGGGTACCTGTGTGGAGAGAAGCAAACACCACTCTATTTTGTGCATCAGATGCTAAATCCTATGATACAGAGGTACATAAT---GTTTGGGCCACACATGCCTGTGTACCTACAGACCCTAACCCACAAGAAGTGGTAATG---GGAAATGTGACAGAAAGTTTTAACATGTGGAAAAATCACATGGTAGAACAGATGCATGAGGATATAATTAGTTTATGGGATCAAAGCCTAAAGCCATGTGTAAGATTAACCCCACTTTGTGTTACTTTAAATTGCAGTAATTATGCTGGAACTAATACCACTGCTATTAATACT---------------------------------------------------------------------------AATACCACTGTCTGGGGGGAAAAGATGGACCCAGGAGAAATAAAAAACTGCTCTTTCAATATCGCC---ACACCCATAAAAGAT------AAGAGGCATCAAGAATATGCATTGTTTTATAAAAGTGATGTAGTACCAATAGATGAGGAT------------------------------AATGATACT---------------------ACCAGTTATAGGTTGATAAGTTGTAACACCTCAGTCATTACACAGGCCTGCCCAAAGGTATCCTTTGAACCAATTCCAATACATTATTGTGCCCCAGCTGGTTTTGCGATTCTAAAGTGT---AATAATAAGACGTTCAATGGAAGTGGACCATGTACAAATGTCAGCACAGTACAATGTACACATGGAATTAAGCCAGTGGTATCAACTCAACTGCTGCTAAATGGCAGTCTAGCAGAGGAG---GAGGTAGTAATTAGATCTGCAAATTTCACGGACAATGCTAAAACTATAATGATACAGCTGAAAGACCCTGTAGAAATTAGTTGTACAAGACCCAATAACAATACAATAAAAGGTATACATATAGGA------------CCAGGGAGA---GCATTTTATACAACAGGACAAGTAATAGGAGATATAAGAAAAGCATATTGTAACATT------AGTAGAGCAAAATGGAATCACACTTTAAGTCAGGTAGTTGAAAAATTAAGA---TTACAATTTCAG------------AATAAAACA---ATAGTCTTTAATCAA---------TCCTCAGGAGGGGACCCAGAAATTGTAATGCACACTTTCAATTGTGGAGGGGAGTTTTTCTATTGCAACTCAACACCACTGTTTAATAGTACTTGG---------------AATGATACAAAAGGGTCA------------------------AATAACACAGTAGGA---------------AATGACACA------------------------------ATCATACTTCAATGCAGGATAAAACAAATTATAAACATGTGGCAGGAAGTAGGCAAAGCAATGTATGCCCCTCCCATCAAAGGAAACATTAGCTGTTCATCAAATATTACAGGGCTGCTATTAACAAGAGATGGTGGTATTGTGGACAAT------AACGATACC---------------------------------GAGACCTTCAGACCTGGAGGAGGAGATATGAGGGACAATTGGAGA---AGTGAATTATATAAATATAAAGTAGTAAAAATTGAACCA---TTAGGAATAGCACCC---ACCAAGGCAAAGAGAAGAGTGGTGCAGAGAGAA---AAAAGAGCAATA---GGA---ATAGGA---GCTATG---TTCCTT---GGG---------TTCTTAGGA---GCAGCAGGAAGCACTATGGGCGCAGCGTCAATG---ACGCTGACGGTACAGGCCAGACAATTATTGTCTGGTATAGTGCAACAGCAGAACAATTTGCTGAGGGCTATTGAGGCGCAACAGCATCTGTTGCAACTCACAGTCTGGGGCGTCAAGCAGCTCCAGGCAAGA---GTCCTGGCTGTGGAAAGATACCTAAAGGATCAACAGCTCCTGGGGATTTGGGGTTGCTCTGGAAAACTCATCTGCACCACTACTGTGCCTTGGAATGTTAGTTGGAGT---------------------------AATAAATCTCTGGATAATATTTGGAAT---GAAATGACCTGGATGGAGTGGGAGAGAGAAATTGAC------AATTACACAGACATAATATACTCCTTAATTGAAGAATCACAGAACCAACAAGACAAGAATGAAAAAGAATTGTTGGAATTGGATAAATGGGACAGTTTGTGGAATTGGTTTAGCATAACAAAGTGGCTGTGGTATATAAGAATATTCATAATGATAGTAGGAGGCTTGGTAGGTTTAAGAATAGTTTTTGCTGTACTTTCTATAGTGAATAGAGTTAGGCAGGGATACTCACCCTTATCGTTTCAGACCCGC---CTCCCAACCCCGAGGGGA------CCCGACAGGCCCGAAGGAATCGAAGAAGAAGGTGGAGACAGAGACAGAGACAGATCCACGACATTAGTGCAAGGATTCTTAGCACTTATCTGGGTCGACCTGAGGAGCCTGTGCATTTTCATCTACCACCGCTTGAGAGACTTACTCTTGATTGTAACGAGGATTGTGGAACTTCTGGGACGCCGG---------------GGGTGGGAACTCCTCAAATATTGGTGG---AATCTCCTACAATATTGG---------------------------------------------------AGTCAGGAACTAAAGAATAGTGCTGTTAGCTTGCTCAACACCACAGCCGTAGCAGTAGCTGAGGGGACAGATAGGGTCATAGAAGCATTACAAAGA------------------GTTGGTAGAGGTATCCTTCATATACCTACAAGAATAAGACAGGGCTTAGAAAGGGCTTTGCTATAA

2.1054.SPD.EU575261 ATG---------GAGATCAGGAGGAATTATCAGCACTTG---------TGGAGATGGGGC------------------------ACCATGCTCCTTGGGTTATTGATGATC------------TGTAATGCTGCA---------GAACAGTTGTGGGTCACAGTATATTATGGGGTACCTGTGTGGAGAGAAGCAAACACCACTCTATTTTGTGCATCAGATGCTAAATCCTATGATACAGAGGTACATAAT---GTTTGGGCCACACATGCCTGTGTACCTACAGACCCTAACCCACAAGAAGTGGTAATG---GGAAATGTGACAGAAAGTTTTAACATGTGGAAAAATCACATGGTAGAACAGATGCATGAGGATATAATTAGTTTATGGGATCAAAGCCTAAAGCCATGTGTAAGATTAACCCCACTTTGTGTTACTTTAAATTGCAGTAATTATGCTGGAACTAATACCACTGCTATTAATACT---------------------------------------------------------------------------AATACCACTGTCTGGGGGGAAAAGATGGACCCAGGAGAAATAAAAAACTGCTCTTTCAATATCGCC---ACACCCATAAAAGAT------AAGAGGCATCAAGAATATGCATTGTTTTATAAAAGTGATGTAGTACCAATAGATGAGGAT------------------------------AATGATACT---------------------ACCAGTTATAGGTTGATAAGTTGTAACACCTCAGTCATTACACAGGCCTGCCCAAAGGTATCCTTTGAACCAATTCCAATACATTATTGTGCCCCAGCTGGTTTTGCGATTCTAAAGTGT---AATAATAAGACGTTCAATGGAAGTGGACCATGTACAAATGTCAGCACAGTACAATGTACACATGGAATTAAGCCAGTGGTATCAACTCAACTGCTGCTAAATGGCAGTCTAGCAGAGGAG---GAGGTAGTAATTAGATCTGCAAATTTCACGGACAATGCTAAAACTATAATGATACAGCTGAAAGACCCTGTAGAAATTAGTTGTACAAGACCCAATAACAATACAATAAAAGGTATACATATAGGA------------CCAGGGAGA---GCATTTTATACAACAGGACAAGTAATAGGAGATATAAGAAAAGCATATTGTAACATT------AGTAGAGCAAAATGGAATCACACTTTAAGTCAGGTAGTTGAAAAATTAAGA---TTACAATTTCAG------------AATAAAACA---ATAGTCTTTAATCAA---------TCCTCAGGAGGGGACCCAGAAATTGTAATGCACACTTTCAATTGTGGAGGGGAGTTTTTCTATTGCAACTCAACACCACTGTTTAATAGTACTTGG---------------AATGATACAAAAGGGTCA------------------------AATAACACAGTAGGA---------------AATGACACA------------------------------ATCATACTTCAATGCAGGATAAAACAAATTATAAACATGTGGCAGGAAGTAGGCAAAGCAATGTATGCCCCTCCCATCAAAGGAAACATTAGCTGTTCATCAAATATTACAGGGCTGCTATTAACAAGAGATGGTGGTATTGTGGACAAT------AACGATACC---------------------------------GAGACCTTCAGACCTGGAGGAGGAGATATGAGGGACAATTGGAGA---AGTGAATTATATAAATATAAAGTAGTAAAAATTGAACCA---TTAGGAATAGCACCC---ACCAAGGCAAAGAGAAGAGTGGTGCAGAGAGAA---AAAAGAGCAATA---GGA---ATAGGA---GCTATG---TTCCTT---GGG---------TTCTTAGGA---GCAGCAGGAAGCACTATGGGCGCAGCGTCAATG---ACGCTGACGGTACAGGCCAGACAATTATTGTCTGGTATAGTGCAACAGCAGAACAATTTGCTGAGGGCTATTGAGGCGCAACAGCATCTGTTGCAACTCACAGTCTGGGGCGTCAAGCAGCTCCAGGCAAGA---GTCCTGGCTGTGGAAAGATACCTAAAGGATCAACAGCTCCTGGGGATTTGGGGTTGCTCTGGAAAACTCATCTGCACCACTACTGTGCCTTGGAATGTTAGTTGGAGT---------------------------AATAAATCTCTGGATAATATTTGGAAT---GAAATGACCTGGATGGAGTGGGAGAGAGAAATTGAC------AATTACACAGACATAATATACTCCTTAATTGAAGAATCACAGAACCAACAAGACAAGAATGAAAAAGAATTGTTGGAATTGGATAAATGGGACAGTTTGTGGAATTGGTTTAGCATAACAAAGTGGCTGTGGTATATAAGAATATTCATAATGATAGTAGGAGGCTTGGTAGGTTTAAGAATAGTTTTTGCTGTACTTTCTATAGTGAATAGAGTTAGGCAGGGATACTCACCCTTATCGTTTCAGACCCGC---CTCCCAACCCCGAGGGGA------CCCGACAGGCCCGAAGGAATCGAAGAAGAAGGTGGAGACAGAGACAGAGACAGATCCACGACATTAGTGCAAGGATTCTTAGCACTTATCTGGGTCGACCTGAGGAGCCTGTGCATTTTCATCTACCACCGCTTGAGAGACTTACTCTTGATTGTAACGAGGATTGTGGAACTTCTGGGACGCCGG---------------GGGTGGGAACTCCTCAAATATTGGTGG---AATCTCCTACAATATTGG---------------------------------------------------AGTCAGGAACTAAAGAATAGTGCTGTTAGCTTGCTCAACACCACAGCCGTAGCAGTAGCTGAGGGGACAGATAGGGTCATAGAAGCATTACAAAGA------------------GTTGGTAGAGGTATCCTTCATATACCTACAAGAATAAGACAGGGCTTAGAAAGGGCTTTGCTATAA

2.1054.SPD.EU575280 ATG---------GAGATCAGGAGGAATTATCAGCACTTG---------TGGAGATGGGGC------------------------ACCATGCTCCTTGGGTTATTGATGATC------------TGTAATGCTGCA---------GAACAGTTGTGGGTCACAGTATATTATGGGGTACCTGTGTGGAGAGAAGCAAACACCACTCTATTTTGTGCATCAGATGCTAAATCCTATGATACAGAGGTACATAAT---GTTTGGGCCACACATGCCTGTGTACCTACAGACCCTAACCCACAAGAAGTGGTAATG---GGAAATGTGACAGAAAGTTTTAACATGTGGAAAAATCACATGGTAGAACAGATGCATGAGGATATAATTAGTTTATGGGATCAAAGCCTAAAGCCATGTGTAAGATTAACCCCACTTTGTGTTACTTTAAATTGCAGTAATTATGCTGGAACTAATACCACTGCTATTAATACT---------------------------------------------------------------------------AATACCACTGTCTGGGGGGAAAAGATGGACCCAGGAGAAATAAAAAACTGCTCTTTCAATATCGCC---ACACCCATAAAAGAT------AAGAGGCATCAAGAATATGCATTGTTTTATAAAAGTGATGTAGTACCAATAGATGAGGAT------------------------------AATGATACT---------------------ACCAGTTATAGGTTGATAAGTTGTAACACCTCAGTCATTACACAGGCCTGCCCAAAGGTATCCTTTGAACCAATTCCAATACATTATTGTGCCCCAGCTGGTTTTGCGATTCTAAAGTGT---AATAATAAGACGTTCAATGGAAGTGGACCATGTACAAATGTCAGCACAGTACAATGTACACATGGAATTAAGCCAGTGGTATCAACTCAACTGCTGCTAAATGGCAGTCTAGCAGAGGAG---GAGGTAGTAATTAGATCTGCAAATTTCACGGACAATGCTAAAACTATAATGATACAGCTGAAAGACCCTGTAGAAATTAGTTGTACAAGACCCAATAACAATACAATAAAAGGTATACATATAGGA------------CCAGGGAGA---GCATTTTATACAACAGGACAAGTAATAGGAGATATAAGAAAAGCATATTGTAACATT------AGTAGAGCAAAATGGAATCACACTTTAAGTCAGGTAGTTGAAAAATTAAGA---TTACAATTTCAG------------AATAAAACA---ATAGTCTTTAATCAA---------TCCTCAGGAGGGGACCCAGAAATTGTAATGCACACTTTCAATTGTGGAGGGGAGTTTTTCTATTGCAACTCAACACCACTGTTTAATAGTACTTGG---------------AATGATACAAAAGGGTCA------------------------AATAACACAGTAGGA---------------AATGACACA------------------------------ATCATACTTCAATGCAGGATAAAACAAATTATAAACATGTGGCAGGAAGTAGGCAAAGCAATGTATGCCCCTCCCATCAAAGGAAACATTAGCTGTTCATCAAATATTACAGGGCTGCTATTAACAAGAGATGGTGGTATTGTGGACAAT------AACGATACC---------------------------------GAGACCTTCAGACCTGGAGGAGGAGATATGAGGGACAATTGGAGA---AGTGAATTATATAAATATAAAGTAGTAAAAATTGAACCA---TTAGGAATAGCACCC---ACCAAGGCAAAGAGAAGAGTGGTGCAGAGAGAA---AAAAGAGCAATA---GGA---ATAGGA---GCTATG---TTCCTT---GGG---------TTCTTAGGA---GCAGCAGGAAGCACTATGGGCGCAGCGTCAATG---ACGCTGACGGTACAGGCCAGACAATTATTGTCTGGTATAGTGCAACAGCAGAACAATTTGCTGAGGGCTATTGAGGCGCAACAGCATCTGTTGCAACTCACAGTCTGGGGCGTCAAGCAGCTCCAGGCAAGA---GTCCTGGCTGTGGAAAGATACCTAAAGGATCAACAGCTCCTGGGGATTTGGGGTTGCTCTGGAAAACTCATCTGCACCACTACTGTGCCTTGGAATGTTAGTTGGAGT---------------------------AATAAATCTCTGGATAATATTTGGAAT---GAAATGACCTGGATGGAGTGGGAGAGAGAAATTGAC------AATTACACAGACATAATATACTCCTTAATTGAAGAATCACAGAACCAACAAGACAAGAATGAAAAAGAATTGTTGGAATTGGATAAATGGGACAGTTTGTGGAATTGGTTTAGCATAACAAAGTGGCTGTGGTATATAAGAATATTCATAATGATAGTAGGAGGCTTGGTAGGTTTAAGAATAGTTTTTGCTGTACTTTCTATAGTGAATAGAGTTAGGCAGGGATACTCACCCTTATCGTTTCAGACCCGC---CTCCCAACCCCGAGGGGA------CCCGACAGGCCCGAAGGAATCGAAGAAGAAGGTGGAGACAGAGACAGAGACAGATCCACGACATTAGTGCAAGGATTCTTAGCACTTATCTGGGTCGACCTGAGGAGCCTGTGCATTTTCATCTACCACCGCTTGAGAGACTTACTCTTGATTGTAACGAGGATTGTGGAACTTCTGGGACGCCGG---------------GGGTGGGAACTCCTCAAATATTGGTGG---AATCTCCTACAATATTGG---------------------------------------------------AGTCAGGAACTAAAGAATAGTGCTGTTAGCTTGCTCAACACCACAGCCGTAGCAGTAGCTGAGGGGACAGATAGGGTCATAGAAGCATTACAAAGA------------------GTTGGTAGAGGTATCCTTCATATACCTACAAGAATAAGACAGGGCTTAGAAAGGGCTTTGCTATAA

2.1054.SPD.EU575269 ATG---------GAGATCAGGAGGAATTATCAGCACTTG---------TGGAGATGGGGC------------------------ACCATGCTCCTTGGGTTATTGATGATC------------TGTAATGCTGCA---------GAACAGTTGTGGGTCACAGTATATTATGGGGTACCTGTGTGGAGAGAAGCAAACACCACTCTATTTTGTGCATCAGATGCTAAATCCTATGATACAGAGGTACATAAT---GTTTGGGCCACACATGCCTGTGTACCTACAGACCCTAACCCACAAGAAGTGGTAATG---GGAAATGTGACAGAAAGTTTTAACATGTGGAAAAATCACATGGTAGAACAGATGCATGAGGATATAATTAGTTTATGGGATCAAAGCCTAAAGCCATGTGTAAGATTAACCCCACTTTGTGTTACTTTAAATTGCAGTAATTATGCTGGAACTAATACCACTGCTATTAATACT---------------------------------------------------------------------------AATACCACTGTCTGGGGGGAAAAGATGGACCCAGGAGAAATAAAAAACTGCTCTTTCAATATCGCC---ACACCCATAAAAGAT------AAGAGGCATCAAGAATATGCATTGTTTTATAAAAGTGATGTAGTACCAATAGATGAGGAT------------------------------AATGATACT---------------------ACCAGTTATAGGTTGATAAGTTGTAACACCTCAGTCATTACACAGGCCTGCCCAAAGGTATCCTTTGAACCAATTCCAATACATTATTGTGCCCCAGCTGGTTTTGCGATTCTAAAGTGT---AATAATAAGACGTTCAATGGAAGTGGACCATGTACAAATGTCAGCACAGTACAATGTACACATGGAATTAAGCCAGTGGTATCAACTCAACTGCTGCTAAATGGCAGTCTAGCAGAGGAG---GAGGTAGTAATTAGATCTGCAAATTTCACGGACAATGCTAAAACTATAATGATACAGCTGAAAGACCCTGTAGAAATTAGTTGTACAAGACCCAATAACAATACAATAAAAGGTATACATATAGGA------------CCAGGGAGA---GCATTTTATACAACAGGACAAGTAATAGGAGATATAAGAAAAGCATATTGTAACATT------AGTAGAGCAAAATGGAATCACACTTTAAGTCAGGTAGTTGAAAAATTAAGA---TTACAATTTCAG------------AATAAAACA---ATAGTCTTTAATCAA---------TCCTCAGGAGGGGACCCAGAAATTGTAATGCACACTTTCAATTGTGGAGGGGAGTTTTTCTATTGCAACTCAACACCACTGTTTAATAGTACTTGG---------------AATGATACAAAAGGGTCA------------------------AATAACACAGTAGGA---------------AATGACACA------------------------------ATCATACTTCAATGCAGGATAAAACAAATTATAAACATGTGGCAGGAAGTAGGCAAAGCAATGTATGCCCCTCCCATCAAAGGAAACATTAGCTGTTCATCAAATATTACAGGGCTGCTATTAACAAGAGATGGTGGTATTGTGGACAAT------AACGATACC---------------------------------GAGACCTTCAGACCTGGAGGAGGAGATATGAGGGACAATTGGAGA---AGTGAATTATATAAATATAAAGTAGTAAAAATTGAACCA---TTAGGAATAGCACCC---ACCAAGGCAAAGAGAAGAGTGGTGCAGAGAGAA---AAAAGAGCAATA---GGA---ATAGGA---GCTATG---TTCCTT---GGG---------TTCTTAGGA---GCAGCAGGAAGCACTATGGGCGCAGCGTCAATG---ACGCTGACGGTACAGGCCAGACAATTATTGTCTGGTATAGTGCAACAGCAGAACAATTTGCTGAGGGCTATTGAGGCGCAACAGCATCTGTTGCAACTCACAGTCTGGGGCGTCAAGCAGCTCCAGGCAAGA---GTCCTGGCTGTGGAAAGATACCTAAAGGATCAACAGCTCCTGGGGATTTGGGGTTGCTCTGGAAAACTCATCTGCACCACTACTGTGCCTTGGAATGTTAGTTGGAGT---------------------------AATAAATCTCTGGATAATATTTGGAAT---GAAATGACCTGGATGGAGTGGGAGAGAGAAATTGAC------AATTACACAGACATAATATACTCCTTAATTGAAGAATCACAGAACCAACAAGACAAGAATGAAAAAGAATTGTTGGAATTGGATAAATGGGACAGTTTGTGGAATTGGTTTAGCATAACAAAGTGGCTGTGGTATATAAGAATATTCATAATGATAGTAGGAGGCTTGGTAGGTTTAAGAATAGTTTTTGCTGTACTTTCTATAGTGAATAGAGTTAGGCAGGGATACTCACCCTTATCGTTTCAGACCCGC---CTCCCAACCCCGAGGGGA------CCCGACAGGCCCGAAGGAATCGAAGAAGAAGGTGGAGACAGAGACAGAGACAGATCCACGACATTAGTGCAAGGATTCTTAGCACTTATCTGGGTCGACCTGAGGAGCCTGTGCATTTTCATCTACCACCGCTTGAGAGACTTACTCTTGATTGTAACGAGGATTGTGGAACTTCTGGGACGCCGG---------------GGGTGGGAACTCCTCAAATATTGGTGG---AATCTCCTACAATATTGG---------------------------------------------------AGTCAGGAACTAAAGAATAGTGCTGTTAGCTTGCTCAACACCACAGCCGTAGCAGTAGCTGAGGGGACAGATAGGGTCATAGAAGCATTACAAAGA------------------GTTGGTAGAGGTATCCTTCATATACCTACAAGAATAAGACAGGGCTTAGAAAGGGCTTTGCTATAA

2.1054.SPD.EU575276 ATG---------GAGATCAGGAGGAATTATCAGCACTTG---------TGGAGATGGGGC------------------------ACCATGCTCCTTGGGTTATTGATGATC------------TGTAATGCTGCA---------GAACAGTTGTGGGTCACAGTATATTATGGGGTACCTGTGTGGAGAGAAGCAAACACCACTCTATTTTGTGCATCAGATGCTAAATCCTATGATACAGAGGTACATAAT---GTTTGGGCCACACATGCCTGTGTACCTACAGACCCTAACCCACAAGAAGTGGTAATG---GGAAATGTGACAGAAAGTTTTAACATGTGGAAAAATCACATGGTAGAACAGATGCATGAGGATATAATTAGTTTATGGGATCAAAGCCTAAAGCCATGTGTAAGATTAACCCCACTTTGTGTTACTTTAAATTGCAGTAATTATGCTGGAACTAATACCACTGCTATTAATACT---------------------------------------------------------------------------AATACCACTGTCTGGGGGGAAAAGATGGACCCAGGAGAAATAAAAAACTGCTCTTTCAATATCGCC---ACACCCATAAAAGAT------AAGAGGCATCAAGAATATGCATTGTTTTATAAAAGTGATGTAGTACCAATAGATGAGGAT------------------------------AATGATACT---------------------ACCAGTTATAGGTTGATAAGTTGTAACACCTCAGTCATTACACAGGCCTGCCCAAAGGTATCCTTTGAACCAATTCCAATACATTATTGTGCCCCAGCTGGTTTTGCGATTCTAAAGTGT---AATAATAAGACGTTCAATGGAAGTGGACCATGTACAAATGTCAGCACAGTACAATGTACACATGGAATTAAGCCAGTGGTATCAACTCAACTGCTGCTAAATGGCAGTCTAGCAGAGGAG---GAGGTAGTAATTAGATCTGCAAATTTCACGGACAATGCTAAAACTATAATGATACAGCTGAAAGACCCTGTAGAAATTAGTTGTACAAGACCCAATAACAATACAATAAAAGGTATACATATAGGA------------CCAGGGAGA---GCATTTTATACAACAGGACAAGTAATAGGAGATATAAGAAAAGCATATTGTAACATT------AGTAGAGCAAAATGGAATCACACTTTAAGTCAGGTAGTTGAAAAATTAAGA---TTACAATTTCAG------------AATAAAACA---ATAGTCTTTAATCAA---------TCCTCAGGAGGGGACCCAGAAATTGTAATGCACACTTTCAATTGTGGAGGGGAGTTTTTCTATTGCAACTCAACACCACTGTTTAATAGTACTTGG---------------AATGATACAAAAGGGTCA------------------------AATAACACAGTAGGA---------------AATGACACA------------------------------ATCATACTTCAATGCAGGATAAAACAAATTATAAACATGTGGCAGGAAGTAGGCAAAGCAATGTATGCCCCTCCCATCAAAGGAAACATTAGCTGTTCATCAAATATTACAGGGCTGCTATTAACAAGAGATGGTGGTATTGTGGACAAT------AACGATACC---------------------------------GAGACCTTCAGACCTGGAGGAGGAGATATGAGGGACAATTGGAGA---AGTGAATTATATAAATATAAAGTAGTAAAAATTGAACCA---TTAGGAATAGCACCC---ACCAAGGCAAAGAGAAGAGTGGTGCAGAGAGAA---AAAAGAGCAATA---GGA---ATAGGA---GCTATG---TTCCTT---GGG---------TTCTTAGGA---GCAGCAGGAAGCACTATGGGCGCAGCGTCAATG---ACGCTGACGGTACAGGCCAGACAATTATTGTCTGGTATAGTGCAACAGCAGAACAATTTGCTGAGGGCTATTGAGGCGCAACAGCATCTGTTGCAACTCACAGTCTGGGGCGTCAAGCAGCTCCAGGCAAGA---GTCCTGGCTGTGGAAAGATACCTAAAGGATCAACAGCTCCTGGGGATTTGGGGTTGCTCTGGAAAACTCATCTGCACCACTACTGTGCCTTGGAATGTTAGTTGGAGT---------------------------AATAAATCTCTGGATAATATTTGGAAT---GAAATGACCTGGATGGAGTGGGAGAGAGAAATTGAC------AATTACACAGACATAATATACTCCTTAATTGAAGAATCACAGAACCAACAAGACAAGAATGAAAAAGAATTGTTGGAATTGGATAAATGGGACAGTTTGTGGAATTGGTTTAGCATAACAAAGTGGCTGTGGTATATAAGAATATTCATAATGATAGTAGGAGGCTTGGTAGGTTTAAGAATAGTTTTTGCTGTACTTTCTATAGTGAATAGAGTTAGGCAGGGATACTCACCCTTATCGTTTCAGACCCGC---CTCCCAACCCCGAGGGGA------CCCGACAGGCCCGAAGGAATCGAAGAAGAAGGTGGAGACAGAGACAGAGACAGATCCACGACATTAGTGCAAGGATTCTTAGCACTTATCTGGGTCGACCTGAGGAGCCTGTGCATTTTCATCTACCACCGCTTGAGAGACTTACTCTTGATTGTAACGAGGATTGTGGAACTTCTGGGACGCCGG---------------GGGTGGGAACTCCTCAAATATTGGTGG---AATCTCCTACAATATTGG---------------------------------------------------AGTCAGGAACTAAAGAATAGTGCTGTTAGCTTGCTCAACACCACAGCCGTAGCAGTAGCTGAGGGGACAGATAGGGTCATAGAAGCATTACAAAGA------------------GTTGGTAGAGGTATCCTTCATATACCTACAAGAATAAGACAGGGCTTAGAAAGGGCTTTGCTATAA

2.1056.SPD.EU575284 ATG---------GAGATCAGGAAGAATTATCAGCACTTG---------TGGAGATGGGGG------------------------ATCATGCTCCTTTGGTTATTAATGAGC------------TGTAGTGCTGAA---------GAAGAAGCGTGGGTCACAGTTTATTATGGGGTACCTGTGTGGAAAGAAGCAGTCACCACTCTATTTTGTGCATCAGACGCAAAAGCATATGATACAGAGGTACATAAT---GTTTGGACCACACATGCCTGTGTACCCACAGACCCCGACCCACAAGAAGTACACATG---GAAAATGTGACAGAAGATTTTAACATGTGGAAAAATAACATGGCAGATCAGATGCATGAGGATATAATCAGTTTATGGGATCAAAGTCTAAAGCCATGTGTAAAATTAACCCCACTCTGTGTTACTTTAAATTGTGCTGATTGGAAGAATAATACTGATACCAATACC---------------------------------------------------------------------------------------AATAGTAGTGTGAGAATAATGGAGAAAGGAGAAATAAAAAACTGCTCTTTCAATATCACC---ACAAACATAAGAGAT------AAGTATCAGAAAGCATATGCACTTTTTTATAAACTTGATGTAGTACCAATAGATGATGAT------------------------------AATGCAACA------GGTAATAATGATACTAGAAACTATAGGTTGATAAGTTGTAACACCTCAGTCATTACACAGGCCTGTCCAAAGGTATCCTTTGAACCAATTCCCATACATTATTGTGCCCCGGCTGGTTTTGCGATTTTAAGGTGT---AATAATAAGACATTCAGTGGAAAAGGACAATGTACAAATGTCAGCACAGTACAATGTACACATGGAATTAAGCCAGTAGTATCAACTCAACTGCTATTAAATGGCAGTCTAGCAGAAGAA---GAGGTAATAATTAGATCTGACAATTTCTCGGACAATGCTAAAACCATAATAGTACATCTAAACAGCTCTGTAGACATTAATTGTACAAGACCAGGCAACAATACAAGAAAAAGTATAACTATAGGA------------CCAGGGAGG---GCATTTTATGCAACAGGAGACATAATAGGAGATATAAGACAAGCACATTGTAACATT------AGTGGAGAAAAATGGAATAACACTTTAAAACAGGTAGTTAAAAAATTAAGA---GAACAATTTGGG------------AATAAAACA---ATAGTCTTTAATCAA---------TCCTCAGGAGGGGACCCAGAAATTACAATGCACACTTTTAATTGTGGAGGGGAATTCTTCTACTGTAATACAGCACAACTGTTTAATAGTACTTGGGAAGCT---------AATAGTACTTGGGAAAATGAT---------------------AATGAAAGGGTAGGTCAC------------AGTAACAAGACT---------------------------ATCATACTACAATGCAGAATAAAACAAATTATAAACATGTGGCAGGAAGTAGGAAAAGCAATGTATGCCCCTCCCATCAGCGGACAGATTAGATGTTCATCAAATATTACGGGGCTGCTATTAACAAGAGATGGTGGTAACGGT------------AACGAGACC---------------------AACCGGACC---GAGGTCTTCAGACCTGGAGGAGGAAATATGAAAGATAACTGGAGA---AGTGAATTATATAAATATAAAGTAGTAAAAATTGAACCA---TTAGGAGTAGCACCC---ACCAGGGCAAAGAGAAGAGTGGTGCAAAGAGAA---AAAAGAGCAGTG---GGA---ATGGGA---GCTTTG---TTCATT---GGG---------TTCTTGTCA---GCAGCAGGAAGCACTATGGGCGCAGCGTCAATG---ACGCTGACGGTACAGGCCAGACAATTATTGTCTGGTATAGTGCAACAGCAGAGCAATTTGCTGAGAGCTATTGAGGCGCAACAACATCTGTTGCAACTCACAGTCTGGGGCATCAAGCAGCTCCAGGCAAGA---GTCCTGGCTGTGGAAAGATACCTAAAGGATCAACAGCTCCTAGGGATTTGGGGTTGCTCTGGAAAACTCATTTGCACCACTACAGTGCCTTGGAATTATAGTTGGAGTCCT------------------------AATAAAACTATGGATGACATTTGGGGT---AACATGACCTGGATGCAATGGGAAAGAGAAATTGAC------AATTATACAGGCATAATATACAGATTAATTGAAATATCGCAAAACCAGCAAGAAAAGAATGAACAAGAATTATTGGAATTAGATAAATGGGCAAGTTTGTGGAATTGGTTTGACATAACAAAGTGGCTGTGGTATATAAAAATATTCATAATGATAATAGGAGGCTTAGTAGGTTTAAGAATAGTCTTTACTGTGCTTTCTATAGTAAATAGAGTTAGGCAGGGATACTCACCATTATCGTTTCAGACCCGC---TTCCCAGCCCCAGGGGGA------CCCGACAGGCCCGAAGGAACAGAAGAAGAAGGTGGAGAGAGAGACAGAGACAGATCCAGTCGATCAGCGGATGGATTCTTAGCAATTATCTGGGTCGATCTGAGGAGCCTGTGCCTGTTCATCTACCACAGCTTGAGAGACTTACTCTTGATTGTAGCAAGGATTGTGGGACTTCTGGGACGCAGG---------------GGGTGGGAACTCCTCAAATATTGGTGG---AATCTCCTCCAGTATTGG---------------------------------------------------AGTCAGGAACTAAAGAATAGTGCTGTTAGCTTGCTGAATGCCACAGCTATAGCAGTAGCTGAGGGGACAGATAGGGTTATAGACATAGTACAAAGA------------------ATTTGCAGAGCTATCCTCCACATACCTAGAAGAATAAGACAGGGCTTTGAAAGGGCTTTGCTATAA

2.1056.SPD.EU575285 ATG---------GAGATCAGGAAGAATTATCAGCACTTG---------TGGAGATGGGGG------------------------ATCATGCTCCTTTGGTTATTAATGAGC------------TGTAGTGCTGAA---------GAAGAAGCGTGGGTCACAGTTTATTATGGGGTACCTGTGTGGAAAGAAGCAGTCACCACTCTATTTTGTGCATCAGACGCAAAAGCATATGATACAGAGGTACATAAT---GTTTGGACCACACATGCCTGTGTACCCACAGACCCCGACCCACAAGAAGTACACATG---GAAAATGTGACAGAAGATTTTAACATGTGGAAAAATAACATGGCAGATCAGATGCATGAGGATATAATCAGTTTATGGGATCAAAGTCTAAAGCCATGTGTAAAATTAACCCCACTCTGTGTTACTTTAAATTGTGCTGATTGGAAGAATAATACTGATACCAATACC---------------------------------------------------------------------------------------AATAGTAGTGTGAGAATAATGGAGAAAGGAGAAATAAAAAACTGCTCTTTCAATATCACC---ACAAACATAAGAGAT------AAGTATCAGAAAGCATATGCACTTTTTTATAAACTTGATGTAGTACCAATAGATGATGAT------------------------------AATGCAACA------GGTAATAATGATACTAGAAACTATAGGTTGATAAGTTGTAACACCTCAGTCATTACACAGGCCTGTCCAAAGGTATCCTTTGAACCAATTCCCATACATTATTGTGCCCCGGCTGGTTTTGCGATTTTAAGGTGT---AATAATAAGACATTCAGTGGAAAAGGACAATGTACAAATGTCAGCACAGTACAATGTACACATGGAATTAAGCCAGTAGTATCAACTCAACTGCTATTAAATGGCAGTCTAGCAGAAGAA---GAGGTAATAATTAGATCTGACAATTTCTCGGACAATGCTAAAACCATAATAGTACATCTAAACAGCTCTGTAGACATTAATTGTACAAGACCAGGCAACAATACAAGAAAAAGTATAACTATAGGA------------CCAGGGAGG---GCATTTTATGCAACAGGAGACATAATAGGAGATATAAGACAAGCACATTGTAACATT------AGTGGAGAAAAATGGAATAACACTTTAAAACAGGTAGTTAAAAAATTAAGA---GAACAATTTGGG------------AATAAAACA---ATAGTCTTTAATCAA---------TCCTCAGGAGGGGACCCAGAAATTACAATGCACACTTTTAATTGTGGAGGGGAATTCTTCTACTGTAATACAGCACAACTGTTTAATAGTACTTGGGAAGCT---------AATAGTACTTGGGAAAATGAT---------------------AATGAAAGGGTAGGTCAC------------AGTAACAAGACT---------------------------ATCATACTACAATGCAGAATAAAACAAATTATAAACATGTGGCAGGAAGTAGGAAAAGCAATGTATGCCCCTCCCATCAGCGGACAGATTAGATGTTCATCAAATATTACGGGGCTGCTATTAACAAGAGATGGTGGTAACGGT------------AACGAGACC---------------------AACCGGACC---GAGGTCTTCAGACCTGGAGGAGGAAATATGAAAGATAACTGGAGA---AGTGAATTATATAAATATAAAGTAGTAAAAATTGAACCA---TTAGGAGTAGCACCC---ACCAGGGCAAAGAGAAGAGTGGTGCAAAGAGAA---AAAAGAGCAGTG---GGA---ATGGGA---GCTTTG---TTCATT---GGG---------TTCTTGTCA---GCAGCAGGAAGCACTATGGGCGCAGCGTCAATG---ACGCTGACGGTACAGGCCAGACAATTATTGTCTGGTATAGTGCAACAGCAGAGCAATTTGCTGAGAGCTATTGAGGCGCAACAACATCTGTTGCAACTCACAGTCTGGGGCATCAAGCAGCTCCAGGCAAGA---GTCCTGGCTGTGGAAAGATACCTAAAGGATCAACAGCTCCTAGGGATTTGGGGTTGCTCTGGAAAACTCATTTGCACCACTACAGTGCCTTGGAATTATAGTTGGAGTCCT------------------------AATAAAACTATGGATGACATTTGGGGT---AACATGACCTGGATGCAATGGGAAAGAGAAATTGAC------AATTATACAGGCATAATATACAGATTAATTGAAATATCGCAAAACCAGCAAGAAAAGAATGAACAAGAATTATTGGAATTAGATAAATGGGCAAGTTTGTGGAATTGGTTTGACATAACAAAGTGGCTGTGGTATATAAAAATATTCATAATGATAATAGGAGGCTTAGTAGGTTTAAGAATAGTCTTTACTGTGCTTTCTATAGTAAATAGAGTTAGGCAGGGATACTCACCATTATCGTTTCAGACCCGC---TTCCCAGCCCCAGGGGGA------CCCGACAGGCCCGAAGGAACAGAAGAAGAAGGTGGAGAGAGAGACAGAGACAGATCCAGTCGATCAGCGGATGGATTCTTAGCAATTATCTGGGTCGATCTGAGGAGCCTGTGCCTGTTCATCTACCACAGCTTGAGAGACTTACTCTTGATTGTAGCAAGGATTGTGGGACTTCTGGGACGCAGG---------------GGGTGGGAACTCCTCAAATATTGGTGG---AATCTCCTCCAGTATTGG---------------------------------------------------AGTCAGGAACTAAAGAATAGTGCTGTTAGCTTGCTGAATGCCACAGCTATAGCAGTAGCTGAGGGGACAGATAGGGTTATAGACATAGTACAAAGA------------------ATTTGCAGAGCTATCCTCCACATACCTAGAAGAATAAGACAGGGCTTTGAAAGGGCTTTGCTATAA

2.1056.SPD.EU575288 ATG---------GAGATCAGGAAGAATTATCAGCACTTG---------TGGAGATGGGGG------------------------ATCATGCTCCTTTGGTTATTAATGAGC------------TGTAGTGCTGAA---------GAAGAAGCGTGGGTCACAGTTTATTATGGGGTACCTGTGTGGAAAGAAGCAGTCACCACTCTATTTTGTGCATCAGACGCAAAAGCATATGATACAGAGGTACATAAT---GTTTGGACCACACATGCCTGTGTACCCACAGACCCCGACCCACAAGAAGTACACATG---GAAAATGTGACAGAAGATTTTAACATGTGGAAAAATAACATGGCAGATCAGATGCATGAGGATATAATCAGTTTATGGGATCAAAGTCTAAAGCCATGTGTAAAATTAACCCCACTCTGTGTTACTTTAAATTGTGCTGATTGGAAGAATAATACTGATACCAATACC---------------------------------------------------------------------------------------AATAGTAGTGTGAGAATAATGGAGAAAGGAGAAATAAAAAACTGCTCTTTCAATATCACC---ACAAACATAAGAGAT------AAGTATCAGAAAGCATATGCACTTTTTTATAAACTTGATGTAGTACCAATAGATGATGAT------------------------------AATGCAACA------GGTAATAATGATACTAGAAACTATAGGTTGATAAGTTGTAACACCTCAGTCATTACACAGGCCTGTCCAAAGGTATCCTTTGAACCAATTCCCATACATTATTGTGCCCCGGCTGGTTTTGCGATTTTAAGGTGT---AATAATAAGACATTCAGTGGAAAAGGACAATGTACAAATGTCAGCACAGTACAATGTACACATGGAATTAAGCCAGTAGTATCAACTCAACTGCTATTAAATGGCAGTCTAGCAGAAGAA---GAGGTAATAATTAGATCTGACAATTTCTCGGACAATGCTAAAACCATAATAGTACATCTAAACAGCTCTGTAGACATTAATTGTACAAGACCAGGCAACAATACAAGAAAAAGTATAACTATAGGA------------CCAGGGAGG---GCATTTTATGCAACAGGAGACATAATAGGAGATATAAGACAAGCACATTGTAACATT------AGTGGAGAAAAATGGAATAACACTTTAAAACAGGTAGTTAAAAAATTAAGA---GAACAATTTGGG------------AATAAAACA---ATAGTCTTTAATCAA---------TCCTCAGGAGGGGACCCAGAAATTACAATGCACACTTTTAATTGTGGAGGGGAATTCTTCTACTGTAATACAGCACAACTGTTTAATAGTACTTGGGAAGCT---------AATAGTACTTGGGAAAATGAT---------------------AATGAAAGGGTAGGTCAC------------AGTAACAAGACT---------------------------ATCATACTACAATGCAGAATAAAACAAATTATAAACATGTGGCAGGAAGTAGGAAAAGCAATGTATGCCCCTCCCATCAGCGGACAGATTAGATGTTCATCAAATATTACGGGGCTGCTATTAACAAGAGATGGTGGTAACGGT------------AACGAGACC---------------------AACCGGACC---GAGGTCTTCAGACCTGGAGGAGGAAATATGAAAGATAACTGGAGA---AGTGAATTATATAAATATAAAGTAGTAAAAATTGAACCA---TTAGGAGTAGCACCC---ACCAGGGCAAAGAGAAGAGTGGTGCAAAGAGAA---AAAAGAGCAGTG---GGA---ATGGGA---GCTTTG---TTCATT---GGG---------TTCTTGTCA---GCAGCAGGAAGCACTATGGGCGCAGCGTCAATG---ACGCTGACGGTACAGGCCAGACAATTATTGTCTGGTATAGTGCAACAGCAGAGCAATTTGCTGAGAGCTATTGAGGCGCAACAACATCTGTTGCAACTCACAGTCTGGGGCATCAAGCAGCTCCAGGCAAGA---GTCCTGGCTGTGGAAAGATACCTAAAGGATCAACAGCTCCTAGGGATTTGGGGTTGCTCTGGAAAACTCATTTGCACCACTACAGTGCCTTGGAATTATAGTTGGAGTCCT------------------------AATAAAACTATGGATGACATTTGGGGT---AACATGACCTGGATGCAATGGGAAAGAGAAATTGAC------AATTATACAGGCATAATATACAGATTAATTGAAATATCGCAAAACCAGCAAGAAAAGAATGAACAAGAATTATTGGAATTAGATAAATGGGCAAGTTTGTGGAATTGGTTTGACATAACAAAGTGGCTGTGGTATATAAAAATATTCATAATGATAATAGGAGGCTTAGTAGGTTTAAGAATAGTCTTTACTGTGCTTTCTATAGTAAATAGAGTTAGGCAGGGATACTCACCATTATCGTTTCAGACCCGC---TTCCCAGCCCCAGGGGGA------CCCGACAGGCCCGAAGGAACAGAAGAAGAAGGTGGAGAGAGAGACAGAGACAGATCCAGTCGATCAGCGGATGGATTCTTAGCAATTATCTGGGTCGATCTGAGGAGCCTGTGCCTGTTCATCTACCACAGCTTGAGAGACTTACTCTTGATTGTAGCAAGGATTGTGGGACTTCTGGGACGCAGG---------------GGGTGGGAACTCCTCAAATATTGGTGG---AATCTCCTCCAGTATTGG---------------------------------------------------AGTCAGGAACTAAAGAATAGTGCTGTTAGCTTGCTGAATGCCACAGCTATAGCAGTAGCTGAGGGGACAGATAGGGTTATAGACATAGTACAAAGA------------------ATTTGCAGAGCTATCCTCCACATACCTAGAAGAATAAGACAGGGCTTTGAAAGGGCTTTGCTATAA

2.1056.SPD.EU575322 ATG---------GAGATCAGGAAGAATTATCAGCACTTG---------TGGAGATGGGGG------------------------ATCATGCTCCTTTGGTTATTAATGAGC------------TGTAGTGCTGAA---------GAAGAAGCGTGGGTCACAGTTTATTATGGGGTACCTGTGTGGAAAGAAGCAGTCACCACTCTATTTTGTGCATCAGACGCAAAAGCATATGATACAGAGGTACATAAT---GTTTGGACCACACATGCCTGTGTACCCACAGACCCCGACCCACAAGAAGTACACATG---GAAAATGTGACAGAAGATTTTAACATGTGGAAAAATAACATGGCAGATCAGATGCATGAGGATATAATCAGTTTATGGGATCAAAGTCTAAAGCCATGTGTAAAATTAACCCCACTCTGTGTTACTTTAAATTGTGCTGATTGGAAGAATAATACTGATACCAATACC---------------------------------------------------------------------------------------AATAGTAGTGTGAGAATAATGGAGAAAGGAGAAATAAAAAACTGCTCTTTCAATATCACC---ACAAACATAAGAGAT------AAGTATCAGAAAGCATATGCACTTTTTTATAAACTTGATGTAGTACCAATAGATGATGAT------------------------------AATGCAACA------GGTAATAATGATACTAGAAACTATAGGTTGATAAGTTGTAACACCTCAGTCATTACACAGGCCTGTCCAAAGGTATCCTTTGAACCAATTCCCATACATTATTGTGCCCCGGCTGGTTTTGCGATTTTAAGGTGT---AATAATAAGACATTCAGTGGAAAAGGACAATGTACAAATGTCAGCACAGTACAATGTACACATGGAATTAAGCCAGTAGTATCAACTCAACTGCTATTAAATGGCAGTCTAGCAGAAGAA---GAGGTAATAATTAGATCTGACAATTTCTCGGACAATGCTAAAACCATAATAGTACATCTAAACAGCTCTGTAGACATTAATTGTACAAGACCAGGCAACAATACAAGAAAAAGTATAACTATAGGA------------CCAGGGAGG---GCATTTTATGCAACAGGAGACATAATAGGAGATATAAGACAAGCACATTGTAACATT------AGTGGAGAAAAATGGAATAACACTTTAAAACAGGTAGTTAAAAAATTAAGA---GAACAATTTGGG------------AATAAAACA---ATAGTCTTTAATCAA---------TCCTCAGGAGGGGACCCAGAAATTACAATGCACACTTTTAATTGTGGAGGGGAATTCTTCTACTGTAATACAGCACAACTGTTTAATAGTACTTGGGAAGCT---------AATAGTACTTGGGAAAATGAT---------------------AATGAAAGGGTAGGTCAC------------AGTAACAAGACT---------------------------ATCATACTACAATGCAGAATAAAACAAATTATAAACATGTGGCAGGAAGTAGGAAAAGCAATGTATTCCCCTCCCATCAGCGGACAGATTAGATGTTCATCAAATATTACGGGGCTGCTATTAACAAGAGATGGTGGTAACGGT------------AACGAGACC---------------------AACCGGACC---GAGGTCTTCAGACCTGGAGGAGGAAATATGAAAGATAACTGGAGA---AGTGAATTATATAAATATAAAGTAGTAAAAATTGAACCA---TTAGGAGTAGCACCC---ACCAGGGCAAAGAGAAGAGTGGTGCAAAGAGAA---AAAAGAGCAGTG---GGA---ATGGGA---GCTTTG---TTCATT---GGG---------TTCTTGTCA---GCAGCAGGAAGCACTATGGGCGCAGCGTCAATG---ACGCTGACGGTACAGGCCAGACAATTATTGTCTGGTATAGTGCAACAGCAGAGCAATTTGCTGAGAGCTATTGAGGCGCAACAACATCTGTTGCAACTCACAGTCTGGGGCATCAAGCAGCTCCAGGCAAGA---GTCCTGGCTGTGGAAAGATACCTAAAGGATCAACAGCTCCTAGGGATTTGGGGTTGCTCTGGAAAACTCATTTGCACCACTACAGTGCCTTGGAATTATAGTTGGAGTCCT------------------------AATAAAACTATGGATGACATTTGGGGT---AACATGACCTGGATGCAATGGGAAAGAGAAATTGAC------AATTATACAGGCATAATATACAGATTAATTGAAATATCGCAAAACCAGCAAGAAAAGAATGAACAAGAATTATTGGAATTAGATAAATGGGCAAGTTTGTGGAATTGGTTTGACATAACAAAGTGGCTGTGGTATATAAAAATATTCATAATGATAATAGGAGGCTTAGTAGGTTTAAGAATAGTCTTTACTGTGCTTTCTATAGTAAATAGAGTTAGGCAGGGATACTCACCATTATCGTTTCAGACCCGC---TTCCCAGCCCCAGGGGGA------CCCGACAGGCCCGAAGGAACAGAAGAAGAAGGTGGAGAGAGAGACAGAGACAGATCCAGTCGATCAGCGGATGGATTCTTAGCAATTATCTGGGTCGATCTGAGGAGCCTGTGCCTGTTCATCTACCACAGCTTGAGAGACTTACTCTTGATTGTAGCAAGGATTGTGGGACTTCTGGGACGCAGG---------------GGGTGGGAACTCCTCAAATATTGGTGG---AATCTCCTCCAGTATTGG---------------------------------------------------AGTCAGGAACTAAAGAATAGTGCTGTTAGCTTGCTGAATGCCACAGCTATAGCAGTAGCTGAGGGGACAGATAGGGTTATAGACATAGTACAAAGA------------------ATTTGCAGAGCTATCCTCCACATACCTAGAAGAATAAGACAGGGCTTTGAAAGGGCTTTGCTATAA

2.1056.SPD.EU575296 ATG---------GAGATCAGGAAGAATTATCAGCACTTG---------TGGAGATGGGGG------------------------ATCATGCTCCTTTGGTTATTAATGAGC------------TGTAGTGCTGAA---------GAAGAAGCGTGGGTCACAGTTTATTATGGGGTACCTGTGTGGAAAGAAGCAGTCACCACTCTATTTTGTGCATCAGACGCAAAAGCATATGATACAGAGGTACATAAT---GTTTGGACCACACATGCCTGTGTACCCACAGACCCCGACCCACAAGAAGTACACATG---GAAAATGTGACAGAAGATTTTAACATGTGGAAAAATAACATGGCAGATCAGATGCATGAGGATATAATCAGTTTATGGGATCAAAGTCTAAAGCCATGTGTAAAATTAACCCCACTCTGTGTTACTTTAAATTGTGCTGATTGGAAGAATAATACTGATACCAATACC---------------------------------------------------------------------------------------AATAGTAGTGTGAGAATAATGGAGAAAGGAGAAATAAAAAACTGCTCTTTCAATATCACC---ACAAACATAAGAGAT------AAGTATCAGAAAGCATATGCACTTTTTTATAAACTTGATGTAGTACCAATAGATGATGAT------------------------------AATGCAACA------GGTAATAATGATACTAGAAACTATAGGTTGATAAGTTGTAACACCTCAGTCATTACACAGGCCTGTCCAAAGGTATCCTTTGAACCAATTCCCATACATTATTGTGCCCCGGCTGGTTTTGCGATTTTAAGGTGT---AATAATAAGACATTCAGTGGAAAAGGACAATGTACAAATGTCAGCACAGTACAATGTACACATGGAATTAAGCCAGTAGTATCAACTCAACTGCTATTAAATGGCAGTCTAGCAGAAGAA---GAGGTAATAATTAGATCTGACAATTTCTCGGACAATGCTAAAACCATAATAGTACATCTAAACAGCTCTGTAGACATTAATTGTACAAGACCAGGCAACAATACAAGAAAAAGTATAACTATAGGA------------CCAGGGAGG---GCATTTTATGCAACAGGAGACATAATAGGAGATATAAGACAAGCACATTGTAACATT------AGTGGAGAAAAATGGAATAACACTTTAAAACAGGTAGTTAAAAAATTAAGA---GAACAATTTGGG------------AATAAAACA---ATAGTCTTTAATCAA---------TCCTCAGGAGGGGACCCAGAAATTACAATGCACACTTTTAATTGTGGAGGGGAATTCTTCTACTGTAATACAGCACAACTGTTTAATAGTACTTGGGAAGCT---------AATAGTACTTGGGAAAATGAT---------------------AATGAAAGGGTAGGTCAC------------AGTAACAAGACT---------------------------ATCATACTACAATGCAGAATAAAACAAATTATAAACATGTGGCAGGAAGTAGGAAAAGCAATGTATGCCCCTCCCATCAGCGGACAGATTAGATGTTCATCAAATATTACGGGGCTGCTATTAACAAGAGATGGTGGTAACGGT------------AACGAGACC---------------------AACCGGACC---GAGGTCTTCAGACCTGGAGGAGGAAATATGAAAGATAACTGGAGA---AGTGAATTATATAAATATAAAGTAGTAAAAATTGAACCA---TTAGGAGTAGCACCC---ACCAGGGCAAAGAGAAGAGTGGTGCAAAGAGAA---AAAAGAGCAGTG---GGA---ATGGGA---GCTTTG---TTCATT---GGG---------TTCTTGTCA---GCAGCAGGAAGCACTATGGGCGCAGCGTCAATG---ACGCTGACGGTACAGGCCAGACAATTATTGTCTGGTATAGTGCAACAGCAGAGCAATTTGCTGAGAGCTATTGAGGCGCAACAACATCTGTTGCAACTCACAGTCTGGGGCATCAAGCAGCTCCAGGCAAGA---GTCCTGGCTGTGGAAAGATACCTAAAGGATCAACAGCTCCTAGGGATTTGGGGTTGCTCTGGAAAACTCATTTGCACCACTACAGTGCCTTGGAATTATAGTTGGAGTCCT------------------------AATAAAACTATGGATGACATTTGGGGT---AACATGACCTGGATGCAATGGGAAAGAGAAATTGAC------AATTATACAGGCATAATATACAGATTAATTGAAATATCGCAAAACCAGCAAGAAAAGAATGAACAAGAATTATTGGAATTAGATAAATGGGCAAGTTTGTGGAATTGGTTTGACATAACAAAGTGGCTGTGGTATATAAAAATATTCATAATGATAATAGGAGGCTTAGTAGGTTTAAGAATAGTCTTTACTGTGCTTTCTATAGTAAATAGAGTTAGGCAGGGATACTCACCATTATCGTTTCAGACCCGC---TTCCCAGCCCCAGGGGGA------CCCGACAGGCCCGAAGGAACAGAAGAAGAAGGTGGAGAGAGAGACAGAGACAGATCCAGTCGATCAGCGGATGGATTCTTAGCAATTATCTGGGTCGATCTGAGGAGCCTGTGCCTGTTCATCTACCACAGCTTGAGAGACTTACTCTTGATTGTAGCAAGGATTGTGGGACTTCTGGGACGCAGG---------------GGGTGGGAACTCCTCAAATATTGGTGG---AATCTCCTCCAGTATTGG---------------------------------------------------AGTCAGGAACTAAAGAATAGTGCTGTTAGCTTGCTGAATGCCACAGCTATAGCAGTAGCTGAGGGGACAGATAGGGTTATAGACATAGTACAAAGA------------------ATTTGCAGAGCTATCCTCCACATACCTAGAAGAATAAGACAGGGCTTTGAAAGGGCTTTGCTATAA

2.1056.SPD.EU575300 ATG---------GAGATCAGGAAGAATTATCAGCACTTG---------TGGAGATGGGGG------------------------ATCATGCTCCTTTGGTTATTAATGAGC------------TGTAGTGCTGAA---------GAAGAAGCGTGGGTCACAGTTTATTATGGGGTACCTGTGTGGAAAGAAGCAGTCACCACTCTATTTTGTGCATCAGACGCAAAAGCATATGATACAGAGGTACATAAT---GTTTGGACCACACATGCCTGTGTACCCACAGACCCCGACCCACAAGAAGTACACATG---GAAAATGTGACAGAAGATTTTAACATGTGGAAAAATAACATGGCAGATCAGATGCATGAGGATATAATCAGTTTATGGGATCAAAGTCTAAAGCCATGTGTAAAATTAACCCCACTCTGTGTTACTTTAAATTGTGCTGATTGGAAGAATAATACTGATACCAATACC---------------------------------------------------------------------------------------AATAGTAGTGTGAGAATAATGGAGAAAGGAGAAATAAAAAACTGCTCTTTCAATATCACC---ACAAACATAAGAGAT------AAGTATCAGAAAGCATATGCACTTTTTTATAAACTTGATGTAGTACCAATAGATGATGAT------------------------------AATGCAACA------GGTAATAATGATACTAGAAACTATAGGTTGATAAGTTGTAACACCTCAGTCATTACACAGGCCTGTCCAAAGGTATCCTTTGAACCAATTCCCATACATTATTGTGCCCCGGCTGGTTTTGCGATTTTAAGGTGT---AATAATAAGACATTCAGTGGAAAAGGACAATGTACAAATGTCAGCACAGTACAATGTACACATGGAATTAAGCCAGTAGTATCAACTCAACTGCTATTAAATGGCAGTCTAGCAGAAGAA---GAGGTAATAATTAGATCTGACAATTTCTCGGACAATGCTAAAACCATAATAGTACATCTAAACAGCTCTGTAGACATTAATTGTACAAGACCAGGCAACAATACAAGAAAAAGTATAACTATAGGA------------CCAGGGAGG---GCATTTTATGCAACAGGAGACATAATAGGAGATATAAGACAAGCACATTGTAACATT------AGTGGAGAAAAATGGAATAACACTTTAAAACAGGTAGTTAAAAAATTAAGA---GAACAATTTGGG------------AATAAAACA---ATAGTCTTTAATCAA---------TCCTCAGGAGGGGACCCAGAAATTACAATGCACACTTTTAATTGTGGAGGGGAATTCTTCTACTGTAATACAGCACAACTGTTTAATAGTACTTGGGAAGCT---------AATAGTACTTGGGAAAATGAT---------------------AATGAAAGGGTAGGTCAC------------AGTAACAAGACT---------------------------ATCATACTACAATGCAGAATAAAACAAATTATAAACATGTGGCAGGAAGTAGGAAAAGCAATGTATGCCCCTCCCATCAGCGGACAGATTAGATGTTCATCAAATATTACGGGGCTGCTATTAACAAGAGATGGTGGTAACGGT------------AACGAGACC---------------------AACCGGACC---GAGGTCTTCAGACCTGGAGGAGGAAATATGAAAGATAACTGGAGA---AGTGAATTATATAAATATAAAGTAGTAAAAATTGAACCA---TTAGGAGTAGCACCC---ACCAGGGCAAAGAGAAGAGTGGTGCAAAGAGAA---AAAAGAGCAGTG---GGA---ATGGGA---GCTTTG---TTCATT---GGG---------TTCTTGTCA---GCAGCAGGAAGCACTATGGGCGCAGCGTCAATG---ACGCTGACGGTACAGGCCAGACAATTATTGTCTGGTATAGTGCAACAGCAGAGCAATTTGCTGAGAGCTATTGAGGCGCAACAACATCTGTTGCAACTCACAGTCTGGGGCATCAAGCAGCTCCAGGCAAGA---GTCCTGGCTGTGGAAAGATACCTAAAGGATCAACAGCTCCTAGGGATTTGGGGTTGCTCTGGAAAACTCATTTGCACCACTACAGTGCCTTGGAATTATAGTTGGAGTCCT------------------------AATAAAACTATGGATGACATTTGGGGT---AACATGACCTGGATGCAATGGGAAAGAGAAATTGAC------AATTATACAGGCATAATATACAGATTAATTGAAATATCGCAAAACCAGCAAGAAAAGAATGAACAAGAATTATTGGAATTAGATAAATGGGCAAGTTTGTGGAATTGGTTTGACATAACAAAGTGGCTGTGGTATATAAAAATATTCATAATGATAATAGGAGGCTTAGTAGGTTTAAGAATAGTCTTTACTGTGCTTTCTATAGTAAATAGAGTTAGGCAGGGATACTCACCATTATCGTTTCAGACCCGC---TTCCCAGCCCCAGGGGGA------CCCGACAGGCCCGAAGGAACAGAAGAAGAAGGTGGAGAGAGAGACAGAGACAGATCCAGTCGATCAGCGGATGGATTCTTAGCAATTATCTGGGTCGATCTGAGGAGCCTGTGCCTGTTCATCTACCACAGCTTGAGAGACTTACTCTTGATTGTAGCAAGGATTGTGGGACTTCTGGGACGCAGG---------------GGGTGGGAACTCCTCAAATATTGGTGG---AATCTCCTCCAGTATTGG---------------------------------------------------AGTCAGGAACTAAAGAATAGTGCTGTTAGCTTGCTGAATGCCACAGCTATAGCAGTAGCTGAGGGGACAGATAGGGTTATAGACATAGTACAAAGA------------------ATTTGCAGAGCTATCCTCCACATACCTAGAAGAATAAGACAGGGCTTTGAAAGGGCTTTGCTATAA

2.1056.SPD.EU575326 ATG---------GAGATCAGGAAGAATTATCAGCACTTG---------TGGAGATGGGGG------------------------ATCATGCTCCTTTGGTTATTAATGAGC------------TGTAGTGCTGAA---------GAAGAAGCGTGGGTCACAGTTTATTATGGGGTACCTGTGTGGAAAGAAGCAGTCACCACTCTATTTTGTGCATCAGACGCAAAAGCATATGATACAGAGGTACATAAT---GTTTGGACCACACATGCCTGTGTACCCACAGACCCCGACCCACAAGAAGTACACATG---GAAAATGTGACAGAAGATTTTAACATGTGGAAAAATAACATGGCAGATCAGATGCATGAGGATATAATCAGTTTATGGGATCAAAGTCTAAAGCCATGTGTAAAATTAACCCCACTCTGTGTTACTTTAAATTGTGCTGATTGGAAGAATAATACTGATACCAATACC---------------------------------------------------------------------------------------AATAGTAGTGTGAGAATAATGGAGAAAGGAGAAATAAAAAACTGCTCTTTCAATATCACC---ACAAACATAAGAGAT------AAGTATCAGAAAGCATATGCACTTTTTTATAAACTTGATGTAGTACCAATAGATGATGAT------------------------------AATGCAACA------GGTAATAATGATACTAGAAACTATAGGTTGATAAGTTGTAACACCTCAGTCATTACACAGGCCTGTCCAAAGGTATCCTTTGAACCAATTCCCATACATTATTGTGCCCCGGCTGGTTTTGCGATTTTAAGGTGT---AATAATAAGACATTCAGTGGAAAAGGACAATGTACAAATGTCAGCACAGTACAATGTACACATGGAATTAAGCCAGTAGTATCAACTCAACTGCTATTAAATGGCAGTCTAGCAGAAGAA---GAGGTAATAATTAGATCTGACAATTTCTCGGACAATGCTAAAACCATAATAGTACATCTAAACAGCTCTGTAGACATTAATTGTACAAGACCAGGCAACAATACAAGAAAAAGTATAACTATAGGA------------CCAGGGAGG---GCATTTTATGCAACAGGAGACATAATAGGAGATATAAGACAAGCACATTGTAACATT------AGTGGAGAAAAATGGAATAACACTTTAAAACAGGTAGTTAAAAAATTAAGA---GAACAATTTGGG------------AATAAAACA---ATAGTCTTTAATCAA---------TCCTCAGGAGGGGACCCAGAAATTACAATGCACACTTTTAATTGTGGAGGGGAATTCTTCTACTGTAATACAGCACAACTGTTTAATAGTACTTGGGAAGCT---------AATAGTACTTGGGAAAATGAT---------------------AATGAAAGGGTAGGTCAC------------AGTAACAAGACT---------------------------ATCATACTACAATGCAGAATAAAACAAATTATAAACATGTGGCAGGAAGTAGGAAAAGCAATGTATGCCCCTCCCATCAGCGGACAGATTAGATGTTCATCAAATATTACGGGGCTGCTATTAACAAGAGATGGTGGTAACGGT------------AACGAGACC---------------------AACCGGACC---GAGGTCTTCAGACCTGGAGGAGGAAATATGAAAGATAACTGGAGA---AGTGAATTATATAAATATAAAGTAGTAAAAATTGAACCA---TTAGGAGTAGCACCC---ACCAGGGCAAAGAGAAGAGTGGTGCAAAGAGAA---AAAAGAGCAGTG---GGA---ATGGGA---GCTTTG---TTCATT---GGG---------TTCTTGTCA---GCAGCAGGAAGCACTATGGGCGCAGCGTCAATG---ACGCTGACGGTACAGGCCAGACAATTATTGTCTGGTATAGTGCAACAGCAGAGCAATTTGCTGAGAGCTATTGAGGCGCAACAACATCTGTTGCAACTCACAGTCTGGGGCATCAAGCAGCTCCAGGCAAGA---GTCCTGGCTGTGGAAAGATACCTAAAGGATCAACAGCTCCTAGGGATTTGGGGTTGCTCTGGAAAACTCATTTGCACCACTACAGTGCCTTGGAATTATAGTTGGAGTCCT------------------------AATAAAACTATGGATGACATTTGGGGT---AACATGACCTGGATGCAATGGGAAAGAGAAATTGAC------AATTATACAGGCATAATATACAGATTAATTGAAATATCGCAAAACCAGCAAGAAAAGAATGAACAAGAATTATTGGAATTAGATAAATGGGCAAGTTTGTGGAATTGGTTTGACATAACAAAGTGGCTGTGGTATATAAAAATATTCATAATGATAATAGGAGGCTTAGTAGGTTTAAGAATAGTCTTTACTGTGCTTTCTATAGTAAATAGAGTTAGGCAGGGATACTCACCATTATCGTTTCAGACCCGC---TTCCCAGCCCCAGGGGGA------CCCGACAGGCCCGAAGGAACAGAAGAAGAAGGTGGAGAGAGAGACAGAGACAGATCCAGTCGATCAGCGGATGGATTCTTAGCAATTATCTGGGTCGATCTGAGGAGCCTGTGCCTGTTCATCTACCACAGCTTGAGAGACTTACTCTTGATTGTAGCAAGGATTGTGGGACTTCTGGGACGCAGG---------------GGGTGGGAACTCCTCAAATATTGGTGG---AATCTCCTCCAGTATTGG---------------------------------------------------AGTCAGGAACTAAAGAATAGTGCTGTTAGCTTGCTGAATGCCACAGCTATAGCAGTAGCTGAGGGGACAGATAGGGTTATAGACATAGTACAAAGA------------------ATTTGCAGAGCTATCCTCCACATACCTAGAAGAATAAGACAGGGCTTTGAAAGGGCTTTGCTATAA

2.1056.SPD.EU575295 ATG---------GAGATCAGGAAGAATTATCAGCACTTG---------TGGAGATGGGGG------------------------ATCATGCTCCTTTGGTTATTAATGAGC------------TGTAGTGCTGAA---------GAAGAAGCGTGGGTCACAGTTTATTATGGGGTACCTGTGTGGAAAGAAGCAGTCACCACTCTATTTTGTGCATCAGACGCAAAAGCATATGATACAGAGGTACATAAT---GTTTGGACCACACATGCCTGTGTACCCACAGACCCCGACCCACAAGAAGTACACATG---GAAAATGTGACAGAAGATTTTAACATGTGGAAAAATAACATGGCAGATCAGATGCATGAGGATATAATCAGTTTATGGGATCAAAGTCTAAAGCCATGTGTAAAATTAACCCCACTCTGTGTTACTTTAAATTGTGCTGATTGGAAGAATAATACTGATACCAATACC---------------------------------------------------------------------------------------AATAGTAGTGTGAGAATAATGGAGAAAGGAGAAATAAAAAACTGCTCTTTCAATATCACC---ACAAACATAAGAGAT------AAGTATCAGAAAGCATATGCACTTTTTTATAAACTTGATGTAGTACCAATAGATGATGAT------------------------------AATGCAACA------GGTAATAATGATACTAGAAACTATAGGTTGATAAGTTGTAACACCTCAGTCATTACACAGGCCTGTCCAAAGGTATCCTTTGAACCAATTCCCATACATTATTGTGCCCCGGCTGGTTTTGCGATTTTAAGGTGT---AATAATAAGACATTCAGTGGAAAAGGACAATGTACAAATGTCAGCACAGTACAATGTACACATGGAATTAAGCCAGTAGTATCAACTCAACTGCTATTAAATGGCAGTCTAGCAGAAGAA---GAGGTAATAATTAGATCTGACAATTTCTCGGACAATGCTAAAACCATAATAGTACATCTAAACAGCTCTGTAGACATTAATTGTACAAGACCAGGCAACAATACAAGAAAAAGTATAACTATAGGA------------CCAGGGAGG---GCATTTTATGCAACAGGAGACATAATAGGAGATATAAGACAAGCACATTGTAACATT------AGTGGAGAAAAATGGAATAACACTTTAAAACAGGTAGTTAAAAAATTAAGA---GAACAATTTGGG------------AATAAAACA---ATAGTCTTTAATCAA---------TCCTCAGGAGGGGACCCAGAAATTACAATGCACACTTTTAATTGTGGAGGGGAATTCTTCTACTGTAATACAGCACAACTGTTTAATAGTACTTGGGAAGCT---------AATAGTACTTGGGAAAATGAT---------------------AATGAAAGGGTAGGTCAC------------AGTAACAAGACT---------------------------ATCATACTACAATGCAGAATAAAACAAATTATAAACATGTGGCAGGAAGTAGGAAAAGCAATGTATGCCCCTCCCATCAGCGGACAGATTAGATGTTCATCAAATATTACGGGGCTGCTATTAACAAGAGATGGTGGTAACGGT------------AACGAGACC---------------------AACCGGACC---GAGGTCTTCAGACCTGGAGGAGGAAATATGAAAGATAACTGGAGA---AGTGAATTATATAAATATAAAGTAGTAAAAATTGAACCA---TTAGGAGTAGCACCC---ACCAGGGCAAAGAGAAGAGTGGTGCAAAGAGAA---AAAAGAGCAGTG---GGA---ATGGGA---GCTTTG---TTCATT---GGG---------TTCTTGTCA---GCAGCAGGAAGCACTATGGGCGCAGCGTCAATG---ACGCTGACGGTACAGGCCAGACAATTATTGTCTGGTATAGTGCAACAGCAGAGCAATTTGCTGAGAGCTATTGAGGCGCAACAACATCTGTTGCAACTCACAGTCTGGGGCATCAAGCAGCTCCAGGCAAGA---GTCCTGGCTGTGGAAAGATACCTAAAGGATCAACAGCTCCTAGGGATTTGGGGTTGCTCTGGAAAACTCATTTGCACCACTACAGTGCCTTGGAATTATAGTTGGAGTCCT------------------------AATAAAACTATGGATGACATTTGGGGT---AACATGACCTGGATGCAATGGGAAAGAGAAATTGAC------AATTATACAGGCATAATATACAGATTAATTGAAATATCGCAAAACCAGCAAGAAAAGAATGAACAAGAATTATTGGAATTAGATAAATGGGCAAGTTTGTGGAATTGGTTTGACATAACAAAGTGGCTGTGGTATATAAAAATATTCATAATGATAATAGGAGGCTTAGTAGGTTTAAGAATAGTCTTTACTGTGCTTTCTATAGTAAATAGAGTTAGGCAGGGATACTCACCATTATCGTTTCAGACCCGC---TTCCCAGCCCCAGGGGGA------CCCGACAGGCCCGAAGGAACAGAAGAAGAAGGTGGAGAGAGAGACAGAGACAGATCCAGTCGATCAGCGGATGGATTCTTAGCAATTATCTGGGTCGATCTGAGGAGCCTGTGCCTGTTCATCTACCACAGCTTGAGAGACTTACTCTTGATTGTAGCAAGGATTGTGGGACTTCTGGGACGCAGG---------------GGGTGGGAACTCCTCAAATATTGGTGG---AATCTCCTCCAGTATTGG---------------------------------------------------AGTCAGGAACTAAAGAATAGTGCTGTTAGCTTGCTGAATGCCACAGCTATAGCAGTAGCTGAGGGGACAGATAGGGTTATAGACATAGTACAAAGA------------------ATTTGCAGAGCTATCCTCCACATACCTAGAAGAATAAGACAGGGCTTTGAAAGGGCTTTGCTATAA

2.1056.SPD.EU575308 ATG---------GAGATCAGGAAGAATTATCAGCACTTG---------TGGAGATGGGGG------------------------ATCATGCTCCTTTGGTTATTAATGAGC------------TGTAGTGCTGAA---------GAAGAAGCGTGGGTCACAGTTTATTATGGGGTACCTGTGTGGAAAGAAGCAGTCACCACTCTATTTTGTGCATCAGACGCAAAAGCATATGATACAGAGGTACATAAT---GTTTGGACCACACATGCCTGTGTACCCACAGACCCCGACCCACAAGAAGTACACATG---GAAAATGTGACAGAAGATTTTAACATGTGGAAAAATAACATGGCAGATCAGATGCATGAGGATATAATCAGTTTATGGGATCAAAGTCTAAAGCCATGTGTAAAATTAACCCCACTCTGTGTTACTTTAAATTGTGCTGATTGGAAGAATAATACTGATACCAATACC---------------------------------------------------------------------------------------AATAGTAGTGTGAGAATAATGGAGAAAGGAGAAATAAAAAACTGCTCTTTCAATATCACC---ACAAACATAAGAGAT------AAGTATCAGAAAGCATATGCACTTTTTTATAAACTTGATGTAGTACCAATAGATGATGAT------------------------------AATGCAACA------GGTAATAATGATACTAGAAACTATAGGTTGATAAGTTGTAACACCTCAGTCATTACACAGGCCTGTCCAAAGGTATCCTTTGAACCAATTCCCATACATTATTGTGCCCCGGCTGGTTTTGCGATTTTAAGGTGT---AATAATAAGACATTCAGTGGAAAAGGACAATGTACAAATGTCAGCACAGTACAATGTACACATGGAATTAAGCCAGTAGTATCAACTCAACTGCTATTAAATGGCAGTCTAGCAGAAGAA---GAGGTAATAATTAGATCTGACAATTTCTCGGACAATGCTAAAACCATAATAGTACATCTAAACAGCTCTGTAGACATTAATTGTACAAGACCAGGCAACAATACAAGAAAAAGTATAACTATAGGA------------CCAGGGAGG---GCATTTTATGCAACAGGAGACATAATAGGAGATATAAGACAAGCACATTGTAACATT------AGTGGAGAAAAATGGAATAACACTTTAAAACAGGTAGTTAAAAAATTAAGA---GAACAATTTGGG------------AATAAAACA---ATAGTCTTTAATCAA---------TCCTCAGGAGGGGACCCAGAAATTACAATGCACACTTTTAATTGTGGAGGGGAATTCTTCTACTGTAATACAGCACAACTGTTTAATAGTACTTGGGAAGCT---------AATAGTACTTGGGAAAATGAT---------------------AATGAAAGGGTAGGTCAC------------AGTAACAAGACT---------------------------ATCATACTACAATGCAGAATAAAACAAATTATAAACATGTGGCAGGAAGTAGGAAAAGCAATGTATGCCCCTCCCATCAGCGGACAGATTAGATGTTCATCAAATATTACGGGGCTGCTATTAACAAGAGATGGTGGTAACGGT------------AACGAGACC---------------------AACCGGACC---GAGGTCTTCAGACCTGGAGGAGGAAATATGAAAGATAACTGGAGA---AGTGAATTATATAAATATAAAGTAGTAAAAATTGAACCA---TTAGGAGTAGCACCC---ACCAGGGCAAAGAGAAGAGTGGTGCAAAGAGAA---AAAAGAGCAGTG---GGA---ATGGGA---GCTTTG---TTCATT---GGG---------TTCTTGTCA---GCAGCAGGAAGCACTATGGGCGCAGCGTCAATG---ACGCTGACGGTACAGGCCAGACAATTATTGTCTGGTATAGTGCAACAGCAGAGCAATTTGCTGAGAGCTATTGAGGCGCAACAACATCTGTTGCAACTCACAGTCTGGGGCATCAAGCAGCTCCAGGCAAGA---GTCCTGGCTGTGGAAAGATACCTAAAGGATCAACAGCTCCTAGGGATTTGGGGTTGCTCTGGAAAACTCATTTGCACCACTACAGTGCCTTGGAATTATAGTTGGAGTCCT------------------------AATAAAACTATGGATGACATTTGGGGT---AACATGACCTGGATGCAATGGGAAAGAGAAATTGAC------AATTATACAGGCATAATATACAGATTAATTGAAATATCGCAAAACCAGCAAGAAAAGAATGAACAAGAATTATTGGAATTAGATAAATGGGCAAGTTTGTGGAATTGGTTTGACATAACAAAGTGGCTGTGGTATATAAAAATATTCATAATGATAATAGGAGGCTTAGTAGGTTTAAGAATAGTCTTTACTGTGCTTTCTATAGTAAATAGAGTTAGGCAGGGATACTCACCATTATCGTTTCAGACCCGC---TTCCCAGCCCCAGGGGGA------CCCGACAGGCCCGAAGGAACAGAAGAAGAAGGTGGAGAGAGAGACAGAGACAGATCCAGTCGATCAGCGGATGGATTCTTAGCAATTATCTGGGTCGATCTGAGGAGCCTGTGCCTGTTCATCTACCACAGCTTGAGAGACTTACTCTTGATTGTAGCAAGGATTGTGGGACTTCTGGGACGCAGG---------------GGGTGGGAACTCCTCAAATATTGGTGG---AATCTCCTCCAGTATTGG---------------------------------------------------AGTCAGGAACTAAAGAATAGTGCTGTTAGCTTGCTGAATGCCACAGCTATAGCAGTAGCTGAGGGGACAGATAGGGTTATAGACATAGTACAAAGA------------------ATTTGCAGAGCTATCCTCCACATACCTAGAAGAATAAGACAGGGCTTTGAAAGGGCTTTGCTATAA

2.1056.SPD.EU575314 ATG---------GAGATCAGGAAGAATTATCAGCACTTG---------TGGAGATGGGGG------------------------ATCATGCTCCTTTGGTTATTAATGAGC------------TGTAGTGCTGAA---------GAAGAAGCGTGGGTCACAGTTTATTATGGGGTACCTGTGTGGAAAGAAGCAGTCACCACTCTATTTTGTGCATCAGACGCAAAAGCATATGATACAGAGGTACATAAT---GTTTGGACCACACATGCCTGTGTACCCACAGACCCCGACCCACAAGAAGTACACATG---GAAAATGTGACAGAAGATTTTAACATGTGGAAAAATAACATGGCAGATCAGATGCATGAGGATATAATCAGTTTATGGGATCAAAGTCTAAAGCCATGTGTAAAATTAACCCCACTCTGTGTTACTTTAAATTGTGCTGATTGGAAGAATAATACTGATACCAATACC---------------------------------------------------------------------------------------AATAGTAGTGTGAGAATAATGGAGAAAGGAGAAATAAAAAACTGCTCTTTCAATATCACC---ACAAACATAAGAGAT------AAGTATCAGAAAGCATATGCACTTTTTTATAAACTTGATGTAGTACCAATAGATGATGAT------------------------------AATGCAACA------GGTAATAATGATACTAGAAACTATAGGTTGATAAGTTGTAACACCTCAGTCATTACACAGGCCTGTCCAAAGGTATCCTTTGAACCAATTCCCATACATTATTGTGCCCCGGCTGGTTTTGCGATTTTAAGGTGT---AATAATAAGACATTCAGTGGAAAAGGACAATGTACAAATGTCAGCACAGTACAATGTACACATGGAATTAAGCCAGTAGTATCAACTCAACTGCTATTAAATGGCAGTCTAGCAGAAGAA---GAGGTAATAATTAGATCTGACAATTTCTCGGACAATGCTAAAACCATAATAGTACATCTAAACAGCTCTGTAGACATTAATTGTACAAGACCAGGCAACAATACAAGAAAAAGTATAACTATAGGA------------CCAGGGAGG---GCATTTTATGCAACAGGAGACATAATAGGAGATATAAGACAAGCACATTGTAACATT------AGTGGAGAAAAATGGAATAACACTTTAAAACAGGTAGTTAAAAAATTAAGA---GAACAATTTGGG------------AATAAAACA---ATAGTCTTTAATCAA---------TCCTCAGGAGGGGACCCAGAAATTACAATGCACACTTTTAATTGTGGAGGGGAATTCTTCTACTGTAATACAGCACAACTGTTTAATAGTACTTGGGAAGCT---------AATAGTACTTGGGAAAATGAT---------------------AATGAAAGGGTAGGTCAC------------AGTAACAAGACT---------------------------ATCATACTACAATGCAGAATAAAACAAATTATAAACATGTGGCAGGAAGTAGGAAAAGCAATGTATGCCCCTCCCATCAGCGGACAGATTAGATGTTCATCAAATATTACGGGGCTGCTATTAACAAGAGATGGTGGTAACGGT------------AACGAGACC---------------------AACCGGACC---GAGGTCTTCAGACCTGGAGGAGGAAATATGAAAGATAACTGGAGA---AGTGAATTATATAAATATAAAGTAGTAAAAATTGAACCA---TTAGGAGTAGCACCC---ACCAGGGCAAAGAGAAGAGTGGTGCAAAGAGAA---AAAAGAGCAGTG---GGA---ATGGGA---GCTTTG---TTCATT---GGG---------TTCTTGTCA---GCAGCAGGAAGCACTATGGGCGCAGCGTCAATG---ACGCTGACGGTACAGGCCAGACAATTATTGTCTGGTATAGTGCAACAGCAGAGCAATTTGCTGAGAGCTATTGAGGCGCAACAACATCTGTTGCAACTCACAGTCTGGGGCATCAAGCAGCTCCAGGCAAGA---GTCCTGGCTGTGGAAAGATACCTAAAGGATCAACAGCTCCTAGGGATTTGGGGTTGCTCTGGAAAACTCATTTGCACCACTACAGTGCCTTGGAATTATAGTTGGAGTCCT------------------------AATAAAACTATGGATGACATTTGGGGT---AACATGACCTGGATGCAATGGGAAAGAGAAATTGAC------AATTATACAGGCATAATATACAGATTAATTGAAATATCGCAAAACCAGCAAGAAAAGAATGAACAAGAATTATTGGAATTAGATAAATGGGCAAGTTTGTGGAATTGGTTTGACATAACAAAGTGGCTGTGGTATATAAAAATATTCATAATGATAATAGGAGGCTTAGTAGGTTTAAGAATAGTCTTTACTGTGCTTTCTATAGTAAATAGAGTTAGGCAGGGATACTCACCATTATCGTTTCAGACCCGC---TTCCCAGCCCCAGGGGGA------CCCGACAGGCCCGAAGGAACAGAAGAAGAAGGTGGAGAGAGAGACAGAGACAGATCCAGTCGATCAGCGGATGGATTCTTAGCAATTATCTGGGTCGATCTGAGGAGCCTGTGCCTGTTCATCTACCACAGCTTGAGAGACTTACTCTTGATTGTAGCAAGGATTGTGGGACTTCTGGGACGCAGG---------------GGGTGGGAACTCCTCAAATATTGGTGG---AATCTCCTCCAGTATTGG---------------------------------------------------AGTCAGGAACTAAAGAATAGTGCTGTTAGCTTGCTGAATGCCACAGCTATAGCAGTAGCTGAGGGGACAGATAGGGTTATAGACATAGTACAAAGA------------------ATTTGCAGAGCTATCCTCCACATACCTAGAAGAATAAGACAGGGCTTTGAAAGGGCTTTGCTATAA

2.1056.SPD.EU575327 ATG---------GAGATCAGGAAGAATTATCAGCACTTG---------TGGAGATGGGGG------------------------ATCATGCTCCTTTGGTTATTAATGAGC------------TGTAGTGCTGAA---------GAAGAAGCGTGGGTCACAGTTTATTATGGGGTACCTGTGTGGAAAGAAGCAGTCACCACTCTATTTTGTGCATCAGACGCAAAAGCATATGATACAGAGGTACATAAT---GTTTGGACCACACATGCCTGTGTACCCACAGACCCCGACCCACAAGAAGTACACATG---GAAAATGTGACAGAAGATTTTAACATGTGGAAAAATAACATGGCAGATCAGATGCATGAGGATATAATCAGTTTATGGGATCAAAGTCTAAAGCCATGTGTAAAATTAACCCCACTCTGTGTTACTTTAAATTGTGCTGATTGGAAGAATAATACTGATACCAATACC---------------------------------------------------------------------------------------AATAGTAGTGTGAGAATAATGGAGAAAGGAGAAATAAAAAACTGCTCTTTCAATATCACC---ACAAACATAAGAGAT------AAGTATCAGAAAGCATATGCACTTTTTTATAAACTTGATGTAGTACCAATAGATGATGAT------------------------------AATGCAACA------GGTAATAATGATACTAGAAACTATAGGTTGATAAGTTGTAACACCTCAGTCATTACACAGGCCTGTCCAAAGGTATCCTTTGAACCAATTCCCATACATTATTGTGCCCCGGCTGGTTTTGCGATTTTAAGGTGT---AATAATAAGACATTCAGTGGAAAAGGACAATGTACAAATGTCAGCACAGTACAATGTACACATGGAATTAAGCCAGTAGTATCAACTCAACTGCTATTAAATGGCAGTCTAGCAGAAGAA---GAGGTAATAATTAGATCTGACAATTTCTCGGACAATGCTAAAACCATAATAGTACATCTAAACAGCTCTGTAGACATTAATTGTACAAGACCAGGCAACAATACAAGAAAAAGTATAACTATAGGA------------CCAGGGAGG---GCATTTTATGCAACAGGAGACATAATAGGAGATATAAGACAAGCACATTGTAACATT------AGTGGAGAAAAATGGAATAACACTTTAAAACAGGTAGTTAAAAAATTAAGA---GAACAATTTGGG------------AATAAAACA---ATAGTCTTTAATCAA---------TCCTCAGGAGGGGACCCAGAAATTACAATGCACACTTTTAATTGTGGAGGGGAATTCTTCTACTGTAATACAGCACAACTGTTTAATAGTACTTGGGAAGCT---------AATAGTACTTGGGAAAATGAT---------------------AATGAAAGGGTAGGTCAC------------AGTAACAAGACT---------------------------ATCATACTACAATGCAGAATAAAACAAATTATAAACATGTGGCAGGAAGTAGGAAAAGCAATGTATGCCCCTCCCATCAGCGGACAGATTAGATTTTCATCAAATATTACGGGGCTGCTATTAACAAGAGATGGTGGTAACGGT------------AACGAGACC---------------------AACCGGACC---GAGGTCTTCAGACCTGGAGGAGGAAATATGAAAGATAACTGGAGA---AGTGAATTATATAAATATAAAGTAGTAAAAATTGAACCA---TTAGGAGTAGCACCC---ACCAGGGCAAAGAGAAGAGTGGTGCAAAGAGAA---AAAAGAGCAGTG---GGA---ATGGGA---GCTTTG---TTCATT---GGG---------TTCTTGTCA---GCAGCAGGAAGCACTATGGGCGCAGCGTCAATG---ACGCTGACGGTACAGGCCAGACAATTATTGTCTGGTATAGTGCAACAGCAGAGCAATTTGCTGAGAGCTATTGAGGCGCAACAACATCTGTTGCAACTCACAGTCTGGGGCATCAAGCAGCTCCAGGCAAGA---GTCCTGGCTGTGGAAAGATACCTAAAGGATCAACAGCTCCTAGGGATTTGGGGTTGCTCTGGAAAACTCATTTGCACCACTACAGTGCCTTGGAATTATAGTTGGAGTCCT------------------------AATAAAACTATGGATGACATTTGGGGT---AACATGACCTGGATGCAATGGGAAAGAGAAATTGAC------AATTATACAGGCATAATATACAGATTAATTGAAATATCGCAAAACCAGCAAGAAAAGAATGAACAAGAATTATTGGAATTAGATAAATGGGCAAGTTTGTGGAATTGGTTTGACATAACAAAGTGGCTGTGGTATATAAAAATATTCATAATGATAATAGGAGGCTTAGTAGGTTTAAGAATAGTCTTTACTGTGCTTTCTATAGTAAATAGAGTTAGGCAGGGATACTCACCATTATCGTTTCAGACCCGC---TTCCCAGCCCCAGGGGGA------CCCGACAGGCCCGAAGGAACAGAAGAAGAAGGTGGAGAGAGAGACAGAGACAGATCCAGTCGATCAGCGGATGGATTCTTAGCAATTATCTGGGTCGATCTGAGGAGCCTGTGCCTGTTCATCTACCACAGCTTGAGAGACTTACTCTTGATTGTAGCAAGGATTGTGGGACTTCTGGGACGCAGG---------------GGGTGGGAACTCCTCAAATATTGGTGG---AATCTCCTCCAGTATTGG---------------------------------------------------AGTCAGGAACTAAAGAATAGTGCTGTTAGCTTGCTGAATGCCACAGCTATAGCAGTAGCTGAGGGGACAGATAGGGTTATAGACATAGTACAAAGA------------------ATTTGCAGAGCTATCCTCCACATACCTAGAAGAATAAGACAGGGCTTTGAAAGGGCTTTGCTATAA

2.1056.SPD.EU575304 ATG---------GAGATCAGGAAGAATTATCAGCACTTG---------TGGAGATGGGGG------------------------ATCATGCTCCTTTGGTTATTAATGAGC------------TGTAGTGCTGAA---------GAAGAAGCGTGGGTCACAGTTTATTATGGGGTACCTGTGTGGAAAGAAGCAGTCACCACTCTATTTTGTGCATCAGACGCAAAAGCATATGATACAGAGGTACATAAT---GTTTGGACCACACATGCCTGTGTACCCACAGACCCCGACCCACAAGAAGTACACATG---GAAAATGTGACAGAAGATTTTAACATGTGGAAAAATAACATGGCAGATCAGATGCATGAGGATATAATCAGTTTATGGGATCAAAGTCTAAAGCCATGTGTAAAATTAACCCCACTCTGTGTTACTTTAAATTGTGCTGATTGGAAGAATAATACTGATACCAATACC---------------------------------------------------------------------------------------AATAGTAGTGTGAGAATAATGGAGAAAGGAGAAATAAAAAACTGCTCTTTCAATATCACC---ACAAACATAAGAGAT------AAGTATCAGAAAGCATATGCACTTTTTTATAAACTTGATGTAGTACCAATAGATGATGAT------------------------------AATGCAACA------GGTAATAATGATACTAGAAACTATAGGTTGATAAGTTGTAACACCTCAGTCATTACACAGGCCTGTCCAAAGGTATCCTTTGAACCAATTCCCATACATTATTGTGCCCCGGCTGGTTTTGCGATTTTAAGGTGT---AATAATAAGACATTCAGTGGAAAAGGACAATGTACAAATGTCAGCACAGTACAATGTACACATGGAATTAAGCCAGTAGTATCAACTCAACTGCTATTAAATGGCAGTCTAGCAGAAGAA---GAGGTAATAATTAGATCTGACAATTTCTCGGACAATGCTAAAACCATAATAGTACATCTAAACAGCTCTGTAGACATTAATTGTACAAGACCAGGCAACAATACAAGAAAAAGTATAACTATAGGA------------CCAGGGAGG---GCATTTTATGCAACAGGAGACATAATAGGAGATATAAGACAAGCACATTGTAACATT------AGTGGAGAAAAATGGAATAACACTTTAAAACAGGTAGTTAAAAAATTAAGA---GAACAATTTGGG------------AATAAAACA---ATAGTCTTTAATCAA---------TCCTCAGGAGGGGACCCAGAAATTACAATGCACACTTTTAATTGTGGAGGGGAATTCTTCTACTGTAATACAGCACAACTGTTTAATAGTACTTGGGAAGCT---------AATAGTACTTGGGAAAATGAT---------------------AATGAAAGGGTAGGTCAC------------AGTAACAAGACT---------------------------ATCATACTACAATGCAGAATAAAACAAATTATAAACATGTGGCAGGAAGTAGGAAAAGCAATGTATGCCCCTCCCATCAGCGGACAGATTAGATGTTCATCAAATATTACGGGGCTGCTATTAACAAGAGATGGTGGTAACGGT------------AACGAGACC---------------------AACCGGACC---GAGGTCTTCAGACCTGGAGGAGGAAATATGAAAGATAACTGGAGA---AGTGAATTATATAAATATAAAGTAGTAAAAATTGAACCA---TTAGGAGTAGCACCC---ACCAGGGCAAAGAGAAGAGTGGTGCAAAGAGAA---AAAAGAGCAGTG---GGA---ATGGGA---GCTTTG---TTCATT---GGG---------TTCTTGTCA---GCAGCAGGAAGCACTATGGGCGCAGCGTCAATG---ACGCTGACGGTACAGGCCAGACAATTATTGTCTGGTATAGTGCAACAGCAGAGCAATTTGCTGAGAGCTATTGAGGCGCAACAACATCTGTTGCAACTCACAGTCTGGGGCATCAAGCAGCTCCAGGCAAGA---GTCCTGGCTGTGGAAAGATACCTAAAGGATCAACAGCTCCTAGGGATTTGGGGTTGCTCTGGAAAACTCATTTGCACCACTACAGTGCCTTGGAATTATAGTTGGAGTCCT------------------------AATAAAACTATGGATGACATTTGGGGT---AACATGACCTGGATGCAATGGGAAAGAGAAATTGAC------AATTATACAGGCATAATATACAGATTAATTGAAATATCGCAAAACCAGCAAGAAAAGAATGAACAAGAATTATTGGAATTAGATAAATGGGCAAGTTTGTGGAATTGGTTTGACATAACAAAGTGGCTGTGGTATATAAAAATATTCATAATGATAATAGGAGGCTTAGTAGGTTTAAGAATAGTCTTTACTGTGCTTTCTATAGTAAATAGAGTTAGGCAGGGATACTCACCATTATCGTTTCAGACCCGC---TTCCCAGCCCCAGGGGGA------CCCGACAGGCCCGAAGGAACAGAAGAAGAAGGTGGAGAGAGAGACAGAGACAGATCCAGTCGATCAGCGGATGGATTCTTAGCAATTATCTGGGTCGATCTGAGGAGCCTGTGCCTGTTCATCTACCACAGCTTGAGAGACTTACTCTTGATTGTAGCAAGGATTGTGGGACTTCTGGGACGCAGG---------------GGGTGGGAACTCCTCAAATATTGGTGG---AATCTCCTCCAGTATTGG---------------------------------------------------AGTCAGGAACTAAAGAATAGTGCTGTTAGCTTGCTGAATGCCACAGCTATAGCAGTAGCTGAGGGGACAGATAGGGTTATAGACATAGTACAAAGA------------------ATTTGCAGAGCTATCCTCCACATACCTAGAAGAATAAGACAGGGCTTTGAAAGGGCTTTGCTATAA

2.1056.SPD.EU575323 ATG---------GAGATCAGGAAGAATTATCAGCACTTG---------TGGAGATGGGGG------------------------ATCATGCTCCTTTGGTTATTAATGAGC------------TGTAGTGCTGAA---------GAAGAAGCGTGGGTCACAGTTTATTATGGGGTACCTGTGTGGAAAGAAGCAGTCACCACTCTATTTTGTGCATCAGACGCAAAAGCATATGATACAGAGGTACATAAT---GTTTGGACCACACATGCCTGTGTACCCACAGACCCCGACCCACAAGAAGTACACATG---GAAAATGTGACAGAAGATTTTAACATGTGGAAAAATAACATGGCAGATCAGATGCATGAGGATATAATCAGTTTATGGGATCAAAGTCTAAAGCCATGTGTAAAATTAACCCCACTCTGTGTTACTTTAAATTGTGCTGATTGGAAGAATAATACTGATACCAATACC---------------------------------------------------------------------------------------AATAGTAGTGTGAGAATAATGGAGAAAGGAGAAATAAAAAACTGCTCTTTCAATATCACC---ACAAACATAAGAGAT------AAGTATCAGAAAGCATATGCACTTTTTTATAAACTTGATGTAGTACCAATAGATGATGAT------------------------------AATGCAACA------GGTAATAATGATACTAGAAACTATAGGTTGATAAGTTGTAACACCTCAGTCATTACACAGGCCTGTCCAAAGGTATCCTTTGAACCAATTCCCATACATTATTGTGCCCCGGCTGGTTTTGCGATTTTAAGGTGT---AATAATAAGACATTCAGTGGAAAAGGACAATGTACAAATGTCAGCACAGTACAATGTACACATGGAATTAAGCCAGTAGTATCAACTCAACTGCTATTAAATGGCAGTCTAGCAGAAGAA---GAGGTAATAATTAGATCTGACAATTTCTCGGACAATGCTAAAACCATAATAGTACATCTAAACAGCTCTGTAGACATTAATTGTACAAGACCAGGCAACAATACAAGAAAAAGTATAACTATAGGA------------CCAGGGAGG---GCATTTTATGCAACAGGAGACATAATAGGAGATATAAGACAAGCACATTGTAACATT------AGTGGAGAAAAATGGAATAACACTTTAAAACAGGTAGTTAAAAAATTAAGA---GAACAATTTGGG------------AATAAAACA---ATAGTCTTTAATCAA---------TCCTCAGGAGGGGACCCAGAAATTACAATGCACACTTTTAATTGTGGAGGGGAATTCTTCTACTGTAATACAGCACAACTGTTTAATAGTACTTGGGAAGCT---------AATAGTACTTGGGAAAATGAT---------------------AATGAAAGGGTAGGTCAC------------AGTAACAAGACT---------------------------ATCATACTACAATGCAGAATAAAACAAATTATAAACATGTGGCAGGAAGTAGGAAAAGCAATGTATGCCCCTCCCATCAGCGGACAGATTAGATGTTCATCAAATATTACGGGGCTGCTATTAACAAGAGATGGTGGTAACGGT------------AACGAGACC---------------------AACCGGACC---GAGGTCTTCAGACCTGGAGGAGGAAATATGAAAGATAACTGGAGA---AGTGAATTATATAAATATAAAGTAGTAAAAATTGAACCA---TTAGGAGTAGCACCC---ACCAGGGCAAAGAGAAGAGTGGTGCAAAGAGAA---AAAAGAGCAGTG---GGA---ATGGGA---GCTTTG---TTCATT---GGG---------TTCTTGTCA---GCAGCAGGAAGCACTATGGGCGCAGCGTCAATG---ACGCTGACGGTACAGGCCAGACAATTATTGTCTGGTATAGTGCAACAGCAGAGCAATTTGCTGAGAGCTATTGAGGCGCAACAACATCTGTTGCAACTCACAGTCTGGGGCATCAAGCAGCTCCAGGCAAGA---GTCCTGGCTGTGGAAAGATACCTAAAGGATCAACAGCTCCTAGGGATTTGGGGTTGCTCTGGAAAACTCATTTGCACCACTACAGTGCCTTGGAATTATAGTTGGAGTCCT------------------------AATAAAACTATGGATGACATTTGGGGT---AACATGACCTGGATGCAATGGGAAAGAGAAATTGAC------AATTATACAGGCATAATATACAGATTAATTGAAATATCGCAAAACCAGCAAGAAAAGAATGAACAAGAATTATTGGAATTAGATAAATGGGCAAGTTTGTGGAATTGGTTTGACATAACAAAGTGGCTGTGGTATATAAAAATATTCATAATGATAATAGGAGGCTTAGTAGGTTTAAGAATAGTCTTTACTGTGCTTTCTATAGTAAATAGAGTTAGGCAGGGATACTCACCATTATCGTTTCAGACCCGC---TTCCCAGCCCCAGGGGGA------CCCGACAGGCCCGAAGGAACAGAAGAAGAAGGTGGAGAGAGAGACAGAGACAGATCCAGTCGATCAGCGGATGGATTCTTAGCAATTATCTGGGTCGATCTGAGGAGCCTGTGCCTGTTCATCTACCACAGCTTGAGAGACTTACTCTTGATTGTAGCAAGGATTGTGGGACTTCTGGGACGCAGG---------------GGGTGGGAACTCCTCAAATATTGGTGG---AATCTCCTCCAGTATTGG---------------------------------------------------AGTCAGGAACTAAAGAATAGTGCTGTTAGCTTGCTGAATGCCACAGCTATAGCAGTAGCTGAGGGGACAGATAGGGTTATAGACATAGTACAAAGA------------------ATTTGCAGAGCTATCCTCCACATACCTAGAAGAATAAGACAGGGCTTTGAAAGGGCTTTGCTATAA

2.1056.SPD.EU575291 ATG---------GAGATCAGGAAGAATTATCAGCACTTG---------TGGAGATGGGGG------------------------ATCATGCTCCTTTGGTTATTAATGAGC------------TGTAGTGCTGAA---------GAAGAAGCGTGGGTCACAGTTTATTATGGGGTACCTGTGTGGAAAGAAGCAGTCACCACTCTATTTTGTGCATCAGACGCAAAAGCATATGATACAGAGGTACATAAT---GTTTGGACCACACATGCCTGTGTACCCACAGACCCCGACCCACAAGAAGTACACATG---GAAAATGTGACAGAAGATTTTAACATGTGGAAAAATAACATGGCAGATCAGATGCATGAGGATATAATCAGTTTATGGGATCAAAGTCTAAAGCCATGTGTAAAATTAACCCCACTCTGTGTTACTTTAAATTGTGCTGATTGGAAGAATAATACTGATACCAATACC---------------------------------------------------------------------------------------AATAGTAGTGTGAGAATAATGGAGAAAGGAGAAATAAAAAACTGCTCTTTCAATATCACC---ACAAACATAAGAGAT------AAGTATCAGAAAGCATATGCACTTTTTTATAAACTTGATGTAGTACCAATAGATGATGAT------------------------------AATGCAACA------GGTAATAATGATACTAGAAACTATAGGTTGATAAGTTGTAACACCTCAGTCATTACACAGGCCTGTCCAAAGGTATCCTTTGAACCAATTCCCATACATTATTGTGCCCCGGCTGGTTTTGCGATTTTAAGGTGT---AATAATAAGACATTCAGTGGAAAAGGACAATGTACAAATGTCAGCACAGTACAATGTACACATGGAATTAAGCCAGTAGTATCAACTCAACTGCTATTAAATGGCAGTCTAGCAGAAGAA---GAGGTAATAATTAGATCTGACAATTTCTCGGACAATGCTAAAACCATAATAGTACATCTAAACAGCTCTGTAGACATTAATTGTACAAGACCAGGCAACAATACAAGAAAAAGTATAACTATAGGA------------CCAGGGAGG---GCATTTTATGCAACAGGAGACATAATAGGAGATATAAGACAAGCACATTGTAACATT------AGTGGAGAAAAATGGAATAACACTTTAAAACAGGTAGTTAAAAAATTAAGA---GAACAATTTGGG------------AATAAAACA---ATAGTCTTTAATCAA---------TCCTCAGGAGGGGACCCAGAAATTACAATGCACACTTTTAATTGTGGAGGGGAATTCTTCTACTGTAATACAGCACAACTGTTTAATAGTACTTGGGAAGCT---------AATAGTACTTGGGAAAATGAT---------------------AATGAAAGGGTAGGTCAC------------AGTAACAAGACT---------------------------ATCATACTACAATGCAGAATAAAACAAATTATAAACATGTGGCAGGAAGTAGGAAAAGCAATGTATGCCCCTCCCATCAGCGGACAGATTAGATGTTCATCAAATATTACGGGGCTGCTATTAACAAGAGATGGTGGTAACGGT------------AACGAGACC---------------------AACCGGACC---GAGGTCTTCAGACCTGGAGGAGGAAATATGAAAGATAACTGGAGA---AGTGAATTATATAAATATAAAGTAGTAAAAATTGAACCA---TTAGGAGTAGCACCC---ACCAGGGCAAAGAGAAGAGTGGTGCAAAGAGAA---AAAAGAGCAGTG---GGA---ATGGGA---GCTTTG---TTCATT---GGG---------TTCTTGTCA---GCAGCAGGAAGCACTATGGGCGCAGCGTCAATG---ACGCTGACGGTACAGGCCAGACAATTATTGTCTGGTATAGTGCAACAGCAGAGCAATTTGCTGAGAGCTATTGAGGCGCAACAACATCTGTTGCAACTAACAGTCTGGGGCATCAAGCAGCTCCAGGCAAGA---GTCCTGGCTGTGGAAAGATACCTAAAGGATCAACAGCTCCTAGGGATTTGGGGTTGCTCTGGAAAACTCATTTGCACCACTACAGTGCCTTGGAATTATAGTTGGAGTCCT------------------------AATAAAACTATGGATGACATTTGGGGT---AACATGACCTGGATGCAATGGGAAAGAGAAATTGAC------AATTATACAGGCATAATATACAGATTAATTGAAATATCGCAAAACCAGCAAGAAAAGAATGAACAAGAATTATTGGAATTAGATAAATGGGCAAGTTTGTGGAATTGGTTTGACATAACAAAGTGGCTGTGGTATATAAAAATATTCATAATGATAATAGGAGGCTTAGTAGGTTTAAGAATAGTCTTTACTGTGCTTTCTATAGTAAATAGAGTTAGGCAGGGATACTCACCATTATCGTTTCAGACCCGC---TTCCCAGCCCCAGGGGGA------CCCGACAGGCCCGAAGGAACAGAAGAAGAAGGTGGAGAGAGAGACAGAGACAGATCCAGTCGATCAGCGGATGGATTCTTAGCAATTATCTGGGTCGATCTGAGGAGCCTGTGCCTGTTCATCTACCACAGCTTGAGAGACTTACTCTTGATTGTAGCAAGGATTGTGGGACTTCTGGGACGCAGG---------------GGGTGGGAACTCCTCAAATATTGGTGG---AATCTCCTCCAGTATTGG---------------------------------------------------AGTCAGGAACTAAAGAATAGTGCTGTTAGCTTGCTGAATGCCACAGCTATAGCAGTAGCTGAGGGGACAGATAGGGTTATAGACATAGTACAAAGA------------------ATTTGCAGAGCTATCCTCCACATACCTAGAAGAATAAGACAGGGCTTTGAAAGGGCTTTGCTATAA

2.1056.SPD.EU575298 ATG---------GAGATCAGGAAGAATTATCAGCACTTG---------TGGAGATGGGGG------------------------ATCATGCTCCTTTGGTTATTAATGAGC------------TGTAGTGCTGAA---------GAAGAAGCGTGGGTCACAGTTTATTATGGGGTACCTGTGTGGAAAGAAGCAGTCACCACTCTATTTTGTGCATCAGACGCAAAAGCATATGATACAGAGGTACATAAT---GTTTGGACCACACATGCCTGTGTACCCACAGACCCCGACCCACAAGAAGTACACATG---GAAAATGTGACAGAAGATTTTAACATGTGGAAAAATAACATGGCAGATCAGATGCATGAGGATATAATCAGTTTATGGGATCAAAGTCTAAAGCCATGTGTAAAATTAACCCCACTCTGTGTTACTTTAAATTGTGCTGATTGGAAGAATAATACTGATACCAATACC---------------------------------------------------------------------------------------AATAGTAGTGTGAGAATAATGGAGAAAGGAGAAATAAAAAACTGCTCTTTCAATATCACC---ACAAACATAAGAGAT------AAGTATCAGAAAGCATATGCACTTTTTTATAAACTTGATGTAGTACCAATAGATGATGAT------------------------------AATGCAACA------GGTAATAATGATACTAGAAACTATAGGTTGATAAGTTGTAACACCTCAGTCATTACACAGGCCTGTCCAAAGGTATCCTTTGAACCAATTCCCATACATTATTGTGCCCCGGCTGGTTTTGCGATTTTAAGGTGT---AATAATAAGACATTCAGTGGAAAAGGACAATGTACAAATGTCAGCACAGTACAATGTACACATGGAATTAAGCCAGTAGTATCAACTCAACTGCTATTAAATGGCAGTCTAGCAGAAGAA---GAGGTAATAATTAGATCTGACAATTTCTCGGACAATGCTAAAACCATAATAGTACATCTAAACAGCTCTGTAGACATTAATTGTACAAGACCAGGCAACAATACAAGAAAAAGTATAACTATAGGA------------CCAGGGAGG---GCATTTTATGCAACAGGAGACATAATAGGAGATATAAGACAAGCACATTGTAACATT------AGTGGAGAAAAATGGAATAACACTTTAAAACAGGTAGTTAAAAAATTAAGA---GAACAATTTGGG------------AATAAAACA---ATAGTCTTTAATCAA---------TCCTCAGGAGGGGACCCAGAAATTACAATGCACACTTTTAATTGTGGAGGGGAATTCTTCTACTGTAATACAGCACAACTGTTTAATAGTACTTGGGAAGCT---------AATAGTACTTGGGAAAATGAT---------------------AATGAAAGGGTAGGTCAC------------AGTAACAAGACT---------------------------ATCATACTACAATGCAGAATAAAACAAATTATAAACATGTGGCAGGAAGTAGGAAAAGCAATGTATGCCCCTCCCATCAGCGGACAGATTAGATGTTCATCAAATATTACGGGGCTGCTATTAGCAAGAGATGGTGGTAACGGT------------AACGAGACC---------------------AACCGGACC---GAGGTCTTCAGACCTGGAGGAGGAAATATGAAAGATAACTGGAGA---AGTGAATTATATAAATATAAAGTAGTAAAAATTGAACCA---TTAGGAGTAGCACCC---ACCAGGGCAAAGAGAAGAGTGGTGCAAAGAGAA---AAAAGAGCAGTG---GGA---ATGGGA---GCTTTG---TTCATT---GGG---------TTCTTGTCA---GCAGCAGGAAGCACTATGGGCGCAGCGTCAATG---ACGCTGACGGTACAGGCCAGACAATTATTGTCTGGTATAGTGCAACAGCAGAGCAATTTGCTGAGAGCTATTGAGGCGCAACAACATCTGTTGCAACTCACAGTCTGGGGCATCAAGCAGCTCCAGGCAAGA---GTCCTGGCTGTGGAAAGATACCTAAAGGATCAACAGCTCCTAGGGATTTGGGGTTGCTCTGGAAAACTCATTTGCACCACTACAGTGCCTTGGAATTATAGTTGGAGTCCT------------------------AATAAAACTATGGATGACATTTGGGGT---AACATGACCTGGATGCAATGGGAAAGAGAAATTGAC------AATTATACAGGCATAATATACAGATTAATTGAAATATCGCAAAACCAGCAAGAAAAGAATGAACAAGAATTATTGGAATTAGATAAATGGGCAAGTTTGTGGAATTGGTTTGACATAACAAAGTGGCTGTGGTATATAAAAATATTCATAATGATAATAGGAGGCTTAGTAGGTTTAAGAATAGTCTTTACTGTGCTTTCTATAGTAAATAGAGTTAGGCAGGGATACTCACCATTATCGTTTCAGACCCGC---TTCCCAGCCCCAGGGGGA------CCCGACAGGCCCGAAGGAACAGAAGAAGAAGGTGGAGAGAGAGACAGAGACAGATCCAGTCGATCAGCGGATGGATTCTTAGCAATTATCTGGGTCGATCTGAGGAGCCTGTGCCTGTTCATCTACCACAGCTTGAGAGACTTACTCTTGATTGTAGCAAGGATTGTGGGACTTCTGGGACGCAGG---------------GGGTGGGAACTCCTCAAATATTGGTGG---AATCTCCTCCAGTATTGG---------------------------------------------------AGTCAGGAACTAAAGAATAGTGCTGTTAGCTTGCTGAATGCCACAGCTATAGCAGTAGCTGAGGGGACAGATAGGGTTATAGACATAGTACAAAGA------------------ATTTGCAGAGCTATCCTCCACATACCTAGAAGAATAAGACAGGGCTTTGAAAGGGCTTTGCTATAA

2.1056.SPD.EU575317 ATG---------GAGATCAGGAAGAATTATCAGCACTTG---------TGGAGATGGGGG------------------------ATCATGCTCCTTTGGTTATTAATGAGC------------TGTAGTGCTGAA---------GAAGAAGCGTGGGTCACAGTTTATTATGGGGTACCTGTGTGGAAAGAAGCAGTCACCACTCTATTTTGTGCATCAGACGCAAAAGCATATGATACAGAGGTACATAAT---GTTTGGACCACACATGCCTGTGTACCCACAGACCCCGACCCACAAGAAGTACACATG---GAAAATGTGACAGAAGATTTTAACATGTGGAAAAATAACATGGCAGATCAGATGCATGAGGATATAATCAGTTTATGGGATCAAAGTCTAAAGCCATGTGTAAAATTAACCCCACTCTGTGTTACTTTAAATTGTGCTGATTGGAAGAATAATACTGATACCAATACC---------------------------------------------------------------------------------------AATAGTAGTGTGAGAATAATGGAGAAAGGAGAAATAAAAAACTGCTCTTTCAATATCACC---ACAAACATAAGAGAT------AAGTATCAGAAAGCATATGCACTTTCTTATAAACTTGATGTAGTACCAATAGATGATGAT------------------------------AATGCAACA------GGTAATAATGATACTAGAAACTATAGGTTGATAAGTTGTAACACCTCAGTCATTACACAGGCCTGTCCAAAGGTATCCTTTGAACCAATTCCCATACATTATTGTGCCCCGGCTGGTTTTGCGATTTTAAGGTGT---AATAATAAGACATTCAGTGGAAAAGGACAATGTACAAATGTCAGCACAGTACAATGTACACATGGAATTAAGCCAGTAGTATCAACTCAACTGCTATTAAATGGCAGTCTAGCAGAAGAA---GAGGTAATAATTAGATCTGACAATTTCTCGGACAATGCTAAAACCATAATAGTACATCTAAACAGCTCTGTAGACATTAATTGTACAAGACCAGGCAACAATACAAGAAAAAGTATAACTATAGGA------------CCAGGGAGG---GCATTTTATGCAACAGGAGACATAATAGGAGATATAAGACAAGCACATTGTAACATT------AGTGGAGAAAAATGGAATAACACTTTAAAACAGGTAGTTAAAAAATTAAGA---GAACAATTTGGG------------AATAAAACA---ATAGTCTTTAATCAA---------TCCTCAGGAGGGGACCCAGAAATTACAATGCACACTTTTAATTGTGGAGGGGAATTCTTCTACTGTAATACAGCACAACTGTTTAATAGTACTTGGGAAGCT---------AATAGTACTTGGGAAAATGAT---------------------AATGAAAGGGTAGGTCAC------------AGTAACAAGACT---------------------------ATCATACTACAATGCAGAATAAAACAAATTATAAACATGTGGCAGGAAGTAGGAAAAGCAATGTATGCCCCTCCCATCAGCGGACAGATTAGATGTTCATCAAATATTACGGGGCTGCTATTAACAAGAGATGGTGGTAACGGT------------AACGAGACC---------------------AACCGGACC---GAGGTCTTCAGACCTGGAGGAGGAAATATGAAAGATAACTGGAGA---AGTGAATTATATAAATATAAAGTAGTAAAAATTGAACCA---TTAGGAGTAGCACCC---ACCAGGGCAAAGAGAAGAGTGGTGCAAAGAGAA---AAAAGAGCAGTG---GGA---ATGGGA---GCTTTG---TTCATT---GGG---------TTCTTGTCA---GCAGCAGGAAGCACTATGGGCGCAGCGTCAATG---ACGCTGACGGTACAGGCCAGACAATTATTGTCTGGTATAGTGCAACAGCAGAGCAATTTGCTGAGAGCTATTGAGGCGCAACAACATCTGTTGCAACTCACAGTCTGGGGCATCAAGCAGCTCCAGGCAAGA---GTCCTGGCTGTGGAAAGATACCTAAAGGATCAACAGCTCCTAGGGATTTGGGGTTGCTCTGGAAAACTCATTTGCACCACTACAGTGCCTTGGAATTATAGTTGGAGTCCT------------------------AATAAAACTATGGATGACATTTGGGGT---AACATGACCTGGATGCAATGGGAAAGAGAAATTGAC------AATTATACAGGCATAATATACAGATTAATTGAAATATCGCAAAACCAGCAAGAAAAGAATGAACAAGAATTATTGGAATTAGATAAATGGGCAAGTTTGTGGAATTGGTTTGACATAACAAAGTGGCTGTGGTATATAAAAATATTCATAATGATAATAGGAGGCTTAGTAGGTTTAAGAATAGTCTTTACTGTGCTTTCTATAGTAAATAGAGTTAGGCAGGGATACTCACCATTATCGTTTCAGACCCGC---TTCCCAGCCCCAGGGGGA------CCCGACAGGCCCGAAGGAACAGAAGAAGAAGGTGGAGAGAGAGACAGAGACAGATCCAGTCGATCAGCGGATGGATTCTTAGCAATTATCTGGGTCGATCTGAGGAGCCTGTGCCTGTTCATCTACCACAGCTTGAGAGACTTACTCTTGATTGTAGCAAGGATTGTGGGACTTCTGGGACGCAGG---------------GGGTGGGAACTCCTCAAATATTGGTGG---AATCTCCTCCAGTATTGG---------------------------------------------------AGTCAGGAACTAAAGAATAGTGCTGTTAGCTTGCTGAATGCCACAGCTATAGCAGTAGCTGAGGGGACAGATAGGGTTATAGACATAGTACAAAGA------------------ATTTGCAGAGCTATCCTCCACATACCTAGAAGAATAAGACAGGGCTTTGAAAGGGCTTTGCTATAA

2.1056.SPD.EU575299 ATG---------GAGATCAGGAAGAATTATCAGCACTTG---------TGGAGATGGGGG------------------------ATCATGCTCCTTTGGTTATTAATGAGC------------TGTAGTGCTGAA---------GAAGAAGCGTGGGTCACAGTTTATTATGGGGTACCTGTGTGGAAAGAAGCAGTCACCACTCTATTTTGTGCATCAGACGCAAAAGCATATGATACAGAGGTACATAAT---GTTTGGACCACACATGCCTGTGTACCCACAGACCCCGACCCACAAGAAGTACACATG---GAAAATGTGACAGAAGATTTTAACATGTGGAAAAATAACATGGCAGATCAGATGCATGAGGATATAATCAGTTTATGGGATCAAAGTCTAAAGCCATGTGTAAAATTAACCCCACTCTGTGTTACTTTAAATTGTGCTGATTGGAAGAATAATACTGATACCAATACC---------------------------------------------------------------------------------------AATAGTAGTGTGAGAATAATGGAGAAAGGAGAAATAAAAAACTGCTCTTTCAATATCACC---ACAAACATAAGAGAT------AAGTATCAGAAAGCATATGCACTTTTTTATAAACTTGATGTAGTACCAATAGATGATGAT------------------------------AATGCAACA------GGTAATAATGATACTAGAAACTATAGGTTGATAAGTTGTAACACCTCAGTCATTACACAGGCCTGTCCAAAGGTATCCTTTGAACCAATTCCCATACATTATTGTGCCCCGGCTGGTTTTGCGATTTTAAGGTGT---AATAATAAGACATTCAGTGGAAAAGGACAATGTACAAATGTCAGCACAGTACAATGTACACATGGAATTAAGCCAGTAGTATCAACTCAACTGCTATTAAATGGCAGTCTAGCAGAAGAA---GAGGTAATAATTAGATCTGACAATTTCTCGGACAATGCTAAAACCATAATAGTACATCTAAACAGCTCTGTAGACATTAATTGTACAAGACCAGGCAACAATACAAGAAAAAGTATAACTATAGGA------------CCAGGGAGG---GCATTTTATGCAACAGGAGACATAATAGGAGATATAAGACAAGCACATTGTAACATT------AGTGGAGAAAAATGGAATAACACTTTAAAACAGGTAGTTAAAAAATTAAGA---GAACAATTTGGG------------AATAAAACA---ATAGTCTTTAATCAA---------TCCTCAGGAGGGGACCCAGAAATTACAATGCACACTTTTAATTGTGGAGGGGAATTCTTCTACTGTAATACAGCACAACTGTTTAATAGTACTTGGGAAGCT---------AATAGTACTTGGGAAAATGAT---------------------AATGAAAGGGTAGGTCAC------------AGTAACAAGACT---------------------------ATCATACTACAATGCAGAATAAAACAAATTATAAACATGTGGCAGGAAGTAGGAAAAGCAATGTATGCCCCTCCCATCAGCGGACAGATTAGATGTTCATCAAATATTACGGGGCTGCTATTAACAAGAGATGGTGGTAACGGT------------AACGAGACC---------------------AACCGGACC---GAGGTCTTCAGACCTGGAGGAGGAAATATGAAAGATAACTGGAGA---AGTGAATTATATAAATATAAAGTAGTAAAAATTGAACCA---TTAGGAGTAGCACCC---ACCAGGGCAAAGAGAAGAGTGGTGCAAAGAGAA---AAAAGAGCAGTG---GGA---ATGGGA---GCTTTG---TTCATT---GGG---------TTCTTGTCA---GCAGCAGGAAGCACTATGGGCGCAGCGTCAATG---ACGCTGACGGTACAGGCCAGACAATTATTGTCTGGTATAGTGCAACAGCAGAGCAATTTGCTGAGAGCTATTGAGGCGCAACAACATCTGTTGCAACTCACAGTCTGGGGCATCAAGCAGCTCCAGGCAAGA---GTCCTGGCTGTGGAAAGATACCTAAAGGATCAACAGCTCCTAGGGATTTGGGGTTGCTCTGGAAAACTCATTTGCACCACTACAGTGCCTTGGAATTATAGTTGGAGTCCT------------------------AATAAAACTATGGATGACATTTGGGGT---AACATGACCTGGATGCAATGGGAAAGAGAAATTGAC------AATTATACAGGCATAATATACAGATTAATTGAAATATCGCAAAACCAGCAAGAAAAGAATGAACAAGAATTATTGGAATTAGATAAATGGGCAAGTTTGTGGAATTGGTTTGACATAACAAAGTGGCTGTGGTATATAAAAATATTCATAATGATAATAGGAGGCTTAGTAGGTTTAAGAATAGTCTTTACTGTGCTTTCTATAGTAAATAGAGTTAGGCAGGGATACTCACCATTATCGTTTCAGACCCGC---TTCCCAGCCCCAGGGGGA------CCCGACAGGCCCGAAGGAACAGAAGAAGAAGGTGGAGAGAGAGACAGAGACAGATCCAGTCGATCAGCGGATGGATTCTTAGCAATTATCTGGGTCGATCTGAGGAGCCTGTGCCTGTTCATCTACCACAGCTTGAGAGACTTACTCTTGATTGTAGCAAGGATTGTGGGACTTCTGGGACGCAGG---------------GGGTGGGAACTCCTCAAATATTGGTGG---AATCTCCTCCAGTATTGG---------------------------------------------------AGTCAGGAACTAAAGAATAGTGCTGTTAGCTTGCTGAATGCCACAGCTATAGCAGTAGCTGAGGGGACAGATAGGGTTATAGACATAGTACAAAGA------------------ATTTGCAGAGCTATCCTCCACATACCTAGAAGAATAAGACAGGGCTTTGAAAGGGCTTTGCTATAA

2.1056.SPD.EU575312 ATG---------GAGATCAGGAAGAATTATCAGCACTTG---------TGGAGATGGGGG------------------------ATCATGCTCCTTTGGTTATTAATGAGC------------TGTAGTGCTGAA---------GAAGAAGCGTGGGTCACAGTTTATTATGGGGTACCTGTGTGGAAAGAAGCAGTCACCACTCTATTTTGTGCATCAGACGCAAAAGCATATGATACAGAGGTACATAAT---GTTTGGACCACACATGCCTGTGTACCCACAGACCCCGACCCACAAGAAGTACACATG---GAAAATGTGACAGAAGATTTTAACATGTGGAAAAATAACATGGCAGATCAGATGCATGAGGATATAATCAGTTTATGGGATCAAAGTCTAAAGCCATGTGTAAAATTAACCCCACTCTGTGTTACTTTAAATTGTGCTGATTGGAAGAATAATACTGATACCAATACC---------------------------------------------------------------------------------------AATAGTAGTGTGAGAATAATGGAGAAAGGAGAAATAAAAAACTGCTCTTTCAATATCACC---ACAAACATAAGAGAT------AAGTATCAGAAAGCATATGCACTTTTTTATAAACTTGATGTAGTACCAATAGATGATGAT------------------------------AATGCAACA------GGTAATAATGATACTAGAAACTATAGGTTGATAAGTTGTAACACCTCAGTCATTACACAGGCCTGTCCAAAGGTATCCTTTGAACCAATTCCCATACATTATTGTGCCCCGGCTGGTTTTGCGATTTTAAGGTGT---AATAATAAGACATTCAGTGGAAAAGGACAATGTACAAATGTCAGCACAGTACAATGTACACATGGAATTAAGCCAGTAGTATCAACTCAACTGCTATTAAATGGCAGTCTAGCAGAAGAA---GAGGTAATAATTAGATCTGACAATTTCTCGGACAATGCTAAAACCATAATAGTACATCTAAACAGCTCTGTAGACATTAATTGTACAAGACCAGGCAACAATACAAGAAAAAGTATAACTATAGGA------------CCAGGGAGG---GCATTTTATGCAACAGGAGACATAATAGGAGATATAAGACAAGCACATTGTAACATT------AGTGGAGAAAAATGGAATAACACTTTAAAACAGGTAGTTAAAAAATTAAGA---GAACAATTTGGG------------AATAAAACA---ATAGTCTTTAATCAA---------TCCTCAGGAGGGGACCCAGAAATTACAATGCACACTTTTAATTGTGGAGGGGAATTCTTCTACTGTAATACAGCACAACTGTTTAATAGTACTTGGGAA---------------------------AATGAT---------------------AATGAAAGGGTAGGTCAC------------AGTAACAAGACT---------------------------ATCATACTACAATGCAGAATAAAACAAATTATAAACATGTGGCAGGAAGTAGGAAAAGCAATGTATGCCCCTCCCATCAGCGGACAGATTAGATGTTCATCAAATATTACGGGGCTGCTATTAACAAGAGATGGTGGTAACGGT------------AACGAGACC---------------------AACCGGACC---GAGGTCTTCAGACCTGGAGGAGGAAATATGAAAGATAACTGGAGA---AGTGAATTATATAAATATAAAGTAGTAAAAATTGAACCA---TTAGGAGTAGCACCC---ACCAGGGCAAAGAGAAGAGTGGTGCAAAGAGAA---AAAAGAGCAGTG---GGA---ATGGGA---GCTTTG---TTCATT---GGG---------TTCTTGTCA---GCAGCAGGAAGCACTATGGGCGCAGCGTCAATG---ACGCTGACGGTACAGGCCAGACAATTATTGTCTGGTATAGTGCAACAGCAGAGCAATTTGCTGAGAGCTATTGAGGCGCAACAACATCTGTTGCAACTCACAGTCTGGGGCATCAAGCAGCTCCAGGCAAGA---GTCCTGGCTGTGGAAAGATACCTAAAGGATCAACAGCTCCTAGGGATTTGGGGTTGCTCTGGAAAACTCATTTGCACCACTACAGTGCCTTGGAATTATAGTTGGAGTCCT------------------------AATAAAACTATGGATGACATTTGGGGT---AACATGACCTGGATGCAATGGGAAAGAGAAATTGAC------AATTATACAGGCATAATATACAGATTAATTGAAATATCGCAAAACCAGCAAGAAAAGAATGAACAAGAATTATTGGAATTAGATAAATGGGCAAGTTTGTGGAATTGGTTTGACATAACAAAGTGGCTGTGGTATATAAAAATATTCATAATGATAATAGGAGGCTTAGTAGGTTTAAGAATAGTCTTTACTGTGCTTTCTATAGTAAATAGAGTTAGGCAGGGATACTCACCATTATCGTTTCAGACCCGC---TTCCCAGCCCCAGGGGGA------CCCGACAGGCCCGAAGGAACAGAAGAAGAAGGTGGAGAGAGAGACAGAGACAGATCCAGTCGATCAGCGGATGGATTCTTAGCAATTATCTGGGTCGATCTGAGGAGCCTGTGCCTGTTCATCTACCACAGCTTGAGAGACTTACTCTTGATTGTAGCAAGGATTGTGGGACTTCTGGGACGCAGG---------------GGGTGGGAACTCCTCAAATATTGGTGG---AATCTCCTCCAGTATTGG---------------------------------------------------AGTCAGGAACTAAAGAATAGTGCTGTTAGCTTGCTGAATGCCACAGCTATAGCAGTAGCTGAGGGGACAGATAGGGTTATAGACATAGTACAAAGA------------------ATTTGCAGAGCTATCCTCCACATACCTAGAAGAATAAGACAGGGCTTTGAAAGGGCTTTGCTATAA

2.1056.SPD.EU575290 ATG---------GAGATCAGGAAGAATTATCAGCACTTG---------TGGAGATGGGGG------------------------ATCATGCTCCTTTGGTTATTAATGAGC------------TGTAGTGCTGAA---------GAAGAAGCGTGGGTCACAGTTTATTATGGGGTACCTGTGTGGAAAGAAGCAGTCACCACTCTATTTTGTGCATCAGACGCAAAAGCATATGATACAGAGGTACATAAT---GTTTGGACCACACATGCCTGTGTACCCACAGACCCCGACCCACAAGAAGTACACATG---GAAAATGTGACAGAAGATTTTAACATGTGGAAAAATAACATGGCAGATCAGATGCATGAGGATATAATCAGTTTATGGGATCAAAGTCTAAAGCCATGTGTAAAATTAACCCCACTCTGTGTTACTTTAAATTGTGCTGATTGGAAGAATAATACTGATACCAATACC---------------------------------------------------------------------------------------AATAGTAGTGTGAGAATAATGGAGAAAGGAGAAATAAAAAACTGCTCTTTCAATATCACC---ACAAACATAAGAGAT------AAGTATCAGAAAGCATATGCACTTTTTTATAAACTTGATGTAGTACCAATAGATGATGAT------------------------------AATGCAACA------GGTAATAATGATACTAGAAACTATAGGTTGATAAGTTGTAACACCTCAGTCATTACACAGGCCTGTCCAAAGGTATCCTTTGAACCAATTCCCATACATTATTGTGCCCCGGCTGGTTTTGCGATTTTAAGGTGT---AATAATAAGACATTCAGTGGAAAAGGACAATGTACAAATGTCAGCACAGTACAATGTACACATGGAATTAAGCCAGTAGTATCAACTCAACTGCTATTAAATGGCAGTCTAGCAGAAGAA---GAGGTAATAATTAGATCTGACAATTTCTCGGACAATGCTAAAACCATAATAGTACATCTAAACAGCTCTGTAGACATTAATTGTACAAGACCAGGCAACAATACAAGAAAAAGTATAACTATAGGA------------CCAGGGAGG---GCATTTTATGCAACAGGAGACATAATAGGAGATATAAGACAAGCACATTGTAACATT------AGTGGAGAAAAATGGAATAACACTTTAAAACAGGTAGTTAAAAAATTAAGA---GAACAATTTGGG------------AATAAAACA---ATAGTCTTTAATCAA---------TCCTCAGGAGGGGACCCAGAAATTACAATGCACACTTTTAATTGTGGAGGGGAATTCTTCTACTGTAATACAGCACAACTGTTTAATAGTACTTGGGAAGCT---------AATAGTACTTGGGAAAATGAT---------------------AATGAAAGGGTAGGTCAC------------AGTAACAAGACT---------------------------ATCATACTACAATGCAGAATAAAACAAATTATAAACATGTGGCAGGAAGTAGGAAAAGCAATGTATGCCCCTCCCATCAGCGGACAGATTAGATGTTCATCAAATATTACGGGGCTGCTATTAACAAGAGATGGTGGTAACGGT------------AACGAGACC---------------------AACCGGACC---GAGGTCTTCAGACCTGGAGGAGGAAATATGAAAGATAACTGGAGA---AGTGAATTATATAAATATAAAGTAGTAAAAATTGAACCA---TTAGGAGTAGCACCC---ACCAGGGCAAAGAGAAGAGTGGTGCAAAGAGAA---AAAAGAGCAGTG---GGA---ATGGGA---GCTTTG---TTCATT---GGG---------TTCTTGTCA---GCAGCAGGAAGCACTATGGGCGCAGCGTCAATG---ACGCTGACGGTACAGGCCAGACAATTATTGTCTGGTATAGTGCAACAGCAGAGCAATTTGCTGAGAGCTATTGAGGCGCAACAACATCTGTTGCAACTCACAGTCTGGGGCATCAAGCAGCTCCAGGCAAGA---GTCCTGGCTGTGGAAAGATACCTAAAGGATCAACAGCTCCTAGGGATTTGGGGTTGCTCTGGAAAACTCATTTGCACCACTACAGTGCCTTGGAATTATAGTTGGAGTCCT------------------------AATAAAACTATGGATGACATTTGGGGT---AACATGACCTGGATGCAATGGGAAAGAGAAATTGAC------AATTATACAGGCATAATATACAGATTAATTGAAATATCGCAAAACCAGCAAGAAAAGAATGAACAAGAATTATTGGAATTAGATAAATGGGCAAGTTTGTGGAATTGGTTTGACATAACAAAGTGGCTGTGGTATATAAAAATATTCATAATGATAATAGGAGGCTTAGTAGGTTTAAGAATAGTCTTTACTGTGCTTTCTATAGTAAATAGAGTTAGGCAGGGATACTCACCATTATCGTTTCAGACCCGC---TTCCCAGCCCCAGGGGGA------CCCGACAGGCCCGAAGGAACAGAAGAAGAAGGTGGAGAGAGAGACAGAGACAGATCCAGTCGATCAGCGGATGGATTCTTAGCAATTATCTGGGTCGATCTGAGGAGCCTGTGCCTGTTCATCTACCACAGCTTGAGAGACTTACTCTTGATTGTAGCAAGGATTGTGGGACTTCTGGGACGCAGG---------------GGGTGGGAACTCCTCAAATATTGGTGG---AATCTCCTCCAGTATTGG---------------------------------------------------AGTCAGGAACTAAAGAATAGTGCTGTTAGCTTGCTGAATGCCACAGCTATAGCAGTAGCTGAGGGGACAGATAGGGTTATAGACATAGTACAAAGA------------------ATTTGCAGAGCTATCCTCCACATACCTAGAAGAATAAGACAGGGCTTTGAAAGGGCTTTGCTATAA

2.1056.SPD.EU575320 ATG---------GAGATCAGGAAGAATTATCAGCACTTG---------TGGAGATGGGGG------------------------ATCATGCTCCTTTGGTTATTAATGAGC------------TGTAGTGCTGAA---------GAAGAAGCGTGGGTCACAGTTTATTATGGGGTACCTGTGTGGAAAGAAGCAGTCACCACTCTATTTTGTGCATCAGACGCAAAAGCATATGATACAGAGGTACATAAT---GTTTGGACCACACATGCCTGTGTACCCACAGACCCCGACCCACAAGAAGTACACATG---GAAAATGTGACAGAAGATTTTAACATGTGGAAAAATAACATGGCAGATCAGATGCATGAGGATATAATCAGTTTATGGGATCAAAGTCTAAAGCCATGTGTAAAATTAACCCCACTCTGTGTTACTTTAAATTGTGCTGATTGGAAGAATAATACTGATACCAATACC---------------------------------------------------------------------------------------AATAGTAGTGTGAGAATAATGGAGAAAGGAGAAATAAAAAACTGCTCTTTCAATATCACC---ACAAACATAAGAGAT------AAGTATCAGAAAGCATATGCACTTTTTTATAAACTTGATGTAGTACCAATAGATGATGAT------------------------------AATGCAACA------GGTAATAATGATACTAGAAACTATAGGTTGATAAGTTGTAACACCTCAGTCATTACACAGGCCTGTCCAAAGGTATCCTTTGAACCAATTCCCATACATTATTGTGCCCCGGCTGGTTTTGCGATTTTAAGGTGT---AATAATAAGACATTCAGTGGAAAAGGACAATGTACAAATGTCAGCACAGTACAATGTACACATGGAATTAAGCCAGTAGTATCAACTCAACTGCTATTAAATGGCAGTCTAGCAGAAGAA---GAGGTAATAATTAGATCTGACAATTTCTCGGACAATGCTAAAACCATAATAGTACATCTAAACAGCTCTGTAGACATTAATTGTACAAGACCAGGCAACAATACAAGAAAAAGTATAACTATAGGA------------CCAGGGAGG---GCATTTTATGCAACAGGAGACATAATAGGAGATATAAGACAAGCACATTGTAACATT------AGTGGAGAAAAATGGAATAACACTTTAAAACAGGTAGTTAAAAAATTAAGA---GAACAATTTGGG------------AATAAAACA---ATAGTCTTTAATCAA---------TCCTCAGGAGGGGACCCAGAAATTACAATGCACACTTTTAATTGTGGAGGGGAATTCTTCTACTGTAATACAGCACAACTGTTTAATAGTACTTGGGAAGCT---------AATAGTACTTGGGAAAATGAT---------------------AATGAAAGGGTAGGTCAC------------AGTAACAAGACT---------------------------ATCATACTACAATGCAGAATAAAACAAATTATAAACATGTGGCAGGAAGTAGGAAAAGCAATGTATGCCCCTCCCATCAGCGGACAGATTAGATGTTCATCAAATATTACGGGGCTGCTATTAACAAGAGATGGTGGTAACGGT------------AACGAGACC---------------------AACCGGACC---GAGGTCTTCAGACCTGGAGGAGGAAATATGAAAGATAACTGGAGA---AGTGAATTATATAAATATAAAGTAGTAAAAATTGAACCA---TTAGGAGTAGCACCC---ACCAGGGCAAAGAGAAGAGTGGTGCAAAGAGAA---AAAAGAGCAGTG---GGA---ATGGGA---GCTTTG---TTCATT---GGG---------TTCTTGTCA---GCAGCAGGAAGCACTATGGGCGCAGCGTCAATG---ACGCTGACGGTACAGGCCAGACAATTATTGTCTGGTATAGTGCAACAGCAGAGCAATTTGCTGAGAGCTATTGAGGCGCAACAACATCTGTTGCAACTCACAGTCTGGGGCATCAAGCAGCTCCAGGCAAGA---GTCCTGGCTGTGGAAAGATACCTAAAGGATCAACAGCTCCTAGGGATTTGGGGTTGCTCTGGAAAACTCATTTGCACCACTACAGTGCCTTGGAATTATAGTTGGAGTCCT------------------------AATAAAACTATGGATGACATTTGGGGT---AACATGACCTGGATGCAATGGGAAAGAGAAATTGAC------AATTATACAGGCATAATATACAGATTAATTGAAATATCGCAAAACCAGCAAGAAAAGAATGAACAAGAATTATTGGAATTAGATAAATGGGCAAGTTTGTGGAATTGGTTTGACATAACAAAGTGGCTGTGGTATATAAAAATATTCATAATGATAATAGGAGGCTTAGTAGGTTTAAGAATAGTCTTTACTGTGCTTTCTATAGTAAATAGAGTTAGGCAGGGATACTCACCATTATCGTTTCAGACCCGC---TTCCCAGCCCCAGGGGGA------CCCGACAGGCCCGAAGGAACAGAAGAAGAAGGTGGAGAGAGAGACAGAGACAGATCCAGTCGATCAGCGGATGGATTCTTAGCAATTATCTGGGTCGATCTGAGGAGCCTGTGCCTGTTCATCTACCACAGCTTGAGAGACTTACTCTTGATTGTAGCAAGGATTGTGGGACTTCTGGGACGCAGG---------------GGGTGGGAACTCCTCAAATATTGGTGG---AATCTCCTCCAGTATTGG---------------------------------------------------AGTCAGGAACTAAAGAATAGTGCTGTTAGCTTGCTGAATGCCACAGCTATAGCAGTAGCTGAGGGGACAGATAGGGTTATAGACATAGTACAAAGA------------------ATTTGCAGAGCTATCCTCCACATACCTAGAAGAATAAGACAGGGCTTTGAAAGGGCTTTGCTATAA

2.1056.SPD.EU575309 ATG---------GAGATCAGGAAGAATTATCAGCACTTG---------TGGAGATGGGGG------------------------ATCATGCTCCTTTGGTTATTAATGAGC------------TGTAGTGCTGAA---------GAAGAAGCGTGGGTCACAGTTTATTATGGGGTACCTGTGTGGAAAGAAGCAGTCACCACTCTATTTTGTGCATCAGACGCAAAAGCATATGATACAGAGGTACATAAT---GTTTGGACCACACATGCCTGTGTACCCACAGACCCCGACCCACAAGAAGTACACATG---GAAAATGTGACAGAAGATTTTAACATGTGGAAAAATAACATGGCAGATCAGATGCATGAGGATATAATCAGTTTATGGGATCAAAGTCTAAAGCCATGTGTAAAATTAACCCCACTCTGTGTTACTTTAAATTGTGCTGATTGGAAGAATAATACTGATACCAATACC---------------------------------------------------------------------------------------AATAGTAGTGTGAGAATAATGGAGAAAGGAGAAATAAAAAACTGCTCTTTCAATATCACC---ACAAACATAAGAGAT------AAGTATCAGAAAGCATATGCACTTTTTTATAAACTTGATGTAGTACCAATAGATGATGAT------------------------------AATGCAACA------GGTAATAATGATACTAGAAACTATAGGTTGATAAGTTGTAACACCTCAGTCATTACACAGGCCTGTCCAAAGGTATCCTTTGAACCAATTCCCATACATTATTGTGCCCCGGCTGGTTTTGCGATTTTAAGGTGT---AATAATAAGACATTCAGTGGAAAAGGACAATGTACAAATGTCAGCACAGTACAATGTACACATGGAATTAAGCCAGTAGTATCAACTCAACTGCTATTAAATGGCAGTCTAGCAGAAGAA---GAGGTAATAATTAGATCTGACAATTTCTCGGACAATGCTAAAACCATAATAGTACATCTAAACAGCTCTGTAGACATTAATTGTACAAGACCAGGCAACAATACAAGAAAAAGTATAACTATAGGA------------CCAGGGAGG---GCATTTTATGCAACAGGAGACATAATAGGAGATATAAGACAAGCACATTGTAACATT------AGTGGAGAAAAATGGAATAACACTTTAAAACAGGTAGTTAAAAAATTAAGA---GAACAATTTGGG------------AATAAAACA---ATAGTCTTTAATCAA---------TCCTCAGGAGGGGACCCAGAAATTACAATGCACACTTTTAATTGTGGAGGGGAATTCTTCTACTGTAATACAGCACAACTGTTTAATAGTACTTGGGAAGCT---------AATAGTACTTGGGAAAATGAT---------------------AATGAAAGGGTAGGTCAC------------AGTAACAAGACT---------------------------ATCATACTACAATGCAGAATAAAACAAATTATAAACATGTGGCAGGAAGTAGGAAAAGCAATGTATGCCCCTCCCATCAGCGGACAGATTAGATGTTCATCAAATATTACGGGGCTGCTATTAACAAGAGATGGTGGTAACGGT------------AACGAGACC---------------------AACCGGACC---GAGGTCTTCAGACCTGGAGGAGGAAATATGAAAGATAACTGGAGA---AGTGAATTATATAAATATAAAGTAGTAAAAATTGAACCA---TTAGGAGTAGCACCC---ACCAGGGCAAAGAGAAGAGTGGTGCAAAGAGAA---AAAAGAGCAGTG---GGA---ATGAGA---GCTTTG---TTCATT---GGG---------TTCTTGTCA---GCAGCAGGAAGCACTATGGGCGCAGCGTCAATG---ACGCTGACGGTACAGGCCAGACAATTATTGTCTGGTATAGTGCAACAGCAGAGCAATTTGCTGAGAGCTATTGAGGCGCAACAACATCTGTTGCAACTCACAGTCTGGGGCATCAAGCAGCTCCAGGCAAGA---GTCCTGGCTGTGGAAAGATACCTAAAGGATCAACAGCTCCTAGGGATTTGGGGTTGCTCTGGAAAACTCATTTGCACCACTACAGTGCCTTGGAATTATAGTTGGAGTCCT------------------------AATAAAACTATGGATGACATTTGGGGT---AACATGACCTGGATGCAATGGGAAAGAGAAATTGAC------AATTATACAGGCATAATATACAGATTAATTGAAATATCGCAAAACCAGCAAGAAAAGAATGAACAAGAATTATTGGAATTAGATAAATGGGCAAGTTTGTGGAATTGGTTTGACATAACAAAGTGGCTGTGGTATATAAAAATATTCATAATGATAATAGGAGGCTTAGTAGGTTTAAGAATAGTCTTTACTGTGCTTTCTATAGTAAATAGAGTTAGGCAGGGATACTCACCATTATCGTTTCAGACCCGC---TTCCCAGCCCCAGGGGGA------CCCGACAGGCCCGAAGGAACAGAAGAAGAAGGTGGAGAGAGAGACAGAGACAGATCCAGTCGATCAGCGGATGGATTCTTAGCAATTATCTGGGTCGATCTGAGGAGCCTGTGCCTGTTCATCTACCACAGCTTGAGAGACTTACTCTTGATTGTAGCAAGGATTGTGGGACTTCTGGGACGCAGG---------------GGGTGGGAACTCCTCAAATATTGGTGG---AATCTCCTCCAGTATTGG---------------------------------------------------AGTCAGGAACTAAAGAATAGTGCTGTTAGCTTGCTGAATGCCACAGCTATAGCAGTAGCTGAGGGGACAGATAGGGTTATAGACATAGTACAAAGA------------------ATTTGCAGAGCTATCCTCCACATACCTAGAAGAATAAGACAGGGCTTTGAAAGGGCTTTGCTATAA

2.1056.SPD.EU575318 ATG---------GAGATCAGGAAGAATTATCAGCACTTG---------TGGAGATGGGGG------------------------ATCATGCTCCTTTGGTTATTAATGAGC------------TGTAGTGCTGAA---------GAAGAAGCGTGGGTCACAGTTTATTATGGGGTACCTGTGTGGAAAGAAGCAGTCACCACTCTATTTTGTGCATCAGACGCAAAAGCATATGATACAGAGGTACATAAT---GTTTGGACCACACATGCCTGTGTACCCACAGACCCCGACCCACAAGAAGTACACATG---GAAAATGTGACAGAAGATTTTAACATGTGGAAAAATAACATGGCAGATCAGATGCATGAGGATATAATCAGTTTATGGGATCAAAGTCTAAAGCCATGTGTAAAATTAACCCCACTCTGTGTTACTTTAAATTGTGCTGATTGGAAGAATAATACTGATACCAATACC---------------------------------------------------------------------------------------AATAGTAGTGTGAGAATAATGGAGAAAGGAGAAATAAAAAACTGCTCTTTCAATATCACC---ACAAACATAAGAGAT------AAGTATCAGAAAGCATATGCACTTTTTTATAAACTTGATGTAGTACCAATAGATGATGAT------------------------------AATGCAACA------GGTAATAATGATACTAGAAACTATAGGTTGATAAGTTGTAACACCTCAGTCATTACACAGGCCTGTCCAAAGGTATCCTTTGAACCAATTCCCATACATTATTGTGCCCCGGCTGGTTTTGCGATTTTAAGGTGT---AATAATAAGACATTCAGTGGAAAAGGACAATGTACAAATGTCAGCACAGTACAATGTACACATGGAATTAAGCCAGTAGTATCAACTCAACTGCTATTAAATGGCAGTCTAGCAGAAGAA---GAGGTAATAATTAGATCTGACAATTTCTCGGACAATGCTAAAACCATAATAGTACATCTAAACAGCTCTGTAGACATTAATTGTACAAGACCAGGCAACAATACAAGAAAAAGTATAACTATAGGA------------CCAGGGAGG---GCATTTTATGCAACAGGAGACATAATAGGAGATATAAGACAAGCACATTGTAACATT------AGTGGAGAAAAATGGAATAACACTTTAAAACAGGTAGTTAAAAAATTAAGA---GAACAATTTGGG------------AATAAAACA---ATAGTCTTTAATCAA---------TCCTCAGGAGGGGACCCAGAAATTACAATGCACACTTTTAATTGTGGAGGGGAATTCTTCTACTGTAATACAGCACAACTGTTTAATAGTACTTGGGAAGCT---------AATAGTACTTGGGAAAATGAT---------------------AATGAAAGGGTAGGTCAC------------AGTAACAAGACT---------------------------ATCATACTACAATGCAGAATAAAACAAATTATAAACATGTGGCAGGAAGTAGGAAAAGCAATGTATGCCCCTCCCATCAGCGGACAGATTAGATGTTCATCAAATATTACGGGGCTGCTATTAACAAGAGATGGTGGTAACGGT------------AACGAGACC---------------------AACCGGACC---GAGGTCTTCAGACCTGGAGGAGGAAATATGAAAGATAACTGGAGA---AGTGAATTATATAAATATAAAGTAGTAAAAATTGAACCA---TTAGGAGTAGCACCC---ACCAGGGCAAAGAGAAGAGTGGTGCAAAGAGAA---AAAAGAGCAGTG---GGA---ATGGGA---GCTTTG---TTCATT---GGG---------TTCTTGTCA---GCAGCAGGAAGCACTATGGGCGCAGCGTCAATG---ACGCTGACGGTACAGGCCAGACAATTATTGTCTGGTATAGTGCAACAGCAGAGCAATTTGCTGAGAGCTATTGAGGCGCAACAACATCTGTTGCAACTCACAGTCTGGGGCATCAAGCAGCTCCAGGCAAGA---GTCCTGGCTGTGGAAAGATACCTAAAGGATCAACAGCTCCTAGGGATTTGGGGTTGCTCTGGAAAACTCATTTGCACCACTACAGTGCCTTGGAATTATAGTTGGAGTCCT------------------------AATAAAACTATGGATGACATTTGGGGT---AACATGACCTGGATGCAATGGGAAAGAGAAATTGAC------AATTATACAGGCATAATATACAGATTAATTGAAATATCGCAAAACCAGCAAGAAAAGAATGAACAAGAATTATTGGAATTAGATAAATGGGCAAGTTTGTGGAATTGGTTTGACATAACAAAGTGGCTGTGGTATATAAAAATATTCATAATGATAATAGGAGGCTTAGTAGGTTTAAGAATAGTCTTTACTGTGCTTTCTATAGTAAATAGAGTTAGGCAGGGATACTCACCATTATCGTTTCAGACCCGC---TTCCCAGCCCCAGGGGGA------CCCGACAGGCCCGAAGGAACAGAAGAAGAAGGTGGAGAGAGAGACAGAGACAGATCCAGTCGATCAGCGGATGGATTCTTAGCAATTATCTGGGTCGATCTGAGGAGCCTGTGCCTGTTCATCTACCACAGCTTGAGAGACTTACTCTTGATTGTAGCAAGGATTGTGGGACTTCTGGGACGCAGG---------------GGGTGGGAACTCCTCAAATATTGGTGG---AATCTCCTCCAGTATTGG---------------------------------------------------AGTCAGGAACTAAAGAATAGTGCTGTTAGCTTGCTGAATGCCACAGCTATAGCAGTAGCTGAGGGGACAGATAGGGTTATAGACATAGTACAAAGA------------------ATTTGCAGAGCTATCCTCCACATACCTAGAAGAATAAGACAGGGCTTTGAAAGGGCTTTGCTATAA

2.1056.SPD.EU575287 ATG---------GAGATCAGGAAGAATTATCAGCACTTG---------TGGAGATGGGGG------------------------ATCATGCTCCTTTGGTTATTAATGAGC------------TGTAGTGCTGAA---------GAAGAAGCGTGGGTCACAGTTTATTATGGGGTACCTGTGTGGAAAGAAGCAGTCACCACTCTATTTTGTGCATCAGACGCAAAAGCATATGATACAGAGGTACATAAT---GTTTGGACCACACATGCCTGTGTACCCACAGACCCCGACCCACAAGAAGTACACATG---GAAAATGTGACAGAAGATTTTAACATGTGGAAAAATAACATGGCAGATCAGATGCATGAGGATATAATCAGTTTATGGGATCAAAGTCTAAAGCCATGTGTAAAATTAACCCCACTCTGTGTTACTTTAAATTGTGCTGATTGGAAGAATAATACTGATACCAATACC---------------------------------------------------------------------------------------AATAGTAGTGTGAGAATAATGGAGAAAGGAGAAATAAAAAACTGCTCTTTCAATATCACC---ACAAACATAAGAGAT------AAGTATCAGAAAGCATATGCACTTTTTTATAAACTTGATGTAGTACCAATAGATGATGAT------------------------------AATGCAACA------GGTAATAATGATACTAGAAACTATAGGTTGATAAGTTGTAACACCTCAGTCATTACACAGGCCTGTCCAAAGGTATCCTTTGAACCAATTCCCATACATTATTGTGCCCCGGCTGGTTTTGCGATTTTAAGGTGT---AATAATAAGACATTCAGTGGAAAAGGACAATGTACAAATGTCAGCACAGTACAATGTACACATGGAATTAAGCCAGTAGTATCAACTCAACTGCTATTAAATGGCAGTCTAGCAGAAGAA---GAGGTAATAATTAGATCTGACAATTTCTCGGACAATGCTAAAACCATAATAGTACATCTAAACAGCTCTGTAGACATTAATTGTACAAGACCAGGCAACAATACAAGAAAAAGTATAACTATAGGA------------CCAGGGAGG---GCATTTTATGCAACAGGAGACATAATAGGAGATATAAGACAAGCACATTGTAACATT------AGTGGAGAAAAATGGAATAACACTTTAAAACAGGTAGTTAAAAAATTAAGA---GAACAATTTGGG------------AATAAAACA---ATAGTCTTTAATCAA---------TCCTCAGGAGGGGACCCAGAAATTACAATGCACACTTTTAATTGTGGAGGGGAATTCTTCTACTGTAATACAGCACAACTGTTTAATAGTACTTGGGAAGCT---------AATAGTACTTGGGAAAATGAT---------------------AATGAAAGGGTAGGTCAC------------AGTAACAAGACT---------------------------ATCATACTACAATGCAGAATAAAACAAATTATAAACATGTGGCAGGAAGTAGGAAAAGCAATGTATGCCCCTCCCATCAGCGGACAGATTAGATGTTCATCAAATATTACGGGGCTGCTATTAACAAGAGATGGTGGTAACGGT------------AACGAGACC---------------------AACCGGACC---GAGGTCTTCAGACCTGGAGGAGGAAATATGAAAGATAACTGGAGA---AGTGAATTATATAAATATAAAGTAGTAAAAATTGAACCA---TTAGGAGTAGCACCC---ACCAGGGCAAAGAGAAGAGTGGTGCAAAGAGAA---AAAAGAGCAGTG---GGA---ATGGGA---GCTTTG---TTCATT---GGG---------TTCTTGTCA---GCAGCAGGAAGCACTATGGGCGCAGCGTCAATG---ACGCTGACGGTACAGGCCAGACAATTATTGTCTGGTATAGTGCAACAGCAGAGCAATTTGCTGAGAGCTATTGAGGCGCAACAACATCTGTTGCAACTCACAGTCTGGGGCATCAAGCAGCTCCAGGCAAGA---GTCCTGGCTGTGGAAAGATACCTAAAGGATCAACAGCTCCTAGGGATTTGGGGTTGCTCTGGAAAACTCATTTGCACCACTACAGTGCCTTGGAATTATAGTTGGAGTCCT------------------------AATAAAACTATGGATGACATTTGGGGT---AACATGACCTGGATGCAATGGGAAAGAGAAATTGAC------AATTATACAGGCATAATATACAGATTAATTGAAATATCGCAAAACCAGCAAGAAAAGAATGAACAAGAATTATTGGAATTAGATAAATGGGCAAGTTTGTGGAATTGGTTTGACATAACAAAGTGGCTGTGGTATATAAAAATATTCATAATGATAATAGGAGGCTTAGTAGGTTTAAGAATAGTCTTTACTGTGCTTTCTATAGTAAATAGAGTTAGGCAGGGATACTCACCATTATCGTTTCAGACCCGC---TTCCCAGCCCCAGGGGGA------CCCGACAGGCCCGAAGGAACAGAAGAAGAAGGTGGAGAGAGAGACAGAGACAGATCCAGTCGATCAGCGGATGGATTCTTAGCAATTATCTGGGTCGATCTGAGGAGCCTGTGCCTGTTCATCTACCACAGCTTGAGAGACTTACTCTTGATTGTAGCAAGGATTGTGGGACTTCTGGGACGCAGG---------------GGGTGGGAACTCCTCAAATATTGGTGG---AATCTCCTCCAGTATTGG---------------------------------------------------AGTCAGAAACTAAAGAATAGTGCTGTTAGCTTGCTGAATGCCACAGCTATAGCAGTAGCTGAGGGGACAGATAGGGTTATAGACATAGTACAAAGA------------------ATTTGCAGAGCTATCCTCCACATACCTAGAAGAATAAGACAGGGCTTTGAAAGGGCTTTGCTATAA

2.1056.SPD.EU575321 ATG---------GAGATCAGGAAGAATTATCAGCACTTG---------TGGAGATGGGGG------------------------ATCATGCTCCTTTGGTTATTAATGAGC------------TGTAGTGCTGAA---------GAAGAAGCGTGGGTCACAGTTTATTATGGGGTACCTGTGTGGAAAGAAGCAGTCACCACTCTATTTTGTGCATCAGACGCAAAAGCATATGATACAGAGGTACATAAT---GTTTGGACCACACATGCCTGTGTACCCACAGACCCCGACCCACAAGAAGTACACATG---GAAAATGTGACAGAAGATTTTAACATGTGGAAAAATAACATGGCAGATCAGATGCATGAGGATATAATCAGTTTATGGGATCAAAGTCTAAAGCCATGTGTAAAATTAACCCCACTCTGTGTTACTTTAAATTGTGCTGATTGGAAGAATAATACTGATACCAATACC---------------------------------------------------------------------------------------AATAGTAGTGTGAGAATAATGGAGAAAGGAGAAATAAAAAACTGCTCTTTCAATATCACC---ACAAACATAAGAGAT------AAGTATCAGAAAGCATATGCACTTTTTTATAAACTTGATGTAGTACCAATAGATGATGAT------------------------------AATGCAACA------GGTAATAATGATACTAGAAACTATAGGTTGATAAGTTGTAACACCTCAGTCATTACACAGGCCTGTCCAAAGGTATCCTTTGAACCAATTCCCATACATTATTGTGCCCCGGCTGGTTTTGCGATTTTAAGGTGT---AATAATAAGACATTCAGTGGAAAAGGACAATGTACAAATGTCAGCACAGTACAATGTACACATGGAATTAAGCCAGTAGTATCAACTCAACTGCTATTAAATGGCAGTCTAGCAGAAGAA---GAGGTAATAATTAGATCTGACAATTTCTCGGACAATGCTAAAACCATAATAGTACATCTAAACAGCTCTGTAGACATTAATTGTACAAGACCAGGCAACAATACAAGAAAAAGTATAACTATAGGA------------CCAGGGAGG---GCATTTTATGCAACAGGAGACATAATAGGAGATATAAGACAAGCACATTGTAACATT------AGTGGAGAAAAATGGAATAACACTTTAAAACAGGTAGTTAAAAAATTAAGA---GAACAATTTGGG------------AATAAAACA---ATAGTCTTTAATCAA---------TCCTCAGGAGGGGACCCAGAAATTACAATGCACACTTTTAATTGTGGAGGGGAATTCTTCTACTGTAATACAGCACAACTGTTTAATAGTACTTGGGAAGCT---------AATAGTACTTGGGAAAATGAT---------------------AATGAAAGGGTAGGTCAC------------AGTAACAAGACT---------------------------ATCATACTACAATGCAGAATAAAACAAATTATAAACATGTGGCAGGAAGTAGGAAAAGCAATGTATGCCCCTCCCATCAGCGGACAGATTAGATGTTCATCAAATATTACGGGGCTGCTATTAACAAGAGATGGTGGTAACGGT------------AACGAGACC---------------------AACCGGACC---GAGGTCTTCAGACCTGGAGGAGGAAATATGAAAGATAACTGGAGA---AGTGAATTATATAAATATAAAGTAGTAAAAATTGAACCA---TTAGGAGTAGCACCC---ACCAGGGCAAAGAGAAGAGTGGTGCAAAGAGAA---AAAAGAGCAGTG---GGA---ATGGGA---GCTTTG---TTCATT---GGG---------TTCTTGTCA---GCAGCAGGAAGCACTATGGGCGCAGCGTCAATG---ACGCTGACGGTACAGGCCAGACAATTATTGTCTGGTATAGTGCAACAGCAGAGCAATTTGCTGAGAGCTATTGAGGCGCAACAACATCTGTTGCAACTCACAGTCTGGGGCATCAAGCAGCTCCAGGCAAGA---GTCCTGGCTGTGGAAAGATACCTAAAGGATCAACAGCTCCTAGGGATTTGGGGTTGCTCTGGAAAACTCATTTGCACCACTACAGTGCCTTGGAATTATAGTTGGAGTCCT------------------------AATAAAACTATGGATGACATTTGGGGT---AACATGACCTGGATGCAATGGGAAAGAGAAATTGAC------AATTATACAGGCATAATATACAGATTAATTGAAATATCGCAAAACCAGCAAGAAAAGAATGAACAAGAATTATTGGAATTAGATAAATGGGCAAGTTTGTGGAATTGGTTTGACATAACAAAGTGGCTGTGGTATATAAAAATATTCATAATGATAATAGGAGGCTTAGTAGGTTTAAGAATAGTCTTTACTGTGCTTTCTATAGTAAATAGAGTTAGGCAGGGATACTCACCATTATCGTTTCAGACCCGC---TTCCCAGCCCCAGGGGGA------CCCGACAGGCCCGAAGGAACAGAAGAAGAAGGTGGAGAGAGAGACAGAGACAGATCCAGTCGATCAGCGGATGGATTCTTAGCAATTATCTGGGTCGATCTGAGGAGCCTGTGCCTGTTCATCTACCACAGCTTGAGAGACTTACTCTTGATTGTAGCAAGGATTGTGGGACTTCTGGGACGCAGG---------------GGGTGGGAACTCCTCAAATATTGGTGG---AATCTCCTCCAGTATTGG---------------------------------------------------AGTCAGGAACTAAAGAATAGTGCTGTTAGCTTGCTGAATGCCACAGCTATAGCAGTAGCTGAGGGGACAGATAGGGTTATAGACATAGTACAAAGA------------------ATTTGCAGAGCTATCCTCCACATACCTAGAAGAATAAGACAGGGCTTTGAAAGGGCTTTGCTATAA

2.1056.SPD.EU575292 ATG---------GAGATCAGGAAGAATTATCAGCACTTG---------TGGAGATGGGGG------------------------ATCATGCTCCTTTGGTTATTAATGAGC------------TGTAGTGCTGAA---------GAAGAAGCGTGGGTCACAGTTTATTATGGGGTACCTGTGTGGAAAGAAGCAGTCACCACTCTATTTTGTGCATCAGACGCAAAAGCATATGATACAGAGGTACATAAT---GTTTGGACCACACATGCCTGTGTACCCACAGACCCCGACCCACAAGAAGTACACATG---GAAAATGTGACAGAAGATTTTAACATGTGGAAAAATAACATGGCAGATCAGATGCATGAGGATATAATCAGTTTATGGGATCAAAGTCTAAAGCCATGTGTAAAATTAACCCCACTCTGTGTTACTTTAAATTGTGCTGATTGGAAGAATAATACTGATACCAATACC---------------------------------------------------------------------------------------AATAGTAGTGTGAGAATAATGGAGAAAGGAGAAATAAAAAACTGCTCTTTCAATATCACC---ACAAACATAAGAGAT------AAGTATCAGAAAGCATATGCACTTTTTTATAAACTTGATGTAGTACCAATAGATGATGAT------------------------------AATGCAACA------GGTAATAATGATACTAGAAACTATAGGTTGATAAGTTGTAACACCTCAGTCATTACACAGGCCTGTCCAAAGGTATCCTTTGAACCAATTCCCATACATTATTGTGCCCCGGCTGGTTTTGCGATTTTAAGGTGT---AATAATAAGACATTCAGTGGAAAAGGACAATGTACAAATGTCAGCACAGTACAATGTACACATGGAATTAAGCCAGTAGTATCAACTCAACTGCTATTAAATGGCAGTCTAGCAGAAGAA---GAGGTAATAATTAGATCTGACAATTTCTCGGACAATGCTAAAACCATAATAGTACATCTAAACAGCTCTGTAGACATTAATTGTACAAGACCAGGCAACAATACAAGAAAAAGTATAACTATAGGA------------CCAGGGAGG---GCATTTTATGCAACAGGAGACATAATAGGAGATATAAGACAAGCACATTGTAACATT------AGTGGAGAAAAATGGAATAACACTTTAAAACAGGTAGTTAAAAAATTAAGA---GAACAATTTGGG------------AATAAAACA---ATAGTCTTTAATCAA---------TCCTCAGGAGGGGACCCAGAAATTACAATGCACACTTTTAATTGTGGAGGGGAATTCTTCTACTGTAATACAGCACAACTGTTTAATAGTACTTGGGAAGCT---------AATAGTACTTGGGAAAATGAT---------------------AATGAAAGGGTAGGTCAC------------AGTAACAAGACT---------------------------ATCATACTACAATGCAGAATAAAACAAATTATAAACATGTGGCAGGAAGTAGGAAAAGCAATGTATGCCCCTCCCATCAGCGGACAGATTAGATGTTCATCAAATATTACGGGGCTGCTATTAACAAGAGATGGTGGTAACGGT------------AACGAGACC---------------------AACCGGACC---GAGGTCTTCAGACCTGGAGGAGGAAATATGAAAGATAACTGGAGA---AGTGAATTATATAAATATAAAGTAGTAAAAATTGAACCA---TTAGGAGTAGCACCC---ACCAGGGCAAAGAGAAGAGTGGTGCAAAGAGAA---AAAAGAGCAGTG---GGA---ATGGGA---GCTTTG---TTCATT---GGG---------TTCTTGTCA---GCAGCAGGAAGCACTATGGGCGCAGCGTCAATG---ACGCTGACGGTACAGGCCAGACAATTATTGTCTGGTATAGTGCAACAGCAGAGCAATTTGCTGAGAGCTATTGAGGCGCAACAACATCTGTTGCAACTCACAGTCTGGGGCATCAAGCAGCTCCAGGCAAGA---GTCCTGGCTGTGGAAAGATACCTAAAGGATCAACAGCTCCTAGGGATTTGGGGTTGCTCTGGAAAACTCATTTGCACCACTACAGTGCCTTGGAATTATAGTTGGAGTCCT------------------------AATAAAACTATGGATGACATTTGGGGT---AACATGACCTGGATGCAATGGGAAAGAGAAATTGAC------AATTATACAGGCATAATATACAGATTAATTGAAATATCGCAAAACCAGCAAGAAAAGAATGAACAAGAATTATTGGAATTAGATAAATGGGCAAGTTTGTGGAATTGGTTTGACATAACAAAGTGGCTGTGGTATATAAAAATATTCATAATGATAATAGGAGGCTTAGTAGGTTTAAGAATAGTCTTTACTGTGCTTTCTATAGTAAATAGAGTTAGGCAGGGATACTCACCATTATCGTTTCAGACCCGC---TTCCCAGCCCCAGGGGGA------CCCGACAGGCCCGAAGGAACAGAAGAAGAAGGTGGAGAGAGAGACAGAGACAGATCCAGTCGATCAGCGGATGGATTCTTAGCAATTATCTGGGTCGATCTGAGGAGCCTGTGCCTGTTCATCTACCACAGCTTGAGAGACTTACTCTTGATTGTAGCAAGGATTGTGGGACTTCTGGGACGCAGG---------------GGGTGGGAACTCCTCAAATATTGGTGG---AATCTCCTCCAGTATTGG---------------------------------------------------AGTCAGGAACTAAAGAATAGTGCTGTTAGCTTGCTGAATGCCACAGCTATAGCAGTAGCTGAGGGGACAGATAGGGTTATAGACATAGTACAAAGA------------------ATTTGCAGAGCTATCCTCCACATACCTAGAAGAATAAGACAGGGCTTTGAAAGGGCTTTGCTATAA

2.1056.SPD.EU575303 ATG---------GAGATCAGGAAGAATTATCAGCACTTG---------TGGAGATGGGGG------------------------ATCATGCTCCTTTGGTTATTAATGAGC------------TGTAGTGCTGAA---------GAAGAAGCGTGGGTCACAGTTTATTATGGGGTACCTGTGTGGAAAGAAGCAGTCACCACTCTATTTTGTGCATCAGACGCAAAAGCATATGATACAGAGGTACATAAT---GTTTGGACCACACATGCCTGTGTACCCACAGACCCCGACCCACAAGAAGTACACATG---GAAAATGTGACAGAAGATTTTAACATGTGGAAAAATAACATGGCAGATCAGATGCATGAGGATATAATCAGTTTATGGGATCAAAGTCTAAAGCCATGTGTAAAATTAACCCCACTCTGTGTTACTTTAAATTGTGCTGATTGGAAGAATAATACTGATACCAATACC---------------------------------------------------------------------------------------AATAGTAGTGTGAGAATAATGGAGAAAGGAGAAATAAAAAACTGCTCTTTCAATATCACC---ACAAACATAAGAGAT------AAGTATCAGAAAGCATATGCACTTTTTTATAAACTTGATGTAGTACCAATAGATGATGAT------------------------------AATGCAACA------GGTAATAATGATACTAGAAACTATAGGTTGATAAGTTGTAACACCTCAGTCATTACACAGGCCTGTCCAAAGGTATCCTTTGAACCAATTCCCATACATTATTGTGCCCCGGCTGGTTTTGCGATTTTAAGGTGT---AATAATAAGACATTCAGTGGAAAAGGACAATGTACAAATGTCAGCACAGTACAATGTACACATGGAATTAAGCCAGTAGTATCAACTCAACTGCTATTAAATGGCAGTCTAGCAGAAGAA---GAGGTAATAATTAGATCTGACAATTTCTCGGACAATGCTAAAACCATAATAGTACATCTAAACAGCTCTGTAGACATTAATTGTACAAGACCAGGCAACAATACAAGAAAAAGTATAACTATAGGA------------CCAGGGAGG---GCATTTTATGCAACAGGAGACATAATAGGAGATATAAGACAAGCACATTGTAACATT------AGTGGAGAAAAATGGAATAACACTTTAAAACAGGTAGTTAAAAAATTAAGA---GAACAATTTGGG------------AATAAAACA---ATAGTCTTTAATCAA---------TCCTCAGGAGGGGACCCAGAAATTACAATGCACACTTTTAATTGTGGAGGGGAATTCTTCTACTGTAATACAGCACAACTGTTTAATAGTACTTGGGAAGCT---------AATAGTACTTGGGAAAATGAT---------------------AATGAAAGGGTAGGTCAC------------AGTAACAAGACT---------------------------ATCATACTACAATGCAGAATAAAACAAATTATAAACATGTGGCAGGAAGTAGGAAAAGCAATGTATGCCCCTCCCATCAGCGGACAGATTAGATGTTCATCAAATATTACGGGGCTGCTATTAACAAGAGATGGTGGTAACGGT------------AACGAGACC---------------------AACCGGACC---GAGGTCTTCAGACCTGGAGGAGGAAATATGAAAGATAACTGGAGA---AGTGAATTATATAAATATAAAGTAGTAAAAATTGAACCA---TTAGGAGTAGCACCC---ACCAGGGCAAAGAGAAGAGTGGTGCAAAGAGAA---AAAAGAGCAGTG---GGA---ATGGGA---GCTTTG---TTCATT---GGG---------TTCTTGTCA---GCAGCAGGAAGCACTATGGGCGCAGCGTCAATG---ACGCTGACGGTACAGGCCAGACAATTATTGTCTGGTATAGTGCAACAGCAGAGCAATTTGCTGAGAGCTATTGAGGCGCAACAACATCTGTTGCAACTCACAGTCTGGGGCATCAAGCAGCTCCAGGCAAGA---GTCCTGGCTGTGGAAAGATACCTAAAGGATCAACAGCTCCTAGGGATTTGGGGTTGCTCTGGAAAACTCATTTGCACCACTACAGTGCCTTGGAATTATAGTTGGAGTCCT------------------------AATAAAACTATGGATGACATTTGGGGT---AACATGACCTGGATGCAATGGGAAAGAGAAATTGAC------AATTATACAGGCATAATATACAGATTAATTGAAATATCGCAAAACCAGCAAGAAAAGAATGAACAAGAATTATTGGAATTAGATAAATGGGCAAGTTTGTGGAATTGGTTTGACATAACAAAGTGGCTGTGGTATATAAAAATATTCATAATGATAATAGGAGGCTTAGTAGGTTTAAGAATAGTCTTTACTGTGCTTTCTATAGTAAATAGAGTTAGGCAGGGATACTCACCATTATCGTTTCAGACCCGC---TTCCCAGCCCCAGGGGGA------CCCGACAGGCCCGAAGGAACAGAAGAAGAAGGTGGAGAGAGAGACAGAGACAGATCCAGTCGATCAGCGGATGGATTCTTAGCAATTATCTGGGTCGATCTGAGGAGCCTGTGCCTGTTCATCTACCACAGCTTGAGAGACTTACTCTTGATTGTAGCAAGGATTGTGGGACTTCTGGGACGCAGG---------------GGGTGGGAACTCCTCAAATATTGGTGG---AATCTCCTCCAGTATTGG---------------------------------------------------AGTCAGGAACTAAAGAATAGTGCTGTTAGCTTGCTGAATGCCACAGCTATAGCAGTAGCTGAGGGGACAGATAGGGTTATAGACATAGTACAAAGA------------------ATTTGCAGAGCTATCCTCCACATACCTAGAAGAATAAGACAGGGCTTTGAAAGGGCTTTGCTATAA

2.1056.SPD.EU575293 ATG---------GAGATCAGGAAGAATTATCAGCACTTG---------TGGAGATGGGGG------------------------ATCATGCTCCTTTGGTTATTAATGAGC------------TGTAGTGCTGAA---------GAAGAAGCGTGGGTCACAGTTTATTATGGGGTACCTGTGTGGAAAGAAGCAGTCACCACTCTATTTTGTGCATCAGACGCAAAAGCATATGATACAGAGGTACATAAT---GTTTGGACCACACATGCCTGTGTACCCACAGACCCCGACCCACAAGAAGTACACATG---GAAAATGTGACAGAAGATTTTAACATGTGGAAAAATAACATGGCAGATCAGATGCATGAGGATATAATCAGTTTATGGGATCAAAGTCTAAAGCCATGTGTAAAATTAACCCCACTCTGTGTTACTTTAAATTGTGCTGATTGGAAGAATAATACTGATACCAATACC---------------------------------------------------------------------------------------AATAGTAGTGTGAGAATAATGGAGAAAGGAGAAATAAAAAACTGCTCTTTCAATATCACC---ACAAACATAAGAGAT------AAGTATCAGAAAGCATATGCACTTTTTTATAAACTTGATGTAGTACCAATAGATGATGAT------------------------------AATGCAACA------GGTAATAATGATACTAGAAACTATAGGTTGATAAGTTGTAACACCTCAGTCATTACACAGGCCTGTCCAAAGGTATCCTTTGAACCAATTCCCATACATTATTGTGCCCCGGCTGGTTTTGCGATTTTAAGGTGT---AATAATAAGACATTCAGTGGAAAAGGACAATGTACAAATGTCAGCACAGTACAATGTACACATGGAATTAAGCCAGTAGTATCAACTCAACTGCTATTAAATGGCAGTCTAGCAGAAGAA---GAGGTAATAATTAGATCTGACAATTTCTCGGACAATGCTAAAACCATAATAGTACATCTAAACAGCTCTGTAGACATTAATTGTACAAGACCAGGCAACAATACAAGAAAAAGTATAACTATAGGA------------CCAGGGAGG---GCATTTTATGCAACAGGAGACATAATAGGAGATATAAGACAAGCACATTGTAACATT------AGTGGAGAAAAATGGAATAACACTTTAAAACAGGTAGTTAAAAAATTAAGA---GAACAATTTGGG------------AATAAAACA---ATAGTCTTTAATCAA---------TCCTCAGGAGGGGACCCAGAAATTACAATGCACACTTTTAATTGTGGAGGGGAATTCTTCTACTGTAATACAGCACAACTGTTTAATAGTACTTGGGAAGCT---------AATAGTACTTGGGAAAATGAT---------------------AATGAAAGGGTAGGTCAC------------AGTAACAAGACT---------------------------ATCATACTACAATGCAGAATAAAACAAATTATAAACATGTGGCAGGAAGTAGGAAAAGCAATGTATGCCCCTCCCATCAGCGGACAGATTAGATGTTCATCAAATATTACGGGGCTGCTATTAACAAGAGATGGTGGTAACGGT------------AACGAGACC---------------------AACCGGACC---GAGGTCTTCAGACCTGGAGGAGGAAATATGAAAGATAACTGGAGA---AGTGAATTATATAAATATAAAGTAGTAAAAATTGAACCA---TTAGGAGTAGCACCC---ACCAGGGCAAAGAGAAGAGTGGTGCAAAGAGAA---AAAAGAGCAGTG---GGA---ATGGGA---GCTTTG---TTCATT---GGG---------TTCTTGTCA---GCAGCAGGAAGCACTATGGGCGCAGCGTCAATG---ACGCTGACGGTACAGGCCAGACAATTATTGTCTGGTATAGTGCAACAGCAGAGCAATTTGCTGAGAGCTATTGAGGCGCAACAACATCTGTTGCAACTCACAGTCTGGGGCATCAAGCAGCTCCAGGCAAGA---GTCCTGGCTGTGGAAAGATACCTAAAGGATCAACAGCTCCTAGGGATTTGGGGTTGCTCTGGAAAACTCATTTGCACCACTACAGTGCCTTGGAATTATAGTTGGAGTCCT------------------------AATAAAACTATGGATGACATTTGGGGT---AACATGACCTGGATGCAATGGGAAAGAGAAATTGAC------AATTATACAGGCATAATATACAGATTAATTGAAATATCGCAAAACCAGCAAGAAAAGAATGAACAAGAATTATTGGAATTAGATAAATGGGCAAGTTTGTGGAATTGGTTTGACATAACAAAGTGGCTGTGGTATATAAAAATATTCATAATGATAATAGGAGGCTTAGTAGGTTTAAGAATAGTCTTTACTGTGCTTTCTATAGTAAATAGAGTTAGGCAGGGATACTCACCATTATCGTTTCAGACCCGC---TTCCCAGCCCCAGGGGGA------CCCGACAGGCCCGAAGGAACAGAAGAAGAAGGTGGAGAGAGAGACAGAGACAGATCCAGTCGATCAGCGGATGGATTCTTAGCAATTATCTGGGTCGATCTGAGGAGCCTGTGCCTGTTCATCTACCACAGCTTGAGAGACTTACTCTTGATTGTAGCAAGGATTGTGGGACTTCTGGGACGCAGG---------------GGGTGGGAACTCCTCAAATATTGGTGG---AATCTCCTCCAGTATTGG---------------------------------------------------AGTCAGGAACTAAAGAATAGTGCTGTTAGCTTGCTGAATGCCACAGCTATAGCAGTAGCTGAGGGGACAGATAGGGTTATAGACATAGTACAAAGA------------------ATTTGCAGAGCTATCCTCCACATACCTAGAAGAATAAGACAGGGCTTTGAAAGGGCTTTGCTATAA

2.1056.SPD.EU575286 ATG---------GAGATCAGGAAGAATTATCAGCACTTG---------TGGAGATGGGGG------------------------ATCATGCTCCTTTGGTTATTAATGAGC------------TGTAGTGCTGAA---------GAAGAAGCGTGGGTCACAGTTTATTATGGGGTACCTGTGTGGAAAGAAGCAGTCACCACTCTATTTTGTGCATCAGACGCAAAAGCATATGATACAGAGGTACATAAT---GTTTGGACCACACATGCCTGTGTACCCACAGACCCCGACCCACAAGAAGTACACATG---GAAAATGTGACAGAAGATTTTAACATGTGGAAAAATAACATGGCAGATCAGATGCATGAGGATATAATCAGTTTATGGGATCAAAGTCTAAAGCCATGTGTAAAATTAACCCCACTCTGTGTTACTTTAAATTGTGCTGATTGGAAGAATAATACTGATACCAATACC---------------------------------------------------------------------------------------AATAGTAGTGTGAGAATAATGGAGAAAGGAGAAATAAAAAACTGCTCTTTCAATATCACC---ACAAACATAAGAGAT------AAGTATCAGAAAGCATATGCACTTTTTTATAAACTTGATGTAGTACCAATAGATGATGAT------------------------------AATGCAACA------GGTAATAATGATACTAGAAACTATAGGTTGATAAGTTGTAACACCTCAGTCATTACACAGGCCTGTCCAAAGGTATCCTTTGAACCAATTCCCATACATTATTGTGCCCCGGCTGGTTTTGCGATTTTAAGGTGT---AATAATAAGACATTCAGTGGAAAAGGACAATGTACAAATGTCAGCACAGTACAATGTACACATGGAATTAAGCCAGTAGTATCAACTCAACTGCTATTAAATGGCAGTCTAGCAGAAGAA---GAGGTAATAATTAGATCTGACAATTTCTCGGACAATGCTAAAACCATAATAGTACATCTAAACAGCTCTGTAGACATTAATTGTACAAGACCAGGCAACAATACAAGAAAAAGTATAACTATAGGA------------CCAGGGAGG---GCATTTTATGCAACAGGAGACATAATAGGAGATATAAGACAAGCACATTGTAACATT------AGTGGAGAAAAATGGAATAACACTTTAAAACAGGTAGTTAAAAAATTAAGA---GAACAATTTGGG------------AATAAAACA---ATAGTCTTTAATCAA---------TCCTCAGGAGGGGACCCAGAAATTACAATGCACACTTTTAATTGTGGAGGGGAATTCTTCTACTGTAATACAGCACAACTGTTTAATAGTACTTGGGAAGCT---------AATAGTACTTGGGAAAATGAT---------------------AATGAAAGGGTAGGTCAC------------AGTAACAAGACT---------------------------ATCATACTACAATGCAGAATAAAACAAATTATAAACATGTGGCAGGAAGTAGGAAAAGCAATGTATGCCCCTCCCATCAGCGGACAGATTAGATGTTCATCAAATATTACGGGGCTGCTATTAACAAGAGATGGTGGTAACGGT------------AACGAGACC---------------------AACCGGACC---GAGGTCTTCAGACCTGGAGGAGGAAATATGAAAGATAACTGGAGA---AGTGAATTATATAAATATAAAGTAGTAAAAATTGAACCA---TTAGGAGTAGCACCC---ACCAGGGCAAAGAGAAGAGTGGTGCAAAGAGAA---AAAAGAGCAGTG---GGA---ATGGGA---GCTTTG---TTCATT---GGG---------TTCTTGTCA---GCAGCAGGAAGCACTATGGGCGCAGCGTCAATG---ACGCTGACGGTACAGGCCAGACAATTATTGTCTGGTATAGTGCAACAGCAGAGCAATTTGCTGAGAGCTATTGAGGCGCAACAACATCTGTTGCAACTCACAGTCTGGGGCATCAAGCAGCTCCAGGCAAGA---GTCCTGGCTGTGGAAAGATACCTAAAGGATCAACAGCTCCTAGGGATTTGGGGTTGCTCTGGAAAACTCATTTGCACCACTACAGTGCCTTGGAATTATAGTTGGAGTCCT------------------------AATAAAACTATGGATGACATTTGGGGT---AACATGACCTGGATGCAATGGGAAAGAGAAATTGAC------AATTATACAGGCATAATATACAGATTAATTGAAATATCGCAAAACCAGCAAGAAAAGAATGAACAAGAATTATTGGAATTAGATAAATGGGCAAGTTTGTGGAATTGGTTTGACATAACAAAGTGGCTGTGGTATATAAAAATATTCATAATGATAATAGGAGGCTTAGTAGGTTTAAGAATAGTCTTTACTGTGCTTTCTATAGTAAATAGAGTTAGGCAGGGATACTCACCATTATCGTTTCAGACCCGC---TTCCCAGCCCCAGGGGGA------CCCGACAGGCCCGAAGGAACAGAAGAAGAAGGTGGAGAGAGAGACAGAGACAGATCCAGTCGATCAGCGGATGGATTCTTAGCAATTATCTGGGTCGATCTGAGGAGCCTGTGCCTGTTCATCTACCACAGCTTGAGAGACTTACTCTTGATTGTAGCAAGGATTGTGGGACTTCTGGGACGCAGG---------------GGGTGGGAACTCCTCAAATATTGGTGG---AATCTCCTCCAGTATTGG---------------------------------------------------AGTCAGGAACTAAAGAATAGTGCTGTTAGCTTGCTGAATGCCACAGCTATAGCAGTAGCTGAGGGGACAGATAGGGTTATAGACATAGTACAAAGA------------------ATTTGCAGAGCTATCCTCCACATACCTAGAAGAATAAGACAGGGCTTTGAAAGGGCTTTGCTATAA

2.1056.SPD.EU575316 ATG---------GAGATCAGGAAGAATTATCAGCACTTG---------TGGAGATGGGGG------------------------ATCATGCTCCTTTGGTTATTAATGAGC------------TGTAGTGCTGAA---------GAAGAAGCGTGGGTCACAGTTTATTATGGGGTACCTGTGTGGAAAGAAGCAGTCACCACTCTATTTTGTGCATCAGACGCAAAAGCATATGATACAGAGGTACATAAT---GTTTGGACCACACATGCCTGTGTACCCACAGACCCCGACCCACAAGAAGTACACATG---GAAAATGTGACAGAAGATTTTAACATGTGGAAAAATAACATGGCAGATCAGATGCATGAGGATATAATCAGTTTATGGGATCAAAGTCTAAAGCCATGTGTAAAATTAACCCCACTCTGTGTTACTTTAAATTGTGCTGATTGGAAGAATAATACTGATACCAATACC---------------------------------------------------------------------------------------AATAGTAGTGTGAGAATAATGGAGAAAGGAGAAATAAAAAACTGCTCTTTCAATATCACC---ACAAACATAAGAGAT------AAGTATCAGAAAGCATATGCACTTTTTTATAAACTTGATGTAGTACCAATAGATGATGAT------------------------------AATGCAACA------GGTAATAATGATACTAGAAACTATAGGTTGATAAGTTGTAACACCTCAGTCATTACACAGGCCTGTCCAAAGGTATCCTTTGAACCAATTCCCATACATTATTGTGCCCCGGCTGGTTTTGCGATTTTAAGGTGT---AATAATAAGACATTCAGTGGAAAAGGACAATGTACAAATGTCAGCACAGTACAATGTACACATGGAATTAAGCCAGTAGTATCAACTCAACTGCTATTAAATGGCAGTCTAGCAGAAGAA---GAGGTAATAATTAGATCTGACAATTTCTCGGACAATGCTAAAACCATAATAGTACATCTAAACAGCTCTGTAGACATTAATTGTACAAGACCAGGCAACAATACAAGAAAAAGTATAACTATAGGA------------CCAGGGAGG---GCATTTTATGCAACAGGAGACATAATAGGAGATATAAGACAAGCACATTGTAACATT------AGTGGAGAAAAATGGAATAACACTTTAAAACAGGTAGTTAAAAAATTAAGA---GAACAATTTGGG------------AATAAAACA---ATAGTCTTTAATCAA---------TCCTCAGGAGGGGACCCAGAAATTACAATGCACACTTTTAATTGTGGAGGGGAATTCTTCTACTGTAATACAGCACAACTGTTTAATAGTACTTGGGAAGCT---------AATAGTACTTGGGAAAATGAT---------------------AATGAAAGGGTAGGTCAC------------AGTAACAAGACT---------------------------ATCATACTACAATGCAGAATAAAACAAATTATAAACATGTGGCAGGAAGTAGGAAAAGCAATGTATGCCCCTCCCATCAGCGGACAGATTAGATGTTCATCAAATATTACGGGGCTGCTATTAACAAGAGATGGTGGTAACGGT------------AACGAGACC---------------------AACCGGACC---GAGGTCTTCAGACCTGGAGGAGGAAATATGAAAGATAACTGGAGA---AGTGAATTATATAAATATAAAGTAGTAAAAATTGAACCA---TTAGGAGTAGCACCC---ACCAGGGCAAAGAGAAGAGTGGTGCAAAGAGAA---AAAAGAGCAGTG---GGA---ATGGGA---GCTTTG---TTCATT---GGG---------TTCTTGTCA---GCAGCAGGAAGCACTATGGGCGCAGCGTCAATG---ACGCTGACGGTACAGGCCAGACAATTATTGTCTGGTATAGTGCAACAGCAGAGCAATTTGCTGAGAGCTATTGAGGCGCAACAACATCTGTTGCAACTCACAGTCTGGGGCATCAAGCAGCTCCAGGCAAGA---GTCCTGGCTGTGGAAAGATACCTAAAGGATCAACAGCTCCTAGGGATTTGGGGTTGCTCTGGAAAACTCATTTGCACCACTACAGTGCCTTGGAATTATAGTTGGAGTCCT------------------------AATAAAACTATGGATGACATTTGGGGT---AACATGACCTGGATGCAATGGGAAAGAGAAATTGAC------AATTATACAGGCATAATATACAGATTAATTGAAATATCGCAAAACCAGCAAGAAAAGAATGAACAAGAATTATTGGAATTAGATAAATGGGCAAGTTTGTGGAATTGGTTTGACATAACAAAGTGGCTGTGGTATATAAAAATATTCATAATGATAATAGGAGGCTTAGTAGGTTTAAGAATAGTCTTTACTGTGCTTTCTATAGTAAATAGAGTTAGGCAGGGATACTCACCATTATCGTTTCAGACCCGC---TTCCCAGCCCCAGGGGGA------CCCGACAGGCCCGAAGGAACAGAAGAAGAAGGTGGAGAGAGAGACAGAGACAGATCCAGTCGATCAGCGGATGGATTCTTAGCAATTATCTGGGTCGATCTGAGGAGCCTGTGCCTGTTCATCTACCACAGCTTGAGAGACTTACTCTTGATTGTAGCAAGGATTGTGGGACTTCTGGGACGCAGG---------------GGGTGGGAACTCCTCAAATATTGGTGG---AATCTCCTCCAGTATTGG---------------------------------------------------AGTCAGGAACTAAAGAATAGTGCTGTTAGCTTGCTGAATGCCACAGCTATAGCAGTAGCTGAGGGGACAGATAGGGTTATAGACATAGTACAAAGA------------------ATTTGCAGAGCTATCCTCCACATACCTAGAAGAATAAGACAGGGCTTTGAAAGGGCTTTGCTATAA

2.1056.SPD.EU575297 ATG---------GAGATCAGGAAGAATTATCAGCACTTG---------TGGAGATGGGGG------------------------ATCATGCTCCTTTGGTTATTAATGAGC------------TGTAGTGCTGAA---------GAAGAAGCGTGGGTCACAGTTTATTATGGGGTACCTGTGTGGAAAGAAGCAGTCACCACTCTATTTTGTGCATCAGACGCAAAAGCATATGATACAGAGGTACATAAT---GTTTGGACCACACATGCCTGTGTACCCACAGACCCCGACCCACAAGAAGTACACATG---GAAAATGTGACAGAAGATTTTAACATGTGGAAAAATAACATGGCAGATCAGATGCATGAGGATATAATCAGTTTATGGGATCAAAGTCTAAAGCCATGTGTAAAATTAACCCCACTCTGTGTTACTTTAAATTGTGCTGATTGGAAGAATAATACTGATACCAATACC---------------------------------------------------------------------------------------AATAGTAGTGTGAGAATAATGGAGAAAGGAGAAATAAAAAACTGCTCTTTCAATATCACC---ACAAACATAAGAGAT------AAGTATCAGAAAGCATATGCACTTTTTTATAAACTTGATGTAGTACCAATAGATGATGAT------------------------------AATGCAACA------GGTAATAATGATACTAGAAACTATAGGTTGATAAGTTGTAACACCTCAGTCATTACACAGGCCTGTCCAAAGGTATCCTTTGAACCAATTCCCATACATTATTGTGCCCCGGCTGGTTTTGCGATTTTAAGGTGT---AATAATAAGACATTCAGTGGAAAAGGACAATGTACAAATGTCAGCACAGTACAATGTACACATGGAATTAAGCCAGTAGTATCAACTCAACTGCTATTAAATGGCAGTCTAGCAGAAGAA---GAGGTAATAATTAGATCTGACAATTTCTCGGACAATGCTAAAACCATAATAGTACATCTAAACAGCTCTGTAGACATTAATTGTACAAGACCAGGCAACAATACAAGAAAAAGTATAACTATAGGA------------CCAGGGAGG---GCATTTTATGCAACAGGAGACATAATAGGAGATATAAGACAAGCACATTGTAACATT------AGTGGAGAAAAATGGAATAACACTTTAAAACAGGTAGTTAAAAAATTAAGA---GAACAATTTGGG------------AATAAAACA---ATAGTCTTTAATCAA---------TCCTCAGGAGGGGACCCAGAAATTACAATGCACACTTTTAATTGTGGAGGGGAATTCTTCTACTGTAATACAGCACAACTGTTTAATAGTACTTGGGAAGCT---------AATAGTACTTGGGAAAATGAT---------------------AATGAAAGGGTAGGTCAC------------AGTAACAAGACT---------------------------ATCATACTACAATGCAGAATAAAACAAATTATAAACATGTGGCAGGAAGTAGGAAAAGCAATGTATGCCCCTCCCATCAGCGGACAGATTAGATGTTCATCAAATATTACGGGGCTGCTATTAACAAGAGATGGTGGTAACGGT------------AACGAGACC---------------------AACCGGACC---GAGGTCTTCAGACCTGGAGGAGGAAATATGAAAGATAACTGGAGA---AGTGAATTATATAAATATAAAGTAGTAAAAATTGAACCA---TTAGGAGTAGCACCC---ACCAGGGCAAAGAGAAGAGTGGTGCAAAGAGAA---AAAAGAGCAGTG---GGA---ATGGGA---GCTTTG---TTCATT---GGG---------TTCTTGTCA---GCAGCAGGAAGCACTATGGGCGCAGCGTCAATG---ACGCTGACGGTACAGGCCAGACAATTATTGTCTGGTATAGTGCAACAGCAGAGCAATTTGCTGAGAGCTATTGAGG--CAACAACATCTGTTGCAACTCACAGTCTGGGGCATCAAGCAGCTCCAGGCAAGA---GTCCTGGCTGTGGAAAGATACCTAAAGGATCAACAGCTCCTAGGGATTTGGGGTTGCTCTGGAAAACTCATTTGCACCACTACAGTGCCTTGGAATTATAGTTGGAGTCCT------------------------AATAAAACTATGGATGACATTTGGGGT---AACATGACCTGGATGCAATGGGAAAGAGAAATTGAC------AATTATACAGGCATAATATACAGATTAATTGAAATATCGCAAAACCAGCAAGAAAAGAATGAACAAGAATTATTGGAATTAGATAAATGGGCAAGTTTGTGGAATTGGTTTGACATAACAAAGTGGCTGTGGTATATAAAAATATTCATAATGATAATAGGAGGCTTAGTAGGTTTAAGAATAGTCTTTACTGTGCTTTCTATAGTAAATAGAGTTAGGCAGGGATACTCACCATTATCGTTTCAGACCCGC---TTCCCAGCCCCAGGGGGA------CCCGACAGGCCCGAAGGAACAGAAGAAGAAGGTGGAGAGAGAGACAGAGACAGATCCAGTCGATCAGCGGATGGATTCTTAGCAATTATCTGGGTCGATCTGAGGAGCCTGTGCCTGTTCATCTACCACAGCTTGAGAGACTTACTCTTGATTGTAGCAAGGATTGTGGGACTTCTGGGACGCAGG---------------GGGTGGGAACTCCTCAAATATTGGTGG---AATCTCCTCCAGTATTGG---------------------------------------------------AGTCAGGAACTAAAGAATAGTGCTGTTAGCTTGCTGAATGCCACAGCTATAGCAGTAGCTGAGGGGACAGATAGGGTTATAGACATAGTACAAAGA------------------ATTTGCAGAGCTATCCTCCACATACCTAGAAGAATAAGACAGGGCTTTGAAAGGGCTTTGCTATAA

2.1056.SPD.EU575328 ATG---------GAGATCAGGAAGAATTATCAGCACTTG---------TGGAGATGGGGG------------------------ATCATGCTCCTTTGGTTATTAATGAGC------------TGTAGTGCTGAA---------GAAGAAGCGTGGGTCACAGTTTATTATGGGGTACCTGTGTGGAAAGAAGCAGTCACCACTCTATTTTGTGCATCAGACGCAAAAGCATATGATACAGAGGTACATAAT---GTTTGGACCACACATGCCTGTGTACCCACAGACCCCGACCCACAAGAAGTACACATG---GAAAATGTGACAGAAGATTTTAACATGTGGAAAAATAACATGGCAGATCAGATGCATGAGGATATAATCAGTTTATGGGATCAAAGTCTAAAGCCATGTGTAAAATTAACCCCACTCTGTGTTACTTTAAATTGTGCTGATTGGAAGAATAATACTGATACCAATACC---------------------------------------------------------------------------------------AATAGTAGTGTGAGAATAATGGAGAAAGGAGAAATAAAAAACTGCTCTTTCAATATCACC---ACAAACATAAGAGAT------AAGTATCAGAAAGCATATGCACTTTTTTATAAACTTGATGTAGTACCAATAGATGATGAT------------------------------AATGCAACA------GGTAATAATGATACTAGAAACTATAGGTTGATAAGTTGTAACACCTCAGTCATTACACAGGCCTGTCCAAAGGTATCCTTTGAACCAATTCCCATACATTATTGTGCCCCGGCTGGTTTTGCGATTTTAAGGTGT---AATAATAAGACATTCAGTGGAAAAGGACAATGTACAAATGTCAGCACAGTACAATGTACACATGGAATTAAGCCAGTAGTATCAACTCAACTGCTATTAAATGGCAGTCTAGCAGAAGAA---GAGGTAATAATTAGATCTGACAATTTCTCGGACAATGCTAAAACCATAATAGTACATCTAAACAGCTCTGTAGACATTAATTGTACAAGACCAGGCAACAATACAAGAAAAAGTATAACTATAGGA------------CCAGGGAGG---GCATTTTATGCAACAGGAGACATAATAGGAGATATAAGACAAGCACATTGTAACATT------AGTGGAGAAAAATGGAATAACACTTTAAAACAGGTAGTTAAAAAATTAAGA---GAACAATTTGGG------------AATAAAACA---ATAGTCTTTAATCAA---------TCCTCAGGAGGGGACCCAGAAATTACAATGCACACTTTTAATTGTGGAGGGGAATTCTTCTACTGTAATACAGCACAACTGTTTAATAGTACTTGGGAAGCT---------AATAGTACTTGGGAAAATGAT---------------------AATGAAAGGGTAGGTCAC------------AGTAACAAGACT---------------------------ATCATACTACAATGCAGAATAAAACAAATTATAAACATGTGGCAGGAAGTAGGAAAAGCAATGTATGCCCCTCCCATCAGCGGACAGATTAGATGTTCATCAAATATTACGGGGCTGCTATTAACAAGAGATGGTGGTAACGGT------------AACGAGACC---------------------AACCGGACC---GAGGTCTTCAGACCTGGAGGAGGAAATATGAAAGATAACTGGAGA---AGTGAATTATATAAATATAAAGTAGTAAAAATTGAACCA---TTAGGAGTAGCACCC---ACCAGGGCAAAGAGAAGAGTGGTGCAAAGAGAA---AAAAGAGCAGTG---GGA---ATGGGA---GCTTTG---TTCATT---GGG---------TTCTTGTCA---GCAGCAGGAAGCACTATGGGCGCAGCGTCAATG---ACGCTGACGGTACAGGCCAGACAATTATTGTCTGGTATAGTGCAACAGCAGAGCAATTTGCTGAGAGCTATTGAGGCGCAACAACATCTGTTGCAACTCACAGTCTGGGGCATCAAGCTGCTCCAGGCAAGA---GTCCTGGCTGTGGAAAGATACCTAAAGGATCAACAGCTCCTAGGGATTTGGGGTTGCTCTGGAAAACTCATTTGCACCACTACAGTGCCTTGGAATTATAGTTGGAGTCCT------------------------AATAAAACTATGGATGACATTTGGGGT---AACATGACCTGGATGCAATGGGAAAGAGAAATTGAC------AATTATACAGGCATAATATACAGATTAATTGAAATATCGCAAAACCAGCAAGAAAAGAATGAACAAGAATTATTGGAATTAGATAAATGGGCAAGTTTGTGGAATTGGTTTGACATAACAAAGTGGCTGTGGTATATAAAAATATTCATAATGATAATAGGAGGCTTAGTAGGTTTAAGAGTAGTCTTTACTGTGCTTTCTATAGTAAATAGAGTTAGGCAGGGATACTCACCATTATCGTTTCAGACCCGC---TTCCCAGCCCCAGGGGGA------CCCGACAGGCCCGAAGGAACAGAAGAAGAAGGTGGAGAGAGAGACAGAGACAGATCCAGTCGATCAGCGGATGGATTCTTAGCAATTATCTGGGTCGATCTGAGGAGCCTGTGCCTGTTCATCTACCACAGCTTGAGAGACTTACTCTTGATTGTAGCAAGGATTGTGGGACTTCTGGGACGCAGG---------------GGGTGGGAACTCCTCAAATATTGGTGG---AATCTCCTCCAGTATTGG---------------------------------------------------AGTCAGGAACTAAAGAATAGTGCTGTTAGCTTGCTGAATGCCACAGCTATAGCAGTAGCTGAGGGGACAGATAGGGTTATAGACATAGTACAAAGA------------------ATTTGCAGAGCTATCCTCCACATACCTAGAAGAATAAGACAGGGCTTTGAAAGGGCTTTGCTATAA

2.1056.SPD.EU575325 ATG---------GAGATCAGGAAGAATTATCAGCACTTG---------TGGAGATGGGGG------------------------ATCATGCTCCTTTGGTTATTAATGAGC------------TGTAGTGCTGAA---------GAAGAAGCGTGGGTCACAGTTTATTATGGGGTACCTGTGTGGAAAGAAGCAGTCACCACTCTATTTTGTGCATCAGACGCAAAAGCATATGATACAGAGGTACATAAT---GTTTGGACCACACATGCCTGTGTACCCACAGACCCCGACCCACAAGAAGTACACATG---GAAAATGTGACAGAAGATTTTAACATGTGGAAAAATAACATGGCAGATCAGATGCATGAGGATATAATCAGTTTATGGGATCAAAGTCTAAAGCCATGTGTAAAATTAACCCCACTCTGTGTTACTTTAAATTGTGCTGATTGGAAGAATAATACTGATACCAATACC---------------------------------------------------------------------------------------AATAGTAGTGTGAGAATAATGGAGAAAGGAGAAATAAAAAACTGCTCTTTCAATATCACC---ACAAACATAAGAGAT------AAGTATCAGAAAGCATATGCACTTTTTTATAAACTTGATGTAGTACCAATAGATGATGAT------------------------------AATGCAACA------GGTAATAATGATACTAGAAACTATAGGTTGATAAGTTGTAACACCTCAGTCATTACACAGGCCTGTCCAAAGGTATCCTTTGAACCAATTCCCATACATTATTGTGCCCCGGCTGGTTTTGCGATTTTAAGGTGT---AATAATAAGACATTCAGTGGAAAAGGACAATGTACAAATGTCAGCACAGTACAATGTACACATGGAATTAAGCCAGTAGTATCAACTCAACTGCTATTAAATGGCAGTCTAGCAGAAGAA---GAGGTAATAATTAGATCTGACAATTTCTCGGACAATGCTAAAACCATAATAGTACATCTAAACAGCTCTGTAGACATTAATTGTACAAGACCAGGCAACAATACAAGAAAAAGTATAACTATAGGA------------CCAGGGAGG---GCATTTTATGCAACAGGAGACATAATAGGAGATATAAGACAAGCACATTGTAACATT------AGTGGAGAAAAATGGAATAACACTTTAAAACAGGTAGTTAAAAAATTAAGA---GAACAATTTGGG------------AATAAAACA---ATAGTCTTTAATCAA---------TCCTCAGGAGGGGACCCAGAAATTACAATGCACACTTTTAATTGTGGAGGGGAATTCTTCTACTGTAATACAGCACAACTGTTTAATAGTACTTGGGAAGCT---------AATAGTACTTGGGAAAATGAT---------------------AATGAAAGGGTAGGTCAC------------AGTAACAAGACT---------------------------ATCATACTACAATGCAGAATAAAACAAATTATAAACATGTGGCAGGAAGTAGGAAAAGCAATGTATGCCCCTCCCATCAGCGGACAGATTAGATGTTCATCAAATATTACGGGGCTGCTATTAACAAGAGATGGTGGTAACGGT------------AACGAGACC---------------------AACCGGACC---GAGGTCTTCAGACCTGGAGGAGGAAATATGAAAGATAACTGGAGA---AGTGAATTATATAAATATAAAGTAGTAAAAATTGAACCA---TTAGGAGTAGCACCC---ACCAGGGCAAAGAGAAGAGTGGTGCAAAGAGAA---AAAAGAGCAGTG---GGA---ATGGGA---GCTTTG---TTCATT---GGG---------TTCTTGTCA---GCAGCAGGAAGCACTATGGGCGCAGCGTCAATG---ACGCTGACGGTACAGGCCAGACAATTATTGTCTGGTATAGTGCAACAGCAGAGCAATTTGCTGAGAGCTATTGAGGCGCAACAACATCTGTTGCAACTCACAGTCTGGGGCATCAAGCAGCTCCAGGCAAGA---GTCCTGGCTGTGGAAAGATACCTAAAGGATCAACAGCTCCTAGGGATTTGGGGTTGCTCTGGAAAACTCATTTGCACCACTACAGTGCCTTGGAATTATAGTTGGAGTCCT------------------------AATAAAACTATGGATGACATTTGGGGT---AACATGACCTGGATGCAATGGGAAAGAGAAATTGAC------AATTATACAGGCATAATATACAGATTAATTGAAATATGGCAAAACCAGCAAGAAAAGAATGAACAAGAATTATTGGAATTAGATAAATGGGCAAGTTTGTGGAATTGGTTTGACATAACAAAGTGGCTGTGGTATATAAAAATATTCATAATGATAATAGGAGGCTTAGTAGGTTTAAGAATAGTCTTTACTGTGCTTTCTATAGTAAATAGAGTTAGGCAGGGATACTCACCATTATCGTTTCAGACCCGC---TTCCCAGCCCCAGGGGGA------CCCGACAGGCCCGAAGGAACAGAAGAAGAAGGTGGAGAGAGAGACAGAGACAGATCCAGTCGATCAGCGGATGGATTCTTAGCAATTATCTGGGTCGATCTGAGGAGCCTGTGCCTGTTCATCTACCACAGCTTGAGAGACTTACTCTTGATTGTAGCAAGGATTGTGGGACTTCTGGGACGCAGG---------------GGGTGGGAACTCCTCAAATATTGGTGG---AATCTCCTCCAGTATTGG---------------------------------------------------AGTCAGGAACTAAAGAATAGTGCTGTTAGCTTGCTGAATGCCACAGCTATAGCAGTAGCTGAGGGGACAGATAGGGTTATAGACATAGTACAAAGA------------------ATTTGCAGAGCTATCCTCCACATACCTAGAAGAATAAGACAGGGCTTTGAAAGGGCTTTGCTATAA

2.1056.SPD.EU575319 ATG---------GAGATCAGGAAGAATTATCAGCACTTG---------TGGAGATGGGGG------------------------ATCATGCTCCTTTGGTTATTAATGAGC------------TGTAGTGCTGAA---------GAAGAAGCGTGGGTCACAGTTTATTATGGGGTACCTGTGTGGAAAGAAGCAGTCACCACTCTATTTTGTGCATCAGACGCAAAAGCATATGATACAGAGGTACATAAT---GTTTGGACCACACATGCCTGTGTACCCACAGACCCCGACCCACAAGAAGTACACATG---GAAAATGTGACAGAAGATTTTAACATGTGGAAAAATAACATGGCAGATCAGATGCATGAGGATATAATCAGTTTATGGGATCAAAGTCTAAAGCCATGTGTAAAATTAACCCCACTCTGTGTTACTTTAAATTGTGCTGATTGGAAGAATAATACTGATACCAATACC---------------------------------------------------------------------------------------AATAGTAGTGTGAGAATAATGGAGAAAGGAGAAATAAAAAACTGCTCTTTCAATATCACC---ACAAACATAAGAGAT------AAGTATCAGAAAGCATATGCACTTTTTTATAAACTTGATGTAGTACCAATAGATGATGAT------------------------------AATGCAACA------GGTAATAATGATACTAGAAACTATAGGTTGATAAGTTGTAACACCTCAGTCATTACACAGGCCTGTCCAAAGGTATCCTTTGAACCAATTCCCATACATTATTGTGCCCCGGCTGGTTTTGCGATTTTAAGGTGT---AATAATAAGACATTCAGTGGAAAAGGACAATGTACAAATGTCAGCACAGTACAATGTACACATGGAATTAAGCCAGTAGTATCAACTCAACTGCTATTAAATGGCAGTCTAGCAGAAGAA---GAGGTAATAATTAGATCTGACAATTTCTCGGACAATGCTAAAACCATAATAGTACATCTAAACAGCTCTGTAGACATTAATTGTACAAGACCAGGCAACAATACAAGAAAAAGTATAACTATAGGA------------CCAGGGAGG---GCATTTTATGCAACAGGAGACATAATAGGAGATATAAGACAAGCACATTGTAACATT------AGTGGAGAAAAATGGAATAACACTTTAAAACAGGTAGTTAAAAAATTAAGA---GAACAATTTGGG------------AATAAAACA---ATAGTCTTTAATCAA---------TCCTCAGGAGGGGACCCAGAAATTACAATGCACACTTTTAATTGTGGAGGGGAATTCTTCTACTGTAATACAGCACAACTGTTTAATAGTACTTGGGAAGCT---------AATAGTACTTGGGAAAATGAT---------------------AATGAAAGGGTAGGTCAC------------AGTAACAAGACT---------------------------ATCATACTACAATGCAGAATAAAACAAATTATAAACATGTGGCAGGAAGTAGGAAAAGCAATGTATGCCCCTCCCATCAGCGGACAGATTAGATGTTCATCAAATATTACGGGGCTGCTATTAACAAGAGATGGTGGTAACGGT------------AACGAGACC---------------------AACCGGACC---GAGGTCTTCAGACCTGGAGGAGGAAATATGAAAGATAACTGGAGA---AGTGAATTATATAAATATAAAGTAGTAAAAATTGAACCA---TTAGGAGTAGCACCC---ACCAGGGCAAAGAGAAGAGTGGTGCAAAGAGAA---AAAAGAGCAGTG---GGA---ATGGGA---GCTTTG---TTCATT---GGG---------TTCTTGTCA---GCAGCAGGAAGCACTATGGGCGCAGCGTCAATG---ACGCTGACGGTACAGGCCAGACAATTATTGTCTGGTATAGTGCAACAGCAGAGCAATTTGCTGAGAGCTATTGAGGCGCAACAACATCTGTTGCAACTCACAGTCTGGGGCATCAAGCAGCTCCAGGCAAGA---GTCCTGGCTGTGGAAAGATACCTAAAGGATCAACAGCTCCTAGGGATTTGGGGTTGCTCTGGAAAACTCATTTGCACCACTACAGTGCCTTGGAATTATAGTTGGAGTCCT------------------------AATAAAACTATGGATGACATTTGGGGT---AACATGACCTGGATGCAATGGGAAAGAGAAATTGAC------AATTATACAGGCATAATATACAGATTAATTGAAATATCGCAAAACCAGCAAGAAAAGAATGAACAAGAATTATTGGAATTAGATAAATGGGCAAGTTTGTGGAATTGGTTTGACATAACAAAGTGGCTGTGGTATATAAAAATATTCATAATGATAATAGGAGGCTTAGTAGGTTTAAGAATAGTCTTTACTGTGCTTTCTATAGTAAATAGAGTTAGGCAGGGATACTCACCATTATCGTTTCAGACCCGC---TTCCCAGCCCCAGGGGGA------CCCGACAGGCCCGAAGGAACAGAAGAAGAAGGTGGAGAGAGAGACAGAGACAGATCCAGTCGATCAGCGGATGGATTCTTAGCAATTATCTGGGTCGATCTGAGGAGCCTGTGCCTGTTCATCTACCACAGCTTGAGAGACTTACTCTTGATTGTAGCAAGGATTGTGGGACTTCTGGGACGCAGG---------------GGGTGGGAACTCCTCAAATATTGGTGG---AATCTCCTCCAGTATTGG---------------------------------------------------AGTCAGGAACTAAAGAATAGTGCTGTTAGCTTGCTGAATGCCACAGCTATAGCAGTAGCTGAGGGGACAGATAGGGTTATAGACATAGTACAAAGA------------------ATTTGCAGAGCTATCCTCCACATACCTAGAAGAATAAGACAGGGCTTTGAAAGGGCTTTGCTATAA

2.1056.SPD.EU575307 ATG---------GAGATCAGGAAGAATTATCAGCACTTG---------TGGAGATGGGGG------------------------ATCATGCTCCTTTGGTTATTAATGAGC------------TGTAGTGCTGAA---------GAAGAAGCGTGGGTCACAGTTTATTATGGGGTACCTGTGTGGAAAGAAGCAGTCACCACTCTATTTTGTGCATCAGACGCAAAAGCATATGATACAGAGGTACATAAT---GTTTGGACCACACATGCCTGTGTACCCACAGACCCCGACCCACAAGAAGTACACATG---GAAAATGTGACAGAAGATTTTAACATGTGGAAAAATAACATGGCAGATCAGATGCATGAGGATATAATCAGTTTATGGGATCAAAGTCTAAAGCCATGTGTAAAATTAACCCCACTCTGTGTTACTTTAAATTGTGCTGATTGGAAGAATAATACTGATACCAATACC---------------------------------------------------------------------------------------AATAGTAGTGTGAGAATAATGGAGAAAGGAGAAATAAAAAACTGCTCTTTCAATATCACC---ACAAACATAAGAGAT------AAGTATCAGAAAGCATATGCACTTTTTTATAAACTTGATGTAGTACCAATAGATGATGAT------------------------------AATGCAACA------GGTAATAATGATACTAGAAACTATAGGTTGATAAGTTGTAACACCTCAGTCATTACACAGGCCTGTCCAAAGGTATCCTTTGAACCAATTCCCATACATTATTGTGCCCCGGCTGGTTTTGCGATTTTAAGGTGT---AATAATAAGACATTCAGTGGAAAAGGACAATGTACAAATGTCAGCACAGTACAATGTACACATGGAATTAAGCCAGTAGTATCAACTCAACTGCTATTAAATGGCAGTCTAGCAGAAGAA---GAGGTAATAATTAGATCTGACAATTTCTCGGACAATGCTAAAACCATAATAGTACATCTAAACAGCTCTGTAGACATTAATTGTACAAGACCAGGCAACAATACAAGAAAAAGTATAACTATAGGA------------CCAGGGAGG---GCATTTTATGCAACAGGAGACATAATAGGAGATATAAGACAAGCACATTGTAACATT------AGTGGAGAAAAATGGAATAACACTTTAAAACAGGTAGTTAAAAAATTAAGA---GAACAATTTGGG------------AATAAAACA---ATAGTCTTTAATCAA---------TCCTCAGGAGGGGACCCAGAAATTACAATGCACACTTTTAATTGTGGAGGGGAATTCTTCTACTGTAATACAGCACAACTGTTTAATAGTACTTGGGAAGCT---------AATAGTACTTGGGAAAATGAT---------------------AATGAAAGGGTAGGTCAC------------AGTAACAAGACT---------------------------ATCATACTACAATGCAGAATAAAACAAATTATAAACATGTGGCAGGAAGTAGGAAAAGCAATGTATGCCCCTCCCATCAGCGGACAGATTAGATGTTCATCAAATATTACGGGGCTGCTATTAACAAGAGATGGTGGTAACGGT------------AACGAGACC---------------------AACCGGACC---GAGGTCTTCAGACCTGGAGGAGGAAATATGAAAGATAACTGGAGA---AGTGAATTATATAAATATAAAGTAGTAAAAATTGAACCA---TTAGGAGTAGCACCC---ACCAGGGCAAAGAGAAGAGTGGTGCAAAGAGAA---AAAAGAGCAGTG---GGA---ATGGGA---GCTTTG---TTCATT---GGG---------TTCTTGTCA---GCAGCAGGAAGCACTATGGGCGCAGCGTCAATG---ACGCTGACGGTACAGGCCAGACAATTATTGTCTGGTATAGTGCAACAGCAGAGCAATTTGCTGAGAGCTATTGAGGCGCAACAACATCTGTTGCAACTCACAGTCTGGGGCATCAAGCAGCTCCAGGCAAGA---GTCCTGGCTGTGGAAAGATACCTAAAGGATCAACAGCTCCTAGGGATTTGGGGTTGCTCTGGAAAACTCATTTGCACCACTACAGTGCCTTGGAATTATAGTTGGAGTCCT------------------------AATAAAACTATGGATGACATTTGGGGT---AACATGACCTGGATGCAATGGGAAAGAGAAATTGAC------AATTATACAGGCATAATATACAGATTAATTGAAATATCGCAAAACCAGCAAGAAAAGAATGAACAAGAATTATTGGAATTAGATAAATGGGCAAGTTTGTGGAATTGGTTTGACATAACAAAGTGGCTGTGGTATATAAAAATATTCATAATGATAATAGGAGGCTTAGTAGGTTTAAGAATAGTCTTTACTGTGCTTTCTATAGTAAATAGAGTTAGGCAGGGATACTCACCATTATCGTTTCAGACCCGC---TTCCCAGCCCCAGGGGGA------CCCGACAGGCCCGAAGGAACAGAAGAAGAAGGTGGAGAGAGAGACAGAGACAGATCCAGTCGATCAGCGGATGGATTCTTAGCAATTATCTGGGTCGATCTGAGGAGCCTGTGCCTGTTCATCTACCACAGCTTGAGAGACTTACTCTTGATTGTAGCAAGGATTGTGGGACTTCTGGGACGCAGG---------------GGGTGGGAACTCCTCAAATATTGGTGG---AATCTCCTCCAGTATTGG---------------------------------------------------AGTCAGGAACTAAAGAATAGTGCTGTTAGCTTGCTGAATGCCACAGCTATAGCAGTAGCTGAGGGGACAGATAGGGTTATAGACATAGTACAAAGA------------------ATTTGCAGAGCTATCCTCCACATACCTAGAAGAATAAGACAGGGCTTTGAAAGGGCTTTGCTATAA

2.1056.SPD.EU575306 ATG---------GAGATCAGGAAGAATTATCAGCACTTG---------TGGAGATGGGGG------------------------ATCATGCTCCTTTGGTTATTAATGAGC------------TGTAGTGCTGAA---------GAAGAAGCGTGGGTCACAGTTTATTATGGGGTACCTGTGTGGAAAGAAGCAGTCACCACTCTATTTTGTGCATCAGACGCAAAAGCATATGATACAGAGGTACATAAT---GTTTGGACCACACATGCCTGTGTACCCACAGACCCCGACCCACAAGAAGTACACATG---GAAAATGTGACAGAAGATTTTAACATGTGGAAAAATAACATGGCAGATCAGATGCATGAGGATATAATCAGTTTATGGGATCAAAGTCTAAAGCCATGTGTAAAATTAACCCCACTCTGTGTTACTTTAAATTGTGCTGATTGGAAGAATAATACTGATACCAATACC---------------------------------------------------------------------------------------AATAGTAGTGTGAGAATAATGGAGAAAGGAGAAATAAAAAACTGCTCTTTCAATATCACC---ACAAACATAAGAGAT------AAGTATCAGAAAGCATATGCACTTTTTTATAAACTTGATGTAGTACCAATAGATGATGAT------------------------------AATGCAACA------GGTAATAATGATACTAGAAACTATAGGTTGATAAGTTGTAACACCTCAGTCATTACACAGGCCTGTCCAAAGGTATCCTTTGAACCAATTCCCATACATTATTGTGCCCCGGCTGGTTTTGCGATTTTAAGGTGT---AATAATAAGACATTCAGTGGAAAAGGACAATGTACAAATGTCAGCACAGTACAATGTACACATGGAATTAAGCCAGTAGTATCAACTCAACTGCTATTAAATGGCAGTCTAGCAGAAGAA---GAGGTAATAATTAGATCTGACAATTTCTCGGACAATGCTAAAACCATAATAGTACATCTAAACAGCTCTGTAGACATTAATTGTACAAGACCAGGCAACAATACAAGAAAAAGTATAACTATAGGA------------CCAGGGAGG---GCATTTTATGCAACAGGAGACATAATAGGAGATATAAGACAAGCACATTGTAACATT------AGTGGAGAAAAATGGAATAACACTTTAAAACAGGTAGTTAAAAAATTAAGA---GAACAATTTGGG------------AATAAAACA---ATAGTCTTTAATCAA---------TCCTCAGGAGGGGACCCAGAAATTACAATGCACACTTTTAATTGTGGAGGGGAATTCTTCTACTGTAATACAGCACAACTGTTTAATAGTACTTGGGAAGCT---------AATAGTACTTGGGAAAATGAT---------------------AATGAAAGGGTAGGTCAC------------AGTAACAAGACT---------------------------ATCATACTACAATGCAGAATAAAACAAATTATAAACATGTGGCAGGAAGTAGGAAAAGCAATGTATGCCCCTCCCATCAGCGGACAGATTAGATGTTCATCAAATATTACGGGGCTGCTATTAACAAGAGATGGTGGTAACGGT------------AACGAGACC---------------------AACCGGACC---GAGGTCTTCAGACCTGGAGGAGGAAATATGAAAGATAACTGGAGA---AGTGAATTATATAAATATAAAGTAGTAAAAATTGAACCA---TTAGGAGTAGCACCC---ACCAGGGCAAAGAGAAGAGTGGTGCAAAGAGAA---AAAAGAGCAGTG---GGA---ATGGGA---GCTTTG---TTCATT---GGG---------TTCTTGTCA---GCAGCAGGAAGCACTATGGGCGCAGCGTCAATG---ACGCTGACGGTACAGGCCAGACAATTATTGTCTGGTATAGTGCAACAGCAGAGCAATTTGCTGAGAGCTATTGAGGCGCAACAACATCTGTTGCAACTCACAGTCTGGGGCATCAAGCAGCTCCAGGCAAGA---GTCCTGGCTGTGGAAAGATACCTAAAGGATCAACAGCTCCTAGGGATTTGGGGTTGCTCTGGAAAACTCATTTGCACCACTACAGTGCCTTGGAATTATAGTTGGAGTCCT------------------------AATAAAACTATGGATGACATTTGGGGT---AACATGACCTGGATGCAATGGGAAAGAGAAATTGAC------AATTATACAGGCATAATATACAGATTAATTGAAATATCGCAAAACCAGCAAGAAAAGAATGAACAAGAATTATTGGAATTAGATAAATGGGCAAGTTTGTGGAATTGGTTTGACATAACAAAGTGGCTGTGGTATATAAAAATATTCATAATGATAATAGGAGGCTTAGTAGGTTTAAGAATAGTCTTTACTGTGCTTTCTATAGTAAATAGAGTTAGGCAGGGATACTCACCATTATCGTTTCAGACCCGC---TTCCCAGCCCCAGGGGGA------CCCGACAGGCCCGAAGGAACAGAAGAAGAAGGTGGAGAGAGAGACAGAGACAGATCCAGTCGATCAGCGGATGGATTCTTAGCAATTATCTGGGTCGATCTGAGGAGCCTGTGCCTGTTCATCTACCACAGCTTGAGAGACTTACTCTTGATTGTAGCAAGGATTGTGGGACTTCTGGGATGCAGG---------------GGGTGGGAACTCCTCAAATATTGGTGG---AATCTCCTCCAGTATTGG---------------------------------------------------AGTCAGGAACTAAAGAATAGTGCTGTTAGCTTGCTGAATGCCACAGCTATAGCAGTAGCTGAGGGGACAGATAGGGTTATAGACATAGTACAAAGA------------------ATTTGCAGAGCTATCCTCCACATACCTAGAAGAATAAGACAGGGCTTTGAAAGGGCTTTGCTATAA

2.1056.SPD.EU575310 ATG---------GAGATCAGGAAGAATTATCAGCACTTG---------TGGAGATGGGGG------------------------ATCATGCTCCTTTGGTTATTAATGAGC------------TGTAGTGCTGAA---------GAAGAAGCGTGGGTCACAGTTTATTATGGGGTACCTGTGTGGAAAGAAGCAGTCACCACTCTATTTTGTGCATCAGACGCAAAAGCATATGATACAGAGGTACATAAT---GTTTGGACCACACATGCCTGTGTACCCACAGACCCCGACCCACAAGAAGTACACATG---GAAAATGTGACAGAAGATTTTAACATGTGGAAAAATAACATGGCAGATCAGATGCATGAGGATATAATCAGTTTATGGGATCAAAGTCTAAAGCCATGTGTAAAATTAACCCCACTCTGTGTTACTTTAAATTGTGCTGATTGGAAGAATAATACTGATACCAATACC---------------------------------------------------------------------------------------AATAGTAGTGTGAGAATAATGGAGAAAGGAGAAATAAAAAACTGCTCTTTCAATATCACC---ACAAACATAAGAGAT------AAGTATCAGAAAGCATATGCACTTTTTTATAAACTTGATGTAGTACCAATAGATGATGAT------------------------------AATGCAACA------GGTAATAATGATACTAGAAACTATAGGTTGATAAGTTGTAACACCTCAGTCATTACACAGGCCTGTCCAAAGGTATCCTTTGAACCAATTCCCATACATTATTGTGCCCCGGCTGGTTTTGCGATTTTAAGGTGT---AATAATAAGACATTCAGTGGAAAAGGACAATGTACAAATGTCAGCACAGTACAATGTACACATGGAATTAAGCCAGTAGTATCAACTCAACTGCTATTAAATGGCAGTCTAGCAGAAGAA---GAGGTAATAATTAGATCTGACAATTTCTCGGACAATGCTAAAACCATAATAGTACATCTAAACAGCTCTGTAGACATTAATTGTACAAGACCAGGCAACAATACAAGAAAAAGTATAACTATAGGA------------CCAGGGAGG---GCATTTTATGCAACAGGAGACATAATAGGAGATATAAGACAAGCACATTGTAACATT------AGTGGAGAAAAATGGAATAACACTTTAAAACAGGTAGTTAAAAAATTAAGA---GAACAATTTGGG------------AATAAAACA---ATAGTCTTTAATCAA---------TCCTCAGGAGGGGACCCAGAAATTACAATGCACACTTTTAATTGTGGAGGGGAATTCTTCTACTGTAATACAGCACAACTGTTTAATAGTACTTGGGAAGCT---------AATAGTACTTGGGAAAATGAT---------------------AATGAAAGGGTAGGTCAC------------AGTAACAAGACT---------------------------ATCATACTACAATGCAGAATAAAACAAATTATAAACATGTGGCAGGAAGTAGGAAAAGCAATGTATGCCCCTCCCATCAGCGGACAGATTAGATGTTCATCAAATATTACGGGGCTGCTATTAACAAGAGATGGTGGTAACGGT------------AACGAGACC---------------------AACCGGACC---GAGGTCTTCAGACCTGGAGGAGGAAATATGAAAGATAACTGGAGA---AGTGAATTATATAAATATAAAGTAGTAAAAATTGAACCA---TTAGGAGTAGCACCC---ACCAGGGCAAAGAGAAGAGTGGTGCAAAGAGAA---AAAAGAGCAGTG---GGA---ATGGGA---GCTTTG---TTCATT---GGG---------TTCTTGTCA---GCAGCAGGAAGCACTATGGGCGCAGCGTCAATG---ACGCTGACGGTACAGGCCAGACAATTATTGTCTGGTATAGTGCAACAGCAGAGCAATTTGCTGAGAGCTATTGAGGCGCAACAACATCTGTTGCAACTCACAGTCTGGGGCATCAAGCAGCTCCAGGCAAGA---GTCCTGGCTGTGGAAAGATACCTAAAGGATCAACAGCTCCTAGGGATTTGGGGTTGCTCTGGAAAACTCATTTGCACCACTACAGTGCCTTGGAATTATAGTTGGAGTCCT------------------------AATAAAACTATGGATGACATTTGGGGT---AACATGACCTGGATGCAATGGGAAAGAGAAATTGAC------AATTATACAGGCATAATATACAGATTAATTGAAATATCGCAAAACCAGCAAGAAAAGAATGAACAAGAATTATTGGAATTAGATAAATGGGCAAGTTTGTGGAATTGGTTTGACATAACAAAGTGGCTGTGGTATATAAAAATATTCATAATGATAATAGGAGGCTTAGTAGGTTTAAGAATAGTCTTTACTGTGCTTTCTATAGTAAATAGAGTTAGGCAGGGATACTCACCATTATCGTTTCAGACCCGC---TTCCCAGCCCCAGGGGGA------CCCGACAGGCCCGAAGGAACAGAAGAAGAAGGTGGAGAGAGAGACAGAGACAGATCCAGTCGATCAGCGGATGGATTCTTAGCAATTATCTGGGTCGATCTGAGGAGCCTGTGCCTGTTCATCTACCACAGCTTGAGAGACTTACTCTTGATTGTAGCAAGGATTGTGGGACTTCTGGGACGCAGG---------------GGGTGGGAACTCCTCAAATATTGGTGG---AATCTCCTCCAGTATTGG---------------------------------------------------AGTCAGGAACTAAAGAATAGTGCTGTTAGCTTGCTGAATGCCACAGCTATAGCAGTAGCTGAGGGGACAGATAGGGTTATAGACATAGTACAAAGA------------------ATTTGCAGAGCTATCCTCCACATACCTAGAAGAATAAGACAGGGCTTTGAAAGGGCTTTGCTATAA

2.1056.SPD.EU575324 ATG---------GAGATCAGGAAGAATTATCAGCACTTG---------TGGAGATGGGGG------------------------ATCATGCTCCTTTGGTTATTAATGAGC------------TGTAGTGCTGAA---------GAAGAAGCGTGGGTCACAGTTTATTATGGGGTACCTGTGTGGAAAGAAGCAGTCACCACTCTATTTTGTGCATCAGACGCAAAAGCATATGATACAGAGGTACATAAT---GTTTGGACCACACATGCCTGTGTACCCACAGACCCCGACCCACAAGAAGTACACATG---GAAAATGTGACAGAAGATTTTAACATGTGGAAAAATAACATGGCAGATCAGATGCATGAGGATATAATCAGTTTATGGGATCAAAGTCTAAAGCCATGTGTAAAATTAACCCCACTCTGTGTTACTTTAAATTGTGCTGATTGGAAGAATAATACTGATACCAATACC---------------------------------------------------------------------------------------AATAGTAGTGTGAGAATAATGGAGAAAGGAGAAATAAAAAACTGCTCTTTCAATATCACC---ACAAACATAAGAGAT------AAGTATCAGAAAGCATATGCACTTTTTTATAAACTTGATGTAGTACCAATAGATGATGAT------------------------------AATGCAACA------GGTAATAATGATACTAGAAACTATAGGTTGATAAGTTGTAACACCTCAGTCATTACACAGGCCTGTCCAAAGGTATCCTTTGAACCAATTCCCATACATTATTGTGCCCCGGCTGGTTTTGCGATTTTAAGGTGT---AATAATAAGACATTCAGTGGAAAAGGACAATGTACAAATGTCAGCACAGTACAATGTACACATGGAATTAAGCCAGTAGTATCAACTCAACTGCTATTAAATGGCAGTCTAGCAGAAGAA---GAGGTAATAATTAGATCTGACAATTTCTCGGACAATGCTAAAACCATAATAGTACATCTAAACAGCTCTGTAGACATTAATTGTACAAGACCAGGCAACAATACAAGAAAAAGTATAACTATAGGA------------CCAGGGAGG---GCATTTTATGCAACAGGAGACATAATAGGAGATATAAGACAAGCACATTGTAACATT------AGTGGAGAAAAATGGAATAACACTTTAAAACAGGTAGTTAAAAAATTAAGA---GAACAATTTGGG------------AATAAAACA---ATAGTCTTTAATCAA---------TCCTCAGGAGGGGACCCAGAAATTACAATGCACACTTTTAATTGTGGAGGGGAATTCTTCTACTGTAATACAGCACAACTGTTTAATAGTACTTGGGAAGCT---------AATAGTACTTGGGAAAATGAT---------------------AATGAAAGGGTAGGTCAC------------AGTAACAAGACT---------------------------ATCATACTACAATGCAGAATAAAACAAATTATAAACATGTGGCAGGAAGTAGGAAAAGCAATGTATGCCCCTCCCATCAGCGGACAGATTAGATGTTCATCAAATATTACGGGGCTGCTATTAACAAGAGATGGTGGTAACGGT------------AACGAGACC---------------------AACCGGACC---GAGGTCTTCAGACCTGGAGGAGGAAATATGAAAGATAACTGGAGA---AGTGAATTATATAAATATAAAGTAGTAAAAATTGAACCA---TTAGGAGTAGCACCC---ACCAGGGCAAAGAGAAGAGTGGTGCAAAGAGAA---AAAAGAGCAGTG---GGA---ATGGGA---GCTTTG---TTCATT---GGG---------TTCTTGTCA---GCAGCAGGAAGCACTATGGGCGCAGCGTCAATG---ACGCTGACGGTACAGGCCAGACAATTATTGTCTGGTATAGTGCAACAGCAGAGCAATTTGCTGAGAGCTATTGAGGCGCAACAACATCTGTTGCAACTCACAGTCTGGGGCATCAAGCAGCTCCAGGCAAGA---GTCCTGGCTGTGGAAAGATACCTAAAGGATCAACAGCTCCTAGGGATTTGGGGTTGCTCTGGAAAACTCATTTGCACCACTACAGTGCCTTGGAATTATAGTTGGAGTCCT------------------------AATAAAACTATGGATGACATTTGGGGT---AACATGACCTGGATGCAATGGGAAAGAGAAATTGAC------AATTATACAGGCATAATATACAGATTAATTGAAATATCGCAAAACCAGCAAGAAAAGAATGAACAAGAATTATTGGAATTAGATAAATGGGCAAGTTTGTGGAATTGGTTTGACATAACAAAGTGGCTGTGGTATATAAAAATATTCATAATGATAATAGGAGGCTTAGTAGGTTTAAGAATAGTCTTTACTGTGCTTTCTATAGTAAATAGAGTTAGGCAGGGATACTCACCATTATCGTTTCAGACCCGC---TTCCCAGCCCCAGGGGGA------CCCGACAGGCCCGAAGGAACAGAAGAAGAAGGTGGAGAGAGAGACAGAGACAGATCCAGTCGATCAGCGGATGGATTCTTAGCAATTATCTGGGTCGATCTGAGGAGCCTGTGCCTGTTCATCTACCACAGCTTGAGAGACTTACTCTTGATTGTAGCAAGGATTGTGGGACTTCTGGGACGCAGG---------------GGGTGGGAACTCCTCAAATATTGGTGG---AATCTCCTCCAGTATTGG---------------------------------------------------AGTCAGGAACTAAAGAATAGTGCTGTTAGCTTGCTGAATGCCACAGCTATAGCAGTAGCTGAGGGGACAGATAGGGTTATAGACATAGTACAAAGA------------------ATTTGCAGAGCTATCCTCCACATACCTAGAAGAATAAGACAGGGCTTTGAAAGGGCTTTGCTATAA

2.1056.SPD.EU575294 ATG---------GAGATCAGGAAGAATTATCAGCACTTG---------TGGAGATGGGGG------------------------ATCATGCTCCTTTGGTTATTAATGAGC------------TGTAGTGCTGAA---------GAAGAAGCGTGGGTCACAGTTTATTATGGGGTACCTGTGTGGAAAGAAGCAGTCACCACTCTATTTTGTGCATCAGACGCAAAAGCATATGATACAGAGGTACATAAT---GTTTGGACCACACATGCCTGTGTACCCACAGACCCCGACCCACAAGAAGTACACATG---GAAAATGTGACAGAAGATTTTAACATGTGGAAAAATAACATGGCAGATCAGATGCATGAGGATATAATCAGTTTATGGGATCAAAGTCTAAAGCCATGTGTAAAATTAACCCCACTCTGTGTTACTTTAAATTGTGCTGATTGGAAGAATAATACTGATACCAATACC---------------------------------------------------------------------------------------AATAGTAGTGTGAGAATAATGGAGAAAGGAGAAATAAAAAACTGCTCTTTCAATATCACC---ACAAACATAAGAGAT------AAGTATCAGAAAGCATATGCACTTTTTTATAAACTTGATGTAGTACCAATAGATGATGAT------------------------------AATGCAACA------GGTAATAATGATACTAGAAACTATAGGTTGATAAGTTGTAACACCTCAGTCATTACACAGGCCTGTCCAAAGGTATCCTTTGAACCAATTCCCATACATTATTGTGCCCCGGCTGGTTTTGCGATTTTAAGGTGT---AATAATAAGACATTCAGTGGAAAAGGACAATGTACAAATGTCAGCACAGTACAATGTACACATGGAATTAAGCCAGTAGTATCAACTCAACTGCTATTAAATGGCAGTCTAGCAGAAGAA---GAGGTAATAATTAGATCTGACAATTTCTCGGACAATGCTAAAACCATAATAGTACATCTAAACAGCTCTGTAGACATTAATTGTACAAGACCAGGCAACAATACAAGAAAAAGTATAACTATAGGA------------CCAGGGAGG---GCATTTTATGCAACAGGAGACATAATAGGAGATATAAGACAAGCACATTGTAACATT------AGTGGAGAAAAATGGAATAACACTTTAAAACAGGTAGTTAAAAAATTAAGA---GAACAATTTGGG------------AATAAAACA---ATAGTCTTTAATCAA---------TCCTCAGGAGGGGACCCAGAAATTACAATGCACACTTTTAATTGTGGAGGGGAATTCTTCTACTGTAATACAGCACAACTGTTTAATAGTACTTGGGAAGCT---------AATAGTACTTGGGAAAATGAT---------------------AATGAAAGGGTAGGTCAC------------AGTAACAAGACT---------------------------ATCATACTACAATGCAGAATAAAACAAATTATAAACATGTGGCAGGAAGTAGGAAAAGCAATGTATGCCCCTCCCATCAGCGGACAGATTAGATGTTCATCAAATATTACGGGGCTGCTATTAACAAGAGATGGTGGTAACGGT------------AACGAGACC---------------------AACCGGACC---GAGGTCTTCAGACCTGGAGGAGGAAATATGAAAGATAACTGGAGA---AGTGAATTATATAAATATAAAGTAGTAAAAATTGAACCA---TTAGGAGTAGCACCC---ACCAGGGCAAAGAGAAGAGTGGTGCAAAGAGAA---AAAAGAGCAGTG---GGA---ATGGGA---GCTTTG---TTCATT---GGG---------TTCTTGTCA---GCAGCAGGAAGCACTATGGGCGCAGCGTCAATG---ACGCTGACGGTACAGGCCAGACAATTATTGTCTGGTATAGTGCAACAGCAGAGCAATTTGCTGAGAGCTATTGAGGCGCAACAACATCTGTTGCAACTCACAGTCTGGGGCATCAAGCAGCTCCAGGCAAGA---GTCCTGGCTGTGGAAAGATACCTAAAGGATCAACAGCTCCTAGGGATTTGGGGTTGCTCTGGAAAACTCATTTGCACCACTACAGTGCCTTGGAATTATAGTTGGAGTCCT------------------------AATAAAACTATGGATGACATTTGGGGT---AACATGACCTGGATGCAATGGGAAAGAGAAATTGAC------AATTATACAGGCATAATATACAGATTAATTGAAATATCGCAAAACCAGCAAGAAAAGAATGAACAAGAATTATTGGAATTAGATAAATGGGCAAGTTTGTGGAATTGGTTTGACATAACAAAGTGGCTGTGGTATATAAAAATATTCATAATGATAATAGGAGGCTTAGTAGGTTTAAGAATAGTCTTTACTGTGCTTTCTATAGTAAATAGAGTTAGGCAGGGATACTCACCATTATCGTTTCAGACCCGC---TTCCCAGCCCCAGGGGGA------CCCGACAGGCCCGAAGGAACAGAAGAAGAAGGTGGAGAGAGAGACAGAGACAGATCCAGTCGATCAGCGGATGGATTCTTAGCAATTATCTGGGTCGATCTGAGGAGCCTGTGCCTGTTCATCTACCACAGCTTGAGAGACTTACTCTTGATTGTAGCAAGGATTGTGGGACTTCTGGGACGCAGG---------------GGGTGGGAACTCCTCAAATATTGGTGG---AATCTCCTCCAGTATTGG---------------------------------------------------AGTCAGGAACTAAAGAATAGTGCTGTTAGCTTGCTGAATGCCACAGCTATAGCAGTAGCTGAGGGGACAGATAGGGTTATAGACATAGTACAAAGA------------------ATTTGCAGAGCTATCCTCCACATACCTAGAAGAATAAGACAGGGCTTTGAAAGGGCTTTGCTATAA

2.1056.SPD.EU575289 ATG---------GAGATCAGGAAGAATTATCAGCACTTG---------TGGAGATGGGGG------------------------ATCATGCTCCTTTGGTTATTAATGAGC------------TGTAGTGCTGAA---------GAAGAAGCGTGGGTCACAGTTTATTATGGGGTACCTGTGTGGAAAGAAGCAGTCACCACTCTATTTTGTGCATCAGACGCAAAAGCATATGATACAGAGGTACATAAT---GTTTGGACCACACATGCCTGTGTACCCACAGACCCCGACCCACAAGAAGTACACATG---GAAAATGTGACAGAAGATTTTAACATGTGGAAAAATAACATGGCAGATCAGATGCATGAGGATATAATCAGTTTATGGGATCAAAGTCTAAAGCCATGTGTAAAATTAACCCCACTCTGTGTTACTTTAAATTGTGCTGATTGGAAGAATAATACTGATACCAATACC---------------------------------------------------------------------------------------AATAGTAGTGTGAGAATAATGGAGAAAGGAGAAATAAAAAACTGCTCTTTCAATATCACC---ACAAACATAAGAGAT------AAGTATCAGAAAGCATATGCACTTTTTTATAAACTTGATGTAGTACCAATAGATGATGAT------------------------------AATGCAACA------GGTAATAATGATACTAGAAACTATAGGTTGATAAGTTGTAACACCTCAGTCATTACACAGGCCTGTCCAAAGGTATCCTTTGAACCAATTCCCATACATTATTGTGCCCCGGCTGGTTTTGCGATTTTAAGGTGT---AATAATAAGACATTCAGTGGAAAAGGACAATGTACAAATGTCAGCACAGTACAATGTACACATGGAATTAAGCCAGTAGTATCAACTCAACTGCTATTAAATGGCAGTCTAGCAGAAGAA---GAGGTAATAATTAGATCTGACAATTTCTCGGACAATGCTAAAACCATAATAGTACATCTAAACAGCTCTGTAGACATTAATTGTACAAGACCAGGCAACAATACAAGAAAAAGTATAACTATAGGA------------CCAGGGAGG---GCATTTTATGCAACAGGAGACATAATAGGAGATATAAGACAAGCACATTGTAACATT------AGTGGAGAAAAATGGAATAACACTTTAAAACAGGTAGTTAAAAAATTAAGA---GAACAATTTGGG------------AATAAAACA---ATAGTCTTTAATCAA---------TCCTCAGGAGGGGACCCAGAAATTACAATGCACACTTTTAATTGTGGAGGGGAATTCTTCTACTGTAATACAGCACAACTGTTTAATAGTACTTGGGAAGCT---------AATAGTACTTGGGAAAATGAT---------------------AATGAAAGGGTAGGTCAC------------AGTAACAAGACT---------------------------ATCATACTACAATGCAGAATAAAACAAATTATAAACATGTGGCAGGAAGTAGGAAAAGCAATGTATGCCCCTCCCATCAGCGGACAGATTAGATGTTCATCAAATATTACGGGGCTGCTATTAACAAGAGATGGTGGTAACGGT------------AACGAGACC---------------------AACCGGACC---GAGGTCTTCAGACCTGGAGGAGGAAATATGAAAGATAACTGGAGA---AGTGAATTATATAAATATAAAGTAGTAAAAATTGAACCA---TTAGGAGTAGCACCC---ACCAGGGCAAAGAGAAGAGTGGTGCAAAGAGAA---AAAAGAGCAGTG---GGA---ATGGGA---GCTTTG---TTCATT---GGG---------TTCTTGTCA---GCAGCAGGAAGCACTATGGGCGCAGCGTCAATG---ACGCTGACGGTACAGGCCAGACAATTATTGTCTGGTATAGTGCAACAGCAGAGCAATTTGCTGAGAGCTATTGAGGCGCAACAACATCTGTTGCAACTCACAGTCTGGGGCATCAAGCAGCTCCAGGCAAGA---GTCCTGGCTGTGGAAAGATACCTAAAGGATCAACAGCTCCTAGGGATTTGGGGTTGCTCTGGAAAACTCATTTGCACCACTACAGTGCCTTGGAATTATAGTTGGAGTCCT------------------------AATAAAACTATGGATGACATTTGGGGT---AACATGACCTGGATGCAATGGGAAAGAGAAATTGAC------AATTATACAGGCATAATATACAGATTAATTGAAATATCGCAAAACCAGCAAGAAAAGAATGAACAAGAATTATTGGAATTAGATAAATGGGCAAGTTTGTGGAATTGGTTTGACATAACAAAGTGGCTGTGGTATATAAAAATATTCATAATGATAATAGGAGGCTTAGTAGGTTTAAGAATAGTCTTTACTGTGCTTTCTATAGTAAATAGAGTTAGGCAGGGATACTCACCATTATCGTTTCAGACCCGCTTCTTCCCAGCCCCAGGGGGA------CCCGACAGGCCCGAAGGAACAGAAGAAGAAGGTGGAGAGAGAGACAGAGACAGATCCAGTCGATCAGCGGATGGATTCTTAGCAATTATCTGGGTCGATCTGAGGAGCCTGTGCCTGTTCATCTACCACAGCTTGAGAGACTTACTCTTGATTGTAGCAAGGATTGTGGGACTTCTGGGACGCAGG---------------GGGTGGGAACTCCTCAAATATTGGTGG---AATCTCCTCCAGTATTGG---------------------------------------------------AGTCAGGAACTAAAGAATAGTGCTGTTAGCTTGCTGAATGCCACAGCTATAGCAGTAGCTGAGGGGACAGATAGGGTTATAGACATAGTACAAAGA------------------ATTTGCAGAGCTATCCTCCACATACCTAGAAGAATAAGACAGGGCTTTGAAAGGGCTTTGCTATAA

2.1056.SPD.EU575305 ATG---------GAGATCAGGAAGAATTATCAGCACTTG---------TGGAGATGGGGG------------------------ATCATGCTCCTTTGGTTATTAATGAGC------------TGTAGTGCTGAA---------GAAGAAGCGTGGGTCACAGTTTATTATGGGGTACCTGTGTGGAAAGAAGCAGTCACCACTCTATTTTGTGCATCAGACGCAAAAGCATATGATACAGAGGTACATAAT---GTTTGGACCACACATGCCTGTGTACCCACAGACCCCGACCCACAAGAAGTACACATG---GAAAATGTGACAGAAGATTTTAACATGTGGAAAAATAACATGGCAGATCAGATGCATGAGGATATAATCAGTTTATGGGATCAAAGTCTAAAGCCATGTGTAAAATTAACCCCACTCTGTGTTACTTTAAATTGTGCTGATTGGAAGAATAATACTGATACCAATACC---------------------------------------------------------------------------------------AATAGTAGTGTGAGAATAATGGAGAAAGGAGAAATAAAAAACTGCTCTTTCAATATCACC---ACAAACATAAGAGAT------AAGTATCAGAAAGCATATGCACTTTTTTATAAACTTGATGTAGTACCAATAGATGATGAT------------------------------AATGCAACA------GGTAATAATGATACTAGAAACTATAGGTTGATAAGTTGTAACACCTCAGTCATTACACAGGCCTGTCCAAAGGTATCCTTTGAACCAATTCCCATACATTATTGTGCCCCGGCTGGTTTTGCGATTTTAAGGTGT---AATAATAAGACATTCAGTGGAAAAGGACAATGTACAAATGTCAGCACAGTACAATGTACACATGGAATTAAGCCAGTAGTATCAACTCAACTGCTATTAAATGGCAGTCTAGCAGAAGAA---GAGGTAATAATTAGATCTGACAATTTCTCGGACAATGCTAAAACCATAATAGTACATCTAAACAGCTCTGTAGACATTAATTGTACAAGACCAGGCAACAATACAAGAAAAAGTATAACTATAGGA------------CCAGGGAGG---GCATTTTATGCAACAGGAGACATAATAGGAGATATAAGACAAGCACATTGTAACATT------AGTGGAGAAAAATGGAATAACACTTTAAAACAGGTAGTTAAAAAATTAAGA---GAACAATTTGGG------------AATAAAACA---ATAGTCTTTAATCAA---------TCCTCAGGAGGGGACCCAGAAATTACAATGCACACTTTTAATTGTGGAGGGGAATTCTTCTACTGTAATACAGCACAACTGTTTAATAGTACTTGGGAAGCT---------AATAGTACTTGGGAAAATGAT---------------------AATGAAAGGGTAGGTCAC------------AGTAACAAGACT---------------------------ATCATACTACAATGCAGAATAAAACAAATTATAAACATGTGGCAGGAAGTAGGAAAAGCAATGTATGCCCCTCCCATCAGCGGACAGATTAGATGTTCATCAAATATTACGGGGCTGCTATTAACAAGAGATGGTGGTAACGGT------------AACGAGACC---------------------AACCGGACC---GAGGTCTTCAGACCTGGAGGAGGAAATATGAAAGATAACTGGAGA---AGTGAATTATATAAATATAAAGTAGTAAAAATTGAACCA---TTAGGAGTAGCACCC---ACCAGGGCAAAGAGAAGAGTGGTGCAAAGAGAA---AAAAGAGCAGTG---GGA---ATGGGA---GCTTTG---TTCATT---GGG---------TTCTTGTCA---GCAGCAGGAAGCACTATGGGCGCAGCGTCAATG---ACGCTGACGGTACAGGCCAGACAATTATTGTCTGGTATAGTGCAACAGCAGAGCAATTTGCTGAGAGCTATTGAGGCGCAACAACATCTGTTGCAACTCACAGTCTGGGGCATCAAGCAGCTCCAGGCAAGA---GTCCTGGCTGTGGAAAGATACCTAAAGGATCAACAGCTCCTAGGGATTTGGGGTTGCTCTGGAAAACTCATTTGCACCACTACAGTGCCTTGGAATTATAGTTGGAGTCCT------------------------AATAAAACTATGGATGACATTTGGGGT---AACATGACCTGGATGCAATGGGAAAGAGAAATTGAC------AATTATACAGGCATAATATACAGATTAATTGAAATATCGCAAAACCAGCAAGAAAAGAATGAACAAGAATTATTGGAATTAGATAAATGGGCAAGTTTGTGGAATTGGTTTGACATAACAAAGTGGCTGTGGTATATAAAAATATTCATAATGATAATAGGAGGCTTAGTAGGTTTAAGAATAGTCTTTACTGTGCTTTCTATAGTAAATAGAGTTAGGCAGGGATACTCACCATTATCGTTTCAGACCCGC---TTCCCAGCCCCAGGGGGA------CCCGACAGGCCCGAAGGAACAGAAGAAGAAGGTGGAGAGAGAGACAGAGACAGATCCAGTCGATCAGCGGATGGATTCTTAGCAATTATCTGGGTCGATCTGAGGAGCCTGTGCCTGTTCATCTACCACAGCTTGAGAGACTTACTCTTGATTGTAGCAAGGATTGTGGGACTTCTGGGACGCAGG---------------GGGTGGGAACTCCTCAAATATTGGTGG---AATCTCCTCCAGTATTGG---------------------------------------------------AGTCAGGAACTAAAGAATAGTGCTGTTAGCTTGCTGAATGCCACAGCTATAGCAGTAGCTGAGGGGACAGATAGGGTTATAGACATAGTACAAAGA------------------ATTTGCAGAGCTATCCTCCACATACCTAGAAGAATAAGACAGGGCTTTGAAAGGGCTTTGCTATAA

2.1056.SPD.EU575283 ATG---------GAGATCAGGAAGAATTATCAGCACTTG---------TGGAGATGGGGG------------------------ATCATGCTCCTTTGGTTATTAATGAGC------------TGTAGTGCTGAA---------GAAGAAGCGTGGGTCACAGTTTATTATGGGGTACCTGTGTGGAAAGAAGCAGTCACCACTCTATTTTGTGCATCAGACGCAAAAGCATATGATACAGAGGTACATAAT---GTTTGGACCACACATGCCTGTGTACCCACAGACCCCGACCCACAAGAAGTACACATG---GAAAATGTGACAGAAGATTTTAACATGTGGAAAAATAACATGGCAGATCAGATGCATGAGGATATAATCAGTTTATGGGATCAAAGTCTAAAGCCATGTGTAAAATTAACCCCACTCTGTGTTACTTTAAATTGTGCTGATTGGAAGAATAATACTGATACCAATACC---------------------------------------------------------------------------------------AATAGTAGTGTGAGAATAATGGAGAAAGGAGAAATAAAAAACTGCTCTTTCAATATCACC---ACAAACATAAGAGAT------AAGTATCAGAAAGCATATGCACTTTTTTATAAACTTGATGTAGTACCAATAGATGATGAT------------------------------AATGCAACA------GGTAATAATGATACTAGAAACTATAGGTTGATAAGTTGTAACACCTCAGTCATTACACAGGCCTGTCCAAAGGTATCCTTTGAACCAATTCCCATACATTATTGTGCCCCGGCTGGTTTTGCGATTTTAAGGTGT---AATAATAAGACATTCAGTGGAAAAGGACAATGTACAAATGTCAGCACAGTACAATGTACACATGGAATTAAGCCAGTAGTATCAACTCAACTGCTATTAAATGGCAGTCTAGCAGAAGAA---GAGGTAATAATTAGATCTGACAATTTCTCGGACAATGCTAAAACCATAATAGTACATCTAAACAGCTCTGTAGACATTAATTGTACAAGACCAGGCAACAATACAAGAAAAAGTATAACTATAGGA------------CCAGGGAGG---GCATTTTATGCAACAGGAGACATAATAGGAGATATAAGACAAGCACATTGTAACATT------AGTGGAGAAAAATGGAATAACACTTTAAAACAGGTAGTTAAAAAATTAAGA---GAACAATTTGGG------------AATAAAACA---ATAGTCTTTAATCAA---------TCCTCAGGAGGGGACCCAGAAATTACAATGCACACTTTTAATTGTGGAGGGGAATTCTTCTACTGTAATACAGCACAACTGTTTAATAGTACTTGGGAAGCT---------AATAGTACTTGGGAAAATGAT---------------------AATGAAAGGGTAGGTCAC------------AGTAACAAGACT---------------------------ATCATACTACAATGCAGAATAAAACAAATTATAAACATGTGGCAGGAAGTAGGAAAAGCAATGTATGCCCCTCCCATCAGCGGACAGATTAGATGTTCATCAAATATTACGGGGCTGCTATTAACAAGAGATGGTGGTAACGGT------------AACGAGACC---------------------AACCGGACC---GAGGTCTTCAGACCTGGAGGAGGAAATATGAAAGATAACTGGAGA---AGTGAATTATATAAATATAAAGTAGTAAAAATTGAACCA---TTAGGAGTAGCACCC---ACCAGGGCAAAGAGAAGAGTGGTGCAAAGAGAA---AAAAGAGCAGTG---GGA---ATGGGA---GCTTTG---TTCATT---GGG---------TTCTTGTCA---GCAGCAGGAAGCACTATGGGCGCAGCGTCAATG---ACGCTGACGGTACAGGCCAGACAATTATTGTCTGGTATAGTGCAACAGCAGAGCAATTTGCTGAGAGCTATTGAGGCGCAACAACATCTGTTGCAACTCACAGTCTGGGGCATCAAGCAGCTCCAGGCAAGA---GTCCTGGCTGTGGAAAGATACCTAAAGGATCAACAGCTCCTAGGGATTTGGGGTTGCTCTGGAAAACTCATTTGCACCACTACAGTGCCTTGGAATTATAGTTGGAGTCCT------------------------AATAAAACTATGGATGACATTTGGGGT---AACATGACCTGGATGCAATGGGAAAGAGAAATTGAC------AATTATACAGGCATAATATACAGATTAATTGAAATATCGCAAAACCAGCAAGAAAAGAATGAACAAGAATTATTGGAATTAGATAAATGGGCAAGTTTGTGGAATTGGTTTGACATAACAAAGTGGCTGTGGTATATAAAAATATTCATAATGATAATAGGAGGCTTAGTAGGTTTAAGAATAGTCTTTACTGTGCTTTCTATAGTAAATAGAGTTAGGCAGGGATACTCACCATTATCGTTTCAGACCCGC---TTCCCAGCCCCAGGGGGA------CCCGACAGGCCCGAAGGAACAGAAGAAGAAGGTGGAGAGAGAGACAGAGACAGATCCAGTCGATCAGCGGATGGATTCTTAGCAATTATCTGGGTCGATCTGAGGAGCCTGTGCCTGTTCATCTACCACAGCTTGAGAGACTTACTCTTGATTGTAGCAAGGATTGTGGGACTTCTGGGACGCAGG---------------GGGTGGGAACTCCTCAAATATTGGTGG---AATCTCCTCCAGTATTGG---------------------------------------------------AGTCAGGAACTAAAGAATAGTGCTGTTAGCTTGCTGAATGCCACAGCTATAGCAGTAGCTGAGGGGACAGATAGGGTTATAGACATAGTACAAAGA------------------ATTTGCAGAGCTATCCTCCACATACCTAGAAGAATAAGACAGGGCTTTGAAAGGGCTTTGCTATAA

2.1056.SPD.EU575315 ATG---------GAGATCAGGAAGAATTATCAGCACTTG---------TGGAGATGGGGG------------------------ATCATGCTCCTTTGGTTATTAATGAGC------------TGTAGTGCTGAA---------GAAGAAGCGTGGGTCACAGTTTATTATGGGGTACCTGTGTGGAAAGAAGCAGTCACCACTCTATTTTGTGCATCAGACGCAAAAGCATATGATACAGAGGTATATAAT---GTTTGGACCACACATGCCTGTGTACCCACAGACCCCGACCCACAAGAAGTACACATG---GAAAATGTGACAGAAGATTTTAACATGTGGAAAAATAACATGGCAGATCAGATGCATGAGGATATAATCAGTTTATGGGATCAAAGTCTAAAGCCATGTGTAAAATTAACCCCACTCTGTGTTACTTTAAATTGTGCTGATTGGAAGAATAATACTGATACCAATACC---------------------------------------------------------------------------------------AATAGTAGTGTGAGAATAATGGAGAAAGGAGAAATAAAAAACTGCTCTTTCAATATCACC---ACAAACATAAGAGAT------AAGTATCAGAAAGCATATGCACTTTTTTATAAACTTGATGTAGTACCAATAGATGATGAT------------------------------AATGCAACA------GGTAATAATGATACTAGAAACTATAGGTTGATAAGTTGTAACACCTCAGTCATTACACAGGCCTGTCCAAAGGTATCCTTTGAACCAATTCCCATACATTATTGTGCCCCGGCTGGTTTTGCGATTTTAAGGTGT---AATAATAAGACATTCAGTGGAAAAGGACAATGTACAAATGTCAGCACAGTACAATGTACACATGGAATTAAGCCAGTAGTATCAACTCAACTGCTATTAAATGGCAGTCTAGCAGAAGAA---GAGGTAATAATTAGATCTGACAATTTCTCGGACAATGCTAAAACCATAATAGTACATCTAAACAGCTCTGTAGACATTAATTGTACAAGACCAGGCAACAATACAAGAAAAAGTATAACTATAGGA------------CCAGGGAGG---GCATTTTATGCAACAGGAGACATAATAGGAGATATAAGACAAGCACATTGTAACATT------AGTGGAGAAAAATGGAATAACACTTTAAAACAGGTAGTTAAAAAATTAAGA---GAACAATTTGGG------------AATAAAACA---ATAGTCTTTAATCAA---------TCCTCAGGAGGGGACCCAGAAATTACAATGCACACTTTTAATTGTGGAGGGGAATTCTTCTACTGTAATACAGCACAACTGTTTAATAGTACTTGGGAAGCT---------AATAGTACTTGGGAAAATGAT---------------------AATGAAAGGGTAGGTCAC------------AGTAACAAGACT---------------------------ATCATACTACAATGCAGAATAAAACAAATTATAAACATGTGGCAGGAAGTAGGAAAAGCAATGTATGCCCCTCCCATCAGCGGACAGATTAGATGTTCATCAAATATTACGGGGCTGCTATTAACAGGAGATGGTGGTAACGGT------------AACGAGACC---------------------AACCGGACC---GAGGTCTTCAGACCTGGAGGAGGAAATATGAAAGATAACTGGAGA---AGTGAATTATATAAATATAAAGTAGTAAAAATTGAACCA---TTAGGAGTAGCACCC---ACCAGGGCAAAGAGAAGAGTGGTGCAAAGAGAA---AAAAGAGCAGTG---GGA---ATGGGA---GCTTTG---TTCATT---GGG---------TTCTTGTCA---GCAGCAGGAAGCACTATGGGCGCAGCGTCAATG---ACGCTGACGGTACAGGCCAGACAATTATTGTCTGGTATAGTGCAACAGCAGAGCAATTTGCTGAGAGCTATTGAGGCGCAACAACATCTGTTGCAACTCACAGTCTGGGGCATCAAGCAGCTCCAGGCAAGA---GTCCTGGCTGTGGAAAGATACCTAAAGGATCAACAGCTCCTAGGGATTTGGGGTTGCTCTGGAAAACTCATTTGCACCACTACAGTGCCTTGGAATTATAGTTGGAGTCCT------------------------AATAAAACTATGGATGACATTTGGGGT---AACATGACCTGGATGCAATGGGAAAGAGAAATTGAC------AATTATACAGGCATAATATACAGATTAATTGAAATATCGCAAAACCAGCAAGAAAAGAATGAACAAGAATTATTGGAATTAGATAAATGGGCAAGTTTGTGGAATTGGTTTGACATAACAAAGTGGCTGTGGTATATAAAAATATTCATAATGATAATAGGAGGCTTAGTAGGTTTAAGAATAGTCTTTACTGTGCTTTCTATAGTAAATAGAGTTAGGCAGGGATACTCACCATTATCGTTTCAGACCCGC---TTCCCAGCCCCAGGGGGA------CCCGACAGGCCCGAAGGAACAGAAGAAGAAGGTGGAGAGAGAGACAGAGACAGATCCAGTCGATCAGCGGATGGATTCTTAGCAATTATCTGGGTCGATCTGAGGAGCCTGTGCCTGTTCATCTACCACAGCTTGAGAGACTTACTCTTGATTGTAGCAAGGATTGTGGGACTTCTGGGACGCAGG---------------GGGTGGGAACTCCTCAAATATTGGTGG---AATCTCCTCCAGTATTGG---------------------------------------------------AGTCAGGAACTAAAGAATAGTGCTGTTAGCTTGCTGAATGCCACAGCTATAGCAGTAGCTGAGGGGACAGATAGGGTTATAGACATAGTACAAAGA------------------ATTTGCAGAGCTATCCTCCACATACCTAGAAGAATAAGACAGGGCTTTGAAAGGGCTTTGCTATAA

2.1056.SPD.EU575311 ATG---------GAGATCAGGAAGAATTATCAGCACTTG---------TGGAGATGGGGG------------------------ATCATGCTCCTTTGGTTATTAATGAGC------------TGTAGTGCTGAA---------GAAGAAGCGTGGGTCACAGTTTATTATGGGGTACCTGTGTGGAAAGAAGCAGTCACCACTCTATTTTGTGCATCAGACGCAAAAGCATATGATACAGAGGTACATAAT---GTTTGGACCACACATGCCTGTGTACCCACAGACCCCGACCCACAAGAAGTACACATG---GAAAATGTGACAGAAGATTTTAACATGTGGAAAAATAACATGGCAGATCAGATGCATGAGGATATAATCAGTTTATGGGATCAAAGTCTAAAGCCATGTGTAAAATTAACCCCACTCTGTGTTACTTTAAATTGTGCTGATTGGAAGAATAATACTGATACCAATACC---------------------------------------------------------------------------------------AATAGTAGTGTGAGAATAATGGAGAAAGGAGAAATAAAAAACTGCTCTTTCAATATCACC---ACAAACATAAGAGAT------AAGTATCAGAAAGCATATGCACTTTTTTATAAACTTGATGTAGTACCAATAGATGATGAT------------------------------AATGCAACA------GGTAATAATGATACTAGAAACTATAGGTTGATAAGTTGTAACACCTCAGTCATTACACAGGCCTGTCCAAAGGTATCCTTTGAACCAATTCCCATACATTATTGTGCCCCGGCTGGTTTTGCGATTTTAAGGTGT---AATAATAAGACATTCAGTGGAAAAGGACAATGTACAAATGTCAGCACAGTACAATGTACACATGGAATTAAGCCAGTAGTATCAACTCAACTGCTATTAAATGGCAGTCTAGCAGAAGAA---GAGGTAATAATTAGATCTGACAATTTCTCGGACAATGCTAAAACCATAATAGTACATCTAAACAGCTCTGTAGACATTAATTGTACAAGACCAGGCAACAATACAAGAAAAAGTATAACTATAGGA------------CCAGGGAGG---GCATTTTATGCAACAGGAGACATAATAGGAGATATAAGACAAGCACATTGTAACATT------AGTGGAGAAAAATGGAATAACACTTTAAAACAGGTAGTTAAAAAATTAAGA---GAACAATTTGGG------------AATAAAACA---ATAGTCTTTAATCAA---------TCCTCAGGAGGGGACCCAGAAATTACAATGCACACTTTTAATTGTGGAGGGGAATTCTTCTACTGTAATACAGCACAACTGTTTAATAGTACTTGGGAAGCT---------AATAGTACTTGGGAAAATGAT---------------------AATGAAAGGGTAGGTCAC------------AGTAACAAGACT---------------------------ATCATACTACAATGCAGAATAAAACAAATTATAAACATGTGGCAGGAAGTAGGAAAAGCAATGTATGCCCCTCCCATCAGCGGACAAATTAGATGTTCATCAAATATTACGGGGCTGCTATTAACAAGAGATGGTGGTAACGGT------------AACGAGACC---------------------AACCGGACC---GAGGTCTTCAGACCTGGAGGAGGAAATATGAAAGATAACTGGAGA---AGTGAATTATATAAATATAAAGTAGTAAAAATTGAACCA---TTAGGAGTAGCACCC---ACCAGGGCAAAGAGAAGAGTGGTGCAAAGAGAA---AAAAGAGCAGTG---GGA---ATGGGA---GCTTTG---TTCATT---GGG---------TTCTTGTCA---GCAGCAGGAAGCACTATGGGCGCAGCGTCAATG---ACGCTGACGGTACAGGCCAGACAATTATTGTCTGGTATAGTGCAACAGCAGAGCAATTTGCTGAGAGCTATTGAGGCGCAACAACATCTGTTGCAACTCACAGTCTGGGGCATCAAGCAGCTCCAGGCAAGA---GTCCTGGCTGTGGAAAGATACCTAAAGGATCAACAGCTCCTAGGGATTTGGGGTTGCTCTGGAAAACTCATTTGCACCACTACAGTGCCTTGGAATTATAGTTGGAGTCCT------------------------AATAAAACTATGGATGACATTTGGGGT---AACATGACCTGGATGCAATGGGAAAGAGAAATTGAC------AATTATACAGGCATAATATACAGATTAATTGAAATATCGCAAAACCAGCAAGAAAAGAATGAACAAGAATTATTGGAATTAGATAAATGGGCAAGTTTGTGGAATTGGTTTGACATAACAAAGTGGCTGTGGTATATAAAAATATTCATAATGATAATAGGAGGCTTAGTAGGTTTAAGAATAGTCTTTACTGTGCTTTCTATAGTAAATAGAGTTAGGCAGGGATACTCACCATTATCGTTTCAGACCCGC---TTCCCAGCCCCAGGGGGA------CCCGACAGGCCCGAAGGAACAGAAGAAGAAGGTGGAGAGAGAGACAGAGACAGATCCAGTCGATCAGCGGATGGATTCTTAGCAATTATCTGGGTCGATCTGAGGAGCCTGTGCCTGTTCATCTACCACAGCTTGAGAGACTTACTCTTGATTGTAGCAAGGATTGTGGGACTTCTGGGACGCAGG---------------GGGTGGGAACTCCTCAAATATTGGTGG---AATCTCCTCCAGTATTGG---------------------------------------------------AGTCAGGAACTAAAGAATAGTGCTGTTAGCTTGCTGAATGCCACAGCTATAGCAGTAGCTGAGGGGACAGATAGGGTTATAGACATAGTACAAAGA------------------ATTTGCAGAGCTATCCTCCACATACCTAGAAGAATAAGACAGGGCTTTGAAAGGGCTTTGCTATAA

2.1056.SPD.EU575301 ATG---------GAGATCAGGAAGAATTATCAGCACTTG---------TGGAGATGGGGG------------------------ATCATGCTCCTTTGGTTATTAATGAGC------------TGTAGTGCTGAA---------GAAGAAGCGTGGGTCACAGTTTATTATGGGGTACCTGTGTGGAAAGAAGCAGTCACCACTCTATTTTGTGCATCAGACGCAAAAGCATATGATACAGAGGTACATAAT---GTTTGGACCACACATGCCTGTGTACCCACAGACCCCGACCCACAAGAAGTACACATG---GAAAATGTGACAGAAGATTTTAACATGTGGAAAAATAACATGGCAGATCAGATGCATGAGGATATAATCAGTTTATGGGATCAAAGTCTAAAGCCATGTGTAAAATTAACCCCACTCTGTGTTACTTTAAATTGTGCTGATTGGAAGAATAATACTGATACCAATACC---------------------------------------------------------------------------------------AATAGTAGTGTGAGAATAATGGAGAAAGGAGAAATAAAAAACTGCTCTTTCAATATCACC---ACAAACATAAGAGAT------AAGTATCAGAAAGCATATGCACTTTTTTATAAACTTGATGTAGTACCAATAGATGATGAT------------------------------AATGCAACA------GGTAATAATGATACTAGAAACTATAGGTTGATAAGTTGTAACACCTCAGTCATTACACAGGCCTGTCCAAAGGTATCCTTTGAACCAATTCCCATACATTATTGTGCCCCGGCTGGTTTTGCGATTTTAAGGTGT---AATAATAAGACATTCAGTGGAAAAGGACAATGTACAAATGTCAGCACAGTACAATGTACACATGGAATTAAGCCAGTAGTATCAACTCAACTGCTATTAAATGGCAGTCTAGCAGAAGAA---GAGGTAATAATTAGATCTGACAATTTCTCGGACAATGCTAAAACCATAATAGTACATCTAAACAGCTCTGTAGACATTAATTGTACAAGACCAGGCAACAATACAAGAAAAAGTATAACTATAGGA------------CCAGGGAGG---GCATTTTATGCAACAGGAGACATAATAGGAGATATAAGACAAGCACATTGTAACATT------AGTGGAGAAAAATGGAATAACACTTTAAAACAGGTAGTTAAAAAATTAAGA---GAACAATTTGGG------------AATAAAACA---ATAGTCTTTAATCAA---------TCCTCAGGAGGGGACCCAGAAATTACAATGCACACTTTTAATTGTGGAGGGGAATTCTTCTACTGTAATACAGCACAACTGTTTAATAGTACTTGGGAAGCT---------AATAGTACTTGGGAAAATGAT---------------------AATGAAAGGGTAGGTCAC------------AGTAACAAGACT---------------------------ATCATACTACAATGCAGAATAAAACAAATTATAAACATGTGGCAGGAAGTAGGAAAAGCAATGTATGCCCCTCCCATCAGCGGACAGATTAGATGTTCATCAAATATTACGGGGCTGCTATTAACAAGAGATGGTGGTAACGGT------------AACGAGACC---------------------AACCGGACC---GAGGTCTTCAGACCTGGAGGAGGAAATATGAAAGATAACTGGAGA---AGTGAATTATATAAATATAAAGTAGTAAAAATTGAACCA---TTAGGAGTAGCACCC---ACCAGGGCAAAGAGAAGAGTGGTGCAAAGAGAA---AAAAGAGCAGTG---GGA---ATGGGA---GCTTTG---TTCATT---GGG---------TTCTTGTCA---GCAGCAGGAAGCACTATGGGCGCAGCGTCAATG---ACGCTGACGGTACAGGCCAGACAATTATTGTCTGGTATAGTGCAACAGCAGAGCAATTTGCTGAGAGCTATTGAGGCGCAACAACATCTGTTGCAACTCACAGTCTGGGGCATCAAGCAGCTCCAGGCAAGA---GTCCTGGCTGTGGAAAGATACCTAAAGGATCAACAGCTCCTAGGGATTTGGGGTTGCTCTGGAAAACTCATTTGCACCACTACAGTGCCTTGGAATTATAGTTGGAGTCCT------------------------AATAAAACTATGGATGACATTTGGGGT---AACATGACCTGGATGCAATGGGAAAGAGAAATTGAC------AATTATACAGGCATAATATACAGATTAATTGAAATATCGCAAAACCAGCAAGAAAAGAATGAACAAGAATTATTGGAATTAGATAAATGGGCAAGTTTGTGGAATTGGTTTGACATAACAAAGTGGCTGTGGTATATAAAAATATTCATAATGATAATAGGAGGCTTAGTAGGTTTAAGAATAGTCTTTACTGTGCTTTCTATAGTAAATAGAGTTAGGCAGGGATACTCACCATTATCGTTTCAGACCCGC---TTCCCAGCCCCAGGGGGA------CCCGACAGGCCCGAAGGAACAGAAGAAGAAGGTGGAGAGAGAGACAGAGACAGATCCAGTCGATCAGCGGATGGATTCTTAGCAATTATCTGGGTCGATCTGAGGAGCCTGTGCCTGTTCATCTACCACAGCTTGAGAGACTTACTCTTGATTGTAGCAAGGATTGTGGGACTTCTGGGACGCAGG---------------GGGTGGGAACTCCTCAAATATTGGTGG---AATCTCCTCCAGTATTGG---------------------------------------------------AGTCAGGAACTAAAGAATAGTGCTGTTAGCTTGCTGAATGCCACAGCTATAGCAGTAGCTGAGGGGACAGATAGGGTTATAGACATAGTACAAAGA------------------ATTTGCAGAGCTATCCTCCACATACCTAGAAGAATAAGACAGGGCTTTGAAAGGGCTTTGCTATAA

2.1056.SPD.EU575313 ATG---------GAGATCAGGAAGAATTATCAGCACTTG---------TGGAGATGGGGG------------------------ATCATGCTCCTTTGGTTATTAATGAGC------------TGTAGTGCTGAA---------GAAGAAGCGTGGGTCACAGTTTATTATGGGGTACCTGTGTGGAAAGAAGCAGTCACCACTCTATTTTGTGCATCAGACGCAAAAGCATATGATACAGAGGTACATAAT---GTTTGGACCACACATGCCTGTGTACCCACAGACCCCGACCCACAAGAAGTACACATG---GAAAATGTGACAGAAGATTTTAACATGTGGAAAAATAACATGGCAGATCAGATGCATGAGGATATAATCAGTTTATGGGATCAAAGTCTAAAGCCATGTGTAAAATTAACCCCACTCTGTGTTACTTTAAATTGTGCTGATTGGAAGAATAATACTGATACCAATACC---------------------------------------------------------------------------------------AATAGTAGTGTGAGAATAATGGAGAAAGGAGAAATAAAAAACTGCTCTTTCAATATCACC---ACAAACATAAGAGAT------AAGTATCAGAAAGCATATGCACTTTTTTATAAACTTGATGTAGTACCAATAGATGATGAT------------------------------AATGCAACA------GGTAATAATGATACTAGAAACTATAGGTTGATAAGTTGTAACACCTCAGTCATTACACAGGCCTGTCCAAAGGTATCCTTTGAACCAATTCCCATACATTATTGTGCCCCGGCTGGTTTTGCGATTTTAAGGTGT---AATAATAAGACATTCAGTGGAAAAGGACAATGTACAAATGTCAGCACAGTACAATGTACACATGGAATTAAGCCAGTAGTATCAACTCAACTGCTATTAAATGGCAGTCTAGCAGAAGAA---GAGGTAATAATTAGATCTGACAATTTCTCGGACAATGCTAAAACCATAATAGTACATCTAAACAGCTCTGTAGACATTAATTGTACAAGACCAGGCAACAATACAAGAAAAAGTATAACTATAGGA------------CCAGGGAGG---GCATTTTATGCAACAGGAGACATAATAGGAGATATAAGACAAGCACATTGTAACATT------AGTGGAGAAAAATGGAATAACACTTTAAAACAGGTAGTTAAAAAATTAAGA---GAACAATTTGGG------------AATAAAACA---ATAGTCTTTAATCAA---------TCCTCAGGAGGGGACCCAGAAATTACAATGCACACTTTTAATTGTGGAGGGGAATTCTTCTACTGTAATACAGCACAACTGTTTAATAGTACTTGGGAAGCT---------AATAGTACTTGGGAAAATGAT---------------------AATGAAAGGGTAGGTCAC------------AGTAACAAGACT---------------------------ATCATACTACAATGCAGAATAAAACAAATTATAAACATGTGGCAGGAAGTAGGAAAAGCAATGTATGCCCCTCCCATCAGCGGACAGATTAGATGTTCATCAAATATTACGGGGCTGCTATTAACAAGAGATGGTGGTAACGGTGGT---------AACGAGACC---------------------AACCGGACC---GAGGTCTTCAGACCTGGAGGAGGAAATATGAAAGATAACTGGAGA---AGTGAATTATATAAATATAAAGTAGTAAAAATTGAACCA---TTAGGAGTAGCACCC---ACCAGGGCAAAGAGAAGAGTGGTGCAAAGAGAA---AAAAGAGCAGTG---GGA---ATGGGA---GCTTTG---TTCATT---GGG---------TTCTTGTCA---GCAGCAGGAAGCACTATGGGCGCAGCGTCAATG---ACGCTGACGGTACAGGCCAGACAATTATTGTCTGGTATAGTGCAACAGCAGAGCAATTTGCTGAGAGCTATTGAGGCGCAACAACATCTGTTGCAACTCACAGTCTGGGGCATCAAGCAGCTCCAGGCAAGA---GTCCTGGCTGTGGAAAGATACCTAAAGGATCAACAGCTCCTAGGGATTTGGGGTTGCTCTGGAAAACTCATTTGCACCACTACAGTGCCTTGGAATTATAGTTGGAGTCCT------------------------AATAAAACTATGGATGACATTTGGGGT---AACATGACCTGGATGCAATGGGAAAGAGAAATTGAC------AATTATACAGGCATAATATACAGATTAATTGAAATATCGCAAAACCAGCAAGAAAAGAATGAACAAGAATTATTGGAATTAGATAAATGGGCAAGTTTGTGGAATTGGTTTGACATAACAAAGTGGCTGTGGTATATAAAAATATTCATAATGATAATAGGAGGCTTAGTAGGTTTAAGAATAGTCTTTACTGTGCTTTCTATAGTAAATAGAGTTAGGCAGGGATACTCACCATTATCGTTTCAGACCCGC---TTCCCAGCCCCAGGGGGA------CCCGACAGGCCCGAAGGAACAGAAGAAGAAGGTGGAGAGAGAGACAGAGACAGATCCAGTCGATCAGCGGATGGATTCTTAGCAATTATCTGGGTCGATCTGAGGAGCCTGTGCCTGTTCATCTACCACAGCTTGAGAGACTTACTCTTGATTGTAGCAAGGATTGTGGGACTTCTGGGACGCAGG---------------GGGTGGGAACTCCTCAAATATTGGTGG---AATCTCCTCCAGTATTGG---------------------------------------------------AGTCAGGAACTAAAGAATAGTGCTGTTAGCTTGCTGAATGCCACAGCTATAGCAGTAGCTGAGGGGACAGATAGGGTTATAGACATAGTACAAAGA------------------ATTTGCAGAGCTATCCTCCACATACCTAGAAGAATAAGACAGGGCTTTGAAAGGGCTTTGCTATAA

2.1056.SPD.EU575302 ATG---------GAGATCAGGAAGAATTATCAGCACTTG---------TGGAGATGGGGG------------------------ATCATGCTCCTTTGGTTATTAATGAGC------------TGTAGTGCTGAA---------GAAGAAGCGTGGGTCACAGTTTATTATGGGGTACCTGTGTGGAAAGAAGCAGTCACCACTCTATTTTGTGCATCAGACGCAAAAGCATATGATACAGAGGTACATAAT---GTTTGGACCACACATGCCTGTGTACCCACAGACCCCGACCCACAAGAAGTACACATG---GAAAATGTGACAGAAGATTTTAACATGTGGAAAAATAACATGGCAGATCAGATGCATGAGGATATAATCAGTTTATGGGATCAAAGTCTAAAGCCATGTGTAAAATTAACCCCACTCTGTGTTACTTTAAATTGTGCTGATTGGAAGAATAATACTGATACCAATACC---------------------------------------------------------------------------------------AATAGTAGTGTGAGAATAATGGAGAAAGGAGAAATAAAAAACTGCTCTTTCAATATCACC---ACAAACATAAGAGAT------AAGTATCAGAAAGCATATGCACTTTTTTATAAACTTGATGTAGTACCAATAGATGATGAT------------------------------AATGCAACA------GGTAATAATGATACTAGAAACTATAGGTTGATAAGTTGTAACACCTCAGTCATTACACAGGCCTGTCCAAAGGTATCCTTTGAACCAATTCCCATACATTATTGTGCCCCGGCTGGTTTTGCGATTTTAAGGTGT---AATAATAAGACATTCAGTGGAAAAGGACAATGTACAAATGTCAGCACAGTACAATGTACACATGGAATTAAGCCAGTAGTATCAACTCAACTGCTATTAAATGGCAGTCTAGCAGAAGAA---GAGGTAATAATTAGATCTGACAATTTCTCGGACAATGCTAAAACCATAATAGTACATCTAAACAGCTCTGTAGACATTAATTGTACAAGACCAGGCAACAATACAAGAAAAAGTATAACTATAGGA------------CCAGGGAGG---GCATTTTATGCAACAGGAGACATAATAGGAGATATAAGACAAGCACATTGTAACATT------AGTGGAGAAAAATGGAATAACACTTTAAAACAGGTAGTTAAAAAATTAAGA---GAACAATTTGGG------------AATAAAACA---ATAGTCTTTAATCAA---------TCCTCAGGAGGGGACCCAGAAATTACAATGCACACTTTTAATTGTGGAGGGGAATTCTTCTACTGTAATACAGCACAACTGTTTAATAGTACTTGGGAAGCT---------AATAGTACTTGGGAAAATGAT---------------------AATGAAAGGGTAGGTCAC------------AGTAACAAGACT---------------------------ATCATACTACAATGCAGAATAAAACAAATTATAAACATGTGGCAGGAAGTAGGAAAAGCAATGTATGCCCCTCCCATCAGTGGACAGATTAGATGTTCATCAAATATTACGGGGCTGCTATTAACAAGAGATGGTGGTAACGGT------------AACGAGACC---------------------AACCGGACC---GAGGTCTTCAGACCTGGAGGAGGAAATATGAAAGATAACTGGAGA---AGTGAATTATATAAATATAAAGTAGTAAAAATTGAACCA---TTAGGAGTAGCACCC---ACCAGGGCAAAGAGAAGAGTGGTGCAAAGAGAA---AAAAGAGCAGTG---GGA---ATGGGA---GCTTTG---TTCATT---GGG---------TTCTTGTCA---GCAGCAGGAAGCACTATGGGCGCAGCGTCAATG---ACGCTGACGGTACAGGCCAGACAATTATTGTCTGGTATAGTGCAACAGCAGAGCAATTTGCTGAGAGCTATTGAGGCGCAACAACATCTGTTGCAACTCACAGTCTGGGGCATCAAGCAGCTCCAGGCAAGA---GTCCTGGCTGTGGAAAGATACCTAAAGGATCAACAGCTCCTAGGGATTTGGGGTTGCTCTGGAAAACTCATTTGCACCACTACAGTGCCTTGGAATTATAGTTGGAGTCCT------------------------AATAAAACTATGGATGACATTTGGGGT---AACATGACCTGGATGCAATGGGAAAGAGAAATTGAC------AATTATACAGGCATAATATACAGATTAATTGAAATATCGCAAAACCAGCAAGAAAAGAATGAACAAGAATTATTGGAATTAGATAAATGGGCAAGTTTGTGGAATTGGTTTGACATAACAAAGTGGCTGTGGTATATAAAAATATTCATAATGATAATAGGAGGCTTAGTAGGTTTAAGAATAGTCTTTACTGTGCTTTTTATAGTAAATAGAGTTAGGCAGGGATACTCACCATTATCGTTTCAGACCCGC---TTCCCAGCCCCAGGGGGA------CCCGACAGGCCCGAAGGAACAGAAGAAGAAGGTGGAGAGAGAGACAGAGACAGATCCAGTCGATCAGCGGATGGATTCTTAGCAATTATCTGGGTCGATCTGAGGAGCCTGTGCCTGTTCATCTACCACAGCTTGAGAGACTTACTCTTGATTGTAGCAAGGATTGTGGGACTTCTGGGACGCAGG---------------GGGTGGGAACTCCTCAAATATTGGTGG---AATCTCCTCCAGTATTGG---------------------------------------------------AGTCAGGAACTAAAGAATAGTGCTGTTAGCTTGCTGAATGCCACAGCTATAGCAGTAGCTGAGGGGACAGATAGGGTTATAGACATAGTACAAAGA------------------ATTTGCAGAGCTATCCTCCACATACCTAGAAGAATAAGACAGGGCTTTGAAAGGGCTTTGCTATAA

2.12008.SPD.EU575462 ATGAGAGTGATGGAGATCAGGAGGAACTATCAGCTCTTG---------TGGAAAGGGGGC------------------------ATCTTGCTCCTTGGGATGTTAATGATC------------TGTAATACTTCA---------GAAAAATTGTGGGTCACAGTCTATTATGGGGTACCTGTGTGGAAAGAAGCAACCACCACTCTATTTTGTGCCTCAGATGCTAAAGGACATGAGACAGAGGTACATAAT---GTCTGGGCCACACATGCCTGTGTACCCACAGACCCCAACCCACAAGAAATAGTATTA---AGAAATGTGACAGAAGAGTTTAACATATGGAAAAATAACATGGTAAAACAGATGCATGAGGATATAATCAGTTTATGGGATCAAAGCCTAAAGCCATGTGTAAAATTAACCCCACTCTGTGTCACTTTAAAGTGCACTGATGATTGGAATACTACTAAAATCATTGAGAATCGGAGTGCTACTGGAACCAATGTGACGAGT---------GCTCCTACTAGTACCCCTGTGACAACTGAGACTACAACCCGTAATAGTAGTGAGGGGAAATTAATGGAGATAAAAGAAATGAAAAACTGCTCTTTCAAGGTCACC---TCAAACGTAAGAGAT------AAGGTGCAGGAAGAATATGCACTTTTTTATAAATTTGATATAATACCAATAGGCAATGAT------------------------------AATAATACT------------------AATACCAGTTATAGGTTGATAAATTGCAACACCTCAGTCATTACACAAGCCTGTCCAAAAGTATCCTTTGAGCCAATTCCCATACATTATTGTGCCCCGGCTGGTTTTGCGATTCTAAAGTGT---AATGATAAGGAGTTCAATGGAACAGGACTGTGTACAAATGTTAGCACAGTACAATGTACACATGGAATTAGGCCAGTAGTGTCAACACAACTACTGTTAAATGGCAGTCTAGCAGAAGAA---AAGATAGTAATTAGATCTGAAAACATCACGAACAATGCTAAAACCATAATAGTACATCTGAACGAACCTATAAAAATTAATTGTACAAGACCCAGCAACAATACAAGAAAAAGTATACCTATAGGA------------CCAGGGAGA---GCATTTTATGCAACAGGAGAAATAATAGGAGATATAAGACAAGCACATTGTAACATT------AGTCAAAAAGATTGGAATACCACCTTAGAACGGGTAGTTGAAACATTAAGA---AGAACATTTGGG------------AATAAAACA---ATAATATTTGATCGA---------TCCTCAGGAGGGGACCCAGAAATTACAATGCACAGTTTTAATTGTGGAGGGGAATTTTTCTACTGTAATACATCATCACTGTTTAATAGTACTTGGGGCAGT---------AATGGTACTTGGAAGGGTACA---------------------AATGGCACTAGAGACGAA---------------------------------------------------ATCACACTCCAATGCAGAATAAAACAAATTATAAACATGTGGCAGGAAGTAGGAAAAGCAATGTATGCCCCTCCCATCAGAGGAAATATTAGCTGCTCATCAAATATTACTGGGCTGCTATTAACAAGAGATGGTGGTACTGGCAAG---------AACACTGCCCAG------------------------AAC---GAGACCTTCAGACCTGGAGGAGGAAATATGAAAGACAATTGGAGA---AATGAGTTATATAAATATAAAGTAGTAAAAATTAAACCA---TTAGGAGTAGCACCC---ACCAAGGCAAGGAGAAGAGTGGTGCAGAGAGAA---AAAAGAGCAGTG---GGA---ATAGGA---GCTGTG---TTCCTT---GGG---------TTCTTGTCA---GCAGCAGGAAGCACTATGGGCGCAGCGTCAATG---ACGCTGACGGTACAGGCCAGACAATTATTGTCTGGTATAGTGCAACAGCAAAGCAACTTGCTGAGGGCTATTGAGGCGCAACAGCATCTGTTGCAACTCACAGTCTGGGGCATCAAGCAGCTCCAGGCAAGA---GTCTTGGCTGTGAAAAGATACCTAAAGGATCAACAGCTCCTAGGGATTTGGGGTTGCTCTGAAAAACGCATCTGCACCACTAATGTGCCTTGGAATACTAGTTGGAGT---------------------------AATAAATCTCTGAATGATATTTGGGAT---AACATGACCTGGATGCAGTGGGAAAAAGAAATTGAC------AATTACACAAGCATAATATACACTTTAATTGAAGAATCGCAGAACCAGCAAGAAAAGAATGAACAGGAGTTATTGAAATTAGATAAATGGGCAAATTTGTGGAATTGGTTTTCCATAACACAATGGCTGTGGTATATAAAAATATTCATAATGATAGTAGGAGGCTTGATAGGTTTAAGAATAGTTTTTACTGTGCTTTCTATAGTGAATAGAGTTAGGCAGGGATACTCACCATTATCGTTTCAGACCCAC---CTCCCAGCCCAGAGGGGA------CACGACAGGCCCGAAGGAATCGAAGAAGAAGGTGGAGAGAAAGACAGAGACAGATCCGGAAGATTAGTGGATGGATTGTTGACAATTATCTGGGTCGACCTACGGAGCCTGTGCCTCTTCAGCTACCACCGCTTGAGAGACTTACTCTTGATTGTAACGAGGATTGTGGAACTTCTGGGACGCAGG---------------GGGTGGGAAGCCCTCAAATATTGGTGG---AATCTCCTTCAGTATTGG---------------------------------------------------GGTCAGGAACTAAAGAATAGTGCTGTTAGCTTGCTCAATGTCACAGCTATAGCAGTAGCTGAGGGAACAGATAGGATTATAGAAGTAGTACAAAGA------------------GCTTGTAGAGCTATTCGCAACATACCTGTAAGAATCAGACAGGGCTTGGAAAGGCTTTTGCTATAA

2.12008.SPD.EU575441 ATGAGAGTGATGGAGATCAGGAGGAACTATCAGCTCTTG---------TGGAAAGGGGGC------------------------ATCTTGCTCCTTGGGATGTTAATGATC------------TGTAATACTTCA---------GAAAAATTGTGGGTCACAGTCTATTATGGGGTACCTGTGTGGAAAGAAGCAACCACCACTCTATTTTGTGCCTCAGATGCTAAAGGACATGAGACAGAGGTACATAAT---GTCTGGGCCACACATGCCTGTGTACCCACAGACCCCAACCCACAAGAAATAGTATTG---AGAAATGTGACAGAAGAGTTTAACATATGGAAAAATAACATGGTAGAACAGATGCATGAGGATATAATCAGTTTATGGGATCAAAGCCTAAAGCCATGTGTAAAATTAACCCCACTCTGTGTCACTTTAAAGTGCACTGATGATTGGAATACTACTAAAATCATTGAGAATCGGAGTGCTACTGGAACCAATGTGACGAGT---------GCTCCTACTAGTACCCCTGTGACAACTGAGACTACAACCCGTAATAGTAGTGAGGGGAGATTAATGGAGATAAAAGAAATGAAAAACTGCTCTTTCAAGGTCACC---TCAAACGTAAGAGAT------AAGGTGCAGGAAGAATATGCACTTTTTTATAAATTTGATATAATACCAATAGGCAATGAT------------------------------AATAATACT------------------AATACCAGTTATAGGTTGATAAATTGCAACACCTCAGTCATTACACAAGCCTGTCCAAAAGTATCCTTTGAGCCAATTCCCATACATTATTGTGCCCCGGCTGGTTTTGCGATTCTAAAGTGT---AATGATAAGGAGTTCAATGGAACAGGACTGTGTACAAATGTTAGCACAGTACAATGTACACATGGAATTAGGCCAGTAGTGTCAACACAACTACTGTTAAATGGCAGTCTAGCAGAAGAA---AAGATAGTAATTAGATCTGAAAACATCACGAACAATGCTAAAACCATAATAGTACATCTGAACGAACCTATAAAAATTAATTGTACAAGACCCAGCAACAATACAAGAAAAAGTATACCTATAGGA------------CCAGGGAGA---GCATTTTATGCAACAGGAGAAATAATAGGAGATATAAGACAAGCACATTGTAACATT------AGTCAAAAAGATTGGAATACCACCTTAGAACGGGTAGTTGAAACATTAAGA---AGAACATTTGGG------------AATAAAACA---ATAATATTTGATCGA---------TCCTCAGGAGGGGACCCAGAAATTACAATGCACAGTTTTAATTGTGGAGGGGAATTTTTCTACTGTAATACATCATCACTGTTTAATAGTACTTGGGGCAGT---------AATGGTACTTGGAAGGGTACA---------------------AATGGCACTAGAGACGAA---------------------------------------------------ATCACACTCCAATGCAGAATAAAACAAATTATAAACATGTGGCAGGAAGTAGGAAAAGCAATGTATGCCCCTCCCATCAGAGGAAATATTAGCTGCTCATCAAATATTACTGGGCTGCTATTAACAAGAGATGGTGGTACTGGCAAG---------AACACTGCCCAG------------------------AAC---GAGACCTTCAGACCTGGAGGAGGAAATATGAAAGACAATTGGAGA---AATGAGTTATATAAATATAAAGTAGTAAAAATTGAACCA---TTAGGAGTAGCACCC---ACCAAGGCAAGGAGAAGAGTGGTGCAGAGAGAAA--AAAAGAGCAGTG---GGA---ATAGGA---GCTGTG---TTCCTT---GGG---------TTCTTGTCA---GCAGCAGGAAGCACTATGGGCGCAGCGTCAATG---ACGCTGACGGTACAGGCCAGACAATTATTGTCTGGTATAGTGCAACAGCAAAGCAACTTGCTGAGGGCTATTGAGGCGCAACAGCATCTGTTGCAACTCACAGTCTGGGGCATCAAGCAGCTCCAGGCAAGA---GTCTTGGCTGTGGAAAGATACCTAAAGGATCAACAGCTCCTAGGGATTTGGGGTTGCTCTGGAAAACGCATCTGCACCACTAATGTGCCTTGGAATACTAGTTGGAGT---------------------------AATAAATCTCTGAATGATATTTGGGAT---AACATGACCTGGATGCAGTGGGAAAAAGAAATTGAC------AATTACACAAGCATAATATACACTTTAATTGAAGAATCGCAGAACCAGCAAGAAAAGAATGAACAGGAGTTATTGGAATTAGATAAATGGGCAAATTTGTGGAATTGGTTTTCCATAACACAATGGCTGTGGTATATAAAAATATTCATAATGATAGTAGGAGGCTTGATAGGTTTAAGAATAGTTTTTACTGTGCTTTCTATAGTGAATAGAGTTAGGCAGGGATACTCACCATTATCGTTTCAGACCCAC---CTCCCAGCCCAGAGGGAA------CACGACAGGCCCGAAGGAATCGAAGAAGAAGGTGGAGAGAGAGACAGAGACAGATCCGGAAGATTAGTGGATGGATTGTTGACAATTATCTGGGTCGACCTACGGAGCCTGTGCCTCTTCAGCTACCACCGCTTGAGAGACTTACTCTTGATTGTAACGAGGATTGTGGAACTTCTGGGACGCAGG---------------GGGTGGGAAGCCCTCAAATATTGGTGG---AATCTCCTTCAGTATTGG---------------------------------------------------GGTCAGGAACTAAAGAATAGTGCTGTTAGCTTGCTCAATGTCACAGCTATAGCAGTAGCTGAGGGAACAGATAGGATTATAGAAGTAGTACAAAGA------------------GCTTGTAGAGCTATTCGCAACATACCTGTAAGAATCAGACAGGGCTTGGAAAGGCTTTTGCTATAA

2.12008.SPD.EU575460 ATGAGAGTGATGGAGATCAGGAGGAACTATCAGCTCTTG---------TGGAAAGGGGGC------------------------ATCTTGCTCCTTGGGATGTTAATGATC------------TGTAATACTTCA---------GAAAAATTGTGGGTCACAGTCTATTATGGGGTACCTGTGTGGAAAGAAGCAACCACCACTCTATTTTGTGCCTCAGATGCTAAAGGACATGAGACAGAGGTACATAAT---GTCTGGGCCACACATGCCTGTGTACCCACAGACCCCAACCCACAAGAAATAGTATTG---AGAAATGTGACAGAAGAGTTTAACATATGGAAAAATAACATGGTAGAACAGATGCATGAGGATATAATCAGTTTATGGGATCAAAGCCTAAAGCCATGTGTAAAATTAACCCCACTCTGTGTCACTTTAAAGTGCACTGATGATTGGAATACTACTAAAATCATTGAGAATCGGAGTGCTACTGGAACCAATGTGACGAGT---------GCTCCTACTAGTACCCCTGTGACAACTGAGACTACAACCCGTAATAGTAGTGAGGGGAGATTAATGGAGATAAAAGAAATGAAAAACTGCTCTTTCAAGGTCACC---TCAAACGTAAGAGAT------AAGGTGCAGGAAGAATATGCACTTTTTTATAAATTTGATATAATACCAATAGGCAATGAT------------------------------AATAATACT------------------AATACCAGTTATAGGTTGATAAATTGCAACACCTCAGTCATTACACAAGCCTGTCCAAAAGTATCCTTTGAGCCAATTCCCATACATTATTGTGCCCCGGCTGGTTTTGCGATTCTAAAGTGT---AATGATAAGGAGTTCAATGGAACAGGACTGTGTACAAATGTTAGCACAGTACAATGTACACATGGAATTAGGCCAGTAGTGTCAACACAACTACTGTTAAATGGCAGTCTAGCAGAAGAA---AAGATAGTAATTAGATCTGAAAACATCACGAACAATGCTAAAACCATAATAGTACATCTGAACGAACCTATAAAAATTAATTGTACAAGACCCAGCAACAATACAAGAAAAAGTATACCTATAGGA------------CCAGGGAGA---GCATTTTATGCAACAGGAGAAATAATAGGAGATATAAGACAAGCACATTGTAACATT------AGTCAAAAAGATTGGAATACCACCTTAGAACGGGTAGTTGAAACATTAAGA---AGAACATTTGGG------------AATAAAACA---ATAATATTTGATCGA---------TCCTCAGGAGGGGACCCAGAAATTACAATGCACAGTTTTAATTGTGGAGGGGAATTTTTCTACTGTAATACATCATCACTGTTTAATAGTACTTGGGGCAGT---------AATGGTACTTGGAAGGGTACA---------------------AATGGCACTAGAGACGAA---------------------------------------------------ATCACACTCCAATGCAGAATAAAACAAATTATAAACATGTGGCAGGAAGTAGGAAAAGCAATGTATGCCCCTCCCATCAGAGGAAATATTAGCTGCTCATCAAATATTACTGGGCTGCTATTAACAAGAGATGGTGGTACTGGCAAG---------AACACTGCCCAG------------------------AAC---GAGACCTTCAGACCTGGAGGAGGAAATATGAAAGACAATTGGAGA---AATGAGTTATATAAATATAAAGTAGTAAAAATTGAACCA---TTAGGAGTAGCACCC---ACCAAGGCAAGGAGAAGAGTGGTGCAGAGAGAA---AAAAGAGCAGTG---GGA---ATAGGA---GCTGTG---TTCCTT---GGG---------TTCTTGTCA---GCAGCAGGAAGCACTATGGGCGCAGCGTCAATG---ACGCTGACGGTACAGGCCAGACAATTATTGTCTGGTATAGTGCAACAGCAAAGCAACTTGCTGAGGGCTATTGAGGCGCAACAGCATCTGTTGCAACTCACAGTCTGGGGCATCAAGCAGCTCCAGGCAAGA---GTCTTGGCTGTGGAAAGATACCTAAAGGATCAACAGCTCCTAGGGATTTGGGGTTGCTCTGGAAAACGCATCTGCACCACTAATGTGCCTTGGAATACTAGTTGGAGT---------------------------AATAAATCTCTGAATGATATTTGGGAT---AACATGACCTGGATGCAGTGGGAAAAAGAAATTGAC------AATTACACAAGCATAATATACACTTTAATTGAAGAATCGCAGAACCAGCAAGAAAAGAATGAACAGGAGTTATTGGAATTAGATAAATGGGCAAATTTGTGGAATTGGTTTTCCATAACACAATGGCTGTGGTATATAAAAATATTCATAATGATAGTAGGAGGCTTGATAGGTTTAAGAATAGTTTTTACTGTGCTTTCTATAGTGAATAGAGTTAGGCAGGGATACTCACCATTATCGTTTCAGACCTAC---CTCCCAGCCCAGAGGGGA------CACGACAGGCCCGAAGGAATCGAAGAAGAAGGTGGAGAGAGAGACAGAGACAGATCCGGAAGATTAGTGGATGGATTGTTGACAATTATCTGGGTCGACCTACGGAGCCTGTGCCTCTTCAGCTACCACCGCTTGAGAGACTTACTCTTGATTGTAACGAGGATTGTGGAACTTCTGGGACGCAGG---------------GGGTGGGAAGCCCTCAAATATTGGTGG---AATCTCCTTCAGTATTGG---------------------------------------------------GGTCAGGAACTAAAGAATAGTGCTGTTAGCTTGCTCAATGTCACAGCTATAGCAGTAGCTGAGGGAACAGATAGGATTATAGAAGTAGTACAAAGA------------------GCTTGTAGAGCTATTCGCAACATACCTGTAAGAATCAGACAGGGCTTGGAAAGGCTTTTGCTATAA

2.12008.SPD.EU575446 ATGAGAGTGATGGAGATCAGGAGGAACTATCAGCTCTTG---------TGGAAAGGGGGC------------------------ATCTTGCTCCTTGGGATGTTAATGATC------------TGTAATACTTCA---------GAAAAATTGTGGGTCACAGTCTATTATGGGGTACCTGTGTGGAAAGAAGCAACCACCACTCTATTTTGTGCCTCAGATGCTAAAGGACATGAGACAGAGGTACATAAT---GTCTGGGCCACACATGCCTGTGTACCCACAGACCCCAACCCACAAGAAATAGTATTG---AGAAATGTGACAGAAGAGTTTAACATATGGAAAAATAACATGGTAGAACAGATGCATGAGGATATAATCAGTTTATGGGATCAAAGCCTAAAGCCATGTGTAAAATTAACCCCACTCTGTGTCACTTTAAAGTGCACTGATGATTGGAATACTACTAAAATCATTGAGAATCGGAGTGCTACTGGAACCAATGTGACGAGT---------GCTCCTACTAGTACCCCTGTGACAACTGAGACTACAACCCGTAATAGTAGTGAGGGGAGATTAATGGAGATAAAAGAAATGAAAAACTGCTCTTTCAAGGTCACC---TCAAACGTAAGAGAT------AAGGTGCAGGAAGAATATGCACTTTTTTATAAATTTGATATAATACCAATAGGCAATGAT------------------------------AATAATACT------------------AATACCAGTTATAGGTTGATAAATTGCAACACCTCAGTCATTACACAAGCCTGTCCAAAAGTATCCTTTGAGCCAATTCCCATACATTATTGTGCCCCGGCTGGTTTTGCGATTCTAAAGTGT---AATGATAAGGAGTTCAATGGAACAGGACTGTGTACAAATGTTAGCACAGTACAATGTACACATGGAATTAGGCCAGTAGTGTCAACACAACTACTGTTAAATGGCAGTCTAGCAGAAGAA---AAGATAGTAATTAGATCTGAAAACATCACGAACAATGCTAAAACCATAATAGTACATCTGAACGAACCTATAAAAATTAATTGTACAAGACCCAGCAACAATACAAGAAAAAGTATACCTATAGGA------------CCAGGGAGA---GCATTTTATGCAACAGGAGAAATAATAGGAGATATAAGACAAGCACATTGTAACATT------AGTCAAAAAGATTGGAATACCACCTTAGAACGGGTAGTTGAAACATTAAGA---AGAACATTTGGG------------AATAAAACA---ATAATATTTGATCGA---------TCCTCAGGAGGGGACCCAGAAATTACAATGCACAGTTTTAATTGTGGAGGGGAATTTTTCTACTGTAATACATCATCACTGTTTAATAGTACTTGGGGCAGT---------AATGGTACTTGGAAGGGTACA---------------------AATGGCACTAGAGACGAA---------------------------------------------------ATCACACTCCAATGCAGAATAAAACAAATTATAAACATGTGGCAGGAAGTAGGAAAAGCAATGTATGCCCCTCCCATCAGAGGAAATATTAGCTGCTCATCAAATATTACTGGGCTGCTATTAACAAGAGATGGTGGTACTGGCAAG---------AACACTGCCCAG------------------------AAC---GAGACCTTCAGACCTGGAGGAGGAAATATGAAAGACAATTGGAGA---AATGAGTTATATAAATATAAAGTAGTAAAAATTGAACCA---TTAGGAGTAGCACCC---ACCAAGGCAAGGAGAAGAGTGGTGCAGAGAGAA---AAAAGAGCAGTG---GGA---ATAGGA---GCTGTG---TTCCTT---GGG---------TTCTTGTCA---GCAGCAGGAAGCACTATGGGCGCAGCGTCAATG---ACGCTGACGGTACAGGCCAGACAATTATTGTCTGGTATAGTGCAACAGCAAAGCAACTTGCTGAGGGCTATTGAGGCGCAACAGCATCTGTTGCAACTCACAGTCTGGGGCATCAAGCAGCTCCAGGCAAGA---GTCTTGGCTGTGGAAAGATACCTAAAGGATCAACAGCTCCTAGGGATTTGGGGTTGCTCTGGAAAACGCATCTGCACCACTAATGTGCCTTGGAATACTAGTTGGAGT---------------------------AATAAATCTCTGAATGATATTTGGGAT---AACATGACCTGGATGCAGTGGGAAAAAGAAATTGAC------AATTACACAAGCATAATATACACTTTAATTGAAGAATCGCAGAACCAGCAAGAAAAGAATGAACAGGAGTTATTGGAATTAGATAAATGGGCAAATTTGTGGAATTGGTTTTCCATAACACAATGGCTGTGGTATATAAAAATATTCATAATGATAGTAGGAGGCTTGATAGGTTTAAGAATAGTTTTTACTGTGCTTTCTATAGTGAATAGAGTTAGGCAGGGATACTCACCATTATCGTTTCAGACCCAC---CTCCCAGCCCAGAGGGGA------CACGACAGGCCCGAAGGAATCGAAGAAGAAGGTGGAGAGAGAGACAGAGACAGATCCGGAAGATTAGTGGATGGATTGTTGACAATTATCTGGGTCGACCTACGGAGCCTGTGCCTCTTCAGCTACCACCGCTTGAGAGACTTACTCTTGATTGTAACGAGGATTGTGGAACTTCTGGGACGCAGG---------------GGGTGGGAAGCCCTCAAATATTGGTGG---AATCTCCTTCAGTATTGG---------------------------------------------------GGTCAGGAACTAAAGAATAGTGCTGTTAGCTTGCTCAATGTCGCAGCTATAGCAGTAGCTGAGGGAACAGATAGGATTATAGAAGTAGTACAAAGA------------------GCTTGTAGAGCTATTCGCAACATACCTGTAAGAATCAGACAGGGCTTGGAAAGGCTTTTGCTATAA

2.12008.SPD.EU575456 ATGAGAGTGATGGAGATCAGGAGGAACTATCAGCTCTTG---------TGGAAAGGGGGC------------------------ATCTTGCTCCTTGGGATGTTAATGATC------------TGTAATACTTCA---------GAAAAATTGTGGGTCACAGTCTATTATGGGGTACCTGTGTGGAAAGAAGCAACCACCACTCTATTTTGTGCCTCAGATGCTAAAGGACATGAGACAGAGGTACATAAT---GTCTGGGCCACACATGCCTGTGTACCCACAGACCCCAACCCACAAGAAATAGTATTG---AGAAATGTGACAGAAGAGTTTAACATATGGAAAAATAACATGGTAGAACAGATGCATGAGGATATAATCAGTTTATGGGATCAAAGCCTAAAGCCATGTGTAAAATTAACCCCACTCTGTGTCACTTTAAAGTGCACTGATGATTGGAATACTACTAAAATCATTGAGAATCGGAGTGCTACTGGAACCAATGTGACGAGT---------GCTCCTACTAGTACCCCTGTGACAACTGAGACTACAACCCGTAATAGTAGTGAGGGGAGATTAATGGAGATAAAAGAAATGAAAAACTGCTCTTTCAAGGTCACC---TCAAACGTAAGAGAT------AAGGTGCAGGAAGAATATGCACTTTTTTATAAATTTGATATAATACCAATAGGCAATGAT------------------------------AATAATACT------------------AATACCAGTTATAGGTTGATAAATTGCAACACCTCAGTCATTACACAAGCCTGTCCAAAAGTATCCTTTGAGCCAATTCCCATACATTATTGTGCCCCGGCTGGTTTTGCGATTCTAAAGTGT---AATGATAAGGAGTTCAATGGAACAGGACTGTGTACAAATGTTAGCACAGTACAATGTACACATGGAATTAGGCCAGTAGTGTCAACACAACTACTGTTAAATGGCAGTCTAGCAGAAGAA---AAGATAGTAATTAGATCTGAAAACATCACGAACAATGCTAAAACCATAATAGTACATCTGAACGAACCTATAAAAATTAATTGTACAAGACCCAGCAACAATACAAGAAAAAGTATACCTATAGGA------------CCAGGGAGA---GCATTTTATGCAACAGGAGAAATAATAGGAGATATAAGACAAGCACATTGTAACATT------AGTCAAAAAGATTGGAATACCACCTTAGAACGGGTAGTTGAAACATTAAGA---AGAACATTTGGG------------AATAAAACA---ATAATATTTGATCGA---------TCCTCAGGAGGGGACCCAGAAATTACAATGCACAGTTTTAATTGTGGAGGGGAATTTTTCTACTGTAATACATCATCACTGTTTAATAGTACTTGGGGCAGT---------AATGGTACTTGGAAGGGTACA---------------------AATGGCACTAGAGACGAA---------------------------------------------------ATCACACTCCAATGCAGAATAAAACAAATTATAAACATGTGGCAGGAAGTAGGAAAAGCAATGTATGCCCCTCCCATCAGAGGAAATATTAGCTGCTCATCAAATATTACTGGGCTGCTATTAACAAGAGATGGTGGTACTGGCAAG---------AACACTGCCCAG------------------------AAC---GAGACCTTCAGACCTGGAGGAGGAAATATGAAAGACAATTGGAGA---AATGAGTTATATAAATATAAAGTAGTAAAAATTGAACCA---TTAGGAGTAGCACCC---ACCAAGGCAAGGAGAAGAGTGGTGCAGAGAGAA---AAAAGAGCAGTG---GGA---ATAGGA---GCTGTG---TTCCTT---GGG---------TTCTTGTCA---GCAGCAGGAAGCACTATGGGCGCAGCGTCAATG---ACGCTGACGGTACAGGCCAGACAATTATTGTCTGGTATAGTGCAACAGCAAAGCAACTTGCTGAGGGCTATTGAGGCGCAACAGCATCTGTTGCAACTCACAGTCTGGGGCATCAAGCAGCTCCAGGCAAGA---GTCTTGGCTGTGGAAAGATACCTAAAGGATCAACAGCTCCTAGGGATTTGGGGTTGCTCTGGAAAACGCATCTGCACCACTAATGTGCCTTGGAATACTAGTTGGAGT---------------------------AATAAATCTCTGAATGATATTTGGGAT---AACATGACCTGGATGCAGTGGGAAAAAGAAATTGAC------AATTACACAAGCATAATATACACTTTAATTGAAGAATCGCAGAACCAGCAAGAAAAGAATGAACAGGAGTTATTGGAATTAGATAAATGGGCAAATTTGTGGAATTGGTTTTCCATAACACAATGGCTGTGGTATATAAAAATATTCATAATGATAGTAGGAGGCTTGATAGGTTTAAGAATAGTTTTTACTGTGCTTTCTATAGTGAATAGAGTTAGGCAGGGATACTCACCATTATCGTTTCAGACCCAC---CTCCCAGCCCAGAGGGGA------CACGACAGGCCCGAAGGAATCGAAGAAGAAGGTGGAGAGAGAGACAGAGACAGATCCGGAAGATTAGTGGATGGATTGTTGACAATTATCTGGGTCGACCTACGGAGCCTGTGCCTCTTCAGCTACCACCGCTTGAGAGACTTACTCTTGATTGTAACGAGGATTGTGGAACTTCTGGGACGCAGG---------------GGGTGGGAAGCCCTCAAATATTGGTGG---AATCTCCTTCAGTATTGG---------------------------------------------------GGTCAGGAACTAAAGAATAGTGCTGTTAGCTTGCTCAATGTCACAGCTATAGCAGTAGCTGAGGGAACAGATAGGATTATAGAAGTAGTACAAAGA------------------GCTTGTAGAGCTATTCGCAACATACCTGTAAGAATCAGACAGGGCTTGGAAAGGCTTTTGCTATAA

2.12008.SPD.EU575439 ATGAGAGTGATGGAGATCAGGAGGAACTATCAGCTCTTG---------TGGAAAGGGGGC------------------------ATCTTGCTCCTTGGGATGTTAATGATC------------TGTAATACTTCA---------GAAAAATTGTGGGTCACAGTCTATTATGGGGTACCTGTGTGGAAAGAAGCAACCACCACTCTATTTTGTGCCTCAGATGCTAAAGGACATGAGACAGAGGTACATAAT---GTCTGGGCCACACATGCCTGTGTACCCACAGACCCCAACCCACAAGAAATAGTATTG---AGAAATGTGACAGAAGAGTTTAACATATGGAAAAATAACATGGTAGAACAGATGCATGAGGATATAATCAGTTTATGGGATCAAAGCCTAAAGCCATGTGTAAAATTAACCCCACTCTGTGTCACTTTAAAGTGCACTGATGATTGGAATACTACTAAAATCATTGAGAATCGGAGTGCTACTGGAACCAATGTGACGAGT---------GCTCCTACTAGTACCCCTGTGACAACTGAGACTACAACCCGTAATAGTAGTGAGGGGAGATTAATGGAGATAAAAGAAATGAAAAACTGCTCTTTCAAGGTCACC---TCAAACGTAAGAGAT------AAGGTGCAGGAAGAATATGCACTTTTTTATAAATTTGATATAATACCAATAGGCAATGAT------------------------------AATAATACT------------------AATACCAGTTATAGGTTGATAAATTGCAACACCTCAGTCATTACACAAGCCTGTCCAAAAGTATCCTTTGAGCCAATTCCCATACATTATTGTGCCCCGGCTGGTTTTGCGATTCTAAAGTGT---AATGATAAGGAGTTCAATGGAACAGGACTGTGTACAAATGTTAGCACAGTACAATGTACACATGGAATTAGGCCAGTAGTGTCAACACAACTACTGTTAAATGGCAGTCTAGCAGAAGAA---AAGATAGTAATTAGATCTGAAAACATCACGAACAATGCTAAAACCATAATAGTACATCTGAACGAACCTATAAAAATTAATTGTACAAGACCCAGCAACAATACAAGAAAAAGTATACCTATAGGA------------CCAGGGAGA---GCATTTTATGCAACAGGAGAAATAATAGGAGATATAAGACAAGCACATTGTAACATT------AGTCAAAAAGATTGGAATACCACCTTAGAACGGGTAGTTGAAACATTAAGA---AGAACATTTGGG------------AATAAAACA---ATAATATTTGATCGA---------TCCTCAGGAGGGGACCCAGAAATTACAATGCACAGTTTTAATTGTGGAGGGGAATTTTTCTACTGTAATACATCATCACTGTTTAATAGTACTTGGGGCAGT---------AATGGTACTTGGAAGGGTACA---------------------AATGGCACTAGAGACGAA---------------------------------------------------ATCACACTCCAATGCAGAATAAAACAAATTATAAACATGTGGCAGGAAGTAGGAAAAGCAATGTATGCCCCTCCCATCAGAGGAAATATTAGCTGCTCATCAAATATTACTGGGCTGCTATTAACAAGAGATGGTGGTACTGGCAAG---------AACACTGCCCAG------------------------AAC---GAGACCTTCAGACCTGGAGGAGGAAATATGAAAGACAATTGGAGA---AATGAGTTATATAAATATAAAGTAGTAAAAATTGAACCA---TTAGGAGTAGCACCC---ACCAAGGCAAGGAGAAGAGTGGTGCAGAGAGAA---AAAAGAGCAGTG---GGA---ATAGGA---GCTGTG---TTCCTT---GGG---------TTCTTGTCA---GCAGCAGGAAGCACTATGGGCGCAGCGTCAATG---ACGCTGACGGTACAGGCCAGACAATTATTGTCTGGTATAGTGCAACAGCAAAGCAACTTGCTGAGGGCTATTGAGGCGCAACAGCATCTGTTGCAACTCACAGTCTGGGGCATCAAGCAGCTCCAGGCAAGA---GTCTTGGCTGTGGAAAGATACCTAAAGGATCAACAGCTCCTAGGGATTTGGGGTTGCTCTGGAAAACGCATCTGCACCACTAATGTGCCTTGGAATACTAGTTGGAGT---------------------------AATAAATCTCTGAATGATATTTGGGAT---AACATGACCTGGATGCAGTGGGAAAAAGAAATTGAC------AATTACACAAGCATAATATACACTTTAATTGAAGAATCGCAGAACCAGCAAGAAAAGAATGAACAGGAGTTATTGGAATTAGATAAATGGGCAAATTTGTGGAATTGGTTTTCCATAACACAATGGCTGTGGTATATAAAAATATTCATAATGATAGTAGGAGGCTTGATAGGTTTAAGAATAGTTTTTACTGTGCTTTCTATAGTGAATAGAGTTAGGCAGGGATACTCACCATTATCGTTTCAGACCCAC---CTCCCAGCCCAGAGGGGA------CACGACAGGCCCGAAGGAATCGAAGAAGAAGGTGGAGAGAGAGACAGAGACAGATCCGGAAGATTAGTGGATGGATTGTTGACAATTATCTGGGTCGACCTACGGAGCCTGTGCCTCTTCAGCTACCACCGCTTGAGAGACTTACTCTTGATTGTAACGAGGATTGTGGAACTTCTGGGACGCAGG---------------GGGTGGGAAGCCCTCAAATATTGGTGG---AATCTCCTTCAGTATTGG---------------------------------------------------GGTCAGGAACTAAAGAATAGTGCTGTTAGCTTGCTCAATGTCACAGCTATAGCAGTAGCTGAGGGAACAGATAGGATTATAGAAGTAGTACAAAGA------------------GCTTGTAGAGCTATTCGCAACATACCTGTAAGAATCAGACAGGGCTTGGAAAGGCTTTTGCTATAA

2.12008.SPD.EU575440 ATGAGAGTGATGGAGATCAGGAGGAACTATCAGCTCTTG---------TGGAAAGGGGGC------------------------ATCTTGCTCCTTGGGATGTTAATGATC------------TGTAATACTTCA---------GAAAAATTGTGGGTCACAGTCTATTATGGGGTACCTGTGTGGAAAGAAGCAACCACCACTCTATTTTGTGCCTCAGATGCTAAAGGACATGAGACAGAGGTACATAAT---GTCTGGGCCACACATGCCTGTGTACCCACAGACCCCAACCCACAAGAAATAGTATTG---AGAAATGTGACAGAAGAGTTTAACATATGGAAAAATAACATGGTAGAACAGATGCATGAGGATATAATCAGTTTATGGGATCAAAGCCTAAAGCCATGTGTAAAATTAACCCCACTCTGTGTCACTTTAAAGTGCACTGATGATTGGAATACTACTAAAATCATTGAGAATCGGAGTGCTACTGGAACCAATGTGACGAGT---------GCTCCTACTAGTACCCCTGTGACAACTGAGACTACAACCCGTAATAGTAGTGAGGGGAGATTAATGGAGATAAAAGAAATGAAAAACTGCTCTTTCAAGGTCACC---TCAAACGTAAGAGAT------AAGGTGCAGGAAGAATATGCACTTTTTTATAAATTTGATATAATACCAATAGGCAATGAT------------------------------AATAATACT------------------AATACCAGTTATAGGTTGATAAATTGCAACACCTCAGTCATTACACAAGCCTGTCCAAAAGTATCCTTTGAGCCAATTCCCATACATTATTGTGCCCCGGCTGGTTTTGCGATTCTAAAGTGT---AATGATAAGGAGTTCAATGGAACAGGACTGTGTACAAATGTTAGCACAGTACAATGTACACATGGAATTAGGCCAGTAGTGTCAACACAACTACTGTTAAATGGCAGTCTAGCAGAAGAA---AAGATAGTAATTAGATCTGAAAACATCACGAACAATGCTAAAACCATAATAGTACATCTGAACGAACCTATAAAAATTAATTGTACAAGACCCAGCAACAATACAAGAAAAAGTATACCTATAGGA------------CCAGGGAGA---GCATTTTATGCAACAGGAGAAATAATAGGAGATATAAGACAAGCACATTGTAACATT------AGTCAAAAAGATTGGAATACCACCTTAGAACGGGTAGTTGAAACATTAAGA---AGAACATTTGGG------------AATAAAACA---ATAATATTTGATCGA---------TCCTCAGGAGGGGACCCAGAAATTACAATGCACAGTTTTAATTGTGGAGGGGAATTTTTCTACTGTAATACATCATCACTGTTTAATAGTACTTGGGGCAGT---------AATGGTACTTGGAAGGGTACA---------------------AATGGCACTAGAGACGAA---------------------------------------------------ATCACACTCCAATGCAGAATAAAACAAATTATAAACATGTGGCAGGAAGTAGGAAAAGCAATGTATGCCCCTCCCATCAGAGGAAATATTAGCTGCTCATCAAATATTACTGGGCTGCTATTAACAAGAGATGGTGGTACTGGCAAG---------AACACTGCCCAG------------------------AAC---GAGACCTTCAGACCTGGAGGAGGAAATATGAAAGACAATTGGAGA---AATGAGTTATATAAATATAAAGTAGTAAAAATTGAACCA---TTAGGAGTAGCACCC---ACCAAGGCAAGGAGAAGAGTGGTGCAGAGAGAA---AAAAGAGCAGTG---GGA---ATAGGA---GCTGTG---TTCCTT---GGG---------TTCTTGTCA---GCAGCAGGAAGCACTATGGGCGCAGCGTCAATG---ACGCTGACGGTACAGGCCAGACAATTATTGTCTGGTATAGTGCAACAGCAAAGCAACTTGCTGAGGGCTATTGAGGCGCAACAGCATCTGTTGCAACTCACAGTCTGGGGCATCAAGCAGCTCCAGGCAAGA---GTCTTGGCTGTGGAAAGATACCTAAAGGATCAACAGCTCCTAGGGATTTGGGGTTGCTCTGGAAAACGCATCTGCACCACTAATGTGCCTTGGAATACTAGTTGGAGT---------------------------AATAAATCTCTGAATGATATTTGGGAT---AACATGACCTGGATGCAGTGGGAAAAAGAAATTGAC------AATTACACAAGCATAATATACACTTTAATTGAAGAATCGCAGAACCAGCAAGAAAAGAATGAACAGGAGTTATTGGAATTAGATAAATGGGCAAATTTGTGGAATTGGTTTTCCATAACACAATGGCTGTGGTATATAAAAATATTCATAATGATAGTAGGAGGCTTGATAGGTTTAAGAATAGTTTTTACTGTGCTTTCTATAGTGAATAGAGTTAGGCAGGGATACTCACCATTATCGTTTCAGACCCAC---CTCCCAGCCCAGAGGGGA------CACGACAGGCCCGAAGGAATCGAAGAAGAAGGTGGAGAGAGAGACAGAGACAGATCCGGAAGATTAGTGGATGGATTGTTGACAATTATCTGGGTCGACCTACGGAGCCTGTGCCTCTTCAGCTACCACCGCTTGAGAGACTTACTCTTGATTGTAACGAGGATTGTGGAACTTCTGGGACGCAGG---------------GGGTGGGAAGCCCTCAAATATTGGTGG---AATCTCCTTCAGTATTGG---------------------------------------------------GGTCAGGAACTAAAGAATAGTGCTGTTAGCTTGCTCAATGTCACAGCTATAGCAGTAGCTGAGGGAACAGATAGGATTATAGAAGTAGTACAAAGA------------------GCTTGTAGAGCTATTCGCAACATACCTGTAAGAATCAGACAGGGCTTGGAAAGGCTTTTGCTATAA

2.12008.SPD.EU575450 ATGAGAGTGATGGAGATCAGGAGGAACTATCAGCTCTTG---------TGGAAAGGGGGC------------------------ATCTTGCTCCTTGGGATGTTAATGATC------------TGTAATACTTCA---------GAAAAATTGTGGGTCACAGTCTATTATGGGGTACCTGTGTGGAAAGAAGCAACCACCACTCTATTTTGTGCCTCAGATGCTAAAGGACATGAGACAGAGGTACATAAT---GTCTGGGCCACACATGCCTGTGTACCCACAGACCCCAACCCACAAGAAATAGTATTG---AGAAATGTGACAGAAGAGTTTAACATATGGAAAAATAACATGGTAGAACAGATGCATGAGGATATAATCAGTTTATGGGATCAAAGCCTAAAGCCATGTGTAAAATTAACCCCACTCTGTGTCACTTTAAAGTGCACTGATGATTGGAATACTACTAAAATCATTGAGAATCGGAGTGCTACTGGAACCAATGTGACGAGT---------GCTCCTACTAGTACCCCTGTGACAACTGAGACTACAACCCGTAATAGTAGTGAGGGGAGATTAATGGAGATAAAAGAAATGAAAAACTGCTCTTTCAAGGTCACC---TCAAACGTAAGAGAT------AAGGTGCAGGAAGAATATGCACTTTTTTATAAATTTGATATAATACCAATAGGCAATGAT------------------------------AATAATACT------------------AATACCAGTTATAGGTTGATAAATTGCAACACCTCAGTCATTACACAAGCCTGTCCAAAAGTATCCTTTGAGCCAATTCCCATACATTATTGTGCCCCGGCTGGTTTTGCGATTCTAAAGTGT---AATGATAAGGAGTTCAATGGAACAGGACTGTGTACAAATGTTAGCACAGTACAATGTACACATGGAATTAGGCCAGTAGTGTCAACACAACTACTGTTAAATGGCAGTCTAGCAGAAGAA---AAGATAGTAATTAGATCTGAAAACATCACGAACAATGCTAAAACCATAATAGTACATCTGAACGAACCTATAAAAATTAATTGTACAAGACCCAGCAACAATACAAGAAAAAGTATACCTATAGGA------------CCAGGGAGA---GCATTTTATGCAACAGGAGAAATAATAGGAGATATAAGACAAGCACATTGTAACATT------AGTCAAAAAGATTGGAATACCACCTTAGAACGGGTAGTTGAAACATTAAGA---AGAACATTTGGG------------AATAAAACA---ATAATATTTGATCGA---------TCCTCAGGAGGGGACCCAGAAATTACAATGCACAGTTTTAATTGTGGAGGGGAATTTTTCTACTGTAATACATCATCACTGTTTAATAGTACTTGGGGCAGT---------AATGGTACTTGGAAGGGTACA---------------------AATGGCACTAGAGACGAA---------------------------------------------------ATCACACTCCAATGCAGAATAAAACAAATTATAAACATGTGGCAGGAAGTAGGAAAAGCAATGTATGCCCCTCCCATCAGAGGAAATATTAGCTGCTCATCAAATATTACTGGGCTGCTATTAACAAGAGATGGTGGTACTGGCAAG---------AACACTGCCCAG------------------------AAC---GAGACCTTCAGACCTGGAGGAGGAAATATGAAAGACAATTGGAGA---AATGAGTTATATAAATATAAAGTAGTAAAAATTGAACCA---TTAGGAGTAGCACCC---ACCAAGGCAAGGAGAAGAGTGGTGCAGAGAGAA---AAAAGAGCAGTG---GGA---ATAGGA---GCTGTG---TTCCTT---GGG---------TTCTTGTCA---GCAGCAGGAAGCACTATGGGCGCAGCGTCAATG---ACGCTGACGGTACAGGCCAGACAATTATTGTCTGGTATAGTGCAACAGCAAAGCAACTTGCTGAGGGCTATTGAGGCGCAACAGCATCTGTTGCAACTCACAGTCTGGGGCATCAAGCAGCTCCAGGCAAGA---GTCTTGGCTGTGGAAAGATACCTAAAGGATCAACAGCTCCTAGGGATTTGGGGTTGCTCTGGAAAACGCATCTGCACCACTAATGTGCCTTGGAATACTAGTTGGAGT---------------------------AATAAATCTCTGAATGATATTTGGGAT---AACATGACCTGGATGCAGTGGGAAAAAGAAATTGAC------AATTACACAAGCATAATATACACTTTAATTGAAGAATCGCAGAACCAGCAAGAAAAGAATGAACAGGAGTTATTGGAATTAGATAAATGGGCAAATTTGTGGAATTGGTTTTCCATAACACAATGGCTGTGGTATATAAAAATATTCATAATGATAGTAGGAGGCTTGATAGGTTTAAGAATAGTTTTTACTGTGCTTTCTATAGTGAATAGAGTTAGGCAGGGATACTCACCATTATCGTTTCAGACCCAC---CTCCCAGCCCAGAGGGGA------CACGACAGGCCCGAAGGAATCGAAGAAGAAGGTGGAGAGAGAGACAGAGACAGATCCGGAAGATTAGTGGATGGATTGTTGACAATTATCTGGGTCGACCTACGGAGCCTGTGCCTCTTCAGCTACCACCGCTTGAGAGACTTACTCTTGATTGTAACGAGGATTGTGGAACTTCTGGGACGCAGG---------------GGGTGGGAAGCCCTCAAATATTGGTGG---AATCTCCTTCAGTATTGG---------------------------------------------------GGTCAGGAACTAAAGAATAGTGCTGTTAGCTTGCTCAATGTCACAGCTATAGCAGTAGCTGAGGGAACAGATAGGATTATAGAAGTAGTACAAAGA------------------GCTTGTAGAGCTATTCGCAACATACCTGTAAGAATCAGACAGGGCTTGGAAAGGCTTTTGCTATAA

2.12008.SPD.EU575454 ATGAGAGTGATGGAGATCAGGAGGAACTATCAGCTCTTG---------TGGAAAGGGGGC------------------------ATCTTGCTCCTTGGGATGTTAATGATC------------TGTAATACTTCA---------GAAAAATTGTGGGTCACAGTCTATTATGGGGTACCTGTGTGGAAAGAAGCAACCACCACTCTATTTTGTGCCTCAGATGCTAAAGGACATGAGACAGAGGTACATAAT---GTCTGGGCCACACATGCCTGTGTACCCACAGACCCCAACCCACAAGAAATAGTATTG---AGAAATGTGACAGAAGAGTTTAACATATGGAAAAATAACATGGTAGAACAGATGCATGAGGATATAATCAGTTTATGGGATCAAAGCCTAAAGCCATGTGTAAAATTAACCCCACTCTGTGTCACTTTAAAGTGCACTGATGATTGGAATACTACTAAAATCATTGAGAATCGGAGTGCTACTGGAACCAATGTGACGAGT---------GCTCCTACTAGTACCCCTGTGACAACTGAGACTACAACCCGTAATAGTAGTGAGGGGAGATTAATGGAGATAAAAGAAATGAAAAACTGCTCTTTCAAGGTCACC---TCAAACGTAAGAGAT------AAGGTGCAGGAAGAATATGCACTTTTTTATAAATTTGATATAATACCAATAGGCAATGAT------------------------------AATAATACT------------------AATACCAGTTATAGGTTGATAAATTGCAACACCTCAGTCATTACACAAGCCTGTCCAAAAGTATCCTTTGAGCCAATTCCCATACATTATTGTGCCCCGGCTGGTTTTGCGATTCTAAAGTGT---AATGATAAGGAGTTCAATGGAACAGGACTGTGTACAAATGTTAGCACAGTACAATGTACACATGGAATTAGGCCAGTAGTGTCAACACAACTACTGTTAAATGGCAGTCTAGCAGAAGAA---AAGATAGTAATTAGATCTGAAAACATCACGAACAATGCTAAAACCATAATAGTACATCTGAACGAACCTATAAAAATTAATTGTACAAGACCCAGCAACAATACAAGAAAAAGTATACCTATAGGA------------CCAGGGAGA---GCATTTTATGCAACAGGAGAAATAATAGGAGATATAAGACAAGCACATTGTAACATT------AGTCAAAAAGATTGGAATACCACCTTAGAACGGGTAGTTGAAACATTAAGA---AGAACATTTGGG------------AATAAAACA---ATAATATTTGATCGA---------TCCTCAGGAGGGGACCCAGAAATTACAATGCACAGTTTTAATTGTGGAGGGGAATTTTTCTACTGTAATACATCATCACTGTTTAATAGTACTTGGGGCAGT---------AATGGTACTTGGAAGGGTACA---------------------AATGGCACTAGAGACGAA---------------------------------------------------ATCACACTCCAATGCAGAATAAAACAAATTATAAACATGTGGCAGGAAGTAGGAAAAGCAATGTATGCCCCTCCCATCAGAGGAAATATTAGCTGCTCATCAAATATTACTGGGCTGCTATTAACAAGAGATGGTGGTACTGGCAAG---------AACACTGCCCAG------------------------AAC---GAGACCTTCAGACCTGGAGGAGGAAATATGAAAGACAATTGGAGA---AATGAGTTATATAAATATAAAGTAGTAAAAATTGAACCA---TTAGGAGTAGCACCC---ACCAAGGC-AGGAGAAGAGTGGTGCAGAGAGAA---AAAAGAGCAGTG---GGA---ATAGGA---GCTGTG---TTCCTT---GGG---------TTCTTGTCA---GCAGCAGGAAGCACTATGGGCGCAGCGTCAATG---ACGCTGACGGTACAGGCCAGACAATTATTGTCTGGTATAGTGCAACAGCAAAGCAACTTGCTGAGGGCTATTGAGGCGCAACAGCATCTGTTGCAACTCACAGTCTGGGGCATCAAGCAGCTCCAGGCAAGA---GTCTTGGCTGTGGAAAGATACCTAAAGGATCAACAGCTCCTAGGGATTTGGGGTTGCTCTGGAAAACGCATCTGCACCACTAATGTGCCTTGGAATACTAGTTGGAGT---------------------------AATAAATCTCTGAATGATATTTGGGAT---AACATGACCTGGATGCAGTGGGAAAAAGAAATTGAC------AATTACACAAGCATAATATACACTTTAATTGAAGAATCGCAGAACCAGCAAGAAAAGAATGAACAGGAGTTATTGGAATTAGATAAATGGGCAAATTTGTGGAATTGGTTTTCCATAACACAATGGCTGTGGTATATAAAAATATTCATAATGATAGTAGGAGGCTTGATAGGTTTAAGAATAGTTTTTACTGTGCTTTCTATAGTGAATAGAGTTAGGCAGGGATACTCACCATTATCGTTTCAGACCCAC---CTCCCAGCCCAGAGGGGA------CACGACAGGCCCGAAGGAATCGAAGAAGAAGGTGGAGAGAGAGACAGAGACAGATCCGGAAGATTAGTGGATGGATTGTTGACAATTATCTGGGTCGACCTACGGAGCCTGTGCCTCTTCAGCTACCACCGCTTGAGAGACTTACTCTTGATTGTAACGAGGATTGTGGAACTTCTGGGACGCAGG---------------GGGTGGGAAGCCCTCAAATATTGGTGG---AATCTCCTTCAGTATTGG---------------------------------------------------GGTCAGGAACTAAAGAATAGTGCTGTTAGCTTGCTCAATGTCACAGCTATAGCAGTAGCTGAGGGAACAGATAGGATTATAGAAGTAGTACAAAGA------------------GCTTGTAGAGCTATTCGCAACATACCTGTAAGAATCAGACAGGGCTTGGAAAGGCTTTTGCTATAA

2.12008.SPD.EU575447 ATGAGAGTGATGGAGATCAGGAGGAACTATCAGCTCTTG---------TGGAAAGGGGGC------------------------ATCTTGCTCCTTGGGATGTTAATGATC------------TGTAATACTTCA---------GAAAAATTGTGGGTCACAGTCTATTATGGGGTACCTGTGTGGAAAGAAGCAACCACCACTCTATTTTGTGCCTCAGATGCTAAAGGACATGAGACAGAGGTACATAAT---GTCTGGGCCACACATGCCTGTGTACCCACAGACCCCAACCCACAAGAAATAGTATTG---AGAAATGTGACAGAAGAGTTTAACATATGGAAAAATAACATGGTAGAACAGATGCATGAGGATATAATCAGTTTATGGGATCAAAGCCTAAAGCCATGTGTAAAATTAACCCCACTCTGTGTCACTTTAAAGTGCACTGATGATTGGAATACTACTAAAATCATTGAGAATCGGAGTGCTACTGGAACCAATGTGACGAGT---------GCTCCTACTAGTACCCCTGTGACAACTGAGACTACAACCCGTAATAGTAGTGAGGGGAGATTAATGGAGATAAAAGAAATGAAAAACTGCTCTTTCAAGGTCACC---TCAAACGTAAGAGAT------AAGGTGCAGGAAGAATATGCACTTTTTTATAAATTTGATATAATACCAATAGGCAATGAT------------------------------AATAATACT------------------AATACCAGTTATAGGTTGATAAATTGCAACACCTCAGTCATTACACAAGCCTGTCCAAAAGTATCCTTTGAGCCAATTCCCATACATTATTGTGCCCCGGCTGGTTTTGCGATTCTAAAGTGT---AATGATAAGGAGTTCAATGGAACAGGACTGTGTACAAATGTTAGCACAGTACAATGTACACATGGAATTAGGCCAGTAGTGTCAACACAACTACTGTTAAATGGCAGTCTAGCAGAAGAA---AAGATAGTAATTAGATCTGAAAACATCACGAACAATGCTAAAACCATAATAGTACATCTGAACGAACCTATAAAAATTAATTGTACAAGACCCAGCAACAATACAAGAAAAAGTATACCTATAGGA------------CCAGGGAGA---GCATTTTATGCAACAGGAGAAATAATAGGAGATATAAGACAAGCACATTGTAACATT------AGTCAAAAAGATTGGAATACCACCTTAGAACGGGTAGTTGAAACATTAAGA---AGAACATTTGGG------------AATAAAACA---ATAATATTTGATCGA---------TCCTCAGGAGGGGACCCAGAAATTACAATGCACAGTTTTAATTGTGGAGGGGAATTTTTCTACTGTAATACATCATCACTGTTTAATAGTACTTGGGGCAGT---------AATGGTACTTGGAAGGGTACA---------------------AATGGCACTAGAGACGAA---------------------------------------------------ATCACACTCCAATGCAGAATAAAACAAATTATAAACATGTGGCAGGAAGTAGGAAAAGCAATGTATGCCCCTCCCATCAGAGGAAATATTAGCTGCTCATCAAATATTACTGGGCTGCTATTAACAAGAGATGGTGGTACTGGCAAG---------AACACTGCCCAG------------------------AAC---GAGACCTTCAGACCTGGAGGAGGAAATATGAAAGACAATTGGAGA---AATGAGTTATATAAATATAAAGTAGTAAAAATTGAACCA---TTAGGAGTAGCACCC---ACCAAGGCAAGGAGAAGAGTGGTGCAGAGAGAA---AAAAGAGCAGTG---GGA---ATAGGA---GCTGTG---TTCCTT---GGG---------TTCTTGTCA---GCAGCAGGAAGCACTATGGGCGCAGCGTCAATG---ACGCTGACGGTACAGGCCAGACAATTATTGTCTGGTATAGTGCAACAGCAAAGCAACTTGCTGAGGGCTATTGAGGCGCAACAGCATCTGTTGCAACTCACAGTCTGGGGCATCAAGCAGCTCCAGGCAAGA---GTCTTGGCTGTGGAAAGATACCTAAAGGATCAACAGCTCCTAGGGATTTGGGGTTGCTCTGGAAAACGCATCTGCACCACTAATGTGCCTTGGAATACTAGTTGGAGT---------------------------AATAAATCTCTGAATGATATTTGGGAT---AACATGACCTGGATGCAGTGGGAAAAAGAAATTGAC------AATTACACAAGCATAATATACACTTTAATTGAAGAATCGCAGAACCAGCAAGAAAAGAATGAACAGGAGTTATTGGAATTAGATAAATGGGCAAATTTGTGGAATTGGTTTTCCATAACACAATGGCTGTGGTATATAAAAATATTCATAATGATAGTAGGAGGCTTGATAGGTTTAAGAATAGTTTTTACTGTGCTTTCTATAGTGAATAGAGTTAGGCAGGGATACTCACCATTATCGTTTCAGACCCAC---CTCCCAGCCCAGAGGGGA------CACGACAGGCCCGAAGGAATCGAAGAAGAAGGTGGAGAGAGAGACAGAGACAGATCCGGAAGATTAGTGGATGGATTGTTGACAATTATCTGGGTCGACCTACGGAGCCTGTGCCTCTTCAGCTACCACCGCTTGAGAGACTTACTCTTGATTGTAACGAGGATTGTGGAACTTCTGGGACGCAGG---------------GGGTGGGAAGCCCTCAAATATTGGTGG---AATCTCCTTCAGTATTGG---------------------------------------------------GGTCAGGAACTAAAGAATAGTGCTGTTAGCTTGCTCAATGTCACAGCTATAGCAGTAGCTGAGGGAACAGATAGGATTATAGAAGTAGTACAAAGA------------------GCTTGTAGAGCTATTCGCAACATACCTGTAAGAATCAGACAGGGCTTGGAAAGGCTTTTGCTATAA

2.12008.SPD.EU575458 ATGAGAGTGATGGAGATCAGGAGGAACTATCAGCTCTTG---------TGGAAAGGGGGC------------------------ATCTTGCTCCTTGGGATGTTAATGATC------------TGTAATACTTCA---------GAAAAATTGTGGGTCACAGTCTATTATGGGGTACCTGTGTGGAAAGAAGCAACCACCACTCTATTTTGTGCCTCAGATGCTAAAGGACATGAGACAGAGGTACATAAT---GTCTGGGCCACACATGCCTGTGTACCCACAGACCCCAACCCACAAGAAATAGTATTG---AGAAATGTGACAGAAGAGTTTAACATATGGAAAAATAACATGGTAGAACAGATGCATGAGGATATAATCAGTTTATGGGATCAAAGCCTAAAGCCATGTGTAAAATTAACCCCACTCTGTGTCACTTTAAAGTGCACTGATGATTGGAATACTACTAAAATCATTGAGAATCGGAGTGCTACTGGAACCAATGTGACGAGT---------GCTCCTACTAGTACCCCTGTGACAACTGAGACTACAACCCGTAATAGTAGTGAGGGGAGATTAATGGAGATAAAAGAAATGAAAAACTGCTCTTTCAAGGTCACC---TCAAACGTAAGAGAT------AAGGTGCAGGAAGAATATGCACTTTTTTATAAATTTGATATAATACCAATAGGCAATGAT------------------------------AATAATACT------------------AATACCAGTTATAGGTTGATAAATTGCAACACCTCAGTCATTACACAAGCCTGTCCAAAAGTATCCTTTGAGCCAATTCCCATACATTATTGTGCCCCGGCTGGTTTTGCGATTCTAAAGTGT---AATGATAAGGAGTTCAATGGAACAGGACTGTGTACAAATGTTAGCACAGTACAATGTACACATGGAATTAGGCCAGTAGTGTCAACACAACTACTGTTAAATGGCAGTCTAGCAGAAGAA---AAGATAGTAATTAGATCTGAAAACATCACGAACAATGCTAAAACCATAATAGTACATCTGAACGAACCTATAAAAATTAATTGTACAAGACCCAGCAACAATACAAGAAAAAGTATACCTATAGGA------------CCAGGGAGA---GCATTTTATGCAACAGGAGAAATAATAGGAGATATAAGACAAGCACATTGTAACATT------AGTCAAAAAGATTGGAATACCACCTTAGAACGGGTAGTTGAAACATTAAGA---AGAACATTTGGG------------AATAAAACA---ATAATATTTGATCGA---------TCCTCAGGAGGGGACCCAGAAATTACAATGCACAGTTTTAATTGTGGAGGGGAATTTTTCTACTGTAATACATCATCACTGTTTAATAGTACTTGGGGCAGT---------AATGGTACTTGGAAGGGTACA---------------------AATGGCACTAGAGACGAA---------------------------------------------------ATCACACTCCAATGCAGAATAAAACAAATTATAAACATGTGGCAGGAAGTAGGAAAAGCAATGTATGCCCCTCCCATCAGAGGAAATATTAGCTGCTCATCAAATATTACTGGGCTGCTATTAACAAGAGATGGTGGTACTGGCAAG---------AACACTGCCCAG------------------------AAC---GAGACCTTCAGACCTGGAGGAGGAAATATGAAAGACAATTGGAGA---AATGAGTTATATAAATATAAAGTAGTAAAAATTGAACCA---TTAGGAGTAGCACCC---ACCAAGGCAAGGAGAAGAGTGGTGCAGAGAGAA---AAAAGAGCAGTG---GGA---ATAGGA---GCTGTG---TTCCTT---GGG---------TTCTTGTCA---GCAGCAGGAAGCACTATGGGCGCAGCGTCAATG---ACGCTGACGGTACAGGCCAGACAATTATTGTCTGGTATAGTGCAACAGCAAAGCAACTTGCTGAGGGCTATTGAGGCGCAACAGCATCTGTTGCAACTCACAGTCTGGGGCATCAAGCAGCTCCAGGCAAGA---GTCTTGGCTGTGGAAAGATACCTAAAGGATCAACAGCTCCTAGGGATTTGGGGTTGCTCTGGAAAACGCATCTGCACCACTAATGTGCCTTGGAATACTAGTTGGAGT---------------------------AATAAATCTCTGAATGATATTTGGGAT---AACATGACCTGGATGCAGTGGGAAAAAGAAATTGAC------AATTACACAAGCATAATATACACTTTAATTGAAGAATCGCAGAACCAGCAAGAAAAGAATGAACAGGAGTTATTGGAATTAGATAAATGGGCAAATTTGTGGAATTGGTTTTCCATAACACAATGGCTGTGGTATATAAAAATATTCATAATGATAGTAGGAGGCTTGATAGGTTTAAGAATAGTTTTTACTGTGCTTTCTATAGTGAATAGAGTTAGGCAGGGATACTCACCATTATCGTTTCAGACCCAC---CTCCCAGCCCAGAGGGGA------CACGACAGGCCCGAAGGAATCGAAGAAGAAGGTGGAGAGAGAGACAGAGACAGATCCGGAAGATTAGTGGATGGATTGTTGACAATTATCTGGGTCGACCTACGGAGCCTGTGCCTCTTCAGCTACCACCGCTTGAGAGACTTACTCTTGATTGTAACGAGGATTGTGGAACTTCTGGGACGCAGG---------------GGGTGGGAAGCCCTCAAATATTGGTGG---AATCTCCTTCAGTATTGG---------------------------------------------------GGTCAGGAACTAAAGAATAGTGCTGTTAGCTTGCTCAATGTCACAGCTATAGCAGTAGCTGAGGGAACAGATAGGATTATAGAAGTAGTACAAAGA------------------GCTTGTAGAGCTATTCGCAACATACCTGTAAGAATCAGACAGGGCTTGGAAAGGCTTTTGCTATAA

2.12008.SPD.EU575465 ATGAGAGTGATGGAGATCAGGAGGAACTATCAGCTCTTG---------TGGAAAGGGGGC------------------------ATCTTGCTCCTTGGGATGTTAATGATC------------TGTAATACTTCA---------GAAAAATTGTGGGTCACAGTCTATTATGGGGTACCTGTGTGGAAAGAAGCAACCACCACTCTATTTTGTGCCTCAGATGCTAAAGGACATGAGACAGAGGTACATAAT---GTCTGGGCCACACATGCCTGTGTACCCACAGACCCCAACCCACAAGAAATAGTATTG---AGAAATGTGACAGAAGAGTTTAACATATGGAAAAATAACATGGTAGAACAGATGCATGAGGATATAATCAGTTTATGGGATCAAAGCCTAAAGCCATGTGTAAAATTAACCCCACTCTGTGTCACTTTAAAGTGCACTGATGATTGGAATACTACTAAAATCATTGAGAATCGGAGTGCTACTGGAACCAATGTGACGAGT---------GCTCCTACTAGTACCCCTGTGACAACTGAGACTACAACCCGTAATAGTAGTGAGGGGAGATTAATGGAGATAAAAGAAATGAAAAACTGCTCTTTCAAGGTCACC---TCAAACGTAAGAGAT------AAGGTGCAGGAAGAATATGCACTTTTTTATAAATTTGATATAATACCAATAGGCAATGAT------------------------------AATAATACT------------------AATACCAGTTATAGGTTGATAAATTGCAACACCTCAGTCATTACACAAGCCTGTCCAAAAGTATCCTTTGAGCCAATTCCCATACATTATTGTGCCCCGGCTGGTTTTGCGATTCTAAAGTGT---AATGATAAGGAGTTCAATGGAACAGGACTGTGTACAAATGTTAGCACAGTACAATGTACACATGGAATTAGGCCAGTAGTGTCAACACAACTACTGTTAAATGGCAGTCTAGCAGAAGAA---AAGATAGTAATTAGATCTGAAAACATCACGAACAATGCTAAAACCATAATAGTACATCTGAACGAACCTATAAAAATTAATTGTACAAGACCCAGCAACAATACAAGAAAAAGTATACCTATAGGA------------CCAGGGAGA---GCATTTTATGCAACAGGAGAAATAATAGGAGATATAAGACAAGCACATTGTAACATT------AGTCAAAAAGATTGGAATACCACCTTAGAACGGGTAGTTGAAACATTAAGA---AGAACATTTGGG------------AATAAAACA---ATAATATTTGATCGA---------TCCTCAGGAGGGGACCCAGAAATTACAATGCACAGTTTTAATTGTGGAGGGGAATTTTTCTACTGTAATACATCATCACTGTTTAATAGTACTTGGGGCAGT---------AATGGTACTTGGAAGGGTACA---------------------AATGGCACTAGAGACGAA---------------------------------------------------ATCACACTCCAATGCAGAATAAAACAAATTATAAACATGTGGCAGGAAGTAGGAAAAGCAATGTATGCCCCTCCCATCAGAGGAAATATTAGCTGCTCATCAAATATTACTGGGCTGCTATTAACAAGAGATGGTGGTACTGGCAAG---------AACACTGCCCAG------------------------AAC---GAGACCTTCAGACCTGGAGGAGGAAATATGAAAGACAATTGGAGA---AATGAGTTATATAAATATAAAGTAGTAAAAATTGAACCA---TTAGGAGTAGCACCC---ACCAAGGCAAGGAGAAGAGTGGTGCAGAGAGAA---AAAAGAGCAGTG---GGA---ATAGGA---GCTGTG---TTCCTT---GGG---------TTCTTGTCA---GCAGCAGGAAGCACTATGGGCGCAGCGTCAATG---ACGCTGACGGTACAGGCCAGACAATTATTGTCTGGTATAGTGCAACAGCAAAGCAACTTGCTGAGGGCTATTGAGGCGCAACAGCATCTGTTGCAACTCACAGTCTGGGGCATCAAGCAGCTCCAGGCAAGA---GTCTTGGCTGTGGAAAGATACCTAAAGGATCAACAGCTCCTAGGGATTTGGGGTTGCTCTGGAAAACGCATCTGCACCACTAATGTGCCTTGGAATACTAGTTGGAGT---------------------------AATAAATCTCTGAATGATATTTGGGAT---AACATGACCTGGATGCAGTGGGAAAAAGAAATTGAC------AATTACACAAGCATAATATACACTTTAATTGAAGAATCGCAGAACCAGCAAGAAAAGAATGAACAGGAGTTATTGGAATTAGATAAATGGGCAAATTTGTGGAATTGGTTTTCCATAACACAATGGCTGTGGTATATAAAAATATTCATAATGATAGTAGGAGGCTTGATAGGTTTAAGAATAGTTTTTACTGTGCTTTCTATAGTGAATAGAGTTAGGCAGGGATACTCACCATTATCGTTTCAGACCCAC---CTCCCAGCCCAGAGGGGA------CACGACAGGCCCGAAGGAATCGAAGAAGAAGGTGGAGAGAGAGACAGAGACAGATCCGGAAGATTAGTGGATGGATTGTTGACAATTATCTGGGTCGACCTACGGAGCCTGTGCCTCTTCAGCTACCACCGCTTGAGAGACTTACTCTTGATTGTAACGAGGATTGTGGAACTTCTGGGACGCAGG---------------GGGTGGGAAGCCCTCAAATATTGGTGG---AATCTCCTTCAGTATTGG---------------------------------------------------GGTCAGGAACTAAAGAATAGTGCTGTTAGCTTGCTCAATGTCACAGCTATAGCAGTAGCTGAGGGAACAGATAGGATTATAGAAGTAGTACAAAGA------------------GCTTGTAGAGCTATTCGCAACATACCTGTAAGAATCAGACAGGGCTTGGAAAGGCTTTTGCTATAA

2.12008.SPD.EU575451 ATGAGAGTGATGGAGATCAGGAGGAACTATCAGCTCTTG---------TGGAAAGGGGGC------------------------ATCTTGCTCCTTGGGATGTTAATGATC------------TGTAATACTTCA---------GAAAAATTGTGGGTCACAGTCTATTATGGGGTACCTGTGTGGAAAGAAGCAACCACCACTCTATTTTGTGCCTCAGATGCTAAAGGACATGAGACAGAGGTACATAAT---GTCTGGGCCACACATGCCTGTGTACCCACAGACCCCAACCCACAAGAAATAGTATTG---AGAAATGTGACAGAAGAGTTTAACATATGGAAAAATAACATGGTAGAACAGATGCATGAGGATATAATCAGTTTATGGGATCAAAGCCTAAAGCCATGTGTAAAATTAACCCCACTCTGTGTCACTTTAAAGTGCACTGATGATTGGAATACTACTAAAATCATTGAGAATCGGAGTGCTACTGGAACCAATGTGACGAGT---------GCTCCTACTAGTACCCCTGTGACAACTGAGACTACAACCCGTAATAGTAGTGAGGGGAGATTAATGGAGATAAAAGAAATGAAAAACTGCTCTTTCAAGGTCACC---TCAAACGTAAGAGAT------AAGGTGCAGGAAGAATATGCACTTTTTTATAAATTTGATATAATACCAATAGGCAATGAT------------------------------AATAATACT------------------AATACCAGTTATAGGTTGATAAATTGCAACACCTCAGTCATTACACAAGCCTGTCCAAAAGTATCCTTTGAGCCAATTCCCATACATTATTGTGCCCCGGCTGGTTTTGCGATTCTAAAGTGT---AATGATAAGGAGTTCAATGGAACAGGACTGTGTACAAATGTTAGCACAGTACAATGTACACATGGAATTAGGCCAGTAGTGTCAACACAACTACTGTTAAATGGCAGTCTAGCAGAAGAA---AAGATAGTAATTAGATCTGAAAACATCACGAACAATGCTAAAACCATAATAGTACATCTGAACGAACCTATAAAAATTAATTGTACAAGACCCAGCAACAATACAAGAAAAAGTATACCTATAGGA------------CCAGGGAGA---GCATTTTATGCAACAGGAGAAATAATAGGAGATATAAGACAAGCACATTGTAACATT------AGTCAAAAAGATTGGAATACCACCTTAGAACGGGTAGTTGAAACATTAAGA---AGAACATTTGGG------------AATAAAACA---ATAATATTTGATCGA---------TCCTCAGGAGGGGACCCAGAAATTACAATGCACAGTTTTAATTGTGGAGGGGAATTTTTCTACTGTAATACATCATCACTGTTTAATAGTACTTGGGGCAGT---------AATGGTACTTGGAAGGGTACA---------------------AATGGCACTAGAGACGAA---------------------------------------------------ATCACACTCCAATGCAGAATAAAACAAATTATAAACATGTGGCAGGAAGTAGGAAAAGCAATGTATGCCCCTCCCATCAGAGGAAATATTAGCTGCTCATCAAATATTACTGGGCTGCTATTAACAAGAGATGGTGGTACTGGCAAG---------AACACTGCCCAG------------------------AAC---GAGACCTTCAGACCTGGAGGAGGAAATATGAAAGACAATTGGAGA---AATGAGTTATATAAATATAAAGTAGTAAAAATTGAACCA---TTAGGAGTAGCACCC---ACCAAGGCAAGGAGAAGAGTGGTGCAGAGAGAA---AAAAGAGCAGTG---GGA---ATAGGA---GCTGTG---TTCCTT---GGG---------TTCTTGTCA---GCAGCAGGAAGCACTATGGGCGCAGCGTCAATG---ACGCTGACGGTACAGGCCAGACAATTATTGTCTGGTATAGTGCAACAGCAAAGCAACTTGCTGAGGGCTATTGAGGCGCAACAGCATCTGTTGCAACTCACAGTCTGGGGCATCAAGCAGCTCCAGGCAAGA---GTCTTGGCTGTGGAAAGATACCTAAAGGATCAACAGCTCCTAGGGATTTGGGGTTGCTCTGGAAAACGCATCTGCACCACTAATGTGCCTTGGAATACTAGTTGGAGT---------------------------AATAAATCTCTGAATGATATTTGGGAT---AACATGACCTGGATGCAGTGGGAAAAAGAAATTGAC------AATTACACAAGCATAATATACACTTTAATTGAAGAATCGCAGAACCAGCAAGAAAAGAATGAACAGGAGTTATTGGAATTAGATAAATGGGCAAATTTGTGGAATTGGTTTTCCATAACACAATGGCTGTGGTATATAAAAATATTCATAATGATAGTAGGAGGCTTGATAGGTTTAAGAATAGTTTTTACTGTGCTTTCTATAGTGAATAGAGTTAGGCAGGGATACTCACCATTATCGTTTCAGACCCAC---CTCCCAGCCCAGAGGGGA------CACGACAGGCCCGAAGGAATCGAAGAAGAAGGTGGAGAGAGAGACAGAGACAGATCCGGAAGATTAGTGGATGGATTGTTGACAATTATCTGGGTCGACCTACGGAGCCTGTGCCTCTTCAGCTACCACCGCTTGAGAGACTTACTCTTGATTGTAACGAGGATTGTGGAACTTCTGGGACGCAGG---------------GGGTGGGAAGCCCTCAAATATTGGTGG---AATCTCCTTCAGTATTGG---------------------------------------------------GGTCAGGAACTAAAGAATAGTGCTGTTAGCTTGCTCAATGTCACAGCTATAGCAGTAGCTGAGGGAACAGATAGGATTATAGAAGTAGTACAAAGA------------------GCTTGTAGAGCTATTCGCAACATACCTGTAAGAATCAGACAGGGCTTGGAAAGGCTTTTGCTATAA

2.12008.SPD.EU575464 ATGAGAGTGATGGAGATCAGGAGGAACTATCAGCTCTTG---------TGGAAAGGGGGC------------------------ATCTTGCTCCTTGGGATGTTAATGATC------------TGTAATACTTCA---------GAAAAATTGTGGGTCACAGTCTATTATGGGGTACCTGTGTGGAAAGAAGCAACCACCACTCTATTTTGTGCCTCAGATGCTAAAGGACATGAGACAGAGGTACATAAT---GTCTGGGCCACACATGCCTGTGTACCCACAGACCCCAACCCACAAGAAATAGTATTG---AGAAATGTGACAGAAGAGTTTAACATATGGAAAAATAACATGGTAGAACAGATGCATGAGGATATAATCAGTTTATGGGATCAAAGCCTAAAGCCATGTGTAAAATTAACCCCACTCTGTGTCACTTTAAAGTGCACTGATGATTGGAATACTACTAAAATCATTGAGAATCGGAGTGCTACTGGAACCAATGTGACGAGT---------GCTCCTACTAGTACCCCTGTGACAACTGAGACTACAACCCGTAATAGTAGTGAGGGGAGATTAATGGAGATAAAAGAAATGAAAAACTGCTCTTTCAAGGTCACC---TCAAACGTAAGAGAT------AAGGTGCAGGAAGAATATGCACTTTTTTATAAATTTGATATAATACCAATAGGCAATGAT------------------------------AATAATACT------------------AATACCAGTTATAGGTTGATAAATTGCAACACCTCAGTCATTACACAAGCCTGTCCAAAAGTATCCTTTGAGCCAATTCCCATACATTATTGTGCCCCGGCTGGTTTTGCGATTCTAAAGTGT---AATGATAAGGAGTTCAATGGAACAGGACTGTGTACAAATGTTAGCACAGTACAATGTACACATGGAATTAGGCCAGTAGTGTCAACACAACTACTGTTAAATGGCAGTCTAGCAGAAGAA---AAGATAGTAATTAGATCTGAAAACATCACGAACAATGCTAAAACCATAATAGTACATCTGAACGAACCTATAAAAATTAATTGTACAAGACCCAGCAACAATACAAGAAAAAGTATACCTATAGGA------------CCAGGGAGA---GCATTTTATGCAACAGGAGAAATAATAGGAGATATAAGACAAGCACATTGTAACATT------AGTCAAAAAGATTGGAATACCACCTTAGAACGGGTAGTTGAAACATTAAGA---AGAACATTTGGG------------AATAAAACA---ATAATATTTGATCGA---------TCCTCAGGAGGGGACCCAGAAATTACAATGCACAGTTTTAATTGTGGAGGGGAATTTTTCTACTGTAATACATCATCACTGTTTAATAGTACTTGGGGCAGT---------AATGGTACTTGGAAGGGTACA---------------------AATGGCACTAGAGACGAA---------------------------------------------------ATCACACTCCAATGCAGAATAAAACAAATTATAAACATGTGGCAGGAAGTAGGAAAAGCAATGTATGCCCCTCCCATCAGAGGAAATATTAGCTGCTCATCAAATATTACTGGGCTGCTATTAACAAGAGATGGTGGTACTGGCAAG---------AACACTGCCCAG------------------------AAC---GAGACCTTCAGACCTGGAGGAGGAAATATGAAAGACAATTGGAGA---AATGAGTTATATAAATATAAAGTAGTAAAAATTGAACCA---TTAGGAGTAGCACCC---ACCAAGGCAAGGAGAAGAGTGGTGCAGAGAGAA---AAAAGAGCAGTG---GGA---ATAGGA---GCTGTG---TTCCTT---GGG---------TTCTTGTCA---GCAGCAGGAAGCACTATGGGCGCAGCGTCAATG---ACGCTGACGGTACAGGCCAGACAATTATTGTCTGGTATAGTGCAACAGCAAAGCAACTTGCTGAGGGCTATTGAGGCGCAACAGCATCTGTTGCAACTCACAGTCTGGGGCATCAAGCAGCTCCAGGCAAGA---GTCTTGGCTGTGGAAAGATACCTAAAGGATCAACAGCTCCTAGGGATTTGGGGTTGCTCTGGAAAACGCATCTGCACCACTAATGTGCCTTGGAATACTAGTTGGAGT---------------------------AATAAATCTCTGAATGATATTTGGGAT---AACATGACCTGGATGCAGTGGGAAAAAGAAATTGAC------AATTACACAAGCATAATATACACTTTAATTGAAGAATCGCAGAACCAGCAAGAAAAGAATGAACAGGAGTTATTGGAATTAGATAAATGGGCAAATTTGTGGAATTGGTTTTCCATAACACAATGGCTGTGGTATATAAAAATATTCATAATGATAGTAGGAGGCTTGATAGGTTTAAGAATAGTTTTTACTGTGCTTTCTATAGTGAATAGAGTTAGGCAGGGATACTCACCATTATCGTTTCAGACCCAC---CTCCCAGCCCAGAGGGGA------CACGACAGGCCCGAAGGAATCGAAGAAGAAGGTGGAGAGAGAGACAGAGACAGATCCGGAAGATTAGTGGATGGATTGTTGACAATTATCTGGGTCGACCTACGGAGCCTGTGCCTCTTCAGCTACCACCGCTTGAGAGACTTACTCTTGATTGTAACGAGGATTGTGGAACTTCTGGGACGCAGG---------------GGGTGGGAAGCCCTCAAATATTGGTGG---AATCTCCTTCAGTATTGG---------------------------------------------------GGTCAGGAACTAAAGAATAGTGCTGTTAGCTTGCTCAATGTCACAGCTATAGCAGTAGCTGAGGGAACAGATAGGATTATAGAAGTAGTACAAAGA------------------GCTTGTAGAGCTATTCGCAACATACCTGTAAGAATCAGACAGGGCTTGGAAAGGCTTTTGCTATAA

2.12008.SPD.EU575455 ATGAGAGTGATGGAGATCAGGAGGAACTATCAGCTCTTG---------TGGAAAGGGGGC------------------------ATCTTGCTCCTTGGGATGTTAATGATC------------TGTAATACTTCA---------GAAAAATTGTGGGTCACAGTCTATTATGGGGTACCTGTGTGGAAAGAAGCAACCACCACTCTATTTTGTGCCTCAGATGCTAAAGGACATGAGACAGAGGTACATAAT---GTCTGGGCCACACATGCCTGTGTACCCACAGACCCCAACCCACAAGAAATAGTATTG---AGAAATGTGACAGAAGAGTTTAACATATGGAAAAATAACATGGTAGAACAGATGCATGAGGATATAATCAGTTTATGGGATCAAAGCCTAAAGCCATGTGTAAAATTAACCCCACTCTGTGTCACTTTAAAGTGCACTGATGATTGGAATACTACTAAAATCATTGAGAATCGGAGTGCTACTGGAACCAATGTGACGAGT---------GCTCCTACTAGTACCCCTGTGACAACTGAGACTACAACCCGTAATAGTAGTGAGGGGAGATTAATGGAGATAAAAGAAATGAAAAACTGCTCTTTCAAGGTCACC---TCAAACGTAAGAGAT------AAGGTGCAGGAAGAATATGCACTTTTTTATAAATTTGATATAATACCAATAGGCAATGAT------------------------------AATAATACT------------------AATACCAGTTATAGGTTGATAAATTGCAACACCTCAGTCATTACACAAGCCTGTCCAAAAGTATCCTTTGAGCCAATTCCCATACATTATTGTGCCCCGGCTGGTTTTGCGATTCTAAAGTGT---AATGATAAGGAGTTCAATGGAACAGGACTGTGTACAAATGTTAGCACAGTACAATGTACACATGGAATTAGGCCAGTAGTGTCAACACAACTACTGTTAAATGGCAGTCTAGCAGAAGAA---AAGATAGTAATTAGATCTGAAAACATCACGAACAATGCTAAAACCATAATAGTACATCTGAACGAACCTATAAAAATTAATTGTACAAGACCCAGCAACAATACAAGAAAAAGTATACCTATAGGA------------CCAGGGAGA---GCATTTTATGCAACAGGAGAAATAATAGGAGATATAAGACAAGCACATTGTAACATT------AGTCAAAAAGATTGGAATACCACCTTAGAACGGGTAGTTGAAACATTAAGA---AGAACATTTGGG------------AATAAAACA---ATAATATTTGATCGA---------TCCTCAGGAGGGGACCCAGAAATTACAATGCACAGTTTTAATTGTGGAGGGGAATTTTTCTACTGTAATACATCATCACTGTTTAATAGTACTTGGGGCAGT---------AATGGTACTTGGAAGGGTACA---------------------AATGGCACTAGAGACGAA---------------------------------------------------ATCACACTCCAATGCAGAATAAAACAAATTATAAACATGTGGCAGGAAGTAGGAAAAGCAATGTATGCCCCTCCCATCAGAGGAAATATTAGCTGCTCATCAAATATTACTGGGCTGCTATTAACAAGAGATGGTGGTACTGGCAAG---------AACACTGCCCAG------------------------AAC---GAGACCTTCAGACCTGGAGGAGGAAATATGAAAGACAATTGGAGA---AATGAGTTATATAAATATAAAGTAGTAAAAATTGAACCA---TTAGGAGTAGCACCC---ACCAAGGCAAGGAGAAGAGTGGTGCAGAGAGAA---AAAAGAGCAGTG---GGA---ATAGGA---GCTGTG---TTCCTT---GGG---------TTCTTGTCA---GCAGCAGGAAGCACTATGGGCGCAGCGTCAATG---ACGCTGACGGTACAGGCCAGACAATTATTGTCTGGTATAGTGCAACAGCAAAGCAACTTGCTGAGGGCTATTGAGGCGCAACAGCATCTGTTGCAACTCACAGTCTGGGGCATCAAGCAGCTCCAGGCAAGA---GTCTTGGCTGTGGAAAGATACCTAAAGGATCAACAGCTCCTAGGGATTTGGGGTTGCTCTGGAAAACGCATCTGCACCACTAATGTGCCTTGGAATACTAGTTGGAGT---------------------------AATAAATCTCTGAATGATATTTGGGAT---AACATGACCTGGATGCAGTGGGAAAAAGAAATTGAC------AATTACACAAGCATAATATACACTTTAATTGAAGAATCGCAGAACCAGCAAGAAAAGAATGAACAGGAGTTATTGGAATTAGATAAATGGGCAAATTTGTGGAATTGGTTTTCCATAACACAATGGCTGTGGTATATAAAAATATTCATAATGATAGTAGGAGGCTTGATAGGTTTAAGAATAGTTTTTACTGTGCTTTCTATAGTGAATAGAGTTAGGCAGGGATACTCACCATTATCGTTTCAGACCCAC---CTCCCAGCCCAGAGGGGA------CACGACAGGCCCGAAGGAATCGAAGAAGAAGGTGGAGAGAGAGACAGAGACAGATCCGGAAGATTAGTGGATGGATTGTTGACAATTATCTGGGTCGACCTACGGAGCCTGTGCCTCTTCAGCTACCACCGCTTGAGAGACTTACTCTTGATTGTAACGAGGATTGTGGAACTTCTGGGACGCAGG---------------GGGTGGGAAGCCCTCAAATATTGGTGG---AATCTCCTTCAGTATTGG---------------------------------------------------GGTCAGGAACTAAAGAATAGTGCTGTTAGCTTGCTCAATGTCACAGCTATAGCAGTAGCTGAGGGAACAGATAGGATTATAGAAGTAGTACAAAGA------------------GCTTGTAGAGCTATTCGCAACATACCTGTAAGAATCAGACAGGGCTTGGAAAGGCTTTTGCTATAA

2.12008.SPD.EU575459 ATGAGAGTGATGGAGATCAGGAGGAACTATCAGCTCTTG---------TGGAAAGGGGGC------------------------ATCTTGCTCCTTGGGATGTTAATGATC------------TGTAATACTTCA---------GAAAAATTGTGGGTCACAGTCTATTATGGGGTACCTGTGTGGAAAGAAGCAACCACCACTCTATTTTGTGCCTCAGATGCTAAAGGACATGAGACAGAGGTACATAAT---GTCTGGGCCACACATGCCTGTGTACCCACAGACCCCAACCCACAAGAAATAGTATTG---AGAAATGTGACAGAAGAGTTTAACATATGGAAAAATAACATGGTAGAACAGATGCATGAGGATATAATCAGTTTATGGGATCAAAGCCTAAAGCCATGTGTAAAATTAACCCCACTCTGTGTCACTTTAAAGTGCACTGATGATTGGAATACTACTAAAATCATTGAGAATCGGAGTGCTACTGGAACCAATGTGACGAGT---------GCTCCTACTAGTACCCCTGTGACAACTGAGACTACAACCCGTAATAGTAGTGAGGGGAGATTAATGGAGATAAAAGAAATGAAAAACTGCTCTTTCAAGGTCACC---TCAAACGTAAGAGAT------AAGGTGCAGGAAGAATATGCACTTTTTTATAAATTTGATATAATACCAATAGGCAATGAT------------------------------AATAATACT------------------AATACCAGTTATAGGTTGATAAATTGCAACACCTCAGTCATTACACAAGCCTGTCCAAAAGTATCCTTTGAGCCAATTCCCATACATTATTGTGCCCCGGCTGGTTTTGCGATTCTAAAGTGT---AATGATAAGGAGTTCAATGGAACAGGACTGTGTACAAATGTTAGCACAGTACAATGTACACATGGAATTAGGCCAGTAGTGTCAACACAACTACTGTTAAATGGCAGTCTAGCAGAAGAA---AAGATAGTAATTAGATCTGAAAACATCACGAACAATGCTAAAACCATAATAGTACATCTGAACGAACCTATAAAAATTAATTGTACAAGACCCAGCAACAATACAAGAAAAAGTATACCTATAGGA------------CCAGGGAGA---GCATTTTATGCAACAGGAGAAATAATAGGAGATATAAGACAAGCACATTGTAACATT------AGTCAAAAAGATTGGAATACCACCTTAGAACGGGTAGTTGAAACATTAAGA---AGAACATTTGGG------------AATAAAACA---ATAATATTTGATCGA---------TCCTCAGGAGGGGACCCAGAAATTACAATGCACAGTTTTAATTGTGGAGGGGAATTTTTCTACTGTAATACATCATCACTGTTTAATAGTACTTGGGGCAGT---------AATGGTACTTGGAAGGGTACA---------------------AATGGCACTAGAGACGAA---------------------------------------------------ATCACACTCCAA----------AACAAATTATAAACATGTGGCAGGAAGTAGGAAAAGCAATGTATGCCCCTCCCATCAGAGGAAATATTAGCTGCTCATCAAATATTACTGGGCTGCTATTAACAAGAGATGGTGGTACTGGCAAG---------AACACTGCCCAG------------------------AAC---GAGACCTTCAGACCTGGAGGAGGAAATATGAAAGACAATTGGAGA---AATGAGTTATATAAATATAAAGTAGTAAAAATTGAACCA---TTAGGAGTAGCACCC---ACCAAGGCAAGGAGAAGAGTGGTGCAGAGAGAA---AAAAGAGCAGTG---GGA---ATAGGA---GCTGTG---TTCCTT---GGG---------TTCTTGTCA---GCAGCAGGAAGCACTATGGGCGCAGCGTCAATG---ACGCTGACGGTACAGGCCAGACAATTATTGTCTGGTATAGTGCAACAGCAAAGCAACTTGCTGAGGGCTATTGAGGCGCAACAGCATCTGTTGCAACTCACAGTCTGGGGCATCAAGCAGCTCCAGGCAAGA---GTCTTGGCTGTGGAAAGATACCTAAAGGATCAACAGCTCCTAGGGATTTGGGGTTGCTCTGGAAAACGCATCTGCACCACTAATGTGCCTTGGAATACTAGTTGGAGT---------------------------AATAAATCTCTGAATGATATTTGGGAT---AACATGACCTGGATGCAGTGGGAAAAAGAAATTGAC------AATTACACAAGCATAATATACACTTTAATTGAAGAATCGCAGAACCAGCAAGAAAAGAATGAACAGGAGTTATTGGAATTAGATAAATGGGCAAATTTGTGGAATTGGTTTTCCATAACACAATGGCTGTGGTATATAAAAATATTCATAATGATAGTAGGAGGCTTGATAGGTTTAAGAATAGTTTTTACTGTGCTTTCTATAGTGAATAGAGTTAGGCAGGGATACTCACCATTATCGTTTCAGACCCAC---CTCCCAGCCCAGAGGGGA------CACGACAGGCCCGAAGGAATCGAAGAAGAAGGTGGAGAGAGAGACAGAGACAGATCCGGAAGATTAGTGGATGGATTGTTGACAATTATCTGGGTCGACCTACGGAGCCTGTGCCTCTTCAGCTACCACCGCTTGAGAGACTTACTCTTGATTGTAACGAGGATTGTGGAACTTCTGGGACGCAGG---------------GGGTGGGAAGCCCTCAAATATTGGTGG---AATCTCCTTCAGTATTGG---------------------------------------------------GGTCAGGAACTAAAGAATAGTGCTGTTAGCTTGCTCAATGTCACAGCTATAGCAGTAGCTGAGGGAACAGATAGGATTATAGAAGTAGTACAAAGA------------------GCTTGTAGAGCTATTCGCAACATACCTGTAAGAATCAGACAGGGCTTGGAAAGGCTTTTGCTATAA

2.12008.SPD.EU575444 ATGAGAGTGATGGAGATCAGGAGGAACTATCAGCTCTTG---------TGGAAAGGGGGC------------------------ATCTTGCTCCTTGGGATGTTAATGATC------------TGTAATACTTCA---------GAAAAATTGTGGGTCACAGTCTATTATGGGGTACCTGTGTGGAAAGAAGCAACCACCACTCTATTTTGTGCCTCAGATGCTAAAGGACATGAGACAGAGGTACATAAT---GTCTGGGCCACACATGCCTGTGTACCCACAGACCCCAACCCACAAGAAATAGTATTG---AGAAATGTGACAGAAGAGTTTAACATATGGAAAAATAACATGGTAGAACAGATGCATGAGGATATAATCAGTTTATGGGATCAAAGCCTAAAGCCATGTGTAAAATTAACCCCACTCTGTGTCACTTTAAAGTGCACTGATGATTGGAATACTACTAAAATCATTGAGAATCGGAGTGCTACTGGAACCAATGTGACGAGT---------GCTCCTACTAGTACCCCTGTGACAACTGAGACTACAACCCGTAATAGTAGTGAGGGGAGATTAATGGAGATAAAAGAAATGAAAAACTGCTCTTTCAAGGTCACC---TCAAACGTAAGAGAT------AAGGTGCAGGAAGAATATGCACTTTTTTATAAATTTGATATAATACCAATAGGCAATGAT------------------------------AATAATACT------------------AATACCAGTTATAGGTTGATAAATTGCAACACCTCAGTCATTACACAAGCCTGTCCAAAAGTATCCTTTGAGCCAATTCCCATACATTATTGTGCCCCGGCTGGTTTTGCGATTCTAAAGTGT---AATGATAAGGAGTTCAATGGAACAGGACTGTGTACAAATGTTAGCACAGTACAATGTACACATGGAATTAGGCCAGTAGTGTCAACACAACTACTGTTAAATGGCAGTCTAGCAGAAGAA---AAGATAGTAATTAGATCTGAAAACATCACGAACAATGCTAAAACCATAATAGTACATCTGAACGAACCTATAAAAATTAATTGTACAAGACCCAGCAACAATACAAGAAAAAGTATACCTATAGGA------------CCAGGGAGA---GCATTTTATGCAACAGGAGAAATAATAGGAGATATAAGACAAGCACATTGTAACATT------AGTCAAAAAGATTGGAATACCACCTTAGAACGGGTAGTTGAAACATTAAGA---AGAACATTTGGG------------AATAAAACA---ATAATATTTGATCGA---------TCCTCAGGAGGGGACCCAGAAATTACAATGCACAGTTTTAATTGTGGAGGGGAATTTTTCTACTGTAATACATCATCACTGTTTAATAGTACTTGGGGCAGT---------AATGGTACTTGGAAGGGTACA---------------------AATGGCACTAGAGACGAA---------------------------------------------------ATCACACTCCAATGCAGAATAAAACAAATTATAAACATGTGGCAGGAAGTAGGAAAAGCAATGTATGCCCCTCCCATCAGAGGAAATATTAGCTGCTCATCAAATATTACTGGGCTGCTATTAACAAGAGATGGTGGTACTGGCAAG---------AACACTGCCCAG------------------------AAC---GAGACCTTCAGACCTGGAGGAGGAAATATGAAAGACAATTGGAGA---AATGAGTTATATAAATATAAAGTAGTAAAAATTGAACCA---TTAGGAGTAGCACCC---ACCAAGGCAAGGAGAAGAGTGGTGCAGAGAGAA---AAAAGAGCAGTG---GGA---ATAGGA---GCTGTG---TTCCTT---GGG---------TTCTTGTCA---GCAGCAGGAAGCACTATGGGCGCAGCGTCAATG---ACGCTGACGGTACAGGCCAGACAATTATTGTCTGGTATAGTGCAACAGCAAAGCAACTTGCTGAGGGCTATTGAGGCGCAACAGCATCTGTTGCAACTCACAGTCTGGGGCATCAAGCAGCTCCAGGCAAGA---GTCTTGGCTGTGGAAAGATACCTAAAGGATCAACAGCTCCTAGGGATTTGGGGTTGCTCTGGAAAACGCATCTGCACCACTAATGTGCCTTGGAATACTAGTTGGAGT---------------------------AATAAATCTCTGAATGATATTTGGGAT---AACATGACCTGGATGCAGTGGGAAAAAGAAATTGAC------AATTACACAAGCATAATATACACTTTAATTGAAGAATCGCAGAACCAGCAAGAAAAGAATGAACAGGAGTTATTGGAATTAGATAAATGGGCAAATTTGTGGAATTGGTTTTCCATAACACAATGGCTGTGGTATATAAAAATATTCATAATGATAGTAGGAGGCTTGATAGGTTTAAGAATAGTTTTTACTGTGCTTTCTATAGTGAATAGAGTTAGGCAGGGATACTCACCATTATCGTTTCAGACCCAC---CTCCCAGCCCAGAGGGGA------CACGACAGGCCCGAAGGAATCGAAGAAGAAGGTGGAGAGAGAGACAGAGACAGATCCGGAAGATTAGTGGATGGATTGTTGACAATTATCTGGGTCGACCTACGGAGCCTGTGCCTCTTCAGCTACCACCGCTTGAGAGACTTACTCTTGATTGTAACGAGGATTGTGGAACTTCTGGGACGCAGG---------------GGGTGGGAAGCCCTCAAATATTGGTGG---AATCTCCTTCAGTATTGG---------------------------------------------------GGTCAGGAACTAAAGAATAGTGCTGTTAGCTTGCTCAATGTCACAGCTATAGCAGTAGCTGAGGGAACAGATAGGATTATAGAAGTAGTACAAAGA------------------GCTTGTAGAGCTATTCGCAACATACCTGTAAGAATCAGACAGGGCTTGGAAAGGCTTTTGCTATAA

2.12008.SPD.EU575449 ATGAGAGTGATGGAGATCAGGAGGAACTATCAGCTCTTG---------TGGAAAGGGGGC------------------------ATCTTGCTCCTTGGGATGTTAATGATC------------TGTAATACTTCA---------GAAAAATTGTGGGTCACAGTCTATTATGGGGTACCTGTGTGGAAAGAAGCAACCACCACTCTATTTTGTGCCTCAGATGCTAAAGGACATGAGACAGAGGTACATAAT---GTCTGGGCCACACATGCCTGTGTACCCACAGACCCCAACCCACAAGAAATAGTATTG---AGAAATGTGACAGAAGAGTTTAACATATGGAAAAATAACATGGTAGAACAGATGCATGAGGATATAATCAGTTTATGGGATCAAAGCCTAAAGCCATGTGTAAAATTAACCCCACTCTGTGTCACTTTAAAGTGCACTGATGATTGGAATACTACTAAAATCATTGAGAATCGGAGTGCTACTGGAACCAATGTGACGAGT---------GCTCCTACTAGTACCCCTGTGACAACTGAGACTACAACCCGTAATAGTAGTGAGGGGAGATTAATGGAGATAAAAGAAATGAAAAACTGCTCTTTCAAGGTCACC---TCAAACGTAAGAGAT------AAGGTGCAGGAAGAATATGCACTTTTTTATAAATTTGATATAATACCAATAGGCAATGAT------------------------------AATAATACT------------------AATACCAGTTATAGGTTGATAAATTGCAACACCTCAGTCATTACACAAGCCTGTCCAAAAGTATCCTTTGAGCCAATTCCCATACATTATTGTGCCCCGGCTGGTTTTGCGATTCTAAAGTGT---AATGATAAGGAGTTCAATGGAACAGGACTGTGTACAAATGTTAGCACAGTACAATGTACACATGGAATTAGGCCAGTAGTGTCAACACAACTACTGTTAAATGGCAGTCTAGCAGAAGAA---AAGATAGTAATTAGATCTGAAAACATCACGAACAATGCTAAAACCATAATAGTACATCTGAACGAACCTATAAAAATTAATTGTACAAGACCCAGCAACAATACAAGAAAAAGTATACCTATAGGA------------CCAGGGAGA---GCATTTTATGCAACAGGAGAAATAATAGGAGATATAAGACAAGCACATTGTAACATT------AGTCAAAAAGATTGGAATACCACCTTAGAACGGGTAGTTGAAACATTAAGA---AGAACATTTGGG------------AATAAAACA---ATAATATTTGATCGA---------TCCTCAGGAGGGGACCCAGAAATTACAATGCACAGTTTTAATTGTGGAGGGGAATTTTTCTACTGTAATACATCATCACTGTTTAATAGTACTTGGGGCAGT---------AATGGTACTTGGAAGGGTACA---------------------AATGGCACTAGAGACGAA---------------------------------------------------ATCACACTCCAATGCAGAATAAAACAAATTATAAACATGTGGCAGGAAGTAGGAAAAGCAATGTATGCCCCTCCCATCAGAGGAAATATTAGCTGCTCATCAAATATTACTGGGCTGCTATTAACAAGAGATGGTGGTACTGGCAAG---------AACACTGCCCAG------------------------AAC---GAGACCTTCAGACCTGGAGGAGGAAATATGAAAGACAATTGGAGA---AATGAGTTATATAAATATAAAGTAGTAAAAATTGAACCA---TTAGGAGTAGCACCC---ACCAAGGCAAGGAGAAGAGTGGTGCAGAGAGAA---AAAAGAGCAGTG---GGA---ATAGGA---GCTGTG---TTCCTT---GGG---------TTCTTGTCA---GCAGCAGGAAGCACTATGGGCGCAGCGTCAATG---ACGCTGACGGTACAGGCCAGACAATTATTGTCTGGTATAGTGCAACAGCAAAGCAACTTGCTGAGGGCTATTGAGGCGCAACAGCATCTGTTGCAACTCACAGTCTGGGGCATCAAGCAGCTCCAGGCAAGA---GTCTTGGCTGTGGAAAGATACCTAAAGGATCAACAGCTCCTAGGGATTTGGGGTTGCTCTGGAAAACGCATCTGCACCACTAATGTGCCTTGGAATACTAGTTGGAGT---------------------------AATAAATCTCTGAATGATATTTGGGAT---AACATGACCTGGATGCAGTGGGAAAAAGAAATTGAC------AATTACACAAGCATAATATACACTTTAATTGAAGAATCGCAGAACCAGCAAGAAAAGAATGAACAGGAGTTATTGGAATTAGATAAATGGGCAAATTTGTGGAATTGGTTTTCCATAACACAATGGCTGTGGTATATAAAAATATTCATAATGATAGTAGGAGGCTTGATAGGTTTAAGAATAGTTTTTACTGTGCTTTCTATAGTGAATAGAGTTAGGCAGGGATACTCACCATTATCGTTTCAGACCCAC---CTCCCAGCCCAGAGGGGA------CACGACAGGCCCGAAGGAATCGAAGAAGAAGGTGGAGAGAGAGACAGAGACAGATCCGGAAGATTAGTGGATGGATTGTTGACAATTATCTGGGTCGACCTACGGAGCCTGTGCCTCTTCAGCTACCACCGCTTGAGAGACTTACTCTTGATTGTAACGAGGATTGTGGAACTTCTGGGACGCAGG---------------GGGTGGGAAGCCCTCAAATATTGGTGG---AATCTCCTTCAGTATTGG---------------------------------------------------GGTCAGGAACTAAAGAATAGTGCTGTTAGCTTGCTCAATGTCACAGCTATAGCAGTAGCTGAGGGAACAGATAGGATTATAGAAGTAGTACAAAGA------------------GCTTGTAGAGCTATTCGCAACATACCTGTAAGAATCAGACAGGGCTTGGAAAGGCTTTTGCTATAA

2.12008.SPD.EU575467 ATGAGAGTGATGGAGATCAGGAGGAACTATCAGCTCTTG---------TGGAAAGGGGGC------------------------ATCTTGCTCCTTGGGATGTTAATGATC------------TGTAATACTTCA---------GAAAAATTGTGGGTCACAGTCTATTATGGGGTACCTGTGTGGAAAGAAGCAACCACCACTCTATTTTGTGCCTCAGATGCTAAAGGACATGAGACAGAGGTACATAAT---GTCTGGGCCACACATGCCTGTGTACCCACAGACCCCAACCCACAAGAAATAGTATTG---AGAAATGTGACAGAAGAGTTTAACATATGGAAAAATAACATGGTAGAACAGATGCATGAGGATATAATCAGTTTATGGGATCAAAGCCTAAAGCCATGTGTAAAATTAACCCCACTCTGTGTCACTTTAAAGTGCACTGATGATTGGAATACTACTAAAATCATTGAGAATCGGAGTGCTACTGGAACCAATGTGACGAGT---------GCTCCTACTAGTACCCCTGTGACAACTGAGACTACAACCCGTAATAGTAGTGAGGGGAGATTAATGGAGATAAAAGAAATGAAAAACTGCTCTTTCAAGGTCACC---TCAAACGTAAGAGAT------AAGGTGCAGGAAGAATATGCACTTTTTTATAAATTTGATATAATACCAATAGGCAATGAT------------------------------AATAATACT------------------AATACCAGTTATAGGTTGATAAATTGCAACACCTCAGTCATTACACAAGCCTGTCCAAAAGTATCCTTTGAGCCAATTCCCATACATTATTGTGCCCCGGCTGGTTTTGCGATTCTAAAGTGT---AATGATAAGGAGTTCAATGGAACAGGACTGTGTACAAATGTTAGCACAGTACAATGTACACATGGAATTAGGCCAGTAGTGTCAACACAACTACTGTTAAATGGCAGTCTAGCAGAAGAA---AAGATAGTAATTAGATCTGAAAACATCACGAACAATGCTAAAACCATAATAGTACATCTGAACGAACCTATAAAAATTAATTGTACAAGACCCAGCAACAATACAAGAAAAAGTATACCTATAGGA------------CCAGGGAGA---GCATTTTATGCAACAGGAGAAATAATAGGAGATATAAGACAAGCACATTGTAACATT------AGTCAAAAAGATTGGAATACCACCTTAGAACGGGTAGTTGAAACATTAAGA---AGAACATTTGGG------------AATAAAACA---ATAATATTTGATCGA---------TCCTCAGGAGGGGACCCAGAAATTACAATGCACAGTTTTAATTGTGGAGGGGAATTTTTCTACTGTAATACATCATCACTGTTTAATAGTACTTGGGGCAGT---------AATGGTACTTGGAAGGGTACA---------------------AATGGCACTAGAGACGAA---------------------------------------------------ATCACACTCCAATGCAGAATAAAACAAATTATAAACATGTGGCAGGAAGTAGGAAAAGCAATGTATGCCCCTCCCATCAGAGGAAATATTAGCTGCTCATCAAATATTACTGGGCTGCTATTAACAAGAGATGGTGGTACTGGCAAG---------AACACTGCCCAG------------------------AAC---GAGACCTTCAGACCTGGAGGAGGAAATATGAAAGACAATTGGAGA---AATGAGTTATATAAATATAAAGTAGTAAAAATTGAACCA---TTAGGAGTAGCACCC---ACCAAGGCAAGGAGAAGAGTGGTGCAGAGAGAA---AAAAGAGCAGTG---GGA---ATAGGA---GCTGTG---TTCCTT---GGG---------TTCTTGTCA---GCAGCAGGAAGCACTATGGGCGCAGCGTCAATG---ACGCTGACGGTACAGGCCAGACAATTATTGTCTGGTATAGTGCAACAGCAAAGCAACTTGCTGAGGGCTATTGAGGCGCAACAGCATCTGTTGCAACTCACAGTCTGGGGCATCAAGCAGCTCCAGGCAAGA---GTCTTGGCTGTGGAAAGATACCTAAAGGATCAACAGCTCCTAGGGATTTGGGGTTGCTCTGGAAAACGCATCTGCACCACTAATGTGCCTTGGAATACTAGTTGGAGT---------------------------AATAAATCTCTGAATGATATTTGGGAT---AACATGACCTGGATGCAGTGGGAAAAAGAAATTGAC------AATTACACAAGCATAATATACACTTTAATTGAAGAATCGCAGAACCAGCAAGAAAAGAATGAACAGGAGTTATTGGAATTAGATAAATGGGCAAATTTGTGGAATTGGTTTTCCATAACACAATGGCTGTGGTATATAAAAATATTCATAATGATAGTAGGAGGCTTGATAGGTTTAAGAATAGTTTTTACTGTGCTTTCTATAGTGAATAGAGTTAGGCAGGGATACTCACCATTATCGTTTCAGACCCAC---CTCCCAGCCCAGAGGGGA------CACGACAGGCCCGAAGGAATCGAAGAAGAAGGTGGAGAGAGAGACAGAGACAGATCCGGAAGATTAGTGGATGGATTGTTGACAATTATCTGGGTCGACCTACGGAGCCTGTGCCTCTTCAGCTACCACCGCTTGAGAGACTTACTCTTGATTGTAACGAGGATTGTGGAACTTCTGGGACGCAGG---------------GGGTGGGAAGCCCTCAAATATTGGTGG---AATCTCCTTCAGTATTGG---------------------------------------------------GGTCAGGAACTAAAGAATAGTGCTGTTAGCTTGCTCAATGTCACAGCTATAGCAGTAGCTGAGGGAACAGATAGGATTATAGAAGTAGTACAAAGA------------------GCTTGTAGAGCTATTCGCAACATACCTGTAAGAATCAGACAGGGCTTGGAAAGGCTTTTGCTATAA
[truncated: 4,206,712 more chars]
