# Supplementary material for: Recurrent Signature Patterns in HIV-1 B Clade Envelope Glycoproteins Associated with either Early or Chronic Infections
Source: PLoS Pathog. 2011 Sep 29;7(9):e1002209. doi: 10.1371/journal.ppat.1002209 (PMC3182927; doi:10.1371/journal.ppat.1002209)
Supplement: Table S8 — Distributions of Amino Acid Sets for Cluster 437: Sites 207, 326, 327, 422, 436, 437, 439. Sphere-sets (sets/clusters of amino acid sites within a sphere centered on a surface residue with diameter selected to fit a typical conformational antibody epitope) potentially containing antibody epitopes were evaluated using a generalized linear model fit by generalized estimating equations, to assess a different distribution of amino acid patterns in the acute/early group versus the chronic group, relative to the reference set (the consensus among acute/early sequences). Cluster 437 (comprised of sites 207, 326, 327, 422, 436, 437, 439) had q-value<0.001 and Hom-Bonferroni adjusted p-value<0.001 for the Holdout data, and is noteworthy because the q-value and adjusted p-value are small, and because it includes CD4-induced epitope sites originally described in Wyatt et al. (1998). The table shows the distribution of amino acid sets in cluster 437 for the Original data and for the Holdout data. For the Holdout data several patterns of mutation away from the reference set KIRQAPI are over-represented in the chronic sequences. However, for the Original data the pattern of amino acid mutations was opposite to that for the holdout data, with mutation away from the reference set KIRQAPI slightly over-represented in acute/early sequences. (DOC) [file ppat.1002209.s015.doc]

**Table S8. Distributions of Amino Acid Sets for Cluster 437: Sites 207, 326, 327, 422, 436, 437, 439.**

|  | **Original Data** | |  | **Holdout Data** | |
| --- | --- | --- | --- | --- | --- |
| **AA Pattern** | **Acute/Early** | **Chronic** |  | **Acute/Early** | **Chronic** |
| KIRQAPI | 1207 | 847 |  | 1262 | 1119 |
| –IRQAPI | 0 | 0 |  | 0 | 3 |
| K–––––– | 0 | 0 |  | 0 | 4 |
| K–––API | 0 | 0 |  | 0 | 1 |
| KIGQAPI | 1 | 0 |  | 0 | 0 |
| KIKQAPI | 2 | 0 |  | 0 | 15 |
| KIR–API | 1 | 0 |  | 1 | 2 |
| KIR$API | 0 | 0 |  | 1 | 1 |
| KIR\#API | 1 | 0 |  | 0 | 1 |
| KIRKAPI | 0 | 0 |  | 1 | 0 |
| KIRQAHI | 1 | 0 |  | 0 | 0 |
| KIRQALI | 3 | 0 |  | 1 | 0 |
| KIRQAPT | 0 | 0 |  | 0 | 1 |
| KIRQAPV | 1 | 0 |  | 0 | 10 |
| KIRQASI | 0 | 0 |  | 0 | 5 |
| KIRQATI | 0 | 0 |  | 1 | 0 |
| KIRQ––I | 0 | 0 |  | 0 | 1 |
| KIRQTPI | 1 | 0 |  | 3 | 0 |
| K––QAPI | 0 | 0 |  | 0 | 1 |
| KIRQTSI | 0 | 0 |  | 0 | 11 |
| KIRQVPI | 1 | 0 |  | 0 | 0 |
| KIRRAPI | 1 | 0 |  | 0 | 0 |
| KTRQAPI | 53 | 0 |  | 0 | 6 |
| KVKQAPI | 0 | 0 |  | 0 | 5 |
| KVRQAPI | 0 | 1 |  | 0 | 44 |

**Table S8. Distributions of Amino Acid Sets for Cluster 437: Sites 207, 326, 327, 422, 436, 437, 439*.** Sphere-sets (sets/clusters of amino acid sites within a sphere centered on a surface residue with diameter selected to fit a typical conformational antibody epitope) potentially containing antibody epitopes were evaluated using a generalized linear model fit by generalized estimating equations, to assess a different distribution of amino acid patterns in the acute/early group versus the chronic group, relative to the reference set (the consensus among acute/early sequences). Cluster 437 (comprised of sites 207, 326, 327, 422, 436, 437, 439) had q-value < 0.001 and Hom-Bonferroni adjusted p-value < 0.001 for the Holdout data, and is noteworthy because the q-value and adjusted p-value are small, and because it includes CD4-induced epitope sites originally described in Wyatt et al. (1998). The table shows the distribution of amino acid sets in cluster 437 for the Original data and for the Holdout data. For the Holdout data several patterns of mutation away from the reference set KIRQAPI are over-represented in the chronic sequences. However, for the Original data the pattern of amino acid mutations was opposite to that for the holdout data, with mutation away from the reference set KIRQAPI slightly over-represented in acute/early sequences. Finally, - indicates a gap; $ indicates a stop codon; # indicates a frameshift. If the nucleotide sequence only has a partial codon (e.g., “GC-” or “A-C” or “T-” ), then the amino acid was coded as #.
