# Supplementary material for: Recurrent Signature Patterns in HIV-1 B Clade Envelope Glycoproteins Associated with either Early or Chronic Infections
Source: PLoS Pathog. 2011 Sep 29;7(9):e1002209. doi: 10.1371/journal.ppat.1002209 (PMC3182927; doi:10.1371/journal.ppat.1002209)
Supplement: Text S1 — This part summarizes strategies to define Acute versus Chronic HIV Signature Analysis via Non-Phylogenetically-Corrected Statistical Analyses. (DOC) [file ppat.1002209.s016.doc]

**Supplemental Text S1.**

**Summary of strategies to define Acute versus Chronic HIV Signature Analysis via Non-Phylogenetically-Corrected Statistical Analyses**

**Statistical Methods for Signature Position Analysis**

Objective: To identify signature positions, defined either as positions with different amino acid frequencies among acute and chronic sequences (type B signatures); or as positions with different amino acid divergences from a reference amino acid (type A signatures).

The consensus of the subject-specific consensus sequences of the 26 Fiebig stage I-II acutely infected Original set of subjects was used as the reference sequence in all assessments of type A signatures. A further goal is to identify the particular amino acids at certain positions that are differentially represented in acute versus chronic sequences. Finally, an aim is to identify sets of positions at which certain amino acid patterns are over-represented in acute compared to chronic sequences.

**Data**

The Original and Holdout data-sets described in Table 1 of the main paper were analyzed. As in the main paper, throughout the “acute” sequence set is those sequences sampled at Fiebig Stage I-VI and the “chronic” sequence set is those sequences sampled after Fiebig Stage VI. For these analyses a small number of sequences were removed due to evidence of epidemiological-linkage or to the fact that subjects had both acute and chronic sequences, in which case only the acute sequences were retained. In total 47 acute subjects and 40 chronic subjects were analyzed.

Statistical procedures to identify significant or predictive positions were performed on the original data. The holdout data set was used for verifying whether the positions found significant based on the original data are also significant for the holdout data, and for quantifying the prediction accuracy of predictive models built using the original data.

**Analysis 1: Testing for Type B Signature Positions from Subject-Specific Consensus Sequences**

We compared the 47 subject-specific consensus sequences from acutes and 40 subject-specific consensus sequences from chronics. Two-sample test statistics were applied for each position, with or without stratification by B sub-clade (b/c versus a/d/e, which is equivalent to Trinidad versus non-Trinidad), to provide some protection against bias from phylogenetic confounding. For evaluating type B signatures three statistical testing procedures of Gilbert, Wu, and Jobes (2008) were used, based on the unstandardized Chi-square type statistic, the standardized Chi-square type statistic, and the Kullback-Leibler/mutual information statistic. For evaluating type A signatures the t-statistic type procedure of Gilbert, Wu, and Jobes (2008) was used. Unadjusted p-values for each site were computed using 10,000 permuted data-sets. Positions that are so conserved that it would not be possible to identify a significant signature position were screened out using Tarone's (1990) procedure.

To account for the multiple hypothesis tests across amino acid sites, we computed Holm-Bonferroni family-wise error rate (FWER) adjusted p-values and computed q-values. Both of these were computed on the set of sites that survived the Tarone (1990) screen-out procedure, which is known to be valid for obtaining false positive error rate control.

**Analysis 2: Testing for Type B Signature Positions from All Individual Sequences**

In the second analysis, type B signature positions were evaluated using all of the individual sequences. We applied “pairwise sequence amino acid mismatch tests.” Specifically, for each position we first estimated the probability of inter-subject amino acid mismatch for all pairs of acute sequences, using the observed frequency of mismatch. Second we estimated the probability of inter-subject amino acid mismatch for all pairs of acute versus chronic sequences. We then took the difference of these estimated probabilities as a test statistic. If a position is a signature position, the parameter estimated by this difference will be significantly different from zero. Unadjusted p-values were computed using the nonparametric bootstrap with 10,000 data-sets sampled with replacement. This method provides valid and robust inferences when there are different numbers of sequences per subject, as is the case for the available data.

The analyses were performed on all positions for which at least four subjects have a non-consensus amino acid, and FWER-adjusted p-values and q-values were computed in the same way as described above.

**Analysis 3: Testing for Type A Signature Positions from All Individual Sequences**

In the third analysis, type A signature positions were evaluated using all of the individual sequences. We sought to apply methods that provide valid and robust inferences given that there are different numbers of sequences per subject. To enable this, we evaluated type A signature positions using a generalized linear model fit by generalized estimating equations (GEE).

For amino acid divergence defined by 1 for mismatch and 0 for match, a logit link function was used to relate the marginal mean probability of mismatch to the status of acute versus chronic, adjusting for phylogenetic sub-clade defined above. The GEE method does not require any parametric assumptions. For each position the GEE method provides an estimate of the adjusted odds ratio (OR), interpreted as the odds ratio of a mutation (relative to reference) at the given position for acute versus chronic subjects, controlling for phylogenetic sub-clade. A robust variance estimate of the adjusted OR estimate was used, which was designed to be valid for data with different numbers of sequences per subject. An exchangeable correlation structure of the response for repeated measurements of sequences from the same individual was assumed, in other words we assumed the same correlation among the indicators of mutation for the sequences within a person. The data were used to estimate the correlation matrix separately for acutes and chronics. This is relevant because the greater homogeneity of the acute sequences is expected to make the covariance matrices different for acutes and chronics. For each site a Wald-based unadjusted p-value for a difference in acute versus chronic sequences is computed based on the coefficient estimate divided by the robust standard error.

The GEE analyses were performed on all positions for which at least four subjects have a non-consensus amino acid, and FWER-adjusted p-values and q-values were computed in the same way as described above.

**Analysis 4: Classifying/Predicting Acute or Chronic Status from All Individual Sequences**

We applied threshold gradient descent regularization (TGDR) (Friedman and Popescu, 2004) to evaluate the set of amino acids at certain positions that best predict whether a sequence is from an acute or chronic subject. Ten-fold cross-validation (on the original data set only) was used to optimize the “number of steps” tuning parameter of TGDR.

At each position, the indicators of all amino acids represented in the dataset (relative to the acute consensus amino acid as the reference amino acid) were used as potential predictors of acute/chronic status. All amino acid indicators at a position that were represented in fewer than four subjects were screened out. Of the original set of more than 20,000 indicators, fewer than 1000 remained after the screening. The output of the TGDR method includes measures of classification accuracy, which quantify how well the optimally predictive model predicts acute/chronic status on independent data (held out from the training data set). One hundred random splits of the original data set were made, with 2:1 allocation of subjects into training:test sets. Prediction accuracy was measured by misclassification rates and ROC curves for the ability of models fit on training data to predict acute/chronic status of sequences in the test set. The splits were done at the subject level, so that the sequences within a subject always stay with that subject. For each of a small set of prediction models that performed best on the original data set, the ability of the model to predict acute/chronic status in the holdout dataset was assessed with an ROC curve. An advantage of TGDR is that it has shown good performance to select multiple covariates that work together to predict the outcome. Thus the method can reflect co-varying mutations that “work together” to predict acute/chronic status.

The above model-selection analysis was repeated using additional machine learning methods: the inductive logic methods RIPPER, random forests, and logistic regression. The data are also evaluated with a decision stump, to determine a baseline predictive model (the simplest possible model based on the amino acid character at a single position).

**Analysis 5: Testing for Signature Potential B Cell Spatial Clusters from All Individual Sequences**

We identified spatial clusters of residues on gp120 that might be targeted by antibodies. A sphere was centered at each of 226 exposed surface residues, which were determined based on the HIV literature and measurement of buried surface area. The V1/V2 loops were eliminated because of the lack of known crystal structure, and residues known or expected to be non-exposed in the trimeric spike (inner domain, silent face, core of protein) were also excluded. The gp120 construct in the protein structure comes from the JR-FL strain.

The sphere radius was selected to be 7, 8, 9, 10, or 11 angstroms. This range was selected by analyzing the size of antibody epitopes in a data set of antibody-antigen structures (104 antibody-antigen complexes in the “Epitome” data set, Schlessinger et al., 2006, plus a non-redundant sub-set). Known epitopes overlap significantly with at least one of the 226 spatial clusters. In-dels were handled by removing all deletions from each cluster. Each large insertion (> 5 residues) was defined as its own cluster. If another cluster touched an edge of this insertion, then the edge residue was added to that cluster. Small insertions (≤ 5 residues) were added to any existing clusters that touched the insertion.

Similar to the plan for Analysis 3 above, we evaluated signature sphere-sets using a generalized linear model fit by generalized estimating equations (GEE). For each sphere set the response variable is the number of mismatched amino acids in the sphere set compared to the reference sphere set, which was assumed to be Poisson distributed (and hence the generalized linear model uses a log link function to relate the marginal mean number of mismatches to the status of acute versus chronic, adjusting for phylogenetic sub-clade).

As for Analysis 3 above, a robust variance estimate of the acute/chronic coefficient estimate was used, which ensures validity for data with different numbers of sequences per subject, and an exchangeable correlation structure of the response for repeated measurements of sequences from the same individual was assumed, with correlation matrix estimated separately for acutes and chronics. For each sphere-set a Wald-based unadjusted p-value for a difference in acute versus chronic sequences is computed based on the coefficient estimate divided by the robust standard error.

The GEE analyses were performed on all positions for which at least four subjects have a non-consensus sphere-set, and FWER-adjusted p-values and q-values were computed in the same way as described above.

All statistical tests use 2-sided p-values.

**Results**

Following the main paper, for the results based on hypothesis testing we require a q-value < 0.2 in the original data set and a q-value < 0.3 in the holdout data in order to flag the result as a statistically supported signature.

**Results of Analysis 1**

Based on the original data, one site had q-value < 0.2: position 30, with q-values of 0.04, 0.02, 0.08 (unadjusted p-values of 0.04, 0.02, 0.11) for the un-standardized Chi-square type, standardized Chi-square type, and mutual information-based tests. However the q-values for the holdout data were all above 0.5, and therefore no signature sites were supported by analysis 1.

**Results of Analysis 2**

Based on the original data, 4 sites had a q-value < 0.2 (sites 13, 20, 142g, 346; test statistics for all sites shown in Figure S3, where a large-magnitude test statistic suggests a difference in amino acid distribution at the site for acute versus chronic sequences). None of these sites had a q-value < 0.3 for the holdout data, and therefore no signature sites were supported by analysis 2.

**Results of Analysis 3**

Based on the original data, 3 sites had a q-value < 0.2 (sites 19, 30, 424, with q-values 0.02, 0.001, 0.008 and unadjusted p-values 0.004, < 0.001, 0.002). The estimated odds ratios of mutations away from the reference sequence residues W19, M30, and V424 were 4.5, 7.2, and 4.4, respectively. Based on the holdout data, the q-value was $>$ 0.5 for sites 30 and 424, and was 0.14 (with unadjusted p-value of 0.04) for site 19.

Therefore site 19 was the only statistically supported signature site identified by analysis 3. However, the estimated odds ratio on the holdout data at site 19 was 0.35, which is the opposite association as observed for the original data. Therefore no meaningful statistically significant signature sites were identified based on analysis 3.

Given that site 12 was a highly significant signature based on the phylogenetically-corrected methods used for the main paper, it is interesting to note that the GEE analysis provided some support for it as a signature site. For the original data the q-value was 0.21 and the unadjusted p-value was 0.08, with odds ratio of 2.1; whereas for the holdout data the q-value was 0.5 and the unadjusted p-value was 0.19, with odds ratio of 1.8.

**Results of Analysis 4**

Based on the original data, the TGDR method yields a best-classifying model that has the following six amino acid indicators: L20, M154, A346, V424, N636, I755. Based on the 100 random splits of the original data into 2:1 training:test splits, 100 areas under the ROC curve (AUCs) were computed to evaluate how well the best-model built on the training data predicted the independent test data. The median of the AUC was 0.61, suggesting weak classification accuracy but better than that expected from chance (which would be indicated by an AUC of 0.5). The fifth and ninety-fifth percentiles of the AUC on the independent test data were 0.44 and 0.72. These results are illustrated in Figure S4.

Based on the original data, the best inductive logic predictor was the decision stump, identifying that the best classifier of acute/chronic status based on a single amino acid site was an M at site 30, which predicted acute status with 73.6% accuracy. However, its classification accuracy was only 51.2\% on the holdout data, showing that this signature was not statistically supported. None of the other classification/prediction methods improved in classification power over the decision stump model, probably indicating that the more complex methods were over-training on signals involving amino acid interactions only found in the training sets.

These analyses confirm the conclusion from the main paper that the identified signature sites are only weakly predictive of acute/chronic status.

**Results of Analysis 5**

Figure S5 shows q-values for the analysis of the 226 sphere sets based on all individual sequences, separately for the original data and the holdout data, and Figure S6 shows the corresponding Holm-Bonferroni adjusted p-values. No sphere sets have q-value < 0.2 in the original data, whereas two sphere sets have q-value < 0.2 for the holdout data (Cluster 437 comprised of sites 207, 326, 327, 422, 436, 437, 439 with q < 0.001 and Holm-Bonferroni adjusted p-value < 0.0001; Cluster 264 comprised of sites 249, 262, 264, 265, 267, 484, 485 with q = 0.18 and Holm-Bonferroni adjusted p-value 0.37); thus no sphere sets were statistically supported signatures based on our pre-specified criteria.

Cluster 437 is noteworthy, both because the q-value for the holdout set was small, and because it includes CD4-induced epitope sites originally described in Wyatt et al. (1998). Table S9 and Figure S7 show the distribution of amino acid sets for cluster 437 for the original data and for the holdout data. Several patterns of mutation away from the reference set KIRQAPI were over-represented in the chronic sequences. For the original data, cluster 437 had an unadjusted p-value of 0.005, q-value of 0.84, and Holm-Bonferroni adjusted p-value 1.0; however the pattern of amino acid mutations was opposite to that for the holdout data, with mutation away from the reference set KIRQAPI slightly over-represented in acute sequences.

**Interpretation of Results**

In conclusion, based on the supplementary statistical signature analysis using methods that only accounted for phylogenetic structure through stratification by the two major phylogenetic sub-clades, no statistically robust signature sites or sets of sites were identified that were consistent in the Original data and in the Holdout data. This suggests that signatures are subtle and their detection (based on the limited numbers of subjects that contributed sequence data) required detailed phylogenetic correction. However, these supplementary analyses did lend further support to the strongest signature found from the phylogenetically-corrected analysis (at site 12), albeit not quite reaching the pre-specified levels of statistical significance after correction for multiple testing.

In addition, the fact that a few statistically significant signature sites/sphere sets identified in the Original data had opposite mutation patterns in the Holdout data raises the question as to whether the Holdout data-set was drawn from a similar-enough population as the Original data to facilitate a robust validation of signatures.

**References**

Friedman JH, Popescu BE (2004) Gradient directed regularization for linear regression and classification. Technical Report, Department of Statistics, Stanford University.

Gilbert PB, Wu C, Jobes DV (2008) Genome scanning methods for comparing sequences between groups, with application to HIV vaccine trials. Biometrics 64:198−207.

Nickle D, Heath L, Jensen M, Gilbert P, Mullins J, et al. (2007) HIV-specific probabilistic models of protein evolution. PLoS ONE June 6;2:e503.

Schlessinger A, Ofran Y, Yachdav G, Rost B (2006) Epitome: database of structure-inferred antigenic epitopes. Nucleic Acids Research 34, Suppl 1:D777−D780.

Tarone RE (1990) A modified Bonferroni method for discrete data. Biometrics 46:515−522.

Wyatt R, Kwong PD, Desjardins E, Sweet RW, Robinson J, et al. (1998) The antigenic structure of the HIV gp120 envelope glycoprotein. Nature 393:705−711.
